# Bond topology of chain, ribbon and tube silicates. Part I. Graph-theory generation of infinite one-dimensional arrangements of (*T*O_4_)^*n*−^ tetrahedra

**DOI:** 10.1107/S2053273322001747

**Published:** 2022-04-04

**Authors:** Maxwell Christopher Day, Frank Christopher Hawthorne

**Affiliations:** a Department of Earth Sciences, University of Manitoba, Winnipeg, Manitoba R3T 2N2, Canada

**Keywords:** bond topology, tetrahedra, chain graph, adjacency matrix, vertex set, edge set, silicates, graph generation, isomorphism, translational symmetry

## Abstract

Chain, ribbon and tube silicates are based on one-dimensional polymerizations of (*T*O_4_)^*n*−^ tetrahedra, where *T* = Si^4+^ plus P^5+^, V^5+^, As^5+^, Al^3+^, Fe^3+^ and B^3+^. Such polymerizations may be represented by infinite graphs (designated chain graphs) in which vertices represent tetrahedra and edges represent linkages between tetrahedra. The valence-sum rule of bond-valence theory limits the maximum degree of any vertex to 4 and the number of edges linking two vertices to 1 (corner-sharing tetrahedra). The unit cell (or repeat unit) of the chain graph generates the chain graph through action of translational symmetry operators. The (infinite) chain graph is converted into a finite graph by wrapping edges that exit the unit cell such that they link to vertices within the unit cell that are translationally equivalent to the vertices to which they link in the chain graph, and the wrapped graph preserves all topological information of the chain graph. A symbolic algebra is developed that represents the degree of each vertex in the wrapped graph. The wrapped graph is represented by its adjacency matrix which is modified to indicate the direction of wrapped edges, up (+**c**) or down (−**c**) along the direction of polymerization. The symbolic algebra is used to generate all possible vertex connectivities for graphs with ≤8 vertices. This method of representing chain graphs by finite matrices may now be inverted to generate all non-isomorphic chain graphs with ≤8 vertices for all possible vertex connectivities. *MatLabR2019b* code is provided for computationally intensive steps of this method and ∼3000 finite graphs (and associated adjacency matrices) and ∼1500 chain graphs are generated.

## Abstract 2

Chains of *T*O_4_^*n*−^ tetrahedra are represented as chain graphs in which tetrahedra are represented as vertices and the linkage between tetrahedra is represented as edges. Topologically distinct chain graphs are generated for all possible chain stoichiometries (up to a boundary number of tetrahedra) using the formalisms of graph theory, making possible the comparison of observed chain arrangements with all topologically possible chain arrangements.

## 1. Introduction

Silicon and oxygen are the most abundant elements in the crust and mantle of the Earth and silicates *sensu lato* are the most important constituents of many crust and mantle processes. As part of a larger programme to provide a framework for understanding the atomic scale factors controlling composition, structural variability and paragenesis (occurrence) of silicate minerals in the rocks of the Earth’s crust and mantle, Day & Hawthorne (2020 ▸) presented a structure hierarchy (Hawthorne, 2014 ▸) for chain-silicate minerals in which the silicate moiety consists of (*T*O_4_)^*n*−^ groups that are polymerized infinitely in one dimension to form chains, ribbons and tubes. For simplicity of expression, we denote ‘chains, ribbons and tubes’ as ‘chains’ except where it is necessary to distinguish between these three types of polymerization. Where we refer to a silicate chain, ribbon and/or tube, or other silicate unit (*i.e.* cluster, sheet and/or framework), let it be understood that the unit must contain Si^4+^ but may also contain other tetrahedrally coordinated cations: *e.g.* *T* = P^5+^, V^5+^, As^5+^, Si^4+^, Al^3+^, Fe^3+^ and B^3+^. For simplicity of expression, we refer to such compositions as silicates, whether or not the dominant tetrahedrally coordinated cation is Si^4+^ as we require them to contain Si^4+^ as an essential constituent.

We wish to understand the characteristics of graphs that give rise to different classes of mineral structures: (i) common minerals of high abundance; (ii) common minerals of low abundance; (iii) rare minerals of high abundance; (iv) rare minerals of low abundance; (v) no mineral structures at all. To do this we need to derive all possible one-dimensionally infinite graphs (up to some size limit) and examine what graph characteristics (*a*) prevent, and (*b*) allow, their embedding into Euclidean space with edge lengths and inter-edge angles compatible with crystal structures of specific types of general chemical composition. Our intent is not to predict crystal structures but to examine the factors that allow embedding of graphs into Euclidean space such that their geometrical characteristics are compatible with metrics of crystal structures. Here, we characterize all one-dimensional polymerizations of tetrahedra with periodic symmetry; in a later paper, we will address the issue of embedding these graphs into Euclidean space.

## 2. Terminology

Following Day & Hawthorne (2020 ▸), we define chains, ribbons and tubes as follows:

*Chain*: a structural unit of (*T*O_4_)^*n*−^ tetrahedra that link together infinitely in a single direction, and that can be broken into two parts by eliminating a single linkage between adjacent tetrahedra.

*Ribbon*: a structural unit of (*T*O_4_)^*n*−^ tetrahedra that link together infinitely in a single direction, and that cannot be broken into two parts by eliminating a single linkage between adjacent tetrahedra.

*Tube*: a structural unit of (*T*O_4_)^*n*−^ tetrahedra that link together infinitely in a single direction, and also link orthogonally to the direction of polymerization to form a hollow cylinder.

It can be difficult to differentiate some ribbon arrangements from tube arrangements when shown as graphs in two dimensions (*i.e*. illustrated on the printed page or a screen). Two-dimensional graphs of tubes are always non-planar: they always have apparently intersecting or overlapping edges. Two-dimensional graphs of ribbons and chains are planar and can always be drawn without any intersecting or overlapping edges. Although some graphs of ribbons and chains may show crossed edges, this feature may be removed by changing the view direction of the ribbon or chain and/or moving the vertices of the graph in the plane of the illustration while maintaining the connectivity of the graph; this is not possible for graphs of tubes.

*Cluster*: a zero-dimensional structural unit of linked (*T*O_4_)^*n*−^ tetrahedra that do not extend infinitely in any direction. The graph of a cluster may be planar or non-planar.

*Structural unit*: the strongly bonded part of a structure, con­sisting of oxyanions and low-coordination-number cations (Hawthorne, 1983 ▸, 2015*b* ▸).

*Repeat unit*: that part of a (i) chain, ribbon or tube that can be repeated by translational symmetry to produce the complete chain, ribbon or tube; (ii) finite graph that can be repeated by topological translational symmetry to produce the graph of the complete chain, ribbon or tube.

## 3. Previous work

Since the pioneering work of Wells (*e.g*. 1954 ▸, 1962 ▸, 1977 ▸), there has been much work on the description and generation of simple crystal structures using periodic nets. For example, Klee (1980 ▸) examined tetrahedron polymerizations in silicates and their corresponding periodic nets, and Klein (1996 ▸) generated 2-periodic nets and their corresponding sheets of tetrahedra. The quasi-infinite character of crystal structures presented a problem in their representation as finite graphs. However, this problem was overcome with the use of (finite) quotient graphs (Chung *et al.*, 1984 ▸; Eon, 1998 ▸, 1999 ▸, 2016 ▸; Klee, 2004 ▸). This work involved the description of known crystal structures using nets and the generation of new nets or graphs as potential arrangements of atoms in crystals, and extensive databases of structures and nets are available (*e.g.* Delgado-Friedrichs & O’Keeffe, 2003 ▸; Blatov *et al.*, 2014 ▸). Chung *et al.* (1984 ▸) used quotient graphs to describe and generate nets and this type of approach has been integrated in various software programs including *SYSTRE* (Delgado-Friedrichs & O’Keeffe, 2003 ▸) and *ToposPro* (Blatov *et al.*, 2014 ▸).

Work on silicates has tended to focus on framework and sheet structures (Smith, 1977 ▸, 1978 ▸, 1988 ▸; Liebau, 1985 ▸; Hawthorne, 2015*a* ▸; Hawthorne & Smith, 1986*a* ▸,*b* ▸, 1988 ▸; Hawthorne *et al.*, 2019 ▸; Krivovichev, 2008 ▸, 2009 ▸), and there is no comprehensive description of silicate chains, ribbons and tubes using graphs with one-dimensional periodic symmetry, except for some tubular chain silicates (Rozhdestvenskaya & Krivovichev, 2011 ▸). Previous methods involving quotient graphs have focused on describing and predicting crystal structures, and hence have dealt with graphs that (i) are already embedded in Euclidean space, and (ii) are connected. As (i) we wish to examine the details of embedding graphs into Euclidean space such that their metric properties are compatible with crystal structures, and (ii) do not wish to be restricted to connected graphs as some crystal structures consist of silicate polymerizations that are not connected to each other, we use a different approach to enumerating graphs.

## 4. Graphs

In this work we take a graph-theory approach and will begin by defining our terms as there are some differences in some of these definitions in the literature.

**Figure 1:**
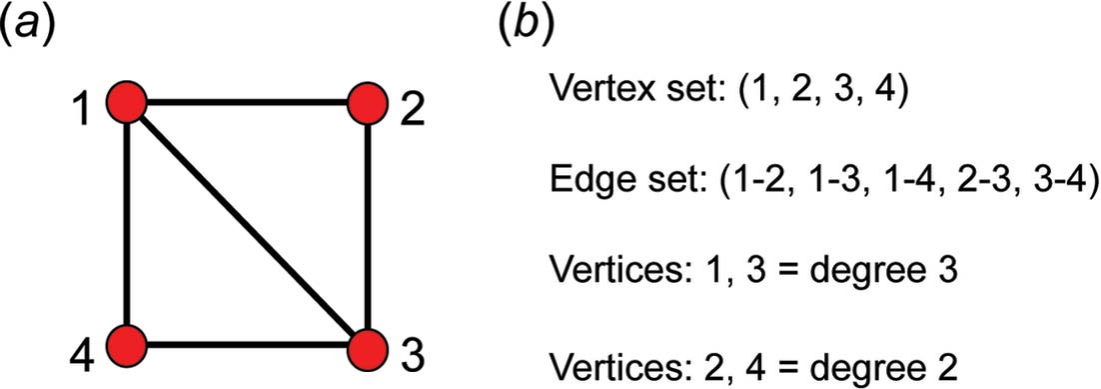
(*a*) A labelled graph with vertices indicated by red circles and edges indicated by black lines, (*b*) the vertex and edge set of the graph. Vertices 1 and 3 are of degree 3 (3-connected) and vertices 2 and 4 are of degree 2 (2-connected).

We may define a graph as a nonempty set of elements, *V*(*G*), called vertices, and a nonempty set of unordered pairs of these vertices, *E*(*G*), called edges (Wilson, 1979 ▸). We may colour the vertices and we may label the vertices. The result is a labelled polychromatic graph, illustrated pictorially in Fig. 1 ▸(*a*) and expressed in terms of its vertex set and edge set in Fig. 1 ▸(*b*). The degree of a vertex is the number of edges incident at that vertex; thus in Fig. 1 ▸(*a*), vertices 1 and 3 are of degree 3 and vertices 2 and 4 are of degree 2. Here we will use graphs to represent tetrahedra and linkages between tetrahedra: the vertex set represents tetrahedra and the edge set represents linkages between tetrahedra. As tetrahedra in silicates link only by sharing corners, vertices in the graphs we use to represent infinite linkages of tetrahedra are linked only by one or zero edges. Simple bond-valence arguments limit the maximum number of silicate tetrahedra to which a single tetrahedron may link to 4, *i.e.* the coordination of bridging O^2−^ ions by tetrahedrally coordinated cations cannot exceed 2 without violating the valence-sum rule (Brown, 2016 ▸). Thus, the maximum degree of a vertex in the graphs we will consider is 4. For convenience, we denote translationally symmetric infinite graphs with vertex degrees 1–4 as chain graphs.

### 4.1. How to represent infinite chains as finite graphs

**Figure 2:**
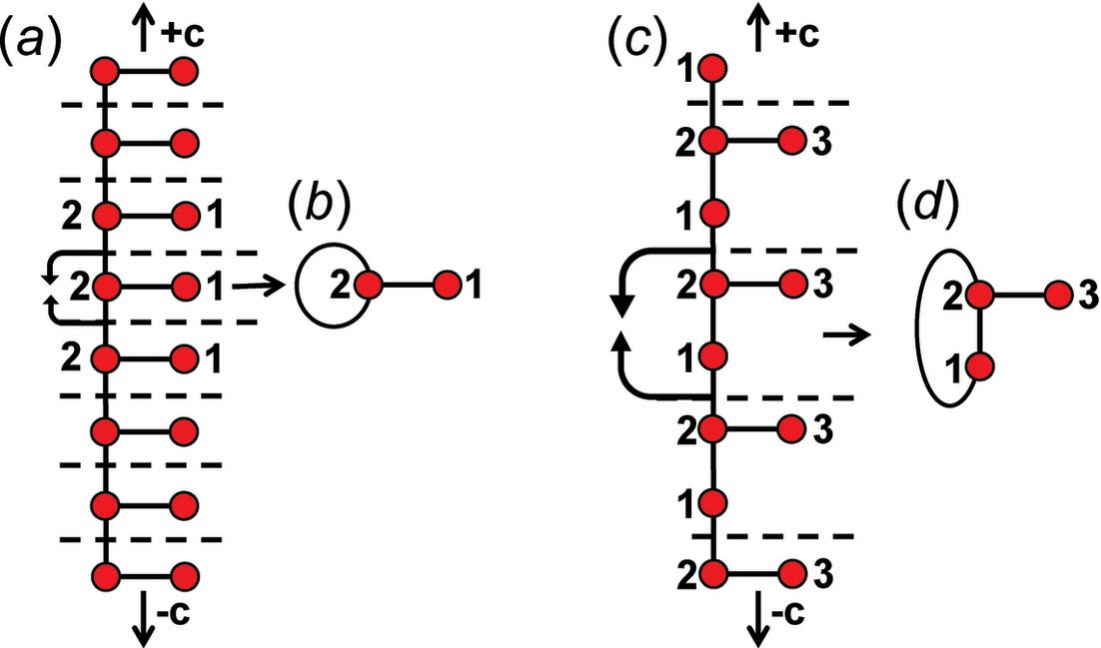
(*a*) An infinite chain graph with vertices of degree 1 and 3, in which repeat units are separated by dashed lines and edge 2–2 extends outside the repeat unit; (*b*) a finite wrapped graph in which edges that extend outside the repeat unit in (*a*) are wrapped to form a loop at vertex 2; (*c*) an infinite chain graph with vertices of degree 1, 2 and 3; (*d*) a finite wrapped graph in which edges that extend outside the repeat unit in (*c*) are wrapped to form a curved edge between vertices 1 and 2. Straight black arrows indicate the infinite extension of chain graphs in the +**c** and −**c** directions. Curved black arrows in (*a*) and (*c*) indicate how edges are wrapped in (*b*) and (*d*), and curved black lines represent wrapped edges in (*b*) and (*d*). Dashed black lines show the repeat units of each chain graph.

As we are dealing with tetrahedron linkages that are (virtually) infinite in one direction, all the graphs that we consider are similarly infinite in one direction. Although infinite, these chain graphs have translational symmetry in the direction of tetrahedron polymerization, and hence we represent the chain graph by a repeat unit that is propagated by the translational symmetry of the chain (see Section 5 below). To do this, we must incorporate the translational symmetry into the finite graph of the repeat unit. We may do this by wrapping the edges that exit the repeat unit in either repeat direction (+**c** and −**c**) such that they join to vertices within the same repeat unit while maintaining the connectivity of the original chain graph (Fig. 2 ▸).

To preserve the information related to the translational symmetry of the chain graph, the wrapped edge in the wrapped finite graph must be indicated; here, a wrapped edge is always shown as a curved line and an edge that is not wrapped (does not exit the repeat unit) is shown as a straight line. There are two different possibilities: (i) in which adjacent repeat units link via edges between identical vertices; and (ii) in which adjacent repeat units link via edges between non-identical vertices. Consider case (i): the graph in Fig. 2 ▸(*a*) consists of a repeat unit containing vertex 1 of degree 1 and vertex 2 of degree 3, and vertex 2 links to translationally equivalent vertices in adjacent repeat units. We may wrap the edges that link to vertices of an adjacent repeat unit along the repeat direction such that they join and form a loop, maintaining the vertex degree 3 for vertex 2 [Fig. 2 ▸(*b*)]. A tetrahedron cannot link to itself and hence the loop must indicate that the vertex corresponding to the tetrahedron links to translationally equivalent vertices (tetrahedra) in both the +**c** and −**c** directions. Consider case (ii): the graph in Fig. 2 ▸(*c*) consists of a repeat unit containing vertex 3 of degree 1, vertex 1 of degree 2 and vertex 2 of degree 3. Vertex 2 links to vertex 1 of the adjacent repeat unit in the +**c** direction and vertex 1 links to vertex 2 of the adjacent repeat unit in the −**c** direction, and hence wrapping produces an additional edge [shown as a curved line in Fig. 2 ▸(*d*)] between vertices 1 and 2 in the repeat unit.

### 4.2. The direction of wrapped edges

**Figure 3:**
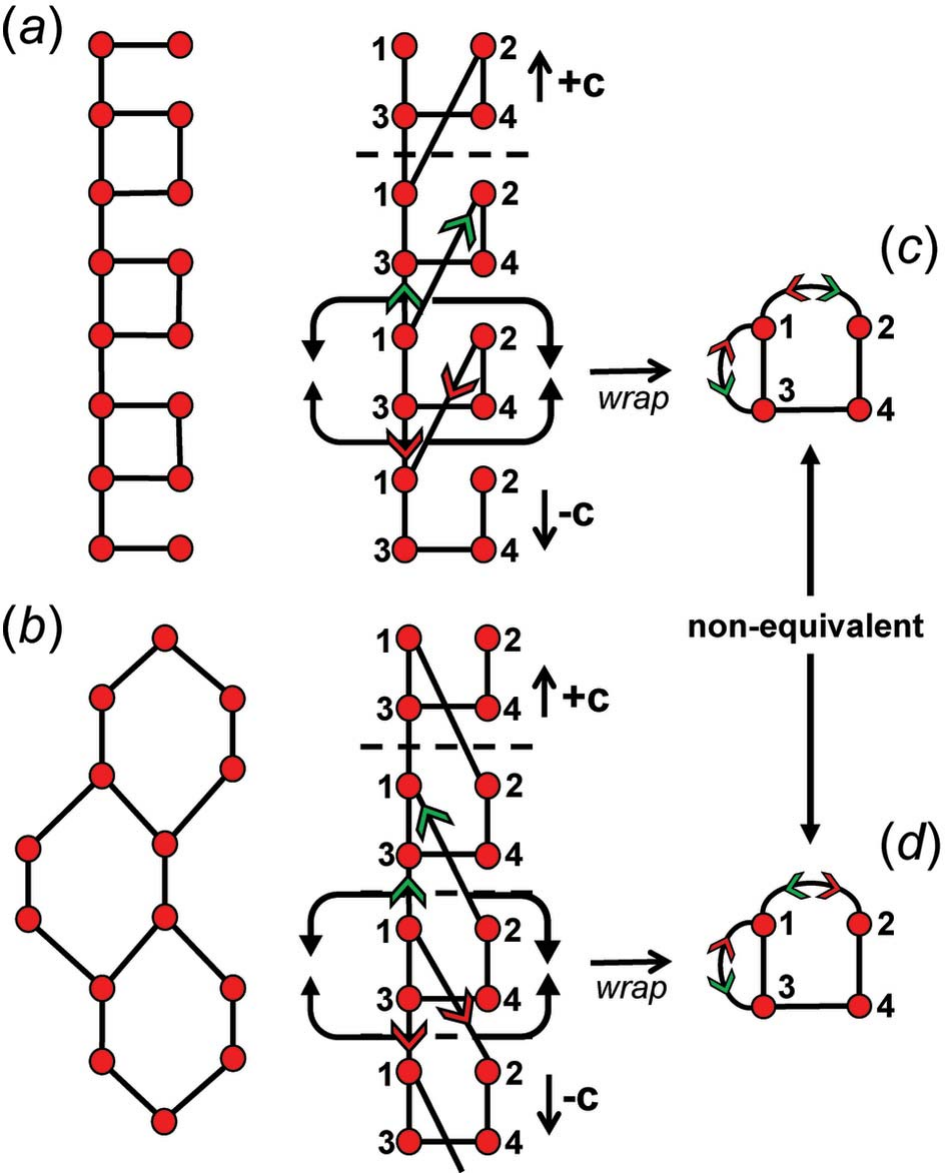
Two topologically distinct (non-isomorphic) chain graphs: (*a*) a chain graph of four-membered rings (squares), and (*b*) a chain graph of six-membered rings (hexagons) in which edges that extend outside the repeat unit in the +**c** and −**c** directions are shown with green and red arrows, respectively. (*c*), (*d*) The wrapped graphical representations of (*a*) and (*b*) are topologically identical (isomorphic) and differentiated by indicating the direction of wrapped (curved) edges with red and green arrows. Straight black arrows in (*a*) and (*b*) indicate the infinite extension of chain graphs in the +**c** and −**c** directions. Curved black arrows indicate how edges are wrapped in (*a*) and (*b*), and curved black lines represent wrapped edges in (*c*) and (*d*). Dashed black lines show the repeat units of each chain graph.

In most cases, all information related to the translational symmetry of a given chain graph can be represented using a finite graph by wrapping and denoting wrapped edges as curved lines; however, this is not always the case. Consider the infinite chain graphs shown in Figs. 3 ▸(*a*) and 3 ▸(*b*); they are topologically different from each other (non-isomorphic) as one contains squares [Fig. 3 ▸(*a*)] and the other contains hexagons [Fig. 3 ▸(*b*)]. However, wrapping each of these infinite graphs produces topologically identical (isomorphic) finite wrapped graphs [Figs. 3 ▸(*c*), 3 ▸(*d*)], in which there is one wrapped edge between vertices 1 and 2, and one wrapped edge between vertices 1 and 3. This example suggests that to completely describe a specific chain graph via wrapping, we need to specify (i) which edges are wrapped (curved lines), and (ii) the direction in which those edges exit the repeat unit. In Fig. 3 ▸(*a*), edges extend from vertex 1 out of the repeat unit to vertex 2 in the +**c** direction, and from vertex 1 out of the repeat unit to vertex 3 in the +**c** direction. This is shown in the corresponding wrapped graph [Fig. 3 ▸(*c*)] by adding a green arrow to each wrapped edge to indicate linkage in the +**c** direction. In Fig. 3 ▸(*a*), edges extend from vertices 2 and 3 out of the repeat unit to vertex 1 in the −**c** direction. This is shown in the corresponding wrapped graph [Fig. 3 ▸(*c*)] by adding a red arrow to each wrapped edge to indicate linkage in the −**c** direction. In Fig. 3 ▸(*b*), an edge extends from vertex 1 to vertex 2 in the −**c** direction and from vertex 2 to vertex 1 in the +**c** direction, and these directions are shown by red and green arrows, respectively, in the corresponding wrapped graph [Fig. 3 ▸(*d*)]. It is apparent that the difference in the direction of wrapped edges linking vertices 1 and 2 is why the infinite graphs in Figs. 3 ▸(*a*) and 3 ▸(*b*) are topologically different. The direction of any wrapped edge must be indicated to ensure that all information contained within the chain graph is represented in the finite wrapped graph.

## 5. Geometrical and topological (graphical) representations of chains

**Figure 4:**
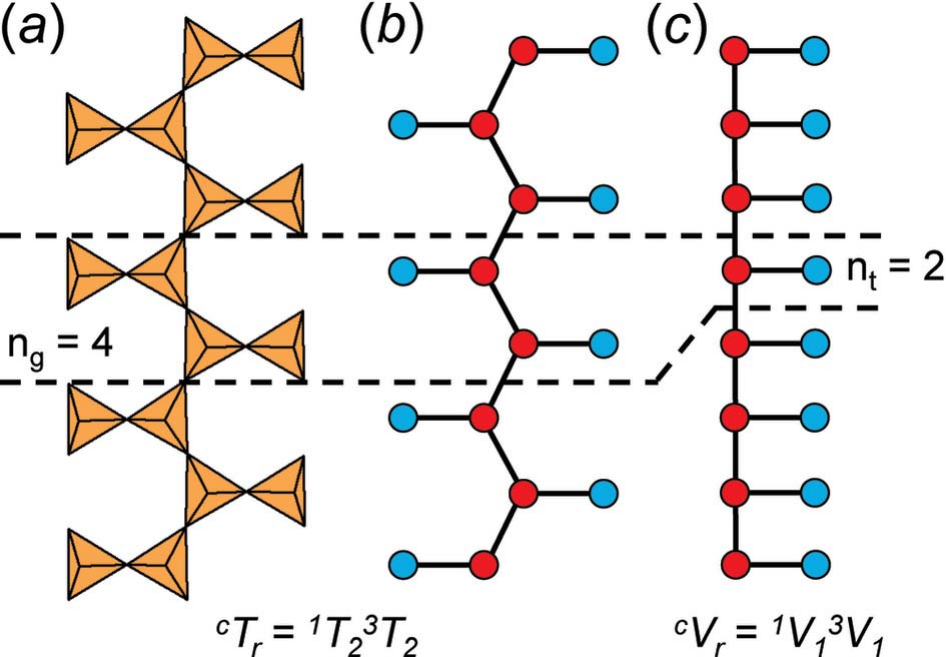
(*a*) Tetrahedron, (*b*) ball-and-stick and (*c*) graphical representations of the chain in astrophyllite-supergroup minerals viewed orthogonally to the *c* axis. Each tetrahedron in (*a*) is represented by a point (ball) in (*b*) and a vertex in (*c*), and all linkages between tetrahedra in (*a*) are represented by lines (sticks) in (*b*) and edges in (*c*) that connect each ball or vertex. Red and blue balls (points) represent 3- and 1-connected vertices, respectively. Dashed black lines show the ^1^ *T*_2_^3^ *T*_2_ geometrical repeat unit (*n*_g_) in (*a*) and (*b*), and the ^1^ *V*_1_^3^ *V*_1_ topological repeat unit (*n*_t_) in (*c*).

Chain-, ribbon- and tube-silicate structures may be shown in three representations:

(i) *Tetrahedron*: (*T*O_4_)^*n*−^ groups are shown as tetrahedra and the original chain geometry is preserved [Fig. 4 ▸(*a*)].

(ii) *Ball-and-stick*: representations in which tetrahedra are represented by points and links between tetrahedra are represented by lines, and the original chain geometry is preserved [Fig. 4 ▸(*b*)].

(iii) *Graphs*: representations in which the chain is reduced to a graph in which tetrahedra are represented by vertices and linkages between tetrahedra are represented by edges, and the original chain geometry is not preserved [Fig. 4 ▸(*c*)].

The geometry of a given chain, ribbon and/or tube may be represented by identifying a single geometrical repeat unit [with *n*_g_ tetrahedra, Fig. 4 ▸(*a*)] as in representations (i) and (ii), and the topology of a given chain, ribbon and/or tube may be represented by identifying a single topological repeat unit [with *n*_t_ vertices, Fig. 4 ▸(*c*)] as in representation (iii). The topological and geometrical repeat units can be linked infinitely by translation in a single direction to produce the original infinite polyhedron, ball-and-stick and graphical representations, respectively.

### 5.1. The geometrical repeat unit

In the tetrahedron and ball-and-stick representations, we assign a geometrical repeat unit in which the geometry of the chain (lengths and angles of linkages between tetrahedra as observed in the mineral or synthetic compound) is preserved. The geometrical repeat unit contains the minimum number of tetrahedra (*n*_g_) required to generate the chain through translation operations. It is necessary to specify the numbers of 1-, 2-, 3- and 4-connected tetrahedra that comprise *n*_g_ to describe the geometrical repeat unit of a chain. To do this, we denote a tetrahedron by *T*, its connectivity by the superscript *c* (*c* = 1–4) and the number of such tetrahedra in the geometrical repeat unit by the subscript *r*. The expression *^c^T_r_* = ^1^ *T_r_* ^2^ *T_r_* ^3^ *T_r_* ^4^ *T_r_* represents all possible connectivities of tetrahedra in the repeat unit of a chain, and the number of terms with *r* ≠ 0 in the *^c^T_r_* expression is defined as its rank. The majority of chains observed in minerals and synthetic compounds contain only 2- and 3-connected vertices (*i.e. ^c^T_r_* has a rank of 2 as *r* = 0 for ^1^ *T_r_* and ^4^ *T_r_*), but some chains also contain 1- and/or 4-connected vertices. As an example, consider the tetrahedron [Fig. 4 ▸(*a*)] and ball-and-stick [Fig. 4 ▸(*b*)] representations of the [Si_4_O_12_]^8−^ chain in the astrophyllite-supergroup minerals (Sokolova *et al.*, 2017 ▸). The ball-and-stick representation shows two types of vertices: (i) 3-connected (red circles), and (ii) 1-connected (blue circles) [Fig. 4 ▸(*b*)]. The geometrical repeat unit contains two of each of these types of ball (*n*_g_ = 4), and the ^c^ *T*_r_ expression for the astrophyllite-type chain is written as *^c^T_r_* = ^1^ *T*_2_^2^ *T*_0_^3^ *T*_2_^4^ *T*_0_ = ^1^ *T*_2_^3^ *T*_2_ (rank = 2) (Day & Hawthorne, 2020 ▸).

### 5.2. The topological repeat unit

**Figure 5:**
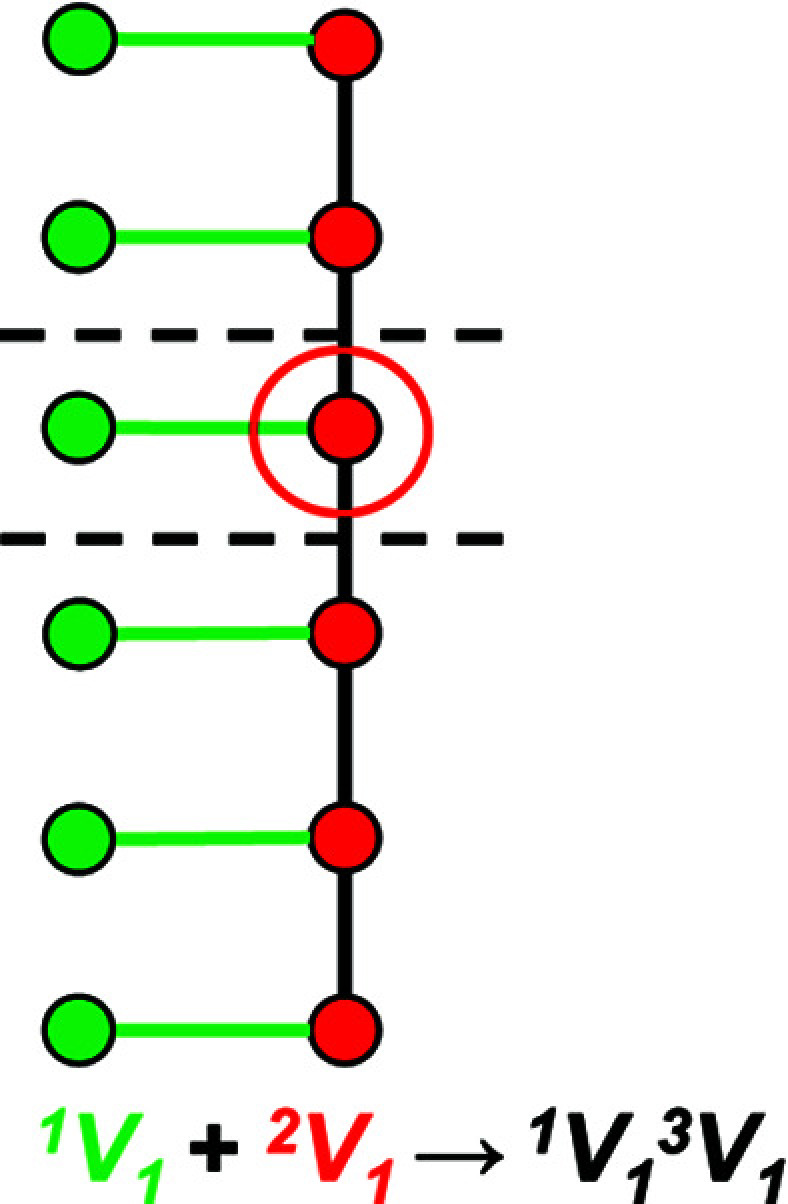
A chain graph with vertex connectivity ^2^ *V*_1_ (red vertices and black edges) to which ^1^ *V*_1_ vertices (green vertices and edges) have been added, forming a chain graph with vertex connectivity ^1^ *V*_1_^3^ *V*_1_. The red circle indicates a vertex that has been changed from degree 2 to 3 by the connection with a 1-connected vertex. Dashed black lines show the repeat unit of the chain graph.

Graphical representations of chains, ribbons and tubes have a topological repeat unit in which only the topological properties are represented. The topological repeat unit contains the minimum number of vertices (*n*_t_) required to generate the chain through infinite linkage in a single direction by translation. By analogy with the geometrical repeat unit, we may describe the topological repeat unit using the expression *^c^V_r_* = ^1^ *V_r_* ^2^ *V_r_* ^3^ *V_r_* ^4^ *V_r_* where *^c^V_r_* denotes the connectivity of vertices (*V*) rather than tetrahedra (*T*). In many chains, tetrahedra are topologically identical but geometrically distinct. This often results in chains with geometrical and topological repeat units that contain different numbers of tetrahedra and vertices. Figs. 4 ▸(*a*), 4 ▸(*b*) show the tetrahedron and ball-and-stick representations of the chain in the astrophyllite-supergroup minerals, where *^c^T_r_* = ^1^ *T*_2_^3^ *T*_2_ is the connectivity of the tetrahedra in the geometrical repeat unit. The graphical representation of the same chain [Fig. 4 ▸(*c*)] has a topological repeat unit that contains only two vertices, as the different directions of branching of the 1-connected vertices in Fig. 4 ▸(*b*) do not affect the topology of the linkage. It follows that we may describe the topological repeat unit in the astrophyllite-supergroup minerals as *^c^V_r_* = ^1^ *V*_1_^3^ *V*_1_. Hence the vertex connectivity, *^c^V_r_*, for a topological repeat unit may be derived by multiplying the values of *r* in the respective *^c^T_r_* expression by *n*_t_/*n*_g_. Note that any graph with an odd number of vertices of odd degree is not possible. For example, the graph with vertex connectivity ^1^ *V*_1_^2^ *V*_1_ has an odd number of vertices of degree 1. If a vertex of degree 1 is connected to a chain with vertex connectivity ^2^ *V*_1_, a vertex of degree 3 is created and the chain no longer has the vertex connectivity ^1^ *V*_1_^2^ *V*_1_ (Fig. 5 ▸); hence this chain is not possible. Instead, a chain with vertex connectivity ^1^ *V*_1_^3^ *V*_1_ is created (Fig. 5 ▸) which has an odd number (one) of vertices of degree 1 and 3 but the total number of vertices with an odd degree (two) is even and therefore a chain graph with vertex connectivity ^1^ *V*_1_^3^ *V*_1_ is possible and is shown in Fig. 5 ▸.

## 6. Adjacency matrices

**Figure 6:**
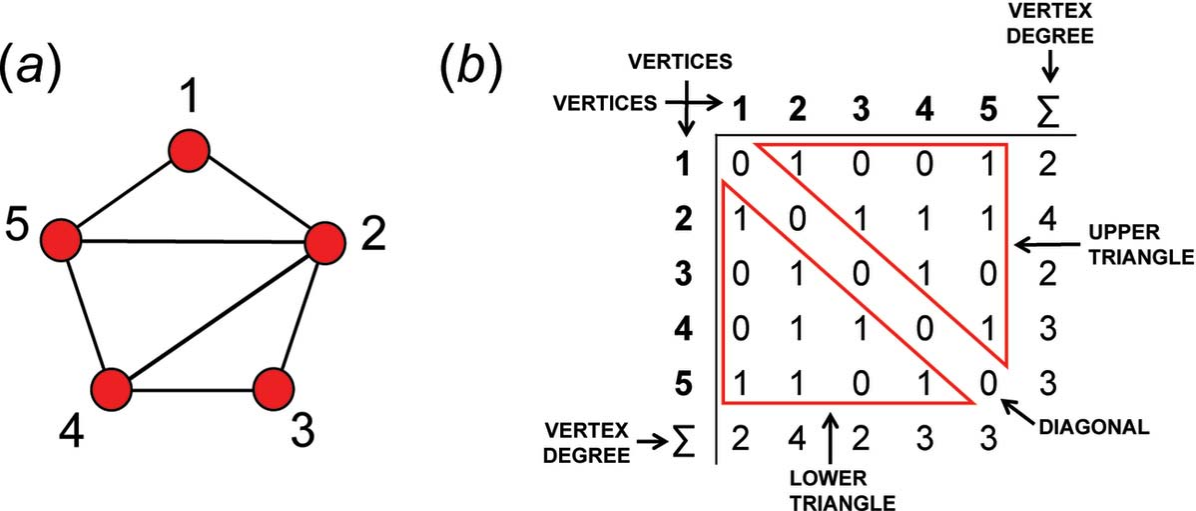
(*a*) A finite graph with vertex connectivity ^2^ *V*_2_^3^ *V*_2_^4^ *V*_1_ and (*b*) the corresponding adjacency matrix in which each row (or column) represents a vertex and the sums of rows and columns equal the degree of the respective vertices. Cells of the upper and lower triangle are outlined in red and separated by diagonal cells.

A finite graph [Fig. 6 ▸(*a*)] may be represented numerically as a matrix [Fig. 6 ▸(*b*)]. The vertices are listed as shown in Fig. 6 ▸(*b*): each column and row of the matrix is associated with a specific vertex, and the corresponding matrix entries denote whether (positive) or not (zero) two vertices are adjacent, that is joined by an edge. The matrix elements denote the edge set, and this matrix is called an adjacency matrix. Conventionally, the diagonal entries of the matrix are zeros, and the edge set of the graph is denoted by the upper (or lower) triangle of matrix entries. The degree of a vertex is the number of edges connected to that vertex, and the sums of the entries in each row and column of the matrix correspond to the degrees of the vertices associated with that row or column. All adjacency matrices for graphs with no directed edges must be symmetrical about the diagonal [Fig. 6 ▸(*b*)]. As discussed above, the maximum degree of a vertex is 4 (*e.g.* vertex 2, Fig. 6 ▸). Wrapped graphs may contain two vertices linked by two edges where one or both of such edges is wrapped (curved).

**Figure 7:**
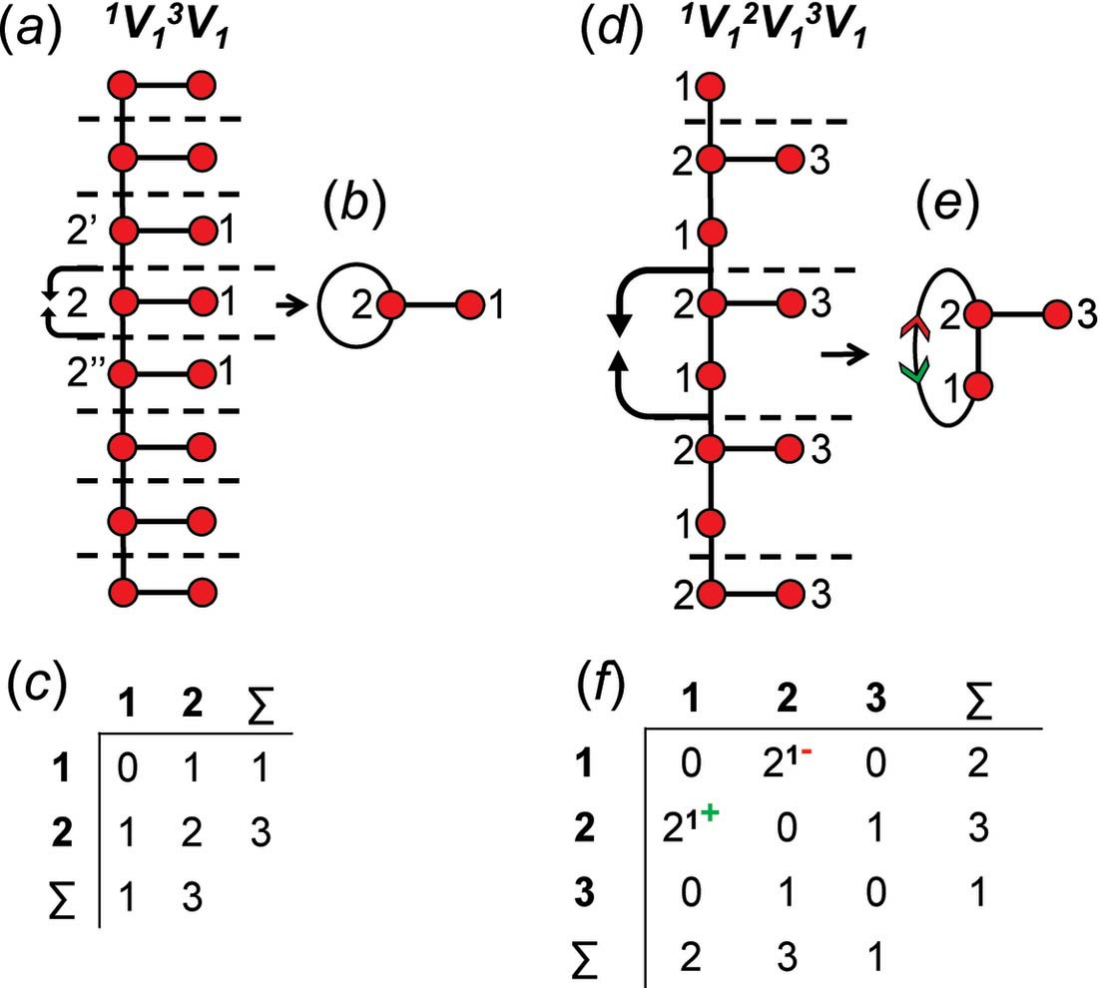
(*a*) A chain graph with vertex connectivity ^1^ *V*_1_^3^ *V*_1_, (*b*) the corresponding wrapped graph and (*c*) the corresponding adjacency matrix. (*d*) A chain graph with vertex connectivity ^1^ *V*_1_^2^ *V*_1_^3^ *V*_1_, (*e*) the corresponding wrapped graph and (*f*) the corresponding adjacency matrix. Curved black arrows indicate how edges are wrapped in (*a*) and (*d*), and curved black lines represent wrapped edges in (*b*) and (*e*). Coloured arrows in (*b*) are not required as the loop on vertex 2 represents linkage to equivalent vertices (2′ and 2′′) in the +**c** and −**c** directions. Green and red arrows in (*e*) and matrix-element superscripts in (*f*) indicate the number and direction of wrapped edges. Dashed black lines show the repeat units of each chain graph.

How do we represent information on wrapping in the adjacency matrix? In the graph in Fig. 7 ▸(*a*), vertex 2 links to vertex 2′ in the +**c** direction and to vertex 2′′ in the −**c** direction, and in the corresponding wrapped graph [Fig. 7 ▸(*b*)] there are two edges incident at vertex 2 which form a loop. We may represent this loop by coding the diagonal entry 2,2 in the adjacency matrix [Fig. 7 ▸(*c*)] as 2, and the degree of vertex 2 is 3 in the chain graph [Fig. 7 ▸(*a*)], the wrapped graph [Fig. 7 ▸(*b*)] and the corresponding adjacency matrix [Fig. 7 ▸(*c*)]. The direction of the looped edge in Fig. 7 ▸(*c*) need not be indicated by a green or red arrow as any looped edge exits the repeat unit in both the +**c** and −**c** directions. In Fig. 7 ▸(*d*), there are two linkages between vertices 1 and 2, one of which occurs within the repeat unit [the straight edge in Fig. 7 ▸(*e*)] and one of which links to vertices of adjacent repeat units in both +**c** and −**c** directions [the curved edge in Fig. 7 ▸(*e*)]. To represent the directions of linkage to adjacent repeat units, the relevant matrix entries are given superscripts which indicate the number and direction of the linkages to adjacent repeat units. Thus entry 1,2 in the adjacency matrix is coded as 2^1−^ [Fig. 7 ▸(*f*)] as one of the two 1–2 edges extends from vertex 1 out of the repeat unit to vertex 2 in the −**c** direction, and the other 1–2 edge occurs within the repeat unit of the infinite chain graph and hence has no superscript. Similarly, entry 2,1 is coded as 2^1+^ [Fig. 7 ▸(*f*)] as one of the two 2–1 edges extends from vertex 2 out of the repeat unit to vertex 1 in the +**c** direction.

**Figure 8:**
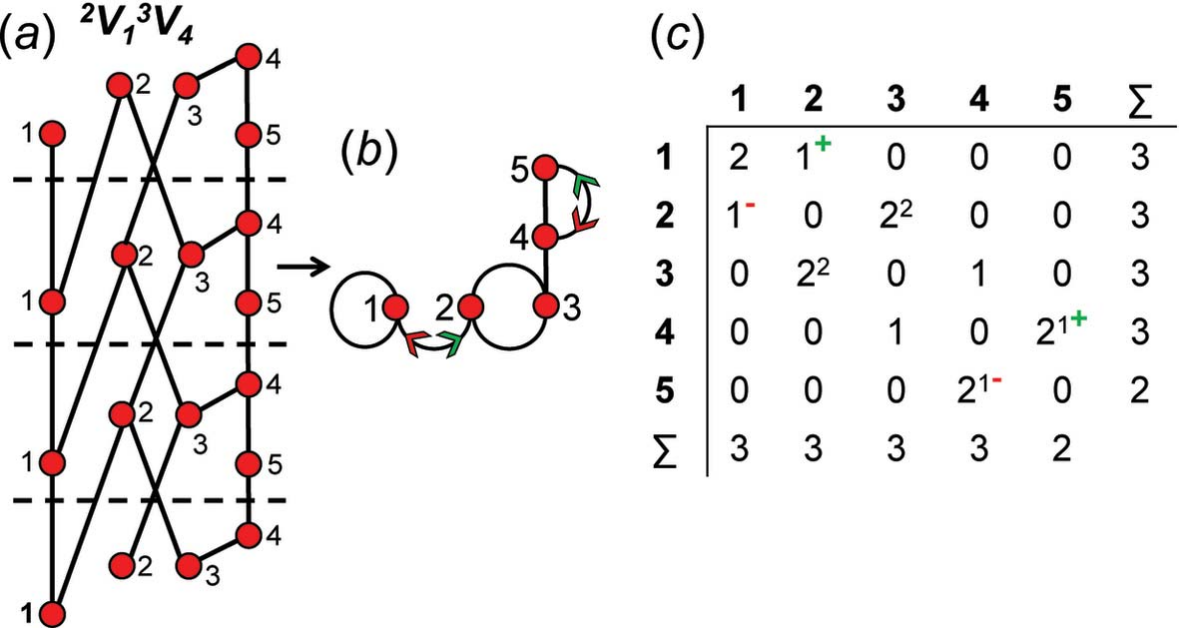
(*a*) A chain graph with vertex connectivity ^2^ *V*_1_^3^ *V*_4_, (*b*) the corresponding wrapped graph and (*c*) the corresponding adjacency matrix. Curved black lines in (*b*) represent wrapped edges in (*a*); green and red arrows in (*b*) and matrix-element superscripts in (*c*) indicate the number and direction of wrapped edges. Note that it is not necessary to indicate the direction of the wrapped edges linking vertices 2 and 3. Dashed black lines show the repeat units of each chain graph.

A more complicated chain graph with vertex connectivity ^2^ *V*_1_^3^ *V*_4_ is shown in Fig. 8 ▸(*a*) and the corresponding wrapped graph and its adjacency matrix are shown in Figs. 8 ▸(*b*) and 8 ▸(*c*), respectively. Entries 1,2 and 2,1 are coded as 1^+^ and 1^−^ to indicate the numbers and directions of the external linkages [Fig. 8 ▸(*c*)]. Vertex 2 links to vertex 3 in both the +**c** and −**c** directions, and similarly vertex 3 links to vertex 2 in both the +**c** and −**c** directions [Fig. 8 ▸(*a*)]. Thus entries 2,3 and 3,2 do not need to indicate directions as the superscript 2^2^ shows that there are two wrapped edges, and these must occur in opposite directions. Vertex 1 links to equivalent vertices in adjacent repeat units and the entry 1,1 is coded 2 [Fig. 8 ▸(*c*)]. There are two edges linking vertices 4 and 5, one of which is wrapped [Fig. 8 ▸(*b*)] and the entries 4,5 and 5,4 are coded 2^1+^ and 2^1−^, respectively [Fig. 8 ▸(*c*)]. When describing a wrapped edge, we need only to refer to the matrix element in the upper triangle (or lower triangle) instead of both as the repeat unit of any chain graph is translationally equivalent to adjacent repeat units such that if *n* edges link to vertices of the adjacent repeat unit in the +**c** direction, *n* edges must also link to vertices of the adjacent repeat unit in the −**c** direction.

## 7. Generation of all non-isomorphic chain graphs

We have developed finite representations of infinite chains using wrapped edges. Now we wish to invert this process and generate all possible non-isomorphic graphs that correspond to infinite chains. A graph consists of two sets, a vertex set and an edge set (Section 4), each of which may include at least one subset. We may treat the generation of all possible non-isomorphic graphs as a two-component process. First, we generate all possible vertex sets and vertex subsets consistent with chains, ribbons and tubes (Section 2), and second, we generate all possible edge sets and edge subsets consistent with each vertex set and vertex subset already defined.

## 8. Generation of vertex sets for chain graphs

**Figure 9:**
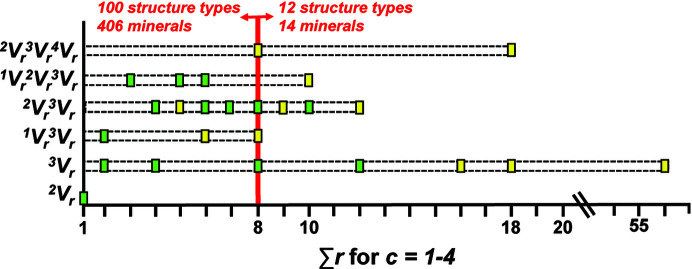
The vertex connectivity, *^c^V_r_*, of observed chain, ribbon and tube topologies as a function of ∑*r* for *c* = 1–4. Most observed topologies have ∑*r* ≤ 8; hence the boundary limit for graph generation has been set to ∑*r* = 8 and is shown by the red line. Squares indicate the *^c^V_r_* expression and ∑*r* for one (yellow) and more than one (green) observed topology. [Data from Day & Hawthorne (2020 ▸).]

Each vertex is characterized by its degree, and we express the degree structure of a vertex set using the vertex connectivity *^c^V_r_* ( = ^1^ *V*_*r*_^2^ *V_r_* ^3^ *V_r_* ^4^ *V_r_*). Thus, we may use *^c^V_r_* to generate all possible vertex sets by writing all possible *^c^V_r_* expressions for chain graphs with vertex degrees *c* = 1, 2, 3 and 4 where *r* = 1 to ∞ by sequentially increasing the values of *c* and *r*. Obviously we need to limit the value of *r* for practical reasons, and we may justify this by observing the distribution of vertex sets in observed chain, ribbon and tube minerals (Fig. 9 ▸). As shown by Day & Hawthorne (2020 ▸), most structures have ∑*r* ≤ 8 and hence we will use 8 as the boundary limit for ∑*r*. We will order the ^1^ *V_r_* ^2^ *V_r_* ^3^ *V_r_* ^4^ *V_r_* expressions in terms of increasing rank (*i.e.* the number of individual *^c^V_r_* values). For a given rank, we sequentially increase the value of *c* for *r* = 1 to 8; Table 1 ▸ shows the *^c^V_r_* expressions produced in this way. For a rank of 1, where *c* = 1, a chain graph is not possible: ^1^ *V*_1_ corresponds to a single vertex; ^1^ *V*_2_ corresponds to a [*T*_2_O_7_] dimer, and no further linkages are possible without changing the value of *c* (Table 1 ▸). Thus, the simplest possible chain arrangement has the vertex connectivity ^2^ *V*_1_, followed by ^2^ *V*_2_, ^2^ *V*_3_, ^2^ *V*_4_ *etc*. For higher ranks, we order *^c^V_r_* first in terms of *c* and then in terms of *r*, hence for a rank of 2: ^2^ *V_r_* ^3^ *V_r_*, we have ^2^ *V*_1_^3^ *V_r_*, ^2^ *V*_2_^3^ *V_r_*, ^2^ *V*_3_^3^ *V* *_r_ etc*.

### 8.1. Determining vertex subsets using characteristic polynomial equations

*^c^V_r_* describes only the degree of the vertices in the repeat unit of a chain graph. Vertices that are isomorphic (symmetrically equivalent) (i) must have the same degree, and (ii) the degrees of neighbouring (next-nearest, next-next-nearest *etc*.) vertices must be identical. Thus, the vertex set of any graph may be divided into subsets of isomorphic vertices.

The characteristic polynomial, *p*(λ), is an equation that describes a square matrix (A) whose eigenvalues are the roots of *p*(λ) and whose trace and determinant are coefficients of the polynomial. *I* is the identity matrix of [A] and λ is a scalar multiple that is an eigenvalue of (A) if (A − λ*I*) = 0. Each characteristic polynomial equation has *n* solutions and therefore *n* eigenvalues (or roots) where *n* equals the dimension of (A). We may write the characteristic polynomial as follows:

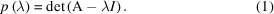




We can determine which vertices of a given graph belong to the same vertex subset by deleting one vertex in turn and calculating the characteristic polynomial equation of the adjacency matrices of the resultant graphs which are referred to as reduced graphs and reduced adjacency matrices. If the characteristic polynomial equations of two reduced graphs are identical, the eigenvalues of the two reduced matrices are identical and hence the two vertices removed from the original graph are isomorphic and belong to the same vertex subset. It follows that one must calculate *n* characteristic polynomial equations for a given *n* × *n* matrix. The characteristic polynomial equations were calculated for all matrices corresponding to each *^c^V_r_* expression using the *MatLabR2019b* code in Appendix *B* (see the supporting information).

**Figure 10:**
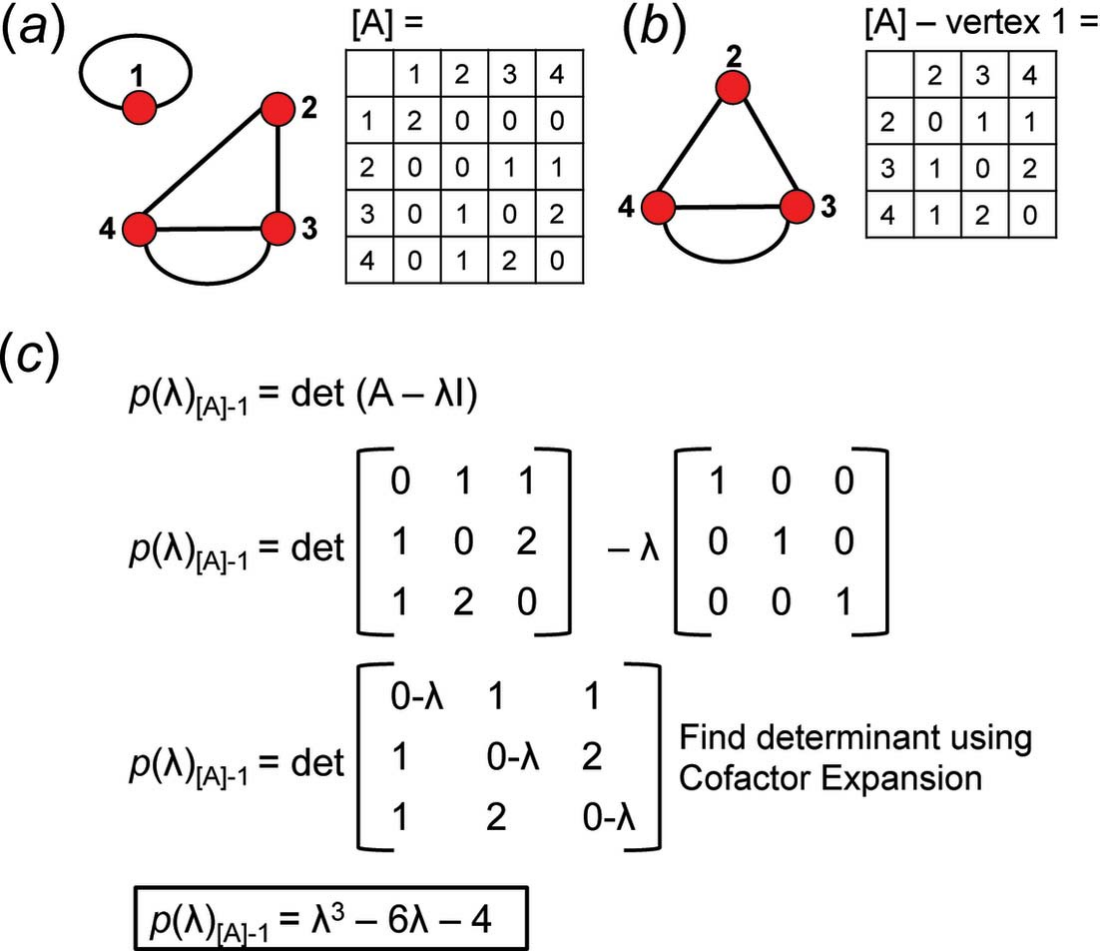
(*a*) A graph with vertex connectivity ^2^ *V*_2_^3^ *V*_2_ and its corresponding adjacency matrix ([A]), (*b*) the reduced graph produced by removing vertex 1 from the graph in (*a*) and the corresponding reduced adjacency matrix ([A]−1), and (*c*) the characteristic polynomial equation for the reduced adjacency matrix in (*b*): [*p*(λ)_[A]−1_].

**Figure 11:**
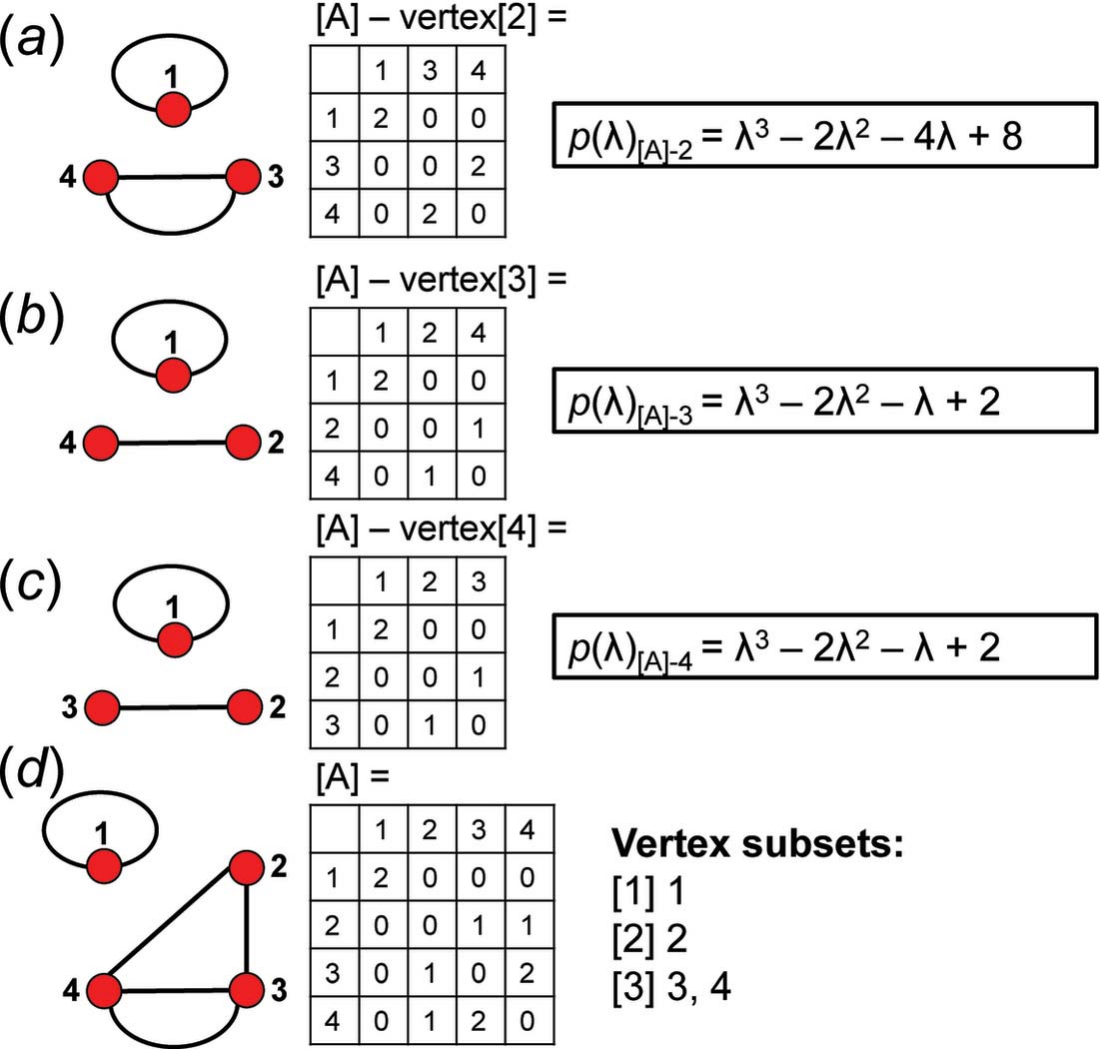
The reduced graphs, corresponding reduced adjacency matrices, and characteristic polynomial equations after the following vertices have been removed from the graph in Fig. 10 ▸(*a*): (*a*) vertex 2, *p*(λ)_[A]−2_, (*b*) vertex 3, *p*(λ)_[A]−3_ and (*c*) vertex 4, *p*(λ)_[A]−4_. (*d*) The original graph, adjacency matrix and the vertex subsets determined using characteristic polynomial equations.

An example of how this calculation is done for a 4 × 4 adjacency matrix is shown in Figs. 10 ▸ and 11 ▸. In Fig. 10 ▸(*a*), vertices 1 and 2 have degree 2 and therefore must belong to different vertex subsets than vertices 3 and 4 which have degree 3. However, one cannot assume vertices of the same degree belong to the same vertex subset until they are confirmed to be isomorphic using characteristic polynomial equations. We begin by removing vertex 1 from the graph and matrix ([A]) shown in Fig. 10 ▸(*a*). The reduced graph and reduced adjacency matrix, [A] − [1], are shown in Fig. 10 ▸(*b*) and the characteristic polynomial equation of this reduced matrix, labelled (λ)_([A]−1)_, is calculated as shown in Fig. 10 ▸(*c*) using the formula above. The reduced matrices after vertices 2, 3 and 4 are removed [labelled (λ)_([A]−2)_, *p*(λ)_([A]−3)_ and *p*(λ)_([A]−4)_, respectively] are shown in Figs. 11 ▸(*a*), 11 ▸(*b*) and 11 ▸(*c*). Here, (λ)_([A]−3)_ and *p*(λ)_([A]−4)_ are identical and hence vertices 3 and 4 are isomorphic and belong to the same vertex subset [Fig. 11 ▸(*d*)]; *p*(λ)_([A]−1)_ and *p*(λ)_([A]−2)_ are different and vertices 1 and 2 are non-isomorphic and belong to different vertex subsets [Fig. 11 ▸(*d*)]. Removal of vertices 3 and 4 in turn results in the same reduced graph and reduced adjacency matrix [Figs. 11 ▸(*b*) and 11 ▸(*c*)].

If the characteristic polynomial equation of an *n* × *n* adjacency matrix is the same as the characteristic polynomial equation of another *n* × *n* adjacency matrix, these matrices (and their corresponding graphs) are isomorphic.

This method is laborious and becomes impractical for larger matrices (graphs with many vertices). For larger matrices, it is more practical to use eigenvector centrality (EVC) heuristics (Meghanathan, 2015 ▸). The EVC of a given vertex is a measure of the degree of that vertex and the degrees of neighbouring vertices. If the EVC values of the vertices of two graphs are not identical, the graphs are non-isomorphic; if the EVC values of the vertices of the two graphs are identical, the graphs are possibly isomorphic. Thus, the EVC method can show that two graphs are non-isomorphic. It cannot definitively show that graphs are isomorphic, although we are not aware of any example of two isomorphic graphs with different EVC values.

## 9. Derivation of matrix-element combinations and generation of edge sets and edge subsets

In Sections 8 and 8.1, we proposed a method for generating all possible *^c^V_r_* expressions (vertex sets) and detecting isomorphism amongst vertices of a given matrix or matrices using characteristic polynomial equations. Now we may begin to derive every distinct adjacency matrix and all possible non-isomorphic finite graphs that correspond to such matrices for all possible *^c^V_r_* expressions (up to the boundary limit). To do this, all unique combinations of the matrix elements 1, 2, 2^1^ and/or 2^2^ must be derived for each *^c^V_r_*. Each matrix-element combination corresponds to one or more distinct matrices and their non-isomorphic finite graphs that are generated as described below. Thus, deriving all matrix-element combinations for a given *^c^V_r_* allows us to generate the set of all non-isomorphic finite graphs that conform to that *^c^V_r_*. Two *n* × *n* matrices are referred to as distinct if their *n* characteristic polynomial equations are not identical. The edge subsets for each of the non-isomorphic finite graphs (for a given *^c^V_r_*) can then be determined using the vertex subsets of such graphs determined using the characteristic polynomial equations as described above. One can then generate all possible non-isomorphic wrapped graphs with specific vertex connectivities, *^c^V_r_*, using the edge subsets of the finite graphs from which the wrapped graphs are generated.

### 9.1. Matrix-element combinations

For a topological repeat unit described by the vertex connectivity *^ci^V_ri_* = ^1^ *V*_*r*1_^2^ *V*_*r*2_^3^ *V*_*r*3_^4^ *V*_*r*4_, the number of edges *e* = ∑*c_i_* *r_i_*/2, *i* = 1, 4. The number of edges in the corresponding adjacency matrix *e*_A_ = *e* × 2 as each edge starts and ends on a vertex and is therefore counted twice. For a specific *^c^V_r_*, we calculate all possible combinations of the matrix elements 0, 1, 2, 2^1^, 2^2^ that sum to the number of edges in the adjacency matrix (*e*_A_) for that *^c^V_r_*. These matrix-element combinations are used to generate a set of *n*-dimensional matrices where *n* is the number of vertices in the *^c^V_r_* expression of interest, and is given by *n* = ∑*r_i_*, *i* = 1, 4. We derive matrix-element combinations that sum to *e*_A_ rather than *e* because we use the diagonal elements of each matrix to store additional information (occupied by 0 or 2) and therefore all matrix positions are considered rather than those in just the upper or lower triangle of the matrix (*i.e.* Fig. 6 ▸). Some of the possible matrix-element combinations for a given *^c^V_r_* may not be valid and need not be considered as they correspond to matrices that violate one or more of the following constraints:

(i) The matrix must be symmetric about the diagonal (*e.g.* Fig. 6 ▸) unless it contains the elements 2^1+^ or 2^1−^.

(ii) The diagonal cells of the matrix can only be occupied by 0 and/or 2, and off-diagonal cells cannot be occupied by 2.

If the matrix accords with these constraints, the associated matrix-element combination is valid and will give one or more finite graphs that correspond to that *^c^V_r_* expression.

**Figure 12:**
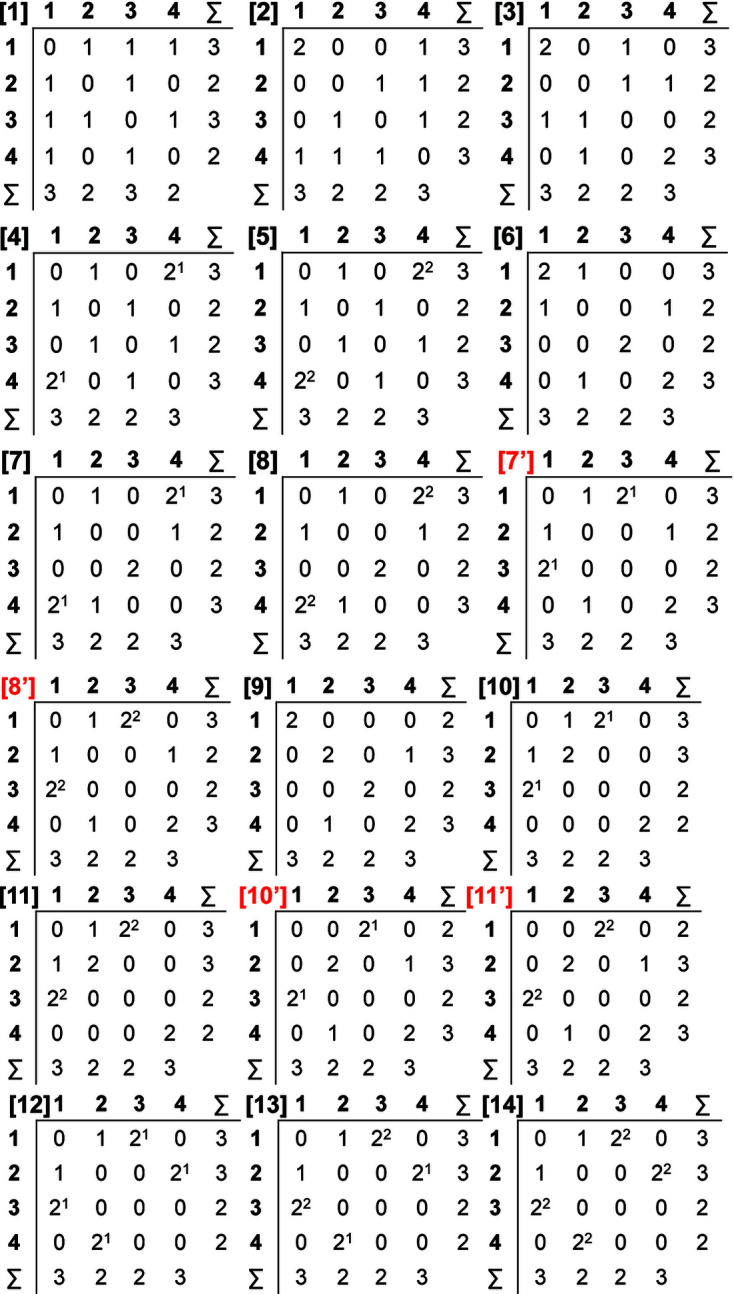
All distinct adjacency matrices that produce proto-graphs with vertex connectivity ^2^ *V*_2_^3^ *V*_2_. The matrix-element combination (4 × 1)(1 × 2)(2 × 2^1^) corresponds to matrices [7] and [7′], (4 × 1)(1 × 2)(2 × 2^2^) corresponds to matrices [8] and [8′], (2 × 1)(2 × 2)(2 × 2^1^) corresponds to matrices [10] and [10′], and (2 × 1)(2 × 2)(2 × 2^2^) corresponds to matrices [11] and [11′]. All other valid matrix-element combinations correspond to a single matrix.

**Figure 13:**
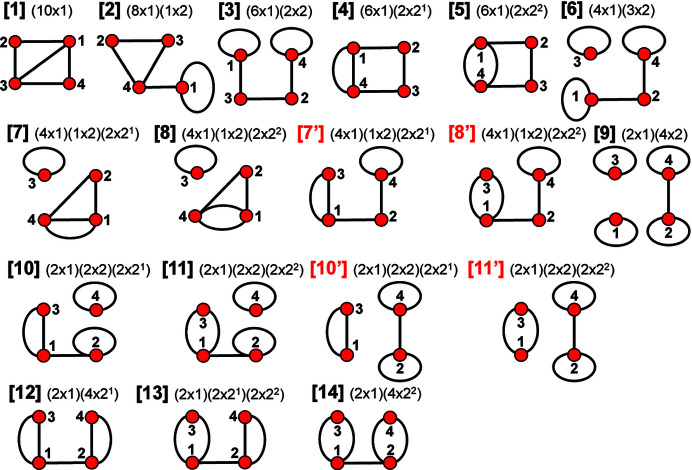
All valid matrix-element combinations and their associated proto-graphs with vertex connectivity ^2^ *V*_2_^3^ *V*_2_. The corresponding adjacency matrices are shown in Fig. 12 ▸.

The following example shows how matrix-element combinations are derived for ^2^ *V*_2_^3^ *V*_2_ and determined to be either valid or invalid. The number of vertices *n* = (2 + 2) is 4, the number of edges *e* is [(2 × 2) + (3 × 2)]/2 = 5, the number of edges in the adjacency matrix *e*_A_ is 5 × 2 = 10, the number of matrix positions is 4^2^ = 16. The possible matrix-element combinations (that sum to *e*_A_ = 10) are shown in Table 2 ▸, labelled [1]–[20]. One can then determine which of these matrix-element combinations are invalid by attempting to construct a 4 × 4 matrix using the 20 element combinations. Table 2 ▸ shows that element combinations [1]–[14] (shown in italics) generate matrices that accord with the constraints listed above and are therefore valid; these matrices are shown in Fig. 12 ▸ and the corresponding non-isomorphic finite graphs are shown in Fig. 13 ▸. Matrix-element combinations [15]–[20] (shown in bold, Table 2 ▸) force at least one 4-connected vertex (a row or column that sums to 4) and only generate matrices that violate constraint (i) and/or correspond to graphs with vertex connectivities that differ from ^2^ *V*_2_^3^ *V*_2_ and therefore need not be considered. Matrix-element combinations are written as (*m*_1_ × 1)(*m*_2_ × 2)(*m*_3_ × 2^1^)(*m*_4_ × 2^2^) where *m_i_* are the numbers of those matrix elements.

### 9.2. Multiplicity of matrix-element combinations and derivation of proto-graphs

Each valid matrix-element combination generates one or more distinct adjacency matrices, depending on the number of vertex subsets generated in each case. Each of these adjacency matrices describes one finite graph. These graphs are not chain graphs but are used to derive chain graphs by systematically unwrapping all combinations of their edges; we will call these graphs proto-graphs. For ^2^ *V*_2_^3^ *V*_2_, there are 14 valid matrix-element combinations (Table 2 ▸). However, Figs. 12 ▸ and 13 ▸ show 18 distinct matrices and non-isomorphic proto-graphs, as matrix-element combinations [7], [8], [10] and [11] each correspond to one additional distinct matrix, labelled [7′], [8′], [10′] and [11′], that each generate an additional non-isomorphic proto-graph (Fig. 13 ▸). For a given *^c^V_r_*, each valid matrix-element combination corresponds to at least one non-isomorphic proto-graph. We may determine how many non-isomorphic proto-graphs correspond to each valid matrix-element combination using the following method.

**Figure 14:**
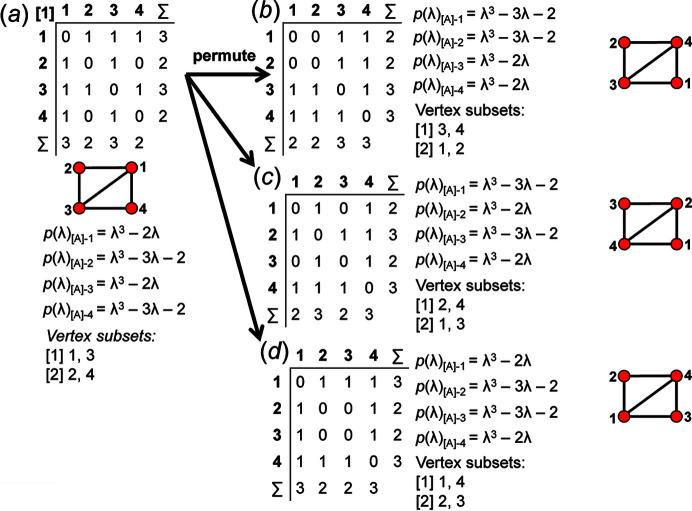
For vertex connectivity ^2^ *V*_2_^3^ *V*_2_, (*a*) the adjacency matrix [1] for the matrix-element combination (10 × 1), the corresponding proto-graph, the characteristic polynomial equations of the reduced proto-graphs and the resulting vertex subsets; (*b*), (*c*) and (*d*) three permutated versions of this adjacency matrix and proto-graph. The characteristic polynomial equations of the reduced adjacency matrices are identical, confirming that these adjacency matrices correspond to isomorphic proto-graphs.

First, we permute the positions of the matrix elements (not just the rows and columns) in one of the matrices that correspond to the valid matrix-element combinations for the specific *^c^V_r_* of interest. For vertex connectivity ^2^ *V*_2_^3^ *V*_2_, there are 14 valid matrix-element combinations (Table 2 ▸), and one permutes the position of the matrix elements in the 14 associated adjacency matrices (Fig. 12 ▸). If any of the valid permuted matrices have non-identical vertex subsets (any vertices associated with different characteristic polynomial equations), such matrices are distinct from the original matrix and will produce an additional non-isomorphic proto-graph. We derive all valid permutations of a given matrix and extract all unique permutations (those with different vertex subsets) using the *MatLabR2019b* code in Appendix *B* (supporting information). Fig. 14 ▸(*a*) shows matrix [1] [matrix-element combination (10 × 1)] (Fig. 12 ▸) and the corresponding proto-graph [1] (Fig. 13 ▸). In Figs. 14 ▸(*b*), 14 ▸(*c*) and 14 ▸(*d*), three permuted versions of the original matrix [1] [Fig. 14 ▸(*a*)] are shown along with the corresponding proto-graphs and the vertex subsets. The vertex subsets for each of the permuted matrices are the same and all proto-graphs are isomorphic as the characteristic polynomial equations for reduced matrices where a vertex of subset [1] has been removed are identical and the characteristic polynomial equations for reduced matrices where a vertex of subset [2] has been removed are identical (Fig. 14 ▸). Labels may be interchanged between vertices of the same subset as such vertices are isomorphic [*e.g.* compare Figs. 14 ▸(*a*) and 14 ▸(*c*)].

**Figure 15:**
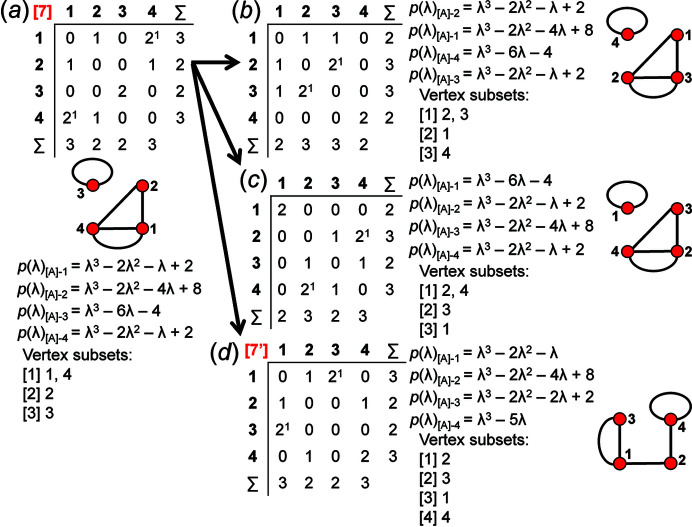
For vertex connectivity ^2^ *V*_2_^3^ *V*_2_, (*a*) adjacency matrix [7] for the matrix-element combination (4 × 1)(1 × 2)(2 × 2^1^), the corresponding proto-graph, the characteristic polynomial equations of the reduced proto-graphs and the resulting vertex subsets. (*b*), (*c*), (*d*) Three permutated versions of this adjacency matrix and proto-graph, and the characteristic polynomial equations of the reduced adjacency matrices. The characteristic polynomial equations of the reduced adjacency matrices in (*b*) and (*c*) are identical to those in (*a*) (adjacency matrix [7]), confirming that the reduced proto-graphs are isomorphic. The characteristic polynomial equations for the reduced adjacency matrices derived from adjacency matrix [7′], shown in (*d*), are different, confirming that this proto-graph is non-isomorphic with the other proto-graphs.

**Figure 16:**
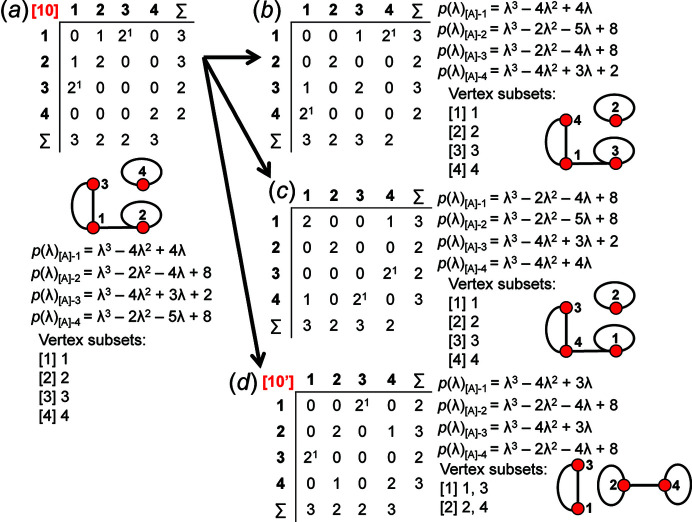
For vertex connectivity ^2^ *V*_2_^3^ *V*_2_, (*a*) adjacency matrix [10] for the matrix-element combination (2 × 1)(2 × 2)(2 × 2^1^), the corresponding proto-graph, the characteristic polynomial equations of the reduced proto-graphs and the resulting vertex subsets. (*b*), (*c*), (*d*) Three permutated versions of this adjacency matrix and proto-graph, and the characteristic polynomial equations of the reduced adjacency matrices. The characteristic polynomial equations of the reduced adjacency matrices in (*b*) and (*c*) are identical to those in (*a*) (adjacency matrix [10]), confirming that these proto-graphs are isomorphic. The characteristic polynomial equations for the reduced adjacency matrices derived from adjacency matrix [10′], shown in (*d*), are different, confirming that this proto-graph is non-isomorphic with the proto-graphs in (*b*) and (*c*).

Although only three of the permuted versions of matrix [1] are shown in Fig. 13 ▸, all permutations correspond to proto-graphs with identical characteristic polynomials, and we therefore conclude that the matrix-element combination (10 × 1) corresponds to only a single proto-graph with vertex connectivity ^2^ *V*_2_^3^ *V*_2_. Next consider Fig. 15 ▸(*a*); here we examine matrix [7] [matrix-element combination (4 × 1)(1 × 2)(2 × 2^1^)] (Fig. 11 ▸) and the corresponding proto-graph [7] (Fig. 12 ▸). Here, the vertex subsets of the permuted matrices in Figs. 15 ▸(*b*) and 15 ▸(*c*) and of the original matrix [7] [Fig. 15 ▸(*a*)] are identical but are different from the vertex subsets of permuted matrix [7′] shown in Fig. 15 ▸(*d*) (indicated by the characteristic polynomial equations). Although there are many more valid permuted versions of matrix [7] compared with what is shown in Fig. 15 ▸, all correspond to proto-graphs with vertex subsets identical to those of either graph [7] or [7′]. Thus the matrix-element combination (4 × 1)(1 × 2)(2 × 2^1^) corresponds to two non-isomorphic proto-graphs (graphs [7] and [7′]; Fig. 12 ▸). Now consider Fig. 16 ▸(*a*) which shows matrix [10] [matrix-element combination (2 × 1)(2 × 2)(2 × 2^1^)] (Fig. 11 ▸) and the corresponding proto-graph [10] (Fig. 12 ▸). Here, the vertex subsets of the permuted matrices in Figs. 16 ▸(*b*) and 16 ▸(*c*) and of the original matrix [10] [Fig. 16 ▸(*a*)] are identical but are different from the vertex subsets of permuted matrix [10′] shown in Fig. 16 ▸(*d*) (indicated by the characteristic polynomial equations). There are two different vertex subsets and we conclude that the matrix-element combination (2 × 1)(2 × 2)(2 × 2^1^) corresponds to two non-isomorphic proto-graphs (graphs [10] and [10′]) (Fig. 12 ▸). For vertex connectivity ^2^ *V*_2_^3^ *V*_2_, 4 of the 14 valid matrix-element combinations, [7], [8], [10] and [11], correspond to two distinct valid matrices (Fig. 11 ▸, [7′], [8′], [10′] and [11′]) and two non-isomorphic proto-graphs (Fig. 12 ▸, [7′], [8′], [10′] and [11′]). Thus, there are (10 × 1) + (4 × 2) = 18 non-isomorphic proto-graphs with vertex connectivity ^2^ *V*_2_^3^ *V*_2_ (Fig. 12 ▸).

### 9.3. Derivation of edge subsets using vertex subsets

**Figure 17:**
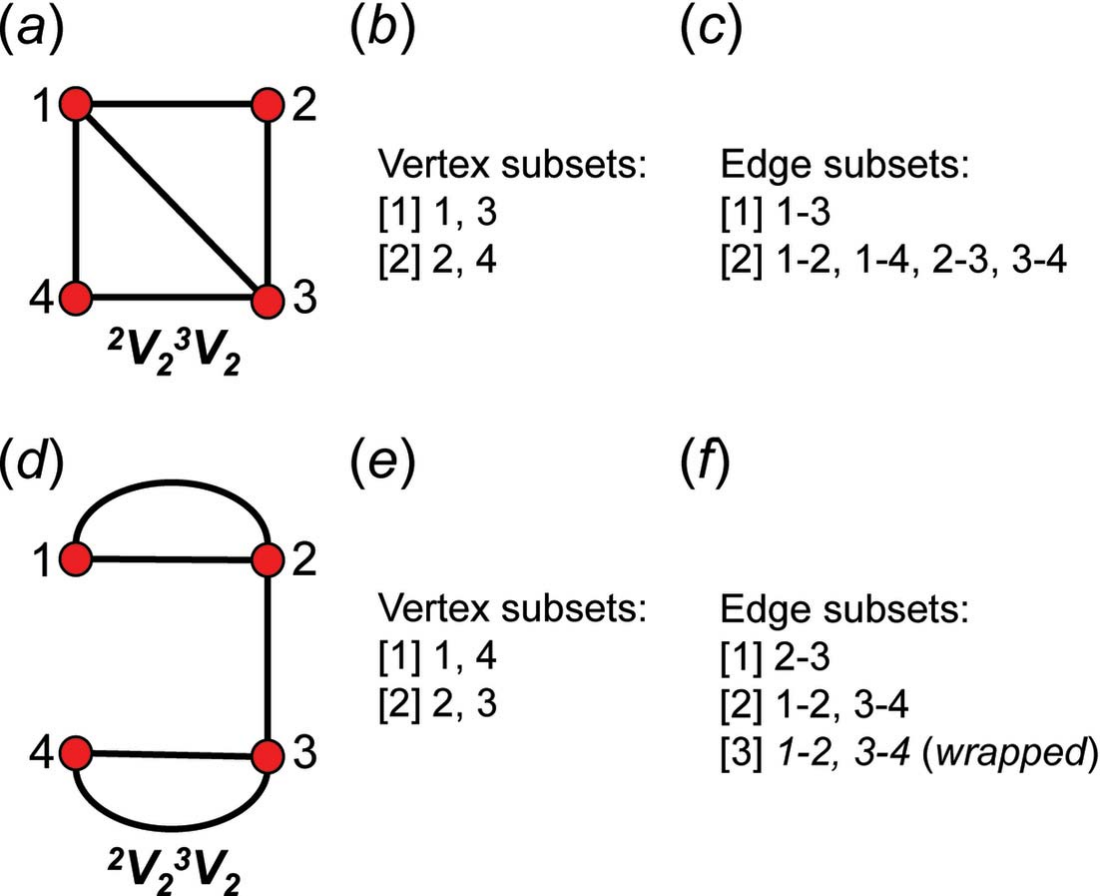
For vertex connectivity ^2^ *V*_2_^3^ *V*_2_, (*a*) the proto-graph for the matrix-element combination (10 × 1), (*b*) the corresponding vertex subsets, (*c*) the corresponding edge subsets. (*d*) The proto-graph for the matrix-element combination (2 × 1)(4 × 2^1^), (*e*) the corresponding vertex subsets, (*f*) the corresponding edge subsets.

Now that we have a method for deriving the set of all distinct valid matrices and corresponding non-isomorphic proto-graphs for a given *^c^V_r_*, the edge subsets for each proto-graph that correspond to such matrices can be determined. Two edges belong to the same subset if and only if they are both wrapped (or both not wrapped) and if the respective vertices to which they link belong to the same vertex subset. Note that wrapped edges are italicized in the edge subsets given in the figures. Consider the proto-graph with vertex connectivity ^2^ *V*_2_^3^ *V*_2_ in Fig. 17 ▸(*a*). The characteristic polynomial equations (Section 8.1) show that vertices 1 and 3 are isomorphic and vertices 2 and 4 are isomorphic, and hence this proto-graph has two vertex subsets [Fig. 17 ▸(*b*)]. This proto-graph has five edges, four of which link a vertex of subset [1] to a vertex of subset [2] and are therefore equivalent and belong to the same edge subset [Fig. 17 ▸(*c*)]. The fifth edge links a vertex of subset [1] to another vertex of subset [1] and therefore belongs to a second edge subset [Fig. 17 ▸(*c*)]. Consider the proto-graph with vertex connectivity ^2^ *V*_2_^3^ *V*_2_ in Fig. 17 ▸(*d*); there are two vertex subsets that each contain two vertices [Fig. 17 ▸(*e*)] and four edges that link vertices of subset [1] to vertices of subset [2] and one edge that links vertices of subset [2]. However, two of the four edges that link vertices of subset [1] to vertices of subset [2] are wrapped and therefore belong to a third edge subset [Fig. 17 ▸(*f*)]. Thus, once the vertex subsets have been established, the edge subsets may be read from the corresponding proto-graph by inspection.

### 9.4. *MatLab* limitations and multiplicity of matrix-element combinations with matrix elements 2^1^ and 2^2^

The method described in Sections 9.1 and 9.2 generates all possible non-isomorphic proto-graphs for a given *^c^V_r_* but is extremely laborious and impractical where *n* = 5–8 as the number of possible matrix-element permutations (and non-isomorphic proto-graphs) increases exponentially as the number and degree of such vertices increase. Consequently, we developed *MatLabR2019b* code (Appendix *B*, supporting information) to derive the set of proto-graphs for a given *^c^V_r_*. All valid matrix-element combinations and their associated proto-graphs that contain the matrix elements 1, 2 and/or 2^2^ are generated using this code. However, this code does not differentiate between curved and straight lines as the memory consumption for such a code is impractically large. Consequently, valid matrix-element combinations that contain the matrix element 2^1^ must be derived manually.

**Figure 18:**
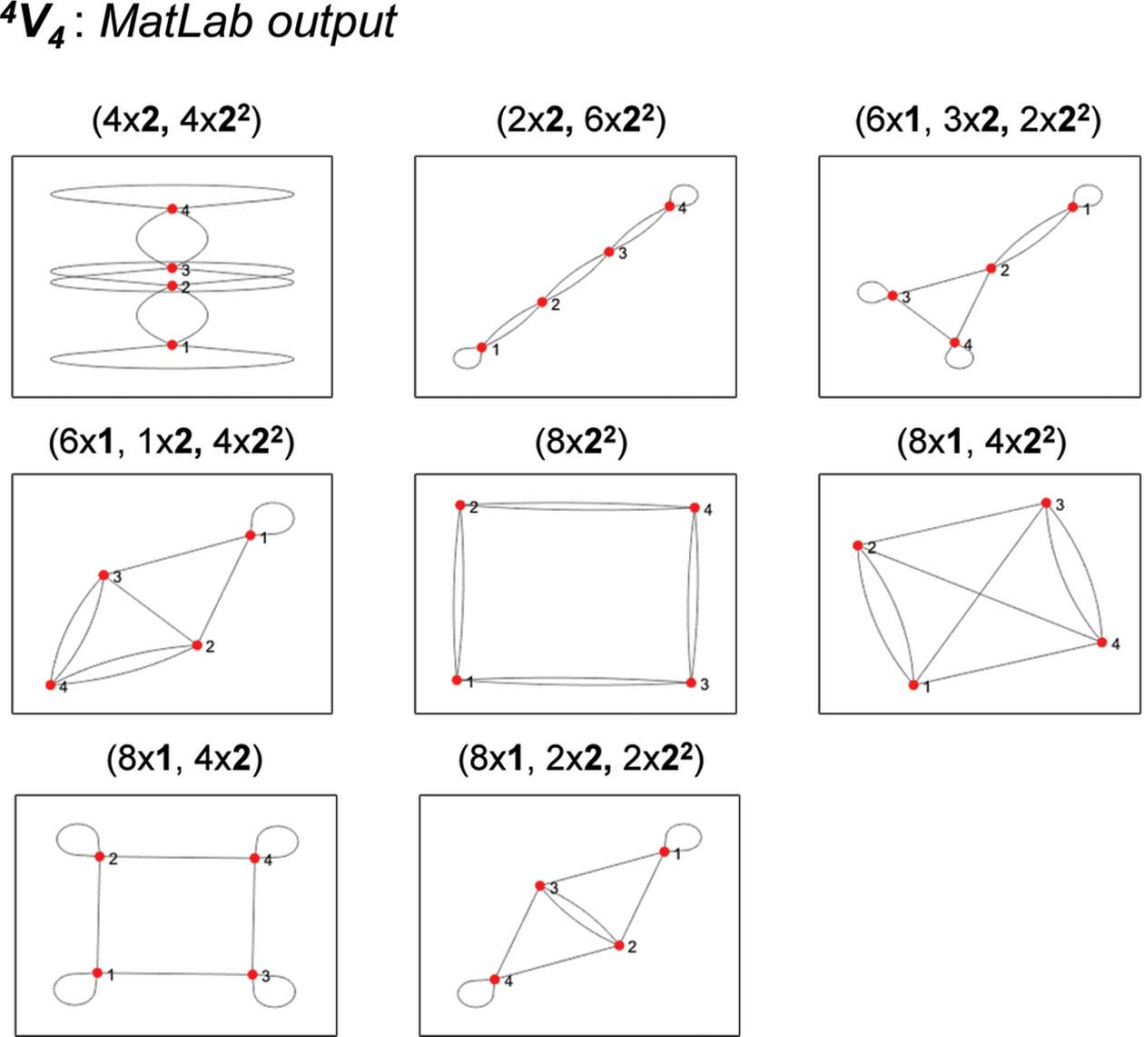
The *MatLabR2019b* output of all possible valid matrix-element combinations and corresponding non-isomorphic proto-graphs for the vertex connectivity ^4^ *V*_4_.

**Figure 19:**
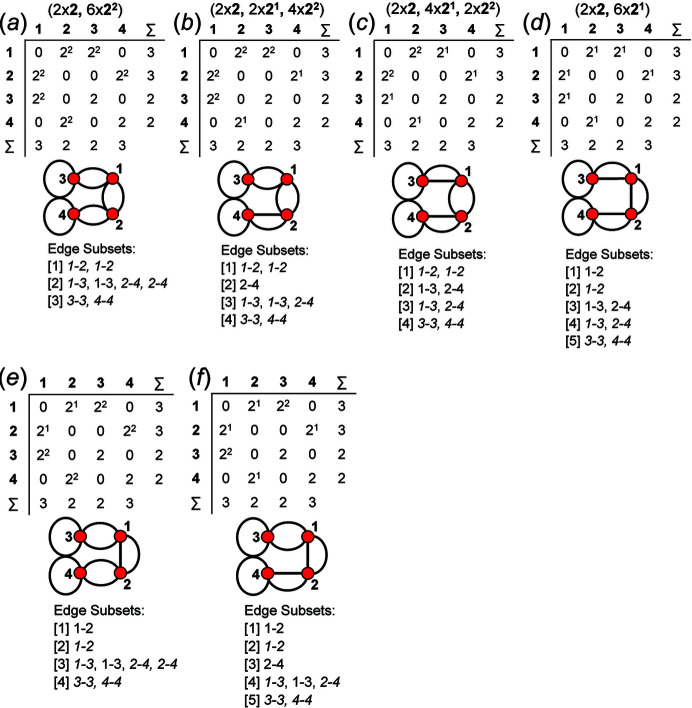
For vertex connectivity ^4^ *V*_4_, the adjacency matrix, corresponding proto-graph and edge subsets for the matrix-element combinations (*a*) (2 × 2)(6 × 2^2^), (*b*) (2 × 2)(2 × 2^1^)(4 × 2^2^), (*c*) (2 × 2)(4 × 2^1^)(2 × 2^2^) and (*d*) (2 × 2)(6 × 2^1^). The matrix-element combinations (2 × 2)(2 × 2^1^)(4 × 2^2^) and (2 × 2)(4 × 2^1^)(2 × 2^2^) correspond to a second distinct matrix and non-isomorphic proto-graph shown in (*e*) and (*f*), respectively. The adjacency matrices and corresponding proto-graphs in (*b*)–(*f*) are not produced by the *MatLabR2019b* code and must be derived manually. In edge subsets, wrapped edges are italicized.

Consider the *MatLabR2019b* output (Appendix *B*, supporting information) for the vertex connectivity ^4^ *V*_4_ in Fig. 18 ▸. Only proto-graphs that correspond to matrix-element combinations with 1, 2 and/or 2^2^ are produced and additional valid matrix-element combinations (and their corresponding proto-graphs) that involve 2^1^ may now be derived. As an example, consider the matrix-element combination (2 × 2)(6 × 2^2^) and the corresponding proto-graph and edge subsets in Fig. 19 ▸(*a*). We begin by converting two of the 2^2^ matrix elements in the matrix in Fig. 19 ▸(*a*) to 2^1^ (change one of the curved 2–4 edges to a straight edge) to generate the matrix-element combination (2 × 2)(2 × 2^1^)(4 × 2^2^) and the corresponding non-isomorphic proto-graph in Fig. 19 ▸(*b*). Next, we convert four of the 2^2^ matrix elements in the matrix in Fig. 19 ▸(*a*) to 2^1^ (the 2–4 and 1–3 edges) to generate the matrix-element combination (2 × 2)(4 × 2^1^)(2 × 2^2^) and the corresponding non-isomorphic proto-graph in Fig. 19 ▸(*c*). Finally, we convert all six of the 2^2^ matrix elements in the matrix in Fig. 19 ▸(*a*) to 2^1^ (the 2–4, 1–3 and 1–2 edges) to generate the matrix-element combination (2 × 2)(6 × 2^1^) and the corresponding non-isomorphic proto-graph in Fig. 19 ▸(*d*). We can then repeat this process for the other matrix-element combinations in Fig. 18 ▸ that contain the matrix element 2^2^. For a given matrix-element combination that contains *n* 2^2^ matrix elements, there are at least *n*/2 additional matrix-element combinations and associated proto-graphs that need to be generated.

For matrix-element combinations that contain both matrix elements 2^1^ and 2^2^, there may be more than one distinct adjacency matrix and non-isomorphic proto-graph. To produce the matrix-element combination (2 × 2)(2 × 2^1^)(4 × 2^2^) [Fig. 19 ▸(*b*)], one of the 2^2^ edges in Figs. 19 ▸(*a*), 2–4, 1–3 or 1–2, must be converted to a 2^1^ edge. However, the 1–3 and 2–4 edges belong to edge subset [2] and the 1–2 edges belong to edge subset [1]. It follows that there are two possible non-isomorphic proto-graphs that conform to the matrix-element combination (2 × 2)(2 × 2^1^)(4 × 2^2^), one in which one of the 2–4 or 1–3 edges is straight (a 2^1^ matrix element) [Fig. 19 ▸(*b*)] and another in which one of the 1–2 edges is straight [Fig. 19 ▸(*e*)]. To produce the matrix-element combination (2 × 2)(4 × 2^1^)(2 × 2^2^) [Fig. 19 ▸(*c*)], two of the 2^2^ edges in Figs. 19 ▸(*a*), 2–4, 1–3 and/or 1–2, must be converted to a 2^1^ edge. Again, there are two possible non-isomorphic proto-graphs. The first [Fig. 19 ▸(*c*)] is produced by selecting both 2^2^ edges from subset [2] [Fig. 19 ▸(*a*)] where one of the 2–4 and 1–3 edges is straight. The second is produced by selecting one 2^2^ edge from subset [1] and another from subset [2] where one of the 2–4 (or 1–3) and 1–2 edges is straight [Fig. 19 ▸(*f*)]. For vertex connectivity ^4^ *V*_4_, all valid matrix-element combinations and adjacency matrices are shown in Appendix *E* (supporting information) and all non-isomorphic proto-graphs in Appendix *F* (supporting information).

All valid matrix-element combinations that contain the matrix element 2^1^ may be derived using the *MatLab* output (*i.e.* Fig. 18 ▸). In some cases, an additional procedure is required to derive multiple non-isomorphic proto-graphs (if they exist) for matrix-element combinations that contain the matrix elements 2^1^ and 2^2^ [as done for the matrix-element combinations in Figs. 19 ▸(*b*) and 19 ▸(*c*)]. As described in Section 9.2, other matrix-element combinations (those that do not contain matrix elements 2^1^ and/or 2^2^) may correspond to more than one non-isomorphic proto-graph but such graphs will be derived by the *MatLabR2019b* code (Appendix *B*, supporting information).

### 9.5. Assigning wrapped edges and unwrapping edges

Next, we assign wrapped edges and all possible directions of unwrapping to proto-graphs to produce directed proto-graphs. A directed proto-graph is a proto-graph in which one or more edges have been assigned as wrapped in either the +**c** or −**c** direction, and is produced by (i) assigning one or more straight edges of a given proto-graph as wrapped in the +**c** or −**c** direction and/or (ii) assigning an unwrapping direction (+**c** or −**c**) to one or more wrapped (curved) edges of a proto-graph.

**Figure 20:**
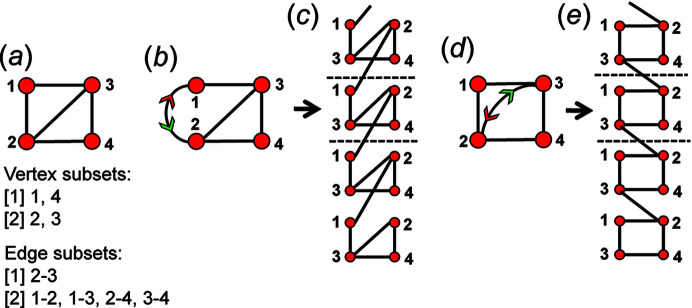
For vertex connectivity ^2^ *V*_2_^3^ *V*_2_, (*a*) a proto-graph for the matrix-element combination (10 × 1) and its vertex and edge subsets, (*b*) the directed proto-graph in which the 1–2 edge (of edge subset [2]) has been assigned as wrapped in the +**c** direction, and (*c*) the resultant chain graph. (*d*) The directed proto-graph in which the 2–3 edge (of edge subset [1]) has been assigned as wrapped in the +**c** direction, and (*e*) the resultant chain graph. Note how unwrapping single edges that belong to different edge subsets results in non-isomorphic chain graphs. Legend as in Fig. 7 ▸; the green and red arrows on the curved 1–2 and 2–3 edges denote the directions in which the edge is to be unwrapped: green = in the +**c** direction, red = in the −**c** direction.

Fig. 20 ▸(*a*) shows a proto-graph, Fig. 20 ▸(*b*) shows a corresponding directed proto-graph in which the 1–2 edge is assigned as wrapped, and Fig. 20 ▸(*c*) shows the corresponding (unwrapped) chain graph. Assigning and unwrapping the 1–3, 2–4 or 3–4 edges results in the same chain graph [Fig. 20 ▸(*c*)] as these edges belong to the same edge subset as edge 1–2 [Fig. 20 ▸(*a*)]. Assigning the 2–3 edge as wrapped results in a different directed proto-graph [Fig. 20 ▸(*d*)] and unwrapping this graph generates a chain graph [Fig. 20 ▸(*e*)] that is non-isomorphic with the chain graph in Fig. 20 ▸(*c*) as the 1–2 and 2–3 edges belong to different edge subsets [Fig. 20 ▸(*a*)]. When unwrapping a single edge in a series of directed proto-graphs generated from a single proto-graph, non-isomorphic chain graphs result only where the unwrapped edges belong to different edge subsets.

## 10. Derivation of all non-isomorphic chain and cluster graphs

For a given *^c^V_r_*, we may use the methods described above to derive all possible non-isomorphic proto-graphs (together with their associated adjacency matrices) and to determine their vertex and edge subsets. A particular proto-graph may then be used to generate all non-isomorphic directed proto-graphs with which it is conformable. This is done in two steps: (i) all curved edges in the proto-graph are assigned directions in which they are to be unwrapped (by assigning green and red arrows, *e.g.* Fig. 20 ▸); (ii) all unique combinations of straight edges in the proto-graph (except for 2^1^ straight edges as unwrapping this straight edge results in the matrix element 2^2^ which we have already produced in other matrix-element combinations) are similarly assigned directions in which they are to be unwrapped. The result of (i) and (ii) is a set of directed proto-graphs. In turn, the wrapped edges of these directed proto-graphs may be unwrapped to generate all non-isomorphic chain and cluster graphs. There are two types of edges that may occur in the directed proto-graphs: curved (wrapped) edges and straight edges. Curved edges must link to vertices of adjacent repeat units and hence curved edges must always be unwrapped when generating chain graphs. As shown in Fig. 20 ▸ (Section 9.5), when assigning a single straight edge as wrapped, only one edge from each subset needs to be chosen to generate the set of all non-isomorphic directed proto-graphs. When assigning more than one straight edge as wrapped, all unique combinations of straight edges (edge combinations) must be derived (irrespective of the edge subset to which they belong) to produce the set of all non-isomorphic directed proto-graphs and unwrapping such directed proto-graphs will generate all non-isomorphic chain graphs conformable with the parent proto-graph. For a given proto-graph, edge combinations and the corresponding directed proto-graphs are referred to as unique if the edges assigned as wrapped do not belong to the same edge subsets and/or if the directions assigned to such edges are different. Once the combinations of straight edges of the proto-graph are assigned as wrapped, those edges are replaced by curved edges and marked with red and green arrows to indicate the directions of unwrapping: green = +**c**, red = −**c**. Where an edge is assigned as wrapped, the direction in which this edge is to be unwrapped is appended to the corresponding matrix element as a superscript as described in Section 6.

### 10.1. Unwrapping edges and redundant unwrappings: vertices of degree 1 and 2

**Figure 21:**
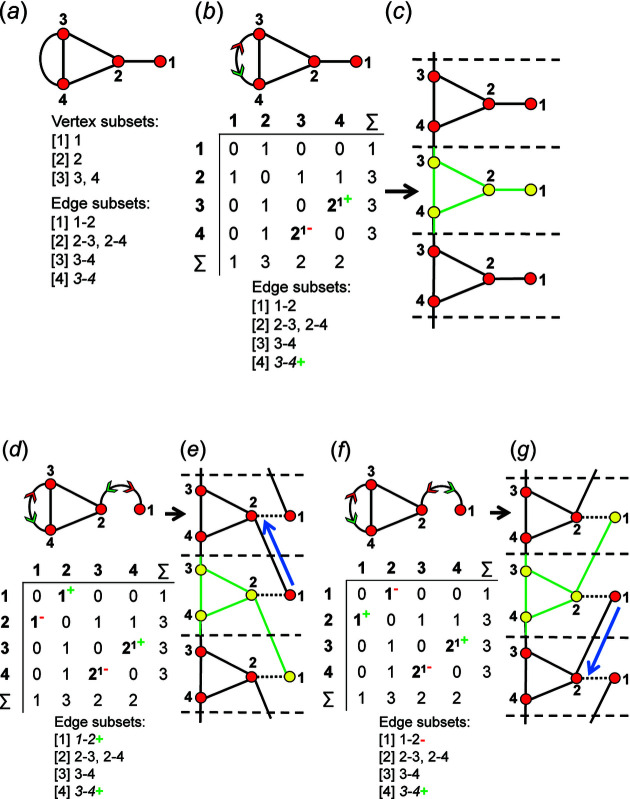
For vertex connectivity ^1^ *V*_1_^3^ *V*_3_, (*a*) a proto-graph for the matrix-element combination (6 × 1)(2 × 2^1^) and the corresponding vertex and edge subsets, (*b*) the directed proto-graph in which the 3–4 edge is assigned as wrapped in the +**c** direction and the corresponding adjacency matrix and edge subsets, and (*c*) the resultant chain graph. (*d*) The directed proto-graph in which the 3–4 and 1–2 edges have been assigned as wrapped in the +**c** direction and the corresponding adjacency matrix and edge subsets, and (*e*) the resultant chain graph. (*f*) The directed proto-graph in which the 3–4 and 1–2 edges have been assigned as wrapped in the +**c** and −**c** directions, respectively, and the corresponding adjacency matrix and edge subsets, and (*g*) the resultant chain graph. Note that the chain graphs in (*e*) and (*g*) are isomorphic with the chain graph in (*c*). The blue arrows show the direction of unwrapping of the 1–2 edge in (*e*) the +**c** direction and in (*g*) the −**c** direction. All edges and vertices of a single repeat unit in each chain graph are shown in green and yellow, respectively. Legend as in Figs. 7 ▸ and 20 ▸.

All unique combinations of edges must be assigned as wrapped for each proto-graph for a given *^c^V_r_* and then unwrapped to produce all non-isomorphic chain graphs that correspond to that *^c^V_r_*. However, many of these edge combinations correspond to directed proto-graphs that, once unwrapped, produce chain graphs that are isomorphic with previously generated chain graphs. Consider the proto-graph with vertex connectivity ^1^ *V*_1_^3^ *V*_3_ shown in Fig. 21 ▸(*a*). This proto-graph has a curved edge linking vertices 3 and 4, and this edge must be assigned coloured arrows to indicate the direction in which it will be unwrapped before straight edges can be assigned as wrapped edges. For edges that are curved in the proto-graph, the direction of unwrapping is not significant; this may be seen in the unwrapped chain graph [Fig. 21 ▸(*c*)] where vertex 3 links to vertex 4 in the +**c** direction and vertex 4 links to vertex 3 in the −**c** direction. Fig. 21 ▸(*b*) shows the directed proto-graph produced by assigning coloured arrows to edge 3–4 and Fig. 21 ▸(*c*) shows the corresponding chain graph in which vertices and edges of a single repeat have been coloured yellow and green, respectively. Fig. 21 ▸(*d*) shows the directed proto-graph produced by assigning the 3–4 and 1–2 edges as wrapped, and Fig. 21 ▸(*e*) shows the corresponding chain graph in which unwrapping of the 1–2 edge in the +**c** direction is denoted by the blue arrow and the original unwrapped 1–2 edge is shown by the dotted line. However, unwrapping the 1–2 edge does not produce a new chain graph [compare Figs. 21 ▸(*c*) and 21 ▸(*e*)]. In Fig. 21 ▸(*f*), the 3–4 and 1–2 edges are also unwrapped, but the 1–2 edge is unwrapped in the −**c** direction; again, we do not produce a new chain graph [Fig. 21 ▸(*g*)] [compare Figs. 21 ▸(*c*), 21 ▸(*e*) and 21 ▸(*g*)]. Vertex 1 has the degree 1 and is connected to the chain by a single 1–2 edge. Therefore, assigning this edge as wrapped in either direction must produce a chain graph isomorphic with the original chain graph in which the 1–2 edge is not unwrapped [Fig. 21 ▸(*c*)]. Unwrapping any edge linked to a vertex of degree 1 [*e.g.* vertex 1 in Figs. 21 ▸(*d*), 21 ▸(*f*)] will produce a chain graph isomorphic with that produced by unwrapping the same directed proto-graph in which this edge is not unwrapped.

**Figure 22:**
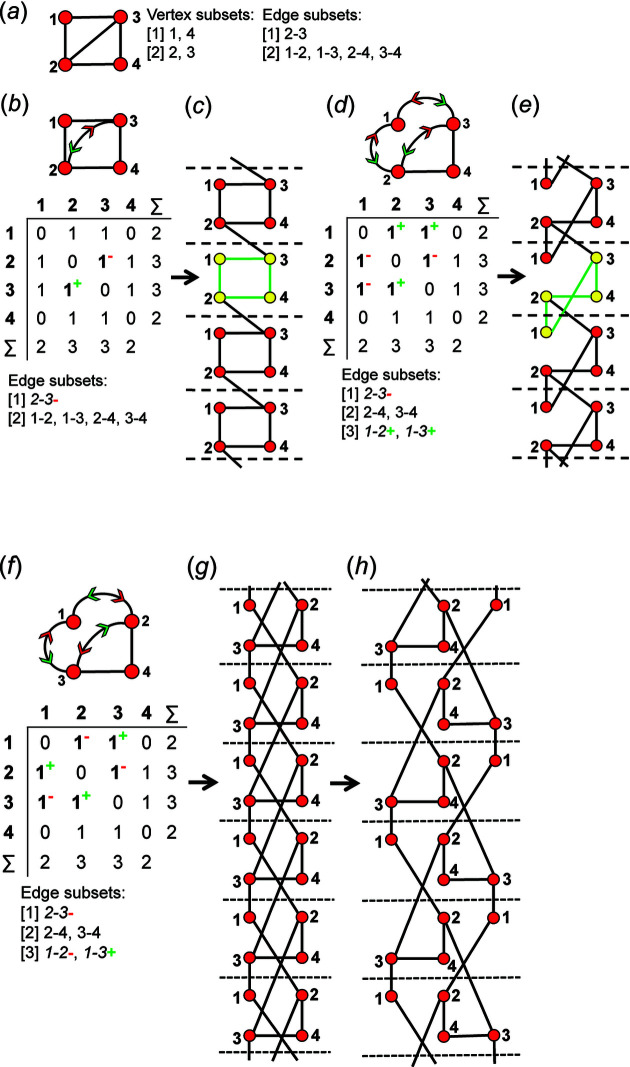
For vertex connectivity ^2^ *V*_2_^3^ *V*_2_, (*a*) a proto-graph [identical to that in Fig. 20 ▸(*a*)] for the matrix-element combination (10 × 1) and the corresponding vertex and edge subsets, (*b*) the directed proto-graph in which the 2–3 edge is assigned as wrapped in the −**c** direction and the corresponding adjacency matrix and edge subsets, and (*c*) the resultant chain graph. (*d*) The directed proto-graph in which the 1–2 and 1–3 edges have been assigned as wrapped in the +**c** direction and the 2–3 edge in the −**c** direction, and the corresponding adjacency matrix and edge subsets, and (*e*) the resultant chain graph. (*f*) The directed proto-graph in which the 2–3 and 1–2 edges have been assigned as wrapped in the −**c** direction and the 1–3 edge in the +**c** direction, and the corresponding adjacency matrix and edge subsets, (*g*) the resultant chain graph, and (*h*) an untangled version of this chain graph. Note that the chain graph in (*e*) is isomorphic with the chain graph in (*c*) as unwrappings involving vertex 1 in (*d*) are redundant. Legend as in Fig. 21 ▸.

Consider the proto-graph with vertex connectivity ^2^ *V*_2_^3^ *V*_2_ in Fig. 22 ▸(*a*); there are no curved edges and we may begin by assigning the 2–3 edge as wrapped, generating the resultant directed proto-graph in Fig. 22 ▸(*b*). Fig. 22 ▸(*c*) shows the chain graph produced by unwrapping the 2–3 edge in the −**c** direction. If the 2–3, 1–2 and 1–3 edges of the same proto-graph are assigned as wrapped, the directed proto-graph shown in Fig. 22 ▸(*d*) is generated. Fig. 22 ▸(*e*) shows the chain graph generated by unwrapping the 2–3 edge in the −**c** direction, the 1–2 edge in the +**c** direction and the 1–3 edge in the +**c** direction; the resultant chain graph [Fig. 22 ▸(*e*)] is isomorphic with the chain graph in Fig. 22 ▸(*c*). On generation of all unique edge combinations for this proto-graph [Fig. 22 ▸(*a*)], edge combinations that correspond to isomorphic chain graphs can be determined by inspection of the corresponding edge subsets. In Fig. 22 ▸(*d*), edges 1–2 and 1–3 are assigned as wrapped and therefore belong to a different edge subset [3] [compare with edge subsets in Figs. 22 ▸(*a*), 22 ▸(*b*)]. These edges are both unwrapped in the +**c** direction and are both linked to vertex 1, and therefore are redundant (may be omitted) and the resultant edge subset (and adjacency matrix) is identical to that in Fig. 22 ▸(*b*). The chain graph in Fig. 22 ▸(*e*) is isomorphic with the chain graph in Fig. 22 ▸(*c*), and hence unwrapping the edge combination (1–2+, 1–3+ and 2–3–) is redundant and the corresponding directed proto-graph [Fig. 22 ▸(*d*)] need not be generated.

The same edges (1–2, 1–3 and 2–3) are assigned as wrapped as in Fig. 22 ▸(*d*), but the 1–2 edge is to be unwrapped in the −**c** direction and the resultant directed proto-graph is shown in Fig. 22 ▸(*f*). The corresponding chain graph [Fig. 22 ▸(*g*)] is non-isomorphic with the chain graphs in Figs. 22 ▸(*c*), 22 ▸(*e*) which is clear from Fig. 22 ▸(*h*) which shows the chain graph in Fig. 22 ▸(*g*) with some of the visual overlap of the edges removed (we refer to this process as untangling). Both edges connected to vertex 1 (1–2 and 1–3) are unwrapped in opposite directions and therefore are not redundant; hence this edge subset [Fig. 22 ▸(*f*)] must be used as the corresponding directed proto-graph may produce a new non-isomorphic chain graph [Figs. 22 ▸(*g*), 22 ▸(*h*)].

**Figure 23:**
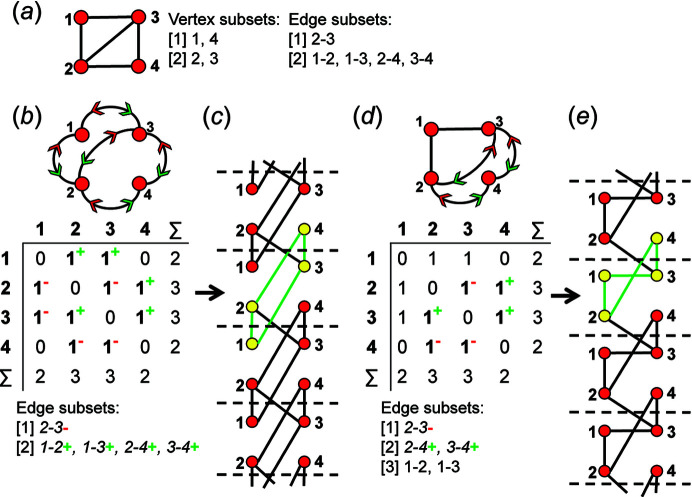
For vertex connectivity ^2^ *V*_2_^3^ *V*_2_, (*a*) a proto-graph [identical to that in Fig. 20 ▸(*a*)] and the corresponding vertex and edge subsets, (*b*) the directed proto-graph in which the 1–2, 1–3, 2–4 and 3–4 edges are assigned as wrapped in the +**c** direction and the 2–3 edge is assigned as wrapped in the −**c** direction, and its corresponding adjacency matrix, and (*c*) the resultant chain graph. (*d*) The directed proto-graph produced by omitting redundant unwrappings involving vertex 1 in (*b*), and its corresponding adjacency matrix and edge subsets, and (*e*) the resultant chain graph. Note that the chain graph in (*e*) is isomorphic with the chain graph in (*c*). Legend as in Figs. 7 ▸ and 21 ▸.

Consider the same proto-graph in Fig. 23 ▸(*a*). We may choose an edge combination in which the 1–2, 1–3, 2–4 and 3–4 edges are assigned as wrapped in the +**c** direction and the 2–3 edge is assigned as wrapped in the −**c** direction to produce the directed proto-graph and corresponding edge subset [Fig. 23 ▸(*b*)]. Unwrapping this graph generates the chain graph in Fig. 23 ▸(*c*). Inspection of this edge subset shows that both edges connected to vertices 1 and 4 are unwrapped in the +**c** direction and therefore are redundant, and this directed proto-graph will not generate a new non-isomorphic chain graph. Unwrapping the edges involving vertex 4 is redundant, and when omitted, the remaining edge subsets are identical to those in Fig. 22 ▸(*d*), and the chain graphs in Figs. 22 ▸(*c*), 22 ▸(*e*) and 23 ▸(*c*) are isomorphic. Alternatively, we may omit unwrapping the edges involving vertex 1 (instead of vertex 4) to produce the edge subsets, adjacency matrix and the corresponding directed proto-graph in Fig. 23 ▸(*d*). Unwrapping this graph generates the chain graph in Fig. 23 ▸(*e*) which is isomorphic with the chain graphs in Figs. 22 ▸(*c*), 22 ▸(*e*) and 23 ▸(*c*). If redundant unwrappings involving vertices 1 and 4 [Fig. 23 ▸(*b*)] are omitted, the remaining edge subset is identical to that in Fig. 22 ▸(*b*). Thus, the edge combinations that correspond to the edge subsets in Figs. 22 ▸(*d*), 23 ▸(*b*) and 23 ▸(*d*) need not be considered as each will generate a chain graph isomorphic with that in Fig. 22 ▸(*c*).

**Figure 24:**
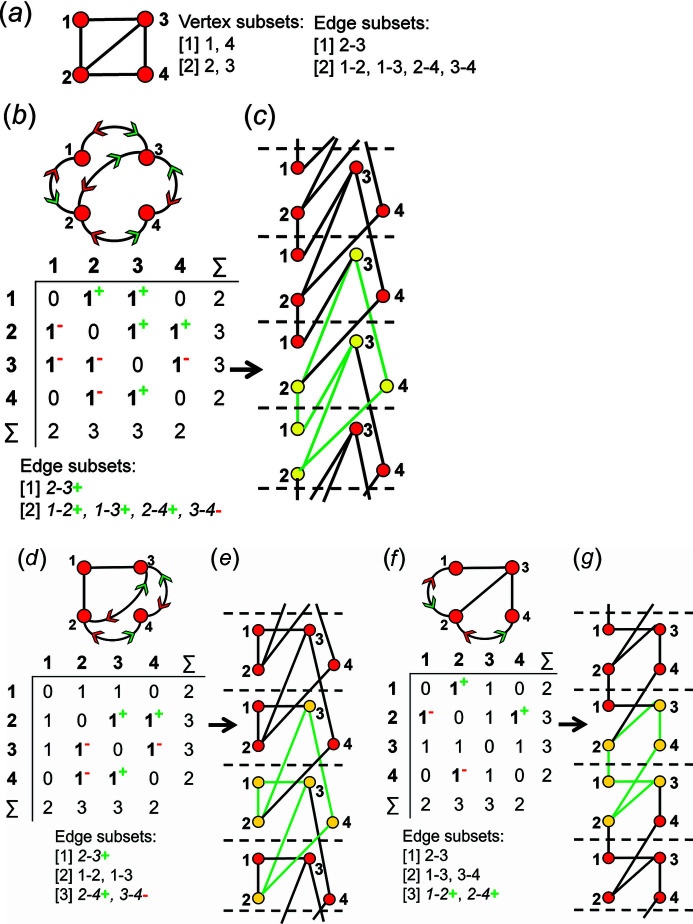
For vertex connectivity ^2^ *V*_2_^3^ *V*_2_, (*a*) a proto-graph [identical to that shown in Fig. 20 ▸(*a*)] and the corresponding vertex and edge subsets, (*b*) the directed proto-graph in which the 1–2, 1–3, 2–3 and 2–4 edges are assigned as wrapped in the +**c** direction and the 3–4 edge is assigned as wrapped in the −**c** direction, its corresponding adjacency matrix and edge subsets, and (*c*) the resultant chain graph. (*d*) The directed proto-graph produced by omitting redundant unwrappings involving vertex 1 in (*b*), its corresponding adjacency matrix and edge subsets, and (*e*) the resultant chain graph. (*f*) The directed proto-graph produced by omitting redundant unwrappings involving vertex 3 in (*b*), its corresponding adjacency matrix and edge subsets, and (*g*) the resultant chain graph. Note that the chain graphs in (*c*), (*e*) and (*g*) are isomorphic. Legend as in Figs. 7 ▸ and 21 ▸.

Consider the same proto-graph in Fig. 24 ▸(*a*). We may choose an edge combination in which the 1–2, 1–3, 2–3 and 2–4 edges are assigned as wrapped in the +**c** direction and the 3–4 edge is assigned as wrapped in the −**c** direction to produce the directed proto-graph and corresponding edge subset in Fig. 24 ▸(*b*). Unwrapping this graph generates the chain graph in Fig. 24 ▸(*c*). Inspection of this edge subset shows that all edges involving vertices 1 and 3 are unwrapped in the +**c** direction, and a new non-isomorphic chain graph is not generated. If we omit the unwrappings involving vertex 1, we produce the edge subsets, adjacency matrix and directed proto-graph in Fig. 24 ▸(*d*). Unwrapping this graph generates the chain graph in Fig. 24 ▸(*e*) which is isomorphic with the chain graph in Fig. 24 ▸(*c*). Alternatively, we may omit unwrappings involving vertex 3 (instead of vertex 1) to produce the edge subsets, adjacency matrix and corresponding directed proto-graph in Fig. 24 ▸(*f*). Unwrapping this graph generates the chain graph in Fig. 24 ▸(*g*) which is isomorphic with the chain graphs in Figs. 24 ▸(*c*), 24 ▸(*e*). The edge combinations that correspond to the edge subsets in Figs. 24 ▸(*b*), 24 ▸(*d*) need not be considered for unwrapping as they will generate chain graphs that are isomorphic with that in Fig. 24 ▸(*g*) which has already been generated thus far in the method as we begin by selecting edge combinations involving 1, 2, 3 edges and so on. The edges involving a specific vertex cannot be unwrapped in the same direction if such a vertex is linked to looped and/or multi-edges. It follows that if a row *n* (or column *n*) in the adjacency matrix contains the matrix elements 2, 2^1^ and/or 2^2^, all edges linked to vertex *n* cannot be unwrapped in the same direction as the matrix elements 2 and 2^2^ indicate unwrapping in both directions and the matrix element 2^1^ indicates one edge that is wrapped and one that is not wrapped.

### 10.2. Unwrapping edges and redundant unwrappings: vertices of degree 3 and 4

**Figure 25:**
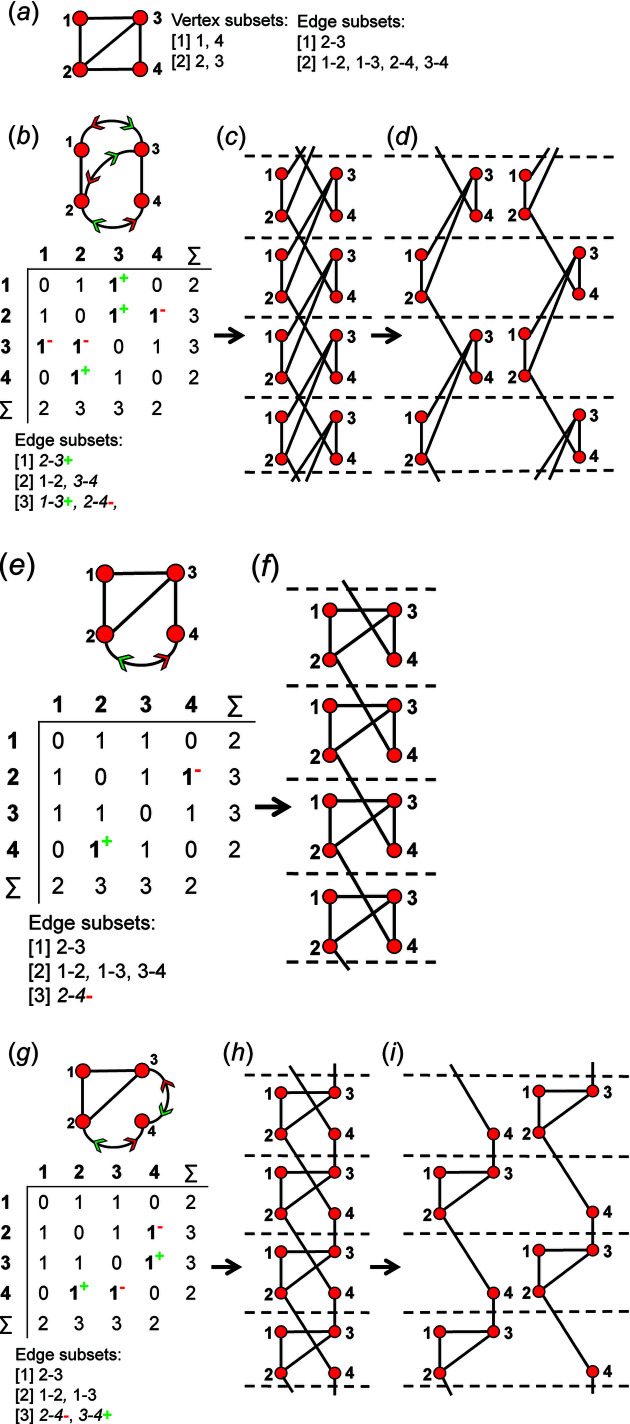
For vertex connectivity ^2^ *V*_2_^3^ *V*_2_, (*a*) a proto-graph [identical to that in Fig. 20 ▸(*a*)] and the corresponding vertex and edge subsets, (*b*) the directed proto-graph in which the 1–3 and 2–3 edges are assigned as wrapped in the +**c** direction and the 2–4 edge in the −**c** direction, its corresponding adjacency matrix and edge subsets, (*c*) the resultant chain graph, (*d*) an untangled version of this chain graph. (*e*) The directed proto-graph produced by omitting redundant unwrappings involving vertex 3 in (*b*), its corresponding adjacency matrix and edge subsets, and (*f*) the resultant chain graph which is non-isomorphic with the chain graph in (*c*). (*g*) The directed proto-graph in which the 2–4 edge is assigned as wrapped in the −**c** direction and the 3–4 edge is assigned as wrapped in the +**c** direction, its corresponding adjacency matrix and edge subsets, (*h*) the resultant chain graph, and (*i*) an untangled version of this chain graph. Note the chain graphs in (*h*) and (*i*) are isomorphic with the chain graphs in (*c*) and (*d*). Legend as in Figs. 7 ▸ and 21 ▸.

Consider the same proto-graph in Fig. 25 ▸(*a*). We may choose an edge combination in which the 1–3 and 2–3 edges are assigned as wrapped in the +**c** direction and the 2–4 edge is assigned as wrapped in the −**c** direction to produce the directed proto-graph and corresponding edge subsets in Fig. 25 ▸(*b*). Unwrapping this graph generates the chain graph in Fig. 25 ▸(*c*) and after untangling, we see that this chain graph consists of two unconnected chain graphs [Fig. 25 ▸(*d*)]. Here, two thirds of the edges involving vertex 3 are unwrapped in the +**c** direction; omitting such unwrappings produces the edge subsets, adjacency matrix and directed proto-graph in Fig. 25 ▸(*e*). However, unwrapping this graph generates the chain graph in Fig. 25 ▸(*f*) which is non-isomorphic with that in Figs. 25 ▸(*c*), 25 ▸(*d*). Thus, unwrappings involved with a given vertex can only be omitted if they are all unwrapped in the same direction (as described in Section 10.1). The edge combination in which the 2–4 edge is assigned as wrapped in the −**c** direction and the 3–4 edge is assigned as wrapped in the +**c** direction produces the directed proto-graph and corresponding edge subset in Fig. 25 ▸(*g*). Unwrapping this graph generates the chain graph in Fig. 25 ▸(*h*) and inspection of the untangled version of this chain graph [Fig. 25 ▸(*i*)] shows that it is isomorphic with that in Figs. 25 ▸(*c*), 25 ▸(*d*). Thus unwrapping non-isomorphic directed proto-graphs may (or may not) produce isomorphic chain graphs. This is the reason why this generating method may produce duplicate chain graphs for a given proto-graph. Note that predicting which directed proto-graphs will result in isomorphic chain graphs before they are unwrapped is not always possible but can be done for most proto-graphs using the rules described in Appendix *A* [App appa].

**Figure 26:**
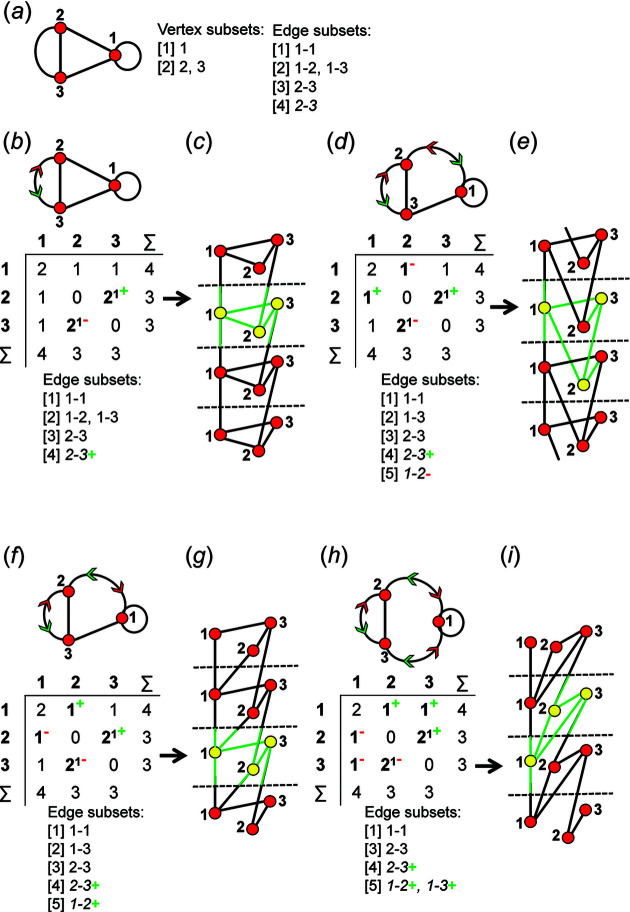
For vertex connectivity ^3^ *V*_2_^4^ *V*_1_, (*a*) a proto-graph for the matrix-element combination (4 × 1)(2 × 1)(2 × 2^1^) and its vertex and edge subsets, (*b*) the directed proto-graph in which the 2–3 edge is assigned as wrapped in the +**c** direction, its corresponding adjacency matrix and edge subsets, and (*c*) the resultant chain graph. (*d*) The directed proto-graph in which the 2–3 edge is assigned as wrapped in the +**c** direction and the 1–2 edge is assigned as wrapped in the −**c** direction, and the corresponding adjacency matrix and edge subsets, and (*e*) the resultant chain graph. (*f*) The directed proto-graph in which the 2–3 and 1–2 edges are assigned as wrapped in the +**c** direction, its corresponding adjacency matrix and edge subsets, and (*g*) the resultant chain graph. (*h*) The directed proto-graph in which the 2–3, 1–2 and 1–3 edges are assigned as wrapped in the +**c** direction, its corresponding adjacency matrix and edge subsets, and (*i*) the resultant chain graph. Note that the chain graphs in (*e*) and (*i*) are isomorphic with the chain graph in (*c*) as the unwrappings involving vertex 2 in (*d*) are redundant and the unwrappings involving vertex 1 in (*h*) are redundant. Legend as in Figs. 7 ▸ and 21 ▸.

Consider the proto-graph in Fig. 26 ▸(*a*) in which one of the two 2–3 edges is curved. Arrows are automatically assigned to this edge to produce the edge subsets, adjacency matrix and directed proto-graph in Fig. 26 ▸(*b*). Unwrapping this graph generates the chain graph in Fig. 26 ▸(*c*). If the 1–2 edge of the directed proto-graph in Fig. 26 ▸(*b*) is assigned as wrapped in the −**c** direction, the directed proto-graph in Fig. 26 ▸(*d*) is produced. Unwrapping this graph generates the chain graph in Fig. 26 ▸(*e*) which is isomorphic with the chain graph in Fig. 26 ▸(*c*). In Fig. 26 ▸(*d*), two thirds of the edges (1–2, 3–2) involved with vertex 2 are unwrapped in the same (+**c**) direction and the third edge is not unwrapped. If we change the unwrapping direction of the 1–2 edge from −**c** [Fig. 26 ▸(*d*)] to +**c**, the edge subset, adjacency matrix and directed proto-graph in Fig. 26 ▸(*f*) are produced. Unwrapping this graph generates the chain graph in Fig. 26 ▸(*g*) which is non-isomorphic with that in Figs. 26 ▸(*c*), 26 ▸(*e*). Here, two thirds of the edges linked to vertex 2 are unwrapped in opposite directions. If the 1–3 edge of the directed proto-graph in Fig. 26 ▸(*f*) is assigned as wrapped in the +**c** direction, the edge subsets, adjacency matrix and directed proto-graph in Fig. 26 ▸(*h*) are produced. Unwrapping this graph generates the chain graph in Fig. 26 ▸(*i*) which is isomorphic with that in Figs. 26 ▸(*c*), 26 ▸(*e*). Here, three quarters of the edges linked to vertex 1 are unwrapped in the same (+**c**) direction (a diagonal matrix element of 2 indicates unwrapping in both directions) and the fourth 1–2 edge is unwrapped in the −**c** direction; two thirds of the three edges linked to vertex 3 are unwrapped in the same direction and the third edge is not unwrapped.

### 10.3. D* vertices and redundant unwrappings

Based on the examples in Sections 10.1 and 10.2, we conclude the following: if more than half the edges that link to a particular vertex (of any degree) of a directed proto-graph are unwrapped in the same direction and the other edges are not unwrapped, a new non-isomorphic chain graph will not be produced. We refer to such a vertex as a D* vertex. Thus, any edge combination that gives rise to edge subset(s) with a D* vertex need not be considered. Note that D* vertices are possible only where the corresponding matrix row (and column) contains only the matrix elements 1 and 2^1^ with the same sign (either all + or all −). Note also that edge combinations such as 1–3–, 1–2– and 1–3+, 1–2+ are redundant as they generate isomorphic chain graphs (with the positive direction of the translation axis reversed) and therefore only one of such edge combinations needs to be considered.

### 10.4. Linear and polygonal branches

**Figure 27:**
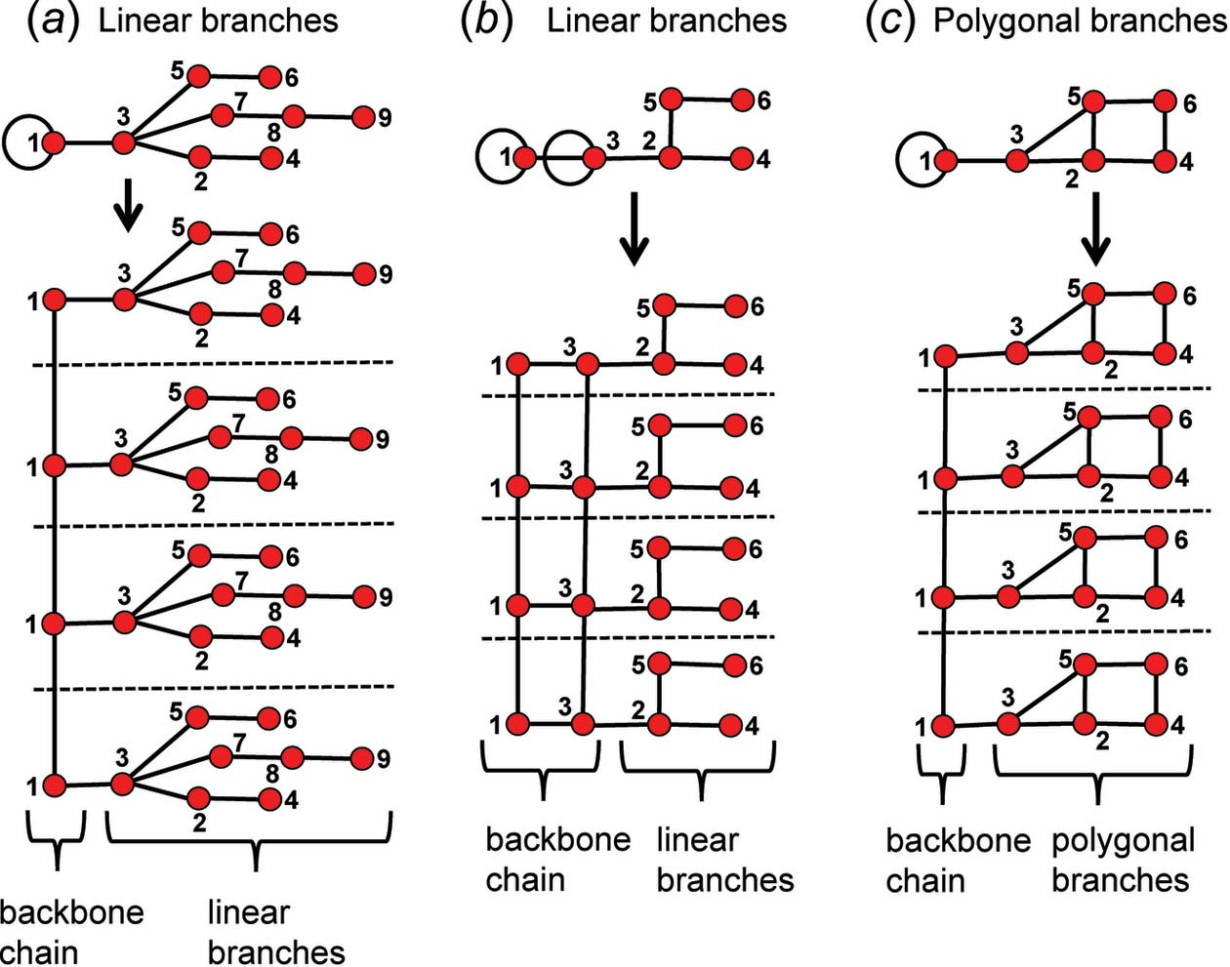
(*a*) A proto-graph and corresponding chain graph with vertex connectivity ^1^ *V*_3_^2^ *V*_4_^3^ *V*_1_^4^ *V*_1_ in which edges 3–5, 3–2, 3–7, 2–4, 5–6, 7–8 and 8–9 and vertices 2 to 9 form linear branches, and edge 1–1 and vertex 1 form the backbone chain. (*b*) A proto-graph and corresponding chain graph with vertex connectivity ^1^ *V*_2_^2^ *V*_1_^3^ *V*_2_^4^ *V*_1_ in which edges 3–2, 2–4, 2–5 and 5–6 and vertices 2, 4, 5 and 6 form linear branches and edges 1–1, 3–3 and 1–3 and vertices 1 and 3 form the backbone chain. (*c*) A proto-graph and corresponding chain graph with vertex connectivity ^2^ *V*_2_^3^ *V*_4_ in which edges 3–2, 3–5, 2–5, 2–4, 5–6 and 4–6 and vertices 2 to 6 form polygonal branches, and edges 1–1 and vertex 1 form the backbone chain. Legend as in Fig. 7 ▸.

A branch is a set of vertices linked to the backbone chain of a chain graph by a single edge. Chain graphs may contain two types of branches: (i) linear branches and (ii) polygonal branches. Linear branches do not contain polygons, and polygonal branches do contain polygons. In Fig. 27 ▸(*a*), vertices 2 to 9 form a linear branch as they are connected to the backbone chain (vertex 1) by one edge (1–3). In Fig. 27 ▸(*b*), vertices 2, 4, 5 and 6 form a linear branch as they are connected to the backbone chain (vertices 1 and 3) by one edge (2–3). In Fig. 27 ▸(*c*), vertices 2, 3, 4, 5 and 6 form a polygonal branch as they are connected to the backbone chain (vertex 1) by one edge (1–3).

**Figure 28:**
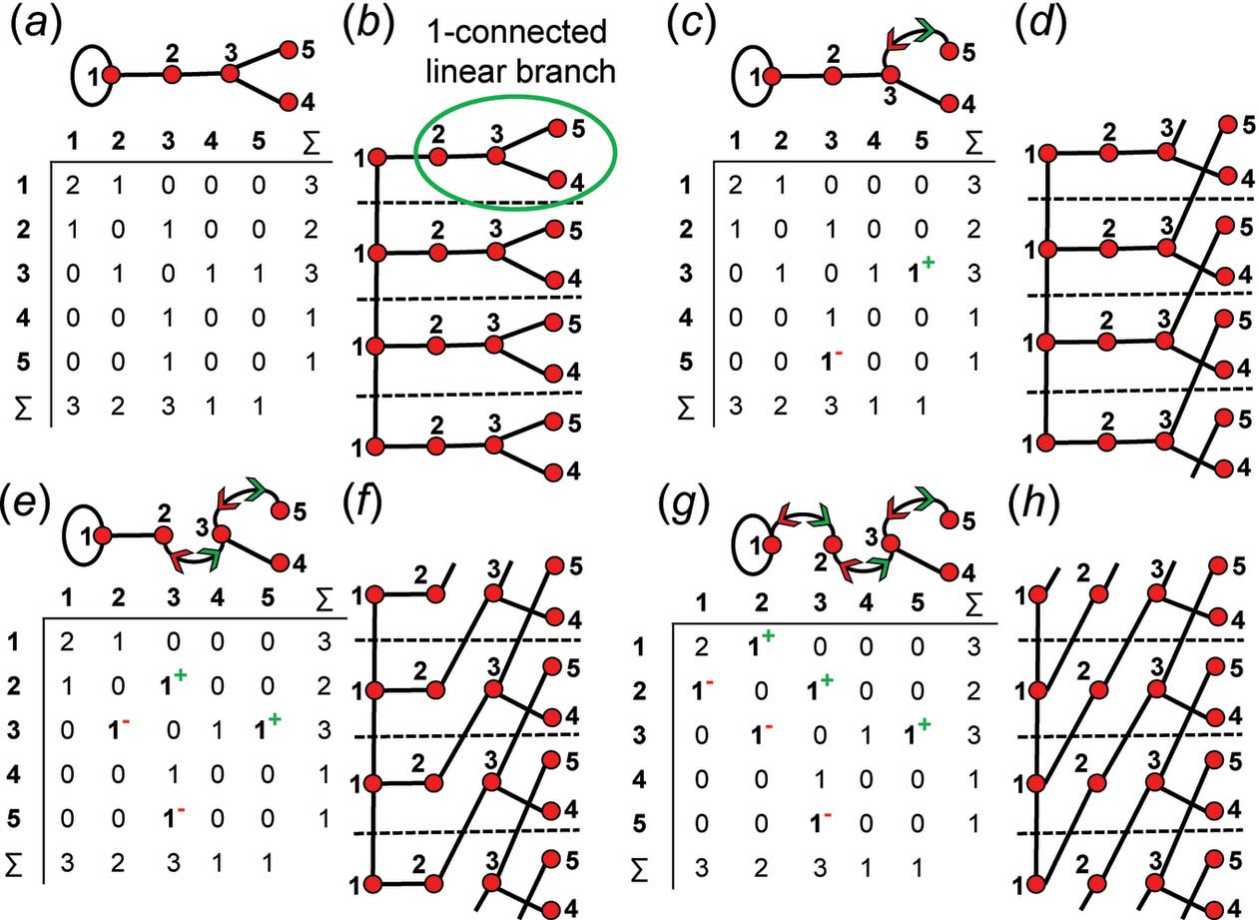
(*a*) A proto-graph with vertex connectivity ^1^ *V*_2_^2^ *V*_1_^3^ *V*_2_ and its corresponding adjacency matrix, and (*b*) the resultant chain graph in which edges 1–2, 2–3, 3–4 and 3–5 and vertices 2 to 5 form linear branches (indicated by the green ellipse). (*c*) A directed proto-graph in which the 3–5 edge is assigned as wrapped in the +**c** direction, its corresponding adjacency matrix, and (*d*) the resultant chain graph. (*e*) The directed proto-graph in which the 2–3 and 3–5 edges are assigned as wrapped in the +**c** direction, the corresponding adjacency matrix, and (*f*) the resultant chain graph. (*g*) The directed proto-graph in which the 1–2, 2–3 and 3–5 edges are assigned as wrapped in the +**c** direction, its corresponding adjacency matrix, and (*h*) the resultant chain graph. Note how chain graphs in (*b*), (*d*), (*f*) and (*h*) are isomorphic. Legend as in Fig. 7 ▸.

Identification of linear branches simplifies the generation of chain graphs as unwrapping any edge (in either direction) of a linear branch (of a directed proto-graph) will never result in a new non-isomorphic chain graph. Consider the proto-graph with vertex connectivity ^1^ *V*_2_^2^ *V*_1_^3^ *V*_2_ in Fig. 28 ▸(*a*). Unwrapping this graph generates the chain graph in Fig. 28 ▸(*b*) in which vertices 2, 3, 4 and 5 form a linear branch connected to the backbone chain by a single edge (1–2). Assigning any edge in such a branch as wrapped will not generate a new non-isomorphic chain graph. If the 3–5 edge is assigned as wrapped in the +**c** direction, the directed proto-graph in Fig. 28 ▸(*c*) is produced. Unwrapping this graph generates the chain graph in Fig. 28 ▸(*d*) which is isomorphic with the chain graph in Fig. 28 ▸(*b*) as vertex 5 is a D* vertex. The directed proto-graph in Fig. 28 ▸(*e*) is produced by assigning the 2–3 and 3–5 edges as wrapped in the +**c** direction. Unwrapping this graph generates the chain graph in Fig. 28 ▸(*f*) which is isomorphic with the chain graph in Figs. 28 ▸(*b*) and 28 ▸(*d*). The directed proto-graph in Fig. 28 ▸(*g*) is produced by assigning the 1–2, 2–3 and 3–5 edges as wrapped in the +**c** direction. Unwrapping this graph generates the chain graph in Fig. 28 ▸(*h*) which is isomorphic with the chain graph in Figs. 28 ▸(*b*), 28 ▸(*d*) and 28 ▸(*f*).

**Figure 29:**
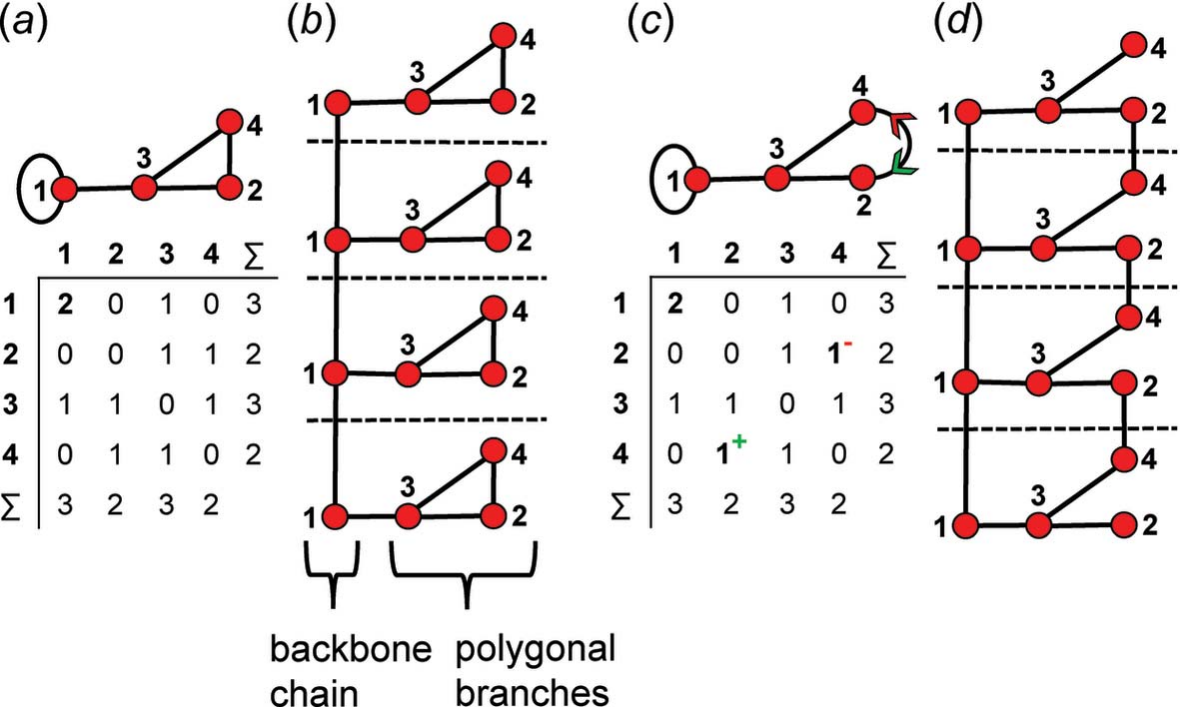
(*a*) A proto-graph with vertex connectivity ^2^ *V*_2_^3^ *V*_2_, its corresponding adjacency matrix, and (*b*) the resultant chain graph in which edges 2–3, 3–4 and 2–4 and vertices 2, 3 and 4 form polygonal branches. (*c*) The directed proto-graph in which the 2–4 edge is assigned as wrapped in the −**c** direction, and (*d*) the resultant chain graph which does not contain branches and is non-isomorphic with the chain graph in (*b*). Legend as in Fig. 7 ▸.

Unwrapping the directed proto-graph in Fig. 29 ▸(*a*) generates a chain graph that contains polygonal branches consisting of three-membered rings [Fig. 29 ▸(*b*)]. In Fig. 29 ▸(*c*), the same directed proto-graph is shown in which the 2–4 edge is assigned as wrapped in the −**c** direction. Unwrapping this graph generates the chain graph in Fig. 29 ▸(*d*) which is non-isomorphic with the chain graph in Fig. 29 ▸(*b*).

Unwrapping any edge of a linear branch will produce a chain graph isomorphic with the chain graph produced by unwrapping the same directed proto-graph in which such an edge is not unwrapped. This is the case irrespective of whether the edge of the linear branch is connected to a D* vertex or not. Therefore, any edge combination (and associated directed proto-graphs) that involves edges of linear branches need not be considered. If the edges of polygonal branches are unwrapped, additional non-isomorphic chain graphs may be generated and unlike linear branches, all unique combinations of such edges must be unwrapped to ensure all non-isomorphic chain graphs are generated.

Any proto-graph and adjacency matrix that contains only the element ‘1’ [matrix-element combination (*m*_1_ × 1)] and does not contain polygons cannot correspond to a chain and may be omitted. Assigning the edges of such a proto-graph as wrapped (in any combination and direction) and then unwrapping such edges, will always produce the original proto-graph and never a non-isomorphic cluster or chain. This simplification is important as it can drastically reduce the time spent generating non-isomorphic chain graphs from a given proto-graph.

### 10.5. Deriving non-isomorphic chain graphs: ^2^ *V*_2_^3^ *V*_2_

Thus far we have derived all valid matrix-element combinations (Table 2 ▸), associated matrices (Fig. 12 ▸) and the corresponding proto-graphs with vertex connectivity ^2^ *V*_2_^3^ *V*_2_ (Fig. 13 ▸) and we have determined which of these element combinations correspond to more than one distinct matrix and non-isomorphic proto-graph. All unique combinations of edges in each of the 18 non-isomorphic proto-graphs that do not result in D* vertices can now be unwrapped to generate all non-isomorphic chain graphs. For vertex connectivity ^2^ *V*_2_^3^ *V*_2_, we give an example of how this is done for the matrix-element combination (10 × 1) in Appendix *C* (supporting information). All other non-isomorphic chain graphs with vertex connectivity ^2^ *V*_2_^3^ *V*_2_ are generated (Appendix *G*, supporting information) by assigning the edges of the other 17 proto-graphs (Fig. 13 ▸) as wrapped in all unique combinations, and then unwrapping the resultant directed proto-graphs. Note that the matrix-element combination (10 × 1) is the most complicated case for ^2^ *V*_2_^3^ *V*_2_ as the number of non-isomorphic directed proto-graphs (and the number of non-isomorphic chain graphs generated) decreases as the number of 1’s in the corresponding matrix-element combination decreases. This happens as the number and direction of wrapped edges associated with matrix elements 2, 2^1^ and 2^2^ are fixed and therefore there are fewer edges that may be assigned as wrapped in the +**c** or −**c** direction, and fewer potential non-isomorphic directed proto-graphs and resultant chain graphs.

### 10.6. Generation of all possible non-isomorphic proto-graphs and chain graphs

**Figure 30:**
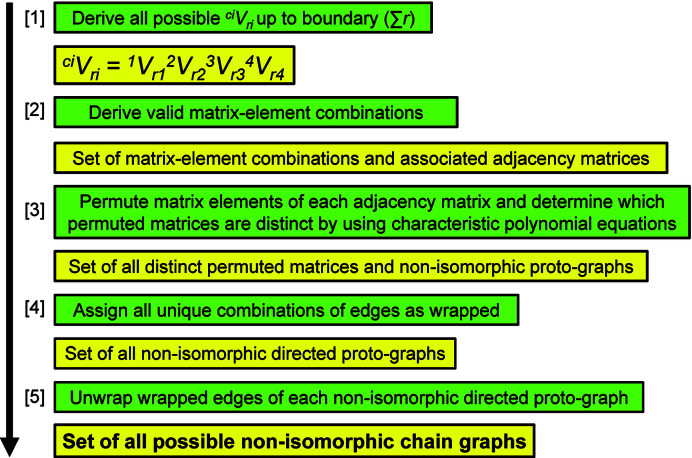
A flow chart of the overall method to derive all non-isomorphic chain graphs from the complete set of possible vertex connectivities ^1^ *V*_*r*1_^2^ *V*_*r*2_^3^ *V*_*r*3_^4^ *V*_*r*4_. Green boxes denote actions and yellow boxes denote the products.

The general procedure for generating all possible non-isomorphic chain graphs is summarized in Fig. 30 ▸. Non-isomorphic proto-graphs were generated with *MatLabR2019b* code that is listed in Appendix *B* (supporting information). In Section 8, a rationale for setting a boundary limit of ∑*r* ≤ 8 is provided. However, we do not generate all proto-graphs and non-isomorphic chain graphs for all vertex connectivities (*^c^V_r_*) for ∑*r* ≤ 8 as the number of non-isomorphic chain graphs increases exponentially with increasing *e* (number of edges), which is higher for larger values of *r* and would result in an impractically large number of chain graphs. For some vertex connectivities, all non-isomorphic chain graphs were generated for ∑*r* ≤ 8 (*e.g.* ^2^ *V*_8_), but for most vertex connectivities, chain graphs were generated for ∑*r* ≤ 4–6 as this results in a set of chain graphs of manageable size. For every selected *^c^V_r_*, all valid matrix-element combinations and their corresponding adjacency matrices are listed in Appendix *E* (supporting information). All proto-graphs that correspond to each adjacency matrix in Appendix *E* are compiled in Appendix *F* (supporting information). In Appendix *C*, we show how to generate all non-isomorphic chain graphs with vertex connectivity ^2^ *V*_2_^3^ *V*_2_ that correspond to the matrix-element combination (10 × 1). All non-isomorphic chain graphs generated using the method described above are compiled in Appendix *G* (supporting information). Clusters are included in Appendix *G* only if they are non-isomorphic with the proto-graph (Appendix *F*) from which they were generated. All possible clusters shown in Appendices *F* and *G* correspond to a matrix-element combination composed entirely of 1’s as (SiO_4_)^4–^ tetrahedra cannot link to themselves (diagonal 2’s) or share edges with other (SiO_4_)^4−^ tetrahedra (off-diagonal 2’s). Appendix *A* [App appa] is a list of the rules for determining which *^c^V_r_* expressions and/or directed proto-graphs may be omitted from consideration as they will not produce additional non-isomorphic chain graphs. Appendix *D* (supporting information) explains how Appendices *E*, *F* and *G* (supporting information) are organized and how to use them.

## 11. Discussion

**Figure 31:**
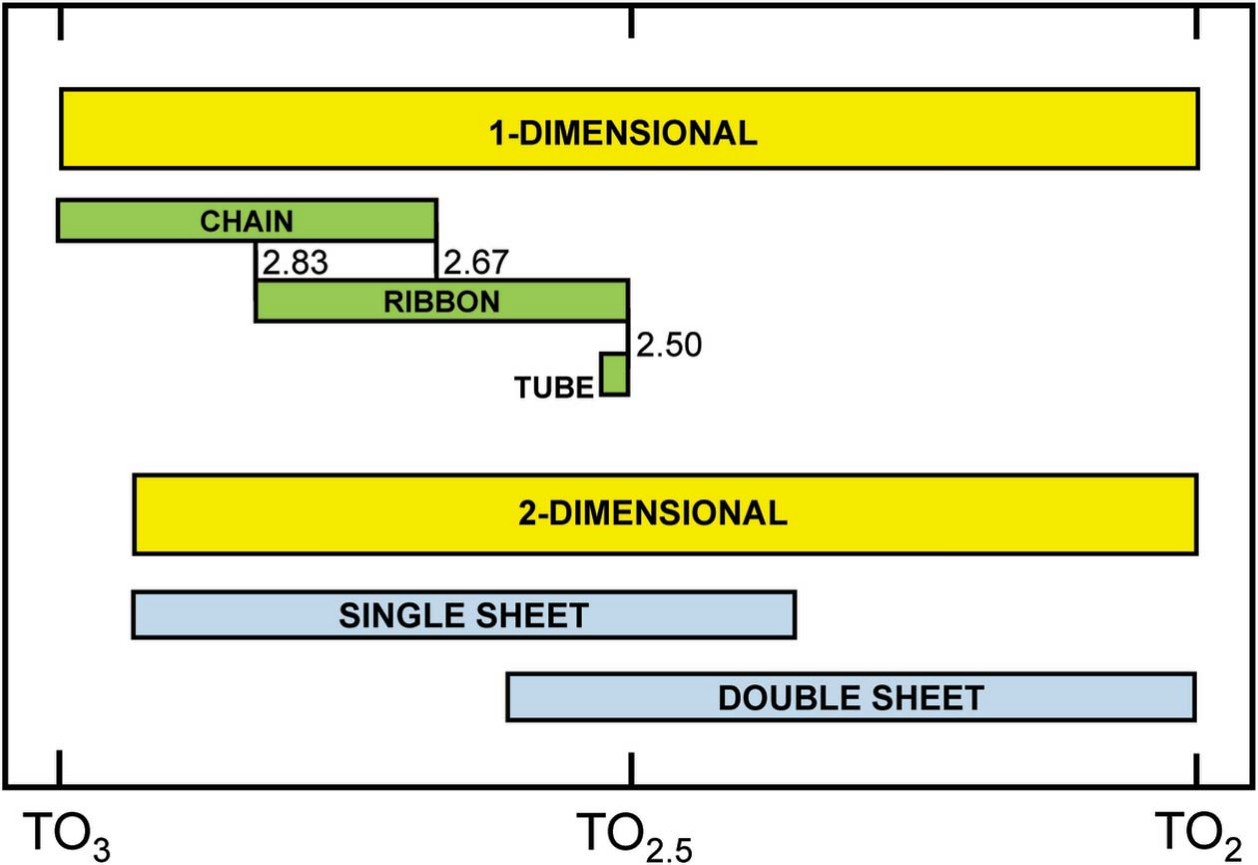
Range of *T*O_*n*_ for chain and sheet silicates. Yellow boxes show the range that is topologically possible for one- and two-dimensional polymerizations of tetrahedra, and the green boxes show the compositional ranges observed in chain- and sheet-silicate minerals. Modified from Day & Hawthorne (2020 ▸).

One often reads in the literature (and in reviews of submitted manuscripts) that one should ‘compare observed structures with other possible structural arrangements’, as if there are only one or two other possible structural arrangements of the same (or similar) stoichiometry. We have shown here that there is generally a large number of topologically possible structural arrangements for specific stoichiometries. Indeed, Day & Hawthorne (2020 ▸) showed that there are ∼50 non-isomorphic chain arrangements of silicate tetrahedra observed in the ∼450 chain-silicate minerals, four of which occur in ∼375 chain silicates and ∼46 of which occur in ∼75 chain silicates. Comparison of these numbers with the numbers of topologically possible chains of even modest stoichiometry suggests that most chain graphs (listed in Appendix *G* for *T*O_*n*_ stoichiometries of *n* ≤ 8) do not occur in minerals or synthetic compounds. Fig. 31 ▸ shows the distribution of possible ranges in *T*O_*n*_ for one- and two-dimensional silicate polymerizations (yellow boxes) and the ranges in *T*O_*n*_ observed in minerals (Day & Hawthorne, 2020 ▸). It is apparent from Fig. 31 ▸ that the chemical compositions of atomic arrangements are controlled (at least in part) by the bond topological characteristics of their arrangements, and that the generation of all possible non-isomorphic chain graphs provides the opportunity to (i) ‘compare observed structures with other possible structural arrangements’, and (ii) examine why some stoichiometries for chain (and sheet) arrangements are topologically possible but not observed in crystal structures.

In Part II of this work, we will examine in detail the factors affecting (i) the occurrence or non-occurrence of specific chain topologies in crystal structures; (ii) the occurrence of some chain topologies in a wide range of crystal structure arrangements and other chain topologies in only one or two structures; and (iii) the occurrence of specific chain topologies in abundant minerals versus the occurrence of other topologies only in rare minerals. Moreover, as *n* in *T*O_*n*_ can be calculated directly from any *^c^V_r_*, one can generate all non-isomorphic chain graphs for any particular chain stoichiometry, explicitly allowing comparison of observed structures with other possible structural arrangements of the same (or related) stoichiometry, and also allowing the prediction of possible structures for specific chemical compositions.

## Supplementary Material

Appendix B: MatLabR2019b code. DOI: 10.1107/S2053273322001747/uv5008sup1.pdf

Appendix C: Example for generating 2V23V2 chain graphs. DOI: 10.1107/S2053273322001747/uv5008sup2.pdf

Appendix D: Instructions for use of Appendices E, F, G. DOI: 10.1107/S2053273322001747/uv5008sup3.pdf

Appendix E: Matrix-element combinations and adjacency matrices. DOI: 10.1107/S2053273322001747/uv5008sup4.pdf

Appendix F: Matrix-element combinations and proto-graphs. DOI: 10.1107/S2053273322001747/uv5008sup5.pdf

Appendix G: Matrix-element combinations and chain graphs. DOI: 10.1107/S2053273322001747/uv5008sup6.pdf

## Figures and Tables

**Table 1:** Hierarchical ordering scheme of *^c^ *V*_r_* values where *r* = 1–∞ and *c* = 1–4

Rank	1	2	3	4
	^1^ *V*_r_	^1^ *V*_r_^2^ *V*_r_	^1^ *V*_r_^2^ *V*_r_^3^ *V*_r_	^1^ *V*_r_^2^ *V*_r_^3^ *V*_r_^4^ *V*_r_
	^2^ *V*_r_	^1^ *V*_r_^3^ *V*_r_	^1^ *V*_r_^2^ *V*_r_^4^ *V*_r_	
	^3^ *V*_r_	^1^ *V*_r_^4^ *V*_r_	^1^ *V*_r_^3^ *V*_r_^4^ *V*_r_	
	^4^ *V*_r_	^2^ *V*_r_^3^ *V*_r_	^2^ *V*_r_^3^ *V*_r_^4^ *V*_r_	
		^2^ *V*_r_^4^ *V*_r_		
		^3^ *V*_r_^4^ *V*_r_		

**Table 2:** Matrix-element combinations for vertex connectivity ^2^ *V*_2_^3^ *V*_2_ Italics: valid matrix-element combination. Bold: invalid matrix-element combination. Matrix-element combinations are written as (*m*_1_ × 1)(*m*_2_ × 2)(*m*_3_ × 2^1^)(*m*_4_ × 2^2^) where *m_i_* are the numbers of those matrix elements, and are numbered with square brackets and cited in the text accordingly.

# of 2’s, 2^1^’s and 2^2^’s					
0	1	2	3	4	5
[*1*] (10 × 1)	[*2*] (8 × 1)(1 × 2)	[*3*] (6 × 1)(2 × 2)	[*6*] (4 × 1)(3 × 2)	[*9*] (2 × 1)(4 × 2)	**[15]** (5 × 2)
		[*4*] (6 × 1)(2 × 2^1^)	[*7*] (4 × 1)(1 × 2)(2 × 2^1^)	[*10*] (2 × 1)(2 × 2)(2 × 2^1^)	**[16]** (1 × 2)(4 × 2^1^)
		[*5*] (6 × 1)(2 × 2^2^)	[*8*] (4 × 1)(1 × 2)(2 × 2^2^)	[*11*] (2 × 1)(2 × 2)(2 × 2^2^)	**[17]** (1 × 2)(2 × 2^1^)(2 × 2^2^)
				[*12*] (2 × 1)(4 × 2^1^)	**[18]** (1 × 2)(4 × 2^2^)
				[*13*] (2 × 1)(2 × 2^1^)(2 × 2^2^)	**[19]** (3 × 2)(2 × 2^1^)
				[*14*] (2 × 1)(4 × 2^2^)	**[20]** (3 × 2)(2 × 2^2^)

**Table 3:** Rules for determining which *^c^V_r_* expressions and/or directed proto-graphs will not produce additional non-isomorphic chain graphs For rules [2] and [6], vertices and edges of a single repeat unit are shown in yellow and green, respectively.

Rule or property	Examples	
[1] Any graph (*^c^V_r_*) with an odd number of vertices of odd degree is not possible. Any *^c^V_r_* where *e*_A_ is odd can be ignored	A ^1^ *V*_2_^2^ *V*_2_^3^ *V*_1_ graph is not possible as *e*_A_ = 9	
[2] Any two isomorphic directed proto-graphs in which the direction of each isomorphic wrapped edge is opposite will generate isomorphic chain graphs in which the translation axis is reversed. Only one of such directed proto-graphs needs to be considered	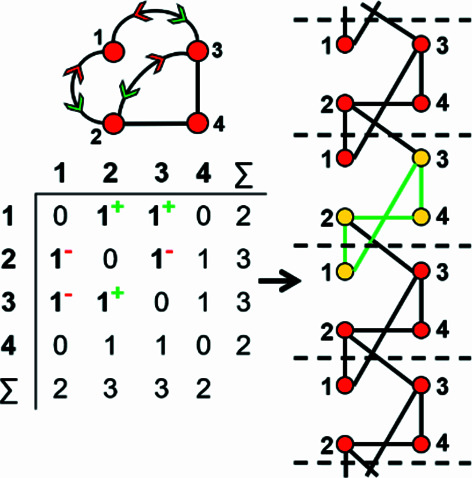	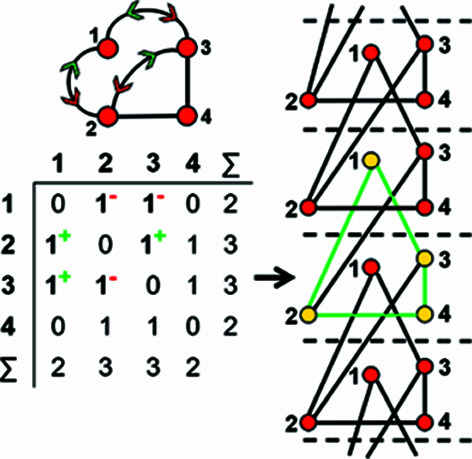
	Two isomorphic ^2^ *V*_2_^3^ *V*_2_ directed proto-graphs in which all equivalent edges are wrapped in opposite directions: the resultant chain graphs are isomorphic	
[3] D* vertices: if all edges connected to a vertex are wrapped in the same direction, that vertex is a D* vertex. Unwrapping a directed proto-graph with a D* vertex will not produce a new non-isomorphic chain graph. Unwrapping any edge linked to a vertex of degree 1 will never result in a new, non-isomorphic chain graph	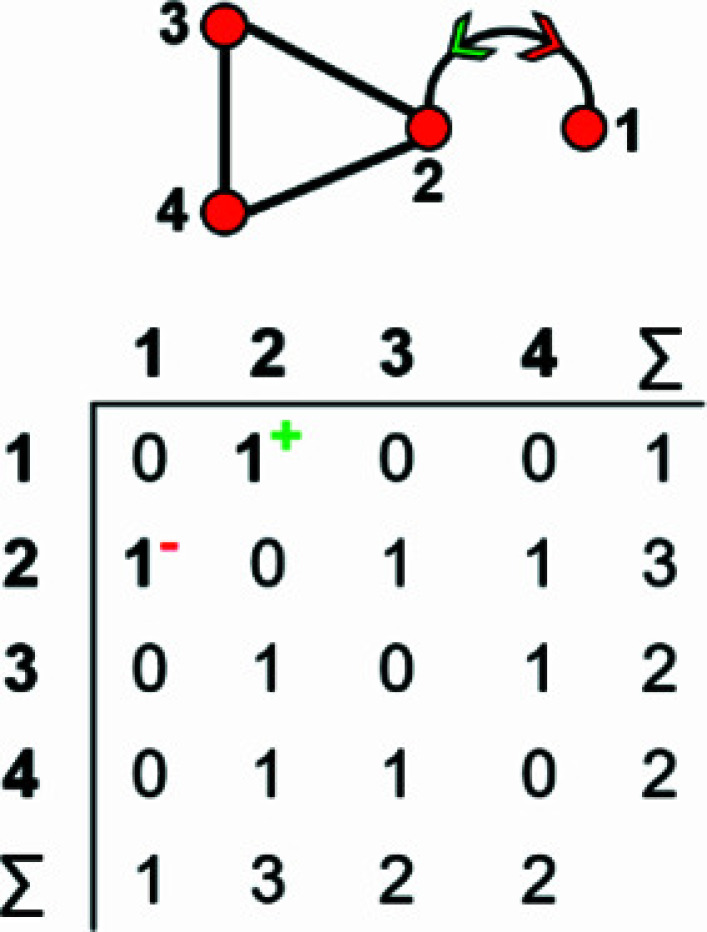	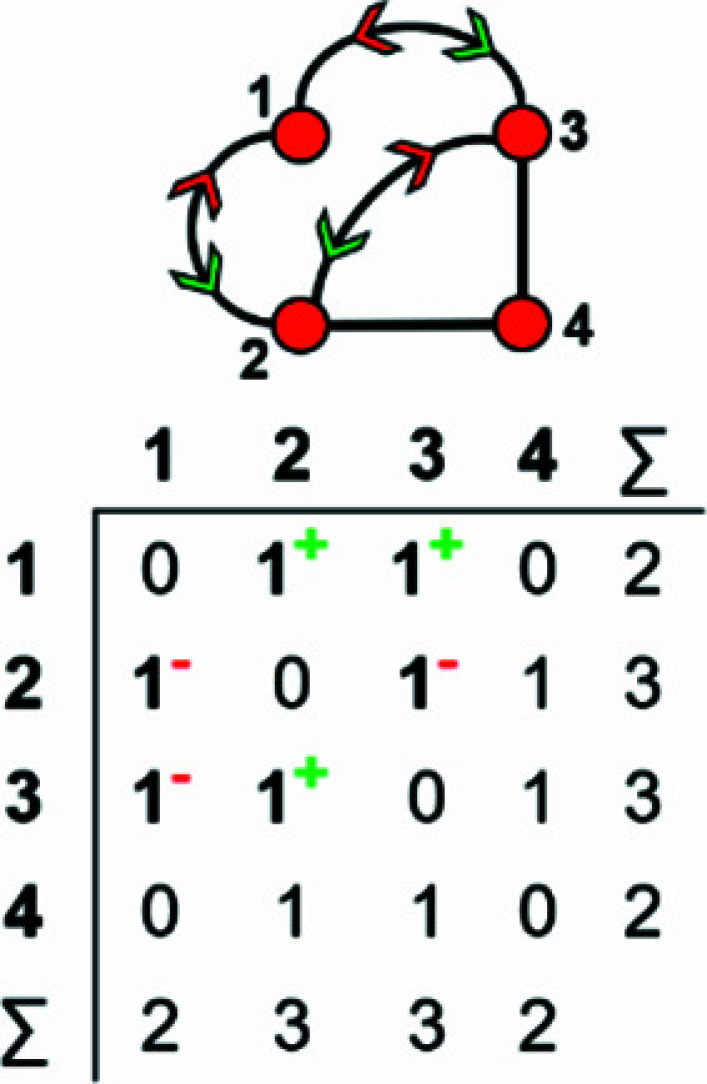
	Vertex 1 is a D* vertex. Assigning the edge of a 1-connected vertex as wrapped is not necessary as it will result in a D* vertex	Vertex 1 is a D* vertex
[4] D* vertices of degree 3: if a vertex of degree 3 has two edges that are wrapped in the same direction and a third edge that is not wrapped, this vertex is a D* vertex and the directed proto-graph will not form a new non-isomorphic chain graph once unwrapped	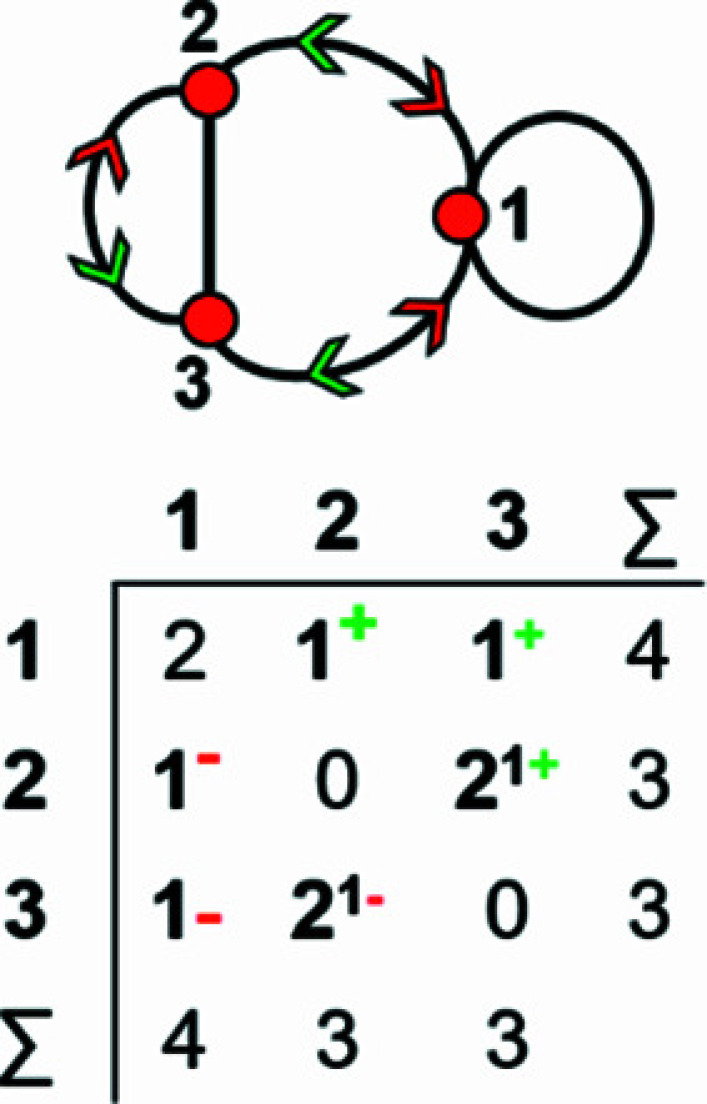	
	Vertex 3 is a D* vertex	
[5] D* vertices of degree 4: if a vertex of degree 4 has three edges that are wrapped in the same direction and a fourth edge that is not wrapped, this vertex is a D* vertex and the directed proto-graph will not form a new non-isomorphic chain graph once unwrapped	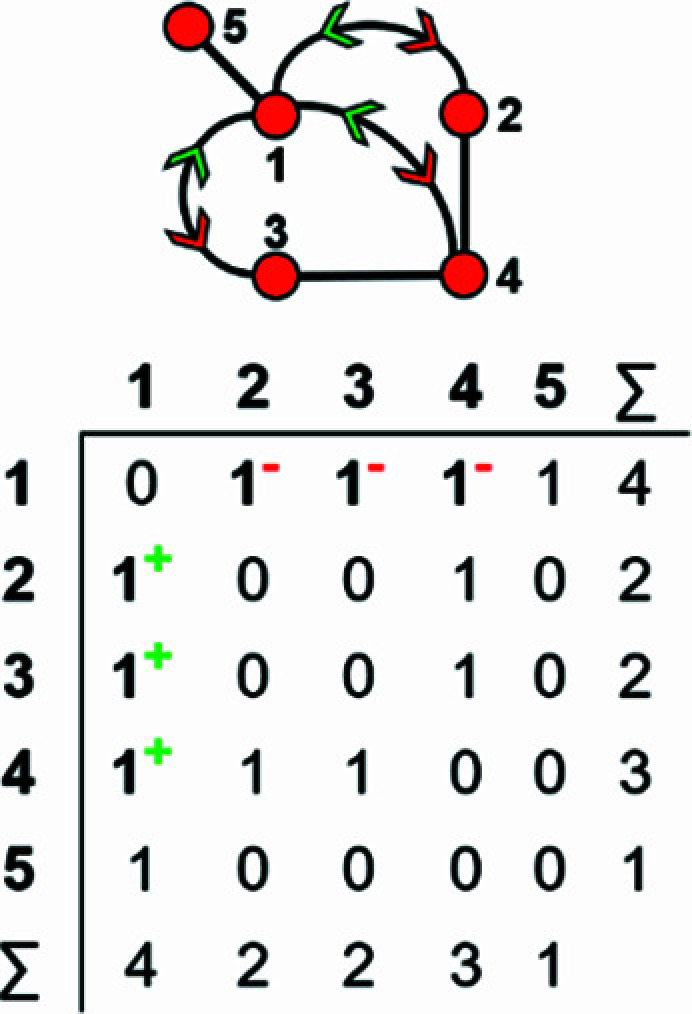	
	Vertex 1 is a D* vertex	
[6] Linear branches: unwrapping any edge of a linear branch will not produce a new non-isomorphic chain graph. This is not true for polygonal branches	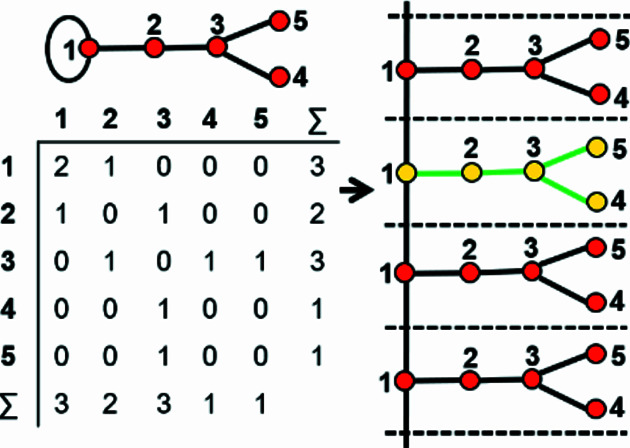	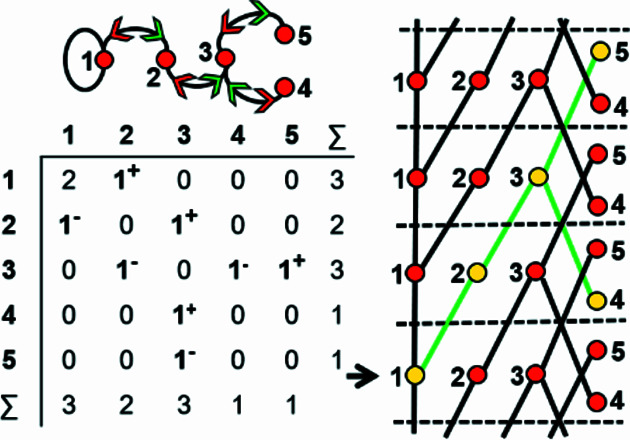
	Edges 1–2, 2–3, 3–5 and 3–4 comprise the linear branch. No edge of this branch is wrapped	Every edge of the linear branch is wrapped and the resultant chain graph is isomorphic with the chain graph to the left

## Appendix A Rules for determining which *^c^V_r_* expressions and/or directed proto-graphs will not produce additional non-isomorphic chain graphs

The details of the rules are provided in the form of Table 3 ▸.
