# Supplementary material for: Bond topology of chain, ribbon and tube silicates. Part I. Graph-theory generation of infinite one-dimensional arrangements of (TO4) n− tetrahedra
Source: Acta Crystallogr A Found Adv. 2022 Apr 4;78(Pt 3):212–33. doi: 10.1107/S2053273322001747 (PMC9062827; doi:10.1107/S2053273322001747)
Supplement: Supplementary file 1 [file a-78-00212-sup1.pdf]

FOUNDATIONS
ADVANCES

Volume 78 (2022)

Supporting information for article:

Bond topology of chain, ribbon and tube silicates. Part I. Graph-theory generation of infinite one-dimensional arrangements of $(TO4)^{n-}$ tetrahedra

Maxwell Christopher Day and Frank Christopher Hawthorne

Appendix B: MatLabR2019b Code

Line descriptions are shown in green

Characteristic polynomial equation of a matrix

```
>> syms x
A = sym([Av]);
polyA = charpoly(A,x)


A = adjacency matrix
Av = adjacency matrix after vertex deletion
```

Permute adjacency matrix and extract all unique permutations

```
>>

% Input matrix

A = [2 2 0 0; 1 2 1 1; 0 1 0 1; 0 1 1 0];

% Get index of lower triangle values (below diagonal)

trilMat = tril(reshape(1:numel(A),size(A)),-1);

trilIdx = trilMat(trilMat>0);

% List all permutations of lower triangle indices

trilPerms = perms(trilIdx);

% List accepted diagonal values

diagPermitted = [0,2];

% List all permutations of diagonal values (with replacement)

diagPerms = permn(diagPermitted,numel(diag(trilMat)));

% Loop through all lower-triangle-permutations and diagonal-permutations

diagMask = logical(eye(size(A)));

upperMask = triu(true(size(A)),1);

lowerMask = tril(true(size(A)),-1);

nPerms = size(trilPerms,1) * size(diagPerms,1); % total number of
permutations

A_PERM = nan([size(A),nPerms]);                 % all permutations
```

```
for i = 1:size(trilPerms,1)
    for j = 1:size(diagPerms,1)
        c = (i-1)*size(diagPerms,1)+j;
        base = zeros(size(A));
        base(trilIdx) = A(trilPerms(i,:));      % permute lower triangle
        base = base + tril(base,-1).';          % reflect lower triangle
base(diagMask) = diagPerms(j,:);        % permute diagonal (not changed, but
comes after reflection)

if ~issymmetric(base)
     % Sanity check: matrix is symmetric
     error('Matrix not symmetic. i = %d  j = %d',i,j)
        end
        A_PERM(:,:,c) = base;                   % store matrix
    end
end
goodRows = ismember(squeeze(sort(sum(A_PERM,2))).',[2 2 3 3],'rows');
A_PERM(:,:,~goodRows) = []
```

To extract distinct matrices from set of permuted matrices

```
>> megaMat =
squeeze(reshape(A_PERM,1,prod(size(A_PERM,[1,2])),size(A_PERM,3))).';
[~, unqRowIdx] = unique(megaMat,'rows');
A_PERM_UNQ = A_PERM(:,:,unqRowIdx)
```

Generate all valid, non-isomorphic proto-graphs for a given ${}^{c}V_{r}$

Remember to set *k* to an appropriate value ($<e_A/2$) for selected ${}^{c}V_{r}$

```
% Enter cVr as degList, i.e. 1V64V2
degList = [1 1 1 1 1 1 4 4];
maxEdgeCount = 2;

% Sort degrees list and get n, e from it
```

```
degList = sort(degList);
n = length(degList);
e = sum(degList)/2;

if e ~= floor(e)
    error('Sum of node degrees must be even')
end

% Generate a complete graph and get all its edges
g = graph(ones(n));
ed = g.Edges.EndNodes;

% Repeat edges as many times as allowed
ed = repelem(ed, maxEdgeCount, 1);

% Split number of edges to iterate over into two parts
k = 4; % Outer loop goes through all combinations of first k edges,
% inner loop goes through all combinations of remaining e-k edges.

firstSetOfRows = nchoosek(1:size(ed, 1), k);

% List of all graphs that are unique among permutations of the node numbers
uniqueGraphs = {};

for ll=1:size(firstSetOfRows, 1)
    firstSet = firstSetOfRows(ll, :);

    % Compute all sets of e unique numbers picked from 1 to the number of
rows of ed.
    remainingEdges = firstSet(end)+1:size(ed, 1);
    if isempty(remainingEdges)
        continue; % can't choose e-k edges from an empty set of available
edges
    elseif isscalar(remainingEdges)
        if e-k == 1
            lastSetsOfRows = remainingEdges;
        else
            continue; % not enough edges left to choose e-k edges from them
        end
    else
        lastSetsOfRows = nchoosek(firstSet(end)+1:size(ed, 1), e-k);
    end

    for ii=1:size(lastSetsOfRows, 1)

        % Construct graph ii
        rowCombination = [firstSet, lastSetsOfRows(ii, :)];
        assert(length(rowCombination) == length(unique(rowCombination)))
        edii = ed(rowCombination, :);
```

```
        g = graph(edii(:, 1), edii(:, 2), [], n);

        if ~isequal(sort(degree(g)), degList')
            % Skip this graph, doesn't satisfy degree condition
            continue;
        end

        % Compare to all existing non-isomorphic graphs
        isNew = true;
        for jj=1:length(uniqueGraphs)
            if isisomorphic(g, uniqueGraphs{jj})
                isNew = false;
                break;
            end
        end

        % It did not match any existing unique graph, add it as a new one
        if isNew
            uniqueGraphs{end+1} = g;
        end
    end
end

% Plot all unique graphs
tiledlayout flow
for ii=1:length(uniqueGraphs)
    nexttile
    plot(uniqueGraphs{ii});
end
```

Note: Remember that this code does not differentiate between curved and straight edges, and thus proto-graphs that correspond to matrix-element combinations that contain the matrix element $\mathbf{2}^1$ must be derived manually (see Section 9.4).
