# Supplementary material for: Bond topology of chain, ribbon and tube silicates. Part I. Graph-theory generation of infinite one-dimensional arrangements of (TO4) n− tetrahedra
Source: Acta Crystallogr A Found Adv. 2022 Apr 4;78(Pt 3):212–33. doi: 10.1107/S2053273322001747 (PMC9062827; doi:10.1107/S2053273322001747)

FOUNDATIONS
ADVANCES

Volume 78 (2022)

Supporting information for article:

Bond topology of chain, ribbon and tube silicates. Part I. Graph-theory generation of infinite one-dimensional arrangements of $(TO4)^{n-}$ tetrahedra

Maxwell Christopher Day and Frank Christopher Hawthorne

Appendix C: Generation of all non-isomorphic chain graphs with vertex connectivity ${}^2V_2{}^3V_2$ for the (10 × 1) matrix-element combination

The following text is repetitive, but it allows the interested reader to follow the generation process in detail for this single example.

Unwrapping 1 edge: We begin by assigning a single edge of the proto-graph in Fig. 32(*a*) as wrapped. When assigning a **single edge** to be unwrapped, all possible non-isomorphic directed proto-graphs are generated simply by assigning an edge as wrapped from each edge subset (Section 9.5) and thus we expect to produce two non-isomorphic directed proto-graphs by assigning one edge as wrapped from subsets [1] and [2] (Fig. 32*a*). The 1–3 edge of subset [1] is assigned as wrapped in the **+c** direction to produce directed proto-graph 1̲ in Fig. 32(*b*). The 1–2 edge of subset [2] is assigned as wrapped to produce directed proto-graph 2̲ in Fig. 32(*c*), which is isomorphic with a directed proto-graph produced by assigned the 1–4, 2–3 or 3–4 edge as wrapped (in either direction) as these edges also belong to edge subset [2]. In Fig. 33(*a*), directed proto-graph 1̲ is unwrapped and the resultant chain graph is shown in Fig. 33(*b*). In Fig. 33(*c*), directed proto-graph 2̲ is unwrapped and the resultant chain graph is shown in Fig. 33(*d*). When selecting a single edge to assign as wrapped, the number of possible non-isomorphic directed proto-graphs is equal to the number of edge subsets in the original proto-graph (Fig. 32). In Fig. 33, both non-isomorphic directed proto-graphs (1̲ and 2̲) generate non-isomorphic chain graphs. However, this is not always the case (as shown below) as two non-isomorphic directed proto-graphs may generate isomorphic chain graphs when **more than one edge** is unwrapped.

Unwrapping 2 edges: When assigning more than one edge as wrapped, one must assign all unique combinations of edges irrespective of the edge subsets to which they belong. We

continue by assigning two edges as wrapped in all possible unique combinations and directions. Edge combinations that result in edge subsets with D* vertices are marked with a red X in the following figures and are omitted from further consideration as they do not produce new non-isomorphic chain graphs when unwrapped.

First, one edge from subset [1] and one edge from subset [2] are assigned as wrapped in all unique combinations (Fig. 34*a*). There are four edge combinations in Fig. 34(*a*) that do not contain D* vertices and correspond to four isomorphic directed proto-graphs (labelled 3) and therefore only one of these directed proto-graphs must be unwrapped to ensure that all non-isomorphic chain graphs are generated for this edge combination. Although the labelling of vertices in each directed proto-graph (Fig. 34) is different, vertices 1 and 3 are isomorphic (vertex subset [1]) and vertices 2 and 4 are isomorphic (vertex subset [2]) and therefore vertex label 1 may be interchanged with vertex label 3 (or vice-versa) and vertex label 2 may be interchanged with vertex label 4 (or vice-versa) without generating a different graph. This graph property is shown in Fig. 34(*a*) where vertices of the same vertex subset (interchangeable vertices) have the same colour.

Next, two edges from subset [2] are assigned as wrapped in all possible unique combinations and directions. These edge combinations are shown in Figs. 34(*b*), (*c*) and (*d*); six of these combinations do not contain D* vertices and correspond to the three non-isomorphic directed proto-graphs: 4 (Fig. 34*b*), 5 (Fig. 34*c*) and 6 (Fig. 34*d*), respectively (the proto-graphs that correspond to edge combinations with D* vertices are not shown in this appendix). In Fig. 35(*a*), graph 3 is unwrapped; the resultant chain graph is shown in Fig. 35(*b*) and an untangled version of this chain graph is shown in Fig. 35(*c*). In Fig. 35(*d*), graph 4 is unwrapped; the resultant chain graph is shown in Fig. 35(*e*), and an untangled version of this chain graph is shown in Fig. 35(*f*). In Fig. 35(*g*), graph 5 is unwrapped and the resultant chain graph is shown in Fig. 35(*h*); untangling this chain graph (Fig. 35*i*) shows that it consists of two identical chains, each isomorphic with the chain graph in Fig. 33(*d*). In Fig. 35(*j*), graph 6 is unwrapped and the

resultant chain graph is shown in Fig. 35(*k*); untangling this chain graph (Fig. 35*l*) shows that it is isomorphic with the chain graph in Fig. 33(*b*). Thus, when unwrapping more than a single edge, any two non-isomorphic directed proto-graphs (*i.e.* Fig. 35*j* and Fig. 33*a*) may or may not produce isomorphic chain graphs once unwrapped, and two isomorphic directed proto-graphs will always produce isomorphic chain graphs once unwrapped. Any two edge combinations in which the direction of wrapped edges is opposite are redundant and only one of such edge combinations need be considered. For example, a proto-graph (Fig. 32*a*) in which the 1–3 and 1–2 edges are assigned as wrapped in the **+c** and **–c** direction, respectively, will result in a chain graph that is isomorphic with the chain graph produced by unwrapping the 1–3 and 1–2 edges in the **–c** and **+c** directions, respectively.

Unwrapping 3 edges: In Figs. 36(*a*)–(*f*), one edge from subset [1] and two edges from subset [2] are assigned as wrapped in all unique combinations. In Fig. 36(*a*), three edge combinations correspond to two non-isomorphic directed proto-graphs: 7 and 8, and in Fig. 36(*b*), three edge combinations correspond to three non-isomorphic directed proto-graphs: 7, 8 and 9 (two of which are isomorphic with 7 and 8 in Fig. 36*a*). In Figs. 36(*c*) and (*d*), two edge combinations correspond to one directed proto-graph: 10, and in Figs. 36(*e*) and (*f*), two edge combinations correspond to one directed proto-graph: 11. In Fig. 37, three edges from subset [2] are assigned as wrapped in all unique combinations and there are four edge combinations that correspond to the directed proto-graph: 12.

In Fig. 38(*a*), directed proto-graph 7 is unwrapped; the resultant chain graphs are shown in Fig. 38(*b*) and are isomorphic with the chain graphs in Figs. 35(*h*), (*i*). In Fig. 38(*c*), directed proto-graph 8 is unwrapped; the resultant chain graph is shown in Fig. 38(*d*) and inspection of the untangled version of these chain graphs (Fig. 38*e*) shows two separate chains, each isomorphic with the chain graph in Fig. 33(*b*). In Fig. 38(*f*), directed proto-graph 9 is unwrapped and the resultant chain graphs (Fig. 38*g*) are isomorphic with the chain graphs in Fig. 38(*b*) and

in Figs. 35(*h*), (*i*). In Fig. 38(*h*), directed proto-graph 10 is unwrapped; the resultant chain graph is shown in Fig. 38(*i*) and an untangled version of this chain graph is shown in Fig. 38(*j*). In Fig. 38(*k*), the directed proto-graph 11 is unwrapped; the resultant chain graphs are shown in Fig. 38(*l*) and inspection of the untangled version of these chain graphs (Fig. 38*m*) shows two separate chains, isomorphic with the chain graphs in Fig. 38(*e*). In Fig. 38(*n*), directed proto-graph 12 is unwrapped; the resultant chain graph is shown in Fig. 38(*o*) and an untangled version of this chain graph is shown in Fig. 38(*p*).

Unwrapping 4 edges: In Figs. 39(*a*)–(*d*), one edge from subset [1] and three edges from subset [2] are assigned as wrapped in all unique combinations and correspond to the four non-isomorphic directed proto-graphs: 13 (Figs. 39*a*, *c*, *d*), 14, (Figs. 39*a*, *b*, *c*), 15 (Figs. 39*b*) and 16 (Figs. 39*d*), respectively. In Fig. 40, four edges from subset [2] are assigned as wrapped in all unique combinations and correspond to the single directed proto-graph: 17.

In Fig 41(*a*), directed proto-graph 13 is unwrapped; the resultant chain graph is shown in Fig. 41(*b*), and an untangled version is shown in Fig. 41(*c*). In Fig. 41(*d*), directed proto-graph 14 is unwrapped; the resultant chain graph is shown in Fig. 41(*e*) and inspection of the untangled version (Fig. 41*f*) shows three separate chains, each isomorphic with the chain graph in Fig. 33(*d*). In Fig 41(*g*), the directed proto-graph 15 is unwrapped; the resultant chain graph is shown in Fig. 41(*h*) and an untangled version is shown in Fig. 41(*i*). In Fig. 41(*j*), directed proto-graph 16 is unwrapped; the resultant chain graphs are shown in Fig. 41(*k*) and inspection of the untangled version of these chains graphs (Fig. 41*l*) shows that they are isomorphic with the chain graphs in Figs. 41(*e*), (*f*). In Fig. 41(*m*), directed proto-graph 17 is unwrapped; the resultant chain graph is shown in Fig. 41(*n*) and the untangled version (Fig. 41*o*) shows two chain graphs, each isomorphic with the chain graph in Fig. 35(*f*).

Unwrapping 5 edges: In Figs. 42(*a*), (*b*), all edges are unwrapped; one edge from subset [1] and four edges from subset [2] are assigned as wrapped in all unique combinations and correspond to the three non-isomorphic directed proto-graphs: 18, 19, (Figs. 42*a*) and 20 (Fig. 42*b*). In Fig 43(*a*), directed proto-graph 18 is unwrapped; the resultant chain graph is shown in Fig. 43(*b*) and an untangled version is shown in Fig. 43(*c*). In Fig 43(*d*), directed proto-graph 19 is unwrapped; the resultant chain graph is shown in Fig. 43(*e*) and an untangled version of this chain graph is shown in Fig. 43(*f*). In Fig. 43(*g*), directed proto-graph 20 is unwrapped; the resultant chain graph is shown in Fig. 43(*h*) and inspection of the untangled version of this chain graph shows three chains, each isomorphic with the chain graph in Fig. 33(*b*). All non-isomorphic chain graphs with vertex connectivity ${}^{2}V_{2}{}^{3}V_{2}$ for the matrix-element combinations (10 × 1) have been derived. One may derive all other non-isomorphic chain graph with this vertex connectivity by completing this method for the other 17 matrix-element combinations (Fig. 13).

Figure 32

For vertex connectivity $^{2}V_{2}{}^{3}V_{2}$, (*a*) the proto-graph for the matrix-element combination (10 × 1) and the corresponding vertex and edge subsets, (*b*) the directed proto-graph 1 in which the 1–3 edge is assigned as wrapped in the **+c** direction and the corresponding edge subsets, and (*c*) the directed proto-graph 2 in which the 1–2 edge is assigned as wrapped in the **+c** direction and the corresponding edge subsets. When assigning one edge of this proto-graph as wrapped, there are two unique edge combinations which result in two non-isomorphic wrapped graphs 1 and 2 and two non-isomorphic chain graphs. Green and red arrows indicate the direction of wrapped (curved) edges.

32

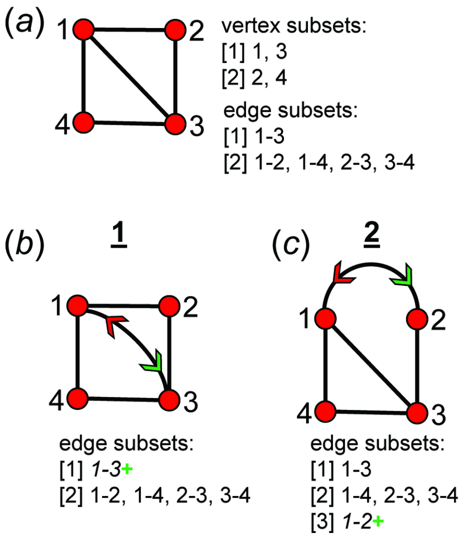

Figure 33

(*a*) Directed proto-graph 1, the corresponding adjacency matrix and edge subsets, and (*b*) the resultant chain graph. (*c*) Directed proto-graph 2, the corresponding adjacency matrix and edge subsets, and (*d*) the resultant chain graph. Green and red arrows and matrix-element superscripts in (*a*) and (*c*) indicate the number and direction of wrapped edges, and dashed black lines in (*b*) and (*d*) outline the repeat units of each chain graph.

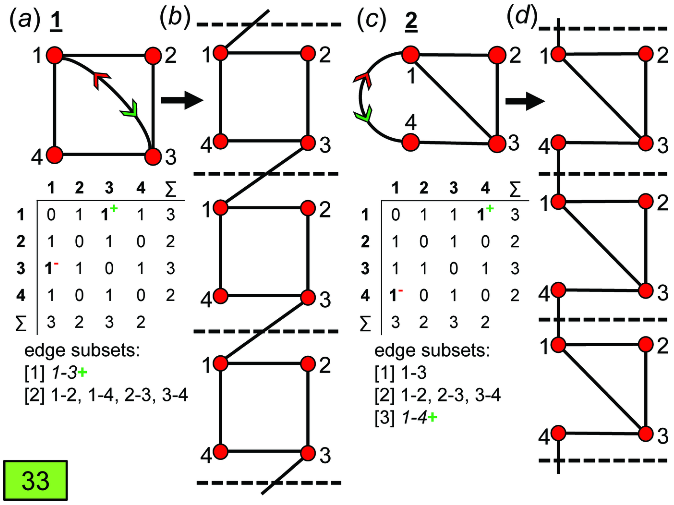

33

Figure 34

(*a*) Edge combinations and the corresponding directed proto-graph 3 produced by assigning one edge from edge subset [1] and one edge from subset [2] as wrapped. Edge combinations and the corresponding directed proto-graphs: (*b*) 4, (*c*) 5 and (*d*) 6, produced by assigning two edges from edge subset [2] as wrapped. Edge combinations that result in D* vertices are marked with a red X and equivalent edge combinations (those that produce isomorphic directed proto-graphs) are labelled using the same underlined number. Green and red arrows indicate the direction of wrapped (curved) edges.

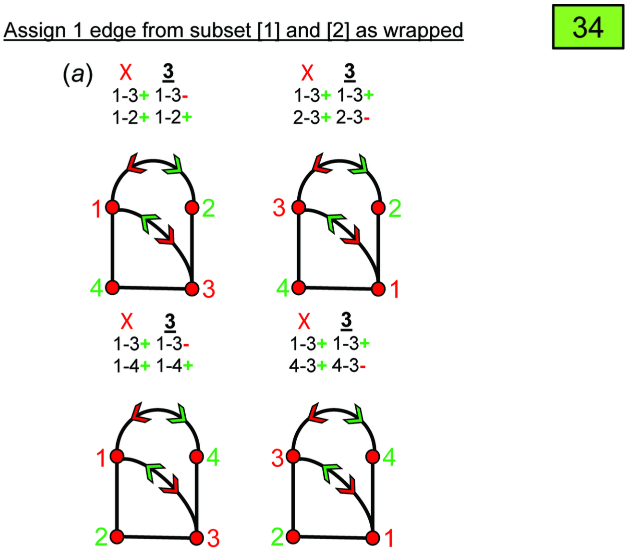

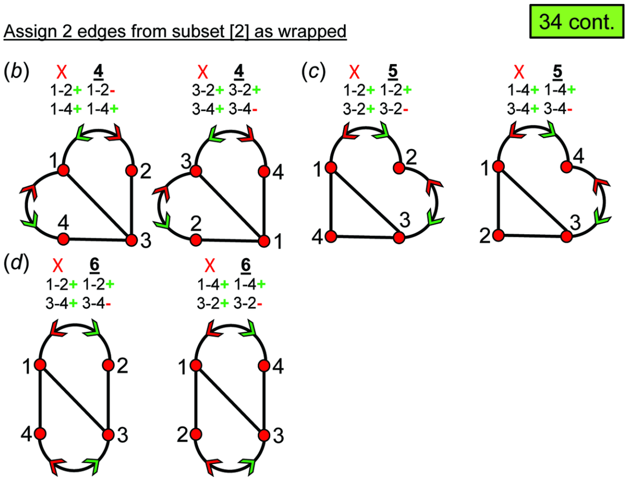

Figure 35

The directed proto-graphs (*a*) 3, (*d*) 4, (*g*) 5 and (*j*) 6, the corresponding adjacency matrices and edge subsets. The chain graphs and untangled chain graphs produced by unwrapping directed proto-graphs (*b*, *c*) 3, (*e*, *f*) 4, (*h*, *i*) 5 and (*k*, *l*) 6, respectively. Legend as in Fig. 33.

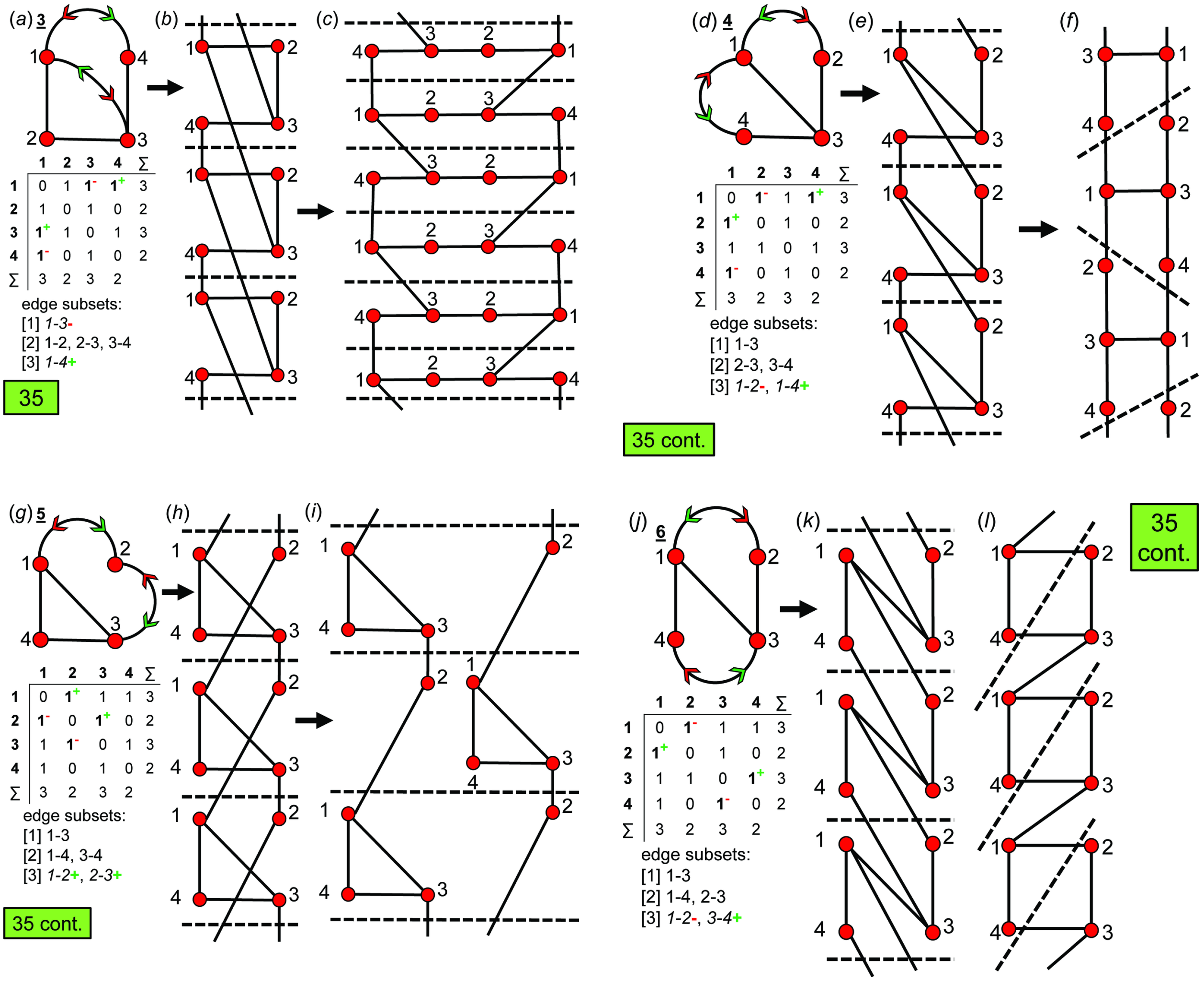

(*a*) 3

	1	2	3	4	Σ
**1**	0	1	1^-	1^+	3
**2**	1	0	1	0	2
**3**	1^+	1	0	1	3
**4**	1^-	0	1	0	2
Σ	3	2	3	2	

edge subsets:
[1] *1-3-*
[2] 1-2, 2-3, 3-4
[3] *1-4+*

(*d*) 4

	1	2	3	4	Σ
**1**	0	1^-	1	1^+	3
**2**	1^+	0	1	0	2
**3**	1	1	0	1	3
**4**	1^-	0	1	0	2
Σ	3	2	3	2	

edge subsets:
[1] 1-3
[2] 2-3, 3-4
[3] *1-2-*, *1-4+*

(*g*) 5

	1	2	3	4	Σ
**1**	0	1^+	1	1	3
**2**	1^-	0	1^+	0	2
**3**	1	1^-	0	1	3
**4**	1	0	1	0	2
Σ	3	2	3	2	

edge subsets:
[1] 1-3
[2] 1-4, 3-4
[3] *1-2+*, *2-3+*

(*j*) 6

	1	2	3	4	Σ
**1**	0	1^-	1	1	3
**2**	1^+	0	1	0	2
**3**	1	1	0	1^+	3
**4**	1	0	1^-	0	2
Σ	3	2	3	2	

edge subsets:
[1] 1-3
[2] 1-4, 2-3
[3] *1-2-*, *3-4+*

Figure 36

Edge combinations and the corresponding directed proto-graphs (*a*) 7 and 8, (*b*) 7, 8 and 9, (*c*) 10, (*d*) 10, (*e*) 11 and (*f*) 11 produced by assigning one edge from edge subset [1] and two edges from subset [2] as wrapped. Legend as in Fig. 34.

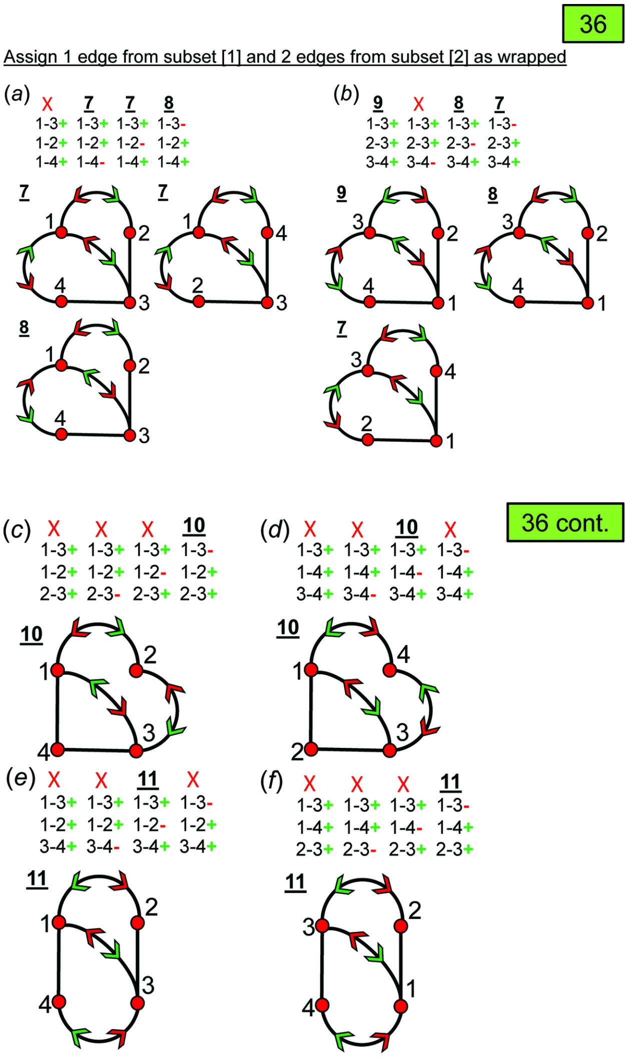

Figure 37

Edge combinations and the corresponding directed proto-graph 12 produced by assigning three edges from edge subset [2] as wrapped. Legend as in Fig. 34.

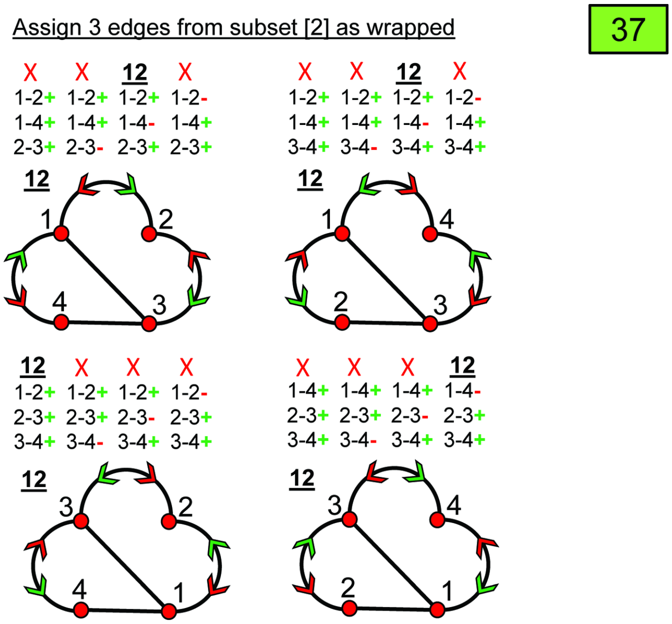

Figure 38

The directed proto-graphs (*a*) 7, (*c*) 8, (*f*) 9, (*h*) 10, (*k*) 11 and (*n*) 12, the corresponding adjacency matrices and edge subsets. The chain graphs and untangled chain graphs produced by unwrapping directed proto-graph (*b*) 7, (*d*, *e*) 8, (*g*) 9, (*i*, *i*) 10, (*l*, *m*) 11 and (*o*, *p*) 12, respectively. Legend as in Fig. 33.

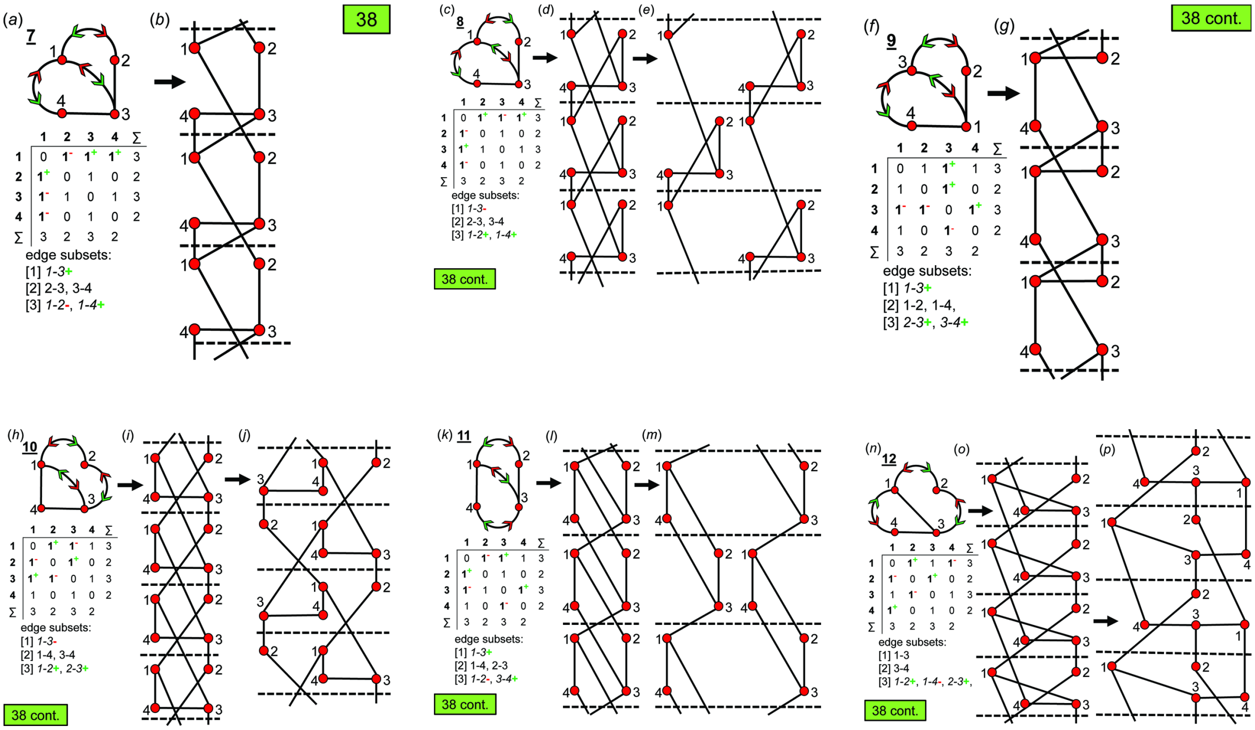

Figure 39

Edge combinations and the corresponding directed proto-graphs (*a*) 13 and 14, (*b*) 14 and 15, (*c*) 13 and 14, and (*d*) 13 and 16, produced by assigning one edge from edge subset [1] and three edges from subset [2] as wrapped. Legend as in Fig. 34.

Assign 1 edge from subset [1] and 3 edges from subset [2] as wrapped

(*a*)

X	X	X	X	**13**	X	**14**	X
1-3+	1-3+	1-3+	1-3+	1-3-	1-3+	1-3+	1-3+
1-2+	1-2+	1-2+	1-2-	1-2+	1-2+	1-2-	1-2-
1-4+	1-4+	1-4-	1-4+	1-4+	1-4-	1-4+	1-4-
2-3+	2-3-	2-3+	2-3+	2-3+	2-3-	2-3-	2-3+

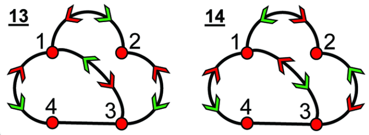

(*b*)

X	X	**14**	X	X	X	X	**15**
1-3+	1-3+	1-3+	1-3+	1-3-	1-3+	1-3+	1-3+
1-2+	1-2+	1-2+	1-2-	1-2+	1-2+	1-2-	1-2-
1-4+	1-4+	1-4-	1-4+	1-4+	1-4-	1-4+	1-4-
3-4+	3-4-	3-4+	3-4+	3-4+	3-4-	3-4-	3-4+

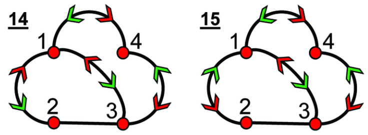

39

39 cont.

(*c*)

X	X	X	X	**14**	X	X	**13**
1-3+	1-3+	1-3+	1-3+	1-3-	1-3+	1-3+	1-3+
1-2+	1-2+	1-2+	1-2-	1-2+	1-2+	1-2-	1-2-
2-3+	2-3+	2-3-	2-3+	2-3+	2-3-	2-3+	2-3-
3-4+	3-4-	3-4+	3-4+	3-4+	3-4-	3-4-	3-4+

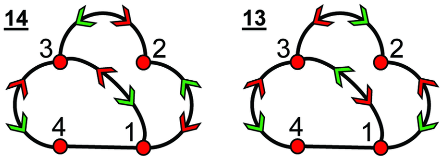

(*d*)

X	X	X	**16**	X	X	X	**13**
1-3+	1-3+	1-3+	1-3+	1-3-	1-3+	1-3+	1-3+
1-4+	1-4+	1-4+	1-4-	1-4+	1-4+	1-4-	1-4-
2-3+	2-3+	2-3-	2-3+	2-3+	2-3-	2-3+	2-3-
3-4+	3-4-	3-4+	3-4+	3-4+	3-4-	3-4-	3-4+

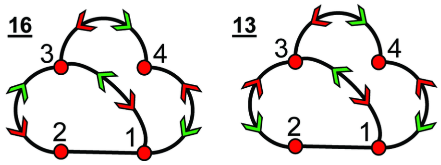

Figure 40

Edge combinations and the corresponding directed proto-graph 17 produced by assigning four edges from edge subset [2]. Legend as in Fig. 34.

40

Assign 4 edges from subset [2] as wrapped

X	X	**17**	X	X	X	X	X
3-4+	3-4+	3-4+	3-4+	3-4-	3-4+	3-4+	3-4+
1-2+	1-2+	1-2+	1-2-	1-2+	1-2+	1-2-	1-2-
1-4+	1-4+	1-4-	1-4+	1-4+	1-4-	1-4+	1-4-
2-3+	2-3-	2-3+	2-3+	2-3+	2-3-	2-3-	2-3+

17

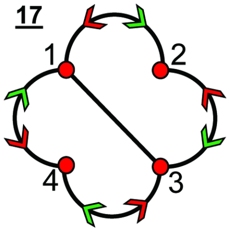

Figure 41

The directed proto-graphs (*a*) 13, (*d*) 14, (*g*) 15, (*j*) 16, and (*m*) 17, the corresponding adjacency matrices and edge subsets. The chain graphs and untangled chain graphs produced by unwrapping directed proto-graphs (*b, c*) 13, (*e*, *f*) 14, (*h, i*) 15, (*k*, *l*) 16 and (*n*, *o*) 17, respectively. Legend as in Fig. 33.

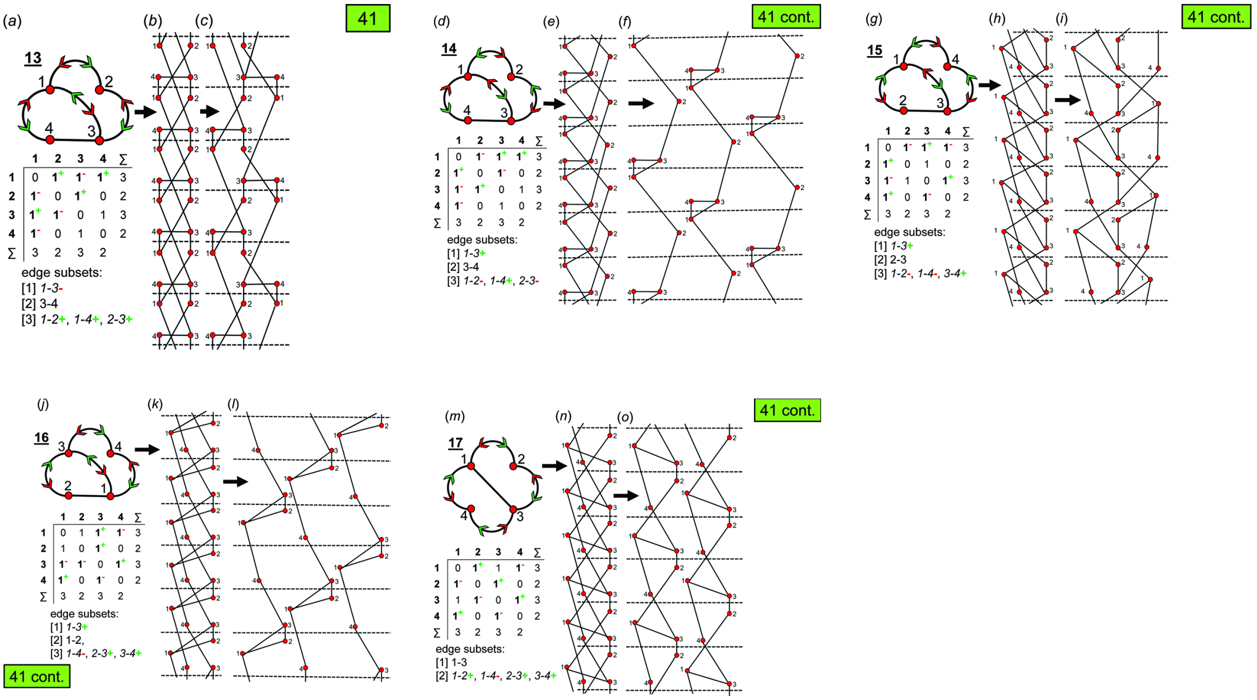

Figure 42

Edge combinations and the corresponding directed proto-graphs (*a*) <u>18</u> and <u>19</u>, and (*b*) <u>20</u>, produced by assigning one edge from edge subset [1] and four edges from subset [2] as wrapped. Legend as in Fig. 34.

42

<u>Assign 1 edge from subset [1] and 4 edges from subset [2] as wrapped</u>

(*a*)

X	X	X	**<u>18</u>**	X	X	X	**<u>19</u>**
3-4+	3-4+	3-4+	3-4+	3-4+	3-4-	3-4+	3-4+
1-2+	1-2+	1-2+	1-2+	1-2-	1-2+	1-2+	1-2+
1-4+	1-4+	1-4+	1-4-	1-4+	1-4+	1-4+	1-4-
2-3+	2-3+	2-3-	2-3+	2-3+	2-3+	2-3-	2-3+
1-3+	1-3-	1-3+	1-3+	1-3+	1-3+	1-3-	1-3-

<u>18</u>

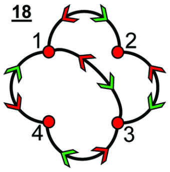

<u>19</u>

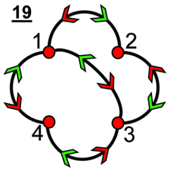

42 cont.

(*b*)

X	**<u>20</u>**	X	X	X	X	X	X
3-4+	3-4-	3-4+	3-4+	3-4-	3-4-	3-4-	3-4+
1-2-	1-2+	1-2+	1-2-	1-2+	1-2-	1-2+	1-2-
1-4+	1-4+	1-4-	1-4+	1-4+	1-4+	1-4-	1-4-
2-3+	2-3+	2-3-	2-3-	2-3-	2-3+	2-3+	2-3+
1-3-	1-3-	1-3-	1-3-	1-3-	1-3-	1-3-	1-3-

<u>20</u>

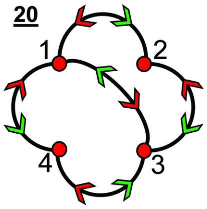

Figure 43

The directed proto-graphs (*a*) 18, (*d*) 19 and (*g*) 20, the corresponding adjacency matrices and edge subsets. The chain graphs and untangled chain graphs produced by unwrapping directed proto-graph (*b, c*) 18, (*e*, *f*) 19 and (*h*, *i*) 20, respectively. Legend as in Fig. 34.

43

(*a*)

18

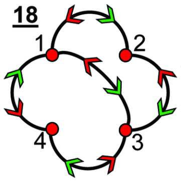

	1	2	3	4	∑
**1**	0	1^+	1^+	1^-	3
**2**	1^-	0	1^+	0	2
**3**	1^-	1^-	0	1^+	3
**4**	1^+	0	1^-	0	2
∑	3	2	3	2	

edge subsets:
[1] *1-3*+
[2] *1-2*+, *1-4*-, *2-3*+, *3-4*+

(*b*) (*c*)

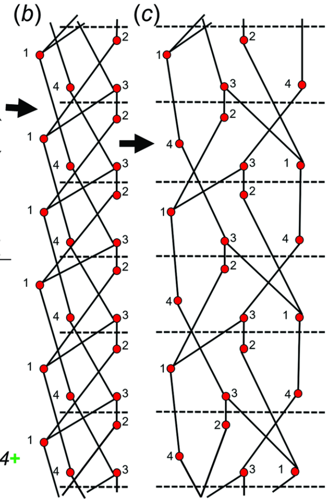

43 cont.

(*d*)

19

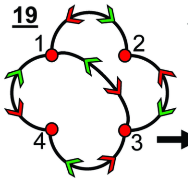

	1	2	3	4	∑
**1**	0	1^+	1^-	1^-	3
**2**	1^-	0	1^+	0	2
**3**	1^+	1^-	0	1^+	3
**4**	1^+	0	1^-	0	2
∑	3	2	3	2	

edge subsets:
[1] *1-3*-
[2] *1-2*+, *1-4*-, *2-3*+, *3-4*+

(*e*) (*f*)

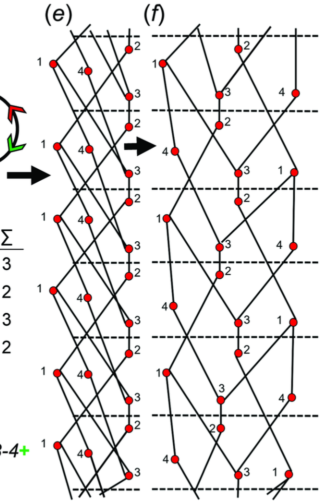

43 cont.

(*g*)

20

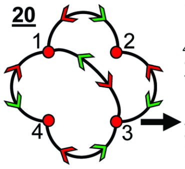

	1	2	3	4	∑
**1**	0	1^+	1^-	1^+	3
**2**	1^-	0	1^+	0	2
**3**	1^+	1^-	0	1^-	3
**4**	1^-	0	1^+	0	2
∑	3	2	3	2	

edge subsets:
[1] *1-3*-
[2] *1-2*+, *1-4*+, *2-3*+, *3-4*-

(*h*) (*i*)

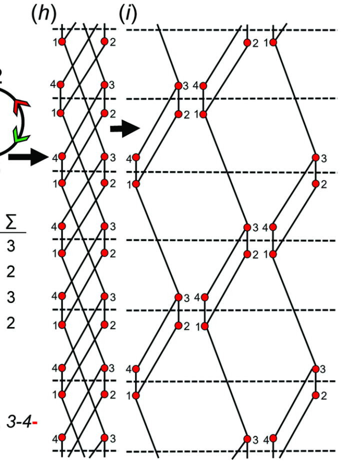

Supplement: Supplementary file 2 [file a-78-00212-sup2.pdf]
