# Supplementary material for: Bond topology of chain, ribbon and tube silicates. Part I. Graph-theory generation of infinite one-dimensional arrangements of (TO4) n− tetrahedra
Source: Acta Crystallogr A Found Adv. 2022 Apr 4;78(Pt 3):212–33. doi: 10.1107/S2053273322001747 (PMC9062827; doi:10.1107/S2053273322001747)
Supplement: Supplementary file 3 [file a-78-00212-sup3.pdf]

Acta Cryst A

FOUNDATIONS
ADVANCES

Volume 78 (2022)

Supporting information for article:

Bond topology of chain, ribbon and tube silicates. Part I. Graph-theory generation of infinite one-dimensional arrangements of $(TO4)^{n-}$ tetrahedra

Maxwell Christopher Day and Frank Christopher Hawthorne

Appendix D: Instructions for Appendices E, F and G

For vertex connectivities (^cV_r) in which $\sum r \leq 8$, all valid matrix-element combinations and their corresponding adjacency matrices are listed in Appendix E. All proto-graphs that correspond to each adjacency matrix in Appendix E are compiled in Appendix F. All non-isomorphic chain graphs are compiled in Appendix G. Clusters will only be included in Appendix G if they are non-isomorphic with the proto-graph (Appendix F) from which they were generated. In Appendices E, F and G, ^cV_r expressions are listed in accordance with the hierarchical ordering scheme given in Table 1 (section 7); note that ^cV_r where e_A is odd cannot form graphs and are not listed in the Appendices. For certain ^cV_r where the number of edges (e) is relatively large, deriving all distinct matrices, the corresponding non-isomorphic proto-graphs and chain graphs is computationally intensive and results in an unmanageable number of graphs, these ^cV_r are indicated as NG (not generated). For some ^cV_r where e is relatively large, all adjacency matrices and proto-graphs are generated but the corresponding chain graphs are not shown and indicated as NG in Appendix G.

For each ^cV_r (where e_A is even) only valid matrix-element combinations are listed and are written in the format (m_1 x **1**, m_2 x **2**, m_3 x $\mathbf{2^1}$, m_4 x $\mathbf{2^2}$) where m_i are the numbers of those matrix elements in the adjacency matrix of interest. In Appendix E, matrix-element combinations that correspond to more than one distinct matrix are listed as (m_1 x **1**, m_2 x **2**, m_3 x $\mathbf{2^1}$, m_4 x $\mathbf{2^2}$) **a,** (m_1 x **1**, m_2 x **2**, m_3 x $\mathbf{2^1}$, m_4 x $\mathbf{2^2}$) **b,** (m_1 x **1**, m_2 x **2**, m_3 x $\mathbf{2^1}$, m_4 x $\mathbf{2^2}$) **c**…etc. and the corresponding proto-graphs are labelled the same way in Appendix F. In Appendix G, matrix-element combinations that correspond to more than one non-isomorphic chain graph are listed as above or as (m_1 x **1**, m_2 x **2**, m_3 x $\mathbf{2^1}$, m_4 x $\mathbf{2^2}$) **a-1,** (m_1 x **1**, m_2 x **2**, m_3 x $\mathbf{2^1}$, m_4 x $\mathbf{2^2}$) **a-2,** (m_1 x **1**, m_2 x **2**, m_3 x $\mathbf{2^1}$, m_4 x $\mathbf{2^2}$) **a-3**…etc. Non-isomorphic directed proto-graphs are not listed in the appendices as the edge combinations (number and direction of wrapped edges) used to generate a given chain graph may be derived by simply constructing an adjacency matrix for any chain graph of interest (section 5).

Example: $^1V_3{}^3V_3$

Consider $^1V_3{}^3V_3$ as an example of how to use Appendices E, F and G. Here, there are eight valid matrix-element combinations that correspond to 15 distinct matrices, and the element combinations that correspond to more than one matrix are labelled as described above in Appendix E. One may then locate the proto-graph to which each of these 15 matrices corresponds in Appendix F, for example the matrix labelled (8 x **1**, 2 x $\mathbf{2^1}$) **b** corresponds to the proto-graph labelled (8 x **1**, 2 x $\mathbf{2^1}$) **b**. One may then locate all non-isomorphic chain graphs generated from a given proto-graph in Appendix G, for example the proto-graph labelled (8 x **1**, 2 x $\mathbf{2^1}$) **b** produces three chain graphs labelled (8 x **1**, 2 x $\mathbf{2^1}$) **b-1,** (8 x **1**, 2 x $\mathbf{2^1}$) **b-2** and (8 x **1**, 2 x $\mathbf{2^1}$) **b-3**.
