# Supplementary material for: Bond topology of chain, ribbon and tube silicates. Part I. Graph-theory generation of infinite one-dimensional arrangements of (TO4) n− tetrahedra
Source: Acta Crystallogr A Found Adv. 2022 Apr 4;78(Pt 3):212–33. doi: 10.1107/S2053273322001747 (PMC9062827; doi:10.1107/S2053273322001747)
Supplement: Supplementary file 4 [file a-78-00212-sup4.pdf]

Acta Cryst A FOUNDATIONS ADVANCES

Volume 78 (2022)

Supporting information for article:

Bond topology of chain, ribbon and tube silicates. Part I. Graph-theory generation of infinite one-dimensional arrangements of $(TO4)^{n-}$ tetrahedra

Maxwell Christopher Day and Frank Christopher Hawthorne

Appendix E. Matrix-element combinations and associated adjacency matrices for vertex connectivities ($^{c}V_{r}$) where $\sum r \leq 8$.

$^{c}V_{r}$	
**Rank 1**	
$^{0}V_{1\text{-}8}$	isolated tetrahedra (nesosilicates)

$^{1}V_{2}$

(2x**1**)

	1	2
1	0	1
2	1	0

$^{1}V_{4}$

(4x**1**)

	1	2	3	4
1	0	1	0	0
2	1	0	0	0
3	0	0	0	1
4	0	0	1	0

$^{1}V_{6}$

(6x**1**)

	1	2	3	4	5	6
1	0	1	0	0	0	0
2	1	0	0	0	0	0
3	0	0	0	1	0	0
4	0	0	1	0	0	0
5	0	0	0	0	0	1
6	0	0	0	0	1	0

$^{1}V_{8}$

(8x**1**)

	1	2	3	4	5	6	7	8
1	0	1	0	0	0	0	0	0
2	1	0	0	0	0	0	0	0
3	0	0	0	1	0	0	0	0
4	0	0	1	0	0	0	0	0
5	0	0	0	0	0	1	0	0
6	0	0	0	0	1	0	0	0
7	0	0	0	0	0	0	0	1
8	0	0	0	0	0	0	1	0

$^2V_1$

(1x**2**)

	1
1	2

$^2V_2$

(2x**2**)

	1	2
1	2	0
2	0	2

(2x$\mathbf{2}^1$)

	1	2
1	0	2^1
2	2^1	0

(2x$\mathbf{2}^2$)

	1	2
1	0	2^2
2	2^2	0

$^2V_3$

(6x**1**)

	1	2	3
1	0	1	1
2	1	0	1
3	1	1	0

(3x**2**)

	1	2	3
1	2	0	0
2	0	2	0
3	0	0	2

(1x**2**, 2x$\mathbf{2}^1$)

	1	2	3
1	0	2^1	0
2	2^1	0	0
3	0	0	2

(1x2, 2x$\mathbf{2}^2$)

	1	2	3
1	0	2^2	0
2	2^2	0	0
3	0	0	2

$^2V_4$

(8x**1**)

	1	2	3	4
1	0	1	0	1
2	1	0	1	0
3	0	1	0	1
4	1	0	1	0

(6x**1**,1x**2**)

	1	2	3	4
1	2	0	0	0
2	0	0	1	1
3	0	1	0	1
4	0	1	1	0

(4x**2**)

	1	2	3	4
1	2	0	0	0
2	0	2	0	0
3	0	0	2	0
4	0	0	0	2

(2x**2**, 2x$\mathbf{2}^1$)

	1	2	3	4
1	0	2^1	0	0
2	2^1	0	0	0
3	0	0	2	0
4	0	0	0	2

(2x**2**, 2x$\mathbf{2}^2$)

	1	2	3	4
1	0	2^2	0	0
2	2^2	0	0	0
3	0	0	2	0
4	0	0	0	2

(4x$\mathbf{2}^1$)

	1	2	3	4
1	0	2^1	0	0
2	2^1	0	0	0
3	0	0	0	2^1
4	0	0	2^1	0

(2x$\mathbf{2}^1$, 2x$\mathbf{2}^2$)

	1	2	3	4
1	0	2^2	0	0
2	2^2	0	0	0
3	0	0	0	2^1
4	0	0	2^1	0

(4x$\mathbf{2}^2$)

	1	2	3	4
1	0	2^2	0	0
2	2^2	0	0	0
3	0	0	0	2^2
4	0	0	2^2	0

$^2V_5$

(10x**1**)

	1	2	3	4	5
1	0	1	1	0	0
2	1	0	0	1	0
3	1	0	0	0	1
4	0	1	0	0	1
5	0	0	1	1	0

(8x**1**, 1x**2**)

	1	2	3	4	5
1	2	0	0	0	0
2	0	0	0	1	1
3	0	0	0	1	1
4	0	1	1	0	0
5	0	1	1	0	0

(6x**1**, 2x**2**)

	1	2	3	4	5
1	2	0	0	0	0
2	0	2	0	0	0
3	0	0	0	1	1
4	0	0	1	0	1
5	0	0	1	1	0

(6x**1**, 2x$\mathbf{2}^1$)

	1	2	3	4	5
1	0	1	1	0	0
2	1	0	1	0	0
3	1	1	0	0	0
4	0	0	0	0	2^1
5	0	0	0	2^1	0

(6x**1**, 2x$\mathbf{2}^2$)

	1	2	3	4	5
1	0	1	1	0	0
2	1	0	1	0	0
3	1	1	0	0	0
4	0	0	0	0	2^2
5	0	0	0	2^2	0

(5x**2**)

	1	2	3	4	5
1	2	0	0	0	0
2	0	2	0	0	0
3	0	0	2	0	0
4	0	0	0	2	0
5	0	0	0	0	2

(3x**2**, 2x$\mathbf{2^1}$)

	1	2	3	4	5
1	0	2^1	0	0	0
2	2^1	0	0	0	0
3	0	0	2	0	0
4	0	0	0	2	0
5	0	0	0	0	2

(3x**2**, 2x$\mathbf{2^2}$)

	1	2	3	4	5
1	0	2^2	0	0	0
2	2^2	0	0	0	0
3	0	0	2	0	0
4	0	0	0	2	0
5	0	0	0	0	2

(1x**2**, 4x$\mathbf{2^1}$)

	1	2	3	4	5
1	0	2^1	0	0	0
2	2^1	0	0	0	0
3	0	0	0	2^1	0
4	0	0	2^1	0	0
5	0	0	0	0	2

(1x**2**, 2x$\mathbf{2^1}$, 2x$\mathbf{2^2}$)

	1	2	3	4	5
1	0	2^1	0	0	0
2	2^1	0	0	0	0
3	0	0	0	2^2	0
4	0	0	2^2	0	0
5	0	0	0	0	2

(1x**2**, 4x$\mathbf{2^2}$)

	1	2	3	4	5
1	0	2^2	0	0	0
2	2^2	0	0	0	0
3	0	0	0	2^2	0
4	0	0	2^2	0	0
5	0	0	0	0	2

$^2V_6$

(12x**1**) **a**

	1	2	3	4	5	6
1	0	1	1	0	0	0
2	1	0	0	1	0	0
3	1	0	0	0	0	1
4	0	1	0	0	1	0
5	0	0	0	1	0	1
6	0	0	1	0	1	0

(12x**1**) **b**

	1	2	3	4	5	6
1	0	1	1	0	0	0
2	1	0	1	0	0	0
3	1	1	0	0	0	0
4	0	0	0	0	1	1
5	0	0	0	1	0	1
6	0	0	0	1	1	0

(10x**1**, 1x**2**)

	1	2	3	4	5	6
1	0	1	1	0	0	0
2	1	0	0	0	1	0
3	1	0	0	1	0	0
4	0	0	1	0	1	0
5	0	1	0	1	0	0
6	0	0	0	0	0	2

(8x**1**, 2x**2**)

	1	2	3	4	5	6
1	2	0	0	0	0	0
2	0	0	0	1	1	0
3	0	0	0	1	1	0
4	0	1	1	0	0	0
5	0	1	1	0	0	0
6	0	0	0	0	0	2

(8x**1**, 2x$\mathbf{2^1}$)

	1	2	3	4	5	6
1	0	1	1	0	0	0
2	1	0	0	1	0	0
3	1	0	0	1	0	0
4	0	1	1	0	0	0
5	0	0	0	0	0	2^1
6	0	0	0	0	2^1	0

(8x**1**, 2x$\mathbf{2^2}$)

	1	2	3	4	5	6
1	0	1	1	0	0	0
2	1	0	0	1	0	0
3	1	0	0	1	0	0
4	0	1	1	0	0	0
5	0	0	0	0	0	2^2
6	0	0	0	0	2^2	0

(6x**1**, 3x**2**)

	1	2	3	4	5	6
1	2	0	0	0	0	0
2	0	0	1	1	0	0
3	0	1	0	1	0	0
4	0	1	1	0	0	0
5	0	0	0	0	2	0
6	0	0	0	0	0	2

(6x**1**, 1x**2**, 2x$\mathbf{2^1}$)

	1	2	3	4	5	6
1	2	0	0	0	0	0
2	0	0	1	1	0	0
3	0	1	0	1	0	0
4	0	1	1	0	0	0
5	0	0	0	0	0	2^1
6	0	0	0	0	2^1	0

(6x**1**, 1x**2**, 2x$\mathbf{2^2}$)

	1	2	3	4	5	6
1	2	0	0	0	0	0
2	0	0	1	1	0	0
3	0	1	0	1	0	0
4	0	1	1	0	0	0
5	0	0	0	0	0	2^2
6	0	0	0	0	2^2	0

(6x**2**)

	1	2	3	4	5	6
1	2	0	0	0	0	0
2	0	2	0	0	0	0
3	0	0	2	0	0	0
4	0	0	0	2	0	0
5	0	0	0	0	2	0
6	0	0	0	0	0	2

(4x**2**, 2x$\mathbf{2^1}$)

	1	2	3	4	5	6
1	0	2^1	0	0	0	0
2	2^1	0	0	0	0	0
3	0	0	2	0	0	0
4	0	0	0	2	0	0
5	0	0	0	0	2	0
6	0	0	0	0	0	2

(4x**2**, 2x$\mathbf{2^2}$)

	1	2	3	4	5	6
1	0	2^2	0	0	0	0
2	2^2	0	0	0	0	0
3	0	0	2	0	0	0
4	0	0	0	2	0	0
5	0	0	0	0	2	0
6	0	0	0	0	0	2

(2x**2**, 4x$\mathbf{2^1}$)

	1	2	3	4	5	6
1	0	2^1	0	0	0	0
2	2^1	0	0	0	0	0
3	0	0	0	2^1	0	0
4	0	0	2^1	0	0	0
5	0	0	0	0	2	0
6	0	0	0	0	0	2

(2x**2**, 2x$\mathbf{2^1}$, 2x$\mathbf{2^2}$)

	1	2	3	4	5	6
1	0	2^1	0	0	0	0
2	2^1	0	0	0	0	0
3	0	0	0	2^2	0	0
4	0	0	2^2	0	0	0
5	0	0	0	0	2	0
6	0	0	0	0	0	2

(2x**2**, 4x$\mathbf{2^2}$)

	1	2	3	4	5	6
1	0	2^2	0	0	0	0
2	2^2	0	0	0	0	0
3	0	0	0	2^2	0	0
4	0	0	2^2	0	0	0
5	0	0	0	0	2	0
6	0	0	0	0	0	2

(6x$\mathbf{2^1}$)

	1	2	3	4	5	6
1	0	2^1	0	0	0	0
2	2^1	0	0	0	0	0
3	0	0	0	2^1	0	0
4	0	0	2^1	0	0	0
5	0	0	0	0	0	2^1
6	0	0	0	0	2^1	0

(4x$\mathbf{2^1}$, 2x$\mathbf{2^2}$)

	1	2	3	4	5	6
1	0	2^1	0	0	0	0
2	2^1	0	0	0	0	0
3	0	0	0	2^1	0	0
4	0	0	2^1	0	0	0
5	0	0	0	0	0	2^2
6	0	0	0	0	2^2	0

(2x$\mathbf{2^1}$, 4x$\mathbf{2^2}$)

	1	2	3	4	5	6
1	0	2^1	0	0	0	0
2	2^1	0	0	0	0	0
3	0	0	0	2^2	0	0
4	0	0	2^2	0	0	0
5	0	0	0	0	0	2^2
6	0	0	0	0	2^2	0

(6x$\mathbf{2^2}$)

	1	2	3	4	5	6
1	0	2^2	0	0	0	0
2	2^2	0	0	0	0	0
3	0	0	0	2^2	0	0
4	0	0	2^2	0	0	0
5	0	0	0	0	0	2^2
6	0	0	0	0	2^2	0

$^{2}V_{7}$

(14x**1**) **a**

	1	2	3	4	5	6	7
1	0	0	1	0	0	0	1
2	0	0	0	1	0	0	1
3	1	0	0	0	0	1	0
4	0	1	0	0	1	0	0
5	0	0	0	1	0	1	0
6	0	0	1	0	1	0	0
7	1	1	0	0	0	0	0

(14x**1**) **b**

	1	2	3	4	5	6	7
1	0	1	0	1	0	0	0
2	1	0	1	0	0	0	0
3	0	1	0	1	0	0	0
4	1	0	1	0	0	0	0
5	0	0	0	0	0	1	1
6	0	0	0	0	1	0	1
7	0	0	0	0	1	1	0

(12x**1**, 1x**2**) **a**

	1	2	3	4	5	6	7
1	0	1	1	0	0	0	0
2	1	0	0	1	0	0	0
3	1	0	0	0	0	1	0
4	0	1	0	0	1	0	0
5	0	0	0	1	0	1	0
6	0	0	1	0	1	0	0
7	0	0	0	0	0	0	2

(12x**1**, 1x**2**) **b**

	1	2	3	4	5	6	7
1	0	1	1	0	0	0	0
2	1	0	1	0	0	0	0
3	1	1	0	0	0	0	0
4	0	0	0	0	1	1	0
5	0	0	0	1	0	1	0
6	0	0	0	1	1	0	0
7	0	0	0	0	0	0	2

(10x**1**, 2x**2**)

	1	2	3	4	5	6	7
1	0	1	1	0	0	0	0
2	1	0	0	1	0	0	0
3	1	0	0	0	1	0	0
4	0	1	0	0	1	0	0
5	0	0	1	1	0	0	0
6	0	0	0	0	0	2	0
7	0	0	0	0	0	0	2

(10x**1**, 2x$\mathbf{2^1}$)

	1	2	3	4	5	6	7
1	0	1	1	0	0	0	0
2	1	0	0	1	0	0	0
3	1	0	0	0	1	0	0
4	0	1	0	0	1	0	0
5	0	0	1	1	0	0	0
6	0	0	0	0	0	0	2^1
7	0	0	0	0	0	2^1	0

(10x**1**, 2x$\mathbf{2^2}$)

	1	2	3	4	5	6	7
1	0	1	1	0	0	0	0
2	1	0	0	1	0	0	0
3	1	0	0	0	1	0	0
4	0	1	0	0	1	0	0
5	0	0	1	1	0	0	0
6	0	0	0	0	0	0	2^2
7	0	0	0	0	0	2^2	0

(8x**1**, 3x**2**)

	1	2	3	4	5	6	7
1	2	0	0	0	0	0	0
2	0	0	0	1	1	0	0
3	0	0	0	1	1	0	0
4	0	1	1	0	0	0	0
5	0	1	1	0	0	0	0
6	0	0	0	0	0	2	0
7	0	0	0	0	0	0	2

(8x**1**, 1x**2**, 2x$\mathbf{2^1}$)

	1	2	3	4	5	6	7
1	2	0	0	0	0	0	0
2	0	0	0	1	1	0	0
3	0	0	0	1	1	0	0
4	0	1	1	0	0	0	0
5	0	1	1	0	0	0	0
6	0	0	0	0	0	0	2^1
7	0	0	0	0	0	2^1	0

(8x**1**, 1x**2**, 2x$\mathbf{2^2}$)

	1	2	3	4	5	6	7
1	2	0	0	0	0	0	0
2	0	0	0	1	1	0	0
3	0	0	0	1	1	0	0
4	0	1	1	0	0	0	0
5	0	1	1	0	0	0	0
6	0	0	0	0	0	0	2^2
7	0	0	0	0	0	2^2	0

(6x**1**, 4x**2**)

	1	2	3	4	5	6	7
1	2	0	0	0	0	0	0
2	0	0	1	1	0	0	0
3	0	1	0	1	0	0	0
4	0	1	1	0	0	0	0
5	0	0	0	0	2	0	0
6	0	0	0	0	0	2	0
7	0	0	0	0	0	0	2

(6x**1**, 2x**2**, 2x$\mathbf{2}^{1}$)

	1	2	3	4	5	6	7
1	2	0	0	0	0	0	0
2	0	0	1	1	0	0	0
3	0	1	0	1	0	0	0
4	0	1	1	0	0	0	0
5	0	0	0	0	2	0	0
6	0	0	0	0	0	0	2^1
7	0	0	0	0	0	2^1	0

(6x**1**, 2x**2**, 2x$\mathbf{2}^{2}$)

	1	2	3	4	5	6	7
1	2	0	0	0	0	0	0
2	0	0	1	1	0	0	0
3	0	1	0	1	0	0	0
4	0	1	1	0	0	0	0
5	0	0	0	0	2	0	0
6	0	0	0	0	0	0	2^2
7	0	0	0	0	0	2^2	0

(6x**1**, 4x$\mathbf{2}^{1}$)

	1	2	3	4	5	6	7
1	0	1	1	0	0	0	0
2	1	0	1	0	0	0	0
3	1	1	0	0	0	0	0
4	0	0	0	0	2^1	0	0
5	0	0	0	2^1	0	0	0
6	0	0	0	0	0	0	2^1
7	0	0	0	0	0	2^1	0

(6x**1**, 2x$\mathbf{2}^{1}$, 2x$\mathbf{2}^{2}$)

	1	2	3	4	5	6	7
1	0	1	1	0	0	0	0
2	1	0	1	0	0	0	0
3	1	1	0	0	0	0	0
4	0	0	0	0	2^1	0	0
5	0	0	0	2^1	0	0	0
6	0	0	0	0	0	0	2^2
7	0	0	0	0	0	2^2	0

(6x**1**, 4x$\mathbf{2}^{2}$)

	1	2	3	4	5	6	7
1	0	1	1	0	0	0	0
2	1	0	1	0	0	0	0
3	1	1	0	0	0	0	0
4	0	0	0	0	2^2	0	0
5	0	0	0	2^2	0	0	0
6	0	0	0	0	0	0	2^2
7	0	0	0	0	0	2^2	0

(7x**2**)

	1	2	3	4	5	6	7
1	2	0	0	0	0	0	0
2	0	2	0	0	0	0	0
3	0	0	2	0	0	0	0
4	0	0	0	2	0	0	0
5	0	0	0	0	2	0	0
6	0	0	0	0	0	2	0
7	0	0	0	0	0	0	2

(5x**2**, 2x$\mathbf{2}^{1}$)

	1	2	3	4	5	6	7
1	2	0	0	0	0	0	0
2	0	2	0	0	0	0	0
3	0	0	2	0	0	0	0
4	0	0	0	2	0	0	0
5	0	0	0	0	2	0	0
6	0	0	0	0	0	0	2^1
7	0	0	0	0	0	2^1	0

(5x**2**, 2x$\mathbf{2}^{2}$)

	1	2	3	4	5	6	7
1	2	0	0	0	0	0	0
2	0	2	0	0	0	0	0
3	0	0	2	0	0	0	0
4	0	0	0	2	0	0	0
5	0	0	0	0	2	0	0
6	0	0	0	0	0	0	2^2
7	0	0	0	0	0	2^2	0

(3x**2**, 4x$\mathbf{2}^{1}$)

	1	2	3	4	5	6	7
1	2	0	0	0	0	0	0
2	0	2	0	0	0	0	0
3	0	0	2	0	0	0	0
4	0	0	0	0	2^1	0	0
5	0	0	0	2^1	0	0	0
6	0	0	0	0	0	0	2^1
7	0	0	0	0	0	2^1	0

(3x**2**, 2x$\mathbf{2}^{1}$, 2x$\mathbf{2}^{2}$)

	1	2	3	4	5	6	7
1	2	0	0	0	0	0	0
2	0	2	0	0	0	0	0
3	0	0	2	0	0	0	0
4	0	0	0	0	2^1	0	0
5	0	0	0	2^1	0	0	0
6	0	0	0	0	0	0	2^2
7	0	0	0	0	0	2^2	0

(3x**2**, 4x$\mathbf{2}^{2}$)

	1	2	3	4	5	6	7
1	2	0	0	0	0	0	0
2	0	2	0	0	0	0	0
3	0	0	2	0	0	0	0
4	0	0	0	0	2^2	0	0
5	0	0	0	2^2	0	0	0
6	0	0	0	0	0	0	2^2
7	0	0	0	0	0	2^2	0

(1x**2**, 6x$\mathbf{2}^{1}$)

	1	2	3	4	5	6	7
1	2	0	0	0	0	0	0
2	0	0	2^1	0	0	0	0
3	0	2^1	0	0	0	0	0
4	0	0	0	0	2^1	0	0
5	0	0	0	2^1	0	0	0
6	0	0	0	0	0	0	2^1
7	0	0	0	0	0	2^1	0

(1x**2**, 4x$\mathbf{2}^{1}$, 2x$\mathbf{2}^{2}$)

	1	2	3	4	5	6	7
1	2	0	0	0	0	0	0
2	0	0	2^1	0	0	0	0
3	0	2^1	0	0	0	0	0
4	0	0	0	0	2^1	0	0
5	0	0	0	2^1	0	0	0
6	0	0	0	0	0	0	2^2
7	0	0	0	0	0	2^2	0

(1x**2**, 2x$\mathbf{2}^{1}$, 4x$\mathbf{2}^{2}$)

	1	2	3	4	5	6	7
1	2	0	0	0	0	0	0
2	0	0	2^1	0	0	0	0
3	0	2^1	0	0	0	0	0
4	0	0	0	0	2^2	0	0
5	0	0	0	2^2	0	0	0
6	0	0	0	0	0	0	2^2
7	0	0	0	0	0	2^2	0

(1x**2**, 6x$\mathbf{2}^{2}$)

	1	2	3	4	5	6	7
1	2	0	0	0	0	0	0
2	0	0	2^2	0	0	0	0
3	0	2^2	0	0	0	0	0
4	0	0	0	0	2^2	0	0
5	0	0	0	2^2	0	0	0
6	0	0	0	0	0	0	2^2
7	0	0	0	0	0	2^2	0

$^{2}V_{8}$

(16x**1**) **a**

	1	2	3	4	5	6	7	8
1	0	0	1	0	0	0	1	0
2	0	0	0	1	0	0	0	1
3	1	0	0	0	0	1	0	0
4	0	1	0	0	1	0	0	0
5	0	0	0	1	0	1	0	0
6	0	0	1	0	1	0	0	0
7	1	0	0	0	0	0	0	1
8	0	1	0	0	0	0	1	0

(16x**1**) **b**

	1	2	3	4	5	6	7	8
1	0	1	0	1	0	0	0	0
2	1	0	1	0	0	0	0	0
3	0	1	0	1	0	0	0	0
4	1	0	1	0	0	0	0	0
5	0	0	0	0	0	1	0	1
6	0	0	0	0	1	0	1	0
7	0	0	0	0	0	1	0	1
8	0	0	0	0	1	0	1	0

(16x**1**) **c**

	1	2	3	4	5	6	7	8
1	0	1	1	0	0	0	0	0
2	1	0	0	1	0	0	0	0
3	1	0	0	0	1	0	0	0
4	0	1	0	0	1	0	0	0
5	0	0	1	1	0	0	0	0
6	0	0	0	0	0	0	1	1
7	0	0	0	0	0	1	0	1
8	0	0	0	0	0	1	1	0

(14x**1**, 1x**2**) **a**

	1	2	3	4	5	6	7	8
1	0	0	1	0	0	0	1	0
2	0	0	0	1	0	0	1	0
3	1	0	0	0	0	1	0	0
4	0	1	0	0	1	0	0	0
5	0	0	0	1	0	1	0	0
6	0	0	1	0	1	0	0	0
7	1	1	0	0	0	0	0	0
8	0	0	0	0	0	0	0	2

(14x**1**, 1x**2**) **b**

	1	2	3	4	5	6	7	8
1	0	1	0	1	0	0	0	0
2	1	0	1	0	0	0	0	0
3	0	1	0	1	0	0	0	0
4	1	0	1	0	0	0	0	0
5	0	0	0	0	0	1	1	0
6	0	0	0	0	1	0	1	0
7	0	0	0	0	1	1	0	0
8	0	0	0	0	0	0	0	2

(12x**1**, 2x**2**) **a**

	1	2	3	4	5	6	7	8
1	0	1	1	0	0	0	0	0
2	1	0	0	1	0	0	0	0
3	1	0	0	0	0	1	0	0
4	0	1	0	0	1	0	0	0
5	0	0	0	1	0	1	0	0
6	0	0	1	0	1	0	0	0
7	0	0	0	0	0	0	2	0
8	0	0	0	0	0	0	0	2

(12x**1**, 2x**2**) **b**

	1	2	3	4	5	6	7	8
1	0	1	1	0	0	0	0	0
2	1	0	1	0	0	0	0	0
3	1	1	0	0	0	0	0	0
4	0	0	0	0	1	1	0	0
5	0	0	0	1	0	1	0	0
6	0	0	0	1	1	0	0	0
7	0	0	0	0	0	0	2	0
8	0	0	0	0	0	0	0	2

(12x**1**, 2x**2**^{1}) **a**

	1	2	3	4	5	6	7	8
1	0	0	1	0	0	1	0	0
2	0	0	0	0	1	1	0	0
3	1	0	0	1	0	0	0	0
4	0	0	1	0	1	0	0	0
5	0	1	0	1	0	0	0	0
6	1	1	0	0	0	0	0	0
7	0	0	0	0	0	0	0	2^1
8	0	0	0	0	0	0	2^1	0

(12x**1**, 2x**2**^{1}) **b**

	1	2	3	4	5	6	7	8
1	0	1	1	0	0	0	0	0
2	1	0	1	0	0	0	0	0
3	1	1	0	0	0	0	0	0
4	0	0	0	0	1	1	0	0
5	0	0	0	1	0	1	0	0
6	0	0	0	1	1	0	0	0
7	0	0	0	0	0	0	0	2^1
8	0	0	0	0	0	0	2^1	0

(12x**1**, 2x**2**^{2}) **a**

	1	2	3	4	5	6	7	8
1	0	0	1	0	0	1	0	0
2	0	0	0	0	1	1	0	0
3	1	0	0	1	0	0	0	0
4	0	0	1	0	1	0	0	0
5	0	1	0	1	0	0	0	0
6	1	1	0	0	0	0	0	0
7	0	0	0	0	0	0	0	2^2
8	0	0	0	0	0	0	2^2	0

(12x**1**, 2x**2**^{2}) **b**

	1	2	3	4	5	6	7	8
1	0	1	1	0	0	0	0	0
2	1	0	1	0	0	0	0	0
3	1	1	0	0	0	0	0	0
4	0	0	0	0	1	1	0	0
5	0	0	0	1	0	1	0	0
6	0	0	0	1	1	0	0	0
7	0	0	0	0	0	0	0	2^2
8	0	0	0	0	0	0	2^2	0

(10x**1**, 3x**2**)

	1	2	3	4	5	6	7	8
1	0	1	1	0	0	0	0	0
2	1	0	0	1	0	0	0	0
3	1	0	0	0	1	0	0	0
4	0	1	0	0	1	0	0	0
5	0	0	1	1	0	0	0	0
6	0	0	0	0	0	2	0	0
7	0	0	0	0	0	0	2	0
8	0	0	0	0	0	0	0	2

(10x**1**, 1x**2**, 2x**2**^{1})

	1	2	3	4	5	6	7	8
1	0	1	1	0	0	0	0	0
2	1	0	0	1	0	0	0	0
3	1	0	0	0	1	0	0	0
4	0	1	0	0	1	0	0	0
5	0	0	1	1	0	0	0	0
6	0	0	0	0	0	0	2^1	0
7	0	0	0	0	0	2^1	0	0
8	0	0	0	0	0	0	0	2

(10x**1**, 1x**2**, 2x**2**^{2})

	1	2	3	4	5	6	7	8
1	0	1	1	0	0	0	0	0
2	1	0	0	1	0	0	0	0
3	1	0	0	0	1	0	0	0
4	0	1	0	0	1	0	0	0
5	0	0	1	1	0	0	0	0
6	0	0	0	0	0	0	2^2	0
7	0	0	0	0	0	2^2	0	0
8	0	0	0	0	0	0	0	2

(8x**1**, 4x**2**)

	1	2	3	4	5	6	7	8
1	2	0	0	0	0	0	0	0
2	0	0	0	1	1	0	0	0
3	0	0	0	1	1	0	0	0
4	0	1	1	0	0	0	0	0
5	0	1	1	0	0	0	0	0
6	0	0	0	0	0	2	0	0
7	0	0	0	0	0	0	2	0
8	0	0	0	0	0	0	0	2

(8x**1**, 2x**2**, 2x**2**^{1})

	1	2	3	4	5	6	7	8
1	0	1	1	0	0	0	0	0
2	1	0	0	1	0	0	0	0
3	1	0	0	1	0	0	0	0
4	0	1	1	0	0	0	0	0
5	0	0	0	0	0	2^1	0	0
6	0	0	0	0	2^1	0	0	0
7	0	0	0	0	0	0	2	0
8	0	0	0	0	0	0	0	2

(8x**1**, 2x**2**, 2x**2**^{2})

	1	2	3	4	5	6	7	8
1	0	1	1	0	0	0	0	0
2	1	0	0	1	0	0	0	0
3	1	0	0	1	0	0	0	0
4	0	1	1	0	0	0	0	0
5	0	0	0	0	0	2^2	0	0
6	0	0	0	0	2^2	0	0	0
7	0	0	0	0	0	0	2	0
8	0	0	0	0	0	0	0	2

(8x**1**, 4x**2**^{1})

	1	2	3	4	5	6	7	8
1	0	1	1	0	0	0	0	0
2	1	0	0	1	0	0	0	0
3	1	0	0	1	0	0	0	0
4	0	1	1	0	0	0	0	0
5	0	0	0	0	0	2^1	0	0
6	0	0	0	0	2^1	0	0	0
7	0	0	0	0	0	0	0	2^1
8	0	0	0	0	0	0	2^1	0

(8x**1**, 2x**2**^{1}, 2x**2**^{2})

	1	2	3	4	5	6	7	8
1	0	1	1	0	0	0	0	0
2	1	0	0	1	0	0	0	0
3	1	0	0	1	0	0	0	0
4	0	1	1	0	0	0	0	0
5	0	0	0	0	0	2^1	0	0
6	0	0	0	0	2^1	0	0	0
7	0	0	0	0	0	0	0	2^2
8	0	0	0	0	0	0	2^2	0

(8x**1**, 4x**2**^{2})

	1	2	3	4	5	6	7	8
1	0	1	1	0	0	0	0	0
2	1	0	0	1	0	0	0	0
3	1	0	0	1	0	0	0	0
4	0	1	1	0	0	0	0	0
5	0	0	0	0	0	2^2	0	0
6	0	0	0	0	2^2	0	0	0
7	0	0	0	0	0	0	0	2^2
8	0	0	0	0	0	0	2^2	0

(6x**1**, 5x**2**)

	1	2	3	4	5	6	7	8
1	2	0	0	0	0	0	0	0
2	0	0	1	1	0	0	0	0
3	0	1	0	1	0	0	0	0
4	0	1	1	0	0	0	0	0
5	0	0	0	0	2	0	0	0
6	0	0	0	0	0	2	0	0
7	0	0	0	0	0	0	2	0
8	0	0	0	0	0	0	0	2

(6x**1**, 3x**2**, 2x$\mathbf{2^{1}}$)

	1	2	3	4	5	6	7	8
1	0	1	1	0	0	0	0	0
2	1	0	1	0	0	0	0	0
3	1	1	0	0	0	0	0	0
4	0	0	0	0	2^1	0	0	0
5	0	0	0	2^1	0	0	0	0
6	0	0	0	0	0	2	0	0
7	0	0	0	0	0	0	2	0
8	0	0	0	0	0	0	0	2

(6x**1**, 3x**2**, 2x$\mathbf{2^{2}}$)

	1	2	3	4	5	6	7	8
1	0	1	1	0	0	0	0	0
2	1	0	1	0	0	0	0	0
3	1	1	0	0	0	0	0	0
4	0	0	0	0	2^2	0	0	0
5	0	0	0	2^2	0	0	0	0
6	0	0	0	0	0	2	0	0
7	0	0	0	0	0	0	2	0
8	0	0	0	0	0	0	0	2

(6x**1**, 1x**2**, 4x$\mathbf{2^{1}}$)

	1	2	3	4	5	6	7	8
1	0	1	1	0	0	0	0	0
2	1	0	1	0	0	0	0	0
3	1	1	0	0	0	0	0	0
4	0	0	0	0	2^1	0	0	0
5	0	0	0	2^1	0	0	0	0
6	0	0	0	0	0	0	2^1	0
7	0	0	0	0	0	2^1	0	0
8	0	0	0	0	0	0	0	2

(6x**1**, 1x**2**, 2x$\mathbf{2^{1}}$, 2x$\mathbf{2^{2}}$)

	1	2	3	4	5	6	7	8
1	0	1	1	0	0	0	0	0
2	1	0	1	0	0	0	0	0
3	1	1	0	0	0	0	0	0
4	0	0	0	0	2^1	0	0	0
5	0	0	0	2^1	0	0	0	0
6	0	0	0	0	0	0	2^2	0
7	0	0	0	0	0	2^2	0	0
8	0	0	0	0	0	0	0	2

(6x**1**, 1x**2**, 4x$\mathbf{2^{2}}$)

	1	2	3	4	5	6	7	8
1	0	1	1	0	0	0	0	0
2	1	0	1	0	0	0	0	0
3	1	1	0	0	0	0	0	0
4	0	0	0	0	2^2	0	0	0
5	0	0	0	2^2	0	0	0	0
6	0	0	0	0	0	0	2^2	0
7	0	0	0	0	0	2^2	0	0
8	0	0	0	0	0	0	0	2

(8x**2**)

	1	2	3	4	5	6	7	8
1	2	0	0	0	0	0	0	0
2	0	2	0	0	0	0	0	0
3	0	0	2	0	0	0	0	0
4	0	0	0	2	0	0	0	0
5	0	0	0	0	2	0	0	0
6	0	0	0	0	0	2	0	0
7	0	0	0	0	0	0	2	0
8	0	0	0	0	0	0	0	2

(6x**2**, 2x$\mathbf{2^{1}}$)

	1	2	3	4	5	6	7	8
1	0	2^1	0	0	0	0	0	0
2	2^1	0	0	0	0	0	0	0
3	0	0	2	0	0	0	0	0
4	0	0	0	2	0	0	0	0
5	0	0	0	0	2	0	0	0
6	0	0	0	0	0	2	0	0
7	0	0	0	0	0	0	2	0
8	0	0	0	0	0	0	0	2

(6x**2**, 2x$\mathbf{2^{2}}$)

	1	2	3	4	5	6	7	8
1	0	2^2	0	0	0	0	0	0
2	2^2	0	0	0	0	0	0	0
3	0	0	2	0	0	0	0	0
4	0	0	0	2	0	0	0	0
5	0	0	0	0	2	0	0	0
6	0	0	0	0	0	2	0	0
7	0	0	0	0	0	0	2	0
8	0	0	0	0	0	0	0	2

(4x**2**, 4x$\mathbf{2^{1}}$)

	1	2	3	4	5	6	7	8
1	0	2^1	0	0	0	0	0	0
2	2^1	0	0	0	0	0	0	0
3	0	0	0	2^1	0	0	0	0
4	0	0	2^1	0	0	0	0	0
5	0	0	0	0	2	0	0	0
6	0	0	0	0	0	2	0	0
7	0	0	0	0	0	0	2	0
8	0	0	0	0	0	0	0	2

(4x**2**, 2x$\mathbf{2^{1}}$, 2x$\mathbf{2^{2}}$)

	1	2	3	4	5	6	7	8
1	0	2^1	0	0	0	0	0	0
2	2^1	0	0	0	0	0	0	0
3	0	0	0	2^2	0	0	0	0
4	0	0	2^2	0	0	0	0	0
5	0	0	0	0	2	0	0	0
6	0	0	0	0	0	2	0	0
7	0	0	0	0	0	0	2	0
8	0	0	0	0	0	0	0	2

(4x**2**, 4x$\mathbf{2^{2}}$)

	1	2	3	4	5	6	7	8
1	0	2^2	0	0	0	0	0	0
2	2^2	0	0	0	0	0	0	0
3	0	0	0	2^2	0	0	0	0
4	0	0	2^2	0	0	0	0	0
5	0	0	0	0	2	0	0	0
6	0	0	0	0	0	2	0	0
7	0	0	0	0	0	0	2	0
8	0	0	0	0	0	0	0	2

(2x**2**, 6x$\mathbf{2^{1}}$)

	1	2	3	4	5	6	7	8
1	0	2^1	0	0	0	0	0	0
2	2^1	0	0	0	0	0	0	0
3	0	0	0	2^1	0	0	0	0
4	0	0	2^1	0	0	0	0	0
5	0	0	0	0	0	2^1	0	0
6	0	0	0	0	2^1	0	0	0
7	0	0	0	0	0	0	2	0
8	0	0	0	0	0	0	0	2

(2x**2**, 4x$\mathbf{2^{1}}$, 2x$\mathbf{2^{2}}$)

	1	2	3	4	5	6	7	8
1	0	2^1	0	0	0	0	0	0
2	2^1	0	0	0	0	0	0	0
3	0	0	0	2^1	0	0	0	0
4	0	0	2^1	0	0	0	0	0
5	0	0	0	0	0	2^2	0	0
6	0	0	0	0	2^2	0	0	0
7	0	0	0	0	0	0	2	0
8	0	0	0	0	0	0	0	2

(2x**2**, 2x$\mathbf{2^{1}}$, 4x$\mathbf{2^{2}}$)

	1	2	3	4	5	6	7	8
1	0	2^1	0	0	0	0	0	0
2	2^1	0	0	0	0	0	0	0
3	0	0	0	2^2	0	0	0	0
4	0	0	2^2	0	0	0	0	0
5	0	0	0	0	0	2^2	0	0
6	0	0	0	0	2^2	0	0	0
7	0	0	0	0	0	0	2	0
8	0	0	0	0	0	0	0	2

(2x**2**, 6x$\mathbf{2^{2}}$)

	1	2	3	4	5	6	7	8
1	0	2^2	0	0	0	0	0	0
2	2^2	0	0	0	0	0	0	0
3	0	0	0	2^2	0	0	0	0
4	0	0	2^2	0	0	0	0	0
5	0	0	0	0	0	2^2	0	0
6	0	0	0	0	2^2	0	0	0
7	0	0	0	0	0	0	2	0
8	0	0	0	0	0	0	0	2

(8x$\mathbf{2^{1}}$)

	1	2	3	4	5	6	7	8
1	0	2^1	0	0	0	0	0	0
2	2^1	0	0	0	0	0	0	0
3	0	0	0	2^1	0	0	0	0
4	0	0	2^1	0	0	0	0	0
5	0	0	0	0	0	2^1	0	0
6	0	0	0	0	2^1	0	0	0
7	0	0	0	0	0	0	0	2^1
8	0	0	0	0	0	0	2^1	0

(6x$\mathbf{2^{1}}$, 2x$\mathbf{2^{2}}$)

	1	2	3	4	5	6	7	8
1	0	2^1	0	0	0	0	0	0
2	2^1	0	0	0	0	0	0	0
3	0	0	0	2^1	0	0	0	0
4	0	0	2^1	0	0	0	0	0
5	0	0	0	0	0	2^1	0	0
6	0	0	0	0	2^1	0	0	0
7	0	0	0	0	0	0	0	2^2
8	0	0	0	0	0	0	2^2	0

(4x$\mathbf{2^{1}}$, 4x$\mathbf{2^{2}}$)

	1	2	3	4	5	6	7	8
1	0	2^1	0	0	0	0	0	0
2	2^1	0	0	0	0	0	0	0
3	0	0	0	2^1	0	0	0	0
4	0	0	2^1	0	0	0	0	0
5	0	0	0	0	0	2^2	0	0
6	0	0	0	0	2^2	0	0	0
7	0	0	0	0	0	0	0	2^2
8	0	0	0	0	0	0	2^2	0

(2x$\mathbf{2^{1}}$, 6x$\mathbf{2^{2}}$)

	1	2	3	4	5	6	7	8
1	0	2^1	0	0	0	0	0	0
2	2^1	0	0	0	0	0	0	0
3	0	0	0	2^2	0	0	0	0
4	0	0	2^2	0	0	0	0	0
5	0	0	0	0	0	2^2	0	0
6	0	0	0	0	2^2	0	0	0
7	0	0	0	0	0	0	0	2^2
8	0	0	0	0	0	0	2^2	0

(8x$\mathbf{2}^2$)

	1	2	3	4	5	6	7	8
1	0	2^2	0	0	0	0	0	0
2	2^2	0	0	0	0	0	0	0
3	0	0	0	2^2	0	0	0	0
4	0	0	2^2	0	0	0	0	0
5	0	0	0	0	0	2^2	0	0
6	0	0	0	0	2^2	0	0	0
7	0	0	0	0	0	0	0	2^2
8	0	0	0	0	0	0	2^2	0

$^3V_2$

(1x**2**, 2x**2**)

	1	2
1	2	1
2	1	2

$^3V_4$

(12x**1**)

	1	2	3	4
1	0	1	1	1
2	1	0	1	1
3	1	1	0	1
4	1	1	1	0

(6x**1**, 3x**2**)

	1	2	3	4
1	0	1	1	1
2	1	2	0	0
3	1	0	2	0
4	1	0	0	2

(6x**1**, 1x**2**, 2x$\mathbf{2}^1$)

	1	2	3	4
1	2	1	0	0
2	1	0	1	1
3	0	1	0	2^1
4	0	1	2^1	0

(6x**1**, 1x**2**, 2x$\mathbf{2}^2$)

	1	2	3	4
1	2	1	0	0
2	1	0	1	1
3	0	1	0	2^2
4	0	1	2^2	0

(4x**1**, 4x**2**)

	1	2	3	4
1	2	1	0	0
2	1	2	0	0
3	0	0	2	1
4	0	0	1	2

(4x**1**, 2x**2**, 2x$\mathbf{2}^1$)

	1	2	3	4
1	0	2^1	1	0
2	2^1	0	0	1
3	1	0	2	0
4	0	1	0	2

(4x**1**, 2x**2**, 2x$\mathbf{2}^2$)

	1	2	3	4
1	0	2^2	1	0
2	2^2	0	0	1
3	1	0	2	0
4	0	1	0	2

(4x**1**, 4x$\mathbf{2}^1$)

	1	2	3	4
1	0	2^1	0	1
2	2^1	0	1	0
3	0	1	0	2^1
4	1	0	2^1	0

(4x**1**, 2x$\mathbf{2}^1$, 2x$\mathbf{2}^2$)

	1	2	3	4
1	0	2^2	0	1
2	2^2	0	1	0
3	0	1	0	2^1
4	1	0	2^1	0

(4x**1**, 4x$\mathbf{2}^2$)

	1	2	3	4
1	0	2^2	0	1
2	2^2	0	1	0
3	0	1	0	2^2
4	1	0	2^2	0

$^3V_6$

NG

$^3V_8$

NG

$^4V_2$

(2x**2**, 2x$\mathbf{2}^1$)

	1	2
1	2	2^1
2	2^1	2

(2x**2**, 2x$\mathbf{2}^2$)

	1	2
1	2	2^2
2	2^2	2

$^4V_3$

(6x**1**, 3x**2**)

	1	2	3
1	2	1	1
2	1	2	1
3	1	1	2

(2x**2**, 4x$\mathbf{2}^1$)

	1	2	3
1	0	2^1	2^1
2	2^1	2	0
3	2^1	0	2

(2x**2**, 2x$\mathbf{2}^1$, 2x$\mathbf{2}^2$)

	1	2	3
1	0	2^2	2^1
2	2^2	2	0
3	2^1	0	2

(2x**2**, 4x$\mathbf{2}^2$)

	1	2	3
1	0	2^2	2^2
2	2^2	2	0
3	2^2	0	2

(6x$\mathbf{2}^1$)

	1	2	3
1	0	2^1	2^1
2	2^1	0	2^1
3	2^1	2^1	0

(4x$\mathbf{2}^1$, 2x$\mathbf{2}^2$)

	1	2	3
1	0	2^1	2^1
2	2^1	0	2^2
3	2^1	2^2	0

(2x$\mathbf{2}^1$, 4x$\mathbf{2}^2$)

	1	2	3
1	0	2^1	2^2
2	2^1	0	2^2
3	2^2	2^2	0

(6x$\mathbf{2}^2$)

	1	2	3
1	0	2^2	2^2
2	2^2	0	2^2
3	2^2	2^2	0

$^{4}V_{4}$

(8x**1**, 4x**2**)

	1	2	3	4
1	2	1	1	0
2	1	2	0	1
3	1	0	2	1
4	0	1	1	2

(8x**1**, 2x**2**, 2x$\mathbf{2^1}$)

	1	2	3	4
1	2	1	1	0
2	1	0	2^1	1
3	1	2^1	0	1
4	0	1	1	2

(8x**1**, 2x**2**, 2x$\mathbf{2^2}$)

	1	2	3	4
1	2	1	1	0
2	1	0	2^2	1
3	1	2^2	0	1
4	0	1	1	2

(8x**1**, 4x$\mathbf{2^1}$)

	1	2	3	4
1	0	1	2^1	1
2	1	0	1	2^1
3	2^1	1	0	1
4	1	2^1	1	0

(8x**1**, 2x$\mathbf{2^1}$, 2x$\mathbf{2^2}$)

	1	2	3	4
1	0	1	2^2	1
2	1	0	1	2^1
3	2^2	1	0	1
4	1	2^1	1	0

(8x**1**, 4x$\mathbf{2^2}$)

	1	2	3	4
1	0	1	2^2	1
2	1	0	1	2^2
3	2^2	1	0	1
4	1	2^2	1	0

(6x**1**, 3x**2**, 2x$\mathbf{2^1}$)

	1	2	3	4
1	2	2^1	0	0
2	2^1	0	1	1
3	0	1	2	1
4	0	1	1	2

(6x**1**, 3x**2**, 2x$\mathbf{2^2}$)

	1	2	3	4
1	2	2^2	0	0
2	2^2	0	1	1
3	0	1	2	1
4	0	1	1	2

(6x**1**, 1x**2**, 4x$\mathbf{2^1}$)

	1	2	3	4
1	2	1	1	0
2	1	0	1	2^1
3	1	1	0	2^1
4	0	2^1	2^1	0

(6x**1**, 1x**2**, 2x$\mathbf{2^1}$, 2x$\mathbf{2^2}$)

	1	2	3	4
1	2	1	1	0
2	1	0	1	2^2
3	1	1	0	2^1
4	0	2^2	2^1	0

(6x**1**, 1x**2**, 4x$\mathbf{2^2}$)

	1	2	3	4
1	2	1	1	0
2	1	0	1	2^2
3	1	1	0	2^2
4	0	2^2	2^2	0

(4x**2**, 4x$\mathbf{2^1}$)

	1	2	3	4
1	2	2^1	0	0
2	2^1	2	0	0
3	0	0	2	2^1
4	0	0	2^1	2

(4x**2**, 2x$\mathbf{2^1}$, 2x$\mathbf{2^2}$)

	1	2	3	4
1	2	2^2	0	0
2	2^2	2	0	0
3	0	0	2	2^1
4	0	0	2^1	2

(4x**2**, 4x$\mathbf{2^2}$)

	1	2	3	4
1	2	2^2	0	0
2	2^2	2	0	0
3	0	0	2	2^2
4	0	0	2^2	2

(2x**2**, 6x$\mathbf{2^1}$)

	1	2	3	4
1	0	2^1	2^1	0
2	2^1	0	0	2^1
3	2^1	0	2	0
4	0	2^1	0	2

(2x**2**, 4x$\mathbf{2^1}$, 2x$\mathbf{2^2}$) a

	1	2	3	4
1	0	2^2	2^1	0
2	2^2	0	0	2^1
3	2^1	0	2	0
4	0	2^1	0	2

(2x**2**, 4x$\mathbf{2^1}$, 2x$\mathbf{2^2}$) b

	1	2	3	4
1	0	2^1	2^2	0
2	2^1	0	0	2^1
3	2^2	0	2	0
4	0	2^1	0	2

(2x**2**, 2x$\mathbf{2^1}$, 4x$\mathbf{2^2}$) a

	1	2	3	4
1	0	2^2	2^2	0
2	2^2	0	0	2^1
3	2^2	0	2	0
4	0	2^1	0	2

(2x**2**, 2x$\mathbf{2^1}$, 4x$\mathbf{2^2}$) b

	1	2	3	4
1	0	2^1	2^2	0
2	2^1	0	0	2^2
3	2^2	0	2	0
4	0	2^2	0	2

(2x**2**, 6x$\mathbf{2^2}$)

	1	2	3	4
1	0	2^2	2^2	0
2	2^2	0	0	2^2
3	2^2	0	2	0
4	0	2^2	0	2

(8x$\mathbf{2^1}$)

	1	2	3	4
1	0	2^1	2^1	0
2	2^1	0	0	2^1
3	2^1	0	0	2^1
4	0	2^1	2^1	0

(6x$\mathbf{2^1}$, 2x$\mathbf{2^2}$)

	1	2	3	4
1	0	2^2	2^1	0
2	2^2	0	0	2^1
3	2^1	0	0	2^1
4	0	2^1	2^1	0

(4x$\mathbf{2^1}$, 4x$\mathbf{2^2}$) a

	1	2	3	4
1	0	2^2	2^1	0
2	2^2	0	0	2^1
3	2^1	0	0	2^2
4	0	2^1	2^2	0

(4x$\mathbf{2^1}$, 4x$\mathbf{2^2}$) **b**

	1	2	3	4
1	0	2^2	2^1	0
2	2^2	0	0	2^2
3	2^1	0	0	2^1
4	0	2^2	2^1	0

(2x$\mathbf{2^1}$, 6x$\mathbf{2^2}$)

	1	2	3	4
1	0	2^2	2^1	0
2	2^2	0	0	2^2
3	2^1	0	0	2^2
4	0	2^2	2^2	0

(8x$\mathbf{2^2}$)

	1	2	3	4
1	0	2^2	2^2	0
2	2^2	0	0	2^2
3	2^2	0	0	2^2
4	0	2^2	2^2	0

$^{4}V_{5}$ NG

$^{4}V_{6}$ NG

$^{4}V_{7}$ NG

$^{4}V_{8}$ NG

Rank 2

$^{1}V_{2}{}^{2}V_{1}$

(4x**1**)

	1	2	3
1	0	1	0
2	1	0	1
3	0	1	0

(2x**1**, 1x**2**)

	1	2	3
1	2	0	0
2	0	0	1
3	0	1	0

$^{1}V_{2}{}^{2}V_{2}$

(6x**1**)

	1	2	3	4
1	0	1	0	1
2	1	0	1	0
3	0	1	0	0
4	1	0	0	0

(4x**1**, 1x**2**)

	1	2	3	4
1	2	0	0	0
2	0	0	1	1
3	0	1	0	0
4	0	1	0	0

(2x**1**, 2x**2**)

	1	2	3	4
1	2	0	0	0
2	0	0	1	0
3	0	1	0	0
4	0	0	0	2

(2x**1**, 2x**2**^{1})

	1	2	3	4
1	0	1	0	0
2	1	0	0	0
3	0	0	0	2^1
4	0	0	2^1	0

(2x**1**, 2x**2**^{2})

	1	2	3	4
1	0	1	0	0
2	1	0	0	0
3	0	0	0	2^2
4	0	0	2^2	0

$^{1}V_{2}{}^{2}V_{3}$

(8x**1**) **a**

	1	2	3	4	5
1	0	0	0	1	0
2	0	0	1	1	0
3	0	1	0	0	1
4	1	1	0	0	0
5	0	0	1	0	0

(8x**1**) **b**

	1	2	3	4	5
1	0	1	1	0	0
2	1	0	1	0	0
3	1	1	0	0	0
4	0	0	0	0	1
5	0	0	0	1	0

(6x**1**, 1x**2**)

	1	2	3	4	5
1	2	0	0	0	0
2	0	0	1	1	0
3	0	1	0	0	1
4	0	1	0	0	0
5	0	0	1	0	0

(4x**1**, 2x**2**)

	1	2	3	4	5
1	2	0	0	0	0
2	0	0	1	1	0
3	0	1	0	0	0
4	0	1	0	0	0
5	0	0	0	0	2

(4x**1**, 2x**2**^{1})

	1	2	3	4	5
1	0	0	0	0	2^1
2	0	0	1	1	0
3	0	1	0	0	0
4	0	1	0	0	0
5	2^1	0	0	0	0

(4x**1**, 2x**2**^{2})

	1	2	3	4	5
1	0	0	0	0	2^2
2	0	0	1	1	0
3	0	1	0	0	0
4	0	1	0	0	0
5	2^2	0	0	0	0

(2x**1**, 3x**2**)

	1	2	3	4	5
1	2	0	0	0	0
2	0	2	0	0	0
3	0	0	2	0	0
4	0	0	0	0	1
5	0	0	0	1	0

(2x**1**, 1x**2**, 2x**2**^{1})

	1	2	3	4	5
1	0	2^1	0	0	0
2	2^1	0	0	0	0
3	0	0	0	1	0
4	0	0	1	0	0
5	0	0	0	0	2

(2x**1**, 1x**2**, 2x**2**^{2})

	1	2	3	4	5
1	0	2^2	0	0	0
2	2^2	0	0	0	0
3	0	0	0	1	0
4	0	0	1	0	0
5	0	0	0	0	2

$^{1}V_{2}{}^{2}V_{4}$

(10x**1**) **a**

	1	2	3	4	5	6
1	0	1	0	0	0	0
2	1	0	1	0	0	0
3	0	1	0	1	0	0
4	0	0	1	0	1	0
5	0	0	0	1	0	1
6	0	0	0	0	1	0

(10x**1**) **b**

	1	2	3	4	5	6
1	0	0	0	1	0	0
2	0	0	1	0	0	1
3	0	1	0	0	1	0
4	1	0	0	0	0	0
5	0	0	1	0	0	1
6	0	1	0	0	1	0

(10x**1**) **c**

	1	2	3	4	5	6
1	0	1	1	0	0	0
2	1	0	1	0	0	0
3	1	1	0	0	0	0
4	0	0	0	0	1	1
5	0	0	0	1	0	0
6	0	0	0	1	0	0

(8x**1**, 1x**2**) **a**

	1	2	3	4	5	6
1	2	0	0	0	0	0
2	0	0	1	0	0	0
3	0	1	0	1	0	0
4	0	0	1	0	1	0
5	0	0	0	1	0	1
6	0	0	0	0	1	0

(8x**1**, 1x**2**) **b**

	1	2	3	4	5	6
1	0	1	1	0	0	0
2	1	0	1	0	0	0
3	1	1	0	0	0	0
4	0	0	0	0	1	0
5	0	0	0	1	0	0
6	0	0	0	0	0	2

(6x**1**, 2x**2**)

	1	2	3	4	5	6
1	2	0	0	0	0	0
2	0	2	0	0	0	0
3	0	0	0	1	0	0
4	0	0	1	0	1	0
5	0	0	0	1	0	1
6	0	0	0	0	1	0

(6x**1**, 2x**2**^{1})

	1	2	3	4	5	6
1	0	1	0	0	0	0
2	1	0	1	0	0	0
3	0	1	0	1	0	0
4	0	0	1	0	0	0
5	0	0	0	0	0	2^1
6	0	0	0	0	2^1	0

(6x**1**, 2x**2**^{2})

	1	2	3	4	5	6
1	0	1	0	0	0	0
2	1	0	1	0	0	0
3	0	1	0	1	0	0
4	0	0	1	0	0	0
5	0	0	0	0	0	2^2
6	0	0	0	0	2^2	0

(4x**1**, 3x**2**)

	1	2	3	4	5	6
1	2	0	0	0	0	0
2	0	2	0	0	0	0
3	0	0	2	0	0	0
4	0	0	0	0	1	0
5	0	0	0	1	0	1
6	0	0	0	0	1	0

(4x**1**, 1x**2**, 2x**2**^{1})

	1	2	3	4	5	6
1	2	0	0	0	0	0
2	0	0	1	0	0	0
3	0	1	0	1	0	0
4	0	0	1	0	0	0
5	0	0	0	0	0	2^1
6	0	0	0	0	2^1	0

(4x**1**, 1x**2**, 2x**2**^{2})

	1	2	3	4	5	6
1	2	0	0	0	0	0
2	0	0	1	0	0	0
3	0	1	0	1	0	0
4	0	0	1	0	0	0
5	0	0	0	0	0	2^2
6	0	0	0	0	2^2	0

(2x**1**, 4x**2**)

	1	2	3	4	5	6
1	0	1	0	0	0	0
2	1	0	0	0	0	0
3	0	0	2	0	0	0
4	0	0	0	2	0	0
5	0	0	0	0	2	0
6	0	0	0	0	0	2

(2x**1**, 2x**2**, 2x**2**^{1})

	1	2	3	4	5	6
1	2	0	0	0	0	0
2	0	0	1	0	0	0
3	0	1	0	0	0	0
4	0	0	0	2	0	0
5	0	0	0	0	0	2^1
6	0	0	0	0	2^1	0

(2x**1**, 2x**2**, 2x**2**^{2})

	1	2	3	4	5	6
1	2	0	0	0	0	0
2	0	0	1	0	0	0
3	0	1	0	0	0	0
4	0	0	0	2	0	0
5	0	0	0	0	0	2^2
6	0	0	0	0	2^2	0

(2x**1**, 4x**2**^{1})

	1	2	3	4	5	6
1	0	1	0	0	0	0
2	1	0	0	0	0	0
3	0	0	0	2^1	0	0
4	0	0	2^1	0	0	0
5	0	0	0	0	0	2^1
6	0	0	0	0	2^1	0

(2x**1**, 2x**2**^{1}, 2x**2**^{2})

	1	2	3	4	5	6
1	0	1	0	0	0	0
2	1	0	0	0	0	0
3	0	0	0	2^1	0	0
4	0	0	2^1	0	0	0
5	0	0	0	0	0	2^2
6	0	0	0	0	2^2	0

(2x**1**, 4x**2**^{2})

	1	2	3	4	5	6
1	0	1	0	0	0	0
2	1	0	0	0	0	0
3	0	0	0	2^2	0	0
4	0	0	2^2	0	0	0
5	0	0	0	0	0	2^2
6	0	0	0	0	2^2	0

$^{1}V_{2}{}^{2}V_{5}$

NG

$^{1}V_{2}{}^{2}V_{6}$

NG

$^{1}V_{4}{}^{2}V_{1}$

(6x**1**)

	1	2	3	4	5
1	0	1	0	0	0
2	1	0	1	0	0
3	0	1	0	0	0
4	0	0	0	0	1
5	0	0	0	1	0

(4x**1**, 1x**2**)

	1	2	3	4	5
1	2	0	0	0	0
2	0	0	1	0	0
3	0	1	0	0	0
4	0	0	0	0	1
5	0	0	0	1	0

$^{1}V_{4}{}^{2}V_{2}$

(8x**1**) **a**

	1	2	3	4	5	6
1	0	1	0	1	0	0
2	1	0	1	0	0	0
3	0	1	0	0	0	0
4	1	0	0	0	0	0
5	0	0	0	0	0	1
6	0	0	0	0	1	0

(8x**1**) **b**

	1	2	3	4	5	6
1	0	1	0	0	0	0
2	1	0	1	0	0	0
3	0	1	0	0	0	0
4	0	0	0	0	1	0
5	0	0	0	1	0	1
6	0	0	0	0	1	0

(6x**1**, 1x**2**)

	1	2	3	4	5	6
1	0	1	0	0	0	0
2	1	0	1	0	0	0
3	0	1	0	0	0	0
4	0	0	0	0	1	0
5	0	0	0	1	0	0
6	0	0	0	0	0	2

(4x**1**, 2x**2**)

	1	2	3	4	5	6
1	2	0	0	0	0	0
2	0	2	0	0	0	0
3	0	0	0	1	0	0
4	0	0	1	0	0	0
5	0	0	0	0	0	1
6	0	0	0	0	1	0

(4x**1**, 2x$\mathbf{2}^{1}$)

	1	2	3	4	5	6
1	0	1	0	0	0	0
2	1	0	0	0	0	0
3	0	0	0	1	0	0
4	0	0	1	0	0	0
5	0	0	0	0	0	2^{1}
6	0	0	0	0	2^{1}	0

(4x**1**, 2x$\mathbf{2}^{2}$)

	1	2	3	4	5	6
1	0	1	0	0	0	0
2	1	0	0	0	0	0
3	0	0	0	1	0	0
4	0	0	1	0	0	0
5	0	0	0	0	0	2^{2}
6	0	0	0	0	2^{2}	0

$^{1}V_{4}{}^{2}V_{3}$

(10x**1**) **a**

	1	2	3	4	5	6	7
1	0	1	1	0	0	0	0
2	1	0	1	0	0	0	0
3	1	1	0	0	0	0	0
4	0	0	0	0	1	0	0
5	0	0	0	1	0	0	0
6	0	0	0	0	0	0	1
7	0	0	0	0	0	1	0

(10x**1**) **b**

	1	2	3	4	5	6	7
1	0	1	1	0	0	0	0
2	1	0	0	1	0	0	0
3	1	0	0	0	1	0	0
4	0	1	0	0	0	0	0
5	0	0	1	0	0	0	0
6	0	0	0	0	0	0	1
7	0	0	0	0	0	1	0

(10x**1**) **c**

	1	2	3	4	5	6	7
1	0	1	1	0	0	0	0
2	1	0	0	1	0	0	0
3	1	0	0	0	0	0	0
4	0	1	0	0	0	0	0
5	0	0	0	0	0	0	1
6	0	0	0	0	0	0	1
7	0	0	0	0	1	1	0

(8x**1**, 1x**2**) **a**

	1	2	3	4	5	6	7
1	0	1	1	0	0	0	0
2	1	0	0	1	0	0	0
3	1	0	0	0	0	0	0
4	0	1	0	0	0	0	0
5	0	0	0	0	2	0	0
6	0	0	0	0	0	0	1
7	0	0	0	0	0	1	0

(8x**1**, 1x**2**) **b**

	1	2	3	4	5	6	7
1	0	1	0	0	0	0	0
2	1	0	1	0	0	0	0
3	0	1	0	0	0	0	0
4	0	0	0	0	1	0	0
5	0	0	0	1	0	1	0
6	0	0	0	0	1	0	0
7	0	0	0	0	0	0	2

(6x**1**, 2x**2**)

	1	2	3	4	5	6	7
1	0	1	0	0	0	0	0
2	1	0	1	0	0	0	0
3	0	1	0	0	0	0	0
4	0	0	0	0	1	0	0
5	0	0	0	1	0	0	0
6	0	0	0	0	0	2	0
7	0	0	0	0	0	0	2

(6x**1**, 2x$\mathbf{2}^{1}$)

	1	2	3	4	5	6	7
1	0	1	0	0	0	0	0
2	1	0	1	0	0	0	0
3	0	1	0	0	0	0	0
4	0	0	0	0	1	0	0
5	0	0	0	1	0	0	0
6	0	0	0	0	0	0	2^{1}
7	0	0	0	0	0	2^{1}	0

(6x**1**, 2x$\mathbf{2}^{2}$)

	1	2	3	4	5	6	7
1	0	1	0	0	0	0	0
2	1	0	1	0	0	0	0
3	0	1	0	0	0	0	0
4	0	0	0	0	1	0	0
5	0	0	0	1	0	0	0
6	0	0	0	0	0	0	2^{2}
7	0	0	0	0	0	2^{2}	0

(4x**1**, 3x**2**)

	1	2	3	4	5	6	7
1	0	1	0	0	0	0	0
2	1	0	0	0	0	0	0
3	0	0	0	1	0	0	0
4	0	0	1	0	0	0	0
5	0	0	0	0	2	0	0
6	0	0	0	0	0	2	0
7	0	0	0	0	0	0	2

(4x**1**, 1x**2**, 2x$\mathbf{2}^{1}$)

	1	2	3	4	5	6	7
1	0	1	0	0	0	0	0
2	1	0	0	0	0	0	0
3	0	0	0	1	0	0	0
4	0	0	1	0	0	0	0
5	0	0	0	0	2	0	0
6	0	0	0	0	0	0	2^{1}
7	0	0	0	0	0	2^{1}	0

(4x**1**, 1x**2**, 2x$\mathbf{2}^{2}$)

	1	2	3	4	5	6	7
1	0	1	0	0	0	0	0
2	1	0	0	0	0	0	0
3	0	0	0	1	0	0	0
4	0	0	1	0	0	0	0
5	0	0	0	0	2	0	0
6	0	0	0	0	0	0	2^{2}
7	0	0	0	0	0	2^{2}	0

${}^1V_4{}^2V_4$

(12x**1**) **a**

	1	2	3	4	5	6	7	8
1	0	1	1	0	0	0	0	0
2	1	0	0	1	0	0	0	0
3	1	0	0	0	0	0	0	0
4	0	1	0	0	0	0	0	0
5	0	0	0	0	0	1	1	0
6	0	0	0	0	1	0	0	1
7	0	0	0	0	1	0	0	0
8	0	0	0	0	0	1	0	0

(12x**1**) **b**

	1	2	3	4	5	6	7	8
1	0	1	1	0	0	0	0	0
2	1	0	0	1	0	0	0	0
3	1	0	0	0	1	0	0	0
4	0	1	0	0	0	0	0	0
5	0	0	1	0	0	0	0	0
6	0	0	0	0	0	0	1	0
7	0	0	0	0	0	1	0	1
8	0	0	0	0	0	0	1	0

(12x**1**) **c**

	1	2	3	4	5	6	7	8
1	0	1	1	0	0	0	0	0
2	1	0	0	1	0	0	0	0
3	1	0	0	0	1	0	0	0
4	0	1	0	0	0	0	0	0
5	0	0	1	0	0	1	0	0
6	0	0	0	0	1	0	0	0
7	0	0	0	0	0	0	0	1
8	0	0	0	0	0	0	1	0

(12x**1**) **d**

	1	2	3	4	5	6	7	8
1	0	1	1	0	0	0	0	0
2	1	0	0	1	0	0	0	0
3	1	0	0	1	0	0	0	0
4	0	1	1	0	0	0	0	0
5	0	0	0	0	0	1	0	0
6	0	0	0	0	1	0	0	0
7	0	0	0	0	0	0	0	1
8	0	0	0	0	0	0	1	0

(12x**1**) **e**

	1	2	3	4	5	6	7	8
1	0	1	0	1	0	0	0	0
2	1	0	0	1	0	0	0	0
3	0	0	0	0	1	0	0	0
4	1	1	0	0	0	0	0	0
5	0	0	1	0	0	1	0	0
6	0	0	0	0	1	0	0	0
7	0	0	0	0	0	0	0	1
8	0	0	0	0	0	0	1	0

(10x**1**, 1x**2**) **a**

	1	2	3	4	5	6	7	8
1	0	1	1	0	0	0	0	0
2	1	0	1	0	0	0	0	0
3	1	1	0	0	0	0	0	0
4	0	0	0	0	1	0	0	0
5	0	0	0	1	0	0	0	0
6	0	0	0	0	0	0	1	0
7	0	0	0	0	0	1	0	0
8	0	0	0	0	0	0	0	2

(10x**1**, 1x**2**) **b**

	1	2	3	4	5	6	7	8
1	0	1	1	0	0	0	0	0
2	1	0	0	1	0	0	0	0
3	1	0	0	0	1	0	0	0
4	0	1	0	0	0	0	0	0
5	0	0	1	0	0	0	0	0
6	0	0	0	0	0	0	1	0
7	0	0	0	0	0	1	0	0
8	0	0	0	0	0	0	0	2

(10x**1**, 1x**2**) **c**

	1	2	3	4	5	6	7	8
1	0	1	1	0	0	0	0	0
2	1	0	0	1	0	0	0	0
3	1	0	0	0	0	0	0	0
4	0	1	0	0	0	0	0	0
5	0	0	0	0	0	0	1	0
6	0	0	0	0	0	0	1	0
7	0	0	0	0	1	1	0	0
8	0	0	0	0	0	0	0	2

(8x**1**, 2x**2**) **a**

	1	2	3	4	5	6	7	8
1	0	1	1	0	0	0	0	0
2	1	0	0	1	0	0	0	0
3	1	0	0	0	0	0	0	0
4	0	1	0	0	0	0	0	0
5	0	0	0	0	0	1	0	0
6	0	0	0	0	1	0	0	0
7	0	0	0	0	0	0	2	0
8	0	0	0	0	0	0	0	2

(8x**1**, 2x**2**) **b**

	1	2	3	4	5	6	7	8
1	0	1	0	0	0	0	0	0
2	1	0	0	1	0	0	0	0
3	0	0	0	0	0	1	0	0
4	0	1	0	0	0	0	0	0
5	0	0	0	0	0	1	0	0
6	0	0	1	0	1	0	0	0
7	0	0	0	0	0	0	2	0
8	0	0	0	0	0	0	0	2

(8x**1**, 2x**2**^{1}) **a**

	1	2	3	4	5	6	7	8
1	0	1	1	0	0	0	0	0
2	1	0	0	1	0	0	0	0
3	1	0	0	0	0	0	0	0
4	0	1	0	0	0	0	0	0
5	0	0	0	0	0	1	0	0
6	0	0	0	0	1	0	0	0
7	0	0	0	0	0	0	0	2^1
8	0	0	0	0	0	0	2^1	0

(8x**1**, 2x**2**^{2}) **a**

	1	2	3	4	5	6	7	8
1	0	1	1	0	0	0	0	0
2	1	0	0	1	0	0	0	0
3	1	0	0	0	0	0	0	0
4	0	1	0	0	0	0	0	0
5	0	0	0	0	0	1	0	0
6	0	0	0	0	1	0	0	0
7	0	0	0	0	0	0	0	2^2
8	0	0	0	0	0	0	2^2	0

(8x**1**, 2x**2**^{1}) **b**

	1	2	3	4	5	6	7	8
1	0	1	0	0	0	0	0	0
2	1	0	1	0	0	0	0	0
3	0	1	0	0	0	0	0	0
4	0	0	0	0	1	0	0	0
5	0	0	0	1	0	1	0	0
6	0	0	0	0	1	0	0	0
7	0	0	0	0	0	0	0	2^1
8	0	0	0	0	0	0	2^1	0

(8x**1**, 2x**2**^{2}) **b**

	1	2	3	4	5	6	7	8
1	0	1	0	0	0	0	0	0
2	1	0	1	0	0	0	0	0
3	0	1	0	0	0	0	0	0
4	0	0	0	0	1	0	0	0
5	0	0	0	1	0	1	0	0
6	0	0	0	0	1	0	0	0
7	0	0	0	0	0	0	0	2^2
8	0	0	0	0	0	0	2^2	0

(6x**1**, 3x**2**)

	1	2	3	4	5	6	7	8
1	0	1	0	0	0	0	0	0
2	1	0	1	0	0	0	0	0
3	0	1	0	0	0	0	0	0
4	0	0	0	0	1	0	0	0
5	0	0	0	1	0	0	0	0
6	0	0	0	0	0	2	0	0
7	0	0	0	0	0	0	2	0
8	0	0	0	0	0	0	0	2

(6x**1**, 1x**2**, 2x**2**^{1})

	1	2	3	4	5	6	7	8
1	0	1	0	0	0	0	0	0
2	1	0	1	0	0	0	0	0
3	0	1	0	0	0	0	0	0
4	0	0	0	0	1	0	0	0
5	0	0	0	1	0	0	0	0
6	0	0	0	0	0	0	2^1	0
7	0	0	0	0	0	2^1	0	0
8	0	0	0	0	0	0	0	2

(6x**1**, 1x**2**, 2x**2**^{2})

	1	2	3	4	5	6	7	8
1	0	1	0	0	0	0	0	0
2	1	0	1	0	0	0	0	0
3	0	1	0	0	0	0	0	0
4	0	0	0	0	1	0	0	0
5	0	0	0	1	0	0	0	0
6	0	0	0	0	0	0	2^2	0
7	0	0	0	0	0	2^2	0	0
8	0	0	0	0	0	0	0	2

(4x**1**, 4x**2**)

	1	2	3	4	5	6	7	8
1	0	1	0	0	0	0	0	0
2	1	0	0	0	0	0	0	0
3	0	0	0	1	0	0	0	0
4	0	0	1	0	0	0	0	0
5	0	0	0	0	2	0	0	0
6	0	0	0	0	0	2	0	0
7	0	0	0	0	0	0	2	0
8	0	0	0	0	0	0	0	2

(4x**1**, 2x**2**, 2x**2**^{1})

	1	2	3	4	5	6	7	8
1	0	1	0	0	0	0	0	0
2	1	0	0	0	0	0	0	0
3	0	0	0	1	0	0	0	0
4	0	0	1	0	0	0	0	0
5	0	0	0	0	2	0	0	0
6	0	0	0	0	0	0	2^1	0
7	0	0	0	0	0	2^1	0	0
8	0	0	0	0	0	0	0	2

(4x**1**, 2x**2**, 2x**2**^{2})

	1	2	3	4	5	6	7	8
1	0	1	0	0	0	0	0	0
2	1	0	0	0	0	0	0	0
3	0	0	0	1	0	0	0	0
4	0	0	1	0	0	0	0	0
5	0	0	0	0	2	0	0	0
6	0	0	0	0	0	0	2^2	0
7	0	0	0	0	0	2^2	0	0
8	0	0	0	0	0	0	0	2

(4x**1**, 4x**2**^{1})

	1	2	3	4	5	6	7	8
1	0	1	0	0	0	0	0	0
2	1	0	0	0	0	0	0	0
3	0	0	0	1	0	0	0	0
4	0	0	1	0	0	0	0	0
5	0	0	0	0	0	2^1	0	0
6	0	0	0	0	2^1	0	0	0
7	0	0	0	0	0	0	0	2^1
8	0	0	0	0	0	0	2^1	0

(4x**1**, 2x**2**^{1}, 2x**2**^{2})

	1	2	3	4	5	6	7	8
1	0	1	0	0	0	0	0	0
2	1	0	0	0	0	0	0	0
3	0	0	0	1	0	0	0	0
4	0	0	1	0	0	0	0	0
5	0	0	0	0	0	2^1	0	0
6	0	0	0	0	2^1	0	0	0
7	0	0	0	0	0	0	0	2^2
8	0	0	0	0	0	0	2^2	0

(4x**1**, 4x**2**^{2})

	1	2	3	4	5	6	7	8
1	0	1	0	0	0	0	0	0
2	1	0	0	0	0	0	0	0
3	0	0	0	1	0	0	0	0
4	0	0	1	0	0	0	0	0
5	0	0	0	0	0	2^2	0	0
6	0	0	0	0	2^2	0	0	0
7	0	0	0	0	0	0	0	2^2
8	0	0	0	0	0	0	2^2	0

$^1V_6{}^2V_1$

(8x**1**)

	1	2	3	4	5	6	7
1	0	1	0	0	0	0	0
2	1	0	1	0	0	0	0
3	0	1	0	0	0	0	0
4	0	0	0	0	1	0	0
5	0	0	0	1	0	0	0
6	0	0	0	0	0	0	1
7	0	0	0	0	0	1	0

(6x**1**, 1x**2**)

	1	2	3	4	5	6	7
1	2	0	0	0	0	0	0
2	0	0	1	0	0	0	0
3	0	1	0	0	0	0	0
4	0	0	0	0	1	0	0
5	0	0	0	1	0	0	0
6	0	0	0	0	0	0	1
7	0	0	0	0	0	1	0

$^1V_6{}^2V_2$

(10x**1**) **a**

	1	2	3	4	5	6	7	8
1	0	1	0	0	0	0	0	0
2	1	0	1	0	0	0	0	0
3	0	1	0	0	0	0	0	0
4	0	0	0	0	1	0	0	0
5	0	0	0	1	0	1	0	0
6	0	0	0	0	1	0	0	0
7	0	0	0	0	0	0	0	1
8	0	0	0	0	0	0	1	0

(10x**1**) **b**

	1	2	3	4	5	6	7	8
1	0	1	1	0	0	0	0	0
2	1	0	0	1	0	0	0	0
3	1	0	0	0	0	0	0	0
4	0	1	0	0	0	0	0	0
5	0	0	0	0	0	1	0	0
6	0	0	0	0	1	0	0	0
7	0	0	0	0	0	0	0	1
8	0	0	0	0	0	0	1	0

(8x**1**, 1x**2**)

	1	2	3	4	5	6	7	8
1	0	1	0	0	0	0	0	0
2	1	0	1	0	0	0	0	0
3	0	1	0	0	0	0	0	0
4	0	0	0	2	0	0	0	0
5	0	0	0	0	0	1	0	0
6	0	0	0	0	1	0	0	0
7	0	0	0	0	0	0	0	1
8	0	0	0	0	0	0	1	0

(6x**1**, 2x$\mathbf{2}^1$)

	1	2	3	4	5	6	7	8
1	0	1	0	0	0	0	0	0
2	1	0	0	0	0	0	0	0
3	0	0	0	1	0	0	0	0
4	0	0	1	0	0	0	0	0
5	0	0	0	0	0	1	0	0
6	0	0	0	0	1	0	0	0
7	0	0	0	0	0	0	0	2^1
8	0	0	0	0	0	0	2^1	0

(6x**1**, 2x$\mathbf{2}^2$)

	1	2	3	4	5	6	7	8
1	0	1	0	0	0	0	0	0
2	1	0	0	0	0	0	0	0
3	0	0	0	1	0	0	0	0
4	0	0	1	0	0	0	0	0
5	0	0	0	0	0	1	0	0
6	0	0	0	0	1	0	0	0
7	0	0	0	0	0	0	0	2^2
8	0	0	0	0	0	0	2^2	0

(6x**1**, 2x**2**)

	1	2	3	4	5	6	7	8
1	0	1	0	0	0	0	0	0
2	1	0	0	0	0	0	0	0
3	0	0	0	1	0	0	0	0
4	0	0	1	0	0	0	0	0
5	0	0	0	0	0	1	0	0
6	0	0	0	0	1	0	0	0
7	0	0	0	0	0	0	2	0
8	0	0	0	0	0	0	0	2

$\boldsymbol{^1V_r{}^3V_r}$

$^1V_1{}^3V_1$

(2x**1**, 1x**2**)

	1	2
1	2	1
2	1	0

$^1V_1{}^3V_3$

(6x**1**, 2x**2**)

	1	2	3	4
1	0	1	1	1
2	1	0	0	0
3	1	0	2	0
4	1	0	0	2

(6x**1**, 2x$\mathbf{2}^1$)

	1	2	3	4
1	0	1	0	0
2	1	0	1	1
3	0	1	0	2^1
4	0	1	2^1	0

(6x**1**, 2x$\mathbf{2}^2$)

	1	2	3	4
1	0	1	0	0
2	1	0	1	1
3	0	1	0	2^2
4	0	1	2^2	0

(4x**1**, 3x**2**)

	1	2	3	4
1	2	1	0	0
2	1	0	0	0
3	0	0	2	1
4	0	0	1	2

(4x**1**, 1x**2**, 2x$\mathbf{2}^1$)

	1	2	3	4
1	0	2^1	1	0
2	2^1	0	0	1
3	1	0	2	0
4	0	1	0	0

(4x**1**, 1x**2**, 2x$\mathbf{2}^2$)

	1	2	3	4
1	0	2^2	1	0
2	2^2	0	0	1
3	1	0	2	0
4	0	1	0	0

$^1V_1{}^3V_5$

(16x**1**)

	1	2	3	4	5	6
1	0	1	1	1	0	0
2	1	0	1	1	0	0
3	1	1	0	0	1	0
4	1	1	0	0	1	0
5	0	0	1	1	0	1
6	0	0	0	0	1	0

(14x**1**, 1x**2**) **a**

	1	2	3	4	5	6
1	0	1	1	1	0	0
2	1	0	1	1	0	0
3	1	1	0	1	0	0
4	1	1	1	0	0	0
5	0	0	0	0	0	1
6	0	0	0	0	1	2

(14x**1**, 1x**2**) **b**

	1	2	3	4	5	6
1	0	1	1	1	0	0
2	1	0	0	1	1	0
3	1	0	0	1	0	1
4	1	1	1	0	0	0
5	0	1	0	0	0	0
6	0	0	1	0	0	2

(12x**1**, 2x**2**)

	1	2	3	4	5	6
1	0	1	1	1	0	0
2	1	2	0	0	0	0
3	1	0	0	1	0	1
4	1	0	1	0	1	0
5	0	0	0	1	0	0
6	0	0	1	0	0	2

(12x**1**, 2x$\mathbf{2}^1$)

	1	2	3	4	5	6
1	0	2^1	1	0	0	0
2	2^1	0	0	1	0	0
3	1	0	0	1	1	0
4	0	1	1	0	1	0
5	0	0	1	1	0	1
6	0	0	0	0	1	0

(12x**1**, 2x$\mathbf{2}^2$)

	1	2	3	4	5	6
1	0	2^2	1	0	0	0
2	2^2	0	0	1	0	0
3	1	0	0	1	1	0
4	0	1	1	0	1	0
5	0	0	1	1	0	1
6	0	0	0	0	1	0

(10x**1**, 3x**2**)

	1	2	3	4	5	6
1	0	1	1	1	0	0
2	1	2	0	0	0	0
3	1	0	0	0	1	1
4	1	0	0	0	0	0
5	0	0	1	0	2	0
6	0	0	1	0	0	2

(10x**1**, 1x2, 2x$\mathbf{2}^1$) **a**

	1	2	3	4	5	6
1	0	2^1	1	0	0	0
2	2^1	0	0	1	0	0
3	1	0	0	1	1	0
4	0	1	1	0	0	1
5	0	0	1	0	2	0
6	0	0	0	1	0	0

(10x**1**, 1x2, 2x$\mathbf{2}^1$) **b**

	1	2	3	4	5	6
1	0	0	2^1	1	0	0
2	0	0	0	0	1	0
3	2^1	0	0	1	0	0
4	1	0	1	0	1	0
5	0	1	0	1	0	1
6	0	0	0	0	1	2

(10x**1**, 1x2, 2x$\mathbf{2}^2$) **a**

	1	2	3	4	5	6
1	0	2^2	1	0	0	0
2	2^2	0	0	1	0	0
3	1	0	0	1	1	0
4	0	1	1	0	0	1
5	0	0	1	0	2	0
6	0	0	0	1	0	0

(10x**1**, 1x2, 2x$\mathbf{2}^2$) **b**

	1	2	3	4	5	6
1	0	0	2^2	1	0	0
2	0	0	0	0	1	0
3	2^2	0	0	1	0	0
4	1	0	1	0	1	0
5	0	1	0	1	0	1
6	0	0	0	0	1	2

(8x**1**, 4x**2**) **a**

	1	2	3	4	5	6
1	0	1	1	1	0	0
2	1	2	0	0	0	0
3	1	0	2	0	0	0
4	1	0	0	2	0	0
5	0	0	0	0	2	1
6	0	0	0	0	1	0

(8x**1**, 4x**2**) **b**

	1	2	3	4	5	6
1	0	1	1	1	0	0
2	1	0	0	0	0	0
3	1	0	2	0	0	0
4	1	0	0	2	0	0
5	0	0	0	0	2	1
6	0	0	0	0	1	2

(8x**1**, 4x**2**) **c**

	1	2	3	4	5	6
1	2	1	0	0	0	0
2	1	2	0	0	0	0
3	0	0	2	1	0	0
4	0	0	1	2	0	0
5	0	0	0	0	0	1
6	0	0	0	0	1	2

(8x**1**, 2x**2**, 2x$\mathbf{2}^1$) **a**

	1	2	3	4	5	6
1	0	2^1	1	0	0	0
2	2^1	0	1	0	0	0
3	1	1	0	1	0	0
4	0	0	1	0	0	0
5	0	0	0	0	2	1
6	0	0	0	0	1	2

(8x**1**, 2x**2**, 2x$\mathbf{2}^1$) **b**

	1	2	3	4	5	6
1	0	2^1	1	0	0	0
2	2^1	0	1	0	0	0
3	1	1	0	1	0	0
4	0	0	1	2	0	0
5	0	0	0	0	2	1
6	0	0	0	0	1	0

(8x**1**, 2x**2**, 2x$\mathbf{2}^1$) **c**

	1	2	3	4	5	6
1	2	0	1	0	0	0
2	0	2	1	0	0	0
3	1	1	0	1	0	0
4	0	0	1	0	2^1	0
5	0	0	0	2^1	0	1
6	0	0	0	0	1	0

(8x**1**, 2x**2**, 2x$\mathbf{2}^1$) **d**

	1	2	3	4	5	6
1	0	0	1	0	0	0
2	0	2	1	0	0	0
3	1	1	0	1	0	0
4	0	0	1	0	2^1	0
5	0	0	0	2^1	0	1
6	0	0	0	0	1	2

(8x**1**, 2x**2**, 2x$\mathbf{2}^2$) **a**

	1	2	3	4	5	6
1	0	2^2	1	0	0	0
2	2^2	0	1	0	0	0
3	1	1	0	1	0	0
4	0	0	1	0	0	0
5	0	0	0	0	2	1
6	0	0	0	0	1	2

(8x**1**, 2x**2**, 2x$\mathbf{2}^2$) **b**

	1	2	3	4	5	6
1	0	2^2	1	0	0	0
2	2^2	0	1	0	0	0
3	1	1	0	1	0	0
4	0	0	1	2	0	0
5	0	0	0	0	2	1
6	0	0	0	0	1	0

(8x**1**, 2x**2**, 2x$\mathbf{2}^2$) **c**

	1	2	3	4	5	6
1	2	0	1	0	0	0
2	0	2	1	0	0	0
3	1	1	0	1	0	0
4	0	0	1	0	2^2	0
5	0	0	0	2^2	0	1
6	0	0	0	0	1	0

(8x**1**, 2x**2**, 2x$\mathbf{2}^2$) **d**

	1	2	3	4	5	6
1	0	0	1	0	0	0
2	0	2	1	0	0	0
3	1	1	0	1	0	0
4	0	0	1	0	2^2	0
5	0	0	0	2^2	0	1
6	0	0	0	0	1	2

(8x**1**, 4x$\mathbf{2}^1$) **a**

	1	2	3	4	5	6
1	0	2^1	1	0	0	0
2	2^1	0	1	0	0	0
3	1	1	0	1	0	0
4	0	0	1	0	2^1	0
5	0	0	0	2^1	0	1
6	0	0	0	0	1	0

(8x**1**, 4x$\mathbf{2}^1$) **b**

	1	2	3	4	5	6
1	0	1	2^1	0	0	0
2	1	0	0	2^1	0	0
3	2^1	0	0	0	0	1
4	0	2^1	0	0	0	1
5	0	0	0	0	0	1
6	0	0	1	1	1	0

(8x**1**, 2x$\mathbf{2}^1$, 2x$\mathbf{2}^2$) **a**

	1	2	3	4	5	6
1	0	2^2	1	0	0	0
2	2^2	0	1	0	0	0
3	1	1	0	1	0	0
4	0	0	1	0	2^1	0
5	0	0	0	2^1	0	1
6	0	0	0	0	1	0

(8x**1**, 2x$\mathbf{2}^1$, 2x$\mathbf{2}^2$) **b**

	1	2	3	4	5	6
1	0	2^1	1	0	0	0
2	2^1	0	1	0	0	0
3	1	1	0	1	0	0
4	0	0	1	0	2^2	0
5	0	0	0	2^2	0	1
6	0	0	0	0	1	0

(8x**1**, 2x$\mathbf{2}^1$, 2x$\mathbf{2}^2$) **c**

	1	2	3	4	5	6
1	0	1	2^2	0	0	0
2	1	0	0	2^1	0	0
3	2^2	0	0	0	0	1
4	0	2^1	0	0	0	1
5	0	0	0	0	0	1
6	0	0	1	1	1	0

(8x**1**, 4x$\mathbf{2}^2$) **a**

	1	2	3	4	5	6
1	0	2^2	1	0	0	0
2	2^2	0	1	0	0	0
3	1	1	0	1	0	0
4	0	0	1	0	2^2	0
5	0	0	0	2^2	0	1
6	0	0	0	0	1	0

(8x**1**, 4x$\mathbf{2}^2$) **b**

	1	2	3	4	5	6
1	0	1	2^2	0	0	0
2	1	0	0	2^2	0	0
3	2^2	0	0	0	0	1
4	0	2^2	0	0	0	1
5	0	0	0	0	0	1
6	0	0	1	1	1	0

(6x**1**, 3x**2**, 2x$\mathbf{2}^1$) **a**

	1	2	3	4	5	6
1	2	1	0	0	0	0
2	1	0	2^1	0	0	0
3	0	2^1	0	1	0	0
4	0	0	1	2	0	0
5	0	0	0	0	0	1
6	0	0	0	0	1	2

(6x**1**, 3x**2**, 2x$\mathbf{2}^{1}$) **b**

	1	2	3	4	5	6
1	2	1	0	0	0	0
2	1	0	2^1	0	0	0
3	0	2^1	0	1	0	0
4	0	0	1	0	0	0
5	0	0	0	0	2	1
6	0	0	0	0	1	2

(6x**1**, 3x**2**, 2x$\mathbf{2}^{2}$) **a**

	1	2	3	4	5	6
1	2	1	0	0	0	0
2	1	0	2^2	0	0	0
3	0	2^2	0	1	0	0
4	0	0	1	2	0	0
5	0	0	0	0	0	1
6	0	0	0	0	1	2

(6x**1**, 3x**2**, 2x$\mathbf{2}^{2}$) **b**

	1	2	3	4	5	6
1	2	1	0	0	0	0
2	1	0	2^2	0	0	0
3	0	2^2	0	1	0	0
4	0	0	1	0	0	0
5	0	0	0	0	2	1
6	0	0	0	0	1	2

(6x**1**, 1x**2**, 4x$\mathbf{2}^{1}$) **a**

	1	2	3	4	5	6
1	0	1	2^1	0	0	0
2	1	0	0	2^1	0	0
3	2^1	0	0	1	0	0
4	0	2^1	1	0	0	0
5	0	0	0	0	2	1
6	0	0	0	0	1	0

(6x**1**, 1x**2**, 4x$\mathbf{2}^{1}$) **b**

	1	2	3	4	5	6
1	2	1	0	0	0	0
2	1	0	2^1	0	0	0
3	0	2^1	0	1	0	0
4	0	0	1	0	2^1	0
5	0	0	0	2^1	0	1
6	0	0	0	0	1	0

(6x**1**, 1x**2**, 2x$\mathbf{2}^{1}$, 2x$\mathbf{2}^{2}$) **a**

	1	2	3	4	5	6
1	0	1	2^2	0	0	0
2	1	0	0	2^1	0	0
3	2^2	0	0	1	0	0
4	0	2^1	1	0	0	0
5	0	0	0	0	2	1
6	0	0	0	0	1	0

(6x**1**, 1x**2**, 2x$\mathbf{2}^{1}$, 2x$\mathbf{2}^{2}$) **b**

	1	2	3	4	5	6
1	2	1	0	0	0	0
2	1	0	2^2	0	0	0
3	0	2^2	0	1	0	0
4	0	0	1	0	2^1	0
5	0	0	0	2^1	0	1
6	0	0	0	0	1	0

(6x**1**, 1x**2**, 2x$\mathbf{2}^{1}$, 2x$\mathbf{2}^{2}$) **c**

	1	2	3	4	5	6
1	2	1	0	0	0	0
2	1	0	2^1	0	0	0
3	0	2^1	0	1	0	0
4	0	0	1	0	2^2	0
5	0	0	0	2^2	0	1
6	0	0	0	0	1	0

(6x**1**, 1x**2**, 4x$\mathbf{2}^{2}$) **a**

	1	2	3	4	5	6
1	0	1	2^2	0	0	0
2	1	0	0	2^2	0	0
3	2^2	0	0	1	0	0
4	0	2^2	1	0	0	0
5	0	0	0	0	2	1
6	0	0	0	0	1	0

(6x**1**, 1x**2**, 4x$\mathbf{2}^{2}$) **b**

	1	2	3	4	5	6
1	2	1	0	0	0	0
2	1	0	2^2	0	0	0
3	0	2^2	0	1	0	0
4	0	0	1	0	2^2	0
5	0	0	0	2^2	0	1
6	0	0	0	0	1	0

$^1V_1{}^3V_7$

NG

$^1V_2{}^3V_2$

(6x**1**, 1x**2**)

	1	2	3	4
1	0	1	1	1
2	1	0	0	0
3	1	0	2	0
4	1	0	0	0

(4x**1**, 2x**2**) **a**

	1	2	3	4
1	0	1	0	0
2	1	0	0	0
3	0	0	2	1
4	0	0	1	2

(4x**1**, 2x**2**) **a**

	1	2	3	4
1	0	1	0	0
2	1	2	0	0
3	0	0	2	1
4	0	0	1	0

(4x**1**, 2x$\mathbf{2}^{1}$)

	1	2	3	4
1	0	2^1	0	1
2	2^1	0	1	0
3	0	1	0	0
4	1	0	0	0

(4x**1**, 2x$\mathbf{2}^{2}$)

	1	2	3	4
1	0	2^2	0	1
2	2^2	0	1	0
3	0	1	0	0
4	1	0	0	0

$^1V_2{}^3V_4$

(14x**1**) **a**

	1	2	3	4	5	6
1	0	1	1	1	0	0
2	1	0	1	1	0	0
3	1	1	0	1	0	0
4	1	1	1	0	0	0
5	0	0	0	0	0	1
6	0	0	0	0	1	0

(14x**1**) **b**

	1	2	3	4	5	6
1	0	1	1	1	0	0
2	1	0	0	1	0	1
3	1	0	0	1	1	0
4	1	1	1	0	0	0
5	0	0	1	0	0	0
6	0	1	0	0	0	0

(12x**1**, 1x**2**)

	1	2	3	4	5	6
1	0	1	1	1	0	0
2	1	2	0	0	0	0
3	1	0	0	1	0	1
4	1	0	1	0	1	0
5	0	0	0	1	0	0
6	0	0	1	0	0	0

(10x**1**, 2x**2**) **a**

	1	2	3	4	5	6
1	0	1	1	1	0	0
2	1	2	0	0	0	0
3	1	0	0	0	1	1
4	1	0	0	0	0	0
5	0	0	1	0	2	0
6	0	0	1	0	0	0

(10x**1**, 2x**2**) **b**

	1	2	3	4	5	6
1	0	1	1	1	0	0
2	1	2	0	0	0	0
3	1	0	0	0	1	1
4	1	0	0	2	0	0
5	0	0	1	0	0	0
6	0	0	1	0	0	0

(10x**1**, 2x$\mathbf{2}^{1}$) **a**

	1	2	3	4	5	6
1	0	2^1	1	0	0	0
2	2^1	0	1	0	0	0
3	1	1	0	1	0	0
4	0	0	1	0	1	1
5	0	0	0	1	0	0
6	0	0	0	1	0	0

(10x**1**, 2x$\mathbf{2}^{1}$) **b**

	1	2	3	4	5	6
1	0	2^1	1	0	0	0
2	2^1	0	0	1	0	0
3	1	0	0	1	1	0
4	0	1	1	0	0	1
5	0	0	1	0	0	0
6	0	0	0	1	0	0

(10x**1**, 2x$\mathbf{2}^{2}$) **a**

	1	2	3	4	5	6
1	0	2^2	1	0	0	0
2	2^2	0	1	0	0	0
3	1	1	0	1	0	0
4	0	0	1	0	1	1
5	0	0	0	1	0	0
6	0	0	0	1	0	0

(10x**1**, 2x$\mathbf{2}^{2}$) **b**

	1	2	3	4	5	6
1	0	2^2	1	0	0	0
2	2^2	0	0	1	0	0
3	1	0	0	1	1	0
4	0	1	1	0	0	1
5	0	0	1	0	0	0
6	0	0	0	1	0	0

(8x**1**, 3x**2**) **a**

	1	2	3	4	5	6
1	0	1	1	1	0	0
2	1	0	0	0	0	0
3	1	0	2	0	0	0
4	1	0	0	0	0	0
5	0	0	0	0	2	1
6	0	0	0	0	1	2

(8x**1**, 3x**2**) **b**

	1	2	3	4	5	6
1	0	1	1	1	0	0
2	1	0	0	0	0	0
3	1	0	2	0	0	0
4	1	0	0	2	0	0
5	0	0	0	0	0	1
6	0	0	0	0	1	2

(8x**1**, 3x**2**) **c**

	1	2	3	4	5	6
1	0	1	1	1	0	0
2	1	2	0	0	0	0
3	1	0	2	0	0	0
4	1	0	0	2	0	0
5	0	0	0	0	0	1
6	0	0	0	0	1	0

(8x**1**, 1x**2**, 2x$\mathbf{2}^{1}$) **a**

	1	2	3	4	5	6
1	0	2^1	1	0	0	0
2	2^1	0	1	0	0	0
3	1	1	0	1	0	0
4	0	0	1	2	0	0
5	0	0	0	0	0	1
6	0	0	0	0	1	0

(8x**1**, 1x**2**, 2x$\mathbf{2}^{1}$) **b**

	1	2	3	4	5	6
1	0	2^1	1	0	0	0
2	2^1	0	1	0	0	0
3	1	1	0	1	0	0
4	0	0	1	0	0	0
5	0	0	0	0	2	1
6	0	0	0	0	1	0

(8x**1**, 1x**2**, 2x$\mathbf{2}^{1}$) **c**

	1	2	3	4	5	6
1	2	0	1	0	0	0
2	0	0	1	0	0	0
3	1	1	0	1	0	0
4	0	0	1	0	2^1	0
5	0	0	0	2^1	0	1
6	0	0	0	0	1	0

(8x**1**, 1x**2**, 2x**2**^{1}) **d**

	1	2	3	4	5	6
1	0	0	1	0	0	0
2	0	0	1	0	0	0
3	1	1	0	1	0	0
4	0	0	1	0	2^1	0
5	0	0	0	2^1	0	1
6	0	0	0	0	1	2

(8x**1**, 1x**2**, 2x**2**^{2}) **a**

	1	2	3	4	5	6
1	0	2^2	1	0	0	0
2	2^2	0	1	0	0	0
3	1	1	0	1	0	0
4	0	0	1	2	0	0
5	0	0	0	0	0	1
6	0	0	0	0	1	0

(8x**1**, 1x**2**, 2x**2**^{2}) **b**

	1	2	3	4	5	6
1	0	2^2	1	0	0	0
2	2^2	0	1	0	0	0
3	1	1	0	1	0	0
4	0	0	1	0	0	0
5	0	0	0	0	2	1
6	0	0	0	0	1	0

(8x**1**, 1x**2**, 2x**2**^{2}) **c**

	1	2	3	4	5	6
1	2	0	1	0	0	0
2	0	0	1	0	0	0
3	1	1	0	1	0	0
4	0	0	1	0	2^2	0
5	0	0	0	2^2	0	1
6	0	0	0	0	1	0

(8x**1**, 1x**2**, 2x**2**^{2}) **d**

	1	2	3	4	5	6
1	0	0	1	0	0	0
2	0	0	1	0	0	0
3	1	1	0	1	0	0
4	0	0	1	0	2^2	0
5	0	0	0	2^2	0	1
6	0	0	0	0	1	2

(6x**1**, 4x**2**) **a**

	1	2	3	4	5	6
1	2	1	0	0	0	0
2	1	2	0	0	0	0
3	0	0	2	1	0	0
4	0	0	1	2	0	0
5	0	0	0	0	0	1
6	0	0	0	0	1	0

(6x**1**, 4x**2**) **b**

	1	2	3	4	5	6
1	2	1	0	0	0	0
2	1	2	0	0	0	0
3	0	0	2	1	0	0
4	0	0	1	0	0	0
5	0	0	0	0	0	1
6	0	0	0	0	1	2

(6x**1**, 2x**2**, 2x**2**^{1}) **a**

	1	2	3	4	5	6
1	0	1	0	0	0	0
2	1	0	2^1	0	0	0
3	0	2^1	0	1	0	0
4	0	0	1	0	0	0
5	0	0	0	0	2	1
6	0	0	0	0	1	2

(6x**1**, 2x**2**, 2x**2**^{1}) **b**

	1	2	3	4	5	6
1	2	1	0	0	0	0
2	1	0	2^1	0	0	0
3	0	2^1	0	1	0	0
4	0	0	1	0	0	0
5	0	0	0	0	0	1
6	0	0	0	0	1	2

(6x**1**, 2x**2**, 2x**2**^{1}) **c**

	1	2	3	4	5	6
1	2	1	0	0	0	0
2	1	0	2^1	0	0	0
3	0	2^1	0	1	0	0
4	0	0	1	2	0	0
5	0	0	0	0	0	1
6	0	0	0	0	1	0

(6x**1**, 2x**2**, 2x**2**^{2}) **a**

	1	2	3	4	5	6
1	0	1	0	0	0	0
2	1	0	2^2	0	0	0
3	0	2^2	0	1	0	0
4	0	0	1	0	0	0
5	0	0	0	0	2	1
6	0	0	0	0	1	2

(6x**1**, 2x**2**, 2x**2**^{2}) **b**

	1	2	3	4	5	6
1	2	1	0	0	0	0
2	1	0	2^2	0	0	0
3	0	2^2	0	1	0	0
4	0	0	1	0	0	0
5	0	0	0	0	0	1
6	0	0	0	0	1	2

(6x**1**, 2x**2**, 2x**2**^{2}) **c**

	1	2	3	4	5	6
1	2	1	0	0	0	0
2	1	0	2^2	0	0	0
3	0	2^2	0	1	0	0
4	0	0	1	2	0	0
5	0	0	0	0	0	1
6	0	0	0	0	1	0

(6x**1**, 4x**2**^{1}) **a**

	1	2	3	4	5	6
1	0	1	0	0	0	0
2	1	0	2^1	0	0	0
3	0	2^1	0	1	0	0
4	0	0	1	0	2^1	0
5	0	0	0	2^1	0	1
6	0	0	0	0	1	0

(6x**1**, 4x**2**^{1}) **b**

	1	2	3	4	5	6
1	0	2^1	0	1	0	0
2	2^1	0	1	0	0	0
3	0	1	0	2^1	0	0
4	1	0	2^1	0	0	0
5	0	0	0	0	0	1
6	0	0	0	0	1	0

(6x**1**, 2x**2**^{1}, 2x**2**^{2}) **a**

	1	2	3	4	5	6
1	0	1	0	0	0	0
2	1	0	2^1	0	0	0
3	0	2^1	0	1	0	0
4	0	0	1	0	2^2	0
5	0	0	0	2^2	0	1
6	0	0	0	0	1	0

(6x**1**, 2x**2**^{1}, 2x**2**^{2}) **b**

	1	2	3	4	5	6
1	0	2^1	0	1	0	0
2	2^1	0	1	0	0	0
3	0	1	0	2^2	0	0
4	1	0	2^2	0	0	0
5	0	0	0	0	0	1
6	0	0	0	0	1	0

(6x**1**, 4x**2**^{2}) **a**

	1	2	3	4	5	6
1	0	1	0	0	0	0
2	1	0	2^2	0	0	0
3	0	2^2	0	1	0	0
4	0	0	1	0	2^2	0
5	0	0	0	2^2	0	1
6	0	0	0	0	1	0

(6x**1**, 4x**2**^{2}) **b**

	1	2	3	4	5	6
1	0	2^2	0	1	0	0
2	2^2	0	1	0	0	0
3	0	1	0	2^2	0	0
4	1	0	2^2	0	0	0
5	0	0	0	0	0	1
6	0	0	0	0	1	0

$^1V_2{}^3V_6$

NG

$^1V_3{}^3V_1$

(6x**1**)

	1	2	3	4
1	0	1	1	1
2	1	0	0	0
3	1	0	0	1
4	1	0	1	0

(4x**1**, 1x2)

	1	2	3	4
1	0	1	0	0
2	1	2	0	0
3	0	0	0	1
4	0	0	1	0

$^1V_3{}^3V_3$

(12x**1**)

	1	2	3	4	5	6
1	0	1	1	1	0	0
2	1	0	1	0	0	1
3	1	1	0	0	1	0
4	1	0	0	0	0	0
5	0	0	1	0	0	0
6	0	1	0	0	0	0

(10x**1**, 1x**2**)

	1	2	3	4	5	6
1	0	1	1	1	0	0
2	1	2	0	0	0	0
3	1	0	0	0	1	1
4	1	0	0	0	0	0
5	0	0	1	0	0	0
6	0	0	1	0	0	0

(8x**1**, 2x**2**) **a**

	1	2	3	4	5	6
1	0	1	1	1	0	0
2	1	2	0	0	0	0
3	1	0	0	0	0	0
4	1	0	0	0	0	0
5	0	0	0	0	2	1
6	0	0	0	0	1	0

(8x**1**, 2x**2**) **b**

	1	2	3	4	5	6
1	0	1	1	1	0	0
2	1	0	0	0	0	0
3	1	0	0	0	0	0
4	1	0	0	0	0	0
5	0	0	0	0	2	1
6	0	0	0	0	1	2

(8x**1**, 2x**2**) **c**

	1	2	3	4	5	6
1	0	1	1	1	0	0
2	1	2	0	0	0	0
3	1	0	2	0	0	0
4	1	0	0	0	0	0
5	0	0	0	0	0	1
6	0	0	0	0	1	0

(8x**1**, 2x**2**^{1}) **a**

	1	2	3	4	5	6
1	0	0	1	0	0	0
2	0	0	1	0	0	0
3	1	1	0	1	0	0
4	0	0	1	0	2^1	0
5	0	0	0	2^1	0	1
6	0	0	0	0	1	0

(8x**1**, 2x**2**^{1}) **b**

	1	2	3	4	5	6
1	0	2^1	1	0	0	0
2	2^1	0	1	0	0	0
3	1	1	0	1	0	0
4	0	0	1	0	0	0
5	0	0	0	0	0	1
6	0	0	0	0	1	0

(8x**1**, 2x**2**^{2}) **a**

	1	2	3	4	5	6
1	0	0	1	0	0	0
2	0	0	1	0	0	0
3	1	1	0	1	0	0
4	0	0	1	0	2^2	0
5	0	0	0	2^2	0	1
6	0	0	0	0	1	0

(8x**1**, 2x**2**^{2}) **b**

	1	2	3	4	5	6
1	0	2^2	1	0	0	0
2	2^2	0	1	0	0	0
3	1	1	0	1	0	0
4	0	0	1	0	0	0
5	0	0	0	0	0	1
6	0	0	0	0	1	0

(6x**1**, 3x**2**) **a**

	1	2	3	4	5	6
1	0	1	0	0	0	0
2	1	2	0	0	0	0
3	0	0	2	1	0	0
4	0	0	1	0	0	0
5	0	0	0	0	0	1
6	0	0	0	0	1	2

(6x**1**, 3x**2**) **b**

	1	2	3	4	5	6
1	0	1	0	0	0	0
2	1	2	0	0	0	0
3	0	0	2	1	0	0
4	0	0	1	2	0	0
5	0	0	0	0	0	1
6	0	0	0	0	1	0

(6x**1**, 1x**2**, 2x**2**^{1}) **a**

	1	2	3	4	5	6
1	0	1	0	0	0	0
2	1	0	2^1	0	0	0
3	0	2^1	0	1	0	0
4	0	0	1	0	0	0
5	0	0	0	0	2	1
6	0	0	0	0	1	0

(6x**1**, 1x**2**, 2x**2**^{1}) **b**

	1	2	3	4	5	6
1	2	1	0	0	0	0
2	1	0	2^1	0	0	0
3	0	2^1	0	1	0	0
4	0	0	1	0	0	0
5	0	0	0	0	0	1
6	0	0	0	0	1	0

(6x**1**, 1x**2**, 2x**2**^{2}) **a**

	1	2	3	4	5	6
1	0	1	0	0	0	0
2	1	0	2^2	0	0	0
3	0	2^2	0	1	0	0
4	0	0	1	0	0	0
5	0	0	0	0	2	1
6	0	0	0	0	1	0

(6x**1**, 1x**2**, 2x**2**^{2}) **b**

	1	2	3	4	5	6
1	2	1	0	0	0	0
2	1	0	2^2	0	0	0
3	0	2^2	0	1	0	0
4	0	0	1	0	0	0
5	0	0	0	0	0	1
6	0	0	0	0	1	0

$^1V_3{}^3V_5$

NG

$^1V_4{}^3V_2$

(10x**1**)

	1	2	3	4	5	6
1	0	0	1	0	0	0
2	0	0	1	0	0	0
3	1	1	0	1	0	0
4	0	0	1	0	1	1
5	0	0	0	1	0	0
6	0	0	0	1	0	0

(8x**1**, 1x**2**) **a**

	1	2	3	4	5	6
1	0	0	1	0	0	0
2	0	0	1	0	0	0
3	1	1	0	1	0	0
4	0	0	1	0	0	0
5	0	0	0	0	0	1
6	0	0	0	0	1	2

(8x**1**, 1x**2**) **b**

	1	2	3	4	5	6
1	0	0	1	0	0	0
2	0	0	1	0	0	0
3	1	1	0	1	0	0
4	0	0	1	2	0	0
5	0	0	0	0	0	1
6	0	0	0	0	1	0

(6x**1**, 2x**2**) **a**

	1	2	3	4	5	6
1	2	1	0	0	0	0
2	1	2	0	0	0	0
3	0	0	0	1	0	0
4	0	0	1	0	0	0
5	0	0	0	0	0	1
6	0	0	0	0	1	0

(6x**1**, 2x**2**) **b**

	1	2	3	4	5	6
1	0	1	0	0	0	0
2	1	2	0	0	0	0
3	0	0	2	1	0	0
4	0	0	1	0	0	0
5	0	0	0	0	0	1
6	0	0	0	0	1	0

(6x**1**, 2x**2**^{1})

	1	2	3	4	5	6
1	0	1	0	0	0	0
2	1	0	2^1	0	0	0
3	0	2^1	0	1	0	0
4	0	0	1	0	0	0
5	0	0	0	0	0	1
6	0	0	0	0	1	0

(6x**1**, 2x**2**^{2})

	1	2	3	4	5	6
1	0	1	0	0	0	0
2	1	0	2^2	0	0	0
3	0	2^2	0	1	0	0
4	0	0	1	0	0	0
5	0	0	0	0	0	1
6	0	0	0	0	1	0

$^1V_4{}^3V_4$

NG

$^1V_5{}^3V_1$

(8x**1**)

	1	2	3	4	5	6
1	0	0	1	0	0	0
2	0	0	1	0	0	0
3	1	1	0	1	0	0
4	0	0	1	0	0	0
5	0	0	0	0	0	1
6	0	0	0	0	1	0

(6x**1**, 1x**2**)

	1	2	3	4	5	6
1	0	0	0	1	0	0
2	0	0	1	0	0	0
3	0	1	0	0	0	0
4	1	0	0	0	0	0
5	0	0	0	0	0	1
6	0	0	0	0	1	2

$^1V_5{}^3V_3$

NG

$^1V_6{}^3V_2$

NG

$^1V_7{}^3V_1$

NG

$^1V_r{}^4V_r$

$^1V_2{}^4V_1$

(4x**1**, 1x**2**)

	1	2	3
1	2	1	1
2	1	0	0
3	1	0	0

$^1V_2{}^4V_2$

(6x**1**, 2x**2**)

	1	2	3	4
1	2	1	1	0
2	1	2	0	1
3	1	0	0	0
4	0	1	0	0

(4x**1**, 1x**2**, 2x$\mathbf{2}^1$)

	1	2	3	4
1	2	2^1	0	0
2	2^1	0	1	1
3	0	1	0	0
4	0	1	0	0

(4x**1**, 1x**2**, 2x$\mathbf{2}^2$)

	1	2	3	4
1	2	2^2	0	0
2	2^2	0	1	1
3	0	1	0	0
4	0	1	0	0

(2x**1**, 2x**2**, 2x$\mathbf{2}^1$)

	1	2	3	4
1	2	2^1	0	0
2	2^1	2	0	0
3	0	0	0	1
4	0	0	1	0

(2x**1**, 2x**2**, 2x$\mathbf{2}^2$)

	1	2	3	4
1	2	2^2	0	0
2	2^2	2	0	0
3	0	0	0	1
4	0	0	1	0

$^1V_2{}^4V_3$

(10x**1**, 2x**2**)

	1	2	3	4	5
1	2	1	1	0	0
2	1	2	1	0	0
3	1	1	0	1	1
4	0	0	1	0	0
5	0	0	1	0	0

(8x**1**, 3x**2**) **a**

	1	2	3	4	5
1	2	1	1	0	0
2	1	2	1	0	0
3	1	1	2	0	0
4	0	0	0	0	1
5	0	0	0	1	0

(8x**1**, 3x**2**) **b**

	1	2	3	4	5
1	2	1	1	0	0
2	1	2	0	1	0
3	1	0	2	0	1
4	0	1	0	0	0
5	0	0	1	0	0

(8x**1**, 1x**2**, 2x$\mathbf{2}^1$)

	1	2	3	4	5
1	0	2^1	1	1	0
2	2^1	0	1	0	1
3	1	1	2	0	0
4	1	0	0	0	0
5	0	1	0	0	0

(8x**1**, 1x**2**, 2x$\mathbf{2}^2$)

	1	2	3	4	5
1	0	2^2	1	1	0
2	2^2	0	1	0	1
3	1	1	2	0	0
4	1	0	0	0	0
5	0	1	0	0	0

(6x**1**, 2x**2**, 2x$\mathbf{2}^1$)

	1	2	3	4	5
1	2	2^1	0	0	0
2	2^1	0	1	1	0
3	0	1	2	0	1
4	0	1	0	0	0
5	0	0	1	0	0

(6x**1**, 2x**2**, 2x$\mathbf{2}^2$)

	1	2	3	4	5
1	2	2^2	0	0	0
2	2^2	0	1	1	0
3	0	1	2	0	1
4	0	1	0	0	0
5	0	0	1	0	0

(6x**1**, 4x$\mathbf{2}^1$)

	1	2	3	4	5
1	0	1	2^1	1	0
2	1	0	2^1	0	1
3	2^1	2^1	0	0	0
4	1	0	0	0	0
5	0	1	0	0	0

(6x**1**, 2x$\mathbf{2}^1$, 2x$\mathbf{2}^2$)

	1	2	3	4	5
1	0	1	2^1	1	0
2	1	0	2^2	0	1
3	2^1	2^2	0	0	0
4	1	0	0	0	0
5	0	1	0	0	0

(6x**1**, 4x$\mathbf{2}^2$)

	1	2	3	4	5
1	0	1	2^2	1	0
2	1	0	2^2	0	1
3	2^2	2^2	0	0	0
4	1	0	0	0	0
5	0	1	0	0	0

(4x**1**, 3x**2**, 2x$\mathbf{2}^1$)

	1	2	3	4	5
1	2	2^1	0	0	0
2	2^1	2	0	0	0
3	0	0	2	1	1
4	0	0	1	0	0
5	0	0	1	0	0

(4x**1**, 3x**2**, 2x$\mathbf{2}^2$)

	1	2	3	4	5
1	2	2^2	0	0	0
2	2^2	2	0	0	0
3	0	0	2	1	1
4	0	0	1	0	0
5	0	0	1	0	0

(4x**1**, 1x**2**, 4x$\mathbf{2}^1$)

	1	2	3	4	5
1	2	2^1	0	0	0
2	2^1	0	2^1	0	0
3	0	2^1	0	1	1
4	0	0	1	0	0
5	0	0	1	0	0

(4x**1**, 1x**2**, 2x$\mathbf{2}^1$, 2x$\mathbf{2}^2$)

	1	2	3	4	5
1	2	2^2	0	0	0
2	2^2	0	2^1	0	0
3	0	2^1	0	1	1
4	0	0	1	0	0
5	0	0	1	0	0

(4x**1**, 1x**2**, 4x$\mathbf{2}^2$)

	1	2	3	4	5
1	2	2^2	0	0	0
2	2^2	0	2^2	0	0
3	0	2^2	0	1	1
4	0	0	1	0	0
5	0	0	1	0	0

(2x**1**, 2x**2**, 4x$\mathbf{2}^1$)

	1	2	3	4	5
1	2	2^1	0	0	0
2	2^1	0	2^1	0	0
3	0	2^1	2	0	0
4	0	0	0	0	1
5	0	0	0	1	0

(2x**1**, 2x**2**, 2x$\mathbf{2}^1$, 2x$\mathbf{2}^2$)

	1	2	3	4	5
1	2	2^2	0	0	0
2	2^2	0	2^1	0	0
3	0	2^1	2	0	0
4	0	0	0	0	1
5	0	0	0	1	0

(2x**1**, 2x**2**, 4x$\mathbf{2}^2$)

	1	2	3	4	5
1	2	2^2	0	0	0
2	2^2	0	2^2	0	0
3	0	2^2	2	0	0
4	0	0	0	0	1
5	0	0	0	1	0

(2x**1**, 6x$\mathbf{2}^1$)

	1	2	3	4	5
1	0	2^1	2^1	0	0
2	2^1	0	2^1	0	0
3	2^1	2^1	0	0	0
4	0	0	0	0	1
5	0	0	0	1	0

(2x**1**, 4x$\mathbf{2}^1$, 2x$\mathbf{2}^2$)

	1	2	3	4	5
1	0	2^1	2^2	0	0
2	2^1	0	2^1	0	0
3	2^2	2^1	0	0	0
4	0	0	0	0	1
5	0	0	0	1	0

(2x**1**, 2x$\mathbf{2}^1$, 4x$\mathbf{2}^2$)

	1	2	3	4	5
1	0	2^2	2^2	0	0
2	2^2	0	2^1	0	0
3	2^2	2^1	0	0	0
4	0	0	0	0	1
5	0	0	0	1	0

(2x**1**, 6x$\mathbf{2}^2$)

	1	2	3	4	5
1	0	2^2	2^2	0	0
2	2^2	0	2^2	0	0
3	2^2	2^2	0	0	0
4	0	0	0	0	1
5	0	0	0	1	0

$^1V_2{}^4V_4$

(14x**1**, 2x**2**)

	1	2	3	4	5	6
1	2	0	1	1	0	0
2	0	2	1	1	0	0
3	1	1	0	1	1	0
4	1	1	1	0	0	1
5	0	0	1	0	0	0
6	0	0	0	1	0	0

(14x**1**, 2x$\mathbf{2}^1$)

	1	2	3	4	5	6
1	0	2^1	1	1	0	0
2	2^1	0	1	1	0	0
3	1	1	0	1	1	0
4	1	1	1	0	0	1
5	0	0	1	0	0	0
6	0	0	0	1	0	0

(14x**1**, 2x$\mathbf{2}^2$)

	1	2	3	4	5	6
1	0	2^2	1	1	0	0
2	2^2	0	1	1	0	0
3	1	1	0	1	1	0
4	1	1	1	0	0	1
5	0	0	1	0	0	0
6	0	0	0	1	0	0

(12x**1**, 3x**2**) **a**

	1	2	3	4	5	6
1	2	1	1	0	0	0
2	1	2	0	1	0	0
3	1	0	2	1	0	0
4	0	1	1	0	1	1
5	0	0	0	1	0	0
6	0	0	0	1	0	0

(12x**1**, 3x**2**) **b**

	1	2	3	4	5	6
1	2	0	1	1	0	0
2	0	2	0	1	1	0
3	1	0	2	1	0	0
4	1	1	1	0	0	1
5	0	1	0	0	0	0
6	0	0	0	1	0	0

(12x**1**, 1x**2**, 2x**2**^{1}) **a**

	1	2	3	4	5	6
1	2	1	1	0	0	0
2	1	0	2^1	1	0	0
3	1	2^1	0	1	0	0
4	0	1	1	0	1	1
5	0	0	0	1	0	0
6	0	0	0	1	0	0

(12x**1**, 1x**2**, 2x**2**^{1}) **b**

	1	2	3	4	5	6
1	2	1	1	0	0	0
2	1	0	1	2^1	0	0
3	1	1	0	1	1	0
4	0	2^1	1	0	0	1
5	0	0	1	0	0	0
6	0	0	0	1	0	0

(12x**1**, 1x**2**, 2x**2**^{2}) **a**

	1	2	3	4	5	6
1	2	1	1	0	0	0
2	1	0	2^2	1	0	0
3	1	2^2	0	1	0	0
4	0	1	1	0	1	1
5	0	0	0	1	0	0
6	0	0	0	1	0	0

(12x**1**, 1x**2**, 2x**2**^{2}) **b**

	1	2	3	4	5	6
1	2	1	1	0	0	0
2	1	0	1	2^2	0	0
3	1	1	0	1	1	0
4	0	2^2	1	0	0	1
5	0	0	1	0	0	0
6	0	0	0	1	0	0

(10x**1**, 4x**2**) **a**

	1	2	3	4	5	6
1	2	1	1	0	0	0
2	1	2	0	1	0	0
3	1	0	2	1	0	0
4	0	1	1	2	0	0
5	0	0	0	0	0	1
6	0	0	0	0	1	0

(10x**1**, 4x**2**) **b**

	1	2	3	4	5	6
1	2	1	1	0	0	0
2	1	2	1	0	0	0
3	1	1	2	0	0	0
4	0	0	0	0	1	0
5	0	0	0	1	2	1
6	0	0	0	0	1	0

(10x**1**, 4x**2**) **c**

	1	2	3	4	5	6
1	0	1	0	0	0	0
2	1	2	1	0	0	0
3	0	1	2	1	0	0
4	0	0	1	2	1	0
5	0	0	0	1	2	1
6	0	0	0	0	1	0

(10x**1**, 2x**2**, 2x**2**^{1}) **a**

	1	2	3	4	5	6
1	2	1	1	0	0	0
2	1	2	0	1	0	0
3	1	0	0	2^1	1	0
4	0	1	2^1	0	0	1
5	0	0	1	0	0	0
6	0	0	0	1	0	0

(10x**1**, 2x**2**, 2x**2**^{1}) **b**

	1	2	3	4	5	6
1	2	1	1	0	0	0
2	1	0	2^1	1	0	0
3	1	2^1	0	1	0	0
4	0	1	1	2	0	0
5	0	0	0	0	0	1
6	0	0	0	0	1	0

(10x**1**, 2x**2**, 2x**2**^{1}) **c**

	1	2	3	4	5	6
1	2	1	1	0	0	0
2	1	0	1	2^1	0	0
3	1	1	0	0	1	1
4	0	2^1	0	2	0	0
5	0	0	1	0	0	0
6	0	0	1	0	0	0

(10x**1**, 2x**2**, 2x**2**^{1}) **d**

	1	2	3	4	5	6
1	2	1	1	0	0	0
2	1	0	1	2^1	0	0
3	1	1	2	0	0	0
4	0	2^1	0	0	1	1
5	0	0	0	1	0	0
6	0	0	0	1	0	0

(10x**1**, 2x**2**, 2x**2**^{1}) **e**

	1	2	3	4	5	6
1	2	1	1	0	0	0
2	1	0	2^1	1	0	0
3	1	2^1	0	0	0	1
4	0	1	0	2	1	0
5	0	0	0	1	0	0
6	0	0	1	0	0	0

(10x**1**, 2x**2**, 2x**2**^{2}) **a**

	1	2	3	4	5	6
1	2	1	1	0	0	0
2	1	2	0	1	0	0
3	1	0	0	2^2	1	0
4	0	1	2^2	0	0	1
5	0	0	1	0	0	0
6	0	0	0	1	0	0

(10x**1**, 2x**2**, 2x**2**^{2}) **b**

	1	2	3	4	5	6
1	2	1	1	0	0	0
2	1	0	2^2	1	0	0
3	1	2^2	0	1	0	0
4	0	1	1	2	0	0
5	0	0	0	0	0	1
6	0	0	0	0	1	0

(10x**1**, 2x**2**, 2x**2**^{2}) **c**

	1	2	3	4	5	6
1	2	1	1	0	0	0
2	1	0	1	2^2	0	0
3	1	1	0	0	1	1
4	0	2^2	0	2	0	0
5	0	0	1	0	0	0
6	0	0	1	0	0	0

(10x**1**, 2x**2**, 2x**2**^{2}) **d**

	1	2	3	4	5	6
1	2	1	1	0	0	0
2	1	0	1	2^2	0	0
3	1	1	2	0	0	0
4	0	2^2	0	0	1	1
5	0	0	0	1	0	0
6	0	0	0	1	0	0

(10x**1**, 2x**2**, 2x**2**^{2}) **e**

	1	2	3	4	5	6
1	2	1	1	0	0	0
2	1	0	2^2	1	0	0
3	1	2^2	0	0	0	1
4	0	1	0	2	1	0
5	0	0	0	1	0	0
6	0	0	1	0	0	0

(10x**1**, 4x**2**^{1}) **a**

	1	2	3	4	5	6
1	0	2^1	1	1	0	0
2	2^1	0	1	1	0	0
3	1	1	0	2^1	0	0
4	1	1	2^1	0	0	0
5	0	0	0	0	0	1
6	0	0	0	0	1	0

(10x**1**, 4x**2**^{1}) **b**

	1	2	3	4	5	6
1	0	2^1	1	0	1	0
2	2^1	0	1	1	0	0
3	1	1	0	2^1	0	0
4	0	1	2^1	0	0	1
5	1	0	0	0	0	0
6	0	0	0	1	0	0

(10x**1**, 4x**2**^{1}) **c**

	1	2	3	4	5	6
1	0	1	1	0	1	1
2	1	0	1	2^1	0	0
3	1	1	0	2^1	0	0
4	0	2^1	2^1	0	0	0
5	1	0	0	0	0	0
6	1	0	0	0	0	0

(10x**1**, 2x**2**^{1}, 2x**2**^{2}) **a**

	1	2	3	4	5	6
1	0	2^1	1	1	0	0
2	2^1	0	1	1	0	0
3	1	1	0	2^2	0	0
4	1	1	2^2	0	0	0
5	0	0	0	0	0	1
6	0	0	0	0	1	0

(10x**1**, 2x**2**^{1}, 2x**2**^{2}) **b**

	1	2	3	4	5	6
1	0	2^1	1	0	1	0
2	2^1	0	1	1	0	0
3	1	1	0	2^2	0	0
4	0	1	2^2	0	0	1
5	1	0	0	0	0	0
6	0	0	0	1	0	0

(10x**1**, 2x**2**^{1}, 2x**2**^{2}) **c**

	1	2	3	4	5	6
1	0	1	1	0	1	1
2	1	0	1	2^1	0	0
3	1	1	0	2^2	0	0
4	0	2^1	2^2	0	0	0
5	1	0	0	0	0	0
6	1	0	0	0	0	0

(10x**1**, 4x**2**^{2}) **a**

	1	2	3	4	5	6
1	0	2^2	1	1	0	0
2	2^2	0	1	1	0	0
3	1	1	0	2^2	0	0
4	1	1	2^2	0	0	0
5	0	0	0	0	0	1
6	0	0	0	0	1	0

(10x**1**, 4x**2**^{2}) **b**

	1	2	3	4	5	6
1	0	2^2	1	0	1	0
2	2^2	0	1	1	0	0
3	1	1	0	2^2	0	0
4	0	1	2^2	0	0	1
5	1	0	0	0	0	0
6	0	0	0	1	0	0

(10x**1**, 4x**2**^{2}) **c**

	1	2	3	4	5	6
1	0	1	1	0	1	1
2	1	0	1	2^2	0	0
3	1	1	0	2^2	0	0
4	0	2^2	2^2	0	0	0
5	1	0	0	0	0	0
6	1	0	0	0	0	0

(8x**1**, 3x**2**, 2x**2**^{1}) **a**

	1	2	3	4	5	6
1	2	1	1	0	0	0
2	1	0	1	2^1	0	0
3	1	1	2	0	0	0
4	0	2^1	0	2	0	0
5	0	0	0	0	0	1
6	0	0	0	0	1	0

(8x**1**, 3x**2**, 2x**2**^{1}) **b**

	1	2	3	4	5	6
1	0	1	0	0	0	0
2	1	2	1	0	0	0
3	0	1	0	1	0	2^1
4	0	0	1	2	1	0
5	0	0	0	1	0	0
6	0	0	2^1	0	0	2

(8x**1**, 3x**2**, 2x**2**^{1}) **c**

	1	2	3	4	5	6
1	0	1	0	0	0	0
2	1	2	1	0	0	0
3	0	1	2	1	0	0
4	0	0	1	0	1	2^1
5	0	0	0	1	0	0
6	0	0	0	2^1	0	2

(8x**1**, 3x**2**, 2x**2**^{2}) **a**

	1	2	3	4	5	6
1	2	1	1	0	0	0
2	1	0	1	2^2	0	0
3	1	1	2	0	0	0
4	0	2^2	0	2	0	0
5	0	0	0	0	0	1
6	0	0	0	0	1	0

(8x**1**, 3x**2**, 2x**2**^{2}) **b**

	1	2	3	4	5	6
1	0	1	0	0	0	0
2	1	2	1	0	0	0
3	0	1	0	1	0	2^2
4	0	0	1	2	1	0
5	0	0	0	1	0	0
6	0	0	2^2	0	0	2

(8x**1**, 3x**2**, 2x**2**^{2}) **c**

	1	2	3	4	5	6
1	0	1	0	0	0	0
2	1	2	1	0	0	0
3	0	1	2	1	0	0
4	0	0	1	0	1	2^2
5	0	0	0	1	0	0
6	0	0	0	2^2	0	2

(8x**1**, 1x**2**, 4x**2**^{1}) **a**

	1	2	3	4	5	6
1	2	1	1	0	0	0
2	1	0	1	2^1	0	0
3	1	1	0	2^1	0	0
4	0	2^1	2^1	0	0	0
5	0	0	0	0	0	1
6	0	0	0	0	1	0

(8x**1**, 1x**2**, 4x**2**^{1}) **b**

	1	2	3	4	5	6
1	2	1	1	0	0	0
2	1	0	0	2^1	0	1
3	1	0	0	2^1	1	0
4	0	2^1	2^1	0	0	0
5	0	0	1	0	0	0
6	0	1	0	0	0	0

(8x**1**, 1x**2**, 4x**2**^{1}) **c**

	1	2	3	4	5	6
1	0	1	1	0	2^1	0
2	1	0	2^1	1	0	0
3	1	2^1	0	0	0	1
4	0	1	0	0	0	0
5	2^1	0	0	0	2	0
6	0	0	1	0	0	0

(8x**1**, 1x**2**, 4x**2**^{1}) **d**

	1	2	3	4	5	6
1	0	2^1	1	0	1	0
2	2^1	0	2^1	0	0	0
3	1	2^1	0	0	0	1
4	0	0	0	0	1	0
5	1	0	0	1	2	0
6	0	0	1	0	0	0

(8x**1**, 1x**2**, 2x**2**^{1}, 2x**2**^{2}) **a**

	1	2	3	4	5	6
1	2	1	1	0	0	0
2	1	0	1	2^1	0	0
3	1	1	0	2^2	0	0
4	0	2^1	2^2	0	0	0
5	0	0	0	0	0	1
6	0	0	0	0	1	0

(8x**1**, 1x**2**, 2x**2**^{1}, 2x**2**^{2}) **b**

	1	2	3	4	5	6
1	2	1	1	0	0	0
2	1	0	0	2^1	0	1
3	1	0	0	2^2	1	0
4	0	2^1	2^2	0	0	0
5	0	0	1	0	0	0
6	0	1	0	0	0	0

(8x**1**, 1x**2**, 2x**2**^{1}, 2x**2**^{2}) **c**

	1	2	3	4	5	6
1	0	1	1	0	2^1	0
2	1	0	2^2	1	0	0
3	1	2^2	0	0	0	1
4	0	1	0	0	0	0
5	2^1	0	0	0	2	0
6	0	0	1	0	0	0

(8x**1**, 1x**2**, 2x**2**^{1}, 2x**2**^{2}) **d**

	1	2	3	4	5	6
1	0	1	1	0	2^2	0
2	1	0	2^1	1	0	0
3	1	2^1	0	0	0	1
4	0	1	0	0	0	0
5	2^2	0	0	0	2	0
6	0	0	1	0	0	0

(8x**1**, 1x**2**, 2x**2**^{1}, 2x**2**^{2}) **e**

	1	2	3	4	5	6
1	0	2^2	1	0	1	0
2	2^2	0	2^1	0	0	0
3	1	2^1	0	0	0	1
4	0	0	0	0	1	0
5	1	0	0	1	2	0
6	0	0	1	0	0	0

(8x**1**, 1x**2**, 2x**2**^{1}, 2x**2**^{2}) **f**

	1	2	3	4	5	6
1	0	2^1	1	0	1	0
2	2^1	0	2^2	0	0	0
3	1	2^2	0	0	0	1
4	0	0	0	0	1	0
5	1	0	0	1	2	0
6	0	0	1	0	0	0

(8x**1**, 1x**2**, 4x**2**^{2}) **a**

	1	2	3	4	5	6
1	2	1	1	0	0	0
2	1	0	1	2^2	0	0
3	1	1	0	2^2	0	0
4	0	2^2	2^2	0	0	0
5	0	0	0	0	0	1
6	0	0	0	0	1	0

(8x**1**, 1x**2**, 4x**2**^{2}) **b**

	1	2	3	4	5	6
1	2	1	1	0	0	0
2	1	0	0	2^2	0	1
3	1	0	0	2^2	1	0
4	0	2^2	2^2	0	0	0
5	0	0	1	0	0	0
6	0	1	0	0	0	0

(8x**1**, 1x**2**, 4x**2**^{2}) **c**

	1	2	3	4	5	6
1	0	1	1	0	2^2	0
2	1	0	2^2	1	0	0
3	1	2^2	0	0	0	1
4	0	1	0	0	0	0
5	2^2	0	0	0	2	0
6	0	0	1	0	0	0

(8x**1**, 1x**2**, 4x**2**^{2}) **d**

	1	2	3	4	5	6
1	0	2^2	1	0	1	0
2	2^2	0	2^2	0	0	0
3	1	2^2	0	0	0	1
4	0	0	0	0	1	0
5	1	0	0	1	2	0
6	0	0	1	0	0	0

(6x**1**, 4x**2**, 2x**2**^{1})

	1	2	3	4	5	6
1	0	1	0	0	0	0
2	1	2	1	0	0	0
3	0	1	2	1	0	0
4	0	0	1	0	0	0
5	0	0	0	0	2	2^1
6	0	0	0	0	2^1	2

(6x**1**, 4x**2**, 2x**2**^{2})

	1	2	3	4	5	6
1	0	1	0	0	0	0
2	1	2	1	0	0	0
3	0	1	2	1	0	0
4	0	0	1	0	0	0
5	0	0	0	0	2	2^2
6	0	0	0	0	2^2	2

(6x**1**, 2x**2**, 4x**2**^{1}) **a**

	1	2	3	4	5	6
1	2	2^1	0	0	0	0
2	2^1	0	2^1	0	0	0
3	0	2^1	0	1	0	1
4	0	0	1	2	1	0
5	0	0	0	1	0	0
6	0	0	1	0	0	0

(6x**1**, 2x**2**, 4x**2**^{1}) **b**

	1	2	3	4	5	6
1	0	1	0	0	0	0
2	1	0	1	0	2^1	0
3	0	1	0	1	0	2^1
4	0	0	1	0	0	0
5	0	2^1	0	0	2	0
6	0	0	2^1	0	0	2

(6x**1**, 2x**2**, 2x**2**^{1}, 2x**2**^{2}) **a**

	1	2	3	4	5	6
1	2	2^1	0	0	0	0
2	2^1	0	2^2	0	0	0
3	0	2^2	0	1	0	1
4	0	0	1	2	1	0
5	0	0	0	1	0	0
6	0	0	1	0	0	0

(6x**1**, 2x**2**, 2x**2**^{1}, 2x**2**^{2}) **b**

	1	2	3	4	5	6
1	2	2^2	0	0	0	0
2	2^2	0	2^1	0	0	0
3	0	2^1	0	1	0	1
4	0	0	1	2	1	0
5	0	0	0	1	0	0
6	0	0	1	0	0	0

(6x**1**, 2x**2**, 2x**2**^{1}, 2x**2**^{2}) **c**

	1	2	3	4	5	6
1	0	1	0	0	0	0
2	1	0	1	0	2^2	0
3	0	1	0	1	0	2^1
4	0	0	1	0	0	0
5	0	2^2	0	0	2	0
6	0	0	2^1	0	0	2

(6x**1**, 2x**2**, 4x**2**^{2}) **a**

	1	2	3	4	5	6
1	2	2^2	0	0	0	0
2	2^2	0	2^2	0	0	0
3	0	2^2	0	1	0	1
4	0	0	1	2	1	0
5	0	0	0	1	0	0
6	0	0	1	0	0	0

(6x**1**, 2x**2**, 4x**2**^{2}) **b**

	1	2	3	4	5	6
1	0	1		0	0	0
2	1	0	1	0	2^2	0
3	0	1	0	1	0	2^2
4	0	0	1	0	0	0
5	0	2^2	0	0	2	0
6	0	0	2^2	0	0	2

(6x**1**, 6x**2**^{1})

	1	2	3	4	5	6
1	0	2^1	0	1	0	1
2	2^1	0	2^1	0	0	0
3	0	2^1	0	2^1	0	0
4	1	0	2^1	0	1	0
5	0	0	0	1	0	0
6	1	0	0	0	0	0

(6x**1**, 4x**2**^{1}, 2x**2**^{2}) **a**

	1	2	3	4	5	6
1	0	2^1	0	1	0	1
2	2^1	0	2^1	0	0	0
3	0	2^1	0	2^2	0	0
4	1	0	2^2	0	1	0
5	0	0	0	1	0	0
6	1	0	0	0	0	0

(6x**1**, 4x**2**^{1}, 2x**2**^{2}) **b**

	1	2	3	4	5	6
1	0	2^1	0	1	0	1
2	2^1	0	2^2	0	0	0
3	0	2^2	0	2^1	0	0
4	1	0	2^1	0	1	0
5	0	0	0	1	0	0
6	1	0	0	0	0	0

(6x**1**, 2x**2**^{1}, 4x**2**^{2}) **a**

	1	2	3	4	5	6
1	0	2^2	0	1	0	1
2	2^2	0	2^1	0	0	0
3	0	2^1	0	2^2	0	0
4	1	0	2^2	0	1	0
5	0	0	0	1	0	0
6	1	0	0	0	0	0

(6x**1**, 2x**2**^{1}, 4x**2**^{2}) **b**

	1	2	3	4	5	6
1	0	2^2	0	1	0	1
2	2^2	0	2^2	0	0	0
3	0	2^2	0	2^1	0	0
4	1	0	2^1	0	1	0
5	0	0	0	1	0	0
6	1	0	0	0	0	0

(6x**1**, 6x**2**^{2})

	1	2	3	4	5	6
1	0	2^2	0	1	0	1
2	2^2	0	2^2	0	0	0
3	0	2^2	0	2^2	0	0
4	1	0	2^2	0	1	0
5	0	0	0	1	0	0
6	1	0	0	0	0	0

(4x**1**, 3x**2**, 4x**2**^{1}) **a**

	1	2	3	4	5	6
1	0	1	0	0	0	0
2	1	2	1	0	0	0
3	0	1	0	0	0	0
4	0	0	0	2	2^1	0
5	0	0	0	2^1	0	2^1
6	0	0	0	0	2^1	2

(4x**1**, 3x**2**, 4x**2**^{1}) **b**

	1	2	3	4	5	6
1	0	0	1	0	0	0
2	0	2	2^1	0	0	0
3	1	2^1	0	1	0	0
4	0	0	1	0	0	0
5	0	0	0	0	2	2^1
6	0	0	0	0	2^1	2

(4x**1**, 3x**2**, 2x**2**^{1}, 2x**2**^{2}) **a**

	1	2	3	4	5	6
1	0	1	0	0	0	0
2	1	2	1	0	0	0
3	0	1	0	0	0	0
4	0	0	0	2	2^1	0
5	0	0	0	2^1	0	2^2
6	0	0	0	0	2^2	2

(4x**1**, 3x**2**, 2x**2**^{1}, 2x**2**^{2}) **b**

	1	2	3	4	5	6
1	0	0	1	0	0	0
2	0	2	2^2	0	0	0
3	1	2^2	0	1	0	0
4	0	0	1	0	0	0
5	0	0	0	0	2	2^1
6	0	0	0	0	2^1	2

(4x**1**, 3x**2**, 2x**2**^{1}, 2x**2**^{2}) **c**

	1	2	3	4	5	6
1	0	0	1	0	0	0
2	0	2	2^1	0	0	0
3	1	2^1	0	1	0	0
4	0	0	1	0	0	0
5	0	0	0	0	2	2^2
6	0	0	0	0	2^2	2

(4x**1**, 3x**2**, 4x**2**^{2}) **a**

	1	2	3	4	5	6
1	0	1	0	0	0	0
2	1	2	1	0	0	0
3	0	1	0	0	0	0
4	0	0	0	2	2^2	0
5	0	0	0	2^2	0	2^2
6	0	0	0	0	2^2	2

(4x**1**, 3x**2**, 4x**2**^{2}) **b**

	1	2	3	4	5	6
1	0	0	1	0	0	0
2	0	2	2^2	0	0	0
3	1	2^2	0	1	0	0
4	0	0	1	0	0	0
5	0	0	0	0	2	2^2
6	0	0	0	0	2^2	2

(4x**1**, 1x**2**, 6x**2**^{1}) **a**

	1	2	3	4	5	6
1	2	2^1	1	0	0	0
2	2^1	0	2^1	0	0	0
3	0	2^1	0	2^1	0	0
4	0	0	2^1	0	1	1
5	0	0	0	1	0	0
6	0	0	0	1	0	0

(4x**1**, 1x**2**, 6x**2**^{1}) **b**

	1	2	3	4	5	6
1	0	2^1	2^1	0	0	0
2	2^1	0	2^1	0	0	0
3	2^1	2^1	0	0	0	0
4	0	0	0	0	1	0
5	0	0	0	1	2	1
6	0	0	0	0	1	0

(4x**1**, 1x**2**, 4x**2**^{1}, 2x**2**^{2}) **a**

	1	2	3	4	5	6
1	2	2^1	1	0	0	0
2	2^1	0	2^2	0	0	0
3	0	2^2	0	2^1	0	0
4	0	0	2^1	0	1	1
5	0	0	0	1	0	0
6	0	0	0	1	0	0

(4x**1**, 1x**2**, 4x**2**^{1}, 2x**2**^{2}) **b**

	1	2	3	4	5	6
1	2	2^1	1	0	0	0
2	2^1	0	2^1	0	0	0
3	0	2^1	0	2^2	0	0
4	0	0	2^2	0	1	1
5	0	0	0	1	0	0
6	0	0	0	1	0	0

(4x**1**, 1x**2**, 4x**2**^{1}, 2x**2**^{2}) **c**

	1	2	3	4	5	6
1	2	2^2	1	0	0	0
2	2^2	0	2^1	0	0	0
3	0	2^1	0	2^1	0	0
4	0	0	2^1	0	1	1
5	0	0	0	1	0	0
6	0	0	0	1	0	0

(4x**1**, 1x**2**, 4x**2**^{1}, 2x**2**^{2}) **d**

	1	2	3	4	5	6
1	0	2^1	2^1	0	0	0
2	2^1	0	2^2	0	0	0
3	2^1	2^2	0	0	0	0
4	0	0	0	0	1	0
5	0	0	0	1	2	1
6	0	0	0	0	1	0

(4x**1**, 1x**2**, 2x**2**^{1}, 4x**2**^{2}) **a**

	1	2	3	4	5	6
1	2	2^1	1	0	0	0
2	2^1	0	2^2	0	0	0
3	0	2^2	0	2^2	0	0
4	0	0	2^2	0	1	1
5	0	0	0	1	0	0
6	0	0	0	1	0	0

(4x**1**, 1x**2**, 2x**2**^{1}, 4x**2**^{2}) **b**

	1	2	3	4	5	6
1	2	2^2	1	0	0	0
2	2^2	0	2^2	0	0	0
3	0	2^2	0	2^1	0	0
4	0	0	2^1	0	1	1
5	0	0	0	1	0	0
6	0	0	0	1	0	0

(4x**1**, 1x**2**, 2x**2**^{1}, 4x**2**^{2}) **c**

	1	2	3	4	5	6
1	2	2^2	1	0	0	0
2	2^2	0	2^1	0	0	0
3	0	2^1	0	2^2	0	0
4	0	0	2^2	0	1	1
5	0	0	0	1	0	0
6	0	0	0	1	0	0

(4x**1**, 1x**2**, 2x**2**^{1}, 4x**2**^{2}) **d**

	1	2	3	4	5	6
1	0	2^2	2^1	0	0	0
2	2^2	0	2^2	0	0	0
3	2^1	2^2	0	0	0	0
4	0	0	0	0	1	0
5	0	0	0	1	2	1
6	0	0	0	0	1	0

(4x**1**, 1x**2**, 6x**2**^{2}) **a**

	1	2	3	4	5	6
1	2	2^2	1	0	0	0
2	2^2	0	2^2	0	0	0
3	0	2^2	0	2^2	0	0
4	0	0	2^2	0	1	1
5	0	0	0	1	0	0
6	0	0	0	1	0	0

(4x**1**, 1x**2**, 6x**2**^{2}) **b**

	1	2	3	4	5	6
1	0	2^2	2^2	0	0	0
2	2^2	0	2^2	0	0	0
3	2^2	2^2	0	0	0	0
4	0	0	0	0	1	0
5	0	0	0	1	2	1
6	0	0	0	0	1	0

(2x**1**, 4x**2**, 4x**2**^{1})

	1	2	3	4	5	6
1	0	1	0	0	0	0
2	1	0	0	0	0	0
3	0	0	2	2^1	0	0
4	0	0	2^1	2	0	0
5	0	0	0	0	2	2^1
6	0	0	0	0	2^1	2

(2x**1**, 4x**2**, 2x**2**^{1}, 2x**2**^{2})

	1	2	3	4	5	6
1	0	1	0	0	0	0
2	1	0	0	0	0	0
3	0	0	2	2^2	0	0
4	0	0	2^2	2	0	0
5	0	0	0	0	2	2^1
6	0	0	0	0	2^1	2

(2x**1**, 4x**2**, 4x**2**^{2})

	1	2	3	4	5	6
1	0	1	0	0	0	0
2	1	0	0	0	0	0
3	0	0	2	2^2	0	0
4	0	0	2^2	2	0	0
5	0	0	0	0	2	2^2
6	0	0	0	0	2^2	2

(2x**1**, 2x**2**, 6x**2**^{1})

	1	2	3	4	5	6
1	0	1	0	0	0	0
2	1	0	0	0	0	0
3	0	0	2	2^1	0	0
4	0	0	2^1	0	0	2^1
5	0	0	0	0	2	2^1
6	0	0	0	2^1	2^1	0

(2x**1**, 2x**2**, 4x**2**^{1}, 2x**2**^{2}) **a**

	1	2	3	4	5	6
1	0	1	0	0	0	0
2	1	0	0	0	0	0
3	0	0	2	2^2	0	0
4	0	0	2^2	0	0	2^1
5	0	0	0	0	2	2^1
6	0	0	0	2^1	2^1	0

(2x**1**, 2x**2**, 4x**2**^{1}, 2x**2**^{2}) **b**

	1	2	3	4	5	6
1	0	1	0	0	0	0
2	1	0	0	0	0	0
3	0	0	2	2^1	0	0
4	0	0	2^1	0	0	2^2
5	0	0	0	0	2	2^1
6	0	0	0	2^2	2^1	0

(2x**1**, 2x**2**, 2x**2**^{1}, 4x**2**^{2}) **a**

	1	2	3	4	5	6
1	0	1	0	0	0	0
2	1	0	0	0	0	0
3	0	0	2	2^2	0	0
4	0	0	2^2	0	0	2^1
5	0	0	0	0	2	2^2
6	0	0	0	2^1	2^2	0

(2x**1**, 2x**2**, 2x**2**^{1}, 4x**2**^{2}) **b**

	1	2	3	4	5	6
1	0	1	0	0	0	0
2	1	0	0	0	0	0
3	0	0	2	2^2	0	0
4	0	0	2^2	0	0	2^2
5	0	0	0	0	2	2^1
6	0	0	0	2^2	2^1	0

(2x**1**, 2x**2**, 6x**2**^{2})

	1	2	3	4	5	6
1	0	1	0	0	0	0
2	1	0	0	0	0	0
3	0	0	2	2^2	0	0
4	0	0	2^2	0	0	2^2
5	0	0	0	0	2	2^2
6	0	0	0	2^2	2^2	0

(2x**1**, 8x**2**^{1})

	1	2	3	4	5	6
1	0	1	0	0	0	0
2	1	0	0	0	0	0
3	0	0	0	2^1	2^1	0
4	0	0	2^1	0	0	2^1
5	0	0	2^1	0	0	2^1
6	0	0	0	2^1	2^1	0

(2x**1**, 6x**2^1**, 2x**2^2**)

	1	2	3	4	5	6
1	0	1	0	0	0	0
2	1	0	0	0	0	0
3	0	0	0	2^1	2^2	0
4	0	0	2^1	0	0	2^1
5	0	0	2^2	0	0	2^1
6	0	0	0	2^1	2^1	0

(2x**1**, 4x**2^1**, 4x**2^2**) **a**

	1	2	3	4	5	6
1	0	1	0	0	0	0
2	1	0	0	0	0	0
3	0	0	0	2^1	2^2	0
4	0	0	2^1	0	0	2^2
5	0	0	2^2	0	0	2^1
6	0	0	0	2^2	2^1	0

(2x**1**, 4x**2^1**, 4x**2^2**) **b**

	1	2	3	4	5	6
1	0	1	0	0	0	0
2	1	0	0	0	0	0
3	0	0	0	2^1	2^2	0
4	0	0	2^1	0	0	2^1
5	0	0	2^2	0	0	2^2
6	0	0	0	2^1	2^2	0

(2x**1**, 2x**2^1**, 6x**2^2**)

	1	2	3	4	5	6
1	0	1	0	0	0	0
2	1	0	0	0	0	0
3	0	0	0	2^1	2^2	0
4	0	0	2^1	0	0	2^2
5	0	0	2^2	0	0	2^2
6	0	0	0	2^2	2^2	0

(2x**1**, 8x**2^2**)

	1	2	3	4	5	6
1	0	1	0	0	0	0
2	1	0	0	0	0	0
3	0	0	0	2^2	2^2	0
4	0	0	2^2	0	0	2^2
5	0	0	2^2	0	0	2^2
6	0	0	0	2^2	2^2	0

$^1V_2{}^4V_5$: NG

$^1V_2{}^4V_6$: NG

$^1V_4{}^4V_1$

(8x**1**)

	1	2	3	4	5
1	0	1	1	1	1
2	1	0	0	0	0
3	1	0	0	0	0
4	1	0	0	0	0
5	1	0	0	0	0

(6x**1**, 1x**2**)

	1	2	3	4	5
1	0	1	0	0	0
2	1	2	1	0	0
3	0	1	0	0	0
4	0	0	0	0	1
5	0	0	0	1	0

$^1V_4{}^4V_2$

(10x**1**, 1x**2**)

	1	2	3	4	5	6
1	0	1	0	0	0	0
2	1	0	1	1	0	1
3	0	1	2	0	1	0
4	0	1	0	0	0	0
5	0	0	1	0	0	0
6	0	1	0	0	0	0

(8x**1**, 2x**2**) **a**

	1	2	3	4	5	6
1	0	1	0	0	0	0
2	1	2	1	0	0	0
3	0	1	2	0	1	0
4	0	0	0	0	0	1
5	0	0	1	0	0	0
6	0	0	0	1	0	0

(8x**1**, 2x**2**) **b**

	1	2	3	4	5	6
1	0	1	0	0	0	0
2	1	2	1	0	0	0
3	0	1	0	0	0	0
4	0	0	0	0	1	0
5	0	0	0	1	2	1
6	0	0	0	0	1	0

(8x**1**, 2x**2^1**)

	1	2	3	4	5	6
1	0	1	0	0	0	0
2	1	0	1	2^1	0	0
3	0	1	0	0	0	0
4	0	2^1	0	0	1	1
5	0	0	0	1	0	0
6	0	0	0	1	0	0

(8x**1**, 2x**2^2**)

	1	2	3	4	5	6
1	0	1	0	0	0	0
2	1	0	1	2^2	0	0
3	0	1	0	0	0	0
4	0	2^2	0	0	1	1
5	0	0	0	1	0	0
6	0	0	0	1	0	0

(6x**1**, 1x**2**, 2x**2^1**)

	1	2	3	4	5	6
1	2	2^1	0	0	0	0
2	2^1	0	1	1	0	0
3	0	1	0	0	0	0
4	0	1	0	0	0	0
5	0	0	0	0	0	1
6	0	0	0	0	1	0

(6x**1**, 1x**2**, 2x**2^2**)

	1	2	3	4	5	6
1	2	2^2	0	0	0	0
2	2^2	0	1	1	0	0
3	0	1	0	0	0	0
4	0	1	0	0	0	0
5	0	0	0	0	0	1
6	0	0	0	0	1	0

(4x**1**, 2x**2**, 2x**2^1**)

	1	2	3	4	5	6
1	2	2^1	0	0	0	0
2	2^1	2	0	0	0	0
3	0	0	0	1	0	0
4	0	0	1	0	0	0
5	0	0	0	0	0	1
6	0	0	0	0	1	0

(4x**1**, 2x**2**, 2x**2^2**)

	1	2	3	4	5	6
1	2	2^2	0	0	0	0
2	2^2	2	0	0	0	0
3	0	0	0	1	0	0
4	0	0	1	0	0	0
5	0	0	0	0	0	1
6	0	0	0	0	1	0

$^1V_4{}^4V_3$: NG

$^1V_4{}^4V_4$: NG

$^1V_6{}^4V_1$

(10x**1**)

	1	2	3	4	5	6	7
1	0	1	1	1	1	0	0
2	1	0	0	0	0	0	0
3	1	0	0	0	0	0	0
4	1	0	0	0	0	0	0
5	1	0	0	0	0	0	0
6	0	0	0	0	0	0	1
7	0	0	0	0	0	1	0

(8x**1**, 1x**2**)

	1	2	3	4	5	6	7
1	0	1	0	0	0	0	0
2	1	2	1	0	0	0	0
3	0	1	0	0	0	0	0
4	0	0	0	0	1	0	0
5	0	0	0	1	0	0	0
6	0	0	0	0	0	0	1
7	0	0	0	0	0	1	0

$^1V_6{}^4V_2$: NG

$^2V_r{}^3V_r$

$^{2}V_{1}{}^{3}V_{2}$

(4x**1**, 2x**2**)

	1	2	3
1	2	1	0
2	1	0	1
3	0	1	2

(4x**1**, 2x**2**^{1})

	1	2	3
1	0	2^1	1
2	2^1	0	1
3	1	1	0

(4x**1**, 2x**2**^{2})

	1	2	3
1	0	2^2	1
2	2^2	0	1
3	1	1	0

(2x**1**, 3x**2**)

	1	2	3
1	2	1	0
2	1	2	0
3	0	0	2

(2x**1**, 1x**2**, 2x**2**^{1})

	1	2	3
1	2	1	0
2	1	0	2^1
3	0	2^1	0

(2x**1**, 1x**2**, 2x**2**^{2})

	1	2	3
1	2	1	0
2	1	0	2^2
3	0	2^2	0

$^{2}V_{1}{}^{3}V_{4}$

(14x**1**)

	1	2	3	4	5
1	0	1	1	1	0
2	1	0	1	1	0
3	1	1	0	0	1
4	1	1	0	0	1
5	0	0	1	1	0

(12x**1**, 1x**2**) **a**

	1	2	3	4	5
1	0	1	1	1	0
2	1	0	1	1	0
3	1	1	0	1	0
4	1	1	1	0	0
5	0	0	0	0	2

(12x**1**, 1x**2**) **b**

	1	2	3	4	5
1	0	1	1	0	0
2	1	0	1	1	0
3	1	1	0	1	0
4	0	1	1	0	1
5	0	0	0	1	2

(10x**1**, 2x**2**)

	1	2	3	4	5
1	0	1	1	0	0
2	1	0	1	1	0
3	1	1	0	0	1
4	0	1	0	2	0
5	0	0	1	0	2

(10x**1**, 2x**2**^{1})

	1	2	3	4	5
1	0	2^1	1	0	0
2	2^1	0	0	1	0
3	1	0	0	1	1
4	0	1	1	0	1
5	0	0	1	1	0

(10x**1**, 2x**2**^{2})

	1	2	3	4	5
1	0	2^2	1	0	0
2	2^2	0	0	1	0
3	1	0	0	1	1
4	0	1	1	0	1
5	0	0	1	1	0

(8x**1**, 3x**2**)

	1	2	3	4	5
1	0	1	1	1	0
2	1	2	0	0	0
3	1	0	0	0	1
4	1	0	0	2	0
5	0	0	1	0	2

(8x**1**, 1x**2**, 2x**2**^{1}) **a**

	1	2	3	4	5
1	0	2^1	1	0	0
2	2^1	0	0	1	0
3	1	0	0	1	1
4	0	1	1	0	0
5	0	0	1	0	2

(8x**1**, 1x**2**, 2x**2**^{1}) **b**

	1	2	3	4	5
1	0	2^1	1	0	0
2	2^1	0	1	0	0
3	1	1	0	1	0
4	0	0	1	0	1
5	0	0	0	1	2

(8x**1**, 1x**2**, 2x**2**^{2}) **a**

	1	2	3	4	5
1	0	2^2	1	0	0
2	2^2	0	0	1	0
3	1	0	0	1	1
4	0	1	1	0	0
5	0	0	1	0	2

(8x**1**, 1x**2**, 2x**2**^{2}) **b**

	1	2	3	4	5
1	0	2^2	1	0	0
2	2^2	0	1	0	0
3	1	1	0	1	0
4	0	0	1	0	1
5	0	0	0	1	2

(6x**1**, 4x**2**) **a**

	1	2	3	4	5
1	2	0	1	0	0
2	0	2	0	1	0
3	1	0	0	0	1
4	0	1	0	2	0
5	0	0	1	0	2

(6x**1**, 4x**2**) **b**

	1	2	3	4	5
1	2	0	1	0	0
2	0	2	0	0	0
3	1	0	0	1	1
4	0	0	1	2	0
5	0	0	1	0	2

(6x**1**, 2x**2**, 2x**2**^{1}) **a**

	1	2	3	4	5
1	0	2^1	1	0	0
2	2^1	0	1	0	0
3	1	1	0	0	0
4	0	0	0	2	1
5	0	0	0	1	2

(6x**1**, 2x**2**, 2x**2**^{1}) **b**

	1	2	3	4	5
1	0	2^1	1	0	0
2	2^1	0	1	0	0
3	1	1	0	1	0
4	0	0	1	2	0
5	0	0	0	0	2

(6x**1**, 2x**2**, 2x**2**^{1}) **c**

	1	2	3	4	5
1	0	2^1	0	0	0
2	2^1	0	1	0	0
3	0	1	0	1	1
4	0	0	1	2	0
5	0	0	1	0	2

(6x**1**, 2x**2**, 2x**2**^{1}) **d**

	1	2	3	4	5
1	0	2^1	0	1	0
2	2^1	0	1	0	0
3	0	1	0	0	1
4	1	0	0	2	0
5	0	0	1	0	2

(6x**1**, 2x**2**, 2x**2**^{2}) **a**

	1	2	3	4	5
1	0	2^2	1	0	0
2	2^2	0	1	0	0
3	1	1	0	0	0
4	0	0	0	2	1
5	0	0	0	1	2

(6x**1**, 2x**2**, 2x**2**^{2}) **b**

	1	2	3	4	5
1	0	2^2	1	0	0
2	2^2	0	1	0	0
3	1	1	0	1	0
4	0	0	1	2	0
5	0	0	0	0	2

(6x**1**, 2x**2**, 2x**2**^{2}) **c**

	1	2	3	4	5
1	0	2^2	0	0	0
2	2^2	0	1	0	0
3	0	1	0	1	1
4	0	0	1	2	0
5	0	0	1	0	2

(6x**1**, 2x**2**, 2x**2**^{1}) **d**

	1	2	3	4	5
1	0	2^2	0	1	0
2	2^2	0	1	0	0
3	0	1	0	0	1
4	1	0	0	2	0
5	0	0	1	0	2

(6x**1**, 4x**2**^{1}) **a**

	1	2	3	4	5
1	0	2^1	1	0	0
2	2^1	0	1	0	0
3	1	1	0	1	0
4	0	0	1	0	2^1
5	0	0	0	2^1	0

(6x**1**, 4x**2**^{1}) **b**

	1	2	3	4	5
1	0	1	0	0	2^1
2	1	0	2^1	0	0
3	0	2^1	0	1	0
4	0	0	1	0	1
5	2^1	0	0	1	0

(6x**1**, 2x**2**^{1}, 2x**2**^{2}) **a**

	1	2	3	4	5
1	0	2^2	1	0	0
2	2^2	0	1	0	0
3	1	1	0	1	0
4	0	0	1	0	2^1
5	0	0	0	2^1	0

(6x**1**, 2x**2**^{1}, 2x**2**^{2}) **b**

	1	2	3	4	5
1	0	2^1	1	0	0
2	2^1	0	1	0	0
3	1	1	0	1	0
4	0	0	1	0	2^2
5	0	0	0	2^2	0

(6x**1**, 2x**2**^{1}, 2x**2**^{2}) **c**

	1	2	3	4	5
1	0	1	0	0	2^2
2	1	0	2^1	0	0
3	0	2^1	0	1	0
4	0	0	1	0	1
5	2^2	0	0	1	0

(6x**1**, 4x**2**^{2}) **a**

	1	2	3	4	5
1	0	2^2	1	0	0
2	2^2	0	1	0	0
3	1	1	0	1	0
4	0	0	1	0	2^2
5	0	0	0	2^2	0

(6x**1**, 4x**2**^{2}) **b**

	1	2	3	4	5
1	0	1	0	0	2^2
2	1	0	2^2	0	0
3	0	2^2	0	1	0
4	0	0	1	0	1
5	2^2	0	0	1	0

(4x**1**, 5x**2**)

	1	2	3	4	5
1	2	0	0	0	0
2	0	2	1	0	0
3	0	1	2	0	0
4	0	0	0	2	1
5	0	0	0	1	2

(4x**1**, 3x**2**, 2x**2**^{1}) **a**

	1	2	3	4	5
1	0	2^1	0	0	0
2	2^1	0	1	0	0
3	0	1	2	0	0
4	0	0	0	2	1
5	0	0	0	1	2

(4x**1**, 3x**2**, 2x**2**^{1}) **b**

	1	2	3	4	5
1	0	2^1	0	1	0
2	2^1	0	1	0	0
3	0	1	2	0	0
4	1	0	0	2	0
5	0	0	0	0	2

(4x**1**, 3x**2**, 2x**2**^{2}) **a**

	1	2	3	4	5
1	0	2^2	0	0	0
2	2^2	0	1	0	0
3	0	1	2	0	0
4	0	0	0	2	1
5	0	0	0	1	2

(4x**1**, 3x**2**, 2x**2**^{2}) **b**

	1	2	3	4	5
1	0	2^2	0	1	0
2	2^2	0	1	0	0
3	0	1	2	0	0
4	1	0	0	2	0
5	0	0	0	0	2

(4x**1**, 1x**2**, 4x**2**^{1}) **a**

	1	2	3	4	5
1	0	2^1	1	0	0
2	2^1	0	0	1	0
3	1	0	0	2^1	0
4	0	1	2^1	0	0
5	0	0	0	0	2

(4x**1**, 1x**2**, 4x**2**^{1}) **b**

	1	2	3	4	5
1	0	2^1	0	1	0
2	2^1	0	1	0	0
3	0	1	0	0	2^1
4	1	0	0	2	0
5	0	0	2^1	0	0

(4x**1**, 1x**2**, 2x**2**^{1}, 2x**2**^{2}) **a**

	1	2	3	4	5
1	0	2^1	1	0	0
2	2^1	0	0	1	0
3	1	0	0	2^2	0
4	0	1	2^2	0	0
5	0	0	0	0	2

(4x**1**, 1x**2**, 2x**2**^{1}, 2x**2**^{1}) **b**

	1	2	3	4	5
1	0	2^2	0	1	0
2	2^2	0	1	0	0
3	0	1	0	0	2^1
4	1	0	0	2	0
5	0	0	2^1	0	0

(4x**1**, 1x**2**, 2x**2**^{1}, 2x**2**^{1}) **c**

	1	2	3	4	5
1	0	2^1	0	1	0
2	2^1	0	1	0	0
3	0	1	0	0	2^2
4	1	0	0	2	0
5	0	0	2^2	0	0

(4x**1**, 1x**2**, 4x**2**^{2}) **a**

	1	2	3	4	5
1	0	2^2	1	0	0
2	2^2	0	0	1	0
3	1	0	0	2^2	0
4	0	1	2^2	0	0
5	0	0	0	0	2

(4x**1**, 1x**2**, 4x**2**^{2}) **b**

	1	2	3	4	5
1	0	2^2	0	1	0
2	2^2	0	1	0	0
3	0	1	0	0	2^2
4	1	0	0	2	0
5	0	0	2^2	0	0

$^2V_1{}^3V_6$

NG

$^2V_2{}^3V_2$

(10x**1**)

	1	2	3	4
1	0	1	1	1
2	1	0	1	0
3	1	1	0	1
4	1	0	1	0

(8x**1**, 1x**2**)

	1	2	3	4
1	0	1	1	0
2	1	0	1	0
3	1	1	0	1
4	0	0	1	2

(6x**1**, 2x**2**)

	1	2	3	4
1	0	1	0	1
2	1	0	1	0
3	0	1	2	0
4	1	0	0	2

(6x**1**, 2x**2**^{1})

	1	2	3	4
1	0	2^1	0	1
2	2^1	0	1	0
3	0	1	0	1
4	1	0	1	0

(6x**1**, 2x**2**^{2})

	1	2	3	4
1	0	2^2	0	1
2	2^2	0	1	0
3	0	1	0	1
4	1	0	1	0

(4x**1**, 3x**2**)

	1	2	3	4
1	0	1	0	1
2	1	2	0	0
3	0	0	2	0
4	1	0	0	2

(4x**1**, 1x**2**, 2x**2**^{1}) **a**

	1	2	3	4
1	0	2^1	1	0
2	2^1	0	1	0
3	1	1	0	0
4	0	0	0	2

(4x**1**, 1x**2**, 2x**2**^{1}) **b**

	1	2	3	4
1	0	1	0	2^1
2	1	0	1	0
3	0	1	2	0
4	2^1	0	0	0

(4x**1**, 1x**2**, 2x**2**^{2}) **a**

	1	2	3	4
1	0	2^2	1	0
2	2^2	0	1	0
3	1	1	0	0
4	0	0	0	2

(4x**1**, 1x**2**, 2x**2**^{2}) **b**

	1	2	3	4
1	0	1	0	2^2
2	1	0	1	0
3	0	1	2	0
4	2^2	0	0	0

(2x**1**, 4x**2**)

	1	2	3	4
1	2	0	0	1
2	0	2	0	0
3	0	0	2	0
4	1	0	0	2

(2x**1**, 2x**2**, 2x**2**^{1}) **a**

	1	2	3	4
1	0	2^1	0	0
2	2^1	0	0	0
3	0	0	2	1
4	0	0	1	2

(2x**1**, 2x**2**, 2x**2**^{1}) **b**

	1	2	3	4
1	0	1	0	2^1
2	1	2	0	0
3	0	0	2	0
4	2^1	0	0	0

(2x**1**, 2x**2**, 2x**2**^{2}) **a**

	1	2	3	4
1	0	2^2	0	0
2	2^2	0	0	0
3	0	0	2	1
4	0	0	1	2

(2x**1**, 2x**2**, 2x**2**^{2}) **b**

	1	2	3	4
1	0	1	0	2^2
2	1	2	0	0
3	0	0	2	0
4	2^2	0	0	0

(2x**1**, 4x**2**^{1})

	1	2	3	4
1	0	1	0	2^1
2	1	0	2^1	0
3	0	2^1	0	0
4	2^1	0	0	0

(2x**1**, 2x**2**^{1}, 2x**2**^{2})

	1	2	3	4
1	0	1	0	2^2
2	1	0	2^1	0
3	0	2^1	0	0
4	2^2	0	0	0

(2x**1**, 4x**2**^{2})

	1	2	3	4
1	0	1	0	2^2
2	1	0	2^2	0
3	0	2^2	0	0
4	2^2	0	0	0

$^2V_2{}^3V_4$

(16x**1**) **a**

	1	2	3	4	5	6
1	0	0	1	1	1	0
2	0	0	1	1	1	0
3	1	1	0	0	0	1
4	1	1	0	0	0	1
5	1	1	0	0	0	0
6	0	0	1	1	0	0

(16x**1**) **b**

	1	2	3	4	5	6
1	0	1	0	1	1	0
2	1	0	1	0	1	0
3	0	1	0	1	0	1
4	1	0	1	0	0	1
5	1	1	0	0	0	0
6	0	0	1	1	0	0

(16x**1**) **c**

	1	2	3	4	5	6
1	0	1	0	1	0	0
2	1	0	1	0	0	0
3	0	1	0	0	1	1
4	1	0	0	0	1	1
5	0	0	1	1	0	1
6	0	0	1	1	1	0

(16x**1**) **d**

	1	2	3	4	5	6
1	0	1	0	1	0	0
2	1	0	1	0	0	1
3	0	1	0	1	0	1
4	1	0	1	0	1	0
5	0	0	0	1	0	1
6	0	1	1	0	1	0

(14x**1**, 1x**2**) **a**

	1	2	3	4	5	6
1	0	1	1	1	0	0
2	1	0	1	1	0	0
3	1	1	0	0	1	0
4	1	1	0	0	1	0
5	0	0	1	1	0	0
6	0	0	0	0	0	2

(14x**1**, 1x**2**) **b**

	1	2	3	4	5	6
1	0	1	1	1	0	0
2	1	0	1	0	0	0
3	1	1	0	1	0	0
4	1	0	1	0	1	0
5	0	0	0	1	0	1
6	0	0	0	0	1	2

(14x**1**, 1x**2**) **c**

	1	2	3	4	5	6
1	0	1	0	1	0	0
2	1	0	1	0	1	0
3	0	1	0	1	1	0
4	1	0	1	0	0	1
5	0	1	1	0	0	0
6	0	0	0	1	0	2

(14x**1**, 1x**2**) **d**

	1	2	3	4	5	6
1	0	0	1	1	0	0
2	0	0	1	1	0	0
3	1	1	0	0	1	0
4	1	1	0	0	1	0
5	0	0	1	1	0	1
6	0	0	0	0	1	2

(14x**1**, 1x**2**) **e**

	1	2	3	4	5	6
1	0	1	0	1	0	0
2	1	0	1	0	1	0
3	0	1	0	1	1	0
4	1	0	1	0	0	0
5	0	1	1	0	0	1
6	0	0	0	0	1	2

(12x**1**, 2x**2**) **a**

	1	2	3	4	5	6
1	0	1	1	1	0	0
2	1	0	1	1	0	0
3	1	1	0	1	0	0
4	1	1	1	0	0	0
5	0	0	0	0	2	0
6	0	0	0	0	0	2

(12x**1**, 2x**2**) **b**

	1	2	3	4	5	6
1	0	1	0	1	0	0
2	1	0	1	0	0	0
3	0	1	0	1	0	1
4	1	0	1	0	1	0
5	0	0	0	1	2	0
6	0	0	1	0	0	2

(12x**1**, 2x**2**) **c**

	1	2	3	4	5	6
1	0	1	0	1	0	0
2	1	0	1	0	0	1
3	0	1	0	1	0	0
4	1	0	1	0	1	0
5	0	0	0	1	2	0
6	0	1	0	0	0	2

(12x**1**, 2x**2**) **d**

	1	2	3	4	5	6
1	0	1	0	1	0	0
2	1	0	0	1	0	1
3	0	0	2	0	1	0
4	1	1	0	0	1	0
5	0	0	1	1	0	0
6	0	1	0	0	0	2

(12x**1**, 2x**2**) **e**

	1	2	3	4	5	6
1	0	1	1	1	0	0
2	1	0	1	0	0	0
3	1	1	0	1	0	0
4	1	0	1	0	1	0
5	0	0	0	1	2	0
6	0	0	0	0	0	2

(12x**1**, 2x**2**) **f**

	1	2	3	4	5	6
1	0	1	0	1	0	0
2	1	0	0	1	0	0
3	0	0	2	0	1	0
4	1	1	0	0	1	0
5	0	0	1	1	0	1
6	0	0	0	0	1	2

(12x**1**, 2x**2**) **g**

	1	2	3	4	5	6
1	0	1	1	1	0	0
2	1	0	1	0	0	0
3	1	1	0	1	0	0
4	1	0	1	0	0	0
5	0	0	0	0	2	1
6	0	0	0	0	1	2

(12x**1**, 2x$\mathbf{2}^{1}$) **a**

	1	2	3	4	5	6
1	0	1	1	1	0	0
2	1	0	1	1	0	0
3	1	1	0	1	0	0
4	1	1	1	0	0	0
5	0	0	0	0	0	2^{1}
6	0	0	0	0	2^{1}	0

(12x**1**, 2x$\mathbf{2}^{1}$) **b**

	1	2	3	4	5	6
1	0	1	0	1	0	0
2	1	0	1	0	0	0
3	0	1	0	1	0	1
4	1	0	1	0	1	0
5	0	0	0	1	0	2^{1}
6	0	0	1	0	2^{1}	0

(12x**1**, 2x$\mathbf{2}^{1}$) **c**

	1	2	3	4	5	6
1	0	0	1	1	0	0
2	0	0	1	1	0	0
3	1	1	0	0	0	1
4	1	1	0	0	1	0
5	0	0	0	1	0	2^{1}
6	0	0	1	0	2^{1}	0

(12x**1**, 2x$\mathbf{2}^{1}$) **d**

	1	2	3	4	5	6
1	0	1	1	1	0	0
2	1	0	1	0	0	0
3	1	1	0	1	0	0
4	1	0	1	0	1	0
5	0	0	0	1	0	2^{1}
6	0	0	0	0	2^{1}	0

(12x**1**, 2x$\mathbf{2}^{1}$) **e**

	1	2	3	4	5	6
1	0	1	1	1	0	0
2	1	0	1	0	0	0
3	1	1	0	0	0	1
4	1	0	0	0	1	0
5	0	0	0	1	0	2^{1}
6	0	0	1	0	2^{1}	0

(12x**1**, 2x$\mathbf{2}^{1}$) **f**

	1	2	3	4	5	6
1	0	1	1	1	0	0
2	1	0	1	0	0	0
3	1	1	0	0	0	0
4	1	0	0	0	1	1
5	0	0	0	1	0	2^{1}
6	0	0	0	1	2^{1}	0

(12x**1**, 2x$\mathbf{2}^{2}$) **a**

	1	2	3	4	5	6
1	0	1	1	1	0	0
2	1	0	1	1	0	0
3	1	1	0	1	0	0
4	1	1	1	0	0	0
5	0	0	0	0	0	2^{2}
6	0	0	0	0	2^{2}	0

(12x**1**, 2x$\mathbf{2}^{2}$) **b**

	1	2	3	4	5	6
1	0	1	0	1	0	0
2	1	0	1	0	0	0
3	0	1	0	1	0	1
4	1	0	1	0	1	0
5	0	0	0	1	0	2^{2}
6	0	0	1	0	2^{2}	0

(12x**1**, 2x$\mathbf{2}^{2}$) **c**

	1	2	3	4	5	6
1	0	0	1	1	0	0
2	0	0	1	1	0	0
3	1	1	0	0	0	1
4	1	1	0	0	1	0
5	0	0	0	1	0	2^{2}
6	0	0	1	0	2^{2}	0

(12x**1**, 2x$\mathbf{2}^{2}$) **d**

	1	2	3	4	5	6
1	0	1	1	1	0	0
2	1	0	1	0	0	0
3	1	1	0	1	0	0
4	1	0	1	0	1	0
5	0	0	0	1	0	2^{2}
6	0	0	0	0	2^{2}	0

(12x**1**, 2x$\mathbf{2}^{2}$) **e**

	1	2	3	4	5	6
1	0	1	1	1	0	0
2	1	0	1	0	0	0
3	1	1	0	0	0	1
4	1	0	0	0	1	0
5	0	0	0	1	0	2^{2}
6	0	0	1	0	2^{2}	0

(12x**1**, 2x$\mathbf{2}^{2}$) **f**

	1	2	3	4	5	6
1	0	1	1	1	0	0
2	1	0	1	0	0	0
3	1	1	0	0	0	0
4	1	0	0	0	1	1
5	0	0	0	1	0	2^{2}
6	0	0	0	1	2^{2}	0

(10x**1**, 3x**2**) **a**

	1	2	3	4	5	6
1	0	1	0	1	0	1
2	1	0	1	0	0	0
3	0	1	2	0	0	0
4	1	0	0	0	1	0
5	0	0	0	1	2	0
6	1	0	0	0	0	2

(10x**1**, 3x**2**) **b**

	1	2	3	4	5	6
1	0	1	1	0	0	1
2	1	0	1	0	0	0
3	1	1	0	1	0	0
4	0	0	1	2	0	0
5	0	0	0	0	2	0
6	1	0	0	0	0	2

(10x**1**, 3x**2**) **c**

	1	2	3	4	5	6
1	0	1	0	1	0	1
2	1	0	1	0	0	0
3	0	1	0	0	1	0
4	1	0	0	2	0	0
5	0	0	1	0	2	0
6	1	0	0	0	0	2

(10x**1**, 3x**2**) **d**

	1	2	3	4	5	6
1	0	1	1	0	0	1
2	1	0	1	0	0	0
3	1	1	0	0	0	0
4	0	0	0	2	1	0
5	0	0	0	1	2	0
6	1	0	0	0	0	2

(10x**1**, 1x**2**, 2x$\mathbf{2}^{1}$) **a**

	1	2	3	4	5	6
1	0	2^{1}	0	1	0	0
2	2^{1}	0	1	0	0	0
3	0	1	0	1	1	0
4	1	0	1	0	1	0
5	0	0	1	1	0	0
6	0	0	0	0	0	2

(10x**1**, 1x**2**, 2x$\mathbf{2}^{1}$) **b**

	1	2	3	4	5	6
1	0	2^{1}	0	1	0	0
2	2^{1}	0	1	0	0	0
3	0	1	0	1	0	0
4	1	0	1	0	1	0
5	0	0	0	1	0	1
6	0	0	0	0	1	2

(10x**1**, 1x**2**, 2x$\mathbf{2}^{1}$) **c**

	1	2	3	4	5	6
1	0	2^{1}	0	1	0	0
2	2^{1}	0	0	1	0	0
3	0	0	2	0	0	1
4	1	1	0	0	1	0
5	0	0	0	1	0	1
6	0	0	1	0	1	0

(10x**1**, 1x**2**, 2x**2**^{1}) **d**

	1	2	3	4	5	6
1	0	2^{1}	0	1	0	0
2	2^{1}	0	1	0	0	0
3	0	1	0	0	1	0
4	1	0	0	0	1	0
5	0	0	1	1	0	1
6	0	0	0	0	1	2

(10x**1**, 1x**2**, 2x**2**^{1}) **e**

	1	2	3	4	5	6
1	0	1	0	2^{1}	0	0
2	1	0	1	0	0	0
3	0	1	0	0	1	0
4	2^{1}	0	0	0	1	0
5	0	0	1	1	0	1
6	0	0	0	0	1	2

(10x**1**, 1x**2**, 2x**2**^{1}) **f**

	1	2	3	4	5	6
1	0	1	0	1	0	0
2	1	0	1	1	0	0
3	0	1	2	0	0	0
4	1	1	0	0	1	0
5	0	0	0	1	0	2^{1}
6	0	0	0	0	2^{1}	0

(10x**1**, 1x**2**, 2x**2**^{1}) **g**

	1	2	3	4	5	6
1	0	1	0	1	0	0
2	1	0	0	1	0	0
3	0	0	2	0	0	1
4	1	1	0	0	1	0
5	0	0	0	1	0	2^{1}
6	0	0	1	0	2^{1}	0

(10x**1**, 1x**2**, 2x**2**^{2}) **a**

	1	2	3	4	5	6
1	0	2^{2}	0	1	0	0
2	2^{2}	0	1	0	0	0
3	0	1	0	1	1	0
4	1	0	1	0	1	0
5	0	0	1	1	0	0
6	0	0	0	0	0	2

(10x**1**, 1x**2**, 2x**2**^{2}) **b**

	1	2	3	4	5	6
1	0	2^{2}	0	1	0	0
2	2^{2}	0	1	0	0	0
3	0	1	0	1	0	0
4	1	0	1	0	1	0
5	0	0	0	1	0	1
6	0	0	0	0	1	2

(10x**1**, 1x**2**, 2x**2**^{2}) **c**

	1	2	3	4	5	6
1	0	2^{2}	0	1	0	0
2	2^{2}	0	0	1	0	0
3	0	0	2	0	0	1
4	1	1	0	0	1	0
5	0	0	0	1	0	1
6	0	0	1	0	1	0

(10x**1**, 1x**2**, 2x**2**^{2}) **d**

	1	2	3	4	5	6
1	0	2^{2}	0	1	0	0
2	2^{2}	0	1	0	0	0
3	0	1	0	0	1	0
4	1	0	0	0	1	0
5	0	0	1	1	0	1
6	0	0	0	0	1	2

(10x**1**, 1x**2**, 2x**2**^{2}) **e**

	1	2	3	4	5	6
1	0	1	0	2^{2}	0	0
2	1	0	1	0	0	0
3	0	1	0	0	1	0
4	2^{2}	0	0	0	1	0
5	0	0	1	1	0	1
6	0	0	0	0	1	2

(10x**1**, 1x**2**, 2x**2**^{2}) **f**

	1	2	3	4	5	6
1	0	1	0	1	0	0
2	1	0	1	1	0	0
3	0	1	2	0	0	0
4	1	1	0	0	1	0
5	0	0	0	1	0	2^{2}
6	0	0	0	0	2^{2}	0

(10x**1**, 1x**2**, 2x**2**^{2}) **g**

	1	2	3	4	5	6
1	0	1	0	1	0	0
2	1	0	0	1	0	0
3	0	0	2	0	0	1
4	1	1	0	0	1	0
5	0	0	0	1	0	2^{2}
6	0	0	1	0	2^{2}	0

(8x**1**, 4x**2**) **a**

	1	2	3	4	5	6
1	0	1	0	1	0	0
2	1	2	0	0	0	0
3	0	0	2	0	0	1
4	1	0	0	2	0	0
5	0	0	0	0	2	1
6	0	0	1	0	1	0

(8x**1**, 4x**2**) **b**

	1	2	3	4	5	6
1	0	1	0	0	0	1
2	1	2	0	0	0	0
3	0	0	2	0	0	1
4	0	0	0	2	0	0
5	0	0	0	0	2	1
6	1	0	1	0	1	0

(8x**1**, 4x**2**) **c**

	1	2	3	4	5	6
1	0	1	0	0	0	1
2	1	2	0	0	0	0
3	0	0	2	1	0	0
4	0	0	1	2	0	0
5	0	0	0	0	2	1
6	1	0	0	0	1	0

(8x**1**, 2x**2**, 2x**2**^{1}) **a**

	1	2	3	4	5	6
1	0	2^{1}	0	1	0	0
2	2^{1}	0	0	1	0	0
3	0	0	2	0	0	0
4	1	1	0	0	1	0
5	0	0	0	1	0	1
6	0	0	0	0	1	2

(8x**1**, 2x**2**, 2x**2**^{1}) **b**

	1	2	3	4	5	6
1	0	2^{1}	0	1	0	0
2	2^{1}	0	1	0	0	0
3	0	1	0	0	0	1
4	1	0	0	0	1	0
5	0	0	0	1	2	0
6	0	0	1	0	0	2

(8x**1**, 2x**2**, 2x**2**^{1}) **c**

	1	2	3	4	5	6
1	0	2^{1}	0	1	0	0
2	2^{1}	0	0	1	0	0
3	0	0	2	0	1	0
4	1	1	0	0	0	0
5	0	0	1	0	0	1
6	0	0	0	0	1	2

(8x**1**, 2x**2**, 2x**2**^{1}) **d**

	1	2	3	4	5	6
1	0	2^{1}	0	1	0	0
2	2^{1}	0	1	0	0	0
3	0	1	0	1	0	0
4	1	0	1	0	1	0
5	0	0	0	1	2	0
6	0	0	0	0	0	2

(8x**1**, 2x**2**, 2x**2**^{1}) **e**

	1	2	3	4	5	6
1	0	0	0	2^{1}	0	0
2	0	2	0	0	1	0
3	0	0	2	0	0	1
4	2^{1}	0	0	0	1	0
5	0	1	0	1	0	1
6	0	0	1	0	1	0

(8x**1**, 2x**2**, 2x**2**^{1}) **f**

	1	2	3	4	5	6
1	2	0	0	1	0	0
2	0	2	0	1	0	0
3	0	0	0	0	0	2^{1}
4	1	1	0	0	1	0
5	0	0	0	1	0	1
6	0	0	2^{1}	0	1	0

(8x**1**, 2x**2**, 2x**2**^{1}) **g**

	1	2	3	4	5	6
1	0	1	0	2^{1}	0	0
2	1	0	1	0	0	0
3	0	1	0	0	0	1
4	2^{1}	0	0	0	1	0
5	0	0	0	1	2	0
6	0	0	1	0	0	2

(8x**1**, 2x**2**, 2x**2**^{1}) **h**

	1	2	3	4	5	6
1	0	2^{1}	0	1	0	0
2	2^{1}	0	1	0	0	0
3	0	1	0	1	0	0
4	1	0	1	0	0	0
5	0	0	0	0	2	1
6	0	0	0	0	1	2

(8x**1**, 2x**2**, 2x**2**^{2}) **a**

	1	2	3	4	5	6
1	0	2^{2}	0	1	0	0
2	2^{2}	0	0	1	0	0
3	0	0	2	0	0	0
4	1	1	0	0	1	0
5	0	0	0	1	0	1
6	0	0	0	0	1	2

(8x**1**, 2x**2**, 2x**2**^{2}) **b**

	1	2	3	4	5	6
1	0	2^{2}	0	1	0	0
2	2^{2}	0	1	0	0	0
3	0	1	0	0	0	1
4	1	0	0	0	1	0
5	0	0	0	1	2	0
6	0	0	1	0	0	2

(8x**1**, 2x**2**, 2x**2**^{2}) **c**

	1	2	3	4	5	6
1	0	2^{2}	0	1	0	0
2	2^{2}	0	0	1	0	0
3	0	0	2	0	1	0
4	1	1	0	0	0	0
5	0	0	1	0	0	1
6	0	0	0	0	1	2

(8x**1**, 2x**2**, 2x**2**^{2}) **d**

	1	2	3	4	5	6
1	0	2^{2}	0	1	0	0
2	2^{2}	0	1	0	0	0
3	0	1	0	1	0	0
4	1	0	1	0	1	0
5	0	0	0	1	2	0
6	0	0	0	0	0	2

(8x**1**, 2x**2**, 2x**2**^{2}) **e**

	1	2	3	4	5	6
1	0	0	0	2^{2}	0	0
2	0	2	0	0	1	0
3	0	0	2	0	0	1
4	2^{2}	0	0	0	1	0
5	0	1	0	1	0	1
6	0	0	1	0	1	0

(8x**1**, 2x**2**, 2x**2**^{2}) **f**

	1	2	3	4	5	6
1	2	0	0	1	0	0
2	0	2	0	1	0	0
3	0	0	0	0	0	2^{2}
4	1	1	0	0	1	0
5	0	0	0	1	0	1
6	0	0	2^{2}	0	1	0

(8x**1**, 2x**2**, 2x**2**^{2}) **g**

	1	2	3	4	5	6
1	0	1	0	2^{2}	0	0
2	1	0	1	0	0	0
3	0	1	0	0	0	1
4	2^{2}	0	0	0	1	0
5	0	0	0	1	2	0
6	0	0	1	0	0	2

(8x**1**, 2x**2**, 2x**2**^{2}) **h**

	1	2	3	4	5	6
1	0	2^{2}	0	1	0	0
2	2^{2}	0	1	0	0	0
3	0	1	0	1	0	0
4	1	0	1	0	0	0
5	0	0	0	0	2	1
6	0	0	0	0	1	2

(8x**1**, 4x**2^1**) **a**

	1	2	3	4	5	6
1	0	2^1	0	1	0	0
2	2^1	0	1	0	0	0
3	0	1	0	0	0	1
4	1	0	0	0	1	0
5	0	0	0	1	0	2^1
6	0	0	1	0	2^1	0

(8x**1**, 4x**2^1**) **b**

	1	2	3	4	5	6
1	0	2^1	0	1	0	0
2	2^1	0	1	0	0	0
3	0	1	0	0	0	2^1
4	1	0	0	0	1	0
5	0	0	0	1	0	1
6	0	0	2^1	0	1	0

(8x**1**, 4x**2^1**) **c**

	1	2	3	4	5	6
1	0	2^1	0	1	0	0
2	2^1	0	1	0	0	0
3	0	1	0	1	0	0
4	1	0	1	0	1	0
5	0	0	0	1	0	2^1
6	0	0	0	0	2^1	0

(8x**1**, 4x**2^1**) **d**

	1	2	3	4	5	6
1	0	2^1	0	1	0	0
2	2^1	0	0	1	0	0
3	0	0	0	0	1	2^1
4	1	1	0	0	0	0
5	0	0	1	0	0	1
6	0	0	2^1	0	1	0

(8x**1**, 4x**2^1**) **e**

	1	2	3	4	5	6
1	0	2^1	0	1	0	0
2	2^1	0	0	1	0	0
3	0	0	0	0	0	2^1
4	1	1	0	0	1	0
5	0	0	0	1	0	1
6	0	0	2^1	0	1	0

(8x**1**, 2x**2^1**, 2x**2^2**) **a**

	1	2	3	4	5	6
1	0	2^1	0	1	0	0
2	2^1	0	1	0	0	0
3	0	1	0	0	0	1
4	1	0	0	0	1	0
5	0	0	0	1	0	2^2
6	0	0	1	0	2^2	0

(8x**1**, 2x**2^1**, 2x**2^2**) **b**

	1	2	3	4	5	6
1	0	2^1	0	1	0	0
2	2^1	0	1	0	0	0
3	0	1	0	0	0	2^2
4	1	0	0	0	1	0
5	0	0	0	1	0	1
6	0	0	2^2	0	1	0

(8x**1**, 2x**2^1**, 2x**2^2**) **c**

	1	2	3	4	5	6
1	0	2^1	0	1	0	0
2	2^1	0	1	0	0	0
3	0	1	0	1	0	0
4	1	0	1	0	1	0
5	0	0	0	1	0	2^2
6	0	0	0	0	2^2	0

(8x**1**, 2x**2^1**, 2x**2^2**) **d**

	1	2	3	4	5	6
1	0	2^2	0	1	0	0
2	2^2	0	1	0	0	0
3	0	1	0	1	0	0
4	1	0	1	0	1	0
5	0	0	0	1	0	2^1
6	0	0	0	0	2^1	0

(8x**1**, 2x**2^1**, 2x**2^2**) **e**

	1	2	3	4	5	6
1	0	2^2	0	1	0	0
2	2^2	0	0	1	0	0
3	0	0	0	0	1	2^1
4	1	1	0	0	0	0
5	0	0	1	0	0	1
6	0	0	2^1	0	1	0

(8x**1**, 2x**2^1**, 2x**2^2**) **f**

	1	2	3	4	5	6
1	0	2^1	0	1	0	0
2	2^1	0	0	1	0	0
3	0	0	0	0	0	2^2
4	1	1	0	0	1	0
5	0	0	0	1	0	1
6	0	0	2^2	0	1	0

(8x**1**, 2x**2^1**, 2x**2^2**) **g**

	1	2	3	4	5	6
1	0	2^2	0	1	0	0
2	2^2	0	0	1	0	0
3	0	0	0	0	0	2^1
4	1	1	0	0	1	0
5	0	0	0	1	0	1
6	0	0	2^1	0	1	0

(8x**1**, 4x**2^2**) **a**

	1	2	3	4	5	6
1	0	2^2	0	1	0	0
2	2^2	0	1	0	0	0
3	0	1	0	0	0	1
4	1	0	0	0	1	0
5	0	0	0	1	0	2^2
6	0	0	1	0	2^2	0

(8x**1**, 4x**2^2**) **b**

	1	2	3	4	5	6
1	0	2^2	0	1	0	0
2	2^2	0	1	0	0	0
3	0	1	0	0	0	2^2
4	1	0	0	0	1	0
5	0	0	0	1	0	1
6	0	0	2^2	0	1	0

(8x**1**, 4x**2^2**) **c**

	1	2	3	4	5	6
1	0	2^2	0	1	0	0
2	2^2	0	1	0	0	0
3	0	1	0	1	0	0
4	1	0	1	0	1	0
5	0	0	0	1	0	2^2
6	0	0	0	0	2^2	0

(8x**1**, 4x**2^2**) **d**

	1	2	3	4	5	6
1	0	2^2	0	1	0	0
2	2^2	0	0	1	0	0
3	0	0	0	0	1	2^2
4	1	1	0	0	0	0
5	0	0	1	0	0	1
6	0	0	2^2	0	1	0

(8x**1**, 4x**2^2**) **e**

	1	2	3	4	5	6
1	0	2^2	0	1	0	0
2	2^2	0	0	1	0	0
3	0	0	0	0	0	2^2
4	1	1	0	0	1	0
5	0	0	0	1	0	1
6	0	0	2^2	0	1	0

(6x**1**, 5x**2**) **a**

	1	2	3	4	5	6
1	2	0	0	0	0	0
2	0	2	0	0	0	0
3	0	0	2	0	1	0
4	0	0	0	2	1	0
5	0	0	1	1	0	1
6	0	0	0	0	1	2

(6x**1**, 5x**2**) **b**

	1	2	3	4	5	6
1	2	1	0	0	0	0
2	1	2	0	0	0	0
3	0	0	2	0	0	0
4	0	0	0	2	1	0
5	0	0	0	1	0	1
6	0	0	0	0	1	2

(6x**1**, 3x**2**, 2x**2^1**) **a**

	1	2	3	4	5	6
1	2	1	0	0	0	0
2	1	2	0	0	0	0
3	0	0	0	2^1	0	0
4	0	0	2^1	0	1	0
5	0	0	0	1	0	1
6	0	0	0	0	1	2

(6x**1**, 3x**2**, 2x**2^1**) **b**

	1	2	3	4	5	6
1	2	1	0	0	0	0
2	1	2	0	0	0	0
3	0	0	0	2^1	1	0
4	0	0	2^1	0	1	0
5	0	0	1	1	0	0
6	0	0	0	0	0	2

(6x**1**, 3x**2**, 2x**2^1**) **c**

	1	2	3	4	5	6
1	2	0	0	0	1	0
2	0	2	1	0	0	0
3	0	1	0	2^1	0	0
4	0	0	2^1	0	1	0
5	1	0	0	1	0	0
6	0	0	0	0	0	2

(6x**1**, 3x**2**, 2x**2^1**) **d**

	1	2	3	4	5	6
1	0	1	0	0	1	0
2	1	2	0	0	0	0
3	0	0	0	2^1	0	1
4	0	0	2^1	0	0	0
5	1	0	0	0	2	0
6	0	0	1	0	0	2

(6x**1**, 3x**2**, 2x**2^1**) **e**

	1	2	3	4	5	6
1	0	2^1	0	0	0	0
2	2^1	0	0	0	0	0
3	0	0	2	0	1	0
4	0	0	0	2	1	0
5	0	0	1	1	0	1
6	0	0	0	0	1	2

(6x**1**, 3x**2**, 2x**2^1**) **f**

	1	2	3	4	5	6
1	2	0	0	0	0	0
2	0	0	0	0	0	2^1
3	0	0	2	0	1	0
4	0	0	0	2	1	0
5	0	0	1	1	0	1
6	0	2^1	0	0	1	0

(6x**1**, 3x**2**, 2x**2^1**) **g**

	1	2	3	4	5	6
1	2	0	0	0	0	0
2	0	2	0	0	0	0
3	0	0	0	2^1	1	0
4	0	0	2^1	0	1	0
5	0	0	1	1	0	1
6	0	0	0	0	1	2

(6x**1**, 3x**2**, 2x**2^2**) **a**

	1	2	3	4	5	6
1	2	1	0	0	0	0
2	1	2	0	0	0	0
3	0	0	0	2^2	0	0
4	0	0	2^2	0	1	0
5	0	0	0	1	0	1
6	0	0	0	0	1	2

(6x**1**, 3x**2**, 2x**2^2**) **b**

	1	2	3	4	5	6
1	2	1	0	0	0	0
2	1	2	0	0	0	0
3	0	0	0	2^2	1	0
4	0	0	2^2	0	1	0
5	0	0	1	1	0	0
6	0	0	0	0	0	2

(6x**1**, 3x**2**, 2x**2^2**) **c**

	1	2	3	4	5	6
1	2	0	0	0	1	0
2	0	2	1	0	0	0
3	0	1	0	2^2	0	0
4	0	0	2^2	0	1	0
5	1	0	0	1	0	0
6	0	0	0	0	0	2

(6x**1**, 3x**2**, 2x**2^2**) **d**

	1	2	3	4	5	6
1	0	1	0	0	1	0
2	1	2	0	0	0	0
3	0	0	0	2^2	0	1
4	0	0	2^2	0	0	0
5	1	0	0	0	2	0
6	0	0	1	0	0	2

(6x**1**, 3x**2**, 2x**2**^{2}) **e**

	1	2	3	4	5	6
1	0	2^2	0	0	0	0
2	2^2	0	0	0	0	0
3	0	0	2	0	1	0
4	0	0	0	2	1	0
5	0	0	1	1	0	1
6	0	0	0	0	1	2

(6x**1**, 3x**2**, 2x**2**^{2}) **f**

	1	2	3	4	5	6
1	2	0	0	0	0	0
2	0	0	0	0	0	2^2
3	0	0	2	0	1	0
4	0	0	0	2	1	0
5	0	0	1	1	0	1
6	0	2^2	0	0	1	0

(6x**1**, 3x**2**, 2x**2**^{2}) **g**

	1	2	3	4	5	6
1	2	0	0	0	0	0
2	0	2	0	0	0	0
3	0	0	0	2^2	1	0
4	0	0	2^2	0	1	0
5	0	0	1	1	0	1
6	0	0	0	0	1	2

(6x**1**, 1x**2**, 4x**2**^{1}) **a**

	1	2	3	4	5	6
1	2	1	0	0	0	0
2	1	0	0	0	0	2^1
3	0	0	0	2^1	0	0
4	0	0	2^1	0	1	0
5	0	0	0	1	0	1
6	0	2^1	0	0	1	0

(6x**1**, 1x**2**, 4x**2**^{1}) **b**

	1	2	3	4	5	6
1	2	1	0	0	0	0
2	1	0	0	0	0	2^1
3	0	0	0	2^1	1	0
4	0	0	2^1	0	1	0
5	0	0	1	1	0	0
6	0	2^1	0	0	0	0

(6x**1**, 1x**2**, 4x**2**^{1}) **c**

	1	2	3	4	5	6
1	2	0	0	0	1	0
2	0	0	0	0	0	2^1
3	0	0	0	2^1	1	0
4	0	0	2^1	0	1	0
5	1	0	1	1	0	0
6	0	2^1	0	0	0	0

(6x**1**, 1x**2**, 4x**2**^{1}) **d**

	1	2	3	4	5	6
1	2	0	0	0	1	0
2	0	0	0	0	0	2^1
3	0	0	0	2^1	0	0
4	0	0	2^1	0	1	0
5	1	0	0	1	0	1
6	0	2^1	0	0	1	0

(6x**1**, 1x**2**, 4x**2**^{1}) **e**

	1	2	3	4	5	6
1	2	1	0	0	0	0
2	1	0	0	0	0	1
3	0	0	0	2^1	0	0
4	0	0	2^1	0	1	0
5	0	0	0	1	0	2^1
6	0	1	0	0	2^1	0

(6x**1**, 1x**2**, 4x**2**^{1}) **f**

	1	2	3	4	5	6
1	2	0	0	0	0	0
2	0	0	0	0	0	2^1
3	0	0	0	2^1	1	0
4	0	0	2^1	0	1	0
5	0	0	1	1	0	1
6	0	2^1	0	0	1	0

(6x**1**, 1x**2**, 4x**2**^{1}) **g**

	1	2	3	4	5	6
1	2	0	0	0	0	0
2	0	0	0	0	1	2^1
3	0	0	0	2^1	0	1
4	0	0	2^1	0	1	0
5	0	1	0	1	0	0
6	0	2^1	1	0	0	0

(6x**1**, 1x**2**, 2x**2**^{1}, 2x**2**^{2}) **a**

	1	2	3	4	5	6
1	2	1	0	0	0	0
2	1	0	0	0	0	2^1
3	0	0	0	2^2	0	0
4	0	0	2^2	0	1	0
5	0	0	0	1	0	1
6	0	2^1	0	0	1	0

(6x**1**, 1x**2**, 2x**2**^{1}, 2x**2**^{2}) **b**

	1	2	3	4	5	6
1	2	1	0	0	0	0
2	1	0	0	0	0	2^2
3	0	0	0	2^1	0	0
4	0	0	2^1	0	1	0
5	0	0	0	1	0	1
6	0	2^2	0	0	1	0

(6x**1**, 1x**2**, 2x**2**^{1}, 2x**2**^{2}) **c**

	1	2	3	4	5	6
1	2	1	0	0	0	0
2	1	0	0	0	0	2^1
3	0	0	0	2^2	1	0
4	0	0	2^2	0	1	0
5	0	0	1	1	0	0
6	0	2^1	0	0	0	0

(6x**1**, 1x**2**, 2x**2**^{1}, 2x**2**^{2}) **d**

	1	2	3	4	5	6
1	2	1	0	0	0	0
2	1	0	0	0	0	2^2
3	0	0	0	2^1	1	0
4	0	0	2^1	0	1	0
5	0	0	1	1	0	0
6	0	2^2	0	0	0	0

(6x**1**, 1x**2**, 2x**2**^{1}, 2x**2**^{2}) **e**

	1	2	3	4	5	6
1	2	0	0	0	1	0
2	0	0	0	0	0	2^1
3	0	0	0	2^2	1	0
4	0	0	2^2	0	1	0
5	1	0	1	1	0	0
6	0	2^1	0	0	0	0

(6x**1**, 1x**2**, 2x**2**^{1}, 2x**2**^{2}) **f**

	1	2	3	4	5	6
1	2	0	0	0	1	0
2	0	0	0	0	0	2^2
3	0	0	0	2^1	1	0
4	0	0	2^1	0	1	0
5	1	0	1	1	0	0
6	0	2^2	0	0	0	0

(6x**1**, 1x**2**, 2x**2**^{1}, 2x**2**^{2}) **g**

	1	2	3	4	5	6
1	2	0	0	0	1	0
2	0	0	0	0	0	2^2
3	0	0	0	2^1	0	0
4	0	0	2^1	0	1	0
5	1	0	0	1	0	1
6	0	2^2	0	0	1	0

(6x**1**, 1x**2**, 2x**2**^{1}, 2x**2**^{2}) **h**

	1	2	3	4	5	6
1	2	1	0	0	0	0
2	1	0	0	0	0	1
3	0	0	0	2^2	0	0
4	0	0	2^2	0	1	0
5	0	0	0	1	0	2^1
6	0	1	0	0	2^1	0

(6x**1**, 1x**2**, 2x**2**^{1}, 2x**2**^{2}) **i**

	1	2	3	4	5	6
1	2	1	0	0	0	0
2	1	0	0	0	0	1
3	0	0	0	2^1	0	0
4	0	0	2^1	0	1	0
5	0	0	0	1	0	2^2
6	0	1	0	0	2^2	0

(6x**1**, 1x**2**, 2x**2**^{1}, 2x**2**^{2}) **j**

	1	2	3	4	5	6
1	2	0	0	0	0	0
2	0	0	0	0	0	2^1
3	0	0	0	2^2	1	0
4	0	0	2^2	0	1	0
5	0	0	1	1	0	1
6	0	2^1	0	0	1	0

(6x**1**, 1x**2**, 2x**2**^{1}, 2x**2**^{2}) **k**

	1	2	3	4	5	6
1	2	0	0	0	0	0
2	0	0	0	0	0	2^2
3	0	0	0	2^1	1	0
4	0	0	2^1	0	1	0
5	0	0	1	1	0	1
6	0	2^2	0	0	1	0

(6x**1**, 1x**2**, 2x**2**^{1}, 2x**2**^{2}) **l**

	1	2	3	4	5	6
1	2	0	0	0	0	0
2	0	0	0	0	1	2^1
3	0	0	0	2^2	0	1
4	0	0	2^2	0	1	0
5	0	1	0	1	0	0
6	0	2^1	1	0	0	0

(6x**1**, 1x**2**, 4x**2**^{2}) **a**

	1	2	3	4	5	6
1	2	1	0	0	0	0
2	1	0	0	0	0	2^2
3	0	0	0	2^2	0	0
4	0	0	2^2	0	1	0
5	0	0	0	1	0	1
6	0	2^2	0	0	1	0

(6x**1**, 1x**2**, 4x**2**^{2}) **b**

	1	2	3	4	5	6
1	2	1	0	0	0	0
2	1	0	0	0	0	2^2
3	0	0	0	2^2	1	0
4	0	0	2^2	0	1	0
5	0	0	1	1	0	0
6	0	2^2	0	0	0	0

(6x**1**, 1x**2**, 4x**2**^{2}) **c**

	1	2	3	4	5	6
1	2	0	0	0	1	0
2	0	0	0	0	0	2^2
3	0	0	0	2^2	1	0
4	0	0	2^2	0	1	0
5	1	0	1	1	0	0
6	0	2^2	0	0	0	0

(6x**1**, 1x**2**, 4x**2**^{2}) **d**

	1	2	3	4	5	6
1	2	0	0	0	1	0
2	0	0	0	0	0	2^2
3	0	0	0	2^2	0	0
4	0	0	2^2	0	1	0
5	1	0	0	1	0	1
6	0	2^2	0	0	1	0

(6x**1**, 1x**2**, 4x**2**^{2}) **e**

	1	2	3	4	5	6
1	2	1	0	0	0	0
2	1	0	0	0	0	1
3	0	0	0	2^2	0	0
4	0	0	2^2	0	1	0
5	0	0	0	1	0	2^2
6	0	1	0	0	2^2	0

(6x**1**, 1x**2**, 4x**2**^{2}) **f**

	1	2	3	4	5	6
1	2	0	0	0	0	0
2	0	0	0	0	0	2^2
3	0	0	0	2^2	1	0
4	0	0	2^2	0	1	0
5	0	0	1	1	0	1
6	0	2^2	0	0	1	0

(6x**1**, 1x**2**, 4x**2**^{2}) **g**

	1	2	3	4	5	6
1	2	0	0	0	0	0
2	0	0	0	0	1	2^2
3	0	0	0	2^2	0	1
4	0	0	2^2	0	1	0
5	0	1	0	1	0	0
6	0	2^2	1	0	0	0

(4x**1**, 4x**2**, 2x**2**^{1}) **a**

	1	2	3	4	5	6
1	2	1	0	0	0	0
2	1	2	0	0	0	0
3	0	0	0	2^1	0	0
4	0	0	2^1	0	0	0
5	0	0	0	0	2	1
6	0	0	0	0	1	2

(4x**1**, 4x**2**, 2x**2**^{1}) **b**

	1	2	3	4	5	6
1	2	0	0	0	0	0
2	0	2	1	0	0	0
3	0	1	0	2^1	0	0
4	0	0	2^1	0	0	0
5	0	0	0	0	2	1
6	0	0	0	0	1	2

(4x**1**, 4x**2**, 2x**2**^{1}) **c**

	1	2	3	4	5	6
1	2	0	0	1	0	0
2	0	2	1	0	0	0
3	0	1	0	2^1	0	0
4	1	0	2^1	0	0	0
5	0	0	0	0	2	0
6	0	0	0	0	0	2

(4x**1**, 4x**2**, 2x**2**^{2}) **a**

	1	2	3	4	5	6
1	2	1	0	0	0	0
2	1	2	0	0	0	0
3	0	0	0	2^2	0	0
4	0	0	2^2	0	0	0
5	0	0	0	0	2	1
6	0	0	0	0	1	2

(4x**1**, 4x**2**, 2x**2**^{2}) **b**

	1	2	3	4	5	6
1	2	0	0	0	0	0
2	0	2	1	0	0	0
3	0	1	0	2^2	0	0
4	0	0	2^2	0	0	0
5	0	0	0	0	2	1
6	0	0	0	0	1	2

(4x**1**, 4x**2**, 2x**2**^{2}) **c**

	1	2	3	4	5	6
1	2	0	0	1	0	0
2	0	2	1	0	0	0
3	0	1	0	2^2	0	0
4	1	0	2^2	0	0	0
5	0	0	0	0	2	0
6	0	0	0	0	0	2

(4x**1**, 2x**2**, 4x**2**^{1}) **a**

	1	2	3	4	5	6
1	0	2^1	0	1	0	0
2	2^1	0	0	0	0	0
3	0	0	0	2^1	0	0
4	1	0	2^1	0	0	0
5	0	0	0	0	2	1
6	0	0	0	0	1	2

(4x**1**, 2x**2**, 4x**2**^{1}) **b**

	1	2	3	4	5	6
1	0	2^1	0	0	1	0
2	2^1	0	0	0	0	1
3	0	0	0	2^1	0	0
4	0	0	2^1	0	0	0
5	1	0	0	0	2	0
6	0	1	0	0	0	2

(4x**1**, 2x**2**, 4x**2**^{1}) **c**

	1	2	3	4	5	6
1	0	2^1	0	1	0	0
2	2^1	0	0	0	0	1
3	0	0	0	2^1	0	0
4	1	0	2^1	0	0	0
5	0	0	0	0	2	0
6	0	1	0	0	0	2

(4x**1**, 2x**2**, 4x**2**^{1}) **d**

	1	2	3	4	5	6
1	0	2^1	0	0	0	0
2	2^1	0	0	0	0	1
3	0	0	0	2^1	1	0
4	0	0	2^1	0	0	0
5	0	0	1	0	2	0
6	0	1	0	0	0	2

(4x**1**, 2x**2**, 4x**2**^{1}) **e**

	1	2	3	4	5	6
1	0	2^1	0	1	0	0
2	2^1	0	1	0	0	0
3	0	1	0	2^1	0	0
4	1	0	2^1	0	0	0
5	0	0	0	0	2	0
6	0	0	0	0	0	2

(4x**1**, 2x**2**, 2x**2**^{1}, 2x**2**^{2}) **a**

	1	2	3	4	5	6
1	0	2^1	0	1	0	0
2	2^1	0	0	0	0	0
3	0	0	0	2^2	0	0
4	1	0	2^2	0	0	0
5	0	0	0	0	2	1
6	0	0	0	0	1	2

(4x**1**, 2x**2**, 2x**2**^{1}, 2x**2**^{2}) **b**

	1	2	3	4	5	6
1	0	2^1	0	0	1	0
2	2^1	0	0	0	0	1
3	0	0	0	2^2	0	0
4	0	0	2^2	0	0	0
5	1	0	0	0	2	0
6	0	1	0	0	0	2

(4x**1**, 2x**2**, 2x**2**^{1}, 2x**2**^{2}) **c**

	1	2	3	4	5	6
1	0	2^2	0	0	1	0
2	2^2	0	0	0	0	1
3	0	0	0	2^1	0	0
4	0	0	2^1	0	0	0
5	1	0	0	0	2	0
6	0	1	0	0	0	2

(4x**1**, 2x**2**, 2x**2**^{1}, 2x**2**^{2}) **d**

	1	2	3	4	5	6
1	0	2^1	0	1	0	0
2	2^1	0	0	0	0	1
3	0	0	0	2^2	0	0
4	1	0	2^2	0	0	0
5	0	0	0	0	2	0
6	0	1	0	0	0	2

(4x**1**, 2x**2**, 2x**2**^{1}, 2x**2**^{2}) **e**

	1	2	3	4	5	6
1	0	2^2	0	1	0	0
2	2^2	0	0	0	0	1
3	0	0	0	2^1	0	0
4	1	0	2^1	0	0	0
5	0	0	0	0	2	0
6	0	1	0	0	0	2

(4x**1**, 2x**2**, 2x**2**^{1}, 2x**2**^{2}) **f**

	1	2	3	4	5	6
1	0	2^2	0	0	0	0
2	2^2	0	0	0	0	1
3	0	0	0	2^1	1	0
4	0	0	2^1	0	0	0
5	0	0	1	0	2	0
6	0	1	0	0	0	2

(4x**1**, 2x**2**, 2x**2**^{1}, 2x**2**^{2}) **g**

	1	2	3	4	5	6
1	0	2^1	0	1	0	0
2	2^1	0	1	0	0	0
3	0	1	0	2^2	0	0
4	1	0	2^2	0	0	0
5	0	0	0	0	2	0
6	0	0	0	0	0	2

(4x**1**, 2x**2**, 4x**2**^{2}) **a**

	1	2	3	4	5	6
1	0	2^2	0	1	0	0
2	2^2	0	0	0	0	0
3	0	0	0	2^2	0	0
4	1	0	2^2	0	0	0
5	0	0	0	0	2	1
6	0	0	0	0	1	2

(4x**1**, 2x**2**, 2x**2**^{1}, 2x**2**^{2}) **b**

	1	2	3	4	5	6
1	0	2^2	0	0	1	0
2	2^2	0	0	0	0	1
3	0	0	0	2^2	0	0
4	0	0	2^2	0	0	0
5	1	0	0	0	2	0
6	0	1	0	0	0	2

(4x**1**, 2x**2**, 4x**2**^{2}) **c**

	1	2	3	4	5	6
1	0	2^2	0	1	0	0
2	2^2	0	0	0	0	1
3	0	0	0	2^2	0	0
4	1	0	2^2	0	0	0
5	0	0	0	0	2	0
6	0	1	0	0	0	2

(4x**1**, 2x**2**, 4x**2**^{2}) **d**

	1	2	3	4	5	6
1	0	2^2	0	0	0	0
2	2^2	0	0	0	0	1
3	0	0	0	2^2	1	0
4	0	0	2^2	0	0	0
5	0	0	1	0	2	0
6	0	1	0	0	0	2

(4x**1**, 2x**2**, 4x**2**^{2}) **e**

	1	2	3	4	5	6
1	0	2^2	0	1	0	0
2	2^2	0	1	0	0	0
3	0	1	0	2^2	0	0
4	1	0	2^2	0	0	0
5	0	0	0	0	2	0
6	0	0	0	0	0	2

(4x**1**, 6x**2**^{1}) **a**

	1	2	3	4	5	6
1	0	2^1	0	1	0	0
2	2^1	0	1	0	0	0
3	0	1	0	2^1	0	0
4	1	0	2^1	0	0	0
5	0	0	0	0	0	2^1
6	0	0	0	0	2^1	0

(4x**1**, 6x**2**^{1}) **b**

	1	2	3	4	5	6
1	0	2^1	0	0	0	0
2	2^1	0	1	0	0	0
3	0	1	0	2^1	0	0
4	0	0	2^1	0	1	0
5	0	0	0	1	0	2^1
6	0	0	0	0	2^1	0

(4x**1**, 4x**2**^{1}, 2x**2**^{2}) **a**

	1	2	3	4	5	6
1	0	2^1	0	1	0	0
2	2^1	0	1	0	0	0
3	0	1	0	2^1	0	0
4	1	0	2^1	0	0	0
5	0	0	0	0	0	2^2
6	0	0	0	0	2^2	0

(4x**1**, 4x**2**^{1}, 2x**2**^{2}) **b**

	1	2	3	4	5	6
1	0	2^1	0	1	0	0
2	2^1	0	1	0	0	0
3	0	1	0	2^2	0	0
4	1	0	2^2	0	0	0
5	0	0	0	0	0	2^1
6	0	0	0	0	2^1	0

(4x**1**, 4x**2**^{1}, 2x**2**^{2}) **c**

	1	2	3	4	5	6
1	0	2^1	0	0	0	0
2	2^1	0	1	0	0	0
3	0	1	0	2^1	0	0
4	0	0	2^1	0	1	0
5	0	0	0	1	0	2^2
6	0	0	0	0	2^2	0

(4x**1**, 4x**2**^{1}, 2x**2**^{2}) **d**

	1	2	3	4	5	6
1	0	2^1	0	0	0	0
2	2^1	0	1	0	0	0
3	0	1	0	2^2	0	0
4	0	0	2^2	0	1	0
5	0	0	0	1	0	2^1
6	0	0	0	0	2^1	0

(4x**1**, 2x**2**^{1}, 4x**2**^{2}) **a**

	1	2	3	4	5	6
1	0	2^1	0	1	0	0
2	2^1	0	1	0	0	0
3	0	1	0	2^2	0	0
4	1	0	2^2	0	0	0
5	0	0	0	0	0	2^2
6	0	0	0	0	2^2	0

(4x**1**, 2x**2**^{1}, 4x**2**^{2}) **b**

	1	2	3	4	5	6
1	0	2^2	0	1	0	0
2	2^2	0	1	0	0	0
3	0	1	0	2^2	0	0
4	1	0	2^2	0	0	0
5	0	0	0	0	0	2^1
6	0	0	0	0	2^1	0

(4x**1**, 2x**2**^{1}, 4x**2**^{2}) **c**

	1	2	3	4	5	6
1	0	2^2	0	0	0	0
2	2^2	0	1	0	0	0
3	0	1	0	2^1	0	0
4	0	0	2^1	0	1	0
5	0	0	0	1	0	2^2
6	0	0	0	0	2^2	0

(4x**1**, 2x**2**^{1}, 4x**2**^{2}) **d**

	1	2	3	4	5	6
1	0	2^1	0	0	0	0
2	2^1	0	1	0	0	0
3	0	1	0	2^2	0	0
4	0	0	2^2	0	1	0
5	0	0	0	1	0	2^2
6	0	0	0	0	2^2	0

(4x**1**, 6x**2**^{2}) **a**

	1	2	3	4	5	6
1	0	2^2	0	1	0	0
2	2^2	0	1	0	0	0
3	0	1	0	2^2	0	0
4	1	0	2^2	0	0	0
5	0	0	0	0	0	2^2
6	0	0	0	0	2^2	0

(4x**1**, 6x**2**^{2}) **b**

	1	2	3	4	5	6
1	0	2^2	0	0	0	0
2	2^2	0	1	0	0	0
3	0	1	0	2^2	0	0
4	0	0	2^2	0	1	0
5	0	0	0	1	0	2^2
6	0	0	0	0	2^2	0

$^2V_2{}^3V_6$ NG

$^2V_2{}^3V_8$ NG

$^2V_3{}^3V_2$

(12x**1**) **a**

	1	2	3	4	5
1	0	0	1	1	0
2	0	0	1	1	0
3	1	1	0	0	1
4	1	1	0	0	1
5	0	0	1	1	0

(12x**1**) **b**

	1	2	3	4	5
1	0	1	0	1	0
2	1	0	1	0	0
3	0	1	0	1	1
4	1	0	1	0	1
5	0	0	1	1	0

(10x**1**, 1x**2**) **a**

	1	2	3	4	5
1	0	1	0	1	0
2	1	0	1	1	0
3	0	1	0	1	0
4	1	1	1	0	0
5	0	0	0	0	2

(10x**1**, 1x**2**) **b**

	1	2	3	4	5
1	0	1	0	1	0
2	1	0	0	1	0
3	0	0	0	1	1
4	1	1	1	0	0
5	0	0	1	0	2

(10x**1**, 1x**2**) **c**

	1	2	3	4	5
1	0	1	0	1	0
2	1	0	1	0	0
3	0	1	0	1	1
4	1	0	1	0	0
5	0	0	1	0	2

(8x**1**, 2x**2**) **a**

	1	2	3	4	5
1	0	1	0	1	0
2	1	2	0	0	0
3	0	0	0	1	1
4	1	0	1	0	0
5	0	0	1	0	2

(8x**1**, 2x**2**) **b**

	1	2	3	4	5
1	0	1	1	0	0
2	1	0	1	0	0
3	1	1	0	0	0
4	0	0	0	2	1
5	0	0	0	1	2

(8x**1**, 2x**2**) **c**

	1	2	3	4	5
1	0	0	1	1	0
2	0	2	0	0	0
3	1	0	0	1	1
4	1	0	1	0	0
5	0	0	1	0	2

(8x**1**, 2x**2**^{1}) **a**

	1	2	3	4	5
1	0	0	1	1	0
2	0	0	1	0	2^1
3	1	1	0	1	0
4	1	0	1	0	0
5	0	2^1	0	0	0

(8x**1**, 2x**2**^{1}) **b**

	1	2	3	4	5
1	0	0	1	1	0
2	0	0	1	0	2^1
3	1	1	0	0	0
4	1	0	0	0	1
5	0	2^1	0	1	0

(8x**1**, 2x**2**^{2}) **a**

	1	2	3	4	5
1	0	0	1	1	0
2	0	0	1	0	2^2
3	1	1	0	1	0
4	1	0	1	0	0
5	0	2^2	0	0	0

(8x**1**, 2x**2**^{2}) **b**

	1	2	3	4	5
1	0	0	1	1	0
2	0	0	1	0	2^2
3	1	1	0	0	0
4	1	0	0	0	1
5	0	2^2	0	1	0

(6x**1**, 3x**2**)

	1	2	3	4	5
1	0	1	0	1	0
2	1	2	0	0	0
3	0	0	2	1	0
4	1	0	1	0	0
5	0	0	0	0	2

(6x**1**, 1x**2**, 2x**2**^{1}) **a**

	1	2	3	4	5
1	2	0	0	0	0
2	0	0	1	0	2^1
3	0	1	0	1	0
4	0	0	1	0	1
5	0	2^1	0	1	0

(6x**1**, 1x**2**, 2x**2**^{1}) **b**

	1	2	3	4	5
1	2	0	0	1	0
2	0	0	1	0	2^1
3	0	1	0	1	0
4	1	0	1	0	0
5	0	2^1	0	0	0

(6x**1**, 1x**2**, 2x**2**^{2}) **a**

	1	2	3	4	5
1	2	0	0	0	0
2	0	0	1	0	2^2
3	0	1	0	1	0
4	0	0	1	0	1
5	0	2^2	0	1	0

(6x**1**, 1x**2**, 2x**2**^{2}) **b**

	1	2	3	4	5
1	2	0	0	1	0
2	0	0	1	0	2^2
3	0	1	0	1	0
4	1	0	1	0	0
5	0	2^2	0	0	0

(4x**1**, 4x**2**)

	1	2	3	4	5
1	2	0	0	1	0
2	0	2	0	0	0
3	0	0	2	1	0
4	1	0	1	0	0
5	0	0	0	0	2

(4x**1**, 2x**2**, 2x**2**^{1}) **a**

	1	2	3	4	5
1	2	0	0	0	0
2	0	0	1	0	2^1
3	0	1	0	0	1
4	0	0	0	2	0
5	0	2^1	1	0	0

(4x**1**, 2x**2**, 2x**2**^{1}) **b**

	1	2	3	4	5
1	2	0	0	1	0
2	0	0	0	0	2^1
3	0	0	2	1	0
4	1	0	1	0	0
5	0	2^1	0	0	0

(4x**1**, 2x**2**, 2x**2**^{1}) **c**

	1	2	3	4	5
1	0	0	0	1	1
2	0	0	0	0	2^1
3	0	0	2	0	0
4	1	0	0	2	0
5	1	2^1	0	0	0

(4x**1**, 2x**2**, 2x**2**^{2}) **a**

	1	2	3	4	5
1	2	0	0	0	0
2	0	0	1	0	2^2
3	0	1	0	0	1
4	0	0	0	2	0
5	0	2^2	1	0	0

(4x**1**, 2x**2**, 2x**2**^{2}) **b**

	1	2	3	4	5
1	2	0	0	1	0
2	0	0	0	0	2^2
3	0	0	2	1	0
4	1	0	1	0	0
5	0	2^2	0	0	0

(4x**1**, 2x**2**, 2x**2**^{2}) **c**

	1	2	3	4	5
1	0	0	0	1	1
2	0	0	0	0	2^2
3	0	0	2	0	0
4	1	0	0	2	0
5	1	2^2	0	0	0

(4x**1**, 4x**2**^{1}) **a**

	1	2	3	4	5
1	0	0	1	2^1	0
2	0	0	1	0	2^1
3	1	1	0	0	0
4	2^1	0	0	0	0
5	0	2^1	0	0	0

(4x**1**, 4x**2**^{1}) **b**

	1	2	3	4	5
1	0	0	0	2^1	0
2	0	0	1	0	2^1
3	0	1	0	0	1
4	2^1	0	0	0	0
5	0	2^1	1	0	0

(4x**1**, 2x**2**^{1}, 2x**2**^{2}) **a**

	1	2	3	4	5
1	0	0	1	2^1	0
2	0	0	1	0	2^2
3	1	1	0	0	0
4	2^1	0	0	0	0
5	0	2^2	0	0	0

(4x**1**, 2x**2**^{1}, 2x**2**^{2}) **b**

	1	2	3	4	5
1	0	0	0	2^1	0
2	0	0	1	0	2^2
3	0	1	0	0	1
4	2^1	0	0	0	0
5	0	2^2	1	0	0

(4x**1**, 2x**2**^{1}, 2x**2**^{2}) **c**

	1	2	3	4	5
1	0	0	0	2^2	0
2	0	0	1	0	2^1
3	0	1	0	0	1
4	2^2	0	0	0	0
5	0	2^1	1	0	0

(4x**1**, 4x**2**^{2}) **a**

	1	2	3	4	5
1	0	0	1	2^2	0
2	0	0	1	0	2^2
3	1	1	0	0	0
4	2^2	0	0	0	0
5	0	2^2	0	0	0

(4x**1**, 4x**2**^{2}) **b**

	1	2	3	4	5
1	0	0	0	2^2	0
2	0	0	1	0	2^2
3	0	1	0	0	1
4	2^2	0	0	0	0
5	0	2^2	1	0	0

(2x**1**, 5x**2**)

	1	2	3	4	5
1	2	0	0	1	0
2	0	2	0	0	0
3	0	0	2	0	0
4	1	0	0	2	0
5	0	0	0	0	2

(2x**1**, 3x**2**, 2x**2**^{1}) **a**

	1	2	3	4	5
1	2	0	0	0	0
2	0	0	1	0	2^1
3	0	1	2	0	0
4	0	0	0	2	0
5	0	2^1	0	0	0

(2x**1**, 3x**2**, 2x**2**^{1}) **b**

	1	2	3	4	5
1	2	0	1	0	0
2	0	0	0	0	2^1
3	1	0	2	0	0
4	0	0	0	2	0
5	0	2^1	0	0	0

(2x**1**, 3x**2**, 2x**2**^{2}) **a**

	1	2	3	4	5
1	2	0	0	0	0
2	0	0	1	0	2^2
3	0	1	2	0	0
4	0	0	0	2	0
5	0	2^2	0	0	0

(2x**1**, 3x**2**, 2x**2**^{2}) **b**

	1	2	3	4	5
1	2	0	1	0	0
2	0	0	0	0	2^2
3	1	0	2	0	0
4	0	0	0	2	0
5	0	2^2	0	0	0

(2x**1**, 1x**2**, 4x**2**^{1}) **a**

	1	2	3	4	5
1	0	0	1	2^1	0
2	0	0	2^1	0	0
3	1	2^1	0	0	0
4	2^1	0	0	0	0
5	0	0	0	0	2

(2x**1**, 1x**2**, 4x**2**^{1}) **b**

	1	2	3	4	5
1	0	0	0	2^1	0
2	0	0	2^1	0	0
3	0	2^1	0	0	0
4	2^1	0	0	0	1
5	0	0	0	1	2

(2x**1**, 1x**2**, 2x**2**^{1}, 2x**2**^{2}) **a**

	1	2	3	4	5
1	0	0	1	2^1	0
2	0	0	2^2	0	0
3	1	2^2	0	0	0
4	2^1	0	0	0	0
5	0	0	0	0	2

(2x**1**, 1x**2**, 2x**2**^{1}, 2x**2**^{2}) **b**

	1	2	3	4	5
1	0	0	0	2^1	0
2	0	0	2^2	0	0
3	0	2^2	0	0	0
4	2^1	0	0	0	1
5	0	0	0	1	2

(2x**1**, 1x**2**, 2x**2**^{1}, 2x**2**^{2}) **c**

	1	2	3	4	5
1	0	0	0	2^2	0
2	0	0	2^1	0	0
3	0	2^1	0	0	0
4	2^2	0	0	0	1
5	0	0	0	1	2

(2x**1**, 1x**2**, 4x**2**^{2}) **a**

	1	2	3	4	5
1	0	0	1	2^2	0
2	0	0	2^2	0	0
3	1	2^2	0	0	0
4	2^2	0	0	0	0
5	0	0	0	0	2

(2x**1**, 1x**2**, 4x**2**^{2}) **b**

	1	2	3	4	5
1	0	0	0	2^2	0
2	0	0	2^2	0	0
3	0	2^2	0	0	0
4	2^2	0	0	0	1
5	0	0	0	1	2

$^{2}V_3{}^{3}V_4$

NG

$^{2}V_4{}^{3}V_2$

(14x**1**) **a**

	1	2	3	4	5	6
1	0	0	1	1	0	0
2	0	0	1	1	0	0
3	1	1	0	0	0	1
4	1	1	0	0	1	0
5	0	0	0	1	0	1
6	0	0	1	0	1	0

(14x**1**) **b**

	1	2	3	4	5	6
1	0	1	0	1	0	0
2	1	0	1	0	0	0
3	0	1	0	1	0	1
4	1	0	1	0	1	0
5	0	0	0	1	0	1
6	0	0	1	0	1	0

(14x**1**) **c**

	1	2	3	4	5	6
1	0	1	1	0	0	0
2	1	0	1	0	0	0
3	1	1	0	1	0	0
4	0	0	1	0	1	1
5	0	0	0	1	0	1
6	0	0	0	1	1	0

(14x**1**) **d**

	1	2	3	4	5	6
1	0	1	1	1	0	0
2	1	0	1	0	0	0
3	1	1	0	0	0	1
4	1	0	0	0	1	0
5	0	0	0	1	0	1
6	0	0	1	0	1	0

(12x**1**, 1x**2**) **a**

	1	2	3	4	5	6
1	0	0	1	1	0	0
2	0	0	1	1	0	0
3	1	1	0	0	1	0
4	1	1	0	0	1	0
5	0	0	1	1	0	0
6	0	0	0	0	0	2

(12x**1**, 1x**2**) **b**

	1	2	3	4	5	6
1	0	1	0	1	0	0
2	1	0	1	0	0	0
3	0	1	0	1	1	0
4	1	0	1	0	1	0
5	0	0	1	1	0	0
6	0	0	0	0	0	2

(12x**1**, 1x**2**) **c**

	1	2	3	4	5	6
1	0	1	0	1	0	0
2	1	0	1	0	0	0
3	0	1	0	1	0	0
4	1	0	1	0	1	0
5	0	0	0	1	0	1
6	0	0	0	0	1	2

(12x**1**, 1x**2**) **d**

	1	2	3	4	5	6
1	0	1	1	1	0	0
2	1	0	1	0	0	0
3	1	1	0	0	0	0
4	1	0	0	0	1	0
5	0	0	0	1	0	1
6	0	0	0	0	1	2

(12x**1**, 1x**2**) **e**

	1	2	3	4	5	6
1	0	1	0	1	0	0
2	1	0	1	0	0	0
3	0	1	0	0	1	0
4	1	0	0	0	1	0
5	0	0	1	1	0	1
6	0	0	0	0	1	2

(10x**1**, 2x**2**) **a**

	1	2	3	4	5	6
1	0	1	0	1	0	0
2	1	0	1	1	0	0
3	0	1	0	1	0	0
4	1	1	1	0	0	0
5	0	0	0	0	2	0
6	0	0	0	0	0	2

(10x**1**, 2x**2**) **b**

	1	2	3	4	5	6
1	2	1	0	0	0	0
2	1	0	1	0	0	0
3	0	1	0	1	0	0
4	0	0	1	0	1	0
5	0	0	0	1	0	1
6	0	0	0	0	1	2

(10x**1**, 2x**2**) **c**

	1	2	3	4	5	6
1	2	0	0	0	0	0
2	0	0	1	1	0	0
3	0	1	0	1	0	0
4	0	1	1	0	1	0
5	0	0	0	1	0	1
6	0	0	0	0	1	2

(10x**1**, 2x**2**) **d**

	1	2	3	4	5	6
1	0	1	0	1	0	0
2	1	0	1	0	0	0
3	0	1	0	1	0	0
4	1	0	1	0	1	0
5	0	0	0	1	2	0
6	0	0	0	0	0	2

(10x**1**, 2x**2**) **e**

	1	2	3	4	5	6
1	0	1	1	0	0	0
2	1	0	1	0	0	0
3	1	1	0	0	0	0
4	0	0	0	2	1	0
5	0	0	0	1	0	1
6	0	0	0	0	1	2

(10x**1**, 2x**2**) **f**

	1	2	3	4	5	6
1	0	1	0	1	0	0
2	1	0	1	0	0	0
3	0	1	0	1	0	0
4	1	0	1	0	0	0
5	0	0	0	0	2	1
6	0	0	0	0	1	2

(10x**1**, 2x$\mathbf{2}^{1}$) **a**

	1	2	3	4	5	6
1	0	1	0	1	0	0
2	1	0	1	1	0	0
3	0	1	0	1	0	0
4	1	1	1	0	0	0
5	0	0	0	0	0	2^1
6	0	0	0	0	2^1	0

(10x**1**, 2x$\mathbf{2}^{1}$) **b**

	1	2	3	4	5	6
1	0	1	0	1	0	0
2	1	0	1	1	0	0
3	0	1	0	0	0	1
4	1	1	0	0	0	0
5	0	0	0	0	0	2^1
6	0	0	1	0	2^1	0

(10x**1**, 2x$\mathbf{2}^{1}$) **c**

	1	2	3	4	5	6
1	0	1	0	1	0	0
2	1	0	1	0	0	0
3	0	1	0	1	0	1
4	1	0	1	0	0	0
5	0	0	0	0	0	2^1
6	0	0	1	0	2^1	0

(10x**1**, 2x$\mathbf{2}^{1}$) **d**

	1	2	3	4	5	6
1	0	1	0	1	0	0
2	1	0	1	0	0	0
3	0	1	0	0	0	1
4	1	0	0	0	1	0
5	0	0	0	1	0	2^1
6	0	0	1	0	2^1	0

(10x**1**, 2x$\mathbf{2}^{1}$) **e**

	1	2	3	4	5	6
1	0	1	0	1	0	0
2	1	0	0	1	0	0
3	0	0	0	0	1	1
4	1	1	0	0	0	0
5	0	0	1	0	0	2^1
6	0	0	1	0	2^1	0

(10x**1**, 2x$\mathbf{2}^{2}$) **a**

	1	2	3	4	5	6
1	0	1	0	1	0	0
2	1	0	1	1	0	0
3	0	1	0	1	0	0
4	1	1	1	0	0	0
5	0	0	0	0	0	2^2
6	0	0	0	0	2^2	0

(10x**1**, 2x$\mathbf{2}^{2}$) **b**

	1	2	3	4	5	6
1	0	1	0	1	0	0
2	1	0	1	1	0	0
3	0	1	0	0	0	1
4	1	1	0	0	0	0
5	0	0	0	0	0	2^2
6	0	0	1	0	2^2	0

(10x**1**, 2x$\mathbf{2}^{2}$) **c**

	1	2	3	4	5	6
1	0	1	0	1	0	0
2	1	0	1	0	0	0
3	0	1	0	1	0	1
4	1	0	1	0	0	0
5	0	0	0	0	0	2^2
6	0	0	1	0	2^2	0

(10x**1**, 2x$\mathbf{2}^{2}$) **d**

	1	2	3	4	5	6
1	0	1	0	1	0	0
2	1	0	1	0	0	0
3	0	1	0	0	0	1
4	1	0	0	0	1	0
5	0	0	0	1	0	2^2
6	0	0	1	0	2^2	0

(10x**1**, 2x$\mathbf{2}^{2}$) **e**

	1	2	3	4	5	6
1	0	1	0	1	0	0
2	1	0	0	1	0	0
3	0	0	0	0	1	1
4	1	1	0	0	0	0
5	0	0	1	0	0	2^2
6	0	0	1	0	2^2	0

(8x**1**, 3x**2**) **a**

	1	2	3	4	5	6
1	2	0	0	0	0	0
2	0	2	1	0	0	0
3	0	1	0	1	0	0
4	0	0	1	0	1	0
5	0	0	0	1	0	1
6	0	0	0	0	1	2

(8x**1**, 3x**2**) **b**

	1	2	3	4	5	6
1	2	0	0	0	0	0
2	0	2	0	0	0	0
3	0	0	0	1	1	0
4	0	0	1	0	1	0
5	0	0	1	1	0	1
6	0	0	0	0	1	2

(8x**1**, 3x**2**) **c**

	1	2	3	4	5	6
1	2	1	0	0	0	0
2	1	2	0	0	0	0
3	0	0	0	1	1	0
4	0	0	1	0	1	0
5	0	0	1	1	0	0
6	0	0	0	0	0	2

(8x**1**, 1x**2**, 2x$\mathbf{2}^{1}$) **a**

	1	2	3	4	5	6
1	2	0	0	0	0	0
2	0	0	1	1	0	0
3	0	1	0	1	0	1
4	0	1	1	0	0	0
5	0	0	0	0	0	2^1
6	0	0	1	0	2^1	0

(8x**1**, 1x**2**, 2x$\mathbf{2}^{1}$) **b**

	1	2	3	4	5	6
1	2	0	0	0	0	0
2	0	0	1	1	0	0
3	0	1	0	0	0	1
4	0	1	0	0	1	0
5	0	0	0	1	0	2^1
6	0	0	1	0	2^1	0

(8x**1**, 1x**2**, 2x$\mathbf{2}^{1}$) **c**

	1	2	3	4	5	6
1	0	1	0	1	0	0
2	1	2	0	0	0	0
3	0	0	0	1	0	1
4	1	0	1	0	0	0
5	0	0	0	0	0	2^1
6	0	0	1	0	2^1	0

(8x**1**, 1x**2**, 2x$\mathbf{2}^{1}$) **d**

	1	2	3	4	5	6
1	0	1	1	1	0	0
2	1	2	0	0	0	0
3	1	0	0	1	0	0
4	1	0	1	0	0	0
5	0	0	0	0	0	2^1
6	0	0	0	0	2^1	0

(8x**1**, 1x**2**, 2x$\mathbf{2}^{1}$) **e**

	1	2	3	4	5	6
1	0	1	0	1	0	0
2	1	0	0	1	0	0
3	0	0	2	0	0	1
4	1	1	0	0	0	0
5	0	0	0	0	0	2^1
6	0	0	1	0	2^1	0

(8x**1**, 1x**2**, 2x$\mathbf{2}^{2}$) **a**

	1	2	3	4	5	6
1	2	0	0	0	0	0
2	0	0	1	1	0	0
3	0	1	0	1	0	1
4	0	1	1	0	0	0
5	0	0	0	0	0	2^2
6	0	0	1	0	2^2	0

(8x**1**, 1x**2**, 2x$\mathbf{2}^{2}$) **b**

	1	2	3	4	5	6
1	2	0	0	0	0	0
2	0	0	1	1	0	0
3	0	1	0	0	0	1
4	0	1	0	0	1	0
5	0	0	0	1	0	2^2
6	0	0	1	0	2^2	0

(8x**1**, 1x**2**, 2x$\mathbf{2}^{2}$) **c**

	1	2	3	4	5	6
1	0	1	0	1	0	0
2	1	2	0	0	0	0
3	0	0	0	1	0	1
4	1	0	1	0	0	0
5	0	0	0	0	0	2^2
6	0	0	1	0	2^2	0

(8x**1**, 1x**2**, 2x$\mathbf{2}^{2}$) **d**

	1	2	3	4	5	6
1	0	1	1	1	0	0
2	1	2	0	0	0	0
3	1	0	0	1	0	0
4	1	0	1	0	0	0
5	0	0	0	0	0	2^2
6	0	0	0	0	2^2	0

(8x**1**, 1x**2**, 2x$\mathbf{2}^{2}$) **e**

	1	2	3	4	5	6
1	0	1	0	1	0	0
2	1	0	0	1	0	0
3	0	0	2	0	0	1
4	1	1	0	0	0	0
5	0	0	0	0	0	2^2
6	0	0	1	0	2^2	0

(6x**1**, 4x**2**)

	1	2	3	4	5	6
1	2	0	0	0	0	0
2	0	2	0	0	0	0
3	0	0	2	1	0	0
4	0	0	1	0	1	0
5	0	0	0	1	0	1
6	0	0	0	0	1	2

(6x**1**, 2x**2**, 2x$\mathbf{2}^{1}$) **a**

	1	2	3	4	5	6
1	2	0	0	0	0	0
2	0	2	0	0	0	0
3	0	0	0	1	0	1
4	0	0	1	0	1	0
5	0	0	0	1	0	2^1
6	0	0	1	0	2^1	0

(6x**1**, 2x**2**, 2x$\mathbf{2}^{1}$) **b**

	1	2	3	4	5	6
1	2	0	0	1	0	0
2	0	2	0	0	0	0
3	0	0	0	1	0	1
4	1	0	1	0	0	0
5	0	0	0	0	0	2^1
6	0	0	1	0	2^1	0

(6x**1**, 2x**2**, 2x$\mathbf{2}^{1}$) **c**

	1	2	3	4	5	6
1	2	0	0	1	0	0
2	0	2	1	0	0	0
3	0	1	0	1	0	0
4	1	0	1	0	0	0
5	0	0	0	0	0	2^1
6	0	0	0	0	2^1	0

(6x**1**, 2x**2**, 2x$\mathbf{2}^{2}$) **a**

	1	2	3	4	5	6
1	2	0	0	0	0	0
2	0	2	0	0	0	0
3	0	0	0	1	0	1
4	0	0	1	0	1	0
5	0	0	0	1	0	2^2
6	0	0	1	0	2^2	0

(6x**1**, 2x**2**, 2x$\mathbf{2}^{2}$) **b**

	1	2	3	4	5	6
1	2	0	0	1	0	0
2	0	2	0	0	0	0
3	0	0	0	1	0	1
4	1	0	1	0	0	0
5	0	0	0	0	0	2^2
6	0	0	1	0	2^2	0

(6x**1**, 2x**2**, 2x$\mathbf{2}^{2}$) **c**

	1	2	3	4	5	6
1	2	0	0	1	0	0
2	0	2	1	0	0	0
3	0	1	0	1	0	0
4	1	0	1	0	0	0
5	0	0	0	0	0	2^2
6	0	0	0	0	2^2	0

(6x**1**, 4x**2**^{1}) **a**

	1	2	3	4	5	6
1	0	2^1	0	1	0	0
2	2^1	0	0	0	0	0
3	0	0	0	1	0	1
4	1	0	1	0	0	0
5	0	0	0	0	0	2^1
6	0	0	1	0	2^1	0

(6x**1**, 4x**2**^{1}) **b**

	1	2	3	4	5	6
1	0	2^1	0	1	0	0
2	2^1	0	1	0	0	0
3	0	1	0	1	0	0
4	1	0	1	0	0	0
5	0	0	0	0	0	2^1
6	0	0	0	0	2^1	0

(6x**1**, 2x**2**^{1}, 2x**2**^{2}) **a**

	1	2	3	4	5	6
1	0	2^1	0	1	0	0
2	2^1	0	0	0	0	0
3	0	0	0	1	0	1
4	1	0	1	0	0	0
5	0	0	0	0	0	2^2
6	0	0	1	0	2^2	0

(6x**1**, 2x**2**^{1}, 2x**2**^{2}) **b**

	1	2	3	4	5	6
1	0	2^1	0	1	0	0
2	2^1	0	1	0	0	0
3	0	1	0	1	0	0
4	1	0	1	0	0	0
5	0	0	0	0	0	2^2
6	0	0	0	0	2^2	0

(6x**1**, 2x**2**^{1}, 2x**2**^{2}) **c**

	1	2	3	4	5	6
1	0	2^2	0	1	0	0
2	2^2	0	1	0	0	0
3	0	1	0	1	0	0
4	1	0	1	0	0	0
5	0	0	0	0	0	2^1
6	0	0	0	0	2^1	0

(6x**1**, 4x**2**^{2}) **a**

	1	2	3	4	5	6
1	0	2^2	0	1	0	0
2	2^2	0	0	0	0	0
3	0	0	0	1	0	1
4	1	0	1	0	0	0
5	0	0	0	0	0	2^2
6	0	0	1	0	2^2	0

(6x**1**, 4x**2**^{2}) **b**

	1	2	3	4	5	6
1	0	2^2	0	1	0	0
2	2^2	0	1	0	0	0
3	0	1	0	1	0	0
4	1	0	1	0	0	0
5	0	0	0	0	0	2^2
6	0	0	0	0	2^2	0

(4x**1**, 5x**2**)

	1	2	3	4	5	6
1	2	0	0	0	0	0
2	0	2	0	0	0	0
3	0	0	2	0	0	0
4	0	0	0	2	1	0
5	0	0	0	1	0	1
6	0	0	0	0	1	2

(4x**1**, 3x**2**, 2x**2**^{1}) **a**

	1	2	3	4	5	6
1	2	0	0	0	0	0
2	0	2	0	0	0	0
3	0	0	0	0	1	1
4	0	0	0	2	0	0
5	0	0	1	0	0	2^1
6	0	0	1	0	2^1	0

(4x**1**, 3x**2**, 2x**2**^{1}) **b**

	1	2	3	4	5	6
1	2	0	0	0	0	0
2	0	2	1	0	0	0
3	0	1	0	0	0	1
4	0	0	0	2	0	0
5	0	0	0	0	0	2^1
6	0	0	1	0	2^1	0

(4x**1**, 3x**2**, 2x**2**^{1}) **c**

	1	2	3	4	5	6
1	2	0	1	0	0	0
2	0	2	1	0	0	0
3	1	1	0	0	0	0
4	0	0	0	2	0	0
5	0	0	0	0	0	2^1
6	0	0	0	0	2^1	0

(4x**1**, 3x**2**, 2x**2**^{2}) **a**

	1	2	3	4	5	6
1	2	0	0	0	0	0
2	0	2	0	0	0	0
3	0	0	0	0	1	1
4	0	0	0	2	0	0
5	0	0	1	0	0	2^2
6	0	0	1	0	2^2	0

(4x**1**, 3x**2**, 2x**2**^{2}) **b**

	1	2	3	4	5	6
1	2	0	0	0	0	0
2	0	2	1	0	0	0
3	0	1	0	0	0	1
4	0	0	0	2	0	0
5	0	0	0	0	0	2^2
6	0	0	1	0	2^2	0

(4x**1**, 3x**2**, 2x**2**^{2}) **c**

	1	2	3	4	5	6
1	2	0	1	0	0	0
2	0	2	1	0	0	0
3	1	1	0	0	0	0
4	0	0	0	2	0	0
5	0	0	0	0	0	2^2
6	0	0	0	0	2^2	0

(4x**1**, 1x**2**, 4x**2**^{1}) **a**

	1	2	3	4	5	6
1	2	0	0	0	0	0
2	0	0	0	2^1	0	0
3	0	0	0	1	0	1
4	0	2^1	1	0	0	0
5	0	0	0	0	0	2^1
6	0	0	1	0	2^1	0

(4x**1**, 1x**2**, 4x**2**^{1}) **b**

	1	2	3	4	5	6
1	2	0	0	0	0	0
2	0	0	0	2^1	0	0
3	0	0	0	0	1	1
4	0	2^1	0	0	0	0
5	0	0	1	0	0	2^1
6	0	0	1	0	2^1	0

(4x**1**, 1x**2**, 4x**2**^{1}) **c**

	1	2	3	4	5	6
1	2	0	1	0	0	0
2	0	0	0	2^1	0	0
3	1	0	0	0	0	1
4	0	2^1	0	0	0	0
5	0	0	0	0	0	2^1
6	0	0	1	0	2^1	0

(4x**1**, 1x**2**, 2x**2**^{1}, 2x**2**^{2}) **a**

	1	2	3	4	5	6
1	2	0	0	0	0	0
2	0	0	0	2^1	0	0
3	0	0	0	1	0	1
4	0	2^1	1	0	0	0
5	0	0	0	0	0	2^2
6	0	0	1	0	2^2	0

(4x**1**, 1x**2**, 2x**2**^{1}, 2x**2**^{2}) **b**

	1	2	3	4	5	6
1	2	0	0	0	0	0
2	0	0	0	2^1	0	0
3	0	0	0	0	1	1
4	0	2^1	0	0	0	0
5	0	0	1	0	0	2^2
6	0	0	1	0	2^2	0

(4x**1**, 1x**2**, 2x**2**^{1}, 2x**2**^{2}) **c**

	1	2	3	4	5	6
1	2	0	0	0	0	0
2	0	0	0	2^2	0	0
3	0	0	0	0	1	1
4	0	2^2	0	0	0	0
5	0	0	1	0	0	2^1
6	0	0	1	0	2^1	0

(4x**1**, 1x**2**, 2x**2**^{1}, 2x**2**^{2}) **d**

	1	2	3	4	5	6
1	2	0	1	0	0	0
2	0	0	0	2^1	0	0
3	1	0	0	0	0	1
4	0	2^1	0	0	0	0
5	0	0	0	0	0	2^2
6	0	0	1	0	2^2	0

(4x**1**, 1x**2**, 2x**2**^{1}, 2x**2**^{2}) **e**

	1	2	3	4	5	6
1	2	0	1	0	0	0
2	0	0	0	2^2	0	0
3	1	0	0	0	0	1
4	0	2^2	0	0	0	0
5	0	0	0	0	0	2^1
6	0	0	1	0	2^1	0

(4x**1**, 1x**2**, 4x**2**^{2}) **a**

	1	2	3	4	5	6
1	2	0	0	0	0	0
2	0	0	0	2^2	0	0
3	0	0	0	1	0	1
4	0	2^2	1	0	0	0
5	0	0	0	0	0	2^2
6	0	0	1	0	2^2	0

(4x**1**, 1x**2**, 4x**2**^{2}) **b**

	1	2	3	4	5	6
1	2	0	0	0	0	0
2	0	0	0	2^2	0	0
3	0	0	0	0	1	1
4	0	2^2	0	0	0	0
5	0	0	1	0	0	2^2
6	0	0	1	0	2^2	0

(4x**1**, 1x**2**, 4x**2**^{2}) **c**

	1	2	3	4	5	6
1	2	0	1	0	0	0
2	0	0	0	2^2	0	0
3	1	0	0	0	0	1
4	0	2^2	0	0	0	0
5	0	0	0	0	0	2^2
6	0	0	1	0	2^2	0

(2x**1**, 6x**2**)

	1	2	3	4	5	6
1	2	0	0	0	0	0
2	0	2	0	0	0	0
3	0	0	2	0	0	0
4	0	0	0	2	0	0
5	0	0	0	0	2	1
6	0	0	0	0	1	2

(2x**1**, 4x**2**, 2x**2**^{1}) **a**

	1	2	3	4	5	6
1	2	0	0	0	0	0
2	0	2	0	0	0	0
3	0	0	2	0	0	1
4	0	0	0	2	0	0
5	0	0	0	0	0	2^1
6	0	0	1	0	2^1	0

(2x**1**, 4x**2**, 2x**2**^{1}) **b**

	1	2	3	4	5	6
1	2	0	0	0	0	0
2	0	2	1	0	0	0
3	0	1	2	0	0	0
4	0	0	0	2	0	0
5	0	0	0	0	0	2^1
6	0	0	0	0	2^1	0

(2x**1**, 4x**2**, 2x**2**^{2}) **a**

	1	2	3	4	5	6
1	2	0	0	0	0	0
2	0	2	0	0	0	0
3	0	0	2	0	0	1
4	0	0	0	2	0	0
5	0	0	0	0	0	2^2
6	0	0	1	0	2^2	0

(2x**1**, 4x**2**, 2x**2**^{2}) **b**

	1	2	3	4	5	6
1	2	0	0	0	0	0
2	0	2	1	0	0	0
3	0	1	2	0	0	0
4	0	0	0	2	0	0
5	0	0	0	0	0	2^2
6	0	0	0	0	2^2	0

(2x**1**, 2x**2**, 4x**2**^{1}) **a**

	1	2	3	4	5	6
1	2	0	0	0	0	0
2	0	0	0	2^1	0	0
3	0	0	0	1	0	2^1
4	0	2^1	1	0	0	0
5	0	0	0	0	2	0
6	0	0	2^1	0	0	0

(2x**1**, 2x**2**, 4x**2**^{1}) **b**

	1	2	3	4	5	6
1	2	0	1	0	0	0
2	0	0	0	2^1	0	0
3	1	0	0	0	0	2^1
4	0	2^1	0	0	0	0
5	0	0	0	0	2	0
6	0	0	2^1	0	0	0

(2x**1**, 2x**2**, 4x**2**^{1}) **c**

	1	2	3	4	5	6
1	2	0	0	0	1	0
2	0	0	0	2^1	0	0
3	0	0	0	0	0	2^1
4	0	2^1	0	0	0	0
5	1	0	0	0	2	0
6	0	0	2^1	0	0	0

(2x**1**, 2x**2**, 2x**2**^{1}, 2x**2**^{2}) **a**

	1	2	3	4	5	6
1	2	0	0	0	0	0
2	0	0	0	2^1	0	0
3	0	0	0	1	0	2^2
4	0	2^1	1	0	0	0
5	0	0	0	0	2	0
6	0	0	2^2	0	0	0

(2x**1**, 2x**2**, 2x**2**^{1}, 2x**2**^{2}) **b**

	1	2	3	4	5	6
1	2	0	1	0	0	0
2	0	0	0	2^1	0	0
3	1	0	0	0	0	2^2
4	0	2^1	0	0	0	0
5	0	0	0	0	2	0
6	0	0	2^2	0	0	0

(2x**1**, 2x**2**, 2x**2**^{1}, 2x**2**^{2}) **c**

	1	2	3	4	5	6
1	2	0	1	0	0	0
2	0	0	0	2^2	0	0
3	1	0	0	0	0	2^1
4	0	2^2	0	0	0	0
5	0	0	0	0	2	0
6	0	0	2^1	0	0	0

(2x**1**, 2x**2**, 2x**2**^{1}, 2x**2**^{2}) **d**

	1	2	3	4	5	6
1	2	0	0	0	1	0
2	0	0	0	2^1	0	0
3	0	0	0	0	0	2^2
4	0	2^1	0	0	0	0
5	1	0	0	0	2	0
6	0	0	2^2	0	0	0

(2x**1**, 2x**2**, 4x**2**^{2}) **a**

	1	2	3	4	5	6
1	2	0	0	0	0	0
2	0	0	0	2^2	0	0
3	0	0	0	1	0	2^2
4	0	2^2	1	0	0	0
5	0	0	0	0	2	0
6	0	0	2^2	0	0	0

(2x**1**, 2x**2**, 4x**2**^{2}) **b**

	1	2	3	4	5	6
1	2	0	1	0	0	0
2	0	0	0	2^2	0	0
3	1	0	0	0	0	2^2
4	0	2^2	0	0	0	0
5	0	0	0	0	2	0
6	0	0	2^2	0	0	0

(2x**1**, 2x**2**, 4x**2**^{2}) **c**

	1	2	3	4	5	6
1	2	0	0	0	1	0
2	0	0	0	2^2	0	0
3	0	0	0	0	0	2^2
4	0	2^2	0	0	0	0
5	1	0	0	0	2	0
6	0	0	2^2	0	0	0

(2x**1**, 6x**2**^{1})

	1	2	3	4	5	6
1	0	0	0	0	2^1	0
2	0	0	0	2^1	0	0
3	0	0	0	1	0	2^1
4	0	2^1	1	0	0	0
5	2^1	0	0	0	0	0
6	0	0	2^1	0	0	0

(2x**1**, 4x**2**^{1}, 2x**2**^{2}) **a**

	1	2	3	4	5	6
1	0	0	0	0	2^1	0
2	0	0	0	2^1	0	0
3	0	0	0	1	0	2^2
4	0	2^1	1	0	0	0
5	2^1	0	0	0	0	0
6	0	0	2^2	0	0	0

(2x**1**, 4x**2**^{1}, 2x**2**^{2}) **b**

	1	2	3	4	5	6
1	0	0	0	0	2^2	0
2	0	0	0	2^1	0	0
3	0	0	0	1	0	2^1
4	0	2^1	1	0	0	0
5	2^2	0	0	0	0	0
6	0	0	2^1	0	0	0

(2x**1**, 2x**2**^{1}, 4x**2**^{2}) **a**

	1	2	3	4	5	6
1	0	0	0	0	2^2	0
2	0	0	0	2^1	0	0
3	0	0	0	1	0	2^2
4	0	2^1	1	0	0	0
5	2^2	0	0	0	0	0
6	0	0	2^2	0	0	0

(2x**1**, 2x**2**^{1}, 4x**2**^{2}) **b**

	1	2	3	4	5	6
1	0	0	0	0	2^1	0
2	0	0	0	2^2	0	0
3	0	0	0	1	0	2^2
4	0	2^2	1	0	0	0
5	2^1	0	0	0	0	0
6	0	0	2^2	0	0	0

(2x**1**, 6x**2**^{2})

	1	2	3	4	5	6
1	0	0	0	0	2^2	0
2	0	0	0	2^2	0	0
3	0	0	0	1	0	2^2
4	0	2^2	1	0	0	0
5	2^2	0	0	0	0	0
6	0	0	2^2	0	0	0

$^2V_4{}^3V_4$ NG

$^2V_5{}^3V_2$ NG

$^2V_6{}^3V_2$ NG

$^2V_r{}^4V_r$

$^2V_1{}^4V_1$

(1x**2**, 2x**2**^{1})

	1	2
1	0	2^1
2	2^1	2

(1x**2**, 2x**2**^{2})

	1	2
1	0	2^2
2	2^2	2

$^2V_1{}^4V_2$

(6x**1**, 2x**2**)

	1	2	3
1	2	1	1
2	1	2	1
3	1	1	0

(3x**2**, 2x**2**^{1})

	1	2	3
1	2	2^1	0
2	2^1	2	0
3	0	0	2

(3x**2**, 2x**2**^{2})

	1	2	3
1	2	2^2	0
2	2^2	2	0
3	0	0	2

(1x**2**, 4x**2**^{1})

	1	2	3
1	2	2^1	0
2	2^1	0	2^1
3	0	2^1	0

(1x**2**, 2x**2**^{1}, 2x**2**^{2}) **a**

	1	2	3
1	2	2^1	0
2	2^1	0	2^2
3	0	2^2	0

(1x**2**, 2x**2**^{1}, 2x**2**^{2}) **b**

	1	2	3
1	2	2^2	0
2	2^2	0	2^1
3	0	2^1	0

(1x**2**, 4x**2**^{2})

	1	2	3
1	2	2^2	0
2	2^2	0	2^2
3	0	2^2	0

$^2V_1{}^4V_3$

(8x**1**, 3x**2**)

	1	2	3	4
1	2	1	0	1
2	1	2	1	0
3	0	1	2	1
4	1	0	1	0

(8x**1**, 1x**2**, 2x**2**^{1})

	1	2	3	4
1	2	1	0	1
2	1	0	1	2^1
3	0	1	0	1
4	1	2^1	1	0

(8x**1**, 1x**2**, 2x**2**^{2})

	1	2	3	4
1	2	1	0	1
2	1	0	1	2^2
3	0	1	0	1
4	1	2^2	1	0

(6x**1**, 4x**2**)

	1	2	3	4
1	2	1	1	0
2	1	2	1	0
3	1	1	2	0
4	0	0	0	2

(6x**1**, 2x**2**, 2x**2**^{1}) **a**

	1	2	3	4
1	2	1	0	1
2	1	0	2^1	1
3	0	2^1	0	0
4	1	1	0	2

(6x**1**, 2x**2**, 2x**2**^{1}) **b**

	1	2	3	4
1	2	1	0	1
2	1	0	2^1	1
3	0	2^1	2	0
4	1	1	0	0

(6x**1**, 2x**2**, 2x**2**^{2}) **a**

	1	2	3	4
1	2	1	0	1
2	1	0	2^2	1
3	0	2^2	0	0
4	1	1	0	2

(6x**1**, 2x**2**, 2x**2**^{2}) **b**

	1	2	3	4
1	2	1	0	1
2	1	0	2^2	1
3	0	2^2	2	0
4	1	1	0	0

(6x**1**, 4x**2**^{1})

	1	2	3	4
1	0	1	1	2^1
2	1	0	1	0
3	1	1	0	2^1
4	2^1	0	2^1	0

(6x**1**, 2x**2**^{1}, 2x**2**^{2})

	1	2	3	4
1	0	1	1	2^2
2	1	0	1	0
3	1	1	0	2^1
4	2^2	0	2^1	0

(6x**1**, 4x**2**^{2})

	1	2	3	4
1	0	1	1	2^2
2	1	0	1	0
3	1	1	0	2^2
4	2^2	0	2^2	0

(3x**2**, 4x**2**^{1}) **a**

	1	2	3	4
1	2	0	0	2^1
2	0	2	0	0
3	0	0	2	2^1
4	2^1	0	2^1	0

(3x**2**, 4x**2**^{1}) **b**

	1	2	3	4
1	2	2^1	0	0
2	2^1	2	0	0
3	0	0	2	2^1
4	0	0	2^1	0

(3x**2**, 2x**2**^{1}, 2x**2**^{2}) **a**

	1	2	3	4
1	2	0	0	2^2
2	0	2	0	0
3	0	0	2	2^1
4	2^2	0	2^1	0

(3x**2**, 2x**2**^{1}, 2x**2**^{2}) **b**

	1	2	3	4
1	2	2^1	0	0
2	2^1	2	0	0
3	0	0	2	2^2
4	0	0	2^2	0

(3x**2**, 2x**2**^{1}, 2x**2**^{2}) **c**

	1	2	3	4
1	2	2^2	0	0
2	2^2	2	0	0
3	0	0	2	2^1
4	0	0	2^1	0

(3x**2**, 4x**2**^{2}) **a**

	1	2	3	4
1	2	0	0	2^2
2	0	2	0	0
3	0	0	2	2^2
4	2^2	0	2^2	0

(3x**2**, 4x**2**^{2}) **b**

	1	2	3	4
1	2	2^2	0	0
2	2^2	2	0	0
3	0	0	2	2^2
4	0	0	2^2	0

(1x**2**, 6x**2**^{1}) **a**

	1	2	3	4
1	0	0	2^1	2^1
2	0	2	0	0
3	2^1	0	0	2^1
4	2^1	0	2^1	0

(1x**2**, 6x**2**^{1}) **b**

	1	2	3	4
1	0	0	0	2^1
2	0	2	2^1	0
3	0	2^1	0	2^1
4	2^1	0	2^1	0

(1x**2**, 4x**2**^{1}, 2x**2**^{2}) **a**

	1	2	3	4
1	0	0	2^2	2^1
2	0	2	0	0
3	2^2	0	0	2^1
4	2^1	0	2^1	0

(1x**2**, 4x**2**^{1}, 2x**2**^{2}) **b**

	1	2	3	4
1	0	0	0	2^1
2	0	2	2^2	0
3	0	2^2	0	2^1
4	2^1	0	2^1	0

(1x**2**, 4x**2**^{1}, 2x**2**^{2}) **c**

	1	2	3	4
1	0	0	0	2^1
2	0	2	2^1	0
3	0	2^1	0	2^2
4	2^1	0	2^2	0

(1x**2**, 4x**2**^{1}, 2x**2**^{2}) **d**

	1	2	3	4
1	0	0	0	2^2
2	0	2	2^1	0
3	0	2^1	0	2^1
4	2^2	0	2^1	0

(1x**2**, 2x**2**^{1}, 4x**2**^{2}) **a**

	1	2	3	4
1	0	0	2^1	2^2
2	0	2	0	0
3	2^1	0	0	2^2
4	2^2	0	2^2	0

(1x**2**, 2x**2**^{1}, 4x**2**^{2}) **b**

	1	2	3	4
1	0	0	0	2^1
2	0	2	2^2	0
3	0	2^2	0	2^2
4	2^1	0	2^2	0

(1x**2**, 2x**2**^{1}, 4x**2**^{2}) **c**

	1	2	3	4
1	0	0	0	2^2
2	0	2	2^2	0
3	0	2^2	0	2^1
4	2^2	0	2^1	0

(1x**2**, 2x**2**^{1}, 4x**2**^{2}) **d**

	1	2	3	4
1	0	0	0	2^2
2	0	2	2^1	0
3	0	2^1	0	2^2
4	2^2	0	2^2	0

(1x**2**, 6x**2**^{2}) **a**

	1	2	3	4
1	0	0	2^2	2^2
2	0	2	0	0
3	2^2	0	0	2^2
4	2^2	0	2^2	0

(1x**2**, 6x**2**^{2}) **b**

	1	2	3	4
1	0	0	0	2^2
2	0	2	2^2	0
3	0	2^2	0	2^2
4	2^2	0	2^2	0

$^2V_1{}^4V_4$

(14x**1**, 2x**2**)

	1	2	3	4	5
1	2	0	1	1	0
2	0	2	1	1	0
3	1	1	0	1	1
4	1	1	1	0	1
5	0	0	1	1	0

(14x**1**, 2x**2**^{1})

	1	2	3	4	5
1	0	1	1	1	1
2	1	0	2^1	1	0
3	1	2^1	0	1	0
4	1	1	1	0	1
5	1	0	0	1	0

(14x**1**, 2x**2**^{2})

	1	2	3	4	5
1	0	1	1	1	1
2	1	0	2^2	1	0
3	1	2^2	0	1	0
4	1	1	1	0	1
5	1	0	0	1	0

(12x**1**, 3x**2**)

	1	2	3	4	5
1	2	1	1	0	0
2	1	2	1	0	0
3	1	1	0	1	1
4	0	0	1	0	1
5	0	0	1	1	2

(12x**1**, 1x**2**, 2x**2**^{1})

	1	2	3	4	5
1	2	0	1	1	0
2	0	0	2^1	1	1
3	1	2^1	0	1	0
4	1	1	1	0	1
5	0	1	0	1	0

(12x**1**, 1x**2**, 2x**2**^{2})

	1	2	3	4	5
1	2	0	1	1	0
2	0	0	2^2	1	1
3	1	2^2	0	1	0
4	1	1	1	0	1
5	0	1	0	1	0

(10x**1**, 4x**2**)

	1	2	3	4	5
1	2	0	1	1	0
2	0	2	1	0	1
3	1	1	2	0	0
4	1	0	0	0	1
5	0	1	0	1	2

(10x**1**, 2x**2**, 2x**2**^{1}) **a**

	1	2	3	4	5
1	0	1	0	2^1	1
2	1	0	1	0	0
3	0	1	2	1	0
4	2^1	0	1	0	1
5	1	0	0	1	2

(10x**1**, 2x**2**, 2x**2**^{1}) **b**

	1	2	3	4	5
1	0	1	0	2^1	1
2	1	2	1	0	0
3	0	1	2	1	0
4	2^1	0	1	0	1
5	1	0	0	1	0

(10x**1**, 2x**2**, 2x**2**^{2}) **a**

	1	2	3	4	5
1	0	1	0	2^2	1
2	1	0	1	0	0
3	0	1	2	1	0
4	2^2	0	1	0	1
5	1	0	0	1	2

(10x**1**, 2x**2**, 2x**2**^{2}) **b**

	1	2	3	4	5
1	0	1	0	2^2	1
2	1	2	1	0	0
3	0	1	2	1	0
4	2^2	0	1	0	1
5	1	0	0	1	0

(10x**1**, 4x**2**^{1})

	1	2	3	4	5
1	0	2^1	1	0	1
2	2^1	0	1	1	0
3	1	1	0	2^1	0
4	0	1	2^1	0	1
5	1	0	0	1	0

(10x**1**, 2x**2**^{1}, 2x**2**^{2})

	1	2	3	4	5
1	0	2^1	1	0	1
2	2^1	0	1	1	0
3	1	1	0	2^2	0
4	0	1	2^2	0	1
5	1	0	0	1	0

(10x**1**, 4x**2**^{2})

	1	2	3	4	5
1	0	2^2	1	0	1
2	2^2	0	1	1	0
3	1	1	0	2^2	0
4	0	1	2^2	0	1
5	1	0	0	1	0

(8x**1**, 5x**2**)

	1	2	3	4	5
1	2	1	0	1	0
2	1	2	1	0	0
3	0	1	2	1	0
4	1	0	1	2	0
5	0	0	0	0	2

(8x**1**, 3x**2**, 2x**2**^{1}) **a**

	1	2	3	4	5
1	2	0	1	1	0
2	0	2	1	1	0
3	1	1	0	2^1	0
4	1	1	2^1	0	0
5	0	0	0	0	2

(8x**1**, 3x**2**, 2x**2**^{1}) **b**

	1	2	3	4	5
1	2	0	1	1	0
2	0	0	1	1	2^1
3	1	1	2	0	0
4	1	1	0	2	0
5	0	2^1	0	0	0

(8x**1**, 3x**2**, 2x**2**^{1}) **c**

	1	2	3	4	5
1	2	0	1	1	0
2	0	0	1	1	2^1
3	1	1	0	0	0
4	1	1	0	2	0
5	0	2^1	0	0	2

(8x**1**, 3x**2**, 2x**2**^{1}) **d**

	1	2	3	4	5
1	0	0	1	1	0
2	0	0	1	1	2^1
3	1	1	2	0	0
4	1	1	0	2	0
5	0	2^1	0	0	2

(8x**1**, 3x**2**, 2x**2**^{2}) **a**

	1	2	3	4	5
1	2	0	1	1	0
2	0	2	1	1	0
3	1	1	0	2^2	0
4	1	1	2^2	0	0
5	0	0	0	0	2

(8x**1**, 3x**2**, 2x**2**^{2}) **b**

	1	2	3	4	5
1	2	0	1	1	0
2	0	0	1	1	2^2
3	1	1	2	0	0
4	1	1	0	2	0
5	0	2^2	0	0	0

(8x**1**, 3x**2**, 2x**2**^{2}) **c**

	1	2	3	4	5
1	2	0	1	1	0
2	0	0	1	1	2^2
3	1	1	0	0	0
4	1	1	0	2	0
5	0	2^2	0	0	2

(8x**1**, 3x**2**, 2x**2**^{2}) **d**

	1	2	3	4	5
1	0	0	1	1	0
2	0	0	1	1	2^2
3	1	1	2	0	0
4	1	1	0	2	0
5	0	2^2	0	0	2

(8x**1**, 1x**2**, 4x**2**^{1}) **a**

	1	2	3	4	5
1	2	0	1	1	0
2	0	0	1	1	2^1
3	1	1	0	2^1	0
4	1	1	2^1	0	0
5	0	2^1	0	0	0

(8x**1**, 1x**2**, 4x**2**^{1}) **b**

	1	2	3	4	5
1	0	0	1	1	0
2	0	0	1	1	2^1
3	1	1	0	2^1	0
4	1	1	2^1	0	0
5	0	2^1	0	0	2

(8x**1**, 1x**2**, 4x**2**^{1}) **c**

	1	2	3	4	5
1	0	1	0	1	2^1
2	1	0	1	0	0
3	0	1	2	1	0
4	1	0	1	0	2^1
5	2^1	0	0	2^1	0

(8x**1**, 1x**2**, 4x**2**^{1}) **d**

	1	2	3	4	5
1	0	2^1	1	1	0
2	2^1	0	1	1	0
3	1	1	0	2^1	0
4	1	1	2^1	0	0
5	0	0	0	0	2

(8x**1**, 1x**2**, 4x**2**^{1}) **e**

	1	2	3	4	5
1	2	0	1	1	0
2	0	0	1	1	0
3	1	1	0	0	2^1
4	1	1	0	0	2^1
5	0	0	2^1	2^1	0

(8x**1**, 1x**2**, 2x**2**^{1}, 2x**2**^{2}) **a**

	1	2	3	4	5
1	2	0	1	1	0
2	0	0	1	1	2^1
3	1	1	0	2^2	0
4	1	1	2^2	0	0
5	0	2^1	0	0	0

(8x**1**, 1x**2**, 2x**2**^{1}, 2x**2**^{2}) **b**

	1	2	3	4	5
1	2	0	1	1	0
2	0	0	1	1	2^2
3	1	1	0	2^1	0
4	1	1	2^1	0	0
5	0	2^2	0	0	0

(8x**1**, 1x**2**, 2x**2**^{1}, 2x**2**^{2}) **c**

	1	2	3	4	5
1	0	0	1	1	0
2	0	0	1	1	2^1
3	1	1	0	2^2	0
4	1	1	2^2	0	0
5	0	2^1	0	0	2

(8x**1**, 1x**2**, 2x**2**^{1}, 2x**2**^{2}) **d**

	1	2	3	4	5
1	0	0	1	1	0
2	0	0	1	1	2^2
3	1	1	0	2^1	0
4	1	1	2^1	0	0
5	0	2^2	0	0	2

(8x**1**, 1x**2**, 2x**2**^{1}, 2x**2**^{2}) **e**

	1	2	3	4	5
1	0	1	0	1	2^2
2	1	0	1	0	0
3	0	1	2	1	0
4	1	0	1	0	2^1
5	2^2	0	0	2^1	0

(8x**1**, 1x**2**, 2x**2**^{1}, 2x**2**^{2}) **f**

	1	2	3	4	5
1	0	1	0	1	2^1
2	1	0	1	0	0
3	0	1	2	1	0
4	1	0	1	0	2^2
5	2^1	0	0	2^2	0

(8x**1**, 1x**2**, 2x**2**^{1}, 2x**2**^{2}) **g**

	1	2	3	4	5
1	0	2^1	1	1	0
2	2^1	0	1	1	0
3	1	1	0	2^2	0
4	1	1	2^2	0	0
5	0	0	0	0	2

(8x**1**, 1x**2**, 2x**2**^{1}, 2x**2**^{2}) **h**

	1	2	3	4	5
1	2	0	1	1	0
2	0	0	1	1	0
3	1	1	0	0	2^2
4	1	1	0	0	2^1
5	0	0	2^2	2^1	0

(8x**1**, 1x**2**, 4x**2**^{2}) **a**

	1	2	3	4	5
1	2	0	1	1	0
2	0	0	1	1	2^2
3	1	1	0	2^2	0
4	1	1	2^2	0	0
5	0	2^2	0	0	0

(8x**1**, 1x**2**, 4x**2**^{2}) **b**

	1	2	3	4	5
1	0	0	1	1	0
2	0	0	1	1	2^2
3	1	1	0	2^2	0
4	1	1	2^2	0	0
5	0	2^2	0	0	2

(8x**1**, 1x**2**, 4x**2**^{2}) **c**

	1	2	3	4	5
1	0	1	0	1	2^2
2	1	0	1	0	0
3	0	1	2	1	0
4	1	0	1	0	2^2
5	2^2	0	0	2^2	0

(8x**1**, 1x**2**, 4x**2**^{2}) **d**

	1	2	3	4	5
1	0	2^2	1	1	0
2	2^2	0	1	1	0
3	1	1	0	2^2	0
4	1	1	2^2	0	0
5	0	0	0	0	2

(8x**1**, 1x**2**, 4x$\mathbf{2}^2$) **e**

	1	2	3	4	5
1	2	0	1	1	0
2	0	0	1	1	0
3	1	1	0	0	2^2
4	1	1	0	0	2^2
5	0	0	2^2	2^2	0

(6x**1**, 4x**2**, 2x$\mathbf{2}^1$) **a**

	1	2	3	4	5
1	2	0	1	1	0
2	0	0	0	0	2^1
3	1	0	2	1	0
4	1	0	1	2	0
5	0	2^1	0	0	2

(6x**1**, 4x**2**, 2x$\mathbf{2}^1$) **b**

	1	2	3	4	5
1	2	0	1	1	0
2	0	2	2^1	0	0
3	1	2^1	0	1	0
4	1	0	1	2	0
5	0	0	0	0	2

(6x**1**, 4x**2**, 2x$\mathbf{2}^1$) **c**

	1	2	3	4	5
1	2	0	1	1	0
2	0	2	0	0	2^1
3	1	0	0	1	0
4	1	0	1	2	0
5	0	2^1	0	0	2

(6x**1**, 4x**2**, 2x$\mathbf{2}^2$) **a**

	1	2	3	4	5
1	2	0	1	1	0
2	0	0	0	0	2^2
3	1	0	2	1	0
4	1	0	1	2	0
5	0	2^2	0	0	2

(6x**1**, 4x**2**, 2x$\mathbf{2}^2$) **b**

	1	2	3	4	5
1	2	0	1	1	0
2	0	2	2^2	0	0
3	1	2^2	0	1	0
4	1	0	1	2	0
5	0	0	0	0	2

(6x**1**, 4x**2**, 2x$\mathbf{2}^2$) **c**

	1	2	3	4	5
1	2	0	1	1	0
2	0	2	0	0	2^2
3	1	0	0	1	0
4	1	0	1	2	0
5	0	2^2	0	0	2

(6x**1**, 2x**2**, 4x$\mathbf{2}^1$) **a**

	1	2	3	4	5
1	0	0	1	1	0
2	0	2	2^1	0	0
3	1	2^1	0	1	0
4	1	0	1	0	2^1
5	0	0	0	2^1	2

(6x**1**, 2x**2**, 4x$\mathbf{2}^1$) **b**

	1	2	3	4	5
1	2	0	1	1	0
2	0	0	2^1	0	0
3	1	2^1	0	1	0
4	1	0	1	0	2^1
5	0	0	0	2^1	2

(6x**1**, 2x**2**, 4x$\mathbf{2}^1$) **c**

	1	2	3	4	5
1	2	0	1	1	0
2	0	0	2^1	0	2^1
3	1	2^1	0	1	0
4	1	0	1	0	0
5	0	2^1	0	0	2

(6x**1**, 2x**2**, 4x$\mathbf{2}^1$) **d**

	1	2	3	4	5
1	2	0	1	1	0
2	0	0	2^1	0	2^1
3	1	2^1	0	1	0
4	1	0	1	2	0
5	0	2^1	0	0	0

(6x**1**, 2x**2**, 4x$\mathbf{2}^1$) **e**

	1	2	3	4	5
1	2	0	1	1	0
2	0	0	2^1	2^1	0
3	1	2^1	0	1	0
4	1	2^1	1	0	0
5	0	0	0	0	2

(6x**1**, 2x**2**, 2x$\mathbf{2}^1$, 2x$\mathbf{2}^2$) **a**

	1	2	3	4	5
1	0	0	1	1	0
2	0	2	2^1	0	0
3	1	2^1	0	1	0
4	1	0	1	0	2^2
5	0	0	0	2^2	2

(6x**1**, 2x**2**, 2x$\mathbf{2}^1$, 2x$\mathbf{2}^2$) **b**

	1	2	3	4	5
1	2	0	1	1	0
2	0	0	2^1	0	0
3	1	2^1	0	1	0
4	1	0	1	0	2^2
5	0	0	0	2^2	2

(6x**1**, 2x**2**, 2x$\mathbf{2}^1$, 2x$\mathbf{2}^2$) **c**

	1	2	3	4	5
1	2	0	1	1	0
2	0	0	2^2	0	0
3	1	2^2	0	1	0
4	1	0	1	0	2^1
5	0	0	0	2^1	2

(6x**1**, 2x**2**, 2x$\mathbf{2}^1$, 2x$\mathbf{2}^2$) **d**

	1	2	3	4	5
1	2	0	1	1	0
2	0	0	2^1	0	2^2
3	1	2^1	0	1	0
4	1	0	1	0	0
5	0	2^2	0	0	2

(6x**1**, 2x**2**, 2x$\mathbf{2}^1$, 2x$\mathbf{2}^2$) **e**

	1	2	3	4	5
1	2	0	1	1	0
2	0	0	2^2	0	2^1
3	1	2^2	0	1	0
4	1	0	1	0	0
5	0	2^1	0	0	2

(6x**1**, 2x**2**, 2x$\mathbf{2}^1$, 2x$\mathbf{2}^2$) **f**

	1	2	3	4	5
1	2	0	1	1	0
2	0	0	2^1	0	2^2
3	1	2^1	0	1	0
4	1	0	1	2	0
5	0	2^2	0	0	0

(6x**1**, 2x**2**, 2x$\mathbf{2}^1$, 2x$\mathbf{2}^2$) **g**

	1	2	3	4	5
1	2	0	1	1	0
2	0	0	2^2	0	2^1
3	1	2^2	0	1	0
4	1	0	1	2	0
5	0	2^1	0	0	0

(6x**1**, 2x**2**, 2x$\mathbf{2}^1$, 2x$\mathbf{2}^2$) **h**

	1	2	3	4	5
1	2	0	1	1	0
2	0	0	2^2	2^1	0
3	1	2^2	0	1	0
4	1	2^1	1	0	0
5	0	0	0	0	2

(6x**1**, 2x**2**, 4x$\mathbf{2}^2$) **a**

	1	2	3	4	5
1	0	0	1	1	0
2	0	2	2^2	0	0
3	1	2^2	0	1	0
4	1	0	1	0	2^2
5	0	0	0	2^2	2

(6x**1**, 2x**2**, 4x$\mathbf{2}^2$) **b**

	1	2	3	4	5
1	2	0	1	1	0
2	0	0	2^2	0	0
3	1	2^2	0	1	0
4	1	0	1	0	2^2
5	0	0	0	2^2	2

(6x**1**, 2x**2**, 4x$\mathbf{2}^2$) **c**

	1	2	3	4	5
1	2	0	1	1	0
2	0	0	2^2	0	2^2
3	1	2^2	0	1	0
4	1	0	1	0	0
5	0	2^2	0	0	2

(6x**1**, 2x**2**, 4x$\mathbf{2}^2$) **d**

	1	2	3	4	5
1	2	0	1	1	0
2	0	0	2^2	0	2^2
3	1	2^2	0	1	0
4	1	0	1	2	0
5	0	2^2	0	0	0

(6x**1**, 2x**2**, 4x$\mathbf{2}^2$) **e**

	1	2	3	4	5
1	2	0	1	1	0
2	0	0	2^2	2^2	0
3	1	2^2	0	1	0
4	1	2^2	1	0	0
5	0	0	0	0	2

(6x**1**, 6x$\mathbf{2}^1$) **a**

	1	2	3	4	5
1	0	0	1	1	0
2	0	0	2^1	0	2^1
3	1	2^1	0	1	0
4	1	0	1	0	2^1
5	0	2^1	0	2^1	0

(6x**1**, 6x$\mathbf{2}^1$) **b**

	1	2	3	4	5
1	0	0	0	2^1	0
2	0	0	2^1	0	2^1
3	0	2^1	0	1	1
4	2^1	0	1	0	1
5	0	2^1	1	1	0

(6x**1**, 4x$\mathbf{2}^1$, 2x$\mathbf{2}^2$) **a**

	1	2	3	4	5
1	0	0	1	1	0
2	0	0	2^1	0	2^1
3	1	2^1	0	1	0
4	1	0	1	0	2^2
5	0	2^1	0	2^2	0

(6x**1**, 4x$\mathbf{2}^1$, 2x$\mathbf{2}^2$) **b**

	1	2	3	4	5
1	0	0	1	1	0
2	0	0	2^1	0	2^2
3	1	2^1	0	1	0
4	1	0	1	0	2^1
5	0	2^2	0	2^1	0

(6x**1**, 4x$\mathbf{2}^1$, 2x$\mathbf{2}^2$) **c**

	1	2	3	4	5
1	0	0	0	2^1	0
2	0	0	2^2	0	2^1
3	0	2^2	0	1	1
4	2^1	0	1	0	1
5	0	2^1	1	1	0

(6x**1**, 4x$\mathbf{2}^1$, 2x$\mathbf{2}^2$) **d**

	1	2	3	4	5
1	0	0	0	2^2	0
2	0	0	2^1	0	2^1
3	0	2^1	0	1	1
4	2^2	0	1	0	1
5	0	2^1	1	1	0

(6x**1**, 2x$\mathbf{2}^1$, 4x$\mathbf{2}^2$) **a**

	1	2	3	4	5
1	0	0	1	1	0
2	0	0	2^2	0	2^1
3	1	2^2	0	1	0
4	1	0	1	0	2^2
5	0	2^1	0	2^2	0

(6x**1**, 2x$\mathbf{2}^1$, 4x$\mathbf{2}^2$) **b**

	1	2	3	4	5
1	0	0	1	1	0
2	0	0	2^1	0	2^2
3	1	2^1	0	1	0
4	1	0	1	0	2^2
5	0	2^2	0	2^2	0

(6x**1**, 2x$\mathbf{2}^1$, 4x$\mathbf{2}^2$) **c**

	1	2	3	4	5
1	0	0	0	2^2	0
2	0	0	2^2	0	2^1
3	0	2^2	0	1	1
4	2^2	0	1	0	1
5	0	2^1	1	1	0

(6x**1**, 2x$\mathbf{2}^1$, 4x$\mathbf{2}^2$) **d**

	1	2	3	4	5
1	0	0	0	2^1	0
2	0	0	2^2	0	2^2
3	0	2^2	0	1	1
4	2^1	0	1	0	1
5	0	2^2	1	1	0

(6x1, 6x2^2) a

	1	2	3	4	5
1	0	0	1	1	0
2	0	0	2^2	0	2^2
3	1	2^2	0	1	0
4	1	0	1	0	2^2
5	0	2^2	0	2^2	0

(6x1, 6x2^1) b

	1	2	3	4	5
1	0	0	0	2^2	0
2	0	0	2^2	0	2^2
3	0	2^2	0	1	1
4	2^2	0	1	0	1
5	0	2^2	1	1	0

(5x2, 4x2^1)

	1	2	3	4	5
1	2	0	0	0	0
2	0	2	2^1	0	0
3	0	2^1	2	0	0
4	0	0	0	2	2^1
5	0	0	0	2^1	2

(5x2, 2x2^1, 2x2^2)

	1	2	3	4	5
1	2	0	0	0	0
2	0	2	2^2	0	0
3	0	2^2	2	0	0
4	0	0	0	2	2^1
5	0	0	0	2^1	2

(5x2, 4x2^2)

	1	2	3	4	5
1	2	0	0	0	0
2	0	2	2^2	0	0
3	0	2^2	2	0	0
4	0	0	0	2	2^2
5	0	0	0	2^2	2

(3x2, 6x2^1) a

	1	2	3	4	5
1	2	0	0	2^1	0
2	0	0	2^1	0	2^1
3	0	2^1	2	0	0
4	2^1	0	0	0	0
5	0	2^1	0	0	2

(3x2, 6x2^1) b

	1	2	3	4	5
1	2	0	0	0	2^1
2	0	0	2^1	0	2^1
3	0	2^1	2	0	0
4	0	0	0	2	0
5	2^1	2^1	0	0	0

(3x2, 6x2^1) c

	1	2	3	4	5
1	2	0	0	2^1	0
2	0	0	2^1	0	2^1
3	0	2^1	0	0	0
4	2^1	0	0	2	0
5	0	2^1	0	0	2

(3x2, 4x2^1, 2x2^2) a

	1	2	3	4	5
1	2	0	0	2^2	0
2	0	0	2^1	0	2^1
3	0	2^1	2	0	0
4	2^2	0	0	0	0
5	0	2^1	0	0	2

(3x2, 4x2^1, 2x2^2) b

	1	2	3	4	5
1	2	0	0	2^1	0
2	0	0	2^2	0	2^1
3	0	2^2	2	0	0
4	2^1	0	0	0	0
5	0	2^1	0	0	2

(3x2, 4x2^1, 2x2^2) c

	1	2	3	4	5
1	2	0	0	0	2^2
2	0	0	2^1	0	2^1
3	0	2^1	2	0	0
4	0	0	0	2	0
5	2^2	2^1	0	0	0

(3x2, 4x2^1, 2x2^2) d

	1	2	3	4	5
1	2	0	0	0	2^1
2	0	0	2^1	0	2^2
3	0	2^1	2	0	0
4	0	0	0	2	0
5	2^1	2^2	0	0	0

(3x2, 4x2^1, 2x2^2) e

	1	2	3	4	5
1	2	0	0	2^2	0
2	0	0	2^1	0	2^1
3	0	2^1	0	0	0
4	2^2	0	0	2	0
5	0	2^1	0	0	2

(3x2, 4x2^1, 2x2^2) f

	1	2	3	4	5
1	2	0	0	2^1	0
2	0	0	2^2	0	2^1
3	0	2^2	0	0	0
4	2^1	0	0	2	0
5	0	2^1	0	0	2

(3x2, 4x2^1, 2x2^2) g

	1	2	3	4	5
1	2	0	0	2^1	0
2	0	0	2^1	0	2^2
3	0	2^1	0	0	0
4	2^1	0	0	2	0
5	0	2^2	0	0	2

(3x2, 2x2^1, 4x2^2) a

	1	2	3	4	5
1	2	0	0	2^2	0
2	0	0	2^1	0	2^2
3	0	2^1	2	0	0
4	2^2	0	0	0	0
5	0	2^2	0	0	2

(3x2, 2x2^1, 4x2^2) b

	1	2	3	4	5
1	2	0	0	2^1	0
2	0	0	2^2	0	2^2
3	0	2^2	2	0	0
4	2^1	0	0	0	0
5	0	2^2	0	0	2

(3x2, 2x2^1, 4x2^2) c

	1	2	3	4	5
1	2	0	0	0	2^2
2	0	0	2^1	0	2^2
3	0	2^1	2	0	0
4	0	0	0	2	0
5	2^2	2^2	0	0	0

(3x2, 2x2^1, 4x2^2) d

	1	2	3	4	5
1	2	0	0	0	2^2
2	0	0	2^2	0	2^1
3	0	2^2	2	0	0
4	0	0	0	2	0
5	2^2	2^1	0	0	0

(3x2, 2x2^1, 4x2^2) e

	1	2	3	4	5
1	2	0	0	2^2	0
2	0	0	2^2	0	2^1
3	0	2^2	0	0	0
4	2^2	0	0	2	0
5	0	2^1	0	0	2

(3x2, 2x2^1, 4x2^2) f

	1	2	3	4	5
1	2	0	0	2^2	0
2	0	0	2^1	0	2^2
3	0	2^1	0	0	0
4	2^2	0	0	2	0
5	0	2^2	0	0	2

(3x2, 2x2^1, 4x2^2) g

	1	2	3	4	5
1	2	0	0	2^1	0
2	0	0	2^2	0	2^2
3	0	2^2	0	0	0
4	2^1	0	0	2	0
5	0	2^2	0	0	2

(3x2, 6x2^2) a

	1	2	3	4	5
1	2	0	0	2^2	0
2	0	0	2^2	0	2^2
3	0	2^2	2	0	0
4	2^2	0	0	0	0
5	0	2^2	0	0	2

(3x2, 6x2^2) b

	1	2	3	4	5
1	2	0	0	0	2^2
2	0	0	2^2	0	2^2
3	0	2^2	2	0	0
4	0	0	0	2	0
5	2^2	2^2	0	0	0

(3x2, 6x2^2) c

	1	2	3	4	5
1	2	0	0	2^2	0
2	0	0	2^2	0	2^2
3	0	2^2	0	0	0
4	2^2	0	0	2	0
5	0	2^2	0	0	2

(1x2, 8x2^1) a

	1	2	3	4	5
1	0	0	0	2^1	0
2	0	0	2^1	0	2^1
3	0	2^1	2	0	0
4	2^1	0	0	0	2^1
5	0	2^1	0	2^1	0

(1x2, 8x2^1) b

	1	2	3	4	5
1	2	0	0	2^1	0
2	0	0	2^1	0	2^1
3	0	2^1	0	0	2^1
4	2^1	0	0	0	0
5	0	2^1	2^1	0	0

(1x2, 8x2^1) c

	1	2	3	4	5
1	2	0	0	0	0
2	0	0	2^1	0	2^1
3	0	2^1	0	2^1	0
4	0	0	2^1	0	2^1
5	0	2^1	0	2^1	0

(1x2, 6x2^1, 2x2^2) a

	1	2	3	4	5
1	0	0	0	2^2	0
2	0	0	2^1	0	2^1
3	0	2^1	2	0	0
4	2^2	0	0	0	2^1
5	0	2^1	0	2^1	0

(1x2, 6x2^1, 2x2^2) b

	1	2	3	4	5
1	0	0	0	2^1	0
2	0	0	2^1	0	2^1
3	0	2^1	2	0	0
4	2^1	0	0	0	2^2
5	0	2^1	0	2^2	0

(1x2, 6x2^1, 2x2^2) c

	1	2	3	4	5
1	0	0	0	2^1	0
2	0	0	2^1	0	2^2
3	0	2^1	2	0	0
4	2^1	0	0	0	2^1
5	0	2^2	0	2^1	0

(1x2, 6x2^1, 2x2^2) d

	1	2	3	4	5
1	0	0	0	2^2	0
2	0	0	2^2	0	2^1
3	0	2^2	2	0	0
4	2^2	0	0	0	2^1
5	0	2^1	0	2^1	0

(1x2, 6x2^1, 2x2^2) e

	1	2	3	4	5
1	2	0	0	2^1	0
2	0	0	2^1	0	2^2
3	0	2^1	0	0	2^1
4	2^1	0	0	0	0
5	0	2^2	2^1	0	0

(1x2, 6x2^1, 2x2^2) f

	1	2	3	4	5
1	2	0	0	2^2	0
2	0	0	2^1	0	2^1
3	0	2^1	0	0	2^1
4	2^2	0	0	0	0
5	0	2^1	2^1	0	0

(1x2, 6x2^1, 2x2^2) g

	1	2	3	4	5
1	2	0	0	0	0
2	0	0	2^1	0	2^2
3	0	2^1	0	2^1	0
4	0	0	2^1	0	2^1
5	0	2^2	0	2^1	0

(1x**2**, 4x**2**^{1}, 4x**2**^{2}) **a**

	1	2	3	4	5
1	0	0	0	2^2	0
2	0	0	2^1	0	2^1
3	0	2^1	2	0	0
4	2^2	0	0	0	2^2
5	0	2^1	0	2^2	0

(1x**2**, 4x**2**^{1}, 4x**2**^{2}) **b**

	1	2	3	4	5
1	0	0	0	2^2	0
2	0	0	2^1	0	2^2
3	0	2^1	2	0	0
4	2^2	0	0	0	2^1
5	0	2^2	0	2^1	0

(1x**2**, 4x**2**^{1}, 4x**2**^{2}) **c**

	1	2	3	4	5
1	0	0	0	2^2	0
2	0	0	2^2	0	2^1
3	0	2^2	2	0	0
4	2^2	0	0	0	2^1
5	0	2^1	0	2^1	0

(1x**2**, 4x**2**^{1}, 4x**2**^{2}) **d**

	1	2	3	4	5
1	0	0	0	2^1	0
2	0	0	2^1	0	2^2
3	0	2^1	2	0	0
4	2^1	0	0	0	2^2
5	0	2^2	0	2^2	0

(1x**2**, 4x**2**^{1}, 4x**2**^{2}) **e**

	1	2	3	4	5
1	0	0	0	2^1	0
2	0	0	2^2	0	2^1
3	0	2^2	2	0	0
4	2^1	0	0	0	2^2
5	0	2^1	0	2^2	0

(1x**2**, 4x**2**^{1}, 4x**2**^{2}) **f**

	1	2	3	4	5
1	0	0	0	2^1	0
2	0	0	2^2	0	2^2
3	0	2^2	2	0	0
4	2^1	0	0	0	2^1
5	0	2^2	0	2^1	0

(1x**2**, 4x**2**^{1}, 4x**2**^{2}) **g**

	1	2	3	4	5
1	2	0	0	2^1	0
2	0	0	2^1	0	2^2
3	0	2^1	0	0	2^2
4	2^1	0	0	0	0
5	0	2^2	2^2	0	0

(1x**2**, 4x**2**^{1}, 4x**2**^{2}) **h**

	1	2	3	4	5
1	2	0	0	2^2	0
2	0	0	2^1	0	2^2
3	0	2^1	0	0	2^1
4	2^2	0	0	0	0
5	0	2^2	2^1	0	0

(1x**2**, 4x**2**^{1}, 4x**2**^{2}) **i**

	1	2	3	4	5
1	2	0	0	0	0
2	0	0	2^1	0	2^2
3	0	2^1	0	2^2	0
4	0	0	2^2	0	2^1
5	0	2^2	0	2^1	0

(1x**2**, 4x**2**^{1}, 4x**2**^{2}) **j**

	1	2	3	4	5
1	2	0	0	0	0
2	0	0	2^1	0	2^1
3	0	2^1	0	2^2	0
4	0	0	2^2	0	2^2
5	0	2^1	0	2^2	0

(1x**2**, 2x**2**^{1}, 6x**2**^{2}) **a**

	1	2	3	4	5
1	0	0	0	2^2	0
2	0	0	2^1	0	2^2
3	0	2^1	2	0	0
4	2^2	0	0	0	2^2
5	0	2^2	0	2^2	0

(1x**2**, 2x**2**^{1}, 6x**2**^{2}) **b**

	1	2	3	4	5
1	0	0	0	2^2	0
2	0	0	2^2	0	2^1
3	0	2^2	2	0	0
4	2^2	0	0	0	2^2
5	0	2^1	0	2^2	0

(1x**2**, 2x**2**^{1}, 6x**2**^{2}) **c**

	1	2	3	4	5
1	0	0	0	2^1	0
2	0	0	2^2	0	2^2
3	0	2^2	2	0	0
4	2^1	0	0	0	2^2
5	0	2^2	0	2^2	0

(1x**2**, 2x**2**^{1}, 6x**2**^{2}) **d**

	1	2	3	4	5
1	0	0	0	2^2	0
2	0	0	2^2	0	2^2
3	0	2^2	2	0	0
4	2^2	0	0	0	2^1
5	0	2^2	0	2^1	0

(1x**2**, 2x**2**^{1}, 6x**2**^{2}) **e**

	1	2	3	4	5
1	2	0	0	2^1	0
2	0	0	2^2	0	2^2
3	0	2^2	0	0	2^2
4	2^1	0	0	0	0
5	0	2^2	2^2	0	0

(1x**2**, 2x**2**^{1}, 6x**2**^{2}) **f**

	1	2	3	4	5
1	2	0	0	2^2	0
2	0	0	2^1	0	2^2
3	0	2^1	0	0	2^2
4	2^2	0	0	0	0
5	0	2^2	2^2	0	0

(1x**2**, 2x**2**^{1}, 6x**2**^{2}) **g**

	1	2	3	4	5
1	2	0	0	0	0
2	0	0	2^2	0	2^2
3	0	2^2	0	2^2	0
4	0	0	2^2	0	2^1
5	0	2^2	0	2^1	0

(1x**2**, 8x**2**^{2}) **a**

	1	2	3	4	5
1	0	0	0	2^2	0
2	0	0	2^2	0	2^2
3	0	2^2	2	0	0
4	2^2	0	0	0	2^2
5	0	2^2	0	2^2	0

(1x**2**, 8x**2**^{2}) **b**

	1	2	3	4	5
1	2	0	0	2^2	0
2	0	0	2^2	0	2^2
3	0	2^2	0	0	2^2
4	2^2	0	0	0	0
5	0	2^2	2^2	0	0

(1x**2**, 8x**2**^{2}) **c**

	1	2	3	4	5
1	2	0	0	0	0
2	0	0	2^2	0	2^2
3	0	2^2	0	2^2	0
4	0	0	2^2	0	2^2
5	0	2^2	0	2^2	0

$^2V_1{}^4V_5$ NG

$^2V_1{}^4V_6$ NG

$^2V_1{}^4V_7$ NG

$^2V_2{}^4V_1$

(6x**1**, 1x**2**)

	1	2	3
1	0	1	1
2	1	0	1
3	1	1	2

(2x**2**, 2x**2**^{1})

	1	2	3
1	2	2^1	0
2	2^1	0	0
3	0	0	2

(2x**2**, 2x**2**^{2})

	1	2	3
1	2	2^2	0
2	2^2	0	0
3	0	0	2

(4x**2**^{1})

	1	2	3
1	0	2^1	0
2	2^1	0	2^1
3	0	2^1	0

(2x**2**^{1}, 2x**2**^{2})

	1	2	3
1	0	2^2	0
2	2^2	0	2^1
3	0	2^1	0

(4x**2**^{2})

	1	2	3
1	0	2^2	0
2	2^2	0	2^2
3	0	2^2	0

$^2V_2{}^4V_2$

(8x**1**, 2x**2**) **a**

	1	2	3	4
1	0	1	1	0
2	1	0	0	1
3	1	0	2	1
4	0	1	1	2

(8x**1**, 2x**2**) **b**

	1	2	3	4
1	0	1	1	0
2	1	2	0	1
3	1	0	2	1
4	0	1	1	0

(8x**1**, 2x**2**^{1})

	1	2	3	4
1	0	1	1	2^1
2	1	0	0	1
3	1	0	0	1
4	2^1	1	1	0

(8x**1**, 2x**2**^{2})

	1	2	3	4
1	0	1	1	2^2
2	1	0	0	1
3	1	0	0	1
4	2^2	1	1	0

(6x**1**, 3x**2**)

	1	2	3	4
1	0	1	1	0
2	1	2	1	0
3	1	1	2	0
4	0	0	0	2

(6x**1**, 1x**2**, 2x**2^1**) **a**

	1	2	3	4
1	0	1	1	0
2	1	2	1	0
3	1	1	0	2^1
4	0	0	2^1	0

(6x**1**, 1x**2**, 2x**2^1**) **b**

	1	2	3	4
1	0	1	1	0
2	1	0	1	0
3	1	1	0	2^1
4	0	0	2^1	2

(6x**1**, 1x**2**, 2x**2^2**) **a**

	1	2	3	4
1	0	1	1	0
2	1	2	1	0
3	1	1	0	2^2
4	0	0	2^2	0

(6x**1**, 1x**2**, 2x**2^2**) **b**

	1	2	3	4
1	0	1	1	0
2	1	0	1	0
3	1	1	0	2^2
4	0	0	2^2	2

(4x**2**, 2x**2^1**)

	1	2	3	4
1	2	0	0	0
2	0	2	0	0
3	0	0	2	2^1
4	0	0	2^1	2

(4x**2**, 2x**2^2**)

	1	2	3	4
1	2	0	0	0
2	0	2	0	0
3	0	0	2	2^2
4	0	0	2^2	2

(2x**2**, 4x**2^1**) **a**

	1	2	3	4
1	0	2^1	0	0
2	2^1	0	0	0
3	0	0	2	2^1
4	0	0	2^1	2

(2x**2**, 4x**2^1**) **b**

	1	2	3	4
1	2	0	0	0
2	0	0	0	2^1
3	0	0	2	2^1
4	0	2^1	2^1	0

(2x**2**, 4x**2^1**) **c**

	1	2	3	4
1	2	2^1	0	0
2	2^1	0	0	0
3	0	0	2	2^1
4	0	0	2^1	0

(2x**2**, 2x**2^1**, 2x**2^2**) **a**

	1	2	3	4
1	0	2^1	0	0
2	2^1	0	0	0
3	0	0	2	2^2
4	0	0	2^2	2

(2x**2**, 2x**2^1**, 2x**2^2**) **b**

	1	2	3	4
1	0	2^2	0	0
2	2^2	0	0	0
3	0	0	2	2^1
4	0	0	2^1	2

(2x**2**, 2x**2^1**, 2x**2^2**) **c**

	1	2	3	4
1	2	0	0	0
2	0	0	0	2^1
3	0	0	2	2^2
4	0	2^1	2^2	0

(2x**2**, 2x**2^1**, 2x**2^2**) **d**

	1	2	3	4
1	2	0	0	0
2	0	0	0	2^2
3	0	0	2	2^1
4	0	2^2	2^1	0

(2x**2**, 2x**2^1**, 2x**2^2**) **e**

	1	2	3	4
1	2	2^2	0	0
2	2^2	0	0	0
3	0	0	2	2^1
4	0	0	2^1	0

(2x**2**, 4x**2^2**) **a**

	1	2	3	4
1	0	2^2	0	0
2	2^2	0	0	0
3	0	0	2	2^2
4	0	0	2^2	2

(2x**2**, 4x**2^2**) **b**

	1	2	3	4
1	2	0	0	0
2	0	0	0	2^2
3	0	0	2	2^2
4	0	2^2	2^2	0

(2x**2**, 4x**2^2**) **c**

	1	2	3	4
1	2	2^2	0	0
2	2^2	0	0	0
3	0	0	2	2^2
4	0	0	2^2	0

(6x**2^1**)

	1	2	3	4
1	0	2^1	0	0
2	2^1	0	0	2^1
3	0	0	0	2^1
4	0	2^1	2^1	0

(4x**2^1**, 2x**2^2**) **a**

	1	2	3	4
1	0	2^2	0	0
2	2^2	0	0	2^1
3	0	0	0	2^1
4	0	2^1	2^1	0

(4x**2^1**, 2x**2^2**) **b**

	1	2	3	4
1	0	2^1	0	0
2	2^1	0	0	2^2
3	0	0	0	2^1
4	0	2^2	2^1	0

(2x**2^1**, 4x**2^2**) **a**

	1	2	3	4
1	0	2^2	0	0
2	2^2	0	0	2^1
3	0	0	0	2^2
4	0	2^1	2^2	0

(2x**2^1**, 4x**2^2**) **b**

	1	2	3	4
1	0	2^2	0	0
2	2^2	0	0	2^2
3	0	0	0	2^1
4	0	2^2	2^1	0

(6x**2^2**)

	1	2	3	4
1	0	2^2	0	0
2	2^2	0	0	2^2
3	0	0	0	2^2
4	0	2^2	2^2	0

$^2V_2{}^4V_3$

(14x**1**, 1x**2**)

	1	2	3	4	5
1	2	0	1	1	0
2	0	0	1	1	0
3	1	1	0	1	1
4	1	1	1	0	1
5	0	0	1	1	0

(12x**1**, 2x**2**) **a**

	1	2	3	4	5
1	2	1	1	0	0
2	1	0	1	0	0
3	1	1	0	1	1
4	0	0	1	0	1
5	0	0	1	1	2

(12x**1**, 2x**2**) **b**

	1	2	3	4	5
1	2	1	1	0	0
2	1	2	1	0	0
3	1	1	0	1	1
4	0	0	1	0	1
5	0	0	1	1	0

(12x**1**, 2x**2^1**)

	1	2	3	4	5
1	0	0	1	1	0
2	0	0	2^1	1	1
3	1	2^1	0	1	0
4	1	1	1	0	1
5	0	1	0	1	0

(12x**1**, 2x**2^2**)

	1	2	3	4	5
1	0	0	1	1	0
2	0	0	2^2	1	1
3	1	2^2	0	1	0
4	1	1	1	0	1
5	0	1	0	1	0

(10x**1**, 3x**2**) **a**

	1	2	3	4	5
1	2	0	1	1	0
2	0	2	1	0	1
3	1	1	2	0	0
4	1	0	0	0	1
5	0	1	0	1	0

(10x**1**, 3x**2**) **a**

	1	2	3	4	5
1	2	0	1	1	0
2	0	0	1	0	1
3	1	1	2	0	0
4	1	0	0	0	1
5	0	1	0	1	2

(10x**1**, 1x**2**, 2x**2^1**) **a**

	1	2	3	4	5
1	0	1	0	2^1	1
2	1	0	1	0	0
3	0	1	0	1	0
4	2^1	0	1	0	1
5	1	0	0	1	2

(10x**1**, 1x**2**, 2x**2^1**) **b**

	1	2	3	4	5
1	0	1	0	2^1	1
2	1	0	1	0	0
3	0	1	2	1	0
4	2^1	0	1	0	1
5	1	0	0	1	0

(10x**1**, 1x**2**, 2x**2^2**) **a**

	1	2	3	4	5
1	0	1	0	2^2	1
2	1	0	1	0	0
3	0	1	0	1	0
4	2^2	0	1	0	1
5	1	0	0	1	2

(10x**1**, 1x**2**, 2x**2^2**) **b**

	1	2	3	4	5
1	0	1	0	2^2	1
2	1	0	1	0	0
3	0	1	2	1	0
4	2^2	0	1	0	1
5	1	0	0	1	0

(8x**1**, 4x**2**)

	1	2	3	4	5
1	0	1	0	1	0
2	1	2	1	0	0
3	0	1	2	1	0
4	1	0	1	2	0
5	0	0	0	0	2

(8x**1**, 2x**2**, 2x**2^1**) **a**

	1	2	3	4	5
1	2	0	1	1	0
2	0	0	1	1	2^1
3	1	1	0	0	0
4	1	1	0	0	0
5	0	2^1	0	0	2

(8x**1**, 2x**2**, 2x**2^1**) **b**

	1	2	3	4	5
1	0	0	1	1	0
2	0	0	1	1	2^1
3	1	1	0	0	0
4	1	1	0	2	0
5	0	2^1	0	0	2

(8x**1**, 2x**2**, 2x**2^1**) **c**

	1	2	3	4	5
1	2	0	1	1	0
2	0	0	1	1	2^1
3	1	1	0	0	0
4	1	1	0	2	0
5	0	2^1	0	0	0

(8x**1**, 2x**2**, 2x**2**^{1}) **d**

	1	2	3	4	5
1	0	0	1	1	0
2	0	0	1	1	2^1
3	1	1	2	0	0
4	1	1	0	2	0
5	0	2^1	0	0	0

(8x**1**, 2x**2**, 2x**2**^{1}) **e**

	1	2	3	4	5
1	0	0	1	1	0
2	0	2	1	1	0
3	1	1	0	2^1	0
4	1	1	2^1	0	0
5	0	0	0	0	2

(8x**1**, 2x**2**, 2x**2**^{2}) **a**

	1	2	3	4	5
1	2	0	1	1	0
2	0	0	1	1	2^2
3	1	1	0	0	0
4	1	1	0	0	0
5	0	2^2	0	0	2

(8x**1**, 2x**2**, 2x**2**^{2}) **b**

	1	2	3	4	5
1	0	0	1	1	0
2	0	0	1	1	2^2
3	1	1	0	0	0
4	1	1	0	2	0
5	0	2^2	0	0	2

(8x**1**, 2x**2**, 2x**2**^{2}) **c**

	1	2	3	4	5
1	2	0	1	1	0
2	0	0	1	1	2^2
3	1	1	0	0	0
4	1	1	0	2	0
5	0	2^2	0	0	0

(8x**1**, 2x**2**, 2x**2**^{2}) **d**

	1	2	3	4	5
1	0	0	1	1	0
2	0	0	1	1	2^2
3	1	1	2	0	0
4	1	1	0	2	0
5	0	2^2	0	0	0

(8x**1**, 2x**2**, 2x**2**^{2}) **e**

	1	2	3	4	5
1	0	0	1	1	0
2	0	2	1	1	0
3	1	1	0	2^2	0
4	1	1	2^2	0	0
5	0	0	0	0	2

(8x**1**, 4x**2**^{1}) **a**

	1	2	3	4	5
1	0	1	0	1	2^1
2	1	0	1	0	0
3	0	1	0	1	0
4	1	0	1	0	2^1
5	2^1	0	0	2^1	0

(8x**1**, 4x**2**^{1}) **b**

	1	2	3	4	5
1	0	1	1	0	2^1
2	1	0	0	1	0
3	1	0	0	1	0
4	0	1	1	0	2^1
5	2^1	0	0	2^1	0

(8x**1**, 4x**2**^{1}) **c**

	1	2	3	4	5
1	0	0	1	1	0
2	0	0	1	1	2^1
3	1	1	0	2^1	0
4	1	1	2^1	0	0
5	0	2^1	0	0	0

(8x**1**, 2x**2**^{1}, 2x**2**^{2}) **a**

	1	2	3	4	5
1	0	1	0	1	2^2
2	1	0	1	0	0
3	0	1	0	1	0
4	1	0	1	0	2^1
5	2^2	0	0	2^1	0

(8x**1**, 2x**2**^{1}, 2x**2**^{2}) **b**

	1	2	3	4	5
1	0	1	1	0	2^2
2	1	0	0	1	0
3	1	0	0	1	0
4	0	1	1	0	2^1
5	2^2	0	0	2^1	0

(8x**1**, 2x**2**^{1}, 2x**2**^{2}) **c**

	1	2	3	4	5
1	0	0	1	1	0
2	0	0	1	1	2^1
3	1	1	0	2^2	0
4	1	1	2^2	0	0
5	0	2^1	0	0	0

(8x**1**, 2x**2**^{1}, 2x**2**^{2}) **d**

	1	2	3	4	5
1	0	0	1	1	0
2	0	0	1	1	2^1
3	1	1	0	2^1	0
4	1	1	2^1	0	0
5	0	2^1	0	0	0

(8x**1**, 4x**2**^{2}) **a**

	1	2	3	4	5
1	0	1	0	1	2^2
2	1	0	1	0	0
3	0	1	0	1	0
4	1	0	1	0	2^2
5	2^2	0	0	2^2	0

(8x**1**, 4x**2**^{2}) **b**

	1	2	3	4	5
1	0	1	1	0	2^2
2	1	0	0	1	0
3	1	0	0	1	0
4	0	1	1	0	2^2
5	2^2	0	0	2^2	0

(8x**1**, 4x**2**^{2}) **c**

	1	2	3	4	5
1	0	0	1	1	0
2	0	0	1	1	2^2
3	1	1	0	2^2	0
4	1	1	2^2	0	0
5	0	2^2	0	0	0

(6x**1**, 5x**2**)

	1	2	3	4	5
1	2	0	0	0	0
2	0	2	1	1	0
3	0	1	2	1	0
4	0	1	1	2	0
5	0	0	0	0	2

(6x**1**, 3x**2**, 2x**2**^{1}) **a**

	1	2	3	4	5
1	0	0	0	0	2^1
2	0	2	1	1	0
3	0	1	2	1	0
4	0	1	1	0	0
5	2^1	0	0	0	2

(6x**1**, 3x**2**, 2x**2**^{1}) **b**

	1	2	3	4	5
1	0	0	0	0	2^1
2	0	2	1	1	0
3	0	1	2	1	0
4	0	1	1	2	0
5	2^1	0	0	0	0

(6x**1**, 3x**2**, 2x**2**^{1}) **c**

	1	2	3	4	5
1	2	0	0	0	2^1
2	0	2	1	1	0
3	0	1	0	1	0
4	0	1	1	0	0
5	2^1	0	0	0	2

(6x**1**, 3x**2**, 2x**2**^{1}) **d**

	1	2	3	4	5
1	0	0	0	2^1	0
2	0	2	1	1	0
3	0	1	2	1	0
4	2^1	1	1	0	0
5	0	0	0	0	2

(6x**1**, 3x**2**, 2x**2**^{1}) **e**

	1	2	3	4	5
1	2	0	0	2^1	0
2	0	2	1	1	0
3	0	1	0	1	0
4	2^1	1	1	0	0
5	0	0	0	0	2

(6x**1**, 3x**2**, 2x**2**^{2}) **a**

	1	2	3	4	5
1	0	0	0	0	2^2
2	0	2	1	1	0
3	0	1	2	1	0
4	0	1	1	0	0
5	2^2	0	0	0	2

(6x**1**, 3x**2**, 2x**2**^{2}) **b**

	1	2	3	4	5
1	0	0	0	0	2^2
2	0	2	1	1	0
3	0	1	2	1	0
4	0	1	1	2	0
5	2^2	0	0	0	0

(6x**1**, 3x**2**, 2x**2**^{2}) **c**

	1	2	3	4	5
1	2	0	0	0	2^2
2	0	2	1	1	0
3	0	1	0	1	0
4	0	1	1	0	0
5	2^2	0	0	0	2

(6x**1**, 3x**2**, 2x**2**^{2}) **d**

	1	2	3	4	5
1	0	0	0	2^2	0
2	0	2	1	1	0
3	0	1	2	1	0
4	2^2	1	1	0	0
5	0	0	0	0	2

(6x**1**, 3x**2**, 2x**2**^{2}) **e**

	1	2	3	4	5
1	2	0	0	2^2	0
2	0	2	1	1	0
3	0	1	0	1	0
4	2^2	1	1	0	0
5	0	0	0	0	2

(6x**1**, 1x**2**, 4x**2**^{1}) **a**

	1	2	3	4	5
1	0	0	0	2^1	0
2	0	0	1	1	2^1
3	0	1	2	1	0
4	2^1	1	1	0	0
5	0	2^1	0	0	0

(6x**1**, 1x**2**, 4x**2**^{1}) **b**

	1	2	3	4	5
1	0	0	0	2^1	2^1
2	0	0	1	1	0
3	0	1	0	1	0
4	2^1	1	1	0	0
5	2^1	0	0	0	2

(6x**1**, 1x**2**, 4x**2**^{1}) **c**

	1	2	3	4	5
1	0	0	0	2^1	2^1
2	0	2	1	1	0
3	0	1	0	1	0
4	2^1	1	1	0	0
5	2^1	0	0	0	0

(6x**1**, 1x**2**, 4x**2**^{1}) **d**

	1	2	3	4	5
1	0	0	0	2^1	0
2	0	0	1	1	2^1
3	0	1	0	1	0
4	2^1	1	1	0	0
5	0	2^1	0	0	2

(6x**1**, 1x**2**, 4x**2**^{1}) **e**

	1	2	3	4	5
1	0	1	0	1	2^1
2	1	0	0	1	0
3	0	0	2	0	0
4	1	1	0	0	2^1
5	2^1	0	0	2^1	0

(6x**1**, 1x**2**, 2x**2**^{1}, 2x**2**^{2}) **a**

	1	2	3	4	5
1	0	0	0	2^2	0
2	0	0	1	1	2^1
3	0	1	2	1	0
4	2^2	1	1	0	0
5	0	2^1	0	0	0

(6x**1**, 1x**2**, 2x**2**^{1}, 2x**2**^{2}) **b**

	1	2	3	4	5
1	0	0	0	2^2	2^1
2	0	0	1	1	0
3	0	1	0	1	0
4	2^2	1	1	0	0
5	2^1	0	0	0	2

(6x**1**, 1x**2**, 2x**2**^{1}, 2x**2**^{2}) **c**

	1	2	3	4	5
1	0	0	0	2^1	2^2
2	0	0	1	1	0
3	0	1	0	1	0
4	2^1	1	1	0	0
5	2^2	0	0	0	2

(6x**1**, 1x**2**, 2x**2**^{1}, 2x**2**^{2}) **d**

	1	2	3	4	5
1	0	0	0	2^2	2^1
2	0	2	1	1	0
3	0	1	0	1	0
4	2^2	1	1	0	0
5	2^1	0	0	0	0

(6x**1**, 1x**2**, 2x**2**^{1}, 2x**2**^{2}) **e**

	1	2	3	4	5
1	0	0	0	2^1	2^2
2	0	2	1	1	0
3	0	1	0	1	0
4	2^1	1	1	0	0
5	2^2	0	0	0	0

(6x**1**, 1x**2**, 2x**2**^{1}, 2x**2**^{2}) **f**

	1	2	3	4	5
1	0	0	0	2^2	0
2	0	0	1	1	2^1
3	0	1	0	1	0
4	2^2	1	1	0	0
5	0	2^1	0	0	2

(6x**1**, 1x**2**, 2x**2**^{1}, 2x**2**^{2}) **g**

	1	2	3	4	5
1	0	0	0	2^1	0
2	0	0	1	1	2^2
3	0	1	0	1	0
4	2^1	1	1	0	0
5	0	2^2	0	0	2

(6x**1**, 1x**2**, 2x**2**^{1}, 2x**2**^{2}) **h**

	1	2	3	4	5
1	0	1	0	1	2^1
2	1	0	0	1	0
3	0	0	2	0	0
4	1	1	0	0	2^2
5	2^1	0	0	2^2	0

(6x**1**, 1x**2**, 4x**2**^{2}) **a**

	1	2	3	4	5
1	0	0	0	2^2	0
2	0	0	1	1	2^2
3	0	1	2	1	0
4	2^2	1	1	0	0
5	0	2^2	0	0	0

(6x**1**, 1x**2**, 4x**2**^{2}) **b**

	1	2	3	4	5
1	0	0	0	2^2	2^2
2	0	0	1	1	0
3	0	1	0	1	0
4	2^2	1	1	0	0
5	2^2	0	0	0	2

(6x**1**, 1x**2**, 4x**2**^{2}) **c**

	1	2	3	4	5
1	0	0	0	2^2	2^2
2	0	2	1	1	0
3	0	1	0	1	0
4	2^2	1	1	0	0
5	2^2	0	0	0	0

(6x**1**, 1x**2**, 4x**2**^{2}) **d**

	1	2	3	4	5
1	0	0	0	2^2	0
2	0	0	1	1	2^2
3	0	1	0	1	0
4	2^2	1	1	0	0
5	0	2^2	0	0	2

(6x**1**, 1x**2**, 4x**2**^{2}) **e**

	1	2	3	4	5
1	0	1	0	1	2^2
2	1	0	0	1	0
3	0	0	2	0	0
4	1	1	0	0	2^2
5	2^2	0	0	2^2	0

(4x**2**, 4x**2**^{1}) **a**

	1	2	3	4	5
1	0	0	0	2^1	2^1
2	0	2	0	0	0
3	0	0	2	0	0
4	2^1	0	0	2	0
5	2^1	0	0	0	2

(4x**2**, 4x**2**^{1}) **b**

	1	2	3	4	5
1	0	2^1	0	0	0
2	2^1	2	0	0	0
3	0	0	2	2^1	0
4	0	0	2^1	2	0
5	0	0	0	0	2

(4x**2**, 2x**2**^{1}, 2x**2**^{2}) **a**

	1	2	3	4	5
1	0	0	0	2^2	2^1
2	0	2	0	0	0
3	0	0	2	0	0
4	2^2	0	0	2	0
5	2^1	0	0	0	2

(4x**2**, 2x**2**^{1}, 2x**2**^{2}) **b**

	1	2	3	4	5
1	0	2^1	0	0	0
2	2^1	2	0	0	0
3	0	0	2	2^2	0
4	0	0	2^2	2	0
5	0	0	0	0	2

(4x**2**, 2x**2**^{1}, 2x**2**^{2}) **c**

	1	2	3	4	5
1	0	2^2	0	0	0
2	2^2	2	0	0	0
3	0	0	2	2^1	0
4	0	0	2^1	2	0
5	0	0	0	0	2

(4x**2**, 4x**2**^{2}) **a**

	1	2	3	4	5
1	0	0	0	2^2	2^2
2	0	2	0	0	0
3	0	0	2	0	0
4	2^2	0	0	2	0
5	2^2	0	0	0	2

(4x**2**, 4x**2**^{2}) **b**

	1	2	3	4	5
1	0	2^2	0	0	0
2	2^2	2	0	0	0
3	0	0	2	2^2	0
4	0	0	2^2	2	0
5	0	0	0	0	2

(2x**2**, 6x**2**^{1}) **a**

	1	2	3	4	5
1	0	2^1	2^1	0	0
2	2^1	0	0	0	0
3	2^1	0	0	0	0
4	0	0	0	2	2^1
5	0	0	0	2^1	2

(2x**2**, 6x**2**^{1}) **b**

	1	2	3	4	5
1	0	2^1	2^1	0	0
2	2^1	0	0	0	2^1
3	2^1	0	2	0	0
4	0	0	0	2	0
5	0	2^1	0	0	0

(2x**2**, 6x**2**^{1}) **c**

	1	2	3	4	5
1	0	2^1	2^1	0	0
2	2^1	2	0	0	0
3	2^1	0	2	0	0
4	0	0	0	0	2^1
5	0	0	0	2^1	0

(2x**2**, 6x**2**^{1}) **d**

	1	2	3	4	5
1	0	2^1	2^1	0	0
2	2^1	0	0	0	0
3	2^1	0	2	0	0
4	0	0	0	0	2^1
5	0	0	0	2^1	2

(2x**2**, 6x**2**^{1}) **e**

	1	2	3	4	5
1	0	2^1	2^1	0	0
2	2^1	0	2^1	0	0
3	2^1	2^1	0	0	0
4	0	0	0	2	0
5	0	0	0	0	2

(2x**2**, 4x**2**^{1}, 2x**2**^{2}) **a**

	1	2	3	4	5
1	0	2^1	2^2	0	0
2	2^1	0	0	0	0
3	2^2	0	0	0	0
4	0	0	0	2	2^1
5	0	0	0	2^1	2

(2x**2**, 4x**2**^{1}, 2x**2**^{2}) **b**

	1	2	3	4	5
1	0	2^1	2^1	0	0
2	2^1	0	0	0	0
3	2^1	0	0	0	0
4	0	0	0	2	2^2
5	0	0	0	2^2	2

(2x**2**, 4x**2**^{1}, 2x**2**^{2}) **c**

	1	2	3	4	5
1	0	2^1	2^1	0	0
2	2^1	0	0	0	2^2
3	2^1	0	2	0	0
4	0	0	0	2	0
5	0	2^2	0	0	0

(2x**2**, 4x**2**^{1}, 2x**2**^{2}) **d**

	1	2	3	4	5
1	0	2^2	2^1	0	0
2	2^2	0	0	0	2^1
3	2^1	0	2	0	0
4	0	0	0	2	0
5	0	2^1	0	0	0

(2x**2**, 4x**2**^{1}, 2x**2**^{2}) **e**

	1	2	3	4	5
1	0	2^1	2^2	0	0
2	2^1	0	0	0	2^1
3	2^2	0	2	0	0
4	0	0	0	2	0
5	0	2^1	0	0	0

(2x**2**, 4x**2**^{1}, 2x**2**^{2}) **f**

	1	2	3	4	5
1	0	2^1	2^1	0	0
2	2^1	2	0	0	0
3	2^1	0	2	0	0
4	0	0	0	0	2^2
5	0	0	0	2^2	0

(2x**2**, 4x**2**^{1}, 2x**2**^{2}) **g**

	1	2	3	4	5
1	0	2^2	2^1	0	0
2	2^2	2	0	0	0
3	2^1	0	2	0	0
4	0	0	0	0	2^1
5	0	0	0	2^1	0

(2x**2**, 4x**2**^{1}, 2x**2**^{2}) **h**

	1	2	3	4	5
1	0	2^1	2^1	0	0
2	2^1	0	0	0	0
3	2^1	0	2	0	0
4	0	0	0	0	2^2
5	0	0	0	2^2	2

(2x**2**, 4x**2**^{1}, 2x**2**^{2}) **i**

	1	2	3	4	5
1	0	2^2	2^1	0	0
2	2^2	0	0	0	0
3	2^1	0	2	0	0
4	0	0	0	0	2^1
5	0	0	0	2^1	2

(2x**2**, 4x**2**^{1}, 2x**2**^{2}) **j**

	1	2	3	4	5
1	0	2^1	2^2	0	0
2	2^1	0	0	0	0
3	2^2	0	2	0	0
4	0	0	0	0	2^1
5	0	0	0	2^1	2

(2x**2**, 4x**2**^{1}, 2x**2**^{2}) **k**

	1	2	3	4	5
1	0	2^1	2^2	0	0
2	2^1	0	2^1	0	0
3	2^2	2^1	0	0	0
4	0	0	0	2	0
5	0	0	0	0	2

(2x**2**, 2x**2**^{1}, 4x**2**^{2}) **a**

	1	2	3	4	5
1	0	2^1	2^2	0	0
2	2^1	0	0	0	0
3	2^2	0	0	0	0
4	0	0	0	2	2^2
5	0	0	0	2^2	2

(2x**2**, 2x**2^1**, 4x**2^2**) **b**

	1	2	3	4	5
1	0	2^2	2^2	0	0
2	2^2	0	0	0	0
3	2^2	0	0	0	0
4	0	0	0	2	2^1
5	0	0	0	2^1	2

(2x**2**, 2x**2^1**, 4x**2^2**) **c**

	1	2	3	4	5
1	0	2^2	2^1	0	0
2	2^2	0	0	0	2^2
3	2^1	0	2	0	0
4	0	0	0	2	0
5	0	2^2	0	0	0

(2x**2**, 2x**2^1**, 4x**2^2**) **d**

	1	2	3	4	5
1	0	2^1	2^2	0	0
2	2^1	0	0	0	2^2
3	2^2	0	2	0	0
4	0	0	0	2	0
5	0	2^2	0	0	0

(2x**2**, 2x**2^1**, 4x**2^2**) **e**

	1	2	3	4	5
1	0	2^2	2^2	0	0
2	2^2	0	0	0	2^1
3	2^2	0	2	0	0
4	0	0	0	2	0
5	0	2^1	0	0	0

(2x**2**, 2x**2^1**, 4x**2^2**) **f**

	1	2	3	4	5
1	0	2^1	2^2	0	0
2	2^1	2	0	0	0
3	2^2	0	2	0	0
4	0	0	0	0	2^2
5	0	0	0	2^2	0

(2x**2**, 2x**2^1**, 4x**2^2**) **g**

	1	2	3	4	5
1	0	2^2	2^2	0	0
2	2^2	2	0	0	0
3	2^2	0	2	0	0
4	0	0	0	0	2^1
5	0	0	0	2^1	0

(2x**2**, 2x**2^1**, 4x**2^2**) **h**

	1	2	3	4	5
1	0	2^2	2^1	0	0
2	2^2	0	0	0	0
3	2^1	0	2	0	0
4	0	0	0	0	2^2
5	0	0	0	2^2	2

(2x**2**, 2x**2^1**, 4x**2^2**) **i**

	1	2	3	4	5
1	0	2^1	2^2	0	0
2	2^1	0	0	0	0
3	2^2	0	2	0	0
4	0	0	0	0	2^2
5	0	0	0	2^2	2

(2x**2**, 2x**2^1**, 4x**2^2**) **j**

	1	2	3	4	5
1	0	2^2	2^2	0	0
2	2^2	0	0	0	0
3	2^2	0	2	0	0
4	0	0	0	0	2^1
5	0	0	0	2^1	2

(2x**2**, 2x**2^1**, 4x**2^2**) **k**

	1	2	3	4	5
1	0	2^2	2^2	0	0
2	2^2	0	2^1	0	0
3	2^2	2^1	0	0	0
4	0	0	0	2	0
5	0	0	0	0	2

(2x**2**, 6x**2^2**) **a**

	1	2	3	4	5
1	0	2^2	2^2	0	0
2	2^2	0	0	0	0
3	2^2	0	0	0	0
4	0	0	0	2	2^2
5	0	0	0	2^2	2

(2x**2**, 6x**2^2**) **b**

	1	2	3	4	5
1	0	2^2	2^2	0	0
2	2^2	0	0	0	2^2
3	2^2	0	2	0	0
4	0	0	0	2	0
5	0	2^2	0	0	0

(2x**2**, 6x**2^2**) **c**

	1	2	3	4	5
1	0	2^2	2^2	0	0
2	2^2	2	0	0	0
3	2^2	0	2	0	0
4	0	0	0	0	2^2
5	0	0	0	2^2	0

(2x**2**, 6x**2^2**) **d**

	1	2	3	4	5
1	0	2^2	2^2	0	0
2	2^2	0	0	0	0
3	2^2	0	2	0	0
4	0	0	0	0	2^2
5	0	0	0	2^2	2

(2x**2**, 6x**2^2**) **e**

	1	2	3	4	5
1	0	2^2	2^2	0	0
2	2^2	0	2^2	0	0
3	2^2	2^2	0	0	0
4	0	0	0	2	0
5	0	0	0	0	2

(8x**2^1**) **a**

	1	2	3	4	5
1	0	2^1	2^1	0	0
2	2^1	0	2^1	0	0
3	2^1	2^1	0	0	0
4	0	0	0	0	2^1
5	0	0	0	2^1	0

(8x**2^1**) **b**

	1	2	3	4	5
1	0	2^1	2^1	0	0
2	2^1	0	0	0	2^1
3	2^1	0	0	2^1	0
4	0	0	2^1	0	0
5	0	2^1	0	0	0

(6x**2^1**, 2x**2^2**) **a**

	1	2	3	4	5
1	0	2^1	2^1	0	0
2	2^1	0	2^1	0	0
3	2^1	2^1	0	0	0
4	0	0	0	0	2^2
5	0	0	0	2^2	0

(6x**2^1**, 2x**2^2**) **b**

	1	2	3	4	5
1	0	2^2	2^1	0	0
2	2^2	0	2^1	0	0
3	2^1	2^1	0	0	0
4	0	0	0	0	2^1
5	0	0	0	2^1	0

(6x**2^1**, 2x**2^2**) **c**

	1	2	3	4	5
1	0	2^1	2^1	0	0
2	2^1	0	0	0	2^2
3	2^1	0	0	2^1	0
4	0	0	2^1	0	0
5	0	2^2	0	0	0

(6x**2^1**, 2x**2^2**) **d**

	1	2	3	4	5
1	0	2^1	2^2	0	0
2	2^1	0	0	0	2^1
3	2^2	0	0	2^1	0
4	0	0	2^1	0	0
5	0	2^1	0	0	0

(4x**2^1**, 4x**2^2**) **a**

	1	2	3	4	5
1	0	2^2	2^1	0	0
2	2^2	0	2^1	0	0
3	2^1	2^1	0	0	0
4	0	0	0	0	2^2
5	0	0	0	2^2	0

(4x**2^1**, 4x**2^2**) **b**

	1	2	3	4	5
1	0	2^2	2^2	0	0
2	2^2	0	2^1	0	0
3	2^2	2^1	0	0	0
4	0	0	0	0	2^1
5	0	0	0	2^1	0

(4x**2^1**, 4x**2^2**) **c**

	1	2	3	4	5
1	0	2^2	2^1	0	0
2	2^2	0	0	0	2^2
3	2^1	0	0	2^1	0
4	0	0	2^1	0	0
5	0	2^2	0	0	0

(4x**2^1**, 4x**2^2**) **d**

	1	2	3	4	5
1	0	2^1	2^2	0	0
2	2^1	0	0	0	2^2
3	2^2	0	0	2^1	0
4	0	0	2^1	0	0
5	0	2^2	0	0	0

(4x**2^1**, 4x**2^2**) **e**

	1	2	3	4	5
1	0	2^1	2^1	0	0
2	2^1	0	0	0	2^2
3	2^1	0	0	2^2	0
4	0	0	2^2	0	0
5	0	2^2	0	0	0

(4x**2^1**, 4x**2^2**) **f**

	1	2	3	4	5
1	0	2^2	2^2	0	0
2	2^2	0	0	0	2^1
3	2^2	0	0	2^1	0
4	0	0	2^1	0	0
5	0	2^1	0	0	0

(2x**2^1**, 6x**2^2**) **a**

	1	2	3	4	5
1	0	2^2	2^2	0	0
2	2^2	0	2^1	0	0
3	2^2	2^1	0	0	0
4	0	0	0	0	2^2
5	0	0	0	2^2	0

(2x**2^1**, 6x**2^2**) **b**

	1	2	3	4	5
1	0	2^2	2^2	0	0
2	2^2	0	2^2	0	0
3	2^2	2^2	0	0	0
4	0	0	0	0	2^1
5	0	0	0	2^1	0

(2x**2^1**, 6x**2^2**) **c**

	1	2	3	4	5
1	0	2^2	2^2	0	0
2	2^2	0	0	0	2^2
3	2^2	0	0	2^1	0
4	0	0	2^1	0	0
5	0	2^2	0	0	0

(2x**2^1**, 6x**2^2**) **d**

	1	2	3	4	5
1	0	2^2	2^1	0	0
2	2^2	0	0	0	2^2
3	2^1	0	0	2^2	0
4	0	0	2^2	0	0
5	0	2^2	0	0	0

(8x**2^2**) **a**

	1	2	3	4	5
1	0	2^2	2^2	0	0
2	2^2	0	2^2	0	0
3	2^2	2^2	0	0	0
4	0	0	0	0	2^2
5	0	0	0	2^2	0

(8x**2^2**) **b**

	1	2	3	4	5
1	0	2^2	2^2	0	0
2	2^2	0	0	0	2^2
3	2^2	0	0	2^2	0
4	0	0	2^2	0	0
5	0	2^2	0	0	0

${}^2V_2{}^4V_4$	NG
${}^2V_2{}^4V_5$	NG

$^{2}V_{2}{}^{4}V_{6}$	NG

$^{2}V_{3}{}^{4}V_{1}$

(8x**1**, 1x**2**)

	1	2	3	4
1	0	0	1	1
2	0	0	1	1
3	1	1	0	0
4	1	1	0	2

(6x**1**, 2x**2**)

	1	2	3	4
1	0	1	0	1
2	1	0	0	1
3	0	0	2	0
4	1	1	0	2

(6x**1**, 2x**2**^{1})

	1	2	3	4
1	0	1	0	1
2	1	0	2^1	1
3	0	2^1	0	0
4	1	1	0	0

(6x**1**, 2x**2**^{2})

	1	2	3	4
1	0	1	0	1
2	1	0	2^2	1
3	0	2^2	0	0
4	1	1	0	0

(3x**2**, 2x**2**^{1})

	1	2	3	4
1	2	0	0	0
2	0	2	2^1	0
3	0	2^1	0	0
4	0	0	0	2

(3x**2**, 2x**2**^{2})

	1	2	3	4
1	2	0	0	0
2	0	2	2^2	0
3	0	2^2	0	0
4	0	0	0	2

(1x**2**, 4x**2**^{1}) **a**

	1	2	3	4
1	0	0	0	2^1
2	0	2	2^1	0
3	0	2^1	0	0
4	2^1	0	0	0

(1x**2**, 4x**2**^{1}) **b**

	1	2	3	4
1	2	0	0	0
2	0	0	2^1	0
3	0	2^1	0	2^1
4	0	0	2^1	0

(1x**2**, 2x**2**^{1}, 2x**2**^{2}) **a**

	1	2	3	4
1	0	0	0	2^1
2	0	2	2^2	0
3	0	2^2	0	0
4	2^1	0	0	0

(1x**2**, 2x**2**^{1}, 2x**2**^{2}) **b**

	1	2	3	4
1	0	0	0	2^2
2	0	2	2^1	0
3	0	2^1	0	0
4	2^2	0	0	0

(1x**2**, 4x**2**^{1}) **c**

	1	2	3	4
1	2	0	0	0
2	0	0	2^2	0
3	0	2^2	0	2^1
4	0	0	2^1	0

(1x**2**, 4x**2**^{2}) **a**

	1	2	3	4
1	0	0	0	2^2
2	0	2	2^2	0
3	0	2^2	0	0
4	2^2	0	0	0

(1x**2**, 4x**2**^{2}) **b**

	1	2	3	4
1	2	0	0	0
2	0	0	2^2	0
3	0	2^2	0	2^2
4	0	0	2^2	0

$^{2}V_{3}{}^{4}V_{2}$

(14x**1**)

	1	2	3	4	5
1	0	0	1	1	0
2	0	0	1	1	0
3	1	1	0	1	1
4	1	1	1	0	1
5	0	0	1	1	0

(12x**1**, 1x**2**)

	1	2	3	4	5
1	2	1	1	0	0
2	1	0	1	0	0
3	1	1	0	1	1
4	0	0	1	0	1
5	0	0	1	1	0

(10x**1**, 2x**2**) **a**

	1	2	3	4	5
1	2	0	1	1	0
2	0	0	1	0	1
3	1	1	2	0	0
4	1	0	0	0	1
5	0	1	0	1	0

(10x**1**, 2x**2**) **b**

	1	2	3	4	5
1	2	0	1	1	0
2	0	2	1	0	1
3	1	1	0	0	0
4	1	0	0	0	1
5	0	1	0	1	0

(10x**1**, 2x**2**^{1})

	1	2	3	4	5
1	0	1	0	2^1	1
2	1	0	1	0	0
3	0	1	0	1	0
4	2^1	0	1	0	1
5	1	0	0	1	0

(10x**1**, 2x**2**^{2})

	1	2	3	4	5
1	0	1	0	2^2	1
2	1	0	1	0	0
3	0	1	0	1	0
4	2^2	0	1	0	1
5	1	0	0	1	0

(8x**1**, 3x**2**) **a**

	1	2	3	4	5
1	2	0	0	0	0
2	0	2	1	0	1
3	0	1	2	1	0
4	0	0	1	0	1
5	0	1	0	1	0

(8x**1**, 3x**2**) **b**

	1	2	3	4	5
1	2	0	0	0	0
2	0	0	1	0	1
3	0	1	2	1	0
4	0	0	1	0	1
5	0	1	0	1	2

(8x**1**, 1x**2**, 2x**2**^{1}) **a**

	1	2	3	4	5
1	0	0	1	1	0
2	0	0	1	1	2^1
3	1	1	0	0	0
4	1	1	0	0	0
5	0	2^1	0	0	2

(8x**1**, 1x**2**, 2x**2**^{1}) **b**

	1	2	3	4	5
1	0	0	1	1	0
2	0	0	1	1	2^1
3	1	1	0	0	0
4	1	1	0	2	0
5	0	2^1	0	0	0

(8x**1**, 1x**2**, 2x**2**^{1}) **c**

	1	2	3	4	5
1	2	0	1	1	0
2	0	0	1	1	2^1
3	1	1	0	0	0
4	1	1	0	0	0
5	0	2^1	0	0	0

(8x**1**, 1x**2**, 2x**2**^{1}) **d**

	1	2	3	4	5
1	0	0	1	1	0
2	0	0	1	1	0
3	1	1	0	2^1	0
4	1	1	2^1	0	0
5	0	0	0	0	2

(8x**1**, 1x**2**, 2x**2**^{2}) **a**

	1	2	3	4	5
1	0	0	1	1	0
2	0	0	1	1	2^2
3	1	1	0	0	0
4	1	1	0	0	0
5	0	2^2	0	0	2

(8x**1**, 1x**2**, 2x**2**^{2}) **b**

	1	2	3	4	5
1	0	0	1	1	0
2	0	0	1	1	2^2
3	1	1	0	0	0
4	1	1	0	2	0
5	0	2^2	0	0	0

(8x**1**, 1x**2**, 2x**2**^{2}) **c**

	1	2	3	4	5
1	2	0	1	1	0
2	0	0	1	1	2^2
3	1	1	0	0	0
4	1	1	0	0	0
5	0	2^2	0	0	0

(8x**1**, 1x**2**, 2x**2**^{2}) **d**

	1	2	3	4	5
1	0	0	1	1	0
2	0	0	1	1	0
3	1	1	0	2^2	0
4	1	1	2^2	0	0
5	0	0	0	0	2

(6x**1**, 4x**2**)

	1	2	3	4	5
1	2	0	0	0	0
2	0	2	1	1	0
3	0	1	2	1	0
4	0	1	1	0	0
5	0	0	0	0	2

(6x**1**, 2x**2**, 2x**2**^{1}) **a**

	1	2	3	4	5
1	2	0	0	0	0
2	0	0	1	1	2^1
3	0	1	0	1	0
4	0	1	1	0	0
5	0	2^1	0	0	2

(6x**1**, 2x**2**, 2x**2**^{1}) **b**

	1	2	3	4	5
1	2	0	0	0	0
2	0	0	1	1	2^1
3	0	1	0	1	0
4	0	1	1	2	0
5	0	2^1	0	0	0

(6x**1**, 2x**2**, 2x**2**^{1}) **c**

	1	2	3	4	5
1	0	0	1	1	0
2	0	0	0	0	2^1
3	1	0	0	1	0
4	1	0	1	2	0
5	0	2^1	0	0	2

(6x**1**, 2x**2**, 2x$\mathbf{2}^{1}$) **d**

	1	2	3	4	5
1	0	0	1	1	0
2	0	2	0	0	2^1
3	1	0	0	1	0
4	1	0	1	0	0
5	0	2^1	0	0	2

(6x**1**, 2x**2**, 2x$\mathbf{2}^{1}$) **e**

	1	2	3	4	5
1	2	0	1	1	0
2	0	0	0	0	2^1
3	1	0	0	1	0
4	1	0	1	2	0
5	0	2^1	0	0	0

(6x**1**, 2x**2**, 2x$\mathbf{2}^{2}$) **a**

	1	2	3	4	5
1	2	0	0	0	0
2	0	0	1	1	2^2
3	0	1	0	1	0
4	0	1	1	0	0
5	0	2^2	0	0	2

(6x**1**, 2x**2**, 2x$\mathbf{2}^{2}$) **b**

	1	2	3	4	5
1	2	0	0	0	0
2	0	0	1	1	2^2
3	0	1	0	1	0
4	0	1	1	2	0
5	0	2^2	0	0	0

(6x**1**, 2x**2**, 2x$\mathbf{2}^{2}$) **c**

	1	2	3	4	5
1	0	0	1	1	0
2	0	0	0	0	2^2
3	1	0	0	1	0
4	1	0	1	2	0
5	0	2^2	0	0	2

(6x**1**, 2x**2**, 2x$\mathbf{2}^{2}$) **d**

	1	2	3	4	5
1	0	0	1	1	0
2	0	2	0	0	2^2
3	1	0	0	1	0
4	1	0	1	0	0
5	0	2^2	0	0	2

(6x**1**, 2x**2**, 2x$\mathbf{2}^{2}$) **e**

	1	2	3	4	5
1	2	0	1	1	0
2	0	0	0	0	2^2
3	1	0	0	1	0
4	1	0	1	2	0
5	0	2^2	0	0	0

(6x**1**, 4x$\mathbf{2}^{1}$) **a**

	1	2	3	4	5
1	0	0	0	2^1	2^1
2	0	0	1	1	0
3	0	1	0	1	0
4	2^1	1	1	0	0
5	2^1	0	0	0	0

(6x**1**, 4x$\mathbf{2}^{1}$) **b**

	1	2	3	4	5
1	0	0	0	2^1	0
2	0	0	1	1	2^1
3	0	1	0	1	0
4	2^1	1	1	0	0
5	0	2^1	0	0	0

(6x**1**, 2x$\mathbf{2}^{1}$, 2x$\mathbf{2}^{2}$) **a**

	1	2	3	4	5
1	0	0	0	2^2	2^1
2	0	0	1	1	0
3	0	1	0	1	0
4	2^2	1	1	0	0
5	2^1	0	0	0	0

(6x**1**, 2x$\mathbf{2}^{1}$, 2x$\mathbf{2}^{2}$) **b**

	1	2	3	4	5
1	0	0	0	2^1	2^2
2	0	0	1	1	0
3	0	1	0	1	0
4	2^1	1	1	0	0
5	2^2	0	0	0	0

(6x**1**, 2x$\mathbf{2}^{1}$, 2x$\mathbf{2}^{2}$) **c**

	1	2	3	4	5
1	0	0	0	2^1	0
2	0	0	1	1	2^2
3	0	1	0	1	0
4	2^1	1	1	0	0
5	0	2^2	0	0	0

(6x**1**, 4x$\mathbf{2}^{2}$) **a**

	1	2	3	4	5
1	0	0	0	2^2	2^2
2	0	0	1	1	0
3	0	1	0	1	0
4	2^2	1	1	0	0
5	2^2	0	0	0	0

(6x**1**, 4x$\mathbf{2}^{2}$) **b**

	1	2	3	4	5
1	0	0	0	2^2	0
2	0	0	1	1	2^2
3	0	1	0	1	0
4	2^2	1	1	0	0
5	0	2^2	0	0	0

(5x**2**, 2x$\mathbf{2}^{1}$)

	1	2	3	4	5
1	2	0	0	0	0
2	0	2	0	0	2^1
3	0	0	2	0	0
4	0	0	0	2	0
5	0	2^1	0	0	2

(5x**2**, 2x$\mathbf{2}^{2}$)

	1	2	3	4	5
1	2	0	0	0	0
2	0	2	0	0	2^2
3	0	0	2	0	0
4	0	0	0	2	0
5	0	2^2	0	0	2

(3x**2**, 4x$\mathbf{2}^{1}$) **a**

	1	2	3	4	5
1	0	0	2^1	0	0
2	0	0	0	2^1	0
3	2^1	0	2	0	0
4	0	2^1	0	2	0
5	0	0	0	0	2

(3x**2**, 4x$\mathbf{2}^{1}$) **b**

	1	2	3	4	5
1	0	0	2^1	0	0
2	0	2	0	2^1	0
3	2^1	0	0	0	0
4	0	2^1	0	2	0
5	0	0	0	0	2

(3x**2**, 4x$\mathbf{2}^{1}$) **c**

	1	2	3	4	5
1	2	0	0	0	0
2	0	2	0	2^1	0
3	0	0	0	2^1	0
4	0	2^1	2^1	0	0
5	0	0	0	0	2

(3x**2**, 2x$\mathbf{2}^{1}$, 2x$\mathbf{2}^{2}$) **a**

	1	2	3	4	5
1	0	0	2^1	0	0
2	0	0	0	2^2	0
3	2^1	0	2	0	0
4	0	2^2	0	2	0
5	0	0	0	0	2

(3x**2**, 2x$\mathbf{2}^{1}$, 2x$\mathbf{2}^{2}$) **b**

	1	2	3	4	5
1	0	0	2^2	0	0
2	0	2	0	2^1	0
3	2^2	0	0	0	0
4	0	2^1	0	2	0
5	0	0	0	0	2

(3x**2**, 2x$\mathbf{2}^{1}$, 2x$\mathbf{2}^{2}$) **c**

	1	2	3	4	5
1	0	0	2^1	0	0
2	0	2	0	2^2	0
3	2^1	0	0	0	0
4	0	2^2	0	2	0
5	0	0	0	0	2

(3x**2**, 2x$\mathbf{2}^{1}$, 2x$\mathbf{2}^{2}$) **d**

	1	2	3	4	5
1	2	0	0	0	0
2	0	2	0	2^1	0
3	0	0	0	2^2	0
4	0	2^1	2^2	0	0
5	0	0	0	0	2

(3x**2**, 2x$\mathbf{2}^{1}$, 2x$\mathbf{2}^{2}$) **e**

	1	2	3	4	5
1	2	0	0	0	0
2	0	2	0	2^2	0
3	0	0	0	2^1	0
4	0	2^2	2^1	0	0
5	0	0	0	0	2

(3x**2**, 4x$\mathbf{2}^{2}$) **a**

	1	2	3	4	5
1	0	0	2^2	0	0
2	0	0	0	2^2	0
3	2^2	0	2	0	0
4	0	2^2	0	2	0
5	0	0	0	0	2

(3x**2**, 4x$\mathbf{2}^{2}$) **b**

	1	2	3	4	5
1	0	0	2^2	0	0
2	0	2	0	2^2	0
3	2^2	0	0	0	0
4	0	2^2	0	2	0
5	0	0	0	0	2

(3x**2**, 4x$\mathbf{2}^{2}$) **c**

	1	2	3	4	5
1	2	0	0	0	0
2	0	2	0	2^2	0
3	0	0	0	2^2	0
4	0	2^2	2^2	0	0
5	0	0	0	0	2

(1x**2**, 6x$\mathbf{2}^{1}$) **a**

	1	2	3	4	5
1	2	0	0	0	0
2	0	0	0	2^1	0
3	0	0	0	2^1	2^1
4	0	2^1	2^1	0	0
5	0	0	2^1	0	0

(1x**2**, 6x$\mathbf{2}^{1}$) **b**

	1	2	3	4	5
1	0	2^1	0	0	0
2	2^1	2	0	0	0
3	0	0	0	2^1	2^1
4	0	0	2^1	0	0
5	0	0	2^1	0	0

(1x**2**, 6x$\mathbf{2}^{1}$) **c**

	1	2	3	4	5
1	0	2^1	0	0	0
2	2^1	0	0	0	0
3	0	0	0	2^1	2^1
4	0	0	2^1	2	0
5	0	0	2^1	0	0

(1x**2**, 4x$\mathbf{2}^{1}$, 2x$\mathbf{2}^{2}$) **a**

	1	2	3	4	5
1	2	0	0	0	0
2	0	0	0	2^1	0
3	0	0	0	2^2	2^1
4	0	2^1	2^2	0	0
5	0	0	2^1	0	0

(1x**2**, 4x$\mathbf{2}^{1}$, 2x$\mathbf{2}^{2}$) **b**

	1	2	3	4	5
1	2	0	0	0	0
2	0	0	0	2^2	0
3	0	0	0	2^1	2^1
4	0	2^2	2^1	0	0
5	0	0	2^1	0	0

(1x**2**, 4x$\mathbf{2}^{1}$, 2x$\mathbf{2}^{2}$) **c**

	1	2	3	4	5
1	0	2^2	0	0	0
2	2^2	2	0	0	0
3	0	0	0	2^1	2^1
4	0	0	2^1	0	0
5	0	0	2^1	0	0

(1x**2**, 4x$\mathbf{2}^{1}$, 2x$\mathbf{2}^{2}$) **d**

	1	2	3	4	5
1	0	2^1	0	0	0
2	2^1	2	0	0	0
3	0	0	0	2^2	2^1
4	0	0	2^2	0	0
5	0	0	2^1	0	0

(1x**2**, 4x$\mathbf{2}^{1}$, 2x$\mathbf{2}^{2}$) **e**

	1	2	3	4	5
1	0	2^1	0	0	0
2	2^1	0	0	0	0
3	0	0	0	2^2	2^1
4	0	0	2^2	2	0
5	0	0	2^1	0	0

(1x**2**, 4x$\mathbf{2^1}$, 2x$\mathbf{2^2}$) **f**

	1	2	3	4	5
1	0	2^2	0	0	0
2	2^2	0	0	0	0
3	0	0	0	2^1	2^1
4	0	0	2^1	2	0
5	0	0	2^1	0	0

(1x**2**, 4x$\mathbf{2^1}$, 2x$\mathbf{2^2}$) **g**

	1	2	3	4	5
1	0	2^1	0	0	0
2	2^1	0	0	0	0
3	0	0	0	2^1	2^2
4	0	0	2^1	2	0
5	0	0	2^2	0	0

(1x**2**, 2x$\mathbf{2^1}$, 4x$\mathbf{2^2}$) **a**

	1	2	3	4	5
1	2	0	0	0	0
2	0	0	0	2^2	0
3	0	0	0	2^2	2^1
4	0	2^2	2^2	0	0
5	0	0	2^1	0	0

(1x**2**, 2x$\mathbf{2^1}$, 4x$\mathbf{2^2}$) **b**

	1	2	3	4	5
1	2	0	0	0	0
2	0	0	0	2^2	0
3	0	0	0	2^1	2^2
4	0	2^2	2^1	0	0
5	0	0	2^2	0	0

(1x**2**, 2x$\mathbf{2^1}$, 4x$\mathbf{2^2}$) **c**

	1	2	3	4	5
1	0	2^2	0	0	0
2	2^2	2	0	0	0
3	0	0	0	2^2	2^1
4	0	0	2^2	0	0
5	0	0	2^1	0	0

(1x**2**, 2x$\mathbf{2^1}$, 4x$\mathbf{2^2}$) **d**

	1	2	3	4	5
1	0	2^1	0	0	0
2	2^1	2	0	0	0
3	0	0	0	2^2	2^2
4	0	0	2^2	0	0
5	0	0	2^2	0	0

(1x**2**, 2x$\mathbf{2^1}$, 4x$\mathbf{2^2}$) **e**

	1	2	3	4	5
1	0	2^1	0	0	0
2	2^1	0	0	0	0
3	0	0	0	2^2	2^2
4	0	0	2^2	2	0
5	0	0	2^2	0	0

(1x**2**, 2x$\mathbf{2^1}$, 4x$\mathbf{2^2}$) **f**

	1	2	3	4	5
1	0	2^2	0	0	0
2	2^2	0	0	0	0
3	0	0	0	2^1	2^2
4	0	0	2^1	2	0
5	0	0	2^2	0	0

(1x**2**, 2x$\mathbf{2^1}$, 4x$\mathbf{2^2}$) **g**

	1	2	3	4	5
1	0	2^2	0	0	0
2	2^2	0	0	0	0
3	0	0	0	2^2	2^1
4	0	0	2^2	2	0
5	0	0	2^1	0	0

(1x**2**, 6x$\mathbf{2^2}$) **a**

	1	2	3	4	5
1	2	0	0	0	0
2	0	0	0	2^2	0
3	0	0	0	2^2	2^2
4	0	2^2	2^2	0	0
5	0	0	2^2	0	0

(1x**2**, 6x$\mathbf{2^2}$) **b**

	1	2	3	4	5
1	0	2^2	0	0	0
2	2^2	2	0	0	0
3	0	0	0	2^2	2^2
4	0	0	2^2	0	0
5	0	0	2^2	0	0

(1x**2**, 6x$\mathbf{2^2}$) **c**

	1	2	3	4	5
1	0	2^2	0	0	0
2	2^2	0	0	0	0
3	0	0	0	2^2	2^2
4	0	0	2^2	2	0
5	0	0	2^2	0	0

$^{2}V_{3}{}^{4}V_{3}$ NG

$^{2}V_{3}{}^{4}V_{4}$ NG

$^{2}V_{3}{}^{4}V_{5}$ NG

$^{2}V_{4}{}^{4}V_{1}$

(12x**1**)

	1	2	3	4	5
1	0	1	1	0	0
2	1	0	1	0	0
3	1	1	0	1	1
4	0	0	1	0	1
5	0	0	1	1	0

(10x**1**, 1x**2**)

	1	2	3	4	5
1	0	0	1	1	0
2	0	0	1	0	1
3	1	1	2	0	0
4	1	0	0	0	1
5	0	1	0	1	0

(8x**1**, 2x**2**)

	1	2	3	4	5
1	2	0	0	0	0
2	0	2	1	0	1
3	0	1	0	1	0
4	0	0	1	0	1
5	0	1	0	1	0

(8x**1**, 2x$\mathbf{2^1}$)

	1	2	3	4	5
1	0	0	1	1	0
2	0	0	1	1	2^1
3	1	1	0	0	0
4	1	1	0	0	0
5	0	2^1	0	0	0

(8x**1**, 2x$\mathbf{2^2}$)

	1	2	3	4	5
1	0	0	1	1	0
2	0	0	1	1	2^2
3	1	1	0	0	0
4	1	1	0	0	0
5	0	2^2	0	0	0

(6x**1**, 3x**2**)

	1	2	3	4	5
1	2	0	0	0	0
2	0	0	1	1	0
3	0	1	2	1	0
4	0	1	1	0	0
5	0	0	0	0	2

(6x**1**, 1x**2**, 2x$\mathbf{2^1}$) **a**

	1	2	3	4	5
1	0	0	0	0	2^1
2	0	2	1	1	0
3	0	1	0	1	0
4	0	1	1	0	0
5	2^1	0	0	0	0

(6x**1**, 1x**2**, 2x$\mathbf{2^1}$) **b**

	1	2	3	4	5
1	0	0	0	0	2^1
2	0	0	1	1	0
3	0	1	0	1	0
4	0	1	1	0	0
5	2^1	0	0	0	2

(6x**1**, 1x**2**, 2x$\mathbf{2^1}$) **c**

	1	2	3	4	5
1	2	0	0	0	0
2	0	0	1	1	2^1
3	0	1	0	1	0
4	0	1	1	0	0
5	0	2^1	0	0	0

(6x**1**, 1x**2**, 2x$\mathbf{2^2}$) **a**

	1	2	3	4	5
1	0	0	0	0	2^2
2	0	2	1	1	0
3	0	1	0	1	0
4	0	1	1	0	0
5	2^2	0	0	0	0

(6x**1**, 1x**2**, 2x$\mathbf{2^2}$) **b**

	1	2	3	4	5
1	0	0	0	0	2^2
2	0	0	1	1	0
3	0	1	0	1	0
4	0	1	1	0	0
5	2^2	0	0	0	2

(6x**1**, 1x**2**, 2x$\mathbf{2^2}$) **c**

	1	2	3	4	5
1	2	0	0	0	0
2	0	0	1	1	2^2
3	0	1	0	1	0
4	0	1	1	0	0
5	0	2^2	0	0	0

(4x**2**, 2x$\mathbf{2^1}$)

	1	2	3	4	5
1	0	0	0	0	2^1
2	0	2	0	0	0
3	0	0	2	0	0
4	0	0	0	2	0
5	2^1	0	0	0	2

(4x**2**, 2x$\mathbf{2^2}$)

	1	2	3	4	5
1	0	0	0	0	2^2
2	0	2	0	0	0
3	0	0	2	0	0
4	0	0	0	2	0
5	2^2	0	0	0	2

(2x**2**, 4x$\mathbf{2^1}$) **a**

	1	2	3	4	5
1	0	0	0	0	2^1
2	0	0	0	2^1	0
3	0	0	2	0	0
4	0	2^1	0	0	0
5	2^1	0	0	0	2

(2x**2**, 4x$\mathbf{2^1}$) **b**

	1	2	3	4	5
1	0	0	0	2^1	0
2	0	0	0	2^1	0
3	0	0	2	0	0
4	2^1	2^1	0	0	0
5	0	0	0	0	2

(2x**2**, 2x$\mathbf{2^1}$, 2x$\mathbf{2^2}$) **a**

	1	2	3	4	5
1	0	0	0	0	2^2
2	0	0	0	2^1	0
3	0	0	2	0	0
4	0	2^1	0	0	0
5	2^2	0	0	0	2

(2x**2**, 2x$\mathbf{2^1}$, 2x$\mathbf{2^2}$) **b**

	1	2	3	4	5
1	0	0	0	0	2^1
2	0	0	0	2^2	0
3	0	0	2	0	0
4	0	2^2	0	0	0
5	2^1	0	0	0	2

(2x**2**, 2x$\mathbf{2^1}$, 2x$\mathbf{2^2}$) **c**

	1	2	3	4	5
1	0	0	0	2^1	0
2	0	0	0	2^2	0
3	0	0	2	0	0
4	2^1	2^2	0	0	0
5	0	0	0	0	2

(2x**2**, 4x$\mathbf{2^2}$) **a**

	1	2	3	4	5
1	0	0	0	0	2^2
2	0	0	0	2^2	0
3	0	0	2	0	0
4	0	2^2	0	0	0
5	2^2	0	0	0	2

(2x**2**, 4x$\mathbf{2^2}$) **b**

	1	2	3	4	5
1	0	0	0	2^2	0
2	0	0	0	2^2	0
3	0	0	2	0	0
4	2^2	2^2	0	0	0
5	0	0	0	0	2

(6x$\mathbf{2^1}$)

	1	2	3	4	5
1	0	0	0	2^1	0
2	0	0	0	2^1	0
3	0	0	0	0	2^1
4	2^1	2^1	0	0	0
5	0	0	2^1	0	0

(4x$\mathbf{2^1}$, 2x$\mathbf{2^2}$) **a**

	1	2	3	4	5
1	0	0	0	2^1	0
2	0	0	0	2^2	0
3	0	0	0	0	2^1
4	2^1	2^2	0	0	0
5	0	0	2^1	0	0

(4x$\mathbf{2^1}$, 2x$\mathbf{2^2}$) **b**

	1	2	3	4	5
1	0	0	0	2^1	0
2	0	0	0	2^1	0
3	0	0	0	0	2^2
4	2^1	2^1	0	0	0
5	0	0	2^2	0	0

(2x$\mathbf{2^1}$, 4x$\mathbf{2^2}$) **a**

	1	2	3	4	5
1	0	0	0	2^2	0
2	0	0	0	2^2	0
3	0	0	0	0	2^1
4	2^2	2^2	0	0	0
5	0	0	2^1	0	0

(2x$\mathbf{2^1}$, 4x$\mathbf{2^2}$) **b**

	1	2	3	4	5
1	0	0	0	2^1	0
2	0	0	0	2^2	0
3	0	0	0	0	2^2
4	2^1	2^2	0	0	0
5	0	0	2^2	0	0

(6x$\mathbf{2^2}$)

	1	2	3	4	5
1	0	0	0	2^2	0
2	0	0	0	2^2	0
3	0	0	0	0	2^2
4	2^2	2^2	0	0	0
5	0	0	2^2	0	0

$^2V_4{}^4V_2$

(16x**1**) **a**

	1	2	3	4	5	6
1	0	0	1	1	0	0
2	0	0	1	1	0	0
3	1	1	0	0	1	1
4	1	1	0	0	1	1
5	0	0	1	1	0	0
6	0	0	1	1	0	0

(16x**1**) **b**

	1	2	3	4	5	6
1	0	0	0	1	0	1
2	0	0	1	1	0	0
3	0	1	0	1	1	1
4	1	1	1	0	1	0
5	0	0	1	1	0	0
6	1	0	1	0	0	0

(14x**1**, 1x**2**) **a**

	1	2	3	4	5	6
1	0	0	1	1	0	0
2	0	0	1	1	0	0
3	1	1	0	1	1	0
4	1	1	1	0	1	0
5	0	0	1	1	0	0
6	0	0	0	0	0	2

(14x**1**, 1x**2**) **b**

	1	2	3	4	5	6
1	2	0	1	1	0	0
2	0	0	1	1	1	1
3	1	1	0	0	0	0
4	1	1	0	0	0	0
5	0	1	0	0	0	1
6	0	1	0	0	1	0

(14x**1**, 1x**2**) **c**

	1	2	3	4	5	6
1	0	0	1	1	0	0
2	0	0	1	1	1	1
3	1	1	0	0	0	0
4	1	1	0	2	0	0
5	0	1	0	0	0	1
6	0	1	0	0	1	0

(14x**1**, 1x**2**) **d**

	1	2	3	4	5	6
1	0	0	1	1	0	0
2	0	0	1	1	1	1
3	1	1	0	0	0	0
4	1	1	0	0	0	0
5	0	1	0	0	0	1
6	0	1	0	0	1	2

(12x**1**, 2x**2**) **a**

	1	2	3	4	5	6
1	2	0	1	1	0	0
2	0	0	1	0	0	1
3	1	1	0	0	0	0
4	1	0	0	0	1	0
5	0	0	0	1	0	1
6	0	1	0	0	1	2

(12x**1**, 2x**2**) **b**

	1	2	3	4	5	6
1	2	0	1	1	0	0
2	0	2	1	0	0	1
3	1	1	0	0	0	0
4	1	0	0	0	1	0
5	0	0	0	1	0	1
6	0	1	0	0	1	0

(12x**1**, 2x**2**) **c**

	1	2	3	4	5	6
1	0	0	1	1	0	0
2	0	2	1	0	0	1
3	1	1	0	0	0	0
4	1	0	0	0	1	0
5	0	0	0	1	0	1
6	0	1	0	0	1	2

(12x**1**, 2x**2**) **d**

	1	2	3	4	5	6
1	2	0	0	0	0	0
2	0	0	1	1	1	1
3	0	1	0	1	0	0
4	0	1	1	0	0	0
5	0	1	0	0	0	1
6	0	1	0	0	1	2

(12x**1**, 2x**2**) **e**

	1	2	3	4	5	6
1	2	0	1	1	0	0
2	0	2	0	0	1	1
3	1	0	0	1	0	0
4	1	0	1	0	0	0
5	0	1	0	0	0	1
6	0	1	0	0	1	0

(12x**1**, 2x**2**) **f**

	1	2	3	4	5	6
1	0	0	1	1	0	0
2	0	2	0	0	1	1
3	1	0	0	1	0	0
4	1	0	1	0	0	0
5	0	1	0	0	2	1
6	0	1	0	0	1	0

(12x**1**, 2x$\mathbf{2^1}$) **a**

	1	2	3	4	5	6
1	0	0	0	0	0	2^1
2	0	0	1	1	1	1
3	0	1	0	1	0	0
4	0	1	1	0	0	0
5	0	1	0	0	0	1
6	2^1	1	0	0	1	0

(12x**1**, 2x$\mathbf{2^1}$) **b**

	1	2	3	4	5	6
1	0	1	0	0	1	2^1
2	1	0	0	0	1	0
3	0	0	0	1	0	1
4	0	0	1	0	0	1
5	1	1	0	0	0	0
6	2^1	0	1	1	0	0

(12x**1**, 2x$\mathbf{2^1}$) **c**

	1	2	3	4	5	6
1	0	0	0	1	0	1
2	0	0	1	0	1	0
3	0	1	0	2^1	0	1
4	1	0	2^1	0	1	0
5	0	1	0	1	0	0
6	1	0	1	0	0	0

(12x**1**, 2x$\mathbf{2^1}$) **d**

	1	2	3	4	5	6
1	0	0	1	1	0	0
2	0	0	1	0	0	1
3	1	1	0	2^1	0	0
4	1	0	2^1	0	1	0
5	0	0	0	1	0	1
6	0	1	0	0	1	0

(12x**1**, 2x$\mathbf{2^2}$) **a**

	1	2	3	4	5	6
1	0	0	0	0	0	2^2
2	0	0	1	1	1	1
3	0	1	0	1	0	0
4	0	1	1	0	0	0
5	0	1	0	0	0	1
6	2^2	1	0	0	1	0

(12x**1**, 2x$\mathbf{2^2}$) **b**

	1	2	3	4	5	6
1	0	1	0	0	1	2^2
2	1	0	0	0	1	0
3	0	0	0	1	0	1
4	0	0	1	0	0	1
5	1	1	0	0	0	0
6	2^2	0	1	1	0	0

(12x**1**, 2x$\mathbf{2^2}$) **c**

	1	2	3	4	5	6
1	0	0	0	1	0	1
2	0	0	1	0	1	0
3	0	1	0	2^2	0	1
4	1	0	2^2	0	1	0
5	0	1	0	1	0	0
6	1	0	1	0	0	0

(12x**1**, 2x$\mathbf{2^2}$) **d**

	1	2	3	4	5	6
1	0	0	1	1	0	0
2	0	0	1	0	0	1
3	1	1	0	2^2	0	0
4	1	0	2^2	0	1	0
5	0	0	0	1	0	1
6	0	1	0	0	1	0

(10x**1**, 3x**2**) **a**

	1	2	3	4	5	6
1	2	0	0	0	0	0
2	0	2	1	0	0	1
3	0	1	0	1	0	0
4	0	0	1	0	1	0
5	0	0	0	1	0	1
6	0	1	0	0	1	2

(10x**1**, 3x**2**) **b**

	1	2	3	4	5	6
1	2	0	0	0	0	0
2	0	0	1	0	0	1
3	0	1	2	1	0	0
4	0	0	1	0	1	0
5	0	0	0	1	0	1
6	0	1	0	0	1	2

(10x**1**, 1x**2**, 2x**2**^{1}) **a**

	1	2	3	4	5	6
1	2	0	0	0	0	0
2	0	0	1	0	2^1	1
3	0	1	0	1	0	0
4	0	0	1	0	1	0
5	0	2^1	0	1	0	1
6	0	1	0	0	1	0

(10x**1**, 1x**2**, 2x**2**^{1}) **b**

	1	2	3	4	5	6
1	0	0	0	0	0	2^1
2	0	0	1	0	0	1
3	0	1	2	1	0	0
4	0	0	1	0	1	0
5	0	0	0	1	0	1
6	2^1	1	0	0	1	0

(10x**1**, 1x**2**, 2x**2**^{1}) **c**

	1	2	3	4	5	6
1	0	0	0	0	0	2^1
2	0	0	1	0	0	1
3	0	1	0	1	0	0
4	0	0	1	0	1	0
5	0	0	0	1	2	1
6	2^1	1	0	0	1	0

(10x**1**, 1x**2**, 2x**2**^{1}) **d**

	1	2	3	4	5	6
1	2	0	0	0	0	2^1
2	0	0	1	0	0	1
3	0	1	0	1	0	0
4	0	0	1	0	1	0
5	0	0	0	1	0	1
6	2^1	1	0	0	1	0

(10x**1**, 1x**2**, 2x**2**^{2}) **a**

	1	2	3	4	5	6
1	2	0	0	0	0	0
2	0	0	1	0	2^2	1
3	0	1	0	1	0	0
4	0	0	1	0	1	0
5	0	2^2	0	1	0	1
6	0	1	0	0	1	0

(10x**1**, 1x**2**, 2x**2**^{2}) **b**

	1	2	3	4	5	6
1	0	0	0	0	0	2^2
2	0	0	1	0	0	1
3	0	1	2	1	0	0
4	0	0	1	0	1	0
5	0	0	0	1	0	1
6	2^2	1	0	0	1	0

(10x**1**, 1x**2**, 2x**2**^{2}) **c**

	1	2	3	4	5	6
1	0	0	0	0	0	2^2
2	0	0	1	0	0	1
3	0	1	0	1	0	0
4	0	0	1	0	1	0
5	0	0	0	1	2	1
6	2^2	1	0	0	1	0

(10x**1**, 1x**2**, 2x**2**^{2}) **d**

	1	2	3	4	5	6
1	2	0	0	0	0	2^2
2	0	0	1	0	0	1
3	0	1	0	1	0	0
4	0	0	1	0	1	0
5	0	0	0	1	0	1
6	2^2	1	0	0	1	0

(8x**1**, 4x**2**) **a**

	1	2	3	4	5	6
1	2	0	0	0	0	0
2	0	2	1	0	1	0
3	0	1	0	1	0	0
4	0	0	1	2	1	0
5	0	1	0	1	0	0
6	0	0	0	0	0	2

(8x**1**, 4x**2**) **b**

	1	2	3	4	5	6
1	2	0	0	0	0	0
2	0	0	1	0	1	0
3	0	1	2	1	0	0
4	0	0	1	2	1	0
5	0	1	0	1	0	0
6	0	0	0	0	0	2

(8x**1**, 2x**2**, 2x**2**^{1}) **a**

	1	2	3	4	5	6
1	0	0	1	1	0	0
2	0	0	1	1	0	0
3	1	1	0	2^1	0	0
4	1	1	2^1	0	0	0
5	0	0	0	0	2	0
6	0	0	0	0	0	2

(8x**1**, 2x**2**, 2x**2**^{1}) **b**

	1	2	3	4	5	6
1	0	0	1	1	0	0
2	0	0	1	1	0	2^1
3	1	1	2	0	0	0
4	1	1	0	0	0	0
5	0	0	0	0	2	0
6	0	2^1	0	0	0	0

(8x**1**, 2x**2**, 2x**2**^{1}) **c**

	1	2	3	4	5	6
1	2	0	1	1	0	0
2	0	0	1	1	0	2^1
3	1	1	0	0	0	0
4	1	1	0	0	0	0
5	0	0	0	0	2	0
6	0	2^1	0	0	0	0

(8x**1**, 2x**2**, 2x**2**^{1}) **d**

	1	2	3	4	5	6
1	0	0	1	1	0	0
2	0	0	1	1	0	2^1
3	1	1	0	0	0	0
4	1	1	0	0	0	0
5	0	0	0	0	2	0
6	0	2^1	0	0	0	2

(8x**1**, 2x**2**, 2x**2**^{1}) **e**

	1	2	3	4	5	6
1	0	1	0	1	0	0
2	1	2	1	0	0	0
3	0	1	0	1	0	0
4	1	0	1	2	0	0
5	0	0	0	0	0	2^1
6	0	0	0	0	2^1	0

(8x**1**, 2x**2**, 2x**2**^{1}) **f**

	1	2	3	4	5	6
1	0	1	0	1	0	0
2	1	2	1	0	0	0
3	0	1	2	1	0	0
4	1	0	1	0	0	0
5	0	0	0	0	0	2^1
6	0	0	0	0	2^1	0

(8x**1**, 2x**2**, 2x**2**^{1}) **g**

	1	2	3	4	5	6
1	0	1	0	1	0	0
2	1	0	1	0	0	0
3	0	1	2	1	0	0
4	1	0	1	0	0	0
5	0	0	0	0	0	2^1
6	0	0	0	0	2^1	2

(8x**1**, 2x**2**, 2x**2**^{1}) **h**

	1	2	3	4	5	6
1	0	1	0	1	0	0
2	1	0	1	0	0	0
3	0	1	0	1	0	0
4	1	0	1	0	0	0
5	0	0	0	0	2	2^1
6	0	0	0	0	2^1	2

(8x**1**, 2x**2**, 2x**2**^{2}) **a**

	1	2	3	4	5	6
1	0	0	1	1	0	0
2	0	0	1	1	0	0
3	1	1	0	2^2	0	0
4	1	1	2^2	0	0	0
5	0	0	0	0	2	0
6	0	0	0	0	0	2

(8x**1**, 2x**2**, 2x**2**^{2}) **b**

	1	2	3	4	5	6
1	0	0	1	1	0	0
2	0	0	1	1	0	2^2
3	1	1	2	0	0	0
4	1	1	0	0	0	0
5	0	0	0	0	2	0
6	0	2^2	0	0	0	0

(8x**1**, 2x**2**, 2x**2**^{2}) **c**

	1	2	3	4	5	6
1	2	0	1	1	0	0
2	0	0	1	1	0	2^2
3	1	1	0	0	0	0
4	1	1	0	0	0	0
5	0	0	0	0	2	0
6	0	2^2	0	0	0	0

(8x**1**, 2x**2**, 2x**2**^{2}) **d**

	1	2	3	4	5	6
1	0	0	1	1	0	0
2	0	0	1	1	0	2^2
3	1	1	0	0	0	0
4	1	1	0	0	0	0
5	0	0	0	0	2	0
6	0	2^2	0	0	0	2

(8x**1**, 2x**2**, 2x**2**^{2}) **e**

	1	2	3	4	5	6
1	0	1	0	1	0	0
2	1	2	1	0	0	0
3	0	1	0	1	0	0
4	1	0	1	2	0	0
5	0	0	0	0	0	2^2
6	0	0	0	0	2^2	0

(8x**1**, 2x**2**, 2x**2**^{2}) **f**

	1	2	3	4	5	6
1	0	1	0	1	0	0
2	1	2	1	0	0	0
3	0	1	2	1	0	0
4	1	0	1	0	0	0
5	0	0	0	0	0	2^2
6	0	0	0	0	2^2	0

(8x**1**, 2x**2**, 2x**2**^{2}) **g**

	1	2	3	4	5	6
1	0	1	0	1	0	0
2	1	0	1	0	0	0
3	0	1	2	1	0	0
4	1	0	1	0	0	0
5	0	0	0	0	0	2^2
6	0	0	0	0	2^2	2

(8x**1**, 2x**2**, 2x**2**^{2}) **h**

	1	2	3	4	5	6
1	0	1	0	1	0	0
2	1	0	1	0	0	0
3	0	1	0	1	0	0
4	1	0	1	0	0	0
5	0	0	0	0	2	2^2
6	0	0	0	0	2^2	2

(8x**1**, 4x**2**^{1}) **a**

	1	2	3	4	5	6
1	0	0	1	1	2^1	0
2	0	0	1	1	0	2^1
3	1	1	0	0	0	0
4	1	1	0	0	0	0
5	2^1	0	0	0	0	0
6	0	2^1	0	0	0	0

(8x**1**, 4x**2**^{1}) **b**

	1	2	3	4	5	6
1	0	0	1	1	2^1	0
2	0	0	1	1	0	0
3	1	1	0	0	0	2^1
4	1	1	0	0	0	0
5	2^1	0	0	0	0	0
6	0	0	2^1	0	0	0

(8x**1**, 4x**2**^{1}) **c**

	1	2	3	4	5	6
1	0	0	1	1	0	0
2	0	0	1	1	0	0
3	1	1	0	2^{1}	0	0
4	1	1	2^{1}	0	0	0
5	0	0	0	0	0	2^{1}
6	0	0	0	0	2^{1}	0

(8x**1**, 4x**2**^{1}) **d**

	1	2	3	4	5	6
1	0	0	1	1	0	0
2	0	0	1	1	0	2^{1}
3	1	1	0	0	0	0
4	1	1	0	0	0	0
5	0	0	0	0	0	2^{1}
6	0	2^{1}	0	0	2^{1}	0

(8x**1**, 2x**2**^{1}, 2x**2**^{2}) **a**

	1	2	3	4	5	6
1	0	0	1	1	2^{1}	0
2	0	0	1	1	0	2^{2}
3	1	1	0	0	0	0
4	1	1	0	0	0	0
5	2^{1}	0	0	0	0	0
6	0	2^{2}	0	0	0	0

(8x**1**, 2x**2**^{1}, 2x**2**^{2}) **b**

	1	2	3	4	5	6
1	0	0	1	1	2^{1}	0
2	0	0	1	1	0	0
3	1	1	0	0	0	2^{2}
4	1	1	0	0	0	0
5	2^{1}	0	0	0	0	0
6	0	0	2^{2}	0	0	0

(8x**1**, 2x**2**^{1}, 2x**2**^{2}) **c**

	1	2	3	4	5	6
1	0	0	1	1	0	0
2	0	0	1	1	0	0
3	1	1	0	2^{2}	0	0
4	1	1	2^{2}	0	0	0
5	0	0	0	0	0	2^{1}
6	0	0	0	0	2^{1}	0

(8x**1**, 2x**2**^{1}, 2x**2**^{2}) **d**

	1	2	3	4	5	6
1	0	0	1	1	0	0
2	0	0	1	1	0	0
3	1	1	0	2^{1}	0	0
4	1	1	2^{1}	0	0	0
5	0	0	0	0	0	2^{2}
6	0	0	0	0	2^{2}	0

(8x**1**, 2x**2**^{1}, 2x**2**^{2}) **e**

	1	2	3	4	5	6
1	0	0	1	1	0	0
2	0	0	1	1	0	2^{2}
3	1	1	0	0	0	0
4	1	1	0	0	0	0
5	0	0	0	0	0	2^{1}
6	0	2^{2}	0	0	2^{1}	0

(8x**1**, 2x**2**^{1}, 2x**2**^{2}) **f**

	1	2	3	4	5	6
1	0	0	1	1	0	0
2	0	0	1	1	0	2^{1}
3	1	1	0	0	0	0
4	1	1	0	0	0	0
5	0	0	0	0	0	2^{2}
6	0	2^{1}	0	0	2^{2}	0

(8x**1**, 4x**2**^{2}) **a**

	1	2	3	4	5	6
1	0	0	1	1	2^{2}	0
2	0	0	1	1	0	2^{2}
3	1	1	0	0	0	0
4	1	1	0	0	0	0
5	2^{2}	0	0	0	0	0
6	0	2^{2}	0	0	0	0

(8x**1**, 4x**2**^{2}) **b**

	1	2	3	4	5	6
1	0	0	1	1	2^{2}	0
2	0	0	1	1	0	0
3	1	1	0	0	0	2^{2}
4	1	1	0	0	0	0
5	2^{2}	0	0	0	0	0
6	0	0	2^{2}	0	0	0

(8x**1**, 4x**2**^{2}) **c**

	1	2	3	4	5	6
1	0	0	1	1	0	0
2	0	0	1	1	0	0
3	1	1	0	2^{2}	0	0
4	1	1	2^{2}	0	0	0
5	0	0	0	0	0	2^{2}
6	0	0	0	0	2^{2}	0

(8x**1**, 4x**2**^{2}) **d**

	1	2	3	4	5	6
1	0	0	1	1	0	0
2	0	0	1	1	0	2^{2}
3	1	1	0	0	0	0
4	1	1	0	0	0	0
5	0	0	0	0	0	2^{2}
6	0	2^{2}	0	0	2^{2}	0

(6x**1**, 5x**2**)

	1	2	3	4	5	6
1	2	0	0	0	0	0
2	0	2	0	0	0	0
3	0	0	2	1	1	0
4	0	0	1	2	1	0
5	0	0	1	1	0	0
6	0	0	0	0	0	2

(6x**1**, 3x**2**, 2x**2**^{1}) **a**

	1	2	3	4	5	6
1	2	0	0	2^{1}	0	0
2	0	0	1	1	0	0
3	0	1	0	1	0	0
4	2^{1}	1	1	0	0	0
5	0	0	0	0	2	0
6	0	0	0	0	0	2

(6x**1**, 3x**2**, 2x**2**^{1}) **b**

	1	2	3	4	5	6
1	0	0	0	2^{1}	0	0
2	0	2	1	1	0	0
3	0	1	0	1	0	0
4	2^{1}	1	1	0	0	0
5	0	0	0	0	2	0
6	0	0	0	0	0	2

(6x**1**, 3x**2**, 2x**2**^{1}) **c**

	1	2	3	4	5	6
1	0	0	0	2^{1}	0	0
2	0	2	1	0	0	1
3	0	1	0	0	0	1
4	2^{1}	0	0	0	0	0
5	0	0	0	0	2	0
6	0	1	1	0	0	2

(6x**1**, 3x**2**, 2x**2**^{1}) **d**

	1	2	3	4	5	6
1	0	0	0	2^{1}	0	0
2	0	2	1	0	0	1
3	0	1	0	0	0	1
4	2^{1}	0	0	2	0	0
5	0	0	0	0	2	0
6	0	1	1	0	0	0

(6x**1**, 3x**2**, 2x**2**^{1}) **e**

	1	2	3	4	5	6
1	2	0	0	2^{1}	0	0
2	0	0	1	0	0	1
3	0	1	0	0	0	1
4	2^{1}	0	0	2	0	0
5	0	0	0	0	2	0
6	0	1	1	0	0	0

(6x**1**, 3x**2**, 2x**2**^{2}) **a**

	1	2	3	4	5	6
1	2	0	0	2^{2}	0	0
2	0	0	1	1	0	0
3	0	1	0	1	0	0
4	2^{2}	1	1	0	0	0
5	0	0	0	0	2	0
6	0	0	0	0	0	2

(6x**1**, 3x**2**, 2x**2**^{2}) **b**

	1	2	3	4	5	6
1	0	0	0	2^{2}	0	0
2	0	2	1	1	0	0
3	0	1	0	1	0	0
4	2^{2}	1	1	0	0	0
5	0	0	0	0	2	0
6	0	0	0	0	0	2

(6x**1**, 3x**2**, 2x**2**^{2}) **c**

	1	2	3	4	5	6
1	0	0	0	2^{2}	0	0
2	0	2	1	0	0	1
3	0	1	0	0	0	1
4	2^{2}	0	0	0	0	0
5	0	0	0	0	2	0
6	0	1	1	0	0	2

(6x**1**, 3x**2**, 2x**2**^{2}) **d**

	1	2	3	4	5	6
1	0	0	0	2^{2}	0	0
2	0	2	1	0	0	1
3	0	1	0	0	0	1
4	2^{2}	0	0	2	0	0
5	0	0	0	0	2	0
6	0	1	1	0	0	0

(6x**1**, 3x**2**, 2x**2**^{2}) **e**

	1	2	3	4	5	6
1	2	0	0	2^{2}	0	0
2	0	0	1	0	0	1
3	0	1	0	0	0	1
4	2^{2}	0	0	2	0	0
5	0	0	0	0	2	0
6	0	1	1	0	0	0

(6x**1**, 1x**2**, 4x**2**^{1}) **a**

	1	2	3	4	5	6
1	0	1	1	0	0	0
2	1	0	1	0	0	2^{1}
3	1	1	0	0	0	0
4	0	0	0	2	0	0
5	0	0	0	0	0	2^{1}
6	0	2^{1}	0	0	2^{1}	0

(6x**1**, 1x**2**, 4x**2**^{1}) **b**

	1	2	3	4	5	6
1	0	1	1	0	0	0
2	1	0	1	0	0	2^{1}
3	1	1	0	0	0	0
4	0	0	0	0	2^{1}	0
5	0	0	0	2^{1}	0	0
6	0	2^{1}	0	0	0	2

(6x**1**, 1x**2**, 4x**2**^{1}) **c**

	1	2	3	4	5	6
1	0	1	1	0	0	0
2	1	0	1	0	0	2^{1}
3	1	1	2	0	0	0
4	0	0	0	0	2^{1}	0
5	0	0	0	2^{1}	0	0
6	0	2^{1}	0	0	0	0

(6x**1**, 1x**2**, 4x**2**^{1}) **d**

	1	2	3	4	5	6
1	0	1	1	0	0	0
2	1	0	1	0	0	2^{1}
3	1	1	0	0	0	0
4	0	0	0	0	2^{1}	0
5	0	0	0	2^{1}	2	0
6	0	2^{1}	0	0	0	0

(6x**1**, 1x**2**, 4x**2**^{1}) **e**

	1	2	3	4	5	6
1	0	1	1	2^{1}	0	0
2	1	0	1	0	0	2^{1}
3	1	1	0	0	0	0
4	2^{1}	0	0	0	0	0
5	0	0	0	0	2	0
6	0	2^{1}	0	0	0	0

(6x**1**, 1x**2**, 4x**2**^{1}) **f**

	1	2	3	4	5	6
1	0	0	1	1	0	0
2	0	0	0	0	0	2^{1}
3	1	0	0	1	0	0
4	1	0	1	0	0	0
5	0	0	0	0	2	2^{1}
6	0	2^{1}	0	0	2^{1}	0

(6x**1**, 1x**2**, 4x**2**^{1}) **g**

	1	2	3	4	5	6
1	0	0	1	1	0	0
2	0	0	0	0	0	2^{1}
3	1	0	2	1	0	0
4	1	0	1	0	0	0
5	0	0	0	0	0	2^{1}
6	0	2^{1}	0	0	2^{1}	0

(6x**1**, 1x**2**, 2x$\mathbf{2^1}$, 2x$\mathbf{2^2}$) **a**

	1	2	3	4	5	6
1	0	1	1	0	0	0
2	1	0	1	0	0	2^2
3	1	1	0	0	0	0
4	0	0	0	2	0	0
5	0	0	0	0	0	2^1
6	0	2^2	0	0	2^1	0

(6x**1**, 1x**2**, 2x$\mathbf{2^1}$, 2x$\mathbf{2^2}$) **b**

	1	2	3	4	5	6
1	0	1	1	0	0	0
2	1	0	1	0	0	2^1
3	1	1	0	0	0	0
4	0	0	0	2	0	0
5	0	0	0	0	0	2^2
6	0	2^1	0	0	2^2	0

(6x**1**, 1x**2**, 2x$\mathbf{2^1}$, 2x$\mathbf{2^2}$) **c**

	1	2	3	4	5	6
1	0	1	1	0	0	0
2	1	0	1	0	0	2^2
3	1	1	0	0	0	0
4	0	0	0	0	2^1	0
5	0	0	0	2^1	0	0
6	0	2^2	0	0	0	2

(6x**1**, 1x**2**, 2x$\mathbf{2^1}$, 2x$\mathbf{2^2}$) **d**

	1	2	3	4	5	6
1	0	1	1	0	0	0
2	1	0	1	0	0	2^1
3	1	1	0	0	0	0
4	0	0	0	0	2^2	0
5	0	0	0	2^2	0	0
6	0	2^1	0	0	0	2

(6x**1**, 1x**2**, 2x$\mathbf{2^1}$, 2x$\mathbf{2^2}$) **e**

	1	2	3	4	5	6
1	0	1	1	0	0	0
2	1	0	1	0	0	2^2
3	1	1	2	0	0	0
4	0	0	0	0	2^1	0
5	0	0	0	2^1	0	0
6	0	2^2	0	0	0	0

(6x**1**, 1x**2**, 2x$\mathbf{2^1}$, 2x$\mathbf{2^2}$) **f**

	1	2	3	4	5	6
1	0	1	1	0	0	0
2	1	0	1	0	0	2^1
3	1	1	2	0	0	0
4	0	0	0	0	2^2	0
5	0	0	0	2^2	0	0
6	0	2^1	0	0	0	0

(6x**1**, 1x**2**, 2x$\mathbf{2^1}$, 2x$\mathbf{2^2}$) **g**

	1	2	3	4	5	6
1	0	1	1	0	0	0
2	1	0	1	0	0	2^2
3	1	1	0	0	0	0
4	0	0	0	0	2^1	0
5	0	0	0	2^1	2	0
6	0	2^2	0	0	0	0

(6x**1**, 1x**2**, 2x$\mathbf{2^1}$, 2x$\mathbf{2^2}$) **h**

	1	2	3	4	5	6
1	0	1	1	0	0	0
2	1	0	1	0	0	2^1
3	1	1	0	0	0	0
4	0	0	0	0	2^2	0
5	0	0	0	2^2	2	0
6	0	2^1	0	0	0	0

(6x**1**, 1x**2**, 2x$\mathbf{2^1}$, 2x$\mathbf{2^2}$) **i**

	1	2	3	4	5	6
1	0	1	1	2^1	0	0
2	1	0	1	0	0	2^2
3	1	1	0	0	0	0
4	2^1	0	0	0	0	0
5	0	0	0	0	2	0
6	0	2^2	0	0	0	0

(6x**1**, 1x**2**, 2x$\mathbf{2^1}$, 2x$\mathbf{2^2}$) **j**

	1	2	3	4	5	6
1	0	0	1	1	0	0
2	0	0	0	0	0	2^2
3	1	0	0	1	0	0
4	1	0	1	0	0	0
5	0	0	0	0	2	2^1
6	0	2^2	0	0	2^1	0

(6x**1**, 1x**2**, 2x$\mathbf{2^1}$, 2x$\mathbf{2^2}$) **k**

	1	2	3	4	5	6
1	0	0	1	1	0	0
2	0	0	0	0	0	2^1
3	1	0	0	1	0	0
4	1	0	1	0	0	0
5	0	0	0	0	2	2^2
6	0	2^1	0	0	2^2	0

(6x**1**, 1x**2**, 2x$\mathbf{2^1}$, 2x$\mathbf{2^2}$) **l**

	1	2	3	4	5	6
1	0	0	1	1	0	0
2	0	0	0	0	0	2^2
3	1	0	2	1	0	0
4	1	0	1	0	0	0
5	0	0	0	0	0	2^1
6	0	2^2	0	0	2^1	0

(6x**1**, 1x**2**, 2x$\mathbf{2^1}$, 2x$\mathbf{2^2}$) **m**

	1	2	3	4	5	6
1	0	0	1	1	0	0
2	0	0	0	0	0	2^1
3	1	0	2	1	0	0
4	1	0	1	0	0	0
5	0	0	0	0	0	2^2
6	0	2^1	0	0	2^2	0

(6x**1**, 1x**2**, 4x$\mathbf{2^2}$) **a**

	1	2	3	4	5	6
1	0	1	1	0	0	0
2	1	0	1	0	0	2^2
3	1	1	0	0	0	0
4	0	0	0	2	0	0
5	0	0	0	0	0	2^2
6	0	2^2	0	0	2^2	0

(6x**1**, 1x**2**, 4x$\mathbf{2^2}$) **b**

	1	2	3	4	5	6
1	0	1	1	0	0	0
2	1	0	1	0	0	2^2
3	1	1	0	0	0	0
4	0	0	0	0	2^2	0
5	0	0	0	2^2	0	0
6	0	2^2	0	0	0	2

(6x**1**, 1x**2**, 4x$\mathbf{2^2}$) **c**

	1	2	3	4	5	6
1	0	1	1	0	0	0
2	1	0	1	0	0	2^2
3	1	1	2	0	0	0
4	0	0	0	0	2^2	0
5	0	0	0	2^2	0	0
6	0	2^2	0	0	0	0

(6x**1**, 1x**2**, 4x$\mathbf{2^2}$) **d**

	1	2	3	4	5	6
1	0	1	1	0	0	0
2	1	0	1	0	0	2^2
3	1	1	0	0	0	0
4	0	0	0	0	2^2	0
5	0	0	0	2^2	2	0
6	0	2^2	0	0	0	0

(6x**1**, 1x**2**, 4x$\mathbf{2^2}$) **e**

	1	2	3	4	5	6
1	0	1	1	2^2	0	0
2	1	0	1	0	0	2^2
3	1	1	0	0	0	0
4	2^2	0	0	0	0	0
5	0	0	0	0	2	0
6	0	2^2	0	0	0	0

(6x**1**, 1x**2**, 4x$\mathbf{2^2}$) **f**

	1	2	3	4	5	6
1	0	0	1	1	0	0
2	0	0	0	0	0	2^2
3	1	0	0	1	0	0
4	1	0	1	0	0	0
5	0	0	0	0	2	2^2
6	0	2^2	0	0	2^2	0

(6x**1**, 1x**2**, 4x$\mathbf{2^2}$) **g**

	1	2	3	4	5	6
1	0	0	1	1	0	0
2	0	0	0	0	0	2^2
3	1	0	2	1	0	0
4	1	0	1	0	0	0
5	0	0	0	0	0	2^2
6	0	2^2	0	0	2^2	0

(6x**2**, 2x$\mathbf{2^1}$)

	1	2	3	4	5	6
1	2	2^1	0	0	0	0
2	2^1	2	0	0	0	0
3	0	0	2	0	0	0
4	0	0	0	2	0	0
5	0	0	0	0	2	0
6	0	0	0	0	0	2

(6x**2**, 2x$\mathbf{2^2}$)

	1	2	3	4	5	6
1	2	2^2	0	0	0	0
2	2^2	2	0	0	0	0
3	0	0	2	0	0	0
4	0	0	0	2	0	0
5	0	0	0	0	2	0
6	0	0	0	0	0	2

(4x**2**, 4x$\mathbf{2^1}$) **a**

	1	2	3	4	5	6
1	2	2^1	0	0	0	0
2	2^1	2	0	0	0	0
3	0	0	0	2^1	0	0
4	0	0	2^1	0	0	0
5	0	0	0	0	2	0
6	0	0	0	0	0	2

(4x**2**, 4x$\mathbf{2^1}$) **b**

	1	2	3	4	5	6
1	2	2^1	0	0	0	0
2	2^1	0	0	0	0	0
3	0	0	2	2^1	0	0
4	0	0	2^1	0	0	0
5	0	0	0	0	2	0
6	0	0	0	0	0	2

(4x**2**, 4x$\mathbf{2^1}$) **c**

	1	2	3	4	5	6
1	2	2^1	0	0	0	0
2	2^1	0	2^1	0	0	0
3	0	2^1	0	0	0	0
4	0	0	0	2	0	0
5	0	0	0	0	2	0
6	0	0	0	0	0	2

(4x**2**, 2x$\mathbf{2^1}$, 2x$\mathbf{2^2}$) **a**

	1	2	3	4	5	6
1	2	2^2	0	0	0	0
2	2^2	2	0	0	0	0
3	0	0	0	2^1	0	0
4	0	0	2^1	0	0	0
5	0	0	0	0	2	0
6	0	0	0	0	0	2

(4x**2**, 2x$\mathbf{2^1}$, 2x$\mathbf{2^2}$) **b**

	1	2	3	4	5	6
1	2	2^1	0	0	0	0
2	2^1	2	0	0	0	0
3	0	0	0	2^2	0	0
4	0	0	2^2	0	0	0
5	0	0	0	0	2	0
6	0	0	0	0	0	2

(4x**2**, 2x$\mathbf{2^1}$, 2x$\mathbf{2^2}$) **c**

	1	2	3	4	5	6
1	2	2^2	0	0	0	0
2	2^2	0	0	0	0	0
3	0	0	2	2^1	0	0
4	0	0	2^1	0	0	0
5	0	0	0	0	2	0
6	0	0	0	0	0	2

(4x**2**, 2x$\mathbf{2^1}$, 2x$\mathbf{2^2}$) **d**

	1	2	3	4	5	6
1	2	2^2	0	0	0	0
2	2^2	0	2^1	0	0	0
3	0	2^1	0	0	0	0
4	0	0	0	2	0	0
5	0	0	0	0	2	0
6	0	0	0	0	0	2

(4x**2**, 2x$\mathbf{2^1}$, 2x$\mathbf{2^2}$) **e**

	1	2	3	4	5	6
1	2	2^1	0	0	0	0
2	2^1	0	2^2	0	0	0
3	0	2^2	0	0	0	0
4	0	0	0	2	0	0
5	0	0	0	0	2	0
6	0	0	0	0	0	2

(4x**2**, 4x$\mathbf{2^2}$) **a**

	1	2	3	4	5	6
1	2	2^2	0	0	0	0
2	2^2	2	0	0	0	0
3	0	0	0	2^2	0	0
4	0	0	2^2	0	0	0
5	0	0	0	0	2	0
6	0	0	0	0	0	2

(4x**2**, 4x$\mathbf{2^2}$) **b**

	1	2	3	4	5	6
1	2	2^2	0	0	0	0
2	2^2	0	0	0	0	0
3	0	0	2	2^2	0	0
4	0	0	2^2	0	0	0
5	0	0	0	0	2	0
6	0	0	0	0	0	2

(4x**2**, 4x$\mathbf{2^2}$) **c**

	1	2	3	4	5	6
1	2	2^2	0	0	0	0
2	2^2	0	2^2	0	0	0
3	0	2^2	0	0	0	0
4	0	0	0	2	0	0
5	0	0	0	0	2	0
6	0	0	0	0	0	2

(2x**2**, 6x$\mathbf{2^1}$) **a**

	1	2	3	4	5	6
1	0	2^1	0	2^1	0	0
2	2^1	0	2^1	0	0	0
3	0	2^1	0	0	0	0
4	2^1	0	0	0	0	0
5	0	0	0	0	2	0
6	0	0	0	0	0	2

(2x**2**, 6x$\mathbf{2^1}$) **b**

	1	2	3	4	5	6
1	0	2^1	0	0	0	0
2	2^1	0	2^1	0	0	0
3	0	2^1	0	0	0	0
4	0	0	0	0	0	2^1
5	0	0	0	0	2	0
6	0	0	0	2^1	0	2

(2x**2**, 6x$\mathbf{2^1}$) **c**

	1	2	3	4	5	6
1	2	2^1	0	0	0	0
2	2^1	0	2^1	0	0	0
3	0	2^1	0	0	0	0
4	0	0	0	0	0	2^1
5	0	0	0	0	2	0
6	0	0	0	2^1	0	0

(2x**2**, 6x$\mathbf{2^1}$) **d**

	1	2	3	4	5	6
1	2	2^1	0	0	0	0
2	2^1	0	0	0	0	0
3	0	0	2	2^1	0	0
4	0	0	2^1	0	0	0
5	0	0	0	0	0	2^1
6	0	0	0	0	2^1	0

(2x**2**, 6x$\mathbf{2^1}$) **e**

	1	2	3	4	5	6
1	2	2^1	0	0	0	0
2	2^1	2	0	0	0	0
3	0	0	0	2^1	0	0
4	0	0	2^1	0	0	0
5	0	0	0	0	0	2^1
6	0	0	0	0	2^1	0

(2x**2**, 4x$\mathbf{2^1}$, 2x$\mathbf{2^2}$) **a**

	1	2	3	4	5	6
1	0	2^1	0	2^2	0	0
2	2^1	0	2^1	0	0	0
3	0	2^1	0	0	0	0
4	2^2	0	0	0	0	0
5	0	0	0	0	2	0
6	0	0	0	0	0	2

(2x**2**, 4x$\mathbf{2^1}$, 2x$\mathbf{2^2}$) **b**

	1	2	3	4	5	6
1	0	2^2	0	2^1	0	0
2	2^2	0	2^1	0	0	0
3	0	2^1	0	0	0	0
4	2^1	0	0	0	0	0
5	0	0	0	0	2	0
6	0	0	0	0	0	2

(2x**2**, 4x$\mathbf{2^1}$, 2x$\mathbf{2^2}$) **c**

	1	2	3	4	5	6
1	0	2^1	0	0	0	0
2	2^1	0	2^1	0	0	0
3	0	2^1	0	0	0	0
4	0	0	0	0	0	2^2
5	0	0	0	0	2	0
6	0	0	0	2^2	0	2

(2x**2**, 4x$\mathbf{2^1}$, 2x$\mathbf{2^2}$) **d**

	1	2	3	4	5	6
1	0	2^2	0	0	0	0
2	2^2	0	2^1	0	0	0
3	0	2^1	0	0	0	0
4	0	0	0	0	0	2^1
5	0	0	0	0	2	0
6	0	0	0	2^1	0	2

(2x**2**, 4x$\mathbf{2^1}$, 2x$\mathbf{2^2}$) **e**

	1	2	3	4	5	6
1	2	2^1	0	0	0	0
2	2^1	0	2^1	0	0	0
3	0	2^1	0	0	0	0
4	0	0	0	0	0	2^2
5	0	0	0	0	2	0
6	0	0	0	2^2	0	0

(2x**2**, 4x$\mathbf{2^1}$, 2x$\mathbf{2^2}$) **f**

	1	2	3	4	5	6
1	2	2^2	0	0	0	0
2	2^2	0	2^1	0	0	0
3	0	2^1	0	0	0	0
4	0	0	0	0	0	2^1
5	0	0	0	0	2	0
6	0	0	0	2^1	0	0

(2x**2**, 4x$\mathbf{2^1}$, 2x$\mathbf{2^2}$) **g**

	1	2	3	4	5	6
1	2	2^1	0	0	0	0
2	2^1	0	2^2	0	0	0
3	0	2^2	0	0	0	0
4	0	0	0	0	0	2^1
5	0	0	0	0	2	0
6	0	0	0	2^1	0	0

(2x**2**, 4x$\mathbf{2^1}$, 2x$\mathbf{2^2}$) **h**

	1	2	3	4	5	6
1	2	2^2	0	0	0	0
2	2^2	0	0	0	0	0
3	0	0	2	2^1	0	0
4	0	0	2^1	0	0	0
5	0	0	0	0	0	2^1
6	0	0	0	0	2^1	0

(2x**2**, 4x$\mathbf{2^1}$, 2x$\mathbf{2^2}$) **i**

	1	2	3	4	5	6
1	2	2^1	0	0	0	0
2	2^1	0	0	0	0	0
3	0	0	2	2^1	0	0
4	0	0	2^1	0	0	0
5	0	0	0	0	0	2^2
6	0	0	0	0	2^2	0

(2x**2**, 4x$\mathbf{2^1}$, 2x$\mathbf{2^2}$) **j**

	1	2	3	4	5	6
1	2	2^2	0	0	0	0
2	2^2	2	0	0	0	0
3	0	0	0	2^1	0	0
4	0	0	2^1	0	0	0
5	0	0	0	0	0	2^1
6	0	0	0	0	2^1	0

(2x**2**, 4x$\mathbf{2^1}$, 2x$\mathbf{2^2}$) **k**

	1	2	3	4	5	6
1	2	2^1	0	0	0	0
2	2^1	2	0	0	0	0
3	0	0	0	2^1	0	0
4	0	0	2^1	0	0	0
5	0	0	0	0	0	2^2
6	0	0	0	0	2^2	0

(2x**2**, 2x$\mathbf{2^1}$, 4x$\mathbf{2^2}$) **a**

	1	2	3	4	5	6
1	0	2^1	0	2^2	0	0
2	2^1	0	2^2	0	0	0
3	0	2^2	0	0	0	0
4	2^2	0	0	0	0	0
5	0	0	0	0	2	0
6	0	0	0	0	0	2

(2x**2**, 2x$\mathbf{2^1}$, 4x$\mathbf{2^2}$) **b**

	1	2	3	4	5	6
1	0	2^2	0	2^2	0	0
2	2^2	0	2^1	0	0	0
3	0	2^1	0	0	0	0
4	2^2	0	0	0	0	0
5	0	0	0	0	2	0
6	0	0	0	0	0	2

(2x**2**, 2x$\mathbf{2^1}$, 4x$\mathbf{2^2}$) **c**

	1	2	3	4	5	6
1	0	2^2	0	0	0	0
2	2^2	0	2^1	0	0	0
3	0	2^1	0	0	0	0
4	0	0	0	0	0	2^2
5	0	0	0	0	2	0
6	0	0	0	2^2	0	2

(2x**2**, 2x$\mathbf{2^1}$, 4x$\mathbf{2^2}$) **d**

	1	2	3	4	5	6
1	0	2^2	0	0	0	0
2	2^2	0	2^2	0	0	0
3	0	2^2	0	0	0	0
4	0	0	0	0	0	2^1
5	0	0	0	0	2	0
6	0	0	0	2^1	0	2

(2x**2**, 2x$\mathbf{2^1}$, 4x$\mathbf{2^2}$) **e**

	1	2	3	4	5	6
1	2	2^2	0	0	0	0
2	2^2	0	2^1	0	0	0
3	0	2^1	0	0	0	0
4	0	0	0	0	0	2^2
5	0	0	0	0	2	0
6	0	0	0	2^2	0	0

(2x**2**, 2x$\mathbf{2^1}$, 4x$\mathbf{2^2}$) **f**

	1	2	3	4	5	6
1	2	2^2	0	0	0	0
2	2^2	0	2^2	0	0	0
3	0	2^2	0	0	0	0
4	0	0	0	0	0	2^1
5	0	0	0	0	2	0
6	0	0	0	2^1	0	0

(2x**2**, 2x$\mathbf{2^1}$, 4x$\mathbf{2^2}$) **g**

	1	2	3	4	5	6
1	2	2^1	0	0	0	0
2	2^1	0	2^2	0	0	0
3	0	2^2	0	0	0	0
4	0	0	0	0	0	2^2
5	0	0	0	0	2	0
6	0	0	0	2^2	0	0

(2x**2**, 2x$\mathbf{2^1}$, 4x$\mathbf{2^2}$) **h**

	1	2	3	4	5	6
1	2	2^2	0	0	0	0
2	2^2	0	0	0	0	0
3	0	0	2	2^2	0	0
4	0	0	2^2	0	0	0
5	0	0	0	0	0	2^1
6	0	0	0	0	2^1	0

(2x**2**, 2x$\mathbf{2^1}$, 4x$\mathbf{2^2}$) **i**

	1	2	3	4	5	6
1	2	2^2	0	0	0	0
2	2^2	0	0	0	0	0
3	0	0	2	2^1	0	0
4	0	0	2^1	0	0	0
5	0	0	0	0	0	2^2
6	0	0	0	0	2^2	0

(2x**2**, 2x$\mathbf{2^1}$, 4x$\mathbf{2^2}$) **j**

	1	2	3	4	5	6
1	2	2^2	0	0	0	0
2	2^2	2	0	0	0	0
3	0	0	0	2^2	0	0
4	0	0	2^2	0	0	0
5	0	0	0	0	0	2^1
6	0	0	0	0	2^1	0

(2x**2**, 2x$\mathbf{2^1}$, 4x$\mathbf{2^2}$) **k**

	1	2	3	4	5	6
1	2	2^1	0	0	0	0
2	2^1	2	0	0	0	0
3	0	0	0	2^2	0	0
4	0	0	2^2	0	0	0
5	0	0	0	0	0	2^2
6	0	0	0	0	2^2	0

(2x**2**, 6x**2**^{2}) **a**

	1	2	3	4	5	6
1	0	2^1	0	2^2	0	0
2	2^1	0	2^2	0	0	0
3	0	2^2	0	0	0	0
4	2^2	0	0	0	0	0
5	0	0	0	0	2	0
6	0	0	0	0	0	2

(2x**2**, 6x**2**^{2}) **b**

	1	2	3	4	5	6
1	0	2^2	0	0	0	0
2	2^2	0	2^2	0	0	0
3	0	2^2	0	0	0	0
4	0	0	0	0	0	2^2
5	0	0	0	0	2	0
6	0	0	0	2^2	0	2

(2x**2**, 6x**2**^{2}) **c**

	1	2	3	4	5	6
1	2	2^2	0	0	0	0
2	2^2	0	2^2	0	0	0
3	0	2^2	0	0	0	0
4	0	0	0	0	0	2^2
5	0	0	0	0	2	0
6	0	0	0	2^2	0	0

(2x**2**, 6x**2**^{2}) **d**

	1	2	3	4	5	6
1	2	2^2	0	0	0	0
2	2^2	0	0	0	0	0
3	0	0	2	2^2	0	0
4	0	0	2^2	0	0	0
5	0	0	0	0	0	2^2
6	0	0	0	0	2^2	0

(2x**2**, 6x**2**^{2}) **e**

	1	2	3	4	5	6
1	2	2^2	0	0	0	0
2	2^2	2	0	0	0	0
3	0	0	0	2^2	0	0
4	0	0	2^2	0	0	0
5	0	0	0	0	0	2^2
6	0	0	0	0	2^2	0

(8x**2**^{1}) **a**

	1	2	3	4	5	6
1	0	2^1	0	0	0	0
2	2^1	0	2^1	0	0	0
3	0	2^1	0	0	0	0
4	0	0	0	0	2^1	0
5	0	0	0	2^1	0	2^1
6	0	0	0	0	2^1	0

(8x**2**^{1}) **b**

	1	2	3	4	5	6
1	0	2^1	0	2^1	0	0
2	2^1	0	2^1	0	0	0
3	0	2^1	0	0	0	0
4	2^1	0	0	0	0	0
5	0	0	0	0	0	2^1
6	0	0	0	0	2^1	0

(6x**2**^{1}, 2x**2**^{2}) **a**

	1	2	3	4	5	6
1	0	2^1	0	0	0	0
2	2^1	0	2^1	0	0	0
3	0	2^1	0	0	0	0
4	0	0	0	0	2^1	0
5	0	0	0	2^1	0	2^2
6	0	0	0	0	2^2	0

(6x**2**^{1}, 2x**2**^{2}) **b**

	1	2	3	4	5	6
1	0	2^1	0	2^2	0	0
2	2^1	0	2^1	0	0	0
3	0	2^1	0	0	0	0
4	2^2	0	0	0	0	0
5	0	0	0	0	0	2^1
6	0	0	0	0	2^1	0

(6x**2**^{1}, 2x**2**^{2}) **c**

	1	2	3	4	5	6
1	0	2^2	0	2^1	0	0
2	2^2	0	2^1	0	0	0
3	0	2^1	0	0	0	0
4	2^1	0	0	0	0	0
5	0	0	0	0	0	2^1
6	0	0	0	0	2^1	0

(6x**2**^{1}, 2x**2**^{2}) **d**

	1	2	3	4	5	6
1	0	2^1	0	2^1	0	0
2	2^1	0	2^1	0	0	0
3	0	2^1	0	0	0	0
4	2^1	0	0	0	0	0
5	0	0	0	0	0	2^2
6	0	0	0	0	2^2	0

(4x**2**^{1}, 4x**2**^{2}) **a**

	1	2	3	4	5	6
1	0	2^1	0	0	0	0
2	2^1	0	2^1	0	0	0
3	0	2^1	0	0	0	0
4	0	0	0	0	2^2	0
5	0	0	0	2^2	0	2^2
6	0	0	0	0	2^2	0

(4x**2**^{1}, 4x**2**^{2}) **b**

	1	2	3	4	5	6
1	0	2^2	0	0	0	0
2	2^2	0	2^1	0	0	0
3	0	2^1	0	0	0	0
4	0	0	0	0	2^1	0
5	0	0	0	2^1	0	2^2
6	0	0	0	0	2^2	0

(4x**2**^{1}, 4x**2**^{2}) **c**

	1	2	3	4	5	6
1	0	2^2	0	2^2	0	0
2	2^2	0	2^1	0	0	0
3	0	2^1	0	0	0	0
4	2^2	0	0	0	0	0
5	0	0	0	0	0	2^1
6	0	0	0	0	2^1	0

(4x**2**^{1}, 4x**2**^{2}) **d**

	1	2	3	4	5	6
1	0	2^1	0	2^2	0	0
2	2^1	0	2^2	0	0	0
3	0	2^2	0	0	0	0
4	2^2	0	0	0	0	0
5	0	0	0	0	0	2^1
6	0	0	0	0	2^1	0

(4x**2**^{1}, 4x**2**^{2}) **e**

	1	2	3	4	5	6
1	0	2^1	0	2^2	0	0
2	2^1	0	2^1	0	0	0
3	0	2^1	0	0	0	0
4	2^2	0	0	0	0	0
5	0	0	0	0	0	2^2
6	0	0	0	0	2^2	0

(4x**2**^{1}, 4x**2**^{2}) **f**

	1	2	3	4	5	6
1	0	2^2	0	2^1	0	0
2	2^2	0	2^1	0	0	0
3	0	2^1	0	0	0	0
4	2^1	0	0	0	0	0
5	0	0	0	0	0	2^2
6	0	0	0	0	2^2	0

(2x**2**^{1}, 6x**2**^{2}) **a**

	1	2	3	4	5	6
1	0	2^2	0	0	0	0
2	2^2	0	2^1	0	0	0
3	0	2^1	0	0	0	0
4	0	0	0	0	2^2	0
5	0	0	0	2^2	0	2^2
6	0	0	0	0	2^2	0

(2x**2**^{1}, 6x**2**^{2}) **b**

	1	2	3	4	5	6
1	0	2^2	0	2^2	0	0
2	2^2	0	2^2	0	0	0
3	0	2^2	0	0	0	0
4	2^2	0	0	0	0	0
5	0	0	0	0	0	2^1
6	0	0	0	0	2^1	0

(2x**2**^{1}, 6x**2**^{2}) **c**

	1	2	3	4	5	6
1	0	2^2	0	2^2	0	0
2	2^2	0	2^1	0	0	0
3	0	2^1	0	0	0	0
4	2^2	0	0	0	0	0
5	0	0	0	0	0	2^2
6	0	0	0	0	2^2	0

(2x**2**^{1}, 6x**2**^{2}) **d**

	1	2	3	4	5	6
1	0	2^1	0	2^2	0	0
2	2^1	0	2^2	0	0	0
3	0	2^2	0	0	0	0
4	2^2	0	0	0	0	0
5	0	0	0	0	0	2^2
6	0	0	0	0	2^2	0

(8x**2**^{2}) **a**

	1	2	3	4	5	6
1	0	2^2	0	0	0	0
2	2^2	0	2^2	0	0	0
3	0	2^2	0	0	0	0
4	0	0	0	0	2^2	0
5	0	0	0	2^2	0	2^2
6	0	0	0	0	2^2	0

(8x**2**^{2}) **b**

	1	2	3	4	5	6
1	0	2^2	0	2^2	0	0
2	2^2	0	2^2	0	0	0
3	0	2^2	0	0	0	0
4	2^2	0	0	0	0	0
5	0	0	0	0	0	2^2
6	0	0	0	0	2^2	0

$^2V_4{}^4V_3$ NG

$^2V_4{}^4V_4$ NG

$^2V_5{}^4V_1$

(14x**1**)

	1	2	3	4	5	6
1	0	0	1	1	0	0
2	0	0	1	1	1	1
3	1	1	0	0	0	0
4	1	1	0	0	0	0
5	0	1	0	0	0	1
6	0	1	0	0	1	0

(12x**1**, 1x**2**) **a**

	1	2	3	4	5	6
1	2	0	0	0	0	0
2	0	0	1	1	1	1
3	0	1	0	1	0	0
4	0	1	1	0	0	0
5	0	1	0	0	0	1
6	0	1	0	0	1	0

(12x**1**, 1x**2**) **b**

	1	2	3	4	5	6
1	2	0	1	1	0	0
2	0	0	1	0	0	1
3	1	1	0	0	0	0
4	1	0	0	0	1	0
5	0	0	0	1	0	1
6	0	1	0	0	1	0

(12x**1**, 1x**2**) **c**

	1	2	3	4	5	6
1	2	0	1	1	0	0
2	0	0	0	0	1	1
3	1	0	0	1	0	0
4	1	0	1	0	0	0
5	0	1	0	0	0	1
6	0	1	0	0	1	0

(10x**1**, 2x**2**)

	1	2	3	4	5	6
1	2	0	0	0	0	0
2	0	2	1	0	0	1
3	0	1	0	1	0	0
4	0	0	1	0	1	0
5	0	0	0	1	0	1
6	0	1	0	0	1	0

(10x**1**, 2x**2**^{1})

	1	2	3	4	5	6
1	0	0	0	0	0	2^1
2	0	0	1	0	0	1
3	0	1	0	1	0	0
4	0	0	1	0	1	0
5	0	0	0	1	0	1
6	2^1	1	0	0	1	0

(10x**1**, 2x**2**^{2})

	1	2	3	4	5	6
1	0	0	0	0	0	2^2
2	0	0	1	0	0	1
3	0	1	0	1	0	0
4	0	0	1	0	1	0
5	0	0	0	1	0	1
6	2^2	1	0	0	1	0

(8x**1**, 3x**2**)

	1	2	3	4	5	6
1	2	0	0	0	0	0
2	0	0	1	0	1	0
3	0	1	0	1	0	0
4	0	0	1	2	1	0
5	0	1	0	1	0	0
6	0	0	0	0	0	2

(8x**1**, 1x**2**, 2x**2**^{1}) **a**

	1	2	3	4	5	6
1	0	1	0	1	0	0
2	1	0	1	0	0	0
3	0	1	0	1	0	0
4	1	0	1	0	0	0
5	0	0	0	0	0	2^1
6	0	0	0	0	2^1	2

(8x**1**, 1x**2**, 2x**2**^{1}) **b**

	1	2	3	4	5	6
1	0	1	0	1	0	0
2	1	2	1	0	0	0
3	0	1	0	1	0	0
4	1	0	1	0	0	0
5	0	0	0	0	0	2^1
6	0	0	0	0	2^1	0

(8x**1**, 1x**2**, 2x**2**^{1}) **c**

	1	2	3	4	5	6
1	0	0	1	1	0	0
2	0	0	1	1	0	2^1
3	1	1	0	0	0	0
4	1	1	0	0	0	0
5	0	0	0	0	2	0
6	0	2^1	0	0	0	0

(8x**1**, 1x**2**, 2x**2**^{2}) **a**

	1	2	3	4	5	6
1	0	1	0	1	0	0
2	1	0	1	0	0	0
3	0	1	0	1	0	0
4	1	0	1	0	0	0
5	0	0	0	0	0	2^2
6	0	0	0	0	2^2	2

(8x**1**, 1x**2**, 2x**2**^{2}) **b**

	1	2	3	4	5	6
1	0	1	0	1	0	0
2	1	2	1	0	0	0
3	0	1	0	1	0	0
4	1	0	1	0	0	0
5	0	0	0	0	0	2^2
6	0	0	0	0	2^2	0

(8x**1**, 1x**2**, 2x**2**^{2}) **c**

	1	2	3	4	5	6
1	0	0	1	1	0	0
2	0	0	1	1	0	2^2
3	1	1	0	0	0	0
4	1	1	0	0	0	0
5	0	0	0	0	2	0
6	0	2^2	0	0	0	0

(6x**1**, 4x**2**)

	1	2	3	4	5	6
1	2	0	0	0	0	0
2	0	2	0	0	0	0
3	0	0	0	1	1	0
4	0	0	1	2	1	0
5	0	0	1	1	0	0
6	0	0	0	0	0	2

(6x**1**, 2x**2**, 2x**2**^{1}) **a**

	1	2	3	4	5	6
1	0	0	0	2^1	0	0
2	0	0	1	1	0	0
3	0	1	0	1	0	0
4	2^1	1	1	0	0	0
5	0	0	0	0	2	0
6	0	0	0	0	0	2

(6x**1**, 2x**2**, 2x**2**^{1}) **b**

	1	2	3	4	5	6
1	0	0	0	2^1	0	0
2	0	0	1	0	0	1
3	0	1	0	0	0	1
4	2^1	0	0	2	0	0
5	0	0	0	0	2	0
6	0	1	1	0	0	0

(6x**1**, 2x**2**, 2x**2**^{1}) **c**

	1	2	3	4	5	6
1	0	0	0	2^1	0	0
2	0	0	1	0	0	1
3	0	1	0	0	0	1
4	2^1	0	0	0	0	0
5	0	0	0	0	2	0
6	0	1	1	0	0	2

(6x**1**, 2x**2**, 2x**2**^{2}) **a**

	1	2	3	4	5	6
1	0	0	0	2^2	0	0
2	0	0	1	1	0	0
3	0	1	0	1	0	0
4	2^2	1	1	0	0	0
5	0	0	0	0	2	0
6	0	0	0	0	0	2

(6x**1**, 2x**2**, 2x**2**^{2}) **b**

	1	2	3	4	5	6
1	0	0	0	2^2	0	0
2	0	0	1	0	0	1
3	0	1	0	0	0	1
4	2^2	0	0	2	0	0
5	0	0	0	0	2	0
6	0	1	1	0	0	0

(6x**1**, 2x**2**, 2x**2**^{2}) **c**

	1	2	3	4	5	6
1	0	0	0	2^2	0	0
2	0	0	1	0	0	1
3	0	1	0	0	0	1
4	2^2	0	0	0	0	0
5	0	0	0	0	2	0
6	0	1	1	0	0	2

(6x**1**, 4x**2**^{1}) **a**

	1	2	3	4	5	6
1	0	1	1	0	0	0
2	1	0	1	0	0	2^1
3	1	1	0	0	0	0
4	0	0	0	0	2^1	0
5	0	0	0	2^1	0	0
6	0	2^1	0	0	0	0

(6x**1**, 4x**2**^{1}) **b**

	1	2	3	4	5	6
1	0	0	1	1	0	0
2	0	0	0	0	0	2^1
3	1	0	0	1	0	0
4	1	0	1	0	0	0
5	0	0	0	0	0	2^1
6	0	2^1	0	0	2^1	0

(6x**1**, 2x**2**^{1}, 2x**2**^{2}) **a**

	1	2	3	4	5	6
1	0	1	1	0	0	0
2	1	0	1	0	0	2^2
3	1	1	0	0	0	0
4	0	0	0	0	2^1	0
5	0	0	0	2^1	0	0
6	0	2^2	0	0	0	0

(6x**1**, 2x**2**^{1}, 2x**2**^{2}) **b**

	1	2	3	4	5	6
1	0	1	1	0	0	0
2	1	0	1	0	0	2^1
3	1	1	0	0	0	0
4	0	0	0	0	2^2	0
5	0	0	0	2^2	0	0
6	0	2^1	0	0	0	0

(6x**1**, 2x**2**^{1}, 2x**2**^{2}) **c**

	1	2	3	4	5	6
1	0	0	1	1	0	0
2	0	0	0	0	0	2^1
3	1	0	0	1	0	0
4	1	0	1	0	0	0
5	0	0	0	0	0	2^2
6	0	2^1	0	0	2^2	0

(6x**1**, 4x**2**^{2}) **a**

	1	2	3	4	5	6
1	0	1	1	0	0	0
2	1	0	1	0	0	2^2
3	1	1	0	0	0	0
4	0	0	0	0	2^2	0
5	0	0	0	2^2	0	0
6	0	2^2	0	0	0	0

(6x**1**, 4x**2**^{2}) **b**

	1	2	3	4	5	6
1	0	0	1	1	0	0
2	0	0	0	0	0	2^2
3	1	0	0	1	0	0
4	1	0	1	0	0	0
5	0	0	0	0	0	2^2
6	0	2^2	0	0	2^2	0

(5x**2**, 2x**2**^{1})

	1	2	3	4	5	6
1	2	2^1	0	0	0	0
2	2^1	0	0	0	0	0
3	0	0	2	0	0	0
4	0	0	0	2	0	0
5	0	0	0	0	2	0
6	0	0	0	0	0	2

(5x**2**, 2x**2**^{2})

	1	2	3	4	5	6
1	2	2^2	0	0	0	0
2	2^2	0	0	0	0	0
3	0	0	2	0	0	0
4	0	0	0	2	0	0
5	0	0	0	0	2	0
6	0	0	0	0	0	2

(3x**2**, 4x**2**^{1}) **a**

	1	2	3	4	5	6
1	2	2^1	0	0	0	0
2	2^1	0	0	0	0	0
3	0	0	0	2^1	0	0
4	0	0	2^1	0	0	0
5	0	0	0	0	2	0
6	0	0	0	0	0	2

(3x**2**, 4x**2**^{1}) **b**

	1	2	3	4	5	6
1	0	2^1	0	0	0	0
2	2^1	0	2^1	0	0	0
3	0	2^1	0	0	0	0
4	0	0	0	2	0	0
5	0	0	0	0	2	0
6	0	0	0	0	0	2

(3x**2**, 2x**2**^{1}, 2x**2**^{2}) **a**

	1	2	3	4	5	6
1	2	2^1	0	0	0	0
2	2^1	0	0	0	0	0
3	0	0	0	2^2	0	0
4	0	0	2^2	0	0	0
5	0	0	0	0	2	0
6	0	0	0	0	0	2

(3x**2**, 2x**2**^{1}, 2x**2**^{2}) **b**

	1	2	3	4	5	6
1	2	2^2	0	0	0	0
2	2^2	0	0	0	0	0
3	0	0	0	2^1	0	0
4	0	0	2^1	0	0	0
5	0	0	0	0	2	0
6	0	0	0	0	0	2

(3x**2**, 2x**2**^{1}, 2x**2**^{2}) **c**

	1	2	3	4	5	6
1	0	2^2	0	0	0	0
2	2^2	0	2^1	0	0	0
3	0	2^1	0	0	0	0
4	0	0	0	2	0	0
5	0	0	0	0	2	0
6	0	0	0	0	0	2

(3x**2**, 4x**2**^{2}) **a**

	1	2	3	4	5	6
1	2	2^2	0	0	0	0
2	2^2	0	0	0	0	0
3	0	0	0	2^2	0	0
4	0	0	2^2	0	0	0
5	0	0	0	0	2	0
6	0	0	0	0	0	2

(3x**2**, 4x**2**^{2}) **b**

	1	2	3	4	5	6
1	0	2^2	0	0	0	0
2	2^2	0	2^2	0	0	0
3	0	2^2	0	0	0	0
4	0	0	0	2	0	0
5	0	0	0	0	2	0
6	0	0	0	0	0	2

(1x**2**, 6x**2**^{1}) **a**

	1	2	3	4	5	6
1	0	2^1	0	0	0	0
2	2^1	0	2^1	0	0	0
3	0	2^1	0	0	0	0
4	0	0	0	0	0	2^1
5	0	0	0	0	2	0
6	0	0	0	2^1	0	0

(1x**2**, 6x**2**^{1}) **b**

	1	2	3	4	5	6
1	2	2^1	0	0	0	0
2	2^1	0	0	0	0	0
3	0	0	0	2^1	0	0
4	0	0	2^1	0	0	0
5	0	0	0	0	0	2^1
6	0	0	0	0	2^1	0

(1x**2**, 4x**2**^{1}, 2x**2**^{2}) **a**

	1	2	3	4	5	6
1	0	2^1	0	0	0	0
2	2^1	0	2^1	0	0	0
3	0	2^1	0	0	0	0
4	0	0	0	0	0	2^2
5	0	0	0	0	2	0
6	0	0	0	2^2	0	0

(1x**2**, 4x**2**^{1}, 2x**2**^{2}) **b**

	1	2	3	4	5	6
1	0	2^2	0	0	0	0
2	2^2	0	2^1	0	0	0
3	0	2^1	0	0	0	0
4	0	0	0	0	0	2^1
5	0	0	0	0	2	0
6	0	0	0	2^1	0	0

(1x**2**, 4x**2**^{1}, 2x**2**^{2}) **c**

	1	2	3	4	5	6
1	2	2^2	0	0	0	0
2	2^2	0	0	0	0	0
3	0	0	0	2^1	0	0
4	0	0	2^1	0	0	0
5	0	0	0	0	0	2^1
6	0	0	0	0	2^1	0

(1x**2**, 4x**2**^{1}, 2x**2**^{2}) **d**

	1	2	3	4	5	6
1	2	2^1	0	0	0	0
2	2^1	0	0	0	0	0
3	0	0	0	2^2	0	0
4	0	0	2^2	0	0	0
5	0	0	0	0	0	2^1
6	0	0	0	0	2^1	0

(1x**2**, 2x**2**^{1}, 4x**2**^{2}) **a**

	1	2	3	4	5	6
1	0	2^2	0	0	0	0
2	2^2	0	2^1	0	0	0
3	0	2^1	0	0	0	0
4	0	0	0	0	0	2^2
5	0	0	0	0	2	0
6	0	0	0	2^2	0	0

(1x**2**, 2x**2**^{1}, 4x**2**^{2}) **b**

	1	2	3	4	5	6
1	0	2^2	0	0	0	0
2	2^2	0	2^2	0	0	0
3	0	2^2	0	0	0	0
4	0	0	0	0	0	2^1
5	0	0	0	0	2	0
6	0	0	0	2^1	0	0

(1x**2**, 2x**2**^{1}, 4x**2**^{2}) **c**

	1	2	3	4	5	6
1	2	2^2	0	0	0	0
2	2^2	0	0	0	0	0
3	0	0	0	2^2	0	0
4	0	0	2^2	0	0	0
5	0	0	0	0	0	2^1
6	0	0	0	0	2^1	0

(1x**2**, 2x**2**^{1}, 4x**2**^{2}) **d**

	1	2	3	4	5	6
1	2	2^1	0	0	0	0
2	2^1	0	0	0	0	0
3	0	0	0	2^2	0	0
4	0	0	2^2	0	0	0
5	0	0	0	0	0	2^2
6	0	0	0	0	2^2	0

(1x**2**, 6x**2**^{2}) **a**

	1	2	3	4	5	6
1	0	2^2	0	0	0	0
2	2^2	0	2^2	0	0	0
3	0	2^2	0	0	0	0
4	0	0	0	0	0	2^2
5	0	0	0	0	2	0
6	0	0	0	2^2	0	0

(1x**2**, 6x**2**^{2}) **b**

	1	2	3	4	5	6
1	2	2^2	0	0	0	0
2	2^2	0	0	0	0	0
3	0	0	0	2^2	0	0
4	0	0	2^2	0	0	0
5	0	0	0	0	0	2^2
6	0	0	0	0	2^2	0

$^2V_5{}^4V_2$ NG

$^2V_5{}^4V_3$ NG

$^2V_6{}^4V_1$ NG

$^2V_6{}^4V_2$ NG

$^2V_7{}^4V_1$ NG

$^3V_r{}^4V_r$

$^3V_2{}^4V_1$

(4x**1**, 3x**2**)

	1	2	3
1	2	1	0
2	1	2	1
3	0	1	2

(4x**1**, 1x**2**, 2x**2**^{1})

	1	2	3
1	0	1	2^1
2	1	2	1
3	2^1	1	0

(4x**1**, 1x**2**, 2x**2**^{2})

	1	2	3
1	0	1	2^2
2	1	2	1
3	2^2	1	0

(2x**1**, 2x**2**, 2x**2**^{1})

	1	2	3
1	2	0	2^1
2	0	2	1
3	2^1	1	0

(2x**1**, 2x**2**, 2x**2**^{2})

	1	2	3
1	2	0	2^1
2	0	2	1
3	2^1	1	0

(2x**1**, 4x**2**^{1})

	1	2	3
1	0	1	2^1
2	1	0	2^1
3	2^1	2^1	0

(2x**1**, 2x**2**^{1}, 2x**2**^{2})

	1	2	3
1	0	1	2^1
2	1	0	2^2
3	2^1	2^2	0

(2x**1**, 4x**2**^{2})

	1	2	3
1	0	1	2^2
2	1	0	2^2
3	2^2	2^2	0

$^3V_2{}^4V_2$

(10x**1**, 2x**2**)

	1	2	3	4
1	2	0	1	1
2	0	2	1	1
3	1	1	0	1
4	1	1	1	0

(10x**1**, 2x**2**^{1})

	1	2	3	4
1	0	1	1	1
2	1	0	2^1	1
3	1	2^1	0	1
4	1	1	1	0

(10x**1**, 2x**2**^{2})

	1	2	3	4
1	0	1	1	1
2	1	0	2^2	1
3	1	2^2	0	1
4	1	1	1	0

(8x**1**, 3x**2**)

	1	2	3	4
1	2	1	1	0
2	1	2	1	0
3	1	1	0	1
4	0	0	1	2

(8x**1**, 1x**2**, 2x**2**^{1})

	1	2	3	4
1	2	0	1	1
2	0	0	2^1	1
3	1	2^1	0	1
4	1	1	1	0

(8x**1**, 1x**2**, 2x**2**^{2})

	1	2	3	4
1	2	0	1	1
2	0	0	2^2	1
3	1	2^2	0	1
4	1	1	1	0

(6x**1**, 4x**2**)

	1	2	3	4
1	2	1	1	0
2	1	2	0	0
3	1	0	2	1
4	0	0	1	2

(6x**1**, 2x**2**, 2x**2**^{1}) **a**

	1	2	3	4
1	0	2^1	0	1
2	2^1	0	1	0
3	0	1	2	1
4	1	0	1	2

(6x**1**, 2x**2**, 2x**2**^{1}) **b**

	1	2	3	4
1	0	2^1	1	1
2	2^1	0	1	0
3	1	1	2	0
4	1	0	0	2

(6x**1**, 2x**2**, 2x**2**^{2}) **a**

	1	2	3	4
1	0	2^2	0	1
2	2^2	0	1	0
3	0	1	2	1
4	1	0	1	2

(6x**1**, 2x**2**, 2x**2**^{2}) **b**

	1	2	3	4
1	0	2^2	1	1
2	2^2	0	1	0
3	1	1	2	0
4	1	0	0	2

(6x**1**, 4x**2**^{1})

	1	2	3	4
1	0	0	1	2^1
2	0	0	2^1	1
3	1	2^1	0	1
4	2^1	1	1	0

(6x**1**, 2x**2**^{1}, 2x**2**^{2})

	1	2	3	4
1	0	0	1	2^1
2	0	0	2^2	1
3	1	2^2	0	1
4	2^1	1	1	0

(6x**1**, 4x**2**^{2})

	1	2	3	4
1	0	0	1	2^2
2	0	0	2^2	1
3	1	2^2	0	1
4	2^2	1	1	0

(4x**1**, 3x**2**, 2x**2**^{1}) **a**

	1	2	3	4
1	0	2^1	1	1
2	2^1	2	0	0
3	1	0	2	0
4	1	0	0	2

(4x**1**, 3x**2**, 2x**2**^{1}) **b**

	1	2	3	4
1	0	2^1	1	0
2	2^1	2	0	0
3	1	0	2	1
4	0	0	1	2

(4x**1**, 3x**2**, 2x**2**^{2}) **a**

	1	2	3	4
1	0	2^2	1	1
2	2^2	2	0	0
3	1	0	2	0
4	1	0	0	2

(4x**1**, 3x**2**, 2x**2**^{2}) **b**

	1	2	3	4
1	0	2^2	1	0
2	2^2	2	0	0
3	1	0	2	1
4	0	0	1	2

(4x**1**, 1x**2**, 4x**2**^{1}) **a**

	1	2	3	4
1	0	2^1	1	0
2	2^1	0	1	0
3	1	1	0	2^1
4	0	0	2^1	2

(4x**1**, 1x**2**, 4x**2**^{1}) **b**

	1	2	3	4
1	0	2^1	1	0
2	2^1	0	2^1	0
3	1	2^1	0	1
4	0	0	1	2

(4x**1**, 1x**2**, 4x**2**^{1}) **c**

	1	2	3	4
1	0	2^1	0	1
2	2^1	0	2^1	0
3	0	2^1	0	1
4	1	0	1	2

(4x**1**, 1x**2**, 2x**2**^{1}, 2x**2**^{2}) **a**

	1	2	3	4
1	0	2^2	1	0
2	2^2	0	1	0
3	1	1	0	2^1
4	0	0	2^1	2

(4x**1**, 1x**2**, 2x**2**^{1}, 2x**2**^{2}) **b**

	1	2	3	4
1	0	2^1	1	0
2	2^1	0	1	0
3	1	1	0	2^2
4	0	0	2^2	2

(4x**1**, 1x**2**, 2x**2**^{1}, 2x**2**^{2}) **c**

	1	2	3	4
1	0	2^2	1	0
2	2^2	0	2^1	0
3	1	2^1	0	1
4	0	0	1	2

(4x**1**, 1x**2**, 2x**2**^{1}, 2x**2**^{2}) **d**

	1	2	3	4
1	0	2^1	1	0
2	2^1	0	2^2	0
3	1	2^2	0	1
4	0	0	1	2

(4x**1**, 1x**2**, 2x**2**^{1}, 2x**2**^{2}) **e**

	1	2	3	4
1	0	2^2	0	1
2	2^2	0	2^1	0
3	0	2^1	0	1
4	1	0	1	2

(4x**1**, 1x**2**, 4x**2**^{2}) **a**

	1	2	3	4
1	0	2^2	1	0
2	2^2	0	1	0
3	1	1	0	2^2
4	0	0	2^2	2

(4x**1**, 1x**2**, 4x**2**^{2}) **b**

	1	2	3	4
1	0	2^2	1	0
2	2^2	0	2^2	0
3	1	2^2	0	1
4	0	0	1	2

(4x**1**, 1x**2**, 4x**2**^{2}) **c**

	1	2	3	4
1	0	2^2	0	1
2	2^2	0	2^2	0
3	0	2^2	0	1
4	1	0	1	2

(2x**1**, 4x**2**, 2x**2**^{1})

	1	2	3	4
1	2	1	0	0
2	1	2	0	0
3	0	0	2	2^1
4	0	0	2^1	2

(2x**1**, 4x**2**, 2x**2**^{2})

	1	2	3	4
1	2	1	0	0
2	1	2	0	0
3	0	0	2	2^2
4	0	0	2^2	2

(2x**1**, 2x**2**, 4x**2**^{1}) **a**

	1	2	3	4
1	0	2^1	2^1	0
2	2^1	2	0	0
3	2^1	0	0	1
4	0	0	1	2

(2x**1**, 2x**2**, 4x**2**^{1}) **b**

	1	2	3	4
1	0	2^1	1	0
2	2^1	2	0	0
3	1	0	0	2^1
4	0	0	2^1	2

(2x**1**, 2x**2**, 2x**2**^{1}, 2x**2**^{2}) **a**

	1	2	3	4
1	0	2^2	2^1	0
2	2^2	2	0	0
3	2^1	0	0	1
4	0	0	1	2

(2x**1**, 2x**2**, 2x**2**^{1}, 2x**2**^{2}) **b**

	1	2	3	4
1	0	2^1	2^2	0
2	2^1	2	0	0
3	2^2	0	0	1
4	0	0	1	2

(2x**1**, 2x**2**, 2x**2**^{1}, 2x**2**^{2}) **c**

	1	2	3	4
1	0	2^1	1	0
2	2^1	2	0	0
3	1	0	0	2^2
4	0	0	2^2	2

(2x**1**, 2x**2**, 4x**2**^{2}) **a**

	1	2	3	4
1	0	2^2	2^2	0
2	2^2	2	0	0
3	2^2	0	0	1
4	0	0	1	2

(2x**1**, 2x**2**, 4x**2**^{2}) **b**

	1	2	3	4
1	0	2^2	1	0
2	2^2	2	0	0
3	1	0	0	2^2
4	0	0	2^2	2

(2x**1**, 6x**2**^{1})

	1	2	3	4
1	0	2^1	0	1
2	2^1	0	2^1	0
3	0	2^1	0	2^1
4	1	0	2^1	0

(2x**1**, 4x**2**^{1}, 2x**2**^{2}) **a**

	1	2	3	4
1	0	2^2	0	1
2	2^2	0	2^1	0
3	0	2^1	0	2^1
4	1	0	2^1	0

(2x**1**, 4x**2**^{1}, 2x**2**^{2}) **b**

	1	2	3	4
1	0	2^1	0	1
2	2^1	0	2^2	0
3	0	2^2	0	2^1
4	1	0	2^1	0

(2x**1**, 2x**2**^{1}, 4x**2**^{2}) **a**

	1	2	3	4
1	0	2^2	0	1
2	2^2	0	2^1	0
3	0	2^1	0	2^2
4	1	0	2^2	0

(2x**1**, 2x**2**^{1}, 4x**2**^{2}) **b**

	1	2	3	4
1	0	2^2	0	1
2	2^2	0	2^2	0
3	0	2^2	0	2^1
4	1	0	2^1	0

(2x**1**, 6x**2**^{2})

	1	2	3	4
1	0	2^2	0	1
2	2^2	0	2^2	0
3	0	2^2	0	2^2
4	1	0	2^2	0

$^3V_2{}^4V_3$

(18x**1**)

	1	2	3	4	5
1	0	1	1	0	1
2	1	0	1	1	1
3	1	1	0	1	1
4	0	1	1	0	1
5	1	1	1	1	0

(16x**1**, 1x**2**)

	1	2	3	4	5
1	2	1	0	0	1
2	1	0	1	1	1
3	0	1	0	1	1
4	0	1	1	0	1
5	1	1	1	1	0

(14x**1**, 2x**2**)

	1	2	3	4	5
1	2	0	1	1	0
2	0	0	1	1	1
3	1	1	0	1	1
4	1	1	1	0	0
5	0	1	1	0	2

(14x**1**, 2x$\mathbf{2^1}$)

	1	2	3	4	5
1	0	1	1	0	1
2	1	0	0	2^1	1
3	1	0	0	1	1
4	0	2^1	1	0	1
5	1	1	1	1	0

(14x**1**, 2x$\mathbf{2^2}$)

	1	2	3	4	5
1	0	1	1	0	1
2	1	0	0	2^2	1
3	1	0	0	1	1
4	0	2^2	1	0	1
5	1	1	1	1	0

(12x**1**, 3x**2**) **a**

	1	2	3	4	5
1	2	1	1	0	0
2	1	0	0	1	1
3	1	0	0	1	1
4	0	1	1	2	0
5	0	1	1	0	2

(12x**1**, 3x**2**) **b**

	1	2	3	4	5
1	2	1	0	0	1
2	1	0	1	0	1
3	0	1	2	1	0
4	0	0	1	2	1
5	1	1	0	1	0

(12x**1**, 3x**2**) **c**

	1	2	3	4	5
1	2	0	1	1	0
2	0	2	1	1	0
3	1	1	0	1	0
4	1	1	1	0	1
5	0	0	0	1	2

(12x**1**, 1x**2**, 2x$\mathbf{2^1}$) **a**

	1	2	3	4	5
1	2	1	0	0	1
2	1	0	1	2^1	0
3	0	1	0	1	1
4	0	2^1	1	0	1
5	1	0	1	1	0

(12x**1**, 1x**2**, 2x$\mathbf{2^1}$) **b**

	1	2	3	4	5
1	2	1	0	0	1
2	1	0	1	1	0
3	0	1	0	2^1	1
4	0	1	2^1	0	1
5	1	0	1	1	0

(12x**1**, 1x**2**, 2x$\mathbf{2^1}$) **c**

	1	2	3	4	5
1	0	0	1	2^1	0
2	0	0	1	1	1
3	1	1	0	1	1
4	2^1	1	1	0	0
5	0	1	1	0	2

(12x**1**, 1x**2**, 2x$\mathbf{2^1}$) **d**

	1	2	3	4	5
1	0	0	2^1	1	1
2	0	2	0	1	0
3	2^1	0	0	1	1
4	1	1	1	0	1
5	1	0	1	1	0

(12x**1**, 1x**2**, 2x$\mathbf{2^2}$) **a**

	1	2	3	4	5
1	2	1	0	0	1
2	1	0	1	2^2	0
3	0	1	0	1	1
4	0	2^2	1	0	1
5	1	0	1	1	0

(12x**1**, 1x**2**, 2x$\mathbf{2^2}$) **b**

	1	2	3	4	5
1	2	1	0	0	1
2	1	0	1	1	0
3	0	1	0	2^2	1
4	0	1	2^2	0	1
5	1	0	1	1	0

(12x**1**, 1x**2**, 2x$\mathbf{2^2}$) **c**

	1	2	3	4	5
1	0	0	1	2^2	0
2	0	0	1	1	1
3	1	1	0	1	1
4	2^2	1	1	0	0
5	0	1	1	0	2

(12x**1**, 1x**2**, 2x$\mathbf{2^2}$) **d**

	1	2	3	4	5
1	0	0	2^2	1	1
2	0	2	0	1	0
3	2^2	0	0	1	1
4	1	1	1	0	1
5	1	0	1	1	0

(10x**1**, 4x**2**) **a**

	1	2	3	4	5
1	2	0	1	1	0
2	0	2	1	0	1
3	1	1	0	1	0
4	1	0	1	2	0
5	0	1	0	0	2

(10x**1**, 4x**2**) **b**

	1	2	3	4	5
1	2	0	1	1	0
2	0	2	1	0	0
3	1	1	0	1	1
4	1	0	1	2	0
5	0	0	1	0	2

(10x**1**, 4x**2**) **c**

	1	2	3	4	5
1	2	0	0	1	1
2	0	2	0	1	0
3	0	0	2	1	1
4	1	1	1	0	0
5	1	0	1	0	2

(10x**1**, 2x**2**, 2x$\mathbf{2^1}$) **a**

	1	2	3	4	5
1	2	1	1	0	0
2	1	0	0	1	2^1
3	1	0	0	1	1
4	0	1	1	2	0
5	0	2^1	1	0	0

(10x**1**, 2x**2**, 2x$\mathbf{2^1}$) **b**

	1	2	3	4	5
1	0	0	1	1	1
2	0	2	0	2^1	0
3	1	0	0	1	1
4	1	2^1	1	0	0
5	1	0	1	0	2

(10x**1**, 2x**2**, 2x$\mathbf{2^1}$) **c**

	1	2	3	4	5
1	0	0	1	2^1	1
2	0	2	0	1	0
3	1	0	0	1	1
4	2^1	1	1	0	0
5	1	0	1	0	2

(10x**1**, 2x**2**, 2x$\mathbf{2^1}$) **d**

	1	2	3	4	5
1	0	0	2^1	1	1
2	0	2	0	1	0
3	2^1	0	0	1	1
4	1	1	1	0	0
5	1	0	1	0	2

(10x**1**, 2x**2**, 2x$\mathbf{2^1}$) **e**

	1	2	3	4	5
1	2	0	0	1	1
2	0	2	0	1	0
3	0	0	0	1	2^1
4	1	1	1	0	1
5	1	0	2^1	1	0

(10x**1**, 2x**2**, 2x$\mathbf{2^1}$) **f**

	1	2	3	4	5
1	0	1	0	0	2^1
2	1	0	1	0	1
3	0	1	2	1	0
4	0	0	1	2	1
5	2^1	1	0	1	0

(10x**1**, 2x**2**, 2x$\mathbf{2^1}$) **g**

	1	2	3	4	5
1	2	1	0	0	1
2	1	0	2^1	0	1
3	0	2^1	0	1	0
4	0	0	1	2	1
5	1	1	0	1	0

(10x**1**, 2x**2**, 2x$\mathbf{2^1}$) **h**

	1	2	3	4	5
1	0	1	0	0	2^1
2	1	0	1	1	1
3	0	1	2	1	0
4	0	1	1	2	0
5	2^1	1	0	0	0

(10x**1**, 2x**2**, 2x$\mathbf{2^2}$) **a**

	1	2	3	4	5
1	2	1	1	0	0
2	1	0	0	1	2^2
3	1	0	0	1	1
4	0	1	1	2	0
5	0	2^2	1	0	0

(10x**1**, 2x**2**, 2x$\mathbf{2^2}$) **b**

	1	2	3	4	5
1	0	0	1	1	1
2	0	2	0	2^2	0
3	1	0	0	1	1
4	1	2^2	1	0	0
5	1	0	1	0	2

(10x**1**, 2x**2**, 2x$\mathbf{2^2}$) **c**

	1	2	3	4	5
1	0	0	1	2^2	1
2	0	2	0	1	0
3	1	0	0	1	1
4	2^2	1	1	0	0
5	1	0	1	0	2

(10x**1**, 2x**2**, 2x$\mathbf{2^2}$) **d**

	1	2	3	4	5
1	0	0	2^2	1	1
2	0	2	0	1	0
3	2^2	0	0	1	1
4	1	1	1	0	0
5	1	0	1	0	2

(10x**1**, 2x**2**, 2x$\mathbf{2^2}$) **e**

	1	2	3	4	5
1	2	0	0	1	1
2	0	2	0	1	0
3	0	0	0	1	2^2
4	1	1	1	0	1
5	1	0	2^2	1	0

(10x**1**, 2x**2**, 2x$\mathbf{2^2}$) **f**

	1	2	3	4	5
1	0	1	0	0	2^2
2	1	0	1	0	1
3	0	1	2	1	0
4	0	0	1	2	1
5	2^2	1	0	1	0

(10x**1**, 2x**2**, 2x$\mathbf{2^2}$) **g**

	1	2	3	4	5
1	2	1	0	0	1
2	1	0	2^2	0	1
3	0	2^2	0	1	0
4	0	0	1	2	1
5	1	1	0	1	0

(10x**1**, 2x**2**, 2x$\mathbf{2^2}$) **h**

	1	2	3	4	5
1	0	1	0	0	2^2
2	1	0	1	1	1
3	0	1	2	1	0
4	0	1	1	2	0
5	2^2	1	0	0	0

(10x**1**, 4x**2**^{1}) **a**

	1	2	3	4	5
1	0	2^1	0	0	2^1
2	2^1	0	1	1	0
3	0	1	0	1	1
4	0	1	1	0	1
5	2^1	0	1	1	0

(10x**1**, 4x**2**^{1}) **b**

	1	2	3	4	5
1	0	1	0	0	2^1
2	1	0	2^1	1	0
3	0	2^1	0	1	1
4	0	1	1	0	1
5	2^1	0	1	1	0

(10x**1**, 4x**2**^{1}) **c**

	1	2	3	4	5
1	0	1	0	0	2^1
2	1	0	1	1	0
3	0	1	0	2^1	1
4	0	1	2^1	0	1
5	2^1	0	1	1	0

(10x**1**, 4x**2**^{1}) **d**

	1	2	3	4	5
1	0	0	1	2^1	0
2	0	0	1	1	2^1
3	1	1	0	1	1
4	2^1	1	1	0	0
5	0	2^1	1	0	0

(10x**1**, 2x**2**^{1}, 2x**2**^{2}) **a**

	1	2	3	4	5
1	0	2^2	0	0	2^1
2	2^2	0	1	1	0
3	0	1	0	1	1
4	0	1	1	0	1
5	2^1	0	1	1	0

(10x**1**, 2x**2**^{1}, 2x**2**^{2}) **b**

	1	2	3	4	5
1	0	1	0	0	2^2
2	1	0	2^1	1	0
3	0	2^1	0	1	1
4	0	1	1	0	1
5	2^2	0	1	1	0

(10x**1**, 2x**2**^{1}, 2x**2**^{2}) **c**

	1	2	3	4	5
1	0	1	0	0	2^1
2	1	0	2^2	1	0
3	0	2^2	0	1	1
4	0	1	1	0	1
5	2^1	0	1	1	0

(10x**1**, 2x**2**^{1}, 2x**2**^{2}) **d**

	1	2	3	4	5
1	0	1	0	0	2^2
2	1	0	1	1	0
3	0	1	0	2^1	1
4	0	1	2^1	0	1
5	2^2	0	1	1	0

(10x**1**, 2x**2**^{1}, 2x**2**^{2}) **e**

	1	2	3	4	5
1	0	1	0	0	2^1
2	1	0	1	1	0
3	0	1	0	2^2	1
4	0	1	2^2	0	1
5	2^1	0	1	1	0

(10x**1**, 2x**2**^{1}, 2x**2**^{2}) **f**

	1	2	3	4	5
1	0	0	1	2^1	0
2	0	0	1	1	2^2
3	1	1	0	1	1
4	2^1	1	1	0	0
5	0	2^2	1	0	0

(10x**1**, 4x**2**^{2}) **a**

	1	2	3	4	5
1	0	2^2	0	0	2^2
2	2^2	0	1	1	0
3	0	1	0	1	1
4	0	1	1	0	1
5	2^2	0	1	1	0

(10x**1**, 4x**2**^{2}) **b**

	1	2	3	4	5
1	0	1	0	0	2^2
2	1	0	2^2	1	0
3	0	2^2	0	1	1
4	0	1	1	0	1
5	2^2	0	1	1	0

(10x**1**, 4x**2**^{2}) **c**

	1	2	3	4	5
1	0	1	0	0	2^2
2	1	0	1	1	0
3	0	1	0	2^2	1
4	0	1	2^2	0	1
5	2^2	0	1	1	0

(10x**1**, 4x**2**^{2}) **d**

	1	2	3	4	5
1	0	0	1	2^2	0
2	0	0	1	1	2^2
3	1	1	0	1	1
4	2^2	1	1	0	0
5	0	2^2	1	0	0

(8x**1**, 5x**2**) **a**

	1	2	3	4	5
1	2	0	0	0	1
2	0	2	0	1	0
3	0	0	2	1	1
4	0	1	1	2	0
5	1	0	1	0	2

(8x**1**, 5x**2**) **b**

	1	2	3	4	5
1	2	0	1	0	1
2	0	2	0	1	0
3	1	0	2	0	1
4	0	1	0	2	0
5	1	0	1	0	2

(8x**1**, 3x**2**, 2x**2**^{1}) **a**

	1	2	3	4	5
1	2	0	0	1	1
2	0	2	0	1	0
3	0	0	0	2^1	1
4	1	1	2^1	0	0
5	1	0	1	0	2

(8x**1**, 3x**2**, 2x**2**^{1}) **b**

	1	2	3	4	5
1	2	0	1	1	0
2	0	2	0	1	0
3	1	0	0	2^1	1
4	1	1	2^1	0	0
5	0	0	1	0	2

(8x**1**, 3x**2**, 2x**2**^{1}) **c**

	1	2	3	4	5
1	2	0	1	1	0
2	0	2	0	2^1	0
3	1	0	0	1	1
4	1	2^1	1	0	0
5	0	0	1	0	2

(8x**1**, 3x**2**, 2x**2**^{1}) **d**

	1	2	3	4	5
1	2	0	1	1	0
2	0	0	0	1	2^1
3	1	0	2	1	0
4	1	1	1	0	0
5	0	2^1	0	0	2

(8x**1**, 3x**2**, 2x**2**^{1}) **e**

	1	2	3	4	5
1	2	0	1	1	0
2	0	0	0	2^1	1
3	1	0	2	1	0
4	1	2^1	1	0	0
5	0	1	0	0	2

(8x**1**, 3x**2**, 2x**2**^{1}) **f**

	1	2	3	4	5
1	0	0	1	2^1	0
2	0	2	0	1	1
3	1	0	2	1	0
4	2^1	1	1	0	0
5	0	1	0	0	2

(8x**1**, 3x**2**, 2x**2**^{1}) **g**

	1	2	3	4	5
1	2	1	0	0	1
2	1	2	1	0	0
3	0	1	0	2^1	0
4	0	0	2^1	0	1
5	1	0	0	1	2

(8x**1**, 3x**2**, 2x**2**^{2}) **a**

	1	2	3	4	5
1	2	0	0	1	1
2	0	2	0	1	0
3	0	0	0	2^2	1
4	1	1	2^2	0	0
5	1	0	1	0	2

(8x**1**, 3x**2**, 2x**2**^{2}) **b**

	1	2	3	4	5
1	2	0	1	1	0
2	0	2	0	1	0
3	1	0	0	2^2	1
4	1	1	2^2	0	0
5	0	0	1	0	2

(8x**1**, 3x**2**, 2x**2**^{2}) **c**

	1	2	3	4	5
1	2	0	1	1	0
2	0	2	0	2^2	0
3	1	0	0	1	1
4	1	2^2	1	0	0
5	0	0	1	0	2

(8x**1**, 3x**2**, 2x**2**^{2}) **d**

	1	2	3	4	5
1	2	0	1	1	0
2	0	0	0	1	2^2
3	1	0	2	1	0
4	1	1	1	0	0
5	0	2^2	0	0	2

(8x**1**, 3x**2**, 2x**2**^{2}) **e**

	1	2	3	4	5
1	2	0	1	1	0
2	0	0	0	2^2	1
3	1	0	2	1	0
4	1	2^2	1	0	0
5	0	1	0	0	2

(8x**1**, 3x**2**, 2x**2**^{2}) **f**

	1	2	3	4	5
1	0	0	1	2^2	0
2	0	2	0	1	1
3	1	0	2	1	0
4	2^2	1	1	0	0
5	0	1	0	0	2

(8x**1**, 3x**2**, 2x**2**^{2}) **g**

	1	2	3	4	5
1	2	1	0	0	1
2	1	2	1	0	0
3	0	1	0	2^2	0
4	0	0	2^2	0	1
5	1	0	0	1	2

(8x**1**, 1x**2**, 4x**2**^{1}) **a**

	1	2	3	4	5
1	0	0	1	1	2^1
2	0	2	0	1	0
3	1	0	0	1	2^1
4	1	1	1	0	0
5	2^1	0	2^1	0	0

(8x**1**, 1x**2**, 4x**2**^{1}) **b**

	1	2	3	4	5
1	0	0	1	1	2^1
2	0	2	0	2^1	0
3	1	0	0	1	1
4	1	2^1	1	0	0
5	2^1	0	1	0	0

(8x**1**, 1x**2**, 4x**2**^{1}) **c**

	1	2	3	4	5
1	0	0	1	2^1	1
2	0	2	0	1	0
3	1	0	0	1	2^1
4	2^1	1	1	0	0
5	1	0	2^1	0	0

(8x**1**, 1x**2**, 4x**2**^{1}) **d**

	1	2	3	4	5
1	2	1	0	0	1
2	1	0	1	0	2^1
3	0	1	0	2^1	0
4	0	0	2^1	0	1
5	1	2^1	0	1	0

(8x**1**, 1x**2**, 4x**2**^{1}) **e**

	1	2	3	4	5
1	2	1	0	0	1
2	1	0	2^1	0	1
3	0	2^1	0	2^1	0
4	0	0	2^1	0	1
5	1	1	0	1	0

(8x**1**, 1x**2**, 4x**2**^{1}) **f**

	1	2	3	4	5
1	2	1	0	0	1
2	1	0	2^1	0	1
3	0	2^1	0	1	0
4	0	0	1	0	2^1
5	1	1	0	2^1	0

(8x**1**, 1x**2**, 4x**2**^{1}) **g**

	1	2	3	4	5
1	0	2^1	0	0	1
2	2^1	0	1	0	1
3	0	1	2	1	0
4	0	0	1	0	2^1
5	1	1	0	2^1	0

(8x**1**, 1x**2**, 4x**2**^{1}) **h**

	1	2	3	4	5
1	0	1	2^1	0	0
2	1	0	0	1	2^1
3	2^1	0	0	1	1
4	0	1	1	2	0
5	0	2^1	1	0	0

(8x**1**, 1x**2**, 2x**2**^{1}, 2x**2**^{2}) **a**

	1	2	3	4	5
1	0	0	1	1	2^1
2	0	2	0	1	0
3	1	0	0	1	2^2
4	1	1	1	0	0
5	2^1	0	2^2	0	0

(8x**1**, 1x**2**, 2x**2**^{1}, 2x**2**^{2}) **b**

	1	2	3	4	5
1	0	0	1	1	2^2
2	0	2	0	2^1	0
3	1	0	0	1	1
4	1	2^1	1	0	0
5	2^2	0	1	0	0

(8x**1**, 1x**2**, 2x**2**^{1}, 2x**2**^{2}) **c**

	1	2	3	4	5
1	0	0	1	1	2^1
2	0	2	0	2^2	0
3	1	0	0	1	1
4	1	2^2	1	0	0
5	2^1	0	1	0	0

(8x**1**, 1x**2**, 2x**2**^{1}, 2x**2**^{2}) **d**

	1	2	3	4	5
1	0	0	1	2^1	1
2	0	2	0	1	0
3	1	0	0	1	2^2
4	2^1	1	1	0	0
5	1	0	2^2	0	0

(8x**1**, 1x**2**, 2x**2**^{1}, 2x**2**^{2}) **e**

	1	2	3	4	5
1	0	0	1	2^2	1
2	0	2	0	1	0
3	1	0	0	1	2^1
4	2^2	1	1	0	0
5	1	0	2^1	0	0

(8x**1**, 1x**2**, 2x**2**^{1}, 2x**2**^{2}) **f**

	1	2	3	4	5
1	2	1	0	0	1
2	1	0	1	0	2^1
3	0	1	0	2^2	0
4	0	0	2^2	0	1
5	1	2^1	0	1	0

(8x**1**, 1x**2**, 2x**2**^{1}, 2x**2**^{2}) **g**

	1	2	3	4	5
1	2	1	0	0	1
2	1	0	1	0	2^2
3	0	1	0	2^1	0
4	0	0	2^1	0	1
5	1	2^2	0	1	0

(8x**1**, 1x**2**, 2x**2**^{1}, 2x**2**^{2}) **h**

	1	2	3	4	5
1	2	1	0	0	1
2	1	0	2^1	0	1
3	0	2^1	0	2^2	0
4	0	0	2^2	0	1
5	1	1	0	1	0

(8x**1**, 1x**2**, 2x**2**^{1}, 2x**2**^{2}) **i**

	1	2	3	4	5
1	2	1	0	0	1
2	1	0	2^2	0	1
3	0	2^2	0	2^1	0
4	0	0	2^1	0	1
5	1	1	0	1	0

(8x**1**, 1x**2**, 2x**2**^{1}, 2x**2**^{2}) **j**

	1	2	3	4	5
1	2	1	0	0	1
2	1	0	2^1	0	1
3	0	2^1	0	1	0
4	0	0	1	0	2^2
5	1	1	0	2^2	0

(8x**1**, 1x**2**, 2x**2**^{1}, 2x**2**^{2}) **k**

	1	2	3	4	5
1	0	2^1	0	0	1
2	2^1	0	1	0	1
3	0	1	2	1	0
4	0	0	1	0	2^2
5	1	1	0	2^2	0

(8x**1**, 1x**2**, 2x**2**^{1}, 2x**2**^{2}) **l**

	1	2	3	4	5
1	0	2^2	0	0	1
2	2^2	0	1	0	1
3	0	1	2	1	0
4	0	0	1	0	2^1
5	1	1	0	2^1	0

(8x**1**, 1x**2**, 2x**2**^{1}, 2x**2**^{2}) **m**

	1	2	3	4	5
1	0	1	2^2	0	0
2	1	0	0	1	2^1
3	2^2	0	0	1	1
4	0	1	1	2	0
5	0	2^1	1	0	0

(8x**1**, 1x**2**, 2x**2**^{1}, 2x**2**^{2}) **n**

	1	2	3	4	5
1	0	1	2^1	0	0
2	1	0	0	1	2^2
3	2^1	0	0	1	1
4	0	1	1	2	0
5	0	2^2	1	0	0

(8x**1**, 1x**2**, 4x**2**^{2}) **a**

	1	2	3	4	5
1	0	0	1	1	2^2
2	0	2	0	1	0
3	1	0	0	1	2^2
4	1	1	1	0	0
5	2^2	0	2^2	0	0

(8x**1**, 1x**2**, 4x**2**^{1}) **b**

	1	2	3	4	5
1	0	0	1	1	2^2
2	0	2	0	2^2	0
3	1	0	0	1	1
4	1	2^2	1	0	0
5	2^2	0	1	0	0

(8x**1**, 1x**2**, 4x**2**^{1}) **c**

	1	2	3	4	5
1	0	0	1	2^2	1
2	0	2	0	1	0
3	1	0	0	1	2^2
4	2^2	1	1	0	0
5	1	0	2^2	0	0

(8x**1**, 1x**2**, 4x**2**^{2}) **d**

	1	2	3	4	5
1	2	1	0	0	1
2	1	0	1	0	2^2
3	0	1	0	2^2	0
4	0	0	2^2	0	1
5	1	2^2	0	1	0

(8x**1**, 1x**2**, 4x**2**^{2}) **e**

	1	2	3	4	5
1	2	1	0	0	1
2	1	0	2^2	0	1
3	0	2^2	0	2^2	0
4	0	0	2^2	0	1
5	1	1	0	1	0

(8x**1**, 1x**2**, 4x**2**^{2}) **f**

	1	2	3	4	5
1	2	1	0	0	1
2	1	0	2^2	0	1
3	0	2^2	0	1	0
4	0	0	1	0	2^2
5	1	1	0	2^2	0

(8x**1**, 1x**2**, 4x**2**^{2}) **g**

	1	2	3	4	5
1	0	2^2	0	0	1
2	2^2	0	1	0	1
3	0	1	2	1	0
4	0	0	1	0	2^2
5	1	1	0	2^2	0

(8x**1**, 1x**2**, 4x**2**^{2}) **h**

	1	2	3	4	5
1	0	1	2^2	0	0
2	1	0	0	1	2^2
3	2^2	0	0	1	1
4	0	1	1	2	0
5	0	2^2	1	0	0

(6x**1**, 4x**2**, 2x**2**^{1}) **a**

	1	2	3	4	5
1	2	0	0	0	1
2	0	2	0	2^1	0
3	0	0	2	1	1
4	0	2^1	1	0	0
5	1	0	1	0	2

(6x**1**, 4x**2**, 2x**2**^{1}) **b**

	1	2	3	4	5
1	2	0	1	0	0
2	0	2	0	0	1
3	1	0	0	2^1	1
4	0	0	2^1	2	0
5	0	1	1	0	2

(6x**1**, 4x**2**, 2x**2**^{2}) **a**

	1	2	3	4	5
1	2	0	0	0	1
2	0	2	0	2^2	0
3	0	0	2	1	1
4	0	2^2	1	0	0
5	1	0	1	0	2

(6x**1**, 4x**2**, 2x**2**^{2}) **b**

	1	2	3	4	5
1	2	0	1	0	0
2	0	2	0	0	1
3	1	0	0	2^2	1
4	0	0	2^2	2	0
5	0	1	1	0	2

(6x**1**, 2x**2**, 4x**2**^{1}) **a**

	1	2	3	4	5
1	0	0	2^1	2^1	0
2	0	2	0	1	0
3	2^1	0	0	1	1
4	2^1	1	1	0	0
5	0	0	1	0	2

(6x**1**, 2x**2**, 4x**2**^{1}) **b**

	1	2	3	4	5
1	0	0	2^1	1	0
2	0	2	0	2^1	0
3	2^1	0	0	1	1
4	1	2^1	1	0	0
5	0	0	1	0	2

(6x**1**, 2x**2**, 4x**2**^{1}) **c**

	1	2	3	4	5
1	0	0	0	1	2^1
2	0	2	1	0	0
3	0	1	0	1	2^1
4	1	0	1	2	0
5	2^1	0	2^1	0	0

(6x**1**, 2x**2**, 4x**2**^{1}) **d**

	1	2	3	4	5
1	0	0	0	1	2^1
2	0	2	2^1	0	0
3	0	2^1	0	1	1
4	1	0	1	2	0
5	2^1	0	1	0	0

(6x**1**, 2x**2**, 4x**2**^{1}) **e**

	1	2	3	4	5
1	0	0	1	0	2^1
2	0	2	1	1	0
3	1	1	0	0	2^1
4	0	1	0	2	0
5	2^1	0	2^1	0	0

(6x**1**, 2x**2**, 4x**2**^{1}) **f**

	1	2	3	4	5
1	2	0	1	0	1
2	0	0	1	2^1	0
3	1	1	0	0	2^1
4	0	2^1	0	2	0
5	1	0	2^1	0	0

(6x**1**, 2x**2**, 4x$\mathbf{2}^1$) **g**

	1	2	3	4	5
1	2	1	0	0	1
2	1	2	1	0	0
3	0	1	0	2^1	0
4	0	0	2^1	0	2^1
5	1	0	0	2^1	0

(6x**1**, 2x**2**, 2x$\mathbf{2}^1$, 2x$\mathbf{2}^2$) **a**

	1	2	3	4	5
1	0	0	2^2	2^1	0
2	0	2	0	1	0
3	2^2	0	0	1	1
4	2^1	1	1	0	0
5	0	0	1	0	2

(6x**1**, 2x**2**, 2x$\mathbf{2}^1$, 2x$\mathbf{2}^2$) **b**

	1	2	3	4	5
1	0	0	2^2	1	0
2	0	2	0	2^1	0
3	2^2	0	0	1	1
4	1	2^1	1	0	0
5	0	0	1	0	2

(6x**1**, 2x**2**, 2x$\mathbf{2}^1$, 2x$\mathbf{2}^2$) **c**

	1	2	3	4	5
1	0	0	2^1	1	0
2	0	2	0	2^2	0
3	2^1	0	0	1	1
4	1	2^2	1	0	0
5	0	0	1	0	2

(6x**1**, 2x**2**, 2x$\mathbf{2}^1$, 2x$\mathbf{2}^2$) **d**

	1	2	3	4	5
1	0	0	0	1	2^2
2	0	2	1	0	0
3	0	1	0	1	2^1
4	1	0	1	2	0
5	2^2	0	2^1	0	0

(6x**1**, 2x**2**, 2x$\mathbf{2}^1$, 2x$\mathbf{2}^2$) **e**

	1	2	3	4	5
1	0	0	0	1	2^1
2	0	2	1	0	0
3	0	1	0	1	2^2
4	1	0	1	2	0
5	2^1	0	2^2	0	0

(6x**1**, 2x**2**, 2x$\mathbf{2}^1$, 2x$\mathbf{2}^2$) **f**

	1	2	3	4	5
1	0	0	0	1	2^2
2	0	2	2^1	0	0
3	0	2^1	0	1	1
4	1	0	1	2	0
5	2^2	0	1	0	0

(6x**1**, 2x**2**, 2x$\mathbf{2}^1$, 2x$\mathbf{2}^2$) **g**

	1	2	3	4	5
1	0	0	0	1	2^1
2	0	2	2^2	0	0
3	0	2^2	0	1	1
4	1	0	1	2	0
5	2^1	0	1	0	0

(6x**1**, 2x**2**, 2x$\mathbf{2}^1$, 2x$\mathbf{2}^2$) **h**

	1	2	3	4	5
1	0	0	1	0	2^2
2	0	2	1	1	0
3	1	1	0	0	2^1
4	0	1	0	2	0
5	2^2	0	2^1	0	0

(6x**1**, 2x**2**, 2x$\mathbf{2}^1$, 2x$\mathbf{2}^2$) **i**

	1	2	3	4	5
1	0	0	1	0	2^1
2	0	2	1	1	0
3	1	1	0	0	2^2
4	0	1	0	2	0
5	2^1	0	2^2	0	0

(6x**1**, 2x**2**, 2x$\mathbf{2}^1$, 2x$\mathbf{2}^2$) **j**

	1	2	3	4	5
1	2	0	1	0	1
2	0	0	1	2^1	0
3	1	1	0	0	2^2
4	0	2^1	0	2	0
5	1	0	2^2	0	0

(6x**1**, 2x**2**, 2x$\mathbf{2}^1$, 2x$\mathbf{2}^2$) **k**

	1	2	3	4	5
1	2	0	1	0	1
2	0	0	1	2^2	0
3	1	1	0	0	2^1
4	0	2^2	0	2	0
5	1	0	2^1	0	0

(6x**1**, 2x**2**, 2x$\mathbf{2}^1$, 2x$\mathbf{2}^2$) **l**

	1	2	3	4	5
1	2	1	0	0	1
2	1	2	1	0	0
3	0	1	0	2^1	0
4	0	0	2^1	0	2^2
5	1	0	0	2^2	0

(6x**1**, 2x**2**, 4x$\mathbf{2}^2$) **a**

	1	2	3	4	5
1	0	0	2^2	2^2	0
2	0	2	0	1	0
3	2^2	0	0	1	1
4	2^2	1	1	0	0
5	0	0	1	0	2

(6x**1**, 2x**2**, 4x$\mathbf{2}^2$) **b**

	1	2	3	4	5
1	0	0	2^2	1	0
2	0	2	0	2^2	0
3	2^2	0	0	1	1
4	1	2^2	1	0	0
5	0	0	1	0	2

(6x**1**, 2x**2**, 4x$\mathbf{2}^2$) **c**

	1	2	3	4	5
1	0	0	0	1	2^2
2	0	2	1	0	0
3	0	1	0	1	2^2
4	1	0	1	2	0
5	2^2	0	2^2	0	0

(6x**1**, 2x**2**, 4x$\mathbf{2}^2$) **d**

	1	2	3	4	5
1	0	0	0	1	2^2
2	0	2	2^2	0	0
3	0	2^2	0	1	1
4	1	0	1	2	0
5	2^2	0	1	0	0

(6x**1**, 2x**2**, 4x$\mathbf{2}^2$) **e**

	1	2	3	4	5
1	0	0	1	0	2^2
2	0	2	1	1	0
3	1	1	0	0	2^2
4	0	1	0	2	0
5	2^2	0	2^2	0	0

(6x**1**, 2x**2**, 4x$\mathbf{2}^2$) **f**

	1	2	3	4	5
1	2	0	1	0	1
2	0	0	1	2^2	0
3	1	1	0	0	2^2
4	0	2^2	0	2	0
5	1	0	2^2	0	0

(6x**1**, 2x**2**, 4x$\mathbf{2}^2$) **g**

	1	2	3	4	5
1	2	1	0	0	1
2	1	2	1	0	0
3	0	1	0	2^2	0
4	0	0	2^2	0	2^2
5	1	0	0	2^2	0

(6x**1**, 6x$\mathbf{2}^1$) **a**

	1	2	3	4	5
1	0	2^1	0	0	2^1
2	2^1	0	1	0	1
3	0	1	0	2^1	0
4	0	0	2^1	0	1
5	2^1	1	0	1	0

(6x**1**, 6x$\mathbf{2}^1$) **b**

	1	2	3	4	5
1	0	1	0	0	2^1
2	1	0	2^1	0	1
3	0	2^1	0	2^1	0
4	0	0	2^1	0	1
5	2^1	1	0	1	0

(6x**1**, 4x$\mathbf{2}^1$, 2x$\mathbf{2}^2$) **a**

	1	2	3	4	5
1	0	2^1	0	0	2^1
2	2^1	0	1	0	1
3	0	1	0	2^2	0
4	0	0	2^2	0	1
5	2^1	1	0	1	0

(6x**1**, 4x$\mathbf{2}^1$, 2x$\mathbf{2}^2$) **b**

	1	2	3	4	5
1	0	2^1	0	0	2^2
2	2^1	0	1	0	1
3	0	1	0	2^1	0
4	0	0	2^1	0	1
5	2^2	1	0	1	0

(6x**1**, 4x$\mathbf{2}^1$, 2x$\mathbf{2}^2$) **c**

	1	2	3	4	5
1	0	1	0	0	2^1
2	1	0	2^1	0	1
3	0	2^1	0	2^2	0
4	0	0	2^2	0	1
5	2^1	1	0	1	0

(6x**1**, 4x$\mathbf{2}^1$, 2x$\mathbf{2}^2$) **d**

	1	2	3	4	5
1	0	1	0	0	2^1
2	1	0	2^2	0	1
3	0	2^2	0	2^1	0
4	0	0	2^1	0	1
5	2^1	1	0	1	0

(6x**1**, 4x$\mathbf{2}^1$, 2x$\mathbf{2}^2$) **e**

	1	2	3	4	5
1	0	1	0	0	2^2
2	1	0	2^1	0	1
3	0	2^1	0	2^1	0
4	0	0	2^1	0	1
5	2^2	1	0	1	0

(6x**1**, 2x$\mathbf{2}^1$, 4x$\mathbf{2}^2$) **a**

	1	2	3	4	5
1	0	2^1	0	0	2^2
2	2^1	0	1	0	1
3	0	1	0	2^2	0
4	0	0	2^2	0	1
5	2^2	1	0	1	0

(6x**1**, 2x$\mathbf{2}^1$, 4x$\mathbf{2}^2$) **b**

	1	2	3	4	5
1	0	2^2	0	0	2^2
2	2^2	0	1	0	1
3	0	1	0	2^1	0
4	0	0	2^1	0	1
5	2^2	1	0	1	0

(6x**1**, 2x$\mathbf{2}^1$, 4x$\mathbf{2}^2$) **c**

	1	2	3	4	5
1	0	1	0	0	2^1
2	1	0	2^2	0	1
3	0	2^2	0	2^2	0
4	0	0	2^2	0	1
5	2^1	1	0	1	0

(6x**1**, 2x$\mathbf{2}^1$, 4x$\mathbf{2}^2$) **d**

	1	2	3	4	5
1	0	1	0	0	2^2
2	1	0	2^1	0	1
3	0	2^1	0	2^2	0
4	0	0	2^2	0	1
5	2^2	1	0	1	0

(6x**1**, 2x$\mathbf{2}^1$, 4x$\mathbf{2}^2$) **e**

	1	2	3	4	5
1	0	1	0	0	2^2
2	1	0	2^2	0	1
3	0	2^2	0	2^1	0
4	0	0	2^1	0	1
5	2^2	1	0	1	0

(6x**1**, 6x$\mathbf{2}^2$) **a**

	1	2	3	4	5
1	0	2^2	0	0	2^2
2	2^2	0	1	0	1
3	0	1	0	2^2	0
4	0	0	2^2	0	1
5	2^2	1	0	1	0

(6x**1**, 6x$\mathbf{2}^2$) **b**

	1	2	3	4	5
1	0	1	0	0	2^2
2	1	0	2^2	0	1
3	0	2^2	0	2^2	0
4	0	0	2^2	0	1
5	2^2	1	0	1	0

(4x**1**, 5x**2**, 2x$\mathbf{2}^1$)

	1	2	3	4	5
1	2	0	0	0	1
2	0	2	0	2^1	0
3	0	0	2	0	1
4	0	2^1	0	2	0
5	1	0	1	0	2

(4x**1**, 5x**2**, 2x$\mathbf{2}^2$)

	1	2	3	4	5
1	2	0	0	0	1
2	0	2	0	2^2	0
3	0	0	2	0	1
4	0	2^2	0	2	0
5	1	0	1	0	2

(4x**1**, 3x**2**, 4x$\mathbf{2}^1$) **a**

	1	2	3	4	5
1	2	0	0	0	2^1
2	0	2	0	2^1	0
3	0	0	2	1	1
4	0	2^1	1	0	0
5	2^1	0	1	0	0

(4x**1**, 3x**2**, 4x$\mathbf{2}^1$) **b**

	1	2	3	4	5
1	2	0	0	0	2^1
2	0	2	0	1	0
3	0	0	0	1	2^1
4	0	1	1	2	0
5	2^1	0	2^1	0	0

(4x**1**, 3x**2**, 4x$\mathbf{2}^1$) **c**

	1	2	3	4	5
1	2	0	0	0	2^1
2	0	2	1	0	0
3	0	1	0	1	2^1
4	0	0	1	2	0
5	2^1	0	2^1	0	0

(4x**1**, 3x**2**, 4x$\mathbf{2}^1$) **d**

	1	2	3	4	5
1	2	0	0	0	2^1
2	0	2	1	0	0
3	0	1	0	2^1	1
4	0	0	2^1	2	0
5	2^1	0	1	0	0

(4x**1**, 3x**2**, 4x$\mathbf{2}^1$) **e**

	1	2	3	4	5
1	0	0	1	0	2^1
2	0	2	0	2^1	0
3	1	0	2	0	1
4	0	2^1	0	2	0
5	2^1	0	1	0	0

(4x**1**, 3x**2**, 2x$\mathbf{2}^1$, 2x$\mathbf{2}^2$) **a**

	1	2	3	4	5
1	2	0	0	0	2^2
2	0	2	0	2^1	0
3	0	0	2	1	1
4	0	2^1	1	0	0
5	2^2	0	1	0	0

(4x**1**, 3x**2**, 2x$\mathbf{2}^1$, 2x$\mathbf{2}^2$) **b**

	1	2	3	4	5
1	2	0	0	0	2^2
2	0	2	0	1	0
3	0	0	0	1	2^1
4	0	1	1	2	0
5	2^2	0	2^1	0	0

(4x**1**, 3x**2**, 2x$\mathbf{2}^1$, 2x$\mathbf{2}^2$) **c**

	1	2	3	4	5
1	2	0	0	0	2^1
2	0	2	0	1	0
3	0	0	0	1	2^2
4	0	1	1	2	0
5	2^1	0	2^2	0	0

(4x**1**, 3x**2**, 2x$\mathbf{2}^1$, 2x$\mathbf{2}^2$) **d**

	1	2	3	4	5
1	2	0	0	0	2^1
2	0	2	1	0	0
3	0	1	0	1	2^2
4	0	0	1	2	0
5	2^1	0	2^2	0	0

(4x**1**, 3x**2**, 2x$\mathbf{2}^1$, 2x$\mathbf{2}^2$) **e**

	1	2	3	4	5
1	2	0	0	0	2^2
2	0	2	1	0	0
3	0	1	0	1	2^1
4	0	0	1	2	0
5	2^2	0	2^1	0	0

(4x**1**, 3x**2**, 2x$\mathbf{2}^1$, 2x$\mathbf{2}^2$) **f**

	1	2	3	4	5
1	2	0	0	0	2^2
2	0	2	1	0	0
3	0	1	0	2^1	1
4	0	0	2^1	2	0
5	2^2	0	1	0	0

(4x**1**, 3x**2**, 2x$\mathbf{2}^1$, 2x$\mathbf{2}^2$) **g**

	1	2	3	4	5
1	2	0	0	0	2^1
2	0	2	1	0	0
3	0	1	0	2^2	1
4	0	0	2^2	2	0
5	2^1	0	1	0	0

(4x**1**, 3x**2**, 2x$\mathbf{2}^1$, 2x$\mathbf{2}^2$) **h**

	1	2	3	4	5
1	0	0	1	0	2^2
2	0	2	0	2^1	0
3	1	0	2	0	1
4	0	2^1	0	2	0
5	2^2	0	1	0	0

(4x**1**, 3x**2**, 2x$\mathbf{2}^1$, 2x$\mathbf{2}^2$) **i**

	1	2	3	4	5
1	0	0	1	0	2^1
2	0	2	0	2^2	0
3	1	0	2	0	1
4	0	2^2	0	2	0
5	2^1	0	1	0	0

(4x**1**, 3x**2**, 4x$\mathbf{2}^2$) **a**

	1	2	3	4	5
1	2	0	0	0	2^2
2	0	2	0	2^2	0
3	0	0	2	1	1
4	0	2^2	1	0	0
5	2^2	0	1	0	0

(4x**1**, 3x**2**, 4x$\mathbf{2}^2$) **b**

	1	2	3	4	5
1	2	0	0	0	2^2
2	0	2	0	1	0
3	0	0	0	1	2^2
4	0	1	1	2	0
5	2^2	0	2^2	0	0

(4x**1**, 3x**2**, 4x$\mathbf{2}^2$) **c**

	1	2	3	4	5
1	2	0	0	0	2^2
2	0	2	1	0	0
3	0	1	0	1	2^2
4	0	0	1	2	0
5	2^2	0	2^2	0	0

(4x**1**, 3x**2**, 4x$\mathbf{2}^2$) **d**

	1	2	3	4	5
1	2	0	0	0	2^2
2	0	2	1	0	0
3	0	1	0	2^2	1
4	0	0	2^2	2	0
5	2^2	0	1	0	0

(4x**1**, 3x**2**, 4x$\mathbf{2}^2$) **e**

	1	2	3	4	5
1	0	0	1	0	2^2
2	0	2	0	2^2	0
3	1	0	2	0	1
4	0	2^2	0	2	0
5	2^2	0	1	0	0

(4x**1**, 1x**2**, 6x$\mathbf{2}^1$) **a**

	1	2	3	4	5
1	2	1	0	0	1
2	1	0	2^1	0	0
3	0	2^1	0	2^1	0
4	0	0	2^1	0	2^1
5	1	0	0	2^1	0

(4x**1**, 1x**2**, 6x$\mathbf{2}^1$) **b**

	1	2	3	4	5
1	0	0	0	2^1	2^1
2	0	2	0	1	0
3	0	0	0	1	2^1
4	2^1	1	1	0	0
5	2^1	0	2^1	0	0

(4x**1**, 1x**2**, 6x$\mathbf{2}^1$) **c**

	1	2	3	4	5
1	0	0	0	1	2^1
2	0	2	0	2^1	0
3	0	0	0	1	2^1
4	1	2^1	1	0	0
5	2^1	0	2^1	0	0

(4x**1**, 1x**2**, 6x$\mathbf{2}^1$) **d**

	1	2	3	4	5
1	0	0	1	0	2^1
2	0	0	1	2^1	0
3	1	1	0	0	2^1
4	0	2^1	0	2	0
5	2^1	0	2^1	0	0

(4x**1**, 1x**2**, 6x$\mathbf{2}^1$) **e**

	1	2	3	4	5
1	0	0	1	0	2^1
2	0	0	2^1	2^1	0
3	1	2^1	0	0	1
4	0	2^1	0	2	0
5	2^1	0	1	0	0

(4x**1**, 1x**2**, 4x$\mathbf{2}^1$, 2x$\mathbf{2}^2$) **a**

	1	2	3	4	5
1	2	1	0	0	1
2	1	0	2^1	0	0
3	0	2^1	0	2^2	0
4	0	0	2^2	0	2^1
5	1	0	0	2^1	0

(4x**1**, 1x**2**, 4x$\mathbf{2}^1$, 2x$\mathbf{2}^2$) **b**

	1	2	3	4	5
1	2	1	0	0	1
2	1	0	2^2	0	0
3	0	2^2	0	2^1	0
4	0	0	2^1	0	2^1
5	1	0	0	2^1	0

(4x**1**, 1x**2**, 4x$\mathbf{2}^1$, 2x$\mathbf{2}^2$) **c**

	1	2	3	4	5
1	0	0	0	2^1	2^2
2	0	2	0	1	0
3	0	0	0	1	2^1
4	2^1	1	1	0	0
5	2^2	0	2^1	0	0

(4x**1**, 1x**2**, 4x$\mathbf{2}^1$, 2x$\mathbf{2}^2$) **d**

	1	2	3	4	5
1	0	0	0	2^2	2^1
2	0	2	0	1	0
3	0	0	0	1	2^1
4	2^2	1	1	0	0
5	2^1	0	2^1	0	0

(4x**1**, 1x**2**, 4x$\mathbf{2}^1$, 2x$\mathbf{2}^2$) **e**

	1	2	3	4	5
1	0	0	0	2^1	2^1
2	0	2	0	1	0
3	0	0	0	1	2^2
4	2^1	1	1	0	0
5	2^1	0	2^2	0	0

(4x**1**, 1x**2**, 4x$\mathbf{2}^1$, 2x$\mathbf{2}^2$) **f**

	1	2	3	4	5
1	0	0	0	1	2^1
2	0	2	0	2^1	0
3	0	0	0	1	2^2
4	1	2^1	1	0	0
5	2^1	0	2^2	0	0

(4x**1**, 1x**2**, 4x$\mathbf{2}^1$, 2x$\mathbf{2}^2$) **g**

	1	2	3	4	5
1	0	0	0	1	2^1
2	0	2	0	2^2	0
3	0	0	0	1	2^1
4	1	2^2	1	0	0
5	2^1	0	2^1	0	0

(4x**1**, 1x**2**, 4x$\mathbf{2}^1$, 2x$\mathbf{2}^2$) **h**

	1	2	3	4	5
1	0	0	1	0	2^2
2	0	0	1	2^1	0
3	1	1	0	0	2^1
4	0	2^1	0	2	0
5	2^2	0	2^1	0	0

(4x**1**, 1x**2**, 4x$\mathbf{2}^1$, 2x$\mathbf{2}^2$) **i**

	1	2	3	4	5
1	0	0	1	0	2^1
2	0	0	1	2^1	0
3	1	1	0	0	2^2
4	0	2^1	0	2	0
5	2^1	0	2^2	0	0

(4x**1**, 1x**2**, 4x$\mathbf{2}^1$, 2x$\mathbf{2}^2$) **j**

	1	2	3	4	5
1	0	0	1	0	2^1
2	0	0	1	2^2	0
3	1	1	0	0	2^1
4	0	2^2	0	2	0
5	2^1	0	2^1	0	0

(4x**1**, 1x**2**, 4x$\mathbf{2}^1$, 2x$\mathbf{2}^2$) **k**

	1	2	3	4	5
1	0	0	1	0	2^2
2	0	0	2^1	2^1	0
3	1	2^1	0	0	1
4	0	2^1	0	2	0
5	2^2	0	1	0	0

(4x**1**, 1x**2**, 4x$\mathbf{2}^1$, 2x$\mathbf{2}^2$) **l**

	1	2	3	4	5
1	0	0	1	0	2^1
2	0	0	2^2	2^1	0
3	1	2^2	0	0	1
4	0	2^1	0	2	0
5	2^1	0	1	0	0

(4x**1**, 1x**2**, 4x$\mathbf{2}^1$, 2x$\mathbf{2}^2$) **m**

	1	2	3	4	5
1	0	0	1	0	2^1
2	0	0	2^1	2^2	0
3	1	2^1	0	0	1
4	0	2^2	0	2	0
5	2^1	0	1	0	0

(4x**1**, 1x**2**, 2x$\mathbf{2}^1$, 4x$\mathbf{2}^2$) **a**

	1	2	3	4	5
1	2	1	0	0	1
2	1	0	2^1	0	0
3	0	2^1	0	2^2	0
4	0	0	2^2	0	2^2
5	1	0	0	2^2	0

(4x**1**, 1x**2**, 2x$\mathbf{2}^1$, 4x$\mathbf{2}^2$) **b**

	1	2	3	4	5
1	2	1	0	0	1
2	1	0	2^2	0	0
3	0	2^2	0	2^1	0
4	0	0	2^1	0	2^2
5	1	0	0	2^2	0

(4x**1**, 1x**2**, 2x$\mathbf{2}^1$, 4x$\mathbf{2}^2$) **c**

	1	2	3	4	5
1	0	0	0	2^1	2^2
2	0	2	0	1	0
3	0	0	0	1	2^2
4	2^1	1	1	0	0
5	2^2	0	2^2	0	0

(4x**1**, 1x**2**, 2x$\mathbf{2}^1$, 4x$\mathbf{2}^2$) **d**

	1	2	3	4	5
1	0	0	0	2^2	2^2
2	0	2	0	1	0
3	0	0	0	1	2^1
4	2^2	1	1	0	0
5	2^2	0	2^1	0	0

(4x**1**, 1x**2**, 2x$\mathbf{2}^1$, 4x$\mathbf{2}^2$) **e**

	1	2	3	4	5
1	0	0	0	2^2	2^1
2	0	2	0	1	0
3	0	0	0	1	2^2
4	2^2	1	1	0	0
5	2^1	0	2^2	0	0

(4x**1**, 1x**2**, 2x$\mathbf{2}^1$, 4x$\mathbf{2}^2$) **f**

	1	2	3	4	5
1	0	0	0	1	2^2
2	0	2	0	2^1	0
3	0	0	0	1	2^2
4	1	2^1	1	0	0
5	2^2	0	2^2	0	0

(4x**1**, 1x**2**, 2x$\mathbf{2}^1$, 4x$\mathbf{2}^2$) **g**

	1	2	3	4	5
1	0	0	0	1	2^1
2	0	2	0	2^2	0
3	0	0	0	1	2^2
4	1	2^2	1	0	0
5	2^1	0	2^2	0	0

(4x**1**, 1x**2**, 2x$\mathbf{2}^1$, 4x$\mathbf{2}^2$) **h**

	1	2	3	4	5
1	0	0	1	0	2^2
2	0	0	1	2^1	0
3	1	1	0	0	2^2
4	0	2^1	0	2	0
5	2^2	0	2^2	0	0

(4x**1**, 1x**2**, 2x$\mathbf{2}^1$, 4x$\mathbf{2}^2$) **i**

	1	2	3	4	5
1	0	0	1	0	2^1
2	0	0	1	2^2	0
3	1	1	0	0	2^2
4	0	2^2	0	2	0
5	2^1	0	2^2	0	0

(4x**1**, 1x**2**, 2x$\mathbf{2}^1$, 4x$\mathbf{2}^2$) **j**

	1	2	3	4	5
1	0	0	1	0	2^2
2	0	0	1	2^2	0
3	1	1	0	0	2^1
4	0	2^2	0	2	0
5	2^2	0	2^1	0	0

(4x**1**, 1x**2**, 2x$\mathbf{2}^1$, 4x$\mathbf{2}^2$) **k**

	1	2	3	4	5
1	0	0	1	0	2^2
2	0	0	2^2	2^1	0
3	1	2^2	0	0	1
4	0	2^1	0	2	0
5	2^2	0	1	0	0

(4x**1**, 1x**2**, 2x$\mathbf{2}^1$, 4x$\mathbf{2}^2$) **l**

	1	2	3	4	5
1	0	0	1	0	2^1
2	0	0	2^2	2^2	0
3	1	2^2	0	0	1
4	0	2^2	0	2	0
5	2^1	0	1	0	0

(4x**1**, 1x**2**, 2x$\mathbf{2}^1$, 4x$\mathbf{2}^2$) **m**

	1	2	3	4	5
1	0	0	1	0	2^2
2	0	0	2^1	2^2	0
3	1	2^1	0	0	1
4	0	2^2	0	2	0
5	2^2	0	1	0	0

(4x**1**, 1x**2**, 6x$\mathbf{2}^2$) **a**

	1	2	3	4	5
1	2	1	0	0	1
2	1	0	2^2	0	0
3	0	2^2	0	2^2	0
4	0	0	2^2	0	2^2
5	1	0	0	2^2	0

(4x**1**, 1x**2**, 6x$\mathbf{2}^2$) **b**

	1	2	3	4	5
1	0	0	0	2^2	2^2
2	0	2	0	1	0
3	0	0	0	1	2^2
4	2^2	1	1	0	0
5	2^2	0	2^2	0	0

(4x**1**, 1x**2**, 6x$\mathbf{2}^2$) **c**

	1	2	3	4	5
1	0	0	0	1	2^2
2	0	2	0	2^2	0
3	0	0	0	1	2^2
4	1	2^2	1	0	0
5	2^2	0	2^2	0	0

(4x**1**, 1x**2**, 6x$\mathbf{2}^2$) **d**

	1	2	3	4	5
1	0	0	1	0	2^2
2	0	0	1	2^2	0
3	1	1	0	0	2^2
4	0	2^2	0	2	0
5	2^2	0	2^2	0	0

(4x**1**, 1x**2**, 6x$\mathbf{2}^2$) **e**

	1	2	3	4	5
1	0	0	1	0	2^2
2	0	0	2^2	2^2	0
3	1	2^2	0	0	1
4	0	2^2	0	2	0
5	2^2	0	1	0	0

(2x**1**, 4x**2**, 4x$\mathbf{2}^1$) **a**

	1	2	3	4	5
1	2	0	0	0	2^1
2	0	2	0	1	0
3	0	0	2	0	2^1
4	0	1	0	2	0
5	2^1	0	2^1	0	0

(2x**1**, 4x**2**, 4x$\mathbf{2}^1$) **b**

	1	2	3	4	5
1	2	0	0	0	2^1
2	0	2	0	2^1	0
3	0	0	2	0	1
4	0	2^1	0	2	0
5	2^1	0	1	0	0

(2x**1**, 4x**2**, 2x$\mathbf{2}^1$, 2x$\mathbf{2}^2$) **a**

	1	2	3	4	5
1	2	0	0	0	2^1
2	0	2	0	1	0
3	0	0	2	0	2^2
4	0	1	0	2	0
5	2^1	0	2^2	0	0

(2x**1**, 4x**2**, 2x$\mathbf{2}^1$, 2x$\mathbf{2}^2$) **b**

	1	2	3	4	5
1	2	0	0	0	2^2
2	0	2	0	2^1	0
3	0	0	2	0	1
4	0	2^1	0	2	0
5	2^2	0	1	0	0

(2x**1**, 4x**2**, 2x$\mathbf{2}^1$, 2x$\mathbf{2}^2$) **c**

	1	2	3	4	5
1	2	0	0	0	2^1
2	0	2	0	2^2	0
3	0	0	2	0	1
4	0	2^2	0	2	0
5	2^1	0	1	0	0

(2x**1**, 4x**2**, 4x$\mathbf{2}^2$) **a**

	1	2	3	4	5
1	2	0	0	0	2^2
2	0	2	0	1	0
3	0	0	2	0	2^2
4	0	1	0	2	0
5	2^2	0	2^2	0	0

(2x**1**, 4x**2**, 4x$\mathbf{2}^2$) **b**

	1	2	3	4	5
1	2	0	0	0	2^2
2	0	2	0	2^2	0
3	0	0	2	0	1
4	0	2^2	0	2	0
5	2^2	0	1	0	0

(2x**1**, 2x**2**, 6x$\mathbf{2}^1$) **a**

	1	2	3	4	5
1	0	0	2^1	0	2^1
2	0	2	0	1	0
3	2^1	0	0	0	2^1
4	0	1	0	2	0
5	2^1	0	2^1	0	0

(2x**1**, 2x**2**, 6x$\mathbf{2}^1$) **b**

	1	2	3	4	5
1	0	0	1	0	2^1
2	0	2	0	2^1	0
3	1	0	0	0	2^1
4	0	2^1	0	2	0
5	2^1	0	2^1	0	0

(2x**1**, 2x**2**, 6x$\mathbf{2}^1$) **c**

	1	2	3	4	5
1	2	0	0	0	1
2	0	2	0	2^1	0
3	0	0	0	2^1	2^1
4	0	2^1	2^1	0	0
5	1	0	2^1	0	0

(2x**1**, 2x**2**, 6x$\mathbf{2}^1$) **d**

	1	2	3	4	5
1	2	0	0	0	2^1
2	0	2	0	2^1	0
3	0	0	0	2^1	1
4	0	2^1	2^1	0	0
5	2^1	0	1	0	0

(2x**1**, 2x**2**, 4x$\mathbf{2}^1$, 2x$\mathbf{2}^2$) **a**

	1	2	3	4	5
1	0	0	2^1	0	2^1
2	0	2	0	1	0
3	2^1	0	0	0	2^2
4	0	1	0	2	0
5	2^1	0	2^2	0	0

(2x**1**, 2x**2**, 4x$\mathbf{2}^1$, 2x$\mathbf{2}^2$) **b**

	1	2	3	4	5
1	0	0	1	0	2^1
2	0	2	0	2^1	0
3	1	0	0	0	2^2
4	0	2^1	0	2	0
5	2^1	0	2^2	0	0

(2x**1**, 2x**2**, 4x$\mathbf{2}^1$, 2x$\mathbf{2}^2$) **c**

	1	2	3	4	5
1	0	0	1	0	2^1
2	0	2	0	2^2	0
3	1	0	0	0	2^1
4	0	2^2	0	2	0
5	2^1	0	2^1	0	0

(2x**1**, 2x**2**, 4x**2**^{1}, 2x**2**^{2}) **d**

	1	2	3	4	5
1	2	0	0	0	1
2	0	2	0	2^2	0
3	0	0	0	2^1	2^1
4	0	2^2	2^1	0	0
5	1	0	2^1	0	0

(2x**1**, 2x**2**, 4x**2**^{1}, 2x**2**^{2}) **e**

	1	2	3	4	5
1	2	0	0	0	1
2	0	2	0	2^1	0
3	0	0	0	2^2	2^1
4	0	2^1	2^2	0	0
5	1	0	2^1	0	0

(2x**1**, 2x**2**, 4x**2**^{1}, 2x**2**^{2}) **f**

	1	2	3	4	5
1	2	0	0	0	1
2	0	2	0	2^1	0
3	0	0	0	2^1	2^2
4	0	2^1	2^1	0	0
5	1	0	2^2	0	0

(2x**1**, 2x**2**, 4x**2**^{1}, 2x**2**^{2}) **g**

	1	2	3	4	5
1	2	0	0	0	2^1
2	0	2	0	2^2	0
3	0	0	0	2^1	1
4	0	2^2	2^1	0	0
5	2^1	0	1	0	0

(2x**1**, 2x**2**, 4x**2**^{1}, 2x**2**^{2}) **h**

	1	2	3	4	5
1	2	0	0	0	2^1
2	0	2	0	2^1	0
3	0	0	0	2^2	1
4	0	2^1	2^2	0	0
5	2^1	0	1	0	0

(2x**1**, 2x**2**, 4x**2**^{1}, 2x**2**^{2}) **i**

	1	2	3	4	5
1	2	0	0	0	2^2
2	0	2	0	2^1	0
3	0	0	0	2^1	1
4	0	2^1	2^1	0	0
5	2^2	0	1	0	0

(2x**1**, 2x**2**, 2x**2**^{1}, 4x**2**^{2}) **a**

	1	2	3	4	5
1	0	0	2^1	0	2^2
2	0	2	0	1	0
3	2^1	0	0	0	2^2
4	0	1	0	2	0
5	2^2	0	2^2	0	0

(2x**1**, 2x**2**, 2x**2**^{1}, 4x**2**^{2}) **b**

	1	2	3	4	5
1	0	0	1	0	2^1
2	0	2	0	2^2	0
3	1	0	0	0	2^2
4	0	2^2	0	2	0
5	2^1	0	2^2	0	0

(2x**1**, 2x**2**, 2x**2**^{1}, 4x**2**^{2}) **c**

	1	2	3	4	5
1	0	0	1	0	2^2
2	0	2	0	2^1	0
3	1	0	0	0	2^2
4	0	2^1	0	2	0
5	2^2	0	2^2	0	0

(2x**1**, 2x**2**, 2x**2**^{1}, 4x**2**^{2}) **d**

	1	2	3	4	5
1	2	0	0	0	1
2	0	2	0	2^2	0
3	0	0	0	2^2	2^1
4	0	2^2	2^2	0	0
5	1	0	2^1	0	0

(2x**1**, 2x**2**, 2x**2**^{1}, 4x**2**^{2}) **e**

	1	2	3	4	5
1	2	0	0	0	1
2	0	2	0	2^1	0
3	0	0	0	2^2	2^2
4	0	2^1	2^2	0	0
5	1	0	2^2	0	0

(2x**1**, 2x**2**, 2x**2**^{1}, 4x**2**^{2}) **f**

	1	2	3	4	5
1	2	0	0	0	1
2	0	2	0	2^2	0
3	0	0	0	2^1	2^2
4	0	2^2	2^1	0	0
5	1	0	2^2	0	0

(2x**1**, 2x**2**, 2x**2**^{1}, 4x**2**^{2}) **g**

	1	2	3	4	5
1	2	0	0	0	2^1
2	0	2	0	2^2	0
3	0	0	0	2^2	1
4	0	2^2	2^2	0	0
5	2^1	0	1	0	0

(2x**1**, 2x**2**, 2x**2**^{1}, 4x**2**^{2}) **h**

	1	2	3	4	5
1	2	0	0	0	2^2
2	0	2	0	2^1	0
3	0	0	0	2^2	1
4	0	2^1	2^2	0	0
5	2^2	0	1	0	0

(2x**1**, 2x**2**, 2x**2**^{1}, 4x**2**^{2}) **i**

	1	2	3	4	5
1	2	0	0	0	2^2
2	0	2	0	2^2	0
3	0	0	0	2^1	1
4	0	2^2	2^1	0	0
5	2^2	0	1	0	0

(2x**1**, 2x**2**, 6x**2**^{2}) **a**

	1	2	3	4	5
1	0	0	2^2	0	2^2
2	0	2	0	1	0
3	2^2	0	0	0	2^2
4	0	1	0	2	0
5	2^2	0	2^2	0	0

(2x**1**, 2x**2**, 6x**2**^{2}) **b**

	1	2	3	4	5
1	0	0	1	0	2^2
2	0	2	0	2^2	0
3	1	0	0	0	2^2
4	0	2^2	0	2	0
5	2^2	0	2^2	0	0

(2x**1**, 2x**2**, 6x**2**^{2}) **c**

	1	2	3	4	5
1	2	0	0	0	1
2	0	2	0	2^2	0
3	0	0	0	2^2	2^2
4	0	2^2	2^2	0	0
5	1	0	2^2	0	0

(2x**1**, 2x**2**, 6x**2**^{2}) **d**

	1	2	3	4	5
1	2	0	0	0	2^2
2	0	2	0	2^2	0
3	0	0	0	2^2	1
4	0	2^2	2^2	0	0
5	2^2	0	1	0	0

(2x**1**, 8x**2**^{1})

	1	2	3	4	5
1	0	2^1	0	0	1
2	2^1	0	2^1	0	0
3	0	2^1	0	2^1	0
4	0	0	2^1	0	2^1
5	1	0	0	2^1	0

(2x**1**, 6x**2**^{1}, 2x**2**^{2}) **a**

	1	2	3	4	5
1	0	2^2	0	0	1
2	2^2	0	2^1	0	0
3	0	2^1	0	2^1	0
4	0	0	2^1	0	2^1
5	1	0	0	2^1	0

(2x**1**, 6x**2**^{1}, 2x**2**^{2}) **b**

	1	2	3	4	5
1	0	2^1	0	0	1
2	2^1	0	2^1	0	0
3	0	2^1	0	2^2	0
4	0	0	2^2	0	2^1
5	1	0	0	2^1	0

(2x**1**, 4x**2**^{1}, 4x**2**^{2}) **a**

	1	2	3	4	5
1	0	2^2	0	0	1
2	2^2	0	2^2	0	0
3	0	2^2	0	2^1	0
4	0	0	2^1	0	2^1
5	1	0	0	2^1	0

(2x**1**, 4x**2**^{1}, 4x**2**^{2}) **b**

	1	2	3	4	5
1	0	2^2	0	0	1
2	2^2	0	2^1	0	0
3	0	2^1	0	2^2	0
4	0	0	2^2	0	2^1
5	1	0	0	2^1	0

(2x**1**, 4x**2**^{1}, 4x**2**^{2}) **c**

	1	2	3	4	5
1	0	2^2	0	0	1
2	2^2	0	2^1	0	0
3	0	2^1	0	2^1	0
4	0	0	2^1	0	2^2
5	1	0	0	2^2	0

(2x**1**, 2x**2**^{1}, 6x**2**^{2}) **a**

	1	2	3	4	5
1	0	2^2	0	0	1
2	2^2	0	2^2	0	0
3	0	2^2	0	2^2	0
4	0	0	2^2	0	2^1
5	1	0	0	2^1	0

(2x**1**, 2x**2**^{1}, 6x**2**^{2}) **b**

	1	2	3	4	5
1	0	2^2	0	0	1
2	2^2	0	2^2	0	0
3	0	2^2	0	2^1	0
4	0	0	2^1	0	2^2
5	1	0	0	2^2	0

(2x**1**, 8x**2**^{2})

	1	2	3	4	5
1	0	2^2	0	0	1
2	2^2	0	2^2	0	0
3	0	2^2	0	2^2	0
4	0	0	2^2	0	2^2
5	1	0	0	2^2	0

$^3V_2{}^4V_4$	NG
$^3V_2{}^4V_5$	NG
$^3V_2{}^4V_6$	NG

$^{3}V_{4}{}^{4}V_{1}$

(16x**1**)

	1	2	3	4	5
1	0	1	1	0	1
2	1	0	0	1	1
3	1	0	0	1	1
4	0	1	1	0	1
5	1	1	1	1	0

(14x**1**, 1x**2**) **a**

	1	2	3	4	5
1	2	1	0	0	1
2	1	0	1	1	0
3	0	1	0	1	1
4	0	1	1	0	1
5	1	0	1	1	0

(14x**1**, 1x**2**) **b**

	1	2	3	4	5
1	2	0	0	0	1
2	0	0	1	1	1
3	0	1	0	1	1
4	0	1	1	0	1
5	1	1	1	1	0

(12x**1**, 2x**2**)

	1	2	3	4	5
1	2	0	0	0	1
2	0	0	1	1	1
3	0	1	2	1	0
4	0	1	1	0	1
5	1	1	0	1	0

(12x**1**, 2x**2**^{1})

	1	2	3	4	5
1	0	2^1	0	0	1
2	2^1	0	1	1	0
3	0	1	0	1	1
4	0	1	1	0	1
5	1	0	1	1	0

(12x**1**, 2x**2**^{2})

	1	2	3	4	5
1	0	2^2	0	0	1
2	2^2	0	1	1	0
3	0	1	0	1	1
4	0	1	1	0	1
5	1	0	1	1	0

(10x**1**, 3x**2**)

	1	2	3	4	5
1	2	0	0	0	1
2	0	2	0	1	1
3	0	0	2	1	0
4	0	1	1	0	1
5	1	1	0	1	0

(10x**1**, 1x**2**, 2x**2**^{1}) **a**

	1	2	3	4	5
1	2	1	0	0	1
2	1	0	1	0	1
3	0	1	0	2^1	0
4	0	0	2^1	0	1
5	1	1	0	1	0

(10x**1**, 1x**2**, 2x**2**^{1}) **b**

	1	2	3	4	5
1	2	0	0	0	1
2	0	0	1	1	1
3	0	1	0	2^1	0
4	0	1	2^1	0	1
5	1	1	0	1	0

(10x**1**, 1x**2**, 2x**2**^{2}) **a**

	1	2	3	4	5
1	2	1	0	0	1
2	1	0	1	0	1
3	0	1	0	2^2	0
4	0	0	2^2	0	1
5	1	1	0	1	0

(10x**1**, 1x**2**, 2x**2**^{2}) **b**

	1	2	3	4	5
1	2	0	0	0	1
2	0	0	1	1	1
3	0	1	0	2^2	0
4	0	1	2^2	0	1
5	1	1	0	1	0

(8x**1**, 4x**2**) **a**

	1	2	3	4	5
1	2	0	0	0	1
2	0	2	0	0	1
3	0	0	2	0	1
4	0	0	0	2	1
5	1	1	1	1	0

(8x**1**, 4x**2**) **b**

	1	2	3	4	5
1	2	0	0	0	1
2	0	2	0	0	1
3	0	0	2	1	1
4	0	0	1	2	0
5	1	1	1	0	0

(8x**1**, 2x**2**, 2x**2**^{1}) **a**

	1	2	3	4	5
1	2	0	0	0	1
2	0	2	1	0	1
3	0	1	0	2^1	0
4	0	0	2^1	0	1
5	1	1	0	1	0

(8x**1**, 2x**2**, 2x**2**^{1}) **b**

	1	2	3	4	5
1	2	0	0	0	1
2	0	2	0	0	1
3	0	0	0	2^1	1
4	0	0	2^1	0	1
5	1	1	1	1	0

(8x**1**, 2x**2**, 2x**2**^{1}) **c**

	1	2	3	4	5
1	2	1	0	0	1
2	1	2	0	0	0
3	0	0	0	2^1	1
4	0	0	2^1	0	1
5	1	0	1	1	0

(8x**1**, 2x**2**, 2x**2**^{1}) **d**

	1	2	3	4	5
1	2	0	0	0	1
2	0	2	1	0	0
3	0	1	0	2^1	1
4	0	0	2^1	0	1
5	1	0	1	1	0

(8x**1**, 2x**2**, 2x**2**^{2}) **a**

	1	2	3	4	5
1	2	0	0	0	1
2	0	2	1	0	1
3	0	1	0	2^2	0
4	0	0	2^2	0	1
5	1	1	0	1	0

(8x**1**, 2x**2**, 2x**2**^{2}) **b**

	1	2	3	4	5
1	2	0	0	0	1
2	0	2	0	0	1
3	0	0	0	2^2	1
4	0	0	2^2	0	1
5	1	1	1	1	0

(8x**1**, 2x**2**, 2x**2**^{2}) **c**

	1	2	3	4	5
1	2	1	0	0	1
2	1	2	0	0	0
3	0	0	0	2^2	1
4	0	0	2^2	0	1
5	1	0	1	1	0

(8x**1**, 2x**2**, 2x**2**^{2}) **d**

	1	2	3	4	5
1	2	0	0	0	1
2	0	2	1	0	0
3	0	1	0	2^2	1
4	0	0	2^2	0	1
5	1	0	1	1	0

(8x**1**, 4x**2**^{1}) **a**

	1	2	3	4	5
1	0	2^1	0	0	1
2	2^1	0	1	0	1
3	0	1	0	2^1	0
4	0	0	2^1	0	1
5	1	1	0	1	0

(8x**1**, 4x**2**^{1}) **b**

	1	2	3	4	5
1	0	2^1	0	0	1
2	2^1	0	0	0	1
3	0	0	0	2^1	1
4	0	0	2^1	0	1
5	1	1	1	1	0

(8x**1**, 2x**2**^{1}, 2x**2**^{2}) **a**

	1	2	3	4	5
1	0	2^1	0	0	1
2	2^1	0	1	0	1
3	0	1	0	2^2	0
4	0	0	2^2	0	1
5	1	1	0	1	0

(8x**1**, 2x**2**^{1}, 2x**2**^{2}) **b**

	1	2	3	4	5
1	0	2^2	0	0	1
2	2^2	0	1	0	1
3	0	1	0	2^1	0
4	0	0	2^1	0	1
5	1	1	0	1	0

(8x**1**, 2x**2**^{1}, 2x**2**^{2}) **c**

	1	2	3	4	5
1	0	2^1	0	0	1
2	2^1	0	0	0	1
3	0	0	0	2^2	1
4	0	0	2^2	0	1
5	1	1	1	1	0

(8x**1**, 4x**2**^{2}) **a**

	1	2	3	4	5
1	0	2^2	0	0	1
2	2^2	0	1	0	1
3	0	1	0	2^2	0
4	0	0	2^2	0	1
5	1	1	0	1	0

(8x**1**, 4x**2**^{2}) **b**

	1	2	3	4	5
1	0	2^1	0	0	1
2	2^1	0	0	0	1
3	0	0	0	2^1	1
4	0	0	2^1	0	1
5	1	1	1	1	0

(6x**1**, 5x**2**)

	1	2	3	4	5
1	2	1	0	0	0
2	1	2	0	0	0
3	0	0	2	0	1
4	0	0	0	2	1
5	0	0	1	1	2

(6x**1**, 3x**2**, 2x**2**^{1}) **a**

	1	2	3	4	5
1	2	0	0	0	1
2	0	2	0	0	1
3	0	0	0	2^1	1
4	0	0	2^1	2	0
5	1	1	1	0	0

(6x**1**, 3x**2**, 2x**2**^{1}) **b**

	1	2	3	4	5
1	2	0	0	0	1
2	0	2	0	0	1
3	0	0	0	1	2^1
4	0	0	1	2	0
5	1	1	2^1	0	0

(6x**1**, 3x**2**, 2x**2**^{1}) **c**

	1	2	3	4	5
1	2	0	0	0	1
2	0	2	0	1	0
3	0	0	0	1	2^1
4	0	1	1	2	0
5	1	0	2^1	0	0

(6x**1**, 3x**2**, 2x**2**^{1}) **d**

	1	2	3	4	5
1	2	1	0	0	0
2	1	2	0	0	0
3	0	0	0	1	2^1
4	0	0	1	2	1
5	0	0	2^1	1	0

(6x**1**, 3x**2**, 2x**2**^{2}) **a**

	1	2	3	4	5
1	2	0	0	0	1
2	0	2	0	0	1
3	0	0	0	2^2	1
4	0	0	2^2	2	0
5	1	1	1	0	0

(6x**1**, 3x**2**, 2x**2**^{2}) **b**

	1	2	3	4	5
1	2	0	0	0	1
2	0	2	0	0	1
3	0	0	0	1	2^2
4	0	0	1	2	0
5	1	1	2^2	0	0

(6x1, 3x2, 2x2^2) c

	1	2	3	4	5
1	2	0	0	0	1
2	0	2	0	1	0
3	0	0	0	1	2^2
4	0	1	1	2	0
5	1	0	2^2	0	0

(6x1, 3x2, 2x2^2) d

	1	2	3	4	5
1	2	1	0	0	0
2	1	2	0	0	0
3	0	0	0	1	2^2
4	0	0	1	2	1
5	0	0	2^2	1	0

(6x1, 1x2, 4x2^1) a

	1	2	3	4	5
1	2	0	0	0	1
2	0	0	1	0	2^1
3	0	1	0	2^1	0
4	0	0	2^1	0	1
5	1	2^1	0	1	0

(6x1, 1x2, 4x2^1) b

	1	2	3	4	5
1	2	0	0	0	1
2	0	0	2^1	0	1
3	0	2^1	0	2^1	0
4	0	0	2^1	0	1
5	1	1	0	1	0

(6x1, 1x2, 4x2^1) c

	1	2	3	4	5
1	0	2^1	0	0	1
2	2^1	2	0	0	0
3	0	0	0	2^1	1
4	0	0	2^1	0	1
5	1	0	1	1	0

(6x1, 1x2, 4x2^1) d

	1	2	3	4	5
1	0	1	0	0	2^1
2	1	2	0	0	0
3	0	0	0	2^1	1
4	0	0	2^1	0	1
5	2^1	0	1	1	0

(6x1, 1x2, 4x2^1) e

	1	2	3	4	5
1	2	1	0	0	1
2	1	0	2^1	0	0
3	0	2^1	0	1	0
4	0	0	1	0	2^1
5	1	0	0	2^1	0

(6x1, 1x2, 2x2^1, 2x2^2) a

	1	2	3	4	5
1	2	0	0	0	1
2	0	0	1	0	2^1
3	0	1	0	2^2	0
4	0	0	2^2	0	1
5	1	2^1	0	1	0

(6x1, 1x2, 2x2^1, 2x2^2) b

	1	2	3	4	5
1	2	0	0	0	1
2	0	0	1	0	2^2
3	0	1	0	2^1	0
4	0	0	2^1	0	1
5	1	2^2	0	1	0

(6x1, 1x2, 2x2^1, 2x2^2) c

	1	2	3	4	5
1	2	0	0	0	1
2	0	0	2^1	0	1
3	0	2^1	0	2^2	0
4	0	0	2^2	0	1
5	1	1	0	1	0

(6x1, 1x2, 2x2^1, 2x2^2) d

	1	2	3	4	5
1	0	2^1	0	0	1
2	2^1	2	0	0	0
3	0	0	0	2^2	1
4	0	0	2^2	0	1
5	1	0	1	1	0

(6x1, 1x2, 2x2^1, 2x2^2) e

	1	2	3	4	5
1	0	2^2	0	0	1
2	2^2	2	0	0	0
3	0	0	0	2^1	1
4	0	0	2^1	0	1
5	1	0	1	1	0

(6x1, 1x2, 2x2^1, 2x2^2) f

	1	2	3	4	5
1	0	1	0	0	2^1
2	1	2	0	0	0
3	0	0	0	2^2	1
4	0	0	2^2	0	1
5	2^1	0	1	1	0

(6x1, 1x2, 2x2^1, 2x2^2) g

	1	2	3	4	5
1	0	1	0	0	2^2
2	1	2	0	0	0
3	0	0	0	2^1	1
4	0	0	2^1	0	1
5	2^2	0	1	1	0

(6x1, 1x2, 2x2^1, 2x2^2) h

	1	2	3	4	5
1	2	1	0	0	1
2	1	0	2^1	0	0
3	0	2^1	0	1	0
4	0	0	1	0	2^2
5	1	0	0	2^2	0

(6x1, 1x2, 4x2^2) a

	1	2	3	4	5
1	2	0	0	0	1
2	0	0	1	0	2^2
3	0	1	0	2^2	0
4	0	0	2^2	0	1
5	1	2^2	0	1	0

(6x1, 1x2, 4x2^2) b

	1	2	3	4	5
1	2	0	0	0	1
2	0	0	2^2	0	1
3	0	2^2	0	2^2	0
4	0	0	2^2	0	1
5	1	1	0	1	0

(6x1, 1x2, 4x2^2) c

	1	2	3	4	5
1	0	2^2	0	0	1
2	2^2	2	0	0	0
3	0	0	0	2^2	1
4	0	0	2^2	0	1
5	1	0	1	1	0

(6x1, 1x2, 4x2^2) d

	1	2	3	4	5
1	0	1	0	0	2^2
2	1	2	0	0	0
3	0	0	0	2^2	1
4	0	0	2^2	0	1
5	2^2	0	1	1	0

(6x1, 1x2, 4x2^2) e

	1	2	3	4	5
1	2	1	0	0	1
2	1	0	2^2	0	0
3	0	2^2	0	1	0
4	0	0	1	0	2^2
5	1	0	0	2^2	0

(4x1, 4x2, 2x2^1)

	1	2	3	4	5
1	0	2^1	0	0	1
2	2^1	2	0	0	0
3	0	0	2	1	0
4	0	0	1	2	0
5	1	0	0	0	2

(4x1, 4x2, 2x2^2)

	1	2	3	4	5
1	0	2^2	0	0	1
2	2^2	2	0	0	0
3	0	0	2	1	0
4	0	0	1	2	0
5	1	0	0	0	2

(4x1, 2x2, 4x2^1) a

	1	2	3	4	5
1	0	2^1	0	0	1
2	2^1	0	2^1	0	0
3	0	2^1	0	1	0
4	0	0	1	2	0
5	1	0	0	0	2

(4x1, 2x2, 4x2^1) b

	1	2	3	4	5
1	0	1	0	0	2^1
2	1	0	2^1	0	0
3	0	2^1	0	1	0
4	0	0	1	2	0
5	2^1	0	0	0	2

(4x1, 2x2, 4x2^1) c

	1	2	3	4	5
1	0	2^1	1	0	0
2	2^1	0	2^1	0	0
3	1	2^1	0	0	0
4	0	0	0	2	1
5	0	0	0	1	2

(4x1, 2x2, 2x2^1, 2x2^2) a

	1	2	3	4	5
1	0	2^2	0	0	1
2	2^2	0	2^1	0	0
3	0	2^1	0	1	0
4	0	0	1	2	0
5	1	0	0	0	2

(4x1, 2x2, 2x2^1, 2x2^2) b

	1	2	3	4	5
1	0	1	0	0	2^2
2	1	0	2^1	0	0
3	0	2^1	0	1	0
4	0	0	1	2	0
5	2^2	0	0	0	2

(4x1, 2x2, 2x2^1, 2x2^2) c

	1	2	3	4	5
1	0	1	0	0	2^1
2	1	0	2^2	0	0
3	0	2^2	0	1	0
4	0	0	1	2	0
5	2^1	0	0	0	2

(4x1, 2x2, 2x2^1, 2x2^2) d

	1	2	3	4	5
1	0	2^2	1	0	0
2	2^2	0	2^1	0	0
3	1	2^1	0	0	0
4	0	0	0	2	1
5	0	0	0	1	2

(4x1, 2x2, 4x2^2) a

	1	2	3	4	5
1	0	2^2	0	0	1
2	2^2	0	2^2	0	0
3	0	2^2	0	1	0
4	0	0	1	2	0
5	1	0	0	0	2

(4x1, 2x2, 4x2^2) b

	1	2	3	4	5
1	0	1	0	0	2^2
2	1	0	2^2	0	0
3	0	2^2	0	1	0
4	0	0	1	2	0
5	2^2	0	0	0	2

(4x1, 2x2, 4x2^2) c

	1	2	3	4	5
1	0	2^2	1	0	0
2	2^2	0	2^2	0	0
3	1	2^2	0	0	0
4	0	0	0	2	1
5	0	0	0	1	2

(4x1, 6x2^1)

	1	2	3	4	5
1	0	2^1	0	0	1
2	2^1	0	2^1	0	0
3	0	2^1	0	1	0
4	0	0	1	0	2^1
5	1	0	0	2^1	0

(4x1, 4x2^1, 2x2^2) a

	1	2	3	4	5
1	0	2^1	0	0	1
2	2^1	0	2^2	0	0
3	0	2^2	0	1	0
4	0	0	1	0	2^1
5	1	0	0	2^1	0

(4x1, 4x2^1, 2x2^2) b

	1	2	3	4	5
1	0	2^1	0	0	1
2	2^1	0	2^1	0	0
3	0	2^1	0	1	0
4	0	0	1	0	2^2
5	1	0	0	2^2	0

(4x**1**, 2x**2**^{1}, 4x**2**^{2}) **a**

	1	2	3	4	5
1	0	2^{2}	0	0	1
2	2^{2}	0	2^{2}	0	0
3	0	2^{2}	0	1	0
4	0	0	1	0	2^{1}
5	1	0	0	2^{1}	0

(4x**1**, 2x**2**^{1}, 4x**2**^{2}) **b**

	1	2	3	4	5
1	0	2^{1}	0	0	1
2	2^{1}	0	2^{2}	0	0
3	0	2^{2}	0	1	0
4	0	0	1	0	2^{2}
5	1	0	0	2^{2}	0

(4x**1**, 6x**2**^{2})

	1	2	3	4	5
1	0	2^{2}	0	0	1
2	2^{2}	0	2^{2}	0	0
3	0	2^{2}	0	1	0
4	0	0	1	0	2^{2}
5	1	0	0	2^{2}	0

$^{3}V_{4}{}^{4}V_{2}$	NG
$^{3}V_{4}{}^{4}V_{3}$	NG
$^{3}V_{4}{}^{4}V_{4}$	NG
$^{3}V_{6}{}^{4}V_{1}$	NG
$^{3}V_{6}{}^{4}V_{2}$	NG

Rank 3

$^{1}V_{r}{}^{2}V_{r}{}^{3}V_{r}$

$^{1}V_{1}{}^{2}V_{1}{}^{3}V_{1}$

(4x**1**, 1x**2**)

	1	2	3
1	0	1	1
2	1	0	0
3	1	0	2

(2x**1**, 2x**2**)

	1	2	3
1	0	0	1
2	0	2	0
3	1	0	2

(2x**1**, 2x**2**^{1})

	1	2	3
1	0	1	2^{1}
2	1	0	0
3	2^{1}	0	0

(2x**1**, 2x**2**^{2})

	1	2	3
1	0	1	2^{2}
2	1	0	0
3	2^{2}	0	0

$^{1}V_{1}{}^{2}V_{1}{}^{3}V_{3}$

(12x**1**)

	1	2	3	4	5
1	0	0	0	0	1
2	0	0	1	1	1
3	0	1	0	1	0
4	0	1	1	0	1
5	1	1	0	1	0

(10x**1**, 1x**2**)

	1	2	3	4	5
1	2	0	0	0	1
2	0	0	0	1	1
3	0	0	0	1	0
4	0	1	1	0	1
5	1	1	0	1	0

(8x**1**, 2x**2**) **a**

	1	2	3	4	5
1	2	0	0	0	1
2	0	2	0	0	1
3	0	0	0	1	0
4	0	0	1	0	1
5	1	1	0	1	0

(8x**1**, 2x**2**) **b**

	1	2	3	4	5
1	0	0	0	0	1
2	0	2	0	0	1
3	0	0	2	1	0
4	0	0	1	0	1
5	1	1	0	1	0

(8x**1**, 2x**2**^{1}) **a**

	1	2	3	4	5
1	0	0	0	0	1
2	0	0	1	0	1
3	0	1	0	2^{1}	0
4	0	0	2^{1}	0	1
5	1	1	0	1	0

(8x**1**, 2x**2**^{1}) **b**

	1	2	3	4	5
1	0	1	0	0	1
2	1	0	0	0	0
3	0	0	0	2^{1}	1
4	0	0	2^{1}	0	1
5	1	0	1	1	0

(8x**1**, 2x**2**^{2}) **a**

	1	2	3	4	5
1	0	0	0	0	1
2	0	0	1	0	1
3	0	1	0	2^{2}	0
4	0	0	2^{2}	0	1
5	1	1	0	1	0

(8x**1**, 2x**2**^{2}) **b**

	1	2	3	4	5
1	0	1	0	0	1
2	1	0	0	0	0
3	0	0	0	2^{2}	1
4	0	0	2^{2}	0	1
5	1	0	1	1	0

(6x**1**, 3x**2**) **a**

	1	2	3	4	5
1	2	0	0	0	1
2	0	2	1	0	0
3	0	1	2	0	0
4	0	0	0	0	1
5	1	0	0	1	0

(6x**1**, 3x**2**) **b**

	1	2	3	4	5
1	2	0	0	0	1
2	0	2	0	0	0
3	0	0	2	0	1
4	0	0	0	0	1
5	1	0	1	1	0

(6x**1**, 1x**2**, 2x**2**^{1}) **a**

	1	2	3	4	5
1	0	0	0	0	1
2	0	2	0	0	0
3	0	0	0	2^{1}	1
4	0	0	2^{1}	0	1
5	1	0	1	1	0

(6x**1**, 1x**2**, 2x**2**^{1}) **b**

	1	2	3	4	5
1	0	1	0	0	0
2	1	2	0	0	0
3	0	0	0	2^{1}	1
4	0	0	2^{1}	0	1
5	0	0	1	1	0

(6x**1**, 1x**2**, 2x**2**^{1}) **c**

	1	2	3	4	5
1	0	0	0	0	1
2	0	2	0	0	1
3	0	0	0	2^{1}	0
4	0	0	2^{1}	0	1
5	1	1	0	1	0

(6x**1**, 1x**2**, 2x**2**^{1}) **d**

	1	2	3	4	5
1	0	1	0	0	2^{1}
2	1	2	0	0	0
3	0	0	0	1	0
4	0	0	1	0	1
5	2^{1}	0	0	1	0

(6x**1**, 1x**2**, 2x**2**^{1}) **e**

	1	2	3	4	5
1	0	1	0	0	1
2	1	2	0	0	0
3	0	0	0	1	0
4	0	0	1	0	2^{1}
5	1	0	0	2^{1}	0

(6x**1**, 1x**2**, 2x$\mathbf{2}^2$) **a**

	1	2	3	4	5
1	0	0	0	0	1
2	0	2	0	0	0
3	0	0	0	2^2	1
4	0	0	2^2	0	1
5	1	0	1	1	0

(6x**1**, 1x**2**, 2x$\mathbf{2}^2$) **b**

	1	2	3	4	5
1	0	1	0	0	0
2	1	2	0	0	0
3	0	0	0	2^2	1
4	0	0	2^2	0	1
5	0	0	1	1	0

(6x**1**, 1x**2**, 2x$\mathbf{2}^2$) **c**

	1	2	3	4	5
1	0	0	0	0	1
2	0	2	0	0	1
3	0	0	0	2^2	0
4	0	0	2^2	0	1
5	1	1	0	1	0

(6x**1**, 1x**2**, 2x$\mathbf{2}^2$) **d**

	1	2	3	4	5
1	0	1	0	0	2^2
2	1	2	0	0	0
3	0	0	0	1	0
4	0	0	1	0	1
5	2^2	0	0	1	0

(6x**1**, 1x**2**, 2x$\mathbf{2}^2$) **e**

	1	2	3	4	5
1	0	1	0	0	1
2	1	2	0	0	0
3	0	0	0	1	0
4	0	0	1	0	2^2
5	1	0	0	2^2	0

(4x**1**, 4x**2**)

	1	2	3	4	5
1	2	0	0	0	1
2	0	2	1	0	0
3	0	1	2	0	0
4	0	0	0	2	0
5	1	0	0	0	0

(4x**1**, 2x**2**, 2x$\mathbf{2}^1$) **a**

	1	2	3	4	5
1	0	1	0	0	0
2	1	2	0	0	0
3	0	0	2	1	0
4	0	0	1	0	2^1
5	0	0	0	2^1	0

(4x**1**, 2x**2**, 2x$\mathbf{2}^1$) **b**

	1	2	3	4	5
1	2	1	0	0	0
2	1	2	0	0	0
3	0	0	0	1	0
4	0	0	1	0	2^1
5	0	0	0	2^1	0

(4x**1**, 2x**2**, 2x$\mathbf{2}^1$) **c**

	1	2	3	4	5
1	0	0	0	0	1
2	0	2	0	0	0
3	0	0	2	1	0
4	0	0	1	0	2^1
5	1	0	0	2^1	0

(4x**1**, 2x**2**, 2x$\mathbf{2}^2$) **a**

	1	2	3	4	5
1	0	1	0	0	0
2	1	2	0	0	0
3	0	0	2	1	0
4	0	0	1	0	2^2
5	0	0	0	2^2	0

(4x**1**, 2x**2**, 2x$\mathbf{2}^2$) **b**

	1	2	3	4	5
1	2	1	0	0	0
2	1	2	0	0	0
3	0	0	0	1	0
4	0	0	1	0	2^2
5	0	0	0	2^2	0

(4x**1**, 2x**2**, 2x$\mathbf{2}^2$) **c**

	1	2	3	4	5
1	0	0	0	0	1
2	0	2	0	0	0
3	0	0	2	1	0
4	0	0	1	0	2^2
5	1	0	0	2^2	0

(4x**1**, 4x$\mathbf{2}^1$)

	1	2	3	4	5
1	0	2^1	0	0	1
2	2^1	0	0	0	0
3	0	0	0	1	0
4	0	0	1	0	2^1
5	1	0	0	2^1	0

(4x**1**, 2x$\mathbf{2}^1$, 2x$\mathbf{2}^2$) **a**

	1	2	3	4	5
1	0	2^2	0	0	1
2	2^2	0	0	0	0
3	0	0	0	1	0
4	0	0	1	0	2^1
5	1	0	0	2^1	0

(4x**1**, 2x$\mathbf{2}^1$, 2x$\mathbf{2}^2$) **b**

	1	2	3	4	5
1	0	2^1	0	0	1
2	2^1	0	0	0	0
3	0	0	0	1	0
4	0	0	1	0	2^2
5	1	0	0	2^2	0

(4x**1**, 4x$\mathbf{2}^2$)

	1	2	3	4	5
1	0	2^2	0	0	1
2	2^2	0	0	0	0
3	0	0	0	1	0
4	0	0	1	0	2^2
5	1	0	0	2^2	0

$^{1}V_{1}{}^{2}V_{1}{}^{3}V_{5}$

NG

$^{1}V_{1}{}^{2}V_{2}{}^{3}V_{1}$

(8x**1**)

	1	2	3	4
1	0	1	1	1
2	1	0	1	0
3	1	1	0	0
4	1	0	0	0

(6x**1**, 1x**2**)

	1	2	3	4
1	2	1	0	0
2	1	0	1	0
3	0	1	0	1
4	0	0	1	0

(4x**1**, 2x**2**)

	1	2	3	4
1	2	1	0	0
2	1	0	1	0
3	0	1	0	0
4	0	0	0	2

(4x**1**, 2x$\mathbf{2}^1$)

	1	2	3	4
1	0	2^1	0	0
2	2^1	0	1	0
3	0	1	0	1
4	0	0	1	0

(4x**1**, 2x$\mathbf{2}^2$)

	1	2	3	4
1	0	2^2	0	0
2	2^2	0	1	0
3	0	1	0	1
4	0	0	1	0

(2x**1**, 3x**2**)

	1	2	3	4
1	2	1	0	0
2	1	0	0	0
3	0	0	2	0
4	0	0	0	2

(2x**1**, 1x**2**, 2x$\mathbf{2}^1$) **a**

	1	2	3	4
1	0	2^1	0	0
2	2^1	0	1	0
3	0	1	0	0
4	0	0	0	2

(2x**1**, 1x**2**, 2x$\mathbf{2}^1$) **b**

	1	2	3	4
1	0	2^1	0	0
2	2^1	0	0	0
3	0	0	0	1
4	0	0	1	2

(2x**1**, 1x**2**, 2x$\mathbf{2}^2$) **a**

	1	2	3	4
1	0	2^2	0	0
2	2^2	0	1	0
3	0	1	0	0
4	0	0	0	2

(2x**1**, 1x**2**, 2x$\mathbf{2}^2$) **b**

	1	2	3	4
1	0	2^2	0	0
2	2^2	0	0	0
3	0	0	0	1
4	0	0	1	2

$^{1}V_{1}{}^{2}V_{2}{}^{3}V_{3}$

(14x**1**) **a**

	1	2	3	4	5	6
1	0	1	0	1	0	0
2	1	0	1	0	1	0
3	0	1	0	1	0	0
4	1	0	1	0	1	0
5	0	1	0	1	0	1
6	0	0	0	0	1	0

(14x**1**) **b**

	1	2	3	4	5	6
1	0	0	0	1	1	1
2	0	0	1	0	0	0
3	0	1	0	1	1	0
4	1	0	1	0	0	0
5	1	0	1	0	0	1
6	1	0	0	0	1	0

(14x**1**) **c**

	1	2	3	4	5	6
1	0	0	0	1	1	1
2	0	0	0	0	0	1
3	0	0	0	1	1	0
4	1	0	1	0	0	0
5	1	0	1	0	0	1
6	1	1	0	0	1	0

(14x**1**) **d**

	1	2	3	4	5	6
1	0	0	0	1	1	1
2	0	0	0	0	0	1
3	0	0	0	1	1	0
4	1	0	1	0	1	0
5	1	0	1	1	0	0
6	1	1	0	0	0	0

(12x**1**, 1x**2**) **a**

	1	2	3	4	5	6
1	0	0	0	1	1	1
2	0	2	0	0	1	0
3	0	0	0	1	1	0
4	1	0	1	0	0	0
5	1	1	1	0	0	0
6	1	0	0	0	0	0

(12x**1**, 1x**2**) **b**

	1	2	3	4	5	6
1	0	0	0	1	1	1
2	0	0	1	0	0	0
3	0	1	0	1	1	0
4	1	0	1	0	0	0
5	1	0	1	0	0	0
6	1	0	0	0	0	2

(12x**1**, 1x**2**) **c**

	1	2	3	4	5	6
1	0	0	1	1	1	0
2	0	0	0	0	0	1
3	1	0	0	1	1	0
4	1	0	1	0	0	0
5	1	0	1	0	0	0
6	0	1	0	0	0	2

(12x**1**, 1x**2**) **d**

	1	2	3	4	5	6
1	0	0	1	1	1	0
2	0	0	0	0	1	0
3	1	0	0	1	0	0
4	1	0	1	0	0	0
5	1	1	0	0	0	1
6	0	0	0	0	1	2

(12x**1**, 1x**2**) **e**

	1	2	3	4	5	6
1	0	0	1	1	1	0
2	0	0	1	0	0	0
3	1	1	0	1	0	0
4	1	0	1	0	0	0
5	1	0	0	0	0	1
6	0	0	0	0	1	2

(12x**1**, 1x**2**) **f**

	1	2	3	4	5	6
1	0	0	1	1	1	0
2	0	2	1	0	0	0
3	1	1	0	1	0	0
4	1	0	1	0	0	0
5	1	0	0	0	0	1
6	0	0	0	0	1	0

(12x**1**, 1x**2**) **g**

	1	2	3	4	5	6
1	0	0	1	1	1	0
2	0	2	0	0	0	0
3	1	0	0	1	1	0
4	1	0	1	0	0	0
5	1	0	1	0	0	1
6	0	0	0	0	1	0

(10x**1**, 2x**2**) **a**

	1	2	3	4	5	6
1	0	0	1	1	1	0
2	0	0	0	0	0	1
3	1	0	0	1	0	0
4	1	0	1	0	0	0
5	1	0	0	0	2	0
6	0	1	0	0	0	2

(10x**1**, 2x**2**) **b**

	1	2	3	4	5	6
1	0	0	1	1	1	0
2	0	2	0	0	0	1
3	1	0	0	1	0	0
4	1	0	1	0	0	0
5	1	0	0	0	0	0
6	0	1	0	0	0	2

(10x**1**, 2x**2**) **c**

	1	2	3	4	5	6
1	0	0	1	1	0	1
2	0	2	0	0	0	0
3	1	0	0	1	1	0
4	1	0	1	0	0	0
5	0	0	1	0	0	0
6	1	0	0	0	0	2

(10x**1**, 2x**2**) **d**

	1	2	3	4	5	6
1	0	0	0	1	1	1
2	0	2	0	0	1	0
3	0	0	0	1	0	0
4	1	0	1	0	0	0
5	1	1	0	0	0	0
6	1	0	0	0	0	2

(10x**1**, 2x**2**) **e**

	1	2	3	4	5	6
1	0	0	0	1	1	1
2	0	2	0	0	1	0
3	0	0	2	1	0	0
4	1	0	1	0	0	0
5	1	1	0	0	0	0
6	1	0	0	0	0	0

(10x**1**, 2x**2**) **f**

	1	2	3	4	5	6
1	0	0	0	1	1	0
2	0	2	0	0	1	0
3	0	0	0	1	0	0
4	1	0	1	0	0	0
5	1	1	0	0	0	1
6	0	0	0	0	1	2

(10x**1**, 2x**2**) **g**

	1	2	3	4	5	6
1	0	0	0	1	1	0
2	0	2	0	0	1	0
3	0	0	2	1	0	0
4	1	0	1	0	0	0
5	1	1	0	0	0	1
6	0	0	0	0	1	0

(10x**1**, 2x$\mathbf{2}^{1}$) **a**

	1	2	3	4	5	6
1	0	0	1	1	0	1
2	0	0	0	0	0	2^1
3	1	0	0	1	1	0
4	1	0	1	0	0	0
5	0	0	1	0	0	0
6	1	2^1	0	0	0	0

(10x**1**, 2x$\mathbf{2}^{1}$) **b**

	1	2	3	4	5	6
1	0	0	1	1	0	1
2	0	0	0	0	1	1
3	1	0	0	2^1	0	0
4	1	0	2^1	0	0	0
5	0	1	0	0	0	0
6	1	1	0	0	0	0

(10x**1**, 2x$\mathbf{2}^{1}$) **c**

	1	2	3	4	5	6
1	0	0	1	1	0	1
2	0	0	0	0	1	2^1
3	1	0	0	1	0	0
4	1	0	1	0	0	0
5	0	1	0	0	0	0
6	1	2^1	0	0	0	0

(10x**1**, 2x$\mathbf{2}^{1}$) **d**

	1	2	3	4	5	6
1	0	0	0	1	1	1
2	0	0	0	0	0	1
3	0	0	0	2^1	1	0
4	1	0	2^1	0	0	0
5	1	0	1	0	0	0
6	1	1	0	0	0	0

(10x**1**, 2x$\mathbf{2}^{1}$) **e**

	1	2	3	4	5	6
1	0	0	0	1	0	1
2	0	0	0	0	0	1
3	0	0	0	2^1	1	0
4	1	0	2^1	0	0	0
5	0	0	1	0	0	1
6	1	1	0	0	1	0

(10x**1**, 2x$\mathbf{2}^{1}$) **f**

	1	2	3	4	5	6
1	0	0	0	1	0	1
2	0	0	0	0	0	1
3	0	0	0	1	2^1	0
4	1	0	1	0	0	0
5	0	0	2^1	0	0	1
6	1	1	0	0	1	0

(10x**1**, 2x$\mathbf{2}^{2}$) **a**

	1	2	3	4	5	6
1	0	0	1	1	0	1
2	0	0	0	0	0	2^2
3	1	0	0	1	1	0
4	1	0	1	0	0	0
5	0	0	1	0	0	0
6	1	2^2	0	0	0	0

(10x**1**, 2x$\mathbf{2}^{2}$) **b**

	1	2	3	4	5	6
1	0	0	1	1	0	1
2	0	0	0	0	1	1
3	1	0	0	2^2	0	0
4	1	0	2^2	0	0	0
5	0	1	0	0	0	0
6	1	1	0	0	0	0

(10x**1**, 2x$\mathbf{2}^{2}$) **c**

	1	2	3	4	5	6
1	0	0	1	1	0	1
2	0	0	0	0	1	2^2
3	1	0	0	1	0	0
4	1	0	1	0	0	0
5	0	1	0	0	0	0
6	1	2^2	0	0	0	0

(10x**1**, 2x$\mathbf{2}^{2}$) **d**

	1	2	3	4	5	6
1	0	0	0	1	1	1
2	0	0	0	0	0	1
3	0	0	0	2^2	1	0
4	1	0	2^2	0	0	0
5	1	0	1	0	0	0
6	1	1	0	0	0	0

(10x**1**, 2x$\mathbf{2}^{2}$) **e**

	1	2	3	4	5	6
1	0	0	0	1	0	1
2	0	0	0	0	0	1
3	0	0	0	2^2	1	0
4	1	0	2^2	0	0	0
5	0	0	1	0	0	1
6	1	1	0	0	1	0

(10x**1**, 2x$\mathbf{2}^{2}$) **f**

	1	2	3	4	5	6
1	0	0	0	1	0	1
2	0	0	0	0	0	1
3	0	0	0	1	2^2	0
4	1	0	1	0	0	0
5	0	0	2^2	0	0	1
6	1	1	0	0	1	0

(8x**1**, 3x**2**) **a**

	1	2	3	4	5	6
1	0	0	0	1	1	0
2	0	0	0	0	0	1
3	0	0	2	1	0	0
4	1	0	1	0	0	0
5	1	0	0	0	2	0
6	0	1	0	0	0	2

(8x**1**, 3x**2**) **b**

	1	2	3	4	5	6
1	0	0	0	1	0	0
2	0	0	0	0	1	1
3	0	0	2	1	0	0
4	1	0	1	0	0	0
5	0	1	0	0	2	0
6	0	1	0	0	0	2

(8x**1**, 3x**2**) **c**

	1	2	3	4	5	6
1	0	0	0	1	1	0
2	0	2	0	0	0	1
3	0	0	2	1	0	0
4	1	0	1	0	0	0
5	1	0	0	0	0	0
6	0	1	0	0	0	2

(8x**1**, 3x**2**) **d**

	1	2	3	4	5	6
1	0	0	0	1	0	1
2	0	2	0	0	0	0
3	0	0	2	1	0	0
4	1	0	1	0	1	0
5	0	0	0	1	2	0
6	1	0	0	0	0	0

(8x**1**, 3x**2**) **e**

	1	2	3	4	5	6
1	0	0	0	1	0	1
2	0	2	0	0	0	0
3	0	0	2	1	0	0
4	1	0	1	0	1	0
5	0	0	0	1	0	0
6	1	0	0	0	0	2

(8x**1**, 1x**2**, 2x**2**^{1}) **a**

	1	2	3	4	5	6
1	0	0	0	1	1	1
2	0	2	0	0	0	0
3	0	0	0	2^{1}	1	0
4	1	0	2^{1}	0	0	0
5	1	0	1	0	0	0
6	1	0	0	0	0	0

(8x**1**, 1x**2**, 2x**2**^{1}) **b**

	1	2	3	4	5	6
1	0	0	0	1	1	0
2	0	2	0	0	0	1
3	0	0	0	2^{1}	1	0
4	1	0	2^{1}	0	0	0
5	1	0	1	0	0	0
6	0	1	0	0	0	0

(8x**1**, 1x**2**, 2x**2**^{1}) **c**

	1	2	3	4	5	6
1	0	0	1	1	0	1
2	0	0	0	0	0	1
3	1	0	0	2^{1}	0	0
4	1	0	2^{1}	0	0	0
5	0	0	0	0	2	0
6	1	1	0	0	0	0

(8x**1**, 1x**2**, 2x**2**^{1}) **d**

	1	2	3	4	5	6
1	0	0	1	1	0	0
2	0	0	0	0	1	1
3	1	0	0	2^{1}	0	0
4	1	0	2^{1}	0	0	0
5	0	1	0	0	2	0
6	0	1	0	0	0	0

(8x**1**, 1x**2**, 2x**2**^{1}) **e**

	1	2	3	4	5	6
1	0	0	0	1	0	1
2	0	2	0	0	0	1
3	0	0	0	2^{1}	1	0
4	1	0	2^{1}	0	0	0
5	0	0	1	0	0	0
6	1	1	0	0	0	0

(8x**1**, 1x**2**, 2x**2**^{1}) **f**

	1	2	3	4	5	6
1	0	0	0	2^{1}	0	1
2	0	2	0	0	0	1
3	0	0	0	1	1	0
4	2^{1}	0	1	0	0	0
5	0	0	1	0	0	0
6	1	1	0	0	0	0

(8x**1**, 1x**2**, 2x**2**^{1}) **g**

	1	2	3	4	5	6
1	0	0	0	1	0	2^{1}
2	0	2	0	0	0	1
3	0	0	0	1	1	0
4	1	0	1	0	0	0
5	0	0	1	0	0	0
6	2^{1}	1	0	0	0	0

(8x**1**, 1x**2**, 2x**2**^{1}) **h**

	1	2	3	4	5	6
1	0	0	0	1	0	2^{1}
2	0	2	1	0	0	0
3	0	1	0	1	1	0
4	1	0	1	0	0	0
5	0	0	1	0	0	0
6	2^{1}	0	0	0	0	0

(8x**1**, 1x**2**, 2x**2**^{1}) **i**

	1	2	3	4	5	6
1	0	0	0	1	0	2^{1}
2	0	2	1	0	0	0
3	0	1	0	1	0	0
4	1	0	1	0	1	0
5	0	0	0	1	0	0
6	2^{1}	0	0	0	0	0

(8x**1**, 1x**2**, 2x**2**^{1}) **j**

	1	2	3	4	5	6
1	0	0	0	1	0	2^{1}
2	0	0	1	0	0	0
3	0	1	0	1	0	0
4	1	0	1	0	1	0
5	0	0	0	1	2	0
6	2^{1}	0	0	0	0	0

(8x**1**, 1x**2**, 2x**2**^{2}) **a**

	1	2	3	4	5	6
1	0	0	0	1	1	1
2	0	2	0	0	0	0
3	0	0	0	2^{2}	1	0
4	1	0	2^{2}	0	0	0
5	1	0	1	0	0	0
6	1	0	0	0	0	0

(8x**1**, 1x**2**, 2x**2**^{2}) **b**

	1	2	3	4	5	6
1	0	0	0	1	1	0
2	0	2	0	0	0	1
3	0	0	0	2^{2}	1	0
4	1	0	2^{2}	0	0	0
5	1	0	1	0	0	0
6	0	1	0	0	0	0

(8x**1**, 1x**2**, 2x**2**^{2}) **c**

	1	2	3	4	5	6
1	0	0	1	1	0	1
2	0	0	0	0	0	1
3	1	0	0	2^{2}	0	0
4	1	0	2^{2}	0	0	0
5	0	0	0	0	2	0
6	1	1	0	0	0	0

(8x**1**, 1x**2**, 2x**2**^{2}) **d**

	1	2	3	4	5	6
1	0	0	1	1	0	0
2	0	0	0	0	1	1
3	1	0	0	2^{2}	0	0
4	1	0	2^{2}	0	0	0
5	0	1	0	0	2	0
6	0	1	0	0	0	0

(8x**1**, 1x**2**, 2x**2**^{2}) **e**

	1	2	3	4	5	6
1	0	0	0	1	0	1
2	0	2	0	0	0	1
3	0	0	0	2^{2}	1	0
4	1	0	2^{2}	0	0	0
5	0	0	1	0	0	0
6	1	1	0	0	0	0

(8x**1**, 1x**2**, 2x**2**^{2}) **f**

	1	2	3	4	5	6
1	0	0	0	2^{2}	0	1
2	0	2	0	0	0	1
3	0	0	0	1	1	0
4	2^{2}	0	1	0	0	0
5	0	0	1	0	0	0
6	1	1	0	0	0	0

(8x**1**, 1x**2**, 2x**2**^{2}) **g**

	1	2	3	4	5	6
1	0	0	0	1	0	2^{2}
2	0	2	0	0	0	1
3	0	0	0	1	1	0
4	1	0	1	0	0	0
5	0	0	1	0	0	0
6	2^{2}	1	0	0	0	0

(8x**1**, 1x**2**, 2x**2**^{2}) **h**

	1	2	3	4	5	6
1	0	0	0	1	0	2^{2}
2	0	2	1	0	0	0
3	0	1	0	1	1	0
4	1	0	1	0	0	0
5	0	0	1	0	0	0
6	2^{2}	0	0	0	0	0

(8x**1**, 1x**2**, 2x**2**^{2}) **i**

	1	2	3	4	5	6
1	0	0	0	1	0	2^{2}
2	0	2	1	0	0	0
3	0	1	0	1	0	0
4	1	0	1	0	1	0
5	0	0	0	1	0	0
6	2^{2}	0	0	0	0	0

(8x**1**, 1x**2**, 2x**2**^{2}) **j**

	1	2	3	4	5	6
1	0	0	0	1	0	2^{2}
2	0	0	1	0	0	0
3	0	1	0	1	0	0
4	1	0	1	0	1	0
5	0	0	0	1	2	0
6	2^{2}	0	0	0	0	0

(6x**1**, 4x**2**) **a**

	1	2	3	4	5	6
1	0	0	0	1	0	0
2	0	2	0	0	0	1
3	0	0	2	1	0	0
4	1	0	1	0	0	0
5	0	0	0	0	2	0
6	0	1	0	0	0	2

(6x**1**, 4x**2**) **b**

	1	2	3	4	5	6
1	2	0	0	1	0	0
2	0	2	0	0	0	1
3	0	0	2	1	0	0
4	1	0	1	0	0	0
5	0	0	0	0	2	0
6	0	1	0	0	0	0

(6x**1**, 4x**2**) **c**

	1	2	3	4	5	6
1	2	0	0	1	0	0
2	0	2	0	0	0	0
3	0	0	2	1	0	0
4	1	0	1	0	0	1
5	0	0	0	0	2	0
6	0	0	0	1	0	0

(6x**1**, 2x**2**, 2x**2**^{1}) **a**

	1	2	3	4	5	6
1	0	0	1	1	0	1
2	0	2	0	0	0	0
3	1	0	0	2^{1}	0	0
4	1	0	2^{1}	0	0	0
5	0	0	0	0	2	0
6	1	0	0	0	0	0

(6x**1**, 2x**2**, 2x**2**^{1}) **b**

	1	2	3	4	5	6
1	0	0	1	1	0	0
2	0	2	0	0	0	1
3	1	0	0	2^{1}	0	0
4	1	0	2^{1}	0	0	0
5	0	0	0	0	2	0
6	0	1	0	0	0	0

(6x**1**, 2x**2**, 2x**2**^{1}) **c**

	1	2	3	4	5	6
1	2	0	0	1	0	0
2	0	2	0	0	0	1
3	0	0	0	1	2^{1}	0
4	1	0	1	0	0	0
5	0	0	2^{1}	0	0	0
6	0	1	0	0	0	0

(6x**1**, 2x**2**, 2x**2**^{1}) **d**

	1	2	3	4	5	6
1	0	0	0	1	0	0
2	0	2	0	0	0	1
3	0	0	0	1	2^{1}	0
4	1	0	1	0	0	0
5	0	0	2^{1}	0	0	0
6	0	1	0	0	0	2

(6x**1**, 2x**2**, 2x**2**^{1}) **e**

	1	2	3	4	5	6
1	0	0	0	1	0	1
2	0	2	0	0	0	0
3	0	0	0	2^{1}	1	0
4	1	0	2^{1}	0	0	0
5	0	0	1	0	0	0
6	1	0	0	0	0	2

(6x**1**, 2x**2**, 2x**2**^{1}) **f**

	1	2	3	4	5	6
1	0	0	0	2^{1}	0	1
2	0	2	0	0	0	0
3	0	0	0	1	1	0
4	2^{1}	0	1	0	0	0
5	0	0	1	0	0	0
6	1	0	0	0	0	2

(6x**1**, 2x**2**, 2x**2**^{1}) **g**

	1	2	3	4	5	6
1	2	0	0	0	0	1
2	0	2	0	0	0	1
3	0	0	0	1	2^{1}	0
4	0	0	1	0	0	0
5	0	0	2^{1}	0	0	0
6	1	1	0	0	0	0

(6x**1**, 2x**2**, 2x$\mathbf{2}^1$) **h**

	1	2	3	4	5	6
1	0	0	0	0	0	1
2	0	2	0	0	0	1
3	0	0	0	1	2^1	0
4	0	0	1	2	0	0
5	0	0	2^1	0	0	0
6	1	1	0	0	0	0

(6x**1**, 2x**2**, 2x$\mathbf{2}^1$) **i**

	1	2	3	4	5	6
1	2	0	0	1	0	0
2	0	2	0	0	0	0
3	0	0	0	1	2^1	0
4	1	0	1	0	0	1
5	0	0	2^1	0	0	0
6	0	0	0	1	0	0

(6x**1**, 2x**2**, 2x$\mathbf{2}^1$) **j**

	1	2	3	4	5	6
1	2	0	0	1	0	0
2	0	0	0	1	0	0
3	0	0	2	1	0	0
4	1	1	1	0	0	0
5	0	0	0	0	0	2^1
6	0	0	0	0	2^1	0

(6x**1**, 2x**2**, 2x$\mathbf{2}^2$) **a**

	1	2	3	4	5	6
1	0	0	1	1	0	1
2	0	2	0	0	0	0
3	1	0	0	2^2	0	0
4	1	0	2^2	0	0	0
5	0	0	0	0	2	0
6	1	0	0	0	0	0

(6x**1**, 2x**2**, 2x$\mathbf{2}^2$) **b**

	1	2	3	4	5	6
1	0	0	1	1	0	0
2	0	2	0	0	0	1
3	1	0	0	2^2	0	0
4	1	0	2^2	0	0	0
5	0	0	0	0	2	0
6	0	1	0	0	0	0

(6x**1**, 2x**2**, 2x$\mathbf{2}^2$) **c**

	1	2	3	4	5	6
1	2	0	0	1	0	0
2	0	2	0	0	0	1
3	0	0	0	1	2^2	0
4	1	0	1	0	0	0
5	0	0	2^2	0	0	0
6	0	1	0	0	0	0

(6x**1**, 2x**2**, 2x$\mathbf{2}^2$) **d**

	1	2	3	4	5	6
1	0	0	0	1	0	0
2	0	2	0	0	0	1
3	0	0	0	1	2^2	0
4	1	0	1	0	0	0
5	0	0	2^2	0	0	0
6	0	1	0	0	0	2

(6x**1**, 2x**2**, 2x$\mathbf{2}^2$) **e**

	1	2	3	4	5	6
1	0	0	0	1	0	1
2	0	2	0	0	0	0
3	0	0	0	2^2	1	0
4	1	0	2^2	0	0	0
5	0	0	1	0	0	0
6	1	0	0	0	0	2

(6x**1**, 2x**2**, 2x$\mathbf{2}^2$) **f**

	1	2	3	4	5	6
1	0	0	0	2^2	0	1
2	0	2	0	0	0	0
3	0	0	0	1	1	0
4	2^2	0	1	0	0	0
5	0	0	1	0	0	0
6	1	0	0	0	0	2

(6x**1**, 2x**2**, 2x$\mathbf{2}^2$) **g**

	1	2	3	4	5	6
1	2	0	0	0	0	1
2	0	2	0	0	0	1
3	0	0	0	1	2^2	0
4	0	0	1	0	0	0
5	0	0	2^2	0	0	0
6	1	1	0	0	0	0

(6x**1**, 2x**2**, 2x$\mathbf{2}^2$) **h**

	1	2	3	4	5	6
1	0	0	0	0	0	1
2	0	2	0	0	0	1
3	0	0	0	1	2^2	0
4	0	0	1	2	0	0
5	0	0	2^2	0	0	0
6	1	1	0	0	0	0

(6x**1**, 2x**2**, 2x$\mathbf{2}^2$) **i**

	1	2	3	4	5	6
1	2	0	0	1	0	0
2	0	2	0	0	0	0
3	0	0	0	1	2^2	0
4	1	0	1	0	0	1
5	0	0	2^2	0	0	0
6	0	0	0	1	0	0

(6x**1**, 2x**2**, 2x$\mathbf{2}^2$) **j**

	1	2	3	4	5	6
1	2	0	0	1	0	0
2	0	0	0	1	0	0
3	0	0	2	1	0	0
4	1	1	1	0	0	0
5	0	0	0	0	0	2^2
6	0	0	0	0	2^2	0

(6x**1**, 4x$\mathbf{2}^1$) **a**

	1	2	3	4	5	6
1	0	0	0	1	0	2^1
2	0	0	2^1	0	0	0
3	0	2^1	0	1	0	0
4	1	0	1	0	1	0
5	0	0	0	1	0	0
6	2^1	0	0	0	0	0

(6x**1**, 4x$\mathbf{2}^1$) **b**

	1	2	3	4	5	6
1	0	0	0	2^1	0	1
2	0	0	0	0	2^1	0
3	0	0	0	1	1	0
4	2^1	0	1	0	0	0
5	0	2^1	1	0	0	0
6	1	0	0	0	0	0

(6x**1**, 4x$\mathbf{2}^1$) **c**

	1	2	3	4	5	6
1	0	0	0	1	0	1
2	0	0	0	0	2^1	0
3	0	0	0	2^1	1	0
4	1	0	2^1	0	0	0
5	0	2^1	1	0	0	0
6	1	0	0	0	0	0

(6x**1**, 4x$\mathbf{2}^1$) **d**

	1	2	3	4	5	6
1	0	0	1	1	0	1
2	0	0	0	0	2^1	0
3	1	0	0	2^1	0	0
4	1	0	2^1	0	0	0
5	0	2^1	0	0	0	0
6	1	0	0	0	0	0

(6x**1**, 4x$\mathbf{2}^1$) **e**

	1	2	3	4	5	6
1	0	0	1	1	0	0
2	0	0	0	0	2^1	0
3	1	0	0	2^1	0	0
4	1	0	2^1	0	0	0
5	0	2^1	0	0	0	1
6	0	0	0	0	1	0

(6x**1**, 2x$\mathbf{2}^1$, 2x$\mathbf{2}^2$) **a**

	1	2	3	4	5	6
1	0	0	0	1	0	2^1
2	0	0	2^2	0	0	0
3	0	2^2	0	1	0	0
4	1	0	1	0	1	0
5	0	0	0	1	0	0
6	2^1	0	0	0	0	0

(6x**1**, 2x$\mathbf{2}^1$, 2x$\mathbf{2}^2$) **b**

	1	2	3	4	5	6
1	0	0	0	2^1	0	1
2	0	0	0	0	2^2	0
3	0	0	0	1	1	0
4	2^1	0	1	0	0	0
5	0	2^2	1	0	0	0
6	1	0	0	0	0	0

(6x**1**, 2x$\mathbf{2}^1$, 2x$\mathbf{2}^2$) **c**

	1	2	3	4	5	6
1	0	0	0	2^2	0	1
2	0	0	0	0	2^1	0
3	0	0	0	1	1	0
4	2^2	0	1	0	0	0
5	0	2^1	1	0	0	0
6	1	0	0	0	0	0

(6x**1**, 2x$\mathbf{2}^1$, 2x$\mathbf{2}^2$) **d**

	1	2	3	4	5	6
1	0	0	0	1	0	1
2	0	0	0	0	2^1	0
3	0	0	0	2^2	1	0
4	1	0	2^2	0	0	0
5	0	2^1	1	0	0	0
6	1	0	0	0	0	0

(6x**1**, 2x$\mathbf{2}^1$, 2x$\mathbf{2}^2$) **e**

	1	2	3	4	5	6
1	0	0	0	1	0	1
2	0	0	0	0	2^2	0
3	0	0	0	2^1	1	0
4	1	0	2^1	0	0	0
5	0	2^2	1	0	0	0
6	1	0	0	0	0	0

(6x**1**, 2x$\mathbf{2}^1$, 2x$\mathbf{2}^2$) **f**

	1	2	3	4	5	6
1	0	0	1	1	0	1
2	0	0	0	0	2^1	0
3	1	0	0	2^2	0	0
4	1	0	2^2	0	0	0
5	0	2^1	0	0	0	0
6	1	0	0	0	0	0

(6x**1**, 2x$\mathbf{2}^1$, 2x$\mathbf{2}^2$) **g**

	1	2	3	4	5	6
1	0	0	1	1	0	1
2	0	0	0	0	2^2	0
3	1	0	0	2^1	0	0
4	1	0	2^1	0	0	0
5	0	2^2	0	0	0	0
6	1	0	0	0	0	0

(6x**1**, 2x$\mathbf{2}^1$, 2x$\mathbf{2}^2$) **h**

	1	2	3	4	5	6
1	0	0	1	1	0	0
2	0	0	0	0	2^1	0
3	1	0	0	2^2	0	0
4	1	0	2^2	0	0	0
5	0	2^1	0	0	0	1
6	0	0	0	0	1	0

(6x**1**, 2x$\mathbf{2}^1$, 2x$\mathbf{2}^2$) **i**

	1	2	3	4	5	6
1	0	0	1	1	0	0
2	0	0	0	0	2^2	0
3	1	0	0	2^1	0	0
4	1	0	2^1	0	0	0
5	0	2^2	0	0	0	1
6	0	0	0	0	1	0

(6x**1**, 4x$\mathbf{2}^2$) **a**

	1	2	3	4	5	6
1	0	0	0	1	0	2^2
2	0	0	2^2	0	0	0
3	0	2^2	0	1	0	0
4	1	0	1	0	1	0
5	0	0	0	1	0	0
6	2^2	0	0	0	0	0

(6x**1**, 4x$\mathbf{2}^2$) **b**

	1	2	3	4	5	6
1	0	0	0	2^2	0	1
2	0	0	0	0	2^2	0
3	0	0	0	1	1	0
4	2^2	0	1	0	0	0
5	0	2^2	1	0	0	0
6	1	0	0	0	0	0

(6x**1**, 4x$\mathbf{2}^2$) **c**

	1	2	3	4	5	6
1	0	0	0	1	0	1
2	0	0	0	0	2^2	0
3	0	0	0	2^2	1	0
4	1	0	2^2	0	0	0
5	0	2^2	1	0	0	0
6	1	0	0	0	0	0

(6x**1**, 4x**2**^{2}) **d**

	1	2	3	4	5	6
1	0	0	1	1	0	1
2	0	0	0	0	2^2	0
3	1	0	0	2^2	0	0
4	1	0	2^2	0	0	0
5	0	2^2	0	0	0	0
6	1	0	0	0	0	0

(6x**1**, 4x**2**^{2}) **e**

	1	2	3	4	5	6
1	0	0	1	1	0	0
2	0	0	0	0	2^2	0
3	1	0	0	2^2	0	0
4	1	0	2^2	0	0	0
5	0	2^2	0	0	0	1
6	0	0	0	0	1	0

(4x**1**, 5x**2**)

	1	2	3	4	5	6
1	2	0	0	0	0	0
2	0	2	0	0	0	1
3	0	0	2	1	0	0
4	0	0	1	0	0	0
5	0	0	0	0	2	0
6	0	1	0	0	0	2

(4x**1**, 3x**2**, 2x**2**^{1}) **a**

	1	2	3	4	5	6
1	0	0	0	1	2^1	0
2	0	2	0	0	0	1
3	0	0	2	0	0	0
4	1	0	0	0	0	0
5	2^1	0	0	0	0	0
6	0	1	0	0	0	2

(4x**1**, 3x**2**, 2x**2**^{1}) **b**

	1	2	3	4	5	6
1	0	0	0	1	2^1	0
2	0	2	0	0	0	1
3	0	0	2	0	0	0
4	1	0	0	2	0	0
5	2^1	0	0	0	0	0
6	0	1	0	0	0	0

(4x**1**, 3x**2**, 2x**2**^{1}) **c**

	1	2	3	4	5	6
1	0	0	0	0	2^1	0
2	0	2	0	0	0	1
3	0	0	2	1	0	0
4	0	0	1	0	0	0
5	2^1	0	0	0	0	0
6	0	1	0	0	0	2

(4x**1**, 3x**2**, 2x**2**^{1}) **d**

	1	2	3	4	5	6
1	0	0	0	1	2^1	0
2	0	2	0	0	0	0
3	0	0	2	0	0	0
4	1	0	0	2	0	0
5	2^1	0	0	0	0	1
6	0	0	0	0	1	0

(4x**1**, 3x**2**, 2x**2**^{2}) **a**

	1	2	3	4	5	6
1	0	0	0	1	2^2	0
2	0	2	0	0	0	1
3	0	0	2	0	0	0
4	1	0	0	0	0	0
5	2^2	0	0	0	0	0
6	0	1	0	0	0	2

(4x**1**, 3x**2**, 2x**2**^{2}) **b**

	1	2	3	4	5	6
1	0	0	0	1	2^2	0
2	0	2	0	0	0	1
3	0	0	2	0	0	0
4	1	0	0	2	0	0
5	2^2	0	0	0	0	0
6	0	1	0	0	0	0

(4x**1**, 3x**2**, 2x**2**^{2}) **c**

	1	2	3	4	5	6
1	0	0	0	0	2^2	0
2	0	2	0	0	0	1
3	0	0	2	1	0	0
4	0	0	1	0	0	0
5	2^2	0	0	0	0	0
6	0	1	0	0	0	2

(4x**1**, 3x**2**, 2x**2**^{2}) **d**

	1	2	3	4	5	6
1	0	0	0	1	2^2	0
2	0	2	0	0	0	0
3	0	0	2	0	0	0
4	1	0	0	2	0	0
5	2^2	0	0	0	0	1
6	0	0	0	0	1	0

(4x**1**, 1x**2**, 4x**2**^{1}) **a**

	1	2	3	4	5	6
1	0	0	0	0	0	2^1
2	0	0	0	0	0	1
3	0	0	0	1	2^1	0
4	0	0	1	2	0	0
5	0	0	2^1	0	0	0
6	2^1	1	0	0	0	0

(4x**1**, 1x**2**, 4x**2**^{1}) **b**

	1	2	3	4	5	6
1	0	0	0	0	0	2^1
2	0	0	0	0	1	0
3	0	0	0	1	2^1	0
4	0	0	1	2	0	0
5	0	1	2^1	0	0	0
6	2^1	0	0	0	0	0

(4x**1**, 1x**2**, 4x**2**^{1}) **c**

	1	2	3	4	5	6
1	2	0	0	0	0	1
2	0	0	0	0	2^1	0
3	0	0	0	2^1	1	0
4	0	0	2^1	0	0	0
5	0	2^1	1	0	0	0
6	1	0	0	0	0	0

(4x**1**, 1x**2**, 4x**2**^{1}) **d**

	1	2	3	4	5	6
1	2	0	0	0	0	0
2	0	0	0	0	2^1	1
3	0	0	0	2^1	1	0
4	0	0	2^1	0	0	0
5	0	2^1	1	0	0	0
6	0	1	0	0	0	0

(4x**1**, 1x**2**, 2x**2**^{1}, 2x**2**^{2}) **a**

	1	2	3	4	5	6
1	0	0	0	0	0	2^1
2	0	0	0	0	0	1
3	0	0	0	1	2^2	0
4	0	0	1	2	0	0
5	0	0	2^2	0	0	0
6	2^1	1	0	0	0	0

(4x**1**, 1x**2**, 2x**2**^{1}, 2x**2**^{2}) **b**

	1	2	3	4	5	6
1	0	0	0	0	0	2^2
2	0	0	0	0	0	1
3	0	0	0	1	2^1	0
4	0	0	1	2	0	0
5	0	0	2^1	0	0	0
6	2^2	1	0	0	0	0

(4x**1**, 1x**2**, 2x**2**^{1}, 2x**2**^{2}) **c**

	1	2	3	4	5	6
1	0	0	0	0	0	2^1
2	0	0	0	0	1	0
3	0	0	0	1	2^2	0
4	0	0	1	2	0	0
5	0	1	2^2	0	0	0
6	2^1	0	0	0	0	0

(4x**1**, 1x**2**, 2x**2**^{1}, 2x**2**^{2}) **d**

	1	2	3	4	5	6
1	0	0	0	0	0	2^2
2	0	0	0	0	1	0
3	0	0	0	1	2^1	0
4	0	0	1	2	0	0
5	0	1	2^1	0	0	0
6	2^2	0	0	0	0	0

(4x**1**, 1x**2**, 2x**2**^{1}, 2x**2**^{2}) **e**

	1	2	3	4	5	6
1	2	0	0	0	0	1
2	0	0	0	0	2^2	0
3	0	0	0	2^1	1	0
4	0	0	2^1	0	0	0
5	0	2^2	1	0	0	0
6	1	0	0	0	0	0

(4x**1**, 1x**2**, 2x**2**^{1}, 2x**2**^{2}) **f**

	1	2	3	4	5	6
1	2	0	0	0	0	0
2	0	0	0	0	2^2	1
3	0	0	0	2^1	1	0
4	0	0	2^1	0	0	0
5	0	2^2	1	0	0	0
6	0	1	0	0	0	0

(4x**1**, 1x**2**, 2x**2**^{1}, 2x**2**^{2}) **g**

	1	2	3	4	5	6
1	2	0	0	0	0	0
2	0	0	0	0	2^1	1
3	0	0	0	2^2	1	0
4	0	0	2^2	0	0	0
5	0	2^1	1	0	0	0
6	0	1	0	0	0	0

(4x**1**, 1x**2**, 4x**2**^{2}) **a**

	1	2	3	4	5	6
1	0	0	0	0	0	2^2
2	0	0	0	0	0	1
3	0	0	0	1	2^2	0
4	0	0	1	2	0	0
5	0	0	2^2	0	0	0
6	2^2	1	0	0	0	0

(4x**1**, 1x**2**, 4x**2**^{2}) **b**

	1	2	3	4	5	6
1	0	0	0	0	0	2^2
2	0	0	0	0	1	0
3	0	0	0	1	2^2	0
4	0	0	1	2	0	0
5	0	1	2^2	0	0	0
6	2^2	0	0	0	0	0

(4x**1**, 1x**2**, 4x**2**^{2}) **c**

	1	2	3	4	5	6
1	2	0	0	0	0	1
2	0	0	0	0	2^2	0
3	0	0	0	2^2	1	0
4	0	0	2^2	0	0	0
5	0	2^2	1	0	0	0
6	1	0	0	0	0	0

(4x**1**, 1x**2**, 4x**2**^{2}) **d**

	1	2	3	4	5	6
1	2	0	0	0	0	0
2	0	0	0	0	2^2	1
3	0	0	0	2^2	1	0
4	0	0	2^2	0	0	0
5	0	2^2	1	0	0	0
6	0	1	0	0	0	0

$^1V_1{}^2V_2{}^3V_5$	NG

$^1V_1{}^2V_3{}^3V_1$

(10x**1**) **a**

	1	2	3	4	5
1	0	1	0	1	1
2	1	0	0	0	0
3	0	0	0	1	1
4	1	0	1	0	0
5	1	0	1	0	0

(10x**1**) **b**

	1	2	3	4	5
1	0	1	1	1	0
2	1	0	0	0	1
3	1	0	0	1	0
4	1	0	1	0	0
5	0	1	0	0	0

(8x**1**, 1x**2**) **a**

	1	2	3	4	5
1	0	1	1	1	0
2	1	0	0	0	0
3	1	0	0	1	0
4	1	0	1	0	0
5	0	0	0	0	2

(8x**1**, 1x**2**) **b**

	1	2	3	4	5
1	0	0	1	1	0
2	0	0	0	0	1
3	1	0	0	1	0
4	1	0	1	0	0
5	0	1	0	0	2

(8x**1**, 1x**2**) **c**

	1	2	3	4	5
1	0	1	0	1	0
2	1	0	0	0	1
3	0	0	0	1	0
4	1	0	1	0	0
5	0	1	0	0	2

(6x**1**, 2x**2**)

	1	2	3	4	5
1	0	1	0	1	0
2	1	0	0	0	0
3	0	0	2	1	0
4	1	0	1	0	0
5	0	0	0	0	2

(6x**1**, 2x$\mathbf{2^1}$)

	1	2	3	4	5
1	0	1	0	1	0
2	1	0	0	0	2^1
3	0	0	0	1	0
4	1	0	1	0	0
5	0	2^1	0	0	0

(6x**1**, 2x$\mathbf{2^2}$)

	1	2	3	4	5
1	0	1	0	1	0
2	1	0	0	0	2^2
3	0	0	0	1	0
4	1	0	1	0	0
5	0	2^2	0	0	0

(4x**1**, 3x**2**)

	1	2	3	4	5
1	0	0	0	1	0
2	0	2	0	0	0
3	0	0	2	1	0
4	1	0	1	0	0
5	0	0	0	0	2

(4x**1**, 1x**2**, 2x$\mathbf{2^1}$) **a**

	1	2	3	4	5
1	0	0	0	1	0
2	0	0	0	0	2^1
3	0	0	2	1	0
4	1	0	1	0	0
5	0	2^1	0	0	0

(4x**1**, 1x**2**, 2x$\mathbf{2^1}$) **b**

	1	2	3	4	5
1	0	1	0	1	0
2	1	0	0	0	2^1
3	0	0	2	0	0
4	1	0	0	0	0
5	0	2^1	0	0	0

(4x**1**, 1x**2**, 2x$\mathbf{2^2}$) **a**

	1	2	3	4	5
1	0	0	0	1	0
2	0	0	0	0	2^2
3	0	0	2	1	0
4	1	0	1	0	0
5	0	2^2	0	0	0

(4x**1**, 1x**2**, 2x$\mathbf{2^2}$) **b**

	1	2	3	4	5
1	0	1	0	1	0
2	1	0	0	0	2^2
3	0	0	2	0	0
4	1	0	0	0	0
5	0	2^2	0	0	0

(2x**1**, 4x**2**)

	1	2	3	4	5
1	2	0	0	0	0
2	0	2	0	0	0
3	0	0	2	1	0
4	0	0	1	0	0
5	0	0	0	0	2

(2x**1**, 2x**2**, 2x$\mathbf{2^1}$) **a**

	1	2	3	4	5
1	2	0	0	0	0
2	0	0	0	0	2^1
3	0	0	2	1	0
4	0	0	1	0	0
5	0	2^1	0	0	0

(2x**1**, 2x**2**, 2x$\mathbf{2^1}$) **b**

	1	2	3	4	5
1	0	1	0	0	0
2	1	0	0	0	2^1
3	0	0	2	0	0
4	0	0	0	2	0
5	0	2^1	0	0	0

(2x**1**, 2x**2**, 2x$\mathbf{2^2}$) **a**

	1	2	3	4	5
1	2	0	0	0	0
2	0	0	0	0	2^2
3	0	0	2	1	0
4	0	0	1	0	0
5	0	2^2	0	0	0

(2x**1**, 2x**2**, 2x$\mathbf{2^2}$) **b**

	1	2	3	4	5
1	0	1	0	0	0
2	1	0	0	0	2^2
3	0	0	2	0	0
4	0	0	0	2	0
5	0	2^2	0	0	0

(2x**1**, 4x$\mathbf{2^1}$)

	1	2	3	4	5
1	0	1	0	0	0
2	1	0	0	0	2^1
3	0	0	0	2^1	0
4	0	0	2^1	0	0
5	0	2^1	0	0	0

(2x**1**, 2x$\mathbf{2^1}$, 2x$\mathbf{2^2}$) **a**

	1	2	3	4	5
1	0	1	0	0	0
2	1	0	0	0	2^2
3	0	0	0	2^1	0
4	0	0	2^1	0	0
5	0	2^2	0	0	0

(2x**1**, 2x$\mathbf{2^1}$, 2x$\mathbf{2^2}$) **b**

	1	2	3	4	5
1	0	1	0	0	0
2	1	0	0	0	2^1
3	0	0	0	2^2	0
4	0	0	2^2	0	0
5	0	2^1	0	0	0

(2x**1**, 4x$\mathbf{2^2}$)

	1	2	3	4	5
1	0	1	0	0	0
2	1	0	0	0	2^2
3	0	0	0	2^2	0
4	0	0	2^2	0	0
5	0	2^2	0	0	0

$^1V_1{}^2V_3{}^3V_3$

NG

$^1V_1{}^2V_4{}^3V_1$

(12x**1**) **a**

	1	2	3	4	5	6
1	0	1	0	1	1	0
2	1	0	0	0	0	1
3	0	0	0	1	1	0
4	1	0	1	0	0	0
5	1	0	1	0	0	0
6	0	1	0	0	0	0

(12x**1**) **b**

	1	2	3	4	5	6
1	0	1	0	1	0	0
2	1	0	0	0	1	1
3	0	0	0	1	1	0
4	1	0	1	0	0	0
5	0	1	1	0	0	0
6	0	1	0	0	0	0

(12x**1**) **c**

	1	2	3	4	5	6
1	0	1	1	1	0	0
2	1	0	0	0	0	1
3	1	0	0	1	0	0
4	1	0	1	0	0	0
5	0	0	0	0	0	1
6	0	1	0	0	1	0

(10x**1**, 1x**2**) **a**

	1	2	3	4	5	6
1	0	1	0	1	1	0
2	1	0	0	0	0	0
3	0	0	0	1	1	0
4	1	0	1	0	0	0
5	1	0	1	0	0	0
6	0	0	0	0	0	2

(10x**1**, 1x**2**) **b**

	1	2	3	4	5	6
1	0	1	1	1	0	0
2	1	0	0	0	1	0
3	1	0	0	1	0	0
4	1	0	1	0	0	0
5	0	1	0	0	0	0
6	0	0	0	0	0	2

(10x**1**, 1x**2**) **c**

	1	2	3	4	5	6
1	0	0	1	1	0	0
2	0	0	0	0	1	0
3	1	0	0	1	0	0
4	1	0	1	0	0	0
5	0	1	0	0	0	1
6	0	0	0	0	1	2

(10x**1**, 1x**2**) **d**

	1	2	3	4	5	6
1	0	0	0	1	1	0
2	0	0	0	0	0	1
3	0	0	0	1	1	0
4	1	0	1	0	0	0
5	1	0	1	0	0	0
6	0	1	0	0	0	2

(10x**1**, 1x**2**) **e**

	1	2	3	4	5	6
1	0	1	0	1	0	0
2	1	0	0	0	0	1
3	0	0	0	1	1	0
4	1	0	1	0	0	0
5	0	0	1	0	0	0
6	0	1	0	0	0	2

(8x**1**, 2x**2**) **a**

	1	2	3	4	5	6
1	0	1	1	1	0	0
2	1	0	0	0	0	0
3	1	0	0	1	0	0
4	1	0	1	0	0	0
5	0	0	0	0	2	0
6	0	0	0	0	0	2

(8x**1**, 2x**2**) **b**

	1	2	3	4	5	6
1	0	0	1	1	0	0
2	0	2	0	0	0	0
3	1	0	0	1	0	0
4	1	0	1	0	0	0
5	0	0	0	0	0	1
6	0	0	0	0	1	2

(8x**1**, 2x**2**) **c**

	1	2	3	4	5	6
1	0	1	0	1	0	0
2	1	0	0	0	0	1
3	0	0	0	1	0	0
4	1	0	1	0	0	0
5	0	0	0	0	2	0
6	0	1	0	0	0	2

(8x**1**, 2x$\mathbf{2}^1$) **a**

	1	2	3	4	5	6
1	0	1	1	1	0	0
2	1	0	0	0	0	0
3	1	0	0	1	0	0
4	1	0	1	0	0	0
5	0	0	0	0	0	2^1
6	0	0	0	0	2^1	0

(8x**1**, 2x$\mathbf{2}^1$) **b**

	1	2	3	4	5	6
1	0	0	1	1	0	0
2	0	0	0	0	0	1
3	1	0	0	1	0	0
4	1	0	1	0	0	0
5	0	0	0	0	0	2^1
6	0	1	0	0	2^1	0

(8x**1**, 2x$\mathbf{2}^1$) **c**

	1	2	3	4	5	6
1	0	1	0	1	0	0
2	1	0	0	0	0	2^1
3	0	0	0	1	1	0
4	1	0	1	0	0	0
5	0	0	1	0	0	0
6	0	2^1	0	0	0	0

(8x**1**, 2x$\mathbf{2}^2$) **a**

	1	2	3	4	5	6
1	0	1	1	1	0	0
2	1	0	0	0	0	0
3	1	0	0	1	0	0
4	1	0	1	0	0	0
5	0	0	0	0	0	2^2
6	0	0	0	0	2^2	0

(8x**1**, 2x$\mathbf{2}^2$) **b**

	1	2	3	4	5	6
1	0	0	1	1	0	0
2	0	0	0	0	0	1
3	1	0	0	1	0	0
4	1	0	1	0	0	0
5	0	0	0	0	0	2^2
6	0	1	0	0	2^2	0

(8x**1**, 2x$\mathbf{2}^2$) **c**

	1	2	3	4	5	6
1	0	1	0	1	0	0
2	1	0	0	0	0	2^2
3	0	0	0	1	1	0
4	1	0	1	0	0	0
5	0	0	1	0	0	0
6	0	2^2	0	0	0	0

(6x**1**, 3x**2**)

	1	2	3	4	5	6
1	0	1	0	1	0	0
2	1	0	0	0	0	1
3	0	0	2	0	0	0
4	1	0	0	0	0	0
5	0	0	0	0	2	0
6	0	1	0	0	0	2

(6x**1**, 1x**2**, 2x$\mathbf{2}^1$) **a**

	1	2	3	4	5	6
1	0	1	0	1	0	0
2	1	0	0	0	0	0
3	0	0	2	1	0	0
4	1	0	1	0	0	0
5	0	0	0	0	0	2^1
6	0	0	0	0	2^1	0

(6x**1**, 1x**2**, 2x$\mathbf{2}^1$) **b**

	1	2	3	4	5	6
1	0	1	0	1	0	0
2	1	0	0	0	0	1
3	0	0	2	0	0	0
4	1	0	0	0	0	0
5	0	0	0	0	0	2^1
6	0	1	0	0	2^1	0

(6x**1**, 1x**2**, 2x$\mathbf{2}^2$) **a**

	1	2	3	4	5	6
1	0	1	0	1	0	0
2	1	0	0	0	0	0
3	0	0	2	1	0	0
4	1	0	1	0	0	0
5	0	0	0	0	0	2^2
6	0	0	0	0	2^2	0

(6x**1**, 1x**2**, 2x$\mathbf{2}^2$) **b**

	1	2	3	4	5	6
1	0	1	0	1	0	0
2	1	0	0	0	0	1
3	0	0	2	0	0	0
4	1	0	0	0	0	0
5	0	0	0	0	0	2^2
6	0	1	0	0	2^2	0

(4x**1**, 4x**2**)

	1	2	3	4	5	6
1	0	1	0	0	0	0
2	1	0	0	0	0	1
3	0	0	2	0	0	0
4	0	0	0	2	0	0
5	0	0	0	0	2	0
6	0	1	0	0	0	2

(4x**1**, 2x**2**, 2x$\mathbf{2}^1$) **a**

	1	2	3	4	5	6
1	0	0	0	1	0	0
2	0	2	0	0	0	0
3	0	0	2	1	0	0
4	1	0	1	0	0	0
5	0	0	0	0	0	2^1
6	0	0	0	0	2^1	0

(4x**1**, 2x**2**, 2x$\mathbf{2}^1$) **b**

	1	2	3	4	5	6
1	0	1	0	0	0	0
2	1	0	0	0	0	1
3	0	0	2	0	0	0
4	0	0	0	2	0	0
5	0	0	0	0	0	2^1
6	0	1	0	0	2^1	0

(4x**1**, 2x**2**, 2x$\mathbf{2}^2$) **a**

	1	2	3	4	5	6
1	0	0	0	1	0	0
2	0	2	0	0	0	0
3	0	0	2	1	0	0
4	1	0	1	0	0	0
5	0	0	0	0	0	2^2
6	0	0	0	0	2^2	0

(4x**1**, 2x**2**, 2x$\mathbf{2}^2$) **b**

	1	2	3	4	5	6
1	0	1	0	0	0	0
2	1	0	0	0	0	1
3	0	0	2	0	0	0
4	0	0	0	2	0	0
5	0	0	0	0	0	2^2
6	0	1	0	0	2^2	0

(4x**1**, 4x$\mathbf{2}^1$)

	1	2	3	4	5	6
1	0	1	0	0	0	0
2	1	0	0	0	0	1
3	0	0	0	2^1	0	0
4	0	0	2^1	0	0	0
5	0	0	0	0	0	2^1
6	0	1	0	0	2^1	0

(4x**1**, 2x$\mathbf{2}^1$, 2x$\mathbf{2}^2$) **a**

	1	2	3	4	5	6
1	0	1	0	0	0	0
2	1	0	0	0	0	1
3	0	0	0	2^2	0	0
4	0	0	2^2	0	0	0
5	0	0	0	0	0	2^1
6	0	1	0	0	2^1	0

(4x**1**, 2x$\mathbf{2}^1$, 2x$\mathbf{2}^2$) **b**

	1	2	3	4	5	6
1	0	1	0	0	0	0
2	1	0	0	0	0	1
3	0	0	0	2^1	0	0
4	0	0	2^1	0	0	0
5	0	0	0	0	0	2^2
6	0	1	0	0	2^2	0

(4x**1**, 4x$\mathbf{2}^2$)

	1	2	3	4	5	6
1	0	1	0	0	0	0
2	1	0	0	0	0	1
3	0	0	0	2^2	0	0
4	0	0	2^2	0	0	0
5	0	0	0	0	0	2^2
6	0	1	0	0	2^2	0

(2x**1**, 5x**2**)

	1	2	3	4	5	6
1	2	0	0	0	0	0
2	0	0	0	0	0	1
3	0	0	2	0	0	0
4	0	0	0	2	0	0
5	0	0	0	0	2	0
6	0	1	0	0	0	2

(2x**1**, 3x**2**, 2x$\mathbf{2}^1$) **a**

	1	2	3	4	5	6
1	2	0	0	0	0	0
2	0	2	0	0	0	0
3	0	0	2	1	0	0
4	0	0	1	0	0	0
5	0	0	0	0	0	2^1
6	0	0	0	0	2^1	0

(2x**1**, 3x**2**, 2x$\mathbf{2}^1$) **b**

	1	2	3	4	5	6
1	2	0	0	0	0	0
2	0	0	0	0	0	1
3	0	0	2	0	0	0
4	0	0	0	2	0	0
5	0	0	0	0	0	2^1
6	0	1	0	0	2^1	0

(2x**1**, 3x**2**, 2x$\mathbf{2}^2$) **a**

	1	2	3	4	5	6
1	2	0	0	0	0	0
2	0	2	0	0	0	0
3	0	0	2	1	0	0
4	0	0	1	0	0	0
5	0	0	0	0	0	2^2
6	0	0	0	0	2^2	0

(2x**1**, 3x**2**, 2x$\mathbf{2}^2$) **b**

	1	2	3	4	5	6
1	2	0	0	0	0	0
2	0	0	0	0	0	1
3	0	0	2	0	0	0
4	0	0	0	2	0	0
5	0	0	0	0	0	2^2
6	0	1	0	0	2^2	0

(2x**1**, 1x**2**, 4x$\mathbf{2}^1$) **a**

	1	2	3	4	5	6
1	0	2^1	0	0	0	0
2	2^1	0	0	0	0	0
3	0	0	2	1	0	0
4	0	0	1	0	0	0
5	0	0	0	0	0	2^1
6	0	0	0	0	2^1	0

(2x**1**, 1x**2**, 4x$\mathbf{2}^1$) **b**

	1	2	3	4	5	6
1	0	2^1	0	1	0	0
2	2^1	0	0	0	0	0
3	0	0	2	0	0	0
4	1	0	0	0	0	0
5	0	0	0	0	0	2^1
6	0	0	0	0	2^1	0

(2x**1**, 1x**2**, 2x$\mathbf{2}^1$, 2x$\mathbf{2}^2$) **a**

	1	2	3	4	5	6
1	0	2^2	0	0	0	0
2	2^2	0	0	0	0	0
3	0	0	2	1	0	0
4	0	0	1	0	0	0
5	0	0	0	0	0	2^1
6	0	0	0	0	2^1	0

(2x**1**, 1x**2**, 2x$\mathbf{2}^1$, 2x$\mathbf{2}^2$) **b**

	1	2	3	4	5	6
1	0	2^2	0	1	0	0
2	2^2	0	0	0	0	0
3	0	0	2	0	0	0
4	1	0	0	0	0	0
5	0	0	0	0	0	2^1
6	0	0	0	0	2^1	0

(2x**1**, 1x**2**, 2x**2^1**, 2x**2^2**) **c**

	1	2	3	4	5	6
1	0	2^1	0	1	0	0
2	2^1	0	0	0	0	0
3	0	0	2	0	0	0
4	1	0	0	0	0	0
5	0	0	0	0	0	2^2
6	0	0	0	0	2^2	0

(2x**1**, 1x**2**, 4x**2^2**) **a**

	1	2	3	4	5	6
1	0	2^2	0	0	0	0
2	2^2	0	0	0	0	0
3	0	0	2	1	0	0
4	0	0	1	0	0	0
5	0	0	0	0	0	2^2
6	0	0	0	0	2^2	0

(2x**1**, 1x**2**, 4x**2^2**) **b**

	1	2	3	4	5	6
1	0	2^2	0	1	0	0
2	2^2	0	0	0	0	0
3	0	0	2	0	0	0
4	1	0	0	0	0	0
5	0	0	0	0	0	2^2
6	0	0	0	0	2^2	0

$^1V_1{}^2V_4{}^3V_3$ NG

$^1V_1{}^2V_5{}^3V_1$ NG

$^1V_1{}^2V_6{}^3V_1$ NG

$^1V_2{}^2V_1{}^3V_2$

(10x**1**)

	1	2	3	4	5
1	0	1	1	1	0
2	1	0	0	0	0
3	1	0	0	1	1
4	1	0	1	0	0
5	0	0	1	0	0

(8x**1**, 1x**2**) **a**

	1	2	3	4	5
1	0	1	1	1	0
2	1	0	0	0	1
3	1	0	2	0	0
4	1	0	0	0	0
5	0	1	0	0	0

(8x**1**, 1x**2**) **b**

	1	2	3	4	5
1	0	1	1	1	0
2	1	0	0	0	1
3	1	0	0	0	0
4	1	0	0	0	0
5	0	1	0	0	2

(6x**1**, 2x**2**) **a**

	1	2	3	4	5
1	0	1	1	1	0
2	1	0	0	0	0
3	1	0	2	0	0
4	1	0	0	0	0
5	0	0	0	0	2

(6x**1**, 2x**2**) **b**

	1	2	3	4	5
1	0	1	1	0	0
2	1	0	0	0	0
3	1	0	2	0	0
4	0	0	0	0	1
5	0	0	0	1	2

(6x**1**, 2x**2**) **c**

	1	2	3	4	5
1	0	1	1	0	0
2	1	0	0	0	0
3	1	0	0	0	0
4	0	0	0	2	1
5	0	0	0	1	2

(6x**1**, 2x**2**) **d**

	1	2	3	4	5
1	0	1	1	0	0
2	1	2	0	0	0
3	1	0	2	0	0
4	0	0	0	0	1
5	0	0	0	1	0

(6x**1**, 2x**2^1**) **a**

	1	2	3	4	5
1	0	0	2^1	1	0
2	0	0	0	0	1
3	2^1	0	0	1	0
4	1	0	1	0	0
5	0	1	0	0	0

(6x**1**, 2x**2^1**) **b**

	1	2	3	4	5
1	0	1	0	1	0
2	1	0	0	0	0
3	0	0	0	2^1	1
4	1	0	2^1	0	0
5	0	0	1	0	0

(6x**1**, 2x**2^1**) **c**

	1	2	3	4	5
1	0	1	0	1	1
2	1	0	0	0	0
3	0	0	0	2^1	0
4	1	0	2^1	0	0
5	1	0	0	0	0

(6x**1**, 2x**2^2**) **a**

	1	2	3	4	5
1	0	0	2^2	1	0
2	0	0	0	0	1
3	2^2	0	0	1	0
4	1	0	1	0	0
5	0	1	0	0	0

(6x**1**, 2x**2^2**) **b**

	1	2	3	4	5
1	0	1	0	1	0
2	1	0	0	0	0
3	0	0	0	2^2	1
4	1	0	2^2	0	0
5	0	0	1	0	0

(6x**1**, 2x**2^2**) **c**

	1	2	3	4	5
1	0	1	0	1	1
2	1	0	0	0	0
3	0	0	0	2^2	0
4	1	0	2^2	0	0
5	1	0	0	0	0

(4x**1**, 3x**2**) **a**

	1	2	3	4	5
1	0	0	1	0	0
2	0	2	0	0	0
3	1	0	0	0	0
4	0	0	0	2	1
5	0	0	0	1	2

(4x**1**, 3x**2**) **b**

	1	2	3	4	5
1	0	0	1	0	0
2	0	2	0	0	0
3	1	0	2	0	0
4	0	0	0	0	1
5	0	0	0	1	2

(4x**1**, 1x**2**, 2x**2^1**) **a**

	1	2	3	4	5
1	0	1	0	0	0
2	1	0	0	0	0
3	0	0	0	2^1	1
4	0	0	2^1	0	0
5	0	0	1	0	2

(4x**1**, 1x**2**, 2x**2^1**) **b**

	1	2	3	4	5
1	2	1	0	0	0
2	1	0	0	0	0
3	0	0	0	2^1	1
4	0	0	2^1	0	0
5	0	0	1	0	0

(4x**1**, 1x**2**, 2x**2^1**) **c**

	1	2	3	4	5
1	2	0	0	0	0
2	0	0	0	1	0
3	0	0	0	2^1	1
4	0	1	2^1	0	0
5	0	0	1	0	0

(4x**1**, 1x**2**, 2x**2^2**) **a**

	1	2	3	4	5
1	0	1	0	0	0
2	1	0	0	0	0
3	0	0	0	2^2	1
4	0	0	2^2	0	0
5	0	0	1	0	2

(4x**1**, 1x**2**, 2x**2^2**) **b**

	1	2	3	4	5
1	2	1	0	0	0
2	1	0	0	0	0
3	0	0	0	2^2	1
4	0	0	2^2	0	0
5	0	0	1	0	0

(4x**1**, 1x**2**, 2x**2^2**) **c**

	1	2	3	4	5
1	2	0	0	0	0
2	0	0	0	1	0
3	0	0	0	2^2	1
4	0	1	2^2	0	0
5	0	0	1	0	0

$^1V_2{}^2V_1{}^3V_4$ NG

$^1V_2{}^2V_2{}^3V_2$

(12x**1**) **a**

	1	2	3	4	5	6
1	0	1	1	1	0	0
2	1	0	0	0	0	1
3	1	0	0	1	1	0
4	1	0	1	0	0	0
5	0	0	1	0	0	0
6	0	1	0	0	0	0

(12x**1**) **b**

	1	2	3	4	5	6
1	0	1	1	1	0	0
2	1	0	0	0	1	1
3	1	0	0	1	0	0
4	1	0	1	0	0	0
5	0	1	0	0	0	0
6	0	1	0	0	0	0

(12x**1**) **c**

	1	2	3	4	5	6
1	0	0	1	1	1	0
2	0	0	0	0	0	1
3	1	0	0	1	1	0
4	1	0	1	0	0	0
5	1	0	1	0	0	0
6	0	1	0	0	0	0

(12x**1**) **d**

	1	2	3	4	5	6
1	0	0	0	1	1	0
2	0	0	1	0	0	0
3	0	1	0	1	1	0
4	1	0	1	0	0	0
5	1	0	1	0	0	1
6	0	0	0	0	1	0

(12x**1**) **e**

	1	2	3	4	5	6
1	0	0	0	1	1	0
2	0	0	0	1	0	0
3	0	0	0	1	1	0
4	1	1	1	0	0	0
5	1	0	1	0	0	1
6	0	0	0	0	1	0

(10x**1**, 1x**2**) **a**

	1	2	3	4	5	6
1	0	1	1	1	0	0
2	1	0	0	0	0	0
3	1	0	0	1	1	0
4	1	0	1	0	0	0
5	0	0	1	0	0	0
6	0	0	0	0	0	2

(10x**1**, 1x**2**) **b**

	1	2	3	4	5	6
1	0	1	1	1	0	0
2	1	0	0	0	0	0
3	1	0	0	1	0	0
4	1	0	1	0	0	0
5	0	0	0	0	0	1
6	0	0	0	0	1	2

(10x**1**, 1x**2**) **c**

	1	2	3	4	5	6
1	0	1	1	1	0	0
2	1	2	0	0	0	0
3	1	0	0	1	0	0
4	1	0	1	0	0	0
5	0	0	0	0	0	1
6	0	0	0	0	1	0

(10x**1**, 1x**2**) **d**

	1	2	3	4	5	6
1	0	1	0	1	0	0
2	1	0	0	0	1	1
3	0	0	2	1	0	0
4	1	0	1	0	0	0
5	0	1	0	0	0	0
6	0	1	0	0	0	0

(10x**1**, 1x**2**) **e**

	1	2	3	4	5	6
1	0	1	0	1	0	0
2	1	0	0	0	1	1
3	0	0	0	1	0	0
4	1	0	1	0	0	0
5	0	1	0	0	2	0
6	0	1	0	0	0	0

(10x**1**, 1x**2**) **f**

	1	2	3	4	5	6
1	0	1	0	1	1	0
2	1	0	0	0	0	1
3	0	0	0	1	0	0
4	1	0	1	0	0	0
5	1	0	0	0	0	0
6	0	1	0	0	0	2

(10x**1**, 1x**2**) **g**

	1	2	3	4	5	6
1	0	1	0	1	1	0
2	1	0	0	0	0	1
3	0	0	0	1	0	0
4	1	0	1	0	0	0
5	1	0	0	0	2	0
6	0	1	0	0	0	0

(8x**1**, 2x**2**) **a**

	1	2	3	4	5	6
1	0	1	0	1	0	0
2	1	0	0	0	1	1
3	0	0	2	0	0	0
4	1	0	0	2	0	0
5	0	1	0	0	0	0
6	0	1	0	0	0	0

(8x**1**, 2x**2**) **b**

	1	2	3	4	5	6
1	0	1	0	1	0	0
2	1	0	0	0	1	1
3	0	0	2	0	0	0
4	1	0	0	0	0	0
5	0	1	0	0	2	0
6	0	1	0	0	0	0

(8x**1**, 2x**2**) **c**

	1	2	3	4	5	6
1	0	1	0	1	0	0
2	1	0	0	0	0	1
3	0	0	0	0	1	0
4	1	0	0	2	0	0
5	0	0	1	0	0	0
6	0	1	0	0	0	2

(8x**1**, 2x**2**) **d**

	1	2	3	4	5	6
1	0	1	0	1	0	0
2	1	0	0	0	0	1
3	0	0	2	0	1	0
4	1	0	0	0	0	0
5	0	0	1	0	0	0
6	0	1	0	0	0	2

(8x**1**, 2x**2**) **e**

	1	2	3	4	5	6
1	0	1	0	1	0	0
2	1	0	0	0	0	1
3	0	0	2	0	1	0
4	1	0	0	0	0	0
5	0	0	1	0	2	0
6	0	1	0	0	0	0

(8x**1**, 2x**2**) **f**

	1	2	3	4	5	6
1	0	1	0	1	0	0
2	1	0	0	0	0	0
3	0	0	0	0	1	0
4	1	0	0	2	0	0
5	0	0	1	0	0	1
6	0	0	0	0	1	2

(8x**1**, 2x**2**) **g**

	1	2	3	4	5	6
1	0	1	0	1	0	0
2	1	2	0	0	0	0
3	0	0	0	0	1	0
4	1	0	0	2	0	0
5	0	0	1	0	0	1
6	0	0	0	0	1	0

(8x**1**, 2x**2**^{1}) **a**

	1	2	3	4	5	6
1	0	1	0	1	0	0
2	1	0	0	0	1	1
3	0	0	0	2^1	0	0
4	1	0	2^1	0	0	0
5	0	1	0	0	0	0
6	0	1	0	0	0	0

(8x**1**, 2x**2**^{1}) **b**

	1	2	3	4	5	6
1	0	0	0	1	1	0
2	0	0	0	0	0	1
3	0	0	0	2^1	1	0
4	1	0	2^1	0	0	0
5	1	0	1	0	0	0
6	0	1	0	0	0	0

(8x**1**, 2x**2**^{1}) **c**

	1	2	3	4	5	6
1	0	0	1	1	0	0
2	0	0	0	0	0	1
3	1	0	0	2^1	0	0
4	1	0	2^1	0	0	0
5	0	0	0	0	0	1
6	0	1	0	0	1	0

(8x**1**, 2x**2**^{1}) **d**

	1	2	3	4	5	6
1	0	1	0	2^1	0	0
2	1	0	0	0	0	1
3	0	0	0	1	0	0
4	2^1	0	1	0	0	0
5	0	0	0	0	0	1
6	0	1	0	0	1	0

(8x**1**, 2x**2**^{1}) **e**

	1	2	3	4	5	6
1	0	2^1	0	1	0	0
2	2^1	0	0	0	0	1
3	0	0	0	1	0	0
4	1	0	1	0	0	0
5	0	0	0	0	0	1
6	0	1	0	0	1	0

(8x**1**, 2x**2**^{1}) **f**

	1	2	3	4	5	6
1	0	1	0	1	1	0
2	1	0	0	0	0	1
3	0	0	0	2^1	0	0
4	1	0	2^1	0	0	0
5	1	0	0	0	0	0
6	0	1	0	0	0	0

(8x**1**, 2x**2**^{2}) **a**

	1	2	3	4	5	6
1	0	1	0	1	1	0
2	1	0	0	0	0	1
3	0	0	0	2^2	0	0
4	1	0	2^2	0	0	0
5	1	0	0	0	0	0
6	0	1	0	0	0	0

(8x**1**, 2x**2**^{2}) **b**

	1	2	3	4	5	6
1	0	0	0	1	1	0
2	0	0	0	0	0	1
3	0	0	0	2^2	1	0
4	1	0	2^2	0	0	0
5	1	0	1	0	0	0
6	0	1	0	0	0	0

(8x**1**, 2x**2**^{2}) **c**

	1	2	3	4	5	6
1	0	0	1	1	0	0
2	0	0	0	0	0	1
3	1	0	0	2^2	0	0
4	1	0	2^2	0	0	0
5	0	0	0	0	0	1
6	0	1	0	0	1	0

(8x**1**, 2x**2**^{2}) **d**

	1	2	3	4	5	6
1	0	1	0	2^2	0	0
2	1	0	0	0	0	1
3	0	0	0	1	0	0
4	2^2	0	1	0	0	0
5	0	0	0	0	0	1
6	0	1	0	0	1	0

(8x**1**, 2x**2**^{2}) **e**

	1	2	3	4	5	6
1	0	2^2	0	1	0	0
2	2^2	0	0	0	0	1
3	0	0	0	1	0	0
4	1	0	1	0	0	0
5	0	0	0	0	0	1
6	0	1	0	0	1	0

(8x**1**, 2x$\mathbf{2}^{2}$) **f**

	1	2	3	4	5	6
1	0	1	0	1	1	0
2	1	0	0	0	0	1
3	0	0	0	2^2	0	0
4	1	0	2^2	0	0	0
5	1	0	0	0	0	0
6	0	1	0	0	0	0

(6x**1**, 3x**2**) **a**

	1	2	3	4	5	6
1	0	1	0	0	0	0
2	1	0	0	0	0	1
3	0	0	2	1	0	0
4	0	0	1	2	0	0
5	0	0	0	0	2	0
6	0	1	0	0	0	0

(6x**1**, 3x**2**) **b**

	1	2	3	4	5	6
1	2	1	0	0	0	0
2	1	0	0	0	0	1
3	0	0	0	1	0	0
4	0	0	1	0	0	0
5	0	0	0	0	2	0
6	0	1	0	0	0	2

(6x**1**, 3x**2**) **c**

	1	2	3	4	5	6
1	0	1	0	0	0	0
2	1	0	0	0	0	1
3	0	0	0	1	0	0
4	0	0	1	2	0	0
5	0	0	0	0	2	0
6	0	1	0	0	0	2

(6x**1**, 3x**2**) **d**

	1	2	3	4	5	6
1	0	1	0	0	0	0
2	1	0	0	1	0	1
3	0	0	2	0	0	0
4	0	1	0	2	0	0
5	0	0	0	0	2	0
6	0	1	0	0	0	0

(6x**1**, 1x**2**, 2x$\mathbf{2}^{1}$) **a**

	1	2	3	4	5	6
1	0	1	0	1	0	0
2	1	0	0	0	0	0
3	0	0	0	2^1	0	0
4	1	0	2^1	0	0	0
5	0	0	0	0	0	1
6	0	0	0	0	1	2

(6x**1**, 1x**2**, 2x$\mathbf{2}^{1}$) **b**

	1	2	3	4	5	6
1	0	1	0	1	0	0
2	1	2	0	0	0	0
3	0	0	0	2^1	0	0
4	1	0	2^1	0	0	0
5	0	0	0	0	0	1
6	0	0	0	0	1	0

(6x**1**, 1x**2**, 2x$\mathbf{2}^{1}$) **c**

	1	2	3	4	5	6
1	2	0	0	1	0	0
2	0	0	0	0	0	1
3	0	0	0	2^1	0	0
4	1	0	2^1	0	0	0
5	0	0	0	0	0	1
6	0	1	0	0	1	0

(6x**1**, 1x**2**, 2x$\mathbf{2}^{1}$) **d**

	1	2	3	4	5	6
1	0	0	0	1	0	0
2	0	0	0	0	0	1
3	0	0	0	2^1	0	0
4	1	0	2^1	0	0	0
5	0	0	0	0	2	1
6	0	1	0	0	1	0

(6x**1**, 1x**2**, 2x$\mathbf{2}^{1}$) **e**

	1	2	3	4	5	6
1	0	1	0	2^1	0	0
2	1	0	0	0	0	1
3	0	0	0	1	0	0
4	2^1	0	1	0	0	0
5	0	0	0	0	2	0
6	0	1	0	0	0	0

(6x**1**, 1x**2**, 2x$\mathbf{2}^{1}$) **f**

	1	2	3	4	5	6
1	0	1	0	1	0	1
2	1	0	0	0	0	0
3	0	0	0	2^1	0	0
4	1	0	2^1	0	0	0
5	0	0	0	0	2	0
6	1	0	0	0	0	0

(6x**1**, 1x**2**, 2x$\mathbf{2}^{1}$) **g**

	1	2	3	4	5	6
1	0	1	0	0	1	1
2	1	0	0	0	0	0
3	0	0	0	2^1	0	0
4	0	0	2^1	0	0	0
5	1	0	0	0	0	0
6	1	0	0	0	0	2

(6x**1**, 1x**2**, 2x$\mathbf{2}^{1}$) **h**

	1	2	3	4	5	6
1	0	0	1	1	0	0
2	0	2	0	0	0	0
3	1	0	0	2^1	0	0
4	1	0	2^1	0	0	0
5	0	0	0	0	0	1
6	0	0	0	0	1	0

(6x**1**, 1x**2**, 2x$\mathbf{2}^{2}$) **a**

	1	2	3	4	5	6
1	0	1	0	1	0	0
2	1	0	0	0	0	0
3	0	0	0	2^2	0	0
4	1	0	2^2	0	0	0
5	0	0	0	0	0	1
6	0	0	0	0	1	2

(6x**1**, 1x**2**, 2x$\mathbf{2}^{2}$) **b**

	1	2	3	4	5	6
1	0	1	0	1	0	0
2	1	2	0	0	0	0
3	0	0	0	2^2	0	0
4	1	0	2^2	0	0	0
5	0	0	0	0	0	1
6	0	0	0	0	1	0

(6x**1**, 1x**2**, 2x$\mathbf{2}^{2}$) **c**

	1	2	3	4	5	6
1	2	0	0	1	0	0
2	0	0	0	0	0	1
3	0	0	0	2^2	0	0
4	1	0	2^2	0	0	0
5	0	0	0	0	0	1
6	0	1	0	0	1	0

(6x**1**, 1x**2**, 2x$\mathbf{2}^{2}$) **d**

	1	2	3	4	5	6
1	0	0	0	1	0	0
2	0	0	0	0	0	1
3	0	0	0	2^2	0	0
4	1	0	2^2	0	0	0
5	0	0	0	0	2	1
6	0	1	0	0	1	0

(6x**1**, 1x**2**, 2x$\mathbf{2}^{2}$) **e**

	1	2	3	4	5	6
1	0	1	0	2^2	0	0
2	1	0	0	0	0	1
3	0	0	0	1	0	0
4	2^2	0	1	0	0	0
5	0	0	0	0	2	0
6	0	1	0	0	0	0

(6x**1**, 1x**2**, 2x$\mathbf{2}^{2}$) **f**

	1	2	3	4	5	6
1	0	1	0	1	0	1
2	1	0	0	0	0	0
3	0	0	0	2^2	0	0
4	1	0	2^2	0	0	0
5	0	0	0	0	2	0
6	1	0	0	0	0	0

(6x**1**, 1x**2**, 2x$\mathbf{2}^{2}$) **g**

	1	2	3	4	5	6
1	0	1	0	0	1	1
2	1	0	0	0	0	0
3	0	0	0	2^2	0	0
4	0	0	2^2	0	0	0
5	1	0	0	0	0	0
6	1	0	0	0	0	2

(6x**1**, 1x**2**, 2x$\mathbf{2}^{2}$) **h**

	1	2	3	4	5	6
1	0	0	1	1	0	0
2	0	2	0	0	0	0
3	1	0	0	2^2	0	0
4	1	0	2^2	0	0	0
5	0	0	0	0	0	1
6	0	0	0	0	1	0

(4x**1**, 4x**2**) **a**

	1	2	3	4	5	6
1	2	0	0	0	0	0
2	0	0	0	0	0	1
3	0	0	2	1	0	0
4	0	0	1	2	0	0
5	0	0	0	0	2	0
6	0	1	0	0	0	0

(4x**1**, 4x**2**) **b**

	1	2	3	4	5	6
1	2	0	0	0	0	0
2	0	0	0	0	0	1
3	0	0	0	1	0	0
4	0	0	1	2	0	0
5	0	0	0	0	2	0
6	0	1	0	0	0	2

(4x**1**, 2x**2**, 2x$\mathbf{2}^{1}$) **a**

	1	2	3	4	5	6
1	0	0	0	0	2^1	0
2	0	0	0	0	0	1
3	0	0	2	1	0	0
4	0	0	1	2	0	0
5	2^1	0	0	0	0	0
6	0	1	0	0	0	0

(4x**1**, 2x**2**, 2x$\mathbf{2}^{1}$) **b**

	1	2	3	4	5	6
1	0	0	0	0	2^1	0
2	0	0	0	0	0	1
3	0	0	0	1	0	0
4	0	0	1	2	0	0
5	2^1	0	0	0	0	0
6	0	1	0	0	0	2

(4x**1**, 2x**2**, 2x$\mathbf{2}^{1}$) **c**

	1	2	3	4	5	6
1	0	0	0	0	2^1	0
2	0	2	0	0	0	0
3	0	0	0	1	0	0
4	0	0	1	0	0	0
5	2^1	0	0	0	0	1
6	0	0	0	0	1	2

(4x**1**, 2x**2**, 2x$\mathbf{2}^{1}$) **d**

	1	2	3	4	5	6
1	0	0	0	0	2^1	0
2	0	2	0	0	0	0
3	0	0	2	1	0	0
4	0	0	1	0	0	0
5	2^1	0	0	0	0	1
6	0	0	0	0	1	0

(4x**1**, 2x**2**, 2x$\mathbf{2}^{1}$) **e**

	1	2	3	4	5	6
1	0	1	0	0	2^1	0
2	1	0	0	0	0	0
3	0	0	2	0	0	0
4	0	0	0	2	0	0
5	2^1	0	0	0	0	1
6	0	0	0	0	1	0

(4x**1**, 2x**2**, 2x$\mathbf{2}^{2}$) **a**

	1	2	3	4	5	6
1	0	0	0	0	2^2	0
2	0	0	0	0	0	1
3	0	0	2	1	0	0
4	0	0	1	2	0	0
5	2^2	0	0	0	0	0
6	0	1	0	0	0	0

(4x**1**, 2x**2**, 2x$\mathbf{2}^{2}$) **b**

	1	2	3	4	5	6
1	0	0	0	0	2^2	0
2	0	0	0	0	0	1
3	0	0	0	1	0	0
4	0	0	1	2	0	0
5	2^2	0	0	0	0	0
6	0	1	0	0	0	2

(4x**1**, 2x**2**, 2x$\mathbf{2^2}$) **c**

	1	2	3	4	5	6
1	0	0	0	0	2^2	0
2	0	2	0	0	0	0
3	0	0	0	1	0	0
4	0	0	1	0	0	0
5	2^2	0	0	0	0	1
6	0	0	0	0	1	2

(4x**1**, 2x**2**, 2x$\mathbf{2^2}$) **d**

	1	2	3	4	5	6
1	0	0	0	0	2^2	0
2	0	2	0	0	0	0
3	0	0	2	1	0	0
4	0	0	1	0	0	0
5	2^2	0	0	0	0	1
6	0	0	0	0	1	0

(4x**1**, 2x**2**, 2x$\mathbf{2^2}$) **e**

	1	2	3	4	5	6
1	0	1	0	0	2^2	0
2	1	0	0	0	0	0
3	0	0	2	0	0	0
4	0	0	0	2	0	0
5	2^2	0	0	0	0	1
6	0	0	0	0	1	0

(4x**1**, 4x$\mathbf{2^1}$) **a**

	1	2	3	4	5	6
1	0	0	0	0	2^1	0
2	0	0	2^1	1	0	0
3	0	2^1	0	0	0	0
4	0	1	0	0	0	0
5	2^1	0	0	0	0	1
6	0	0	0	0	1	0

(4x**1**, 4x$\mathbf{2^1}$) **b**

	1	2	3	4	5	6
1	0	0	0	0	1	0
2	0	0	2^1	1	0	0
3	0	2^1	0	0	0	0
4	0	1	0	0	0	2^1
5	1	0	0	0	0	0
6	0	0	0	2^1	0	0

(4x**1**, 4x$\mathbf{2^1}$) **c**

	1	2	3	4	5	6
1	0	0	0	0	2^1	0
2	0	0	1	2^1	0	0
3	0	1	0	0	0	0
4	0	2^1	0	0	0	0
5	2^1	0	0	0	0	1
6	0	0	0	0	1	0

(4x**1**, 2x$\mathbf{2^1}$, 2x$\mathbf{2^2}$) **a**

	1	2	3	4	5	6
1	0	0	0	0	2^2	0
2	0	0	2^1	1	0	0
3	0	2^1	0	0	0	0
4	0	1	0	0	0	0
5	2^2	0	0	0	0	1
6	0	0	0	0	1	0

(4x**1**, 2x$\mathbf{2^1}$, 2x$\mathbf{2^2}$) **b**

	1	2	3	4	5	6
1	0	0	0	0	1	0
2	0	0	2^1	1	0	0
3	0	2^1	0	0	0	0
4	0	1	0	0	0	2^2
5	1	0	0	0	0	0
6	0	0	0	2^2	0	0

(4x**1**, 2x$\mathbf{2^1}$, 2x$\mathbf{2^2}$) **c**

	1	2	3	4	5	6
1	0	0	0	0	2^1	0
2	0	0	1	2^2	0	0
3	0	1	0	0	0	0
4	0	2^2	0	0	0	0
5	2^1	0	0	0	0	1
6	0	0	0	0	1	0

(4x**1**, 2x$\mathbf{2^1}$, 2x$\mathbf{2^2}$) **d**

	1	2	3	4	5	6
1	0	0	0	0	2^2	0
2	0	0	1	2^1	0	0
3	0	1	0	0	0	0
4	0	2^1	0	0	0	0
5	2^2	0	0	0	0	1
6	0	0	0	0	1	0

(4x**1**, 4x$\mathbf{2^2}$) **a**

	1	2	3	4	5	6
1	0	0	0	0	2^2	0
2	0	0	2^2	1	0	0
3	0	2^2	0	0	0	0
4	0	1	0	0	0	0
5	2^2	0	0	0	0	1
6	0	0	0	0	1	0

(4x**1**, 4x$\mathbf{2^2}$) **b**

	1	2	3	4	5	6
1	0	0	0	0	1	0
2	0	0	2^2	1	0	0
3	0	2^2	0	0	0	0
4	0	1	0	0	0	2^2
5	1	0	0	0	0	0
6	0	0	0	2^2	0	0

(4x**1**, 4x$\mathbf{2^2}$) **c**

	1	2	3	4	5	6
1	0	0	0	0	2^2	0
2	0	0	1	2^2	0	0
3	0	1	0	0	0	0
4	0	2^2	0	0	0	0
5	2^2	0	0	0	0	1
6	0	0	0	0	1	0

$^1V_2{}^2V_2{}^3V_4$ NG

$^1V_2{}^2V_3{}^3V_2$ NG

$^1V_2{}^2V_4{}^3V_2$ NG

$^1V_3{}^2V_1{}^3V_1$

(8x**1**)

	1	2	3	4	5
1	0	1	1	1	0
2	1	0	0	0	1
3	1	0	0	0	0
4	1	0	0	0	0
5	0	1	0	0	0

(6x**1**, 1x**2**) **a**

	1	2	3	4	5
1	0	0	0	1	0
2	0	2	0	0	1
3	0	0	0	0	1
4	1	0	0	0	0
5	0	1	1	0	0

(6x**1**, 1x**2**) **b**

	1	2	3	4	5
1	2	0	0	1	0
2	0	0	0	0	1
3	0	0	0	0	1
4	1	0	0	0	0
5	0	1	1	0	0

(6x**1**, 1x**2**) **c**

	1	2	3	4	5
1	2	0	0	0	0
2	0	0	0	0	1
3	0	0	0	0	1
4	0	0	0	0	1
5	0	1	1	1	0

(4x**1**, 2x**2**)

	1	2	3	4	5
1	0	0	0	1	0
2	0	2	0	0	1
3	0	0	2	0	0
4	1	0	0	0	0
5	0	1	0	0	0

(4x**1**, 2x$\mathbf{2^1}$)

	1	2	3	4	5
1	0	1	0	0	0
2	1	0	0	0	0
3	0	0	0	2^1	1
4	0	0	2^1	0	0
5	0	0	1	0	0

(4x**1**, 2x$\mathbf{2^2}$)

	1	2	3	4	5
1	0	1	0	0	0
2	1	0	0	0	0
3	0	0	0	2^2	1
4	0	0	2^2	0	0
5	0	0	1	0	0

$^1V_3{}^2V_1{}^3V_3$ NG

$^1V_3{}^2V_2{}^3V_1$

(10x**1**) **a**

	1	2	3	4	5	6
1	0	1	1	1	0	0
2	1	0	1	0	0	0
3	1	1	0	0	0	0
4	1	0	0	0	0	0
5	0	0	0	0	0	1
6	0	0	0	0	1	0

(10x**1**) **b**

	1	2	3	4	5	6
1	0	1	0	0	0	0
2	1	0	1	1	0	0
3	0	1	0	0	0	1
4	0	1	0	0	0	0
5	0	0	0	0	0	1
6	0	0	1	0	1	0

(10x**1**) **c**

	1	2	3	4	5	6
1	0	1	0	0	0	0
2	1	0	1	0	0	0
3	0	1	0	1	0	1
4	0	0	1	0	0	0
5	0	0	0	0	0	1
6	0	0	1	0	1	0

(8x**1**, 1x**2**) **a**

	1	2	3	4	5	6
1	0	1	0	0	0	0
2	1	0	1	1	0	0
3	0	1	0	0	0	1
4	0	1	0	0	0	0
5	0	0	0	0	2	0
6	0	0	1	0	0	0

(8x**1**, 1x**2**) **b**

	1	2	3	4	5	6
1	0	1	0	1	0	0
2	1	0	1	0	0	0
3	0	1	0	0	0	0
4	1	0	0	0	0	0
5	0	0	0	0	0	1
6	0	0	0	0	1	2

(8x**1**, 1x**2**) **c**

	1	2	3	4	5	6
1	0	1	0	1	0	0
2	1	0	1	0	0	0
3	0	1	2	0	0	0
4	1	0	0	0	0	0
5	0	0	0	0	0	1
6	0	0	0	0	1	0

(8x**1**, 1x**2**) **d**

	1	2	3	4	5	6
1	0	1	0	1	0	0
2	1	0	0	0	0	0
3	0	0	2	0	0	1
4	1	0	0	0	0	0
5	0	0	0	0	0	1
6	0	0	1	0	1	0

(6x**1**, 2x**2**) **a**

	1	2	3	4	5	6
1	0	1	0	0	0	0
2	1	0	0	0	0	0
3	0	0	2	0	0	1
4	0	0	0	2	0	0
5	0	0	0	0	0	1
6	0	0	1	0	1	0

(6x**1**, 2x**2**) **b**

	1	2	3	4	5	6
1	0	1	0	0	0	0
2	1	2	0	0	0	0
3	0	0	0	0	0	1
4	0	0	0	2	0	0
5	0	0	0	0	0	1
6	0	0	1	0	1	0

(6x**1**, 2x**2**) **c**

	1	2	3	4	5	6
1	0	0	0	0	0	1
2	0	2	0	0	0	0
3	0	0	0	0	0	1
4	0	0	0	2	0	0
5	0	0	0	0	0	1
6	1	0	1	0	1	0

(6x**1**, 2x**2**^{1}) **a**

	1	2	3	4	5	6
1	0	1	0	0	0	0
2	1	0	0	0	0	0
3	0	0	0	2^1	0	1
4	0	0	2^1	0	0	0
5	0	0	0	0	0	1
6	0	0	1	0	1	0

(6x**1**, 2x**2**^{1}) **b**

	1	2	3	4	5	6
1	0	1	0	0	0	0
2	1	0	0	0	1	0
3	0	0	0	2^1	0	1
4	0	0	2^1	0	0	0
5	0	1	0	0	0	0
6	0	0	1	0	0	0

(6x**1**, 2x**2**^{1}) **c**

	1	2	3	4	5	6
1	0	0	0	2^1	0	0
2	0	0	0	0	0	1
3	0	0	0	0	0	1
4	2^1	0	0	0	0	0
5	0	0	0	0	0	1
6	0	1	1	0	1	0

(6x**1**, 2x**2**^{2}) **a**

	1	2	3	4	5	6
1	0	1	0	0	0	0
2	1	0	0	0	0	0
3	0	0	0	2^2	0	1
4	0	0	2^2	0	0	0
5	0	0	0	0	0	1
6	0	0	1	0	1	0

(6x**1**, 2x**2**^{2}) **b**

	1	2	3	4	5	6
1	0	1	0	0	0	0
2	1	0	0	0	1	0
3	0	0	0	2^2	0	1
4	0	0	2^2	0	0	0
5	0	1	0	0	0	0
6	0	0	1	0	0	0

(6x**1**, 2x**2**^{2}) **c**

	1	2	3	4	5	6
1	0	0	0	2^2	0	0
2	0	0	0	0	0	1
3	0	0	0	0	0	1
4	2^2	0	0	0	0	0
5	0	0	0	0	0	1
6	0	1	1	0	1	0

(4x**1**, 3x**2**)

	1	2	3	4	5	6
1	0	1	0	0	0	0
2	1	2	0	0	0	0
3	0	0	2	0	0	0
4	0	0	0	2	0	0
5	0	0	0	0	0	1
6	0	0	0	0	1	0

(4x**1**, 1x**2**, 2x**2**^{1}) **a**

	1	2	3	4	5	6
1	0	1	0	0	0	0
2	1	0	0	0	0	0
3	0	0	0	2^1	0	1
4	0	0	2^1	0	0	0
5	0	0	0	0	2	0
6	0	0	1	0	0	0

(4x**1**, 1x**2**, 2x**2**^{1}) **b**

	1	2	3	4	5	6
1	0	1	0	0	0	0
2	1	0	0	0	0	0
3	0	0	0	2^1	0	0
4	0	0	2^1	0	0	0
5	0	0	0	0	2	1
6	0	0	0	0	1	0

(4x**1**, 1x**2**, 2x**2**^{2}) **a**

	1	2	3	4	5	6
1	0	1	0	0	0	0
2	1	0	0	0	0	0
3	0	0	0	2^2	0	1
4	0	0	2^2	0	0	0
5	0	0	0	0	2	0
6	0	0	1	0	0	0

(4x**1**, 1x**2**, 2x**2**^{2}) **b**

	1	2	3	4	5	6
1	0	1	0	0	0	0
2	1	0	0	0	0	0
3	0	0	0	2^2	0	0
4	0	0	2^2	0	0	0
5	0	0	0	0	2	1
6	0	0	0	0	1	0

$^1V_3{}^2V_2{}^3V_3$	NG
$^1V_3{}^2V_3{}^3V_1$	NG
$^1V_3{}^2V_4{}^3V_1$	NG

$^1V_4{}^2V_1{}^3V_2$

(12x**1**) **a**

	1	2	3	4	5	6	7
1	0	0	0	0	0	1	0
2	0	0	0	0	0	1	1
3	0	0	0	0	0	0	1
4	0	0	0	0	0	0	1
5	0	0	0	0	0	1	0
6	1	1	0	0	1	0	0
7	0	1	1	1	0	0	0

(12x**1**) **b**

	1	2	3	4	5	6	7
1	0	1	0	0	0	0	0
2	1	0	0	0	1	0	1
3	0	0	0	0	0	1	1
4	0	0	0	0	0	0	1
5	0	1	0	0	0	0	0
6	0	0	1	0	0	0	0
7	0	1	1	1	0	0	0

(12x**1**) **c**

	1	2	3	4	5	6	7
1	0	0	0	0	1	1	0
2	0	0	0	0	0	1	0
3	0	0	0	0	0	0	1
4	0	0	0	0	1	0	0
5	1	0	0	1	0	1	0
6	1	1	0	0	1	0	0
7	0	0	1	0	0	0	0

(10x**1**, 1x**2**) **a**

	1	2	3	4	5	6	7
1	0	0	0	0	0	1	0
2	0	0	0	1	0	1	1
3	0	0	2	0	0	0	0
4	0	1	0	0	0	0	0
5	0	0	0	0	0	1	0
6	1	1	0	0	1	0	0
7	0	1	0	0	0	0	0

(10x**1**, 1x**2**) **b**

	1	2	3	4	5	6	7
1	0	0	0	0	0	1	0
2	0	0	0	1	0	1	0
3	0	0	2	0	0	0	1
4	0	1	0	0	0	0	0
5	0	0	0	0	0	1	0
6	1	1	0	0	1	0	0
7	0	0	1	0	0	0	0

(10x**1**, 1x**2**) **c**

	1	2	3	4	5	6	7
1	0	0	0	0	0	1	0
2	0	0	0	1	0	1	0
3	0	0	0	0	0	0	1
4	0	1	0	2	0	0	0
5	0	0	0	0	0	1	0
6	1	1	0	0	1	0	0
7	0	0	1	0	0	0	0

(10x**1**, 1x**2**) **d**

	1	2	3	4	5	6	7
1	0	0	0	0	0	1	0
2	0	0	0	1	0	1	0
3	0	0	0	0	0	0	1
4	0	1	0	0	0	0	0
5	0	0	0	0	2	1	0
6	1	1	0	0	1	0	0
7	0	0	1	0	0	0	0

(10x**1**, 1x**2**) **e**

	1	2	3	4	5	6	7
1	0	0	0	0	0	1	0
2	0	0	0	0	0	1	0
3	0	0	0	1	0	0	1
4	0	0	1	0	0	0	0
5	0	0	0	0	2	1	0
6	1	1	0	0	1	0	0
7	0	0	1	0	0	0	0

(10x**1**, 1x**2**) **f**

	1	2	3	4	5	6	7
1	0	0	0	0	0	1	0
2	0	0	0	0	0	1	0
3	0	0	0	1	0	0	1
4	0	0	1	2	0	0	0
5	0	0	0	0	0	1	0
6	1	1	0	0	1	0	0
7	0	0	1	0	0	0	0

(8x**1**, 2x**2**) **a**

	1	2	3	4	5	6	7
1	0	0	0	0	1	0	0
2	0	0	0	0	0	1	0
3	0	0	0	1	0	0	1
4	0	0	1	2	0	0	0
5	1	0	0	0	2	0	0
6	0	1	0	0	0	0	0
7	0	0	1	0	0	0	0

(8x**1**, 2x**2**) **b**

	1	2	3	4	5	6	7
1	2	0	0	0	1	0	0
2	0	0	0	0	0	1	0
3	0	0	0	1	0	0	1
4	0	0	1	0	0	0	0
5	1	0	0	0	2	0	0
6	0	1	0	0	0	0	0
7	0	0	1	0	0	0	0

(8x**1**, 2x**2**) **c**

	1	2	3	4	5	6	7
1	2	0	0	0	1	0	0
2	0	2	0	0	0	1	0
3	0	0	0	1	0	0	1
4	0	0	1	0	0	0	0
5	1	0	0	0	0	0	0
6	0	1	0	0	0	0	0
7	0	0	1	0	0	0	0

(8x**1**, 2x**2**) **d**

	1	2	3	4	5	6	7
1	0	0	0	0	1	0	0
2	0	0	0	0	0	1	0
3	0	0	0	1	0	0	1
4	0	0	1	2	0	0	0
5	1	0	0	0	0	0	0
6	0	1	0	0	0	0	0
7	0	0	1	0	0	0	2

(8x**1**, 2x**2**) **e**

	1	2	3	4	5	6	7
1	2	0	0	0	0	1	0
2	0	0	0	0	0	1	0
3	0	0	0	0	0	0	1
4	0	0	0	2	0	0	0
5	0	0	0	0	0	1	0
6	1	1	0	0	1	0	0
7	0	0	1	0	0	0	0

(8x**1**, 2x**2**) **f**

	1	2	3	4	5	6	7
1	0	0	0	0	0	1	0
2	0	0	0	0	0	1	0
3	0	0	2	0	0	0	1
4	0	0	0	2	0	0	0
5	0	0	0	0	0	1	0
6	1	1	0	0	1	0	0
7	0	0	1	0	0	0	0

(8x**1**, 2x$\mathbf{2}^1$) **a**

	1	2	3	4	5	6	7
1	0	0	1	0	0	0	0
2	0	0	1	0	0	0	0
3	1	1	0	1	0	0	0
4	0	0	1	0	0	0	0
5	0	0	0	0	0	1	0
6	0	0	0	0	1	0	2^1
7	0	0	0	0	0	2^1	0

(8x**1**, 2x$\mathbf{2}^1$) **b**

	1	2	3	4	5	6	7
1	0	0	0	1	0	0	0
2	0	0	1	0	0	0	0
3	0	1	0	0	0	0	0
4	1	0	0	0	0	0	1
5	0	0	0	0	0	1	0
6	0	0	0	0	1	0	2^1
7	0	0	0	1	0	2^1	0

(8x**1**, 2x$\mathbf{2}^1$) **c**

	1	2	3	4	5	6	7
1	0	0	1	0	0	0	0
2	0	0	1	0	0	0	0
3	1	1	0	0	0	0	0
4	0	0	0	0	0	0	1
5	0	0	0	0	0	1	0
6	0	0	0	0	1	0	2^1
7	0	0	0	1	0	2^1	0

(8x**1**, 2x$\mathbf{2}^1$) **d**

	1	2	3	4	5	6	7
1	0	0	0	1	0	0	0
2	0	0	1	0	0	0	0
3	0	1	0	0	0	0	0
4	1	0	0	0	1	0	1
5	0	0	0	1	0	0	0
6	0	0	0	0	0	0	2^1
7	0	0	0	1	0	2^1	0

(8x**1**, 2x$\mathbf{2}^1$) **e**

	1	2	3	4	5	6	7
1	0	0	1	0	0	0	0
2	0	0	0	0	1	0	0
3	1	0	0	0	0	0	0
4	0	0	0	0	0	1	1
5	0	1	0	0	0	0	0
6	0	0	0	1	0	0	2^1
7	0	0	0	1	0	2^1	0

(8x**1**, 2x$\mathbf{2}^2$) **a**

	1	2	3	4	5	6	7
1	0	0	1	0	0	0	0
2	0	0	1	0	0	0	0
3	1	1	0	1	0	0	0
4	0	0	1	0	0	0	0
5	0	0	0	0	0	1	0
6	0	0	0	0	1	0	2^2
7	0	0	0	0	0	2^2	0

(8x**1**, 2x$\mathbf{2}^2$) **b**

	1	2	3	4	5	6	7
1	0	0	0	1	0	0	0
2	0	0	1	0	0	0	0
3	0	1	0	0	0	0	0
4	1	0	0	0	0	0	1
5	0	0	0	0	0	1	0
6	0	0	0	0	1	0	2^2
7	0	0	0	1	0	2^2	0

(8x**1**, 2x$\mathbf{2}^2$) **c**

	1	2	3	4	5	6	7
1	0	0	1	0	0	0	0
2	0	0	1	0	0	0	0
3	1	1	0	0	0	0	0
4	0	0	0	0	0	0	1
5	0	0	0	0	0	1	0
6	0	0	0	0	1	0	2^2
7	0	0	0	1	0	2^2	0

(8x**1**, 2x$\mathbf{2}^2$) **d**

	1	2	3	4	5	6	7
1	0	0	0	1	0	0	0
2	0	0	1	0	0	0	0
3	0	1	0	0	0	0	0
4	1	0	0	0	1	0	1
5	0	0	0	1	0	0	0
6	0	0	0	0	0	0	2^2
7	0	0	0	1	0	2^2	0

(8x**1**, 2x$\mathbf{2}^2$) **e**

	1	2	3	4	5	6	7
1	0	0	1	0	0	0	0
2	0	0	0	0	1	0	0
3	1	0	0	0	0	0	0
4	0	0	0	0	0	1	1
5	0	1	0	0	0	0	0
6	0	0	0	1	0	0	2^2
7	0	0	0	1	0	2^2	0

(6x**1**, 3x**2**) **a**

	1	2	3	4	5	6	7
1	2	0	0	0	1	0	0
2	0	0	0	1	0	0	0
3	0	0	2	0	0	0	0
4	0	1	0	0	0	0	0
5	1	0	0	0	2	0	0
6	0	0	0	0	0	0	1
7	0	0	0	0	0	1	0

(6x**1**, 3x**2**) **b**

	1	2	3	4	5	6	7
1	0	0	0	0	1	0	0
2	0	0	0	1	0	0	0
3	0	0	2	0	0	0	0
4	0	1	0	0	0	0	0
5	1	0	0	0	2	0	0
6	0	0	0	0	0	0	1
7	0	0	0	0	0	1	2

(6x**1**, 1x**2**, 2x$\mathbf{2}^1$) **a**

	1	2	3	4	5	6	7
1	0	1	0	0	0	0	0
2	1	0	0	0	0	0	0
3	0	0	0	1	0	0	0
4	0	0	1	0	0	0	0
5	0	0	0	0	2	1	0
6	0	0	0	0	1	0	2^1
7	0	0	0	0	0	2^1	0

(6x**1**, 1x**2**, 2x$\mathbf{2}^1$) **b**

	1	2	3	4	5	6	7
1	2	1	0	0	0	0	0
2	1	0	0	0	0	0	0
3	0	0	0	1	0	0	0
4	0	0	1	0	0	0	0
5	0	0	0	0	0	1	0
6	0	0	0	0	1	0	2^1
7	0	0	0	0	0	2^1	0

(6x**1**, 1x**2**, 2x$\mathbf{2}^1$) **c**

	1	2	3	4	5	6	7
1	0	0	0	0	0	0	1
2	0	2	0	0	0	0	0
3	0	0	0	1	0	0	0
4	0	0	1	0	0	0	0
5	0	0	0	0	0	1	0
6	0	0	0	0	1	0	2^1
7	1	0	0	0	0	2^1	0

(6x**1**, 1x**2**, 2x**2**^{2}) **a**

	1	2	3	4	5	6	7
1	0	1	0	0	0	0	0
2	1	0	0	0	0	0	0
3	0	0	0	1	0	0	0
4	0	0	1	0	0	0	0
5	0	0	0	0	2	1	0
6	0	0	0	0	1	0	2^2
7	0	0	0	0	0	2^2	0

(6x**1**, 1x**2**, 2x**2**^{2}) **b**

	1	2	3	4	5	6	7
1	2	1	0	0	0	0	0
2	1	0	0	0	0	0	0
3	0	0	0	1	0	0	0
4	0	0	1	0	0	0	0
5	0	0	0	0	0	1	0
6	0	0	0	0	1	0	2^2
7	0	0	0	0	0	2^2	0

(6x**1**, 1x**2**, 2x**2**^{2}) **c**

	1	2	3	4	5	6	7
1	0	0	0	0	0	0	1
2	0	2	0	0	0	0	0
3	0	0	0	1	0	0	0
4	0	0	1	0	0	0	0
5	0	0	0	0	0	1	0
6	0	0	0	0	1	0	2^2
7	1	0	0	0	0	2^2	0

$^1V_4{}^2V_2{}^3V_2$

NG

$^1V_5{}^2V_1{}^3V_1$

(10x**1**) **a**

	1	2	3	4	5	6	7
1	0	1	1	1	0	0	0
2	1	0	0	0	0	0	0
3	1	0	0	0	0	0	0
4	1	0	0	0	0	0	0
5	0	0	0	0	0	1	0
6	0	0	0	0	1	0	1
7	0	0	0	0	0	1	0

(10x**1**) **b**

	1	2	3	4	5	6	7
1	0	1	1	1	0	0	0
2	1	0	0	0	1	0	0
3	1	0	0	0	0	0	0
4	1	0	0	0	0	0	0
5	0	1	0	0	0	0	0
6	0	0	0	0	0	0	1
7	0	0	0	0	0	1	0

(8x**1**, 1x**2**) **a**

	1	2	3	4	5	6	7
1	0	1	0	0	0	0	0
2	1	0	0	0	0	0	0
3	0	0	2	1	0	0	0
4	0	0	1	0	1	0	0
5	0	0	0	1	0	0	0
6	0	0	0	0	0	0	1
7	0	0	0	0	0	1	0

(8x**1**, 1x**2**) **b**

	1	2	3	4	5	6	7
1	2	0	0	0	0	0	0
2	0	0	0	1	0	0	0
3	0	0	0	1	0	0	0
4	0	1	1	0	1	0	0
5	0	0	0	1	0	0	0
6	0	0	0	0	0	0	1
7	0	0	0	0	0	1	0

(8x**1**, 1x**2**) **c**

	1	2	3	4	5	6	7
1	0	1	0	0	0	0	0
2	1	2	0	0	0	0	0
3	0	0	0	1	0	0	0
4	0	0	1	0	1	0	0
5	0	0	0	1	0	0	0
6	0	0	0	0	0	0	1
7	0	0	0	0	0	1	0

(6x**1**, 2x**2**)

	1	2	3	4	5	6	7
1	0	1	0	0	0	0	0
2	1	2	0	0	0	0	0
3	0	0	2	0	0	0	0
4	0	0	0	0	1	0	0
5	0	0	0	1	0	0	0
6	0	0	0	0	0	0	1
7	0	0	0	0	0	1	0

(6x**1**, 2x**2**^{1})

	1	2	3	4	5	6	7
1	0	1	0	0	0	0	0
2	1	0	0	0	0	0	0
3	0	0	0	2^1	0	0	0
4	0	0	2^1	0	1	0	0
5	0	0	0	1	0	0	0
6	0	0	0	0	0	0	1
7	0	0	0	0	0	1	0

(6x**1**, 2x**2**^{2})

	1	2	3	4	5	6	7
1	0	1	0	0	0	0	0
2	1	0	0	0	0	0	0
3	0	0	0	2^2	0	0	0
4	0	0	2^2	0	1	0	0
5	0	0	0	1	0	0	0
6	0	0	0	0	0	0	1
7	0	0	0	0	0	1	0

$^1V_5{}^2V_2{}^3V_1$

NG

$^1V_r{}^2V_r{}^4V_r$

$^1V_2{}^2V_1{}^4V_1$

(6x**1**, 1x**2**)

	1	2	3	4
1	0	1	0	0
2	1	0	0	1
3	0	0	0	1
4	0	1	1	2

(4x**1**, 2x**2**)

	1	2	3	4
1	2	0	0	0
2	0	0	0	1
3	0	0	0	1
4	0	1	1	2

(4x**1**, 2x**2**^{1})

	1	2	3	4
1	0	0	0	1
2	0	0	0	1
3	0	0	0	2^1
4	1	1	2^1	0

(4x**1**, 2x**2**^{2})

	1	2	3	4
1	0	0	0	1
2	0	0	0	1
3	0	0	0	2^1
4	1	1	2^1	0

(2x**1**, 1x**2**, 2x**2**^{1})

	1	2	3	4
1	0	1	0	0
2	1	0	0	0
3	0	0	0	2^1
4	0	0	2^1	2

(2x**1**, 1x**2**, 2x**2**^{2})

	1	2	3	4
1	0	1	0	0
2	1	0	0	0
3	0	0	0	2^2
4	0	0	2^2	2

$^1V_2{}^2V_1{}^4V_2$

(10x**1**, 1x**2**)

	1	2	3	4	5
1	2	1	1	0	0
2	1	0	1	1	1
3	1	1	0	0	0
4	0	1	0	0	0
5	0	1	0	0	0

(8x**1**, 2x**2**) **a**

	1	2	3	4	5
1	2	1	1	0	0
2	1	0	0	1	0
3	1	0	0	0	0
4	0	1	0	2	1
5	0	0	0	1	0

(8x**1**, 2x**2**) **b**

	1	2	3	4	5
1	2	1	1	0	0
2	1	2	1	0	0
3	1	1	0	0	0
4	0	0	0	0	1
5	0	0	0	1	0

(8x**1**, 2x**2**) **c**

	1	2	3	4	5
1	2	1	1	0	0
2	1	2	0	1	0
3	1	0	0	0	0
4	0	1	0	0	1
5	0	0	0	1	0

(8x**1**, 2x**2**^{1})

	1	2	3	4	5
1	0	2^1	1	1	0
2	2^1	0	1	0	1
3	1	1	0	0	0
4	1	0	0	0	0
5	0	1	0	0	0

(8x**1**, 2x$\mathbf{2}^2$)

	1	2	3	4	5
1	0	2^2	1	1	0
2	2^2	0	1	0	1
3	1	1	0	0	0
4	1	0	0	0	0
5	0	1	0	0	0

(6x**1**, 3x**2**)

	1	2	3	4	5
1	2	1	1	0	0
2	1	2	0	1	0
3	1	0	0	0	0
4	0	1	0	0	0
5	0	0	0	0	2

(6x**1**, 1x**2**, 2x$\mathbf{2}^1$) **a**

	1	2	3	4	5
1	0	1	1	0	0
2	1	0	0	2^1	1
3	1	0	0	0	0
4	0	2^1	0	2	0
5	0	1	0	0	0

(6x**1**, 1x**2**, 2x$\mathbf{2}^1$) **b**

	1	2	3	4	5
1	2	1	1	0	0
2	1	0	0	2^1	1
3	1	0	0	0	0
4	0	2^1	0	0	0
5	0	1	0	0	0

(6x**1**, 1x**2**, 2x$\mathbf{2}^2$) **a**

	1	2	3	4	5
1	0	1	1	0	0
2	1	0	0	2^2	1
3	1	0	0	0	0
4	0	2^2	0	2	0
5	0	1	0	0	0

(6x**1**, 1x**2**, 2x$\mathbf{2}^2$) **b**

	1	2	3	4	5
1	2	1	1	0	0
2	1	0	0	2^2	1
3	1	0	0	0	0
4	0	2^2	0	0	0
5	0	1	0	0	0

(4x**1**, 2x**2**, 2x$\mathbf{2}^1$) **a**

	1	2	3	4	5
1	0	0	1	0	0
2	0	0	0	2^1	0
3	1	0	2	0	1
4	0	2^1	0	2	0
5	0	0	1	0	0

(4x**1**, 2x**2**, 2x$\mathbf{2}^1$) **b**

	1	2	3	4	5
1	0	0	0	0	1
2	0	2	0	0	0
3	0	0	0	0	1
4	0	0	0	2	2^1
5	1	0	1	2^1	0

(4x**1**, 2x**2**, 2x$\mathbf{2}^1$) **c**

	1	2	3	4	5
1	0	0	1	0	0
2	0	2	0	2^1	0
3	1	0	0	0	1
4	0	2^1	0	2	0
5	0	0	1	0	0

(4x**1**, 2x**2**, 2x$\mathbf{2}^2$) **a**

	1	2	3	4	5
1	0	0	1	0	0
2	0	0	0	2^2	0
3	1	0	2	0	1
4	0	2^2	0	2	0
5	0	0	1	0	0

(4x**1**, 2x**2**, 2x$\mathbf{2}^2$) **b**

	1	2	3	4	5
1	0	0	0	0	1
2	0	2	0	0	0
3	0	0	0	0	1
4	0	0	0	2	2^2
5	1	0	1	2^2	0

(4x**1**, 2x**2**, 2x$\mathbf{2}^2$) **c**

	1	2	3	4	5
1	0	0	1	0	0
2	0	2	0	2^2	0
3	1	0	0	0	1
4	0	2^2	0	2	0
5	0	0	1	0	0

(4x**1**, 4x$\mathbf{2}^1$)

	1	2	3	4	5
1	0	0	0	0	1
2	0	0	0	2^1	0
3	0	0	0	0	1
4	0	2^1	0	0	2^1
5	1	0	1	2^1	0

(4x**1**, 2x$\mathbf{2}^1$, 2x$\mathbf{2}^2$) **a**

	1	2	3	4	5
1	0	0	0	0	1
2	0	0	0	2^2	0
3	0	0	0	0	1
4	0	2^2	0	0	2^1
5	1	0	1	2^1	0

(4x**1**, 2x$\mathbf{2}^1$, 2x$\mathbf{2}^2$) **b**

	1	2	3	4	5
1	0	0	0	0	1
2	0	0	0	2^1	0
3	0	0	0	0	1
4	0	2^1	0	0	2^2
5	1	0	1	2^2	0

(4x**1**, 4x$\mathbf{2}^2$)

	1	2	3	4	5
1	0	0	0	0	1
2	0	0	0	2^2	0
3	0	0	0	0	1
4	0	2^2	0	0	2^2
5	1	0	1	2^2	0

(2x**1**, 3x**2**, 2x$\mathbf{2}^1$)

	1	2	3	4	5
1	0	0	1	0	0
2	0	2	0	2^1	0
3	1	0	0	0	0
4	0	2^1	0	2	0
5	0	0	0	0	2

(2x**1**, 3x**2**, 2x$\mathbf{2}^2$)

	1	2	3	4	5
1	0	0	1	0	0
2	0	2	0	2^2	0
3	1	0	0	0	0
4	0	2^2	0	2	0
5	0	0	0	0	2

(2x**1**, 1x**2**, 4x$\mathbf{2}^1$)

	1	2	3	4	5
1	0	0	1	0	0
2	0	0	0	2^1	0
3	1	0	0	0	0
4	0	2^1	0	0	2^1
5	0	0	0	2^1	2

(2x**1**, 1x**2**, 2x$\mathbf{2}^1$, 2x$\mathbf{2}^2$) **a**

	1	2	3	4	5
1	0	0	1	0	0
2	0	0	0	2^1	0
3	1	0	0	0	0
4	0	2^1	0	0	2^2
5	0	0	0	2^2	2

(2x**1**, 1x**2**, 2x$\mathbf{2}^1$, 2x$\mathbf{2}^2$) **b**

	1	2	3	4	5
1	0	0	1	0	0
2	0	0	0	2^2	0
3	1	0	0	0	0
4	0	2^2	0	0	2^1
5	0	0	0	2^1	2

(2x**1**, 1x**2**, 4x$\mathbf{2}^2$)

	1	2	3	4	5
1	0	0	1	0	0
2	0	0	0	2^2	0
3	1	0	0	0	0
4	0	2^2	0	0	2^2
5	0	0	0	2^2	2

$^1V_2{}^2V_1{}^4V_3$ NG

$^1V_2{}^2V_1{}^4V_4$ NG

$^1V_2{}^2V_1{}^4V_5$ NG

$^1V_2{}^2V_2{}^4V_1$

(10x**1**)

	1	2	3	4	5
1	0	1	1	0	0
2	1	0	1	1	1
3	1	1	0	0	0
4	0	1	0	0	0
5	0	1	0	0	0

(8x**1**, 1x**2**) **a**

	1	2	3	4	5
1	2	1	1	0	0
2	1	0	1	0	0
3	1	1	0	0	0
4	0	0	0	0	1
5	0	0	0	1	0

(8x**1**, 1x**2**) **b**

	1	2	3	4	5
1	0	1	1	0	0
2	1	2	0	1	0
3	1	0	0	0	0
4	0	1	0	0	1
5	0	0	0	1	0

(8x**1**, 1x**2**) **c**

	1	2	3	4	5
1	0	1	1	0	0
2	1	0	0	1	0
3	1	0	0	0	0
4	0	1	0	2	1
5	0	0	0	1	0

(6x**1**, 2x**2**)

	1	2	3	4	5
1	0	1	1	0	0
2	1	2	0	1	0
3	1	0	0	0	0
4	0	1	0	0	0
5	0	0	0	0	2

(6x**1**, 2x$\mathbf{2}^{1}$)

	1	2	3	4	5
1	0	1	1	0	0
2	1	0	0	2^1	1
3	1	0	0	0	0
4	0	2^1	0	0	0
5	0	1	0	0	0

(6x**1**, 2x$\mathbf{2}^{2}$)

	1	2	3	4	5
1	0	1	1	0	0
2	1	0	0	2^2	1
3	1	0	0	0	0
4	0	2^2	0	0	0
5	0	1	0	0	0

(4x**1**, 3x**2**)

	1	2	3	4	5
1	2	1	1	0	0
2	1	0	0	0	0
3	1	0	0	0	0
4	0	0	0	2	0
5	0	0	0	0	2

(4x**1**, 1x**2**, 2x$\mathbf{2}^{1}$) **a**

	1	2	3	4	5
1	0	0	1	0	0
2	0	0	0	2^1	0
3	1	0	0	0	1
4	0	2^1	0	2	0
5	0	0	1	0	0

(4x**1**, 1x**2**, 2x$\mathbf{2}^{1}$) **b**

	1	2	3	4	5
1	0	0	1	0	0
2	0	0	0	2^1	0
3	1	0	2	0	1
4	0	2^1	0	0	0
5	0	0	1	0	0

(4x**1**, 1x**2**, 2x$\mathbf{2}^{1}$) **c**

	1	2	3	4	5
1	0	0	0	0	1
2	0	2	0	0	0
3	0	0	0	0	1
4	0	0	0	0	2^1
5	1	0	1	2^1	0

(4x**1**, 1x**2**, 2x$\mathbf{2}^{2}$) **a**

	1	2	3	4	5
1	0	0	1	0	0
2	0	0	0	2^2	0
3	1	0	0	0	1
4	0	2^2	0	2	0
5	0	0	1	0	0

(4x**1**, 1x**2**, 2x$\mathbf{2}^{2}$) **b**

	1	2	3	4	5
1	0	0	1	0	0
2	0	0	0	2^2	0
3	1	0	2	0	1
4	0	2^2	0	0	0
5	0	0	1	0	0

(4x**1**, 1x**2**, 2x$\mathbf{2}^{2}$) **c**

	1	2	3	4	5
1	0	0	0	0	1
2	0	2	0	0	0
3	0	0	0	0	1
4	0	0	0	0	2^2
5	1	0	1	2^2	0

(2x**1**, 2x**2**, 2x$\mathbf{2}^{1}$)

	1	2	3	4	5
1	0	0	1	0	0
2	0	2	0	0	0
3	1	0	0	0	0
4	0	0	0	2	2^1
5	0	0	0	2^1	0

(2x**1**, 2x**2**, 2x$\mathbf{2}^{2}$)

	1	2	3	4	5
1	0	0	1	0	0
2	0	2	0	0	0
3	1	0	0	0	0
4	0	0	0	2	2^2
5	0	0	0	2^2	0

(2x**1**, 4x$\mathbf{2}^{1}$)

	1	2	3	4	5
1	0	0	1	0	0
2	0	0	0	2^1	0
3	1	0	0	0	0
4	0	2^1	0	0	2^1
5	0	0	0	2^1	0

(2x**1**, 2x$\mathbf{2}^{1}$, 2x$\mathbf{2}^{2}$)

	1	2	3	4	5
1	0	0	1	0	0
2	0	0	0	2^1	0
3	1	0	0	0	0
4	0	2^1	0	0	2^2
5	0	0	0	2^2	0

(2x**1**, 4x$\mathbf{2}^{2}$)

	1	2	3	4	5
1	0	0	1	0	0
2	0	0	0	2^2	0
3	1	0	0	0	0
4	0	2^2	0	0	2^2
5	0	0	0	2^2	0

${}^1V_2{}^2V_2{}^4V_2$	NG
${}^1V_2{}^2V_2{}^4V_3$	NG
${}^1V_2{}^2V_2{}^4V_4$	NG
${}^1V_2{}^2V_3{}^4V_1$	NG
${}^1V_2{}^2V_3{}^4V_2$	NG
${}^1V_2{}^2V_3{}^4V_3$	NG
${}^1V_2{}^2V_4{}^4V_1$	NG
${}^1V_2{}^2V_4{}^4V_2$	NG
${}^1V_2{}^2V_5{}^4V_1$	NG

${}^1V_4{}^2V_1{}^4V_1$

(10x**1**)

	1	2	3	4	5	6
1	0	1	0	0	0	0
2	1	0	1	1	1	0
3	0	1	0	0	0	0
4	0	1	0	0	0	1
5	0	1	0	0	0	0
6	0	0	0	1	0	0

(8x**1**, 1x**2**) **a**

	1	2	3	4	5	6
1	0	0	1	0	0	0
2	0	0	0	1	0	0
3	1	0	2	0	0	1
4	0	1	0	0	1	0
5	0	0	0	1	0	0
6	0	0	1	0	0	0

(8x**1**, 1x**2**) **b**

	1	2	3	4	5	6
1	0	0	1	0	0	0
2	0	0	0	0	0	1
3	1	0	2	0	0	1
4	0	0	0	0	1	0
5	0	0	0	1	0	0
6	0	1	1	0	0	0

(8x**1**, 1x**2**) **c**

	1	2	3	4	5	6
1	0	1	0	0	0	0
2	1	0	1	1	1	0
3	0	1	0	0	0	0
4	0	1	0	0	0	0
5	0	1	0	0	0	0
6	0	0	0	0	0	2

(6x**1**, 2x**2**)

	1	2	3	4	5	6
1	0	0	1	0	0	0
2	0	0	0	1	0	0
3	1	0	2	0	0	1
4	0	1	0	0	0	0
5	0	0	0	0	2	0
6	0	0	1	0	0	0

(6x**1**, 2x**2**^{1})

	1	2	3	4	5	6
1	0	0	0	0	1	0
2	0	0	0	0	0	1
3	0	0	0	0	1	0
4	0	0	0	0	2^1	0
5	1	0	1	2^1	0	0
6	0	1	0	0	0	0

(6x**1**, 2x**2**^{2})

	1	2	3	4	5	6
1	0	0	0	0	1	0
2	0	0	0	0	0	1
3	0	0	0	0	1	0
4	0	0	0	0	2^2	0
5	1	0	1	2^2	0	0
6	0	1	0	0	0	0

(4x**1**, 1x**2**, 2x**2**^{1})

	1	2	3	4	5	6
1	0	0	1	0	0	0
2	0	0	0	0	0	1
3	1	0	0	0	0	0
4	0	0	0	2	2^1	0
5	0	0	0	2^1	0	0
6	0	1	0	0	0	0

(4x**1**, 1x**2**, 2x**2**^{2})

	1	2	3	4	5	6
1	0	0	1	0	0	0
2	0	0	0	0	0	1
3	1	0	0	0	0	0
4	0	0	0	2	2^2	0
5	0	0	0	2^2	0	0
6	0	1	0	0	0	0

$^1V_4{}^2V_1{}^4V_2$	NG
$^1V_4{}^2V_1{}^4V_3$	NG
$^1V_4{}^2V_2{}^4V_1$	NG
$^1V_4{}^2V_2{}^4V_2$	NG
$^1V_4{}^2V_3{}^4V_1$	NG
$^1V_6{}^2V_1{}^4V_1$	NG
**$^1V_r{}^3V_r{}^4V_r$**	

$^1V_1{}^3V_1{}^4V_1$

(4x**1**, 2x**2**)

	1	2	3
1	0	0	1
2	0	2	1
3	1	1	2

(2x**1**, 1x**2**, 2x**2**^{1})

	1	2	3
1	2	2^1	0
2	2^1	0	1
3	0	1	0

(2x**1**, 1x**2**, 2x**2**^{2})

	1	2	3
1	2	2^2	0
2	2^2	0	1
3	0	1	0

$^1V_1{}^3V_1{}^4V_2$

(8x**1**, 2x**2**)

	1	2	3	4
1	2	1	1	0
2	1	2	1	0
3	1	1	0	1
4	0	0	1	0

(6x**1**, 3x**2**)

	1	2	3	4
1	2	1	1	0
2	1	2	0	1
3	1	0	0	0
4	0	1	0	2

(6x**1**, 1x**2**, 2x**2**^{1})

	1	2	3	4
1	0	1	0	0
2	1	0	1	2^1
3	0	1	2	1
4	0	2^1	1	0

(6x**1**, 1x**2**, 2x**2**^{2})

	1	2	3	4
1	0	1	0	0
2	1	0	1	2^2
3	0	1	2	1
4	0	2^2	1	0

(4x**1**, 2x**2**, 2x**2**^{1}) **a**

	1	2	3	4
1	2	1	0	0
2	1	0	1	2^1
3	0	1	0	0
4	0	2^1	0	2

(4x**1**, 2x**2**, 2x**2**^{1}) **b**

	1	2	3	4
1	2	2^1	0	0
2	2^1	0	0	1
3	0	0	0	1
4	0	1	1	2

(4x**1**, 2x**2**, 2x**2**^{2}) **a**

	1	2	3	4
1	2	1	0	0
2	1	0	1	2^2
3	0	1	0	0
4	0	2^2	0	2

(4x**1**, 2x**2**, 2x**2**^{2}) **b**

	1	2	3	4
1	2	2^2	0	0
2	2^2	0	0	1
3	0	0	0	1
4	0	1	1	2

(4x**1**, 4x**2**^{1})

	1	2	3	4
1	0	1	0	0
2	1	0	1	2^1
3	0	1	0	2^1
4	0	2^1	2^1	0

(4x**1**, 2x**2**^{1}, 2x**2**^{2})

	1	2	3	4
1	0	1	0	0
2	1	0	1	2^1
3	0	1	0	2^2
4	0	2^1	2^2	0

(4x**1**, 4x**2**^{2})

	1	2	3	4
1	0	1	0	0
2	1	0	1	2^2
3	0	1	0	2^2
4	0	2^2	2^2	0

(2x**1**, 3x**2**, 2x**2**^{1})

	1	2	3	4
1	0	0	1	0
2	0	2	0	2^1
3	1	0	2	0
4	0	2^1	0	2

(2x**1**, 3x**2**, 2x**2**^{2})

	1	2	3	4
1	0	0	1	0
2	0	2	0	2^2
3	1	0	2	0
4	0	2^2	0	2

(2x**1**, 1x**2**, 4x**2**^{1})

	1	2	3	4
1	0	0	1	0
2	0	2	0	2^1
3	1	0	0	2^1
4	0	2^1	2^1	0

(2x**1**, 1x**2**, 2x**2**^{1}, 2x**2**^{2})

	1	2	3	4
1	0	0	1	0
2	0	2	0	2^2
3	1	0	0	2^1
4	0	2^2	2^1	0

(2x**1**, 1x**2**, 4x**2**^{2})

	1	2	3	4
1	0	0	1	0
2	0	2	0	2^2
3	1	0	0	2^2
4	0	2^2	2^2	0

$^{1}V_{1}{}^{3}V_{1}{}^{4}V_{3}$

(12x**1**, 2x**2**)

	1	2	3	4	5
1	2	1	0	1	0
2	1	0	1	1	1
3	0	1	2	1	0
4	1	1	1	0	0
5	0	1	0	0	0

(12x**1**, 2x**2**^{1})

	1	2	3	4	5
1	0	1	1	1	0
2	1	0	1	1	1
3	1	1	0	2^1	0
4	1	1	2^1	0	0
5	0	1	0	0	0

(12x**1**, 2x**2**^{2})

	1	2	3	4	5
1	0	1	1	1	0
2	1	0	1	1	1
3	1	1	0	2^2	0
4	1	1	2^2	0	0
5	0	1	0	0	0

(10x**1**, 3x**2**) **a**

	1	2	3	4	5
1	2	1	0	1	0
2	1	0	1	0	1
3	0	1	2	1	0
4	1	0	1	2	0
5	0	1	0	0	0

(10x**1**, 3x**2**) **b**

	1	2	3	4	5
1	0	0	0	0	1
2	0	0	1	1	1
3	0	1	2	1	0
4	0	1	1	2	0
5	1	1	0	0	2

(10x**1**, 3x**2**) **c**

	1	2	3	4	5
1	0	1	0	0	0
2	1	0	1	1	1
3	0	1	2	1	0
4	0	1	1	2	0
5	0	1	0	0	2

(10x**1**, 1x**2**, 2x**2**^{1}) **a**

	1	2	3	4	5
1	2	1	0	1	0
2	1	0	1	1	1
3	0	1	0	2^1	0
4	1	1	2^1	0	0
5	0	1	0	0	0

(10x**1**, 1x**2**, 2x**2**^{1}) **b**

	1	2	3	4	5
1	0	1	1	1	0
2	1	0	2^1	0	1
3	1	2^1	0	1	0
4	1	0	1	2	0
5	0	1	0	0	0

(10x**1**, 1x**2**, 2x**2**^{1}) **c**

	1	2	3	4	5
1	0	1	2^1	1	0
2	1	0	1	0	1
3	2^1	1	0	1	0
4	1	0	1	2	0
5	0	1	0	0	0

(10x**1**, 1x**2**, 2x**2**^{2}) **a**

	1	2	3	4	5
1	2	1	0	1	0
2	1	0	1	1	1
3	0	1	0	2^2	0
4	1	1	2^2	0	0
5	0	1	0	0	0

(10x**1**, 1x**2**, 2x**2**^{2}) **b**

	1	2	3	4	5
1	0	1	1	1	0
2	1	0	2^2	0	1
3	1	2^2	0	1	0
4	1	0	1	2	0
5	0	1	0	0	0

(10x**1**, 1x**2**, 2x**2**^{2}) **c**

	1	2	3	4	5
1	0	1	2^2	1	0
2	1	0	1	0	1
3	2^2	1	0	1	0
4	1	0	1	2	0
5	0	1	0	0	0

(8x**1**, 4x**2**) **a**

	1	2	3	4	5
1	0	0	0	0	1
2	0	2	1	0	1
3	0	1	2	1	0
4	0	0	1	2	0
5	1	1	0	0	2

(8x**1**, 4x**2**) **b**

	1	2	3	4	5
1	0	0	0	0	1
2	0	2	1	1	0
3	0	1	2	1	0
4	0	1	1	2	0
5	1	0	0	0	2

(8x**1**, 2x**2**, 2x**2**^{1}) **a**

	1	2	3	4	5
1	0	0	0	0	1
2	0	0	1	1	2^1
3	0	1	2	1	0
4	0	1	1	2	0
5	1	2^1	0	0	0

(8x**1**, 2x**2**, 2x**2**^{1}) **b**

	1	2	3	4	5
1	0	0	0	0	1
2	0	0	2^1	1	1
3	0	2^1	0	1	0
4	0	1	1	2	0
5	1	1	0	0	2

(8x**1**, 2x**2**, 2x**2**^{1}) **c**

	1	2	3	4	5
1	0	0	1	0	0
2	0	0	1	1	2^1
3	1	1	0	1	0
4	0	1	1	2	0
5	0	2^1	0	0	2

(8x**1**, 2x**2**, 2x**2**^{1}) **d**

	1	2	3	4	5
1	0	0	1	0	0
2	0	0	2^1	1	1
3	1	2^1	0	1	0
4	0	1	1	2	0
5	0	1	0	0	2

(8x**1**, 2x**2**, 2x**2**^{1}) **e**

	1	2	3	4	5
1	0	2^1	1	0	1
2	2^1	0	0	1	0
3	1	0	2	1	0
4	0	1	1	2	0
5	1	0	0	0	0

(8x**1**, 2x**2**, 2x**2**^{2}) **a**

	1	2	3	4	5
1	0	0	0	0	1
2	0	0	1	1	2^2
3	0	1	2	1	0
4	0	1	1	2	0
5	1	2^2	0	0	0

(8x**1**, 2x**2**, 2x**2**^{2}) **b**

	1	2	3	4	5
1	0	0	0	0	1
2	0	0	2^2	1	1
3	0	2^2	0	1	0
4	0	1	1	2	0
5	1	1	0	0	2

(8x**1**, 2x**2**, 2x**2**^{2}) **c**

	1	2	3	4	5
1	0	0	1	0	0
2	0	0	1	1	2^2
3	1	1	0	1	0
4	0	1	1	2	0
5	0	2^2	0	0	2

(8x**1**, 2x**2**, 2x**2**^{2}) **d**

	1	2	3	4	5
1	0	0	1	0	0
2	0	0	2^2	1	1
3	1	2^2	0	1	0
4	0	1	1	2	0
5	0	1	0	0	2

(8x**1**, 2x**2**, 2x**2**^{2}) **e**

	1	2	3	4	5
1	0	2^2	1	0	1
2	2^2	0	0	1	0
3	1	0	2	1	0
4	0	1	1	2	0
5	1	0	0	0	0

(8x**1**, 4x**2**^{1}) **a**

	1	2	3	4	5
1	0	1	1	2^1	0
2	1	0	1	0	1
3	1	1	0	2^1	0
4	2^1	0	2^1	0	0
5	0	1	0	0	0

(8x**1**, 4x**2**^{1}) **b**

	1	2	3	4	5
1	0	1	1	2^1	0
2	1	0	2^1	0	1
3	1	2^1	0	1	0
4	2^1	0	1	0	0
5	0	1	0	0	0

(8x**1**, 2x**2**^{1}, 2x**2**^{2}) **a**

	1	2	3	4	5
1	0	1	1	2^2	0
2	1	0	1	0	1
3	1	1	0	2^1	0
4	2^2	0	2^1	0	0
5	0	1	0	0	0

(8x**1**, 2x**2**^{1}, 2x**2**^{2}) **b**

	1	2	3	4	5
1	0	1	1	2^2	0
2	1	0	2^1	0	1
3	1	2^1	0	1	0
4	2^2	0	1	0	0
5	0	1	0	0	0

(8x**1**, 2x**2**^{1}, 2x**2**^{2}) **c**

	1	2	3	4	5
1	0	1	1	2^2	0
2	1	0	2^2	0	1
3	1	2^2	0	1	0
4	2^2	0	1	0	0
5	0	1	0	0	0

(8x**1**, 4x**2**^{2}) **a**

	1	2	3	4	5
1	0	1	1	2^2	0
2	1	0	1	0	1
3	1	1	0	2^2	0
4	2^2	0	2^2	0	0
5	0	1	0	0	0

(8x**1**, 4x**2**^{2}) **b**

	1	2	3	4	5
1	0	1	1	2^2	0
2	1	0	2^2	0	1
3	1	2^2	0	1	0
4	2^2	0	1	0	0
5	0	1	0	0	0

(6x**1**, 3x**2**, 2x**2**^{1}) **a**

	1	2	3	4	5
1	0	0	0	0	1
2	0	0	1	2^1	1
3	0	1	2	0	0
4	0	2^1	0	2	0
5	1	1	0	0	2

(6x**1**, 3x**2**, 2x**2**^{1}) **b**

	1	2	3	4	5
1	2	0	0	0	1
2	0	0	1	2^1	1
3	0	1	0	0	0
4	0	2^1	0	2	0
5	1	1	0	0	2

(6x**1**, 3x**2**, 2x**2**^{1}) **c**

	1	2	3	4	5
1	2	0	0	0	2^1
2	0	2	1	0	1
3	0	1	2	1	0
4	0	0	1	0	0
5	2^1	1	0	0	0

(6x**1**, 3x**2**, 2x**2**^{2}) **a**

	1	2	3	4	5
1	0	0	0	0	1
2	0	0	1	2^2	1
3	0	1	2	0	0
4	0	2^2	0	2	0
5	1	1	0	0	2

(6x**1**, 3x**2**, 2x$\mathbf{2}^2$) **b**

	1	2	3	4	5
1	2	0	0	0	1
2	0	0	1	2^2	1
3	0	1	0	0	0
4	0	2^2	0	2	0
5	1	1	0	0	2

(6x**1**, 3x**2**, 2x$\mathbf{2}^2$) **c**

	1	2	3	4	5
1	2	0	0	0	2^2
2	0	2	1	0	1
3	0	1	2	1	0
4	0	0	1	0	0
5	2^2	1	0	0	0

(6x**1**, 1x**2**, 4x$\mathbf{2}^1$) **a**

	1	2	3	4	5
1	0	0	1	0	0
2	0	0	1	1	2^1
3	1	1	0	2^1	0
4	0	1	2^1	0	0
5	0	2^1	0	0	2

(6x**1**, 1x**2**, 4x$\mathbf{2}^1$) **b**

	1	2	3	4	5
1	0	0	1	0	0
2	0	0	1	2^1	1
3	1	1	0	2^1	0
4	0	2^1	2^1	0	0
5	0	1	0	0	2

(6x**1**, 1x**2**, 4x$\mathbf{2}^1$) **c**

	1	2	3	4	5
1	0	0	0	0	1
2	0	0	1	2^1	1
3	0	1	0	2^1	0
4	0	2^1	2^1	0	0
5	1	1	0	0	2

(6x**1**, 1x**2**, 4x$\mathbf{2}^1$) **d**

	1	2	3	4	5
1	0	1	0	2^1	0
2	1	2	1	0	0
3	0	1	0	2^1	1
4	2^1	0	2^1	0	0
5	0	0	1	0	0

(6x**1**, 1x**2**, 2x$\mathbf{2}^1$, 2x$\mathbf{2}^2$) **a**

	1	2	3	4	5
1	0	0	1	0	0
2	0	0	1	1	2^2
3	1	1	0	2^1	0
4	0	1	2^1	0	0
5	0	2^2	0	0	2

(6x**1**, 1x**2**, 2x$\mathbf{2}^1$, 2x$\mathbf{2}^2$) **b**

	1	2	3	4	5
1	0	0	1	0	0
2	0	0	1	1	2^1
3	1	1	0	2^2	0
4	0	1	2^2	0	0
5	0	2^1	0	0	2

(6x**1**, 1x**2**, 2x$\mathbf{2}^1$, 2x$\mathbf{2}^2$) **c**

	1	2	3	4	5
1	0	0	1	0	0
2	0	0	1	2^1	1
3	1	1	0	2^2	0
4	0	2^1	2^2	0	0
5	0	1	0	0	2

(6x**1**, 1x**2**, 2x$\mathbf{2}^1$, 2x$\mathbf{2}^2$) **d**

	1	2	3	4	5
1	0	0	1	0	0
2	0	0	1	2^2	1
3	1	1	0	2^1	0
4	0	2^2	2^1	0	0
5	0	1	0	0	2

(6x**1**, 1x**2**, 2x$\mathbf{2}^1$, 2x$\mathbf{2}^2$) **e**

	1	2	3	4	5
1	0	0	0	0	1
2	0	0	1	2^1	1
3	0	1	0	2^2	0
4	0	2^1	2^2	0	0
5	1	1	0	0	2

(6x**1**, 1x**2**, 2x$\mathbf{2}^1$, 2x$\mathbf{2}^2$) **f**

	1	2	3	4	5
1	0	0	0	0	1
2	0	0	1	2^2	1
3	0	1	0	2^1	0
4	0	2^2	2^1	0	0
5	1	1	0	0	2

(6x**1**, 1x**2**, 2x$\mathbf{2}^1$, 2x$\mathbf{2}^2$) **g**

	1	2	3	4	5
1	0	1	0	2^1	0
2	1	2	1	0	0
3	0	1	0	2^2	1
4	2^1	0	2^2	0	0
5	0	0	1	0	0

(6x**1**, 1x**2**, 2x$\mathbf{2}^1$, 2x$\mathbf{2}^2$) **h**

	1	2	3	4	5
1	0	1	0	2^2	0
2	1	2	1	0	0
3	0	1	0	2^1	1
4	2^2	0	2^1	0	0
5	0	0	1	0	0

(6x**1**, 1x**2**, 4x$\mathbf{2}^2$) **a**

	1	2	3	4	5
1	0	0	1	0	0
2	0	0	1	1	2^2
3	1	1	0	2^2	0
4	0	1	2^2	0	0
5	0	2^2	0	0	2

(6x**1**, 1x**2**, 4x$\mathbf{2}^2$) **b**

	1	2	3	4	5
1	0	0	1	0	0
2	0	0	1	2^2	1
3	1	1	0	2^2	0
4	0	2^2	2^2	0	0
5	0	1	0	0	2

(6x**1**, 1x**2**, 4x$\mathbf{2}^2$) **c**

	1	2	3	4	5
1	0	0	0	0	1
2	0	0	1	2^2	1
3	0	1	0	2^2	0
4	0	2^2	2^2	0	0
5	1	1	0	0	2

(6x**1**, 1x**2**, 4x$\mathbf{2}^2$) **d**

	1	2	3	4	5
1	0	1	0	2^2	0
2	1	2	1	0	0
3	0	1	0	2^2	1
4	2^2	0	2^2	0	0
5	0	0	1	0	0

(4x**1**, 4x**2**, 2x$\mathbf{2}^1$)

	1	2	3	4	5
1	2	0	0	0	2^1
2	0	2	1	0	0
3	0	1	2	1	0
4	0	0	1	0	0
5	2^1	0	0	0	2

(4x**1**, 4x**2**, 2x$\mathbf{2}^2$)

	1	2	3	4	5
1	2	0	0	0	2^2
2	0	2	1	0	0
3	0	1	2	1	0
4	0	0	1	0	0
5	2^2	0	0	0	2

(4x**1**, 2x**2**, 4x$\mathbf{2}^1$) **a**

	1	2	3	4	5
1	2	0	0	0	2^1
2	0	0	1	2^1	1
3	0	1	0	0	0
4	0	2^1	0	2	0
5	2^1	1	0	0	0

(4x**1**, 2x**2**, 4x$\mathbf{2}^1$) **b**

	1	2	3	4	5
1	2	0	0	0	2^1
2	0	0	1	1	2^1
3	0	1	0	0	0
4	0	1	0	2	0
5	2^1	2^1	0	0	0

(4x**1**, 2x**2**, 4x$\mathbf{2}^1$) **c**

	1	2	3	4	5
1	2	0	0	0	2^1
2	0	0	1	0	2^1
3	0	1	2	1	0
4	0	0	1	0	0
5	2^1	2^1	0	0	0

(4x**1**, 2x**2**, 2x$\mathbf{2}^1$, 2x$\mathbf{2}^2$) **a**

	1	2	3	4	5
1	2	0	0	0	2^2
2	0	0	1	2^1	1
3	0	1	0	0	0
4	0	2^1	0	2	0
5	2^2	1	0	0	0

(4x**1**, 2x**2**, 2x$\mathbf{2}^1$, 2x$\mathbf{2}^2$) **b**

	1	2	3	4	5
1	2	0	0	0	2^1
2	0	0	1	2^2	1
3	0	1	0	0	0
4	0	2^2	0	2	0
5	2^1	1	0	0	0

(4x**1**, 2x**2**, 2x$\mathbf{2}^1$, 2x$\mathbf{2}^2$) **c**

	1	2	3	4	5
1	2	0	0	0	2^2
2	0	0	1	1	2^1
3	0	1	0	0	0
4	0	1	0	2	0
5	2^2	2^1	0	0	0

(4x**1**, 2x**2**, 2x$\mathbf{2}^1$, 2x$\mathbf{2}^2$) **d**

	1	2	3	4	5
1	2	0	0	0	2^1
2	0	0	1	1	2^2
3	0	1	0	0	0
4	0	1	0	2	0
5	2^1	2^2	0	0	0

(4x**1**, 2x**2**, 2x$\mathbf{2}^1$, 2x$\mathbf{2}^2$) **e**

	1	2	3	4	5
1	2	0	0	0	2^2
2	0	0	1	0	2^1
3	0	1	2	1	0
4	0	0	1	0	0
5	2^2	2^1	0	0	0

(4x**1**, 2x**2**, 2x$\mathbf{2}^1$, 2x$\mathbf{2}^2$) **f**

	1	2	3	4	5
1	2	0	0	0	2^1
2	0	0	1	0	2^2
3	0	1	2	1	0
4	0	0	1	0	0
5	2^1	2^2	0	0	0

(4x**1**, 2x**2**, 4x$\mathbf{2}^2$) **a**

	1	2	3	4	5
1	2	0	0	0	2^2
2	0	0	1	2^2	1
3	0	1	0	0	0
4	0	2^2	0	2	0
5	2^2	1	0	0	0

(4x**1**, 2x**2**, 4x$\mathbf{2}^2$) **b**

	1	2	3	4	5
1	2	0	0	0	2^2
2	0	0	1	1	2^2
3	0	1	0	0	0
4	0	1	0	2	0
5	2^2	2^2	0	0	0

(4x**1**, 2x**2**, 4x$\mathbf{2}^2$) **c**

	1	2	3	4	5
1	2	0	0	0	2^2
2	0	0	1	0	2^2
3	0	1	2	1	0
4	0	0	1	0	0
5	2^2	2^2	0	0	0

(4x**1**, 6x$\mathbf{2}^1$)

	1	2	3	4	5
1	0	2^1	0	2^1	0
2	2^1	0	1	0	0
3	0	1	0	2^1	1
4	2^1	0	2^1	0	0
5	0	0	1	0	0

(4x**1**, 4x$\mathbf{2}^1$, 2x$\mathbf{2}^2$) **a**

	1	2	3	4	5
1	0	2^2	0	2^1	0
2	2^2	0	1	0	0
3	0	1	0	2^1	1
4	2^1	0	2^1	0	0
5	0	0	1	0	0

(4x**1**, 4x$\mathbf{2}^1$, 2x$\mathbf{2}^2$) **b**

	1	2	3	4	5
1	0	2^1	0	2^2	0
2	2^1	0	1	0	0
3	0	1	0	2^1	1
4	2^2	0	2^1	0	0
5	0	0	1	0	0

(4x**1**, 4x$\mathbf{2}^{1}$, 2x$\mathbf{2}^{2}$) **c**

	1	2	3	4	5
1	0	2^1	0	2^1	0
2	2^1	0	1	0	0
3	0	1	0	2^2	1
4	2^1	0	2^2	0	0
5	0	0	1	0	0

(4x**1**, 2x$\mathbf{2}^{1}$, 4x$\mathbf{2}^{2}$) **a**

	1	2	3	4	5
1	0	2^2	0	2^2	0
2	2^2	0	1	0	0
3	0	1	0	2^1	1
4	2^2	0	2^1	0	0
5	0	0	1	0	0

(4x**1**, 2x$\mathbf{2}^{1}$, 4x$\mathbf{2}^{2}$) **b**

	1	2	3	4	5
1	0	2^2	0	2^1	0
2	2^2	0	1	0	0
3	0	1	0	2^2	1
4	2^1	0	2^2	0	0
5	0	0	1	0	0

(4x**1**, 2x$\mathbf{2}^{1}$, 4x$\mathbf{2}^{2}$) **c**

	1	2	3	4	5
1	0	2^1	0	2^2	0
2	2^1	0	1	0	0
3	0	1	0	2^2	1
4	2^2	0	2^2	0	0
5	0	0	1	0	0

(4x**1**, 6x$\mathbf{2}^{2}$)

	1	2	3	4	5
1	0	2^2	0	2^2	0
2	2^2	0	1	0	0
3	0	1	0	2^2	1
4	2^2	0	2^2	0	0
5	0	0	1	0	0

(2x**1**, 3x**2**, 4x$\mathbf{2}^{1}$) **a**

	1	2	3	4	5
1	2	0	0	0	2^1
2	0	2	2^1	0	0
3	0	2^1	0	1	0
4	0	0	1	0	0
5	2^1	0	0	0	2

(2x**1**, 3x**2**, 4x$\mathbf{2}^{1}$) **b**

	1	2	3	4	5
1	0	0	0	0	1
2	0	2	2^1	0	0
3	0	2^1	0	2^1	0
4	0	0	2^1	2	0
5	1	0	0	0	2

(2x**1**, 3x**2**, 2x$\mathbf{2}^{1}$, 2x$\mathbf{2}^{2}$) **a**

	1	2	3	4	5
1	2	0	0	0	2^2
2	0	2	2^1	0	0
3	0	2^1	0	1	0
4	0	0	1	0	0
5	2^2	0	0	0	2

(2x**1**, 3x**2**, 2x$\mathbf{2}^{1}$, 2x$\mathbf{2}^{2}$) **b**

	1	2	3	4	5
1	2	0	0	0	2^1
2	0	2	2^2	0	0
3	0	2^2	0	1	0
4	0	0	1	0	0
5	2^1	0	0	0	2

(2x**1**, 3x**2**, 2x$\mathbf{2}^{1}$, 2x$\mathbf{2}^{2}$) **c**

	1	2	3	4	5
1	0	0	0	0	1
2	0	2	2^2	0	0
3	0	2^2	0	2^1	0
4	0	0	2^1	2	0
5	1	0	0	0	2

(2x**1**, 3x**2**, 4x$\mathbf{2}^{2}$) **a**

	1	2	3	4	5
1	2	0	0	0	2^2
2	0	2	2^2	0	0
3	0	2^2	0	1	0
4	0	0	1	0	0
5	2^2	0	0	0	2

(2x**1**, 3x**2**, 4x$\mathbf{2}^{2}$) **b**

	1	2	3	4	5
1	0	0	0	0	1
2	0	2	2^2	0	0
3	0	2^2	0	2^2	0
4	0	0	2^2	2	0
5	1	0	0	0	2

(2x**1**, 1x**2**, 6x$\mathbf{2}^{1}$) **a**

	1	2	3	4	5
1	0	0	0	0	1
2	0	0	2^1	0	2^1
3	0	2^1	0	2^1	0
4	0	0	2^1	2	0
5	1	2^1	0	0	0

(2x**1**, 1x**2**, 6x$\mathbf{2}^{1}$) **b**

	1	2	3	4	5
1	2	0	0	0	1
2	0	0	2^1	2^1	0
3	0	2^1	0	2^1	0
4	0	2^1	2^1	0	0
5	1	0	0	0	0

(2x**1**, 1x**2**, 4x$\mathbf{2}^{1}$, 2x$\mathbf{2}^{2}$) **a**

	1	2	3	4	5
1	0	0	0	0	1
2	0	0	2^1	0	2^2
3	0	2^1	0	2^1	0
4	0	0	2^1	2	0
5	1	2^2	0	0	0

(2x**1**, 1x**2**, 4x$\mathbf{2}^{1}$, 2x$\mathbf{2}^{2}$) **b**

	1	2	3	4	5
1	0	0	0	0	1
2	0	0	2^2	0	2^1
3	0	2^2	0	2^1	0
4	0	0	2^1	2	0
5	1	2^1	0	0	0

(2x**1**, 1x**2**, 4x$\mathbf{2}^{1}$, 2x$\mathbf{2}^{2}$) **c**

	1	2	3	4	5
1	0	0	0	0	1
2	0	0	2^1	0	2^1
3	0	2^1	0	2^2	0
4	0	0	2^2	2	0
5	1	2^1	0	0	0

(2x**1**, 1x**2**, 4x$\mathbf{2}^{1}$, 2x$\mathbf{2}^{2}$) **d**

	1	2	3	4	5
1	2	0	0	0	1
2	0	0	2^1	2^1	0
3	0	2^1	0	2^2	0
4	0	2^1	2^2	0	0
5	1	0	0	0	0

(2x**1**, 1x**2**, 2x$\mathbf{2}^{1}$, 4x$\mathbf{2}^{2}$) **a**

	1	2	3	4	5
1	0	0	0	0	1
2	0	0	2^2	0	2^2
3	0	2^2	0	2^1	0
4	0	0	2^1	2	0
5	1	2^2	0	0	0

(2x**1**, 1x**2**, 2x$\mathbf{2}^{1}$, 4x$\mathbf{2}^{2}$) **b**

	1	2	3	4	5
1	0	0	0	0	1
2	0	0	2^1	0	2^2
3	0	2^1	0	2^2	0
4	0	0	2^2	2	0
5	1	2^2	0	0	0

(2x**1**, 1x**2**, 2x$\mathbf{2}^{1}$, 4x$\mathbf{2}^{2}$) **c**

	1	2	3	4	5
1	0	0	0	0	1
2	0	0	2^2	0	2^1
3	0	2^2	0	2^2	0
4	0	0	2^2	2	0
5	1	2^1	0	0	0

(2x**1**, 1x**2**, 2x$\mathbf{2}^{1}$, 4x$\mathbf{2}^{2}$) **d**

	1	2	3	4	5
1	2	0	0	0	1
2	0	0	2^2	2^1	0
3	0	2^2	0	2^2	0
4	0	2^1	2^2	0	0
5	1	0	0	0	0

(2x**1**, 1x**2**, 6x$\mathbf{2}^{2}$) **a**

	1	2	3	4	5
1	0	0	0	0	1
2	0	0	2^2	0	2^2
3	0	2^2	0	2^2	0
4	0	0	2^2	2	0
5	1	2^2	0	0	0

(2x**1**, 1x**2**, 6x$\mathbf{2}^{2}$) **b**

	1	2	3	4	5
1	2	0	0	0	1
2	0	0	2^2	2^2	0
3	0	2^2	0	2^2	0
4	0	2^2	2^2	0	0
5	1	0	0	0	0

$^{1}V_{1}{}^{3}V_{1}{}^{4}V_{4}$ NG

$^{1}V_{1}{}^{3}V_{1}{}^{4}V_{5}$ NG

$^{1}V_{1}{}^{3}V_{1}{}^{4}V_{6}$ NG

$^{1}V_{1}{}^{3}V_{3}{}^{4}V_{1}$

(14x**1**)

	1	2	3	4	5
1	0	1	1	1	0
2	1	0	1	1	1
3	1	1	0	1	0
4	1	1	1	0	0
5	0	1	0	0	0

(12x**1**, 1x**2**)

	1	2	3	4	5
1	2	1	0	1	0
2	1	0	1	1	0
3	0	1	0	1	1
4	1	1	1	0	0
5	0	0	1	0	0

(10x**1**, 2x**2**)

	1	2	3	4	5
1	2	1	0	1	0
2	1	0	1	1	0
3	0	1	2	0	0
4	1	1	0	0	1
5	0	0	0	1	0

(10x**1**, 2x$\mathbf{2}^{1}$)

	1	2	3	4	5
1	0	1	0	2^1	0
2	1	0	1	1	0
3	0	1	0	1	1
4	2^1	1	1	0	0
5	0	0	1	0	0

(10x**1**, 2x$\mathbf{2}^{2}$)

	1	2	3	4	5
1	0	1	0	2^2	0
2	1	0	1	1	0
3	0	1	0	1	1
4	2^2	1	1	0	0
5	0	0	1	0	0

(8x**1**, 3x**2**) **a**

	1	2	3	4	5
1	2	0	0	1	0
2	0	2	1	0	0
3	0	1	2	1	0
4	1	0	1	0	1
5	0	0	0	1	0

(8x**1**, 3x**2**) **b**

	1	2	3	4	5
1	2	0	0	1	0
2	0	0	1	0	0
3	0	1	2	1	0
4	1	0	1	0	1
5	0	0	0	1	2

(8x**1**, 3x**2**) **c**

	1	2	3	4	5
1	2	0	0	1	0
2	0	0	0	1	0
3	0	0	2	1	0
4	1	1	1	0	1
5	0	0	0	1	2

(8x**1**, 1x**2**, 2x$\mathbf{2^1}$) **a**

	1	2	3	4	5
1	0	1	0	2^1	0
2	1	2	1	0	0
3	0	1	0	1	1
4	2^1	0	1	0	0
5	0	0	1	0	0

(8x**1**, 1x**2**, 2x$\mathbf{2^1}$) **b**

	1	2	3	4	5
1	0	1	0	2^1	0
2	1	0	1	1	0
3	0	1	2	0	1
4	2^1	1	0	0	0
5	0	0	1	0	0

(8x**1**, 1x**2**, 2x$\mathbf{2^1}$) **c**

	1	2	3	4	5
1	0	1	0	2^1	0
2	1	0	1	1	0
3	0	1	2	0	0
4	2^1	1	0	0	1
5	0	0	0	1	0

(8x**1**, 1x**2**, 2x$\mathbf{2^1}$) **d**

	1	2	3	4	5
1	0	1	0	2^1	0
2	1	0	1	1	0
3	0	1	0	0	0
4	2^1	1	0	0	1
5	0	0	0	1	2

(8x**1**, 1x**2**, 2x$\mathbf{2^1}$) **e**

	1	2	3	4	5
1	0	1	0	2^1	0
2	1	0	1	1	1
3	0	1	2	0	0
4	2^1	1	0	0	0
5	0	1	0	0	0

(8x**1**, 1x**2**, 2x$\mathbf{2^2}$) **a**

	1	2	3	4	5
1	0	1	0	2^2	0
2	1	2	1	0	0
3	0	1	0	1	1
4	2^2	0	1	0	0
5	0	0	1	0	0

(8x**1**, 1x**2**, 2x$\mathbf{2^2}$) **b**

	1	2	3	4	5
1	0	1	0	2^2	0
2	1	0	1	1	0
3	0	1	2	0	1
4	2^2	1	0	0	0
5	0	0	1	0	0

(8x**1**, 1x**2**, 2x$\mathbf{2^2}$) **c**

	1	2	3	4	5
1	0	1	0	2^2	0
2	1	0	1	1	0
3	0	1	2	0	0
4	2^2	1	0	0	1
5	0	0	0	1	0

(8x**1**, 1x**2**, 2x$\mathbf{2^2}$) **d**

	1	2	3	4	5
1	0	1	0	2^2	0
2	1	0	1	1	0
3	0	1	0	0	0
4	2^2	1	0	0	1
5	0	0	0	1	2

(8x**1**, 1x**2**, 2x$\mathbf{2^2}$) **e**

	1	2	3	4	5
1	0	1	0	2^2	0
2	1	0	1	1	1
3	0	1	2	0	0
4	2^2	1	0	0	0
5	0	1	0	0	0

(6x**1**, 4x**2**) **a**

	1	2	3	4	5
1	2	0	0	1	0
2	0	2	1	0	0
3	0	1	0	0	0
4	1	0	0	2	1
5	0	0	0	1	2

(6x**1**, 4x**2**) **b**

	1	2	3	4	5
1	0	0	0	1	0
2	0	2	1	0	0
3	0	1	2	0	0
4	1	0	0	2	1
5	0	0	0	1	2

(6x**1**, 2x**2**, 2x$\mathbf{2^1}$) **a**

	1	2	3	4	5
1	2	0	0	1	0
2	0	2	1	0	0
3	0	1	0	2^1	0
4	1	0	2^1	0	1
5	0	0	0	1	0

(6x**1**, 2x**2**, 2x$\mathbf{2^1}$) **b**

	1	2	3	4	5
1	2	0	0	1	0
2	0	0	1	0	0
3	0	1	0	2^1	0
4	1	0	2^1	0	1
5	0	0	0	1	2

(6x**1**, 2x**2**, 2x$\mathbf{2^1}$) **c**

	1	2	3	4	5
1	2	0	0	1	0
2	0	2	2^1	0	0
3	0	2^1	0	1	0
4	1	0	1	0	1
5	0	0	0	1	0

(6x**1**, 2x**2**, 2x$\mathbf{2^1}$) **d**

	1	2	3	4	5
1	2	0	0	1	1
2	0	0	1	0	0
3	0	1	0	2^1	0
4	1	0	2^1	0	0
5	1	0	0	0	2

(6x**1**, 2x**2**, 2x$\mathbf{2^1}$) **e**

	1	2	3	4	5
1	2	0	0	1	1
2	0	2	1	0	0
3	0	1	0	2^1	0
4	1	0	2^1	0	0
5	1	0	0	0	0

(6x**1**, 2x**2**, 2x$\mathbf{2^1}$) **f**

	1	2	3	4	5
1	0	0	0	0	1
2	0	2	1	1	0
3	0	1	0	2^1	0
4	0	1	2^1	0	0
5	1	0	0	0	2

(6x**1**, 2x**2**, 2x$\mathbf{2^2}$) **a**

	1	2	3	4	5
1	2	0	0	1	0
2	0	2	1	0	0
3	0	1	0	2^2	0
4	1	0	2^2	0	1
5	0	0	0	1	0

(6x**1**, 2x**2**, 2x$\mathbf{2^2}$) **b**

	1	2	3	4	5
1	2	0	0	1	0
2	0	0	1	0	0
3	0	1	0	2^2	0
4	1	0	2^2	0	1
5	0	0	0	1	2

(6x**1**, 2x**2**, 2x$\mathbf{2^2}$) **c**

	1	2	3	4	5
1	2	0	0	1	0
2	0	2	2^2	0	0
3	0	2^2	0	1	0
4	1	0	1	0	1
5	0	0	0	1	0

(6x**1**, 2x**2**, 2x$\mathbf{2^2}$) **d**

	1	2	3	4	5
1	2	0	0	1	1
2	0	0	1	0	0
3	0	1	0	2^2	0
4	1	0	2^2	0	0
5	1	0	0	0	2

(6x**1**, 2x**2**, 2x$\mathbf{2^2}$) **e**

	1	2	3	4	5
1	2	0	0	1	1
2	0	2	1	0	0
3	0	1	0	2^2	0
4	1	0	2^2	0	0
5	1	0	0	0	0

(6x**1**, 2x**2**, 2x$\mathbf{2^2}$) **f**

	1	2	3	4	5
1	0	0	0	0	1
2	0	2	1	1	0
3	0	1	0	2^2	0
4	0	1	2^2	0	0
5	1	0	0	0	2

(6x**1**, 4x$\mathbf{2^1}$) **a**

	1	2	3	4	5
1	0	1	0	2^1	0
2	1	0	2^1	0	0
3	0	2^1	0	1	1
4	2^1	0	1	0	0
5	0	0	1	0	0

(6x**1**, 4x$\mathbf{2^1}$) **b**

	1	2	3	4	5
1	0	2^1	0	2^1	0
2	2^1	0	1	0	0
3	0	1	0	1	1
4	2^1	0	1	0	0
5	0	0	1	0	0

(6x**1**, 4x$\mathbf{2^1}$) **c**

	1	2	3	4	5
1	0	1	0	2^1	0
2	1	0	2^1	1	0
3	0	2^1	0	0	1
4	2^1	1	0	0	0
5	0	0	1	0	0

(6x**1**, 2x$\mathbf{2^1}$, 2x$\mathbf{2^2}$) **a**

	1	2	3	4	5
1	0	1	0	2^2	0
2	1	0	2^1	0	0
3	0	2^1	0	1	1
4	2^2	0	1	0	0
5	0	0	1	0	0

(6x**1**, 2x$\mathbf{2^1}$, 2x$\mathbf{2^2}$) **b**

	1	2	3	4	5
1	0	1	0	2^1	0
2	1	0	2^2	0	0
3	0	2^2	0	1	1
4	2^1	0	1	0	0
5	0	0	1	0	0

(6x**1**, 2x2^1, 2x$\mathbf{2^2}$) **c**

	1	2	3	4	5
1	0	2^1	0	2^2	0
2	2^1	0	1	0	0
3	0	1	0	1	1
4	2^2	0	1	0	0
5	0	0	1	0	0

(6x**1**, 2x$\mathbf{2^1}$, 2x$\mathbf{2^2}$) **d**

	1	2	3	4	5
1	0	1	0	2^2	0
2	1	0	2^1	1	0
3	0	2^1	0	0	1
4	2^2	1	0	0	0
5	0	0	1	0	0

(6x**1**, 2x$\mathbf{2^1}$, 2x$\mathbf{2^2}$) **e**

	1	2	3	4	5
1	0	1	0	2^1	0
2	1	0	2^2	1	0
3	0	2^2	0	0	1
4	2^1	1	0	0	0
5	0	0	1	0	0

(6x**1**, 4x**2^2**) **a**

	1	2	3	4	5
1	0	1	0	2^2	0
2	1	0	2^2	0	0
3	0	2^2	0	1	1
4	2^2	0	1	0	0
5	0	0	1	0	0

(6x**1**, 4x**2^2**) **b**

	1	2	3	4	5
1	0	2^2	0	2^2	0
2	2^2	0	1	0	0
3	0	1	0	1	1
4	2^2	0	1	0	0
5	0	0	1	0	0

(6x**1**, 4x**2^2**) **c**

	1	2	3	4	5
1	0	1	0	2^2	0
2	1	0	2^2	1	0
3	0	2^2	0	0	1
4	2^2	1	0	0	0
5	0	0	1	0	0

(4x**1**, 3x**2**, 2x**2^1**) **a**

	1	2	3	4	5
1	0	0	0	1	0
2	0	2	0	0	1
3	0	0	2	2^1	0
4	1	0	2^1	0	0
5	0	1	0	0	2

(4x**1**, 3x**2**, 2x**2^1**) **b**

	1	2	3	4	5
1	2	0	0	1	0
2	0	2	0	0	1
3	0	0	2	2^1	0
4	1	0	2^1	0	0
5	0	1	0	0	0

(4x**1**, 3x**2**, 2x**2^2**) **a**

	1	2	3	4	5
1	0	0	0	1	0
2	0	2	0	0	1
3	0	0	2	2^2	0
4	1	0	2^2	0	0
5	0	1	0	0	2

(4x**1**, 3x**2**, 2x**2^2**) **b**

	1	2	3	4	5
1	2	0	0	1	0
2	0	2	0	0	1
3	0	0	2	2^2	0
4	1	0	2^2	0	0
5	0	1	0	0	0

(4x**1**, 1x**2**, 4x**2^1**) **a**

	1	2	3	4	5
1	0	0	0	2^1	1
2	0	0	1	0	0
3	0	1	0	2^1	0
4	2^1	0	2^1	0	0
5	1	0	0	0	2

(4x**1**, 1x**2**, 4x**2^1**) **b**

	1	2	3	4	5
1	0	0	0	1	2^1
2	0	0	1	0	0
3	0	1	0	2^1	0
4	1	0	2^1	0	0
5	2^1	0	0	0	2

(4x**1**, 1x**2**, 4x**2^1**) **c**

	1	2	3	4	5
1	0	0	1	2^1	0
2	0	2	0	0	1
3	1	0	0	2^1	0
4	2^1	0	2^1	0	0
5	0	1	0	0	0

(4x**1**, 1x**2**, 2x**2^1**, 2x**2^2**) **a**

	1	2	3	4	5
1	0	0	0	2^2	1
2	0	0	1	0	0
3	0	1	0	2^1	0
4	2^2	0	2^1	0	0
5	1	0	0	0	2

(4x**1**, 1x**2**, 2x**2^1**, 2x**2^2**) **b**

	1	2	3	4	5
1	0	0	0	2^1	1
2	0	0	1	0	0
3	0	1	0	2^2	0
4	2^1	0	2^2	0	0
5	1	0	0	0	2

(4x**1**, 1x**2**, 2x**2^1**, 2x**2^2**) **c**

	1	2	3	4	5
1	0	0	0	1	2^2
2	0	0	1	0	0
3	0	1	0	2^1	0
4	1	0	2^1	0	0
5	2^2	0	0	0	2

(4x**1**, 1x**2**, 2x**2^1**, 2x**2^2**) **d**

	1	2	3	4	5
1	0	0	0	1	2^1
2	0	0	1	0	0
3	0	1	0	2^2	0
4	1	0	2^2	0	0
5	2^1	0	0	0	2

(4x**1**, 1x**2**, 2x**2^1**, 2x**2^2**) **e**

	1	2	3	4	5
1	0	0	1	2^2	0
2	0	2	0	0	1
3	1	0	0	2^1	0
4	2^2	0	2^1	0	0
5	0	1	0	0	0

(4x**1**, 1x**2**, 4x**2^2**) **a**

	1	2	3	4	5
1	0	0	0	2^2	1
2	0	0	1	0	0
3	0	1	0	2^2	0
4	2^2	0	2^2	0	0
5	1	0	0	0	2

(4x**1**, 1x**2**, 4x**2^2**) **b**

	1	2	3	4	5
1	0	0	0	1	2^2
2	0	0	1	0	0
3	0	1	0	2^2	0
4	1	0	2^2	0	0
5	2^2	0	0	0	2

(4x**1**, 1x**2**, 4x**2^2**) **c**

	1	2	3	4	5
1	0	0	1	2^2	0
2	0	2	0	0	1
3	1	0	0	2^2	0
4	2^2	0	2^2	0	0
5	0	1	0	0	0

$^{1}V_{1}{}^{3}V_{3}{}^{4}V_{2}$ NG

$^{1}V_{1}{}^{3}V_{3}{}^{4}V_{3}$ NG

$^{1}V_{1}{}^{3}V_{3}{}^{4}V_{4}$ NG

$^{1}V_{1}{}^{3}V_{5}{}^{4}V_{1}$ NG

$^{1}V_{1}{}^{3}V_{5}{}^{4}V_{2}$ NG

$^{1}V_{2}{}^{3}V_{2}{}^{4}V_{1}$

(10x**1**, 1x**2**)

	1	2	3	4	5
1	2	1	0	1	0
2	1	0	1	1	0
3	0	1	0	0	0
4	1	1	0	0	1
5	0	0	0	1	0

(8x**1**, 2x**2**) **a**

	1	2	3	4	5
1	0	0	0	1	0
2	0	0	0	1	0
3	0	0	2	1	0
4	1	1	1	0	1
5	0	0	0	1	2

(8x**1**, 2x**2**) **b**

	1	2	3	4	5
1	0	0	0	1	0
2	0	2	1	0	0
3	0	1	2	1	0
4	1	0	1	0	1
5	0	0	0	1	0

(8x**1**, 2x**2**) **c**

	1	2	3	4	5
1	0	0	0	1	0
2	0	0	1	0	0
3	0	1	2	1	0
4	1	0	1	0	1
5	0	0	0	1	2

(8x**1**, 2x**2^1**) **a**

	1	2	3	4	5
1	0	2^1	1	0	0
2	2^1	0	1	0	0
3	1	1	0	1	1
4	0	0	1	0	0
5	0	0	1	0	0

(8x**1**, 2x**2^1**) **b**

	1	2	3	4	5
1	0	2^1	1	0	0
2	2^1	0	1	1	0
3	1	1	0	0	1
4	0	1	0	0	0
5	0	0	1	0	0

(8x**1**, 2x**2^2**) **a**

	1	2	3	4	5
1	0	2^2	1	0	0
2	2^2	0	1	0	0
3	1	1	0	1	1
4	0	0	1	0	0
5	0	0	1	0	0

(8x**1**, 2x**2^2**) **b**

	1	2	3	4	5
1	0	2^2	1	0	0
2	2^2	0	1	1	0
3	1	1	0	0	1
4	0	1	0	0	0
5	0	0	1	0	0

(6x**1**, 3x**2**) **a**

	1	2	3	4	5
1	2	0	0	1	0
2	0	2	1	0	0
3	0	1	0	0	0
4	1	0	0	2	1
5	0	0	0	1	0

(6x**1**, 3x**2**) **b**

	1	2	3	4	5
1	0	0	0	1	0
2	0	2	1	0	0
3	0	1	2	0	0
4	1	0	0	2	1
5	0	0	0	1	0

(6x**1**, 3x**2**) **c**

	1	2	3	4	5
1	2	0	0	1	0
2	0	0	1	0	0
3	0	1	0	0	0
4	1	0	0	2	1
5	0	0	0	1	2

(6x**1**, 1x**2**, 2x$\mathbf{2}^{1}$) **a**

	1	2	3	4	5
1	0	0	0	1	0
2	0	2	2^1	0	0
3	0	2^1	0	1	0
4	1	0	1	0	1
5	0	0	0	1	0

(6x**1**, 1x**2**, 2x$\mathbf{2}^{1}$) **b**

	1	2	3	4	5
1	0	0	0	1	0
2	0	2	1	0	0
3	0	1	0	2^1	0
4	1	0	2^1	0	1
5	0	0	0	1	0

(6x**1**, 1x**2**, 2x$\mathbf{2}^{1}$) **c**

	1	2	3	4	5
1	0	0	0	0	1
2	0	0	2^1	1	0
3	0	2^1	0	1	0
4	0	1	1	2	0
5	1	0	0	0	0

(6x**1**, 1x**2**, 2x$\mathbf{2}^{1}$) **d**

	1	2	3	4	5
1	0	0	0	1	0
2	0	0	1	0	0
3	0	1	0	2^1	0
4	1	0	2^1	0	1
5	0	0	0	1	2

(6x**1**, 1x**2**, 2x$\mathbf{2}^{1}$) **e**

	1	2	3	4	5
1	0	0	0	0	1
2	0	0	1	0	0
3	0	1	0	2^1	0
4	0	0	2^1	0	1
5	1	0	0	1	2

(6x**1**, 1x**2**, 2x$\mathbf{2}^{2}$) **a**

	1	2	3	4	5
1	0	0	0	1	0
2	0	2	2^2	0	0
3	0	2^2	0	1	0
4	1	0	1	0	1
5	0	0	0	1	0

(6x**1**, 1x**2**, 2x$\mathbf{2}^{2}$) **b**

	1	2	3	4	5
1	0	0	0	1	0
2	0	2	1	0	0
3	0	1	0	2^2	0
4	1	0	2^2	0	1
5	0	0	0	1	0

(6x**1**, 1x**2**, 2x$\mathbf{2}^{2}$) **c**

	1	2	3	4	5
1	0	0	0	0	1
2	0	0	2^2	1	0
3	0	2^2	0	1	0
4	0	1	1	2	0
5	1	0	0	0	0

(6x**1**, 1x**2**, 2x$\mathbf{2}^{2}$) **d**

	1	2	3	4	5
1	0	0	0	1	0
2	0	0	1	0	0
3	0	1	0	2^2	0
4	1	0	2^2	0	1
5	0	0	0	1	2

(6x**1**, 1x**2**, 2x$\mathbf{2}^{2}$) **e**

	1	2	3	4	5
1	0	0	0	0	1
2	0	0	1	0	0
3	0	1	0	2^2	0
4	0	0	2^2	0	1
5	1	0	0	1	2

(4x**1**, 2x**2**, 2x$\mathbf{2}^{1}$) **a**

	1	2	3	4	5
1	0	0	0	0	1
2	0	2	2^1	0	0
3	0	2^1	0	1	0
4	0	0	1	2	0
5	1	0	0	0	0

(4x**1**, 2x**2**, 2x$\mathbf{2}^{1}$) **b**

	1	2	3	4	5
1	0	0	0	0	1
2	0	2	2^1	0	0
3	0	2^1	0	1	0
4	0	0	1	0	0
5	1	0	0	0	2

(4x**1**, 2x**2**, 2x$\mathbf{2}^{2}$) **a**

	1	2	3	4	5
1	0	0	0	0	1
2	0	2	2^2	0	0
3	0	2^2	0	1	0
4	0	0	1	2	0
5	1	0	0	0	0

(4x**1**, 2x**2**, 2x$\mathbf{2}^{2}$) **b**

	1	2	3	4	5
1	0	0	0	0	1
2	0	2	2^2	0	0
3	0	2^2	0	1	0
4	0	0	1	0	0
5	1	0	0	0	2

(4x**1**, 4x$\mathbf{2}^{1}$) **a**

	1	2	3	4	5
1	0	0	0	0	1
2	0	0	2^1	1	0
3	0	2^1	0	2^1	0
4	0	1	2^1	0	0
5	1	0	0	0	0

(4x**1**, 4x$\mathbf{2}^{1}$) **b**

	1	2	3	4	5
1	0	0	0	0	1
2	0	0	1	0	0
3	0	1	0	2^1	0
4	0	0	2^1	0	2^1
5	1	0	0	2^1	0

(4x**1**, 2x$\mathbf{2}^{1}$, 2x$\mathbf{2}^{2}$) **a**

	1	2	3	4	5
1	0	0	0	0	1
2	0	0	2^1	1	0
3	0	2^1	0	2^2	0
4	0	1	2^2	0	0
5	1	0	0	0	0

(4x**1**, 2x$\mathbf{2}^{1}$, 2x$\mathbf{2}^{2}$) **b**

	1	2	3	4	5
1	0	0	0	0	1
2	0	0	1	0	0
3	0	1	0	2^2	0
4	0	0	2^2	0	2^1
5	1	0	0	2^1	0

(4x**1**, 4x$\mathbf{2}^{2}$) **a**

	1	2	3	4	5
1	0	0	0	0	1
2	0	0	2^2	1	0
3	0	2^2	0	2^2	0
4	0	1	2^2	0	0
5	1	0	0	0	0

(4x**1**, 4x$\mathbf{2}^{2}$) **b**

	1	2	3	4	5
1	0	0	0	0	1
2	0	0	1	0	0
3	0	1	0	2^2	0
4	0	0	2^2	0	2^2
5	1	0	0	2^2	0

$^1V_2{}^3V_2{}^4V_2$ NG

$^1V_2{}^3V_2{}^4V_3$ NG

$^1V_2{}^3V_2{}^4V_4$ NG

$^1V_2{}^3V_4{}^4V_1$ NG

$^1V_2{}^3V_4{}^4V_2$ NG

$^1V_3{}^3V_1{}^4V_1$

(8x**1**, 1x**2**) **a**

	1	2	3	4	5
1	0	0	0	1	0
2	0	0	0	1	0
3	0	0	0	1	0
4	1	1	1	0	1
5	0	0	0	1	2

(8x**1**, 1x**2**) **b**

	1	2	3	4	5
1	0	0	0	1	0
2	0	0	1	0	0
3	0	1	2	1	0
4	1	0	1	0	1
5	0	0	0	1	0

(8x**1**, 1x**2**) **c**

	1	2	3	4	5
1	0	0	0	0	1
2	0	2	1	0	0
3	0	1	2	1	0
4	0	0	1	0	0
5	1	0	0	0	0

(8x**1**, 1x**2**) **d**

	1	2	3	4	5
1	0	0	0	0	1
2	0	0	1	0	0
3	0	1	2	1	0
4	0	0	1	0	0
5	1	0	0	0	2

(6x**1**, 2x$\mathbf{2}^{1}$)

	1	2	3	4	5
1	0	0	0	1	0
2	0	0	1	0	0
3	0	1	0	2^1	0
4	1	0	2^1	0	1
5	0	0	0	1	0

<table>
<tr><td></td><td>(6x**1**, 2x**2**^{2})<table><tr><td></td><td>1</td><td>2</td><td>3</td><td>4</td><td>5</td></tr><tr><td>1</td><td>0</td><td>0</td><td>0</td><td>1</td><td>0</td></tr><tr><td>2</td><td>0</td><td>0</td><td>1</td><td>0</td><td>0</td></tr><tr><td>3</td><td>0</td><td>1</td><td>0</td><td>2^2</td><td>0</td></tr><tr><td>4</td><td>1</td><td>0</td><td>2^2</td><td>0</td><td>1</td></tr><tr><td>5</td><td>0</td><td>0</td><td>0</td><td>1</td><td>0</td></tr></table></td><td>(4x**1**, 1x**2**, 2x**2**^{1})<table><tr><td></td><td>1</td><td>2</td><td>3</td><td>4</td><td>5</td></tr><tr><td>1</td><td>0</td><td>0</td><td>0</td><td>0</td><td>1</td></tr><tr><td>2</td><td>0</td><td>2</td><td>2^1</td><td>0</td><td>0</td></tr><tr><td>3</td><td>0</td><td>2^1</td><td>0</td><td>1</td><td>0</td></tr><tr><td>4</td><td>0</td><td>0</td><td>1</td><td>0</td><td>0</td></tr><tr><td>5</td><td>1</td><td>0</td><td>0</td><td>0</td><td>0</td></tr></table></td><td>(4x**1**, 1x**2**, 2x**2**^{2})<table><tr><td></td><td>1</td><td>2</td><td>3</td><td>4</td><td>5</td></tr><tr><td>1</td><td>0</td><td>0</td><td>0</td><td>0</td><td>1</td></tr><tr><td>2</td><td>0</td><td>2</td><td>2^2</td><td>0</td><td>0</td></tr><tr><td>3</td><td>0</td><td>2^2</td><td>0</td><td>1</td><td>0</td></tr><tr><td>4</td><td>0</td><td>0</td><td>1</td><td>0</td><td>0</td></tr><tr><td>5</td><td>1</td><td>0</td><td>0</td><td>0</td><td>0</td></tr></table></td><td></td><td></td></tr>
<tr><td>$^1V_3{}^3V_1{}^4V_2$</td><td colspan="5">NG</td></tr>
<tr><td>$^1V_3{}^3V_1{}^4V_3$</td><td colspan="5">NG</td></tr>
<tr><td>$^1V_3{}^3V_1{}^4V_4$</td><td colspan="5">NG</td></tr>
<tr><td>$^1V_3{}^3V_3{}^4V_1$</td><td colspan="5">NG</td></tr>
<tr><td>$^1V_3{}^3V_3{}^4V_2$</td><td colspan="5">NG</td></tr>
<tr><td>$^1V_4{}^3V_2{}^4V_1$</td><td colspan="5">NG</td></tr>
<tr><td>$^1V_4{}^3V_2{}^4V_2$</td><td colspan="5">NG</td></tr>
<tr><td>$^1V_5{}^3V_1{}^4V_1$</td><td colspan="5">NG</td></tr>
<tr><td>$^1V_5{}^3V_1{}^4V_2$</td><td colspan="5">NG</td></tr>
<tr><td>$\boldsymbol{^2V_r{}^3V_r{}^4V_r}$</td><td colspan="5"></td></tr>
<tr><td>$^2V_1{}^3V_2{}^4V_1$</td><td>(10x**1**, 1x**2**)<table><tr><td></td><td>1</td><td>2</td><td>3</td><td>4</td></tr><tr><td>1</td><td>0</td><td>1</td><td>0</td><td>1</td></tr><tr><td>2</td><td>1</td><td>0</td><td>1</td><td>1</td></tr><tr><td>3</td><td>0</td><td>1</td><td>2</td><td>1</td></tr><tr><td>4</td><td>1</td><td>1</td><td>1</td><td>0</td></tr></table></td><td>(8x**1**, 2x**2**)<table><tr><td></td><td>1</td><td>2</td><td>3</td><td>4</td></tr><tr><td>1</td><td>2</td><td>1</td><td>0</td><td>0</td></tr><tr><td>2</td><td>1</td><td>0</td><td>1</td><td>1</td></tr><tr><td>3</td><td>0</td><td>1</td><td>2</td><td>1</td></tr><tr><td>4</td><td>0</td><td>1</td><td>1</td><td>0</td></tr></table></td><td>(8x**1**, 2x**2**^{1})<table><tr><td></td><td>1</td><td>2</td><td>3</td><td>4</td></tr><tr><td>1</td><td>0</td><td>1</td><td>0</td><td>1</td></tr><tr><td>2</td><td>1</td><td>0</td><td>2^1</td><td>1</td></tr><tr><td>3</td><td>0</td><td>2^1</td><td>0</td><td>1</td></tr><tr><td>4</td><td>1</td><td>1</td><td>1</td><td>0</td></tr></table></td><td>(8x**1**, 2x**2**^{2})<table><tr><td></td><td>1</td><td>2</td><td>3</td><td>4</td></tr><tr><td>1</td><td>0</td><td>1</td><td>0</td><td>1</td></tr><tr><td>2</td><td>1</td><td>0</td><td>2^1</td><td>1</td></tr><tr><td>3</td><td>0</td><td>2^1</td><td>0</td><td>1</td></tr><tr><td>4</td><td>1</td><td>1</td><td>1</td><td>0</td></tr></table></td><td>(6x**1**, 3x**2**)<table><tr><td></td><td>1</td><td>2</td><td>3</td><td>4</td></tr><tr><td>1</td><td>0</td><td>1</td><td>0</td><td>1</td></tr><tr><td>2</td><td>1</td><td>2</td><td>1</td><td>0</td></tr><tr><td>3</td><td>0</td><td>1</td><td>2</td><td>0</td></tr><tr><td>4</td><td>1</td><td>0</td><td>0</td><td>2</td></tr></table></td></tr>
<tr><td></td><td>(6x**1**, 1x**2**, 2x**2**^{1}) **a**<table><tr><td></td><td>1</td><td>2</td><td>3</td><td>4</td></tr><tr><td>1</td><td>0</td><td>1</td><td>0</td><td>1</td></tr><tr><td>2</td><td>1</td><td>0</td><td>2^1</td><td>0</td></tr><tr><td>3</td><td>0</td><td>2^1</td><td>0</td><td>1</td></tr><tr><td>4</td><td>1</td><td>0</td><td>1</td><td>2</td></tr></table></td><td>(6x**1**, 1x**2**, 2x**2**^{1}) **b**<table><tr><td></td><td>1</td><td>2</td><td>3</td><td>4</td></tr><tr><td>1</td><td>2</td><td>1</td><td>0</td><td>0</td></tr><tr><td>2</td><td>1</td><td>0</td><td>2^1</td><td>1</td></tr><tr><td>3</td><td>0</td><td>2^1</td><td>0</td><td>1</td></tr><tr><td>4</td><td>0</td><td>1</td><td>1</td><td>0</td></tr></table></td><td>(6x**1**, 1x**2**, 2x**2**^{2}) **a**<table><tr><td></td><td>1</td><td>2</td><td>3</td><td>4</td></tr><tr><td>1</td><td>0</td><td>1</td><td>0</td><td>1</td></tr><tr><td>2</td><td>1</td><td>0</td><td>2^2</td><td>0</td></tr><tr><td>3</td><td>0</td><td>2^2</td><td>0</td><td>1</td></tr><tr><td>4</td><td>1</td><td>0</td><td>1</td><td>2</td></tr></table></td><td>(6x**1**, 1x**2**, 2x**2**^{2}) **b**<table><tr><td></td><td>1</td><td>2</td><td>3</td><td>4</td></tr><tr><td>1</td><td>2</td><td>1</td><td>0</td><td>0</td></tr><tr><td>2</td><td>1</td><td>0</td><td>2^2</td><td>1</td></tr><tr><td>3</td><td>0</td><td>2^2</td><td>0</td><td>1</td></tr><tr><td>4</td><td>0</td><td>1</td><td>1</td><td>0</td></tr></table></td><td>(4x**1**, 4x**2**)<table><tr><td></td><td>1</td><td>2</td><td>3</td><td>4</td></tr><tr><td>1</td><td>2</td><td>1</td><td>0</td><td>1</td></tr><tr><td>2</td><td>1</td><td>2</td><td>0</td><td>0</td></tr><tr><td>3</td><td>0</td><td>0</td><td>2</td><td>0</td></tr><tr><td>4</td><td>1</td><td>0</td><td>0</td><td>2</td></tr></table></td></tr>
<tr><td></td><td>(4x**1**, 2x**2**, 2x**2**^{1}) **a**<table><tr><td></td><td>1</td><td>2</td><td>3</td><td>4</td></tr><tr><td>1</td><td>0</td><td>1</td><td>0</td><td>1</td></tr><tr><td>2</td><td>1</td><td>0</td><td>2^1</td><td>0</td></tr><tr><td>3</td><td>0</td><td>2^1</td><td>2</td><td>0</td></tr><tr><td>4</td><td>1</td><td>0</td><td>0</td><td>2</td></tr></table></td><td>(4x**1**, 2x**2**, 2x**2**^{1}) **b**<table><tr><td></td><td>1</td><td>2</td><td>3</td><td>4</td></tr><tr><td>1</td><td>2</td><td>1</td><td>0</td><td>1</td></tr><tr><td>2</td><td>1</td><td>0</td><td>2^1</td><td>0</td></tr><tr><td>3</td><td>0</td><td>2^1</td><td>0</td><td>0</td></tr><tr><td>4</td><td>1</td><td>0</td><td>0</td><td>2</td></tr></table></td><td>(4x**1**, 2x**2**, 2x**2**^{1}) **c**<table><tr><td></td><td>1</td><td>2</td><td>3</td><td>4</td></tr><tr><td>1</td><td>2</td><td>1</td><td>0</td><td>0</td></tr><tr><td>2</td><td>1</td><td>0</td><td>2^1</td><td>1</td></tr><tr><td>3</td><td>0</td><td>2^1</td><td>0</td><td>0</td></tr><tr><td>4</td><td>0</td><td>1</td><td>0</td><td>2</td></tr></table></td><td>(4x**1**, 2x**2**, 2x**2**^{1}) **d**<table><tr><td></td><td>1</td><td>2</td><td>3</td><td>4</td></tr><tr><td>1</td><td>2</td><td>0</td><td>0</td><td>0</td></tr><tr><td>2</td><td>0</td><td>0</td><td>2^1</td><td>1</td></tr><tr><td>3</td><td>0</td><td>2^1</td><td>0</td><td>1</td></tr><tr><td>4</td><td>0</td><td>1</td><td>1</td><td>2</td></tr></table></td><td>(4x**1**, 2x**2**, 2x**2**^{2}) **a**<table><tr><td></td><td>1</td><td>2</td><td>3</td><td>4</td></tr><tr><td>1</td><td>0</td><td>1</td><td>0</td><td>1</td></tr><tr><td>2</td><td>1</td><td>0</td><td>2^2</td><td>0</td></tr><tr><td>3</td><td>0</td><td>2^2</td><td>2</td><td>0</td></tr><tr><td>4</td><td>1</td><td>0</td><td>0</td><td>2</td></tr></table></td></tr>
</table>

(4x**1**, 2x**2**, 2x$\mathbf{2}^{2}$) **b**

	1	2	3	4
1	2	1	0	1
2	1	0	2^2	0
3	0	2^2	0	0
4	1	0	0	2

(4x**1**, 2x**2**, 2x$\mathbf{2}^{2}$) **c**

	1	2	3	4
1	2	1	0	0
2	1	0	2^2	1
3	0	2^2	0	0
4	0	1	0	2

(4x**1**, 2x**2**, 2x$\mathbf{2}^{2}$) **d**

	1	2	3	4
1	2	0	0	0
2	0	0	2^2	1
3	0	2^2	0	1
4	0	1	1	2

(4x**1**, 4x$\mathbf{2}^{1}$) **a**

	1	2	3	4
1	0	1	0	1
2	1	0	2^1	0
3	0	2^1	0	2^1
4	1	0	2^1	0

(4x**1**, 4x$\mathbf{2}^{1}$) **b**

	1	2	3	4
1	0	2^1	0	0
2	2^1	0	1	1
3	0	1	0	2^1
4	0	1	2^1	0

(4x**1**, 2x$\mathbf{2}^{1}$, 2x$\mathbf{2}^{2}$) **a**

	1	2	3	4
1	0	1	0	1
2	1	0	2^1	0
3	0	2^1	0	2^2
4	1	0	2^2	0

(4x**1**, 2x$\mathbf{2}^{1}$, 2x$\mathbf{2}^{2}$) **b**

	1	2	3	4
1	0	2^2	0	0
2	2^2	0	1	1
3	0	1	0	2^1
4	0	1	2^1	0

(4x**1**, 2x$\mathbf{2}^{1}$, 2x$\mathbf{2}^{2}$) **c**

	1	2	3	4
1	0	2^1	0	0
2	2^1	0	1	1
3	0	1	0	2^2
4	0	1	2^2	0

(4x**1**, 4x$\mathbf{2}^{2}$) **a**

	1	2	3	4
1	0	1	0	1
2	1	0	2^2	0
3	0	2^2	0	2^2
4	1	0	2^2	0

(4x**1**, 4x$\mathbf{2}^{2}$) **b**

	1	2	3	4
1	0	2^2	0	0
2	2^2	0	1	1
3	0	1	0	2^2
4	0	1	2^2	0

(2x**1**, 3x**2**, 2x$\mathbf{2}^{1}$) **a**

	1	2	3	4
1	0	2^1	0	1
2	2^1	2	0	0
3	0	0	2	0
4	1	0	0	2

(2x**1**, 3x**2**, 2x$\mathbf{2}^{1}$) **b**

	1	2	3	4
1	2	1	0	0
2	1	2	0	0
3	0	0	2	2^1
4	0	0	2^1	0

(2x**1**, 3x**2**, 2x$\mathbf{2}^{2}$) **a**

	1	2	3	4
1	0	2^2	0	1
2	2^2	2	0	0
3	0	0	2	0
4	1	0	0	2

(2x**1**, 3x**2**, 2x$\mathbf{2}^{2}$) **b**

	1	2	3	4
1	2	1	0	0
2	1	2	0	0
3	0	0	2	2^2
4	0	0	2^2	0

(2x**1**, 1x**2**, 4x$\mathbf{2}^{1}$) **a**

	1	2	3	4
1	0	1	0	2^1
2	1	0	2^1	0
3	0	2^1	2	0
4	2^1	0	0	0

(2x**1**, 1x**2**, 4x$\mathbf{2}^{1}$) **b**

	1	2	3	4
1	0	2^1	0	2^1
2	2^1	0	1	0
3	0	1	2	0
4	2^1	0	0	0

(2x**1**, 1x**2**, 4x$\mathbf{2}^{1}$) **c**

	1	2	3	4
1	0	2^1	0	2^1
2	2^1	0	0	1
3	0	0	2	0
4	2^1	1	0	0

(2x**1**, 1x**2**, 2x$\mathbf{2}^{1}$, 2x$\mathbf{2}^{2}$) **a**

	1	2	3	4
1	0	1	0	2^2
2	1	0	2^1	0
3	0	2^1	2	0
4	2^2	0	0	0

(2x**1**, 1x**2**, 2x$\mathbf{2}^{1}$, 2x$\mathbf{2}^{2}$) **b**

	1	2	3	4
1	0	1	0	2^1
2	1	0	2^2	0
3	0	2^2	2	0
4	2^1	0	0	0

(2x**1**, 1x**2**, 2x$\mathbf{2}^{1}$, 2x$\mathbf{2}^{2}$) **c**

	1	2	3	4
1	0	2^1	0	2^2
2	2^1	0	1	0
3	0	1	2	0
4	2^2	0	0	0

(2x**1**, 1x**2**, 2x$\mathbf{2}^{1}$, 2x$\mathbf{2}^{2}$) **d**

	1	2	3	4
1	0	2^2	0	2^1
2	2^2	0	1	0
3	0	1	2	0
4	2^1	0	0	0

(2x**1**, 1x**2**, 2x$\mathbf{2}^{1}$, 2x$\mathbf{2}^{2}$) **e**

	1	2	3	4
1	0	2^2	0	2^1
2	2^2	0	0	1
3	0	0	2	0
4	2^1	1	0	0

(2x**1**, 1x**2**, 4x$\mathbf{2}^{2}$) **a**

	1	2	3	4
1	0	1	0	2^2
2	1	0	2^2	0
3	0	2^2	2	0
4	2^2	0	0	0

(2x**1**, 1x**2**, 4x$\mathbf{2}^{2}$) **b**

	1	2	3	4
1	0	2^2	0	2^2
2	2^2	0	1	0
3	0	1	2	0
4	2^2	0	0	0

(2x**1**, 1x**2**, 4x$\mathbf{2}^{2}$) **c**

	1	2	3	4
1	0	2^2	0	2^2
2	2^2	0	0	1
3	0	0	2	0
4	2^2	1	0	0

${}^{2}V_1{}^{3}V_2{}^{4}V_2$	NG
${}^{2}V_1{}^{3}V_2{}^{4}V_3$	NG
${}^{2}V_1{}^{3}V_2{}^{4}V_4$	NG
${}^{2}V_1{}^{3}V_2{}^{4}V_5$	NG
${}^{2}V_1{}^{3}V_4{}^{4}V_1$	NG
${}^{2}V_1{}^{3}V_4{}^{4}V_2$	NG
${}^{2}V_1{}^{3}V_4{}^{4}V_3$	NG
${}^{2}V_1{}^{3}V_6{}^{4}V_1$	NG

$^{2}V_{2}{}^{3}V_{2}{}^{4}V_{1}$

(14x**1**)

	1	2	3	4	5
1	0	1	0	1	0
2	1	0	1	1	1
3	0	1	0	0	1
4	1	1	0	0	1
5	0	1	1	1	0

(12x**1**, 1x**2**) **a**

	1	2	3	4	5
1	0	1	0	1	0
2	1	0	1	1	1
3	0	1	0	1	0
4	1	1	1	0	0
5	0	1	0	0	2

(12x**1**, 1x**2**) **b**

	1	2	3	4	5
1	0	1	0	1	0
2	1	0	1	0	1
3	0	1	0	1	1
4	1	0	1	2	0
5	0	1	1	0	0

(12x**1**, 1x**2**) **c**

	1	2	3	4	5
1	0	1	0	1	0
2	1	0	1	0	1
3	0	1	0	1	1
4	1	0	1	0	0
5	0	1	1	0	2

(12x**1**, 1x**2**) **d**

	1	2	3	4	5
1	0	1	0	1	0
2	1	0	1	1	1
3	0	1	2	0	0
4	1	1	0	0	1
5	0	1	0	1	0

(10x**1**, 2x**2**) **a**

	1	2	3	4	5
1	0	1	0	1	0
2	1	0	1	1	1
3	0	1	2	0	0
4	1	1	0	0	0
5	0	1	0	0	2

(10x**1**, 2x**2**) **b**

	1	2	3	4	5
1	0	1	0	1	0
2	1	0	1	0	1
3	0	1	0	1	0
4	1	0	1	2	0
5	0	1	0	0	2

(10x**1**, 2x**2**) **c**

	1	2	3	4	5
1	0	1	0	1	0
2	1	0	1	0	1
3	0	1	2	1	0
4	1	0	1	0	0
5	0	1	0	0	2

(10x**1**, 2x**2**) **d**

	1	2	3	4	5
1	0	1	0	1	0
2	1	0	1	1	0
3	0	1	2	1	0
4	1	1	1	0	0
5	0	0	0	0	2

(10x**1**, 2x**2**) **e**

	1	2	3	4	5
1	0	1	0	1	0
2	1	0	0	1	1
3	0	0	2	0	1
4	1	1	0	2	0
5	0	1	1	0	0

(10x**1**, 2x**2**) **f**

	1	2	3	4	5
1	0	1	0	1	0
2	1	0	0	1	1
3	0	0	2	0	1
4	1	1	0	0	0
5	0	1	1	0	2

(10x**1**, 2x**2**^{1}) **a**

	1	2	3	4	5
1	0	1	0	1	0
2	1	0	1	0	1
3	0	1	0	1	2^1
4	1	0	1	0	0
5	0	1	2^1	0	0

(10x**1**, 2x**2**^{1}) **b**

	1	2	3	4	5
1	0	1	0	1	0
2	1	0	1	0	1
3	0	1	0	2^1	1
4	1	0	2^1	0	0
5	0	1	1	0	0

(10x**1**, 2x**2**^{1}) **c**

	1	2	3	4	5
1	0	1	1	1	0
2	1	0	1	0	2^1
3	1	1	0	1	0
4	1	0	1	0	0
5	0	2^1	0	0	0

(10x**1**, 2x**2**^{1}) **d**

	1	2	3	4	5
1	0	1	0	1	0
2	1	0	1	0	1
3	0	1	0	1	0
4	1	0	1	0	2^1
5	0	1	0	2^1	0

(10x**1**, 2x**2**^{1}) **e**

	1	2	3	4	5
1	0	1	1	1	1
2	1	0	2^1	0	0
3	1	2^1	0	0	0
4	1	0	0	0	1
5	1	0	0	1	0

(10x**1**, 2x**2**^{2}) **a**

	1	2	3	4	5
1	0	1	0	1	0
2	1	0	1	0	1
3	0	1	0	1	2^2
4	1	0	1	0	0
5	0	1	2^2	0	0

(10x**1**, 2x**2**^{2}) **b**

	1	2	3	4	5
1	0	1	0	1	0
2	1	0	1	0	1
3	0	1	0	2^2	1
4	1	0	2^2	0	0
5	0	1	1	0	0

(10x**1**, 2x**2**^{2}) **c**

	1	2	3	4	5
1	0	1	1	1	0
2	1	0	1	0	2^2
3	1	1	0	1	0
4	1	0	1	0	0
5	0	2^2	0	0	0

(10x**1**, 2x**2**^{2}) **d**

	1	2	3	4	5
1	0	1	0	1	0
2	1	0	1	0	1
3	0	1	0	1	0
4	1	0	1	0	2^2
5	0	1	0	2^2	0

(10x**1**, 2x**2**^{2}) **e**

	1	2	3	4	5
1	0	1	1	1	1
2	1	0	2^2	0	0
3	1	2^2	0	0	0
4	1	0	0	0	1
5	1	0	0	1	0

(8x**1**, 3x**2**) **a**

	1	2	3	4	5
1	0	1	0	1	0
2	1	2	0	0	1
3	0	0	2	0	1
4	1	0	0	2	0
5	0	1	1	0	0

(8x**1**, 3x**2**) **b**

	1	2	3	4	5
1	0	1	0	1	0
2	1	0	0	0	1
3	0	0	2	0	1
4	1	0	0	2	0
5	0	1	1	0	2

(8x**1**, 3x**2**) **c**

	1	2	3	4	5
1	0	1	0	1	0
2	1	2	0	1	0
3	0	0	2	0	1
4	1	1	0	0	0
5	0	0	1	0	2

(8x**1**, 3x**2**) **d**

	1	2	3	4	5
1	0	1	0	1	0
2	1	0	0	1	1
3	0	0	2	0	0
4	1	1	0	2	0
5	0	1	0	0	2

(8x**1**, 1x**2**, 2x**2**^{1}) **a**

	1	2	3	4	5
1	0	2^1	0	1	0
2	2^1	0	0	1	1
3	0	0	2	0	1
4	1	1	0	0	0
5	0	1	1	0	0

(8x**1**, 1x**2**, 2x**2**^{1}) **b**

	1	2	3	4	5
1	0	1	0	1	0
2	1	0	0	1	2^1
3	0	0	2	0	1
4	1	1	0	0	0
5	0	2^1	1	0	0

(8x**1**, 1x**2**, 2x**2**^{1}) **c**

	1	2	3	4	5
1	0	1	0	1	0
2	1	0	0	1	1
3	0	0	0	0	2^1
4	1	1	0	2	0
5	0	1	2^1	0	0

(8x**1**, 1x**2**, 2x**2**^{1}) **d**

	1	2	3	4	5
1	0	1	0	1	0
2	1	0	0	1	1
3	0	0	2	0	2^1
4	1	1	0	0	0
5	0	1	2^1	0	0

(8x**1**, 1x**2**, 2x**2**^{1}) **e**

	1	2	3	4	5
1	0	1	0	1	0
2	1	0	0	1	2^1
3	0	0	2	1	0
4	1	1	1	0	0
5	0	2^1	0	0	0

(8x**1**, 1x**2**, 2x**2**^{1}) **f**

	1	2	3	4	5
1	0	2^1	0	1	0
2	2^1	0	1	0	1
3	0	1	0	1	0
4	1	0	1	0	0
5	0	1	0	0	2

(8x**1**, 1x**2**, 2x**2**^{1}) **g**

	1	2	3	4	5
1	0	2^1	0	1	0
2	2^1	0	1	1	0
3	0	1	0	1	0
4	1	1	1	0	0
5	0	0	0	0	2

(8x**1**, 1x**2**, 2x**2**^{1}) **h**

	1	2	3	4	5
1	0	1	0	2^1	0
2	1	0	0	0	1
3	0	0	0	1	1
4	2^1	0	1	0	0
5	0	1	1	0	2

(8x**1**, 1x**2**, 2x**2**^{1}) **i**

	1	2	3	4	5
1	0	1	0	1	0
2	1	0	0	0	1
3	0	0	0	2^1	1
4	1	0	2^1	0	0
5	0	1	1	0	2

(8x**1**, 1x**2**, 2x**2**^{2}) **a**

	1	2	3	4	5
1	0	2^2	0	1	0
2	2^2	0	0	1	1
3	0	0	2	0	1
4	1	1	0	0	0
5	0	1	1	0	0

(8x**1**, 1x**2**, 2x**2**^{2}) **b**

	1	2	3	4	5
1	0	1	0	1	0
2	1	0	0	1	2^2
3	0	0	2	0	1
4	1	1	0	0	0
5	0	2^2	1	0	0

(8x**1**, 1x**2**, 2x**2**^{2}) **c**

	1	2	3	4	5
1	0	1	0	1	0
2	1	0	0	1	1
3	0	0	0	0	2^2
4	1	1	0	2	0
5	0	1	2^2	0	0

(8x**1**, 1x**2**, 2x**2**^{2}) **d**

	1	2	3	4	5
1	0	1	0	1	0
2	1	0	0	1	1
3	0	0	2	0	2^2
4	1	1	0	0	0
5	0	1	2^2	0	0

(8x**1**, 1x**2**, 2x**2**^{2}) **e**

	1	2	3	4	5
1	0	1	0	1	0
2	1	0	0	1	2^2
3	0	0	2	1	0
4	1	1	1	0	0
5	0	2^2	0	0	0

(8x**1**, 1x**2**, 2x**2**^{2}) **f**

	1	2	3	4	5
1	0	2^2	0	1	0
2	2^2	0	1	0	1
3	0	1	0	1	0
4	1	0	1	0	0
5	0	1	0	0	2

(8x**1**, 1x**2**, 2x**2**^{2}) **g**

	1	2	3	4	5
1	0	2^2	0	1	0
2	2^2	0	1	1	0
3	0	1	0	1	0
4	1	1	1	0	0
5	0	0	0	0	2

(8x**1**, 1x**2**, 2x**2**^{2}) **h**

	1	2	3	4	5
1	0	1	0	2^2	0
2	1	0	0	0	1
3	0	0	0	1	1
4	2^2	0	1	0	0
5	0	1	1	0	2

(8x**1**, 1x**2**, 2x**2**^{2}) **i**

	1	2	3	4	5
1	0	1	0	1	0
2	1	0	0	0	1
3	0	0	0	2^2	1
4	1	0	2^2	0	0
5	0	1	1	0	2

(6x**1**, 4x**2**)

	1	2	3	4	5
1	0	1	0	1	0
2	1	2	0	0	1
3	0	0	2	0	0
4	1	0	0	2	0
5	0	1	0	0	2

(6x**1**, 2x**2**, 2x**2**^{1}) **a**

	1	2	3	4	5
1	0	2^1	0	1	0
2	2^1	0	0	1	1
3	0	0	2	0	0
4	1	1	0	0	0
5	0	1	0	0	2

(6x**1**, 2x**2**, 2x**2**^{1}) **b**

	1	2	3	4	5
1	0	2^1	0	0	0
2	2^1	0	0	1	1
3	0	0	2	0	1
4	0	1	0	2	0
5	0	1	1	0	0

(6x**1**, 2x**2**, 2x**2**^{1}) **c**

	1	2	3	4	5
1	0	2^1	0	1	0
2	2^1	0	1	0	0
3	0	1	2	1	0
4	1	0	1	0	0
5	0	0	0	0	2

(6x**1**, 2x**2**, 2x**2**^{1}) **d**

	1	2	3	4	5
1	0	1	0	2^1	0
2	1	2	0	0	1
3	0	0	2	0	1
4	2^1	0	0	0	0
5	0	1	1	0	0

(6x**1**, 2x**2**, 2x**2**^{1}) **e**

	1	2	3	4	5
1	0	1	0	2^1	0
2	1	0	0	0	1
3	0	0	2	0	1
4	2^1	0	0	0	0
5	0	1	1	0	2

(6x**1**, 2x**2**, 2x**2**^{1}) **f**

	1	2	3	4	5
1	0	1	0	2^1	0
2	1	0	0	0	1
3	0	0	2	0	1
4	2^1	0	0	2	0
5	0	1	1	0	0

(6x**1**, 2x**2**, 2x**2**^{2}) **a**

	1	2	3	4	5
1	0	2^2	0	1	0
2	2^2	0	0	1	1
3	0	0	2	0	0
4	1	1	0	0	0
5	0	1	0	0	2

(6x**1**, 2x**2**, 2x**2**^{2}) **b**

	1	2	3	4	5
1	0	2^2	0	0	0
2	2^2	0	0	1	1
3	0	0	2	0	1
4	0	1	0	2	0
5	0	1	1	0	0

(6x**1**, 2x**2**, 2x**2**^{2}) **c**

	1	2	3	4	5
1	0	2^2	0	1	0
2	2^2	0	1	0	0
3	0	1	2	1	0
4	1	0	1	0	0
5	0	0	0	0	2

(6x**1**, 2x**2**, 2x**2**^{2}) **d**

	1	2	3	4	5
1	0	1	0	2^2	0
2	1	2	0	0	1
3	0	0	2	0	1
4	2^2	0	0	0	0
5	0	1	1	0	0

(6x**1**, 2x**2**, 2x**2**^{2}) **e**

	1	2	3	4	5
1	0	1	0	2^2	0
2	1	0	0	0	1
3	0	0	2	0	1
4	2^2	0	0	0	0
5	0	1	1	0	2

(6x**1**, 2x**2**, 2x**2**^{2}) **f**

	1	2	3	4	5
1	0	1	0	2^2	0
2	1	0	0	0	1
3	0	0	2	0	1
4	2^2	0	0	2	0
5	0	1	1	0	0

(6x**1**, 4x**2**^{1}) **a**

	1	2	3	4	5
1	0	2^1	0	1	0
2	2^1	0	0	1	1
3	0	0	0	0	2^1
4	1	1	0	0	0
5	0	1	2^1	0	0

(6x**1**, 4x**2**^{1}) **b**

	1	2	3	4	5
1	0	1	0	2^1	0
2	1	0	1	0	2^1
3	0	1	0	1	0
4	2^1	0	1	0	0
5	0	2^1	0	0	0

(6x**1**, 4x**2**^{1}) **c**

	1	2	3	4	5
1	0	1	0	2^1	0
2	1	0	0	0	1
3	0	0	0	2^1	1
4	2^1	0	2^1	0	0
5	0	1	1	0	0

(6x**1**, 2x**2**^{1}, 2x**2**^{2}) **a**

	1	2	3	4	5
1	0	2^2	0	1	0
2	2^2	0	0	1	1
3	0	0	0	0	2^1
4	1	1	0	0	0
5	0	1	2^1	0	0

(6x**1**, 2x**2**^{1}, 2x**2**^{2}) **b**

	1	2	3	4	5
1	0	2^1	0	1	0
2	2^1	0	0	1	1
3	0	0	0	0	2^2
4	1	1	0	0	0
5	0	1	2^2	0	0

(6x**1**, 2x**2**^{1}, 2x**2**^{2}) **c**

	1	2	3	4	5
1	0	1	0	2^2	0
2	1	0	1	0	2^1
3	0	1	0	1	0
4	2^2	0	1	0	0
5	0	2^1	0	0	0

(6x**1**, 2x**2**^{1}, 2x**2**^{2}) **d**

	1	2	3	4	5
1	0	1	0	2^1	0
2	1	0	1	0	2^2
3	0	1	0	1	0
4	2^1	0	1	0	0
5	0	2^2	0	0	0

(6x**1**, 2x**2**^{1}, 2x**2**^{2}) **e**

	1	2	3	4	5
1	0	1	0	2^2	0
2	1	0	0	0	1
3	0	0	0	2^1	1
4	2^2	0	2^1	0	0
5	0	1	1	0	0

(6x**1**, 4x**2**^{2}) **a**

	1	2	3	4	5
1	0	2^2	0	1	0
2	2^2	0	0	1	1
3	0	0	0	0	2^2
4	1	1	0	0	0
5	0	1	2^2	0	0

(6x**1**, 4x**2**^{2}) **b**

	1	2	3	4	5
1	0	1	0	2^2	0
2	1	0	1	0	2^2
3	0	1	0	1	0
4	2^2	0	1	0	0
5	0	2^2	0	0	0

(6x**1**, 4x**2**^{2}) **c**

	1	2	3	4	5
1	0	1	0	2^2	0
2	1	0	0	0	1
3	0	0	0	2^2	1
4	2^2	0	2^2	0	0
5	0	1	1	0	0

(4x**1**, 5x**2**)

	1	2	3	4	5
1	2	1	0	1	0
2	1	2	0	0	0
3	0	0	2	0	0
4	1	0	0	2	0
5	0	0	0	0	2

(4x**1**, 3x**2**, 2x**2**^{1}) **a**

	1	2	3	4	5
1	2	1	0	1	0
2	1	2	0	0	0
3	0	0	0	0	2^1
4	1	0	0	2	0
5	0	0	2^1	0	0

(4x**1**, 3x**2**, 2x**2**^{1}) **b**

	1	2	3	4	5
1	0	1	0	1	0
2	1	2	0	0	0
3	0	0	2	0	2^1
4	1	0	0	2	0
5	0	0	2^1	0	0

(4x**1**, 3x**2**, 2x**2**^{1}) **c**

	1	2	3	4	5
1	0	1	0	2^1	0
2	1	2	0	0	1
3	0	0	2	0	0
4	2^1	0	0	0	0
5	0	1	0	0	2

(4x**1**, 3x**2**, 2x**2**^{1}) **d**

	1	2	3	4	5
1	0	1	0	2^1	0
2	1	0	0	0	1
3	0	0	2	0	0
4	2^1	0	0	2	0
5	0	1	0	0	2

(4x**1**, 3x**2**, 2x**2**^{1}) **e**

	1	2	3	4	5
1	0	1	0	2^1	0
2	1	2	0	1	0
3	0	0	2	0	0
4	2^1	1	0	0	0
5	0	0	0	0	2

(4x**1**, 3x**2**, 2x**2**^{1}) **f**

	1	2	3	4	5
1	2	0	0	0	1
2	0	2	0	0	1
3	0	0	2	0	0
4	0	0	0	0	2^1
5	1	1	0	2^1	0

(4x**1**, 3x**2**, 2x**2**^{2}) **a**

	1	2	3	4	5
1	2	1	0	1	0
2	1	2	0	0	0
3	0	0	0	0	2^2
4	1	0	0	2	0
5	0	0	2^2	0	0

(4x**1**, 3x**2**, 2x**2**^{2}) **b**

	1	2	3	4	5
1	0	1	0	1	0
2	1	2	0	0	0
3	0	0	2	0	2^2
4	1	0	0	2	0
5	0	0	2^2	0	0

(4x**1**, 3x**2**, 2x**2**^{2}) **c**

	1	2	3	4	5
1	0	1	0	2^2	0
2	1	2	0	0	1
3	0	0	2	0	0
4	2^2	0	0	0	0
5	0	1	0	0	2

(4x**1**, 3x**2**, 2x**2**^{2}) **d**

	1	2	3	4	5
1	0	1	0	2^1	0
2	1	0	0	0	1
3	0	0	2	0	0
4	2^2	0	0	2	0
5	0	1	0	0	2

(4x**1**, 3x**2**, 2x**2**^{2}) **e**

	1	2	3	4	5
1	0	1	0	2^2	0
2	1	2	0	1	0
3	0	0	2	0	0
4	2^2	1	0	0	0
5	0	0	0	0	2

(4x**1**, 3x**2**, 2x**2**^{2}) **f**

	1	2	3	4	5
1	2	0	0	0	1
2	0	2	0	0	1
3	0	0	2	0	0
4	0	0	0	0	2^2
5	1	1	0	2^2	0

(4x**1**, 1x**2**, 4x**2**^{1}) **a**

	1	2	3	4	5
1	0	2^1	0	2^1	0
2	2^1	0	0	0	1
3	0	0	2	0	1
4	2^1	0	0	0	0
5	0	1	1	0	0

(4x**1**, 1x**2**, 4x**2**^{1}) **b**

	1	2	3	4	5
1	0	1	0	2^1	0
2	1	2	0	0	1
3	0	0	0	0	2^1
4	2^1	0	0	0	0
5	0	1	2^1	0	0

(4x**1**, 1x**2**, 4x**2**^{1}) **c**

	1	2	3	4	5
1	0	1	0	2^1	0
2	1	0	0	0	1
3	0	0	0	0	2^1
4	2^1	0	0	2	0
5	0	1	2^1	0	0

(4x**1**, 1x**2**, 4x**2**^{1}) **d**

	1	2	3	4	5
1	0	1	0	2^1	0
2	1	2	0	1	0
3	0	0	0	0	2^1
4	2^1	1	0	0	0
5	0	0	2^1	0	0

(4x**1**, 1x**2**, 4x**2**^{1}) **e**

	1	2	3	4	5
1	0	1	0	2^1	0
2	1	0	0	1	0
3	0	0	0	0	2^1
4	2^1	1	0	0	0
5	0	0	2^1	0	2

(4x**1**, 1x**2**, 4x**2**^{1}) **f**

	1	2	3	4	5
1	0	1	0	2^1	0
2	1	0	0	1	2^1
3	0	0	2	0	0
4	2^1	1	0	0	0
5	0	2^1	0	0	0

(4x**1**, 1x**2**, 4x**2**^{1}) **g**

	1	2	3	4	5
1	0	2^1	0	0	0
2	2^1	0	0	1	1
3	0	0	0	0	2^1
4	0	1	0	2	0
5	0	1	2^1	0	0

(4x**1**, 1x**2**, 4x**2**^{1}) **h**

	1	2	3	4	5
1	0	2^1	0	2^1	0
2	2^1	0	0	0	1
3	0	0	2	0	0
4	2^1	0	0	0	1
5	0	1	0	1	0

(4x**1**, 1x**2**, 2x**2**^{1}, 2x**2**^{2}) **a**

	1	2	3	4	5
1	0	2^1	0	2^2	0
2	2^1	0	0	0	1
3	0	0	2	0	1
4	2^2	0	0	0	0
5	0	1	1	0	0

(4x**1**, 1x**2**, 2x**2**^{1}, 2x**2**^{2}) **b**

	1	2	3	4	5
1	0	2^2	0	2^1	0
2	2^2	0	0	0	1
3	0	0	2	0	1
4	2^1	0	0	0	0
5	0	1	1	0	0

(4x**1**, 1x**2**, 2x**2**^{1}, 2x**2**^{2}) **c**

	1	2	3	4	5
1	0	1	0	2^1	0
2	1	2	0	0	1
3	0	0	0	0	2^2
4	2^1	0	0	0	0
5	0	1	2^2	0	0

(4x**1**, 1x**2**, 2x**2**^{1}, 2x**2**^{2}) **d**

	1	2	3	4	5
1	0	1	0	2^1	0
2	1	0	0	0	1
3	0	0	0	0	2^2
4	2^1	0	0	2	0
5	0	1	2^2	0	0

(4x**1**, 1x**2**, 2x**2**^{1}, 2x**2**^{2}) **e**

	1	2	3	4	5
1	0	1	0	2^2	0
2	1	0	0	0	1
3	0	0	0	0	2^1
4	2^2	0	0	2	0
5	0	1	2^1	0	0

(4x**1**, 1x**2**, 2x**2**^{1}, 2x**2**^{2}) **f**

	1	2	3	4	5
1	0	1	0	2^2	0
2	1	2	0	1	0
3	0	0	0	0	2^1
4	2^2	1	0	0	0
5	0	0	2^1	0	0

(4x**1**, 1x**2**, 2x**2**^{1}, 2x**2**^{2}) **g**

	1	2	3	4	5
1	0	1	0	2^1	0
2	1	2	0	1	0
3	0	0	0	0	2^2
4	2^1	1	0	0	0
5	0	0	2^2	0	0

(4x**1**, 1x**2**, 2x**2**^{1}, 2x**2**^{2}) **h**

	1	2	3	4	5
1	0	1	0	2^2	0
2	1	0	0	1	0
3	0	0	0	0	2^1
4	2^2	1	0	0	0
5	0	0	2^1	0	2

(4x**1**, 1x**2**, 2x**2**^{1}, 2x**2**^{2}) **i**

	1	2	3	4	5
1	0	1	0	2^1	0
2	1	0	0	1	0
3	0	0	0	0	2^2
4	2^1	1	0	0	0
5	0	0	2^2	0	2

(4x**1**, 1x**2**, 2x**2**^{1}, 2x**2**^{2}) **j**

	1	2	3	4	5
1	0	1	0	2^2	0
2	1	0	0	1	2^1
3	0	0	2	0	0
4	2^2	1	0	0	0
5	0	2^1	0	0	0

(4x**1**, 1x**2**, 2x**2**^{1}, 2x**2**^{2}) **k**

	1	2	3	4	5
1	0	1	0	2^1	0
2	1	0	0	1	2^2
3	0	0	2	0	0
4	2^1	1	0	0	0
5	0	2^2	0	0	0

(4x**1**, 1x**2**, 2x**2**^{1}, 2x**2**^{2}) **l**

	1	2	3	4	5
1	0	2^2	0	0	0
2	2^2	0	0	1	1
3	0	0	0	0	2^1
4	0	1	0	2	0
5	0	1	2^1	0	0

(4x**1**, 1x**2**, 2x**2**^{1}, 2x**2**^{2}) **m**

	1	2	3	4	5
1	0	2^1	0	0	0
2	2^1	0	0	1	1
3	0	0	0	0	2^2
4	0	1	0	2	0
5	0	1	2^2	0	0

(4x**1**, 1x**2**, 2x**2**^{1}, 2x**2**^{2}) **n**

	1	2	3	4	5
1	0	2^1	0	2^1	0
2	2^1	0	0	0	1
3	0	0	2	0	0
4	2^1	0	0	0	1
5	0	1	0	1	0

(4x**1**, 1x**2**, 4x**2**^{2}) **a**

	1	2	3	4	5
1	0	2^2	0	2^2	0
2	2^2	0	0	0	1
3	0	0	2	0	1
4	2^2	0	0	0	0
5	0	1	1	0	0

(4x**1**, 1x**2**, 4x**2**^{2}) **b**

	1	2	3	4	5
1	0	1	0	2^2	0
2	1	2	0	0	1
3	0	0	0	0	2^2
4	2^2	0	0	0	0
5	0	1	2^2	0	0

(4x**1**, 1x**2**, 4x**2**^{2}) **c**

	1	2	3	4	5
1	0	1	0	2^2	0
2	1	0	0	0	1
3	0	0	0	0	2^2
4	2^2	0	0	2	0
5	0	1	2^2	0	0

(4x**1**, 1x**2**, 4x**2**^{2}) **d**

	1	2	3	4	5
1	0	1	0	2^2	0
2	1	2	0	1	0
3	0	0	0	0	2^2
4	2^2	1	0	0	0
5	0	0	2^2	0	0

(4x**1**, 1x**2**, 4x**2**^{2}) **e**

	1	2	3	4	5
1	0	1	0	2^2	0
2	1	0	0	1	0
3	0	0	0	0	2^2
4	2^2	1	0	0	0
5	0	0	2^2	0	2

(4x**1**, 1x**2**, 4x**2**^{2}) **f**

	1	2	3	4	5
1	0	1	0	2^2	0
2	1	0	0	1	2^2
3	0	0	2	0	0
4	2^2	1	0	0	0
5	0	2^2	0	0	0

(4x**1**, 1x**2**, 4x**2**^{2}) **g**

	1	2	3	4	5
1	0	2^2	0	0	0
2	2^2	0	0	1	1
3	0	0	0	0	2^2
4	0	1	0	2	0
5	0	1	2^2	0	0

(4x**1**, 1x**2**, 4x**2**^{2}) **h**

	1	2	3	4	5
1	0	2^2	0	2^2	0
2	2^2	0	0	0	1
3	0	0	2	0	0
4	2^2	0	0	0	1
5	0	1	0	1	0

(2x**1**, 4x**2**, 2x**2**^{1}) **a**

	1	2	3	4	5
1	0	2^1	0	1	0
2	2^1	2	0	0	0
3	0	0	2	0	0
4	1	0	0	2	0
5	0	0	0	0	2

(2x**1**, 4x**2**, 2x**2**^{1}) **b**

	1	2	3	4	5
1	0	2^1	0	0	0
2	2^1	2	0	0	0
3	0	0	2	0	0
4	0	0	0	2	1
5	0	0	0	1	2

(2x**1**, 4x**2**, 2x**2**^{2}) **a**

	1	2	3	4	5
1	0	2^2	0	1	0
2	2^2	2	0	0	0
3	0	0	2	0	0
4	1	0	0	2	0
5	0	0	0	0	2

(2x**1**, 4x**2**, 2x**2**^{2}) **b**

	1	2	3	4	5
1	0	2^2	0	0	0
2	2^2	2	0	0	0
3	0	0	2	0	0
4	0	0	0	2	1
5	0	0	0	1	2

(2x**1**, 2x**2**, 4x**2**^{1}) **a**

	1	2	3	4	5
1	0	2^1	0	1	0
2	2^1	2	0	0	0
3	0	0	0	0	2^1
4	1	0	0	2	0
5	0	0	2^1	0	0

(2x**1**, 2x**2**, 4x**2**^{1}) **b**

	1	2	3	4	5
1	0	2^1	0	1	0
2	2^1	0	0	0	0
3	0	0	2	0	2^1
4	1	0	0	2	0
5	0	0	2^1	0	0

(2x**1**, 2x**2**, 4x**2**^{1}) **c**

	1	2	3	4	5
1	0	1	0	2^1	0
2	1	0	2^1	0	0
3	0	2^1	0	0	0
4	2^1	0	0	2	0
5	0	0	0	0	2

(2x**1**, 2x**2**, 4x**2**^{1}) **d**

	1	2	3	4	5
1	0	2^1	0	1	0
2	2^1	0	2^1	0	0
3	0	2^1	0	0	0
4	1	0	0	2	0
5	0	0	0	0	2

(2x**1**, 2x**2**, 4x**2**^{1}) **e**

	1	2	3	4	5
1	0	2^1	0	0	0
2	2^1	0	2^1	0	0
3	0	2^1	0	0	0
4	0	0	0	2	1
5	0	0	0	1	2

(2x**1**, 2x**2**, 4x**2**^{1}) **f**

	1	2	3	4	5
1	0	2^1	1	0	0
2	2^1	0	2^1	0	0
3	1	2^1	0	0	0
4	0	0	0	2	0
5	0	0	0	0	2

(2x**1**, 2x**2**, 2x**2**^{1}, 2x**2**^{2}) **a**

	1	2	3	4	5
1	0	2^1	0	1	0
2	2^1	2	0	0	0
3	0	0	0	0	2^2
4	1	0	0	2	0
5	0	0	2^2	0	0

(2x**1**, 2x**2**, 2x**2**^{1}, 2x**2**^{2}) **b**

	1	2	3	4	5
1	0	2^2	0	1	0
2	2^2	2	0	0	0
3	0	0	0	0	2^1
4	1	0	0	2	0
5	0	0	2^1	0	0

(2x**1**, 2x**2**, 2x**2**^{1}, 2x**2**^{2}) **c**

	1	2	3	4	5
1	0	2^2	0	1	0
2	2^2	0	0	0	0
3	0	0	2	0	2^1
4	1	0	0	2	0
5	0	0	2^1	0	0

(2x**1**, 2x**2**, 2x**2**^{1}, 2x**2**^{2}) **d**

	1	2	3	4	5
1	0	2^1	0	1	0
2	2^1	0	0	0	0
3	0	0	2	0	2^2
4	1	0	0	2	0
5	0	0	2^2	0	0

(2x**1**, 2x**2**, 2x**2**^{1}, 2x**2**^{2}) **e**

	1	2	3	4	5
1	0	1	0	2^2	0
2	1	0	2^1	0	0
3	0	2^1	0	0	0
4	2^2	0	0	2	0
5	0	0	0	0	2

(2x**1**, 2x**2**, 2x**2**^{1}, 2x**2**^{2}) **f**

	1	2	3	4	5
1	0	1	0	2^1	0
2	1	0	2^2	0	0
3	0	2^2	0	0	0
4	2^1	0	0	2	0
5	0	0	0	0	2

(2x**1**, 2x**2**, 2x**2**^{1}, 2x**2**^{2}) **g**

	1	2	3	4	5
1	0	2^2	0	1	0
2	2^2	0	2^1	0	0
3	0	2^1	0	0	0
4	1	0	0	2	0
5	0	0	0	0	2

(2x**1**, 2x**2**, 2x**2**^{1}, 2x**2**^{2}) **h**

	1	2	3	4	5
1	0	2^1	0	1	0
2	2^1	0	2^2	0	0
3	0	2^2	0	0	0
4	1	0	0	2	0
5	0	0	0	0	2

(2x**1**, 2x**2**, 2x**2**^{1}, 2x**2**^{2}) **i**

	1	2	3	4	5
1	0	2^2	0	0	0
2	2^2	0	2^1	0	0
3	0	2^1	0	0	0
4	0	0	0	2	1
5	0	0	0	1	2

(2x**1**, 2x**2**, 2x**2**^{1}, 2x**2**^{2}) **j**

	1	2	3	4	5
1	0	2^1	1	0	0
2	2^1	0	2^2	0	0
3	1	2^2	0	0	0
4	0	0	0	2	0
5	0	0	0	0	2

(2x**1**, 2x**2**, 4x**2**^{2}) **a**

	1	2	3	4	5
1	0	2^2	0	1	0
2	2^2	2	0	0	0
3	0	0	0	0	2^2
4	1	0	0	2	0
5	0	0	2^2	0	0

(2x**1**, 2x**2**, 4x**2**^{2}) **b**

	1	2	3	4	5
1	0	2^2	0	1	0
2	2^2	0	0	0	0
3	0	0	2	0	2^2
4	1	0	0	2	0
5	0	0	2^2	0	0

(2x**1**, 2x**2**, 4x**2**^{2}) **c**

	1	2	3	4	5
1	0	1	0	2^2	0
2	1	0	2^2	0	0
3	0	2^2	0	0	0
4	2^2	0	0	2	0
5	0	0	0	0	2

(2x**1**, 2x**2**, 4x**2**^{2}) **d**

	1	2	3	4	5
1	0	2^2	0	1	0
2	2^2	0	2^2	0	0
3	0	2^2	0	0	0
4	1	0	0	2	0
5	0	0	0	0	2

(2x**1**, 2x**2**, 4x**2**^{2}) **e**

	1	2	3	4	5
1	0	2^2	0	0	0
2	2^2	0	2^2	0	0
3	0	2^2	0	0	0
4	0	0	0	2	1
5	0	0	0	1	2

(2x**1**, 2x**2**, 4x**2**^{2}) **f**

	1	2	3	4	5
1	0	2^2	1	0	0
2	2^2	0	2^2	0	0
3	1	2^2	0	0	0
4	0	0	0	2	0
5	0	0	0	0	2

(2x**1**, 6x**2**^{1}) **a**

	1	2	3	4	5
1	0	2^1	1	0	0
2	2^1	0	2^1	0	0
3	1	2^1	0	0	0
4	0	0	0	0	2^1
5	0	0	0	2^1	0

(2x**1**, 6x**2**^{1}) **b**

	1	2	3	4	5
1	0	1	0	2^1	0
2	1	0	2^1	0	0
3	0	2^1	0	0	2^1
4	2^1	0	0	0	0
5	0	0	2^1	0	0

(2x**1**, 4x**2**^{1}, 2x**2**^{2}) **a**

	1	2	3	4	5
1	0	2^1	1	0	0
2	2^1	0	2^2	0	0
3	1	2^2	0	0	0
4	0	0	0	0	2^1
5	0	0	0	2^1	0

(2x**1**, 4x**2**^{1}, 2x**2**^{2}) **b**

	1	2	3	4	5
1	0	2^1	1	0	0
2	2^1	0	2^1	0	0
3	1	2^1	0	0	0
4	0	0	0	0	2^2
5	0	0	0	2^2	0

(2x**1**, 4x**2**^{1}, 2x**2**^{2}) **c**

	1	2	3	4	5
1	0	1	0	2^2	0
2	1	0	2^1	0	0
3	0	2^1	0	0	2^1
4	2^2	0	0	0	0
5	0	0	2^1	0	0

(2x**1**, 4x**2**^{1}, 2x**2**^{2}) **d**

	1	2	3	4	5
1	0	1	0	2^1	0
2	1	0	2^2	0	0
3	0	2^2	0	0	2^1
4	2^1	0	0	0	0
5	0	0	2^1	0	0

(2x**1**, 4x**2**^{1}, 2x**2**^{2}) **e**

	1	2	3	4	5
1	0	1	0	2^1	0
2	1	0	2^1	0	0
3	0	2^1	0	0	2^2
4	2^1	0	0	0	0
5	0	0	2^2	0	0

(2x**1**, 2x**2**^{1}, 4x**2**^{2}) **a**

	1	2	3	4	5
1	0	2^2	1	0	0
2	2^2	0	2^2	0	0
3	1	2^2	0	0	0
4	0	0	0	0	2^1
5	0	0	0	2^1	0

(2x**1**, 2x**2**^{1}, 4x**2**^{2}) **b**

	1	2	3	4	5
1	0	2^1	1	0	0
2	2^1	0	2^2	0	0
3	1	2^2	0	0	0
4	0	0	0	0	2^2
5	0	0	0	2^2	0

(2x**1**, 2x**2**^{1}, 4x**2**^{2}) **c**

	1	2	3	4	5
1	0	1	0	2^2	0
2	1	0	2^2	0	0
3	0	2^2	0	0	2^1
4	2^2	0	0	0	0
5	0	0	2^1	0	0

(2x**1**, 2x**2**^{1}, 4x**2**^{2}) **d**

	1	2	3	4	5
1	0	1	0	2^2	0
2	1	0	2^1	0	0
3	0	2^1	0	0	2^2
4	2^2	0	0	0	0
5	0	0	2^2	0	0

(2x**1**, 2x**2**^{1}, 4x**2**^{2}) **e**

	1	2	3	4	5
1	0	1	0	2^1	0
2	1	0	2^2	0	0
3	0	2^2	0	0	2^2
4	2^1	0	0	0	0
5	0	0	2^2	0	0

(2x**1**, 6x**2**^{2}) **a**

	1	2	3	4	5
1	0	2^2	1	0	0
2	2^2	0	2^2	0	0
3	1	2^2	0	0	0
4	0	0	0	0	2^2
5	0	0	0	2^2	0

(2x**1**, 6x**2**^{2}) **b**

	1	2	3	4	5
1	0	1	0	2^2	0
2	1	0	2^2	0	0
3	0	2^2	0	0	2^2
4	2^2	0	0	0	0
5	0	0	2^2	0	0

$^2V_2{}^3V_2{}^4V_2$	NG
$^2V_2{}^3V_2{}^4V_3$	NG
$^2V_2{}^3V_2{}^4V_4$	NG
$^2V_2{}^3V_4{}^4V_1$	NG
$^2V_2{}^3V_4{}^4V_2$	NG
$^2V_3{}^3V_2{}^4V_1$	NG
$^2V_3{}^3V_2{}^4V_2$	NG
$^2V_3{}^3V_2{}^4V_3$	NG
$^2V_3{}^3V_4{}^4V_1$	NG
$^2V_4{}^3V_2{}^4V_1$	NG
$^2V_4{}^3V_2{}^4V_2$	NG
$^2V_5{}^3V_2{}^4V_1$	NG

Rank 4

$^1V_r{}^2V_r{}^3V_r{}^4V_r$

$^1V_1{}^2V_1{}^3V_1{}^4V_1$

(8x**1**, 1x**2**)

	1	2	3	4
1	0	1	0	1
2	1	0	1	1
3	0	1	0	0
4	1	1	0	2

(6x**1**, 2x**2**) **a**

	1	2	3	4
1	0	1	0	0
2	1	0	1	0
3	0	1	2	1
4	0	0	1	2

(6x**1**, 2x**2**) **b**

	1	2	3	4
1	0	1	0	0
2	1	2	1	0
3	0	1	0	1
4	0	0	1	2

(6x**1**, 2x**2**^{1})

	1	2	3	4
1	0	1	0	2^1
2	1	0	0	1
3	0	0	0	1
4	2^1	1	1	0

(6x**1**, 2x**2**^{2})

	1	2	3	4
1	0	1	0	2^2
2	1	0	0	1
3	0	0	0	1
4	2^2	1	1	0

(4x**1**, 3x**2**)

	1	2	3	4
1	2	0	0	0
2	0	0	1	0
3	0	1	2	1
4	0	0	1	2

(4x**1**, 1x**2**, 2x**2**^{1}) **a**

	1	2	3	4
1	0	1	0	0
2	1	0	1	0
3	0	1	0	2^1
4	0	0	2^1	2

(4x**1**, 1x**2**, 2x**2**^{1}) **b**

	1	2	3	4
1	0	1	0	0
2	1	2	1	0
3	0	1	0	2^1
4	0	0	2^1	0

(4x**1**, 1x**2**, 2x**2**^{1}) **c**

	1	2	3	4
1	0	0	1	0
2	0	2	1	0
3	1	1	0	2^1
4	0	0	2^1	0

(4x**1**, 1x**2**, 2x**2**^{2}) **a**

	1	2	3	4
1	0	1	0	0
2	1	0	1	0
3	0	1	0	2^2
4	0	0	2^2	2

(4x**1**, 1x**2**, 2x**2**^{2}) **b**

	1	2	3	4
1	0	1	0	0
2	1	2	1	0
3	0	1	0	2^2
4	0	0	2^2	0

(4x**1**, 1x**2**, 2x**2**^{2}) **c**

	1	2	3	4
1	0	0	1	0
2	0	2	1	0
3	1	1	0	2^2
4	0	0	2^2	0

(2x**1**, 2x**2**, 2x**2**^{1}) **a**

	1	2	3	4
1	2	0	0	0
2	0	0	1	0
3	0	1	0	2^1
4	0	0	2^1	2

(2x**1**, 2x**2**, 2x**2**^{1}) **b**

	1	2	3	4
1	2	1	0	0
2	1	0	0	0
3	0	0	0	2^1
4	0	0	2^1	2

(2x**1**, 2x**2**, 2x**2**^{2}) **a**

	1	2	3	4
1	2	0	0	0
2	0	0	1	0
3	0	1	0	2^2
4	0	0	2^2	2

(2x**1**, 2x**2**, 2x**2**^{2}) **b**

	1	2	3	4
1	2	1	0	0
2	1	0	0	0
3	0	0	0	2^2
4	0	0	2^2	2

(2x**1**, 4x**2**^{1})

	1	2	3	4
1	0	1	0	0
2	1	0	2^1	0
3	0	2^1	0	2^1
4	0	0	2^1	0

(2x**1**, 2x**2**^{1}, 2x**2**^{2}) **a**

	1	2	3	4
1	0	1	0	0
2	1	0	2^1	0
3	0	2^1	0	2^2
4	0	0	2^2	0

(2x**1**, 2x**2**^{1}, 2x**2**^{2}) **b**

	1	2	3	4
1	0	1	0	0
2	1	0	2^2	0
3	0	2^2	0	2^1
4	0	0	2^1	0

(2x**1**, 4x**2**^{2})

	1	2	3	4
1	0	1	0	0
2	1	0	2^2	0
3	0	2^2	0	2^2
4	0	0	2^2	0

$^1V_1{}^2V_1{}^3V_1{}^4V_2$

(12x**1**, 1x**2**)

	1	2	3	4	5
1	0	1	0	1	0
2	1	0	1	1	1
3	0	1	2	1	0
4	1	1	1	0	0
5	0	1	0	0	0

(10x**1**, 2x**2**) **a**

	1	2	3	4	5
1	2	1	0	1	0
2	1	0	1	0	1
3	0	1	2	1	0
4	1	0	1	0	0
5	0	1	0	0	0

(10x**1**, 2x**2**) **b**

	1	2	3	4	5
1	2	1	0	1	0
2	1	0	1	0	1
3	0	1	0	1	0
4	1	0	1	2	0
5	0	1	0	0	0

(10x**1**, 2x**2**) **c**

	1	2	3	4	5
1	2	1	0	1	0
2	1	0	0	1	1
3	0	0	0	0	1
4	1	1	0	2	0
5	0	1	1	0	0

(10x**1**, 2x**2**) **d**

	1	2	3	4	5
1	0	1	0	1	0
2	1	0	0	1	1
3	0	0	0	0	1
4	1	1	0	2	0
5	0	1	1	0	2

(10x**1**, 2x**2**) **e**

	1	2	3	4	5
1	0	1	0	1	0
2	1	0	1	1	1
3	0	1	0	0	0
4	1	1	0	2	0
5	0	1	0	0	2

(10x**1**, 2x**2**^{1}) **a**

	1	2	3	4	5
1	0	1	0	1	0
2	1	0	1	1	1
3	0	1	0	2^1	0
4	1	1	2^1	0	0
5	0	1	0	0	0

(10x**1**, 2x**2**^{1}) **b**

	1	2	3	4	5
1	0	1	1	1	0
2	1	0	2^1	0	1
3	1	2^1	0	1	0
4	1	0	1	0	0
5	0	1	0	0	0

(10x**1**, 2x**2**^{1}) **c**

	1	2	3	4	5
1	0	1	2^1	1	0
2	1	0	1	0	1
3	2^1	1	0	1	0
4	1	0	1	0	0
5	0	1	0	0	0

(10x**1**, 2x**2**^{2}) **a**

	1	2	3	4	5
1	0	1	0	1	0
2	1	0	1	1	1
3	0	1	0	2^2	0
4	1	1	2^2	0	0
5	0	1	0	0	0

(10x**1**, 2x**2**^{2}) **b**

	1	2	3	4	5
1	0	1	1	1	0
2	1	0	2^2	0	1
3	1	2^2	0	1	0
4	1	0	1	0	0
5	0	1	0	0	0

(10x**1**, 2x**2**^{2}) **c**

	1	2	3	4	5
1	0	1	2^2	1	0
2	1	0	1	0	1
3	2^2	1	0	1	0
4	1	0	1	0	0
5	0	1	0	0	0

(8x**1**, 3x**2**) **a**

	1	2	3	4	5
1	0	1	0	1	0
2	1	2	0	0	1
3	0	0	2	1	0
4	1	0	1	2	0
5	0	1	0	0	0

(8x**1**, 3x**2**) **b**

	1	2	3	4	5
1	2	1	0	1	0
2	1	0	0	0	1
3	0	0	2	1	0
4	1	0	1	2	0
5	0	1	0	0	0

(8x**1**, 3x**2**) **c**

	1	2	3	4	5
1	2	1	0	1	0
2	1	0	0	0	1
3	0	0	0	1	0
4	1	0	1	2	0
5	0	1	0	0	2

(8x**1**, 3x**2**) **d**

	1	2	3	4	5
1	2	1	0	1	0
2	1	0	0	1	1
3	0	0	2	0	0
4	1	1	0	2	0
5	0	1	0	0	0

(8x**1**, 3x**2**) **e**

	1	2	3	4	5
1	2	1	0	1	0
2	1	0	0	1	0
3	0	0	2	0	1
4	1	1	0	2	0
5	0	0	1	0	0

(8x**1**, 1x**2**, 2x**2**^{1}) **a**

	1	2	3	4	5
1	0	1	0	1	0
2	1	0	2^1	0	1
3	0	2^1	0	1	0
4	1	0	1	2	0
5	0	1	0	0	0

(8x**1**, 1x**2**, 2x**2**^{1}) **b**

	1	2	3	4	5
1	2	1	0	1	0
2	1	0	2^1	0	1
3	0	2^1	0	1	0
4	1	0	1	0	0
5	0	1	0	0	0

(8x**1**, 1x**2**, 2x**2**^{1}) **c**

	1	2	3	4	5
1	0	2^1	0	1	0
2	2^1	0	0	1	1
3	0	0	0	0	1
4	1	1	0	2	0
5	0	1	1	0	0

(8x**1**, 1x**2**, 2x**2**^{1}) **d**

	1	2	3	4	5
1	0	1	0	1	0
2	1	0	0	1	2^1
3	0	0	0	0	1
4	1	1	0	2	0
5	0	2^1	1	0	0

(8x**1**, 1x**2**, 2x**2**^{1}) **e**

	1	2	3	4	5
1	0	2^1	0	1	0
2	2^1	0	0	1	1
3	0	0	0	0	1
4	1	1	0	0	0
5	0	1	1	0	2

(8x**1**, 1x**2**, 2x**2**^{1}) **f**

	1	2	3	4	5
1	0	2^1	0	1	1
2	2^1	0	1	1	0
3	0	1	2	0	0
4	1	1	0	0	0
5	1	0	0	0	0

(8x**1**, 1x**2**, 2x**2**^{1}) **g**

	1	2	3	4	5
1	0	1	0	1	1
2	1	0	2^1	1	0
3	0	2^1	2	0	0
4	1	1	0	0	0
5	1	0	0	0	0

(8x**1**, 1x**2**, 2x**2**^{1}) **h**

	1	2	3	4	5
1	0	1	0	1	1
2	1	0	2^1	1	0
3	0	2^1	0	0	0
4	1	1	0	2	0
5	1	0	0	0	0

(8x**1**, 1x**2**, 2x$\mathbf{2}^2$) **a**

	1	2	3	4	5
1	0	1	0	1	0
2	1	0	2^2	0	1
3	0	2^2	0	1	0
4	1	0	1	2	0
5	0	1	0	0	0

(8x**1**, 1x**2**, 2x$\mathbf{2}^2$) **b**

	1	2	3	4	5
1	2	1	0	1	0
2	1	0	2^2	0	1
3	0	2^2	0	1	0
4	1	0	1	0	0
5	0	1	0	0	0

(8x**1**, 1x**2**, 2x$\mathbf{2}^2$) **c**

	1	2	3	4	5
1	0	2^2	0	1	0
2	2^2	0	0	1	1
3	0	0	0	0	1
4	1	1	0	2	0
5	0	1	1	0	0

(8x**1**, 1x**2**, 2x$\mathbf{2}^2$) **d**

	1	2	3	4	5
1	0	1	0	1	0
2	1	0	0	1	2^2
3	0	0	0	0	1
4	1	1	0	2	0
5	0	2^2	1	0	0

(8x**1**, 1x**2**, 2x$\mathbf{2}^2$) **e**

	1	2	3	4	5
1	0	2^2	0	1	0
2	2^2	0	0	1	1
3	0	0	0	0	1
4	1	1	0	0	0
5	0	1	1	0	2

(8x**1**, 1x**2**, 2x$\mathbf{2}^2$) **f**

	1	2	3	4	5
1	0	2^2	0	1	1
2	2^2	0	1	1	0
3	0	1	2	0	0
4	1	1	0	0	0
5	1	0	0	0	0

(8x**1**, 1x**2**, 2x$\mathbf{2}^2$) **g**

	1	2	3	4	5
1	0	1	0	1	1
2	1	0	2^2	1	0
3	0	2^2	2	0	0
4	1	1	0	0	0
5	1	0	0	0	0

(8x**1**, 1x**2**, 2x$\mathbf{2}^2$) **h**

	1	2	3	4	5
1	0	1	0	1	1
2	1	0	2^2	1	0
3	0	2^2	0	0	0
4	1	1	0	2	0
5	1	0	0	0	0

(6x**1**, 4x**2**)

	1	2	3	4	5
1	2	1	0	1	0
2	1	0	0	0	0
3	0	0	2	1	0
4	1	0	1	2	0
5	0	0	0	0	2

(6x**1**, 2x**2**, 2x$\mathbf{2}^1$) **a**

	1	2	3	4	5
1	2	1	0	1	0
2	1	0	0	0	1
3	0	0	2	2^1	0
4	1	0	2^1	0	0
5	0	1	0	0	0

(6x**1**, 2x**2**, 2x$\mathbf{2}^1$) **b**

	1	2	3	4	5
1	0	1	0	1	0
2	1	2	0	0	1
3	0	0	2	2^1	0
4	1	0	2^1	0	0
5	0	1	0	0	0

(6x**1**, 2x**2**, 2x$\mathbf{2}^1$) **c**

	1	2	3	4	5
1	2	1	0	1	0
2	1	2	0	0	1
3	0	0	0	2^1	0
4	1	0	2^1	0	0
5	0	1	0	0	0

(6x**1**, 2x**2**, 2x$\mathbf{2}^1$) **d**

	1	2	3	4	5
1	0	1	0	0	1
2	1	0	0	0	0
3	0	0	2	0	2^1
4	0	0	0	2	1
5	1	0	2^1	1	0

(6x**1**, 2x**2**, 2x$\mathbf{2}^1$) **e**

	1	2	3	4	5
1	0	1	0	0	1
2	1	2	0	0	0
3	0	0	2	0	2^1
4	0	0	0	0	1
5	1	0	2^1	1	0

(6x**1**, 2x**2**, 2x$\mathbf{2}^1$) **f**

	1	2	3	4	5
1	2	1	0	0	1
2	1	0	0	0	0
3	0	0	0	0	2^1
4	0	0	0	2	1
5	1	0	2^1	1	0

(6x**1**, 2x**2**, 2x$\mathbf{2}^1$) **g**

	1	2	3	4	5
1	2	1	0	0	1
2	1	2	0	0	0
3	0	0	0	0	2^1
4	0	0	0	0	1
5	1	0	2^1	1	0

(6x**1**, 2x**2**, 2x$\mathbf{2}^1$) **h**

	1	2	3	4	5
1	2	0	1	1	0
2	0	0	1	0	0
3	1	1	0	2^1	0
4	1	0	2^1	0	0
5	0	0	0	0	2

(6x**1**, 2x**2**, 2x$\mathbf{2}^2$) **a**

	1	2	3	4	5
1	2	1	0	1	0
2	1	0	0	0	1
3	0	0	2	2^2	0
4	1	0	2^2	0	0
5	0	1	0	0	0

(6x**1**, 2x**2**, 2x$\mathbf{2}^2$) **b**

	1	2	3	4	5
1	0	1	0	1	0
2	1	2	0	0	1
3	0	0	2	2^2	0
4	1	0	2^2	0	0
5	0	1	0	0	0

(6x**1**, 2x**2**, 2x$\mathbf{2}^2$) **c**

	1	2	3	4	5
1	2	1	0	1	0
2	1	2	0	0	1
3	0	0	0	2^2	0
4	1	0	2^2	0	0
5	0	1	0	0	0

(6x**1**, 2x**2**, 2x$\mathbf{2}^2$) **d**

	1	2	3	4	5
1	0	1	0	0	1
2	1	0	0	0	0
3	0	0	2	0	2^2
4	0	0	0	2	1
5	1	0	2^2	1	0

(6x**1**, 2x**2**, 2x$\mathbf{2}^2$) **e**

	1	2	3	4	5
1	0	1	0	0	1
2	1	2	0	0	0
3	0	0	2	0	2^2
4	0	0	0	0	1
5	1	0	2^2	1	0

(6x**1**, 2x**2**, 2x$\mathbf{2}^2$) **f**

	1	2	3	4	5
1	2	1	0	0	1
2	1	0	0	0	0
3	0	0	0	0	2^2
4	0	0	0	2	1
5	1	0	2^2	1	0

(6x**1**, 2x**2**, 2x$\mathbf{2}^2$) **g**

	1	2	3	4	5
1	2	1	0	0	1
2	1	2	0	0	0
3	0	0	0	0	2^2
4	0	0	0	0	1
5	1	0	2^2	1	0

(6x**1**, 2x**2**, 2x$\mathbf{2}^2$) **h**

	1	2	3	4	5
1	2	0	1	1	0
2	0	0	1	0	0
3	1	1	0	2^2	0
4	1	0	2^2	0	0
5	0	0	0	0	2

(6x**1**, 4x$\mathbf{2}^1$) **a**

	1	2	3	4	5
1	0	1	0	1	0
2	1	0	2^1	0	1
3	0	2^1	0	2^1	0
4	1	0	2^1	0	0
5	0	1	0	0	0

(6x**1**, 4x$\mathbf{2}^1$) **b**

	1	2	3	4	5
1	0	2^1	0	2^1	0
2	2^1	0	0	1	1
3	0	0	0	0	1
4	2^1	1	0	0	0
5	0	1	1	0	0

(6x**1**, 4x$\mathbf{2}^1$) **c**

	1	2	3	4	5
1	0	1	0	2^1	1
2	1	0	2^1	1	0
3	0	2^1	0	0	0
4	2^1	1	0	0	0
5	1	0	0	0	0

(6x**1**, 2x$\mathbf{2}^1$, 2x$\mathbf{2}^2$) **a**

	1	2	3	4	5
1	0	1	0	1	0
2	1	0	2^1	0	1
3	0	2^1	0	2^2	0
4	1	0	2^2	0	0
5	0	1	0	0	0

(6x**1**, 2x$\mathbf{2}^1$, 2x$\mathbf{2}^2$) **b**

	1	2	3	4	5
1	0	1	0	1	0
2	1	0	2^2	0	1
3	0	2^2	0	2^1	0
4	1	0	2^1	0	0
5	0	1	0	0	0

(6x**1**, 2x$\mathbf{2}^1$, 2x$\mathbf{2}^2$) **c**

	1	2	3	4	5
1	0	2^1	0	2^2	0
2	2^1	0	0	1	1
3	0	0	0	0	1
4	2^2	1	0	0	0
5	0	1	1	0	0

(6x**1**, 2x$\mathbf{2}^1$, 2x$\mathbf{2}^2$) **d**

	1	2	3	4	5
1	0	2^2	0	2^1	0
2	2^2	0	0	1	1
3	0	0	0	0	1
4	2^1	1	0	0	0
5	0	1	1	0	0

(6x**1**, 2x$\mathbf{2}^1$, 2x$\mathbf{2}^2$) **e**

	1	2	3	4	5
1	0	1	0	2^2	1
2	1	0	2^1	1	0
3	0	2^1	0	0	0
4	2^2	1	0	0	0
5	1	0	0	0	0

(6x**1**, 2x$\mathbf{2}^1$, 2x$\mathbf{2}^2$) **f**

	1	2	3	4	5
1	0	1	0	2^1	1
2	1	0	2^2	1	0
3	0	2^2	0	0	0
4	2^1	1	0	0	0
5	1	0	0	0	0

(6x**1**, 4x$\mathbf{2}^2$) **a**

	1	2	3	4	5
1	0	1	0	1	0
2	1	0	2^2	0	1
3	0	2^2	0	2^2	0
4	1	0	2^2	0	0
5	0	1	0	0	0

(6x**1**, 4x**2**^{2}) **b**

	1	2	3	4	5
1	0	2^2	0	2^2	0
2	2^2	0	0	1	1
3	0	0	0	0	1
4	2^2	1	0	0	0
5	0	1	1	0	0

(6x**1**, 4x**2**^{2}) **c**

	1	2	3	4	5
1	0	1	0	2^2	1
2	1	0	2^2	1	0
3	0	2^2	0	0	0
4	2^2	1	0	0	0
5	1	0	0	0	0

(4x**1**, 3x**2**, 2x**2**^{1}) **a**

	1	2	3	4	5
1	2	1	0	0	0
2	1	2	0	0	1
3	0	0	2	2^1	0
4	0	0	2^1	0	0
5	0	1	0	0	0

(4x**1**, 3x**2**, 2x**2**^{1}) **b**

	1	2	3	4	5
1	2	1	0	0	0
2	1	0	0	0	1
3	0	0	2	2^1	0
4	0	0	2^1	2	0
5	0	1	0	0	0

(4x**1**, 3x**2**, 2x**2**^{1}) **c**

	1	2	3	4	5
1	2	0	0	1	0
2	0	0	0	1	0
3	0	0	2	2^1	0
4	1	1	2^1	0	0
5	0	0	0	0	2

(4x**1**, 3x**2**, 2x**2**^{1}) **d**

	1	2	3	4	5
1	2	1	0	1	0
2	1	0	0	0	0
3	0	0	2	2^1	0
4	1	0	2^1	0	0
5	0	0	0	0	2

(4x**1**, 3x**2**, 2x**2**^{2}) **a**

	1	2	3	4	5
1	2	1	0	0	0
2	1	2	0	0	1
3	0	0	2	2^2	0
4	0	0	2^2	0	0
5	0	1	0	0	0

(4x**1**, 3x**2**, 2x**2**^{2}) **b**

	1	2	3	4	5
1	2	1	0	0	0
2	1	0	0	0	1
3	0	0	2	2^2	0
4	0	0	2^2	2	0
5	0	1	0	0	0

(4x**1**, 3x**2**, 2x**2**^{2}) **c**

	1	2	3	4	5
1	2	0	0	1	0
2	0	0	0	1	0
3	0	0	2	2^2	0
4	1	1	2^2	0	0
5	0	0	0	0	2

(4x**1**, 3x**2**, 2x**2**^{2}) **d**

	1	2	3	4	5
1	2	1	0	1	0
2	1	0	0	0	0
3	0	0	2	2^2	0
4	1	0	2^2	0	0
5	0	0	0	0	2

(4x**1**, 1x**2**, 4x**2**^{1}) **a**

	1	2	3	4	5
1	0	1	0	2^1	0
2	1	2	0	0	1
3	0	0	0	2^1	0
4	2^1	0	2^1	0	0
5	0	1	0	0	0

(4x**1**, 1x**2**, 4x**2**^{1}) **b**

	1	2	3	4	5
1	0	1	0	2^1	0
2	1	0	0	0	1
3	0	0	2	2^1	0
4	2^1	0	2^1	0	0
5	0	1	0	0	0

(4x**1**, 1x**2**, 4x**2**^{1}) **c**

	1	2	3	4	5
1	0	2^1	0	0	1
2	2^1	0	0	0	0
3	0	0	2	0	2^1
4	0	0	0	0	1
5	1	0	2^1	1	0

(4x**1**, 1x**2**, 4x**2**^{1}) **d**

	1	2	3	4	5
1	0	2^1	0	0	1
2	2^1	2	0	0	0
3	0	0	0	0	2^1
4	0	0	0	0	1
5	1	0	2^1	1	0

(4x**1**, 1x**2**, 4x**2**^{1}) **e**

	1	2	3	4	5
1	0	2^1	0	0	2^1
2	2^1	0	0	0	0
3	0	0	2	0	1
4	0	0	0	0	1
5	2^1	0	1	1	0

(4x**1**, 1x**2**, 4x**2**^{1}) **f**

	1	2	3	4	5
1	0	2^1	0	2^1	0
2	2^1	0	0	1	1
3	0	0	2	0	0
4	2^1	1	0	0	0
5	0	1	0	0	0

(4x**1**, 1x**2**, 2x**2**^{1}, 2x**2**^{2}) **a**

	1	2	3	4	5
1	0	1	0	2^1	0
2	1	2	0	0	1
3	0	0	0	2^2	0
4	2^1	0	2^2	0	0
5	0	1	0	0	0

(4x**1**, 1x**2**, 2x**2**^{1}, 2x**2**^{2}) **b**

	1	2	3	4	5
1	0	1	0	2^2	0
2	1	2	0	0	1
3	0	0	0	2^1	0
4	2^2	0	2^1	0	0
5	0	1	0	0	0

(4x**1**, 1x**2**, 2x**2**^{1}, 2x**2**^{2}) **c**

	1	2	3	4	5
1	0	1	0	2^1	0
2	1	0	0	0	1
3	0	0	2	2^2	0
4	2^1	0	2^2	0	0
5	0	1	0	0	0

(4x**1**, 1x**2**, 2x**2**^{1}, 2x**2**^{2}) **d**

	1	2	3	4	5
1	0	1	0	2^2	0
2	1	0	0	0	1
3	0	0	2	2^1	0
4	2^2	0	2^1	0	0
5	0	1	0	0	0

(4x**1**, 1x**2**, 2x**2**^{1}, 2x**2**^{2}) **e**

	1	2	3	4	5
1	0	2^1	0	0	1
2	2^1	0	0	0	0
3	0	0	2	0	2^2
4	0	0	0	0	1
5	1	0	2^2	1	0

(4x**1**, 1x**2**, 2x**2**^{1}, 2x**2**^{2}) **f**

	1	2	3	4	5
1	0	2^2	0	0	1
2	2^2	0	0	0	0
3	0	0	2	0	2^1
4	0	0	0	0	1
5	1	0	2^1	1	0

(4x**1**, 1x**2**, 2x**2**^{1}, 2x**2**^{2}) **g**

	1	2	3	4	5
1	0	2^1	0	0	1
2	2^1	2	0	0	0
3	0	0	0	0	2^2
4	0	0	0	0	1
5	1	0	2^2	1	0

(4x**1**, 1x**2**, 2x**2**^{1}, 2x**2**^{2}) **h**

	1	2	3	4	5
1	0	2^2	0	0	1
2	2^2	2	0	0	0
3	0	0	0	0	2^1
4	0	0	0	0	1
5	1	0	2^1	1	0

(4x**1**, 1x**2**, 2x**2**^{1}, 2x**2**^{2}) **i**

	1	2	3	4	5
1	0	2^2	0	0	2^1
2	2^2	0	0	0	0
3	0	0	2	0	1
4	0	0	0	0	1
5	2^1	0	1	1	0

(4x**1**, 1x**2**, 2x**2**^{1}, 2x**2**^{2}) **j**

	1	2	3	4	5
1	0	2^1	0	0	2^2
2	2^1	0	0	0	0
3	0	0	2	0	1
4	0	0	0	0	1
5	2^2	0	1	1	0

(4x**1**, 1x**2**, 2x**2**^{1}, 2x**2**^{2}) **k**

	1	2	3	4	5
1	0	2^1	0	2^2	0
2	2^1	0	0	1	1
3	0	0	2	0	0
4	2^2	1	0	0	0
5	0	1	0	0	0

(4x**1**, 1x**2**, 2x**2**^{1}, 2x**2**^{2}) **l**

	1	2	3	4	5
1	0	2^2	0	2^1	0
2	2^2	0	0	1	1
3	0	0	2	0	0
4	2^1	1	0	0	0
5	0	1	0	0	0

(4x**1**, 1x**2**, 4x**2**^{2}) **a**

	1	2	3	4	5
1	0	1	0	2^2	0
2	1	2	0	0	1
3	0	0	0	2^2	0
4	2^2	0	2^2	0	0
5	0	1	0	0	0

(4x**1**, 1x**2**, 4x**2**^{2}) **b**

	1	2	3	4	5
1	0	1	0	2^2	0
2	1	0	0	0	1
3	0	0	2	2^2	0
4	2^2	0	2^2	0	0
5	0	1	0	0	0

(4x**1**, 1x**2**, 4x**2**^{2}) **c**

	1	2	3	4	5
1	0	2^2	0	0	1
2	2^2	0	0	0	0
3	0	0	2	0	2^2
4	0	0	0	0	1
5	1	0	2^2	1	0

(4x**1**, 1x**2**, 4x**2**^{2}) **d**

	1	2	3	4	5
1	0	2^2	0	0	1
2	2^2	2	0	0	0
3	0	0	0	0	2^2
4	0	0	0	0	1
5	1	0	2^2	1	0

(4x**1**, 1x**2**, 4x**2**^{2}) **e**

	1	2	3	4	5
1	0	2^2	0	0	2^2
2	2^2	0	0	0	0
3	0	0	2	0	1
4	0	0	0	0	1
5	2^2	0	1	1	0

(4x**1**, 1x**2**, 4x**2**^{2}) **f**

	1	2	3	4	5
1	0	2^2	0	2^2	0
2	2^2	0	0	1	1
3	0	0	2	0	0
4	2^2	1	0	0	0
5	0	1	0	0	0

(2x**1**, 4x**2**, 2x**2**^{1})

	1	2	3	4	5
1	2	1	0	0	0
2	1	0	0	0	0
3	0	0	2	2^1	0
4	0	0	2^1	2	0
5	0	0	0	0	2

(2x**1**, 4x**2**, 2x**2**^{2})

	1	2	3	4	5
1	2	1	0	0	0
2	1	0	0	0	0
3	0	0	2	2^2	0
4	0	0	2^2	2	0
5	0	0	0	0	2

(2x**1**, 2x**2**, 4x**2**^{1}) **a**

	1	2	3	4	5
1	0	1	0	0	0
2	1	0	0	0	2^1
3	0	0	2	2^1	0
4	0	0	2^1	2	0
5	0	2^1	0	0	0

(2x**1**, 2x**2**, 4x**2**^{1}) **b**

	1	2	3	4	5
1	2	2^1	0	0	0
2	2^1	0	0	0	1
3	0	0	2	2^1	0
4	0	0	2^1	0	0
5	0	1	0	0	0

(2x**1**, 2x**2**, 4x**2**^{1}) **c**

	1	2	3	4	5
1	2	2^1	0	0	0
2	2^1	0	0	0	2^1
3	0	0	2	1	0
4	0	0	1	0	0
5	0	2^1	0	0	0

(2x**1**, 2x**2**, 4x**2**^{1}) **d**

	1	2	3	4	5
1	2	2^1	0	0	0
2	2^1	0	0	0	2^1
3	0	0	2	0	0
4	0	0	0	0	1
5	0	2^1	0	1	0

(2x**1**, 2x**2**, 2x**2**^{1}, 2x**2**^{2}) **a**

	1	2	3	4	5
1	0	1	0	0	0
2	1	0	0	0	2^1
3	0	0	2	2^2	0
4	0	0	2^2	2	0
5	0	2^1	0	0	0

(2x**1**, 2x**2**, 2x**2**^{1}, 2x**2**^{2}) **b**

	1	2	3	4	5
1	0	1	0	0	0
2	1	0	0	0	2^2
3	0	0	2	2^1	0
4	0	0	2^1	2	0
5	0	2^2	0	0	0

(2x**1**, 2x**2**, 2x**2**^{1}, 2x**2**^{2}) **c**

	1	2	3	4	5
1	2	2^1	0	0	0
2	2^1	0	0	0	1
3	0	0	2	2^2	0
4	0	0	2^2	0	0
5	0	1	0	0	0

(2x**1**, 2x**2**, 2x**2**^{1}, 2x**2**^{2}) **d**

	1	2	3	4	5
1	2	2^2	0	0	0
2	2^2	0	0	0	1
3	0	0	2	2^1	0
4	0	0	2^1	0	0
5	0	1	0	0	0

(2x**1**, 2x**2**, 2x**2**^{1}, 2x**2**^{2}) **e**

	1	2	3	4	5
1	2	2^2	0	0	0
2	2^2	0	0	0	2^1
3	0	0	2	1	0
4	0	0	1	0	0
5	0	2^1	0	0	0

(2x**1**, 2x**2**, 2x**2**^{1}, 2x**2**^{2}) **f**

	1	2	3	4	5
1	2	2^1	0	0	0
2	2^1	0	0	0	2^2
3	0	0	2	1	0
4	0	0	1	0	0
5	0	2^2	0	0	0

(2x**1**, 2x**2**, 2x**2**^{1}, 2x**2**^{2}) **g**

	1	2	3	4	5
1	2	2^1	0	0	0
2	2^1	0	0	0	2^2
3	0	0	2	0	0
4	0	0	0	0	1
5	0	2^2	0	1	0

(2x**1**, 2x**2**, 2x**2**^{1}, 2x**2**^{2}) **h**

	1	2	3	4	5
1	2	2^2	0	0	0
2	2^2	0	0	0	2^1
3	0	0	2	0	0
4	0	0	0	0	1
5	0	2^1	0	1	0

(2x**1**, 2x**2**, 4x**2**^{2}) **a**

	1	2	3	4	5
1	0	1	0	0	0
2	1	0	0	0	2^2
3	0	0	2	2^2	0
4	0	0	2^2	2	0
5	0	2^2	0	0	0

(2x**1**, 2x**2**, 4x**2**^{2}) **b**

	1	2	3	4	5
1	2	2^2	0	0	0
2	2^2	0	0	0	1
3	0	0	2	2^2	0
4	0	0	2^2	0	0
5	0	1	0	0	0

(2x**1**, 2x**2**, 4x**2**^{2}) **c**

	1	2	3	4	5
1	2	2^2	0	0	0
2	2^2	0	0	0	2^2
3	0	0	2	1	0
4	0	0	1	0	0
5	0	2^2	0	0	0

(2x**1**, 2x**2**, 4x**2**^{2}) **d**

	1	2	3	4	5
1	2	2^2	0	0	0
2	2^2	0	0	0	2^2
3	0	0	2	0	0
4	0	0	0	0	1
5	0	2^2	0	1	0

(2x**1**, 6x**2**^{1})

	1	2	3	4	5
1	0	2^1	0	2^1	0
2	2^1	0	0	0	1
3	0	0	0	2^1	0
4	2^1	0	2^1	0	0
5	0	1	0	0	0

(2x**1**, 4x**2**^{1}, 2x**2**^{2}) **a**

	1	2	3	4	5
1	0	2^1	0	2^1	0
2	2^1	0	0	0	1
3	0	0	0	2^2	0
4	2^1	0	2^2	0	0
5	0	1	0	0	0

(2x**1**, 4x**2**^{1}, 2x**2**^{2}) **b**

	1	2	3	4	5
1	0	2^1	0	2^2	0
2	2^1	0	0	0	1
3	0	0	0	2^1	0
4	2^2	0	2^1	0	0
5	0	1	0	0	0

(2x**1**, 4x**2**^{1}, 2x**2**^{2}) **c**

	1	2	3	4	5
1	0	2^2	0	2^1	0
2	2^2	0	0	0	1
3	0	0	0	2^1	0
4	2^1	0	2^1	0	0
5	0	1	0	0	0

(2x**1**, 2x**2**^{1}, 4x**2**^{2}) **a**

	1	2	3	4	5
1	0	2^1	0	2^2	0
2	2^1	0	0	0	1
3	0	0	0	2^2	0
4	2^2	0	2^2	0	0
5	0	1	0	0	0

(2x**1**, 2x**2**^{1}, 4x**2**^{2}) **b**

	1	2	3	4	5
1	0	2^2	0	2^1	0
2	2^2	0	0	0	1
3	0	0	0	2^2	0
4	2^1	0	2^2	0	0
5	0	1	0	0	0

(2x**1**, 2x**2**^{1}, 4x**2**^{2}) **c**

	1	2	3	4	5
1	0	2^2	0	2^2	0
2	2^2	0	0	0	1
3	0	0	0	2^1	0
4	2^2	0	2^1	0	0
5	0	1	0	0	0

(2x**1**, 6x**2**^{2})

	1	2	3	4	5
1	0	2^2	0	2^2	0
2	2^2	0	0	0	1
3	0	0	0	2^2	0
4	2^2	0	2^2	0	0
5	0	1	0	0	0

$^{1}V_1{}^{2}V_1{}^{3}V_1{}^{4}V_3$	NG
$^{1}V_1{}^{2}V_1{}^{3}V_1{}^{4}V_4$	NG
$^{1}V_1{}^{2}V_1{}^{3}V_1{}^{4}V_5$	NG
$^{1}V_1{}^{2}V_1{}^{3}V_3{}^{4}V_1$	NG
$^{1}V_1{}^{2}V_1{}^{3}V_3{}^{4}V_2$	NG
$^{1}V_1{}^{2}V_1{}^{3}V_3{}^{4}V_3$	NG
$^{1}V_1{}^{2}V_1{}^{3}V_5{}^{4}V_1$	NG

$^1V_1{}^2V_2{}^3V_1{}^4V_1$

(12x**1**)

	1	2	3	4	5
1	0	1	0	1	0
2	1	0	1	1	1
3	0	1	0	1	0
4	1	1	1	0	0
5	0	1	0	0	0

(10x**1**, 1x**2**) **a**

	1	2	3	4	5
1	0	1	0	1	0
2	1	0	1	0	1
3	0	1	2	1	0
4	1	0	1	0	0
5	0	1	0	0	0

(10x**1**, 1x**2**) **b**

	1	2	3	4	5
1	0	1	0	1	0
2	1	0	1	0	1
3	0	1	0	1	0
4	1	0	1	2	0
5	0	1	0	0	0

(10x**1**, 1x**2**) **c**

	1	2	3	4	5
1	0	1	0	1	0
2	1	0	0	1	1
3	0	0	0	0	1
4	1	1	0	2	0
5	0	1	1	0	0

(10x**1**, 1x**2**) **d**

	1	2	3	4	5
1	0	1	0	1	0
2	1	0	0	1	1
3	0	0	0	0	1
4	1	1	0	0	0
5	0	1	1	0	2

(10x**1**, 1x**2**) **e**

	1	2	3	4	5
1	0	1	0	1	0
2	1	0	1	1	1
3	0	1	0	0	0
4	1	1	0	0	0
5	0	1	0	0	2

(8x**1**, 2x**2**) **a**

	1	2	3	4	5
1	0	1	0	1	0
2	1	0	0	1	1
3	0	0	2	0	0
4	1	1	0	2	0
5	0	1	0	0	0

(8x**1**, 2x**2**) **b**

	1	2	3	4	5
1	0	1	0	1	0
2	1	0	0	1	0
3	0	0	2	0	1
4	1	1	0	2	0
5	0	0	1	0	0

(8x**1**, 2x**2**) **c**

	1	2	3	4	5
1	0	1	0	1	0
2	1	0	0	0	1
3	0	0	0	0	1
4	1	0	0	2	0
5	0	1	1	0	2

(8x**1**, 2x**2**) **d**

	1	2	3	4	5
1	0	1	0	1	0
2	1	2	0	0	1
3	0	0	0	0	1
4	1	0	0	2	0
5	0	1	1	0	0

(8x**1**, 2x**2**) **e**

	1	2	3	4	5
1	2	1	0	1	0
2	1	0	0	0	1
3	0	0	0	0	1
4	1	0	0	2	0
5	0	1	1	0	0

(8x**1**, 2x$\mathbf{2}^1$) **a**

	1	2	3	4	5
1	0	1	0	1	0
2	1	0	2^1	0	1
3	0	2^1	0	1	0
4	1	0	1	0	0
5	0	1	0	0	0

(8x**1**, 2x$\mathbf{2}^1$) **b**

	1	2	3	4	5
1	0	1	0	1	0
2	1	0	0	1	1
3	0	0	0	2^1	0
4	1	1	2^1	0	0
5	0	1	0	0	0

(8x**1**, 2x$\mathbf{2}^1$) **c**

	1	2	3	4	5
1	0	1	0	1	0
2	1	0	0	2^1	1
3	0	0	0	0	1
4	1	2^1	0	0	0
5	0	1	1	0	0

(8x**1**, 2x$\mathbf{2}^1$) **d**

	1	2	3	4	5
1	0	1	0	1	0
2	1	0	0	1	2^1
3	0	0	0	0	1
4	1	1	0	0	0
5	0	2^1	1	0	0

(8x**1**, 2x$\mathbf{2}^2$) **a**

	1	2	3	4	5
1	0	1	0	1	0
2	1	0	2^2	0	1
3	0	2^2	0	1	0
4	1	0	1	0	0
5	0	1	0	0	0

(8x**1**, 2x$\mathbf{2}^2$) **b**

	1	2	3	4	5
1	0	1	0	1	0
2	1	0	0	1	1
3	0	0	0	2^2	0
4	1	1	2^2	0	0
5	0	1	0	0	0

(8x**1**, 2x$\mathbf{2}^2$) **c**

	1	2	3	4	5
1	0	1	0	1	0
2	1	0	0	2^2	1
3	0	0	0	0	1
4	1	2^2	0	0	0
5	0	1	1	0	0

(8x**1**, 2x$\mathbf{2}^2$) **d**

	1	2	3	4	5
1	0	1	0	1	0
2	1	0	0	1	2^2
3	0	0	0	0	1
4	1	1	0	0	0
5	0	2^2	1	0	0

(6x**1**, 3x**2**) **a**

	1	2	3	4	5
1	0	1	0	1	0
2	1	2	0	0	1
3	0	0	2	0	0
4	1	0	0	2	0
5	0	1	0	0	0

(6x**1**, 3x**2**) **b**

	1	2	3	4	5
1	2	1	0	1	0
2	1	0	0	0	1
3	0	0	2	0	0
4	1	0	0	2	0
5	0	1	0	0	0

(6x**1**, 1x**2**, 2x$\mathbf{2}^1$) **a**

	1	2	3	4	5
1	0	1	0	2^1	0
2	1	0	0	0	1
3	0	0	0	0	1
4	2^1	0	0	0	0
5	0	1	1	0	2

(6x**1**, 1x**2**, 2x$\mathbf{2}^1$) **b**

	1	2	3	4	5
1	0	1	0	2^1	0
2	1	2	0	0	1
3	0	0	0	0	1
4	2^1	0	0	0	0
5	0	1	1	0	0

(6x**1**, 1x**2**, 2x$\mathbf{2}^1$) **c**

	1	2	3	4	5
1	0	1	0	2^1	0
2	1	0	0	0	1
3	0	0	0	0	1
4	2^1	0	0	2	0
5	0	1	1	0	0

(6x**1**, 1x**2**, 2x$\mathbf{2}^1$) **d**

	1	2	3	4	5
1	0	1	0	1	0
2	1	0	0	2^1	1
3	0	0	2	0	0
4	1	2^1	0	0	0
5	0	1	0	0	0

(6x**1**, 1x**2**, 2x$\mathbf{2}^1$) **e**

	1	2	3	4	5
1	0	1	1	2^1	0
2	1	0	0	0	1
3	1	0	2	0	0
4	2^1	0	0	0	0
5	0	1	0	0	0

(6x**1**, 1x**2**, 2x$\mathbf{2}^1$) **f**

	1	2	3	4	5
1	0	1	1	2^1	0
2	1	0	0	0	1
3	1	0	0	0	0
4	2^1	0	0	0	0
5	0	1	0	0	2

(6x**1**, 1x**2**, 2x$\mathbf{2}^2$) **a**

	1	2	3	4	5
1	0	1	0	2^2	0
2	1	0	0	0	1
3	0	0	0	0	1
4	2^2	0	0	0	0
5	0	1	1	0	2

(6x**1**, 1x**2**, 2x$\mathbf{2}^2$) **b**

	1	2	3	4	5
1	0	1	0	2^2	0
2	1	2	0	0	1
3	0	0	0	0	1
4	2^2	0	0	0	0
5	0	1	1	0	0

(6x**1**, 1x**2**, 2x$\mathbf{2}^2$) **c**

	1	2	3	4	5
1	0	1	0	2^2	0
2	1	0	0	0	1
3	0	0	0	0	1
4	2^2	0	0	2	0
5	0	1	1	0	0

(6x**1**, 1x**2**, 2x$\mathbf{2}^2$) **d**

	1	2	3	4	5
1	0	1	0	1	0
2	1	0	0	2^2	1
3	0	0	2	0	0
4	1	2^2	0	0	0
5	0	1	0	0	0

(6x**1**, 1x**2**, 2x$\mathbf{2}^2$) **e**

	1	2	3	4	5
1	0	1	1	2^2	0
2	1	0	0	0	1
3	1	0	2	0	0
4	2^2	0	0	0	0
5	0	1	0	0	0

(6x**1**, 1x**2**, 2x$\mathbf{2}^2$) **f**

	1	2	3	4	5
1	0	1	1	2^2	0
2	1	0	0	0	1
3	1	0	0	0	0
4	2^2	0	0	0	0
5	0	1	0	0	2

(4x**1**, 4x**2**)

	1	2	3	4	5
1	2	1	0	1	0
2	1	0	0	0	0
3	0	0	2	0	0
4	1	0	0	2	0
5	0	0	0	0	2

(4x**1**, 2x**2**, 2x$\mathbf{2}^1$) **a**

	1	2	3	4	5
1	2	1	0	1	0
2	1	0	0	0	0
3	0	0	0	0	2^1
4	1	0	0	2	0
5	0	0	2^1	0	0

(4x**1**, 2x**2**, 2x**2**^{1}) **b**

	1	2	3	4	5
1	0	1	0	1	0
2	1	0	0	0	0
3	0	0	0	0	2^1
4	1	0	0	2	0
5	0	0	2^1	0	2

(4x**1**, 2x**2**, 2x**2**^{1}) **c**

	1	2	3	4	5
1	0	1	0	1	0
2	1	0	2^1	0	0
3	0	2^1	2	0	0
4	1	0	0	0	0
5	0	0	0	0	2

(4x**1**, 2x**2**, 2x**2**^{1}) **d**

	1	2	3	4	5
1	2	1	0	1	0
2	1	0	2^1	0	0
3	0	2^1	0	0	0
4	1	0	0	0	0
5	0	0	0	0	2

(4x**1**, 2x**2**, 2x**2**^{1}) **e**

	1	2	3	4	5
1	0	1	0	0	0
2	1	0	2^1	1	0
3	0	2^1	0	0	0
4	0	1	0	2	0
5	0	0	0	0	2

(4x**1**, 2x**2**, 2x**2**^{2}) **a**

	1	2	3	4	5
1	2	1	0	1	0
2	1	0	0	0	0
3	0	0	0	0	2^2
4	1	0	0	2	0
5	0	0	2^2	0	0

(4x**1**, 2x**2**, 2x**2**^{2}) **b**

	1	2	3	4	5
1	0	1	0	1	0
2	1	0	0	0	0
3	0	0	0	0	2^2
4	1	0	0	2	0
5	0	0	2^2	0	2

(4x**1**, 2x**2**, 2x**2**^{2}) **c**

	1	2	3	4	5
1	0	1	0	1	0
2	1	0	2^2	0	0
3	0	2^2	2	0	0
4	1	0	0	0	0
5	0	0	0	0	2

(4x**1**, 2x**2**, 2x**2**^{2}) **d**

	1	2	3	4	5
1	2	1	0	1	0
2	1	0	2^2	0	0
3	0	2^2	0	0	0
4	1	0	0	0	0
5	0	0	0	0	2

(4x**1**, 2x**2**, 2x**2**^{2}) **e**

	1	2	3	4	5
1	0	1	0	0	0
2	1	0	2^2	1	0
3	0	2^2	0	0	0
4	0	1	0	2	0
5	0	0	0	0	2

(4x**1**, 4x**2**^{1}) **a**

	1	2	3	4	5
1	0	2^1	0	2^1	0
2	2^1	0	0	0	1
3	0	0	0	0	1
4	2^1	0	0	0	0
5	0	1	1	0	0

(4x**1**, 4x**2**^{1}) **b**

	1	2	3	4	5
1	0	1	1	2^1	0
2	1	0	0	0	2^1
3	1	0	0	0	0
4	2^1	0	0	0	0
5	0	2^1	0	0	0

(4x**1**, 2x**2^1**, 2x**2**^{2}) **a**

	1	2	3	4	5
1	0	2^1	0	2^2	0
2	2^1	0	0	0	1
3	0	0	0	0	1
4	2^2	0	0	0	0
5	0	1	1	0	0

(4x**1**, 2x**2^1**, 2x**2**^{2}) **b**

	1	2	3	4	5
1	0	2^2	0	2^1	0
2	2^2	0	0	0	1
3	0	0	0	0	1
4	2^1	0	0	0	0
5	0	1	1	0	0

(4x**1**, 2x**2^1**, 2x**2**^{2}) **c**

	1	2	3	4	5
1	0	1	1	2^2	0
2	1	0	0	0	2^1
3	1	0	0	0	0
4	2^2	0	0	0	0
5	0	2^1	0	0	0

(4x**1**, 2x**2^1**, 2x**2**^{2}) **d**

	1	2	3	4	5
1	0	1	1	2^1	0
2	1	0	0	0	2^2
3	1	0	0	0	0
4	2^1	0	0	0	0
5	0	2^2	0	0	0

(4x**1**, 4x**2**^{2}) **a**

	1	2	3	4	5
1	0	2^2	0	2^2	0
2	2^2	0	0	0	1
3	0	0	0	0	1
4	2^2	0	0	0	0
5	0	1	1	0	0

(4x**1**, 4x**2**^{2}) **b**

	1	2	3	4	5
1	0	1	1	2^2	0
2	1	0	0	0	2^2
3	1	0	0	0	0
4	2^2	0	0	0	0
5	0	2^2	0	0	0

(2x**1**, 3x**2**, 2x**2**^{1}) **a**

	1	2	3	4	5
1	0	1	0	0	0
2	1	0	2^1	0	0
3	0	2^1	2	0	0
4	0	0	0	2	0
5	0	0	0	0	2

(2x**1**, 3x**2**, 2x**2**^{1}) **b**

	1	2	3	4	5
1	0	0	0	1	0
2	0	0	2^1	0	0
3	0	2^1	2	0	0
4	1	0	0	2	0
5	0	0	0	0	2

(2x**1**, 3x**2**, 2x**2**^{2}) **a**

	1	2	3	4	5
1	0	1	0	0	0
2	1	0	2^2	0	0
3	0	2^2	2	0	0
4	0	0	0	2	0
5	0	0	0	0	2

(2x**1**, 3x**2**, 2x**2**^{2}) **b**

	1	2	3	4	5
1	0	0	0	1	0
2	0	0	2^2	0	0
3	0	2^2	2	0	0
4	1	0	0	2	0
5	0	0	0	0	2

(2x**1**, 1x**2**, 4x**2**^{1}) **a**

	1	2	3	4	5
1	0	1	0	0	0
2	1	0	2^1	0	0
3	0	2^1	2	0	0
4	0	0	0	0	2^1
5	0	0	0	2^1	0

(2x**1**, 1x**2**, 4x**2**^{1}) **b**

	1	2	3	4	5
1	0	1	0	0	0
2	1	0	2^1	0	0
3	0	2^1	0	0	0
4	0	0	0	0	2^1
5	0	0	0	2^1	2

(2x**1**, 1x**2**, 4x**2**^{1}) **c**

	1	2	3	4	5
1	0	2^1	0	0	0
2	2^1	0	2^1	0	0
3	0	2^1	0	0	0
4	0	0	0	0	1
5	0	0	0	1	2

(2x**1**, 1x**2**, 4x**2**^{1}) **d**

	1	2	3	4	5
1	0	2^1	0	1	0
2	2^1	0	2^1	0	0
3	0	2^1	0	0	0
4	1	0	0	0	0
5	0	0	0	0	2

(2x**1**, 1x**2**, 2x**2^1**, 2x**2**^{2}) **a**

	1	2	3	4	5
1	0	1	0	0	0
2	1	0	2^2	0	0
3	0	2^2	2	0	0
4	0	0	0	0	2^1
5	0	0	0	2^1	0

(2x**1**, 1x**2**, 2x**2^1**, 2x**2**^{2}) **b**

	1	2	3	4	5
1	0	1	0	0	0
2	1	0	2^1	0	0
3	0	2^1	2	0	0
4	0	0	0	0	2^2
5	0	0	0	2^2	0

(2x**1**, 1x**2**, 2x**2^1**, 2x**2**^{2}) **c**

	1	2	3	4	5
1	0	1	0	0	0
2	1	0	2^2	0	0
3	0	2^2	0	0	0
4	0	0	0	0	2^1
5	0	0	0	2^1	2

(2x**1**, 1x**2**, 2x**2^1**, 2x**2**^{2}) **d**

	1	2	3	4	5
1	0	1	0	0	0
2	1	0	2^1	0	0
3	0	2^1	0	0	0
4	0	0	0	0	2^2
5	0	0	0	2^2	2

(2x**1**, 1x**2**, 2x**2^1**, 2x**2**^{2}) **e**

	1	2	3	4	5
1	0	2^1	0	0	0
2	2^1	0	2^2	0	0
3	0	2^2	0	0	0
4	0	0	0	0	1
5	0	0	0	1	2

(2x**1**, 1x**2**, 2x**2^1**, 2x**2**^{2}) **f**

	1	2	3	4	5
1	0	2^2	0	1	0
2	2^2	0	2^1	0	0
3	0	2^1	0	0	0
4	1	0	0	0	0
5	0	0	0	0	2

(2x**1**, 1x**2**, 2x**2^1**, 2x**2**^{2}) **g**

	1	2	3	4	5
1	0	2^1	0	1	0
2	2^1	0	2^2	0	0
3	0	2^2	0	0	0
4	1	0	0	0	0
5	0	0	0	0	2

(2x**1**, 1x**2**, 4x**2**^{2}) **a**

	1	2	3	4	5
1	0	1	0	0	0
2	1	0	2^2	0	0
3	0	2^2	2	0	0
4	0	0	0	0	2^2
5	0	0	0	2^2	0

(2x**1**, 1x**2**, 4x**2**^{2}) **b**

	1	2	3	4	5
1	0	1	0	0	0
2	1	0	2^2	0	0
3	0	2^2	0	0	0
4	0	0	0	0	2^2
5	0	0	0	2^2	2

(2x**1**, 1x**2**, 4x**2**^{2}) **c**

	1	2	3	4	5
1	0	2^2	0	0	0
2	2^2	0	2^2	0	0
3	0	2^2	0	0	0
4	0	0	0	0	1
5	0	0	0	1	2

<table>
<tr><td></td><td>(2x1, 1x2, 4x$\mathbf{2}^2$) d
<table>
<tr><td></td><td>1</td><td>2</td><td>3</td><td>4</td><td>5</td></tr>
<tr><td>1</td><td>0</td><td>2^2</td><td>0</td><td>1</td><td>0</td></tr>
<tr><td>2</td><td>2^2</td><td>0</td><td>2^2</td><td>0</td><td>0</td></tr>
<tr><td>3</td><td>0</td><td>2^2</td><td>0</td><td>0</td><td>0</td></tr>
<tr><td>4</td><td>1</td><td>0</td><td>0</td><td>0</td><td>0</td></tr>
<tr><td>5</td><td>0</td><td>0</td><td>0</td><td>0</td><td>2</td></tr>
</table></td><td></td><td></td><td></td><td></td></tr>
<tr><td>${}^{1}V_1{}^{2}V_2{}^{3}V_1{}^{4}V_2$</td><td colspan="5">NG</td></tr>
<tr><td>${}^{1}V_1{}^{2}V_2{}^{3}V_1{}^{4}V_3$</td><td colspan="5">NG</td></tr>
<tr><td>${}^{1}V_1{}^{2}V_2{}^{3}V_1{}^{4}V_4$</td><td colspan="5">NG</td></tr>
<tr><td>${}^{1}V_1{}^{2}V_2{}^{3}V_3{}^{4}V_1$</td><td colspan="5">NG</td></tr>
<tr><td>${}^{1}V_1{}^{2}V_2{}^{3}V_3{}^{4}V_2$</td><td colspan="5">NG</td></tr>
<tr><td>${}^{1}V_1{}^{2}V_3{}^{3}V_1{}^{4}V_1$</td><td colspan="5">NG</td></tr>
<tr><td>${}^{1}V_1{}^{2}V_3{}^{3}V_1{}^{4}V_2$</td><td colspan="5">NG</td></tr>
<tr><td>${}^{1}V_1{}^{2}V_3{}^{3}V_1{}^{4}V_3$</td><td colspan="5">NG</td></tr>
<tr><td>${}^{1}V_1{}^{2}V_3{}^{3}V_3{}^{4}V_1$</td><td colspan="5">NG</td></tr>
<tr><td>${}^{1}V_1{}^{2}V_4{}^{3}V_1{}^{4}V_1$</td><td colspan="5">NG</td></tr>
<tr><td>${}^{1}V_1{}^{2}V_4{}^{3}V_1{}^{4}V_2$</td><td colspan="5">NG</td></tr>
<tr><td>${}^{1}V_1{}^{2}V_5{}^{3}V_1{}^{4}V_1$</td><td colspan="5">NG</td></tr>
<tr><td>${}^{1}V_2{}^{2}V_1{}^{3}V_2{}^{4}V_1$</td><td colspan="5">NG</td></tr>
<tr><td>${}^{1}V_2{}^{2}V_1{}^{3}V_2{}^{4}V_2$</td><td colspan="5">NG</td></tr>
<tr><td>${}^{1}V_2{}^{2}V_1{}^{3}V_2{}^{4}V_3$</td><td colspan="5">NG</td></tr>
<tr><td>${}^{1}V_2{}^{2}V_1{}^{3}V_4{}^{4}V_1$</td><td colspan="5">NG</td></tr>
<tr><td>${}^{1}V_2{}^{2}V_2{}^{3}V_2{}^{4}V_1$</td><td colspan="5">NG</td></tr>
<tr><td>${}^{1}V_2{}^{2}V_2{}^{3}V_2{}^{4}V_2$</td><td colspan="5">NG</td></tr>
<tr><td>${}^{1}V_2{}^{2}V_3{}^{3}V_2{}^{4}V_1$</td><td colspan="5">NG</td></tr>
</table>

$^{1}V_{3}{}^{2}V_{1}{}^{3}V_{1}{}^{4}V_{1}$	NG
$^{1}V_{3}{}^{2}V_{1}{}^{3}V_{1}{}^{4}V_{2}$	NG
$^{1}V_{3}{}^{2}V_{1}{}^{3}V_{1}{}^{4}V_{3}$	NG
$^{1}V_{3}{}^{2}V_{1}{}^{3}V_{3}{}^{4}V_{1}$	NG
$^{1}V_{3}{}^{2}V_{2}{}^{3}V_{1}{}^{4}V_{1}$	NG
$^{1}V_{3}{}^{2}V_{2}{}^{3}V_{1}{}^{4}V_{2}$	NG
$^{1}V_{3}{}^{2}V_{3}{}^{3}V_{1}{}^{4}V_{1}$	NG
$^{1}V_{4}{}^{2}V_{1}{}^{3}V_{2}{}^{4}V_{1}$	NG
$^{1}V_{5}{}^{2}V_{1}{}^{3}V_{1}{}^{4}V_{1}$	NG
Vertex connectivities ($^{c}V_{r}$) where e_A is odd cannot form graphs and are not listed.<br><br>NG = adjacency matrices not generated.	
