# Supplementary material for: Bond topology of chain, ribbon and tube silicates. Part I. Graph-theory generation of infinite one-dimensional arrangements of (TO4) n− tetrahedra
Source: Acta Crystallogr A Found Adv. 2022 Apr 4;78(Pt 3):212–33. doi: 10.1107/S2053273322001747 (PMC9062827; doi:10.1107/S2053273322001747)
Supplement: Supplementary file 5 [file a-78-00212-sup5.pdf]

FOUNDATIONS ADVANCES

Volume 78 (2022)

Supporting information for article:

Bond topology of chain, ribbon and tube silicates. Part I. Graph-theory generation of infinite one-dimensional arrangements of $(TO4)^{n-}$ tetrahedra

Maxwell Christopher Day and Frank Christopher Hawthorne

Appendix F. Matrix element combinations and associated non-isomorphic proto-graphs for vertex connectivities ($^{c}V_{r}$) where $\sum r \leq 8$.

$\boldsymbol{^{c}V_{r}}$				
**Rank 1**				
$^{0}V_{1\text{-}8}$	isolated tetrahedra (nesosilicates)			
$^{1}V_{2}$	(2x**1**) 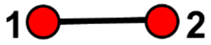			
$^{1}V_{4}$	(4x**1**) 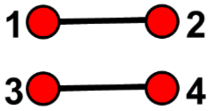			
$^{1}V_{6}$	(6x**1**) 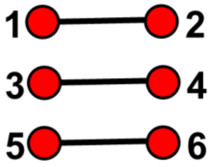			
$^{1}V_{8}$	(8x**1**) 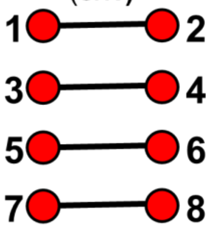			

$^{2}V_{1}$	(1x**2**)			
$^{2}V_{2}$	(2x**2**)	(2x$\mathbf{2}^{1}$)	(2x$\mathbf{2}^{2}$)	
$^{2}V_{3}$	(6x**1**)	(3x**2**)	(1x**2**, 2x$\mathbf{2}^{1}$)	(1x**2**, 2x$\mathbf{2}^{2}$)
$^{2}V_{4}$	(8x**1**)	(6x**1**, 1x**2**)	(4x**2**)	(2x**2**, 2x$\mathbf{2}^{1}$)
	(2x**2**, 2x$\mathbf{2}^{2}$)	(4x$\mathbf{2}^{1}$)	(2x$\mathbf{2}^{1}$, 2x$\mathbf{2}^{2}$)	(4x$\mathbf{2}^{2}$)

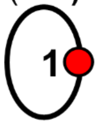

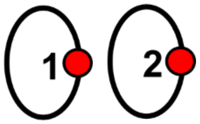

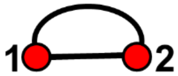

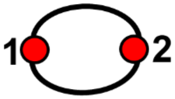

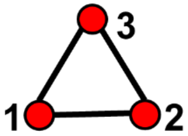

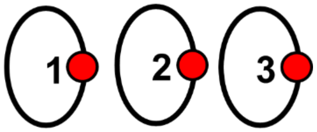

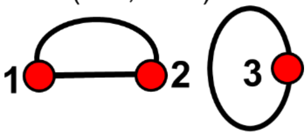

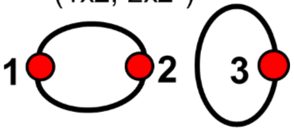

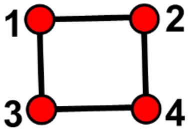

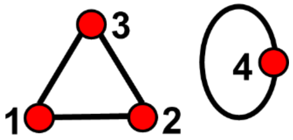

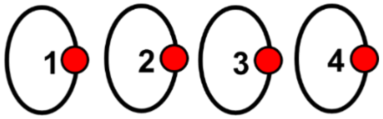

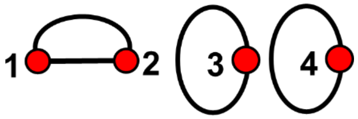

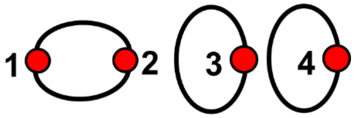

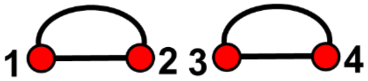

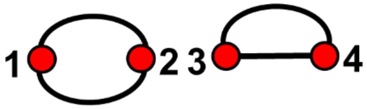

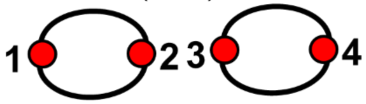

<table>
<tr><td rowspan="3">$^{2}V_{5}$</td><td>(10x1)
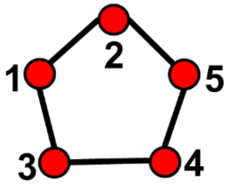
</td><td>(8x1, 1x2)
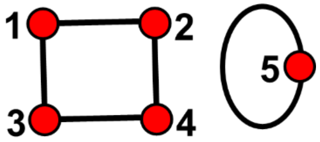
</td><td>(6x1, 2x2)
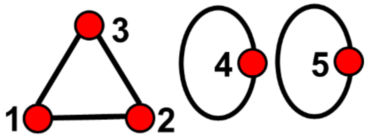
</td><td>(6x1, 2x2^{1})
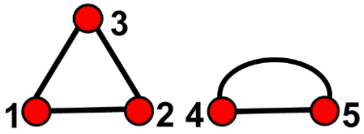
</td></tr>
<tr><td>(6x1, 2x2^{2})
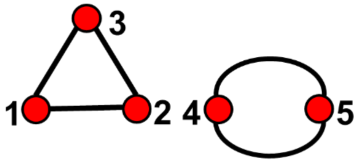
</td><td>(5x2)
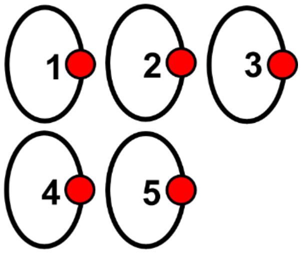
</td><td>(3x2, 2x2^{1})
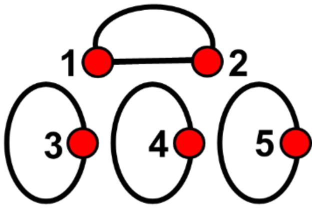
</td><td>(3x2, 2x2^{2})
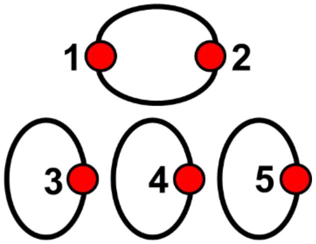
</td></tr>
<tr><td>(1x2, 4x2^{1})
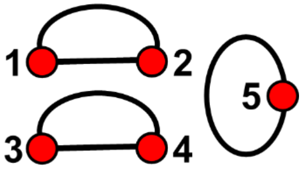
</td><td>(1x2, 2x2^{1}, 2x2^{2})
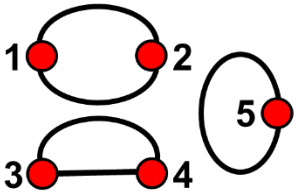
</td><td>(1x2, 4x2^{2})
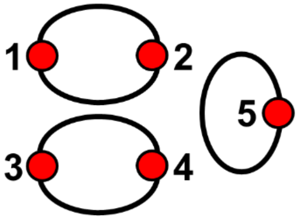
</td><td></td></tr>
<tr><td>$^{2}V_{6}$</td><td>(12x1) a
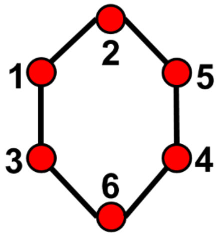
</td><td>(12x1) b
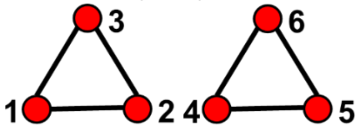
</td><td>(10x1, 1x2)
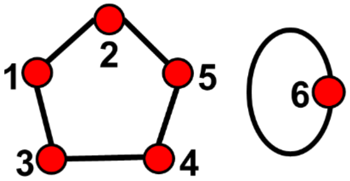
</td><td>(8x1, 2x2)
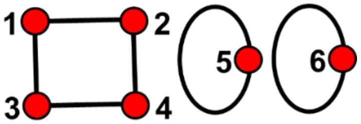
</td></tr>
</table>

	(8x**1**, 2x**2**^{1}) 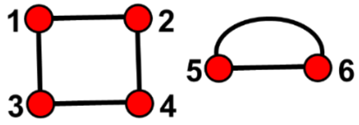	(8x**1**, 2x**2**^{2}) 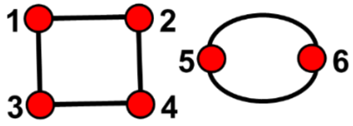	(6x**1**, 3x**2**) 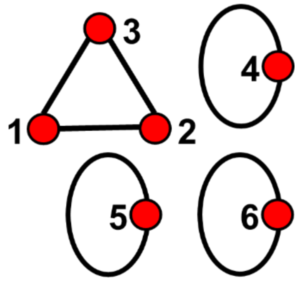	(6x**1**, 1x**2**, 2x**2**^{1}) 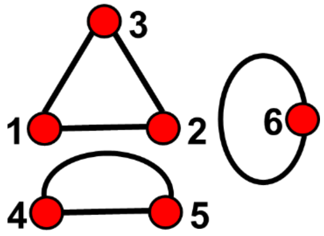
	(6x**1**, 1x**2**, 2x**2**^{2}) 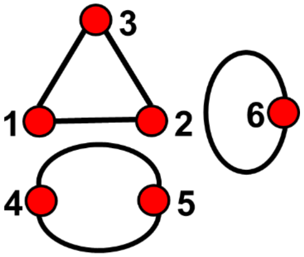	(6x**2**) 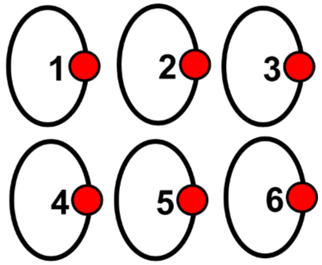	(4x**2**, 2x**2**^{1}) 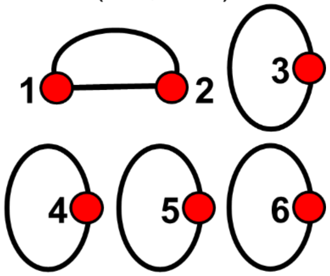	(4x**2**, 2x**2**^{2}) 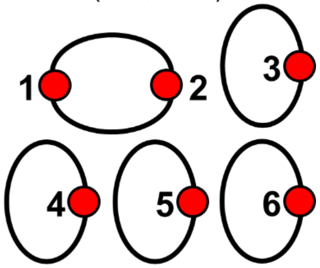
	(2x**2**, 4x**2**^{1}) 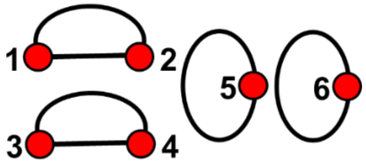	(2x**2**, 2x**2**^{1}, 2x**2**^{2}) 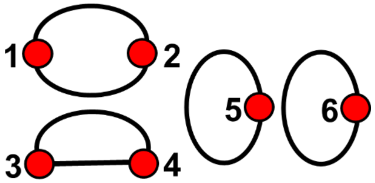	(2x**2**, 4x**2**^{2}) 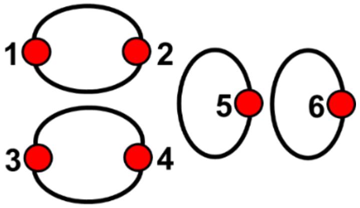	(6x**2**^{1}) 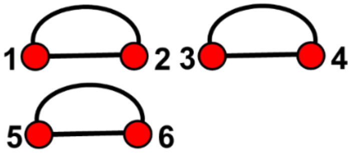

	(4x$\mathbf{2}^1$, 2x$\mathbf{2}^2$) 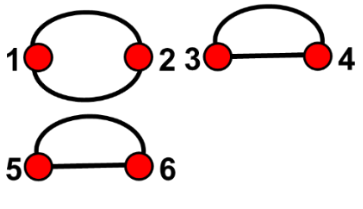	(2x$\mathbf{2}^1$, 4x$\mathbf{2}^2$) 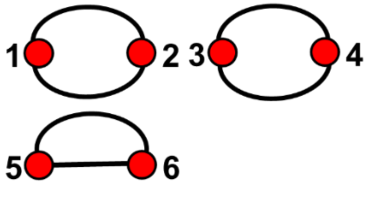	(6x$\mathbf{2}^2$) 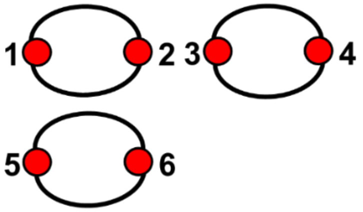	
$^2V_7$	(14x**1**) **a** 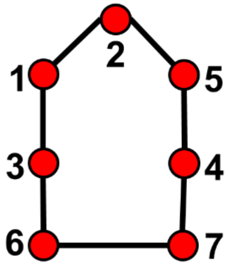	(14x**1**) **b** 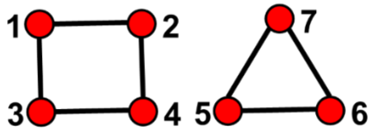	(12x**1**, 1x**2**) **a** 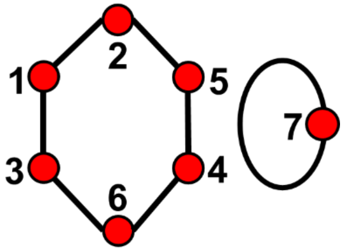	(12x**1**, 1x**2**) **b** 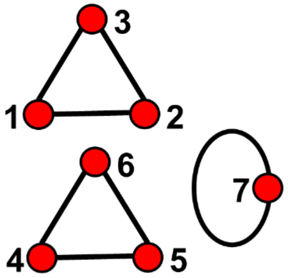
	(10x**1**, 2x**2**) 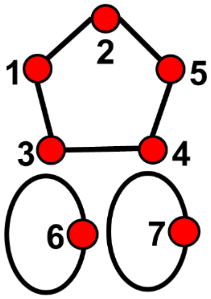	(10x**1**, 2x$\mathbf{2}^1$) 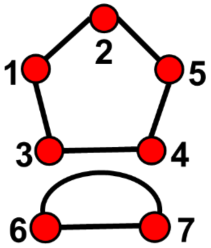	(10x**1**, 2x$\mathbf{2}^2$) 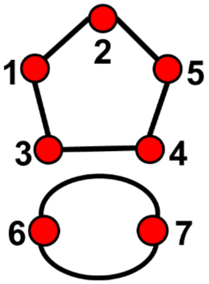	(8x**1**, 3x**2**) 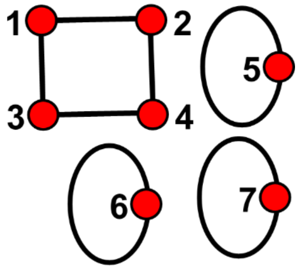

	(8x**1**, 1x**2**, 2x$\mathbf{2}^1$) 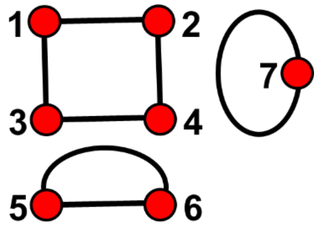	(8x**1**, 1x**2**, 2x$\mathbf{2}^2$) 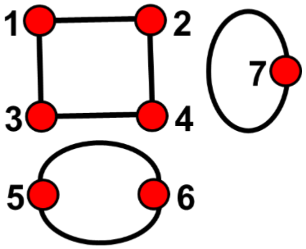	(6x**1**, 4x**2**) 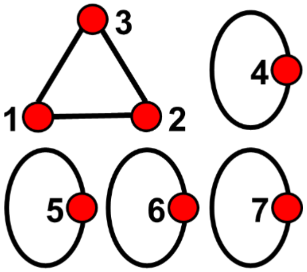	(6x**1**, 2x**2**, 2x$\mathbf{2}^1$) 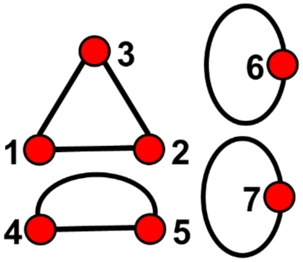
	(6x**1**, 2x**2**, 2x$\mathbf{2}^2$) 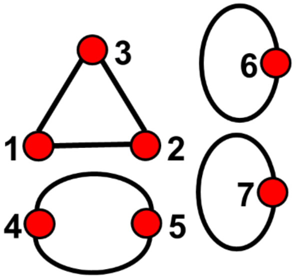	(6x**1**, 4x$\mathbf{2}^1$) 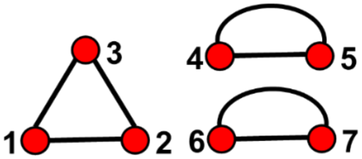	(6x**1**, 2x$\mathbf{2}^1$, 2x$\mathbf{2}^2$) 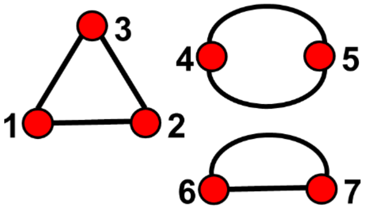	(6x**1**, 4x$\mathbf{2}^2$) 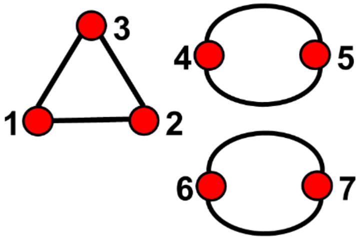
	(7x**2**) 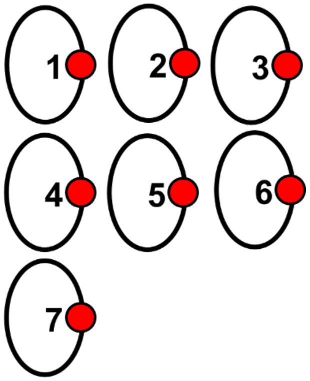	(5x**2**, 2x$\mathbf{2}^1$) 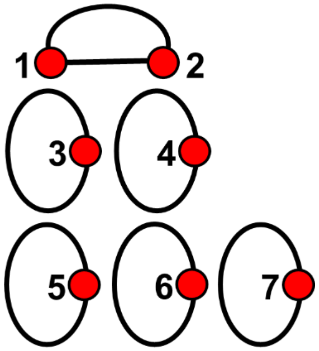	(5x**2**, 2x$\mathbf{2}^2$) 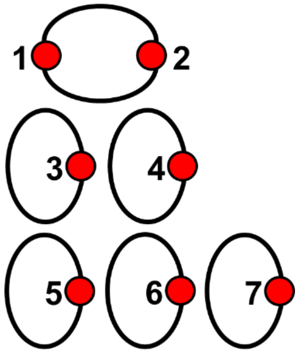	(3x**2**, 4x$\mathbf{2}^1$) 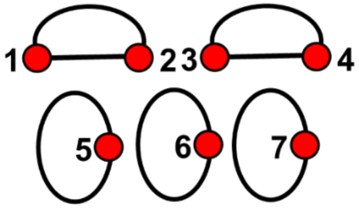

	(3x$\mathbf{2}$, 2x$\mathbf{2}^1$, 2x$\mathbf{2}^2$)<br>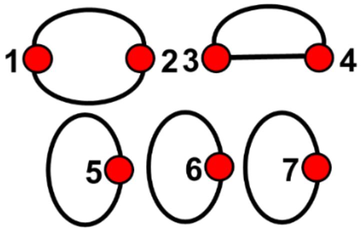	(3x$\mathbf{2}$, 4x$\mathbf{2}^2$)<br>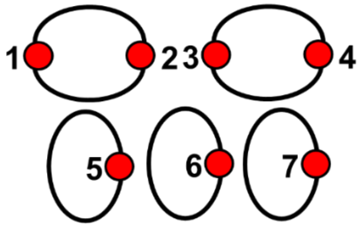	(1x$\mathbf{2}$, 6x$\mathbf{2}^1$)<br>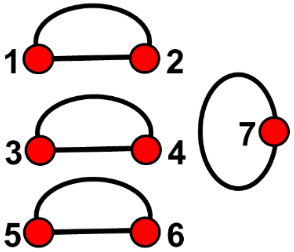	(1x$\mathbf{2}$, 4x$\mathbf{2}^1$, 2x$\mathbf{2}^2$)<br>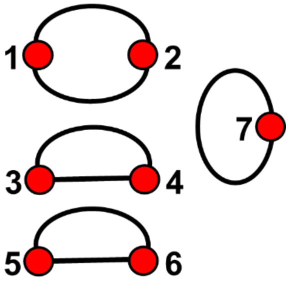
	(1x$\mathbf{2}$, 2x$\mathbf{2}^1$, 4x$\mathbf{2}^2$)<br>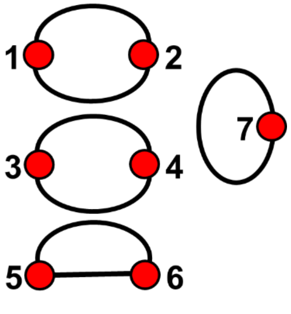	(1x$\mathbf{2}$, 6x$\mathbf{2}^2$)<br>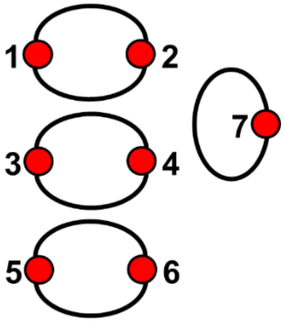		
$^{2}V_{8}$	(16x**1**) **a**<br>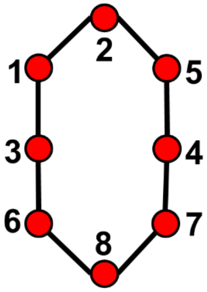	(16x**1**) **b**<br>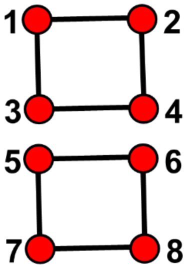	(16x**1**) **c**<br>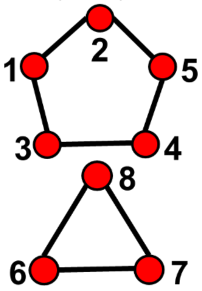	(14x**1**, 1x**2**) **a**<br>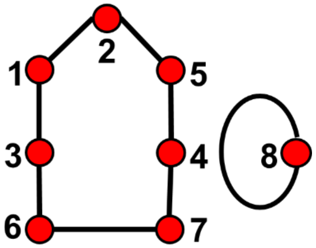

	(14x**1**, 1x**2**) **b** 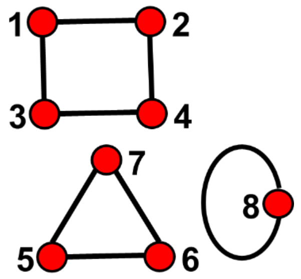	(12x**1**, 2x**2**) **a** 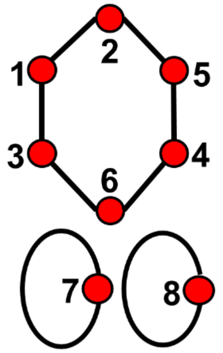	(12x**1**, 2x**2**) **b** 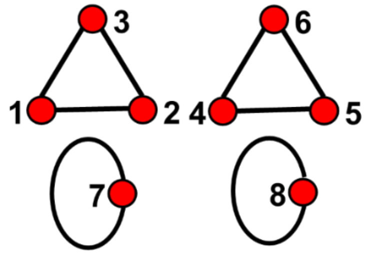	(12x**1**, 2x**2**^{1}) **a** 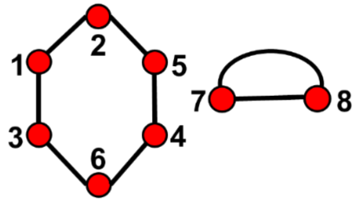
	(12x**1**, 2x**2**^{1}) **b** 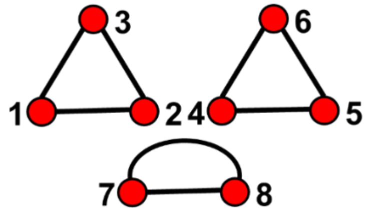	(12x**1**, 2x**2**^{2}) **a** 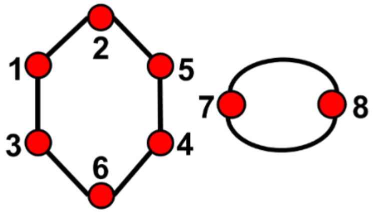	(12x**1**, 2x**2**^{2}) **b** 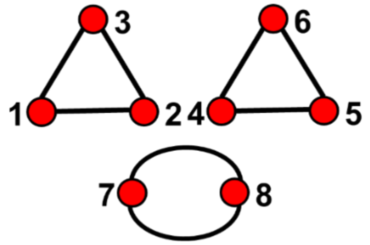	(10x**1**, 3x**2**) 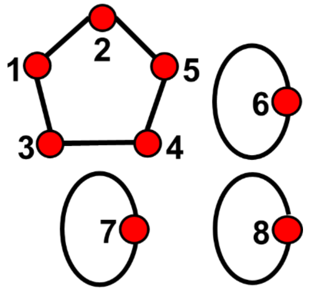
	(10x**1**, 1x**2**, 2x**2**^{1}) 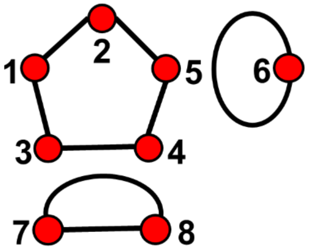	(10x**1**, 1x**2**, 2x**2**^{2}) 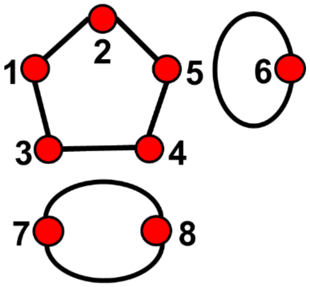	(8x**1**, 4x**2**) 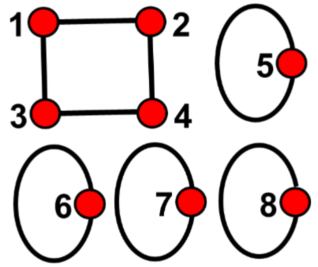	(8x**1**, 2x**2**, 2x**2**^{1}) 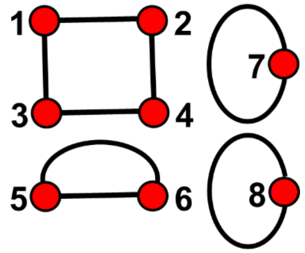

	(8x**1**, 2x**2**, 2x**2**^{2})<br>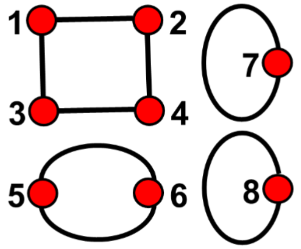	(8x**1**, 4x**2**^{1})<br>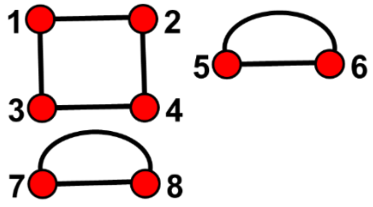	(8x**1**, 2x**2**^{1}, 2x**2**^{2})<br>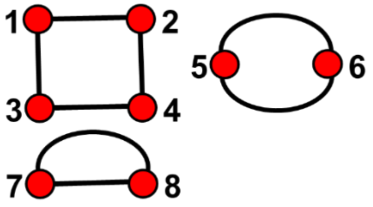	(8x**1**, 4x**2**^{2})<br>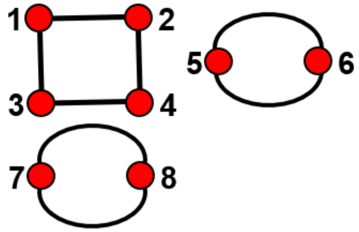
	(6x**1**, 5x**2**)<br>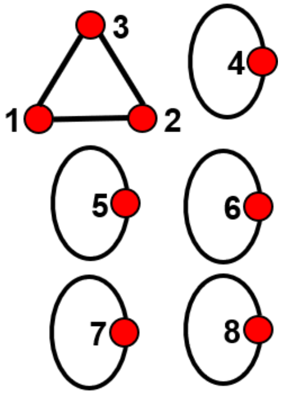	(6x**1**, 3x**2**, 2x**2**^{1})<br>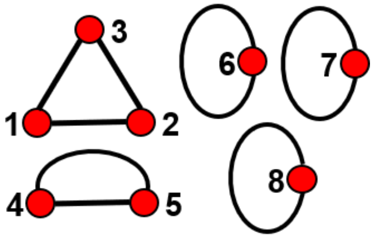	(6x**1**, 3x**2**, 2x**2**^{2})<br>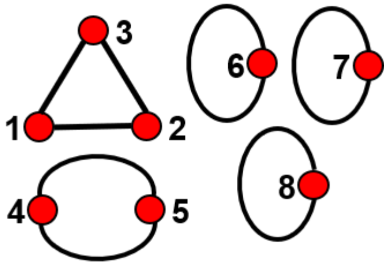	(6x**1**, 1x**2**, 4x**2**^{1})<br>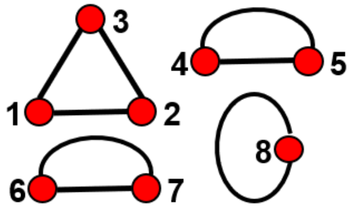
	(6x**1**, 1x**2**, 2x**2**^{1}, 2x**2**^{2})<br>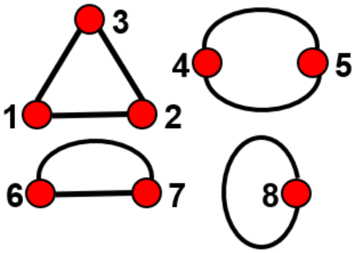	(6x**1**, 1x**2**, 4x**2**^{2})<br>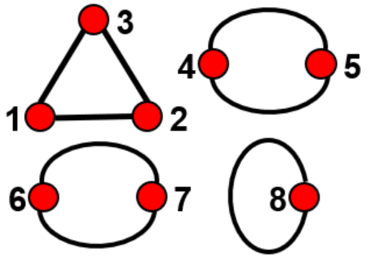	(8x**2**)<br>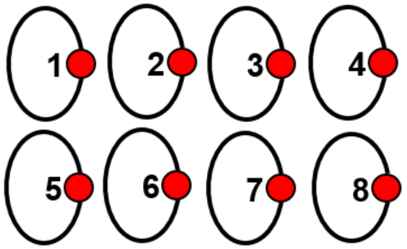	(6x**2**, 2x**2**^{1})<br>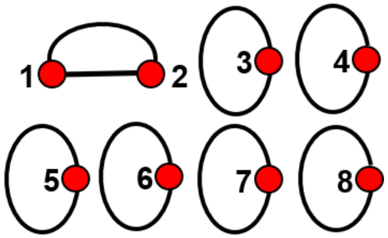

(6x$\mathbf{2}$, 2x$\mathbf{2}^{2}$)

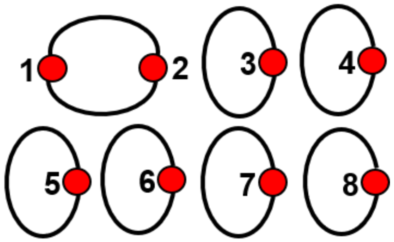

(4x$\mathbf{2}$, 4x$\mathbf{2}^{1}$)

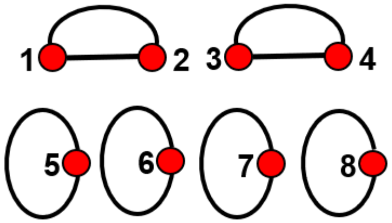

(4x$\mathbf{2}$, 2x$\mathbf{2}^{1}$, 2x$\mathbf{2}^{2}$)

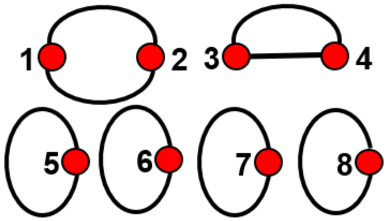

(4x$\mathbf{2}$, 4x$\mathbf{2}^{2}$)

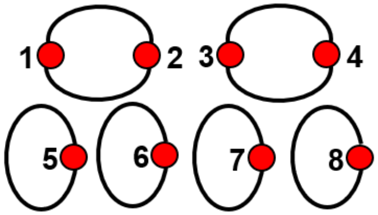

(2x$\mathbf{2}$, 6x$\mathbf{2}^{1}$)

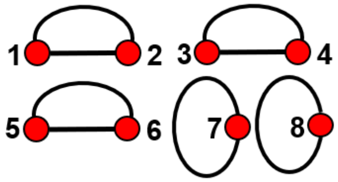

(2x$\mathbf{2}$, 4x$\mathbf{2}^{1}$, 2x$\mathbf{2}^{2}$)

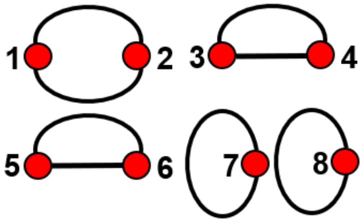

(2x$\mathbf{2}$, 2x$\mathbf{2}^{1}$, 4x$\mathbf{2}^{2}$)

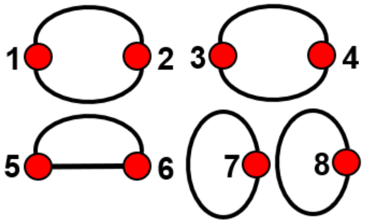

(2x$\mathbf{2}$, 6x$\mathbf{2}^{2}$)

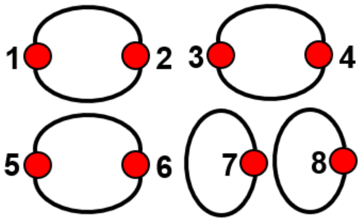

(8x$\mathbf{2}^{1}$)

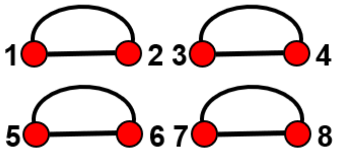

(6x$\mathbf{2}^{1}$, 2x$\mathbf{2}^{2}$)

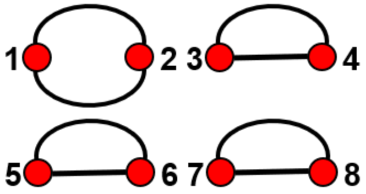

(4x$\mathbf{2}^{1}$, 4x$\mathbf{2}^{2}$)

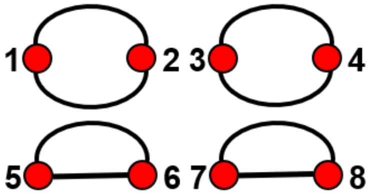

(2x$\mathbf{2}^{1}$, 6x$\mathbf{2}^{2}$)

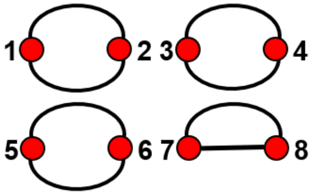

(8x$\mathbf{2}^{2}$)

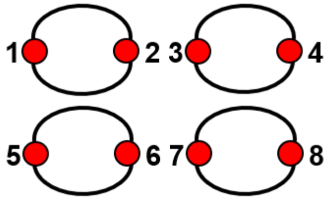

<table>
<tr>
<td>$^{3}V_{2}$</td>
<td>(2x**1**, 2x**2**)
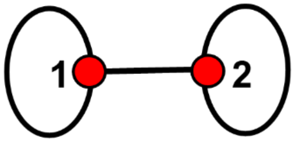</td>
<td></td>
<td></td>
<td></td>
</tr>
<tr>
<td rowspan="2">$^{3}V_{4}$</td>
<td>(12x**1**)
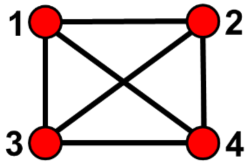</td>
<td>(6x**1**, 3x**2**)
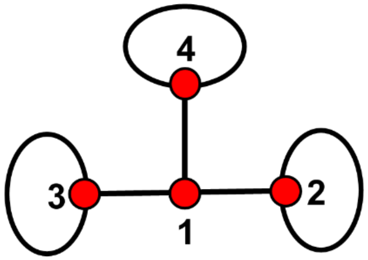</td>
<td>(6x**1**, 1x**2**, 2x$\mathbf{2}^{1}$)
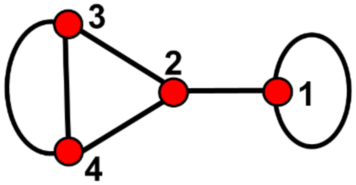</td>
<td>(6x**1**, 1x**2**, 2x$\mathbf{2}^{2}$)
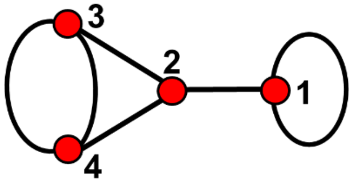</td>
</tr>
<tr>
<td>(4x**1**, 4x**2**)
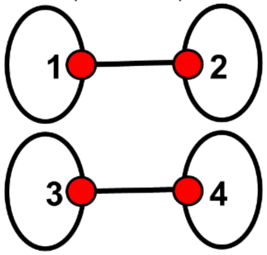</td>
<td>(4x**1**, 2x**2**, 2x$\mathbf{2}^{1}$)
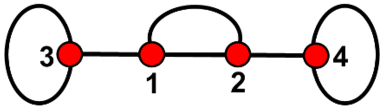</td>
<td>(4x**1**, 2x**2**, 2x$\mathbf{2}^{2}$)
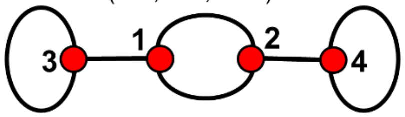</td>
<td>(4x**1**, 4x$\mathbf{2}^{1}$)
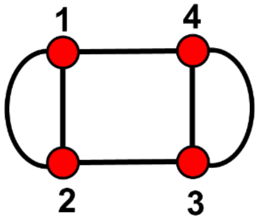</td>
</tr>
</table>

	(4x**1**, 2x**2**^{1}, 2x**2**^{2}) 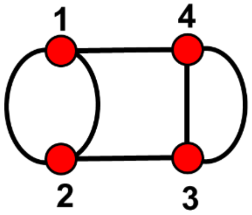	(4x**1**, 4x**2**^{2}) 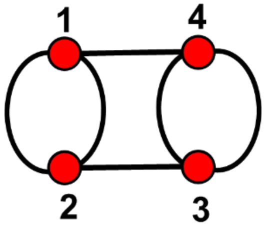		
$^3V_6$				
$^3V_8$				
$^4V_2$	(2x**2**, 2x**2**^{1}) 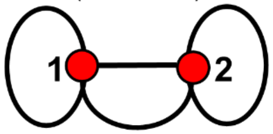	(2x**2**, 2x**2**^{2}) 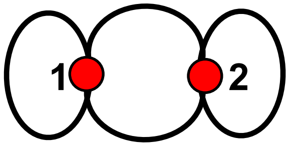		
$^4V_3$	(6x**1**, 3x**2**) 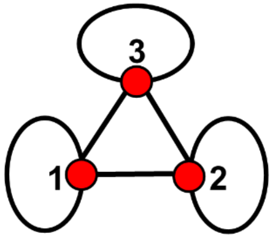	(2x**2**, 4x**2**^{1}) 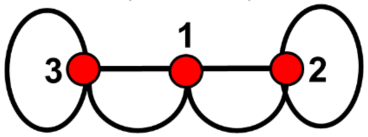	(2x**2**, 2x**2**^{1}, 2x**2**^{2}) 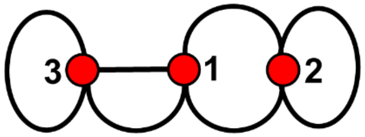	(2x**2**, 4x**2**^{2}) 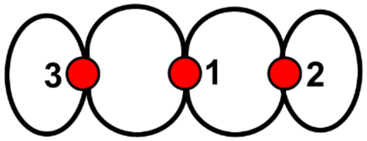

	(6x$\mathbf{2}^1$) 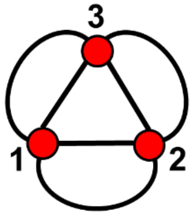	(4x$\mathbf{2}^1$, 2x$\mathbf{2}^2$) 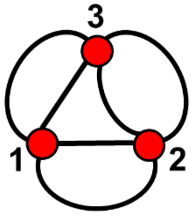	(2x$\mathbf{2}^1$, 4x$\mathbf{2}^2$) 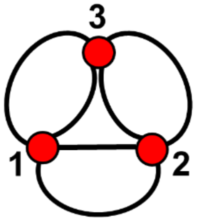	(6x$\mathbf{2}^2$) 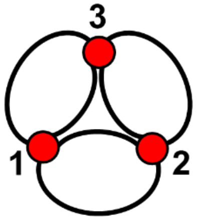
${}^4V_4$	(8x**1**, 4x**2**) 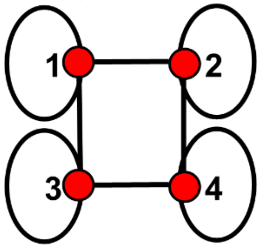	(8x**1**, 2x**2**, 2x$\mathbf{2}^1$) 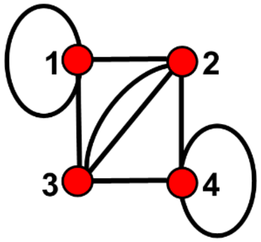	(8x**1**, 2x**2**, 2x$\mathbf{2}^2$) 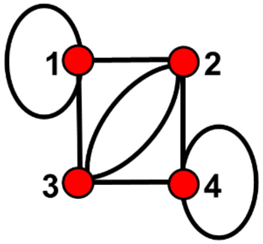	(8x**1**, 4x$\mathbf{2}^1$) 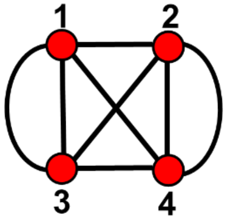
	(8x**1**, 2x$\mathbf{2}^1$, 2x$\mathbf{2}^2$) 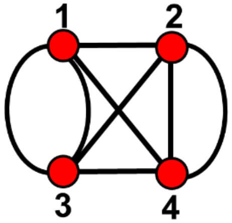	(8x**1**, 4x$\mathbf{2}^2$) 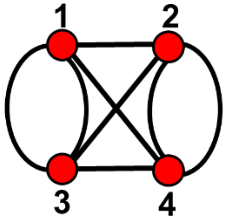	(6x**1**, 3x**2**, 2x$\mathbf{2}^1$) 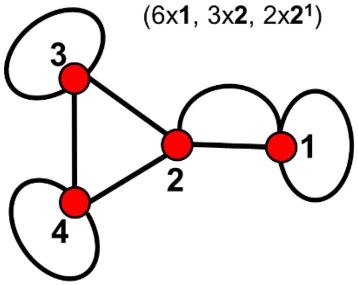	(6x**1**, 3x**2**, 2x$\mathbf{2}^2$) 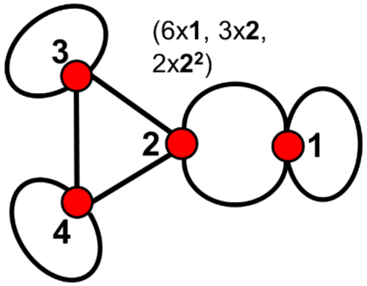

(6x**1**, 1x**2**, 4x$\mathbf{2}^{1}$)

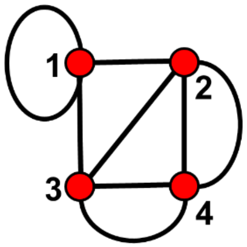

(6x**1**, 1x**2**, 2x$\mathbf{2}^{1}$, 2x$\mathbf{2}^{2}$)

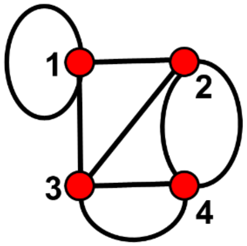

(6x**1**, 1x**2**, 4x$\mathbf{2}^{2}$)

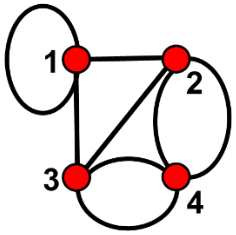

(4x**2**, 4x$\mathbf{2}^{1}$)

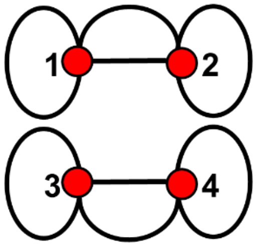

(4x**2**, 2x$\mathbf{2}^{1}$, 2x$\mathbf{2}^{2}$)

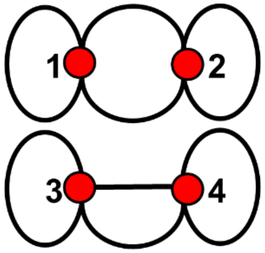

(4x**2**, 4x$\mathbf{2}^{2}$)

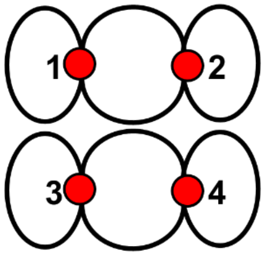

(2x**2**, 6x$\mathbf{2}^{1}$)

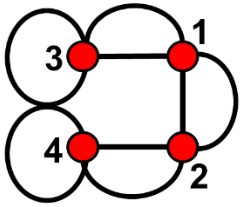

(2x**2**, 4x$\mathbf{2}^{1}$, 2x$\mathbf{2}^{2}$) **a**

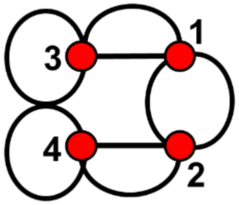

(2x**2**, 4x$\mathbf{2}^{1}$, 2x$\mathbf{2}^{2}$) **b**

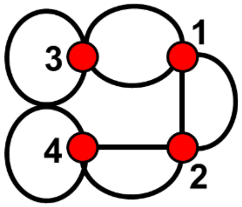

(2x**2**, 2x$\mathbf{2}^{1}$, 4x$\mathbf{2}^{2}$) **a**

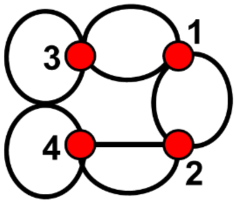

(2x**2**, 2x$\mathbf{2}^{1}$, 4x$\mathbf{2}^{2}$) **b**

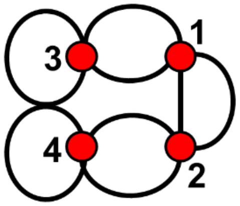

(2x**2**, 6x$\mathbf{2}^{2}$)

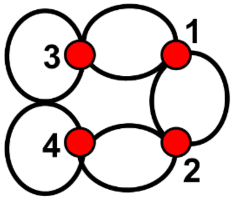

	(8x$\mathbf{2}^1$)<br>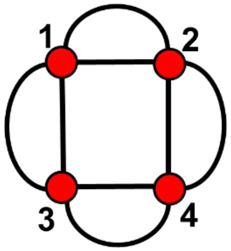	(6x$\mathbf{2}^1$, 2x$\mathbf{2}^2$)<br>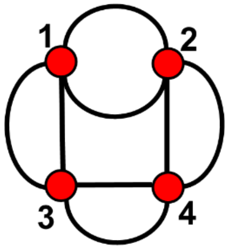	(4x$\mathbf{2}^1$, 4x$\mathbf{2}^2$) **a**<br>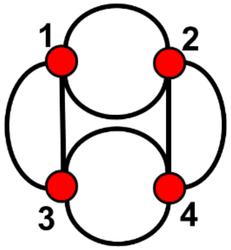	(4x$\mathbf{2}^1$, 4x$\mathbf{2}^2$) **b**<br>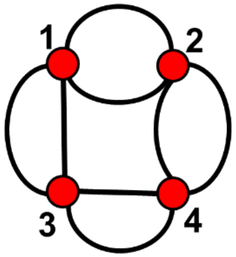
	(2x$\mathbf{2}^1$, 6x$\mathbf{2}^2$)<br>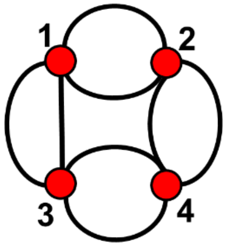	(8x$\mathbf{2}^2$)<br>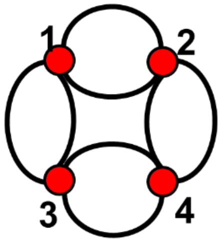		
$^4V_5$	NG			
$^4V_6$	NG			
$^4V_7$	NG			
$^4V_8$	NG			
**Rank 2**				
$^1V_2{}^2V_1$	(4x**1**)<br>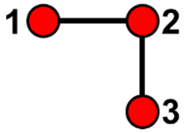	(2x**1**, 1x**2**)<br>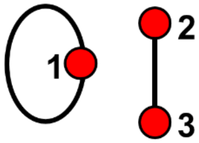		

$^1V_2{}^2V_2$	(6x**1**) 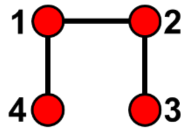	(4x**1**, 1x**2**) 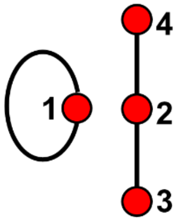	(2x**1**, 2x**2**) 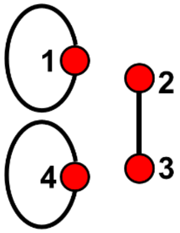	(2x**1**, 2x**2**^{1}) 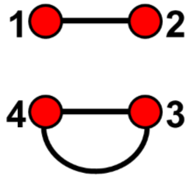
	(2x**1**, 2x**2**^{2}) 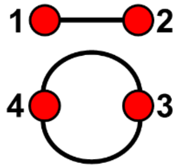			
$^1V_2{}^2V_3$	(8x**1**) **a** 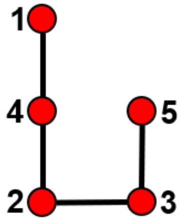	(8x**1**) **b** 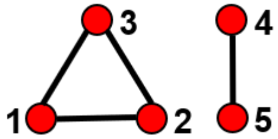	(6x**1**, 1x**2**) 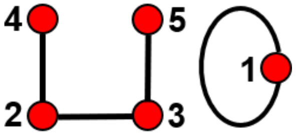	(4x**1**, 2x**2**) 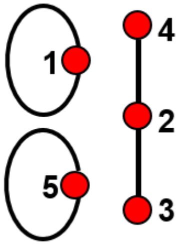
	(4x**1**, 2x**2**^{1}) 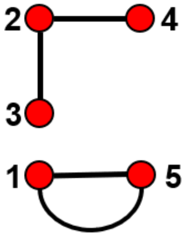	(4x**1**, 2x**2**^{2}) 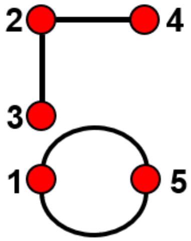	(2x**1**, 3x**2**) 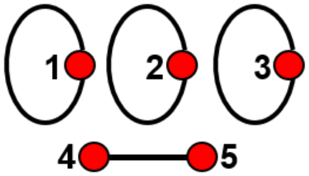	(2x**1**, 1x**2**, 2x**2**^{1}) 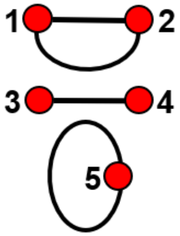

	(2x**1**, 1x**2**, 2x**2**^{2}) 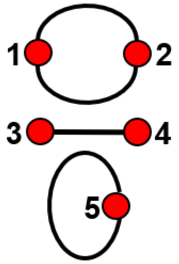			
$^1V_2{}^2V_4$	(10x**1**) **a** 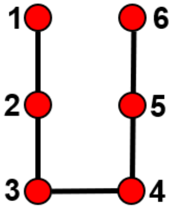	(10x**1**) **b** 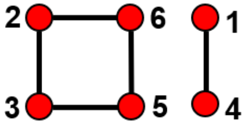	(10x**1**) **c** 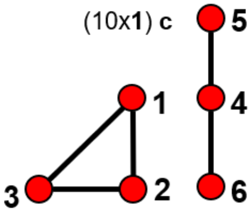	(8x**1**, 1x**2**) **a** 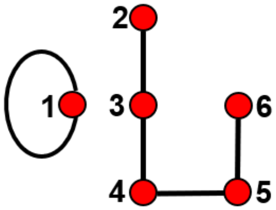
	(8x**1**, 1x**2**) **b** 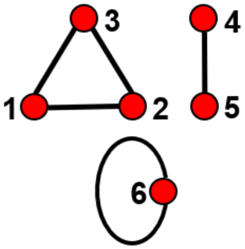	(6x**1**, 2x**2**) 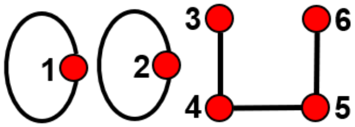	(6x**1**, 2x**2**^{1}) 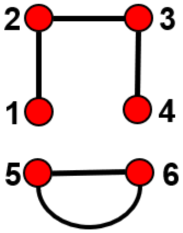	(6x**1**, 2x**2**^{2}) 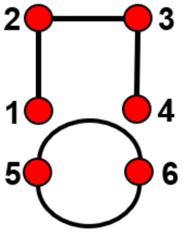
	(4x**1**, 3x**2**) 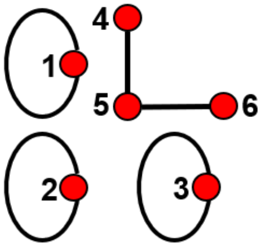	(4x**1**, 1x**2**, 2x**2**^{1}) 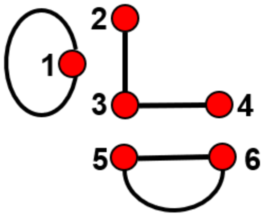	(4x**1**, 1x**2**, 2x**2**^{2}) 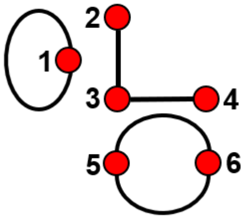	(2x**1**, 4x**2**) 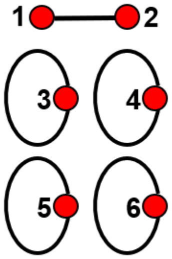

	(2x**1**, 2x**2**, 2x**2**^{1})<br>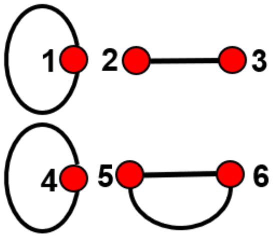	(2x**1**, 2x**2**, 2x**2**^{2})<br>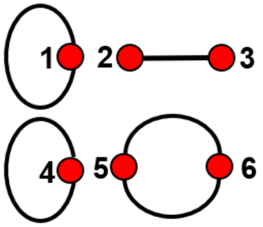	(2x**1**, 4x**2**^{1})<br>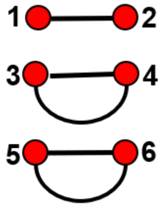	(2x**1**, 2x**2**^{1}, 2x**2**^{2})<br>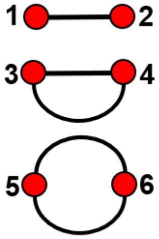
	(2x**1**, 4x**2**^{2})<br>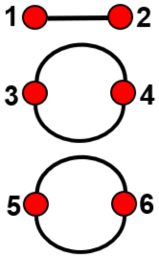			
$^1V_2{}^2V_5$	(12x**1**) **a**<br>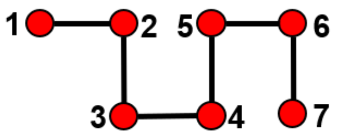	(12x**1**) **b**<br>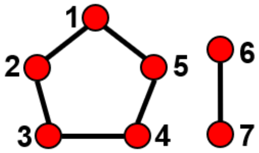	(12x**1**) **c**<br>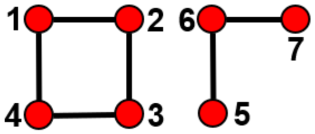	(12x**1**) **d**<br>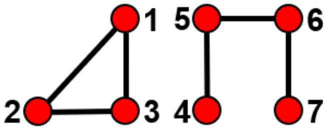
	(10x**1**, 1x**2**) **a**<br>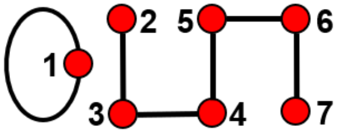	(10x**1**, 1x**2**) **b**<br>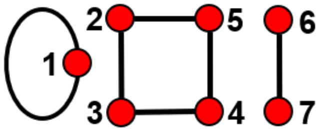	(10x**1**, 1x**2**) **c**<br>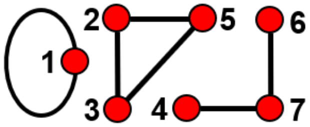	(8x**1**, 2x**2**) **a**<br>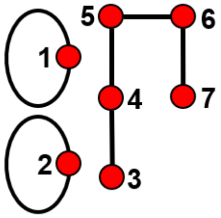

	(8x**1**, 2x**2**) **b** 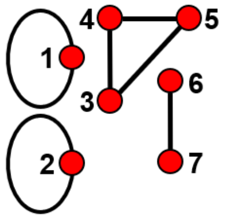	(8x**1**, 2x$\mathbf{2}^1$) **a** 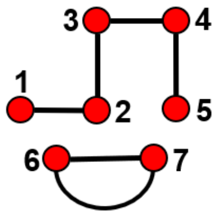	(8x**1**, 2x$\mathbf{2}^1$) **b** 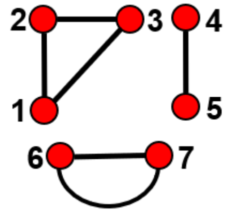	(8x**1**, 2x$\mathbf{2}^2$) **a** 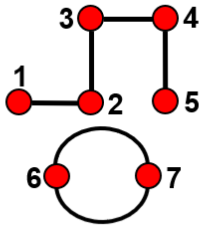
	(8x**1**, 2x$\mathbf{2}^2$) **b** 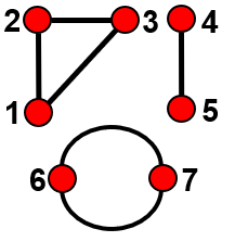	(6x**1**, 3x**2**) 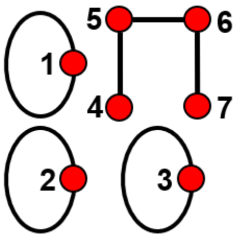	(6x**1**, 1x**2**, 2x$\mathbf{2}^1$) 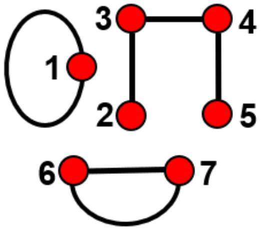	(6x**1**, 1x**2**, 2x$\mathbf{2}^2$) 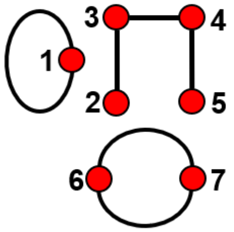
	(4x**1**, 4x**2**) 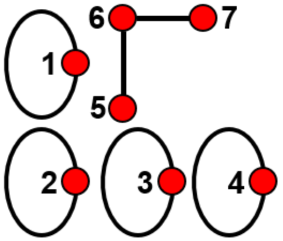	(4x**1**, 2x**2**, 2x$\mathbf{2}^1$) 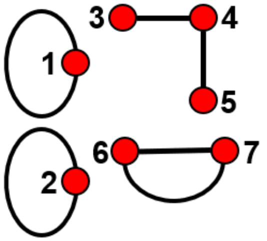	(4x**1**, 2x**2**, 2x$\mathbf{2}^2$) 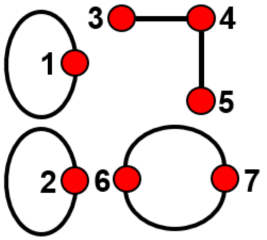	(4x**1**, 4x$\mathbf{2}^1$) 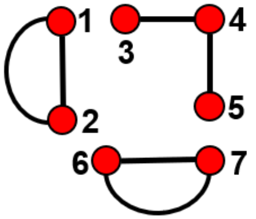
	(4x**1**, 2x$\mathbf{2}^1$, 2x$\mathbf{2}^2$) 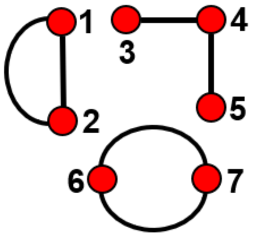	(4x**1**, 4x$\mathbf{2}^2$) 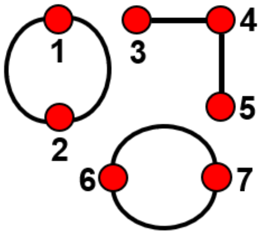	(2x**1**, 5x**2**) 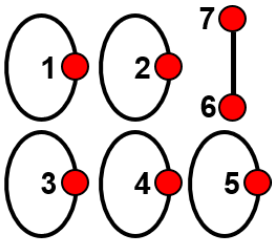	(2x**1**, 3x**2**, 2x$\mathbf{2}^1$) 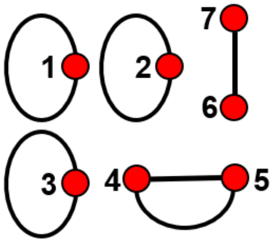

	(2x**1**, 3x**2**, 2x**2**^{2}) 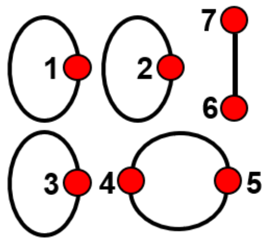	(2x**1**, 1x**2**, 4x**2**^{1}) 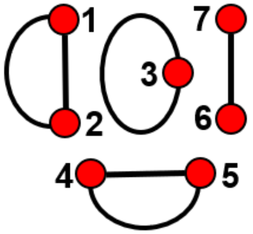	(2x**1**, 1x**2**, 2x**2**^{1}, 2x**2**^{2}) 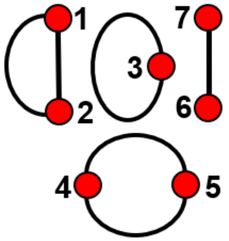	(2x**1**, 1x**2**, 4x**2**^{2}) 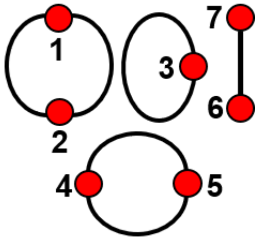
$^{1}V_{2}{}^{2}V_{6}$	NG			
$^{1}V_{4}{}^{2}V_{1}$	(6x**1**) 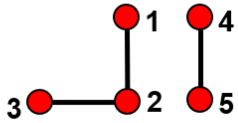	(4x**1**, 1x**2**) 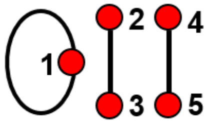		
$^{1}V_{4}{}^{2}V_{2}$	(8x**1**) **a** 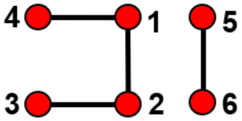	(8x**1**) **b** 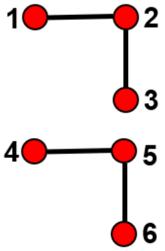	(6x**1**, 1x**2**) 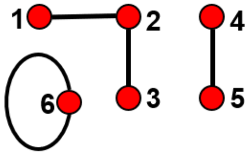	(4x**1**, 2x**2**) 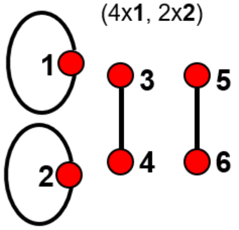
	(4x**1**, 2x**2**^{1}) 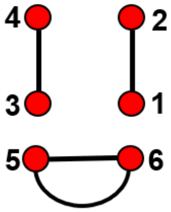	(4x**1**, 2x**2**^{2}) 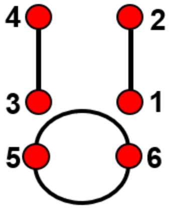		

$^{1}V_{4}{}^{2}V_{3}$

(10x**1**) **a**

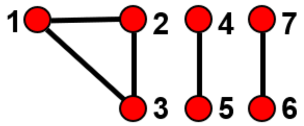

(10x**1**) **b**

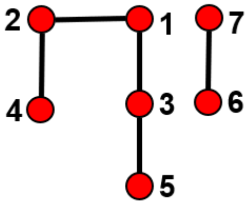

(10x**1**) **c**

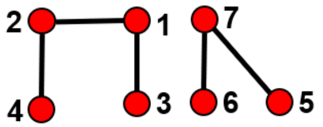

(8x**1**, 1x**2**) **a**

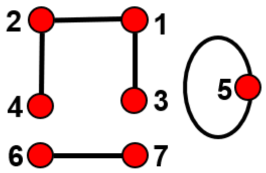

(8x**1**, 1x**2**) **b**

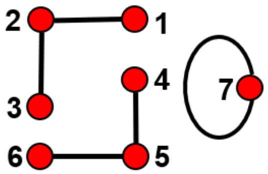

(6x**1**, 2x**2**)

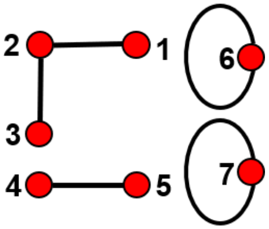

(6x**1**, 2x**2**[1])

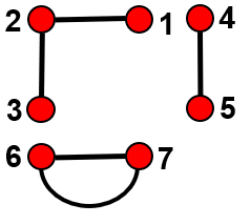

(6x**1**, 2x**2**[2])

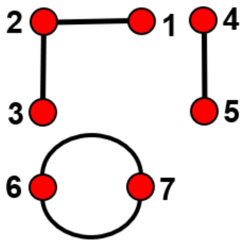

(4x**1**, 3x**2**)

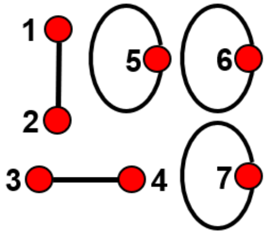

(4x**1**, 1x**2**, 2x**2**[1])

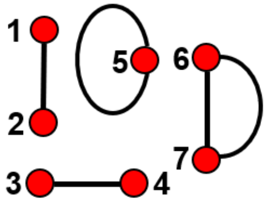

(4x**1**, 1x**2**, 2x**2**[2])

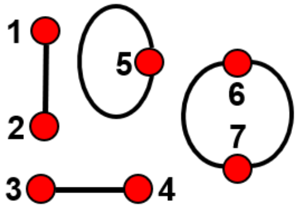

$^{1}V_{4}{}^{2}V_{4}$

(12x**1**) **a**

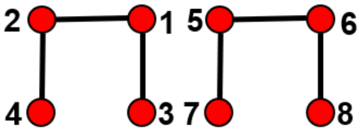

(12x**1**) **b**

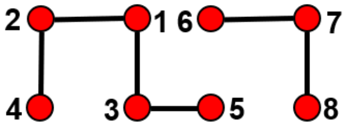

(12x**1**) **c**

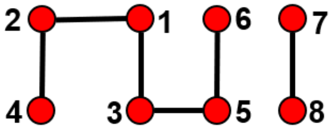

(12x**1**) **d**

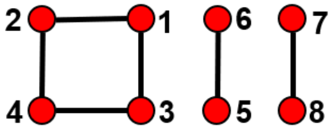

	(12x**1**) **e**<br>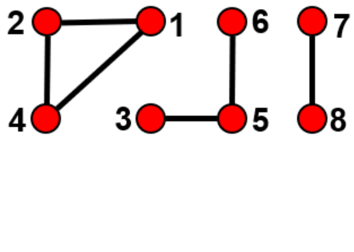	(10x**1**, 1x**2**) **a**<br>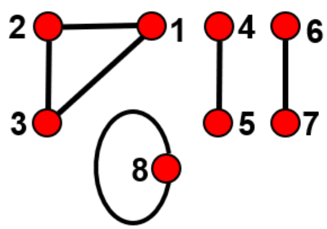	(10x**1**, 1x**2**) **b**<br>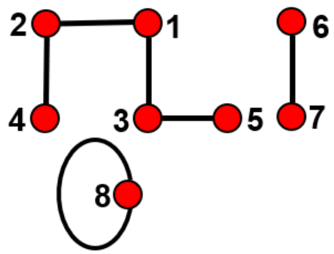	(10x**1**, 1x**2**) **c**<br>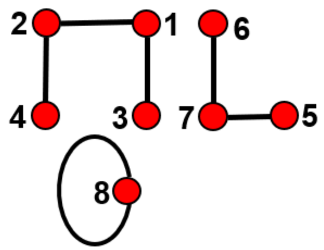
	(8x**1**, 2x**2**) **a**<br>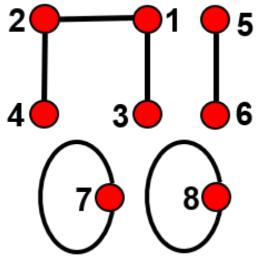	(8x**1**, 2x**2**) **b**<br>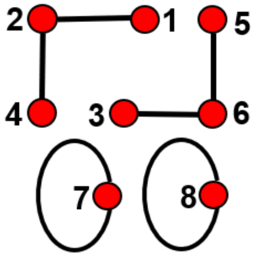	(8x**1**, 2x**2**^{1}) **a**<br>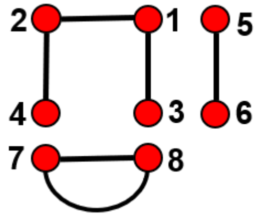	(8x**1**, 2x**2**^{2}) **a**<br>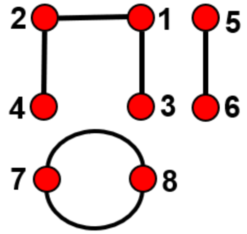
	(8x**1**, 2x**2**^{1}) **b**<br>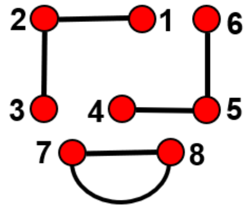	(8x**1**, 2x**2**^{2}) **b**<br>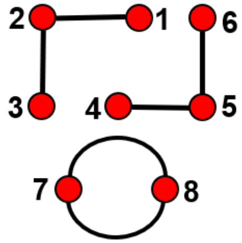	(6x**1**, 3x**2**)<br>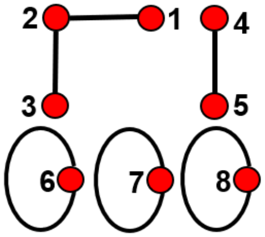	(6x**1**, 1x**2**, 2x**2**^{1})<br>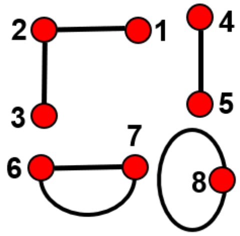
	(6x**1**, 1x**2**, 2x**2**^{2})<br>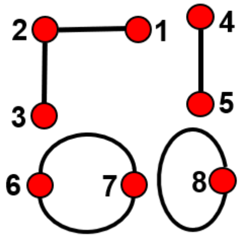	(4x**1**, 4x**2**)<br>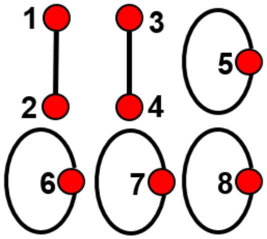	(4x**1**, 2x**2**, 2x**2**^{1})<br>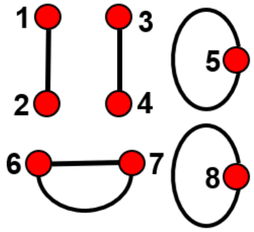	(4x**1**, 2x**2**, 2x**2**^{2})<br>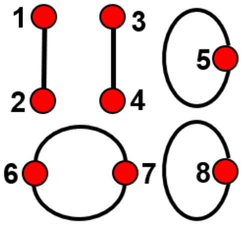

	(4x**1**, 4x$\mathbf{2}^1$) 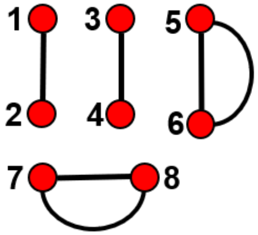	(4x**1**, 2x$\mathbf{2}^1$, 2x$\mathbf{2}^2$) 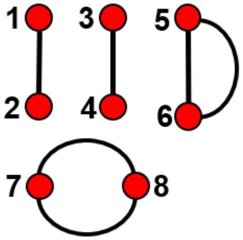	(4x**1**, 4x$\mathbf{2}^2$) 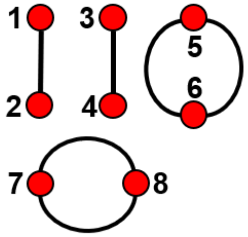	
$^1V_6{}^2V_1$	(8x**1**) 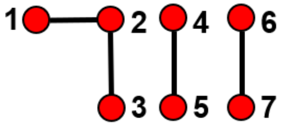	(6x**1**, 1x**2**) 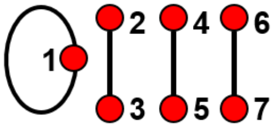		
$^1V_6{}^2V_2$	(10x**1**) **a** 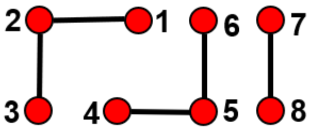	(10x**1**) **b** 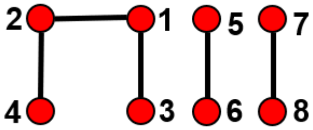	(8x**1**, 1x**2**) 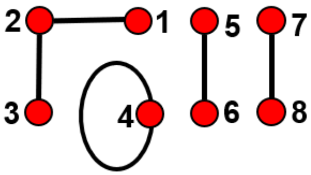	(6x**1**, 2x$\mathbf{2}^1$) 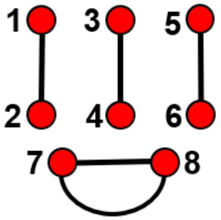
	(6x**1**, 2x$\mathbf{2}^2$) 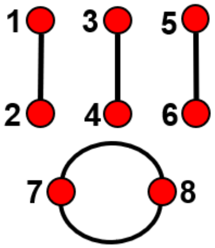	(6x**1**, 2x**2**) 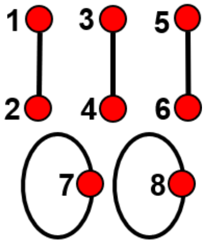		
$\mathbf{^1V_r{}^3V_r}$				

$^{1}V_{1}{}^{3}V_{1}$

(2x**1**, 1x**2**)

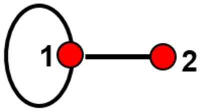

$^{1}V_{1}{}^{3}V_{3}$

(6x**1**, 2x**2**)

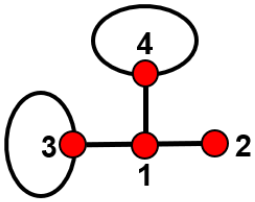

(6x**1**, 2x$\mathbf{2}^{1}$)

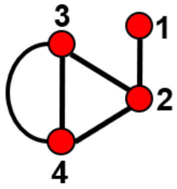

(6x**1**, 2x$\mathbf{2}^{2}$)

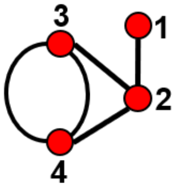

(4x**1**, 3x**2**)

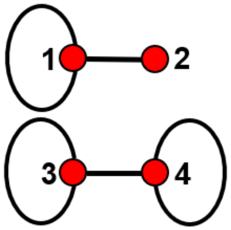

(4x**1**, 1x**2**, 2x$\mathbf{2}^{1}$)

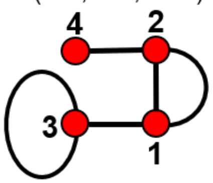

(4x**1**, 1x**2**, 2x$\mathbf{2}^{2}$)

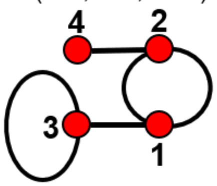

$^{1}V_{1}{}^{3}V_{5}$

(16x**1**)

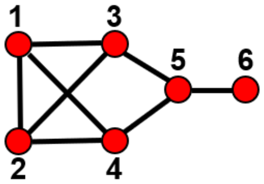

(14x**1**, 1x**2**) **a**

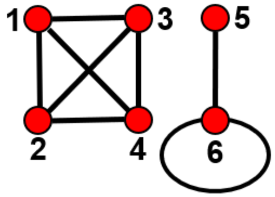

(14x**1**, 1x**2**) **b**

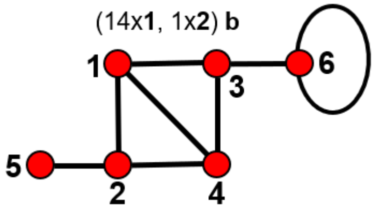

(12x**1**, 2x**2**)

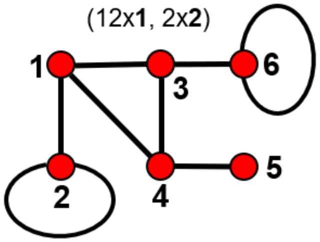

(12x**1**, 2x$\mathbf{2}^{1}$)

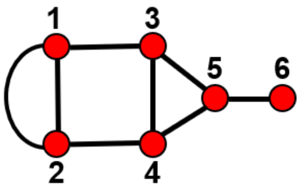

(12x**1**, 2x$\mathbf{2}^{2}$)

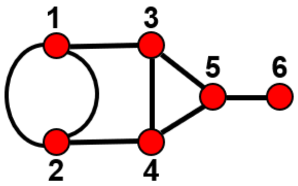

(10x**1**, 3x**2**)

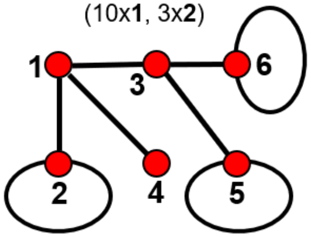

(10x**1**, 1x**2**, 2x$\mathbf{2}^{1}$) **a**

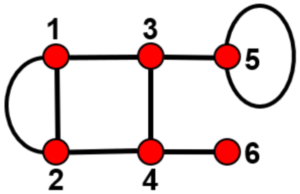

	(10x**1**, 1x**2**, 2x**2**^{1}) **b** 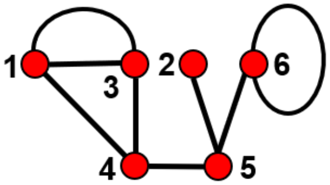	(10x**1**, 1x**2**, 2x**2**^{2}) **a** 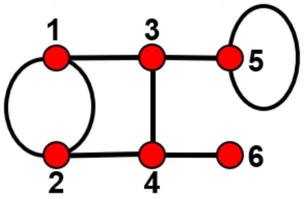	(10x**1**, 1x**2**, 2x**2**^{2}) **b** 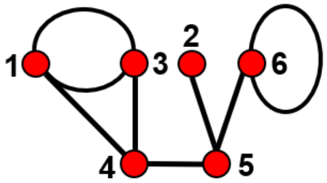	(8x**1**, 4x**2**) **a** 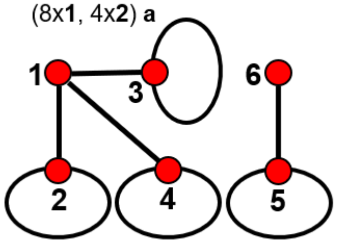
	(8x**1**, 4x**2**) **b** 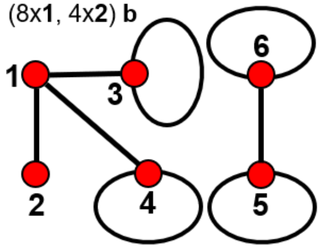	(8x**1**, 4x**2**) **c** 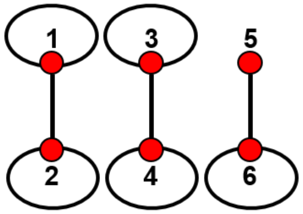	(8x**1**, 2x**2**, 2x**2**^{1}) **a** 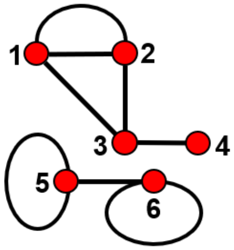	(8x**1**, 2x**2**, 2x**2**^{1}) **b** 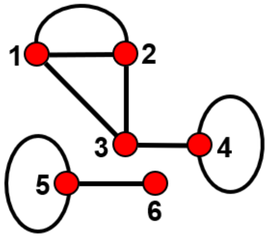
	(8x**1**, 2x**2**, 2x**2**^{1}) **c** 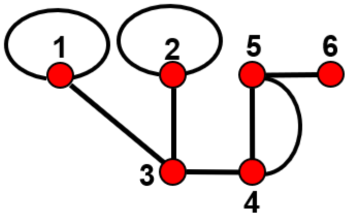	(8x**1**, 2x**2**, 2x**2**^{1}) **d** 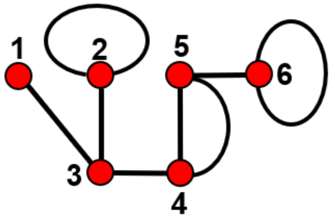	(8x**1**, 2x**2**, 2x**2**^{2}) **a** 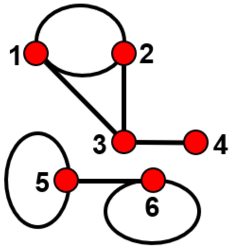	(8x**1**, 2x**2**, 2x**2**^{2}) **b** 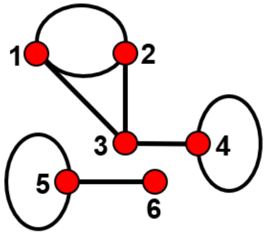
	(8x**1**, 2x**2**, 2x**2**^{2}) **c** 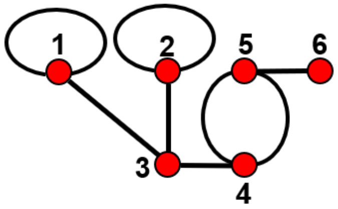	(8x**1**, 2x**2**, 2x**2**^{2}) **d** 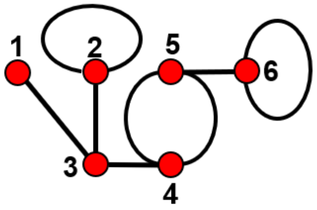	(8x**1**, 4x**2**^{1}) **a** 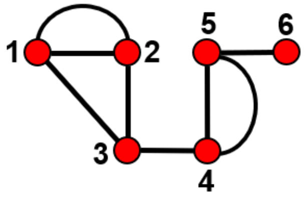	(8x**1**, 4x**2**^{1}) **b** 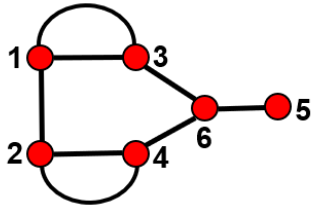

	(8x**1**, 2x**2**^{1}, 2x**2**^{2}) **a** 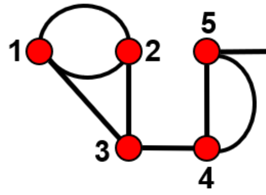	(8x**1**, 2x**2**^{1}, 2x**2**^{2}) **b** 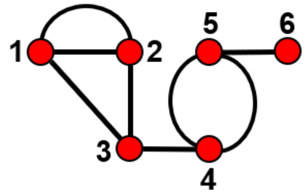	(8x**1**, 2x**2**^{1}, 2x**2**^{2}) **c** 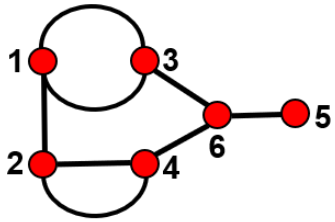	(8x**1**, 4x**2**^{2}) **a** 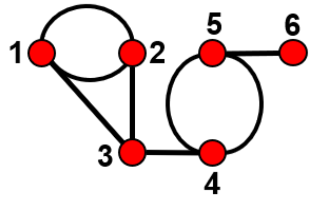
	(8x**1**, 4x**2**^{2}) **b** 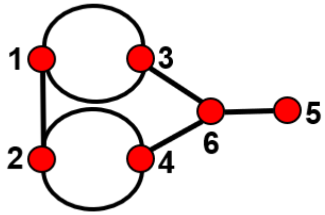	(6x**1**, 3x**2**, 2x**2**^{1}) **a** 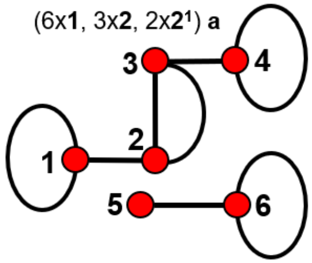	(6x**1**, 3x**2**, 2x**2**^{1}) **b** 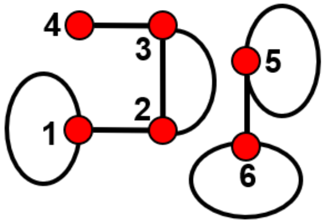	(6x**1**, 3x**2**, 2x**2**^{2}) **a** 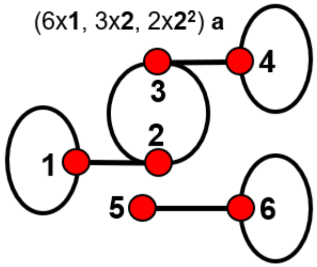
	(6x**1**, 3x**2**, 2x**2**^{2}) **b** 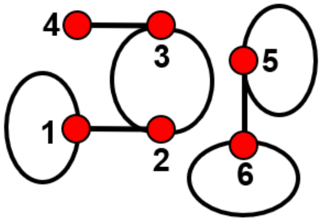	(6x**1**, 1x**2**, 4x**2**^{1}) **a** 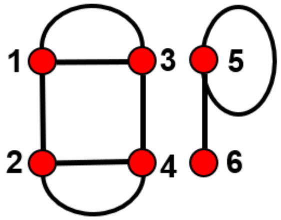	(6x**1**, 1x**2**, 4x**2**^{1}) **b** 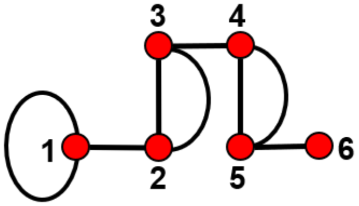	(6x**1**, 1x**2**, 2x**2**^{1}, 2x**2**^{2}) **a** 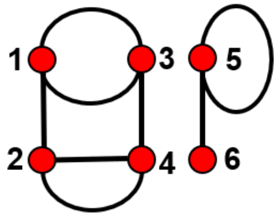
	(6x**1**, 1x**2**, 2x**2**^{1}, 2x**2**^{2}) **b** 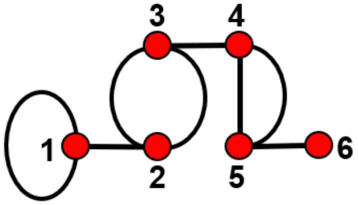	(6x**1**, 1x**2**, 2x**2**^{1}, 2x**2**^{2}) **c** 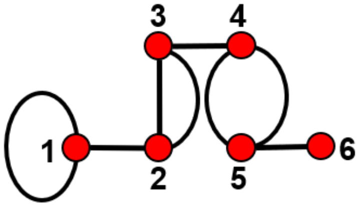	(6x**1**, 1x**2**, 4x**2**^{2}) **a** 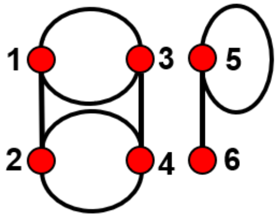	(6x**1**, 1x**2**, 4x**2**^{2}) **b** 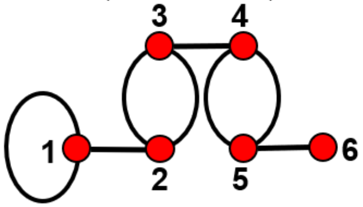
$^1V_1{}^3V_7$	NG			

$^{1}V_{2}{}^{3}V_{2}$	(6x**1**, 1x**2**)<br>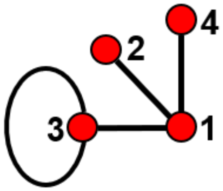	(4x**1**, 2x**2**) **a**<br>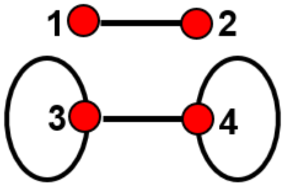	(4x**1**, 2x**2**) **b**<br>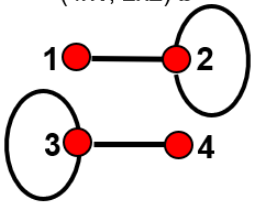	(4x**1**, 2x**2**¹)<br>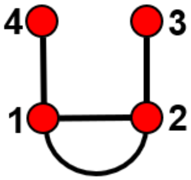
	(4x**1**, 2x**2**²)<br>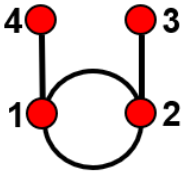			
$^{1}V_{2}{}^{3}V_{4}$	(14x**1**) **a**<br>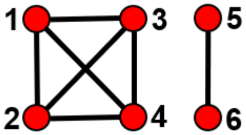	(14x**1**) **b**<br>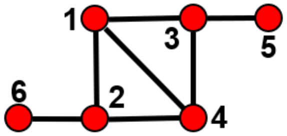	(12x**1**, 1x**2**)<br>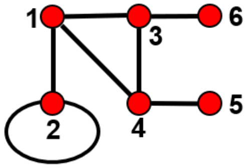	(10x**1**, 2x**2**) **a**<br>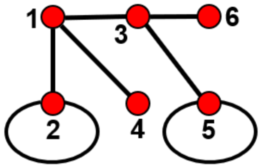
	(10x**1**, 2x**2**) **b**<br>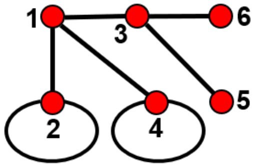	(10x**1**, 2x**2**¹) **a**<br>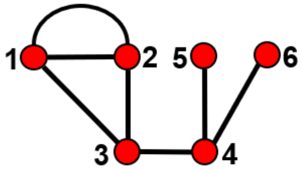	(10x**1**, 2x**2**¹) **b**<br>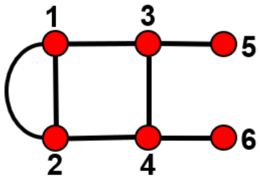	(10x**1**, 2x**2**²) **a**<br>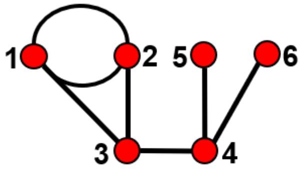
	(10x**1**, 2x**2**²) **b**<br>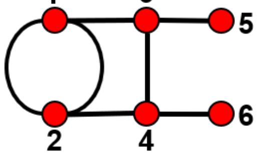	(8x**1**, 3x**2**) **a**<br>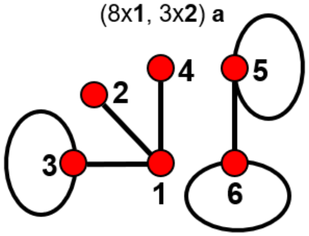	(8x**1**, 3x**2**) **b**<br>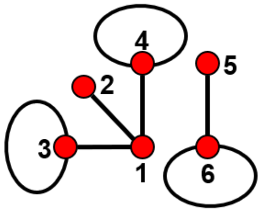	(8x**1**, 3x**2**) **c**<br>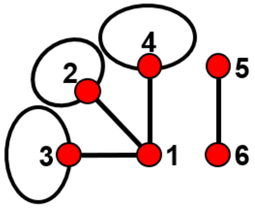

	(8x**1**, 1x**2**, 2x**2**^{1}) **a**<br>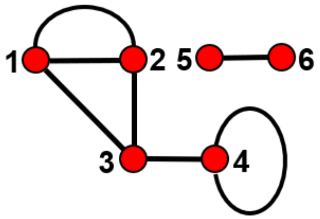	(8x**1**, 1x**2**, 2x**2**^{1}) **b**<br>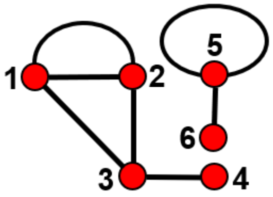	(8x**1**, 1x**2**, 2x**2**^{1}) **c**<br>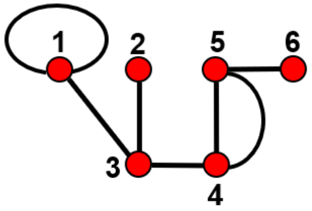	(8x**1**, 1x**2**, 2x**2**^{1}) **d**<br>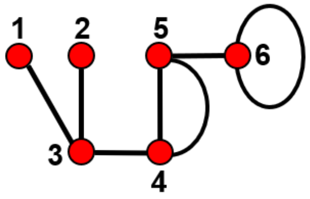
	(8x**1**, 1x**2**, 2x**2**^{2}) **a**<br>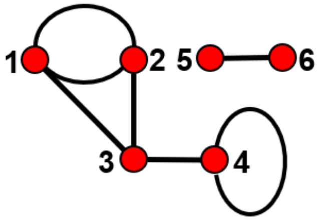	(8x**1**, 1x**2**, 2x**2**^{2}) **b**<br>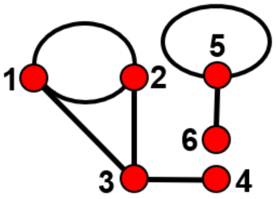	(8x**1**, 1x**2**, 2x**2**^{2}) **c**<br>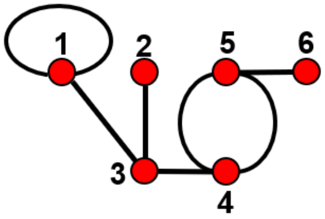	(8x**1**, 1x**2**, 2x**2**^{2}) **d**<br>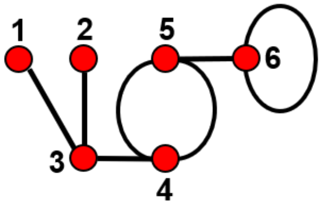
	(6x**1**, 4x**2**) **a**<br>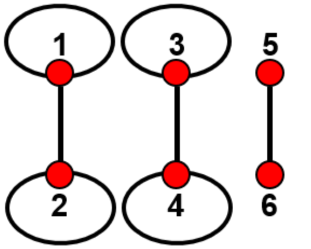	(6x**1**, 4x**2**) **b**<br>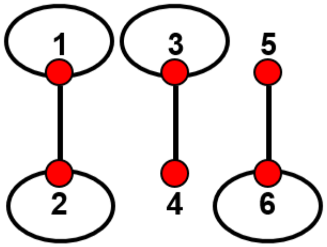	(6x**1**, 2x**2**, 2x**2**^{1}) **a**<br>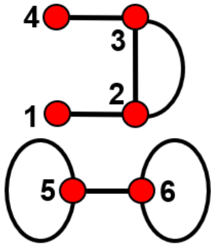	(6x**1**, 2x**2**, 2x**2**^{1}) **b**<br>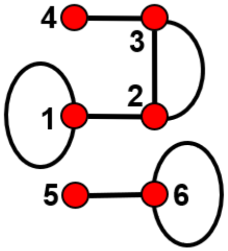
	(6x**1**, 2x**2**, 2x**2**^{1}) **c**<br>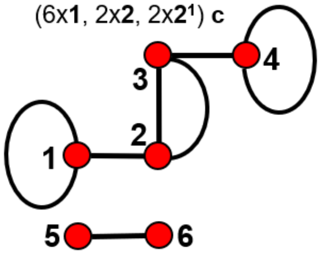	(6x**1**, 2x**2**, 2x**2**^{2}) **a**<br>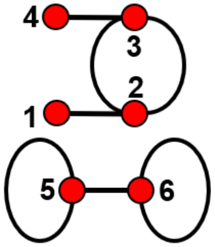	(6x**1**, 2x**2**, 2x**2**^{2}) **b**<br>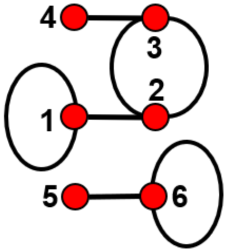	(6x**1**, 2x**2**, 2x**2**^{2}) **c**<br>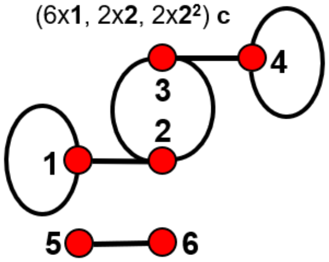

	(6x**1**, 4x**2**^{1}) **a** 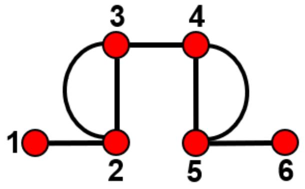	(6x**1**, 4x**2**^{1}) **b** 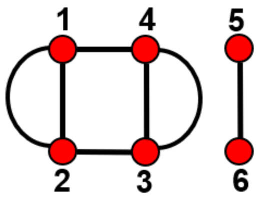	(6x**1**, 2x**2**^{1}, 2x**2**^{2}) **a** 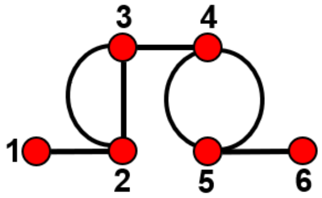	(6x**1**, 2x**2**^{1}, 2x**2**^{2}) **b** 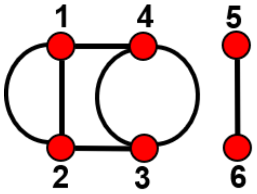
	(6x**1**, 4x**2**^{2}) **a** 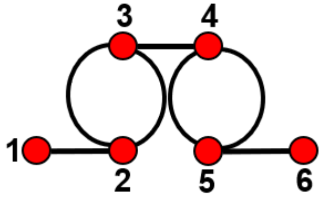	(6x**1**, 4x**2**^{2}) **b** 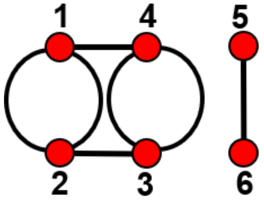		
$^1V_2{}^3V_6$	NG			
$^1V_3{}^3V_1$	(6x**1**) 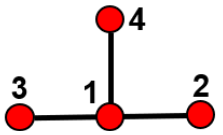	(4x**1**, 1x**2**) 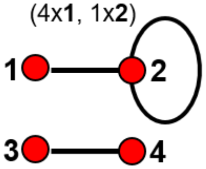		
$^1V_3{}^3V_3$	(12x**1**) 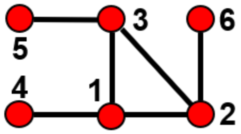	(10x**1**, 1x**2**) 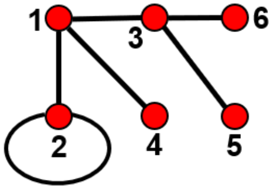	(8x**1**, 2x**2**) **a** 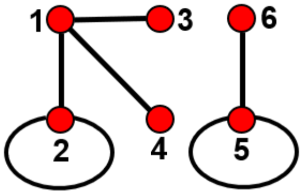	(8x**1**, 2x**2**) **b** 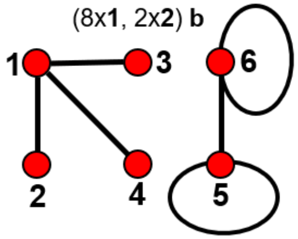

	(8x**1**, 2x**2**) **c** 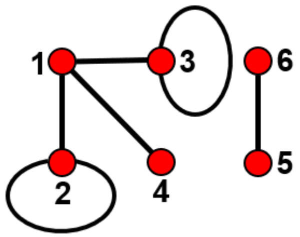	(8x**1**, 2x**2**^{1}) **a** 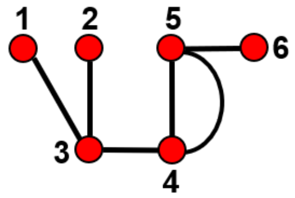	(8x**1**, 2x**2**^{1}) **b** 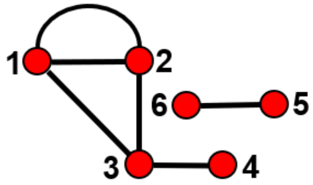	(8x**1**, 2x**2**^{2}) **a** 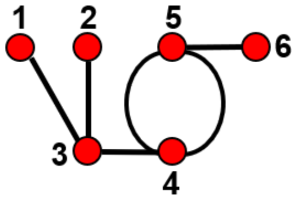
	(8x**1**, 2x**2**^{2}) **b** 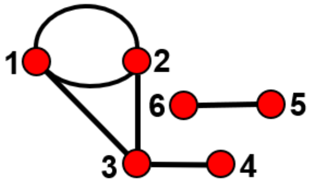	(6x**1**, 3x**2**) **a** 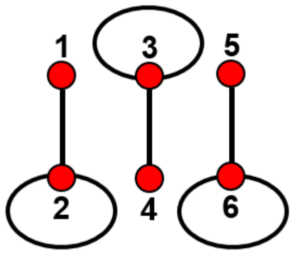	(6x**1**, 3x**2**) **b** 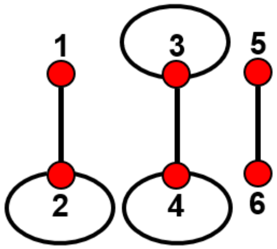	(6x**1**, 1x**2**, 2x**2**^{1}) **a** 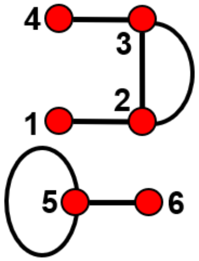
	(6x**1**, 1x**2**, 2x**2**^{1}) **b** 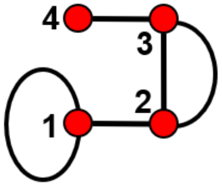	(6x**1**, 1x**2**, 2x**2**^{2}) **a** 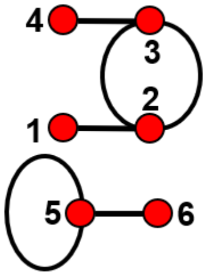	(6x**1**, 1x**2**, 2x**2**^{2}) **b** 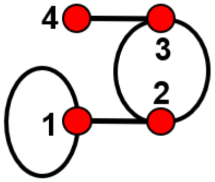	
$^{1}V_{3}{}^{3}V_{5}$	NG			

$^1V_4{}^3V_2$	(10x**1**)<br>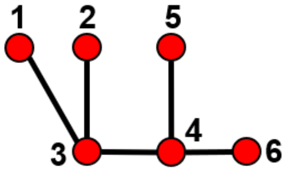	(8x**1**, 1x**2**) **a**<br>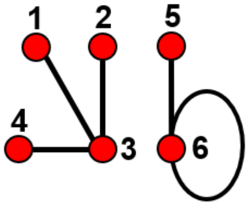	(8x**1**, 1x**2**) **b**<br>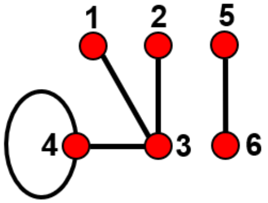	(6x**1**, 2x**2**) **a**<br>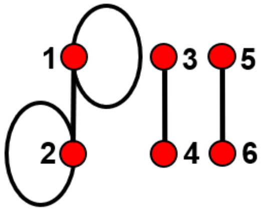
	(6x**1**, 2x**2**) **b**<br>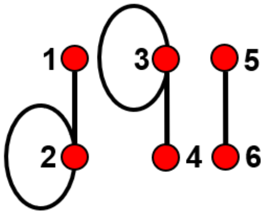	(6x**1**, 2x$\mathbf{2}^1$)<br>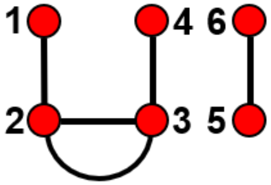	(6x**1**, 2x$\mathbf{2}^2$)<br>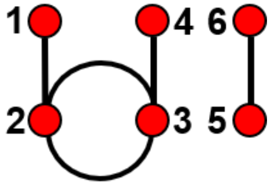	
$^1V_4{}^3V_4$	NG			
$^1V_5{}^3V_1$	(8x**1**)<br>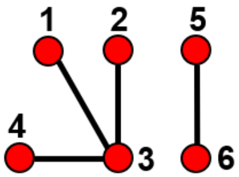	(6x**1**, 1x**2**)<br>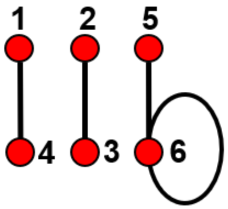		
$^1V_5{}^3V_3$	NG			
$^1V_6{}^3V_2$	NG			
$^1V_7{}^3V_1$	NG			
$\mathbf{^1V_r{}^4V_r}$				
$^1V_2{}^4V_1$	(4x**1**, 1x**2**)<br>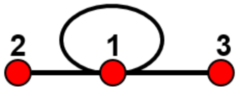			

${}^1V_2{}^4V_2$	(6x**1**, 2x**2**) 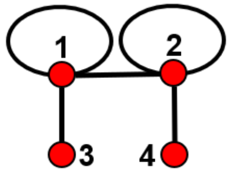	(4x**1**, 1x**2**, 2x**2**^{1}) 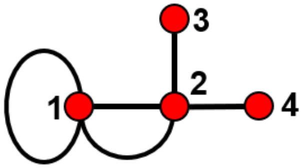	(4x**1**, 1x**2**, 2x**2**^{2}) 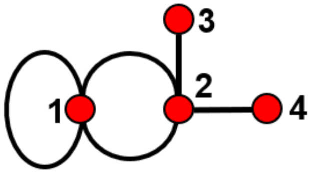	(2x**1**, 2x**2**, 2x**2**^{1}) 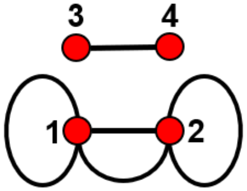
	(2x**1**, 2x**2**, 2x**2**^{2}) 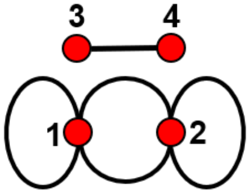			
${}^1V_2{}^4V_3$	(10x**1**, 2x**2**) 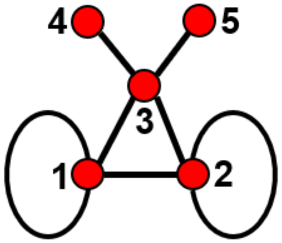	(8x**1**, 3x**2**) **a** 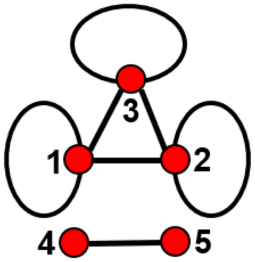	(8x**1**, 3x**2**) **b** 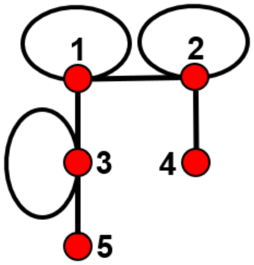	(8x**1**, 1x2, 2x**2**^{1}) 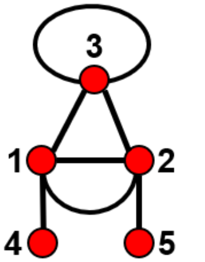
	(8x**1**, 1x2, 2x**2**^{2}) 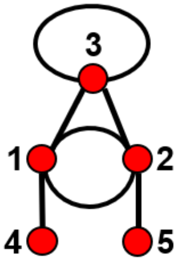	(6x**1**, 2x**2**, 2x**2**^{1}) 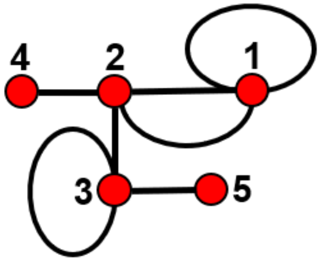	(6x**1**, 2x**2**, 2x**2**^{2}) 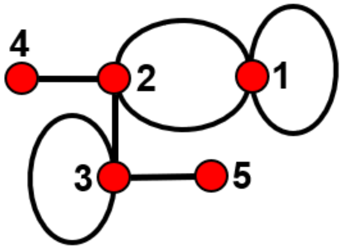	(6x**1**, 4x**2**^{1}) 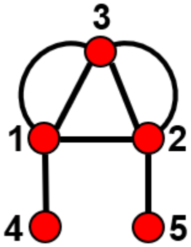

	(6x**1**, 2x$\mathbf{2}^1$, 2x$\mathbf{2}^2$) 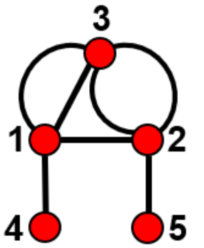	(6x**1**, 4x$\mathbf{2}^2$) 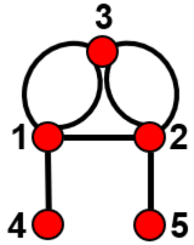	(4x**1**, 3x**2**, 2x$\mathbf{2}^1$) 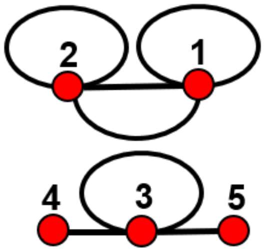	(4x**1**, 3x**2**, 2x$\mathbf{2}^2$) 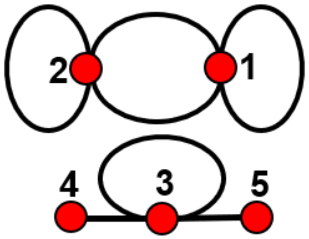
	(4x**1**, 1x**2**, 4x$\mathbf{2}^1$) 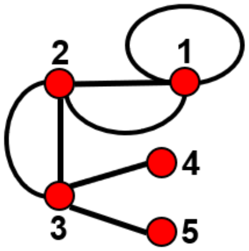	(4x**1**, 1x**2**, 2x$\mathbf{2}^1$, 2x$\mathbf{2}^2$) 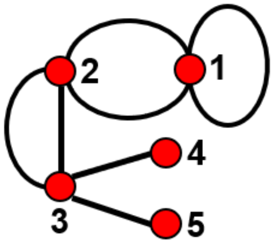	(4x**1**, 1x**2**, 4x$\mathbf{2}^2$) 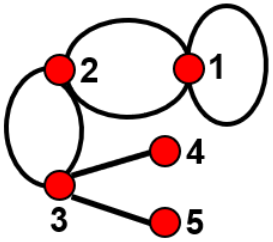	(2x**1**, 2x**2**, 4x$\mathbf{2}^1$) 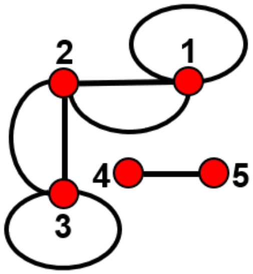
	(2x**1**, 2x**2**, 2x$\mathbf{2}^1$, 2x$\mathbf{2}^2$) 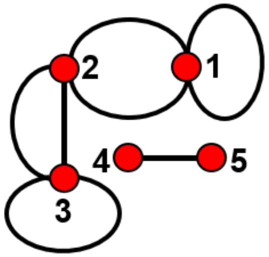	(2x**1**, 2x**2**, 4x$\mathbf{2}^2$) 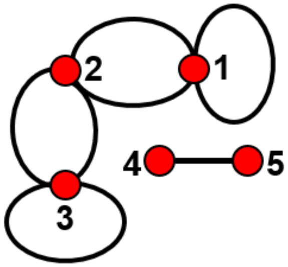	(2x**1**, 6x$\mathbf{2}^1$) 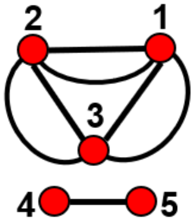	(2x**1**, 4x$\mathbf{2}^1$, 2x$\mathbf{2}^2$) 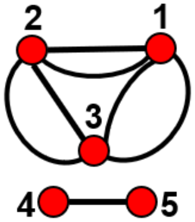

<table>
<tr>
<td></td>
<td>(2x1, 2x2^1, 4x2^2)
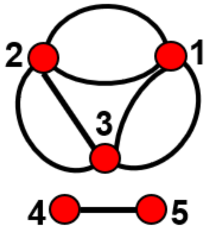
</td>
<td>(2x1, 6x2^2)
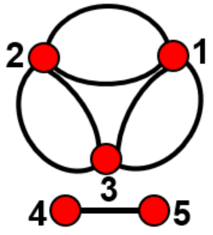
</td>
<td></td>
<td></td>
</tr>
<tr>
<td>$^1V_2{}^4V_4$</td>
<td>(14x1, 2x2)
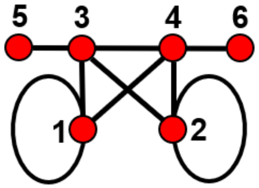
</td>
<td>(14x1, 2x2^1)
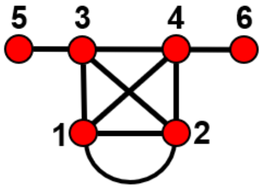
</td>
<td>(14x1, 2x2^2)
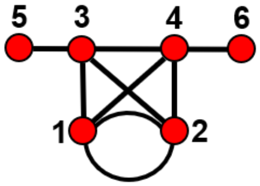
</td>
<td>(12x1, 3x2) a
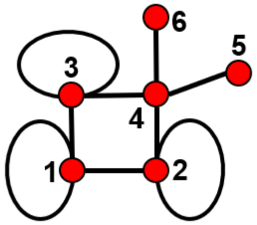
</td>
</tr>
<tr>
<td></td>
<td>(12x1, 3x2) b
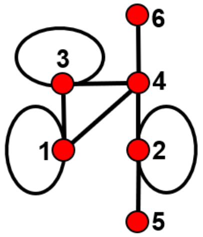
</td>
<td>(12x1, 1x2, 2x2^1) a
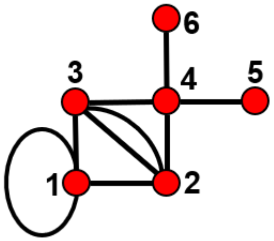
</td>
<td>(12x1, 1x2, 2x2^1) b
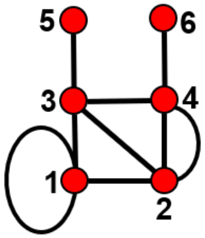
</td>
<td>(12x1, 1x2, 2x2^2) a
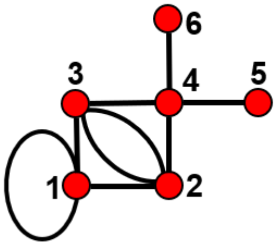
</td>
</tr>
<tr>
<td></td>
<td>(12x1, 1x2, 2x2^2) b
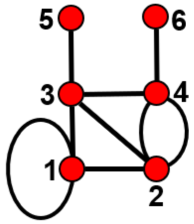
</td>
<td>(10x1, 4x2) a
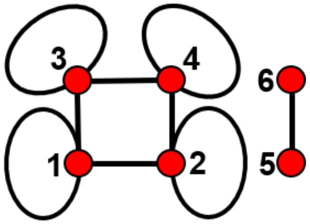
</td>
<td>(10x1, 4x2) b
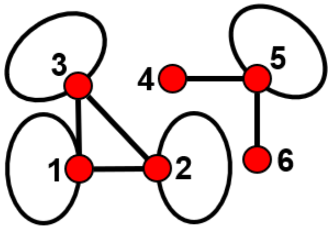
</td>
<td>(10x1, 4x2) c
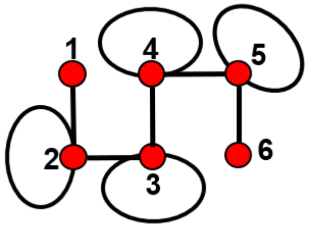
</td>
</tr>
</table>

	(10x**1**, 2x**2**, 2x**2**^{1}) **a** 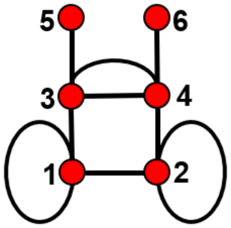	(10x**1**, 2x**2**, 2x**2**^{1}) **b** 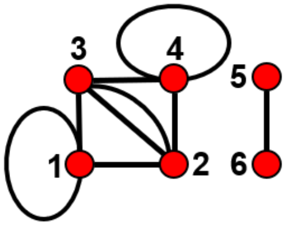	(10x**1**, 2x**2**, 2x**2**^{1}) **c** 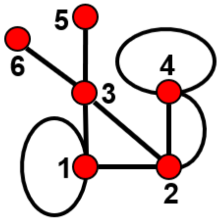	(10x**1**, 2x**2**, 2x**2**^{1}) **d** 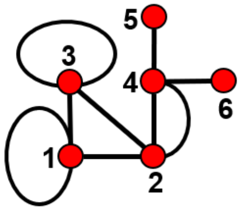
	(10x**1**, 2x**2**, 2x**2**^{1}) **e** 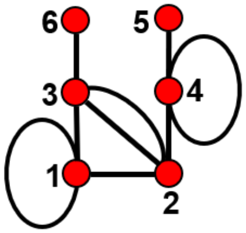	(10x**1**, 2x**2**, 2x**2**^{2}) **a** 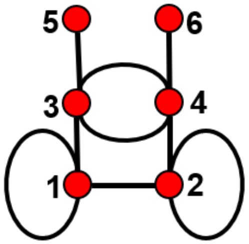	(10x**1**, 2x**2**, 2x**2**^{2}) **b** 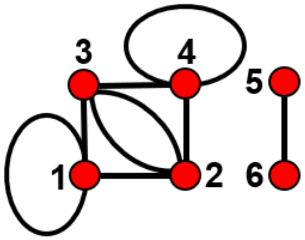	(10x**1**, 2x**2**, 2x**2**^{2}) **c** 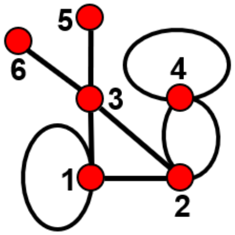
	(10x**1**, 2x**2**, 2x**2**^{2}) **d** 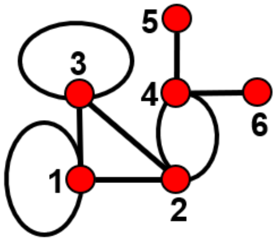	(10x**1**, 2x**2**, 2x**2**^{2}) **e** 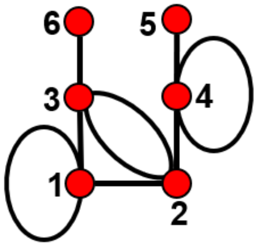	(10x**1**, 4x**2**^{1}) **a** 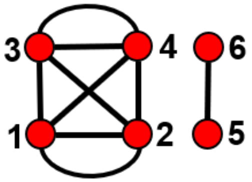	(10x**1**, 4x**2**^{1}) **b** 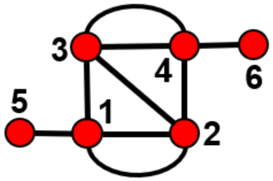
	(10x**1**, 4x**2**^{1}) **c** 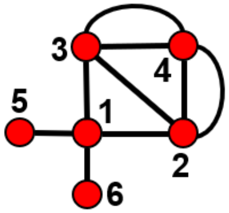	(10x**1**, 2x**2**^{1}, 2x**2**^{2}) **a** 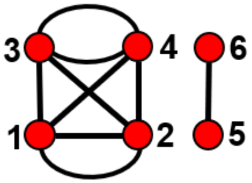	(10x**1**, 2x**2**^{1}, 2x**2**^{2}) **b** 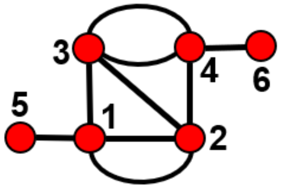	(10x**1**, 2x**2**^{1}, 2x**2**^{2}) **c** 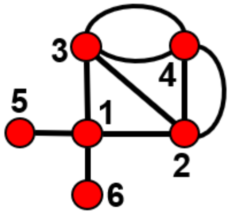

	(10x**1**, 4x**2**^{2}) **a** 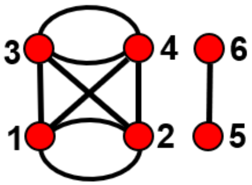	(10x**1**, 4x**2**^{2}) **b** 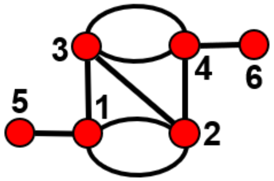	(10x**1**, 4x**2**^{2}) **c** 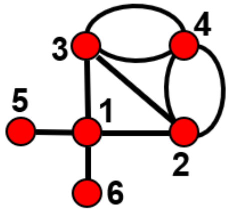	(8x**1**, 3x**2**, 2x**2**^{1}) **a** 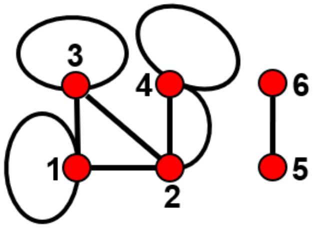
	(8x**1**, 3x**2**, 2x**2**^{1}) **b** 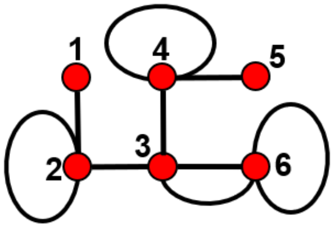	(8x**1**, 3x**2**, 2x**2**^{1}) **c** 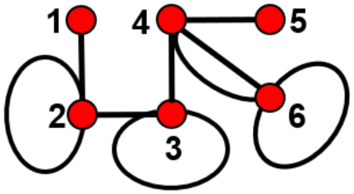	(8x**1**, 3x**2**, 2x**2**^{2}) **a** 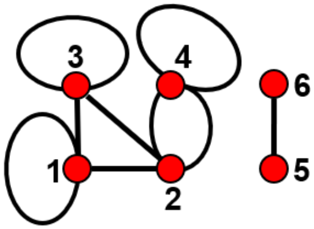	(8x**1**, 3x**2**, 2x**2**^{2}) **b** 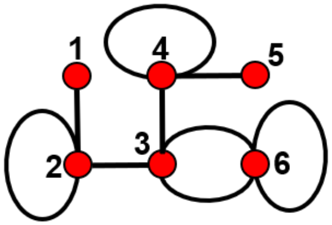
	(8x**1**, 3x**2**, 2x**2**^{2}) **c** 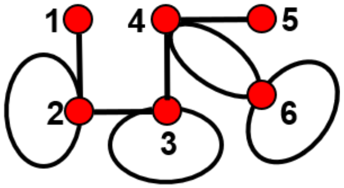	(8x**1**, 1x**2**, 4x**2**^{1}) **a** 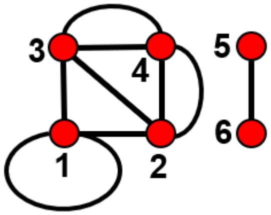	(8x**1**, 1x**2**, 4x**2**^{1}) **b** 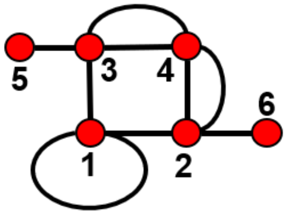	(8x**1**, 1x**2**, 4x**2**^{1}) **c** 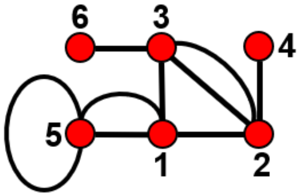
	(8x**1**, 1x**2**, 4x**2**^{1}) **d** 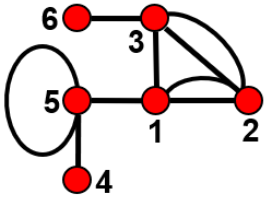	(8x**1**, 1x**2**, 2x**2**^{1}, 2x**2**^{2}) **a** 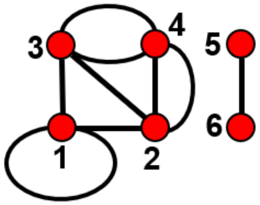	(8x**1**, 1x**2**, 2x**2**^{1}, 2x**2**^{2}) **b** 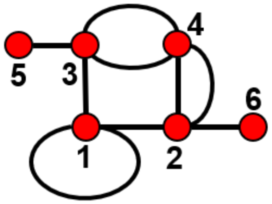	(8x**1**, 1x**2**, 2x**2**^{1}, 2x**2**^{2}) **c** 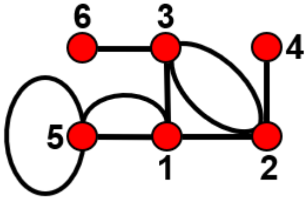

	(8x**1**, 1x**2**, 2x$\mathbf{2}^{1}$, 2x$\mathbf{2}^{2}$) **d**	(8x**1**, 1x**2**, 2x$\mathbf{2}^{1}$, 2x$\mathbf{2}^{2}$) **e**	(8x**1**, 1x**2**, 2x$\mathbf{2}^{1}$, 2x$\mathbf{2}^{2}$) **f**	(8x**1**, 1x**2**, 4x$\mathbf{2}^{2}$) **a**
	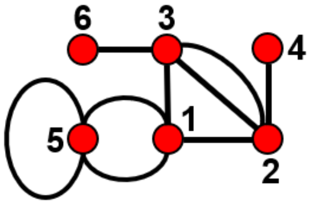	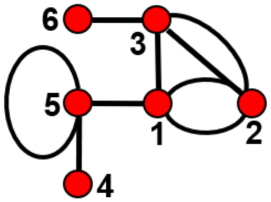	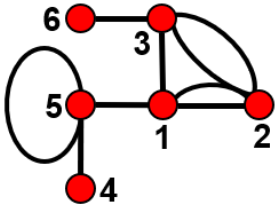	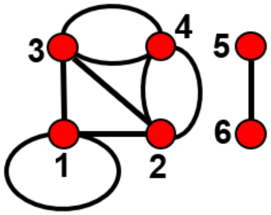
	(8x**1**, 1x**2**, 4x$\mathbf{2}^{2}$) **b**	(8x**1**, 1x**2**, 4x$\mathbf{2}^{2}$) **c**	(8x**1**, 1x**2**, 4x$\mathbf{2}^{2}$) **d**	(6x**1**, 4x**2**, 2x$\mathbf{2}^{1}$)
	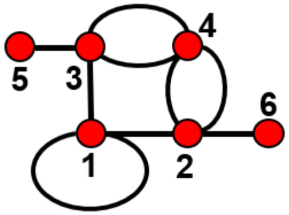	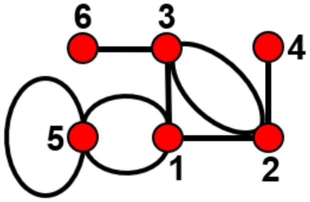	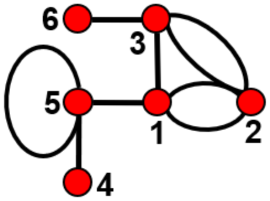	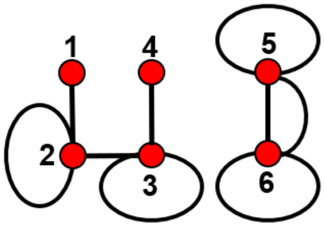
	(6x**1**, 4x**2**, 2x$\mathbf{2}^{2}$)	(6x**1**, 2x**2**, 4x$\mathbf{2}^{1}$) **a**	(6x**1**, 2x**2**, 4x$\mathbf{2}^{1}$) **b**	(6x**1**, 2x**2**, 2x$\mathbf{2}^{1}$, 2x$\mathbf{2}^{2}$) **a**
	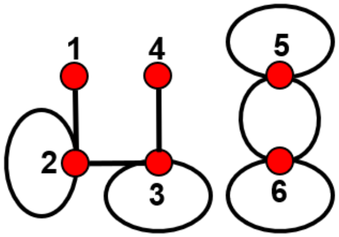	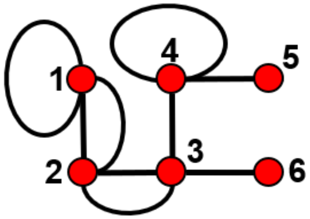	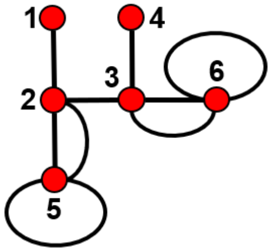	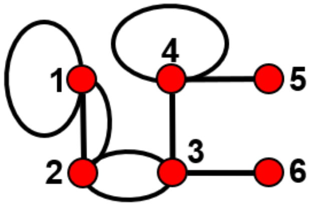
	(6x**1**, 2x**2**, 2x$\mathbf{2}^{1}$, 2x$\mathbf{2}^{2}$) **b**	(6x**1**, 2x**2**, 2x$\mathbf{2}^{1}$, 2x$\mathbf{2}^{2}$) **c**	(6x**1**, 2x**2**, 4x$\mathbf{2}^{2}$) **a**	(6x**1**, 2x**2**, 4x$\mathbf{2}^{2}$) **b**
	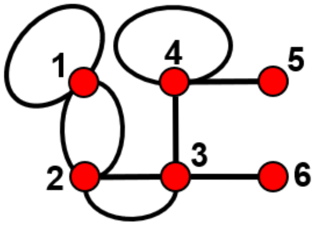	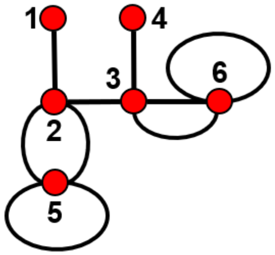	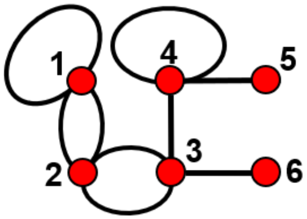	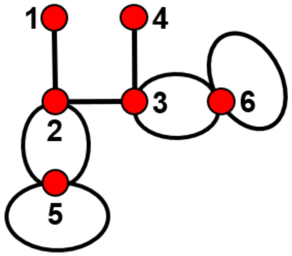

	(6x**1**, 6x**2**^{1}) 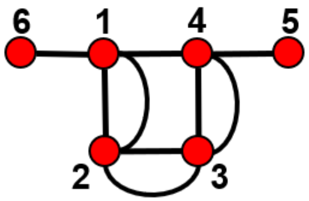	(6x**1**, 4x**2**^{1}, 2x**2**^{2}) **a** 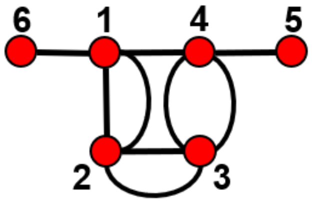	(6x**1**, 4x**2**^{1}, 2x**2**^{2}) **b** 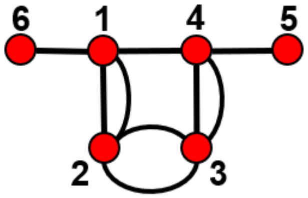	(6x**1**, 2x**2**^{1}, 4x**2**^{2}) **a** 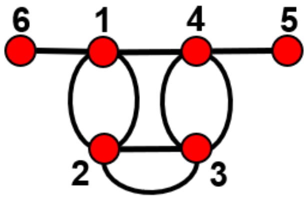
	(6x**1**, 2x**2**^{1}, 4x**2**^{2}) **b** 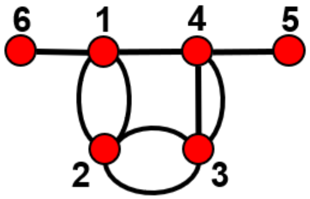	(6x**1**, 6x**2**^{2}) 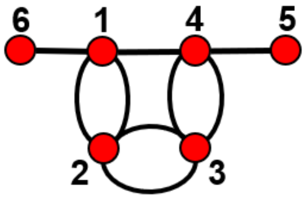	(4x**1**, 3x**2**, 4x**2**^{1}) **a** 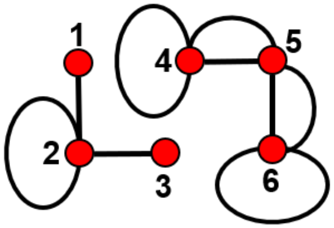	(4x**1**, 3x**2**, 4x**2**^{1}) **b** 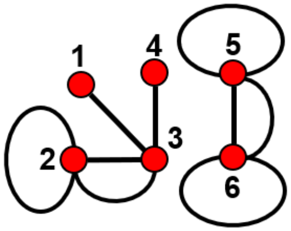
	(4x**1**, 3x**2**, 2x**2**^{1}, 2x**2**^{2}) **a** 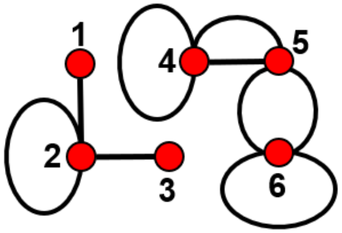	(4x**1**, 3x**2**, 2x**2**^{1}, 2x**2**^{2}) **b** 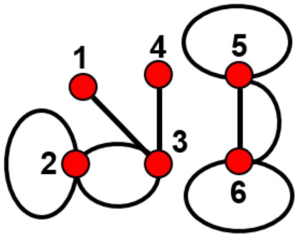	(4x**1**, 3x**2**, 2x**2**^{1}, 2x**2**^{2}) **c** 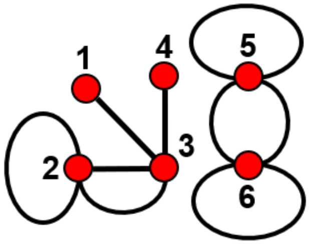	(4x**1**, 3x**2**, 4x**2**^{2}) **a** 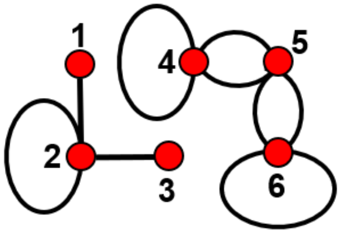
	(4x**1**, 3x**2**, 4x**2**^{2}) **b** 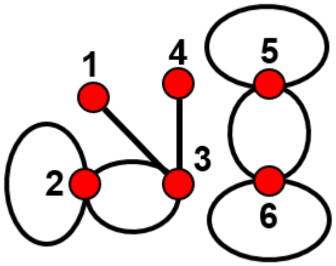	(4x**1**, 1x**2**, 6x**2**^{1}) **a** 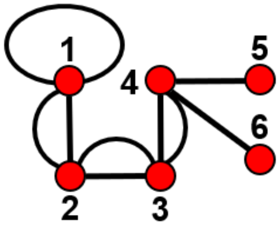	(4x**1**, 1x**2**, 6x**2**^{1}) **b** 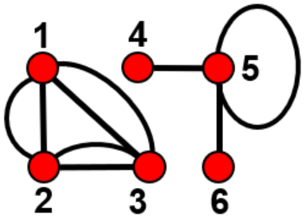	(4x**1**, 1x**2**, 4x**2**^{1}, 2x**2**^{2}) **a** 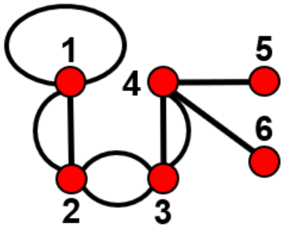

	(4x**1**, 1x**2**, 4x$\mathbf{2}^1$, 2x$\mathbf{2}^2$) **b** 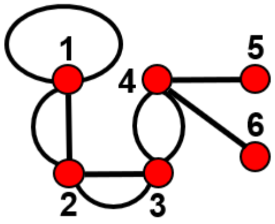	(4x**1**, 1x**2**, 4x$\mathbf{2}^1$, 2x$\mathbf{2}^2$) **c** 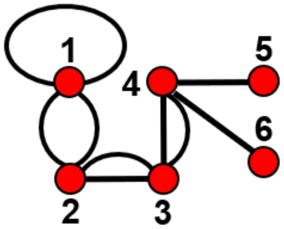	(4x**1**, 1x**2**, 4x$\mathbf{2}^1$, 2x$\mathbf{2}^2$) **d** 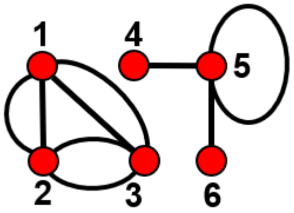	(4x**1**, 1x**2**, 2x$\mathbf{2}^1$, 4x$\mathbf{2}^2$) **a** 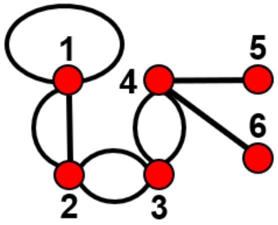
	(4x**1**, 1x**2**, 2x$\mathbf{2}^1$, 4x$\mathbf{2}^2$) **b** 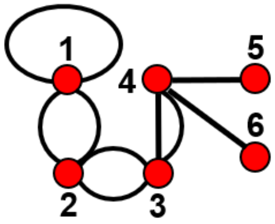	(4x**1**, 1x**2**, 2x$\mathbf{2}^1$, 4x$\mathbf{2}^2$) **c** 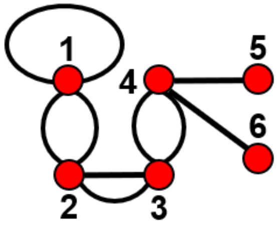	(4x**1**, 1x**2**, 2x$\mathbf{2}^1$, 4x$\mathbf{2}^2$) **d** 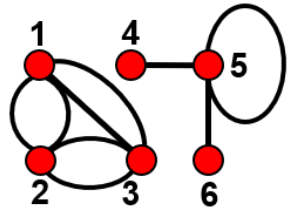	(4x**1**, 1x**2**, 6x$\mathbf{2}^2$) **a** 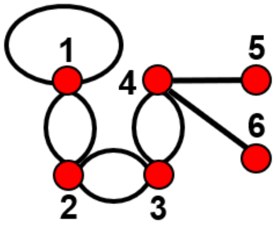
	(4x**1**, 1x**2**, 6x$\mathbf{2}^2$) **b** 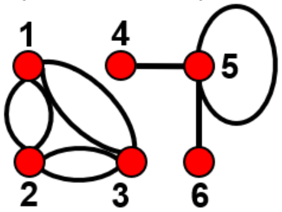	(2x**1**, 4x**2**, 4x$\mathbf{2}^1$) 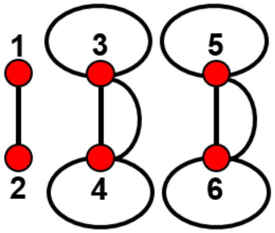	(2x**1**, 4x**2**, 2x$\mathbf{2}^1$, 2x$\mathbf{2}^2$) 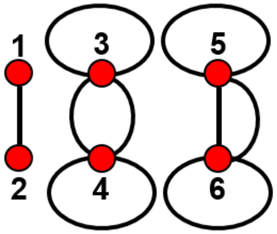	(2x**1**, 4x**2**, 4x$\mathbf{2}^2$) 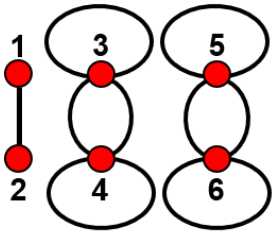

	(2x**1**, 2x**2**, 6x$\mathbf{2}^1$)<br>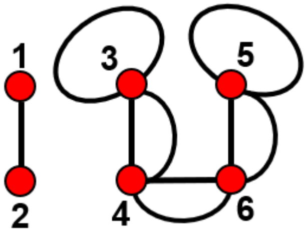	(2x**1**, 2x**2**, 4x$\mathbf{2}^1$, 2x$\mathbf{2}^2$) **a**<br>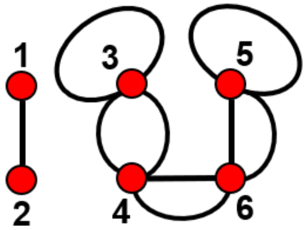	(2x**1**, 2x**2**, 4x$\mathbf{2}^1$, 2x$\mathbf{2}^2$) **b**<br>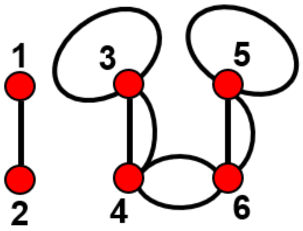	(2x**1**, 2x**2**, 2x$\mathbf{2}^1$, 4x$\mathbf{2}^2$) **a**<br>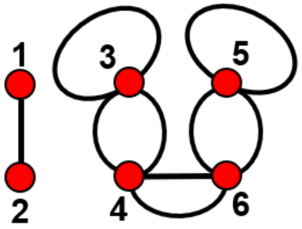
	(2x**1**, 2x**2**, 2x$\mathbf{2}^1$, 4x$\mathbf{2}^2$) **b**<br>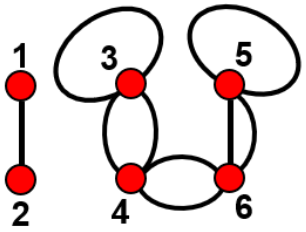	(2x**1**, 2x**2**, 6x$\mathbf{2}^2$)<br>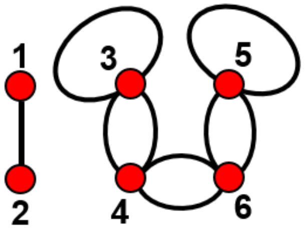	(2x**1**, 8x$\mathbf{2}^1$)<br>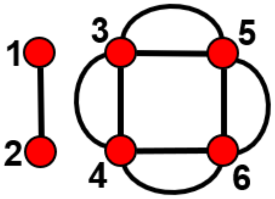	(2x**1**, 6x$\mathbf{2}^1$, 2x$\mathbf{2}^2$)<br>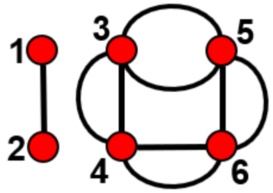
	(2x**1**, 4x$\mathbf{2}^1$, 4x$\mathbf{2}^2$) **a**<br>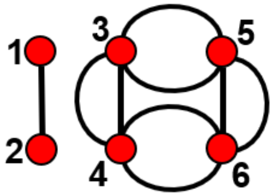	(2x**1**, 4x$\mathbf{2}^1$, 4x$\mathbf{2}^2$) **b**<br>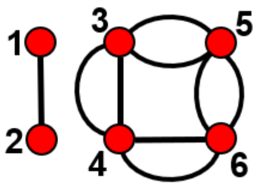	(2x**1**, 2x$\mathbf{2}^1$, 6x$\mathbf{2}^2$)<br>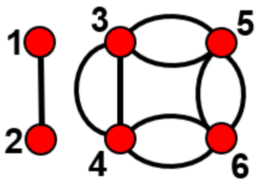	(2x**1**, 8x$\mathbf{2}^2$)<br>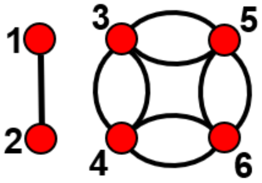
$^1V_2{}^4V_5$	NG			
$^1V_2{}^4V_6$	NG			

$^{1}V_{4}{}^{4}V_{1}$	(8x**1**)<br>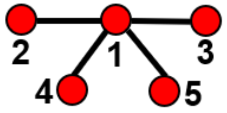	(6x**1**, 1x**2**)<br>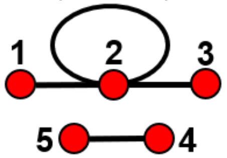		
$^{1}V_{4}{}^{4}V_{2}$	(10x**1**, 1x**2**)<br>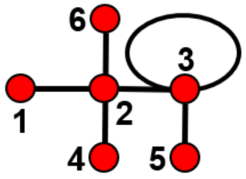	(8x**1**, 2x**2**) **a**<br>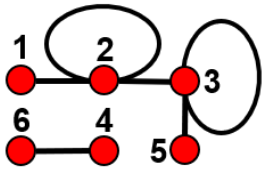	(8x**1**, 2x**2**) **b**<br>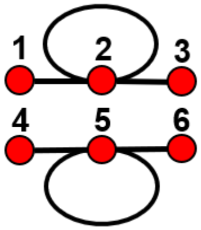	(8x**1**, $2\mathrm{x}\mathbf{2}^{1}$)<br>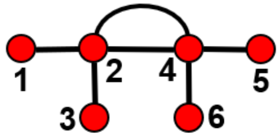
	(8x**1**, $2\mathrm{x}\mathbf{2}^{2}$)<br>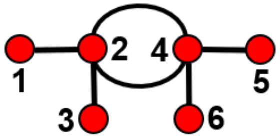	(6x**1**, 1x**2**, $2\mathrm{x}\mathbf{2}^{1}$)<br>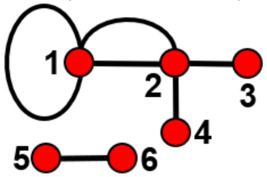	(6x**1**, 1x**2**, $2\mathrm{x}\mathbf{2}^{2}$)<br>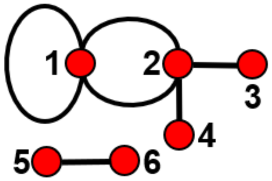	(4x**1**, 2x**2**, $2\mathrm{x}\mathbf{2}^{1}$)<br>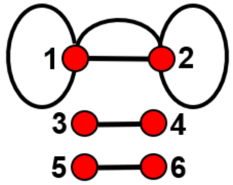
	(4x**1**, 2x**2**, $2\mathrm{x}\mathbf{2}^{2}$)<br>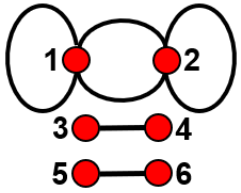			
$^{1}V_{4}{}^{4}V_{3}$	NG			
$^{1}V_{4}{}^{4}V_{4}$	NG			

$^{1}V_{6}{}^{4}V_{1}$	(10x**1**) 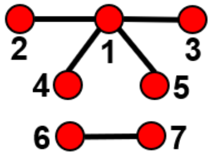	(8x**1**, 1x**2**) 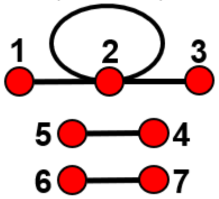		
$^{1}V_{6}{}^{4}V_{2}$	NG			
$\boldsymbol{^{2}V_{r}{}^{3}V_{r}}$				
$^{2}V_{1}{}^{3}V_{2}$	(4x**1**, 2x**2**) 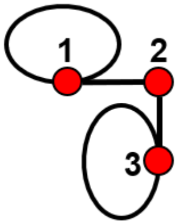	(4x**1**, 2x**2**^{1}) 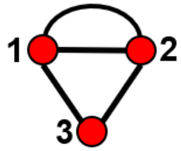	(4x**1**, 2x**2**^{2}) 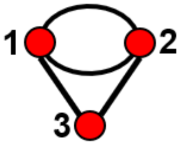	(2x**1**, 3x**2**) 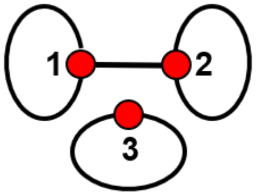
	(2x**1**, 1x**2**, 2x**2**^{1}) 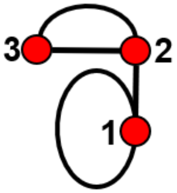	(2x**1**, 1x**2**, 2x**2**^{2}) 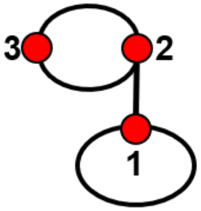		
$^{2}V_{1}{}^{3}V_{4}$	(14x**1**) 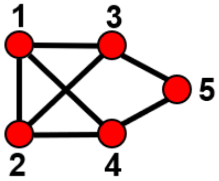	(12x**1**, 1x**2**) **a** 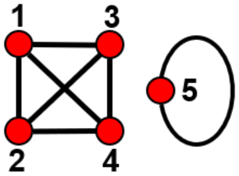	(12x**1**, 1x**2**) **b** 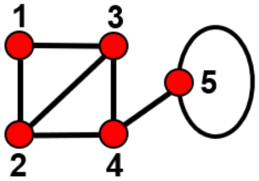	(10x**1**, 2x**2**) 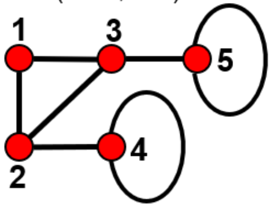

	(10x**1**, 2x**2**^{1}) 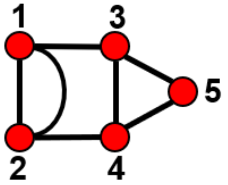	(10x**1**, 2x**2**^{2}) 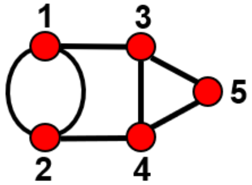	(8x**1**, 3x**2**) 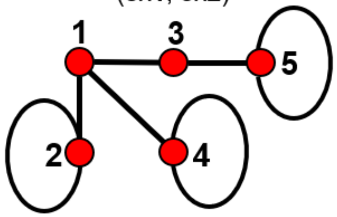	(8x**1**, 1x**2**, 2x**2**^{1}) **a** 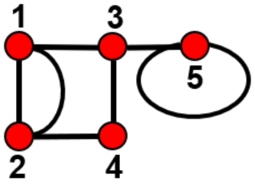
	(8x**1**, 1x**2**, 2x**2**^{1}) **b** 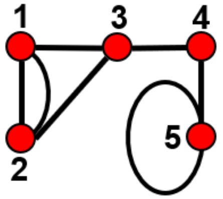	(8x**1**, 1x**2**, 2x**2**^{2}) **a** 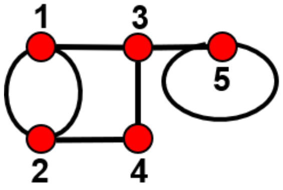	(8x**1**, 1x**2**, 2x**2**^{2}) **b** 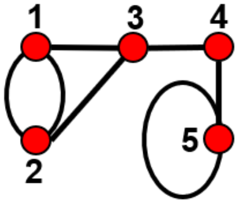	(6x**1**, 4x**2**) **a** 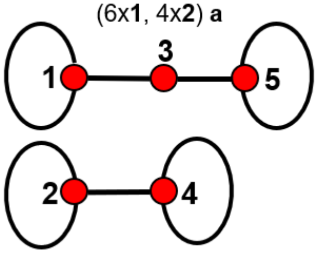
	(6x**1**, 4x**2**) **b** 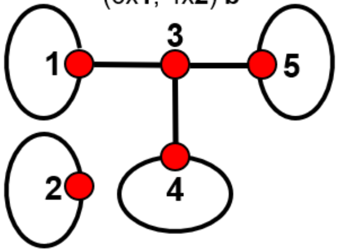	(6x**1**, 2x**2**, 2x**2**^{1}) **a** 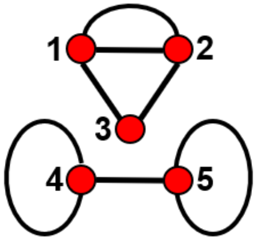	(6x**1**, 2x**2**, 2x**2**^{1}) **b** 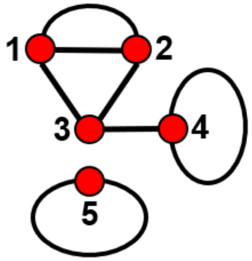	(6x**1**, 2x**2**, 2x**2**^{1}) **c** 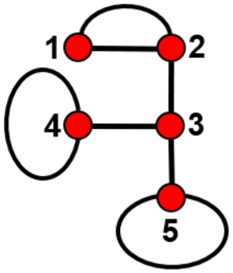
	(6x**1**, 2x**2**, 2x**2**^{1}) **d** 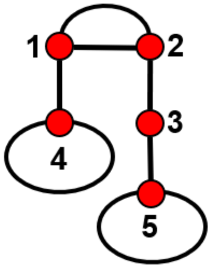	(6x**1**, 2x**2**, 2x**2**^{2}) **a** 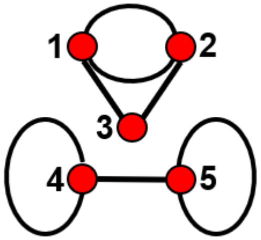	(6x**1**, 2x**2**, 2x**2**^{2}) **b** 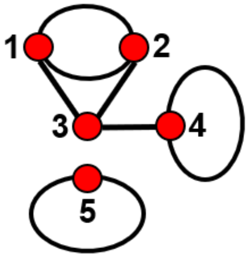	(6x**1**, 2x**2**, 2x**2**^{2}) **c** 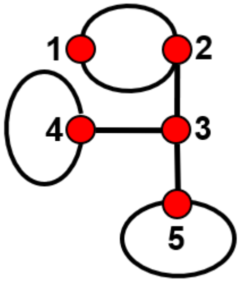

	(6x**1**, 2x**2**, 2x**2**^{2}) **d** 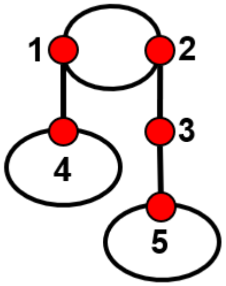	(6x**1**, 4x**2**^{1}) **a** 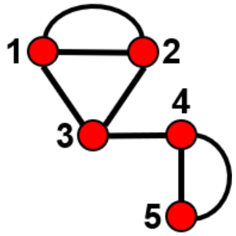	(6x**1**, 4x**2**^{1}) **b** 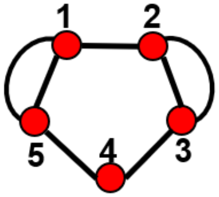	(6x**1**, 2x**2**^{1}, 2x**2**^{2}) **a** 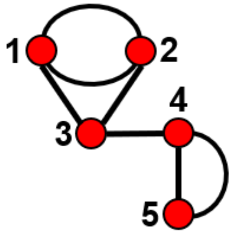
	(6x**1**, 2x**2**^{1}, 2x**2**^{2}) **b** 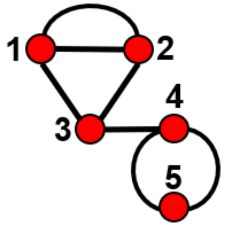	(6x**1**, 2x**2**^{1}, 2x**2**^{2}) **c** 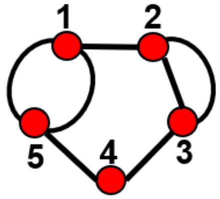	(6x**1**, 4x**2**^{2}) **a** 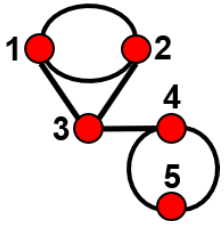	(6x**1**, 4x**2**^{2}) **b** 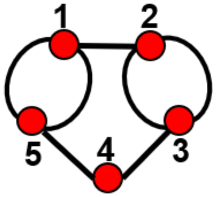
	(4x**1**, 5x**2**) 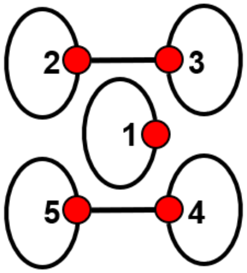	(4x**1**, 3x**2**, 2x**2**^{1}) **a** 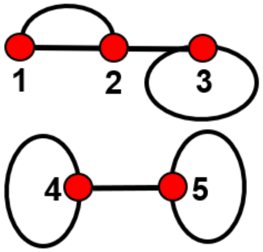	(4x**1**, 3x**2**, 2x**2**^{1}) **b** 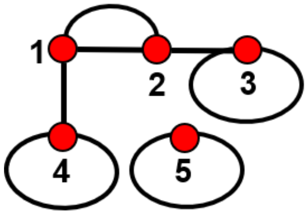	(4x**1**, 3x**2**, 2x**2**^{2}) **a** 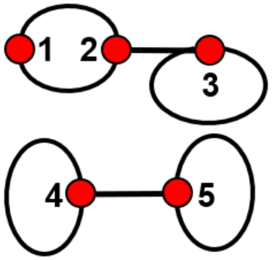
	(4x**1**, 3x**2**, 2x**2**^{2}) **b** 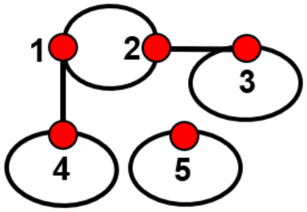	(4x**1**, 1x**2**, 4x**2**^{1}) **a** 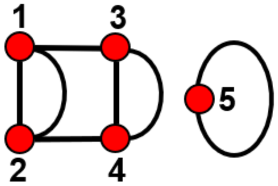	(4x**1**, 1x**2**, 4x**2**^{1}) **b** 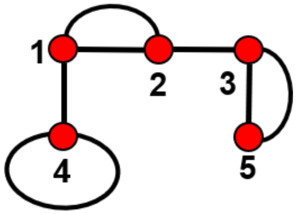	(4x**1**, 1x**2**, 2x**2**^{1}, 2x**2**^{2}) **a** 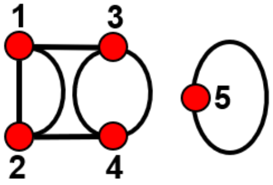

	(4x**1**, 1x**2**, 2x**2**^{1}, 2x**2**^{2}) **b** 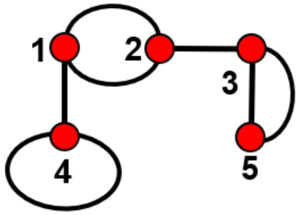	(4x**1**, 1x**2**, 2x**2**^{1}, 2x**2**^{2}) **c** 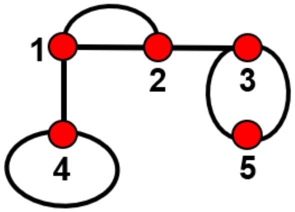	(4x**1**, 1x**2**, 4x**2**^{2}) **a** 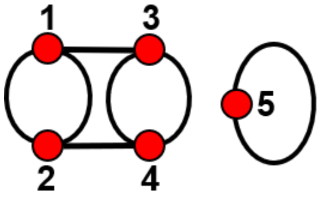	(4x**1**, 1x**2**, 4x**2**^{2}) **b** 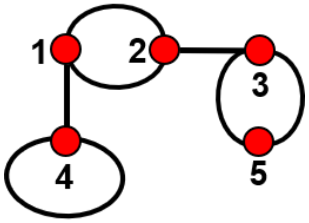
$^2V_1{}^3V_6$	NG			
$^2V_2{}^3V_2$	(10x**1**) 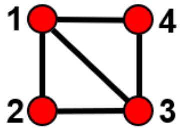	(8x**1**, 1x**2**) 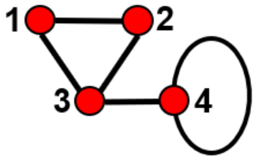	(6x**1**, 2x**2**) 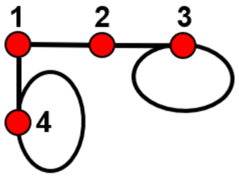	(6x**1**, 2x**2**^{1}) 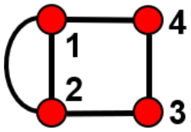
	(6x**1**, 2x**2**^{2}) 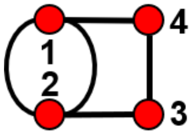	(4x**1**, 3x**2**) 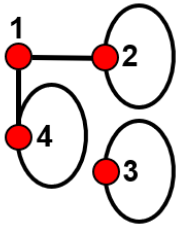	(4x**1**, 1x**2**, 2x**2**^{1}) **a** 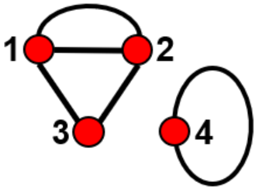	(4x**1**, 1x**2**, 2x**2**^{1}) **b** 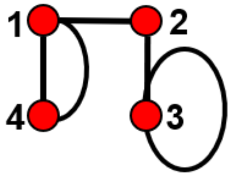
	(4x**1**, 1x**2**, 2x**2**^{2}) **a** 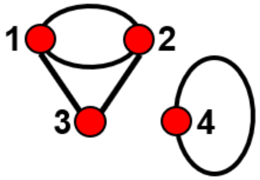	(4x**1**, 1x**2**, 2x**2**^{2}) **b** 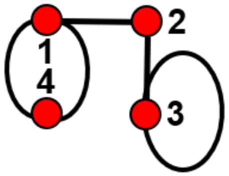	(2x**1**, 4x**2**) 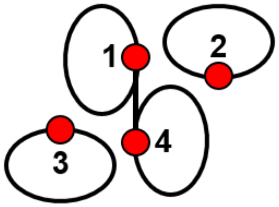	(2x**1**, 2x**2**, 2x**2**^{1}) **a** 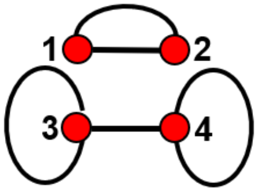

	(2x**1**, 2x**2**, 2x$\mathbf{2}^1$) **b** 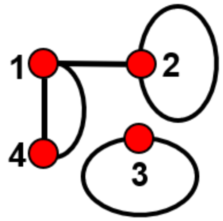	(2x**1**, 2x**2**, 2x$\mathbf{2}^2$) **a** 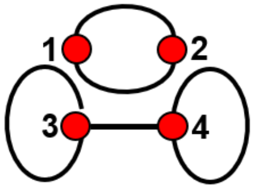	(2x**1**, 2x**2**, 2x$\mathbf{2}^2$) **b** 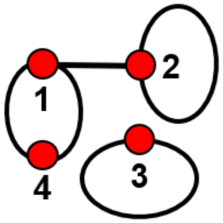	(2x**1**, 4x$\mathbf{2}^1$) 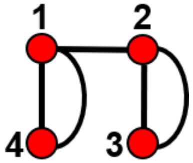
	(2x**1**, 2x$\mathbf{2}^1$, 2x$\mathbf{2}^2$) 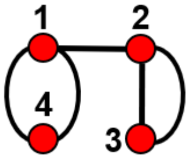	(2x**1**, 4x$\mathbf{2}^2$) 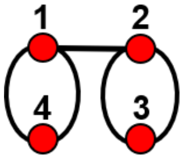		
$^2V_2{}^3V_4$	(16x**1**) **a** 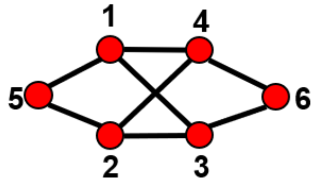	(16x**1**) **b** 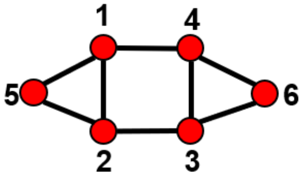	(16x**1**) **c** 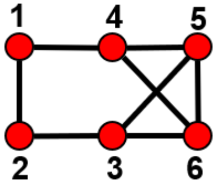	(16x**1**) **d** 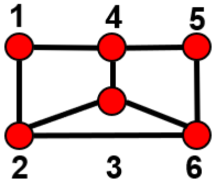
	(14x**1**, 1x**2**) **a** 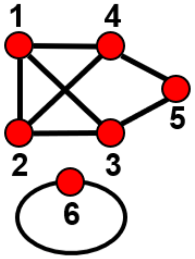	(14x**1**, 1x**2**) **b** 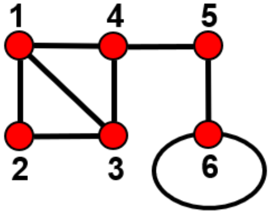	(14x**1**, 1x**2**) **c** 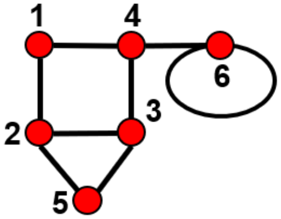	(14x**1**, 1x**2**) **d** 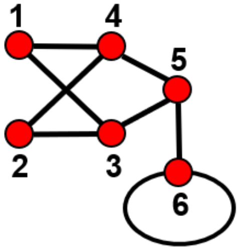

	(14x**1**, 1x**2**) **e**<br>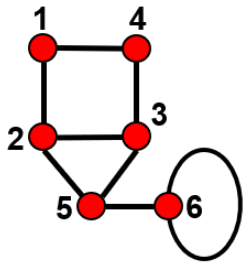	(12x**1**, 2x**2**) **a**<br>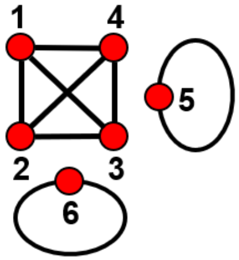	(12x**1**, 2x**2**) **b**<br>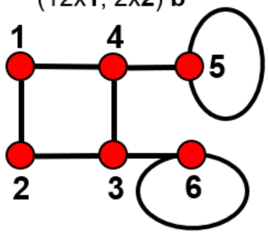	(12x**1**, 2x**2**) **c**<br>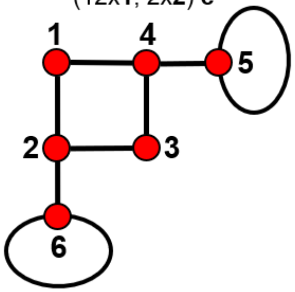
	(12x**1**, 2x**2**) **d**<br>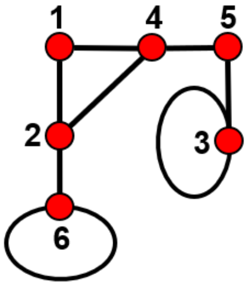	(12x**1**, 2x**2**) **e**<br>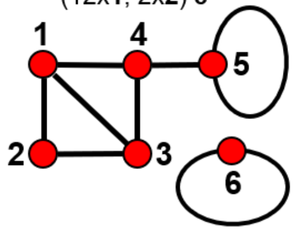	(12x**1**, 2x**2**) **f**<br>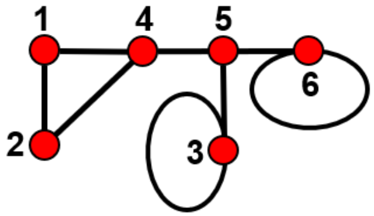	(12x**1**, 2x**2**) **g**<br>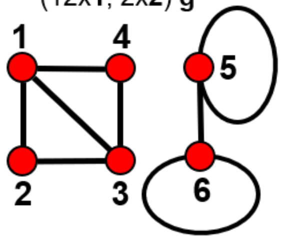
	(12x**1**, 2x**2**^{1}) **a**<br>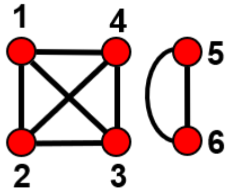	(12x**1**, 2x**2**^{1}) **b**<br>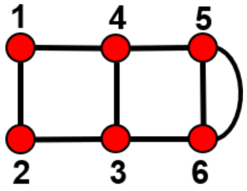	(12x**1**, 2x**2**^{1}) **c**<br>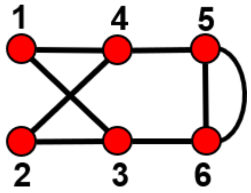	(12x**1**, 2x**2**^{1}) **d**<br>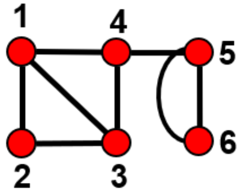
	(12x**1**, 2x**2**^{1}) **e**<br>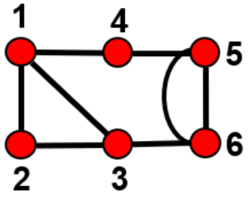	(12x**1**, 2x**2**^{1}) **f**<br>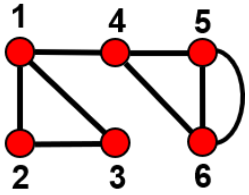	(12x**1**, 2x**2**^{2}) **a**<br>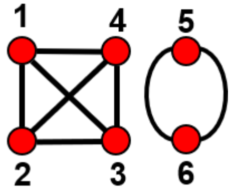	(12x**1**, 2x**2**^{2}) **b**<br>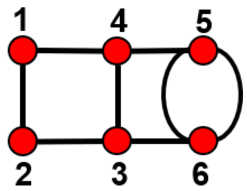

	(12x**1**, 2x$\mathbf{2}^2$) **c** 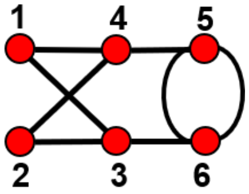	(12x**1**, 2x$\mathbf{2}^2$) **d** 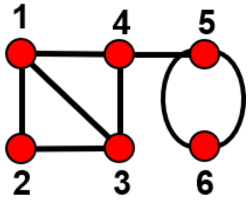	(12x**1**, 2x$\mathbf{2}^2$) **e** 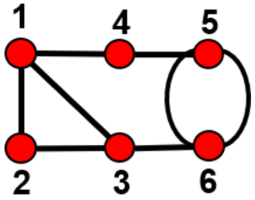	(12x**1**, 2x$\mathbf{2}^2$) **f** 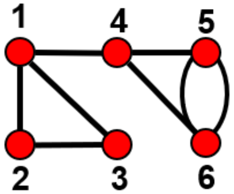
	(10x**1**, 3x**2**) **a** 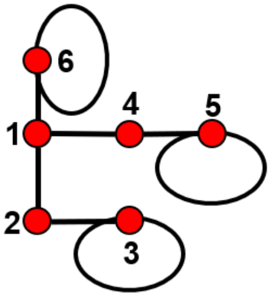	(10x**1**, 3x**2**) **b** 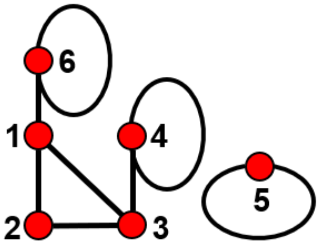	(10x**1**, 3x**2**) **c** 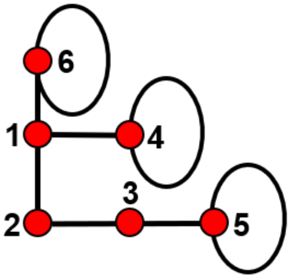	(10x**1**, 3x**2**) **d** 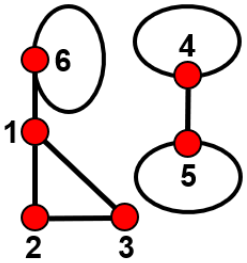
	(10x**1**, 1x**2**, 2x$\mathbf{2}^1$) **a** 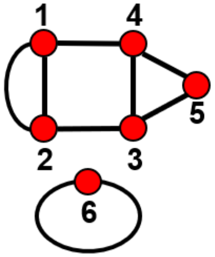	(10x**1**, 1x**2**, 2x$\mathbf{2}^1$) **b** 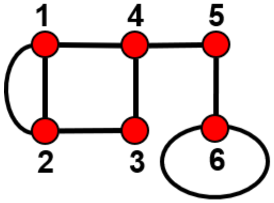	(10x**1**, 1x**2**, 2x$\mathbf{2}^1$) **c** 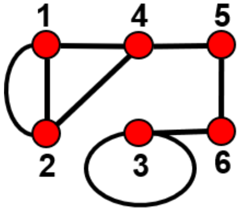	(10x**1**, 1x**2**, 2x$\mathbf{2}^1$) **d** 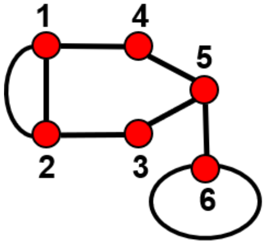

	(10x**1**, 1x**2**, 2x**2**^{1}) **e**<br>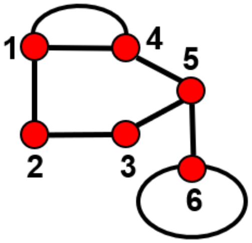	(10x**1**, 1x**2**, 2x**2**^{1}) **f**<br>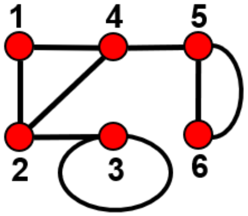	(10x**1**, 1x**2**, 2x**2**^{1}) **g**<br>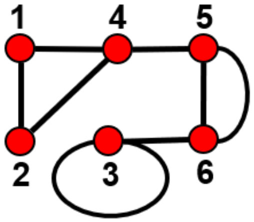	(10x**1**, 1x**2**, 2x**2**^{2}) **a**<br>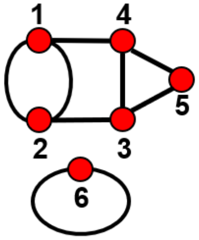
	(10x**1**, 1x**2**, 2x**2**^{2}) **b**<br>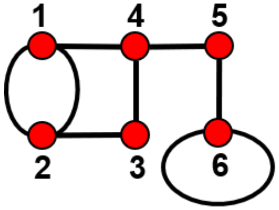	(10x**1**, 1x**2**, 2x**2**^{2}) **c**<br>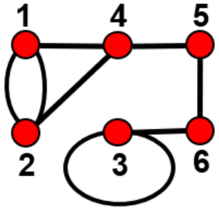	(10x**1**, 1x**2**, 2x**2**^{2}) **d**<br>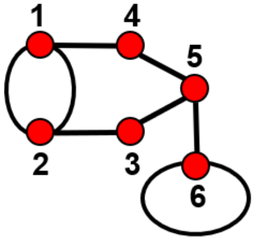	(10x**1**, 1x**2**, 2x**2**^{2}) **e**<br>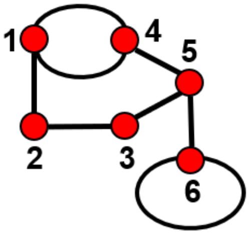
	(10x**1**, 1x**2**, 2x**2**^{2}) **f**<br>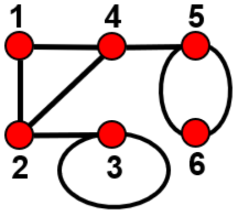	(10x**1**, 1x**2**, 2x**2**^{2}) **g**<br>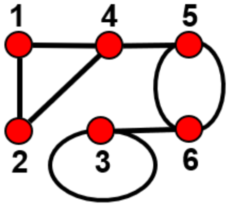	(8x**1**, 4x**2**) **a**<br>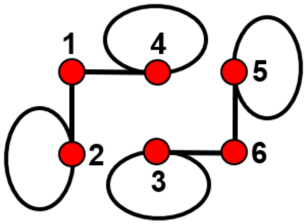	(8x**1**, 4x**2**) **b**<br>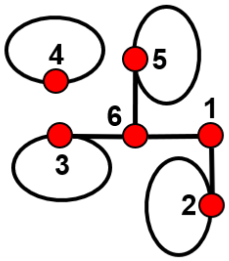

	(8x**1**, 4x**2**) **c** 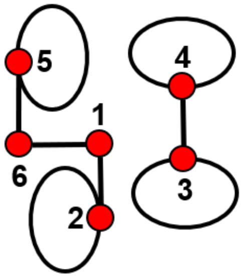	(8x**1**, 2x**2**, 2x**2**^{1}) **a** 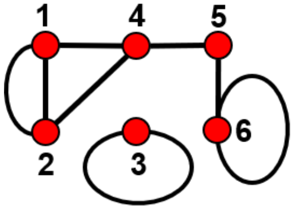	(8x**1**, 2x**2**, 2x**2**^{1}) **b** 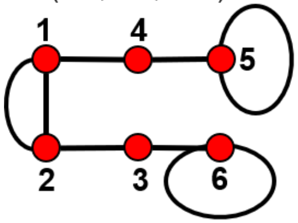	(8x**1**, 2x**2**, 2x**2**^{1}) **c** 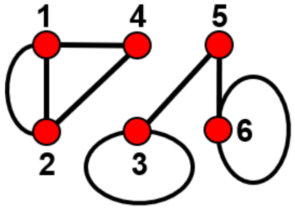
	(8x**1**, 2x**2**, 2x**2**^{1}) **d** 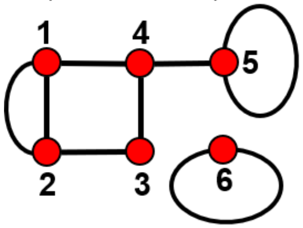	(8x**1**, 2x**2**, 2x**2**^{1}) **e** 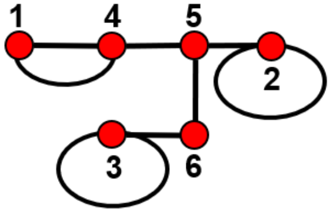	(8x**1**, 2x**2**, 2x**2**^{1}) **f** 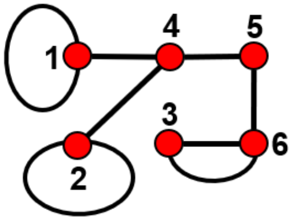	(8x**1**, 2x**2**, 2x**2**^{1}) **g** 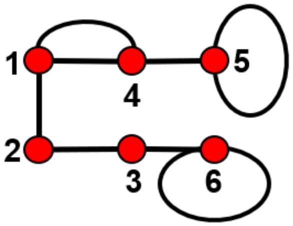
	(8x**1**, 2x**2**, 2x**2**^{1}) **h** 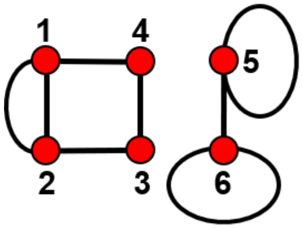	(8x**1**, 2x**2**, 2x**2**^{2}) **a** 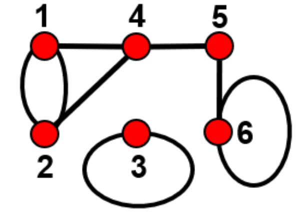	(8x**1**, 2x**2**, 2x**2**^{2}) **b** 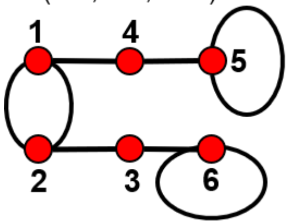	(8x**1**, 2x**2**, 2x**2**^{2}) **c** 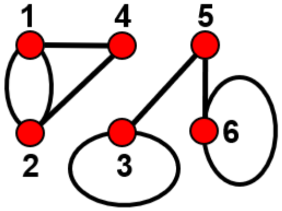
	(8x**1**, 2x**2**, 2x**2**^{2}) **d** 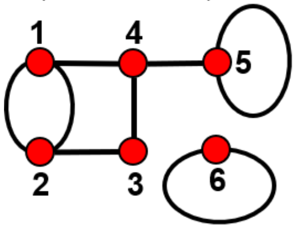	(8x**1**, 2x**2**, 2x**2**^{2}) **e** 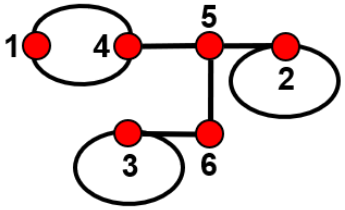	(8x**1**, 2x**2**, 2x**2**^{2}) **f** 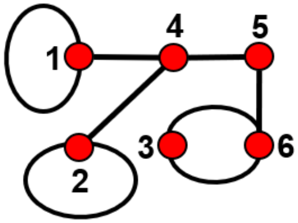	(8x**1**, 2x**2**, 2x**2**^{2}) **g** 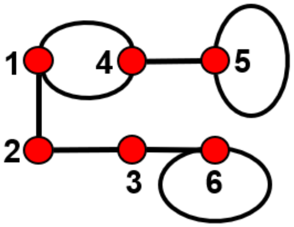

	(8x**1**, 2x**2**, 2x$\mathbf{2}^2$) **h**<br>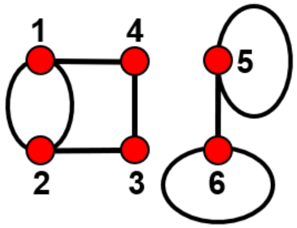	(8x**1**, 4x$\mathbf{2}^1$) **a**<br>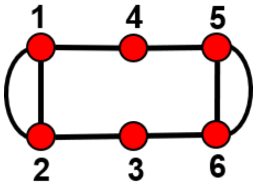	(8x**1**, 4x$\mathbf{2}^1$) **b**<br>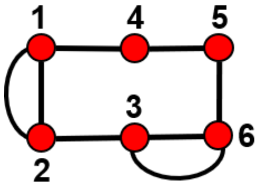	(8x**1**, 4x$\mathbf{2}^1$) **c**<br>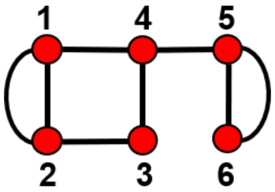
	(8x**1**, 4x$\mathbf{2}^1$) **d**<br>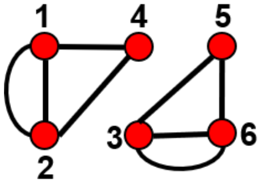	(8x**1**, 4x$\mathbf{2}^1$) **e**<br>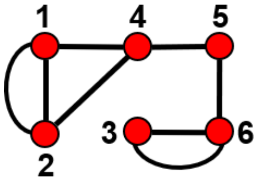	(8x**1**, 2x$\mathbf{2}^1$, 2x$\mathbf{2}^2$) **a**<br>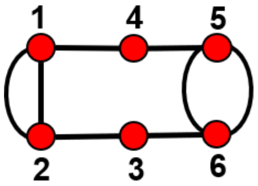	(8x**1**, 2x$\mathbf{2}^1$, 2x$\mathbf{2}^2$) **b**<br>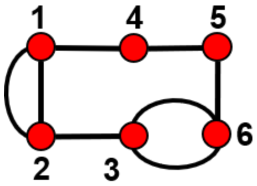
	(8x**1**, 2x$\mathbf{2}^1$, 2x$\mathbf{2}^2$) **c**<br>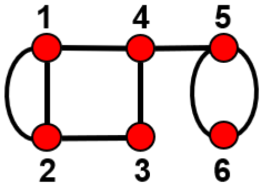	(8x**1**, 2x$\mathbf{2}^1$, 2x$\mathbf{2}^2$) **d**<br>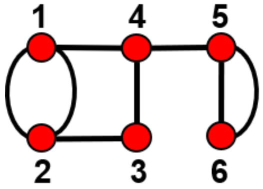	(8x**1**, 2x$\mathbf{2}^1$, 2x$\mathbf{2}^2$) **e**<br>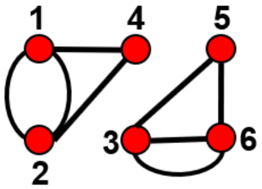	(8x**1**, 2x$\mathbf{2}^1$, 2x$\mathbf{2}^2$) **f**<br>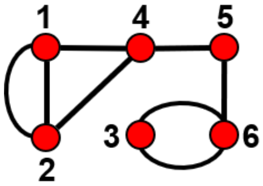
	(8x**1**, 2x$\mathbf{2}^1$, 2x$\mathbf{2}^2$) **g**<br>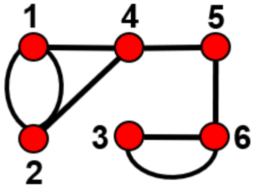	(8x**1**, 4x$\mathbf{2}^2$) **a**<br>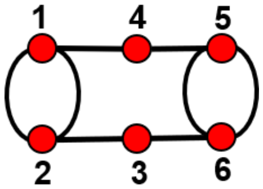	(8x**1**, 4x$\mathbf{2}^2$) **b**<br>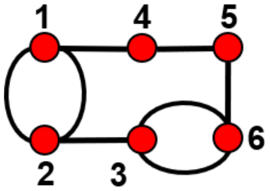	(8x**1**, 4x$\mathbf{2}^2$) **c**<br>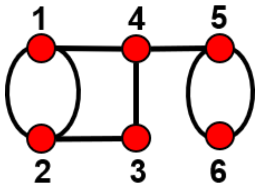

	(8x**1**, 4x**2**^{1}) **d** 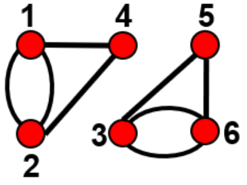	(8x**1**, 4x**2**^{2}) **e** 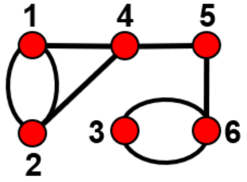	(6x**1**, 5x**2**) **a** 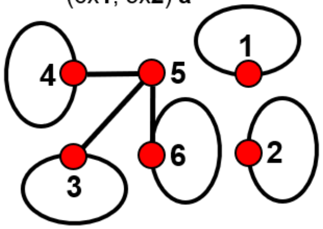	(6x**1**, 5x**2**) **b** 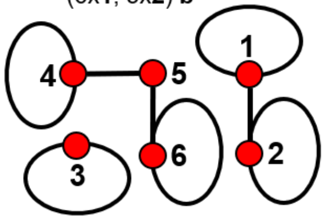
	(6x**1**, 3x**2**, 2x**2**^{1}) **a** 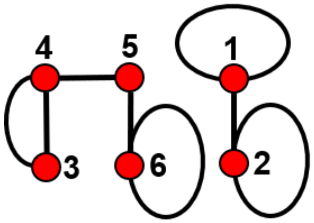	(6x**1**, 3x**2**, 2x**2**^{1}) **b** 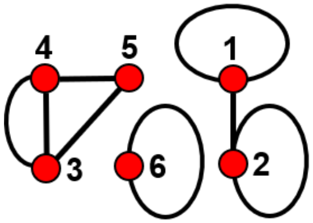	(6x**1**, 3x**2**, 2x**2**^{1}) **c** 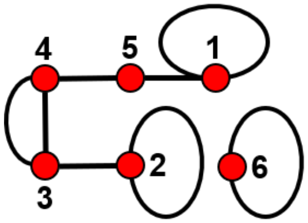	(6x**1**, 3x**2**, 2x**2**^{1}) **d** 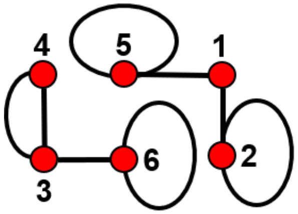
	(6x**1**, 3x**2**, 2x**2**^{1}) **e** 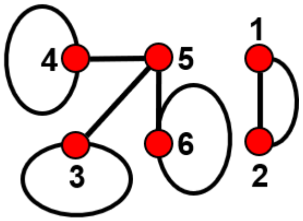	(6x**1**, 3x**2**, 2x**2**^{1}) **f** 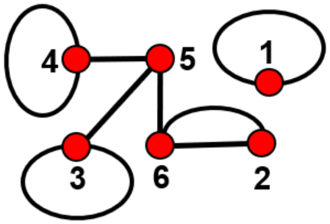	(6x**1**, 3x**2**, 2x**2**^{1}) **g** 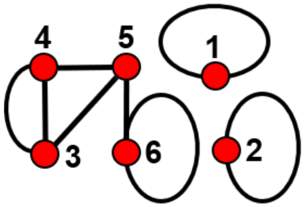	(6x**1**, 3x**2**, 2x**2**^{2}) **a** 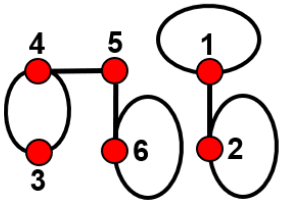
	(6x**1**, 3x**2**, 2x**2**^{2}) **b** 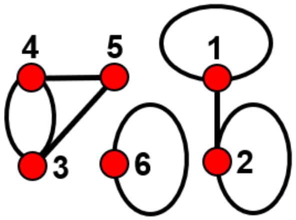	(6x**1**, 3x**2**, 2x**2**^{2}) **c** 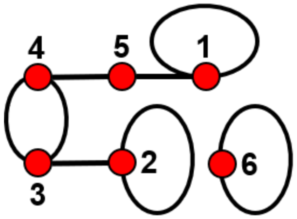	(6x**1**, 3x**2**, 2x**2**^{2}) **d** 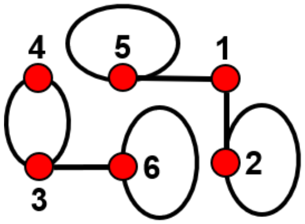	(6x**1**, 3x**2**, 2x**2**^{2}) **e** 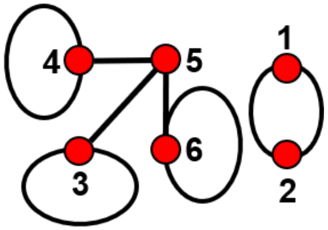

	(6x**1**, 3x**2**, 2x$\mathbf{2}^2$) f 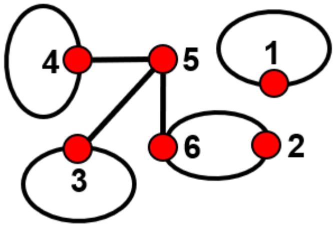	(6x**1**, 3x**2**, 2x$\mathbf{2}^2$) g 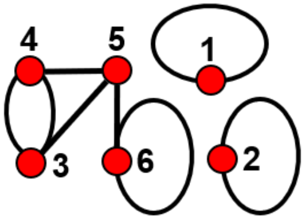	(6x**1**, 1x**2**, 4x$\mathbf{2}^1$) a 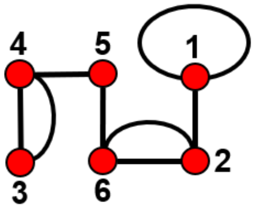	(6x**1**, 1x**2**, 4x$\mathbf{2}^1$) b 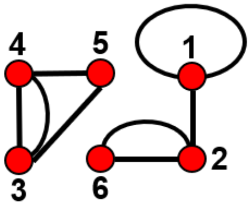
	(6x**1**, 1x**2**, 4x$\mathbf{2}^1$) c 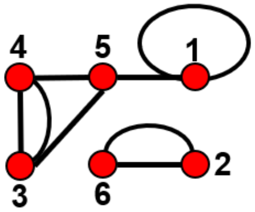	(6x**1**, 1x**2**, 4x$\mathbf{2}^1$) d 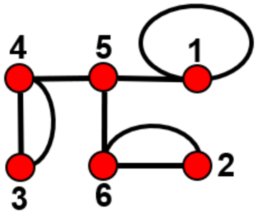	(6x**1**, 1x**2**, 4x$\mathbf{2}^1$) e 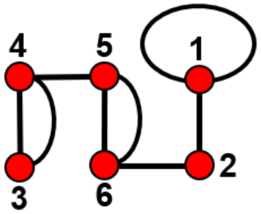	(6x**1**, 1x**2**, 4x$\mathbf{2}^1$) f 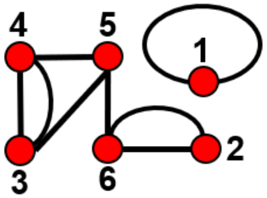
	(6x**1**, 1x**2**, 4x$\mathbf{2}^1$) g 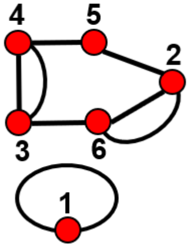	(6x**1**, 1x**2**, 2x$\mathbf{2}^1$, 2x$\mathbf{2}^2$) a 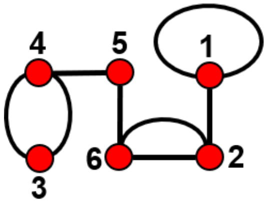	(6x**1**, 1x**2**, 2x$\mathbf{2}^1$, 2x$\mathbf{2}^2$) b 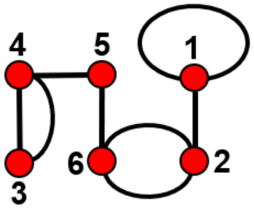	(6x**1**, 1x**2**, 2x$\mathbf{2}^1$, 2x$\mathbf{2}^2$) c 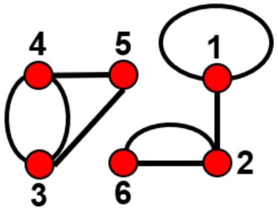
	(6x**1**, 1x**2**, 2x$\mathbf{2}^1$, 2x$\mathbf{2}^2$) d 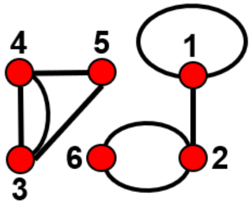	(6x**1**, 1x**2**, 2x$\mathbf{2}^1$, 2x$\mathbf{2}^2$) e 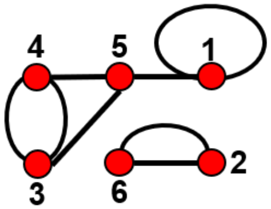	(6x**1**, 1x**2**, 2x$\mathbf{2}^1$, 2x$\mathbf{2}^2$) f 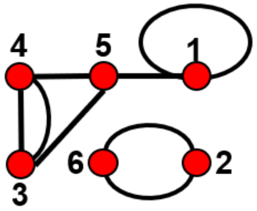	(6x**1**, 1x**2**, 2x$\mathbf{2}^1$, 2x$\mathbf{2}^2$) g 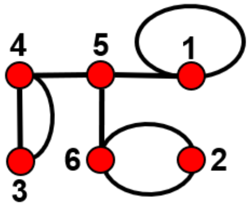

	(6x**1**, 1x**2**, 2x**2**^{1}, 2x**2**^{2}) **h** 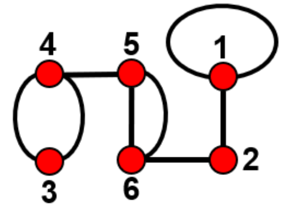	(6x**1**, 1x**2**, 2x**2**^{1}, 2x**2**^{2}) **i** 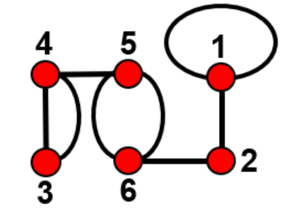	(6x**1**, 1x**2**, 2x**2**^{1}, 2x**2**^{2}) **j** 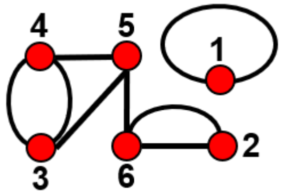	(6x**1**, 1x**2**, 2x**2**^{1}, 2x**2**^{2}) **k** 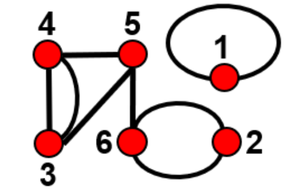
	(6x**1**, 1x**2**, 2x**2**^{1}, 2x**2**^{2}) **l** 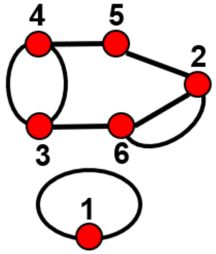	(6x**1**, 1x**2**, 4x**2**^{2}) **a** 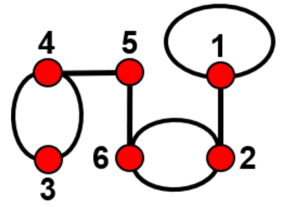	(6x**1**, 1x**2**, 4x**2**^{2}) **b** 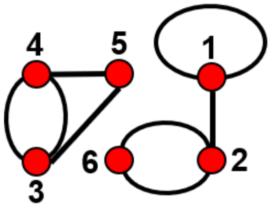	(6x**1**, 1x**2**, 4x**2**^{2}) **c** 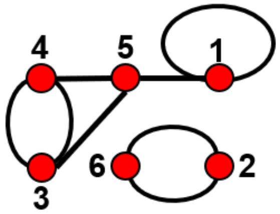
	(6x**1**, 1x**2**, 4x**2**^{2}) **d** 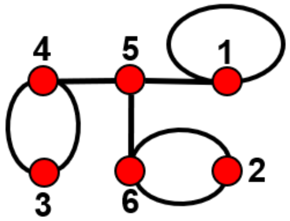	(6x**1**, 1x**2**, 4x**2**^{2}) **e** 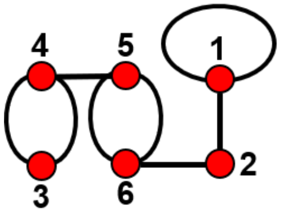	(6x**1**, 1x**2**, 4x**2**^{2}) **f** 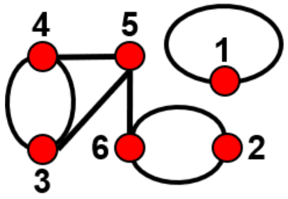	(6x**1**, 1x**2**, 4x**2**^{2}) **g** 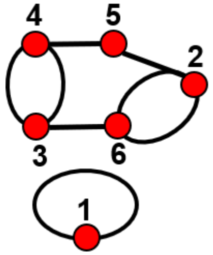
	(4x**1**, 4x**2**, 2x**2**^{1}) **a** 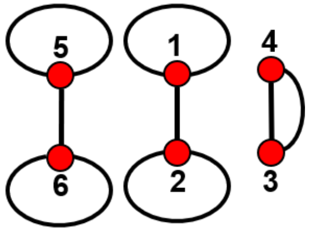	(4x**1**, 4x**2**, 2x**2**^{1}) **b** 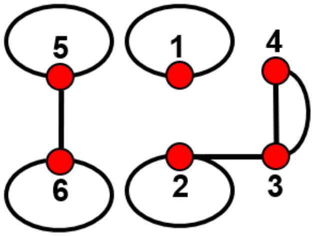	(4x**1**, 4x**2**, 2x**2**^{1}) **c** 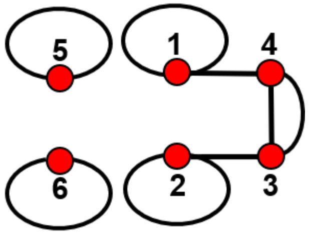	(4x**1**, 4x**2**, 2x**2**^{2}) **a** 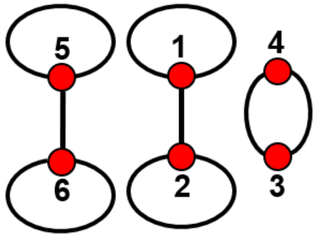

	(4x**1**, 4x**2**, 2x$\mathbf{2}^2$) **b** 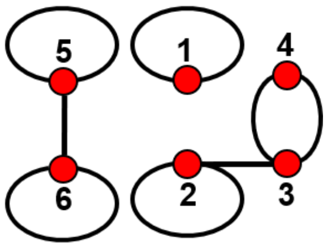	(4x**1**, 4x**2**, 2x$\mathbf{2}^2$) **c** 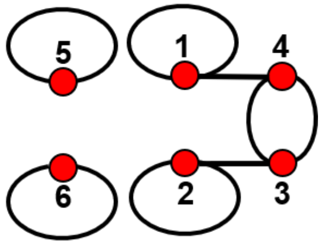	(4x**1**, 2x**2**, 4x$\mathbf{2}^1$) **a** 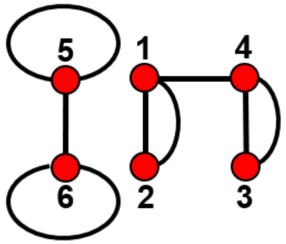	(4x**1**, 2x**2**, 4x$\mathbf{2}^1$) **b** 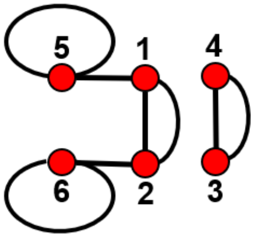
	(4x**1**, 2x**2**, 4x$\mathbf{2}^1$) **c** 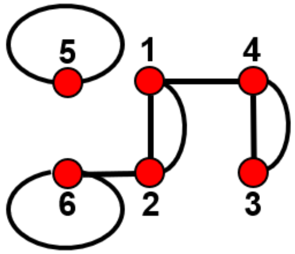	(4x**1**, 2x**2**, 4x$\mathbf{2}^1$) **d** 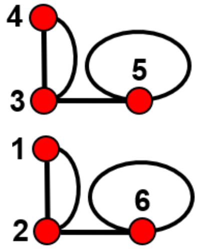	(4x**1**, 2x**2**, 4x$\mathbf{2}^1$) **e** 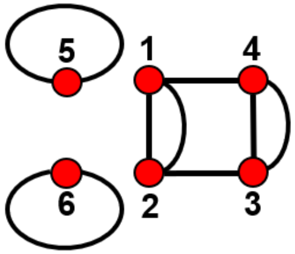	(4x**1**, 2x**2**, 2x$\mathbf{2}^1$, 2x$\mathbf{2}^2$) **a** 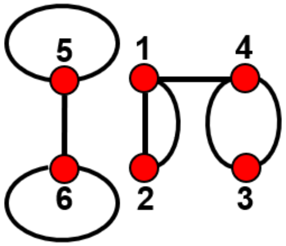
	(4x**1**, 2x**2**, 2x$\mathbf{2}^1$, 2x$\mathbf{2}^2$) **b** 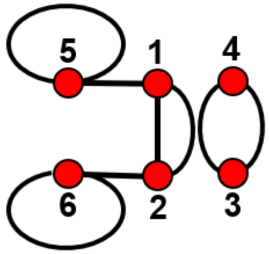	(4x**1**, 2x**2**, 2x$\mathbf{2}^1$, 2x$\mathbf{2}^2$) **c** 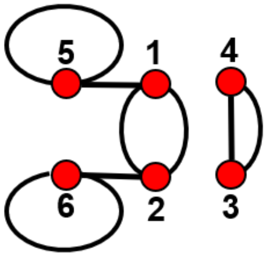	(4x**1**, 2x**2**, 2x$\mathbf{2}^1$, 2x$\mathbf{2}^2$) **d** 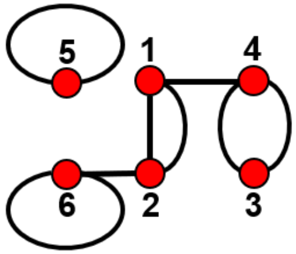	(4x**1**, 2x**2**, 2x$\mathbf{2}^1$, 2x$\mathbf{2}^2$) **e** 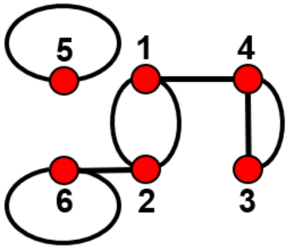

	(4x**1**, 2x**2**, 2x$\mathbf{2}^1$, 2x$\mathbf{2}^2$) **f** 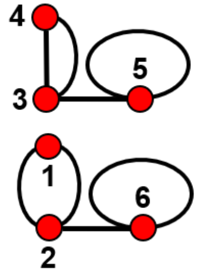	(4x**1**, 2x**2**, 2x$\mathbf{2}^1$, 2x$\mathbf{2}^2$) **g** 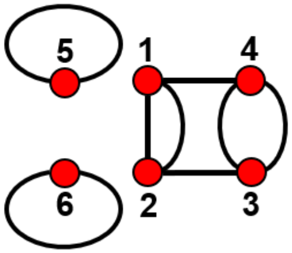	(4x**1**, 2x**2**, 4x$\mathbf{2}^2$) **a** 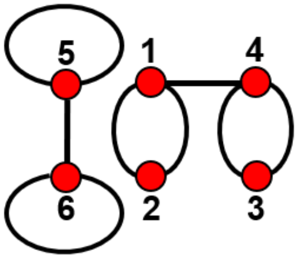	(4x**1**, 2x**2**, 4x$\mathbf{2}^2$) **b** 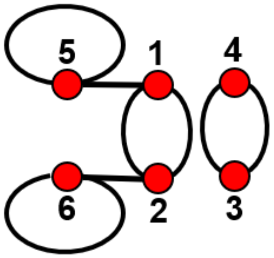
	(4x**1**, 2x**2**, 4x$\mathbf{2}^2$) **c** 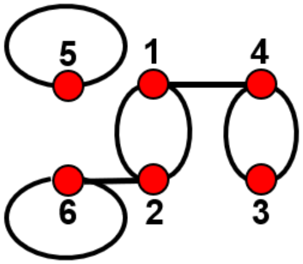	(4x**1**, 2x**2**, 4x$\mathbf{2}^2$) **d** 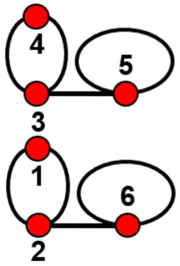	(4x**1**, 2x**2**, 4x$\mathbf{2}^2$) **e** 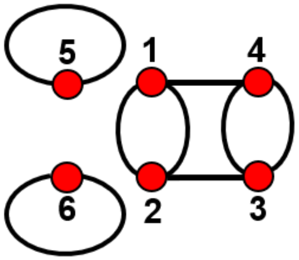	(4x**1**, 6x$\mathbf{2}^1$) **a** 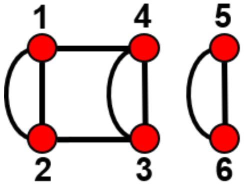
	(4x**1**, 6x$\mathbf{2}^1$) **b** 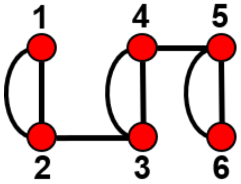	(4x**1**, 4x$\mathbf{2}^1$, 2x$\mathbf{2}^2$) **a** 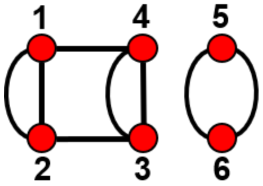	(4x**1**, 4x$\mathbf{2}^1$, 2x$\mathbf{2}^2$) **b** 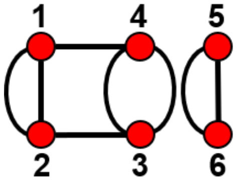	(4x**1**, 4x$\mathbf{2}^1$, 2x$\mathbf{2}^2$) **c** 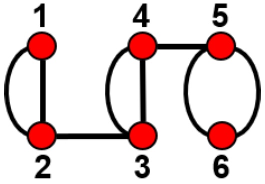

	(4x**1**, 4x**2**^{1}, 2x**2**^{2}) **d** 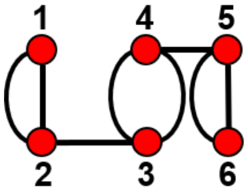	(4x**1**, 2x**2**^{1}, 4x**2**^{2}) **a** 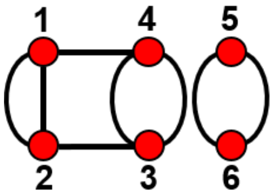	(4x**1**, 2x**2**^{1}, 4x**2**^{2}) **b** 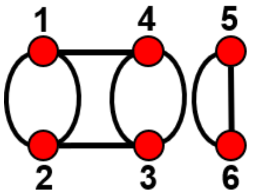	(4x**1**, 2x**2**^{1}, 4x**2**^{2}) **c** 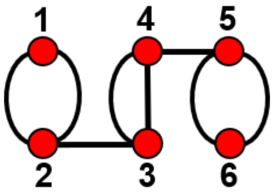
	(4x**1**, 2x**2**^{1}, 4x**2**^{2}) **d** 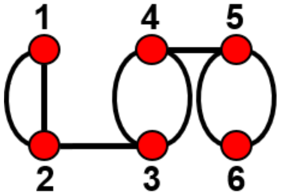	(4x**1**, 6x**2**^{2}) **a** 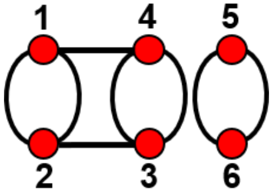	(4x**1**, 6x**2**^{2}) **b** 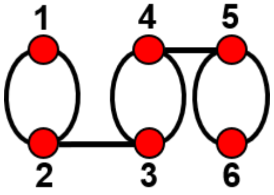	
$^2V_2{}^3V_6$	NG			
$^2V_2{}^3V_8$	NG			
$^2V_3{}^3V_2$	(12x**1**) **a** 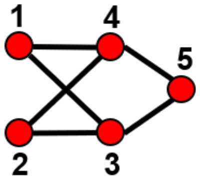	(12x**1**) **b** 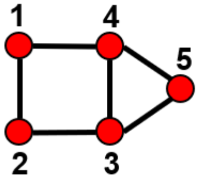	(10x**1**, 1x**2**) **a** 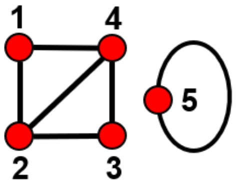	(10x**1**, 1x**2**) **b** 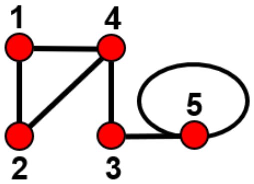
	(10x**1**, 1x**2**) **c** 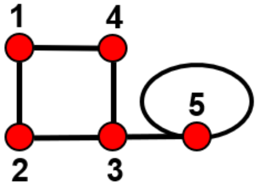	(8x**1**, 2x**2**) **a** 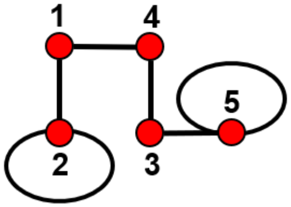	(8x**1**, 2x**2**) **b** 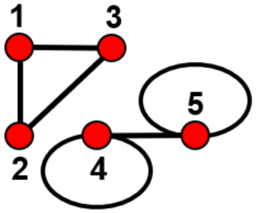	(8x**1**, 2x**2**) **c** 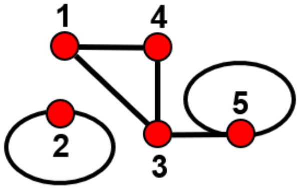

	(8x**1**, 2x**2**^{1}) **a**<br>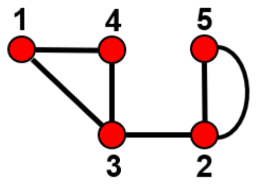	(8x**1**, 2x**2**^{1}) **b**<br>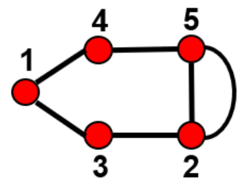	(8x**1**, 2x**2**^{2}) **a**<br>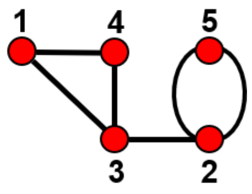	(8x**1**, 2x**2**^{2}) **b**<br>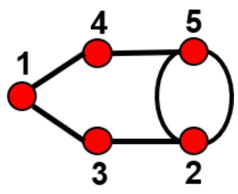
	(6x**1**, 3x**2**)<br>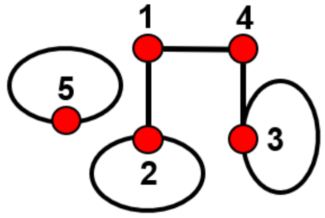	(6x**1**, 1x**2**, 2x**2**^{1}) **a**<br>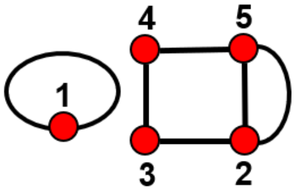	(6x**1**, 1x**2**, 2x**2**^{1}) **b**<br>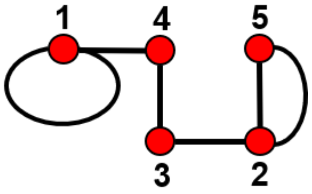	(6x**1**, 1x**2**, 2x**2**^{2}) **a**<br>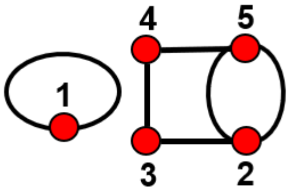
	(6x**1**, 1x**2**, 2x**2**^{2}) **b**<br>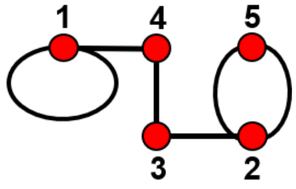	(4x**1**, 4x**2**)<br>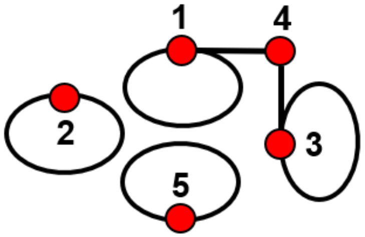	(4x**1**, 2x**2**, 2x**2**^{1}) **a**<br>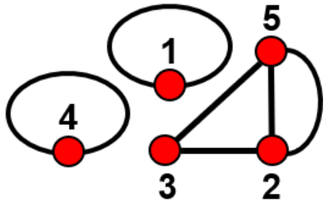	(4x**1**, 2x**2**, 2x**2**^{1}) **b**<br>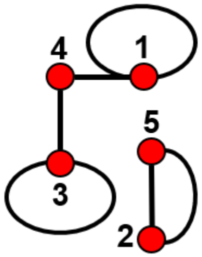
	(4x**1**, 2x**2**, 2x**2**^{1}) **c**<br>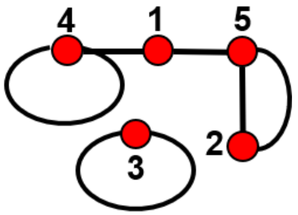	(4x**1**, 2x**2**, 2x**2**^{2}) **a**<br>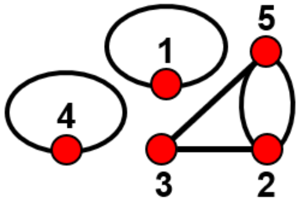	(4x**1**, 2x**2**, 2x**2**^{2}) **b**<br>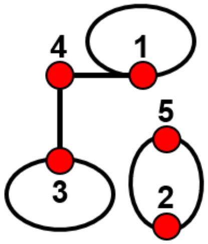	(4x**1**, 2x**2**, 2x**2**^{2}) **c**<br>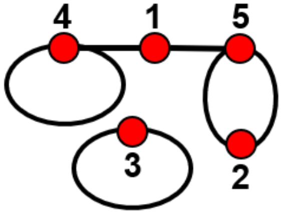

	(4x**1**, 4x**2**[1]) **a** 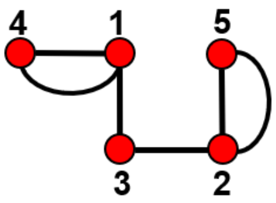	(4x**1**, 4x**2**[1]) **b** 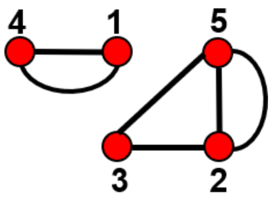	(4x**1**, 2x**2**[1], 2x**2**[2]) **a** 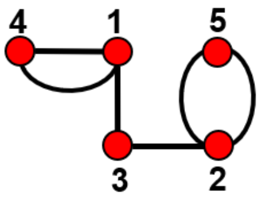	(4x**1**, 2x**2**[1], 2x**2**[2]) **b** 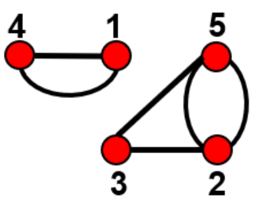
	(4x**1**, 2x**2**[1], 2x**2**[2]) **c** 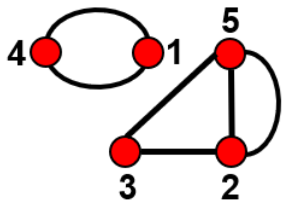	(4x**1**, 4x**2**[2]) **a** 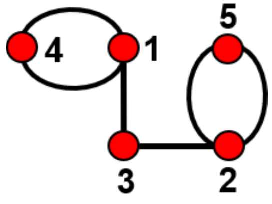	(4x**1**, 4x**2**[2]) **b** 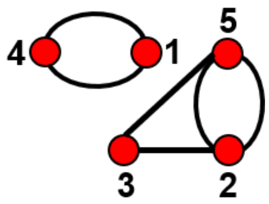	(2x**1**, 5x**2**) 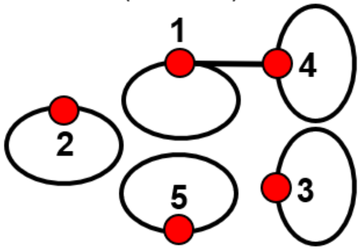
	(2x**1**, 3x**2**, 2x**2**[1]) **a** 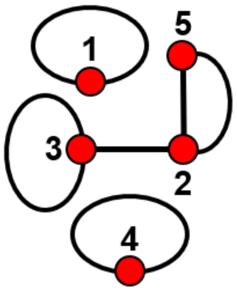	(2x**1**, 3x**2**, 2x**2**[1]) **b** 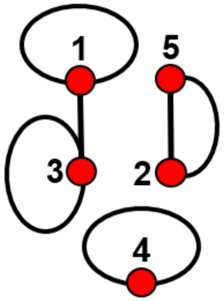	(2x**1**, 3x**2**, 2x**2**[2]) **a** 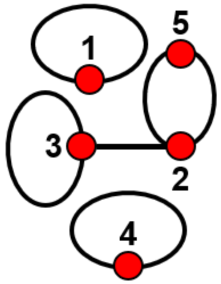	(2x**1**, 3x**2**, 2x**2**[2]) **b** 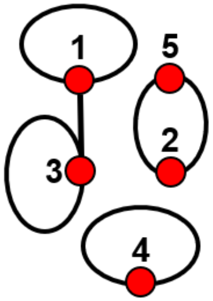
	(2x**1**, 1x**2**, 4x**2**[1]) **a** 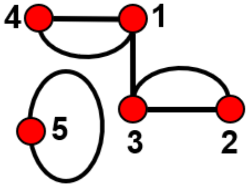	(2x**1**, 1x**2**, 4x**2**[1]) **b** 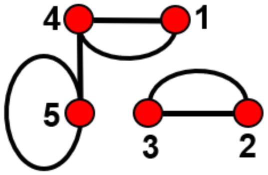	(2x**1**, 1x**2**, 2x**2**[1], 2x**2**[2]) **a** 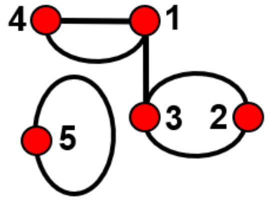	(2x**1**, 1x**2**, 2x**2**[1], 2x**2**[2]) **b** 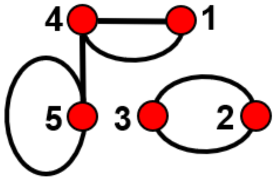

	(2x**1**, 1x**2**, 2x$\mathbf{2}^1$, 2x$\mathbf{2}^2$) **c** 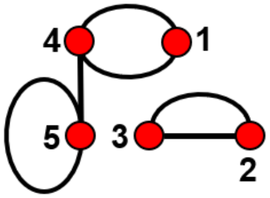	(2x**1**, 1x**2**, 4x$\mathbf{2}^2$) **a** 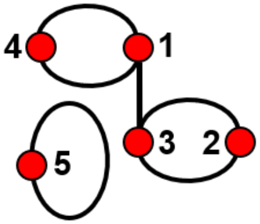	(2x**1**, 1x**2**, 4x$\mathbf{2}^2$) **b** 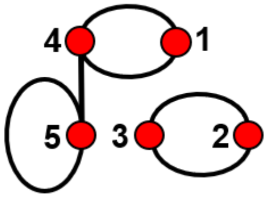	
$^2V_3{}^3V_4$	NG			
$^2V_4{}^3V_2$	(14x**1**) **a** 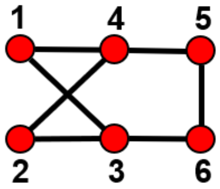	(14x**1**) **b** 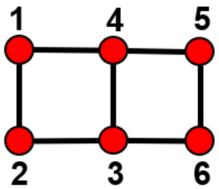	(14x**1**) **c** 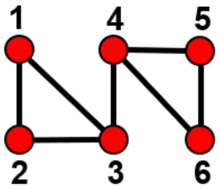	(14x**1**) **d** 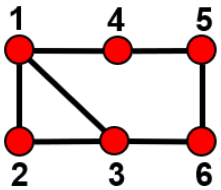
	(12x**1**, 1x**2**) **a** 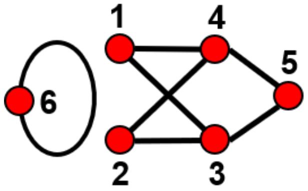	(12x**1**, 1x**2**) **b** 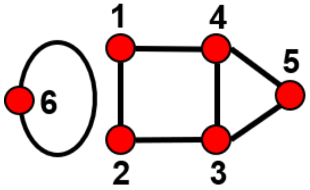	(12x**1**, 1x**2**) **c** 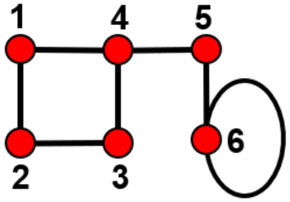	(12x**1**, 1x**2**) **d** 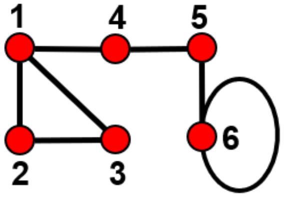
	(12x**1**, 1x**2**) **e** 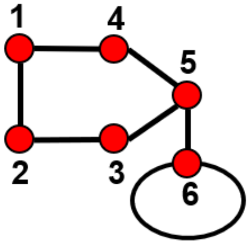	(10x**1**, 2x**2**) **a** 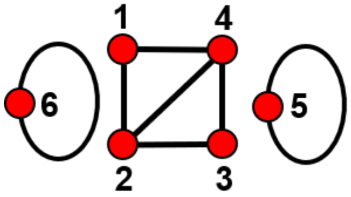	(10x**1**, 2x**2**) **b** 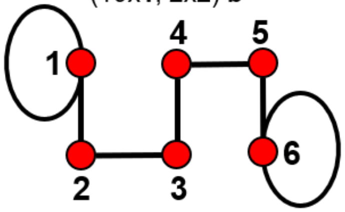	(10x**1**, 2x**2**) **c** 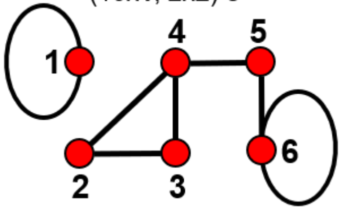

	(10x**1**, 2x**2**) **d** 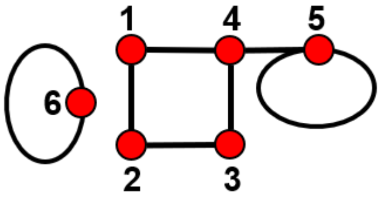	(10x**1**, 2x**2**) **e** 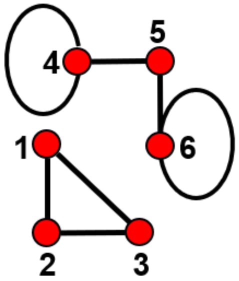	(10x**1**, 2x**2**) **f** 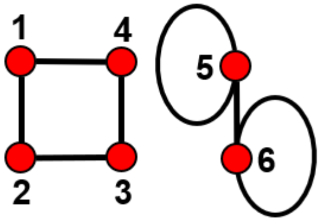	(10x**1**, 2x**2**^{1}) **a** 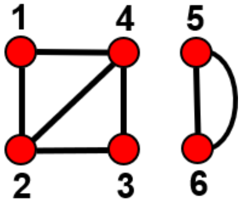
	(10x**1**, 2x**2**^{1}) **b** 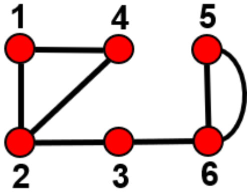	(10x**1**, 2x**2**^{1}) **c** 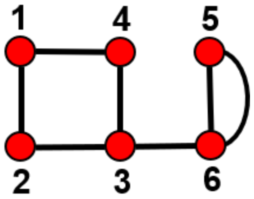	(10x**1**, 2x**2**^{1}) **d** 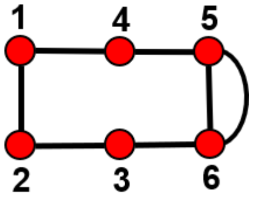	(10x**1**, 2x**2**^{1}) **e** 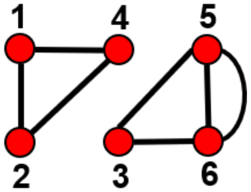
	(10x**1**, 2x**2**^{2}) **a** 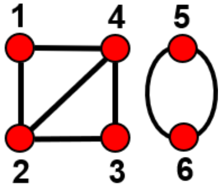	(10x**1**, 2x**2**^{2}) **b** 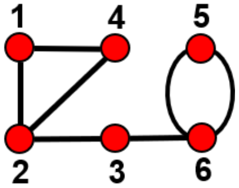	(10x**1**, 2x**2**^{2}) **c** 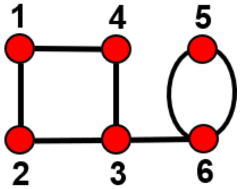	(10x**1**, 2x**2**^{2}) **d** 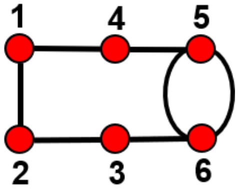
	(10x**1**, 2x**2**^{2}) **e** 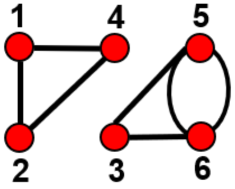	(8x**1**, 3x**2**) **a** 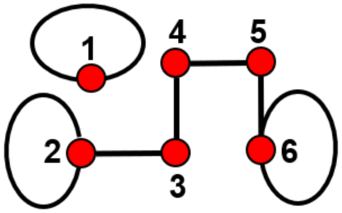	(8x**1**, 3x**2**) **b** 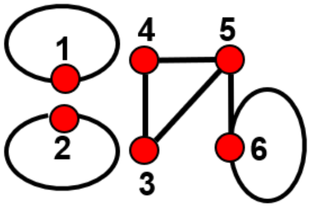	(8x**1**, 3x**2**) **c** 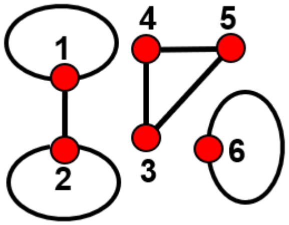

	(8x**1**, 1x**2**, 2x**2**^{1}) **a** 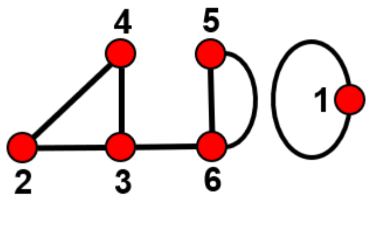	(8x**1**, 1x**2**, 2x**2**^{1}) **b** 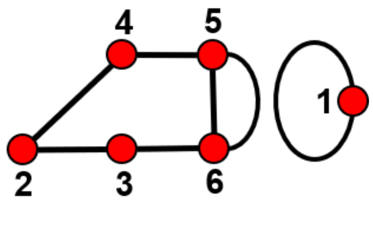	(8x**1**, 1x**2**, 2x**2**^{1}) **c** 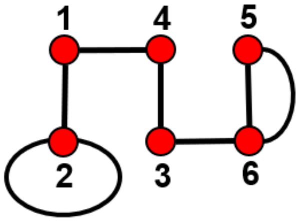	(8x**1**, 1x**2**, 2x**2**^{1}) **d** 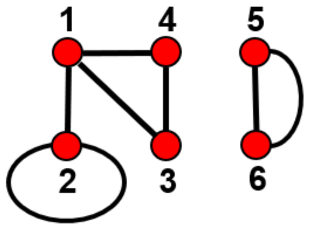
	(8x**1**, 1x**2**, 2x**2**^{1}) **e** 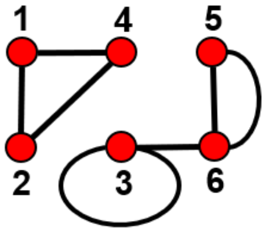	(8x**1**, 1x**2**, 2x**2**^{2}) **a** 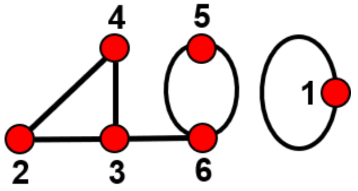	(8x**1**, 1x**2**, 2x**2**^{2}) **b** 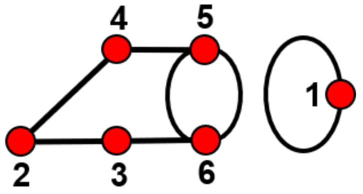	(8x**1**, 1x**2**, 2x**2**^{2}) **c** 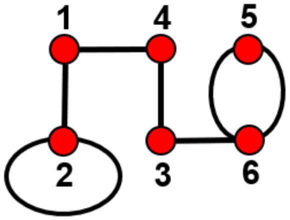
	(8x**1**, 1x**2**, 2x**2**^{2}) **d** 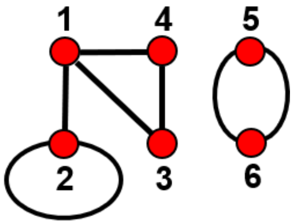	(8x**1**, 1x**2**, 2x**2**^{2}) **e** 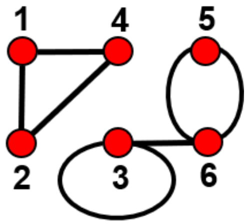	(6x**1**, 4x**2**) 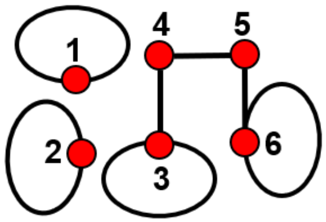	(6x**1**, 2x**2**, 2x**2**^{1}) **a** 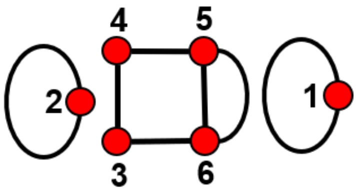
	(6x**1**, 2x**2**, 2x**2**^{1}) **b** 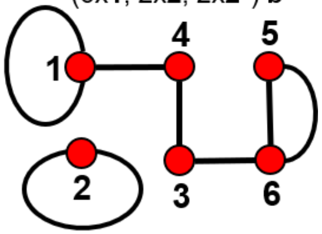	(6x**1**, 2x**2**, 2x**2**^{1}) **c** 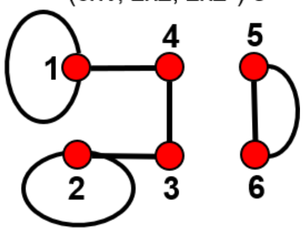	(6x**1**, 2x**2**, 2x**2**^{2}) **a** 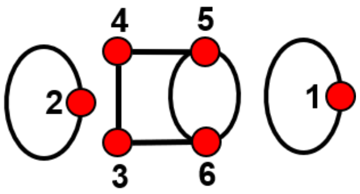	(6x**1**, 2x**2**, 2x**2**^{2}) **b** 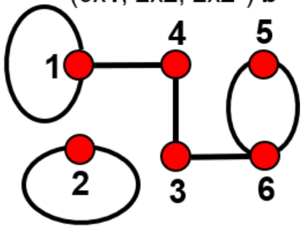

(6x**1**, 2x**2**, 2x$\mathbf{2}^{2}$) **c**

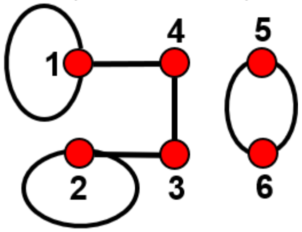

(6x**1**, 4x$\mathbf{2}^{1}$) **a**

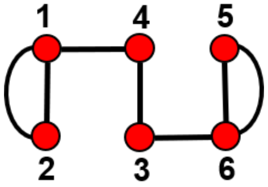

(6x**1**, 4x$\mathbf{2}^{1}$) **b**

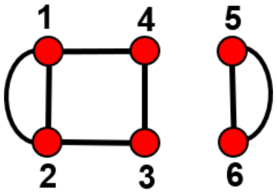

(6x**1**, 2x$\mathbf{2}^{1}$, 2x$\mathbf{2}^{2}$) **a**

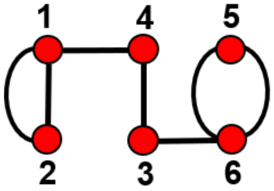

(6x**1**, 2x$\mathbf{2}^{1}$, 2x$\mathbf{2}^{2}$) **b**

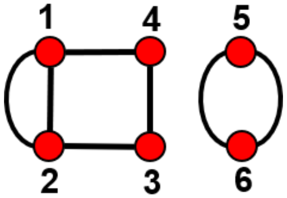

(6x**1**, 2x$\mathbf{2}^{1}$, 2x$\mathbf{2}^{2}$) **c**

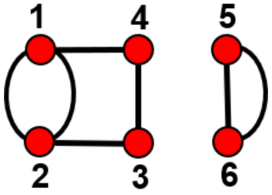

(6x**1**, 4x$\mathbf{2}^{2}$) **a**

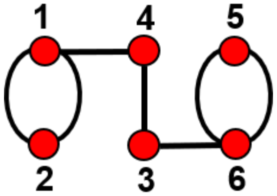

(6x**1**, 4x$\mathbf{2}^{2}$) **b**

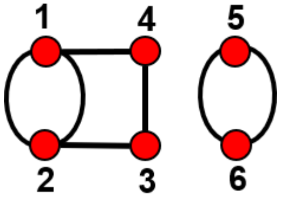

(4x**1**, 5x**2**)

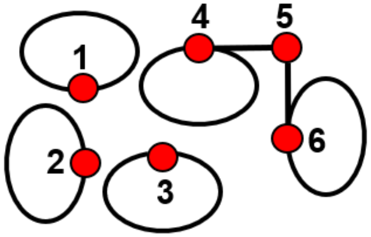

(4x**1**, 3x**2**, 2x$\mathbf{2}^{1}$) **a**

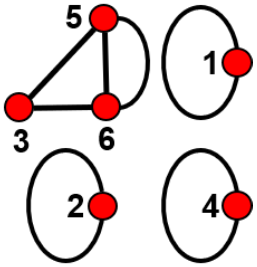

(4x**1**, 3x**2**, 2x$\mathbf{2}^{1}$) **b**

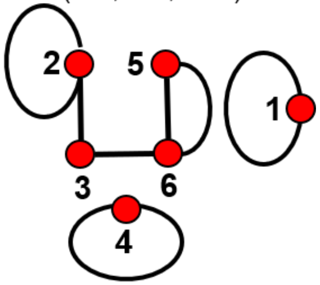

(4x**1**, 3x**2**, 2x$\mathbf{2}^{1}$) **c**

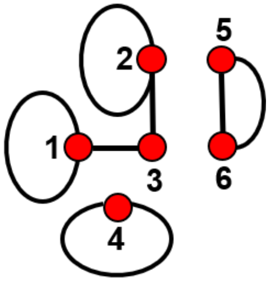

	(4x**1**, 3x**2**, 2x**2**^{2}) **a** 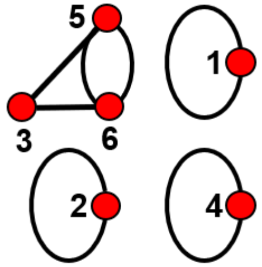	(4x**1**, 3x**2**, 2x**2**^{2}) **b** 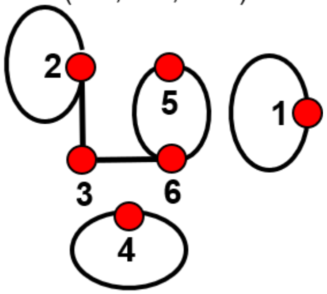	(4x**1**, 3x**2**, 2x**2**^{2}) **c** 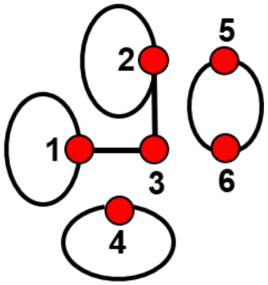	(4x**1**, 1x**2**, 4x**2**^{1}) **a** 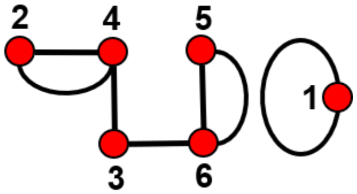
	(4x**1**, 1x**2**, 4x**2**^{1}) **b** 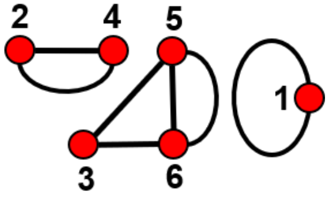	(4x**1**, 1x**2**, 4x**2**^{1}) **c** 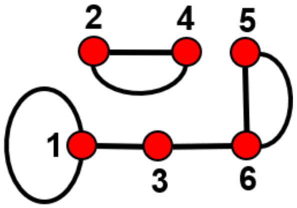	(4x**1**, 1x**2**, 2x**2**^{1}, 2x**2**^{2}) **a** 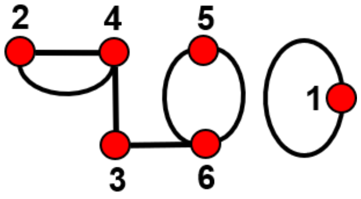	(4x**1**, 1x**2**, 2x**2**^{1}, 2x**2**^{2}) **b** 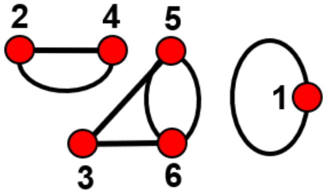
	(4x**1**, 1x**2**, 2x**2**^{1}, 2x**2**^{2}) **c** 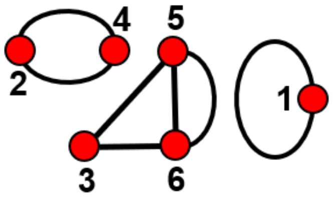	(4x**1**, 1x**2**, 2x**2**^{1}, 2x**2**^{2}) **d** 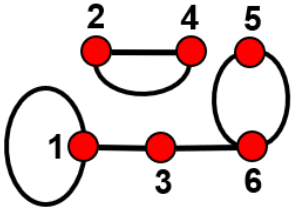	(4x**1**, 1x**2**, 2x**2**^{1}, 2x**2**^{2}) **e** 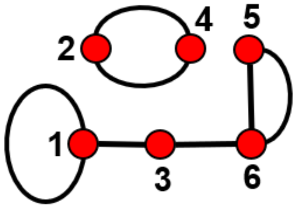	(4x**1**, 1x**2**, 4x**2**^{2}) **a** 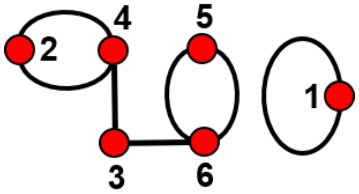
	(4x**1**, 1x**2**, 4x**2**^{2}) **b** 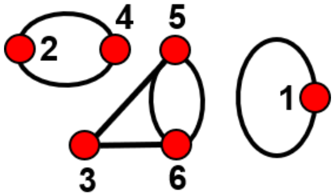	(4x**1**, 1x**2**, 4x**2**^{2}) **c** 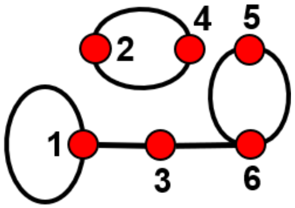	(2x**1**, 6x**2**) 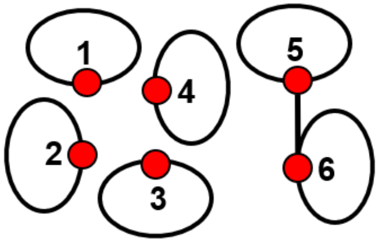	(2x**1**, 4x**2**, 2x**2**^{1}) **a** 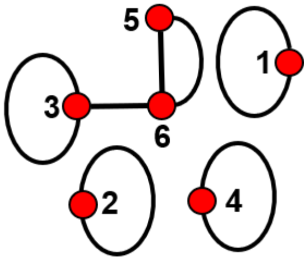

	(2x**1**, 4x**2**, 2x**2**^{1}) **b** 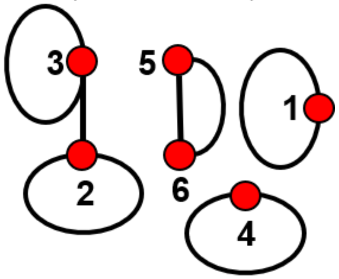	(2x**1**, 4x**2**, 2x**2**^{2}) **a** 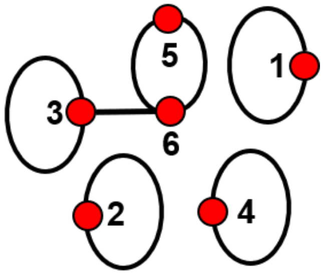	(2x**1**, 4x**2**, 2x**2**^{2}) **b** 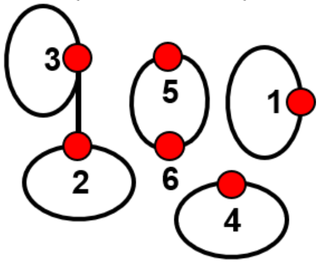	(2x**1**, 2x**2**, 4x**2**^{1}) **a** 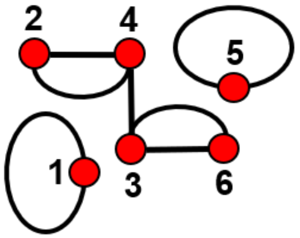
	(2x**1**, 2x**2**, 4x**2**^{1}) **b** 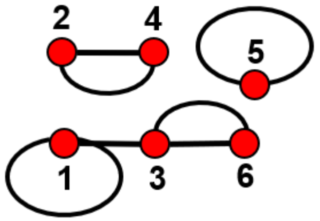	(2x**1**, 2x**2**, 4x**2**^{1}) **c** 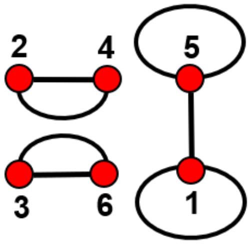	(2x**1**, 2x**2**, 2x**2**^{1}, 2x**2**^{2}) **a** 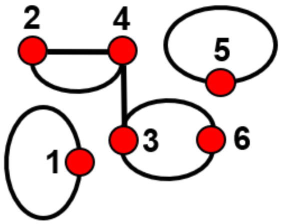	(2x**1**, 2x**2**, 2x**2**^{1}, 2x**2**^{2}) **b** 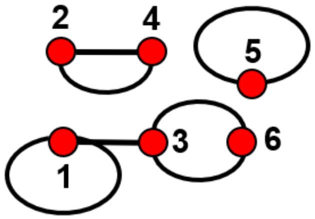
	(2x**1**, 2x**2**, 2x**2**^{1}, 2x**2**^{2}) **c** 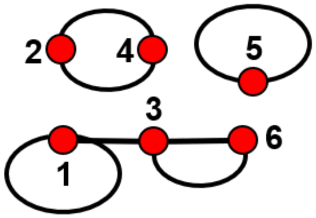	(2x**1**, 2x**2**, 2x**2**^{1}, 2x**2**^{2}) **d** 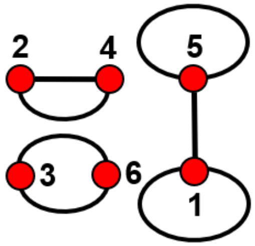	(2x**1**, 2x**2**, 4x**2**^{2}) **a** 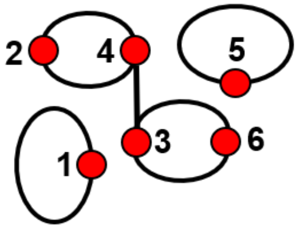	(2x**1**, 2x**2**, 4x**2**^{2}) **b** 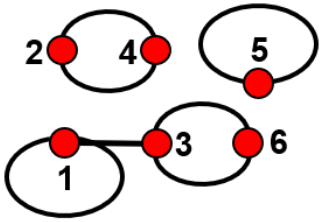

	$(2\text{x}\mathbf{1}, 2\text{x}\mathbf{2}, 4\text{x}\mathbf{2}^{2})$ **c**	$(2\text{x}\mathbf{1}, 6\text{x}\mathbf{2}^{1})$	$(2\text{x}\mathbf{1}, 4\text{x}\mathbf{2}^{1}, 2\text{x}\mathbf{2}^{2})$ **a**	$(2\text{x}\mathbf{1}, 4\text{x}\mathbf{2}^{1}, 2\text{x}\mathbf{2}^{2})$ **b**
	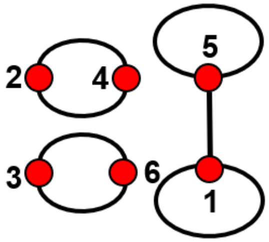	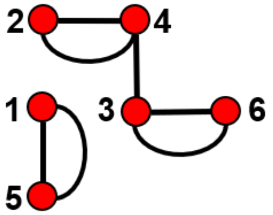	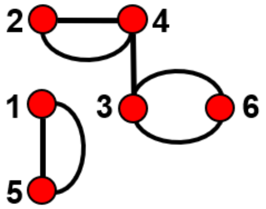	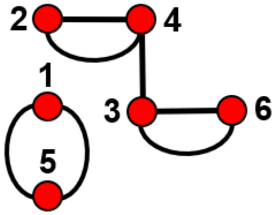
	$(2\text{x}\mathbf{1}, 2\text{x}\mathbf{2}^{1}, 4\text{x}\mathbf{2}^{2})$ **a**	$(2\text{x}\mathbf{1}, 2\text{x}\mathbf{2}^{1}, 4\text{x}\mathbf{2}^{2})$ **b**	$(2\text{x}\mathbf{1}, 6\text{x}\mathbf{2}^{2})$	
	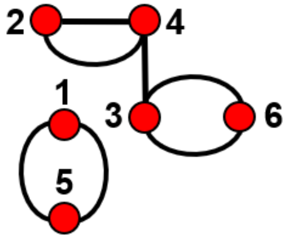	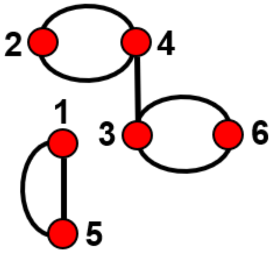	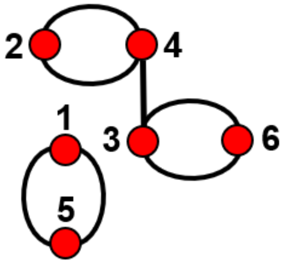	
$^{2}V_{4}{}^{3}V_{4}$	NG			
$^{2}V_{5}{}^{3}V_{2}$	NG			
$^{2}V_{6}{}^{3}V_{2}$	NG			
$\boldsymbol{^{2}V_{r}{}^{4}V_{r}}$				
$^{2}V_{1}{}^{4}V_{1}$	$(1\text{x}\mathbf{2}, 2\text{x}\mathbf{2}^{1})$	$(1\text{x}\mathbf{2}, 2\text{x}\mathbf{2}^{2})$		
	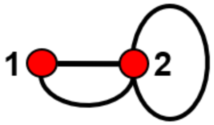	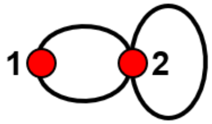		

$^{2}V_{1}{}^{4}V_{2}$	(6x**1**, 2x**2**) 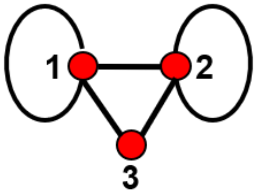	(3x**2**, 2x**2**^{1}) 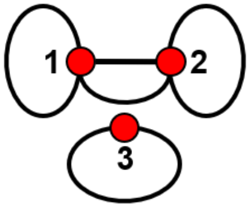	(3x**2**, 2x**2**^{2}) 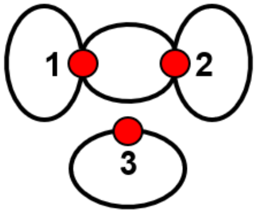	(1x**2**, 4x**2**^{1}) 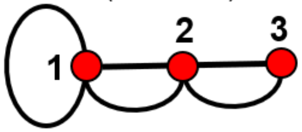
	(1x**2**, 2x**2**^{1}, 2x**2**^{2}) **a** 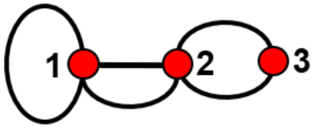	(1x**2**, 2x**2**^{1}, 2x**2**^{2}) **b** 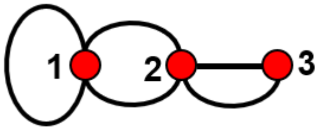	(1x**2**, 4x**2**^{2}) 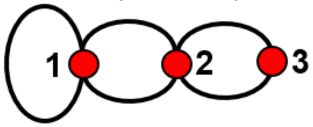	
$^{2}V_{1}{}^{4}V_{3}$	(8x**1**, 3x**2**) 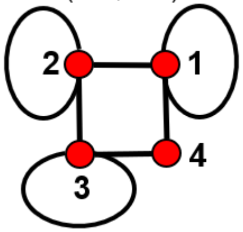	(8x**1**, 1x**2**, 2x**2**^{1}) 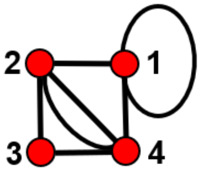	(8x**1**, 1x**2**, 2x**2**^{2}) 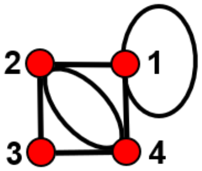	(6x**1**, 4x**2**) 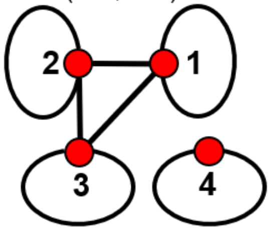
	(6x**1**, 2x**2**, 2x**2**^{1}) **a** 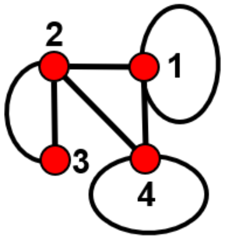	(6x**1**, 2x**2**, 2x**2**^{1}) **b** 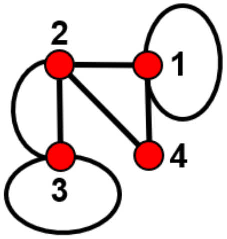	(6x**1**, 2x**2**, 2x**2**^{2}) **a** 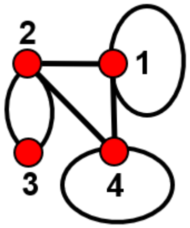	(6x**1**, 2x**2**, 2x**2**^{2}) **b** 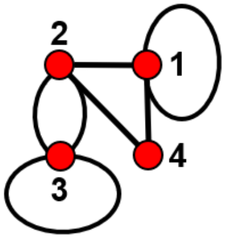

	(6x**1**, 4x**2**^{1}) 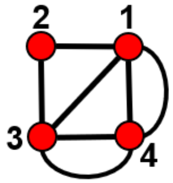	(6x**1**, 2x**2**^{1}, 2x**2**^{2}) 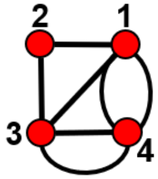	(6x**1**, 4x**2**^{2}) 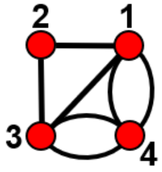	(3x**2**, 4x**2**^{1}) **a** 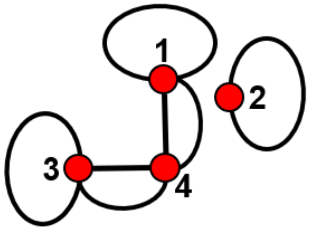
	(3x**2**, 4x**2**^{1}) **b** 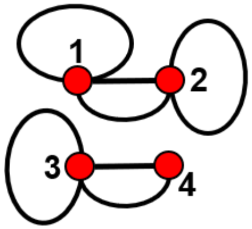	(3x**2**, 2x**2**^{1}, 2x**2**^{2}) **a** 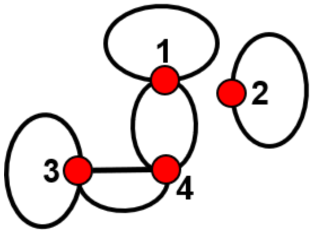	(3x**2**, 2x**2**^{1}, 2x**2**^{2}) **b** 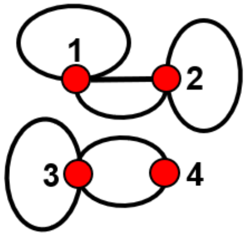	(3x**2**, 2x**2**^{1}, 2x**2**^{2}) **c** 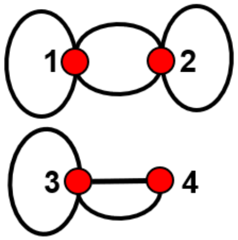
	(3x**2**, 4x**2**^{2}) **a** 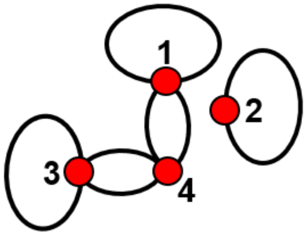	(3x**2**, 4x**2**^{2}) **b** 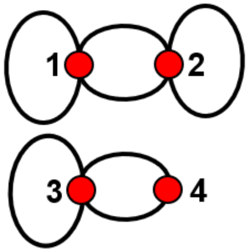	(1x**2**, 6x**2**^{1}) **a** 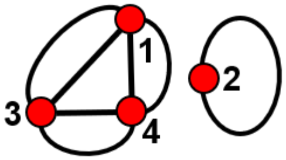	(1x**2**, 6x**2**^{1}) **b** 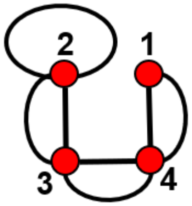
	(1x**2**, 4x**2**^{1}, 2x**2**^{2}) **a** 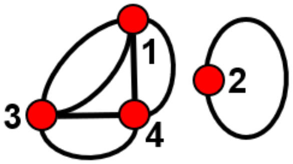	(1x**2**, 4x**2**^{1}, 2x**2**^{2}) **b** 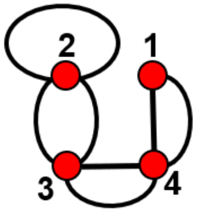	(1x**2**, 4x**2**^{1}, 2x**2**^{2}) **c** 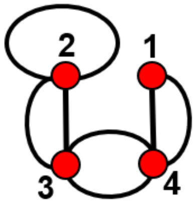	(1x**2**, 4x**2**^{1}, 2x**2**^{2}) **d** 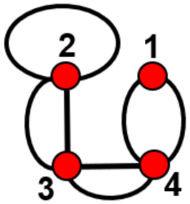

	(1x**2**, 2x**2**^{1}, 4x**2**^{2}) **a** 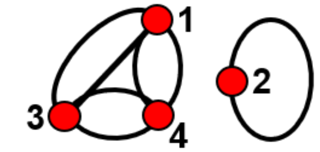	(1x**2**, 2x**2**^{1}, 4x**2**^{2}) **b** 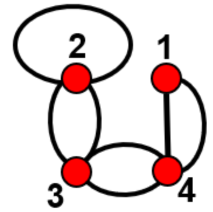	(1x**2**, 2x**2**^{1}, 4x**2**^{2}) **c** 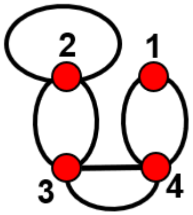	(1x**2**, 2x**2**^{1}, 4x**2**^{2}) **d** 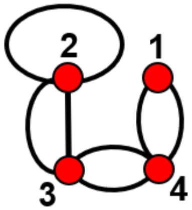
	(1x**2**, 6x**2**^{2}) **a** 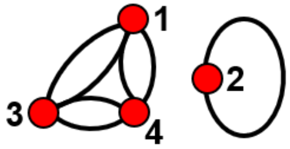	(1x**2**, 6x**2**^{2}) **b** 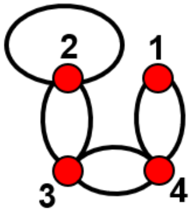		
$^2V_1{}^4V_4$	(14x**1**, 2x**2**) 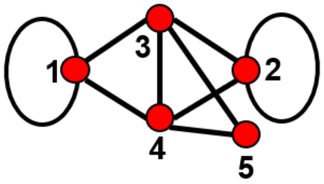	(14x**1**, 2x**2**^{1}) 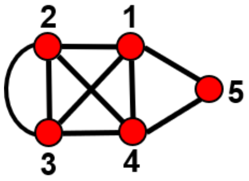	(14x**1**, 2x**2**^{2}) 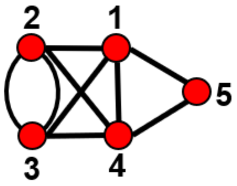	(12x**1**, 3x**2**) 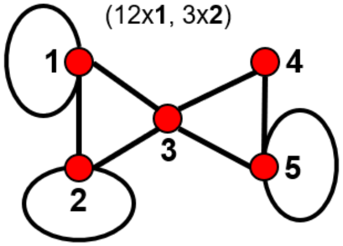
	(12x**1**, 1x**2**, 2x**2**^{1}) 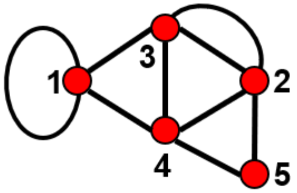	(12x**1**, 1x**2**, 2x**2**^{2}) 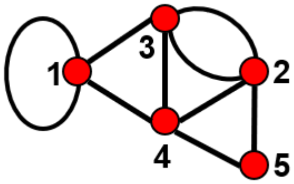	(10x**1**, 4x**2**) 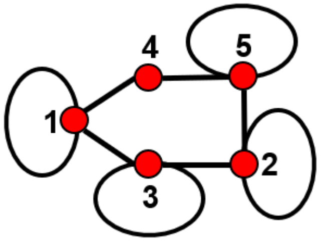	(10x**1**, 2x**2**, 2x**2**^{1}) **a** 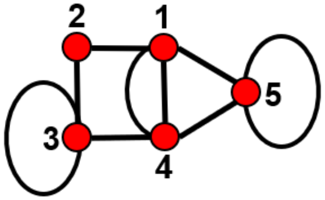

	(10x**1**, 2x**2**, 2x$\mathbf{2}^1$) **b** 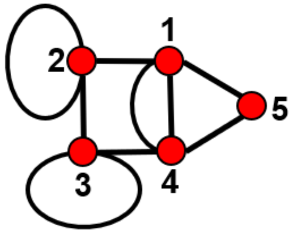	(10x**1**, 2x**2**, 2x$\mathbf{2}^2$) **a** 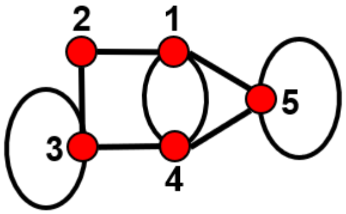	(10x**1**, 2x**2**, 2x$\mathbf{2}^2$) **b** 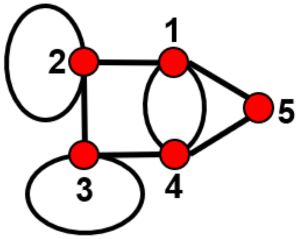	(10x**1**, 4x$\mathbf{2}^1$) 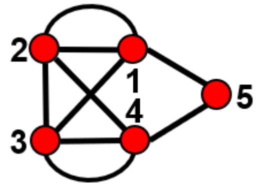
	(10x**1**, 2x$\mathbf{2}^1$, 2x$\mathbf{2}^2$) 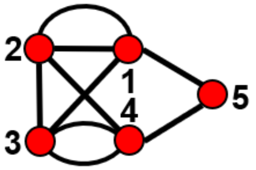	(10x**1**, 4x$\mathbf{2}^2$) 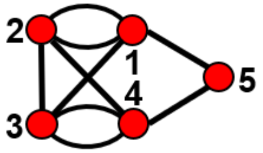	(8x**1**, 5x**2**) 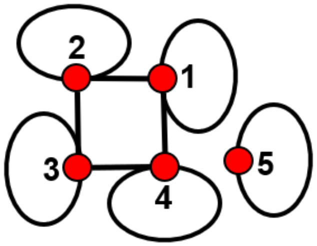	(8x**1**, 3x**2**, 2x$\mathbf{2}^1$) **a** 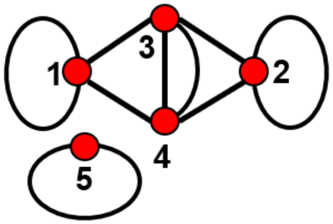
	(8x**1**, 3x**2**, 2x$\mathbf{2}^1$) **b** 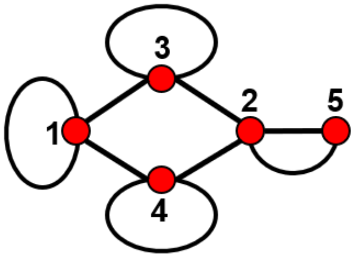	(8x**1**, 3x**2**, 2x$\mathbf{2}^1$) **c** 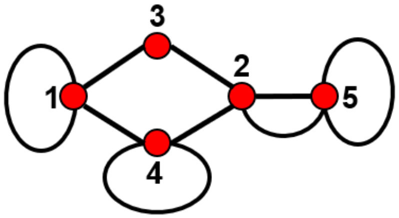	(8x**1**, 3x**2**, 2x$\mathbf{2}^1$) **d** 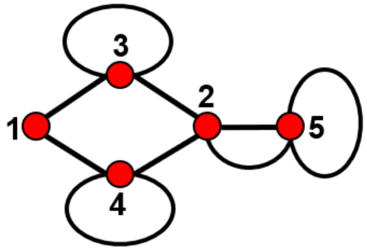	(8x**1**, 3x**2**, 2x$\mathbf{2}^2$) **a** 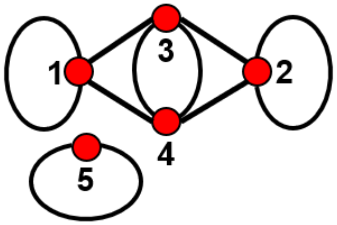
	(8x**1**, 3x**2**, 2x$\mathbf{2}^2$) **b** 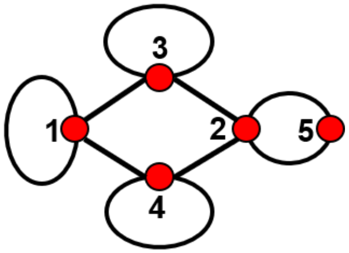	(8x**1**, 3x**2**, 2x$\mathbf{2}^2$) **c** 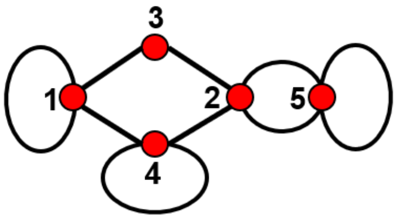	(8x**1**, 3x**2**, 2x$\mathbf{2}^2$) **d** 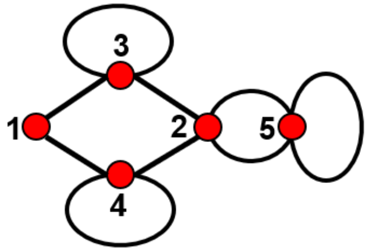	(8x**1**, 1x**2**, 4x$\mathbf{2}^1$) **a** 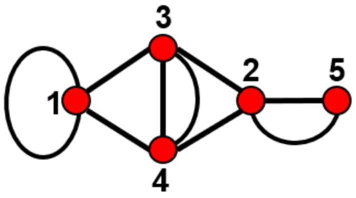

	(8x**1**, 1x**2**, 4x$\mathbf{2}^1$) **b** 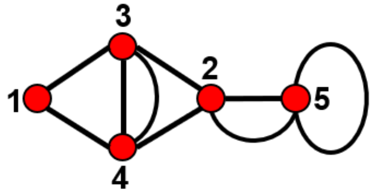	(8x**1**, 1x**2**, 4x$\mathbf{2}^1$) **c** 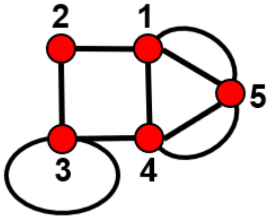	(8x**1**, 1x**2**, 4x$\mathbf{2}^1$) **d** 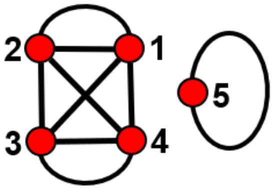	(8x**1**, 1x**2**, 4x$\mathbf{2}^1$) **e** 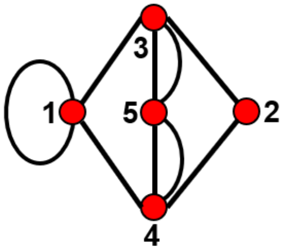
	(8x**1**, 1x**2**, 2x$\mathbf{2}^1$, 2x$\mathbf{2}^2$) **a** 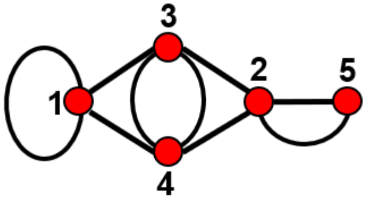	(8x**1**, 1x**2**, 2x$\mathbf{2}^1$, 2x$\mathbf{2}^2$) **b** 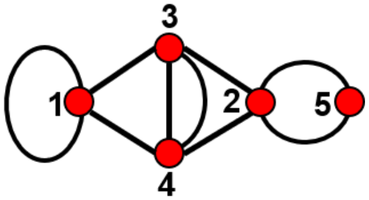	(8x**1**, 1x**2**, 2x$\mathbf{2}^1$, 2x$\mathbf{2}^2$) **c** 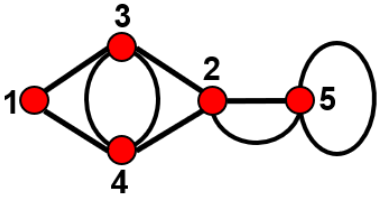	(8x**1**, 1x**2**, 2x$\mathbf{2}^1$, 2x$\mathbf{2}^2$) **d** 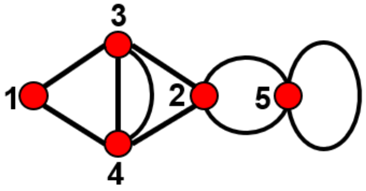
	(8x**1**, 1x**2**, 2x$\mathbf{2}^1$, 2x$\mathbf{2}^2$) **e** 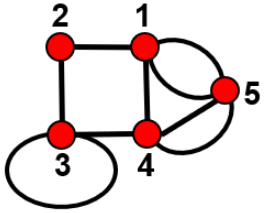	(8x**1**, 1x**2**, 2x$\mathbf{2}^1$, 2x$\mathbf{2}^2$) **f** 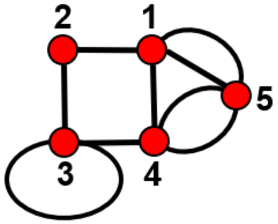	(8x**1**, 1x**2**, 2x$\mathbf{2}^1$, 2x$\mathbf{2}^2$) **g** 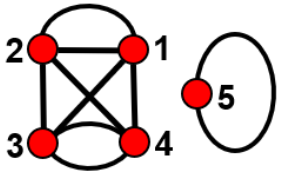	(8x**1**, 1x**2**, 2x$\mathbf{2}^1$, 2x$\mathbf{2}^2$) **h** 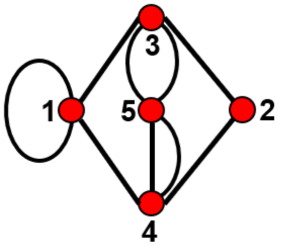
	(8x**1**, 1x**2**, 4x$\mathbf{2}^2$) **a** 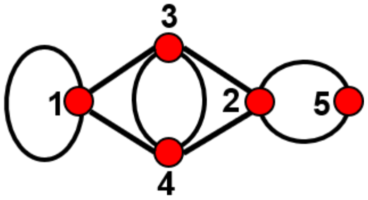	(8x**1**, 1x**2**, 4x$\mathbf{2}^2$) **b** 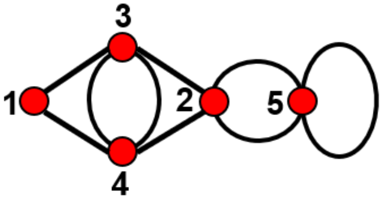	(8x**1**, 1x**2**, 4x$\mathbf{2}^2$) **c** 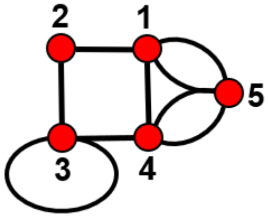	(8x**1**, 1x**2**, 4x$\mathbf{2}^2$) **d** 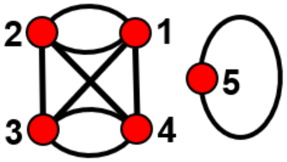

	(8x**1**, 1x**2**, 4x$\mathbf{2}^{2}$) **e**	(6x**1**, 4x**2**, 2x$\mathbf{2}^{1}$) **a**	(6x**1**, 4x**2**, 2x$\mathbf{2}^{1}$) **b**	(6x**1**, 4x**2**, 2x$\mathbf{2}^{1}$) **c**
	(6x**1**, 4x**2**, 2x$\mathbf{2}^{2}$) **a**	(6x**1**, 4x**2**, 2x$\mathbf{2}^{2}$) **b**	(6x**1**, 4x**2**, 2x$\mathbf{2}^{2}$) **c**	(6x**1**, 2x**2**, 4x$\mathbf{2}^{1}$) **a**
	(6x**1**, 2x**2**, 4x$\mathbf{2}^{1}$) **b**	(6x**1**, 2x**2**, 4x$\mathbf{2}^{1}$) **c**	(6x**1**, 2x**2**, 4x$\mathbf{2}^{1}$) **d**	(6x**1**, 2x**2**, 4x$\mathbf{2}^{1}$) **e**

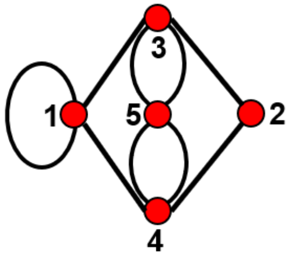

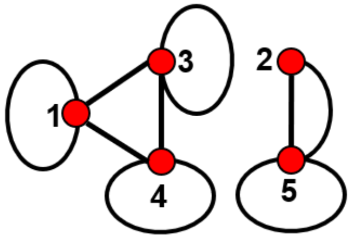

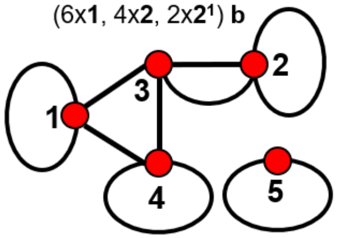

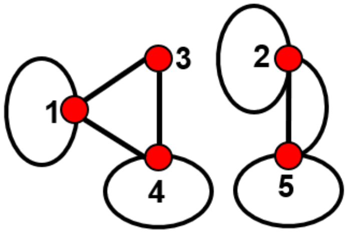

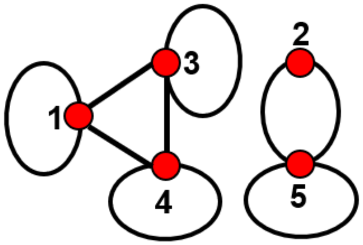

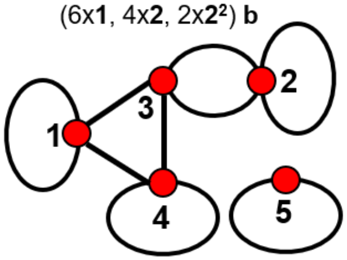

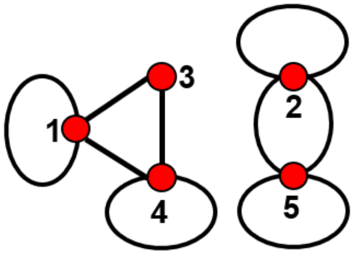

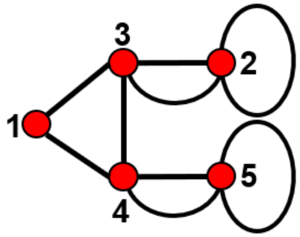

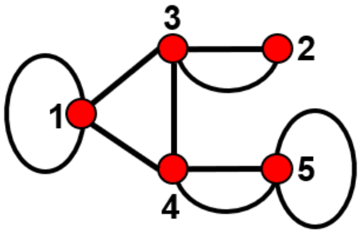

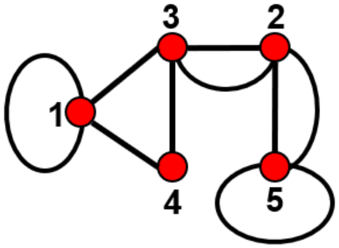

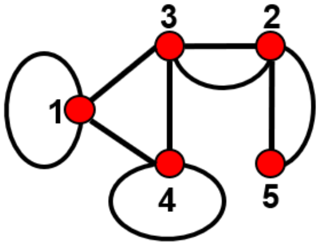

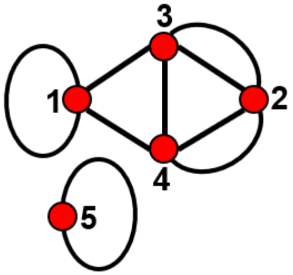

<table>
<tr><td></td><td>(6x1, 2x2, 2x$\mathbf{2}^1$, 2x$\mathbf{2}^2$) a
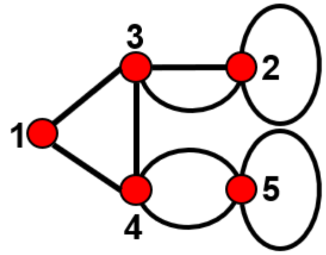</td><td>(6x1, 2x2, 2x$\mathbf{2}^1$, 2x$\mathbf{2}^2$) b
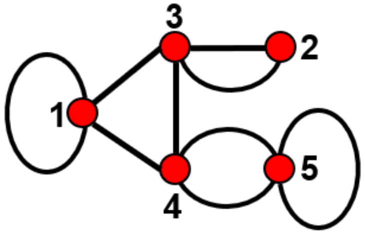</td><td>(6x1, 2x2, 2x$\mathbf{2}^1$, 2x$\mathbf{2}^2$) c
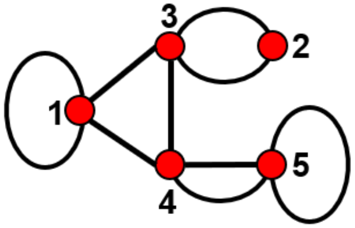</td><td>(6x1, 2x2, 2x$\mathbf{2}^1$, 2x$\mathbf{2}^2$) d
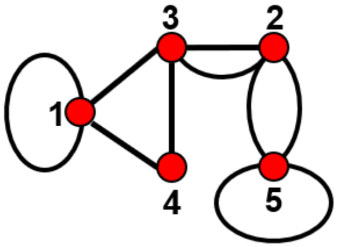</td></tr>
<tr><td></td><td>(6x1, 2x2, 2x$\mathbf{2}^1$, 2x$\mathbf{2}^2$) e
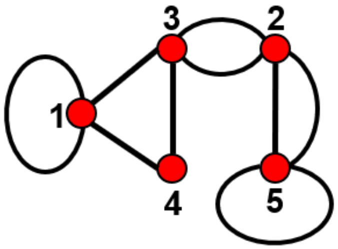</td><td>(6x1, 2x2, 2x$\mathbf{2}^1$, 2x$\mathbf{2}^2$) f
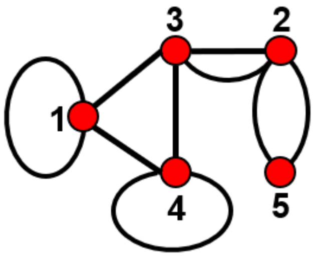</td><td>(6x1, 2x2, 2x$\mathbf{2}^1$, 2x$\mathbf{2}^2$) g
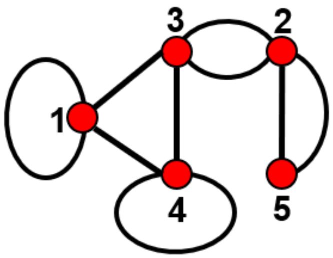</td><td>(6x1, 2x2, 2x$\mathbf{2}^1$, 2x$\mathbf{2}^2$) h
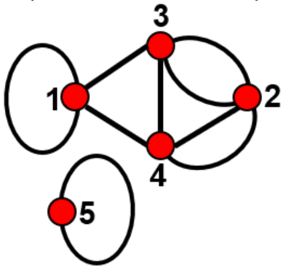</td></tr>
<tr><td></td><td>(6x1, 2x2, 4x$\mathbf{2}^2$) a
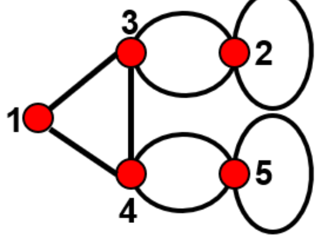</td><td>(6x1, 2x2, 4x$\mathbf{2}^2$) b
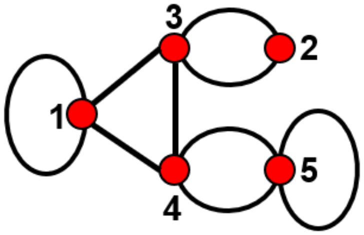</td><td>(6x1, 2x2, 4x$\mathbf{2}^2$) c
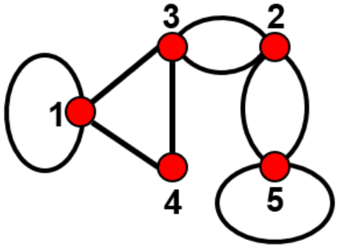</td><td>(6x1, 2x2, 4x$\mathbf{2}^2$) d
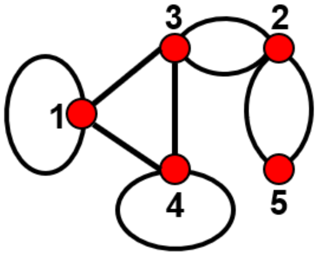</td></tr>
</table>

	(6x**1**, 2x**2**, 4x**2**^{2}) **e** 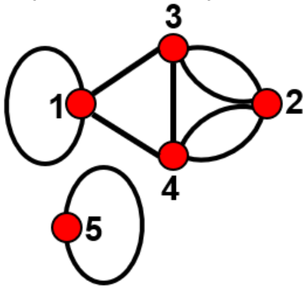	(6x**1**, 6x**2**^{1}) **a** 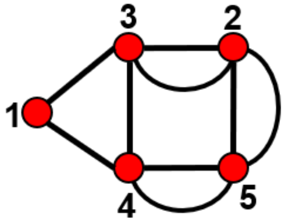	(6x**1**, 6x**2**^{1}) **b** 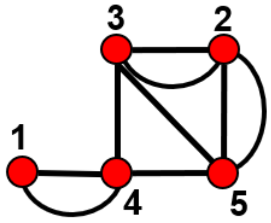	(6x**1**, 4x**2**^{1}, 2x**2**^{2}) **a** 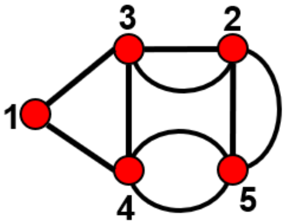
	(6x**1**, 4x**2**^{1}, 2x**2**^{2}) **b** 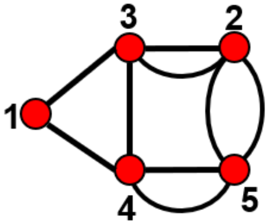	(6x**1**, 4x**2**^{1}, 2x**2**^{2}) **c** 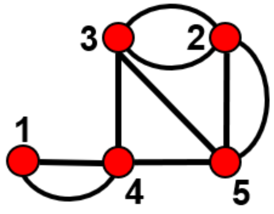	(6x**1**, 4x**2**^{1}, 2x**2**^{2}) **d** 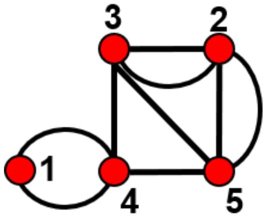	(6x**1**, 2x**2**^{1}, 4x**2**^{2}) **a** 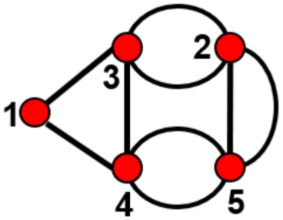
	(6x**1**, 2x**2**^{1}, 4x**2**^{2}) **b** 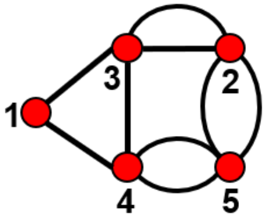	(6x**1**, 2x**2**^{1}, 4x**2**^{2}) **c** 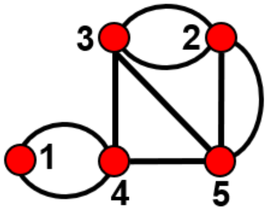	(6x**1**, 2x**2**^{1}, 4x**2**^{2}) **d** 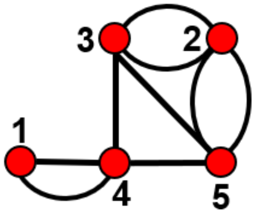	(6x**1**, 6x**2**^{2}) **a** 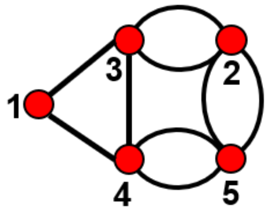

	(6x**1**, 6x**2**^{2}) **b**<br>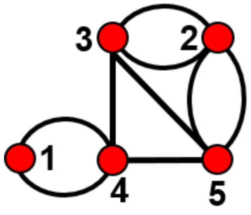	(5x**2**, 4x**2**^{1})<br>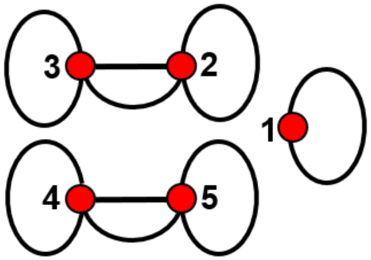	(5x**2**, 2x**2**^{1}, 2x**2**^{2})<br>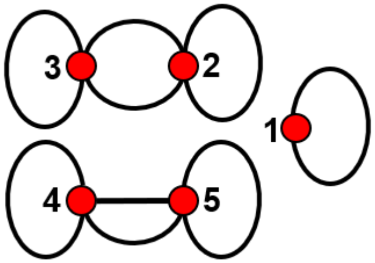	(5x**2**, 4x**2**^{2})<br>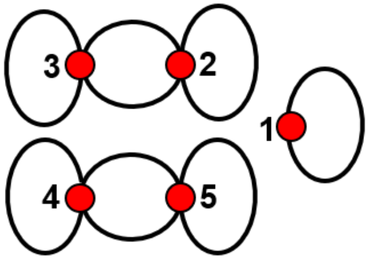
	(3x**2**, 6x**2**^{1}) **a**<br>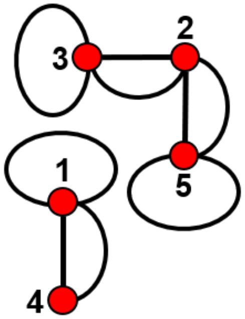	(3x**2**, 6x**2**^{1}) **b**<br>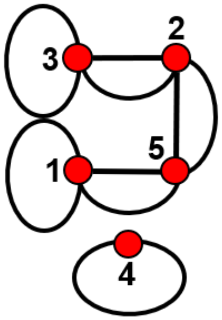	(3x**2**, 6x**2**^{1}) **c**<br>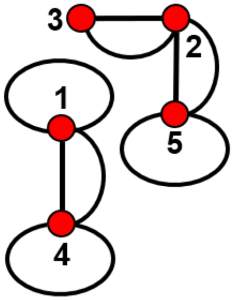	(3x**2**, 4x**2**^{1}, 2x**2**^{2}) **a**<br>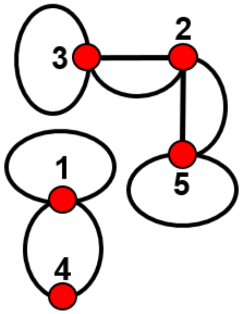
	(3x**2**, 4x**2**^{1}, 2x**2**^{2}) **b**<br>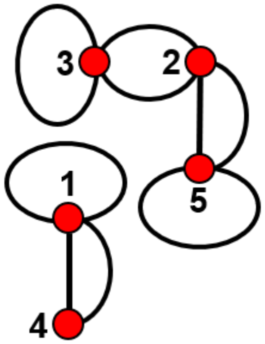	(3x**2**, 4x**2**^{1}, 2x**2**^{2}) **c**<br>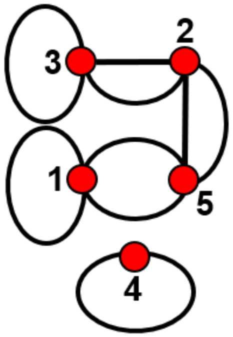	(3x**2**, 4x**2**^{1}, 2x**2**^{2}) **d**<br>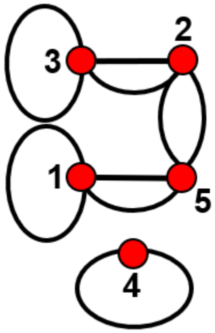	(3x**2**, 4x**2**^{1}, 2x**2**^{2}) **e**<br>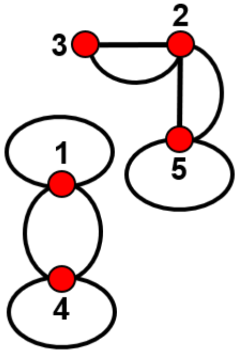

	(3x**2**, 4x**2**^{1}, 2x**2**^{2}) **f** 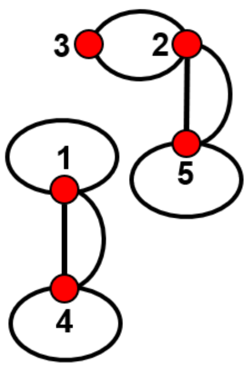	(3x**2**, 4x**2**^{1}, 2x**2**^{2}) **g** 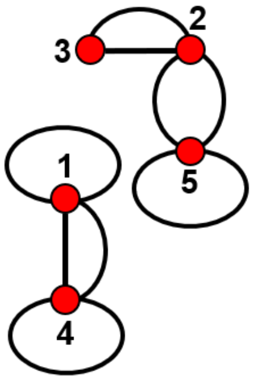	(3x**2**, 2x**2**^{1}, 4x**2**^{2}) **a** 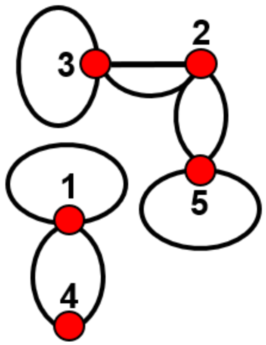	(3x**2**, 2x**2**^{1}, 4x**2**^{2}) **b** 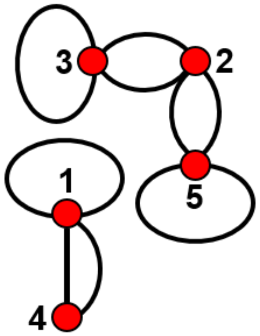
	(3x**2**, 2x**2**^{1}, 4x**2**^{2}) **c** 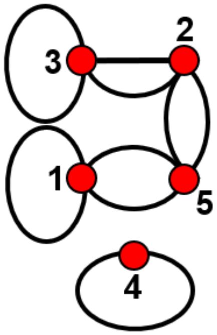	(3x**2**, 2x**2**^{1}, 4x**2**^{2}) **d** 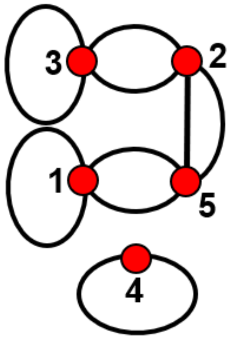	(3x**2**, 2x**2**^{1}, 4x**2**^{2}) **e** 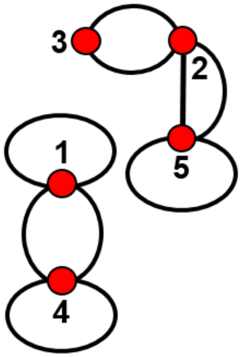	(3x**2**, 2x**2**^{1}, 4x**2**^{2}) **f** 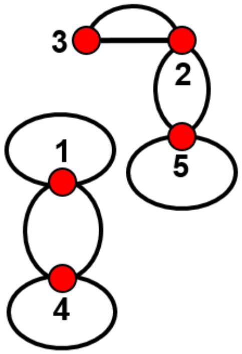

(3x$\mathbf{2}$, 2x$\mathbf{2}^1$, 4x$\mathbf{2}^2$) **g**

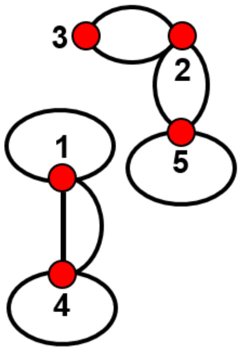

(3x$\mathbf{2}$, 6x$\mathbf{2}^2$) **a**

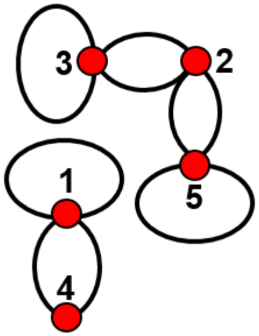

(3x$\mathbf{2}$, 6x$\mathbf{2}^2$) **b**

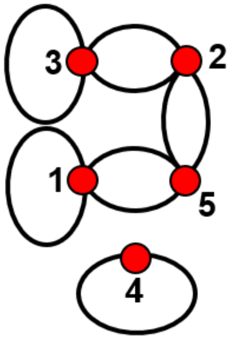

(3x$\mathbf{2}$, 6x$\mathbf{2}^2$) **c**

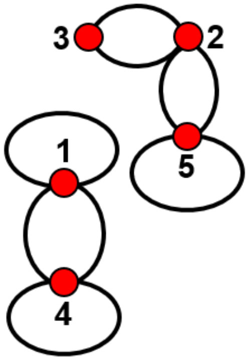

(1x$\mathbf{2}$, 8x$\mathbf{2}^1$) **a**

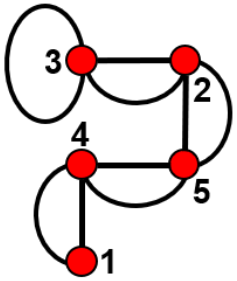

(1x$\mathbf{2}$, 8x$\mathbf{2}^1$) **b**

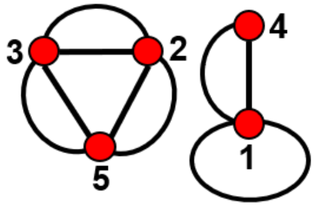

(1x$\mathbf{2}$, 8x$\mathbf{2}^1$) **c**

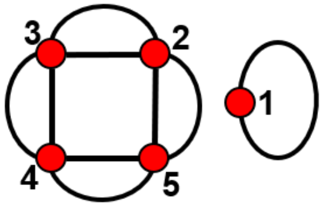

(1x$\mathbf{2}$, 6x$\mathbf{2}^1$, 2x$\mathbf{2}^2$) **a**

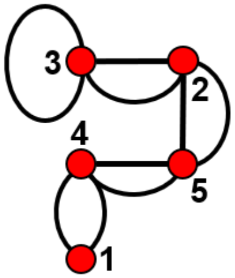

(1x$\mathbf{2}$, 6x$\mathbf{2}^1$, 2x$\mathbf{2}^2$) **b**

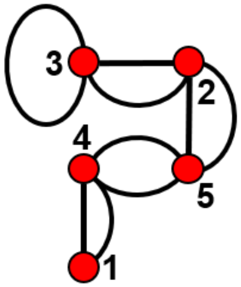

(1x$\mathbf{2}$, 6x$\mathbf{2}^1$, 2x$\mathbf{2}^2$) **c**

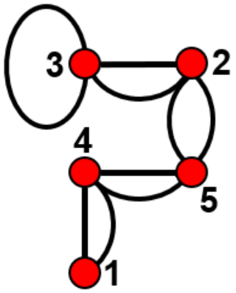

(1x$\mathbf{2}$, 6x$\mathbf{2}^1$, 2x$\mathbf{2}^2$) **d**

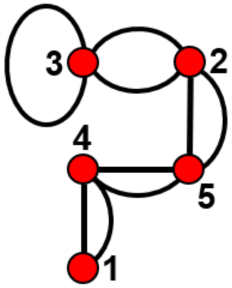

(1x$\mathbf{2}$, 6x$\mathbf{2}^1$, 2x$\mathbf{2}^2$) **e**

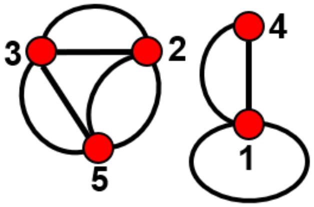

(1x**2**, 6x$\mathbf{2}^1$, 2x$\mathbf{2}^2$) **f**

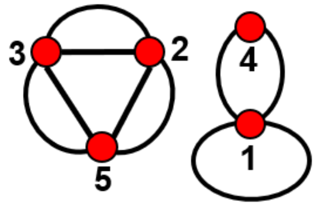

(1x**2**, 6x$\mathbf{2}^1$, 2x$\mathbf{2}^2$) **g**

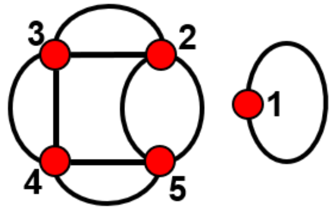

(1x**2**, 4x$\mathbf{2}^1$, 4x$\mathbf{2}^2$) **a**

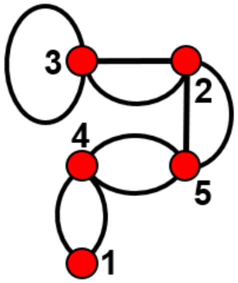

(1x**2**, 4x$\mathbf{2}^1$, 4x$\mathbf{2}^2$) **b**

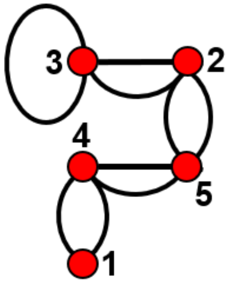

(1x**2**, 4x$\mathbf{2}^1$, 4x$\mathbf{2}^2$) **c**

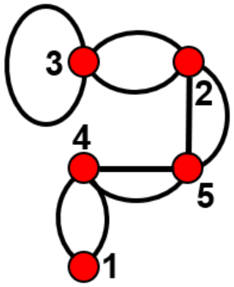

(1x**2**, 4x$\mathbf{2}^1$, 4x$\mathbf{2}^2$) **d**

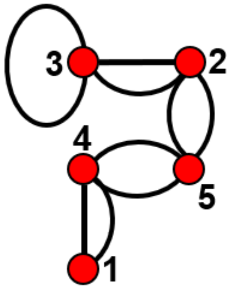

(1x**2**, 4x$\mathbf{2}^1$, 4x$\mathbf{2}^2$) **e**

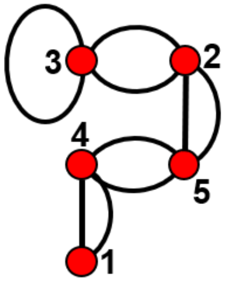

(1x**2**, 4x$\mathbf{2}^1$, 4x$\mathbf{2}^2$) **f**

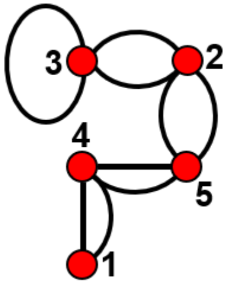

(1x**2**, 4x$\mathbf{2}^1$, 4x$\mathbf{2}^2$) **g**

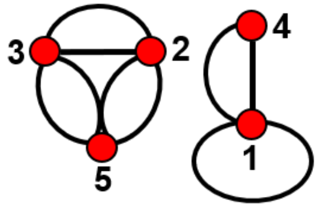

(1x**2**, 4x$\mathbf{2}^1$, 4x$\mathbf{2}^2$) **h**

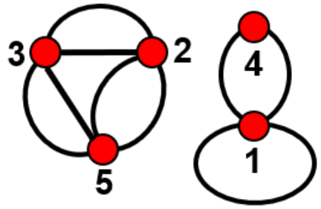

(1x**2**, 4x$\mathbf{2}^1$, 4x$\mathbf{2}^2$) **i**

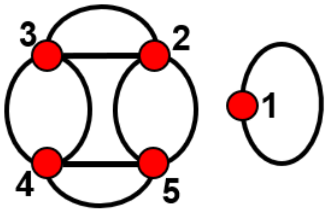

(1x**2**, 4x$\mathbf{2}^1$, 4x$\mathbf{2}^2$) **j**

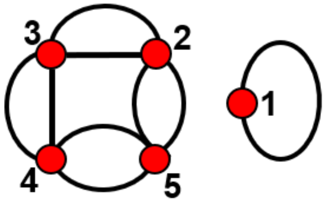

<table>
<tr><td></td><td>(1x2, 2x2^1, 6x2^2) a
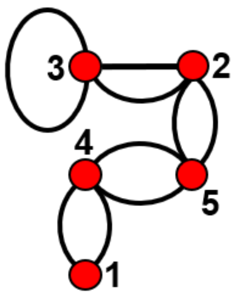</td><td>(1x2, 2x2^1, 6x2^2) b
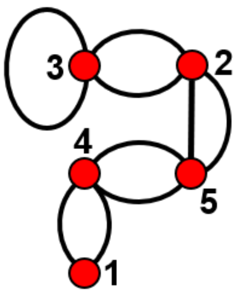</td><td>(1x2, 2x2^1, 6x2^2) c
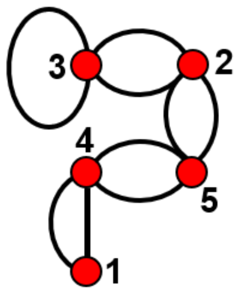</td><td>(1x2, 2x2^1, 6x2^2) d
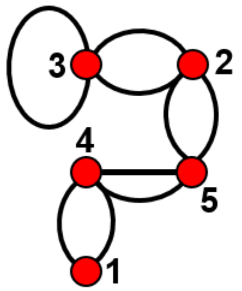</td></tr>
<tr><td></td><td>(1x2, 2x2^1, 6x2^2) e
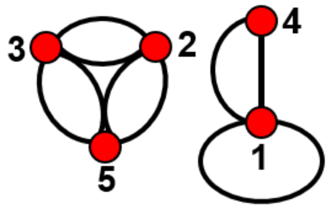</td><td>(1x2, 2x2^1, 6x2^2) f
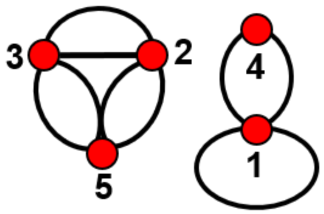</td><td>(1x2, 2x2^1, 6x2^2) g
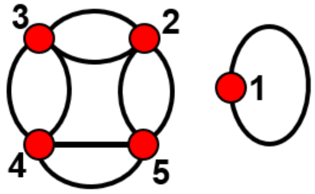</td><td>(1x2, 8x2^2) a
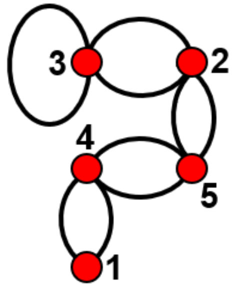</td></tr>
<tr><td></td><td>(1x2, 8x2^2) b
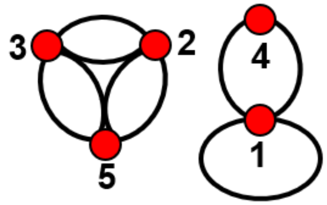</td><td>(1x2, 8x2^2) c
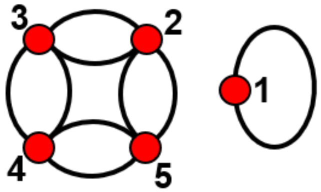</td><td></td><td></td></tr>
<tr><td>$^2V_1{}^4V_5$</td><td colspan="4">NG</td></tr>
<tr><td>$^2V_1{}^4V_6$</td><td colspan="4">NG</td></tr>
<tr><td>$^2V_1{}^4V_7$</td><td colspan="4">NG</td></tr>
</table>

$^2V_2{}^4V_1$	(6x**1**, 1x**2**)<br>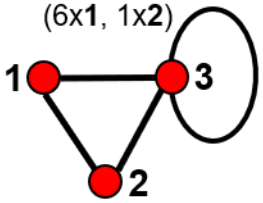	(2x**2**, 2x$\mathbf{2}^1$)<br>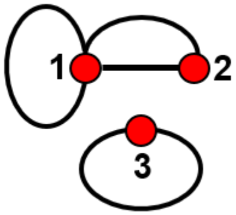	(2x**2**, 2x$\mathbf{2}^2$)<br>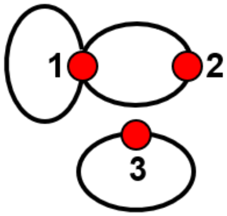	(4x$\mathbf{2}^1$)<br>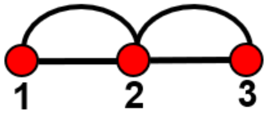
	(2x$\mathbf{2}^1$, 2x$\mathbf{2}^2$)<br>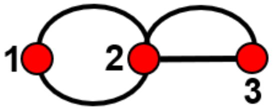	(4x$\mathbf{2}^2$)<br>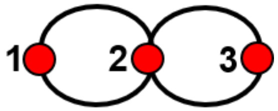		
$^2V_2{}^4V_2$	(8x**1**, 2x**2**) **a**<br>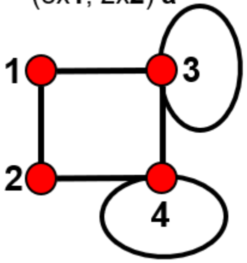	(8x**1**, 2x**2**) **b**<br>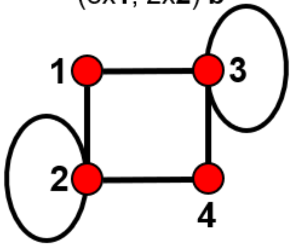	(8x**1**, 2x$\mathbf{2}^1$)<br>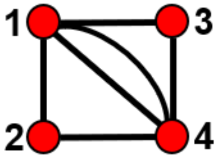	(8x**1**, 2x$\mathbf{2}^2$)<br>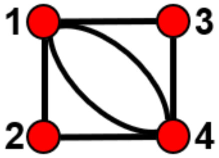
	(6x**1**, 3x**2**)<br>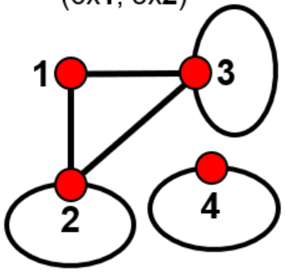	(6x**1**, 1x**2**, 2x$\mathbf{2}^1$) **a**<br>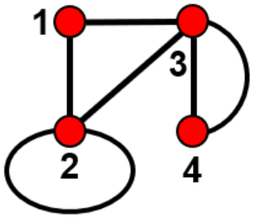	(6x**1**, 1x**2**, 2x$\mathbf{2}^1$) **b**<br>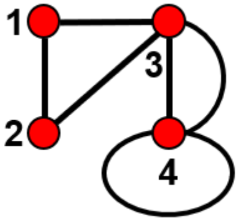	(6x**1**, 1x**2**, 2x$\mathbf{2}^2$) **a**<br>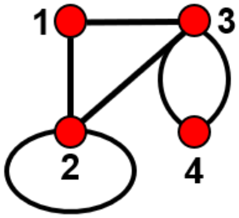

(6x**1**, 1x**2**, 2x$\mathbf{2}^{2}$) **b**

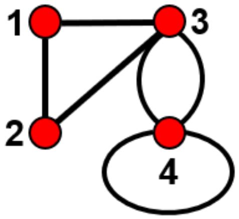

(4x**2**, 2x$\mathbf{2}^{1}$)

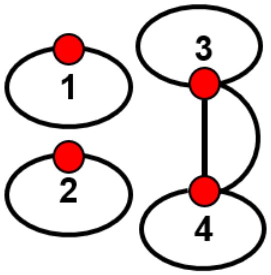

(4x**2**, 2x$\mathbf{2}^{2}$)

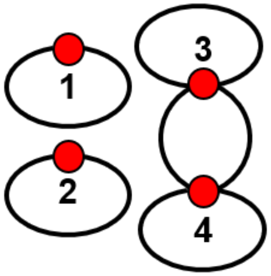

(2x**2**, 4x$\mathbf{2}^{1}$) **a**

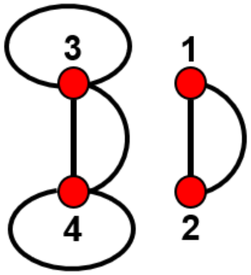

(2x**2**, 4x$\mathbf{2}^{1}$) **b**

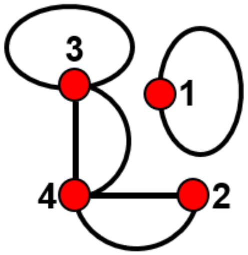

(2x**2**, 4x$\mathbf{2}^{1}$) **c**

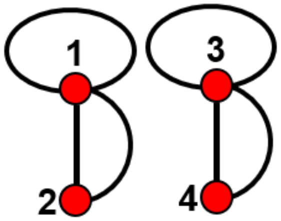

(2x**2**, 2x$\mathbf{2}^{1}$, 2x$\mathbf{2}^{2}$) **a**

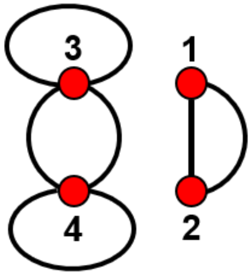

(2x**2**, 2x$\mathbf{2}^{1}$, 2x$\mathbf{2}^{2}$) **b**

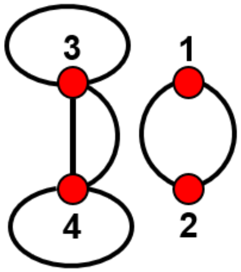

(2x**2**, 2x$\mathbf{2}^{1}$, 2x$\mathbf{2}^{2}$) **c**

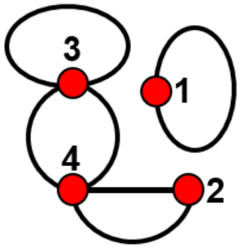

(2x**2**, 2x$\mathbf{2}^{1}$, 2x$\mathbf{2}^{2}$) **d**

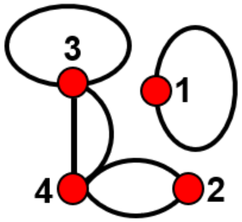

(2x**2**, 2x$\mathbf{2}^{1}$, 2x$\mathbf{2}^{2}$) **e**

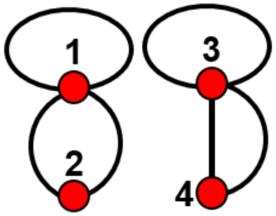

(2x**2**, 4x$\mathbf{2}^{2}$) **a**

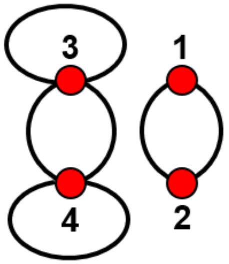

	(2x**2**, 4x$\mathbf{2}^2$) **b** 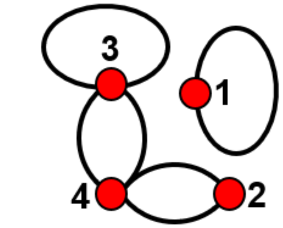	(2x**2**, 4x$\mathbf{2}^2$) **c** 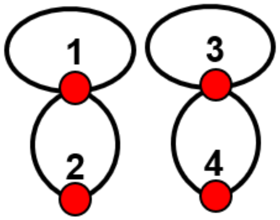	(6x$\mathbf{2}^1$) 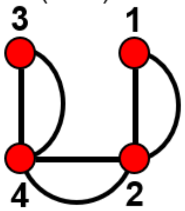	(4x$\mathbf{2}^1$, 2x$\mathbf{2}^2$) **a** 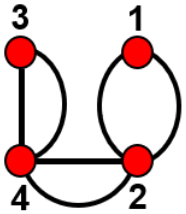
	(4x$\mathbf{2}^1$, 2x$\mathbf{2}^2$) **b** 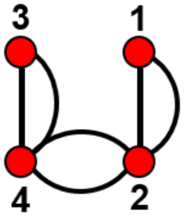	(2x$\mathbf{2}^1$, 4x$\mathbf{2}^2$) **a** 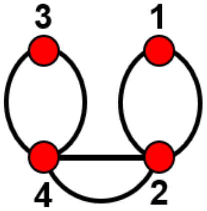	(2x$\mathbf{2}^1$, 4x$\mathbf{2}^2$) **b** 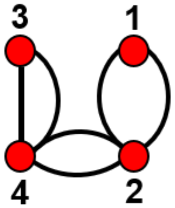	(6x$\mathbf{2}^2$) 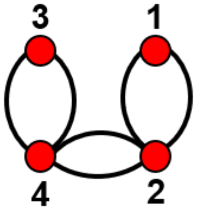
$^2V_2{}^4V_3$	(14x**1**, 1x**2**) 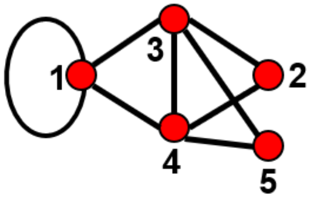	(12x**1**, 2x**2**) **a** 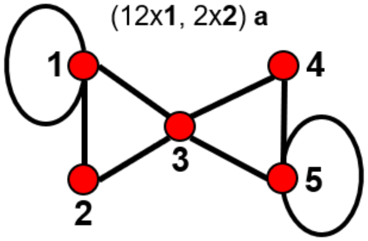	(12x**1**, 2x**2**) **b** 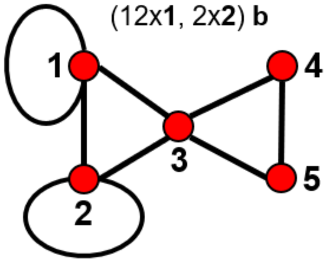	(12x**1**, 2x$\mathbf{2}^1$) 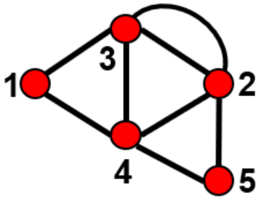
	(12x**1**, 2x$\mathbf{2}^2$) 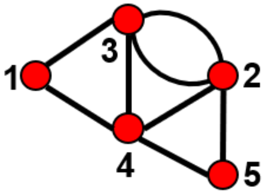	(10x**1**, 3x**2**) **a** 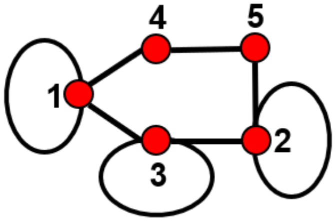	(10x**1**, 3x**2**) **b** 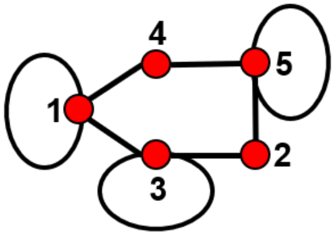	(10x**1**, 1x**2**, 2x$\mathbf{2}^1$) **a** 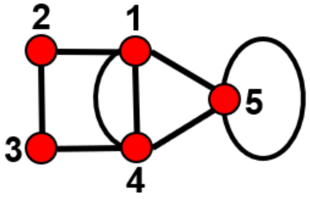

	(10x**1**, 1x**2**, 2x$\mathbf{2}^1$) **b** 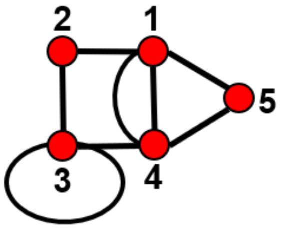	(8x**1**, 4x**2**) 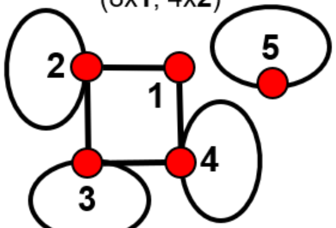	(8x**1**, 2x**2**, 2x$\mathbf{2}^1$) **a** 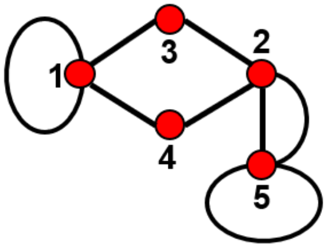	(8x**1**, 2x**2**, 2x$\mathbf{2}^1$) **b** 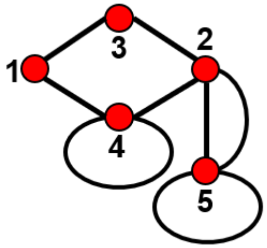
	(8x**1**, 2x**2**, 2x$\mathbf{2}^1$) **c** 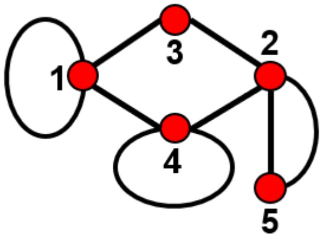	(8x**1**, 2x**2**, 2x$\mathbf{2}^1$) **d** 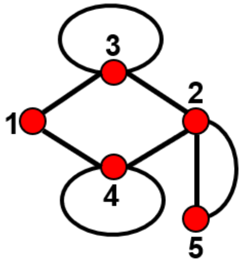	(8x**1**, 2x**2**, 2x$\mathbf{2}^1$) **e** 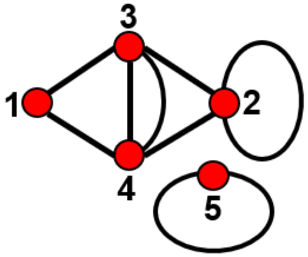	(8x**1**, 2x**2**, 2x$\mathbf{2}^2$) **a** 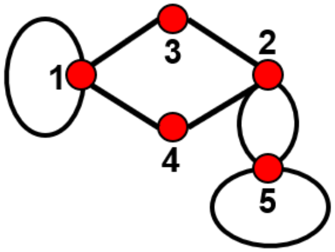
	(8x**1**, 2x**2**, 2x$\mathbf{2}^2$) **b** 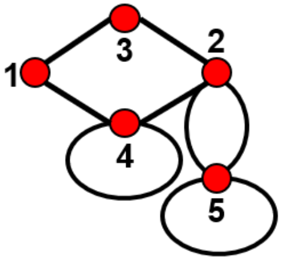	(8x**1**, 2x**2**, 2x$\mathbf{2}^2$) **c** 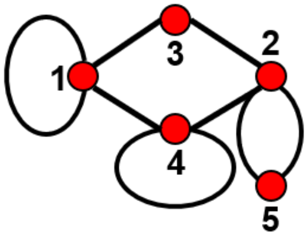	(8x**1**, 2x**2**, 2x$\mathbf{2}^2$) **d** 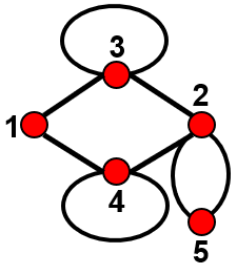	(8x**1**, 2x**2**, 2x$\mathbf{2}^2$) **e** 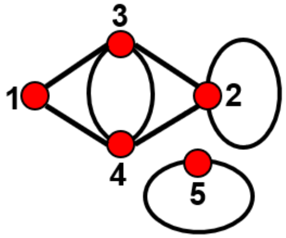

(8x**1**, 4x$\mathbf{2}^{1}$) **a**

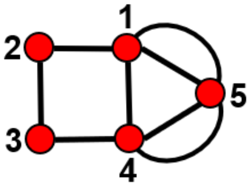

(8x**1**, 4x$\mathbf{2}^{1}$) **b**

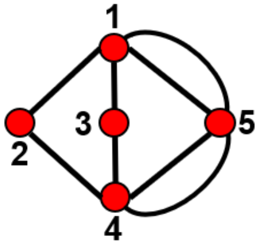

(8x**1**, 4x$\mathbf{2}^{1}$) **c**

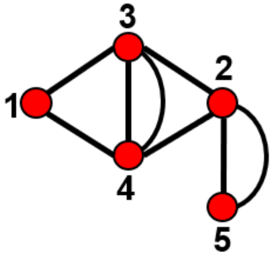

(8x**1**, 2x$\mathbf{2}^{1}$, 2x$\mathbf{2}^{2}$) **a**

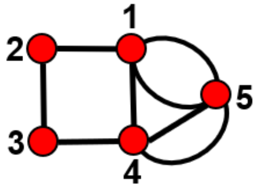

(8x**1**, 2x$\mathbf{2}^{1}$, 2x$\mathbf{2}^{2}$) **b**

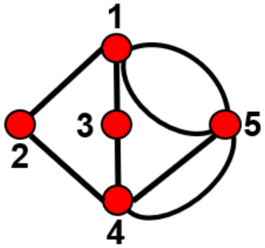

(8x**1**, 2x$\mathbf{2}^{1}$, 2x$\mathbf{2}^{2}$) **c**

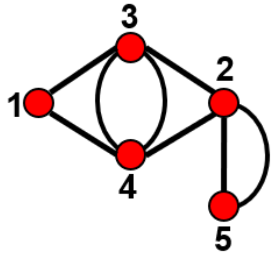

(8x**1**, 2x$\mathbf{2}^{1}$, 2x$\mathbf{2}^{2}$) **d**

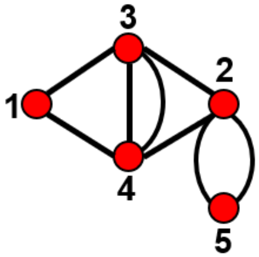

(8x**1**, 4x$\mathbf{2}^{2}$) **a**

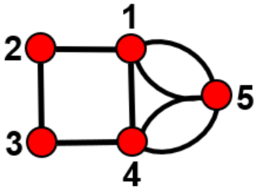

(8x**1**, 4x$\mathbf{2}^{2}$) **b**

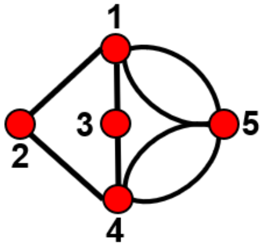

(8x**1**, 4x$\mathbf{2}^{2}$) **c**

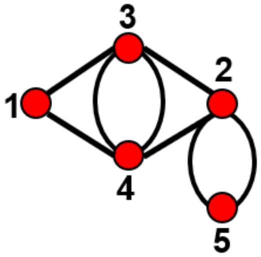

(6x**1**, 5x**2**)

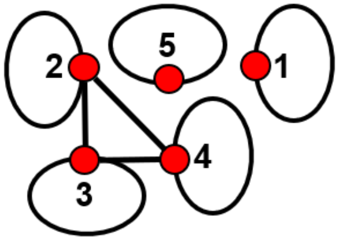

(6x**1**, 3x**2**, 2x$\mathbf{2}^{1}$) **a**

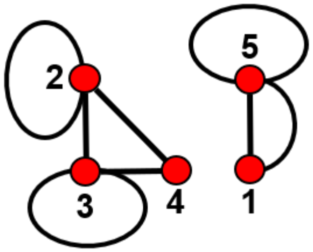

	(6x**1**, 3x**2**, 2x**2**^{1}) **b**<br>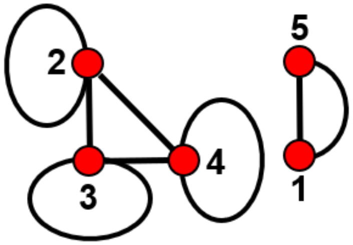	(6x**1**, 3x**2**, 2x**2**^{1}) **c**<br>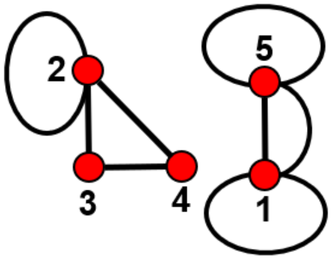	(6x**1**, 3x**2**, 2x**2**^{1}) **d**<br>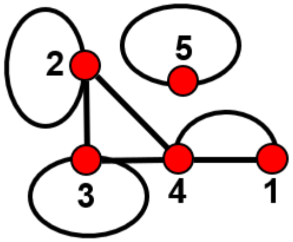	(6x**1**, 3x**2**, 2x**2**^{1}) **e**<br>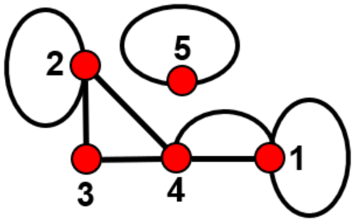
	(6x**1**, 3x**2**, 2x**2**^{2}) **a**<br>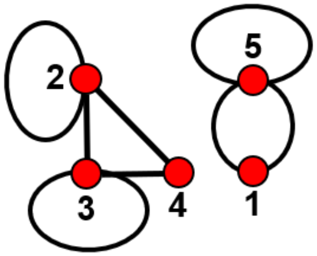	(6x**1**, 3x**2**, 2x**2**^{2}) **b**<br>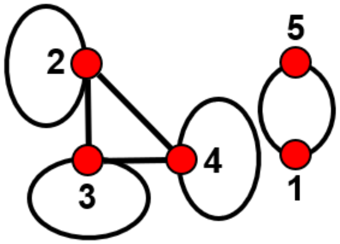	(6x**1**, 3x**2**, 2x**2**^{2}) **c**<br>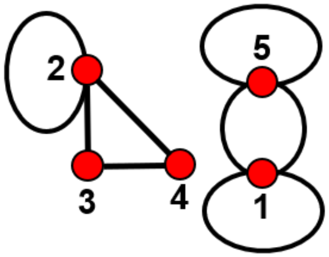	(6x**1**, 3x**2**, 2x**2**^{2}) **d**<br>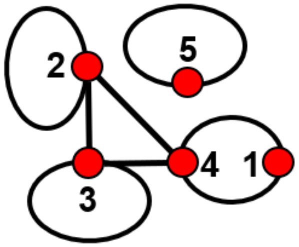
	(6x**1**, 3x**2**, 2x**2**^{2}) **e**<br>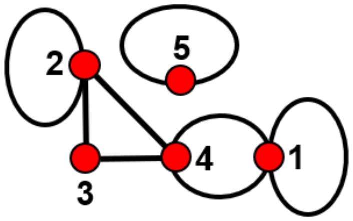	(6x**1**, 1x**2**, 4x**2**^{1}) **a**<br>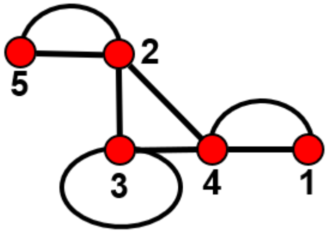	(6x**1**, 1x**2**, 4x**2**^{1}) **b**<br>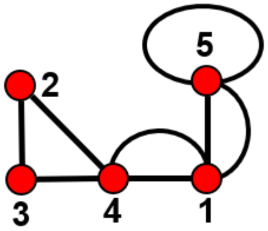	(6x**1**, 1x**2**, 4x**2**^{1}) **c**<br>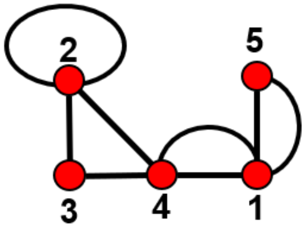

	(6x**1**, 1x**2**, 4x$\mathbf{2}^1$) **d** 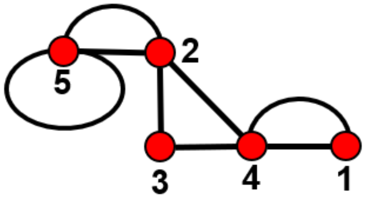	(6x**1**, 1x**2**, 4x$\mathbf{2}^1$) **e** 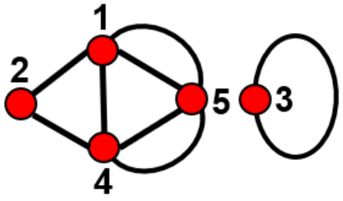	(6x**1**, 1x**2**, 2x$\mathbf{2}^1$, 2x$\mathbf{2}^2$) **a** 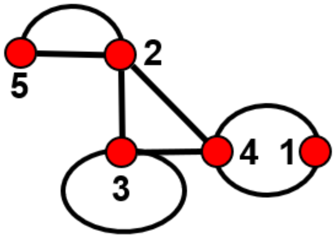	(6x**1**, 1x**2**, 2x$\mathbf{2}^1$, 2x$\mathbf{2}^2$) **b** 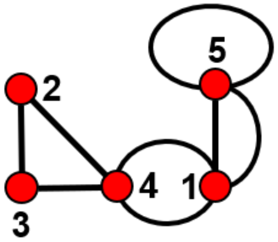
	(6x**1**, 1x**2**, 2x$\mathbf{2}^1$, 2x$\mathbf{2}^2$) **c** 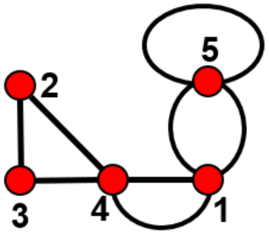	(6x**1**, 1x**2**, 2x$\mathbf{2}^1$, 2x$\mathbf{2}^2$) **d** 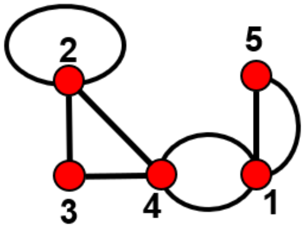	(6x**1**, 1x**2**, 2x$\mathbf{2}^1$, 2x$\mathbf{2}^2$) **e** 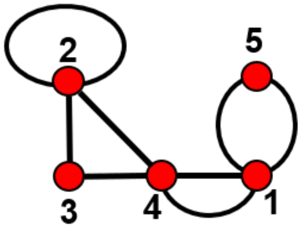	(6x**1**, 1x**2**, 2x$\mathbf{2}^1$, 2x$\mathbf{2}^2$) **f** 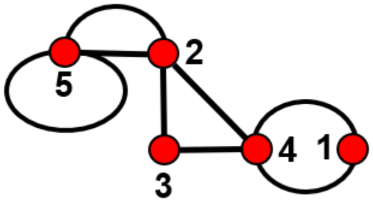
	(6x**1**, 1x**2**, 2x$\mathbf{2}^1$, 2x$\mathbf{2}^2$) **g** 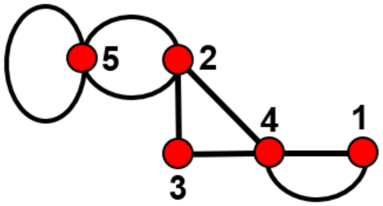	(6x**1**, 1x**2**, 2x$\mathbf{2}^1$, 2x$\mathbf{2}^2$) **h** 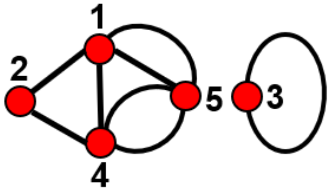	(6x**1**, 1x**2**, 4x$\mathbf{2}^2$) **a** 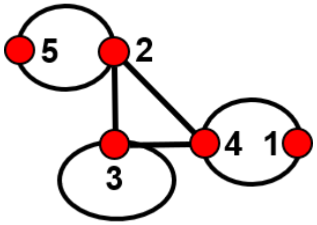	(6x**1**, 1x**2**, 4x$\mathbf{2}^2$) **b** 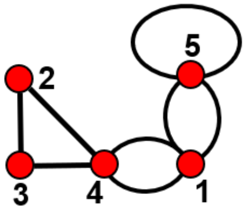

	(6x**1**, 1x**2**, 4x**2**^{2}) **c** 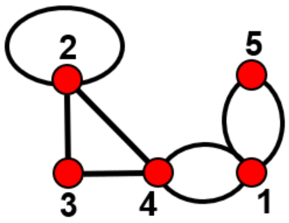	(6x**1**, 1x**2**, 4x**2**^{2}) **d** 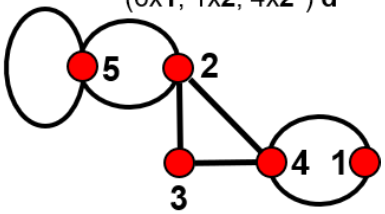	(6x**1**, 1x**2**, 4x**2**^{2}) **e** 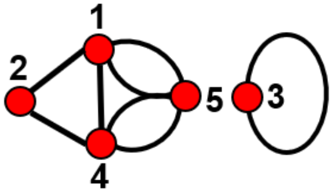	(4x**2**, 4x**2**^{1}) **a** 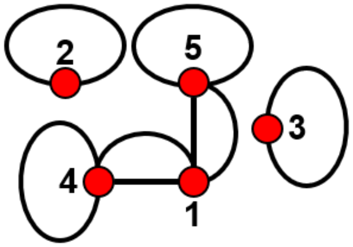
	(4x**2**, 4x**2**^{1}) **b** 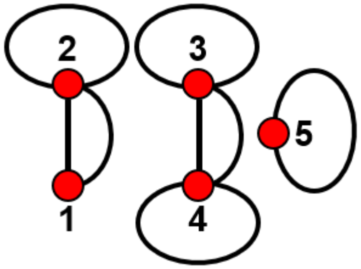	(4x**2**, 2x**2**^{1}, 2x**2**^{2}) **a** 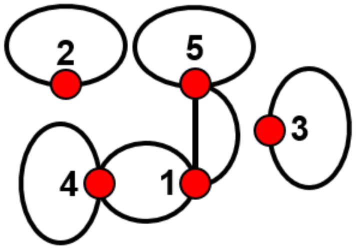	(4x**2**, 2x**2**^{1}, 2x**2**^{2}) **b** 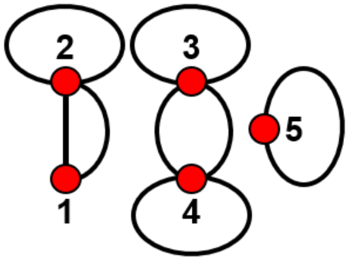	(4x**2**, 2x**2**^{1}, 2x**2**^{2}) **c** 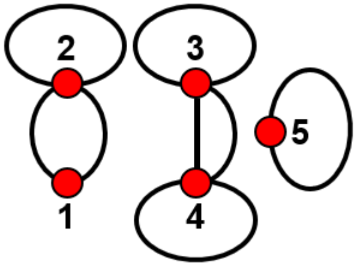
	(4x**2**, 4x**2**^{2}) **a** 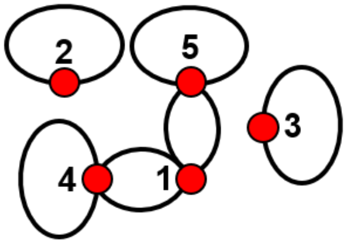	(4x**2**, 4x**2**^{2}) **b** 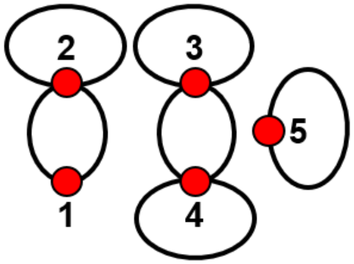	(2x**2**, 6x**2**^{1}) **a** 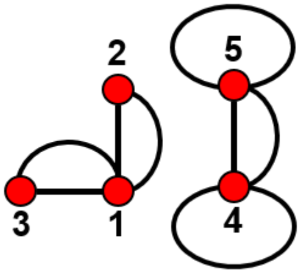	(2x**2**, 6x**2**^{1}) **b** 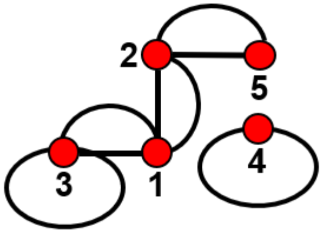

(2x**2**, 6x**2**¹) **c**

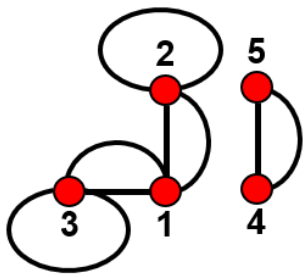

(2x**2**, 6x**2**¹) **d**

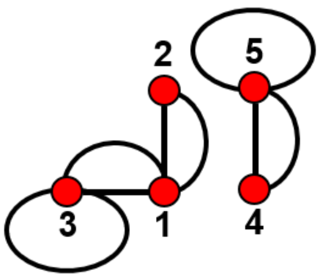

(2x**2**, 6x**2**¹) **e**

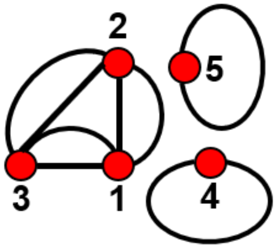

(2x**2**, 4x**2**¹, 2x**2**²) **a**

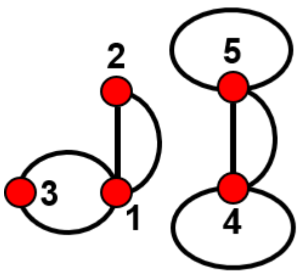

(2x**2**, 4x**2**¹, 2x**2**²) **b**

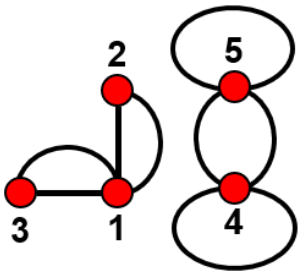

(2x**2**, 4x**2**¹, 2x**2**²) **c**

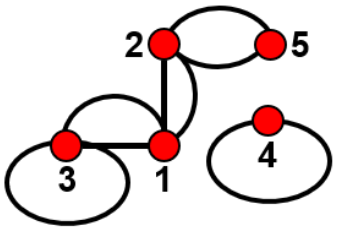

(2x**2**, 4x**2**¹, 2x**2**²) **d**

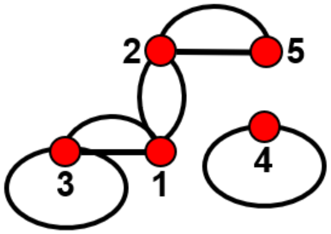

(2x**2**, 4x**2**¹, 2x**2**²) **e**

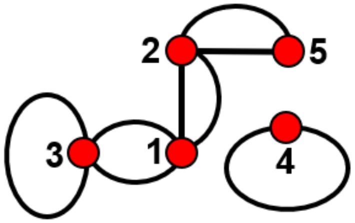

(2x**2**, 4x**2**¹, 2x**2**²) **f**

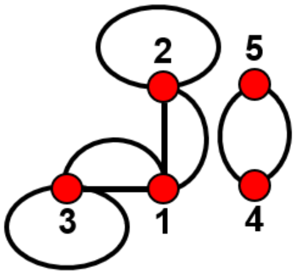

(2x**2**, 4x**2**¹, 2x**2**²) **g**

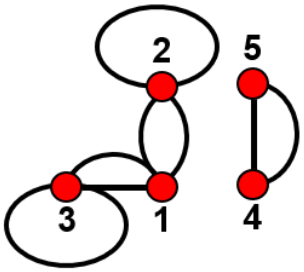

(2x**2**, 4x**2**¹, 2x**2**²) **h**

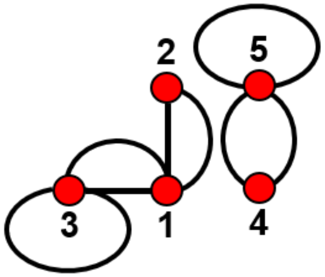

(2x**2**, 4x**2**¹, 2x**2**²) **i**

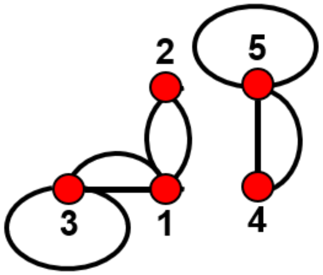

	(2x**2**, 4x**2**^{1}, 2x**2**^{2}) **j** 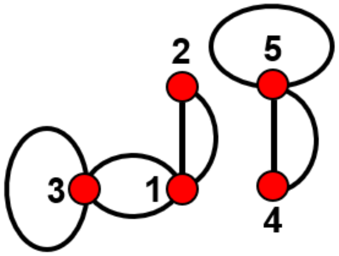	(2x**2**, 4x**2**^{1}, 2x**2**^{2}) **k** 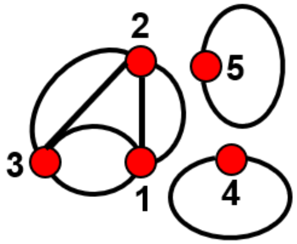	(2x**2**, 2x**2**^{1}, 4x**2**^{2}) **a** 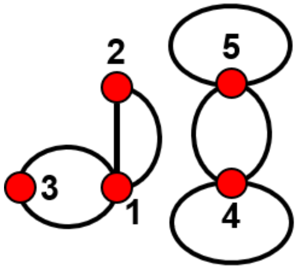	(2x**2**, 2x**2**^{1}, 4x**2**^{2}) **b** 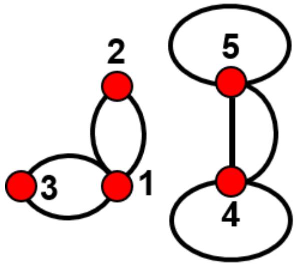
	(2x**2**, 2x**2**^{1}, 4x**2**^{2}) **c** 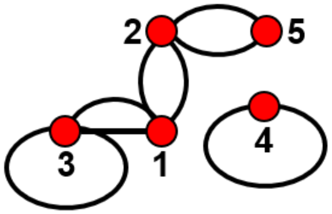	(2x**2**, 2x**2**^{1}, 4x**2**^{2}) **d** 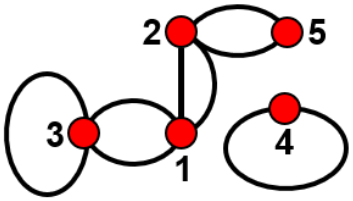	(2x**2**, 2x**2**^{1}, 4x**2**^{2}) **e** 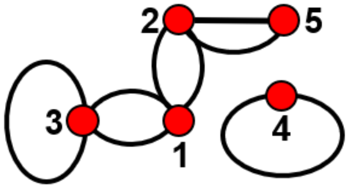	(2x**2**, 2x**2**^{1}, 4x**2**^{2}) **f** 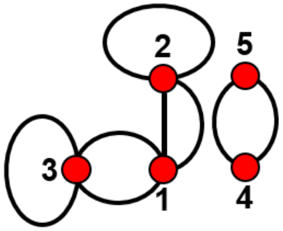
	(2x**2**, 2x**2**^{1}, 4x**2**^{2}) **g** 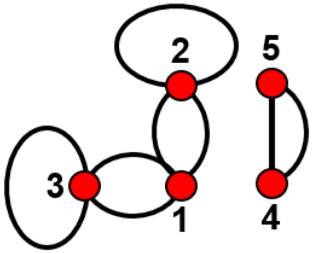	(2x**2**, 2x**2**^{1}, 4x**2**^{2}) **h** 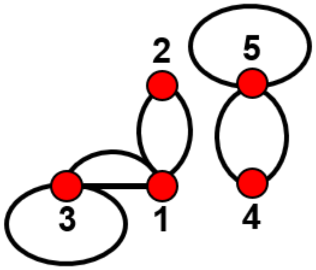	(2x**2**, 2x**2**^{1}, 4x**2**^{2}) **i** 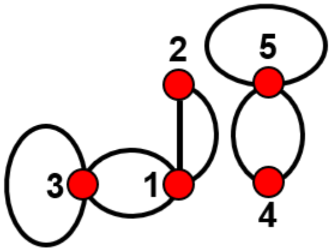	(2x**2**, 2x**2**^{1}, 4x**2**^{2}) **j** 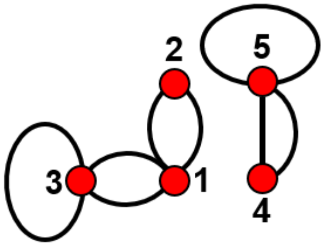

	(2x**2**, 2x$\mathbf{2}^1$, 4x$\mathbf{2}^2$) **k** 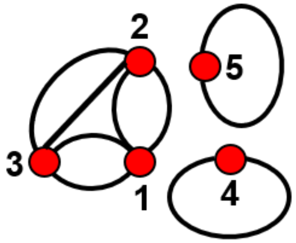	(2x**2**, 6x$\mathbf{2}^2$) **a** 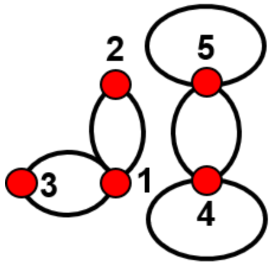	(2x**2**, 6x$\mathbf{2}^2$) **b** 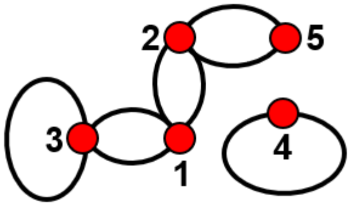	(2x**2**, 6x$\mathbf{2}^2$) **c** 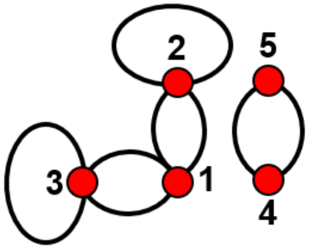
	(2x**2**, 6x$\mathbf{2}^2$) **d** 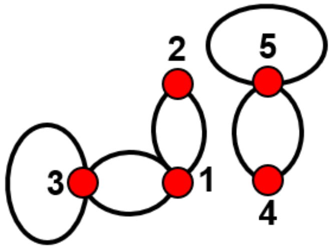	(2x**2**, 6x$\mathbf{2}^2$) **e** 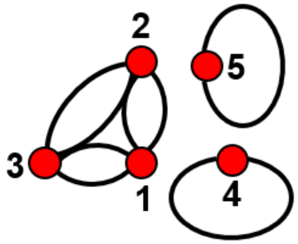	(8x$\mathbf{2}^1$) **a** 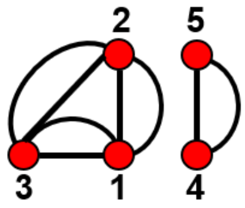	(8x$\mathbf{2}^1$) **b** 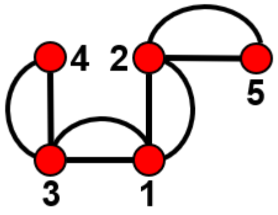
	(6x$\mathbf{2}^1$, 2x$\mathbf{2}^2$) **a** 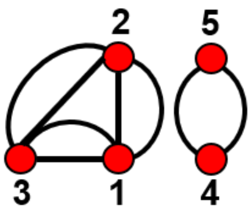	(6x$\mathbf{2}^1$, 2x$\mathbf{2}^2$) **b** 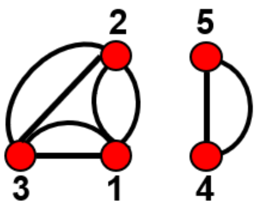	(6x$\mathbf{2}^1$, 2x$\mathbf{2}^2$) **c** 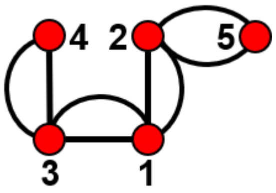	(6x$\mathbf{2}^1$, 2x$\mathbf{2}^2$) **d** 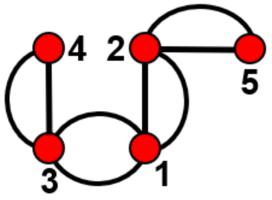
	(4x$\mathbf{2}^1$, 4x$\mathbf{2}^2$) **a** 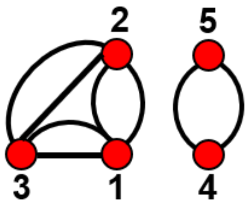	(4x$\mathbf{2}^1$, 4x$\mathbf{2}^2$) **b** 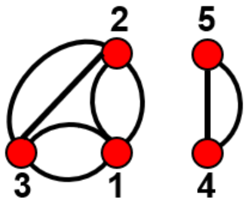	(4x$\mathbf{2}^1$, 4x$\mathbf{2}^2$) **c** 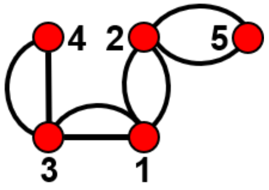	(4x$\mathbf{2}^1$, 4x$\mathbf{2}^2$) **d** 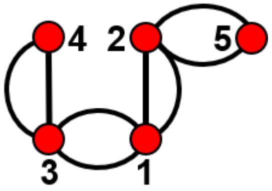

	(4x**2**^{1}, 4x**2**^{2}) **e** 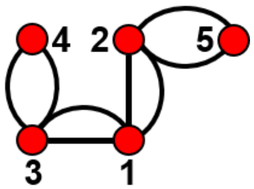	(4x**2**^{1}, 4x**2**^{2}) **f** 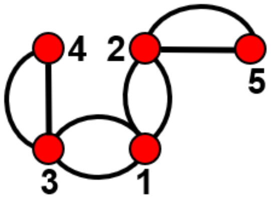	(2x**2**^{1}, 6x**2**^{2}) **a** 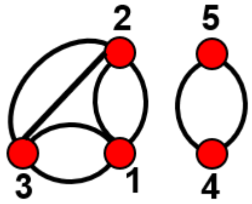	(2x**2**^{1}, 6x**2**^{2}) **b** 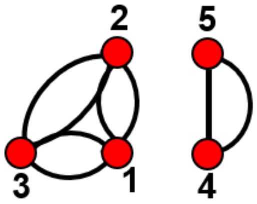
	(2x**2**^{1}, 6x**2**^{2}) **c** 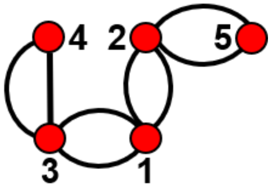	(2x**2**^{1}, 6x**2**^{2}) **d** 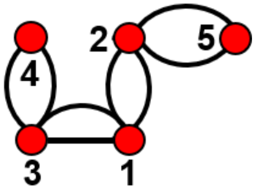	(8x**2**^{2}) **a** 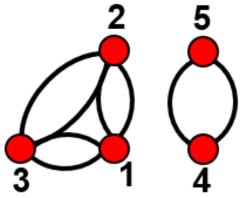	(8x**2**^{2}) **b** 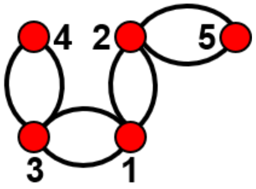
$^2V_2{}^4V_4$	NG			
$^2V_2{}^4V_5$	NG			
$^2V_2{}^4V_6$	NG			
$^2V_3{}^4V_1$	(8x**1**, 1x**2**) 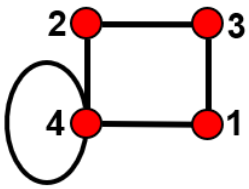	(6x**1**, 2x**2**) 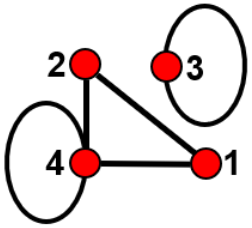	(6x**1**, 2x**2**^{1}) 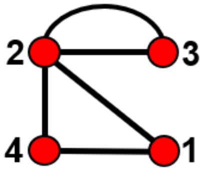	(6x**1**, 2x**2**^{2}) 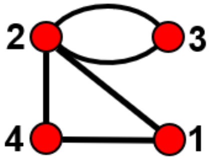

	(3x**2**, 2x**2**^{1})<br>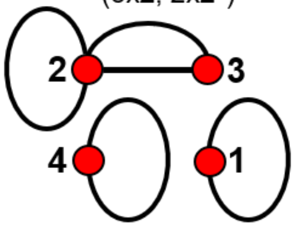	(3x**2**, 2x**2**^{2})<br>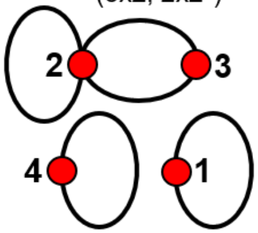	(1x**2**, 4x**2**^{1}) **a**<br>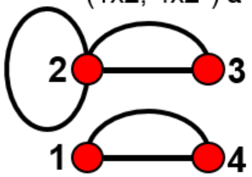	(1x**2**, 4x**2**^{1}) **b**<br>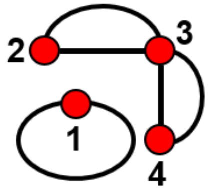
	(1x**2**, 2x**2**^{1}, 2x**2**^{2}) **a**<br>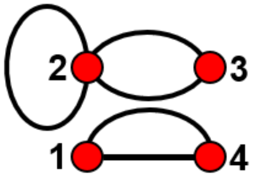	(1x**2**, 2x**2**^{1}, 2x**2**^{2}) **b**<br>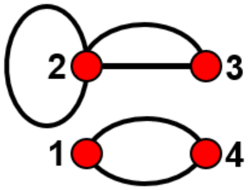	(1x**2**, 2x**2**^{1}, 2x**2**^{2}) **c**<br>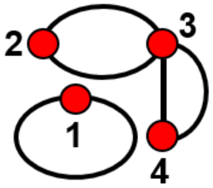	(1x**2**, 4x**2**^{2}) **a**<br>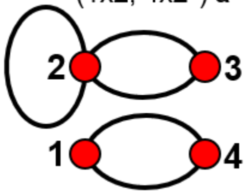
	(1x**2**, 4x**2**^{2}) **b**<br>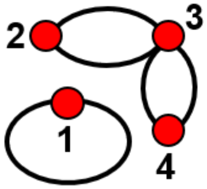			
$^2V_3{}^4V_2$	(14x**1**)<br>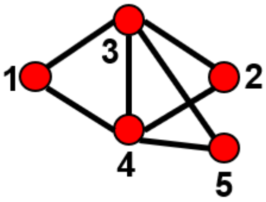	(12x**1**, 1x**2**)<br>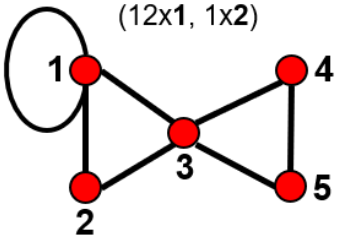	(10x**1**, 2x**2**) **a**<br>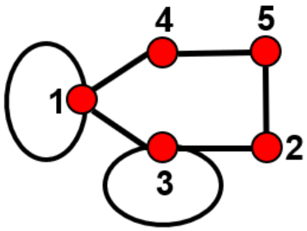	(10x**1**, 2x**2**) **b**<br>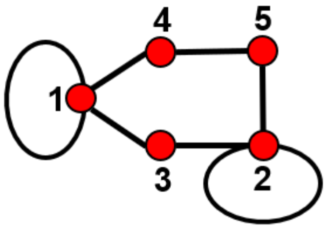

	(10x**1**, 2x**2**^{1})<br>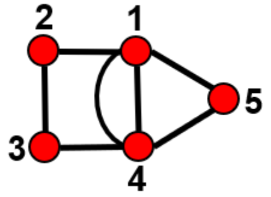	(10x**1**, 2x**2**^{2})<br>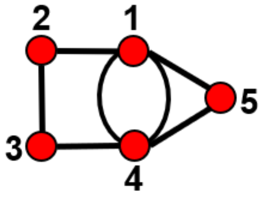	(8x**1**, 3x**2**) **a**<br>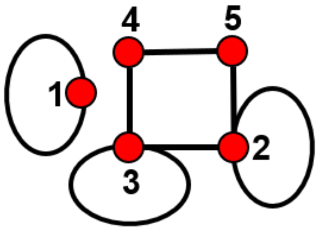	(8x**1**, 3x**2**) **b**<br>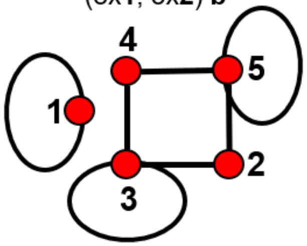
	(8x**1**, 1x**2**, 2x**2**^{1}) **a**<br>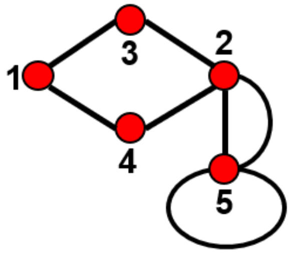	(8x**1**, 1x**2**, 2x**2**^{1}) **b**<br>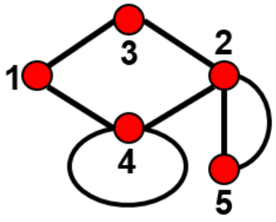	(8x**1**, 1x**2**, 2x**2**^{1}) **c**<br>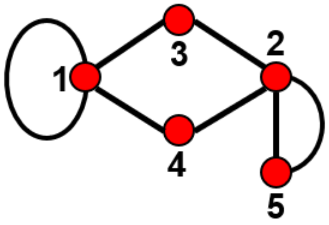	(8x**1**, 1x**2**, 2x**2**^{1}) **d**<br>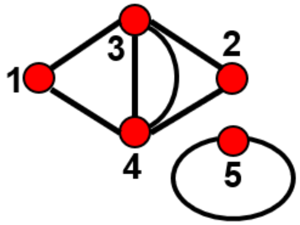
	(8x**1**, 1x**2**, 2x**2**^{2}) **a**<br>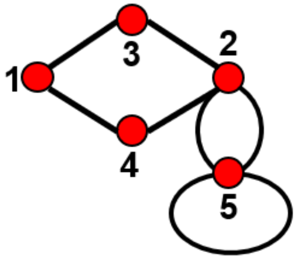	(8x**1**, 1x**2**, 2x**2**^{2}) **b**<br>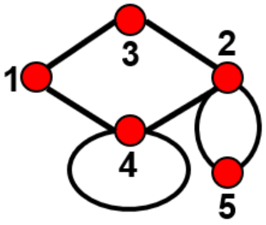	(8x**1**, 1x**2**, 2x**2**^{2}) **c**<br>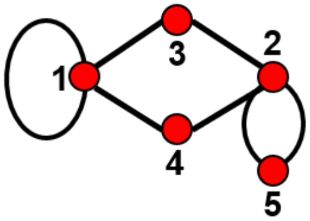	(8x**1**, 1x**2**, 2x**2**^{2}) **d**<br>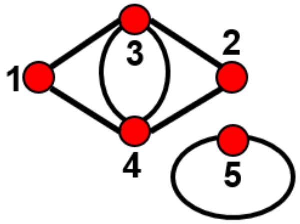
	(6x**1**, 4x**2**)<br>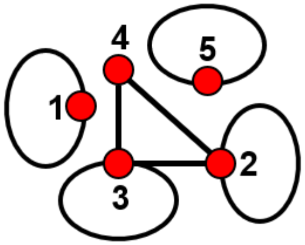	(6x**1**, 2x**2**, 2x**2**^{1}) **a**<br>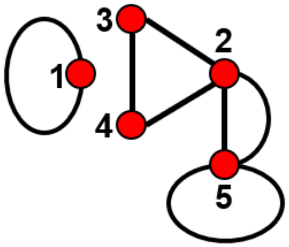	(6x**1**, 2x**2**, 2x**2**^{1}) **b**<br>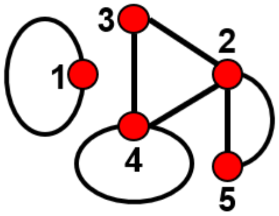	(6x**1**, 2x**2**, 2x**2**^{1}) **c**<br>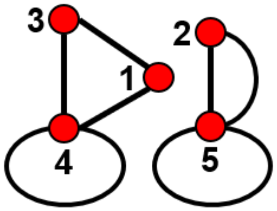

	(6x**1**, 2x**2**, 2x$\mathbf{2}^1$) **d**<br>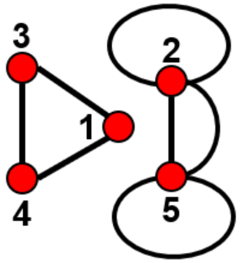	(6x**1**, 2x**2**, 2x$\mathbf{2}^1$) **e**<br>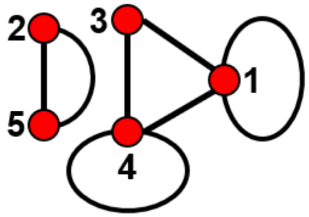	(6x**1**, 2x**2**, 2x$\mathbf{2}^2$) **a**<br>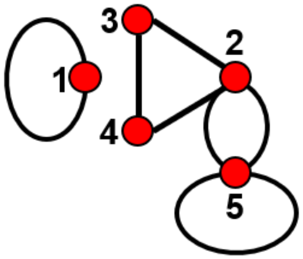	(6x**1**, 2x**2**, 2x$\mathbf{2}^2$) **b**<br>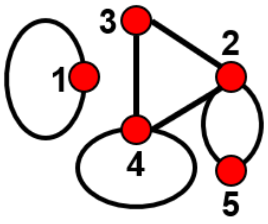
	(6x**1**, 2x**2**, 2x$\mathbf{2}^2$) **c**<br>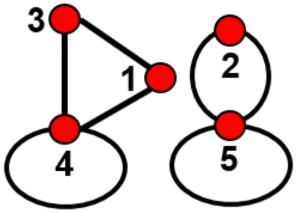	(6x**1**, 2x**2**, 2x$\mathbf{2}^2$) **d**<br>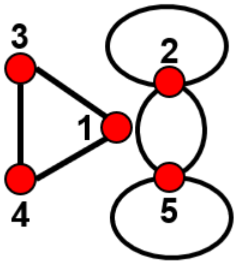	(6x**1**, 2x**2**, 2x$\mathbf{2}^2$) **e**<br>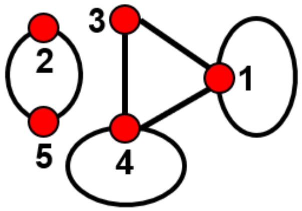	(6x**1**, 4x$\mathbf{2}^1$) **a**<br>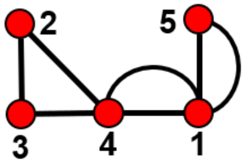
	(6x**1**, 4x$\mathbf{2}^1$) **b**<br>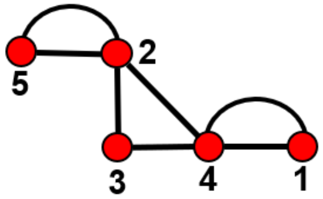	(6x**1**, 2x$\mathbf{2}^1$, 2x$\mathbf{2}^2$) **a**<br>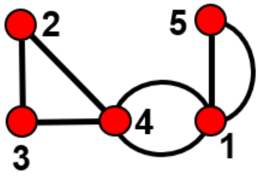	(6x**1**, 2x$\mathbf{2}^1$, 2x$\mathbf{2}^2$) **b**<br>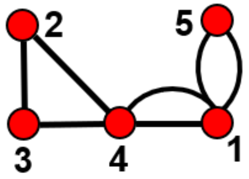	(6x**1**, 2x$\mathbf{2}^1$, 2x$\mathbf{2}^2$) **c**<br>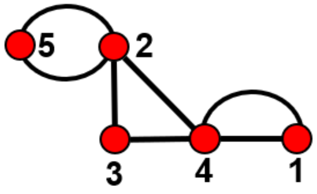

	(6x**1**, 4x**2**^{2}) **a**<br>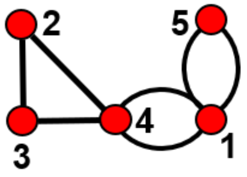	(6x**1**, 4x**2**^{2}) **b**<br>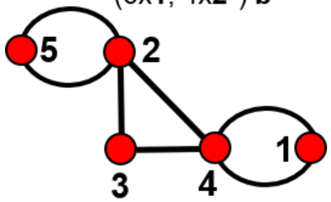	(5x**2**, 2x**2**^{1})<br>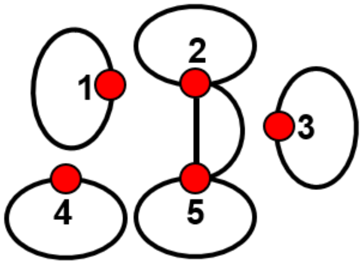	(5x**2**, 2x**2**^{2})<br>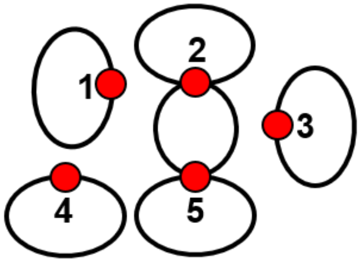
	(3x**2**, 4x**2**^{1}) **a**<br>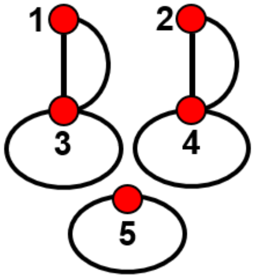	(3x**2**, 4x**2**^{1}) **b**<br>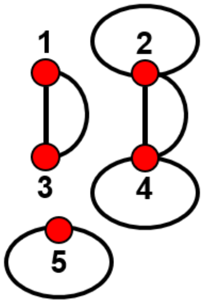	(3x**2**, 4x**2**^{1}) **c**<br>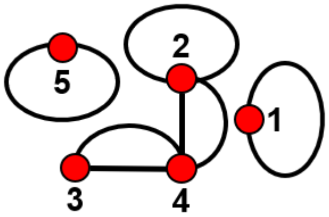	(3x**2**, 2x**2**^{1}**,** 2x**2**^{2}) **a**<br>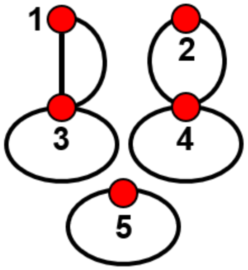
	(3x**2**, 2x**2**^{1}, 2x**2**^{2}) **b**<br>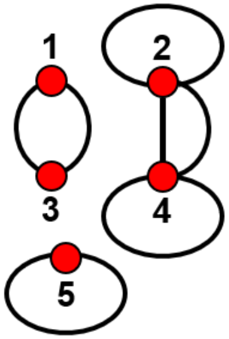	(3x**2**, 2x**2**^{1}, 2x**2**^{2}) **c**<br>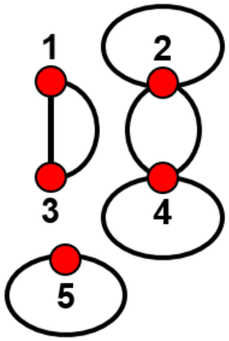	(3x**2**, 2x**2**^{1}, 2x**2**^{2}) **d**<br>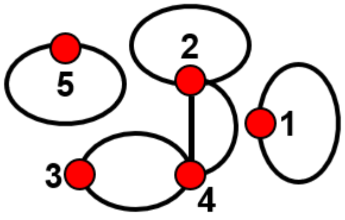	(3x**2**, 2x**2**^{1}, 2x**2**^{2}) **e**<br>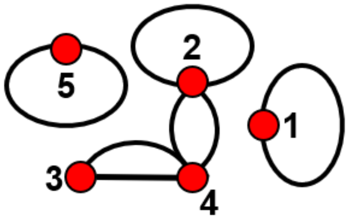

	(3x**2**, 4x**2**^{2}) **a** 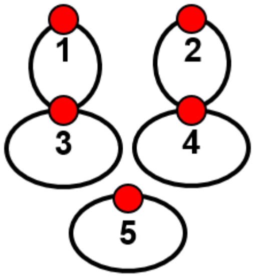	(3x**2**, 4x**2**^{2}) **b** 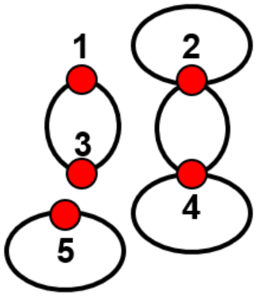	(3x**2**, 4x**2**^{2}) **c** 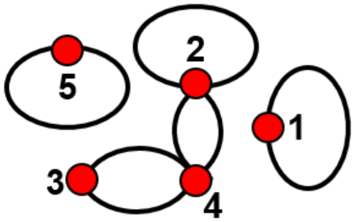	(1x**2**, 6x**2**^{1}) **a** 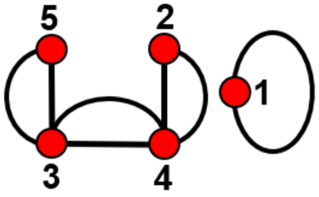
	(1x**2**, 6x**2**^{1}) **b** 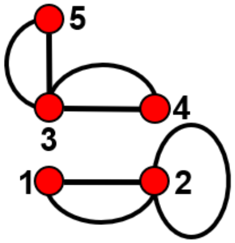	(1x**2**, 6x**2**^{1}) **c** 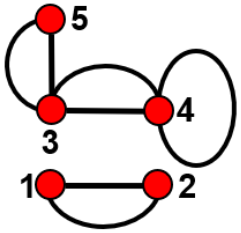	(1x**2**, 4x**2**^{1}, 2x**2**^{2}) **a** 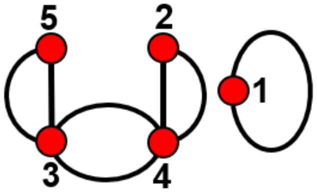	(1x**2**, 4x**2**^{1}, 2x**2**^{2}) **b** 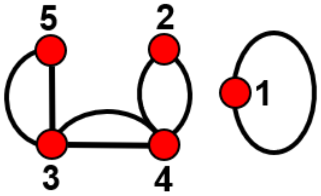
	(1x**2**, 4x**2**^{1}, 2x**2**^{2}) **c** 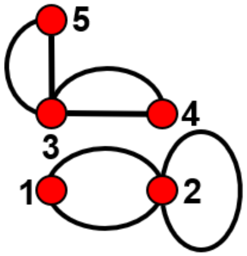	(1x**2**, 4x**2**^{1}, 2x**2**^{2}) **d** 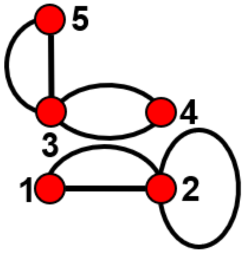	(1x**2**, 4x**2**^{1}, 2x**2**^{2}) **e** 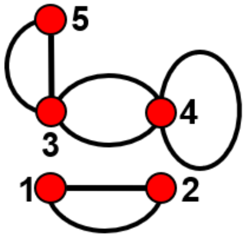	(1x**2**, 4x**2**^{1}, 2x**2**^{2}) **f** 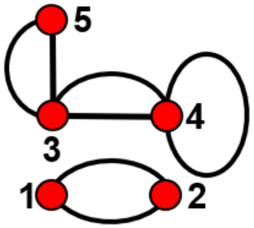

	(1x**2**, 4x$\mathbf{2}^1$, 2x$\mathbf{2}^2$) **g** 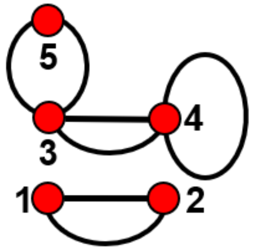	(1x**2**, 2x$\mathbf{2}^1$, 4x$\mathbf{2}^2$) **a** 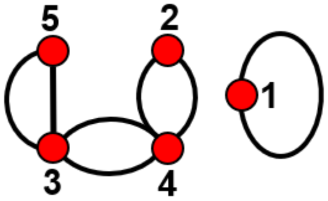	(1x**2**, 2x$\mathbf{2}^1$, 4x$\mathbf{2}^2$) **b** 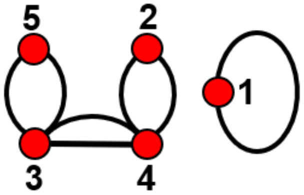	(1x**2**, 2x$\mathbf{2}^1$, 4x$\mathbf{2}^2$) **c** 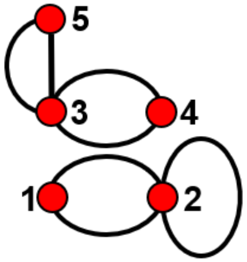
	(1x**2**, 2x$\mathbf{2}^1$, 4x$\mathbf{2}^2$) **d** 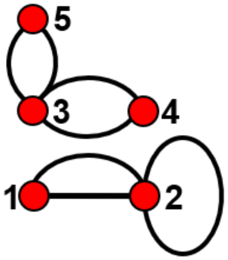	(1x**2**, 2x$\mathbf{2}^1$, 4x$\mathbf{2}^2$) **e** 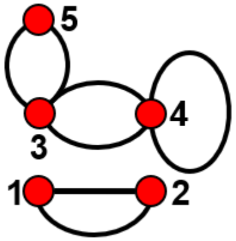	(1x**2**, 2x$\mathbf{2}^1$, 4x$\mathbf{2}^2$) **f** 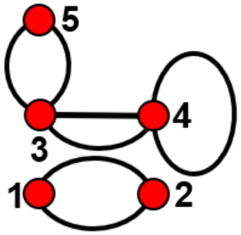	(1x**2**, 2x$\mathbf{2}^1$, 4x$\mathbf{2}^2$) **g** 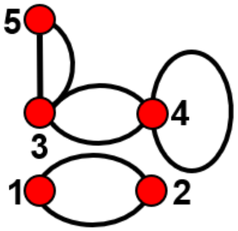
	(1x**2**, 6x$\mathbf{2}^2$) **a** 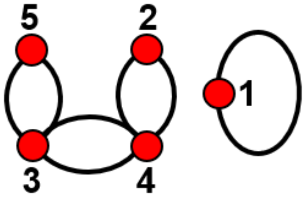	(1x**2**, 6x$\mathbf{2}^2$) **b** 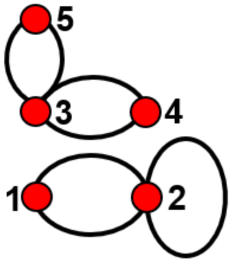	(1x**2**, 6x$\mathbf{2}^2$) **c** 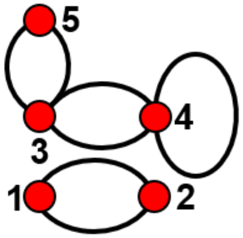	
$^2V_3{}^4V_3$	NG			
$^2V_3{}^4V_4$	NG			
$^2V_3{}^4V_5$	NG			

$^2V_4{}^4V_1$	(12x**1**)<br>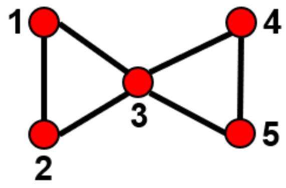	(10x**1**, 1x**2**)<br>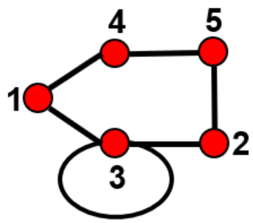	(8x**1**, 2x**2**)<br>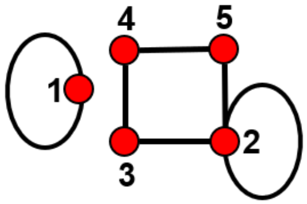	(8x**1**, 2x$\mathbf{2}^1$)<br>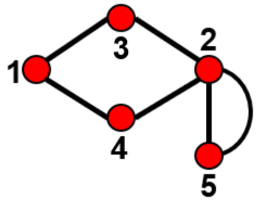
	(8x**1**, 2x$\mathbf{2}^2$)<br>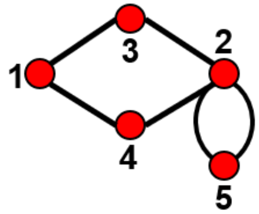	(6x**1**, 3x**2**)<br>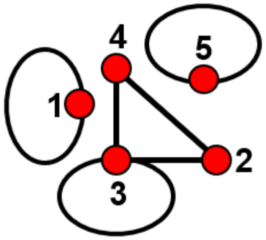	(6x**1**, 1x**2**, 2x$\mathbf{2}^1$) **a**<br>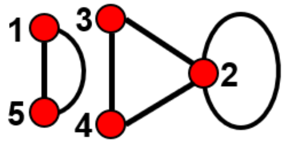	(6x**1**, 1x**2**, 2x$\mathbf{2}^1$) **b**<br>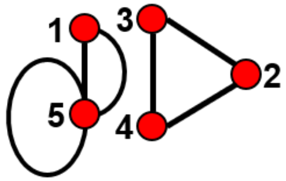
	(6x**1**, 1x**2**, 2x$\mathbf{2}^1$) **c**<br>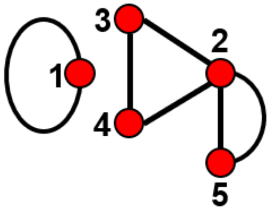	(6x**1**, 1x**2**, 2x$\mathbf{2}^2$) **a**<br>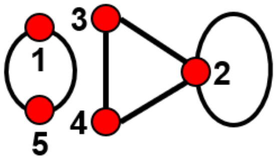	(6x**1**, 1x**2**, 2x$\mathbf{2}^2$) **b**<br>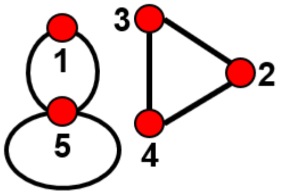	(6x**1**, 1x**2**, 2x$\mathbf{2}^2$) **c**<br>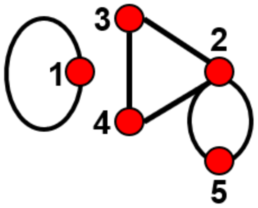
	(4x**2**, 2x$\mathbf{2}^1$)<br>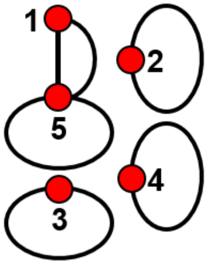	(4x**2**, 2x$\mathbf{2}^2$)<br>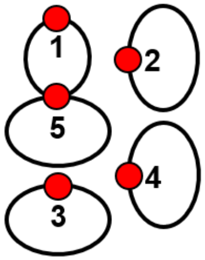	(2x**2**, 4x$\mathbf{2}^1$) **a**<br>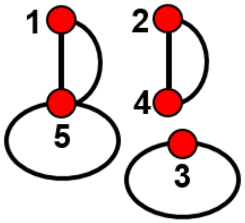	(2x**2**, 4x$\mathbf{2}^1$) **b**<br>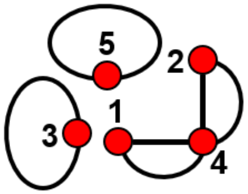

	(2x**2**, 2x$\mathbf{2}^1$, 2x$\mathbf{2}^2$) **a** 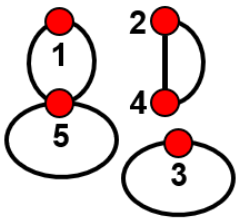	(2x**2**, 2x$\mathbf{2}^1$, 2x$\mathbf{2}^2$) **b** 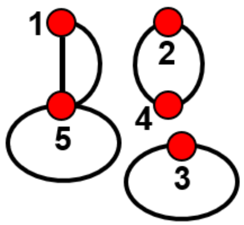	(2x**2**, 2x$\mathbf{2}^1$, 2x$\mathbf{2}^2$) **c** 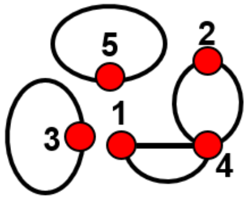	(2x**2**, 4x$\mathbf{2}^2$) **a** 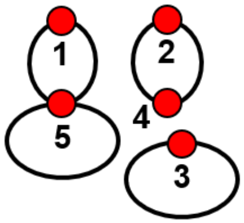
	(2x**2**, 4x$\mathbf{2}^2$) **b** 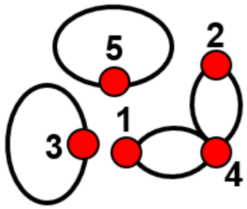	(6x$\mathbf{2}^1$) 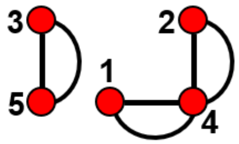	(4x$\mathbf{2}^1$, 2x$\mathbf{2}^2$) **a** 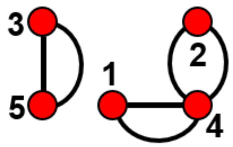	(4x$\mathbf{2}^1$, 2x$\mathbf{2}^2$) **b** 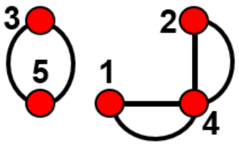
	(2x$\mathbf{2}^1$, 4x$\mathbf{2}^2$) **a** 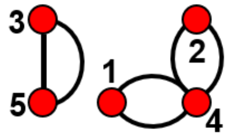	(2x$\mathbf{2}^1$, 4x$\mathbf{2}^2$) **b** 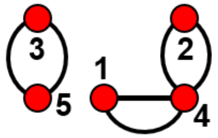	(6x$\mathbf{2}^2$) 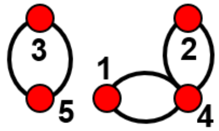	
${}^2V_4{}^4V_2$	(16x**1**) **a** 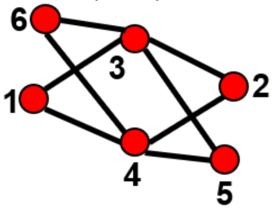	(16x**1**) **b** 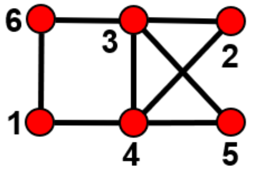	(14x**1**, 1x**2**) **a** 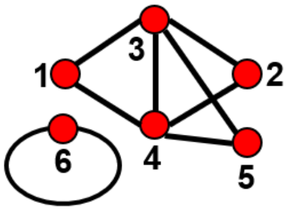	(14x**1**, 1x**2**) **b** 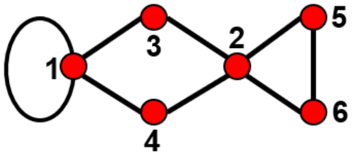

	(14x**1**, 1x**2**) **c** 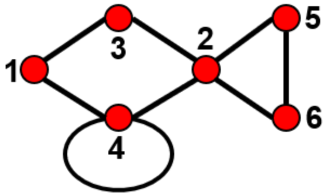	(14x**1**, 1x**2**) **d** 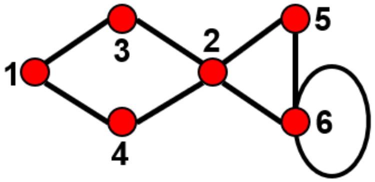	(12x**1**, 2x**2**) **a** 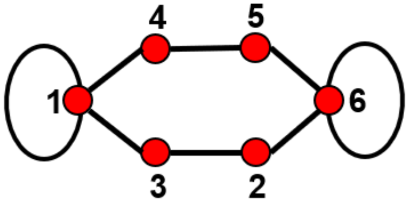	(12x**1**, 2x**2**) **b** 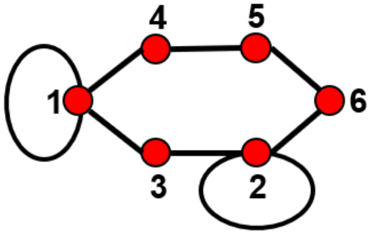
	(12x**1**, 2x**2**) **c** 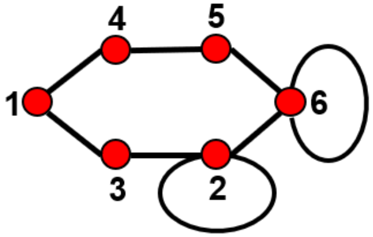	(12x**1**, 2x**2**) **d** 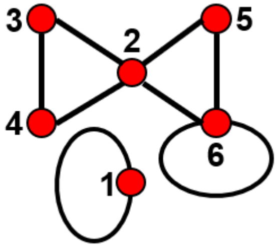	(12x**1**, 2x**2**) **e** 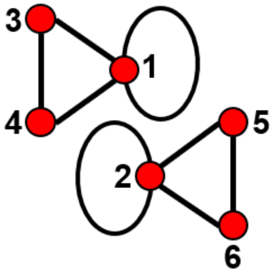	(12x**1**, 2x**2**) **f** 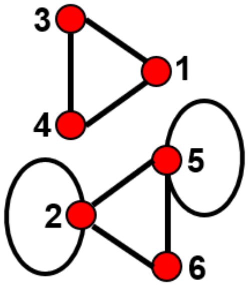
	(12x**1**, 2x$\mathbf{2}^1$) **a** 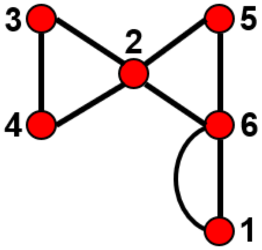	(12x**1**, 2x$\mathbf{2}^1$) **b** 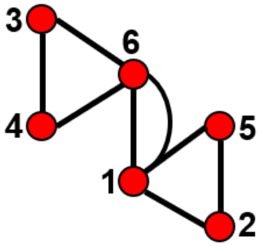	(12x**1**, 2x$\mathbf{2}^1$) **c** 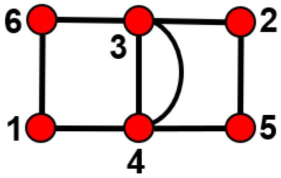	(12x**1**, 2x$\mathbf{2}^1$) **d** 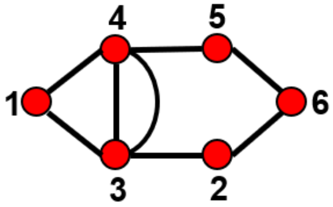

	(12x**1**, 2x**2**^{2}) **a** 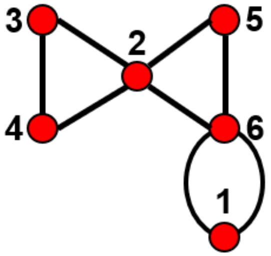	(12x**1**, 2x**2**^{2}) **b** 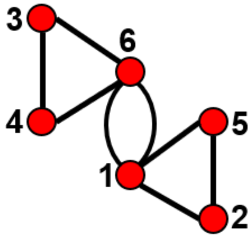	(12x**1**, 2x**2**^{2}) **c** 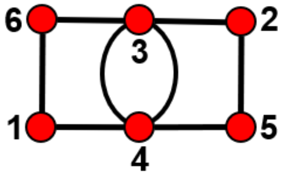	(12x**1**, 2x**2**^{2}) **d** 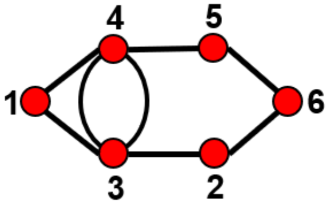
	(10x**1**, 3x**2**) **a** 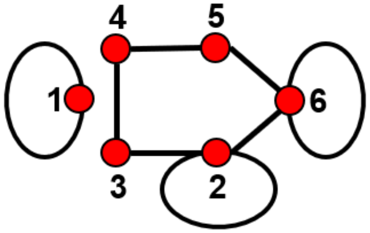	(10x**1**, 3x**2**) **b** 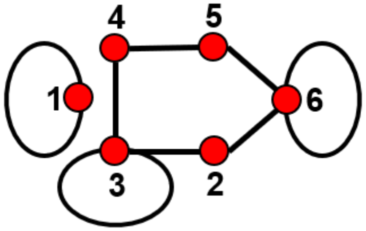	(10x**1**, 1x**2**, 2x**2**^{1}) **a** 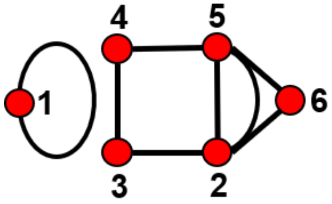	(10x**1**, 1x**2**, 2x**2**^{1}) **b** 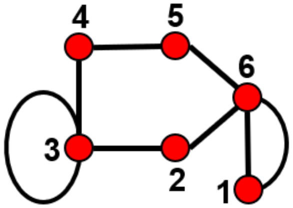
	(10x**1**, 1x**2**, 2x**2**^{1}) **c** 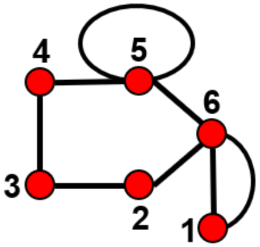	(10x**1**, 1x**2**, 2x**2**^{1}) **d** 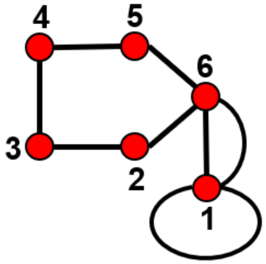	(10x**1**, 1x**2**, 2x**2**^{2}) **a** 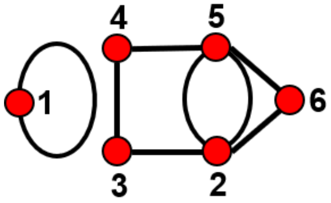	(10x**1**, 1x**2**, 2x**2**^{2}) **b** 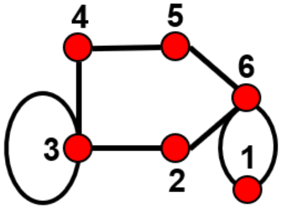

	(10x**1**, 1x**2**, 2x$\mathbf{2}^{2}$) **c**	(10x**1**, 1x**2**, 2x$\mathbf{2}^{2}$) **d**	(8x**1**, 4x**2**) **a**	(8x**1**, 4x**2**) **b**
	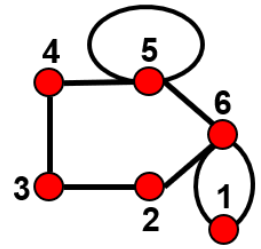	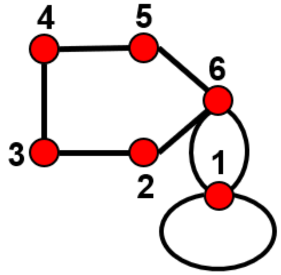	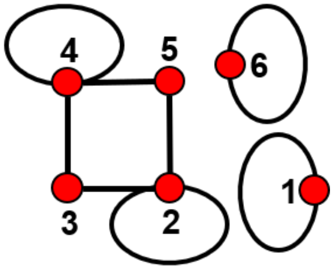	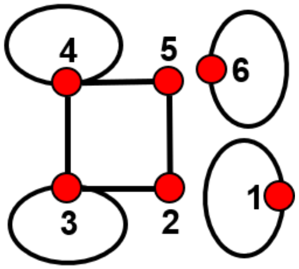
	(8x**1**, 2x**2**, 2x$\mathbf{2}^{1}$) **a**	(8x**1**, 2x**2**, 2x$\mathbf{2}^{1}$) **b**	(8x**1**, 2x**2**, 2x$\mathbf{2}^{1}$) **c**	(8x**1**, 2x**2**, 2x$\mathbf{2}^{1}$) **d**
	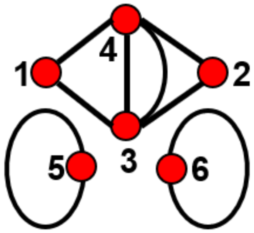	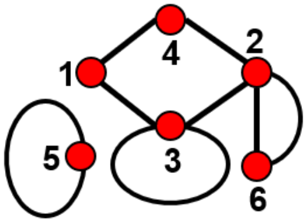	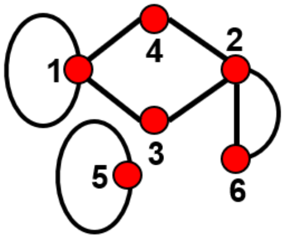	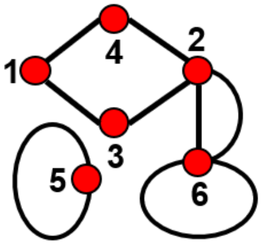
	(8x**1**, 2x**2**, 2x$\mathbf{2}^{1}$) **e**	(8x**1**, 2x**2**, 2x$\mathbf{2}^{1}$) **f**	(8x**1**, 2x**2**, 2x$\mathbf{2}^{1}$) **g**	(8x**1**, 2x**2**, 2x$\mathbf{2}^{1}$) **h**
	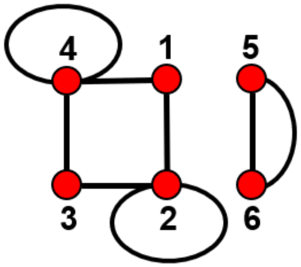	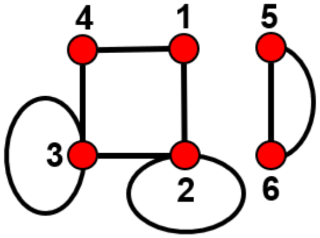	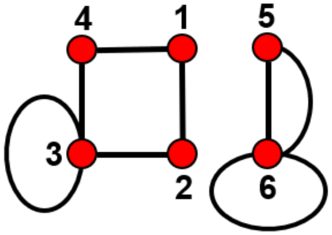	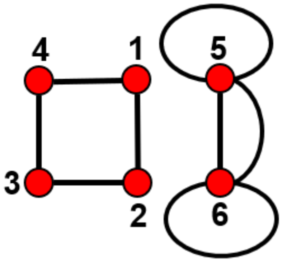

	(8x**1**, 2x**2**, 2x$\mathbf{2}^2$) **a** 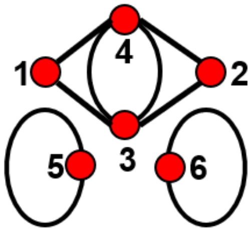	(8x**1**, 2x**2**, 2x$\mathbf{2}^2$) **b** 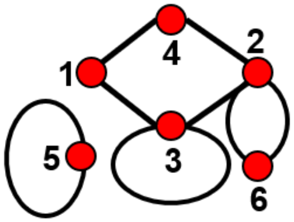	(8x**1**, 2x**2**, 2x$\mathbf{2}^2$) **c** 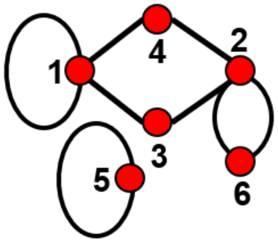	(8x**1**, 2x**2**, 2x$\mathbf{2}^2$) **d** 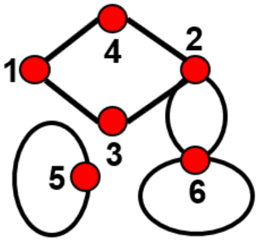
	(8x**1**, 2x**2**, 2x$\mathbf{2}^2$) **e** 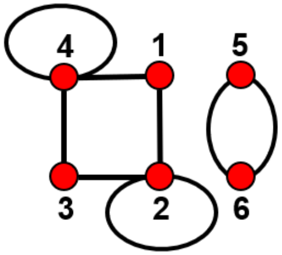	(8x**1**, 2x**2**, 2x$\mathbf{2}^2$) **f** 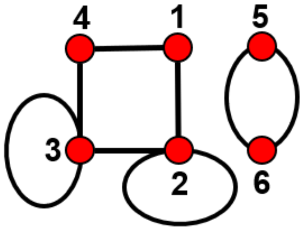	(8x**1**, 2x**2**, 2x$\mathbf{2}^2$) **g** 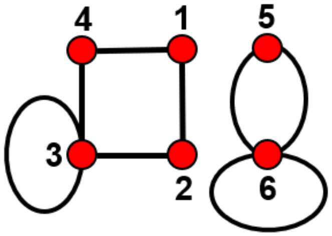	(8x**1**, 2x**2**, 2x$\mathbf{2}^2$) **h** 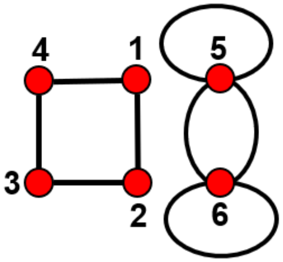
	(8x**1**, 4x$\mathbf{2}^1$) **a** 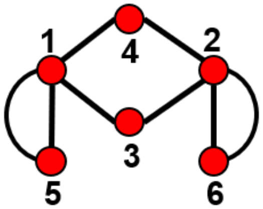	(8x**1**, 4x$\mathbf{2}^1$) **b** 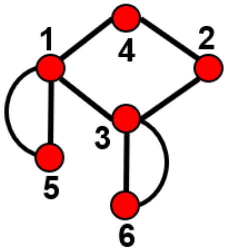	(8x**1**, 4x$\mathbf{2}^1$) **c** 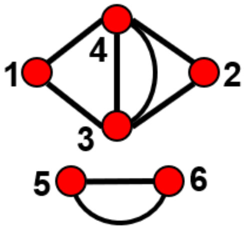	(8x**1**, 4x$\mathbf{2}^1$) **d** 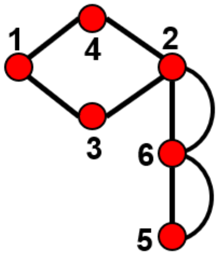

	(8x**1**, 2x$\mathbf{2}^1$, 2x$\mathbf{2}^2$) **a**<br>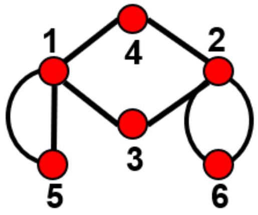	(8x**1**, 2x$\mathbf{2}^1$, 2x$\mathbf{2}^2$) **b**<br>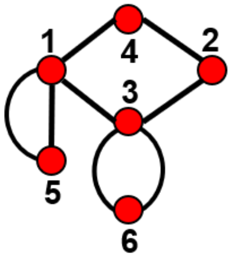	(8x**1**, 2x$\mathbf{2}^1$, 2x$\mathbf{2}^2$) **c**<br>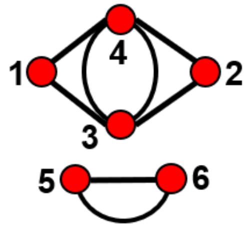	(8x**1**, 2x$\mathbf{2}^1$, 2x$\mathbf{2}^2$) **d**<br>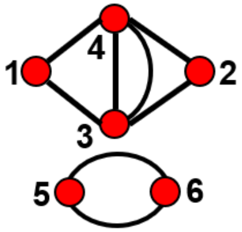
	(8x**1**, 2x$\mathbf{2}^1$, 2x$\mathbf{2}^2$) **e**<br>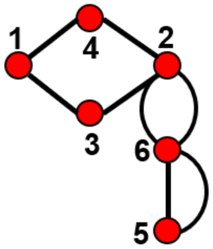	(8x**1**, 2x$\mathbf{2}^1$, 2x$\mathbf{2}^2$) **f**<br>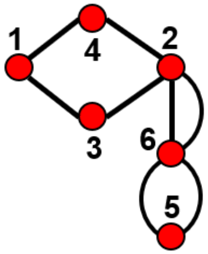	(8x**1**, 4x$\mathbf{2}^2$) **a**<br>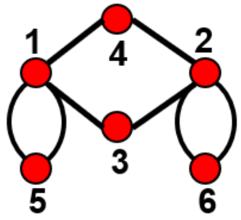	(8x**1**, 4x$\mathbf{2}^2$) **b**<br>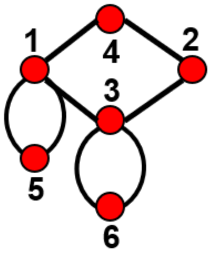
	(8x**1**, 4x$\mathbf{2}^2$) **c**<br>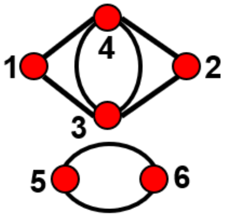	(8x**1**, 4x$\mathbf{2}^2$) **d**<br>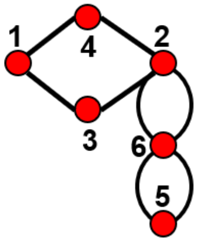	(6x**1**, 5x**2**)<br>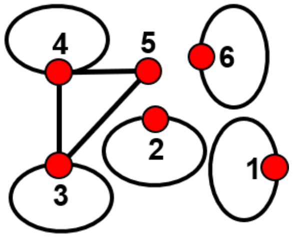	(6x**1**, 3x**2**, 2x$\mathbf{2}^1$) **a**<br>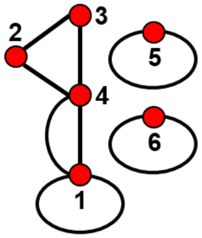

	(6x**1**, 3x**2**, 2x**2**^{1}) **b** 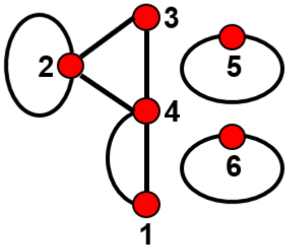	(6x**1**, 3x**2**, 2x**2**^{1}) **c** 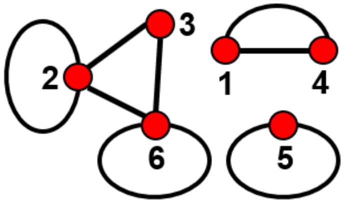	(6x**1**, 3x**2**, 2x**2**^{1}) **d** 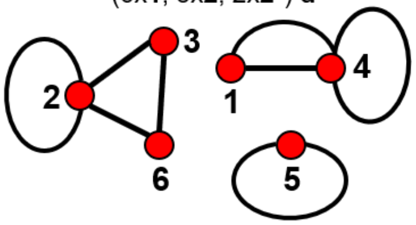	(6x**1**, 3x**2**, 2x**2**^{1}) **e** 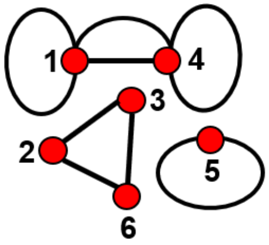
	(6x**1**, 3x**2**, 2x**2**^{2}) **a** 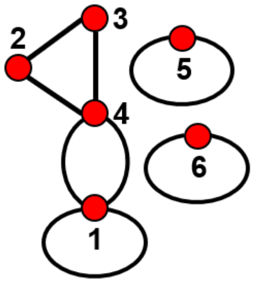	(6x**1**, 3x**2**, 2x**2**^{2}) **b** 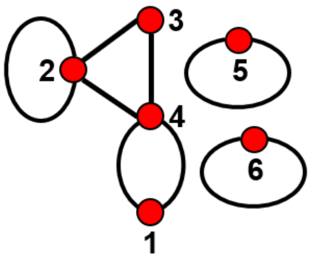	(6x**1**, 3x**2**, 2x**2**^{2}) **c** 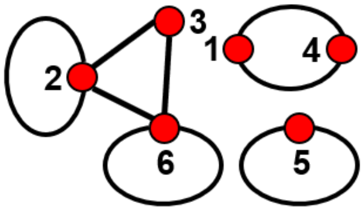	(6x**1**, 3x**2**, 2x**2**^{2}) **d** 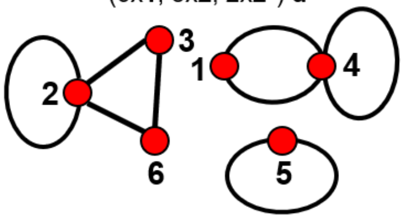
	(6x**1**, 3x**2**, 2x**2**^{2}) **e** 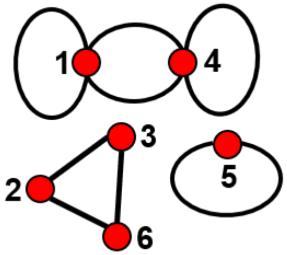	(6x**1**, 1x**2**, 4x**2**^{1}) **a** 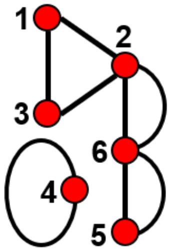	(6x**1**, 1x**2**, 4x**2**^{1}) **b** 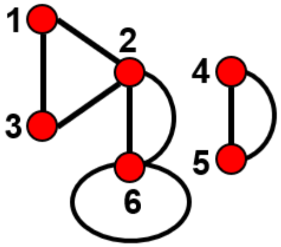	(6x**1**, 1x**2**, 4x**2**^{1}) **c** 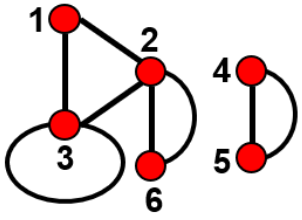

	(6x**1**, 1x**2**, 4x**2**^{1}) **d** 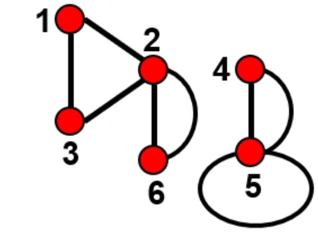	(6x**1**, 1x**2**, 4x**2**^{1}) **e** 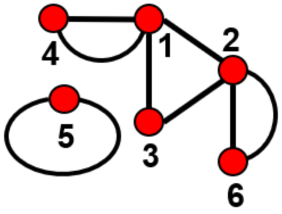	(6x**1**, 1x**2**, 4x**2**^{1}) **f** 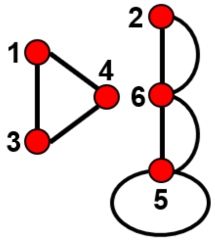	(6x**1**, 1x**2**, 4x**2**^{1}) **g** 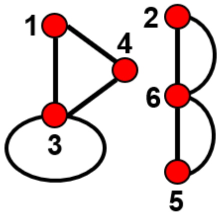
	(6x**1**, 1x**2**, 2x**2**^{1}, 2x**2**^{2}) **a** 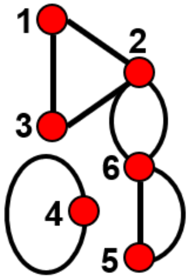	(6x**1**, 1x**2**, 2x**2**^{1}, 2x**2**^{2}) **b** 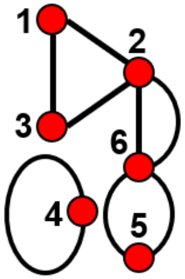	(6x**1**, 1x**2**, 2x**2**^{1}, 2x**2**^{2}) **c** 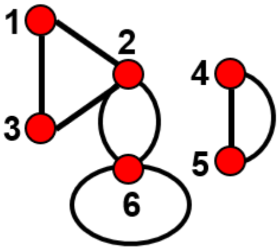	(6x**1**, 1x**2**, 2x**2**^{1}, 2x**2**^{2}) **d** 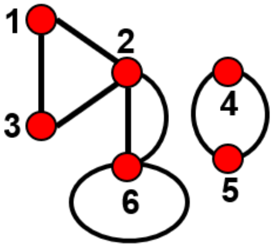
	(6x**1**, 1x**2**, 2x**2**^{1}, 2x**2**^{2}) **e** 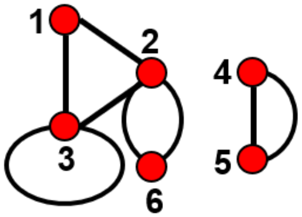	(6x**1**, 1x**2**, 2x**2**^{1}, 2x**2**^{2}) **f** 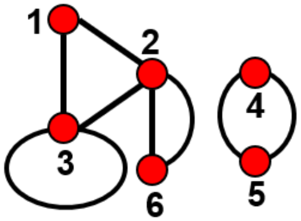	(6x**1**, 1x**2**, 2x**2**^{1}, 2x**2**^{2}) **g** 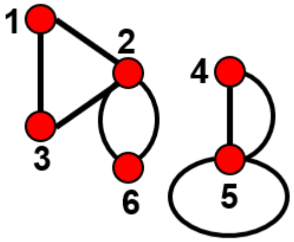	(6x**1**, 1x**2**, 2x**2**^{1}, 2x**2**^{2}) **h** 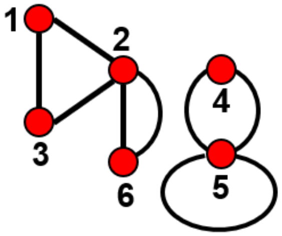

	(6x**1**, 1x**2**, 2x$\mathbf{2}^1$, 2x$\mathbf{2}^2$) **i** 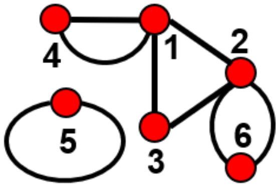	(6x**1**, 1x**2**, 2x$\mathbf{2}^1$, 2x$\mathbf{2}^2$) **j** 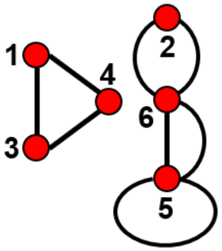	(6x**1**, 1x**2**, 2x$\mathbf{2}^1$, 2x$\mathbf{2}^2$) **k** 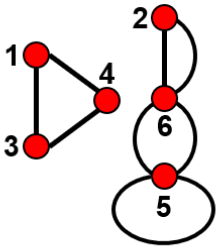	(6x**1**, 1x**2**, 2x$\mathbf{2}^1$, 2x$\mathbf{2}^2$) **l** 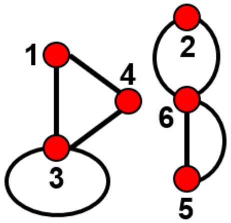
	(6x**1**, 1x**2**, 2x$\mathbf{2}^1$, 2x$\mathbf{2}^2$) **m** 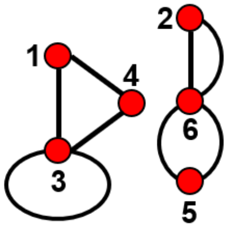	(6x**1**, 1x**2**, 4x$\mathbf{2}^2$) **a** 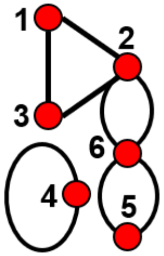	(6x**1**, 1x**2**, 4x$\mathbf{2}^2$) **b** 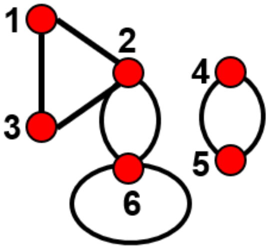	(6x**1**, 1x**2**, 4x$\mathbf{2}^2$) **c** 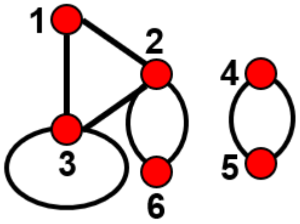
	(6x**1**, 1x**2**, 4x$\mathbf{2}^2$) **d** 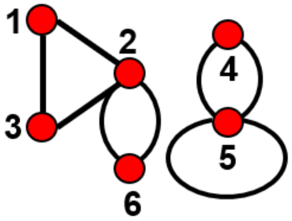	(6x**1**, 1x**2**, 4x$\mathbf{2}^2$) **e** 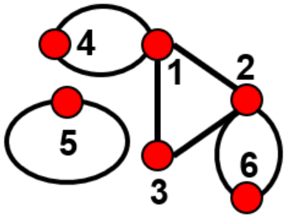	(6x**1**, 1x**2**, 4x$\mathbf{2}^2$) **f** 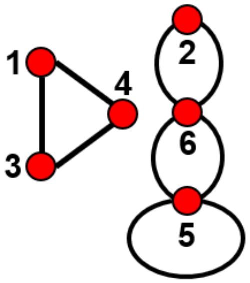	(6x**1**, 1x**2**, 4x$\mathbf{2}^2$) **g** 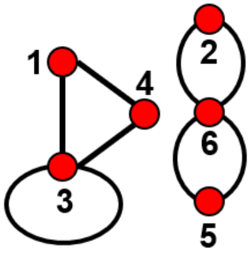

	(6x**2**, 2x$\mathbf{2}^1$)<br>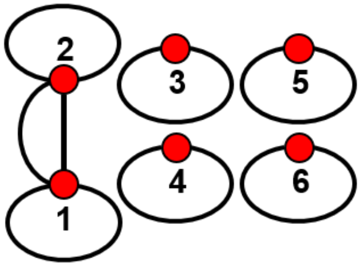	(6x**2**, 2x$\mathbf{2}^2$)<br>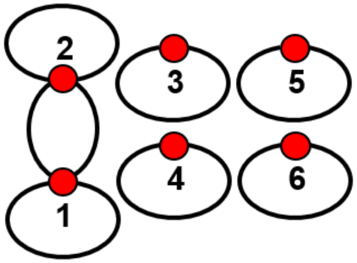	(4x**2**, 4x$\mathbf{2}^1$) **a**<br>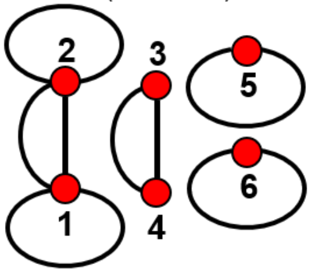	(4x**2**, 4x$\mathbf{2}^1$) **b**<br>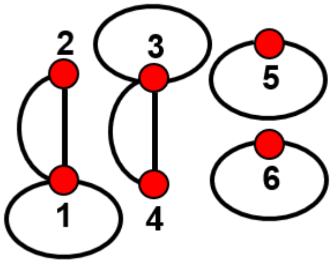
	(4x**2**, 4x$\mathbf{2}^1$) **c**<br>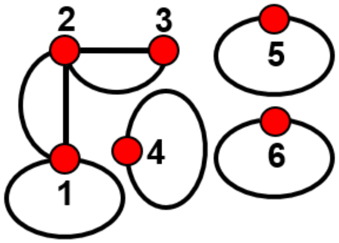	(4x**2**, 2x$\mathbf{2}^1$, 2x$\mathbf{2}^2$) **a**<br>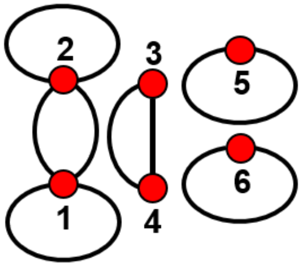	(4x**2**, 2x$\mathbf{2}^1$, 2x$\mathbf{2}^2$) **b**<br>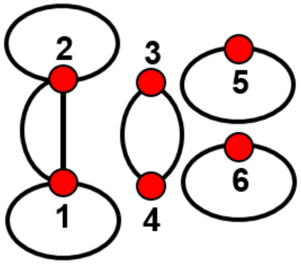	(4x**2**, 2x$\mathbf{2}^1$, 2x$\mathbf{2}^2$) **c**<br>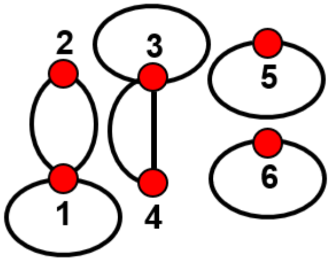
	(4x**2**, 2x$\mathbf{2}^1$, 2x$\mathbf{2}^2$) **d**<br>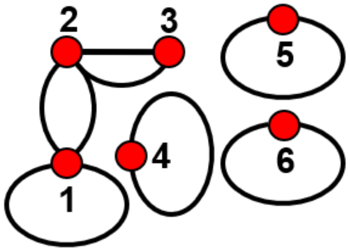	(4x**2**, 2x$\mathbf{2}^1$, 2x$\mathbf{2}^2$) **e**<br>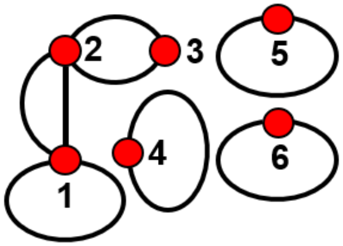	(4x**2**, 4x$\mathbf{2}^2$) **a**<br>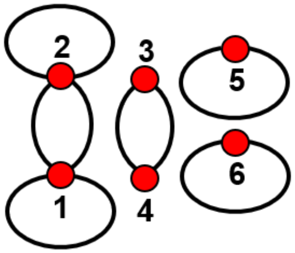	(4x**2**, 4x$\mathbf{2}^2$) **b**<br>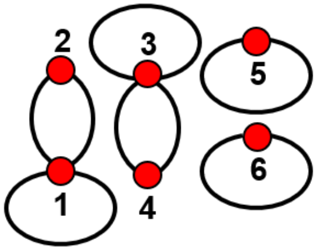

	(4x**2**, 4x**2**^{2}) **c**<br>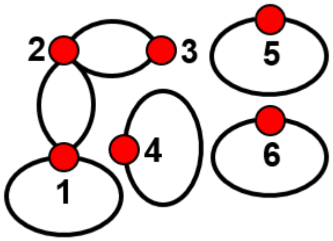	(2x**2**, 6x**2**^{1}) **a**<br>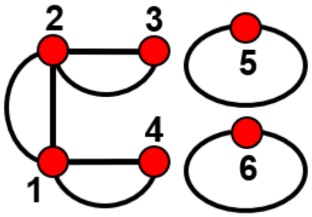	(2x**2**, 6x**2**^{1}) **b**<br>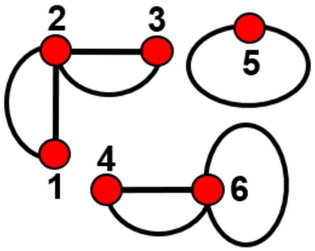	(2x**2**, 6x**2**^{1}) **c**<br>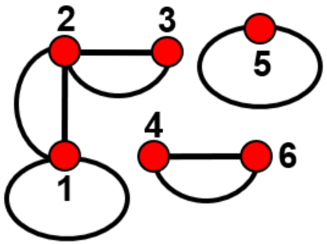
	(2x**2**, 6x**2**^{1}) **d**<br>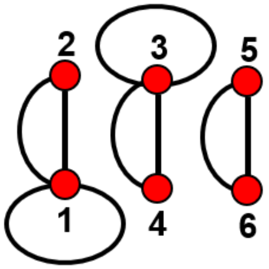	(2x**2**, 6x**2**^{1}) **e**<br>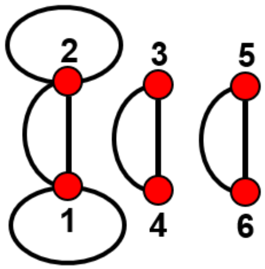	(2x**2**, 4x**2**^{1}, 2x**2**^{2}) **a**<br>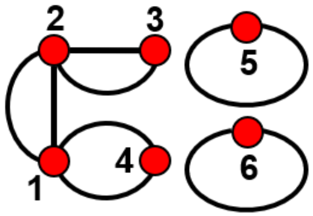	(2x**2**, 4x**2**^{1}, 2x**2**^{2}) **b**<br>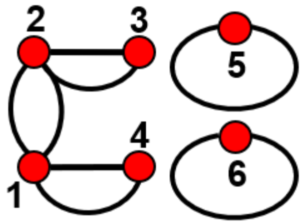
	(2x**2**, 4x**2**^{1}, 2x**2**^{2}) **c**<br>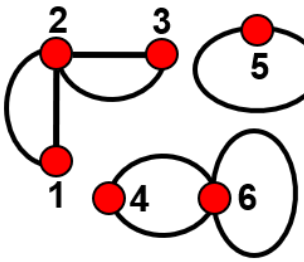	(2x**2**, 4x**2**^{1}, 2x**2**^{2}) **d**<br>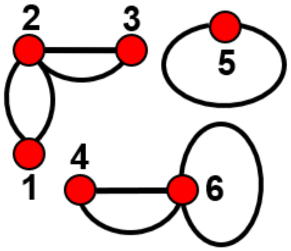	(2x**2**, 4x**2**^{1}, 2x**2**^{2}) **e**<br>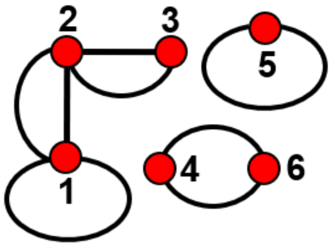	(2x**2**, 4x**2**^{1}, 2x**2**^{2}) **f**<br>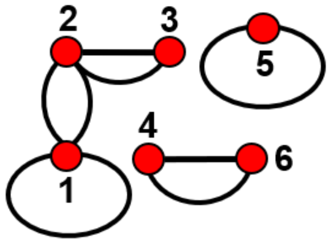

<table>
<tr><td></td><td>(2x$\mathbf{2}$, 4x$\mathbf{2}^1$, 2x$\mathbf{2}^2$) g
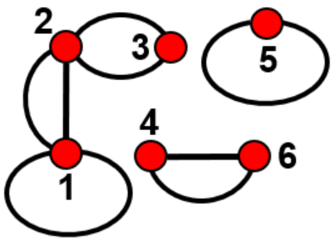</td><td>(2x$\mathbf{2}$, 4x$\mathbf{2}^1$, 2x$\mathbf{2}^2$) h
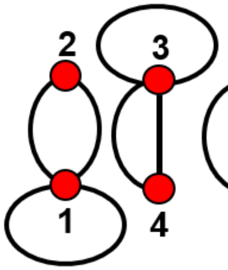</td><td>(2x$\mathbf{2}$, 4x$\mathbf{2}^1$, 2x$\mathbf{2}^2$) i
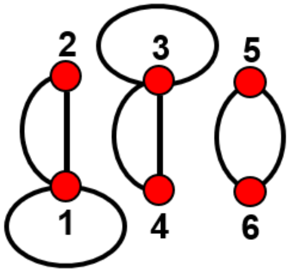</td><td>(2x$\mathbf{2}$, 4x$\mathbf{2}^1$, 2x$\mathbf{2}^2$) j
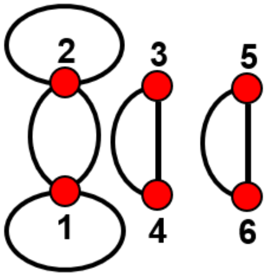</td></tr>
<tr><td></td><td>(2x$\mathbf{2}$, 4x$\mathbf{2}^1$, 2x$\mathbf{2}^2$) k
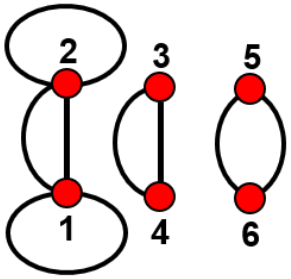</td><td>(2x$\mathbf{2}$, 2x$\mathbf{2}^1$, 4x$\mathbf{2}^2$) a
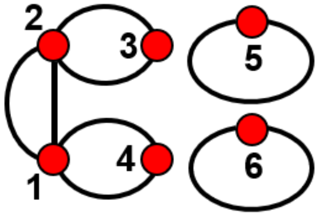</td><td>(2x$\mathbf{2}$, 2x$\mathbf{2}^1$, 4x$\mathbf{2}^2$) b
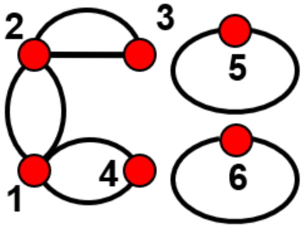</td><td>(2x$\mathbf{2}$, 2x$\mathbf{2}^1$, 4x$\mathbf{2}^2$) c
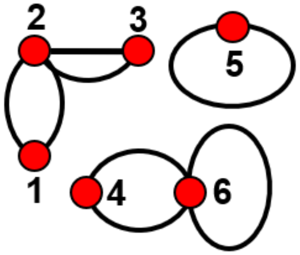</td></tr>
<tr><td></td><td>(2x$\mathbf{2}$, 2x$\mathbf{2}^1$, 4x$\mathbf{2}^2$) d
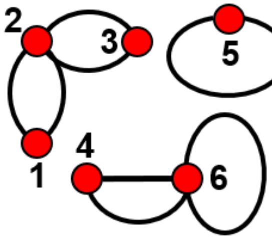</td><td>(2x$\mathbf{2}$, 2x$\mathbf{2}^1$, 4x$\mathbf{2}^2$) e
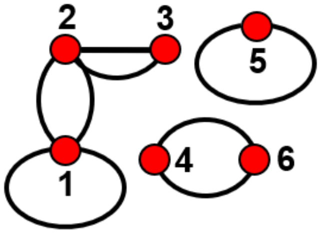</td><td>(2x$\mathbf{2}$, 2x$\mathbf{2}^1$, 4x$\mathbf{2}^2$) f
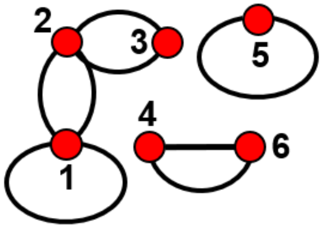</td><td>(2x$\mathbf{2}$, 2x$\mathbf{2}^1$, 4x$\mathbf{2}^2$) g
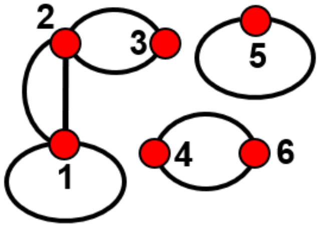</td></tr>
</table>

	(2x**2**, 2x**2**^{1}, 4x**2**^{2}) **h** 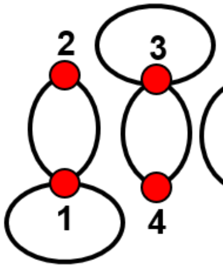	(2x**2**, 2x**2**^{1}, 4x**2**^{2}) **i** 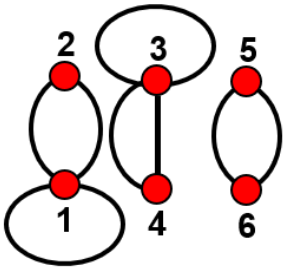	(2x**2**, 2x**2**^{1}, 4x**2**^{2}) **j** 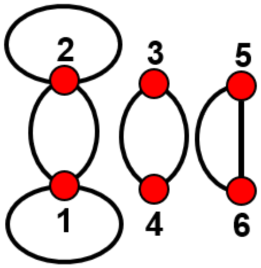	(2x**2**, 2x**2**^{1}, 4x**2**^{2}) **k** 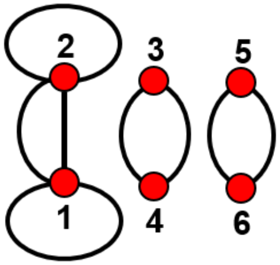
	(2x**2**, 6x**2**^{2}) **a** 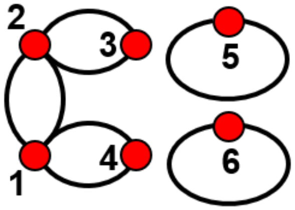	(2x**2**, 6x**2**^{2}) **b** 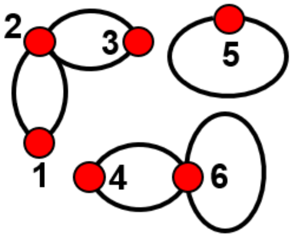	(2x**2**, 6x**2**^{2}) **c** 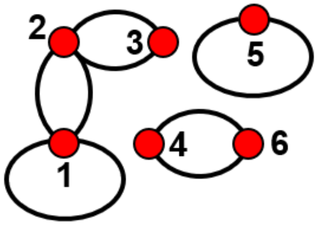	(2x**2**, 6x**2**^{2}) **d** 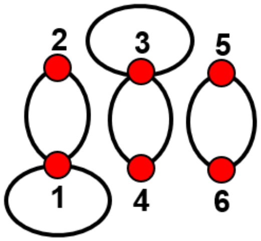
	(2x**2**, 6x**2**^{2}) **e** 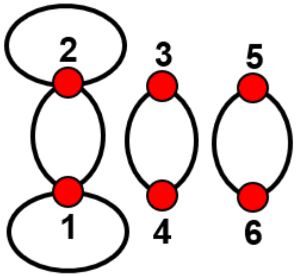	(8x**2**^{1}) **a** 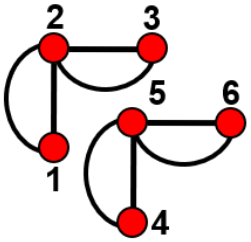	(8x**2**^{1}) **b** 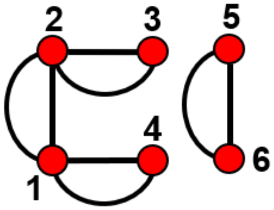	(6x**2**^{1}, 2x**2**^{2}) **a** 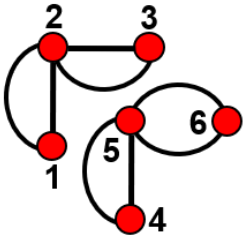

	(6x**2**^{1}, 2x**2**^{2}) **b** 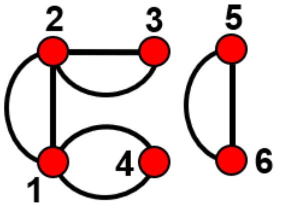	(6x**2**^{1}, 2x**2**^{2}) **c** 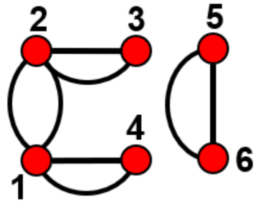	(6x**2**^{1}, 2x**2**^{2}) **d** 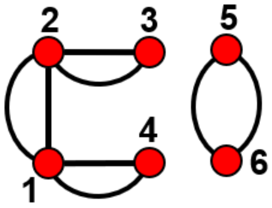	(4x**2**^{1}, 4x**2**^{2}) **a** 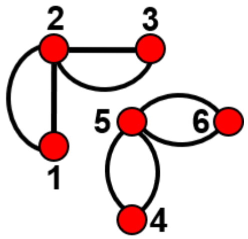
	(4x**2**^{1}, 4x**2**^{2}) **b** 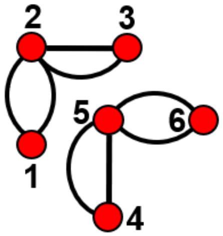	(4x**2**^{1}, 4x**2**^{2}) **c** 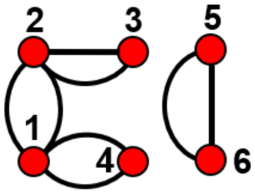	(4x**2**^{1}, 4x**2**^{2}) **d** 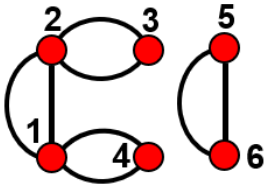	(4x**2**^{1}, 4x**2**^{2}) **e** 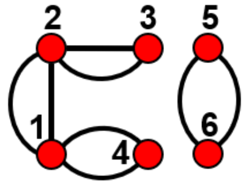
	(4x**2**^{1}, 4x**2**^{2}) **f** 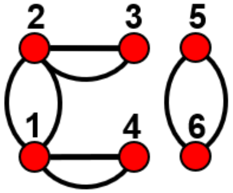	(2x**2**^{1}, 6x**2**^{2}) **a** 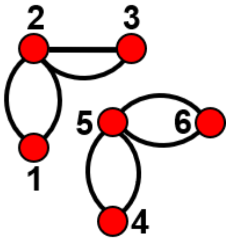	(2x**2**^{1}, 6x**2**^{2}) **b** 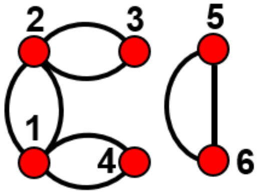	(2x**2**^{1}, 6x**2**^{2}) **c** 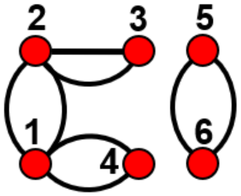

	(2x**2**^{1}, 6x**2**^{2}) **d** 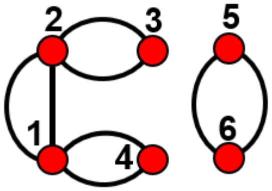	(8x**2**^{2}) **a** 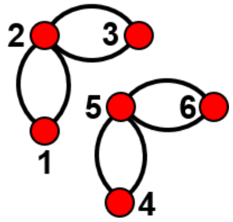	(8x**2**^{2}) **b** 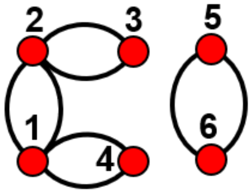	
$^2V_4{}^4V_3$	NG			
$^2V_4{}^4V_4$	NG			
$^2V_5{}^4V_1$	(14x**1**) 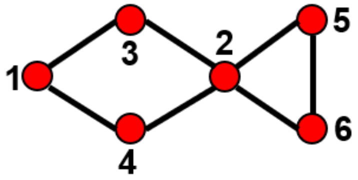	(12x**1**, 1x**2**) **a** 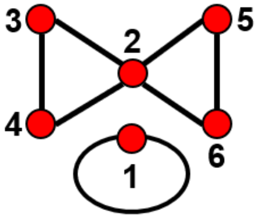	(12x**1**, 1x**2**) **b** 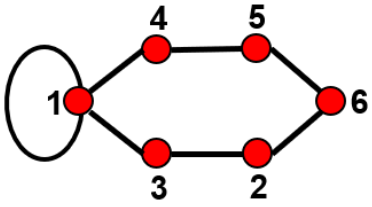	(12x**1**, 1x**2**) **c** 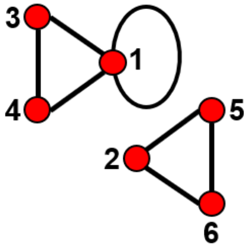
	(10x**1**, 2x**2**) 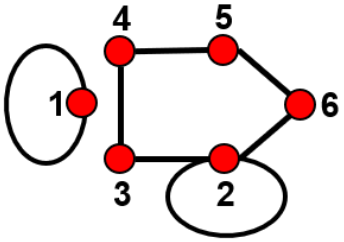	(10x**1**, 2x**2**^{1}) 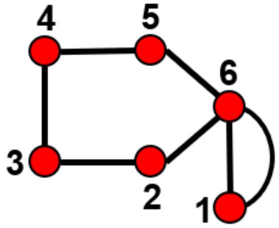	(10x**1**, 2x**2**^{2}) 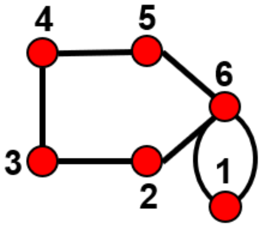	(8x**1**, 3x**2**) 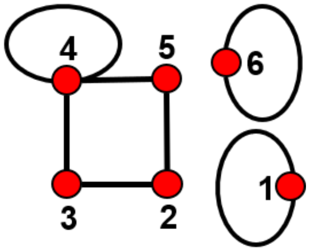

(8x**1**, 1x**2**, 2x$\mathbf{2}^{1}$) **a**

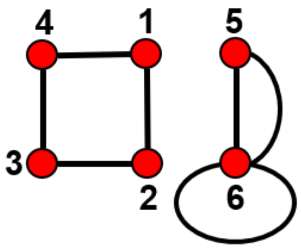

(8x**1**, 1x**2**, 2x$\mathbf{2}^{1}$) **b**

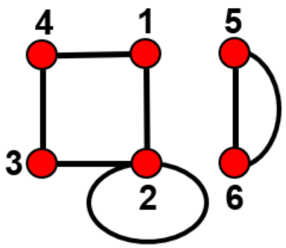

(8x**1**, 1x**2**, 2x$\mathbf{2}^{1}$) **c**

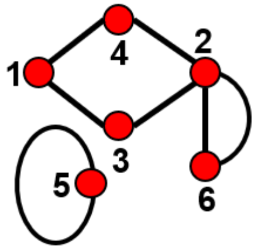

(8x**1**, 1x**2**, 2x$\mathbf{2}^{2}$) **a**

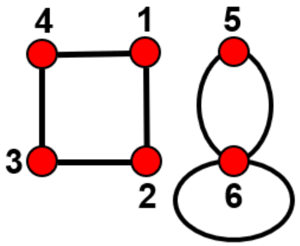

(8x**1**, 1x**2**, 2x$\mathbf{2}^{2}$) **b**

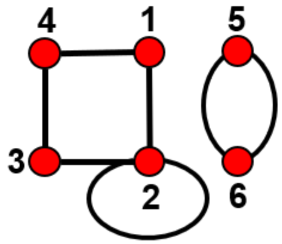

(8x**1**, 1x**2**, 2x$\mathbf{2}^{2}$) **c**

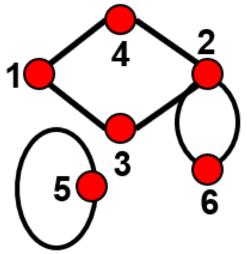

(6x**1**, 4x**2**)

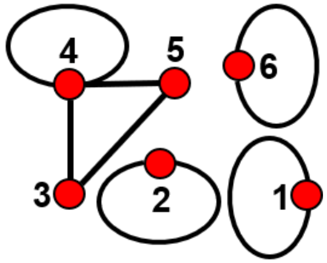

(6x**1**, 2x**2**, 2x$\mathbf{2}^{1}$) **a**

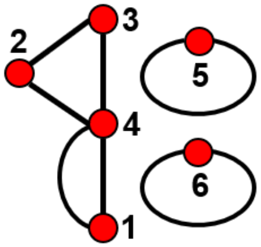

(6x**1**, 2x**2**, 2x$\mathbf{2}^{1}$) **b**

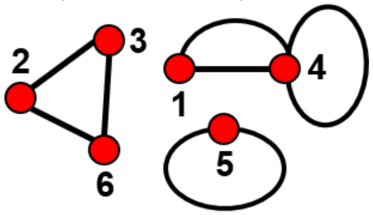

(6x**1**, 2x**2**, 2x$\mathbf{2}^{1}$) **c**

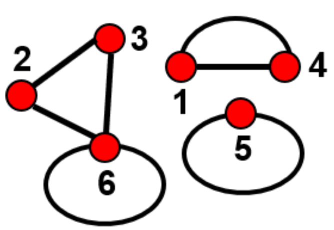

(6x**1**, 2x**2**, 2x$\mathbf{2}^{2}$) **a**

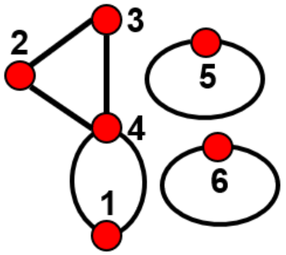

(6x**1**, 2x**2**, 2x$\mathbf{2}^{2}$) **b**

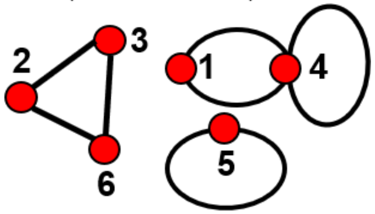

	(6x**1**, 2x**2**, 2x**2**^{2}) **c** 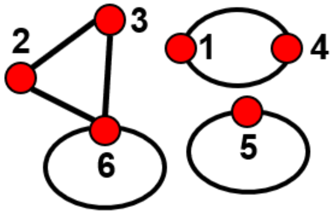	(6x**1**, 4x**2**^{1}) **a** 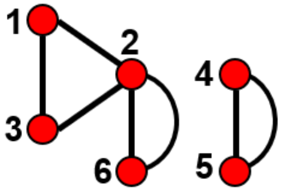	(6x**1**, 4x**2**^{1}) **b** 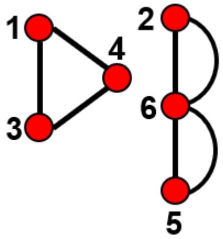	(6x**1**, 2x**2**^{1}, 2x**2**^{2}) **a** 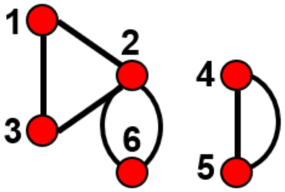
	(6x**1**, 2x**2**^{1}, 2x**2**^{2}) **b** 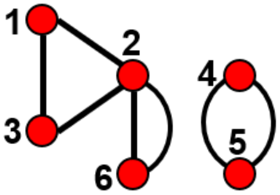	(6x**1**, 2x**2**^{1}, 2x**2**^{2}) **c** 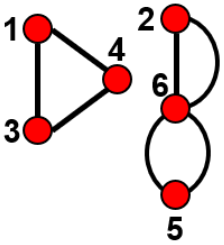	(6x**1**, 4x**2**^{2}) **a** 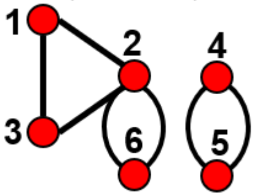	(6x**1**, 4x**2**^{2}) **b** 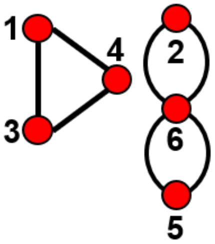
	(5x**2**, 2x**2**^{1}) 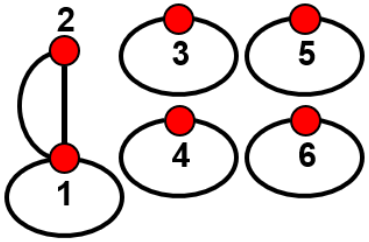	(5x**2**, 2x**2**^{2}) 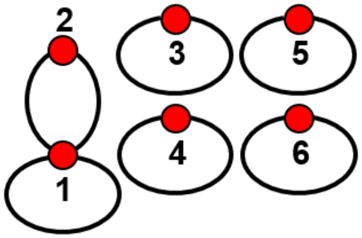	(3x**2**, 4x**2**^{1}) **a** 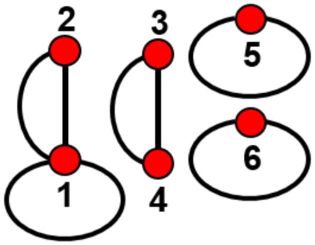	(3x**2**, 4x**2**^{1}) **b** 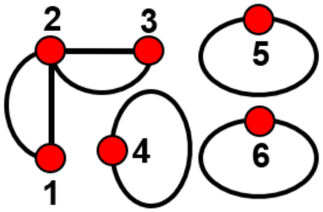

	(3x$\mathbf{2}$, 2x$\mathbf{2}^1$, 2x$\mathbf{2}^2$) **a** 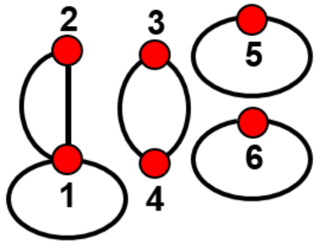	(3x$\mathbf{2}$, 2x$\mathbf{2}^1$, 2x$\mathbf{2}^2$) **b** 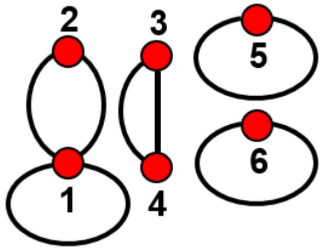	(3x$\mathbf{2}$, 2x$\mathbf{2}^1$, 2x$\mathbf{2}^2$) **c** 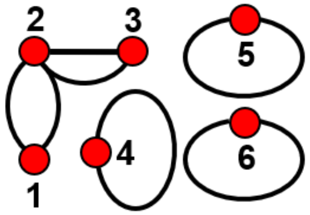	(3x$\mathbf{2}$, 4x$\mathbf{2}^2$) **a** 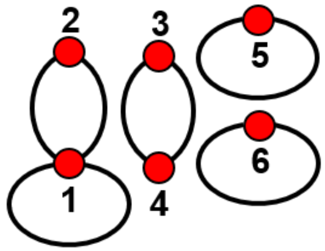
	(3x$\mathbf{2}$, 4x$\mathbf{2}^2$) **b** 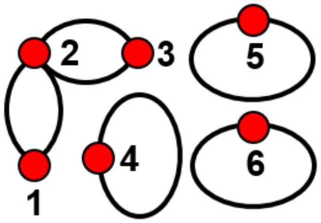	(1x$\mathbf{2}$, 6x$\mathbf{2}^1$) **a** 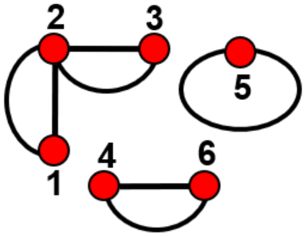	(1x$\mathbf{2}$, 6x$\mathbf{2}^1$) **b** 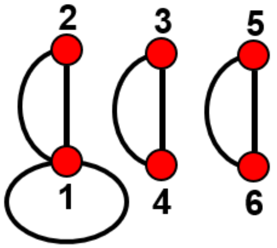	(1x$\mathbf{2}$, 4x$\mathbf{2}^1$, 2x$\mathbf{2}^2$) **a** 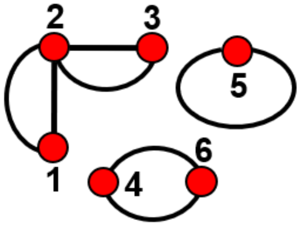
	(1x$\mathbf{2}$, 4x$\mathbf{2}^1$, 2x$\mathbf{2}^2$) **b** 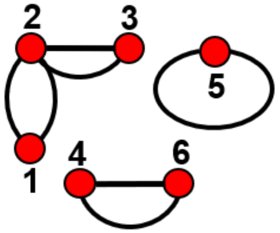	(1x$\mathbf{2}$, 4x$\mathbf{2}^1$, 2x$\mathbf{2}^2$) **c** 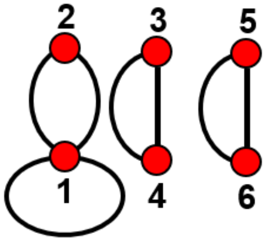	(1x$\mathbf{2}$, 4x$\mathbf{2}^1$, 2x$\mathbf{2}^2$) **d** 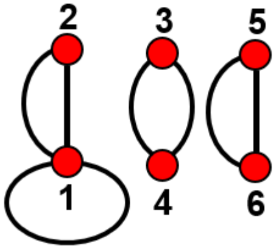	(1x$\mathbf{2}$, 2x$\mathbf{2}^1$, 4x$\mathbf{2}^2$) **a** 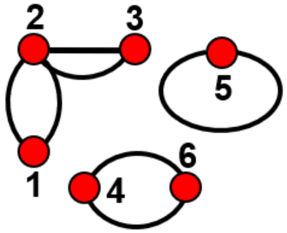

	(1x**2**, 2x$\mathbf{2}^1$, 4x$\mathbf{2}^2$) **b** 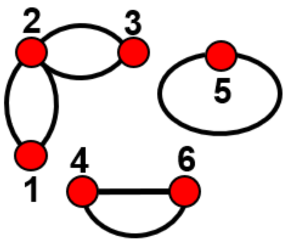	(1x**2**, 2x$\mathbf{2}^1$, 4x$\mathbf{2}^2$) **c** 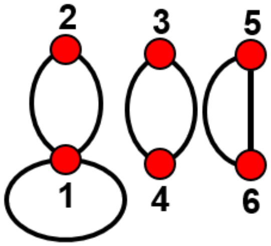	(1x**2**, 2x$\mathbf{2}^1$, 4x$\mathbf{2}^2$) **d** 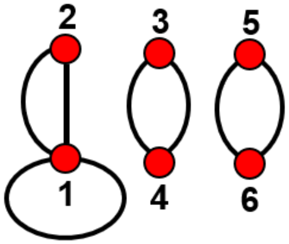	(1x**2**, 6x$\mathbf{2}^2$) **a** 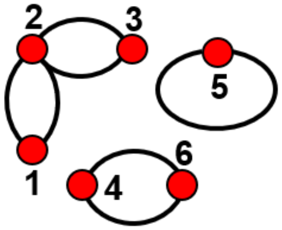
	(1x**2**, 6x$\mathbf{2}^2$) **b** 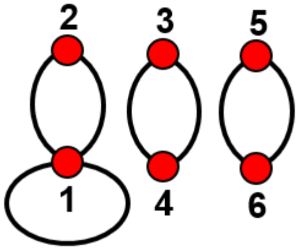			
$^2V_5{}^4V_2$	NG			
$^2V_5{}^4V_3$	NG			
$^2V_6{}^4V_1$	NG			
$^2V_6{}^4V_2$	NG			
$^2V_7{}^4V_1$	NG			
$\mathbf{^3V_r{}^4V_r}$				

$^3V_2{}^4V_1$	(4x**1**, 3x**2**) 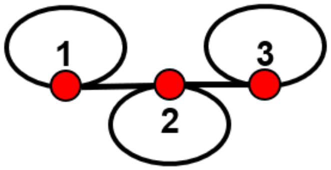	(4x**1**, 1x**2**, 2x**2**^{1}) 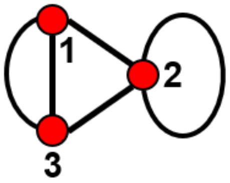	(4x**1**, 1x**2**, 2x**2**^{2}) 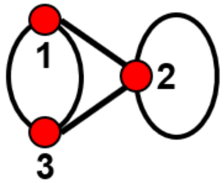	(2x**1**, 2x**2**, 2x**2**^{1}) 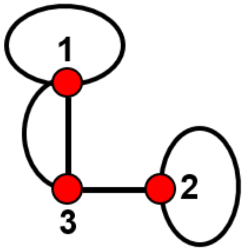
	(2x**1**, 2x**2**, 2x**2**^{2}) 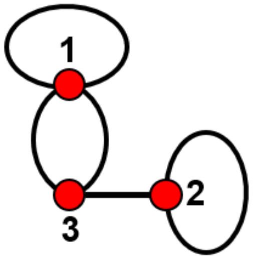	(2x**1**, 4x**2**^{1}) 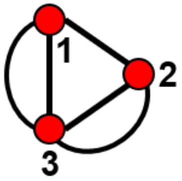	(2x**1**, 2x**2**^{1}, 2x**2**^{2}) 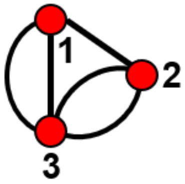	(2x**1**, 4x**2**^{2}) 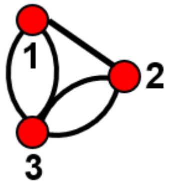
$^3V_2{}^4V_2$	(10x**1**, 2x**2**) 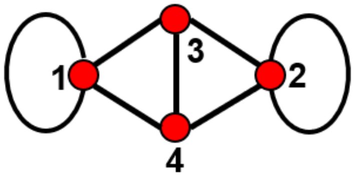	(10x**1**, 2x**2**^{1}) 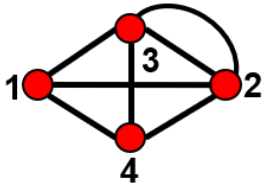	(10x**1**, 2x**2**^{2}) 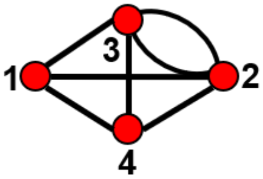	(8x**1**, 3x**2**) 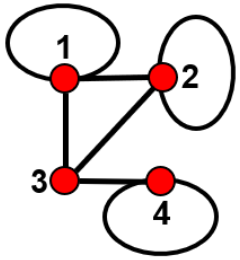

	(8x**1**, 1x**2**, 2x$\mathbf{2}^1$)<br>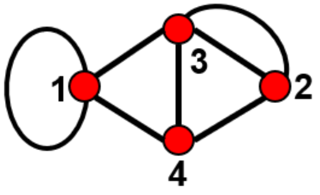	(8x**1**, 1x**2**, 2x$\mathbf{2}^2$)<br>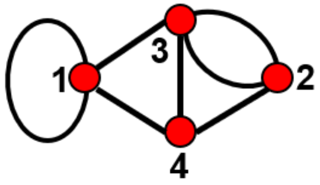	(6x**1**, 4x**2**)<br>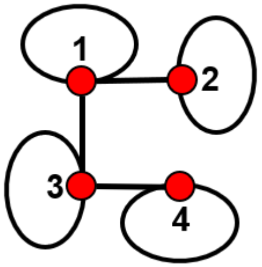	(6x**1**, 2x**2**, 2x$\mathbf{2}^1$) **a**<br>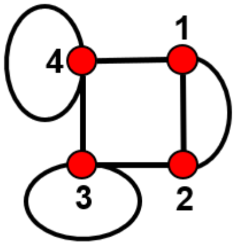
	(6x**1**, 2x**2**, 2x$\mathbf{2}^1$) **b**<br>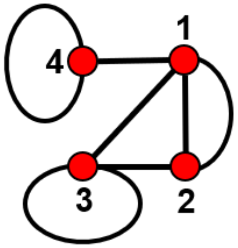	(6x**1**, 2x**2**, 2x$\mathbf{2}^2$) **a**<br>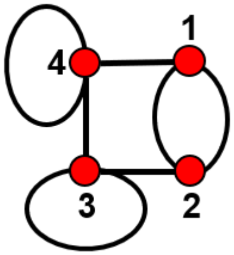	(6x**1**, 2x**2**, 2x$\mathbf{2}^2$) **b**<br>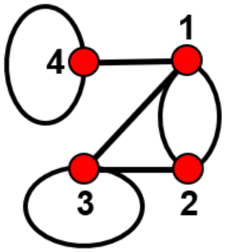	(6x**1**, 4x$\mathbf{2}^1$)<br>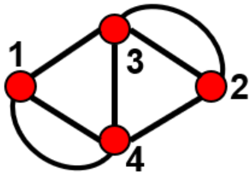
	(6x**1**, 2x$\mathbf{2}^1$, 2x$\mathbf{2}^2$)<br>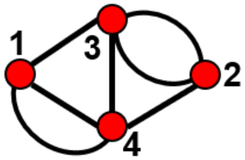	(6x**1**, 4x$\mathbf{2}^2$)<br>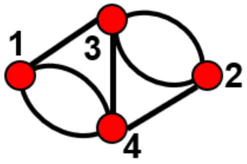	(4x**1**, 3x**2**, 2x$\mathbf{2}^1$) **a**<br>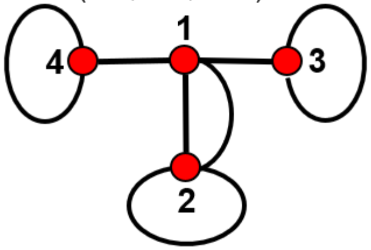	(4x**1**, 3x**2**, 2x$\mathbf{2}^1$) **b**<br>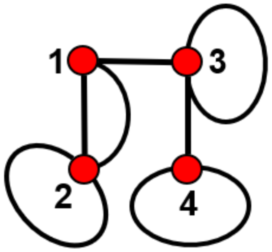

	(4x**1**, 3x**2**, 2x$\mathbf{2}^2$) **a** 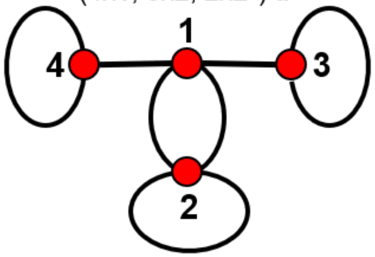	(4x**1**, 3x**2**, 2x$\mathbf{2}^2$) **b** 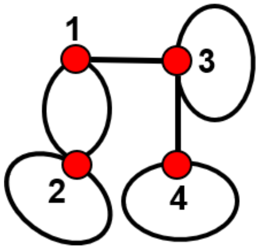	(4x**1**, 1x**2**, 4x$\mathbf{2}^1$) **a** 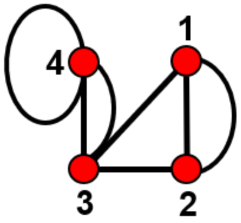	(4x**1**, 1x**2**, 4x$\mathbf{2}^1$) **b** 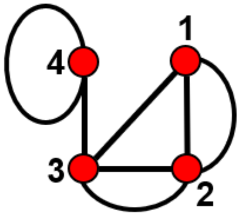
	(4x**1**, 1x**2**, 4x$\mathbf{2}^1$) **c** 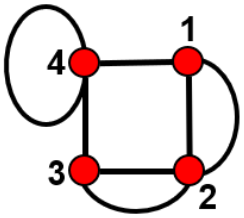	(4x**1**, 1x**2**, 2x$\mathbf{2}^1$, 2x$\mathbf{2}^2$) **a** 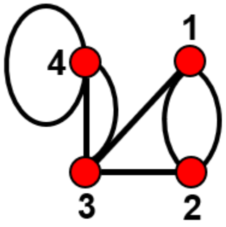	(4x**1**, 1x**2**, 2x$\mathbf{2}^1$, 2x$\mathbf{2}^2$) **b** 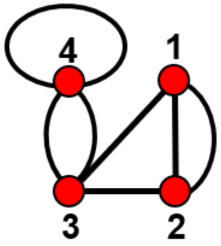	(4x**1**, 1x**2**, 2x$\mathbf{2}^1$, 2x$\mathbf{2}^2$) **c** 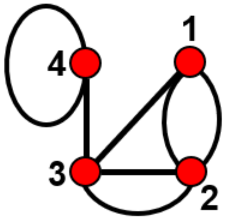
	(4x**1**, 1x**2**, 2x$\mathbf{2}^1$, 2x$\mathbf{2}^2$) **d** 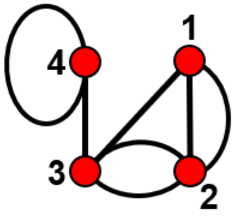	(4x**1**, 1x**2**, 2x$\mathbf{2}^1$, 2x$\mathbf{2}^2$) **e** 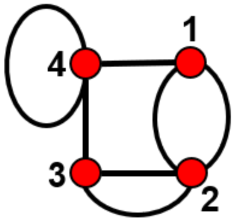	(4x**1**, 1x**2**, 4x$\mathbf{2}^2$) **a** 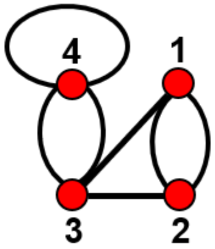	(4x**1**, 1x**2**, 4x$\mathbf{2}^2$) **b** 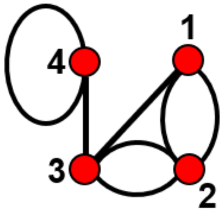

	(4x**1**, 1x**2**, 4x$\mathbf{2}^2$) **c**<br>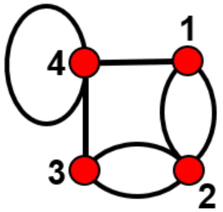	(2x**1**, 4x**2**, 2x$\mathbf{2}^1$)<br>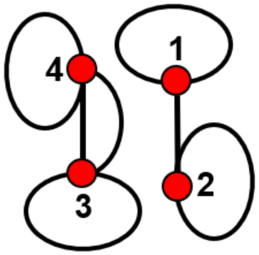	(2x**1**, 4x**2**, 2x$\mathbf{2}^2$)<br>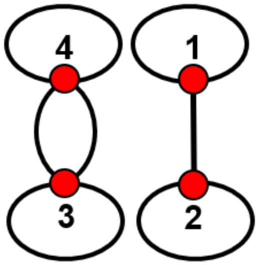	(2x**1**, 2x**2**, 4x$\mathbf{2}^1$) **a**<br>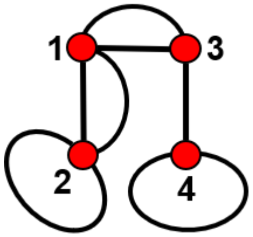
	(2x**1**, 2x**2**, 4x$\mathbf{2}^1$) **b**<br>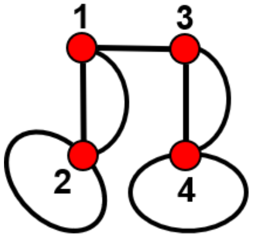	(2x**1**, 2x**2**, 2x$\mathbf{2}^1$, 2x$\mathbf{2}^2$) **a**<br>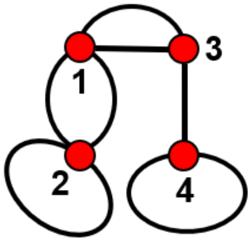	(2x**1**, 2x**2**, 2x$\mathbf{2}^1$, 2x$\mathbf{2}^2$) **b**<br>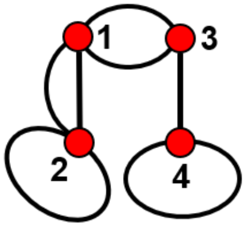	(2x**1**, 2x**2**, 2x$\mathbf{2}^1$, 2x$\mathbf{2}^2$) **c**<br>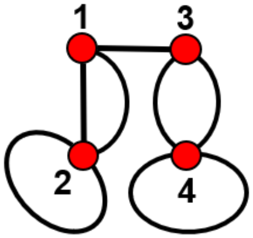
	(2x**1**, 2x**2**, 4x$\mathbf{2}^2$) **a**<br>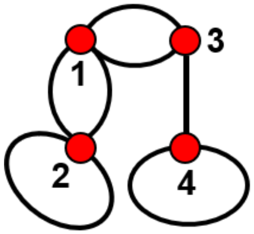	(2x**1**, 2x**2**, 4x$\mathbf{2}^2$) **b**<br>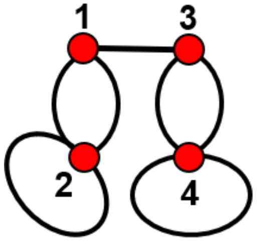	(2x**1**, 6x$\mathbf{2}^1$)<br>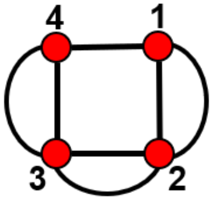	(2x**1**, 4x$\mathbf{2}^1$, 2x$\mathbf{2}^2$) **a**<br>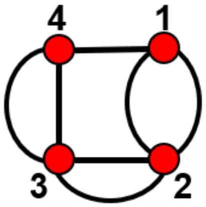
	(2x**1**, 4x$\mathbf{2}^1$, 2x$\mathbf{2}^2$) **b**<br>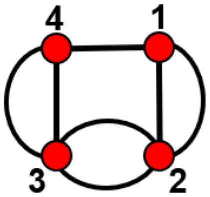	(2x**1**, 2x$\mathbf{2}^1$, 4x$\mathbf{2}^2$) **a**<br>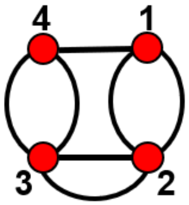	(2x**1**, 2x$\mathbf{2}^1$, 4x$\mathbf{2}^2$) **b**<br>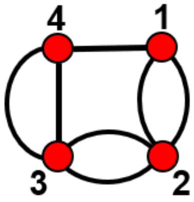	(2x**1**, 6x$\mathbf{2}^2$)<br>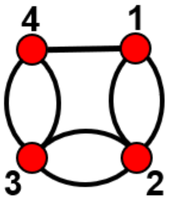

$^3V_2{}^4V_3$	(18x**1**)<br>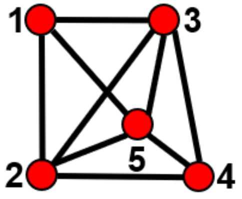	(16x**1**, 1x**2**)<br>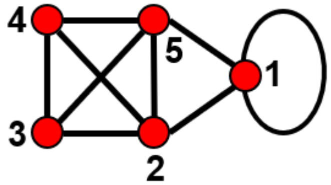	(14x**1**, 2x**2**)<br>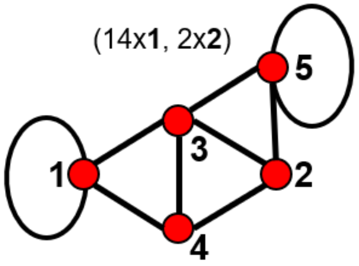	(14x**1**, 2x$\mathbf{2}^1$)<br>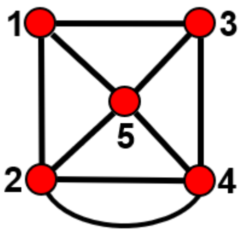
	(14x**1**, 2x$\mathbf{2}^2$)<br>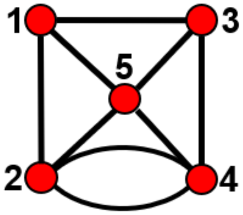	(12x**1**, 3x**2**) **a**<br>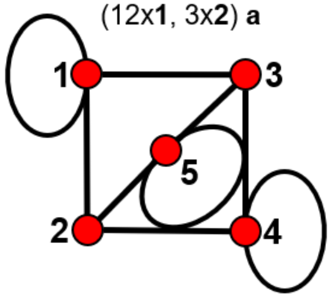	(12x**1**, 3x**2**) **b**<br>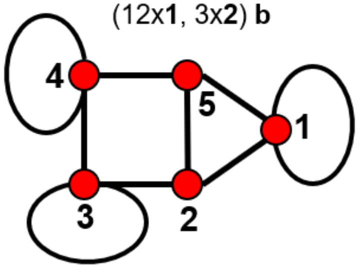	(12x**1**, 3x**2**) **c**<br>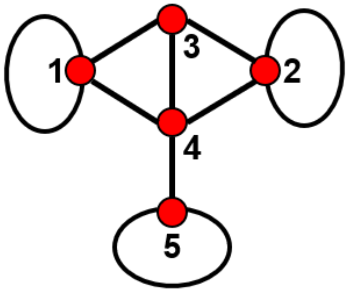
	(12x**1**, 1x**2**, 2x$\mathbf{2}^1$) **a**<br>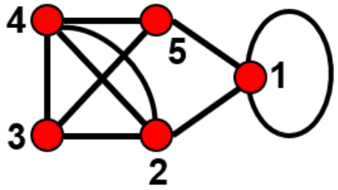	(12x**1**, 1x**2**, 2x$\mathbf{2}^1$) **b**<br>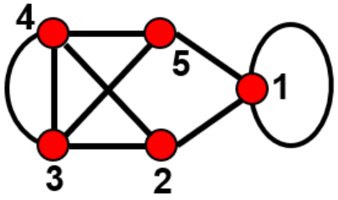	(12x**1**, 1x**2**, 2x$\mathbf{2}^1$) **c**<br>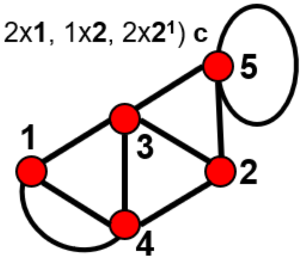	(12x**1**, 1x**2**, 2x$\mathbf{2}^1$) **d**<br>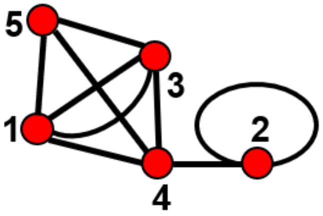

(12x**1**, 1x**2**, 2x$\mathbf{2}^{2}$) **a**

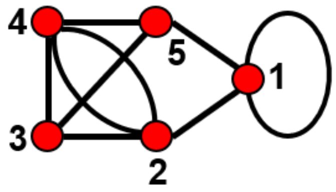

(12x**1**, 1x**2**, 2x$\mathbf{2}^{2}$) **b**

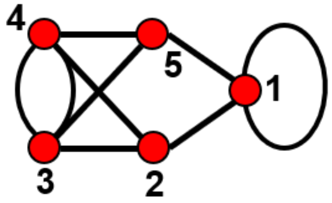

(12x**1**, 1x**2**, 2x$\mathbf{2}^{2}$) **c**

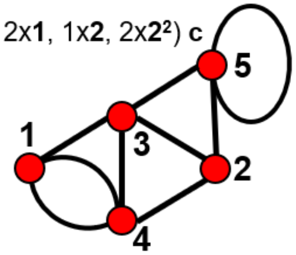

(12x**1**, 1x**2**, 2x$\mathbf{2}^{2}$) **d**

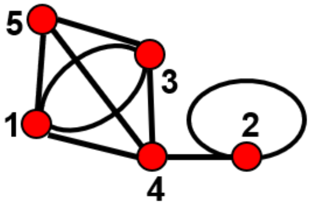

(10x**1**, 4x**2**) **a**

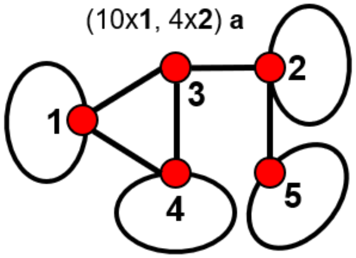

(10x**1**, 4x**2**) **b**

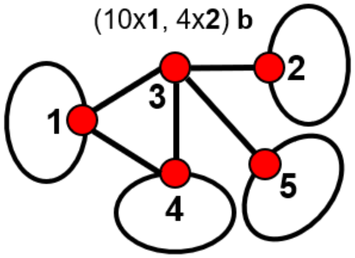

(10x**1**, 4x**2**) **c**

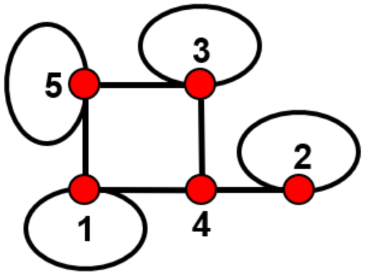

(10x**1**, 2x**2**, 2x$\mathbf{2}^{1}$) **a**

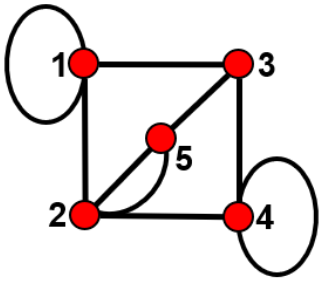

(10x**1**, 2x**2**, 2x$\mathbf{2}^{1}$) **b**

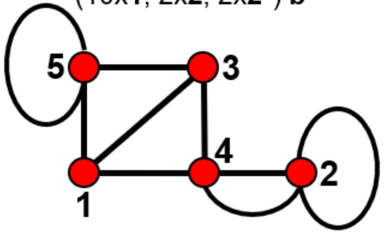

(10x**1**, 2x**2**, 2x$\mathbf{2}^{1}$) **c**

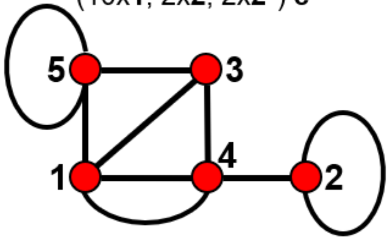

(10x**1**, 2x**2**, 2x$\mathbf{2}^{1}$) **d**

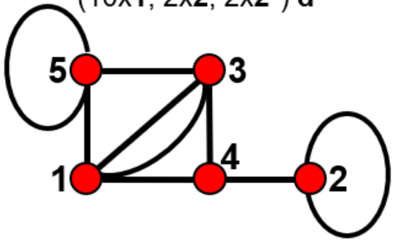

(10x**1**, 2x**2**, 2x$\mathbf{2}^{1}$) **e**

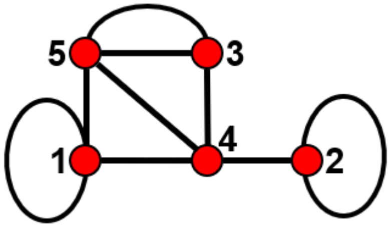

	(10x**1**, 2x**2**, 2x**2**^{1}) **f** 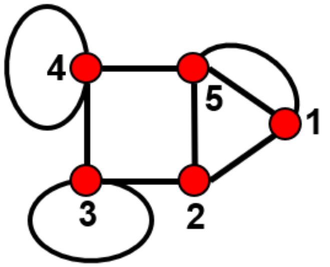	(10x**1**, 2x**2**, 2x**2**^{1}) **g** 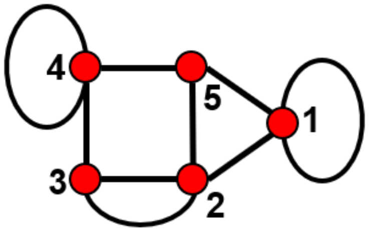	(10x**1**, 2x**2**, 2x**2**^{1}) **h** 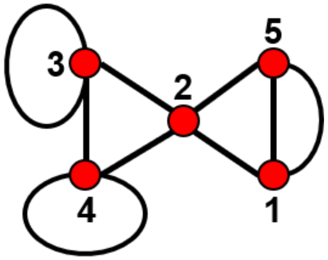	(10x**1**, 2x**2**, 2x**2**^{2}) **a** 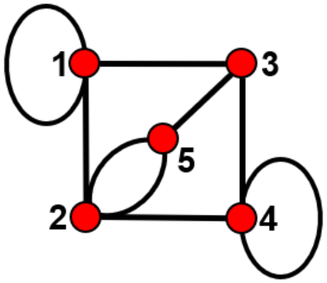
	(10x**1**, 2x**2**, 2x**2**^{2}) **b** 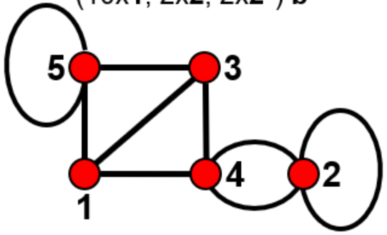	(10x**1**, 2x**2**, 2x**2**^{2}) **c** 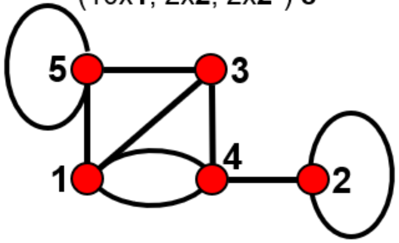	(10x**1**, 2x**2**, 2x**2**^{2}) **d** 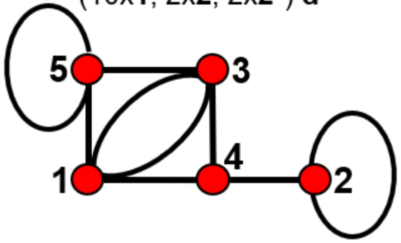	(10x**1**, 2x**2**, 2x**2**^{2}) **e** 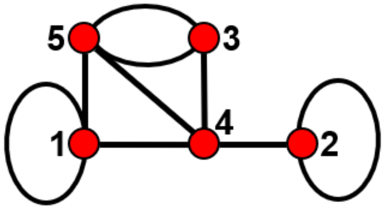
	(10x**1**, 2x**2**, 2x**2**^{2}) **f** 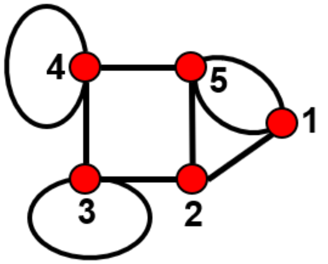	(10x**1**, 2x**2**, 2x**2**^{2}) **g** 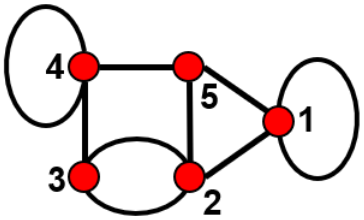	(10x**1**, 2x**2**, 2x**2**^{2}) **h** 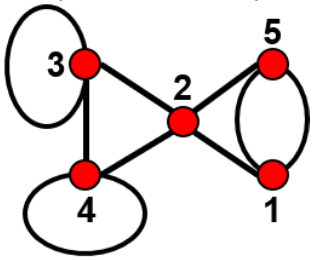	(10x**1**, 4x**2**^{1}) **a** 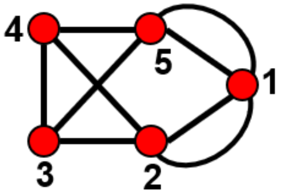

	(10x**1**, 4x**2**^{1}) **b** 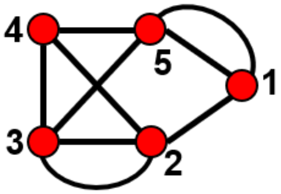	(10x**1**, 4x**2**^{1}) **c** 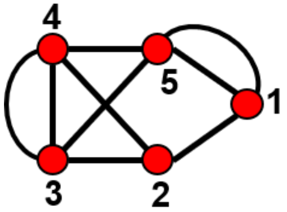	(10x**1**, 4x**2**^{1}) **d** 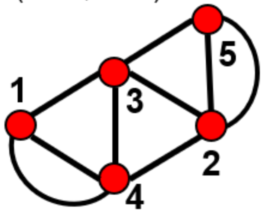	(10x**1**, 2x**2**^{1}, 2x**2**^{2}) **a** 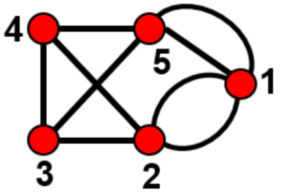
	(10x**1**, 2x**2**^{1}, 2x**2**^{2}) **b** 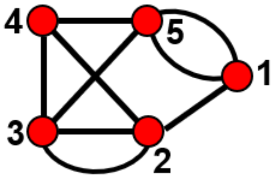	(10x**1**, 2x**2**^{1}, 2x**2**^{2}) **c** 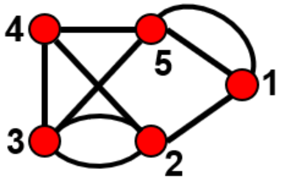	(10x**1**, 2x**2**^{1}, 2x**2**^{2}) **d** 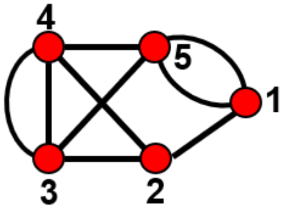	(10x**1**, 2x**2**^{1}, 2x**2**^{2}) **e** 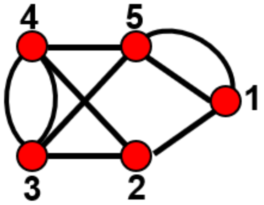
	(10x**1**, 2x**2**^{1}, 2x**2**^{2}) **f** 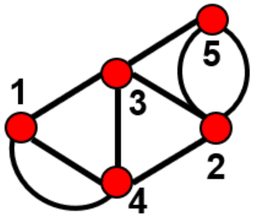	(10x**1**, 4x**2**^{2}) **a** 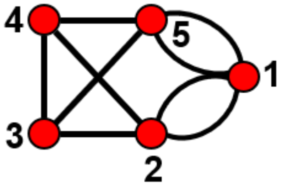	(10x**1**, 4x**2**^{2}) **b** 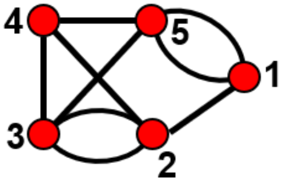	(10x**1**, 4x**2**^{2}) **c** 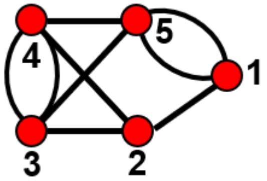
	(10x**1**, 4x**2**^{2}) **d** 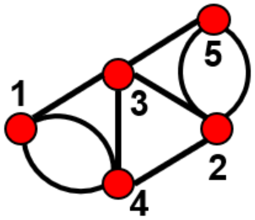	(8x**1**, 5x**2**) **a** 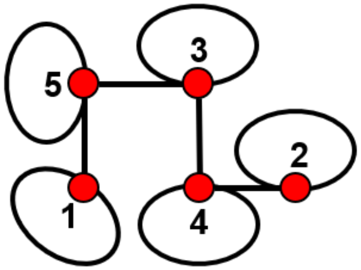	(8x**1**, 5x**2**) **b** 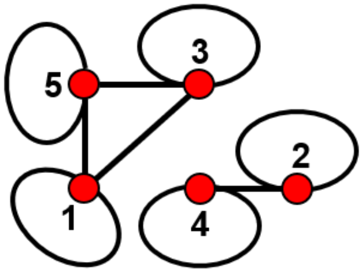	(8x**1**, 3x**2**, 2x**2**^{1}) **a** 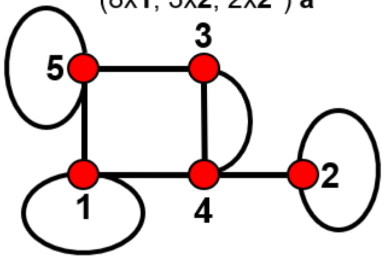

	(8x**1**, 3x**2**, 2x**2**^{1}) **b** 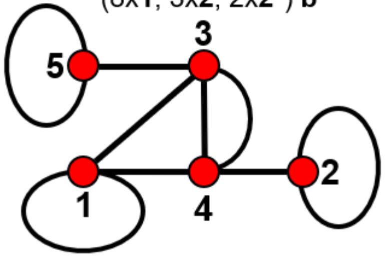	(8x**1**, 3x**2**, 2x**2**^{1}) **c** 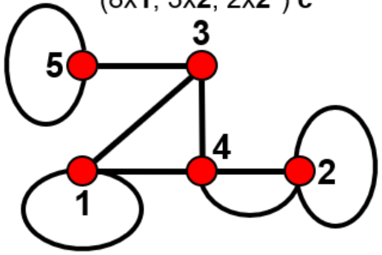	(8x**1**, 3x**2**, 2x**2**^{1}) **d** 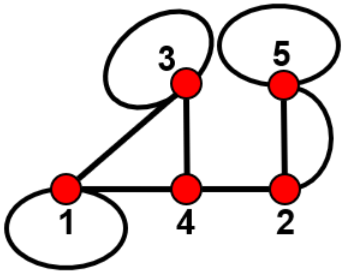	(8x**1**, 3x**2**, 2x**2**^{1}) **e** 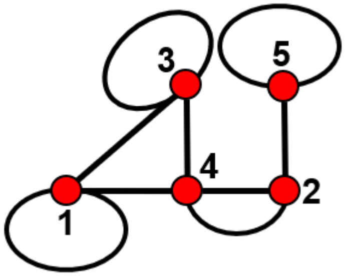
	(8x**1**, 3x**2**, 2x**2**^{1}) **f** 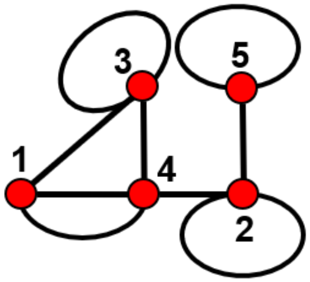	(8x**1**, 3x**2**, 2x**2**^{1}) **g** 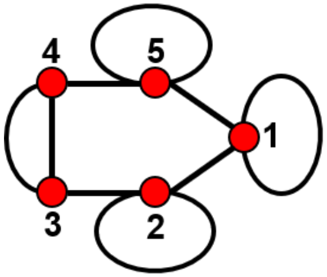	(8x**1**, 3x**2**, 2x**2**^{2}) **a** 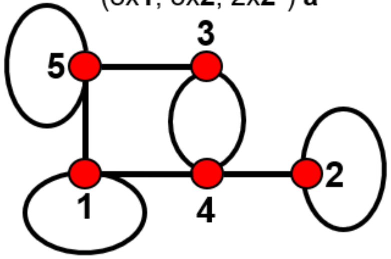	(8x**1**, 3x**2**, 2x**2**^{2}) **b** 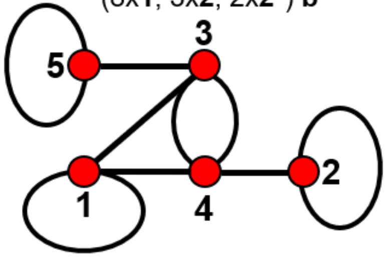
	(8x**1**, 3x**2**, 2x**2**^{2}) **c** 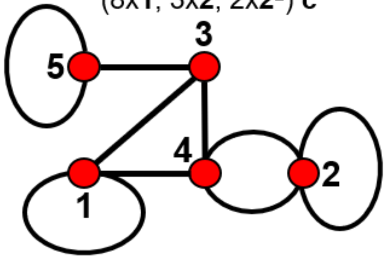	(8x**1**, 3x**2**, 2x**2**^{2}) **d** 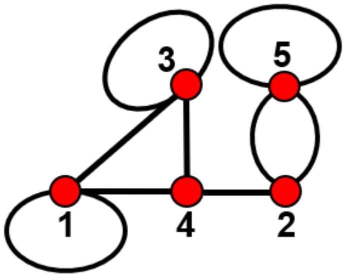	(8x**1**, 3x**2**, 2x**2**^{2}) **e** 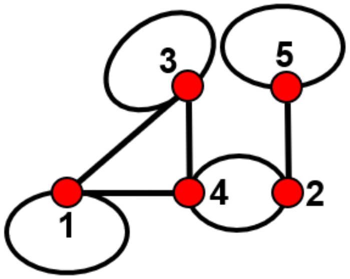	(8x**1**, 3x**2**, 2x**2**^{2}) **f** 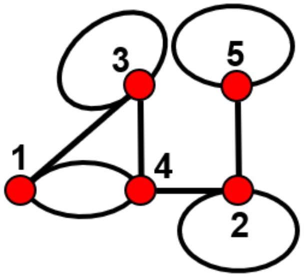

	(8x**1**, 3x**2**, 2x**2**^{2}) **g** 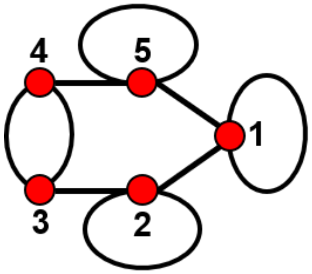	(8x**1**, 1x**2**, 4x**2**^{1}) **a** 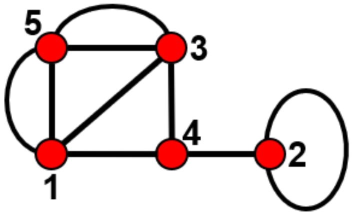	(8x**1**, 1x**2**, 4x**2**^{1}) **b** 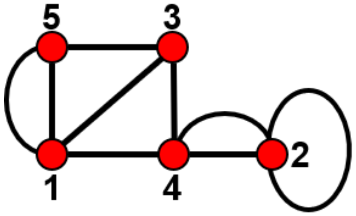	(8x**1**, 1x**2**, 4x**2**^{1}) **c** 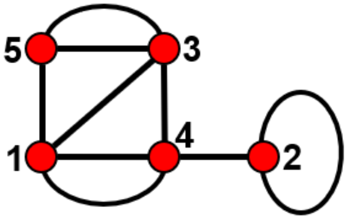
	(8x**1**, 1x**2**, 4x**2**^{1}) **d** 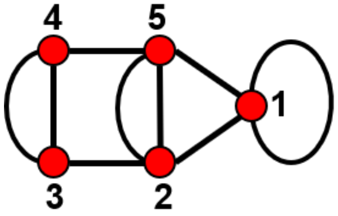	(8x**1**, 1x**2**, 4x**2**^{1}) **e** 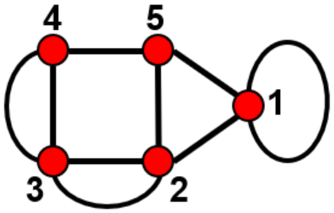	(8x**1**, 1x**2**, 4x**2**^{1}) **f** 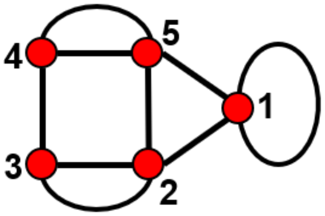	(8x**1**, 1x**2**, 4x**2**^{1}) **g** 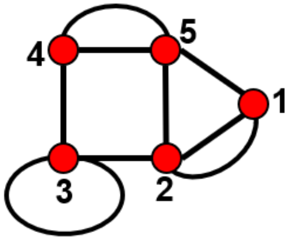
	(8x**1**, 1x**2**, 4x**2**^{1}) **h** 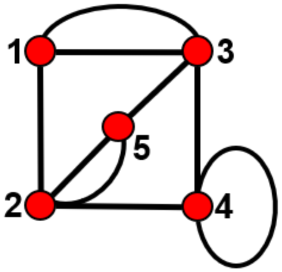	(8x**1**, 1x**2**, 2x**2**^{1}, 2x**2**^{2}) **a** 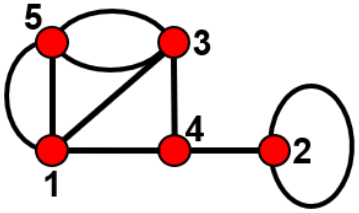	(8x**1**, 1x**2**, 2x**2**^{1}, 2x**2**^{2}) **b** 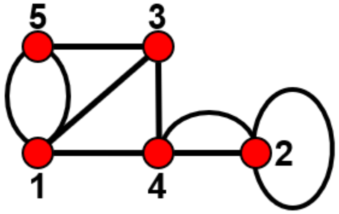	(8x**1**, 1x**2**, 2x**2**^{1}, 2x**2**^{2}) **c** 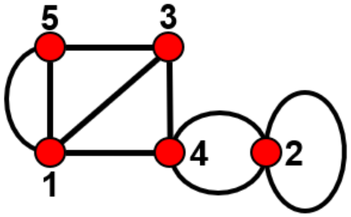

	(8x**1**, 1x**2**, 2x$\mathbf{2}^1$, 2x$\mathbf{2}^2$) **d** 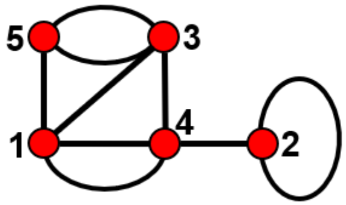	(8x**1**, 1x**2**, 2x$\mathbf{2}^1$, 2x$\mathbf{2}^2$) **e** 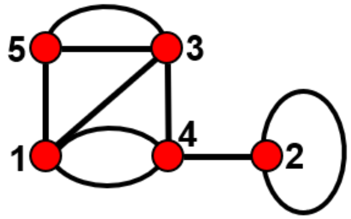	(8x**1**, 1x**2**, 2x$\mathbf{2}^1$, 2x$\mathbf{2}^2$) **f** 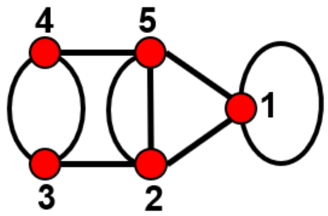	(8x**1**, 1x**2**, 2x$\mathbf{2}^1$, 2x$\mathbf{2}^2$) **g** 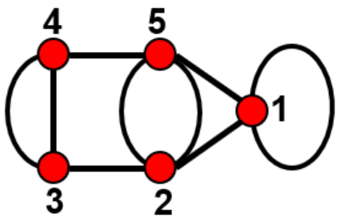
	(8x**1**, 1x**2**, 2x$\mathbf{2}^1$, 2x$\mathbf{2}^2$) **h** 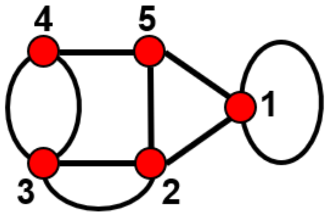	(8x**1**, 1x**2**, 2x$\mathbf{2}^1$, 2x$\mathbf{2}^2$) **i** 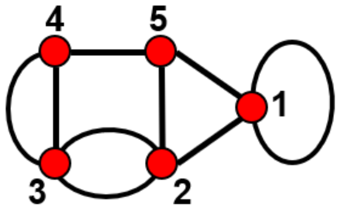	(8x**1**, 1x**2**, 2x$\mathbf{2}^1$, 2x$\mathbf{2}^2$) **j** 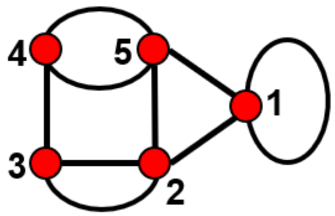	(8x**1**, 1x**2**, 2x$\mathbf{2}^1$, 2x$\mathbf{2}^2$) **k** 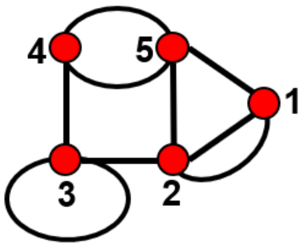
	(8x**1**, 1x**2**, 2x$\mathbf{2}^1$, 2x$\mathbf{2}^2$) **l** 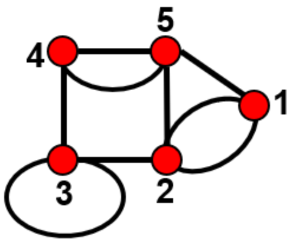	(8x**1**, 1x**2**, 2x$\mathbf{2}^1$, 2x$\mathbf{2}^2$) **m** 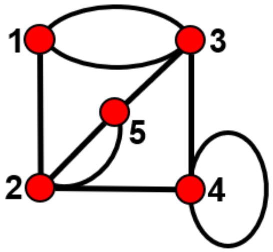	(8x**1**, 1x**2**, 2x$\mathbf{2}^1$, 2x$\mathbf{2}^2$) **n** 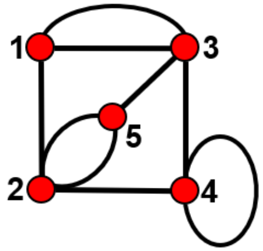	(8x**1**, 1x**2**, 4x$\mathbf{2}^2$) **a** 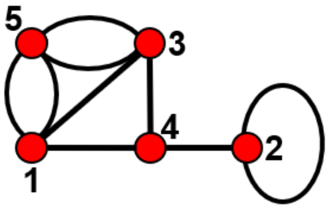
	(8x**1**, 1x**2**, 4x$\mathbf{2}^2$) **b** 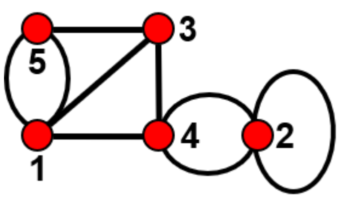	(8x**1**, 1x**2**, 4x$\mathbf{2}^2$) **c** 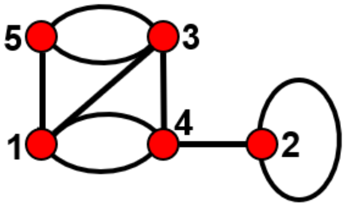	(8x**1**, 1x**2**, 4x$\mathbf{2}^2$) **d** 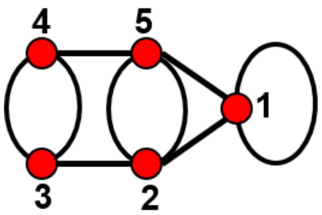	(8x**1**, 1x**2**, 4x$\mathbf{2}^2$) **e** 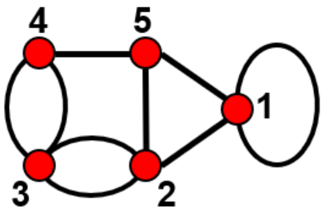

	(8x**1**, 1x**2**, 4x$\mathbf{2}^2$) **f** 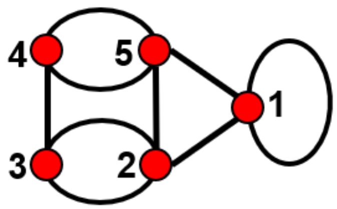	(8x**1**, 1x**2**, 4x$\mathbf{2}^2$) **g** 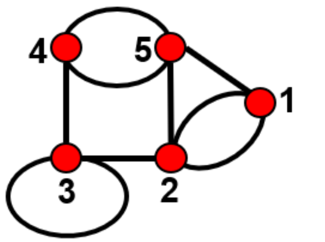	(8x**1**, 1x**2**, 4x$\mathbf{2}^2$) **h** 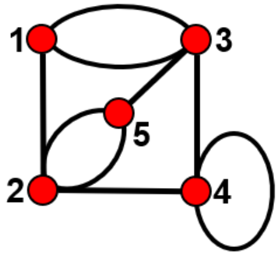	(6x**1**, 4x**2**, 2x$\mathbf{2}^1$) **a** 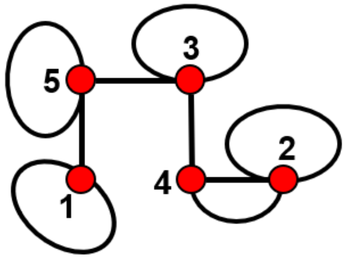
	(6x**1**, 4x**2**, 2x$\mathbf{2}^1$) **b** 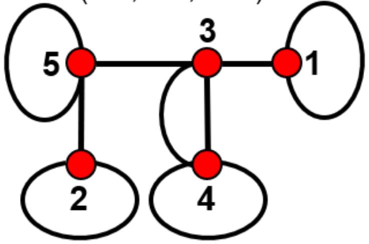	(6x**1**, 4x**2**, 2x$\mathbf{2}^2$) **a** 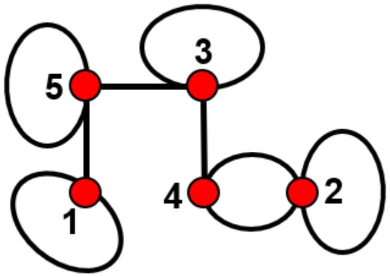	(6x**1**, 4x**2**, 2x$\mathbf{2}^2$) **b** 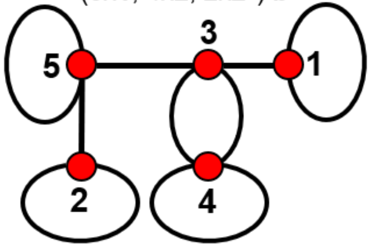	(6x**1**, 2x**2**, 4x$\mathbf{2}^1$) **a** 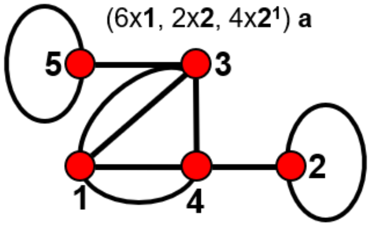
	(6x**1**, 2x**2**, 4x$\mathbf{2}^1$) **b** 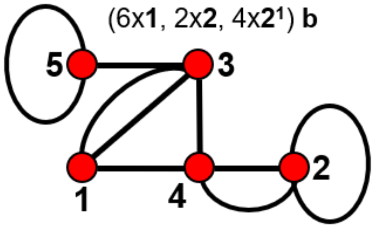	(6x**1**, 2x**2**, 4x$\mathbf{2}^1$) **c** 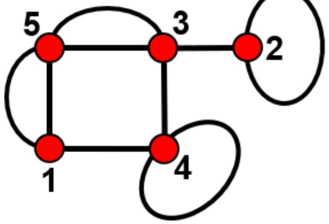	(6x**1**, 2x**2**, 4x$\mathbf{2}^1$) **d** 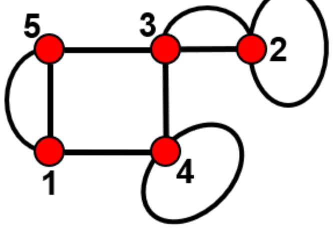	(6x**1**, 2x**2**, 4x$\mathbf{2}^1$) **e** 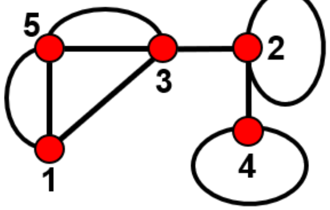
	(6x**1**, 2x**2**, 4x$\mathbf{2}^1$) **f** 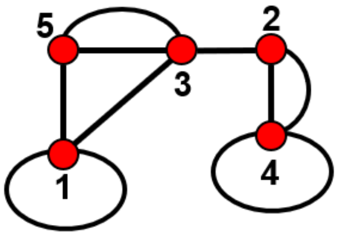	(6x**1**, 2x**2**, 4x$\mathbf{2}^1$) **g** 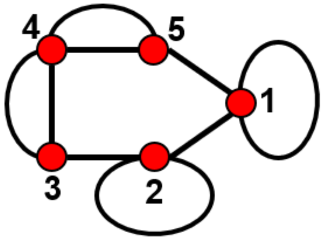	(6x**1**, 2x**2**, 2x$\mathbf{2}^1$, 2x$\mathbf{2}^2$) **a** 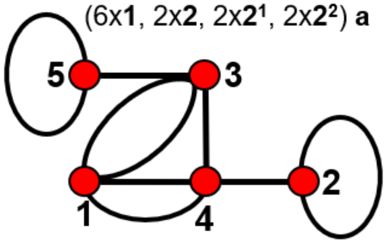	(6x**1**, 2x**2**, 2x$\mathbf{2}^1$, 2x$\mathbf{2}^2$) **b** 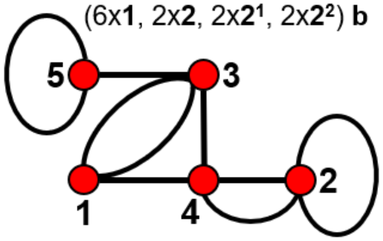

(6x**1**, 2x**2**, 2x$\mathbf{2}^1$, 2x$\mathbf{2}^2$) **c**

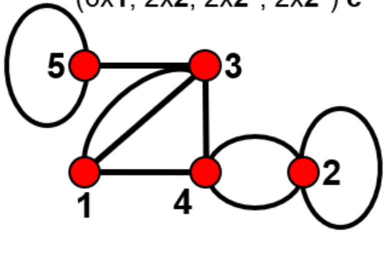

(6x**1**, 2x**2**, 2x$\mathbf{2}^1$, 2x$\mathbf{2}^2$) **d**

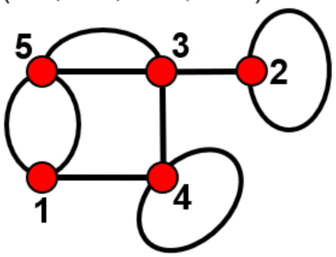

(6x**1**, 2x**2**, 2x$\mathbf{2}^1$, 2x$\mathbf{2}^2$) **e**

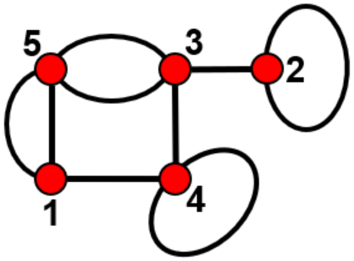

(6x**1**, 2x**2**, 2x$\mathbf{2}^1$, 2x$\mathbf{2}^2$) **f**

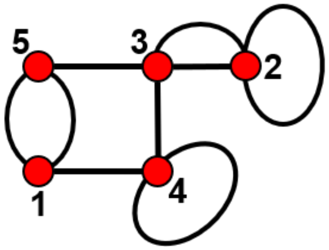

(6x**1**, 2x**2**, 2x$\mathbf{2}^1$, 2x$\mathbf{2}^2$) **g**

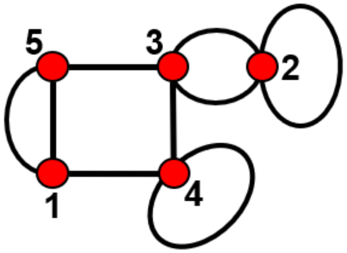

(6x**1**, 2x**2**, 2x$\mathbf{2}^1$, 2x$\mathbf{2}^2$) **h**

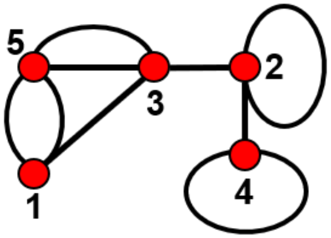

(6x**1**, 2x**2**, 2x$\mathbf{2}^1$, 2x$\mathbf{2}^2$) **i**

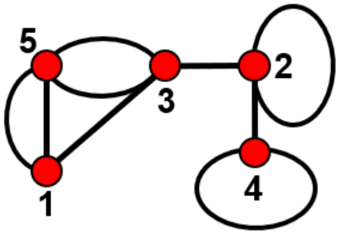

(6x**1**, 2x**2**, 2x$\mathbf{2}^1$, 2x$\mathbf{2}^2$) **j**

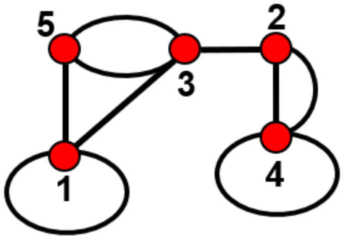

(6x**1**, 2x**2**, 2x$\mathbf{2}^1$, 2x$\mathbf{2}^2$) **k**

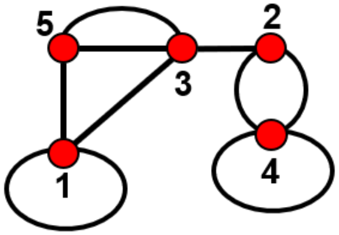

(6x**1**, 2x**2**, 2x$\mathbf{2}^1$, 2x$\mathbf{2}^2$) **l**

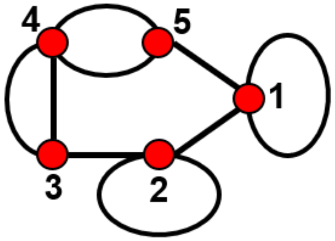

(6x**1**, 2x**2**, 4x$\mathbf{2}^2$) **a**

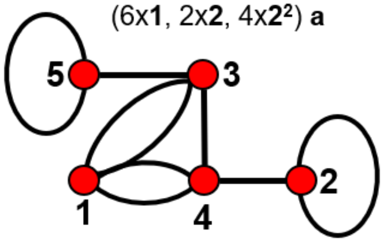

(6x**1**, 2x**2**, 4x$\mathbf{2}^2$) **b**

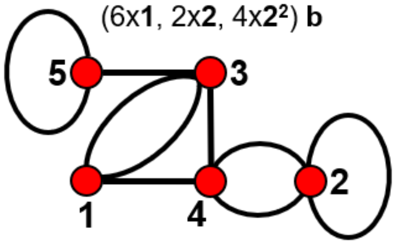

(6x**1**, 2x**2**, 4x$\mathbf{2}^2$) **c**

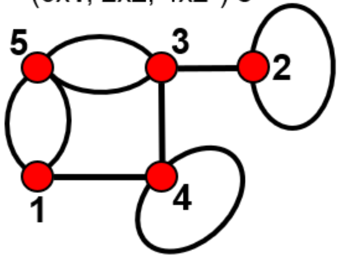

(6x**1**, 2x**2**, 4x$\mathbf{2}^2$) **d**

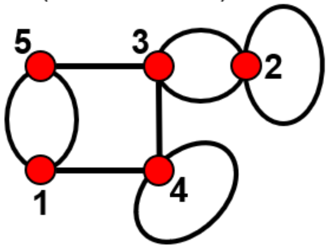

(6x**1**, 2x**2**, 4x$\mathbf{2}^2$) **e**

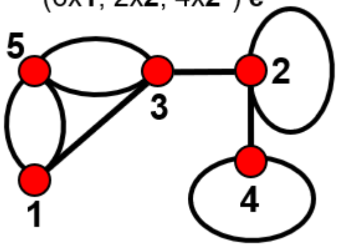

(6x**1**, 2x**2**, 4x$\mathbf{2}^2$) **f**

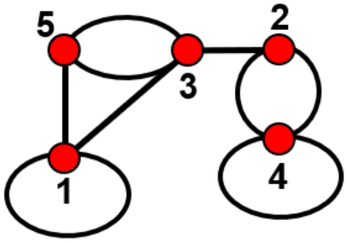

	(6x**1**, 2x**2**, 4x**2**^{2}) g 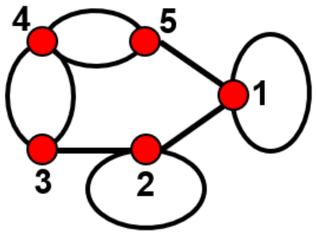	(6x**1**, 6x**2**^{1}) **a** 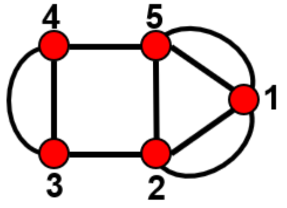	(6x**1**, 6x**2**^{1}) **b** 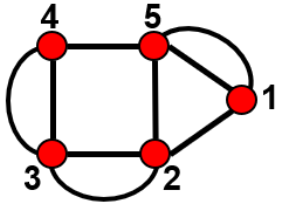	(6x**1**, 4x**2**^{1}, 2x**2**^{2}) **a** 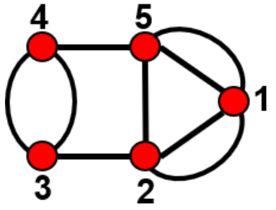
	(6x**1**, 4x**2**^{1}, 2x**2**^{2}) **b** 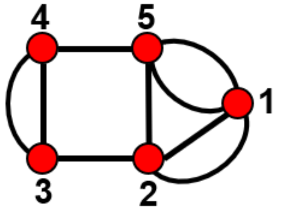	(6x**1**, 4x**2**^{1}, 2x**2**^{2}) **c** 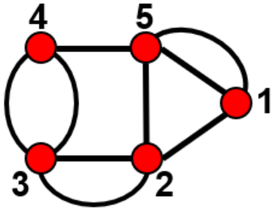	(6x**1**, 4x**2**^{1}, 2x**2**^{2}) **d** 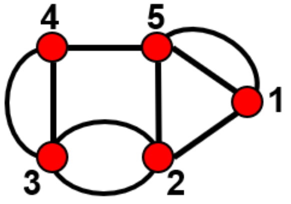	(6x**1**, 4x**2**^{1}, 2x**2**^{2}) **e** 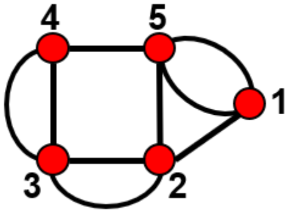
	(6x**1**, 2x**2**^{1}, 4x**2**^{2}) **a** 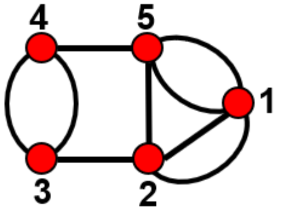	(6x**1**, 2x**2**^{1}, 4x**2**^{2}) **b** 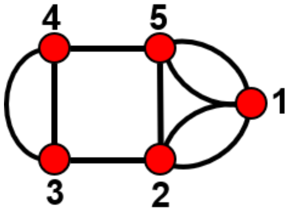	(6x**1**, 2x**2**^{1}, 4x**2**^{2}) **c** 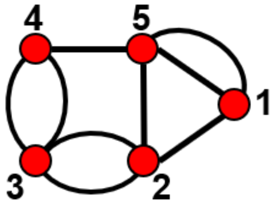	(6x**1**, 2x**2**^{1}, 4x**2**^{2}) **d** 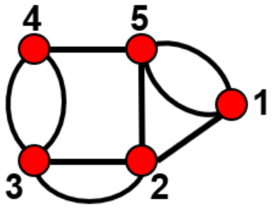
	(6x**1**, 2x**2**^{1}, 4x**2**^{2}) **e** 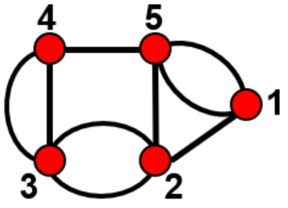	(6x**1**, 6x**2**^{2}) **a** 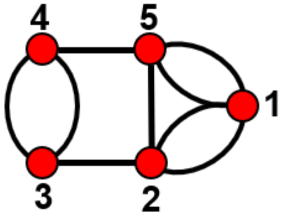	(6x**1**, 6x**2**^{2}) **b** 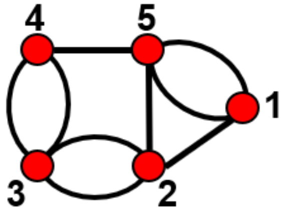	(4x**1**, 5x**2**, 2x**2**^{1}) 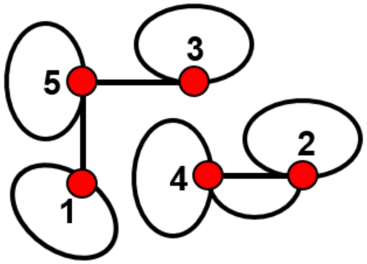

<table>
<tr>
<td></td>
<td>
(4x**1**, 5x**2**, 2x$\mathbf{2}^{2}$)
</td>
<td>
(4x**1**, 3x**2**, 4x$\mathbf{2}^{1}$) **a**
</td>
<td>
(4x**1**, 3x**2**, 4x$\mathbf{2}^{1}$) **b**
</td>
<td>
(4x**1**, 3x**2**, 4x$\mathbf{2}^{1}$) **c**
</td>
</tr>
<tr>
<td></td>
<td>
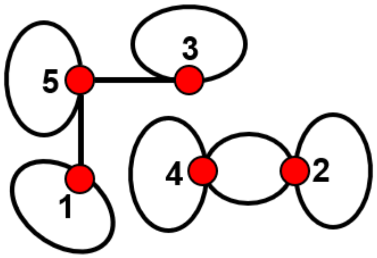
</td>
<td>
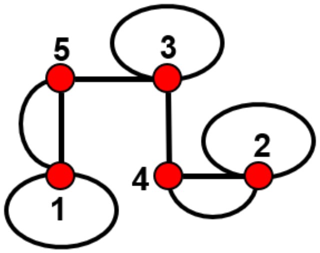
</td>
<td>
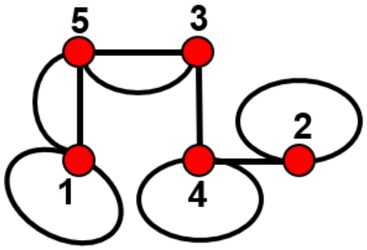
</td>
<td>
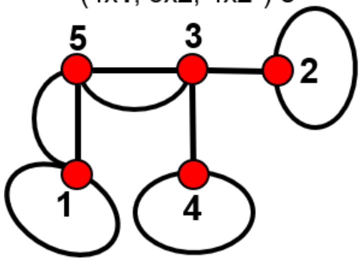
</td>
</tr>
<tr>
<td></td>
<td>
(4x**1**, 3x**2**, 4x$\mathbf{2}^{1}$) **d**
</td>
<td>
(4x**1**, 3x**2**, 4x$\mathbf{2}^{1}$) **e**
</td>
<td>
(4x**1**, 3x**2**, 2x$\mathbf{2}^{1}$, 2x$\mathbf{2}^{2}$) **a**
</td>
<td>
(4x**1**, 3x**2**, 2x$\mathbf{2}^{1}$, 2x$\mathbf{2}^{2}$) **b**
</td>
</tr>
<tr>
<td></td>
<td>
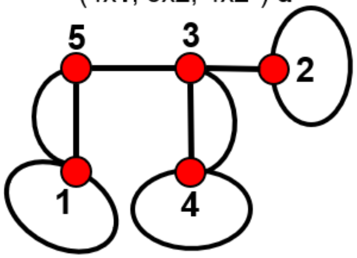
</td>
<td>
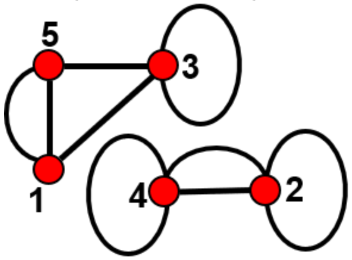
</td>
<td>
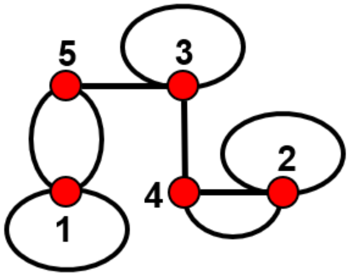
</td>
<td>
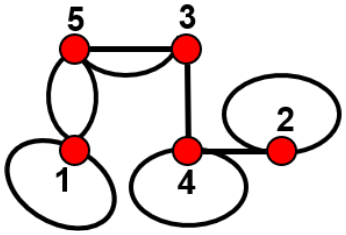
</td>
</tr>
<tr>
<td></td>
<td>
(4x**1**, 3x**2**, 2x$\mathbf{2}^{1}$, 2x$\mathbf{2}^{2}$) **c**
</td>
<td>
(4x**1**, 3x**2**, 2x$\mathbf{2}^{1}$, 2x$\mathbf{2}^{2}$) **d**
</td>
<td>
(4x**1**, 3x**2**, 2x$\mathbf{2}^{1}$, 2x$\mathbf{2}^{2}$) **e**
</td>
<td>
(4x**1**, 3x**2**, 2x$\mathbf{2}^{1}$, 2x$\mathbf{2}^{2}$) **f**
</td>
</tr>
<tr>
<td></td>
<td>
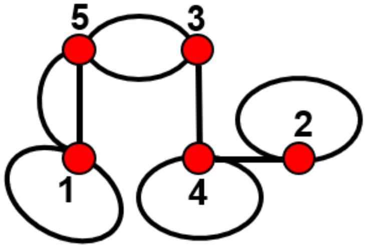
</td>
<td>
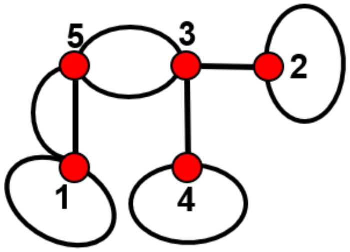
</td>
<td>
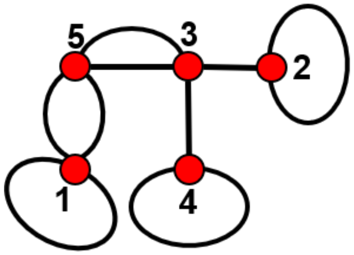
</td>
<td>
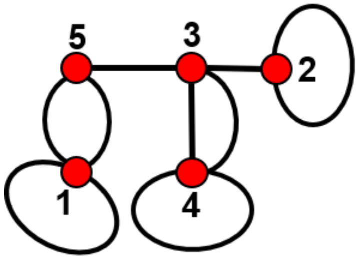
</td>
</tr>
</table>

(4x**1**, 3x**2**, 2x$\mathbf{2}^{1}$, 2x$\mathbf{2}^{2}$) **g**

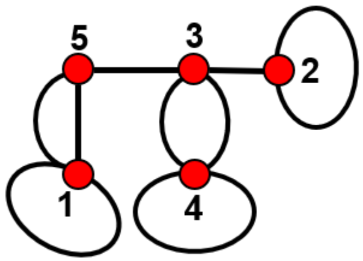

(4x**1**, 3x**2**, 2x$\mathbf{2}^{1}$, 2x$\mathbf{2}^{2}$) **h**

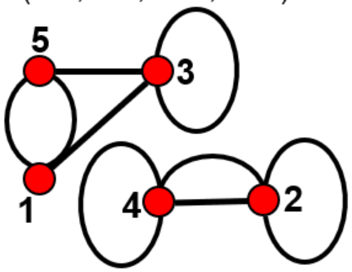

(4x**1**, 3x**2**, 2x$\mathbf{2}^{1}$, 2x$\mathbf{2}^{2}$) **i**

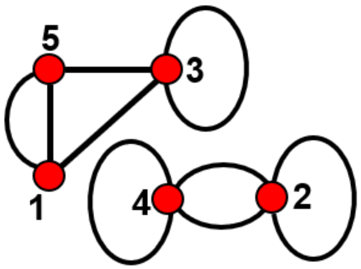

(4x**1**, 3x**2**, 4x$\mathbf{2}^{2}$) **a**

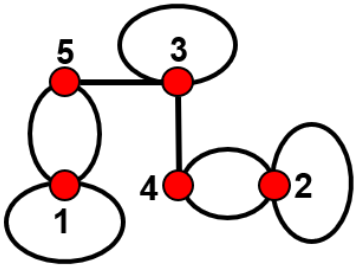

(4x**1**, 3x**2**, 4x$\mathbf{2}^{2}$) **b**

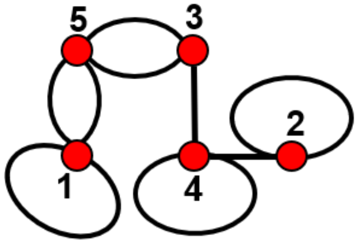

(4x**1**, 3x**2**, 4x$\mathbf{2}^{2}$) **c**

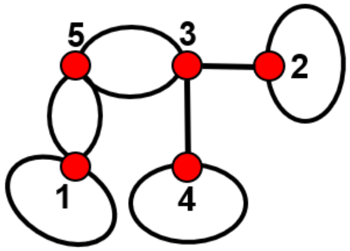

(4x**1**, 3x**2**, 4x$\mathbf{2}^{2}$) **d**

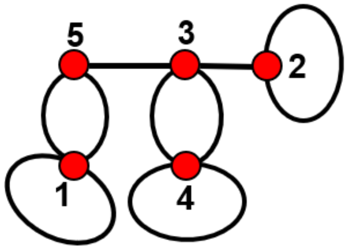

(4x**1**, 3x**2**, 4x$\mathbf{2}^{2}$) **e**

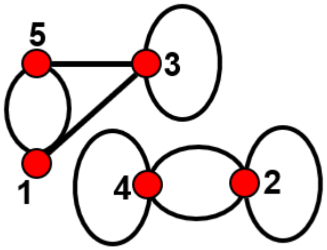

(4x**1**, 1x**2**, 6x$\mathbf{2}^{1}$) **a**

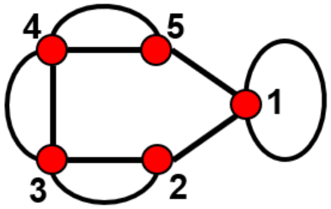

(4x**1**, 1x**2**, 6x$\mathbf{2}^{1}$) **b**

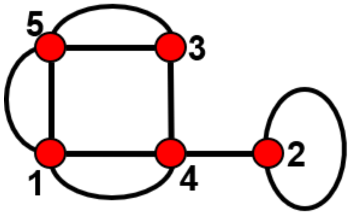

(4x**1**, 1x**2**, 6x$\mathbf{2}^{1}$) **c**

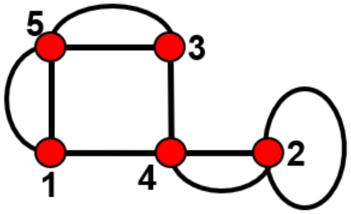

(4x**1**, 1x**2**, 6x$\mathbf{2}^{1}$) **d**

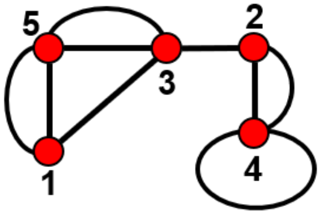

(4x**1**, 1x**2**, 6x$\mathbf{2}^{1}$) **e**

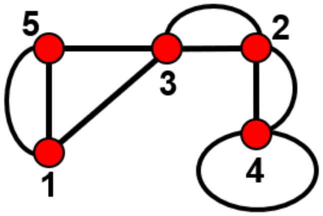

(4x**1**, 1x**2**, 4x$\mathbf{2}^{1}$, 2x$\mathbf{2}^{2}$) **a**

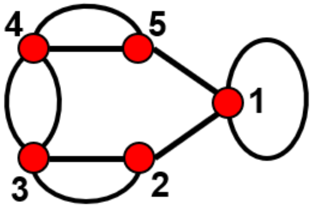

(4x**1**, 1x**2**, 4x$\mathbf{2}^{1}$, 2x$\mathbf{2}^{2}$) **b**

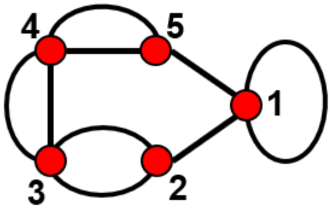

(4x**1**, 1x**2**, 4x$\mathbf{2}^{1}$, 2x$\mathbf{2}^{2}$) **c**

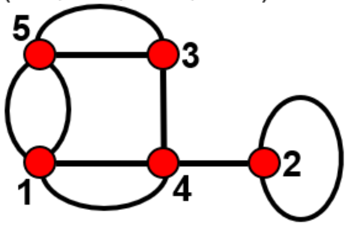

	(4x**1**, 1x**2**, 4x**2**^{1}, 2x**2**^{2}) **d** 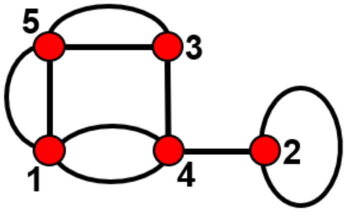	(4x**1**, 1x**2**, 4x**2**^{1}, 2x**2**^{2}) **e** 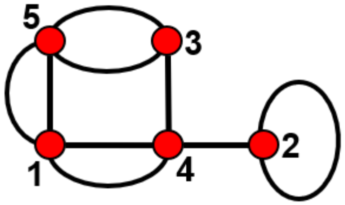	(4x**1**, 1x**2**, 4x**2**^{1}, 2x**2**^{2}) **f** 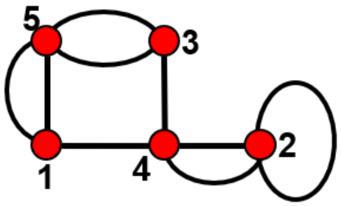	(4x**1**, 1x**2**, 4x**2**^{1}, 2x**2**^{2}) **g** 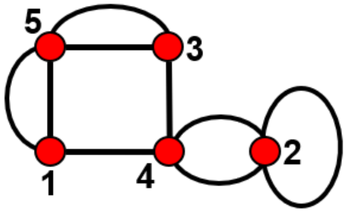
	(4x**1**, 1x**2**, 4x**2**^{1}, 2x**2**^{2}) **h** 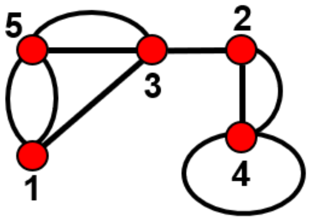	(4x**1**, 1x**2**, 4x**2**^{1}, 2x**2**^{2}) **i** 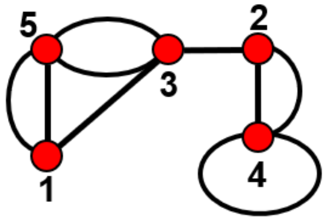	(4x**1**, 1x**2**, 4x**2**^{1}, 2x**2**^{2}) **j** 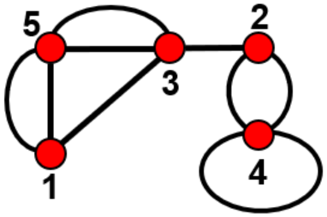	(4x**1**, 1x**2**, 4x**2**^{1}, 2x**2**^{2}) **k** 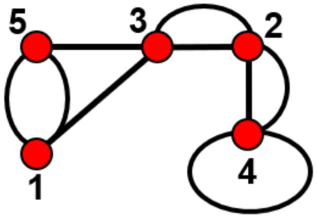
	(4x**1**, 1x**2**, 4x**2**^{1}, 2x**2**^{2}) **l** 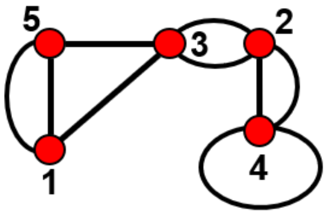	(4x**1**, 1x**2**, 4x**2**^{1}, 2x**2**^{2}) **m** 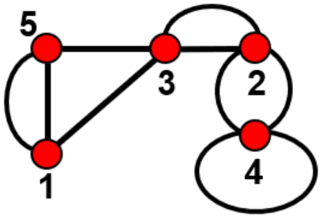	(4x**1**, 1x**2**, 2x**2**^{1}, 4x**2**^{2}) **a** 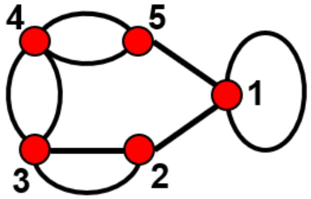	(4x**1**, 1x**2**, 2x**2**^{1}, 4x**2**^{2}) **b** 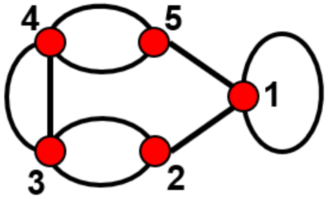
	(4x**1**, 1x**2**, 2x**2**^{1}, 4x**2**^{2}) **c** 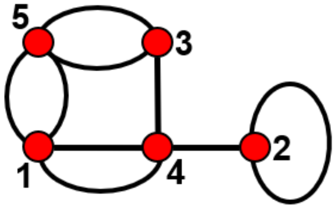	(4x**1**, 1x**2**, 2x**2**^{1}, 4x**2**^{2}) **d** 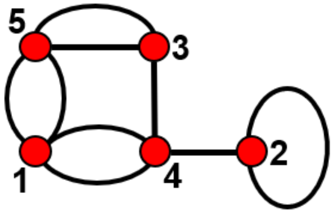	(4x**1**, 1x**2**, 2x**2**^{1}, 4x**2**^{2}) **e** 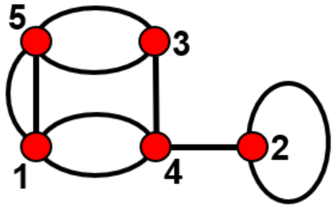	(4x**1**, 1x**2**, 2x**2**^{1}, 4x**2**^{2}) **f** 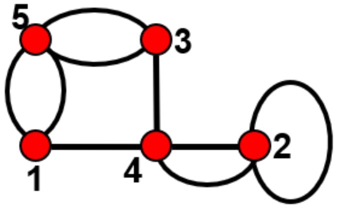

	(4x**1**, 1x**2**, 2x**2**^{1}, 4x**2**^{2}) **g** 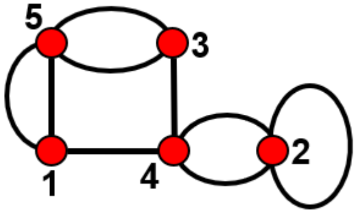	(4x**1**, 1x**2**, 2x**2**^{1}, 4x**2**^{2}) **h** 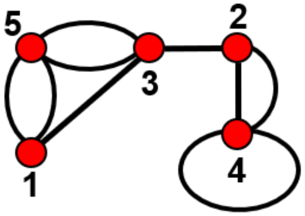	(4x**1**, 1x**2**, 2x**2**^{1}, 4x**2**^{2}) **i** 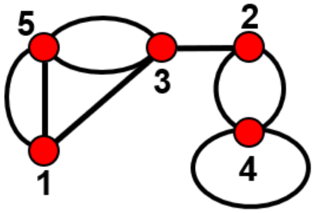	(4x**1**, 1x**2**, 2x**2**^{1}, 4x**2**^{2}) **j** 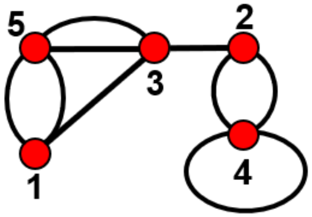
	(4x**1**, 1x**2**, 2x**2**^{1}, 4x**2**^{2}) **k** 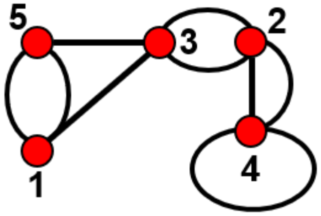	(4x**1**, 1x**2**, 2x**2**^{1}, 4x**2**^{2}) **l** 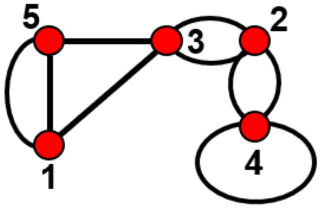	(4x**1**, 1x**2**, 2x**2**^{1}, 4x**2**^{2}) **m** 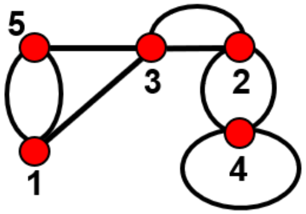	(4x**1**, 1x**2**, 6x**2**^{2}) **a** 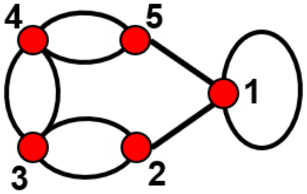
	(4x**1**, 1x**2**, 6x**2**^{2}) **b** 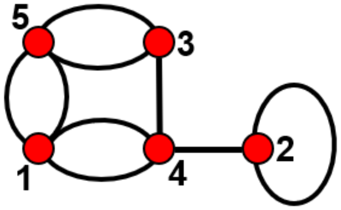	(4x**1**, 1x**2**, 6x**2**^{2}) **c** 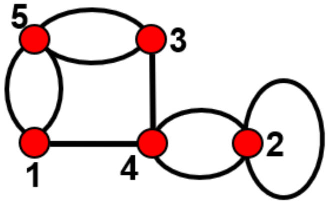	(4x**1**, 1x**2**, 6x**2**^{2}) **d** 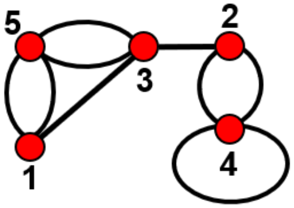	(4x**1**, 1x**2**, 6x**2**^{2}) **e** 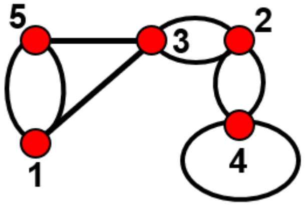
	(2x**1**, 4x**2**, 4x**2**^{1}) **a** 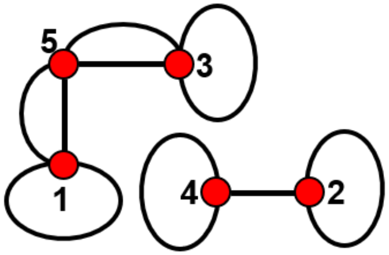	(2x**1**, 4x**2**, 4x**2**^{1}) **b** 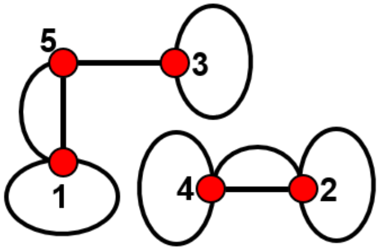	(2x**1**, 4x**2**, 2x**2**^{1}, 2x**2**^{2}) **a** 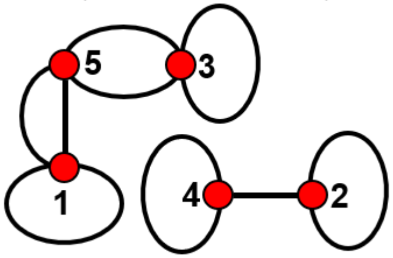	(2x**1**, 4x**2**, 2x**2**^{1}, 2x**2**^{2}) **b** 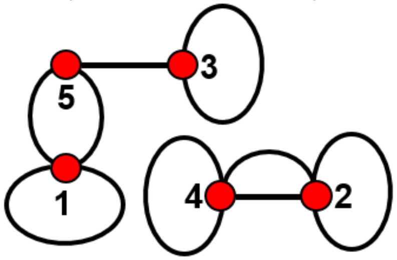

	(2x**1**, 4x**2**, 2x$\mathbf{2}^1$, 2x$\mathbf{2}^2$) **c** 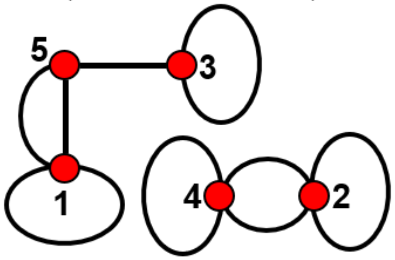	(2x**1**, 4x**2**, 4x$\mathbf{2}^2$) **a** 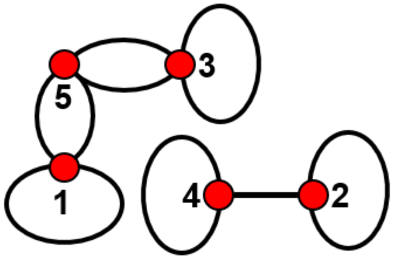	(2x**1**, 4x**2**, 4x$\mathbf{2}^2$) **b** 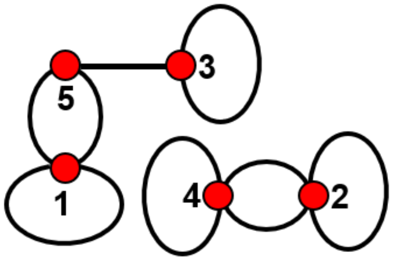	(2x**1**, 2x**2**, 6x$\mathbf{2}^1$) **a** 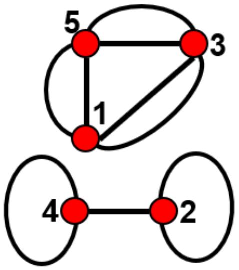
	(2x**1**, 2x**2**, 6x$\mathbf{2}^1$) **b** 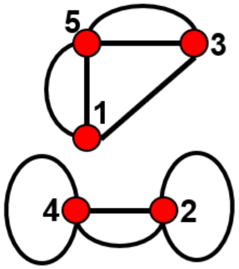	(2x**1**, 2x**2**, 6x$\mathbf{2}^1$) **c** 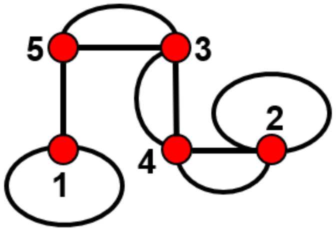	(2x**1**, 2x**2**, 6x$\mathbf{2}^1$) **d** 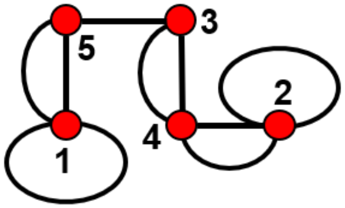	(2x**1**, 2x**2**, 4x$\mathbf{2}^1$, 2x$\mathbf{2}^2$) **a** 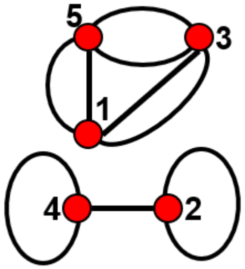
	(2x**1**, 2x**2**, 4x$\mathbf{2}^1$, 2x$\mathbf{2}^2$) **b** 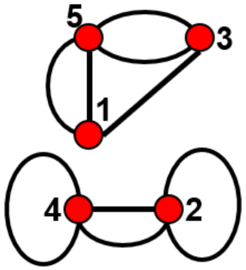	(2x**1**, 2x**2**, 4x$\mathbf{2}^1$, 2x$\mathbf{2}^2$) **c** 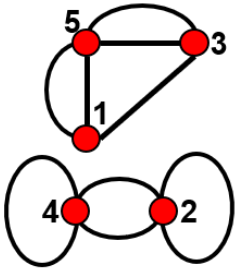	(2x**1**, 2x**2**, 4x$\mathbf{2}^1$, 2x$\mathbf{2}^2$) **d** 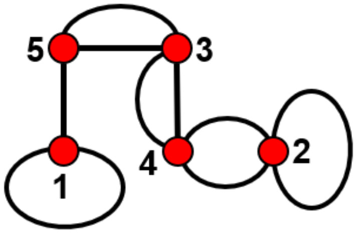	(2x**1**, 2x**2**, 4x$\mathbf{2}^1$, 2x$\mathbf{2}^2$) **e** 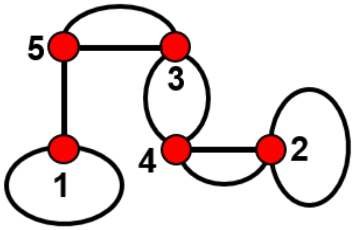

	(2x**1**, 2x**2**, 4x$\mathbf{2}^1$, 2x$\mathbf{2}^2$) **f** 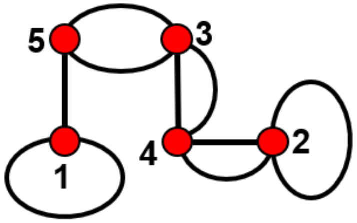	(2x**1**, 2x**2**, 4x$\mathbf{2}^1$, 2x$\mathbf{2}^2$) **g** 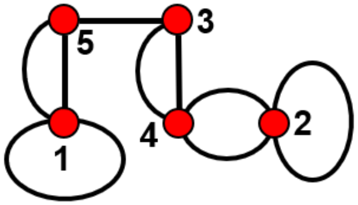	(2x**1**, 2x**2**, 4x$\mathbf{2}^1$, 2x$\mathbf{2}^2$) **h** 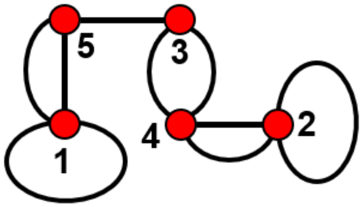	(2x**1**, 2x**2**, 4x$\mathbf{2}^1$, 2x$\mathbf{2}^2$) **i** 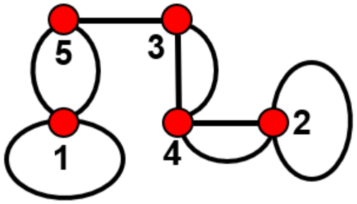
	(2x**1**, 2x**2**, 2x$\mathbf{2}^1$, 4x$\mathbf{2}^2$) **a** 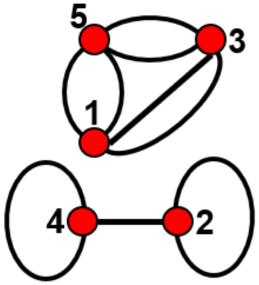	(2x**1**, 2x**2**, 2x$\mathbf{2}^1$, 4x$\mathbf{2}^2$) **b** 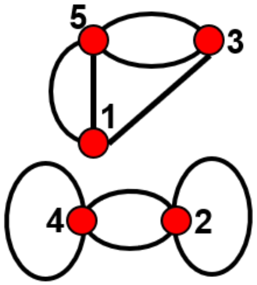	(2x**1**, 2x**2**, 2x$\mathbf{2}^1$, 4x$\mathbf{2}^2$) **c** 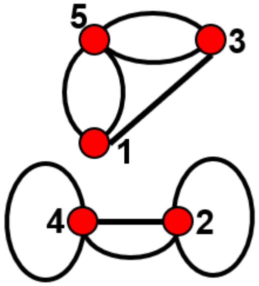	(2x**1**, 2x**2**, 2x$\mathbf{2}^1$, 4x$\mathbf{2}^2$) **d** 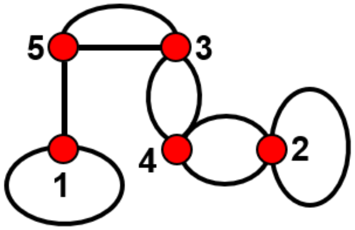
	(2x**1**, 2x**2**, 2x$\mathbf{2}^1$, 4x$\mathbf{2}^2$) **e** 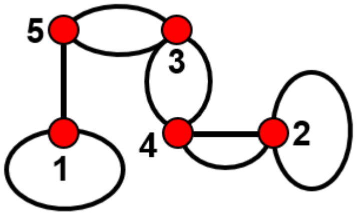	(2x**1**, 2x**2**, 2x$\mathbf{2}^1$, 4x$\mathbf{2}^2$) **f** 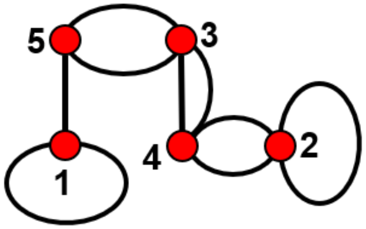	(2x**1**, 2x**2**, 2x$\mathbf{2}^1$, 4x$\mathbf{2}^2$) **g** 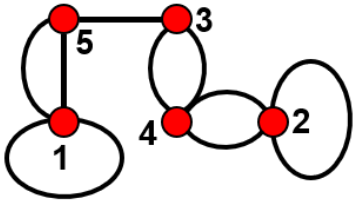	(2x**1**, 2x**2**, 2x$\mathbf{2}^1$, 4x$\mathbf{2}^2$) **h** 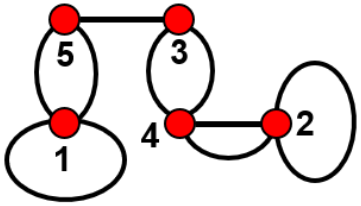

	(2x**1**, 2x**2**, 2x$\mathbf{2}^1$, 4x$\mathbf{2}^2$) **i** 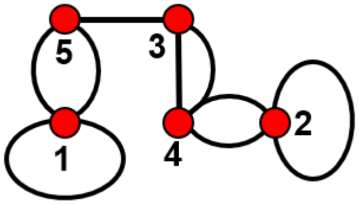	(2x**1**, 2x**2**, 6x$\mathbf{2}^2$) **a** 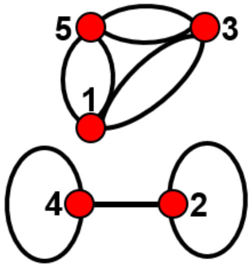	(2x**1**, 2x**2**, 6x$\mathbf{2}^2$) **b** 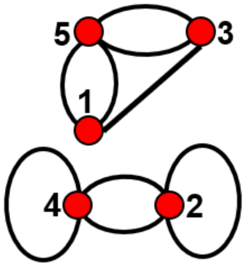	(2x**1**, 2x**2**, 6x$\mathbf{2}^2$) **c** 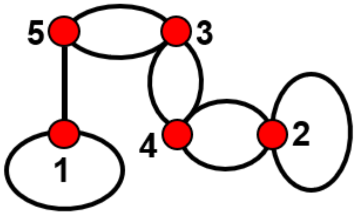
	(2x**1**, 2x**2**, 6x$\mathbf{2}^2$) **d** 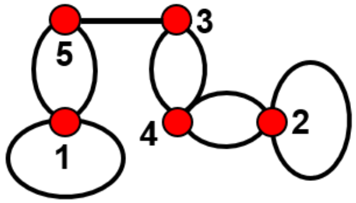	(2x**1**, 8x$\mathbf{2}^1$) 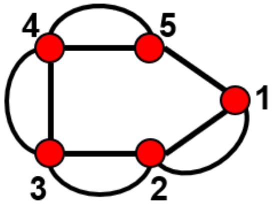	(2x**1**, 6x$\mathbf{2}^1$, 2x$\mathbf{2}^2$) **a** 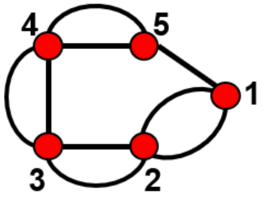	(2x**1**, 6x$\mathbf{2}^1$, 2x$\mathbf{2}^2$) **b** 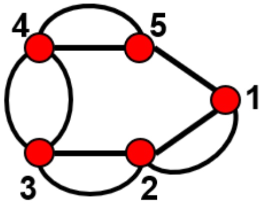
	(2x**1**, 4x$\mathbf{2}^1$, 4x$\mathbf{2}^2$) **a** 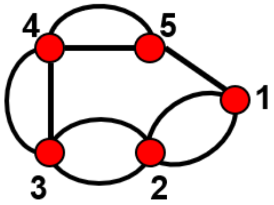	(2x**1**, 4x$\mathbf{2}^1$, 4x$\mathbf{2}^2$) **b** 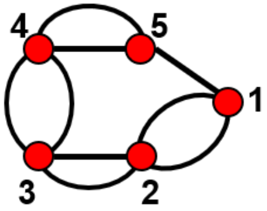	(2x**1**, 4x$\mathbf{2}^1$, 4x$\mathbf{2}^2$) **c** 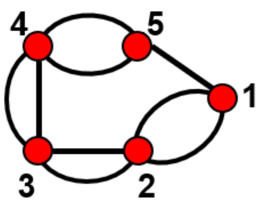	(2x**1**, 2x$\mathbf{2}^1$, 6x$\mathbf{2}^2$) **a** 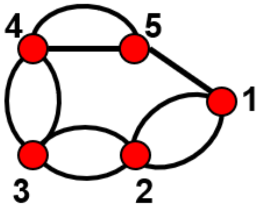
	(2x**1**, 2x$\mathbf{2}^1$, 6x$\mathbf{2}^2$) **b** 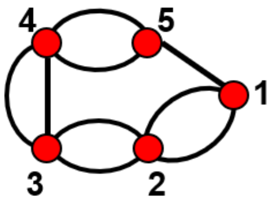	(2x**1**, 8x$\mathbf{2}^2$) 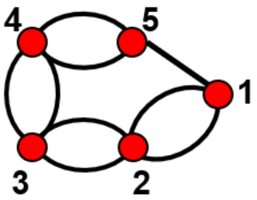		

$^3V_2{}^4V_4$	NG			
$^3V_2{}^4V_5$	NG			
$^3V_2{}^4V_6$	NG			
$^3V_4{}^4V_1$	(16x**1**)<br>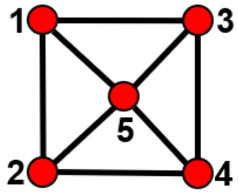	(14x**1**, 1x**2**) **a**<br>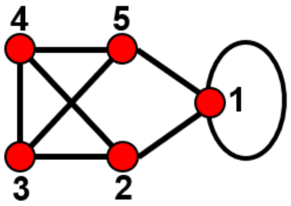	(14x**1**, 1x**2**) **b**<br>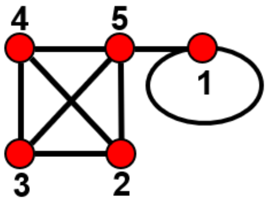	(12x**1**, 2x**2**)<br>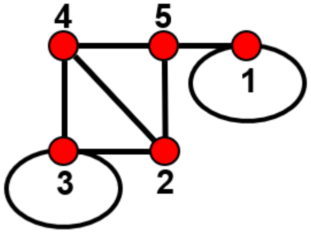
	(12x**1**, 2x**2**^{1})<br>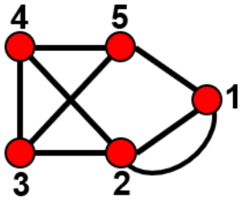	(12x**1**, 2x**2**^{2})<br>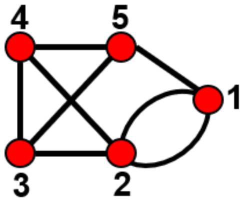	(10x**1**, 3x**2**)<br>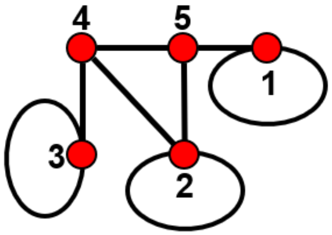	(10x**1**, 1x**2**, 2x**2**^{1}) **a**<br>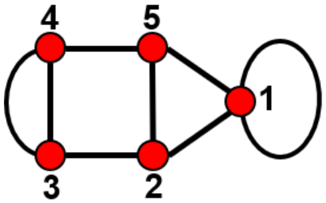
	(10x**1**, 1x**2**, 2x**2**^{1}) **b**<br>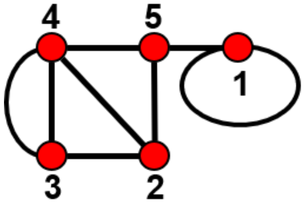	(10x**1**, 1x**2**, 2x**2**^{2}) **a**<br>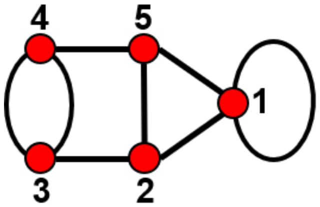	(10x**1**, 1x**2**, 2x**2**^{2}) **b**<br>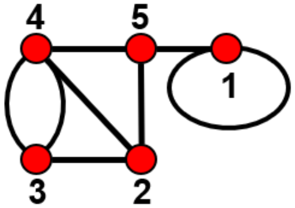	(8x**1**, 4x**2**) **a**<br>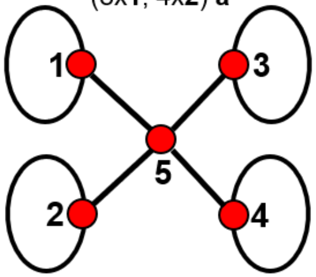

(8x**1**, 4x**2**) **b**

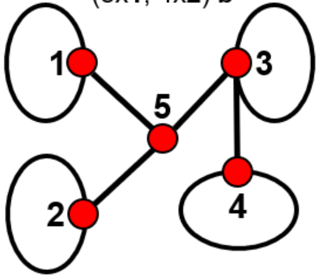

(8x**1**, 2x**2**, 2x**2**^{1}) **a**

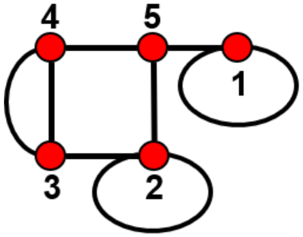

(8x**1**, 2x**2**, 2x**2**^{1}) **b**

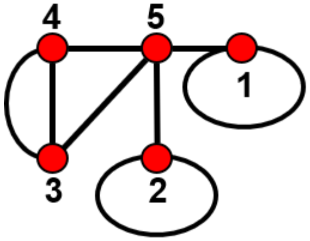

(8x**1**, 2x**2**, 2x**2**^{1}) **c**

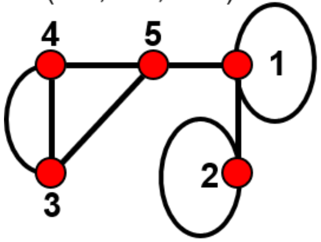

(8x**1**, 2x**2**, 2x**2**^{1}) **d**

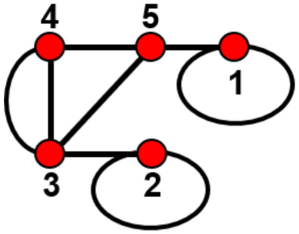

(8x**1**, 2x2, 2x**2**^{2}) **a**

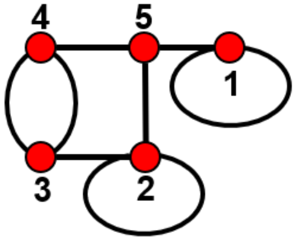

(8x**1**, 2x2, 2x**2**^{2}) **b**

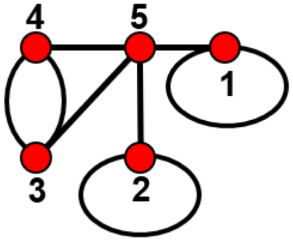

(8x**1**, 2x**2**, 2x**2**^{2}) **c**

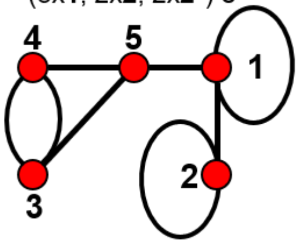

(8x**1**, 2x**2**, 2x**2**^{2}) **d**

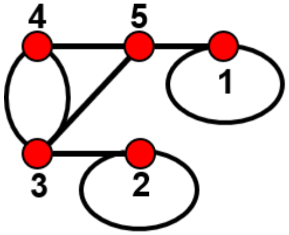

(8x**1**, 4x**2**^{1}) **a**

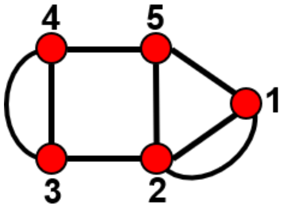

(8x**1**, 4x**2**^{1}) **b**

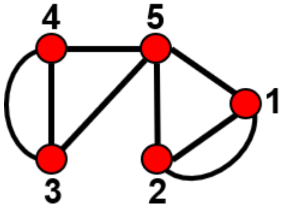

(8x**1**, 2x**2**^{1}, 2x**2**^{2}) **a**

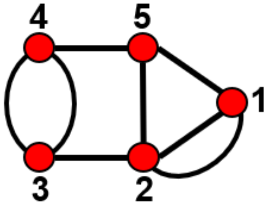

(8x**1**, 2x**2**^{1}, 2x**2**^{2}) **b**

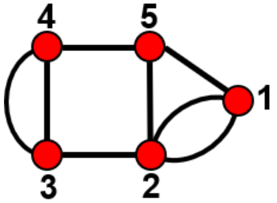

(8x**1**, 2x**2**^{1}, 2x**2**^{2}) **c**

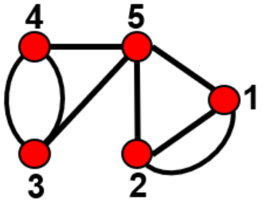

(8x**1**, 4x**2**^{2}) **a**

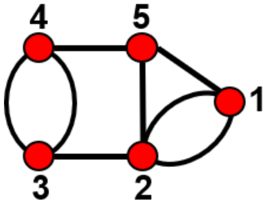

(8x**1**, 4x**2**^{2}) **b**

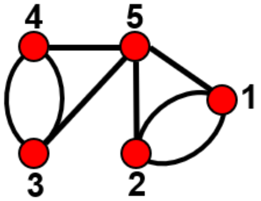

(6x**1**, 5x**2**)

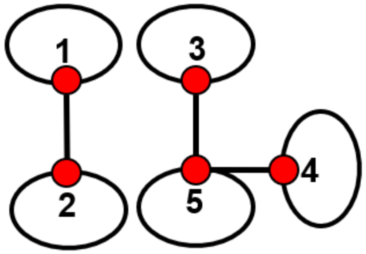

(6x**1**, 3x**2**, 2x$\mathbf{2}^{1}$) **a**

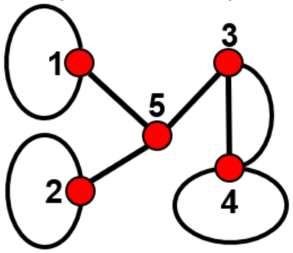

(6x**1**, 3x**2**, 2x$\mathbf{2}^{1}$) **b**

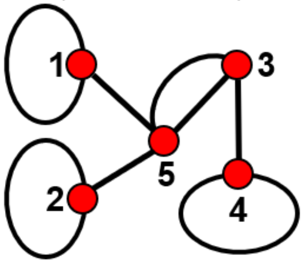

(6x**1**, 3x**2**, 2x$\mathbf{2}^{1}$) **c**

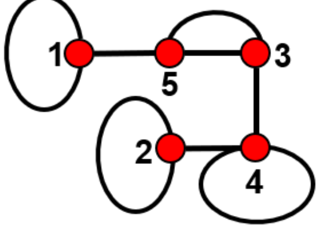

(6x**1**, 3x**2**, 2x$\mathbf{2}^{1}$) **d**

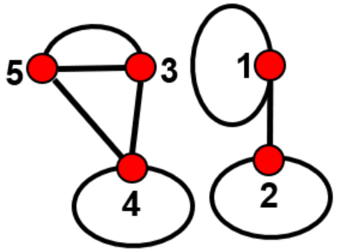

(6x**1**, 3x**2**, 2x$\mathbf{2}^{2}$) **a**

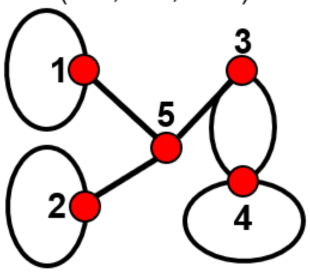

(6x**1**, 3x**2**, 2x$\mathbf{2}^{2}$) **b**

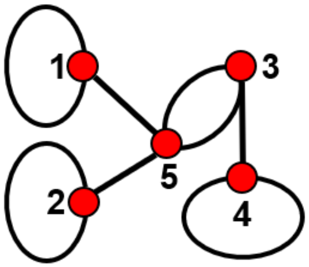

(6x**1**, 3x**2**, 2x$\mathbf{2}^{2}$) **c**

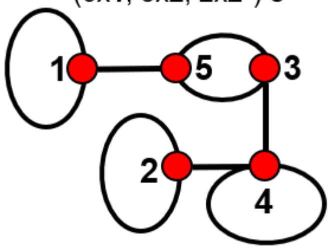

(6x**1**, 3x**2**, 2x$\mathbf{2}^{2}$) **d**

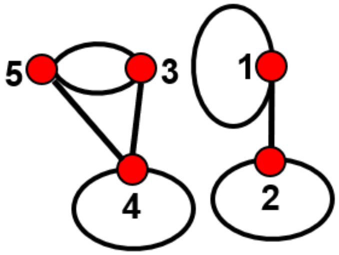

(6x**1**, 1x**2**, 4x$\mathbf{2}^{1}$) **a**

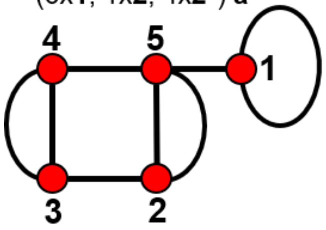

(6x**1**, 1x**2**, 4x$\mathbf{2}^{1}$) **b**

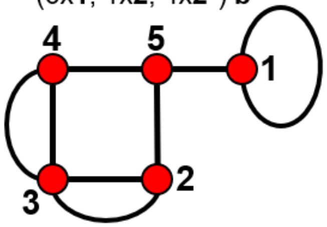

(6x**1**, 1x**2**, 4x$\mathbf{2}^{1}$) **c**

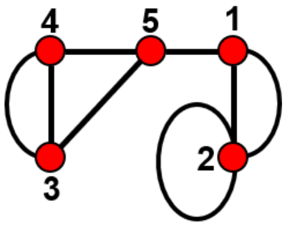

	(6x**1**, 1x**2**, 4x$\mathbf{2}^1$) **d**<br>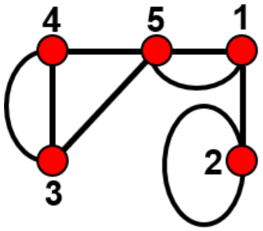	(6x**1**, 1x**2**, 4x$\mathbf{2}^1$) **e**<br>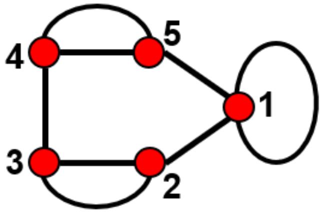	(6x**1**, 1x**2**, 2x$\mathbf{2}^1$, 2x$\mathbf{2}^2$) **a**<br>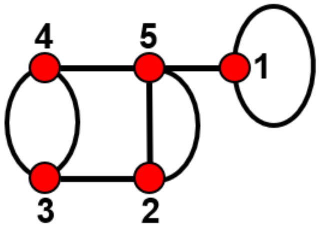	(6x**1**, 1x**2**, 2x$\mathbf{2}^1$, 2x$\mathbf{2}^2$) **b**<br>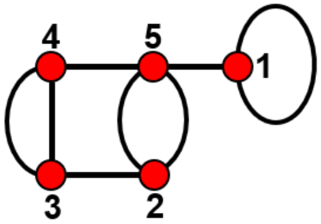
	(6x**1**, 1x**2**, 2x$\mathbf{2}^1$, 2x$\mathbf{2}^2$) **c**<br>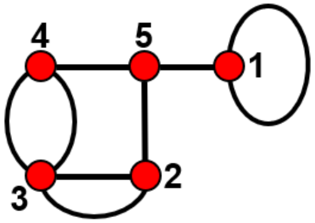	(6x**1**, 1x**2**, 2x$\mathbf{2}^1$, 2x$\mathbf{2}^2$) **d**<br>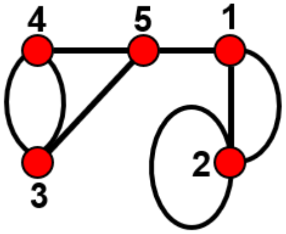	(6x**1**, 1x**2**, 2x$\mathbf{2}^1$, 2x$\mathbf{2}^2$) **e**<br>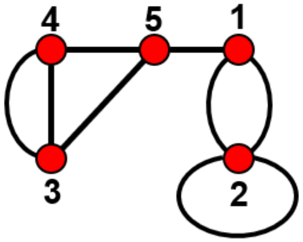	(6x**1**, 1x**2**, 2x$\mathbf{2}^1$, 2x$\mathbf{2}^2$) **f**<br>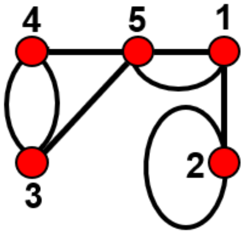
	(6x**1**, 1x**2**, 2x$\mathbf{2}^1$, 2x$\mathbf{2}^2$) **g**<br>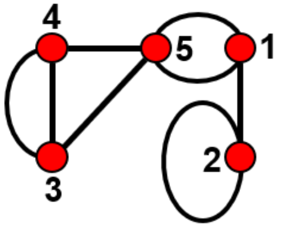	(6x**1**, 1x**2**, 2x$\mathbf{2}^1$, 2x$\mathbf{2}^2$) **h**<br>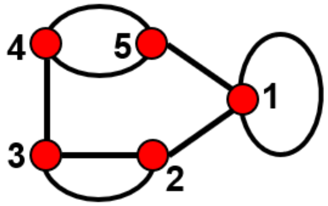	(6x**1**, 1x**2**, 4x$\mathbf{2}^2$) **a**<br>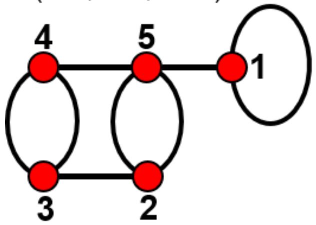	(6x**1**, 1x**2**, 4x$\mathbf{2}^2$) **b**<br>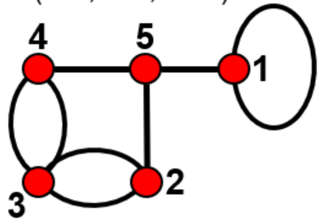
	(6x**1**, 1x**2**, 4x$\mathbf{2}^2$) **c**<br>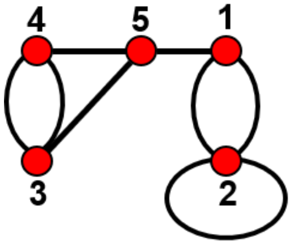	(6x**1**, 1x**2**, 4x$\mathbf{2}^2$) **d**<br>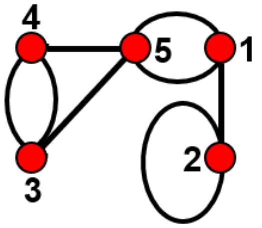	(6x**1**, 1x**2**, 4x$\mathbf{2}^2$) **e**<br>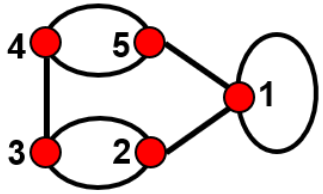	(4x**1**, 4x**2**, 2x$\mathbf{2}^1$)<br>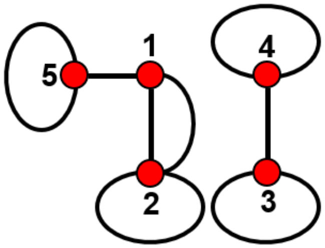

	(4x**1**, 4x**2**, 2x**2**^{2}) 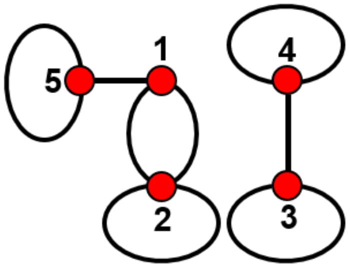	(4x**1**, 2x**2**, 4x**2**^{1}) **a** 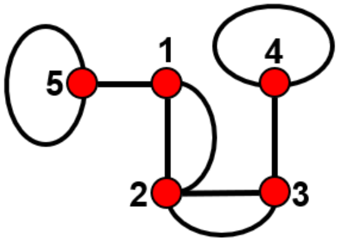	(4x**1**, 2x**2**, 4x**2**^{1}) **b** 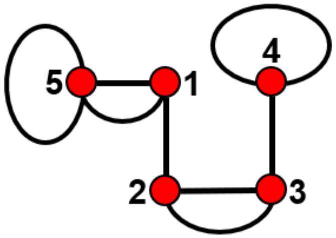	(4x**1**, 2x**2**, 4x**2**^{1}) **c** 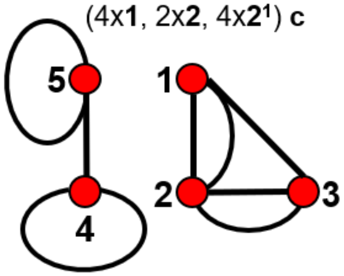
	(4x**1**, 2x**2**, 2x**2**^{1}, 2x**2**^{2}) **a** 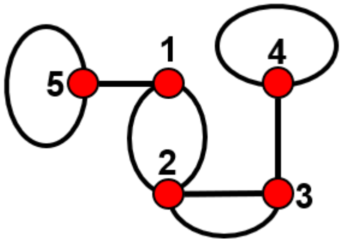	(4x**1**, 2x**2**, 2x**2**^{1}, 2x**2**^{2}) **b** 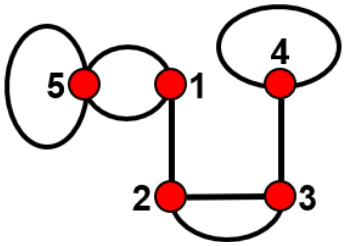	(4x**1**, 2x**2**, 2x**2**^{1}, 2x**2**^{2}) **c** 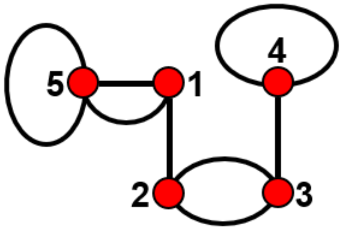	(4x**1**, 2x**2**, 2x**2**^{1}, 2x**2**^{2}) **d** 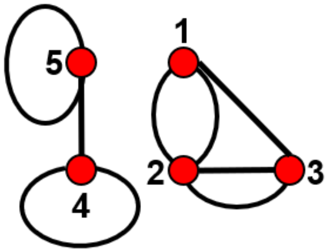
	(4x**1**, 2x**2**, 4x**2**^{2}) **a** 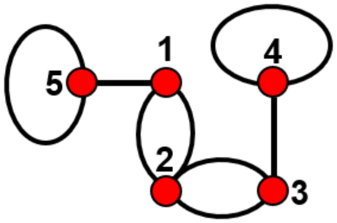	(4x**1**, 2x**2**, 4x**2**^{2}) **b** 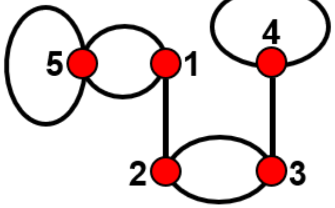	(4x**1**, 2x**2**, 4x**2**^{2}) **c** 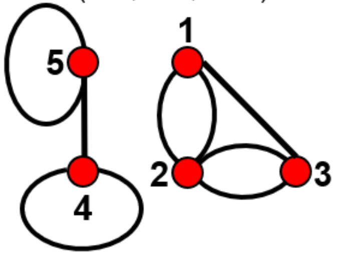	(4x**1**, 6x**2**^{1}) 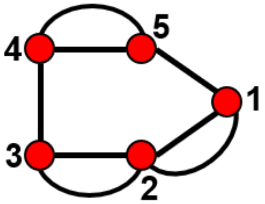

	(4x**1**, 4x**2**^{1}, 2x**2**^{2}) **a** 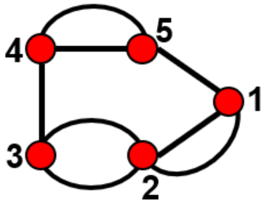	(4x**1**, 4x**2**^{1}, 2x**2**^{2}) **b** 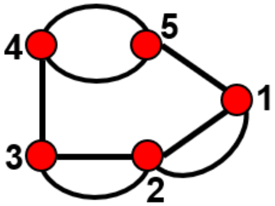	(4x**1**, 2x**2**^{1}, 4x**2**^{2}) **a** 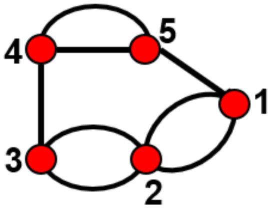	(4x**1**, 2x**2**^{1}, 4x**2**^{2}) **b** 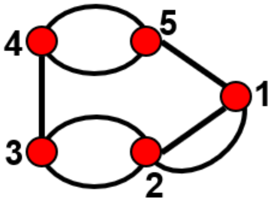
	(4x**1**, 6x**2**^{2}) 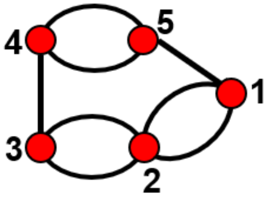			
${}^{3}V_{4}{}^{4}V_{2}$	NG			
${}^{3}V_{4}{}^{4}V_{3}$	NG			
${}^{3}V_{4}{}^{4}V_{4}$	NG			
${}^{3}V_{6}{}^{4}V_{1}$	NG			
${}^{3}V_{6}{}^{4}V_{2}$	NG			
**Rank 3**				
$\boldsymbol{{}^{1}V_{r}{}^{2}V_{r}{}^{3}V_{r}}$				
${}^{1}V_{1}{}^{2}V_{1}{}^{3}V_{1}$	(4x**1**, 1x**2**) 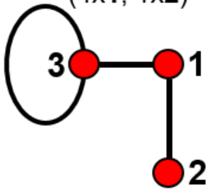	(2x**1**, 2x**2**) 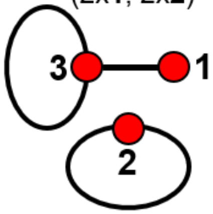	(2x**1**, 2x**2**^{1}) 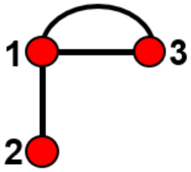	(2x**1**, 2x**2**^{2}) 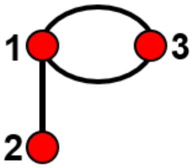

$^{1}V_{1}{}^{2}V_{1}{}^{3}V_{3}$

(12x**1**)

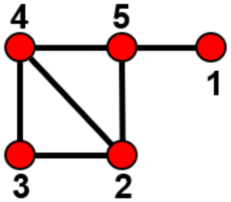

(10x**1**, 1x**2**)

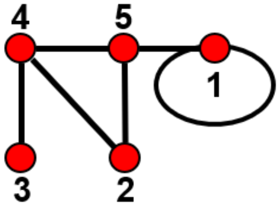

(8x**1**, 2x**2**) **a**

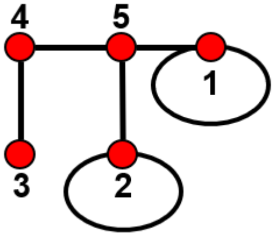

(8x**1**, 2x**2**) **b**

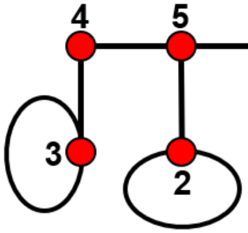

(8x**1**, 2x$\mathbf{2}^{1}$) **a**

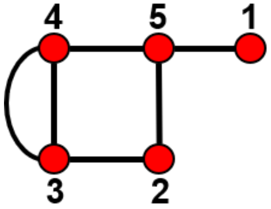

(8x**1**, 2x$\mathbf{2}^{1}$) **b**

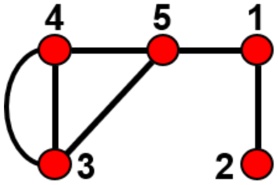

(8x**1**, 2x$\mathbf{2}^{2}$) **a**

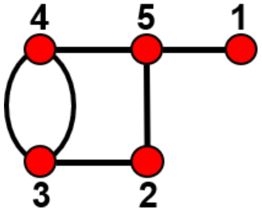

(8x**1**, 2x$\mathbf{2}^{2}$) **b**

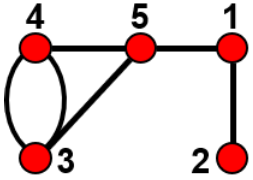

(6x**1**, 3x**2**) **a**

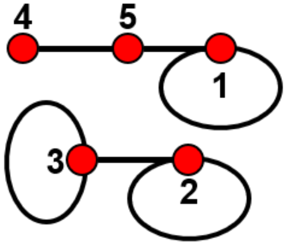

(6x**1**, 3x**2**) **b**

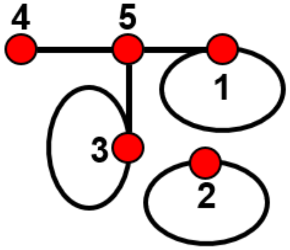

(6x**1**, 1x**2**, 2x$\mathbf{2}^{1}$) **a**

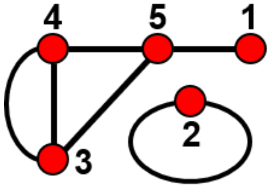

(6x**1**, 1x**2**, 2x$\mathbf{2}^{1}$) **b**

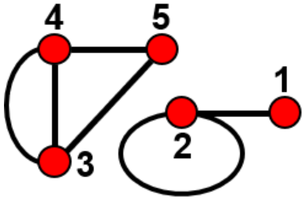

(6x**1**, 1x**2**, 2x$\mathbf{2}^{1}$) **c**

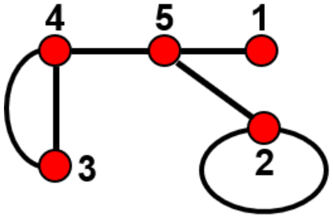

(6x**1**, 1x**2**, 2x$\mathbf{2}^{1}$) **d**

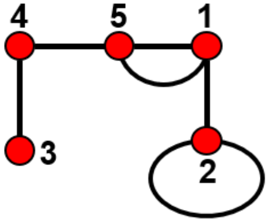

(6x**1**, 1x**2**, 2x$\mathbf{2}^{1}$) **e**

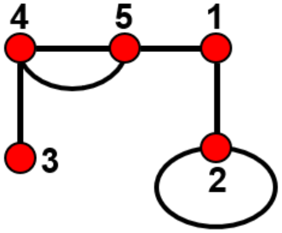

(6x**1**, 1x**2**, 2x$\mathbf{2}^{2}$) **a**

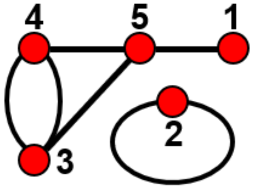

<table>
<tr><td></td><td>(6x1, 1x2, 2x2²) b
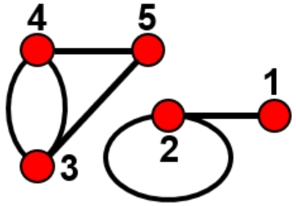</td><td>(6x1, 1x2, 2x2²) c
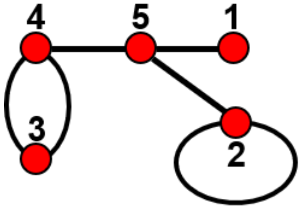</td><td>(6x1, 1x2, 2x2²) d
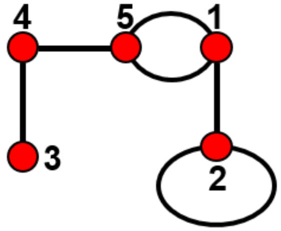</td><td>(6x1, 1x2, 2x2²) e
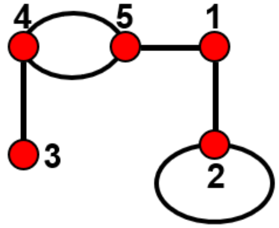</td></tr>
<tr><td></td><td>(4x1, 4x2)
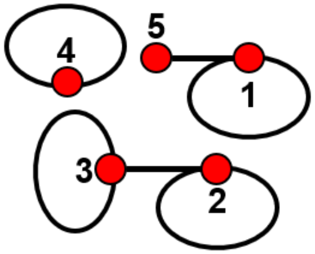</td><td>(4x1, 2x2, 2x2¹) a
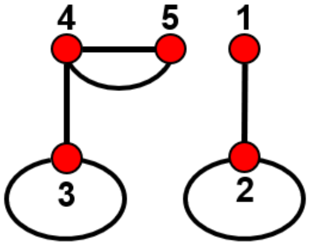</td><td>(4x1, 2x2, 2x2¹) b
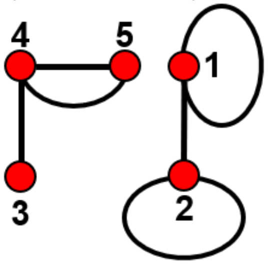</td><td>(4x1, 2x2, 2x2¹) c
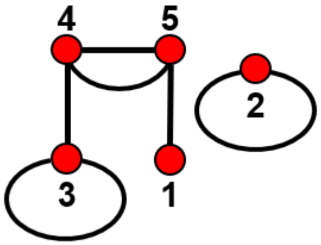</td></tr>
<tr><td></td><td>(4x1, 2x2, 2x2²) a
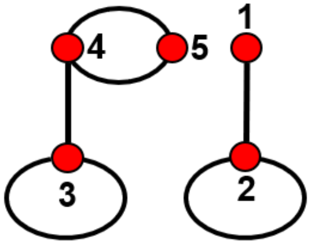</td><td>(4x1, 2x2, 2x2²) b
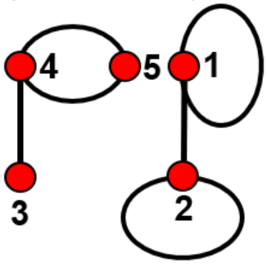</td><td>(4x1, 2x2, 2x2²) c
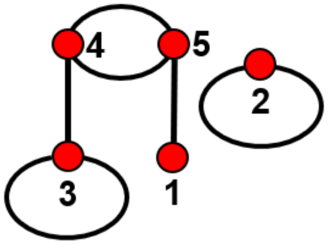</td><td>(4x1, 4x2¹)
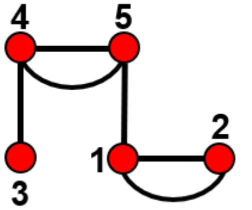</td></tr>
<tr><td></td><td>(4x1, 2x2¹, 2x2²) a
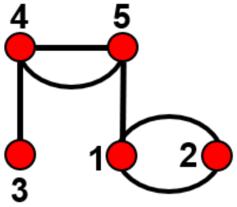</td><td>(4x1, 2x2¹, 2x2²) b
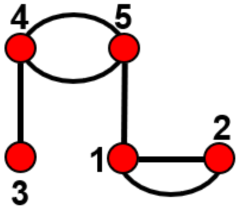</td><td>(4x1, 4x2²)
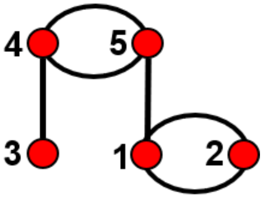</td><td></td></tr>
</table>

$^{1}V_{1}{}^{2}V_{1}{}^{3}V_{5}$	NG			
$^{1}V_{1}{}^{2}V_{2}{}^{3}V_{1}$	(8x**1**)<br>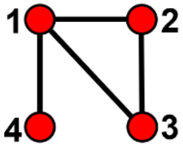	(6x**1**, 1x**2**)<br>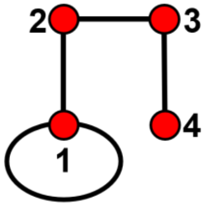	(4x**1**, 2x**2**)<br>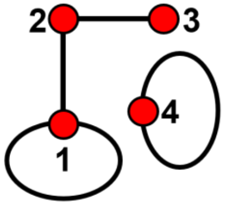	(4x**1**, 2x**2**^{1})<br>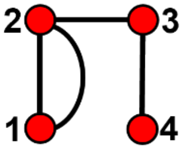
	(4x**1**, 2x**2**^{2})<br>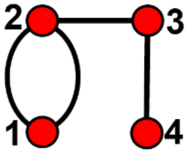	(2x**1**, 3x**2**)<br>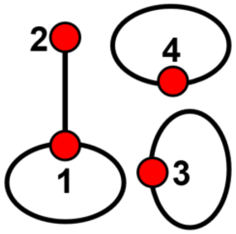	(2x**1**, 1x**2**, 2x**2**^{1}) **a**<br>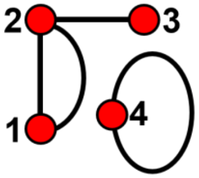	(2x**1**, 1x**2**, 2x**2**^{1}) **b**<br>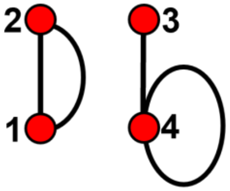
	(2x**1**, 1x**2**, 2x**2**^{2}) **a**<br>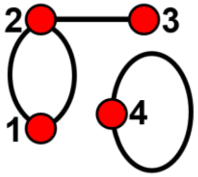	(2x**1**, 1x**2**, 2x**2**^{2}) **b**<br>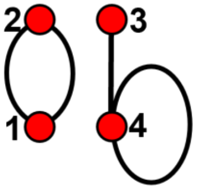		
$^{1}V_{1}{}^{2}V_{2}{}^{3}V_{3}$	(14x**1**) **a**<br>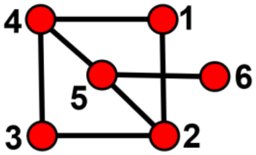	(14x**1**) **b**<br>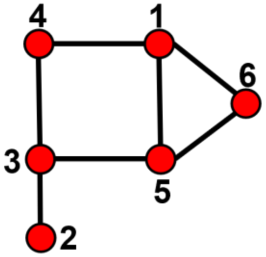	(14x**1**) **c**<br>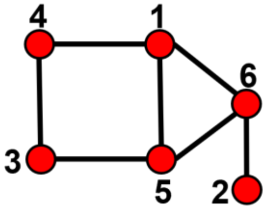	(14x**1**) **d**<br>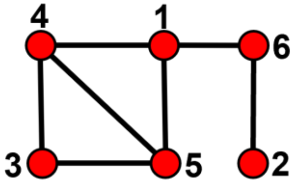

	(12x**1**, 1x**2) a**<br>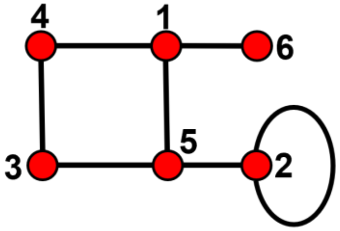	(12x**1**, 1x**2) b**<br>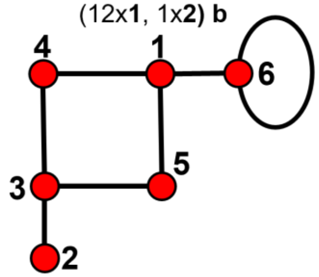	(12x**1**, 1x**2) c**<br>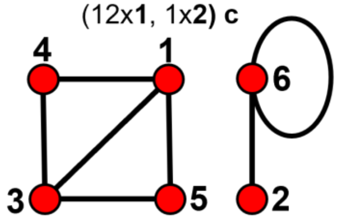	(12x**1**, 1x**2) d**<br>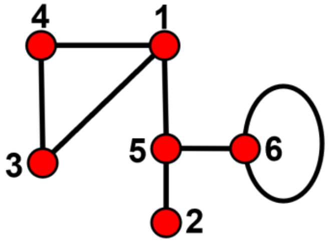
	(12x**1**, 1x**2) e**<br>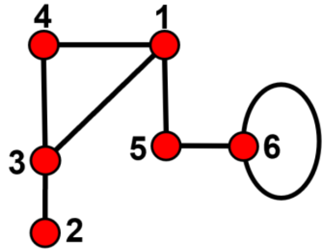	(12x**1**, 1x**2) f**<br>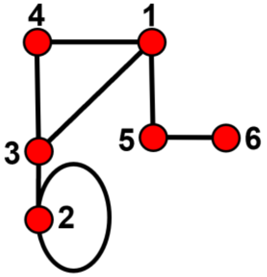	(12x**1**, 1x**2) g**<br>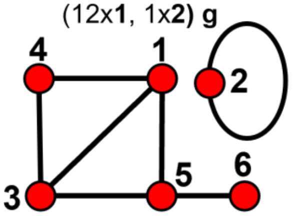	(10x**1**, 2x**2) a**<br>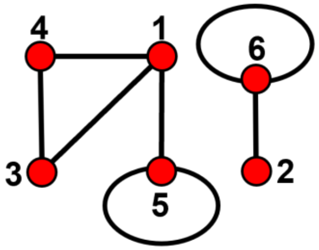
	(10x**1**, 2x**2) b**<br>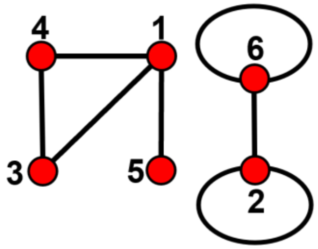	(10x**1**, 2x**2) c**<br>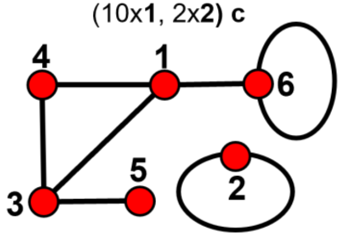	(10x**1**, 2x**2) d**<br>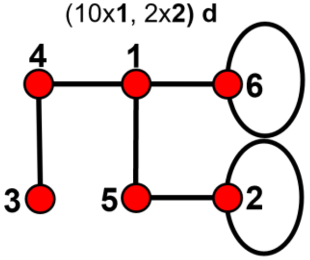	(10x**1**, 2x**2) e**<br>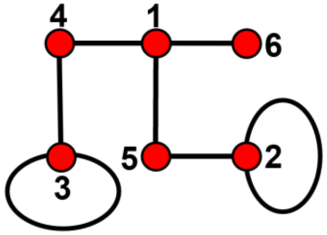

	(10x**1**, 2x**2**) f<br>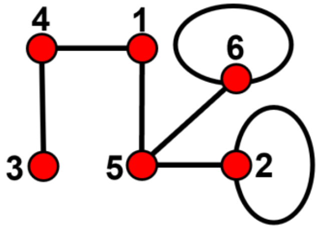	(10x**1**, 2x**2**) g<br>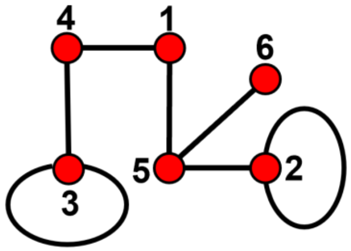	(10x**1**, 2x$\mathbf{2}^1$) **a**<br>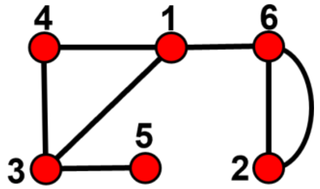	(10x**1**, 2x$\mathbf{2}^1$) **b**<br>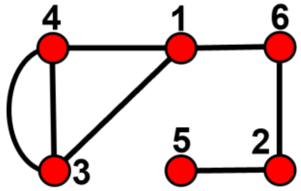
	(10x**1**, 2x$\mathbf{2}^1$) **c**<br>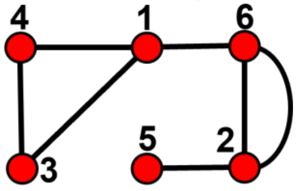	(10x**1**, 2x$\mathbf{2}^1$) **d**<br>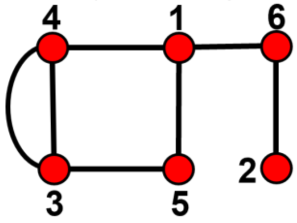	(10x**1**, 2x$\mathbf{2}^1$) **e**<br>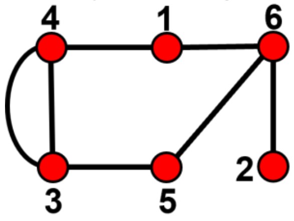	(10x**1**, 2x$\mathbf{2}^1$) **f**<br>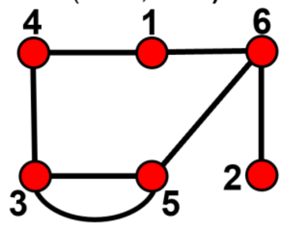
	(10x**1**, 2x$\mathbf{2}^2$) **a**<br>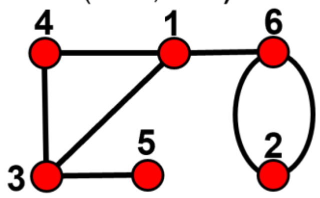	(10x**1**, 2x$\mathbf{2}^2$) **b**<br>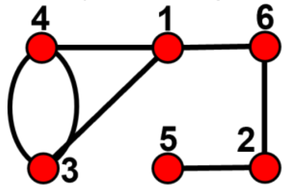	(10x**1**, 2x$\mathbf{2}^2$) **c**<br>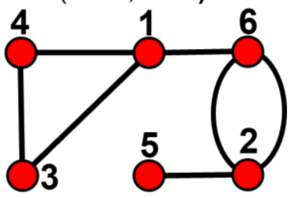	(10x**1**, 2x$\mathbf{2}^2$) **d**<br>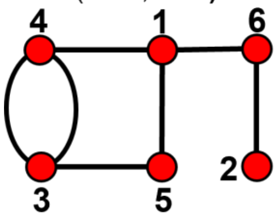
	(10x**1**, 2x$\mathbf{2}^2$) **e**<br>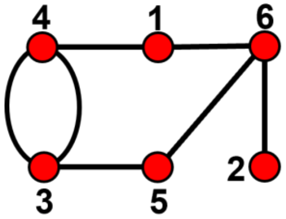	(10x**1**, 2x$\mathbf{2}^2$) **f**<br>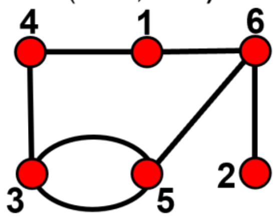	(8x**1**, 3x**2**) a<br>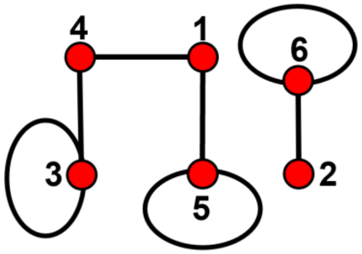	(8x**1**, 3x**2**) b<br>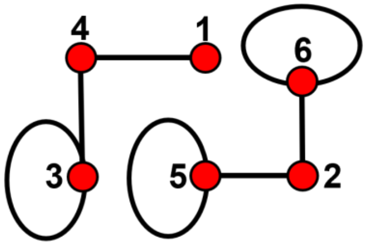

	(8x**1**, 3x**2**) **c** 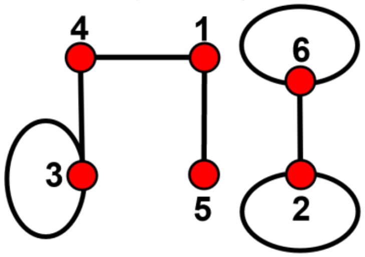	(8x**1**, 3x**2**) **d** 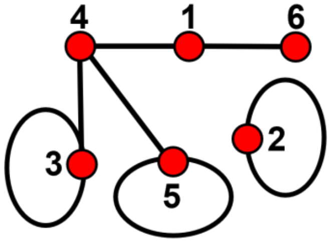	(8x**1**, 3x**2**) **e** 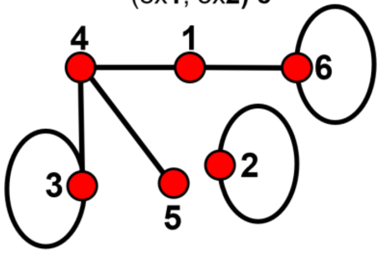	(8x**1**, 1x**2**, 2x$\mathbf{2}^1$) **a** 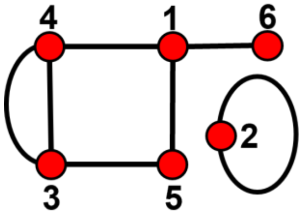
	(8x**1**, 1x**2**, 2x$\mathbf{2}^1$) **b** 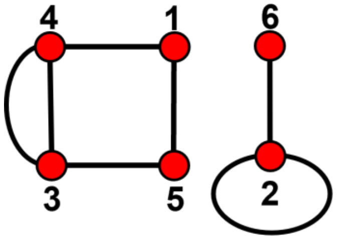	(8x**1**, 1x**2**, 2x$\mathbf{2}^1$) **c** 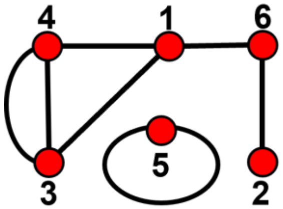	(8x**1**, 1x**2**, 2x$\mathbf{2}^1$) **d** 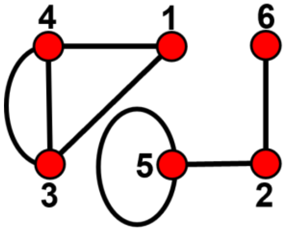	(8x**1**, 1x**2**, 2x$\mathbf{2}^1$) **e** 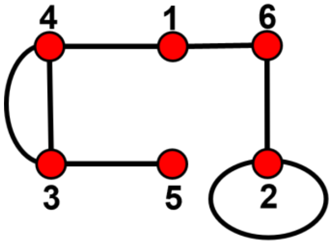
	(8x**1**, 1x**2**, 2x$\mathbf{2}^1$) **f** 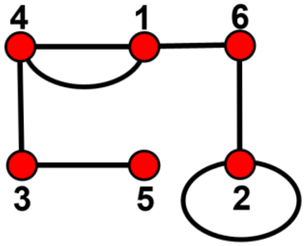	(8x**1**, 1x**2**, 2x$\mathbf{2}^1$) **g** 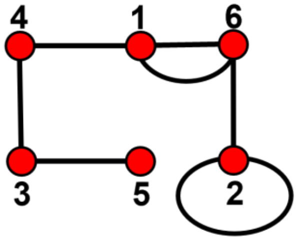	(8x**1**, 1x**2**, 2x$\mathbf{2}^1$) **h** 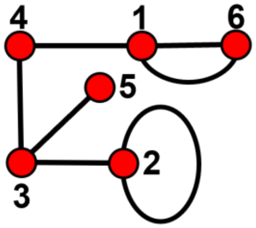	(8x**1**, 1x**2**, 2x$\mathbf{2}^1$) **i** 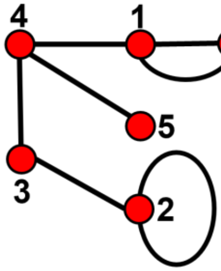

(8x**1**, 1x**2**, 2x$\mathbf{2}^{1}$) **j**

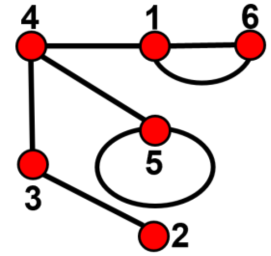

(8x**1**, 1x**2**, 2x$\mathbf{2}^{2}$) **a**

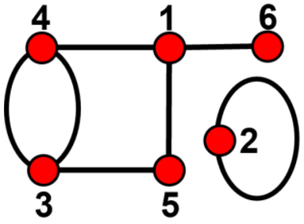

(8x**1**, 1x**2**, 2x$\mathbf{2}^{2}$) **b**

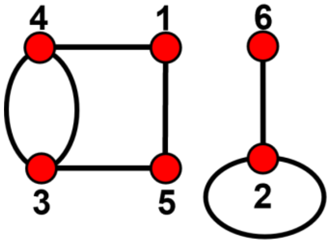

(8x**1**, 1x**2**, 2x$\mathbf{2}^{2}$) **c**

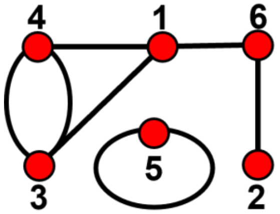

(8x**1**, 1x**2**, 2x$\mathbf{2}^{2}$) **d**

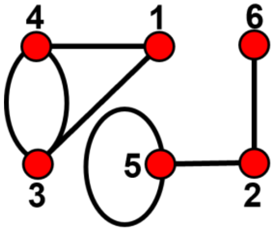

(8x**1**, 1x**2**, 2x$\mathbf{2}^{2}$) **e**

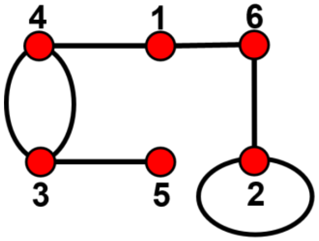

(8x**1**, 1x**2**, 2x$\mathbf{2}^{2}$) **f**

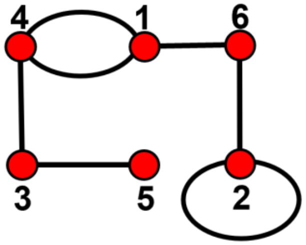

(8x**1**, 1x**2**, 2x$\mathbf{2}^{2}$) **g**

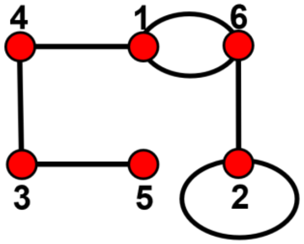

(8x**1**, 1x**2**, 2x$\mathbf{2}^{2}$) **h**

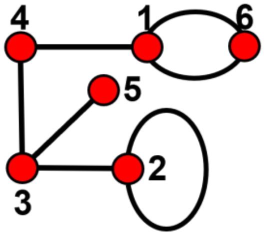

(8x**1**, 1x**2**, 2x$\mathbf{2}^{2}$) **i**

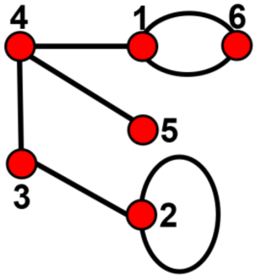

(8x**1**, 1x**2**, 2x$\mathbf{2}^{2}$) **j**

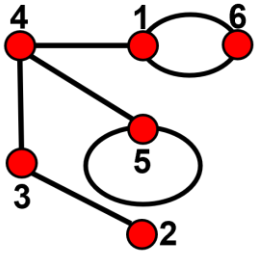

(6x**1**, 4x**2**) **a**

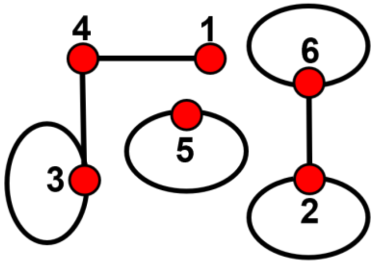

	(6x**1**, 4x**2**) b	(6x**1**, 4x**2**) c	(6x**1**, 2x**2**, 2x$\mathbf{2^1}$) a	(6x**1**, 2x**2**, 2x$\mathbf{2^1}$) b
	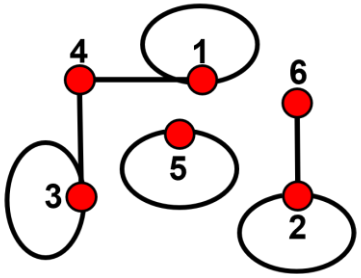	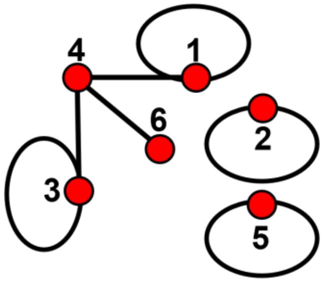	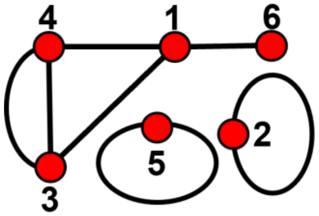	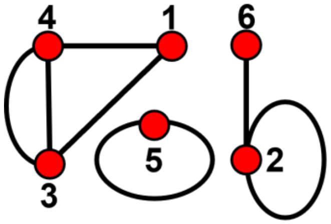
	(8x**1**, 1x**2**, 2x$\mathbf{2^1}$) c	(8x**1**, 1x**2**, 2x$\mathbf{2^1}$) d	(8x**1**, 1x**2**, 2x$\mathbf{2^1}$) e	(8x**1**, 1x**2**, 2x$\mathbf{2^1}$) f
	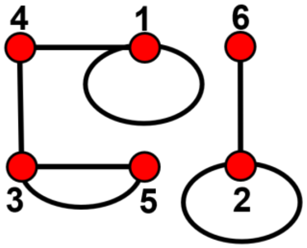	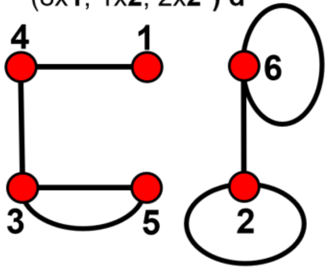	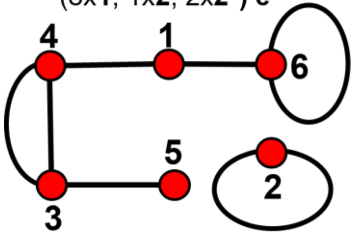	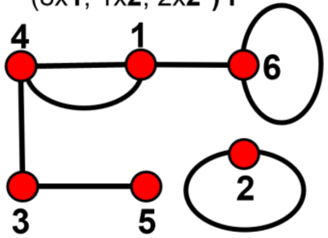
	(8x**1**, 1x**2**, 2x$\mathbf{2^1}$) g	(8x**1**, 1x**2**, 2x$\mathbf{2^1}$) h	(8x**1**, 1x**2**, 2x$\mathbf{2^1}$) i	(8x**1**, 1x**2**, 2x$\mathbf{2^1}$) j
	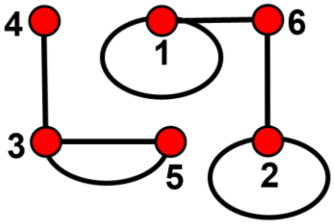	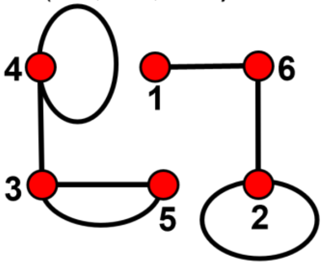	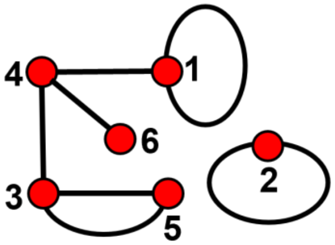	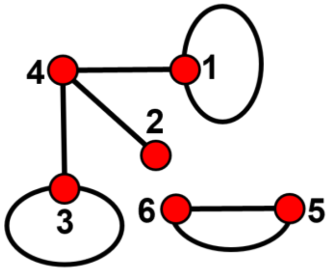

	(6x**1**, 2x**2**, 2x**2**^{2}) a 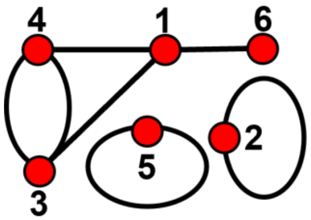	(6x**1**, 2x**2**, 2x**2**^{2}) b 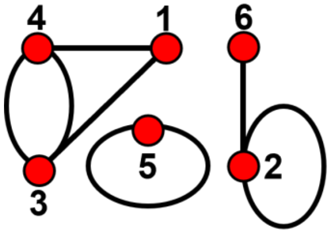	(8x**1**, 1x**2**, 2x**2**^{2}) c 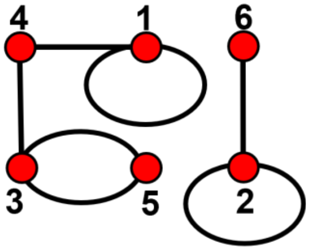	(8x**1**, 1x**2**, 2x**2**^{2}) d 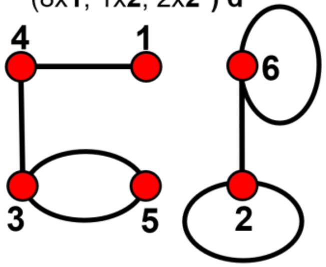
	(8x**1**, 1x**2**, 2x**2**^{2}) e 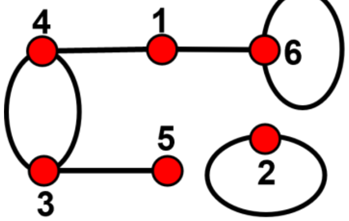	(8x**1**, 1x**2**, 2x**2**^{2}) f 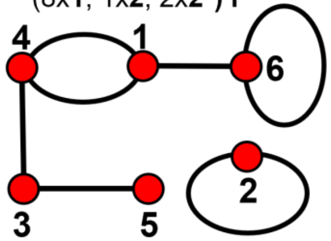	(8x**1**, 1x**2**, 2x**2**^{2}) g 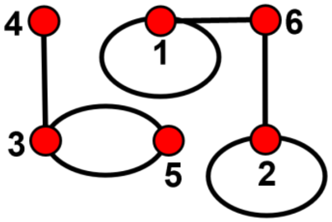	(8x**1**, 1x**2**, 2x**2**^{2}) h 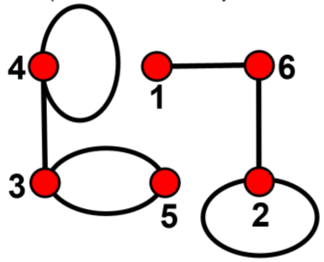
	(8x**1**, 1x**2**, 2x**2**^{2}) i 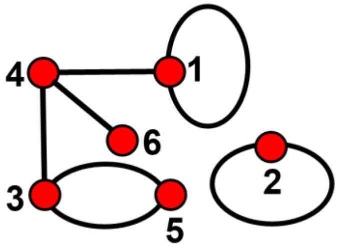	(8x**1**, 1x**2**, 2x**2**^{2}) j 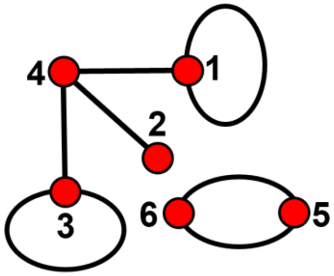	(6x**1**, 4x**2**^{1}) a 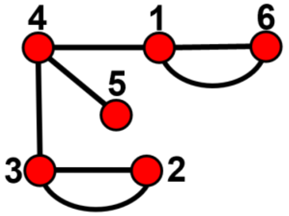	(6x**1**, 4x**2**^{1}) b 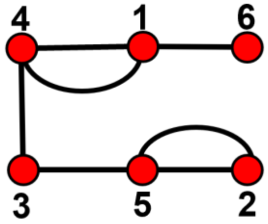

	(6x**1**, 4x$\mathbf{2}^{1}$) **c**	(6x**1**, 4x$\mathbf{2}^{1}$) **d**	(6x**1**, 4x$\mathbf{2}^{1}$) **e**	(6x**1**, 2x$\mathbf{2}^{1}$, 2x$\mathbf{2}^{2}$) **a**
	(6x**1**, 2x$\mathbf{2}^{1}$, 2x$\mathbf{2}^{2}$) **b**	(6x**1**, 2x$\mathbf{2}^{1}$, 2x$\mathbf{2}^{2}$) **c**	(6x**1**, 2x$\mathbf{2}^{1}$, 2x$\mathbf{2}^{2}$) **d**	(6x**1**, 2x$\mathbf{2}^{1}$, 2x$\mathbf{2}^{2}$) **e**
	(6x**1**, 2x$\mathbf{2}^{1}$, 2x$\mathbf{2}^{2}$) **f**	(6x**1**, 2x$\mathbf{2}^{1}$, 2x$\mathbf{2}^{2}$) **g**	(6x**1**, 2x$\mathbf{2}^{1}$, 2x$\mathbf{2}^{2}$) **h**	(6x**1**, 2x$\mathbf{2}^{1}$, 2x$\mathbf{2}^{2}$) **i**

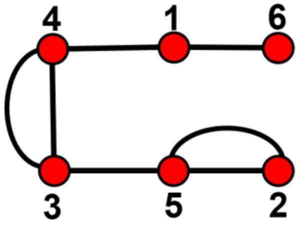
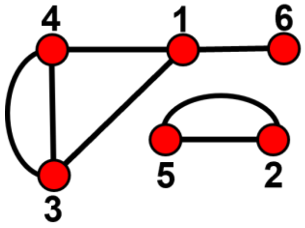
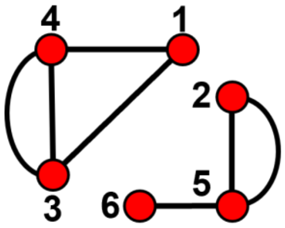
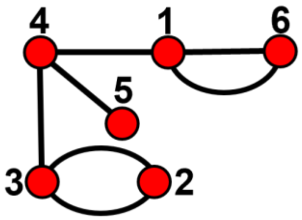
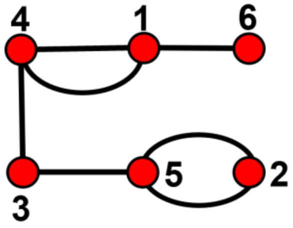
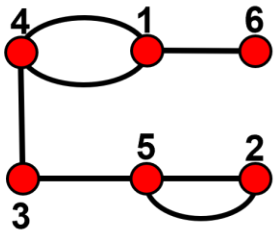
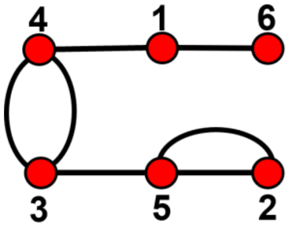
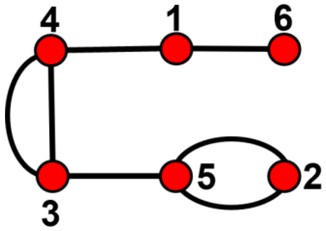
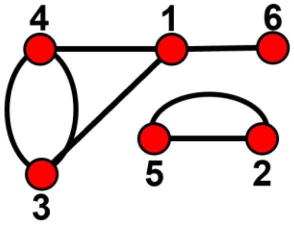
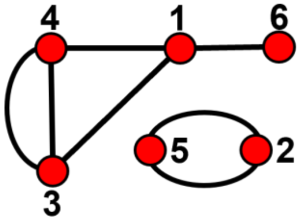
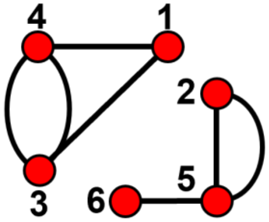
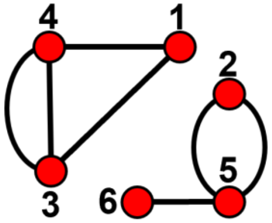

	(6x**1**, 4x$\mathbf{2}^2$) **a** 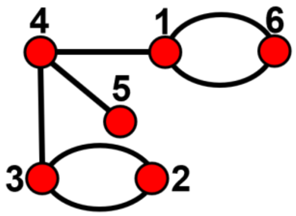	(6x**1**, 4x$\mathbf{2}^2$) **b** 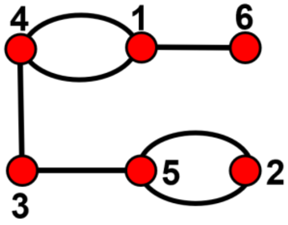	(6x**1**, 4x$\mathbf{2}^2$) **c** 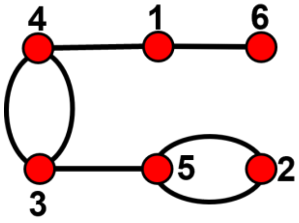	(6x**1**, 4x$\mathbf{2}^2$) **d** 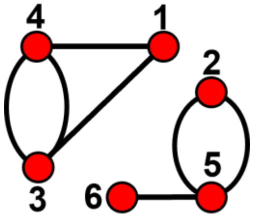
	(6x**1**, 4x$\mathbf{2}^2$) **e** 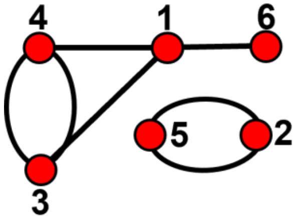	(4x**1**, 5x**2**) 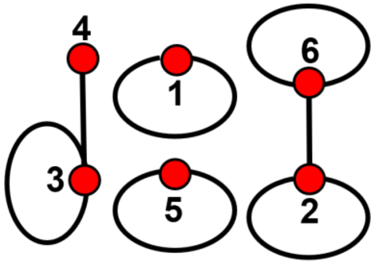	(4x**1**, 3x**2**, 2x$\mathbf{2}^1$) **a** 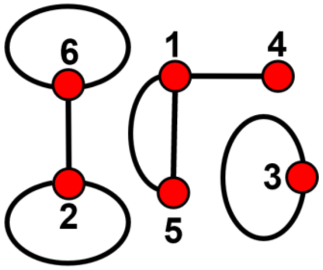	(4x**1**, 3x**2**, 2x$\mathbf{2}^1$) **b** 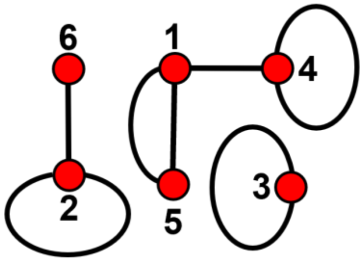
	(4x**1**, 3x**2**, 2x$\mathbf{2}^1$) **c** 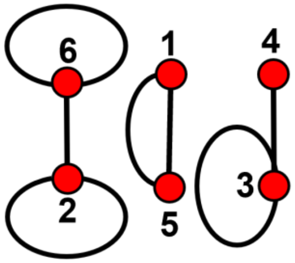	(4x**1**, 3x**2**, 2x$\mathbf{2}^1$) **d** 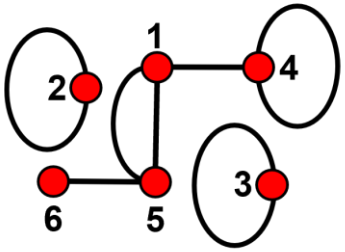	(4x**1**, 3x**2**, 2x$\mathbf{2}^2$) **a** 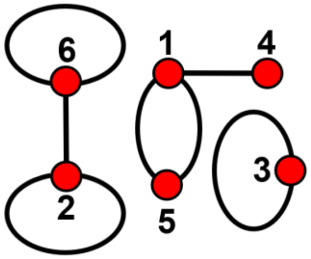	(4x**1**, 3x**2**, 2x$\mathbf{2}^2$) **b** 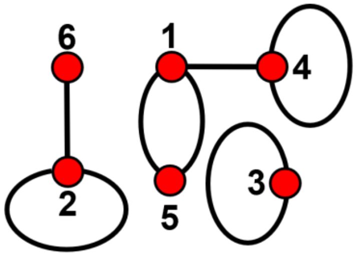

	(4x**1**, 3x**2**, 2x$\mathbf{2}^2$) **c**<br>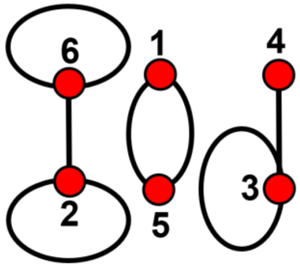	(4x**1**, 3x**2**, 2x$\mathbf{2}^2$) **d**<br>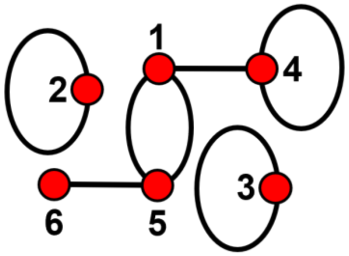	(4x**1**, 1x**2**, 4x$\mathbf{2}^1$) **a**<br>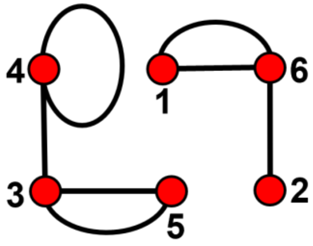	(4x**1**, 1x**2**, 4x$\mathbf{2}^1$) **b**<br>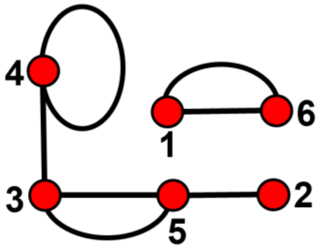
	(4x**1**, 1x**2**, 4x$\mathbf{2}^1$) **c**<br>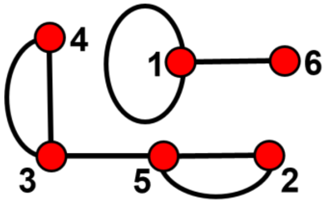	(4x**1**, 1x**2**, 4x$\mathbf{2}^1$) **d**<br>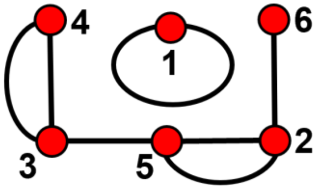	(4x**1**, 1x**2**, 2x$\mathbf{2}^1$, 2x$\mathbf{2}^2$) **a**<br>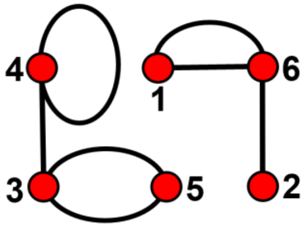	(4x**1**, 1x**2**, 2x$\mathbf{2}^1$, 2x$\mathbf{2}^2$) **b**<br>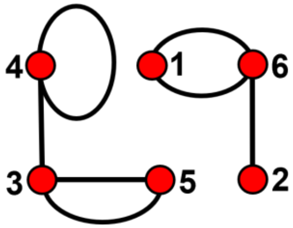
	(4x**1**, 1x**2**, 2x$\mathbf{2}^1$, 2x$\mathbf{2}^2$) **c**<br>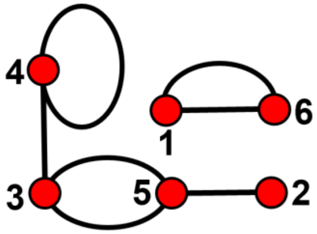	(4x**1**, 1x**2**, 2x$\mathbf{2}^1$, 2x$\mathbf{2}^2$) **d**<br>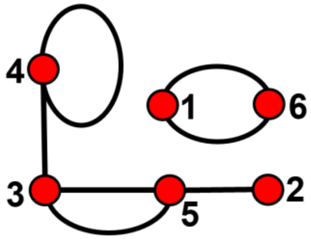	(4x**1**, 1x**2**, 2x$\mathbf{2}^1$, 2x$\mathbf{2}^2$) **e**<br>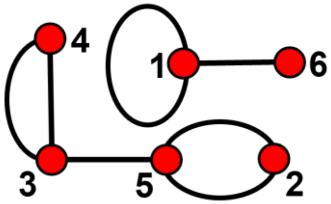	(4x**1**, 1x**2**, 2x$\mathbf{2}^1$, 2x$\mathbf{2}^2$) **f**<br>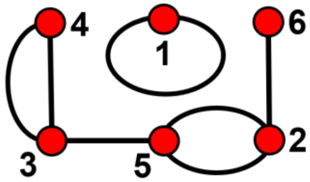

	(4x**1**, 1x**2**, 2x$\mathbf{2}^1$, 2x$\mathbf{2}^2$) **g** 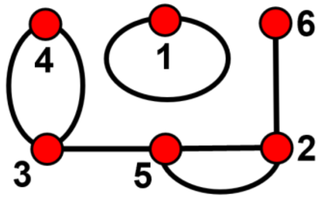	(4x**1**, 1x**2**, 4x$\mathbf{2}^2$) **a** 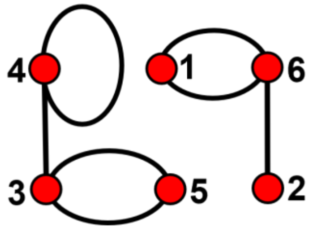	(4x**1**, 1x**2**, 4x$\mathbf{2}^2$) **b** 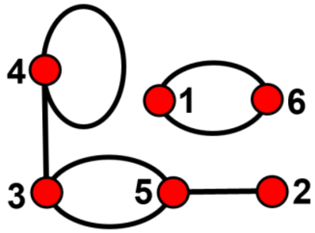	(4x**1**, 1x**2**, 4x$\mathbf{2}^2$) **c** 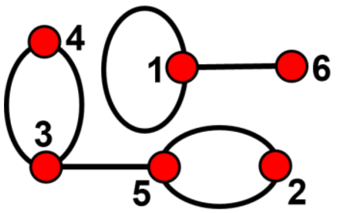
	(4x**1**, 1x**2**, 4x$\mathbf{2}^2$) **d** 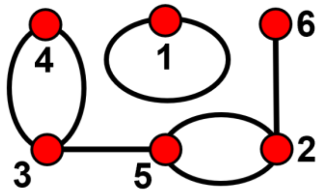			
$^1V_1{}^2V_2{}^3V_5$	NG			
$^1V_1{}^2V_3{}^3V_1$	(10x**1**) **a** 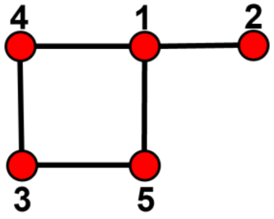	(10x**1**) **b** 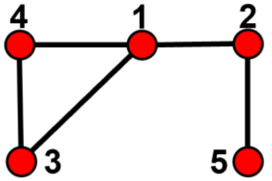	(8x**1**, 1x**2**) **a** 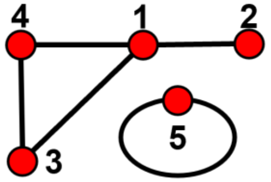	(8x**1**, 1x**2**) **b** 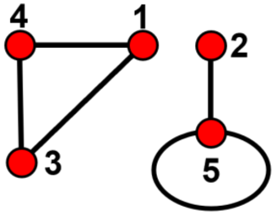
	(8x**1**, 1x**2**) **c** 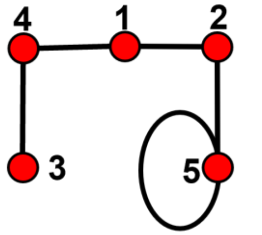	(6x**1**, 2x**2**) 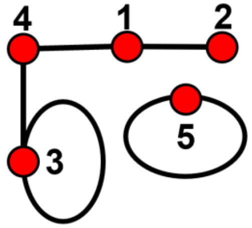	(6x**1**, 2x$\mathbf{2}^1$) 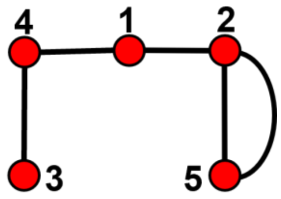	(6x**1**, 2x$\mathbf{2}^2$) 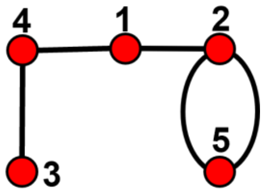

	(4x**1**, 3x**2**) 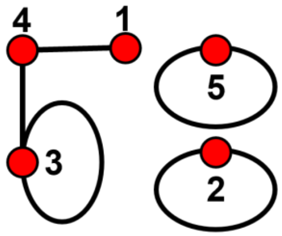	(4x**1**, 1x**2**, 2x**2**^{1}) **a** 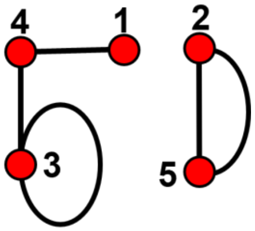	(4x**1**, 1x**2**, 2x**2**^{1}) **b** 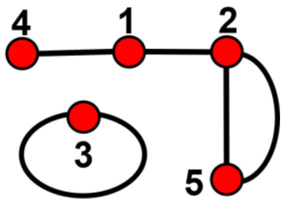	(4x**1**, 1x**2**, 2x**2**^{2}) **a** 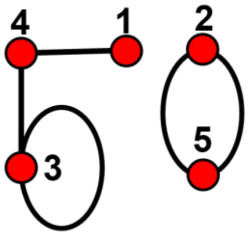
	(4x**1**, 1x**2**, 2x**2**^{2}) **b** 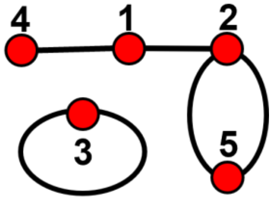	(2x**1**, 4x**2**) 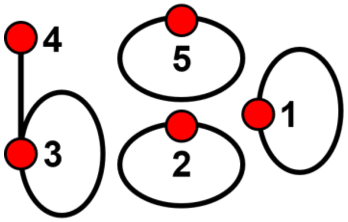	(2x**1**, 2x**2**, 2x**2**^{1}) **a** 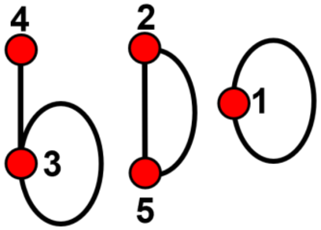	(2x**1**, 2x**2**, 2x**2**^{1}) **b** 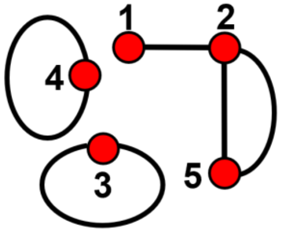
	(2x**1**, 2x**2**, 2x**2**^{2}) **a** 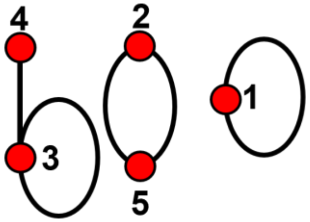	(2x**1**, 2x**2**, 2x**2**^{2}) **b** 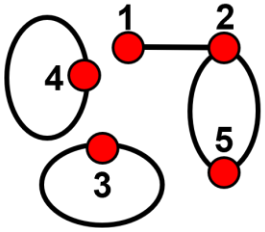	(2x**1**, 4x**2**^{1}) 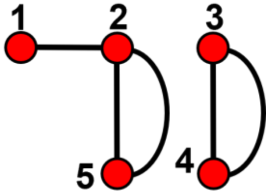	(2x**1**, 2x**2**^{1}, 2x**2**^{2}) **a** 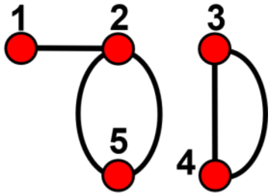
	(2x**1**, 2x**2**^{1}, 2x**2**^{2}) **b** 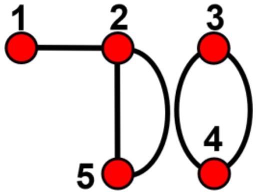	(2x**1**, 4x**2**^{2}) 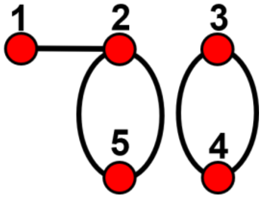		

$^1V_1{}^2V_3{}^3V_3$	NG			
$^1V_1{}^2V_4{}^3V_1$	(12x**1**) **a**<br>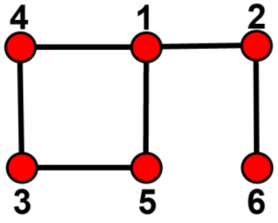	(12x**1**) **b**<br>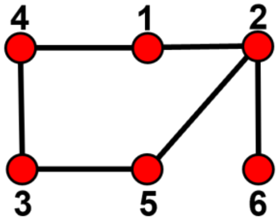	(12x**1**) **c**<br>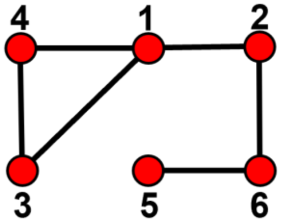	(10x**1**, 1x**2**) **a**<br>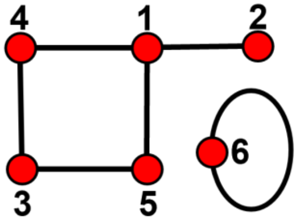
	(10x**1**, 1x**2**) **b**<br>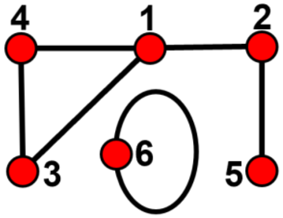	(10x**1**, 1x**2**) **c**<br>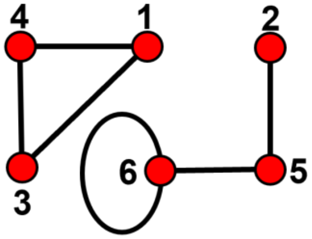	(10x**1**, 1x**2**) **d**<br>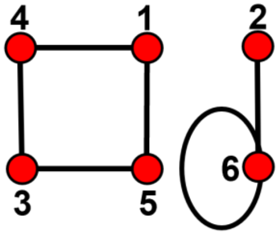	(10x**1**, 1x**2**) **e**<br>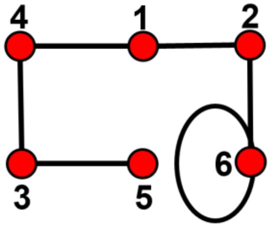
	(8x**1**, 2x**2**) **a**<br>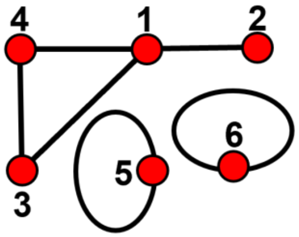	(8x**1**, 2x**2**) **b**<br>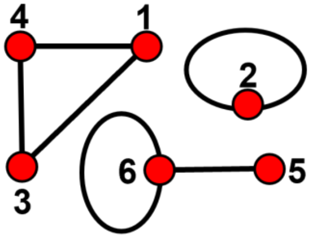	(8x**1**, 2x**2**) **c**<br>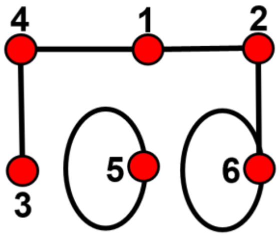	(8x**1**, 2x$\mathbf{2^1}$) **a**<br>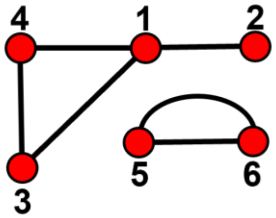

	(8x**1**, 2x**2**^{1}) **b**<br>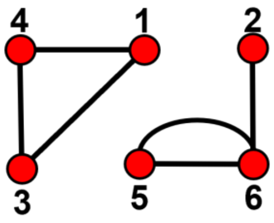	(8x**1**, 2x**2**^{1}) **c**<br>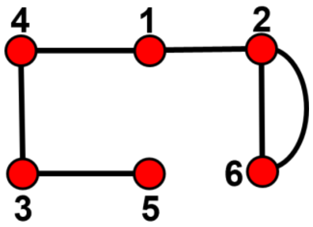	(8x**1**, 2x**2**^{2}) **a**<br>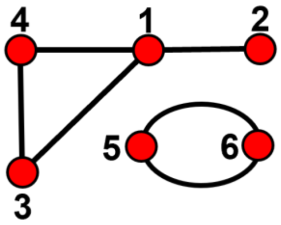	(8x**1**, 2x**2**^{2}) **b**<br>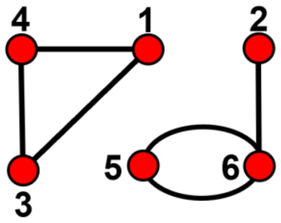
	(8x**1**, 2x**2**^{2}) **c**<br>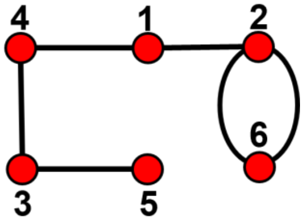	(6x**1**, 3x**2**)<br>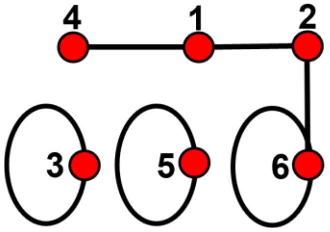	(6x**1**, 1x**2**, 2x**2**^{1}) **a**<br>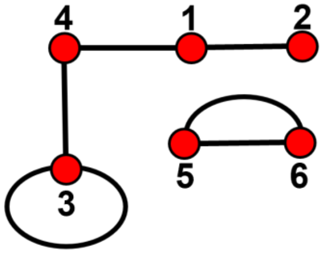	(6x**1**, 1x**2**, 2x**2**^{1}) **b**<br>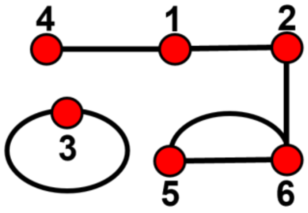
	(6x**1**, 1x**2**, 2x**2**^{2}) **a**<br>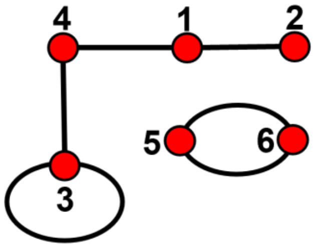	(6x**1**, 1x**2**, 2x**2**^{2}) **b**<br>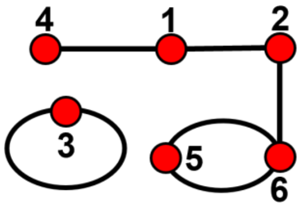	(4x**1**, 4x**2**)<br>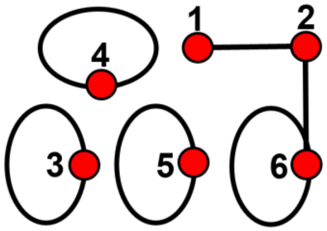	(4x**1**, 2x**2**, 2x**2**^{1}) **a**<br>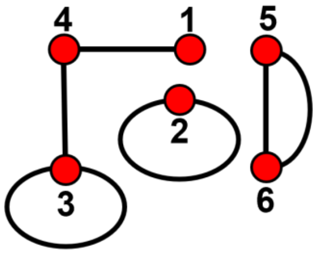

	(4x**1**, 2x**2**, 2x**2**^{1}) **b** 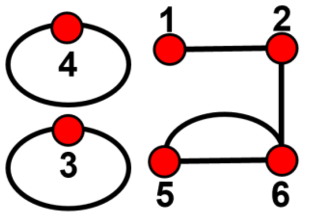	(4x**1**, 2x**2**, 2x**2**^{2}) **a** 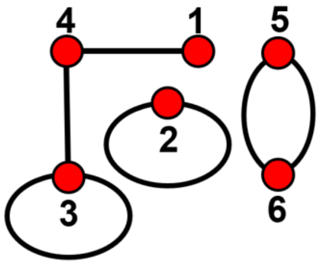	(4x**1**, 2x**2**, 2x**2**^{2}) **b** 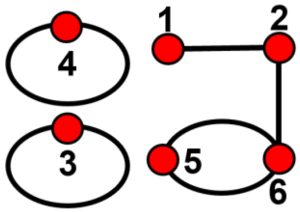	(4x**1**, 4x**2**^{1}) 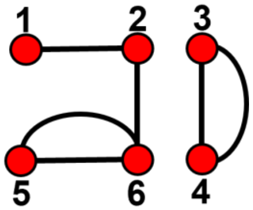
	(4x**1**, 2x**2**^{1}, 2x**2**^{2}) **a** 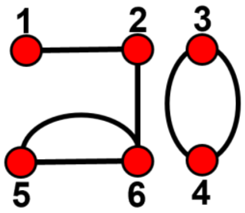	(4x**1**, 2x**2**^{1}, 2x**2**^{2}) **b** 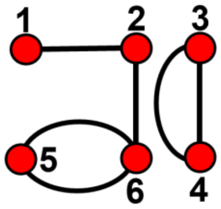	(4x**1**, 4x**2**^{2}) 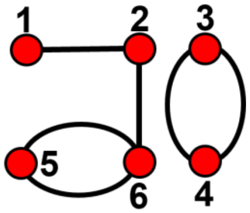	(2x**1**, 5x**2**) 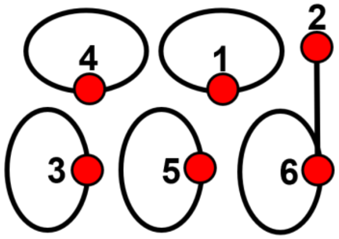
	(2x**1**, 3x**2**, 2x**2**^{1}) **a** 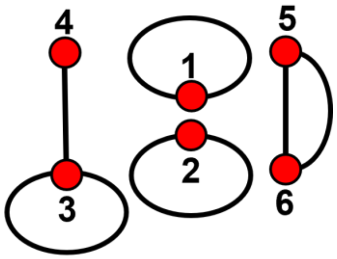	(2x**1**, 3x**2**, 2x**2**^{1}) **b** 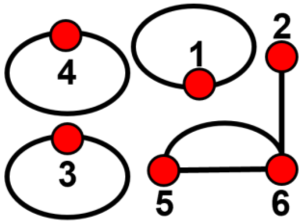	(2x**1**, 3x**2**, 2x**2**^{2}) **a** 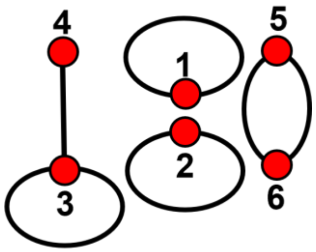	(2x**1**, 3x**2**, 2x**2**^{2}) **b** 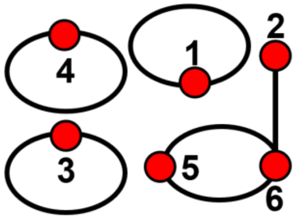

	(2x**1**, 1x**2**, 4x**2**^{1}) **a** 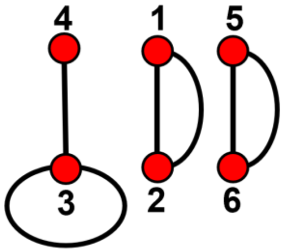	(2x**1**, 1x**2**, 4x**2**^{1}) **b** 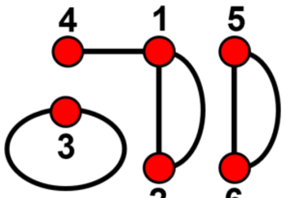	(2x**1**, 1x**2**, 2x**2**^{1}, 2x**2**^{2}) **a** 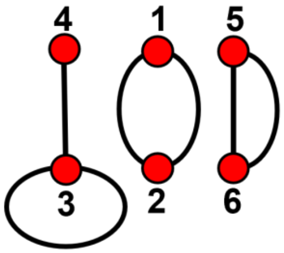	(2x**1**, 1x**2**, 2x**2**^{1}, 2x**2**^{2}) **b** 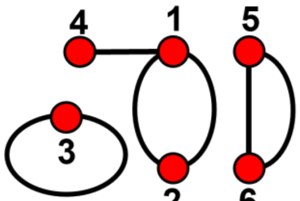
	(2x**1**, 1x**2**, 2x**2**^{1}, 2x**2**^{2}) **c** 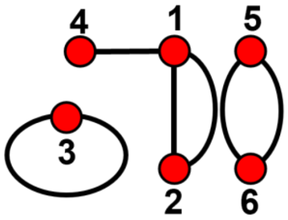	(2x**1**, 1x**2**, 4x**2**^{2}) **a** 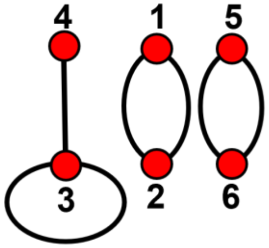	(2x**1**, 1x**2**, 4x**2**^{2}) **b** 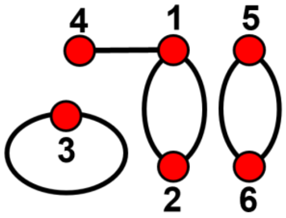	
$^1V_1{}^2V_4{}^3V_3$	NG			
$^1V_1{}^2V_5{}^3V_1$	NG			
$^1V_1{}^2V_6{}^3V_1$	NG			
$^1V_2{}^2V_1{}^3V_2$	(10x**1**) 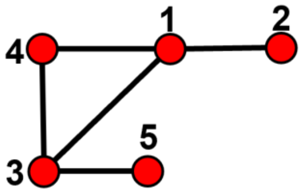	(8x**1**, 1x**2**) **a** 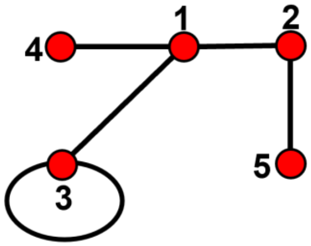	(8x**1**, 1x**2**) **b** 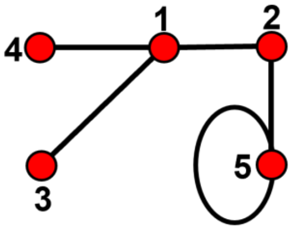	(6x**1**, 2x**2**) **a** 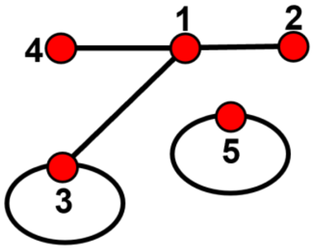

	(6x**1**, 2x**2**) **b** 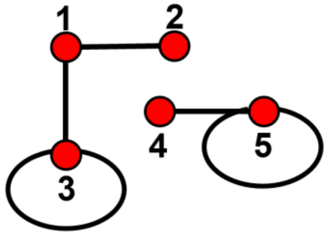	(6x**1**, 2x**2**) **c** 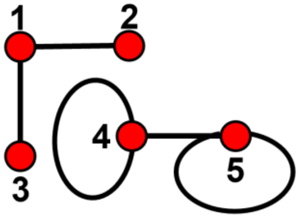	(6x**1**, 2x**2**) **d** 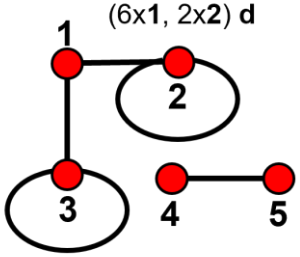	(6x**1**, 2x**2**^{1}) **a** 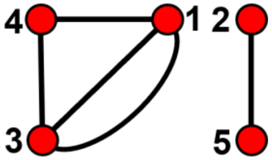
	(6x**1**, 2x**2**^{1}) **b** 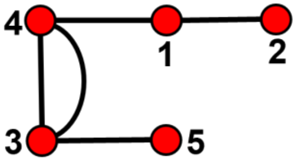	(6x**1**, 2x**2**^{1}) **c** 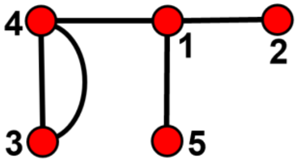	(6x**1**, 2x**2**^{2}) **a** 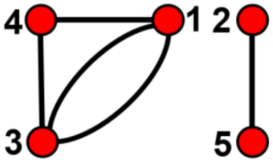	(6x**1**, 2x**2**^{2}) **b** 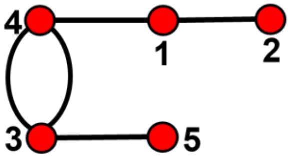
	(6x**1**, 2x**2**^{2}) **c** 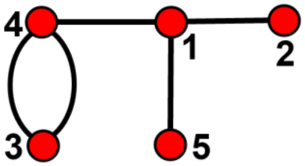	(4x**1**, 3x**2**) **a** 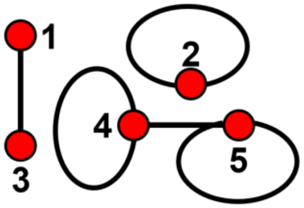	(4x**1**, 3x**2**) **b** 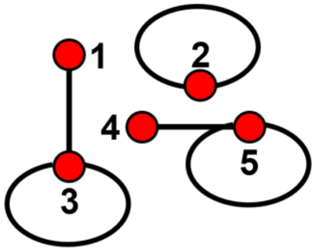	(4x**1**, 1x**2**, 2x**2**^{1}) **a** 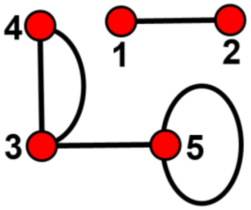
	(4x**1**, 1x**2**, 2x**2**^{1}) **b** 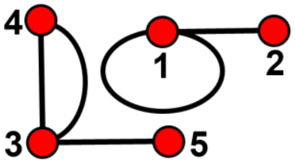	(4x**1**, 1x**2**, 2x**2**^{1}) **c** 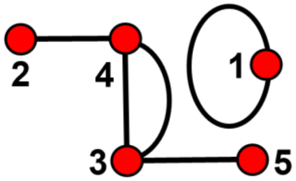	(4x**1**, 1x**2**, 2x**2**^{2}) **a** 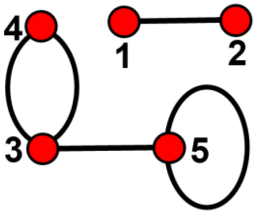	(4x**1**, 1x**2**, 2x**2**^{2}) **b** 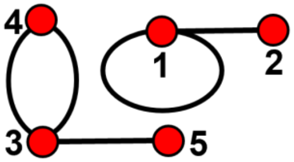

	(4x**1**, 1x**2**, 2x**2**^{2}) **c**<br>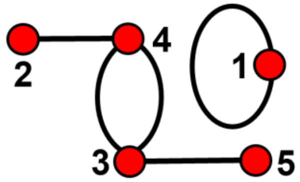 			
$^1V_2{}^2V_1{}^3V_4$	NG			
$^1V_2{}^2V_2{}^3V_2$	(12x**1**) **a**<br>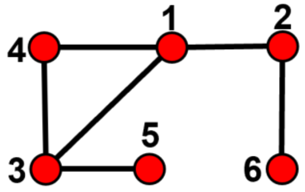 	(12x**1**) **b**<br>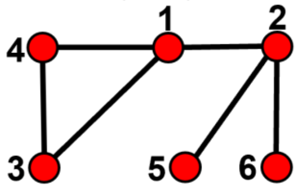 	(12x**1**) **c**<br>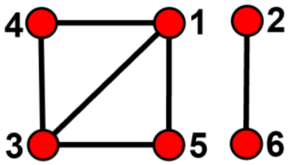 	(12x**1**) **d**<br>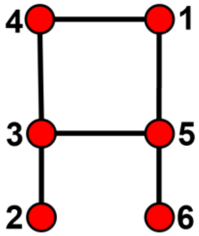
	(12x**1**) **e**<br>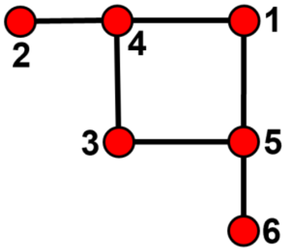 	(10x**1**, 1x**2**) **a**<br>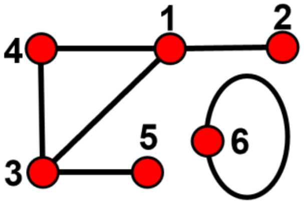 	(10x**1**, 1x**2**) **b**<br>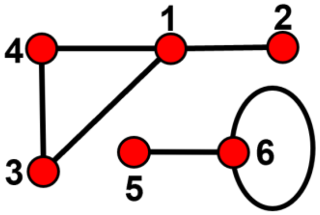 	(10x**1**, 1x**2**) **c**<br>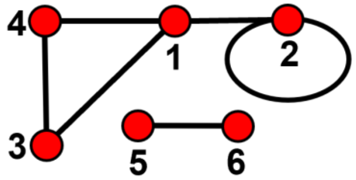

	(10x**1**, 1x**2**) **d** 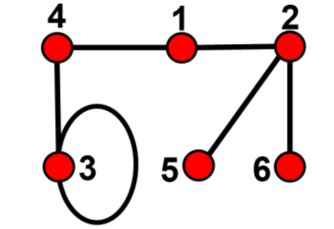	(10x**1**, 1x**2**) **e** 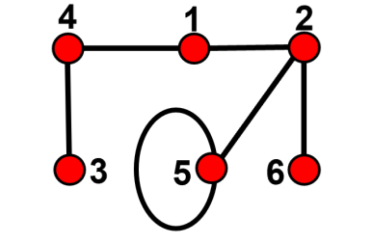	(10x**1**, 1x**2**) **f** 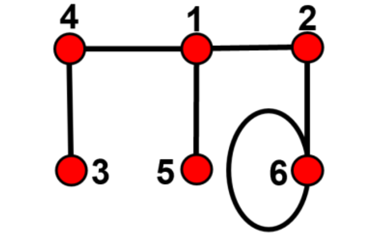	(10x**1**, 1x**2**) **g** 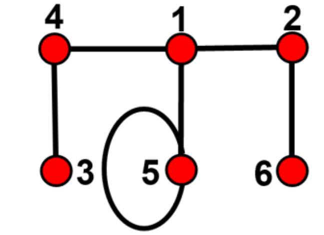
	(8x**1**, 2x**2**) **a** 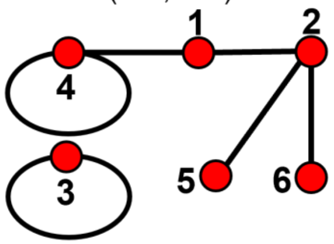	(8x**1**, 2x**2**) **b** 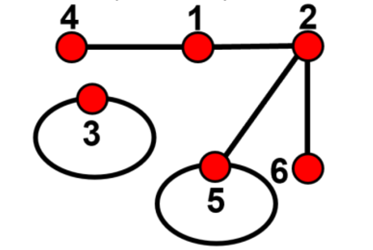	(8x**1**, 2x**2**) **c** 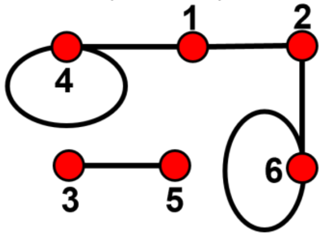	(8x**1**, 2x**2**) **d** 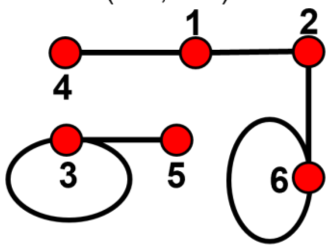
	(8x**1**, 2x**2**) **e** 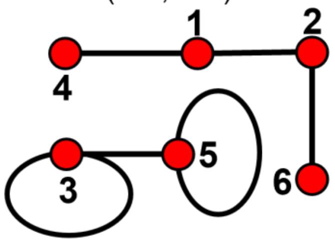	(8x**1**, 2x**2**) **f** 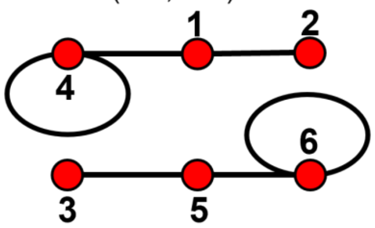	(8x**1**, 2x**2**) **g** 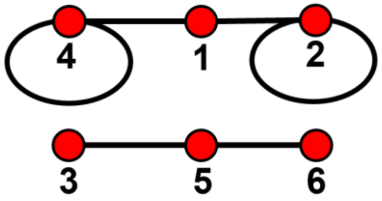	(8x**1**, 2x**2**^{1}) **a** 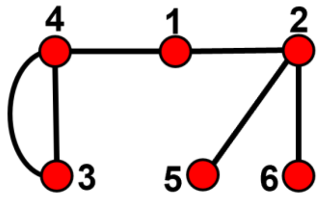
	(8x**1**, 2x**2**^{1}) **b** 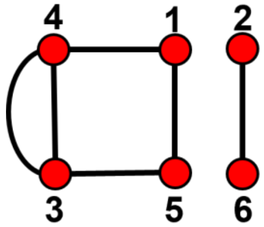	(8x**1**, 2x**2**^{1}) **c** 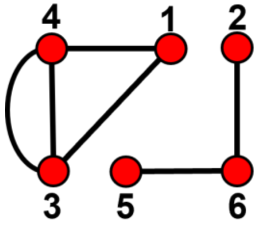	(8x**1**, 2x**2**^{1}) **d** 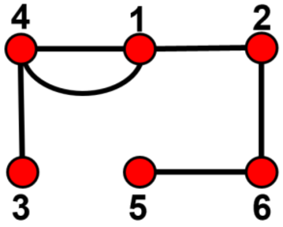	(8x**1**, 2x**2**^{1}) **e** 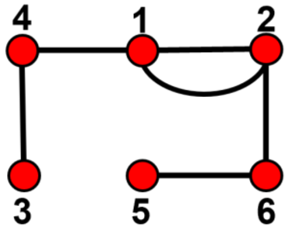

	(8x**1**, 2x**2**^{1}) **f** 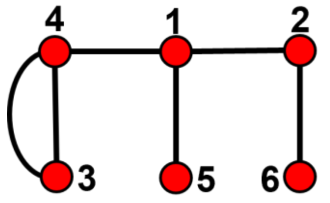	(8x**1**, 2x**2**^{2}) **a** 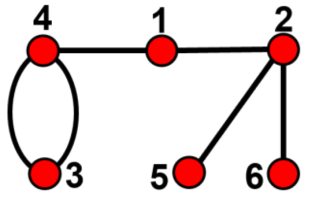	(8x**1**, 2x**2**^{2}) **b** 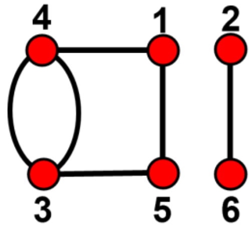	(8x**1**, 2x**2**^{2}) **c** 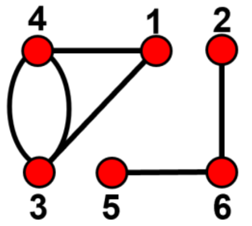
	(8x**1**, 2x**2**^{2}) **d** 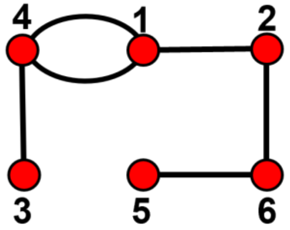	(8x**1**, 2x**2**^{2}) **e** 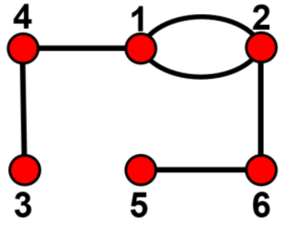	(8x**1**, 2x**2**^{2}) **f** 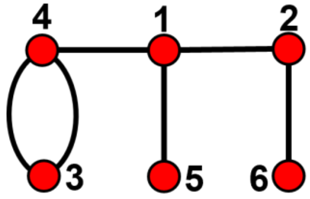	(6x**1**, 3x**2**) **a** 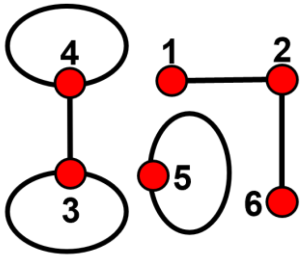
	(6x**1**, 3x**2**) **b** 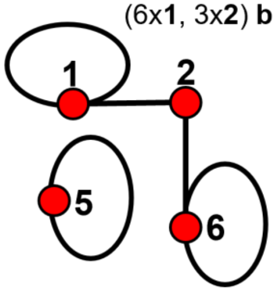	(6x**1**, 3x**2**) **c** 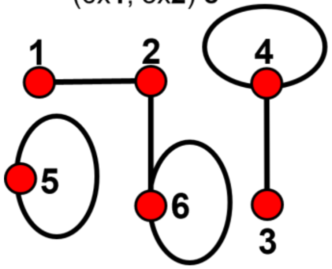	(6x**1**, 3x**2**) **d** 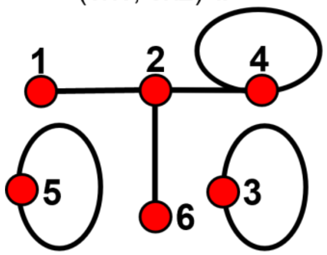	(6x**1**, 1x**2**, 2x**2**^{1}) **a** 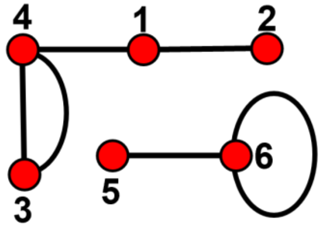

(6x**1**, 1x**2**, 2x**2**^{1}) **b**

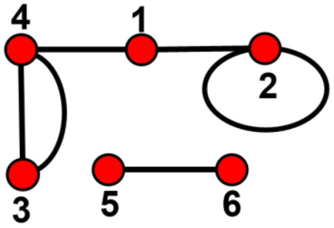

(6x**1**, 1x**2**, 2x**2**^{1}) **c**

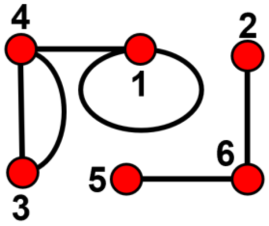

(6x**1**, 1x**2**, 2x**2**^{1}) **d**

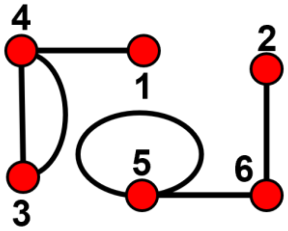

(6x**1**, 1x**2**, 2x**2**^{1}) **e**

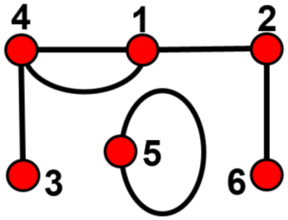

(6x**1**, 1x**2**, 2x**2**^{1}) **f**

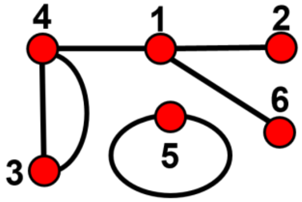

(6x**1**, 1x**2**, 2x**2**^{1}) **g**

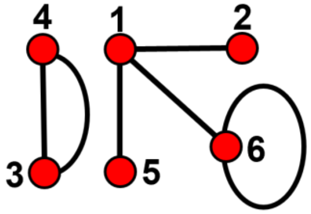

(6x**1**, 1x**2**, 2x**2**^{1}) **h**

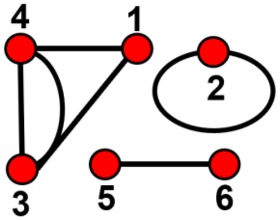

(6x**1**, 1x**2**, 2x**2**^{2}) **a**

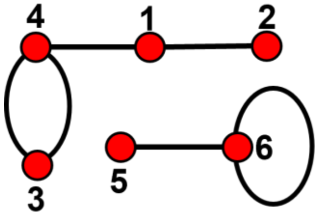

(6x**1**, 1x**2**, 2x**2**^{2}) **b**

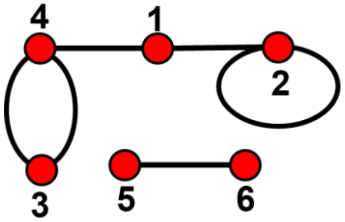

(6x**1**, 1x**2**, 2x**2**^{2}) **c**

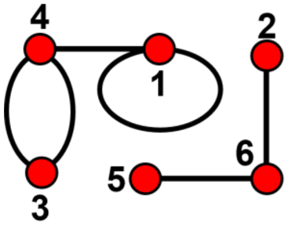

(6x**1**, 1x**2**, 2x**2**^{2}) **d**

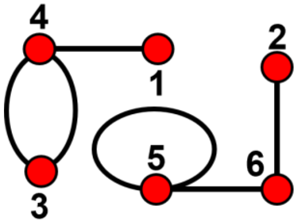

(6x**1**, 1x**2**, 2x**2**^{2}) **e**

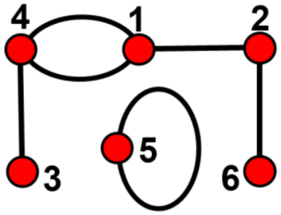

	(6x**1**, 1x**2**, 2x$\mathbf{2}^{2}$) **f**	(6x**1**, 1x**2**, 2x$\mathbf{2}^{2}$) **g**	(6x**1**, 1x**2**, 2x$\mathbf{2}^{2}$) **h**	(4x**1**, 4x**2**) **a**
	(4x**1**, 4x**2**) **b**	(4x**1**, 2x**2**, 2x$\mathbf{2}^{1}$) **a**	(4x**1**, 2x**2**, 2x$\mathbf{2}^{1}$) **b**	(4x**1**, 2x**2**, 2x$\mathbf{2}^{1}$) **c**
	(4x**1**, 2x**2**, 2x$\mathbf{2}^{1}$) **d**	(4x**1**, 2x**2**, 2x$\mathbf{2}^{1}$) **e**	(4x**1**, 2x**2**, 2x$\mathbf{2}^{2}$) **a**	(4x**1**, 2x**2**, 2x$\mathbf{2}^{2}$) **b**

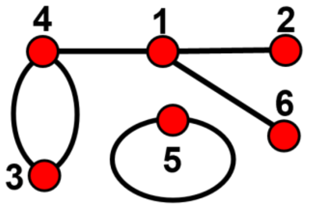

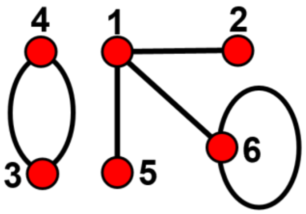

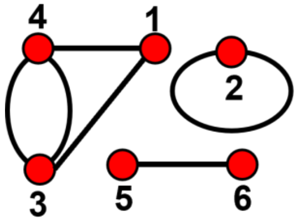

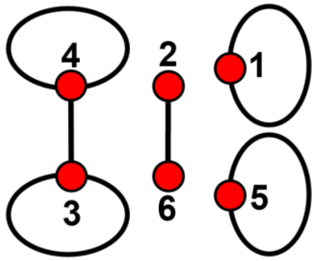

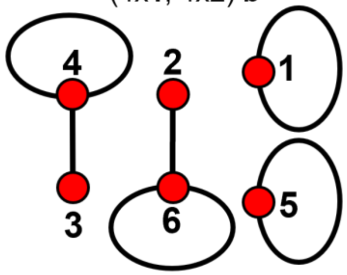

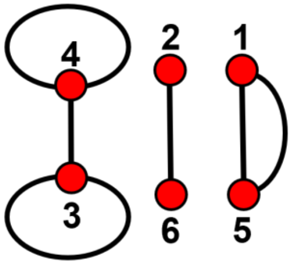

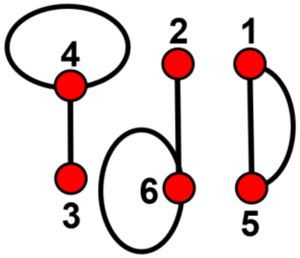

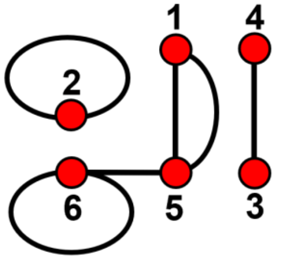

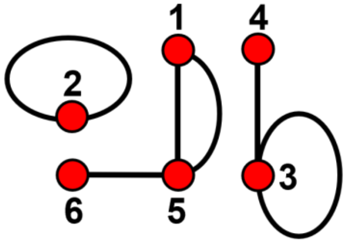

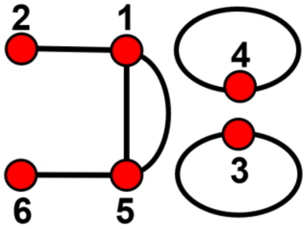

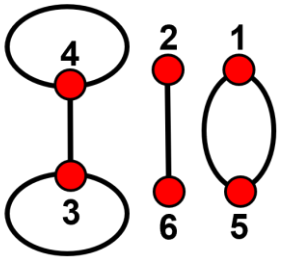

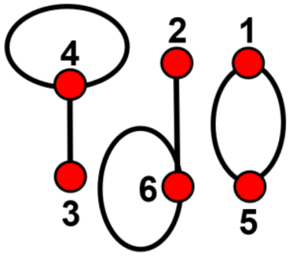

	(4x**1**, 2x**2**, 2x$\mathbf{2}^2$) **c**<br>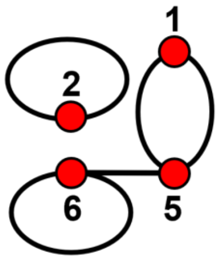	(4x**1**, 2x**2**, 2x$\mathbf{2}^2$) **d**<br>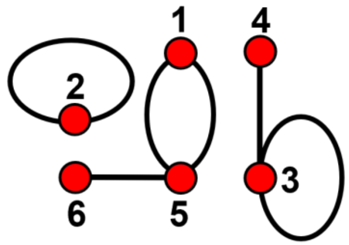	(4x**1**, 2x**2**, 2x$\mathbf{2}^2$) **e**<br>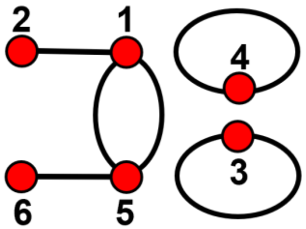	(4x**1**, 4x$\mathbf{2}^1$) **a**<br>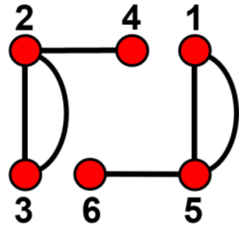
	(4x**1**, 4x$\mathbf{2}^1$) **b**<br>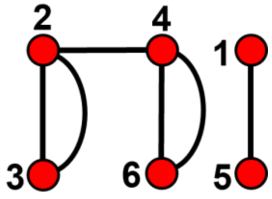	(4x**1**, 4x$\mathbf{2}^1$) **c**<br>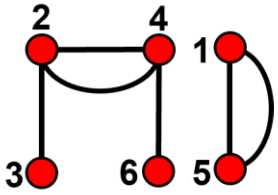	(4x**1**, 2x$\mathbf{2}^1$, 2x$\mathbf{2}^2$) **a**<br>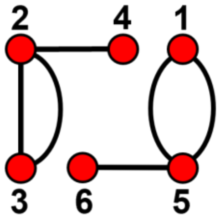	(4x**1**, 2x$\mathbf{2}^1$, 2x$\mathbf{2}^2$) **b**<br>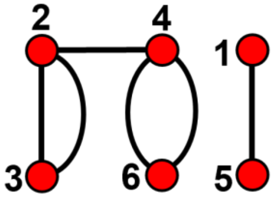
	(4x**1**, 2x$\mathbf{2}^1$, 2x$\mathbf{2}^2$) **c**<br>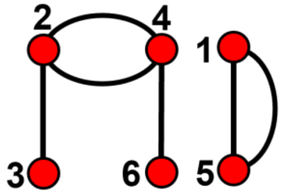	(4x**1**, 2x$\mathbf{2}^1$, 2x$\mathbf{2}^2$) **d**<br>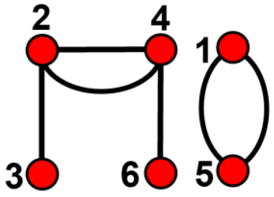	(4x**1**, 4x$\mathbf{2}^2$) **a**<br>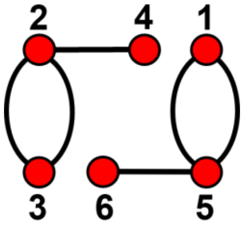	(4x**1**, 4x$\mathbf{2}^2$) **b**<br>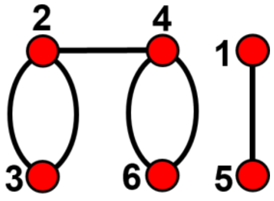
	(4x**1**, 4x$\mathbf{2}^2$) **c**<br>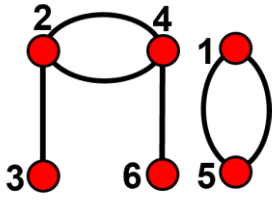			

$^1V_2{}^2V_2{}^3V_4$	NG			
$^1V_2{}^2V_3{}^3V_2$	NG			
$^1V_2{}^2V_4{}^3V_2$	NG			
$^1V_3{}^2V_1{}^3V_1$	(8x**1**) 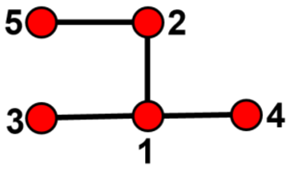	(6x**1**, 1x**2**) **a** 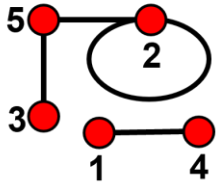	(6x**1**, 1x**2**) **b** 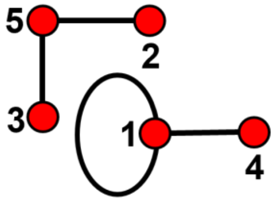	(6x**1**, 1x**2**) **c** 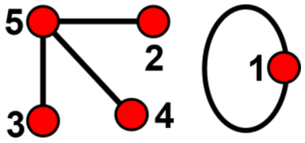
	(4x**1**, 2x**2**) 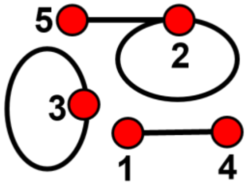	(4x**1**, 2x**2**^{1}) 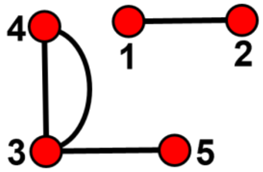	(4x**1**, 2x**2**^{2}) 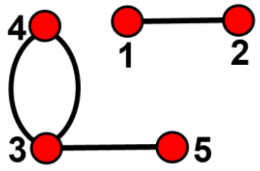	
$^1V_3{}^2V_1{}^3V_3$	NG			
$^1V_3{}^2V_2{}^3V_1$	(10x**1**) **a** 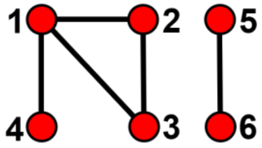	(10x**1**) **b** 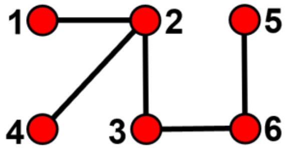	(10x**1**) **c** 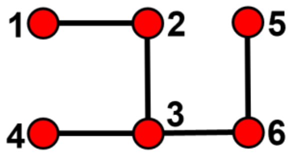	(8x**1**, 1x**2**) **a** 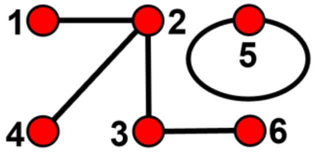

	(8x**1**, 1x**2**) **b** 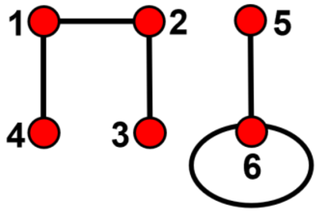	(8x**1**, 1x**2**) **c** 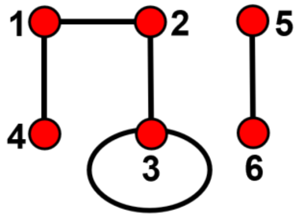	(8x**1**, 1x**2**) **d** 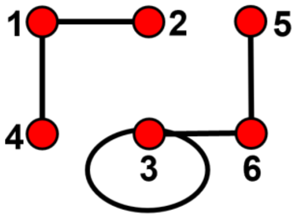	(6x**1**, 2x**2**) **a** 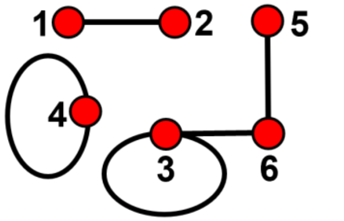
	(6x**1**, 2x**2**) **b** 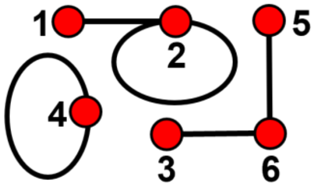	(6x**1**, 2x**2**) **c** 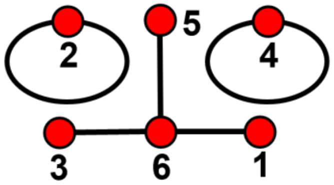	(6x**1**, 2x**2**^{1}) **a** 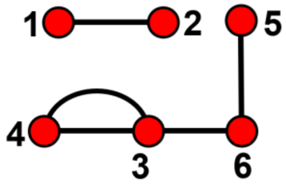	(6x**1**, 2x**2**^{1}) **b** 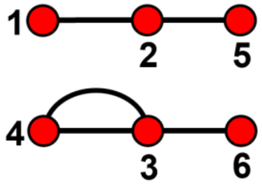
	(6x**1**, 2x**2**^{1}) **c** 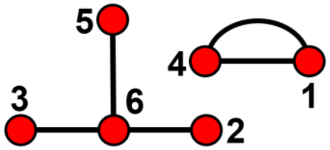	(6x**1**, 2x**2**^{2}) **a** 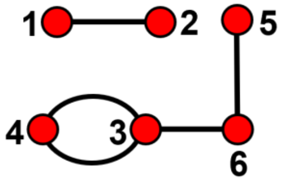	(6x**1**, 2x**2**^{2}) **b** 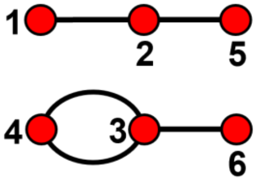	(6x**1**, 2x**2**^{2}) **c** 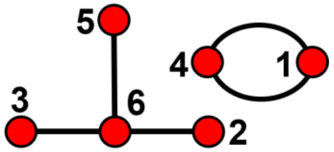
	(4x**1**, 3x**2**) 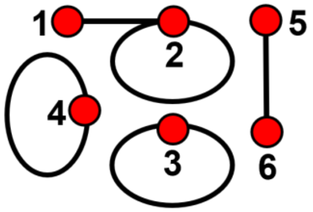	(4x**1**, 1x**2**, 2x**2**^{1}) **a** 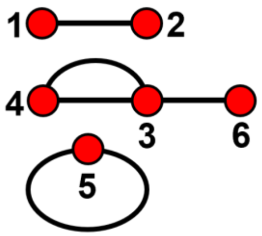	(4x**1**, 1x**2**, 2x**2**^{1}) **b** 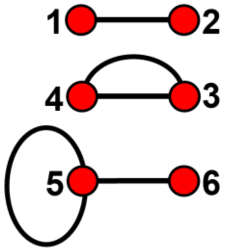	(4x**1**, 1x**2**, 2x**2**^{2}) **a** 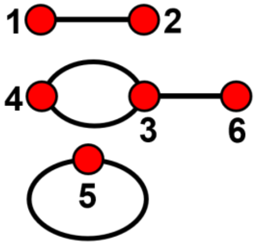

	(4x**1**, 1x**2**, 2x**2**^{2}) **b** 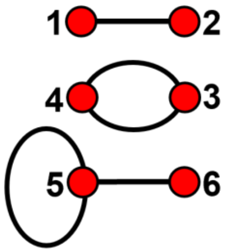			
$^1V_3{}^2V_2{}^3V_3$	NG			
$^1V_3{}^2V_3{}^3V_1$	NG			
$^1V_3{}^2V_4{}^3V_1$	NG			
$^1V_4{}^2V_1{}^3V_2$	(12x**1**) **a** 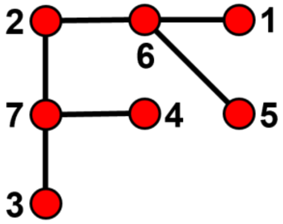	(12x**1**) **b** 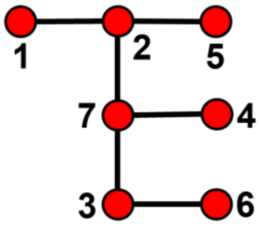	(12x**1**) **c** 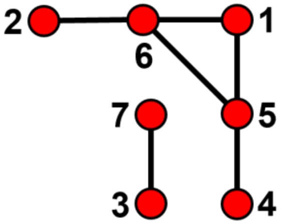	(10x**1**, 1x**2**) **a** 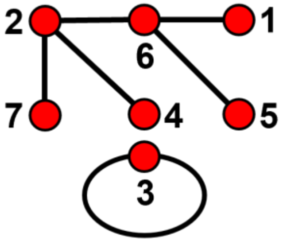
	(10x**1**, 1x**2**) **b** 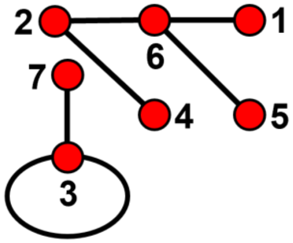	(10x**1**, 1x**2**) **c** 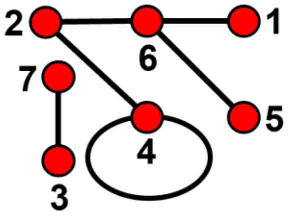	(10x**1**, 1x**2**) **d** 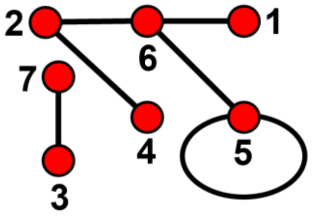	(10x**1**, 1x**2**) **e** 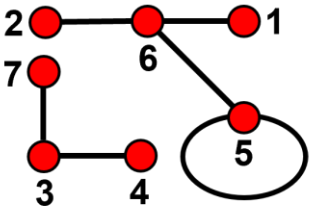

	(10x**1**, 1x**2**) **f**<br>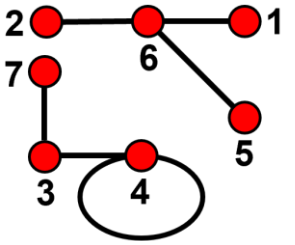	(8x**1**, 2x**2**) **a**<br>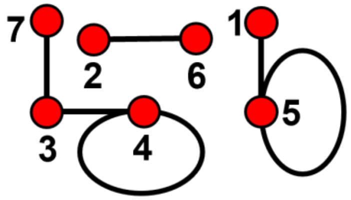	(8x**1**, 2x**2**) **b**<br>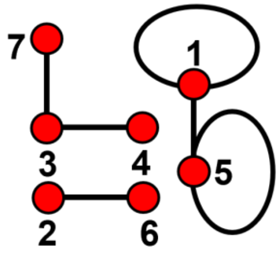	(8x**1**, 2x**2**) **c**<br>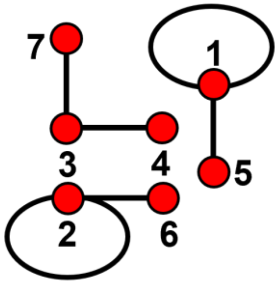
	(8x**1**, 2x**2**) **d**<br>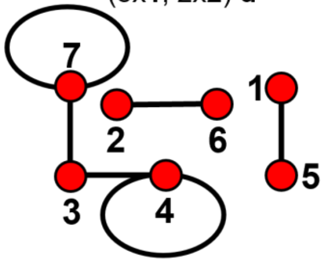	(8x**1**, 2x**2**) **e**<br>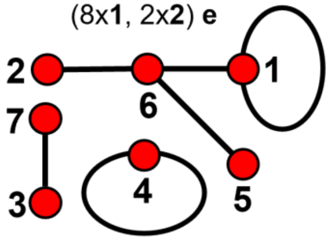	(8x**1**, 2x**2**) **f**<br>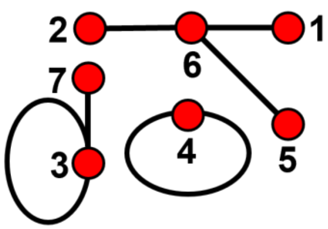	(8x**1**, 2x**2**^{1}) **a**<br>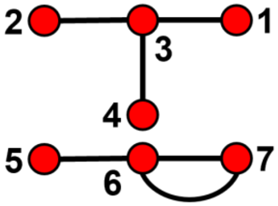
	(8x**1**, 2x**2**^{1}) **b**<br>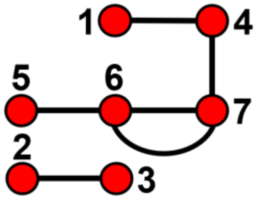	(8x**1**, 2x**2**^{1}) **c**<br>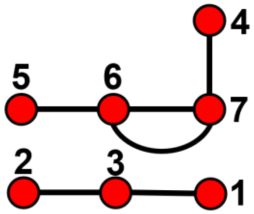	(8x**1**, 2x**2**^{1}) **d**<br>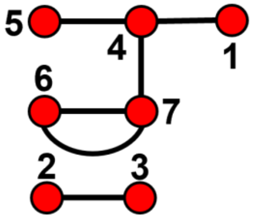	(8x**1**, 2x**2**^{1}) **e**<br>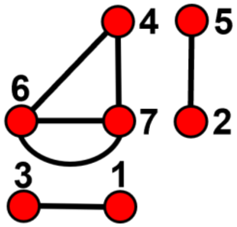

	(8x**1**, 2x**2**^{2}) **a**<br>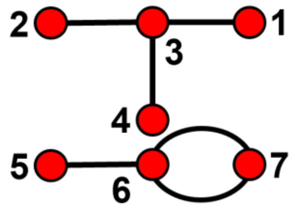	(8x**1**, 2x**2**^{2}) **b**<br>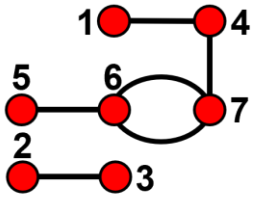	(8x**1**, 2x**2**^{2}) **c**<br>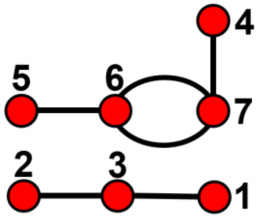	(8x**1**, 2x**2**^{2}) **d**<br>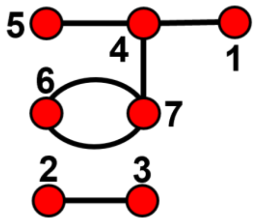
	(8x**1**, 2x**2**^{2}) **e**<br>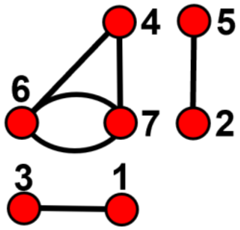	(6x**1**, 3x**2**) **a**<br>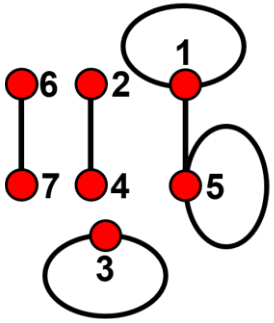	(6x**1**, 3x**2**) **b**<br>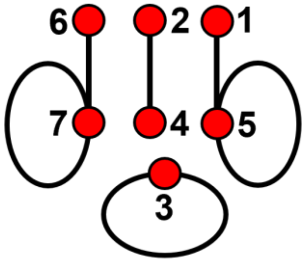	(6x**1**, 1x**2**, 2x**2**^{1}) **a**<br>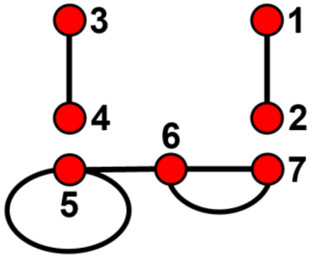
	(6x**1**, 1x**2**, 2x**2**^{1}) **b**<br>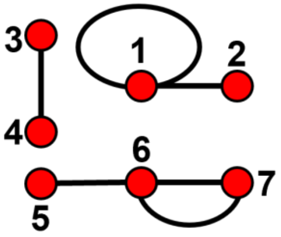	(6x**1**, 1x**2**, 2x**2**^{1}) **c**<br>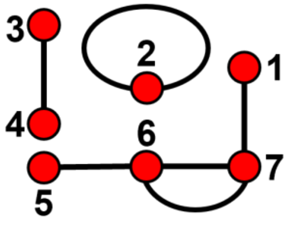	(6x**1**, 1x**2**, 2x**2**^{2}) **a**<br>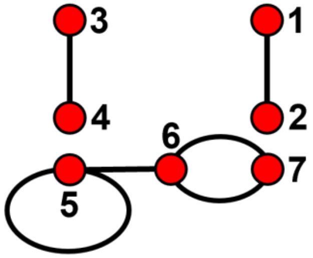	(6x**1**, 1x**2**, 2x**2**^{2}) **b**<br>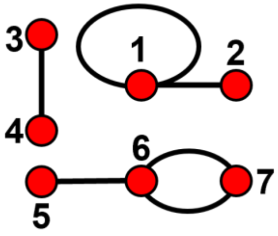

	(6x**1**, 1x**2**, 2x**2**^{2}) **c** 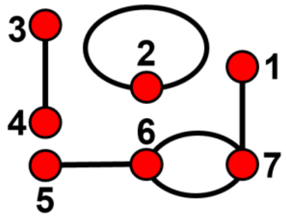			
${}^1V_4{}^2V_2{}^3V_2$	NG			
${}^1V_5{}^2V_1{}^3V_1$	(10x**1**) **a** 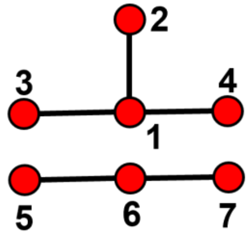	(10x**1**) **b** 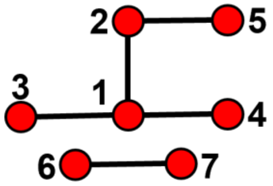	(8x**1**, 1x**2**) **a** 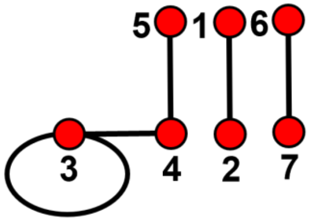	(8x**1**, 1x**2**) **b** 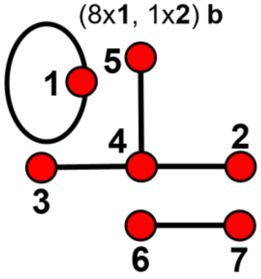
	(8x**1**, 1x**2**) **c** 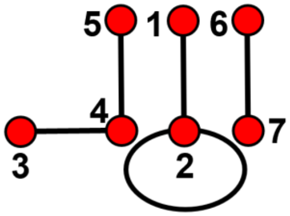	(6x**1**, 2x**2**) 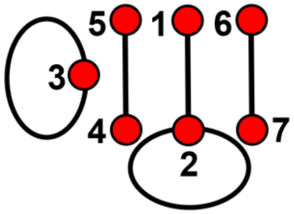	(6x**1**, 2x**2**^{1}) 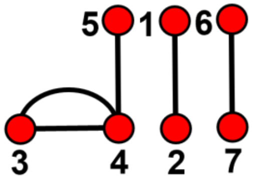	(6x**1**, 2x**2**^{2}) 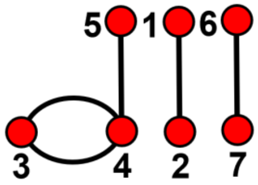
${}^1V_5{}^2V_2{}^3V_1$	NG			
$\boldsymbol{{}^1V_r{}^2V_r{}^4V_r}$				

$^1V_2{}^2V_1{}^4V_1$	(6x**1**, 1x**2**) 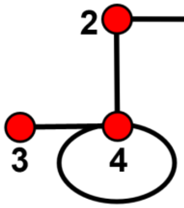	(4x**1**, 2x**2**) 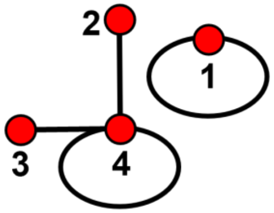	(4x**1**, 2x$\mathbf{2}^1$) 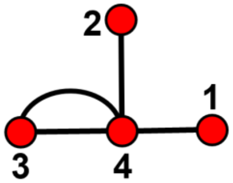	(4x**1**, 2x$\mathbf{2}^2$) 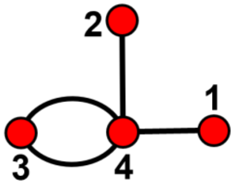
	(2x**1**, 1x**2**, 2x$\mathbf{2}^1$) 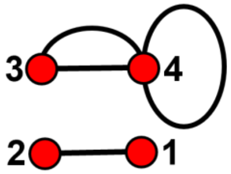	(2x**1**, 1x**2**, 2x$\mathbf{2}^2$) 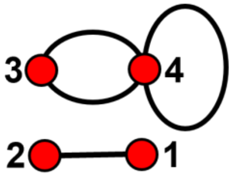		
$^1V_2{}^2V_1{}^4V_2$	(10x**1**, 1x**2**) 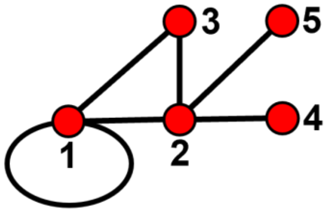	(8x**1**, 2x**2**) **a** 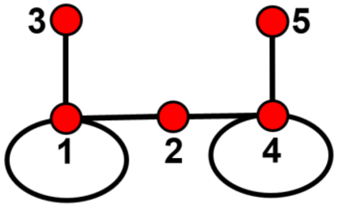	(8x**1**, 2x**2**) **b** 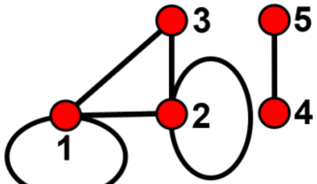	(8x**1**, 2x**2**) **c** 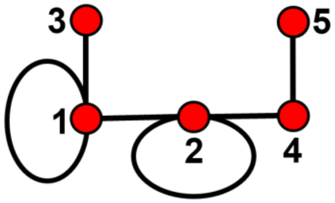
	(8x**1**, 2x$\mathbf{2}^1$) 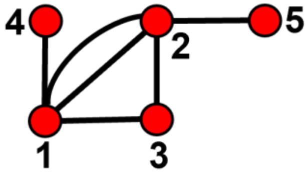	(8x**1**, 2x$\mathbf{2}^2$) 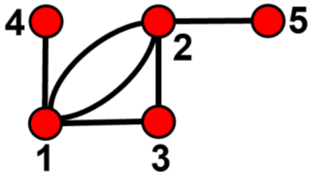	(6x**1**, 3x**2**) 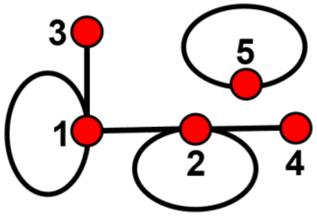	(6x**1**, 1x**2**, 2x$\mathbf{2}^1$) **a** 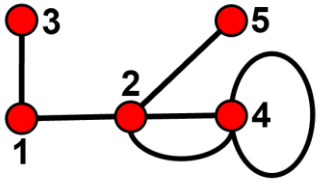

	(6x**1**, 1x**2**, 2x**2**^{1}) **b** 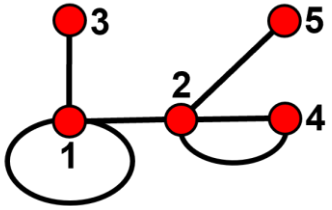	(6x**1**, 1x**2**, 2x**2**^{2}) **a** 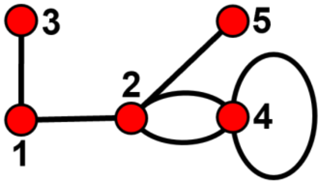	(6x**1**, 1x**2**, 2x**2**^{2}) **b** 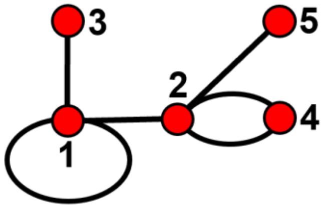	(4x**1**, 2x**2**, 2x**2**^{1}) **a** 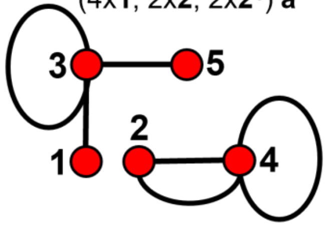
	(4x**1**, 2x**2**, 2x**2**^{1}) **b** 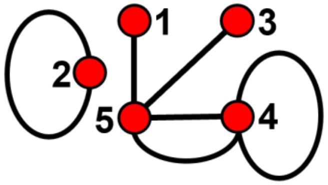	(4x**1**, 2x**2**, 2x**2**^{1}) **c** 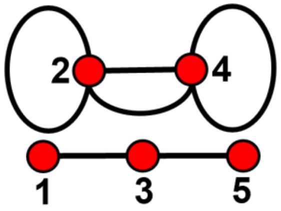	(4x**1**, 2x**2**, 2x**2**^{2}) **a** 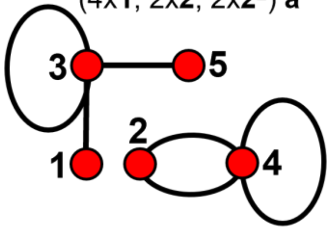	(4x**1**, 2x**2**, 2x**2**^{2}) **b** 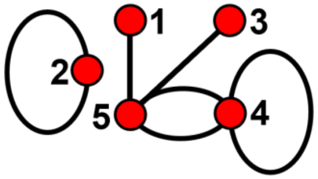
	(4x**1**, 2x**2**, 2x**2**^{2}) **c** 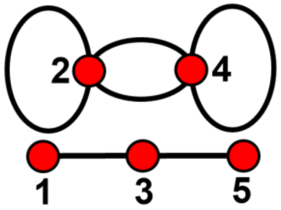	(4x**1**, 4x**2**^{1}) 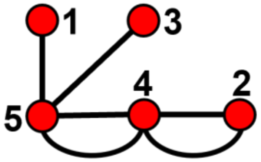	(4x**1**, 2x**2**^{1}, 2x**2**^{2}) **a** 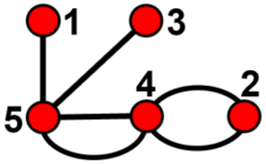	(4x**1**, 2x**2**^{1}, 2x**2**^{2}) **b** 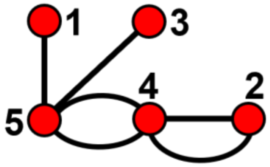
	(4x**1**, 4x**2**^{2}) 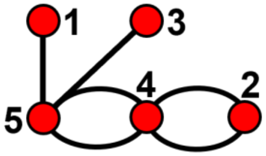	(2x**1**, 3x**2**, 2x**2**^{1}) 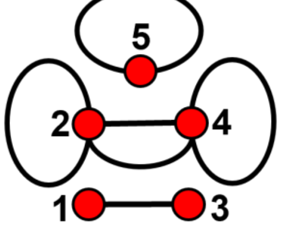	(2x**1**, 3x**2**, 2x**2**^{2}) 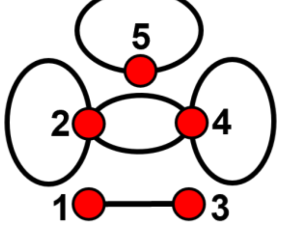	(2x**1**, 1x**2**, 4x**2**^{1}) 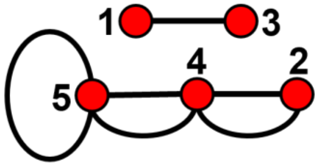

	(2x**1**, 1x**2**, 2x**2**¹, 2x**2**²) **a** 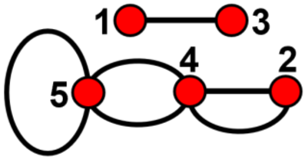	(2x**1**, 1x**2**, 2x**2**¹, 2x**2**²) **b** 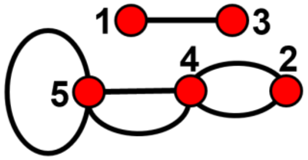	(2x**1**, 1x**2**, 4x**2**²) 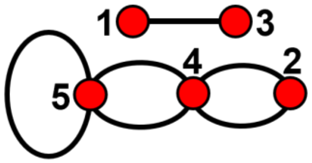	
${}^{1}V_{2}{}^{2}V_{1}{}^{4}V_{3}$	NG			
${}^{1}V_{2}{}^{2}V_{1}{}^{4}V_{4}$	NG			
${}^{1}V_{2}{}^{2}V_{1}{}^{4}V_{5}$	NG			
${}^{1}V_{2}{}^{2}V_{2}{}^{4}V_{1}$	(10x**1**) 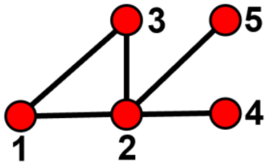	(8x**1**, 1x**2**) **a** 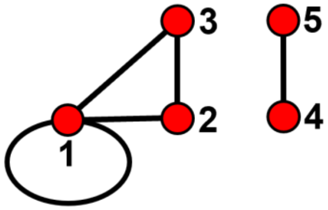	(8x**1**, 1x**2**) **b** 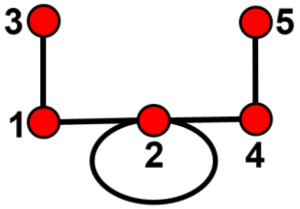	(8x**1**, 1x**2**) **c** 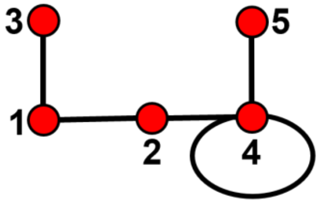
	(6x**1**, 2x**2**) 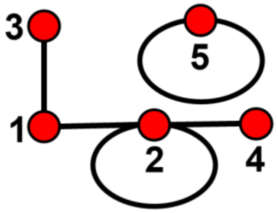	(6x**1**, 2x**2**¹) 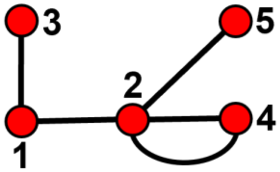	(6x**1**, 2x**2**²) 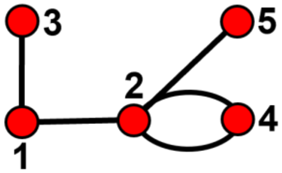	(4x**1**, 3x**2**) 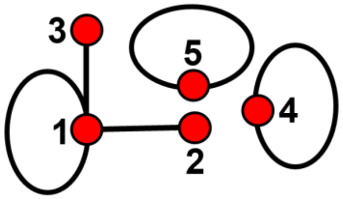
	(4x**1**, 1x**2**, 2x**2**¹) **a** 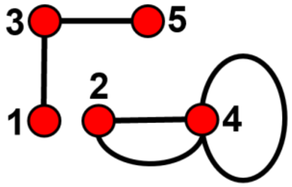	(4x**1**, 1x**2**, 2x**2**¹) **b** 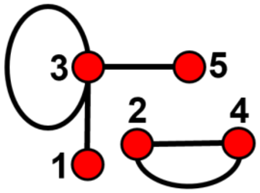	(4x**1**, 1x**2**, 2x**2**¹) **c** 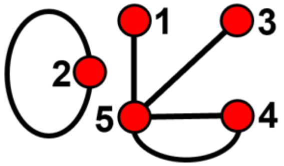	(4x**1**, 1x**2**, 2x**2**²) **a** 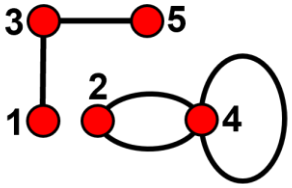

	(4x**1**, 1x**2**, 2x$\mathbf{2}^2$) **b** 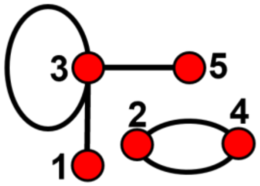	(4x**1**, 1x**2**, 2x$\mathbf{2}^2$) **c** 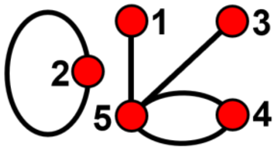	(2x**1**, 2x**2**, 2x$\mathbf{2}^1$) 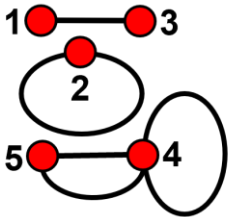	(2x**1**, 2x**2**, 2x$\mathbf{2}^2$) 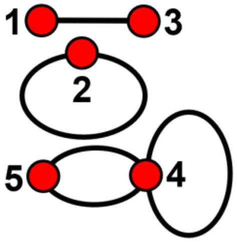
	(2x**1**, 4x$\mathbf{2}^1$) 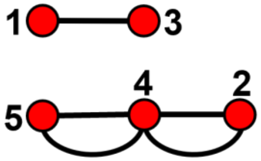	(2x**1**, 2x$\mathbf{2}^1$, 2x$\mathbf{2}^2$) 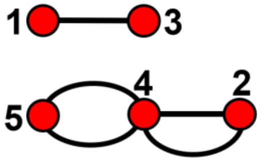	(2x**1**, 4x$\mathbf{2}^2$) 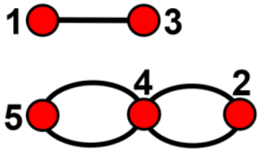	
$^1V_2{}^2V_2{}^4V_2$	NG			
$^1V_2{}^2V_2{}^4V_3$	NG			
$^1V_2{}^2V_2{}^4V_4$	NG			
$^1V_2{}^2V_3{}^4V_1$	NG			
$^1V_2{}^2V_3{}^4V_2$	NG			
$^1V_2{}^2V_3{}^4V_3$	NG			
$^1V_2{}^2V_4{}^4V_1$	NG			
$^1V_2{}^2V_4{}^4V_2$	NG			
$^1V_2{}^2V_5{}^4V_1$	NG			

$^1V_4{}^2V_1{}^4V_1$	(10x**1**) 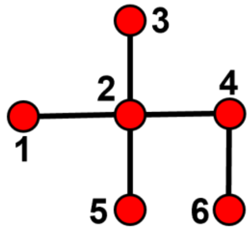	(8x**1**, 1x**2**) **a** 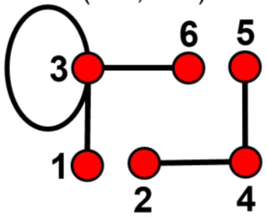	(8x**1**, 1x**2**) **b** 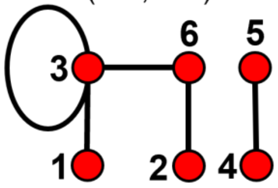	(8x**1**, 1x**2**) **c** 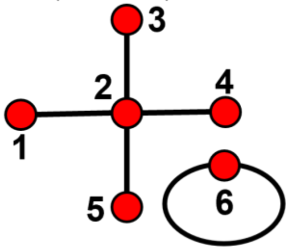
	(6x**1**, 2x**2**) 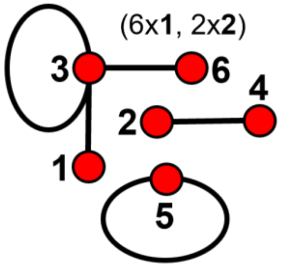	(6x**1**, 2x**2**^{1}) 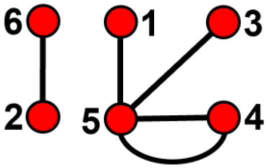	(6x**1**, 2x**2**^{2}) 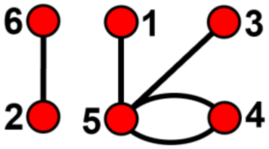	(4x**1**, 1x**2**, 2x**2**^{1}) 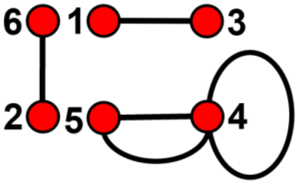
	(4x**1**, 1x**2**, 2x**2**^{2}) 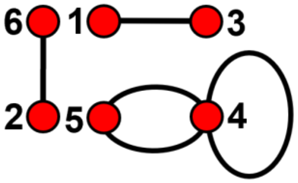			
$^1V_4{}^2V_1{}^4V_2$	NG			
$^1V_4{}^2V_1{}^4V_3$	NG			
$^1V_4{}^2V_2{}^4V_1$	NG			
$^1V_4{}^2V_2{}^4V_2$	NG			
$^1V_4{}^2V_3{}^4V_1$	NG			
$^1V_6{}^2V_1{}^4V_1$	NG			
$\mathbf{^1V_r{}^3V_r{}^4V_r}$				

$^{1}V_{1}{}^{3}V_{1}{}^{4}V_{1}$	(4x**1**, 2x**2**)<br>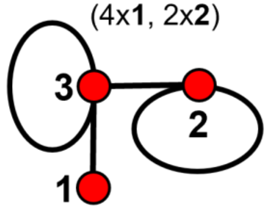	(2x**1**, 1x**2**, 2x**2**^{1})<br>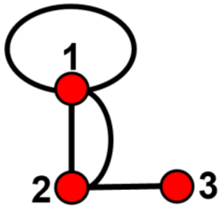	(2x**1**, 1x**2**, 2x**2**^{2})<br>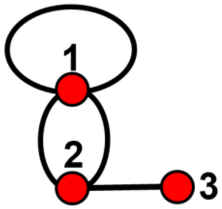	
$^{1}V_{1}{}^{3}V_{1}{}^{4}V_{2}$	(8x**1**, 2x**2**)<br>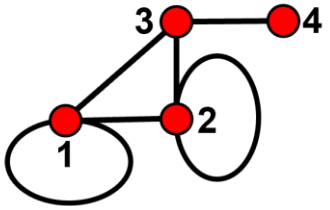	(6x**1**, 3x**2**)<br>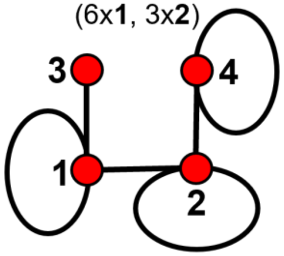	(6x**1**, 1x**2**, 2x**2**^{1})<br>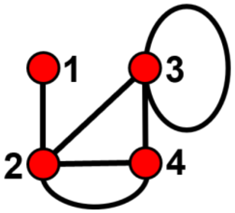	(6x**1**, 1x**2**, 2x**2**^{2})<br>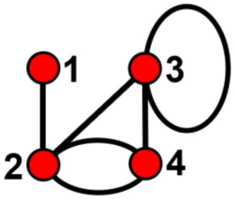
	(4x**1**, 2x**2**, 2x**2**^{1}) **a**<br>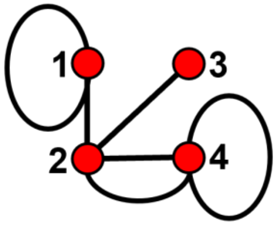	(4x**1**, 2x**2**, 2x**2**^{1}) **b**<br>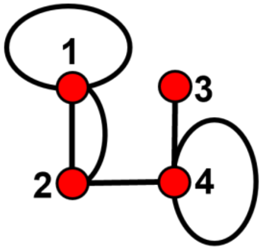	(4x**1**, 2x**2**, 2x**2**^{2}) **a**<br>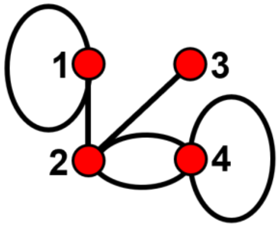	(4x**1**, 2x2, 2x**2**^{2}) **b**<br>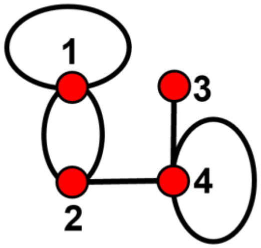
	(4x**1**, 4x**2**^{1})<br>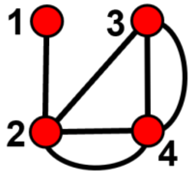	(4x**1**, 2x**2**^{1}, 2x**2**^{2})<br>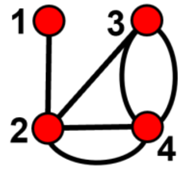	(4x**1**, 4x**2**^{2})<br>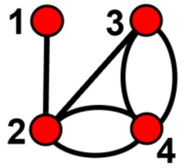	(2x**1**, 3x**2**, 2x**2**^{1})<br>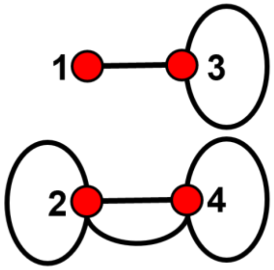

	(2x**1**, 3x**2**, 2x**2**^{2}) 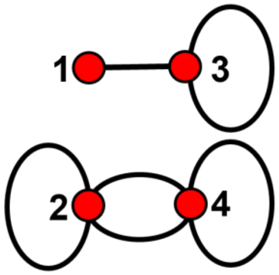	(2x**1**, 1x**2**, 4x**2**^{1}) 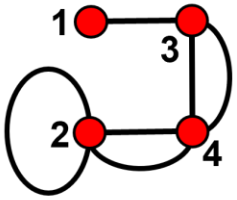	(2x**1**, 1x**2**, 2x**2**^{1}, 2x**2**^{2}) 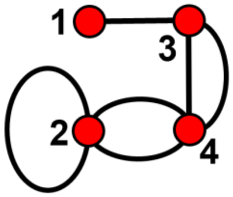	(2x**1**, 1x**2**, 4x**2**^{2}) 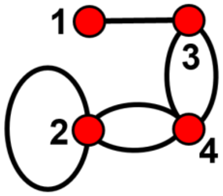
$^{1}V_{1}{}^{3}V_{1}{}^{4}V_{3}$	(12x**1**, 2x**2**) 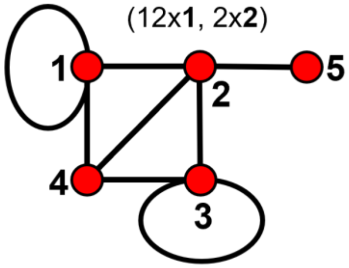	(12x**1**, 2x**2**^{1}) 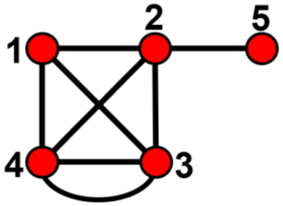	(12x**1**, 2x**2**^{2}) 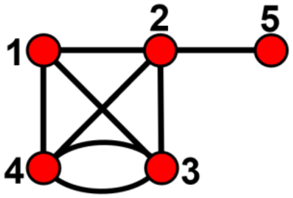	(10x**1**, 3x**2**) **a** 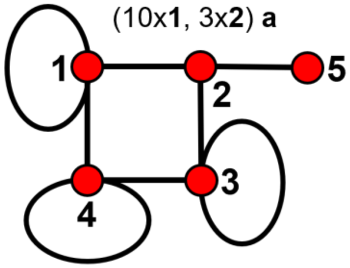
	(10x**1**, 3x**2**) **b** 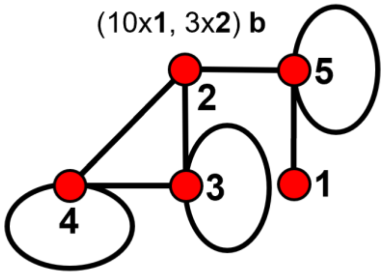	(10x**1**, 3x**2**) **c** 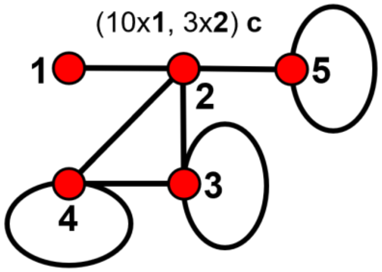	(10x**1**, 1x**2**, 2x**2**^{1}) **a** 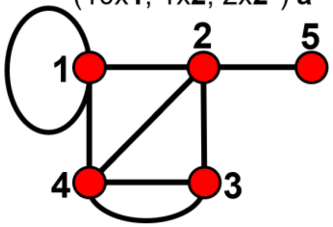	(10x**1**, 1x**2**, 2x**2**^{1}) **b** 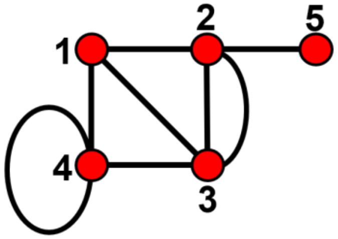
	(10x**1**, 1x**2**, 2x**2**^{1}) **c** 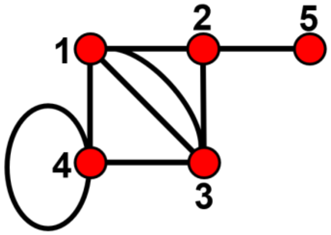	(10x**1**, 1x**2**, 2x**2**^{2}) **a** 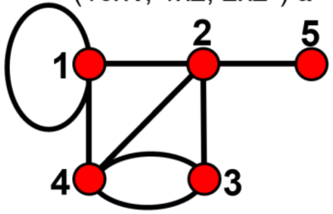	(10x**1**, 1x**2**, 2x**2**^{2}) **b** 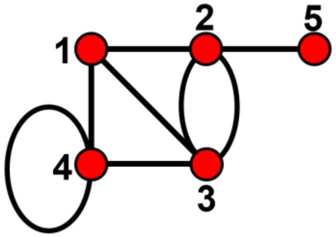	(10x**1**, 1x**2**, 2x**2**^{2}) **c** 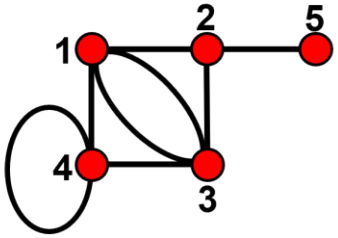

	(8x**1**, 4x**2**) **a** 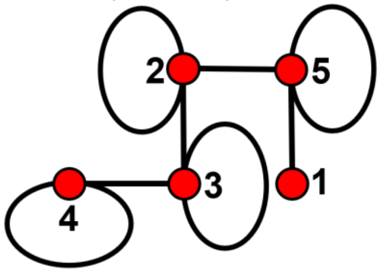	(8x**1**, 4x**2**) **b** 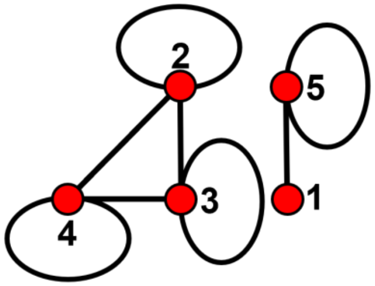	(8x**1**, 2x**2**, 2x**2**^{1}) **a** 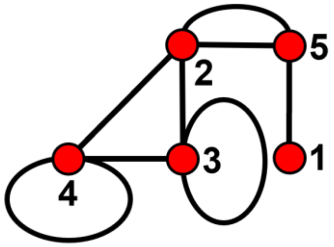	(8x**1**, 2x**2**, 2x**2**^{1}) **b** 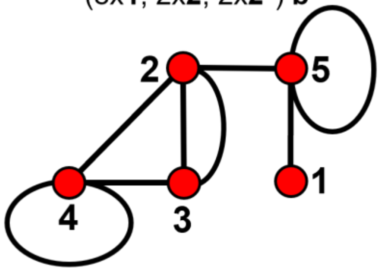
	(8x**1**, 2x**2**, 2x**2**^{1}) **c** 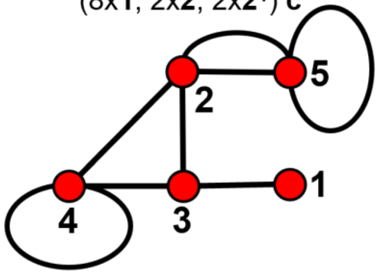	(8x**1**, 2x**2**, 2x**2**^{1}) **d** 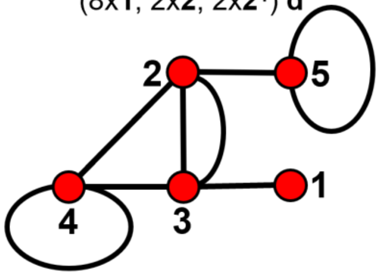	(8x**1**, 2x**2**, 2x**2**^{1}) **e** 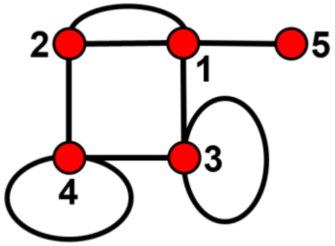	(8x**1**, 2x**2**, 2x**2**^{2}) **a** 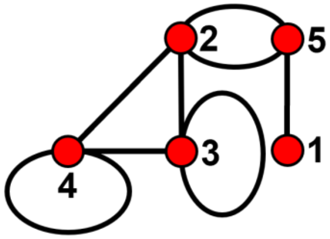
	(8x**1**, 2x**2**, 2x**2**^{2}) **b** 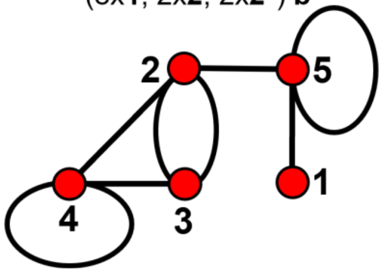	(8x**1**, 2x**2**, 2x**2**^{2}) **c** 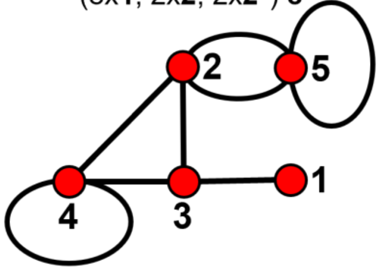	(8x**1**, 2x**2**, 2x**2**^{2}) **d** 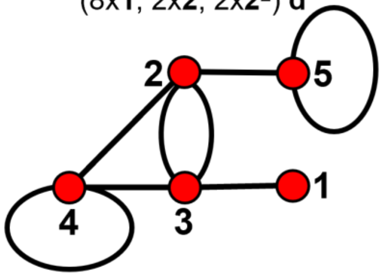	(8x**1**, 2x**2**, 2x**2**^{2}) **e** 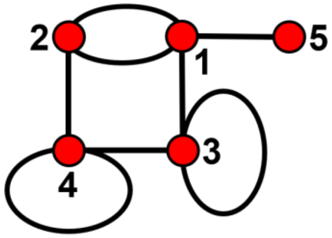

	(8x**1**, 4x**2**^{1}) **a** 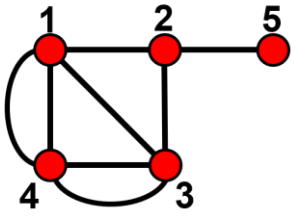	(8x**1**, 4x**2**^{1}) **b** 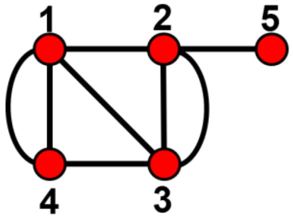	(8x**1**, 2x**2**^{1}, 2x**2**^{2}) **a** 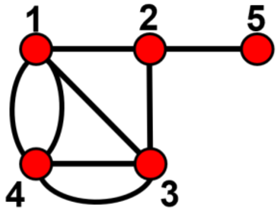	(8x**1**, 2x**2**^{1}, 2x**2**^{2}) **b** 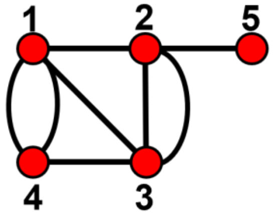
	(8x**1**, 2x**2**^{1}, 2x**2**^{2}) **c** 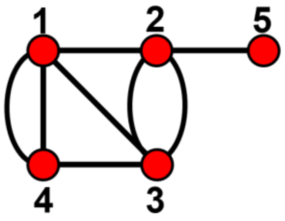	(8x**1**, 4x**2**^{2}) **a** 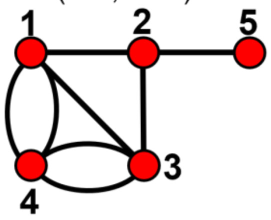	(8x**1**, 4x**2**^{2}) **b** 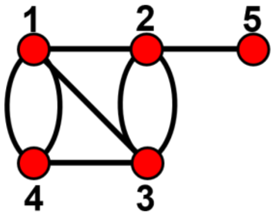	(6x**1**, 3x**2**, 2x**2**^{1}) **a** 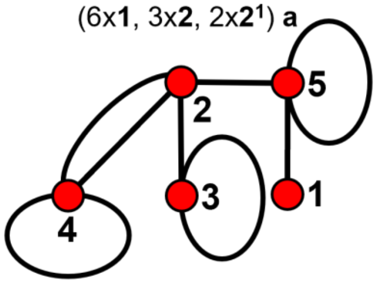
	(6x**1**, 3x**2**, 2x**2**^{1}) **b** 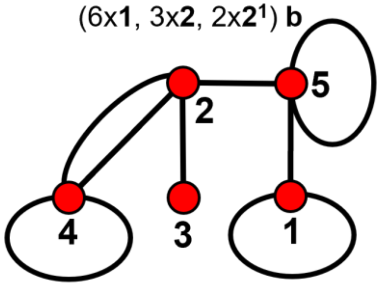	(6x**1**, 3x**2**, 2x**2**^{1}) **c** 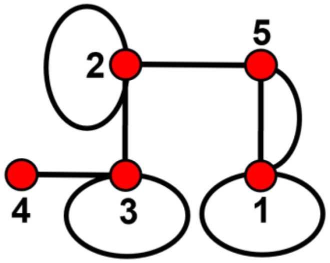	(6x**1**, 3x**2**, 2x**2**^{2}) **a** 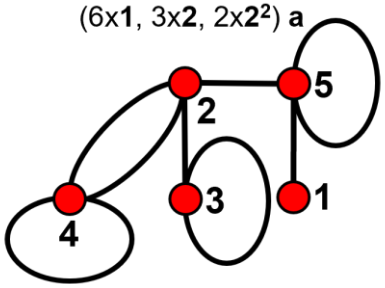	(6x**1**, 3x**2**, 2x**2**^{2}) **b** 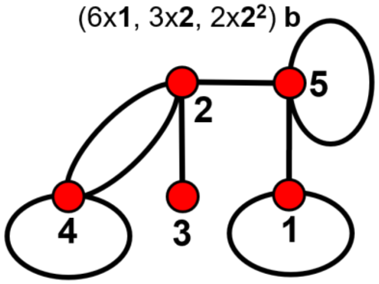

	(6x**1**, 3x**2**, 2x$\mathbf{2}^{2}$) **c**	(6x**1**, 1x**2**, 4x$\mathbf{2}^{1}$) **a**	(6x**1**, 1x**2**, 4x$\mathbf{2}^{1}$) **b**	(6x**1**, 1x**2**, 4x$\mathbf{2}^{1}$) **c**
	(6x**1**, 1x**2**, 4x$\mathbf{2}^{1}$) **d**	(6x**1**, 1x**2**, 2x$\mathbf{2}^{1}$, 2x$\mathbf{2}^{2}$) **a**	(6x**1**, 1x**2**, 2x$\mathbf{2}^{1}$, 2x$\mathbf{2}^{2}$) **b**	(6x**1**, 1x**2**, 2x$\mathbf{2}^{1}$, 2x$\mathbf{2}^{2}$) **c**
	(6x**1**, 1x**2**, 2x$\mathbf{2}^{1}$, 2x$\mathbf{2}^{2}$) **d**	(6x**1**, 1x**2**, 2x$\mathbf{2}^{1}$, 2x$\mathbf{2}^{2}$) **e**	(6x**1**, 1x**2**, 2x$\mathbf{2}^{1}$, 2x$\mathbf{2}^{2}$) **f**	(6x**1**, 1x**2**, 2x$\mathbf{2}^{1}$, 2x$\mathbf{2}^{2}$) **g**
	(6x**1**, 1x**2**, 2x$\mathbf{2}^{1}$, 2x$\mathbf{2}^{2}$) **h**	(6x**1**, 1x**2**, 4x$\mathbf{2}^{2}$) **a**	(6x**1**, 1x**2**, 4x$\mathbf{2}^{2}$) **b**	(6x**1**, 1x**2**, 4x$\mathbf{2}^{2}$) **c**

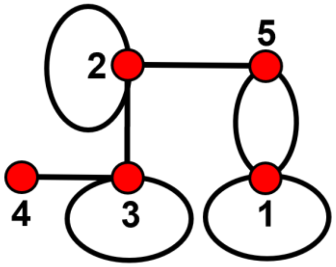

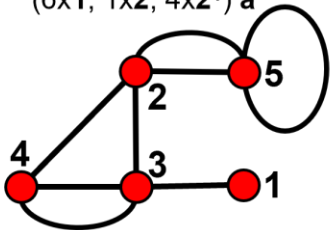

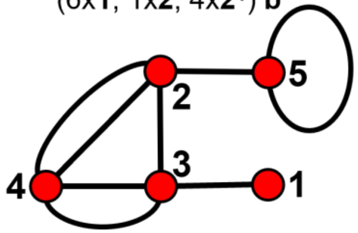

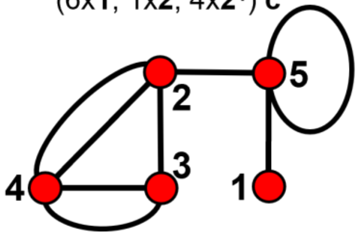

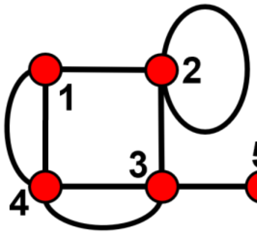

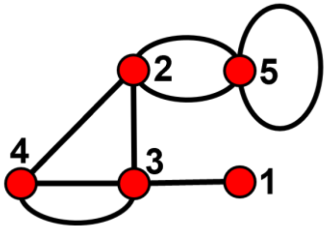

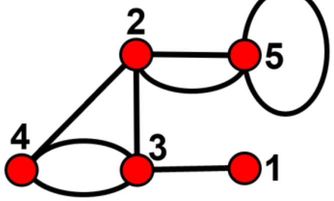

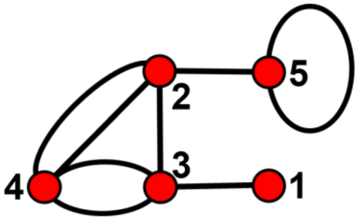

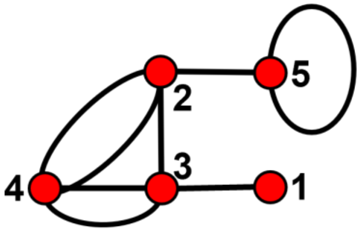

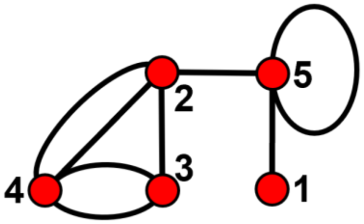

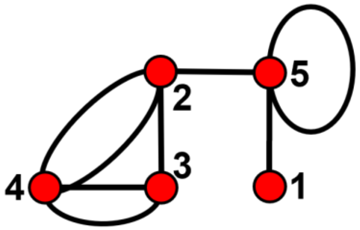

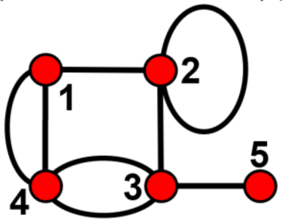

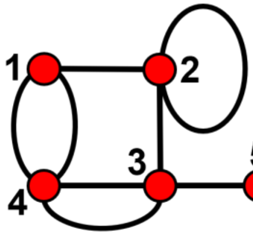

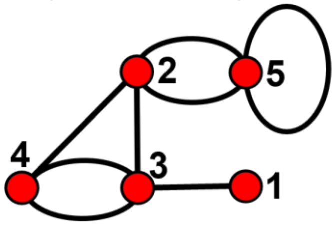

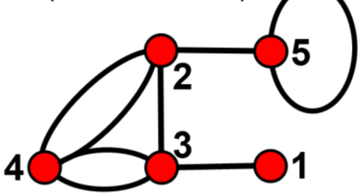

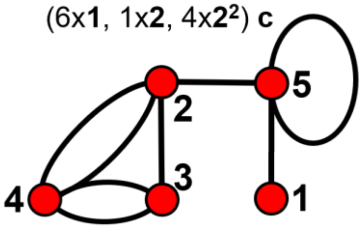

	(6x**1**, 1x**2**, 4x**2**^{2}) **d** 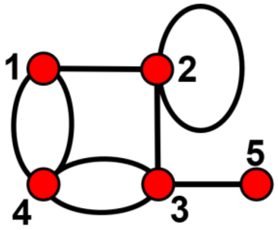	(4x**1**, 4x**2**, 2x**2**^{1}) 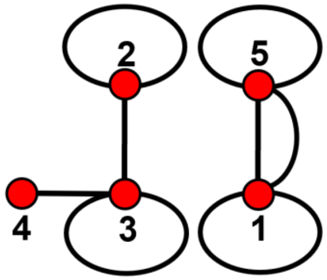	(4x**1**, 4x**2**, 2x**2**^{2}) 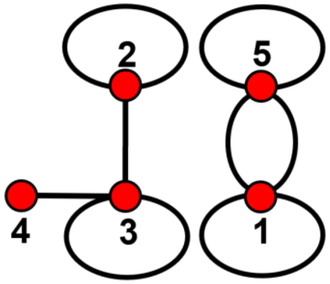	(4x**1**, 2x**2**, 4x**2**^{1}) **a** 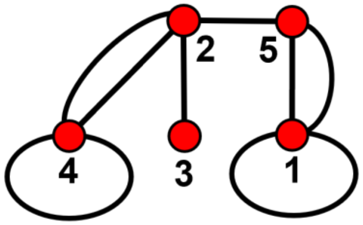
	(4x**1**, 2x**2**, 4x**2**^{1}) **b** 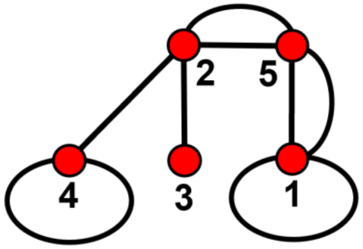	(4x**1**, 2x**2**, 4x**2**^{1}) **c** 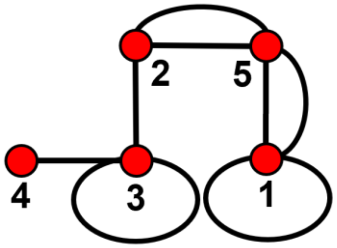	(4x**1**, 2x**2**, 2x**2**^{1}, 2x**2**^{2}) **a** 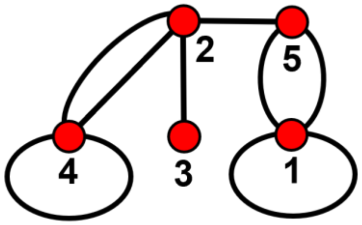	(4x**1**, 2x**2**, 2x**2**^{1}, 2x**2**^{2}) **b** 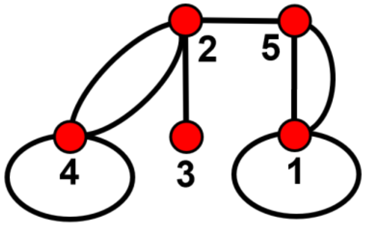
	(4x**1**, 2x**2**, 2x**2**^{1}, 2x**2**^{2}) **c** 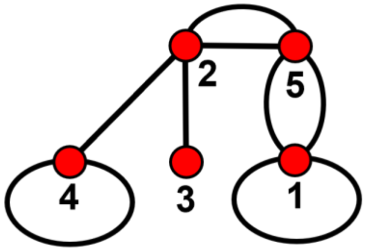	(4x**1**, 2x**2**, 2x**2**^{1}, 2x**2**^{2}) **d** 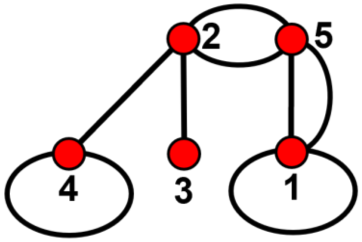	(4x**1**, 2x**2**, 2x**2**^{1}, 2x**2**^{2}) **e** 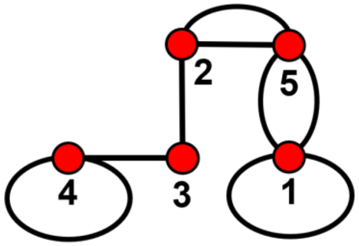	(4x**1**, 2x**2**, 2x**2**^{1}, 2x**2**^{2}) **f** 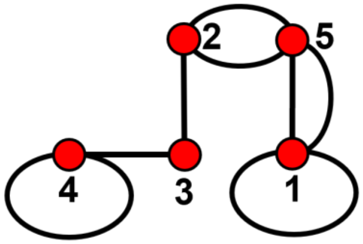

	(4x**1**, 2x**2**, 4x**2**^{2}) **a**<br>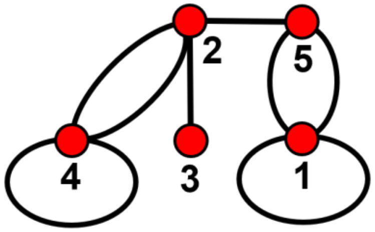 	(4x**1**, 2x**2**, 4x**2**^{2}) **b**<br>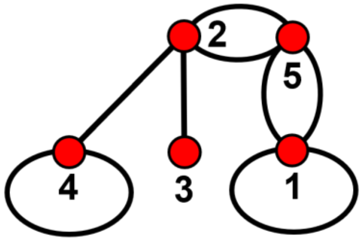 	(4x**1**, 2x**2**, 4x**2**^{2}) **c**<br>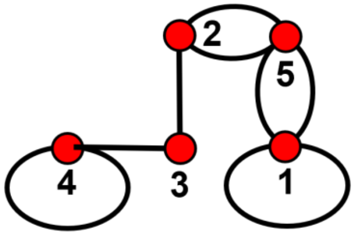 	(4x**1**, 6x**2**^{1})<br>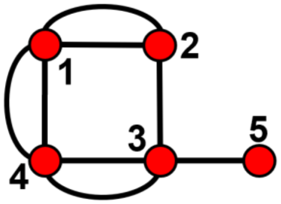
	(4x**1**, 4x**2**^{1}, 2x**2**^{2}) **a**<br>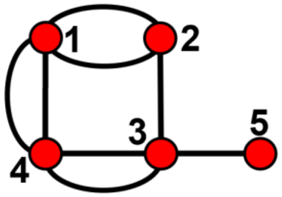 	(4x**1**, 4x**2**^{1}, 2x**2**^{2}) **b**<br>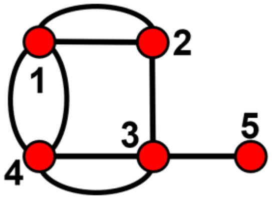 	(4x**1**, 4x**2**^{1}, 2x**2**^{2}) **c**<br>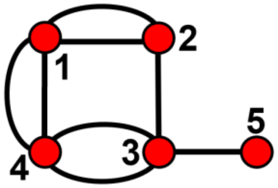 	(4x**1**, 2x**2**^{1}, 4x**2**^{2}) **a**<br>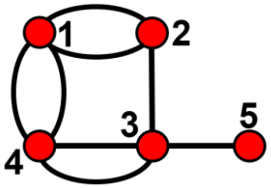
	(4x**1**, 2x**2**^{1}, 4x**2**^{2}) **b**<br>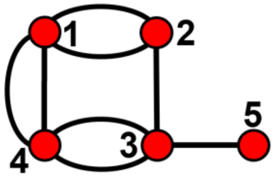 	(4x**1**, 2x**2**^{1}, 4x**2**^{2}) **c**<br>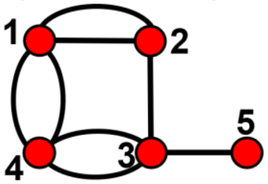 	(4x**1**, 6x**2**^{2})<br>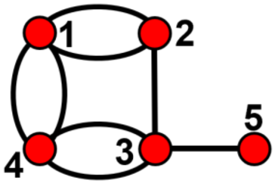 	(2x**1**, 3x**2**, 4x**2**^{1}) **a**<br>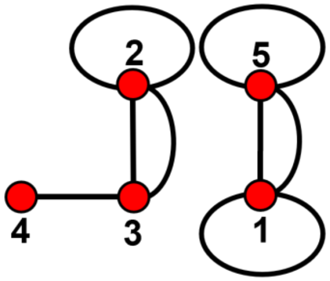

	(2x**1**, 3x**2**, 4x$\mathbf{2}^1$) **b** 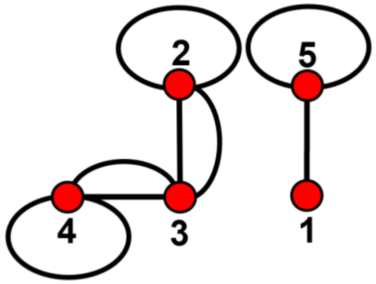	(2x**1**, 3x**2**, 2x$\mathbf{2}^1$, 2x$\mathbf{2}^2$) **a** 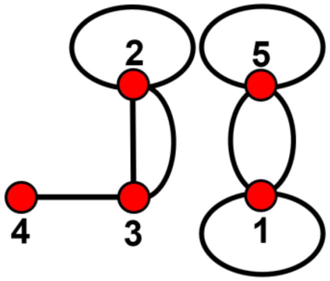	(2x**1**, 3x**2**, 2x$\mathbf{2}^1$, 2x$\mathbf{2}^2$) **b** 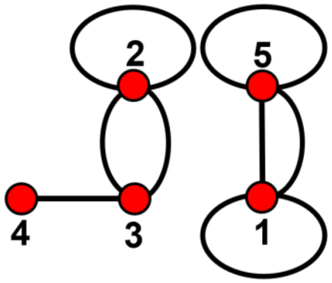	(2x**1**, 3x**2**, 2x$\mathbf{2}^1$, 2x$\mathbf{2}^2$) **c** 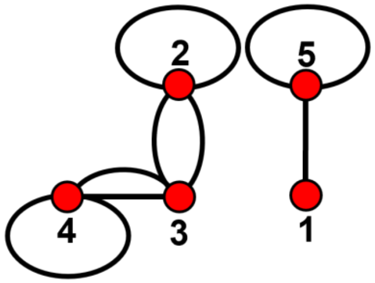
	(2x**1**, 3x**2**, 4x$\mathbf{2}^2$) **a** 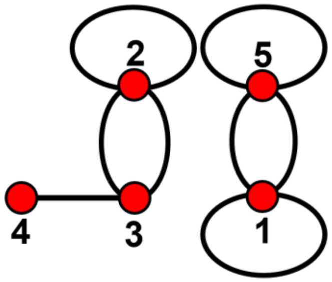	(2x**1**, 3x**2**, 4x$\mathbf{2}^2$) **b** 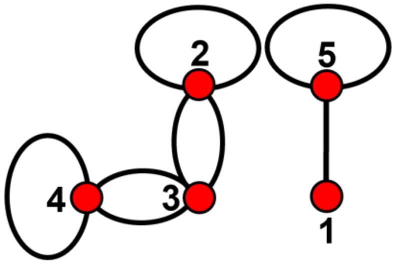	(2x**1**, 1x**2**, 6x$\mathbf{2}^1$) **a** 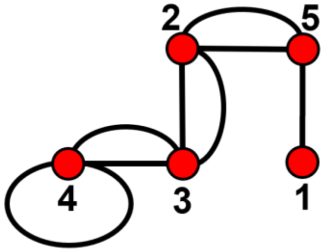	(2x**1**, 1x**2**, 6x$\mathbf{2}^1$) **b** 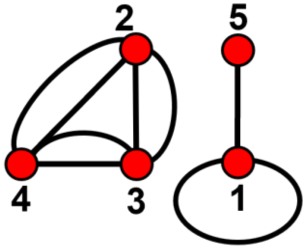
	(2x**1**, 1x**2**, 4x$\mathbf{2}^1$, 2x$\mathbf{2}^2$) **a** 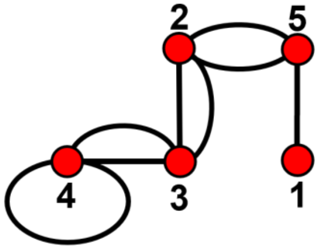	(2x**1**, 1x**2**, 4x$\mathbf{2}^1$, 2x$\mathbf{2}^2$) **b** 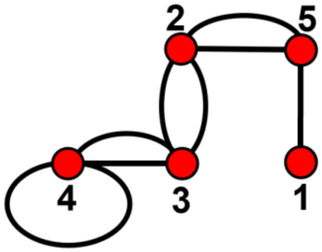	(2x**1**, 1x**2**, 4x$\mathbf{2}^1$, 2x$\mathbf{2}^2$) **c** 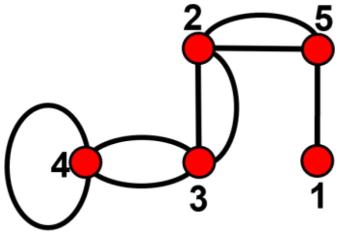	(2x**1**, 1x**2**, 4x$\mathbf{2}^1$, 2x$\mathbf{2}^2$) **d** 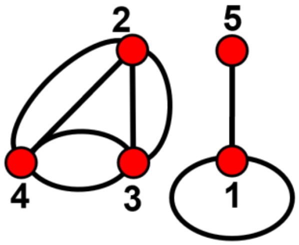

	(2x**1**, 1x**2**, 2x$\mathbf{2}^1$, 4x$\mathbf{2}^2$) **a** 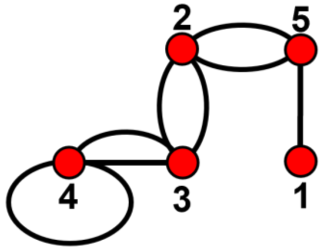	(2x**1**, 1x**2**, 2x$\mathbf{2}^1$, 4x$\mathbf{2}^2$) **b** 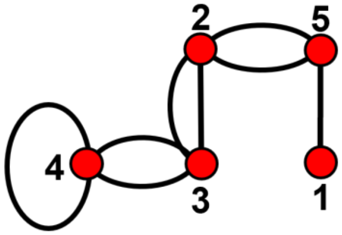	(2x**1**, 1x**2**, 2x$\mathbf{2}^1$, 4x$\mathbf{2}^2$) **c** 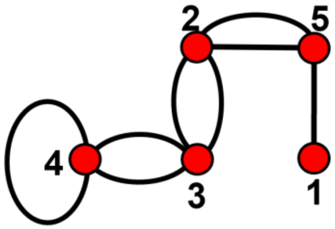	(2x**1**, 1x**2**, 2x$\mathbf{2}^1$, 4x$\mathbf{2}^2$) **d** 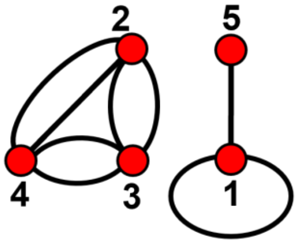
	(2x**1**, 1x**2**, 6x$\mathbf{2}^2$) **a** 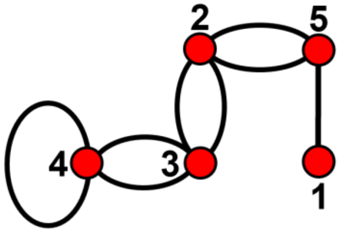	(2x**1**, 1x**2**, 6x$\mathbf{2}^2$) **b** 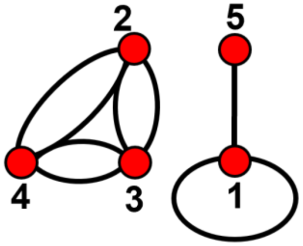		
$^1V_1{}^3V_1{}^4V_4$	NG			
$^1V_1{}^3V_1{}^4V_5$	NG			
$^1V_1{}^3V_1{}^4V_6$	NG			
$^1V_1{}^3V_3{}^4V_1$	(14x**1**) 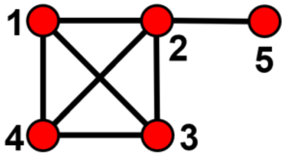	(12x**1**, 1x**2**) 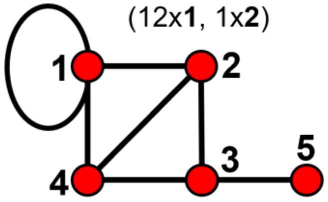	(10x**1**, 2x**2**) 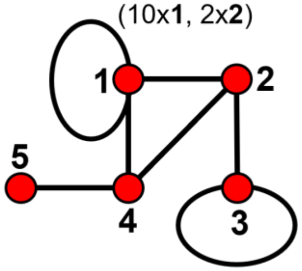	(10x**1**, 2x$\mathbf{2}^1$) 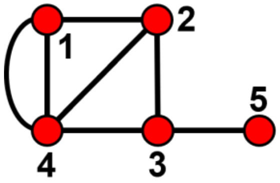

	(10x**1**, 2x$\mathbf{2}^2$) 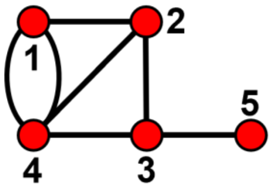	(8x**1**, 3x**2**) **a** 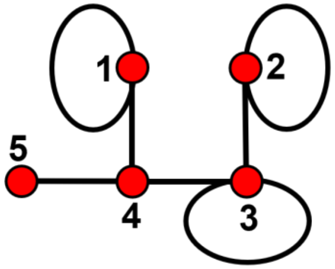	(8x**1**, 3x**2**) **b** 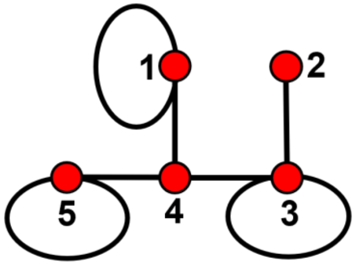	(8x**1**, 3x**2**) **c** 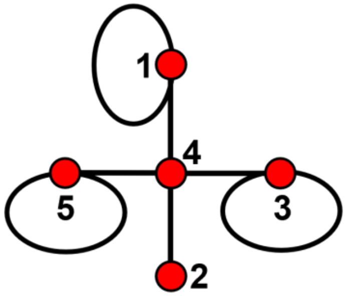
	(8x**1**, 1x**2**, 2x$\mathbf{2}^1$) **a** 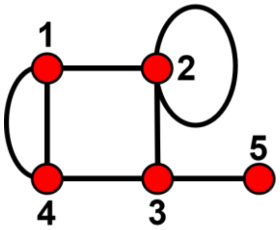	(8x**1**, 1x**2**, 2x$\mathbf{2}^1$) **b** 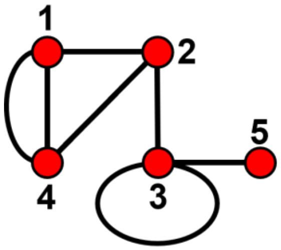	(8x**1**, 1x**2**, 2x$\mathbf{2}^1$) **c** 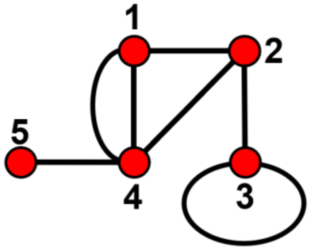	(8x**1**, 1x**2**, 2x$\mathbf{2}^1$) **d** 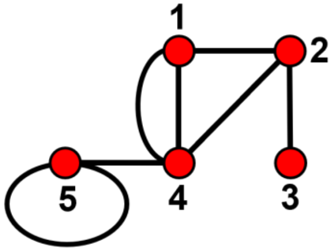
	(8x**1**, 1x**2**, 2x$\mathbf{2}^1$) **e** 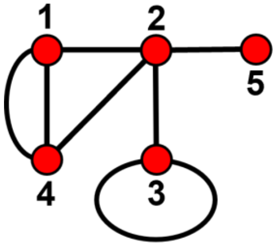	(8x**1**, 1x**2**, 2x$\mathbf{2}^2$) **a** 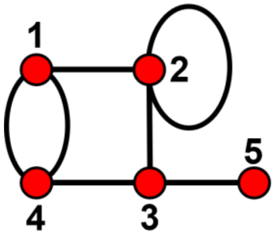	(8x**1**, 1x**2**, 2x$\mathbf{2}^2$) **b** 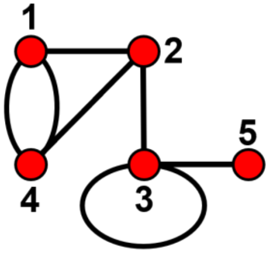	(8x**1**, 1x**2**, 2x$\mathbf{2}^2$) **c** 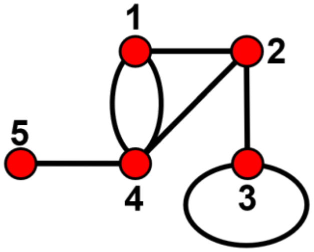

	(8x**1**, 1x**2**, 2x**2**^{2}) **d** 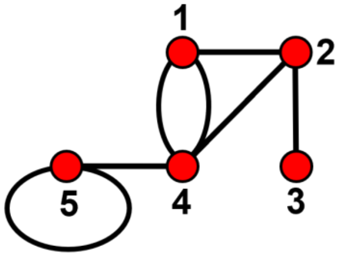	(8x**1**, 1x**2**, 2x**2**^{2}) **e** 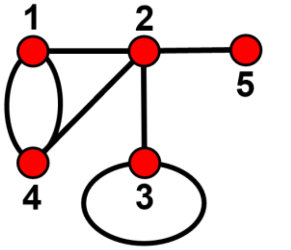	(6x**1**, 4x**2**) **a** 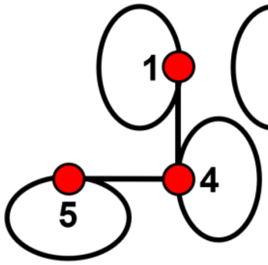	(6x**1**, 4x**2**) **b** 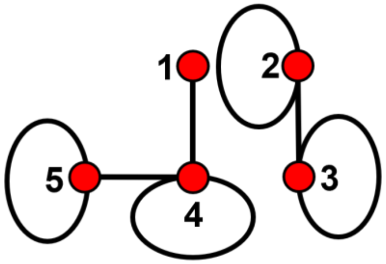
	(6x**1**, 2x**2**, 2x**2**^{1}) **a** 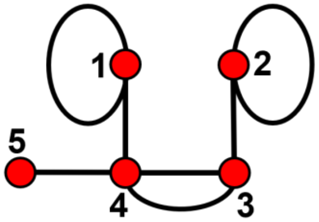	(6x**1**, 2x**2**, 2x**2**^{1}) **b** 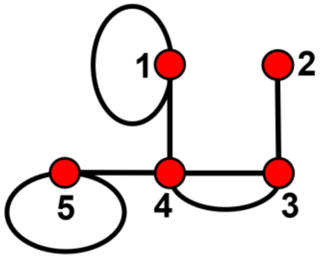	(6x**1**, 2x**2**, 2x**2**^{1}) **c** 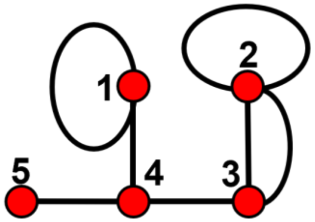	(6x**1**, 2x**2**, 2x**2**^{1}) **d** 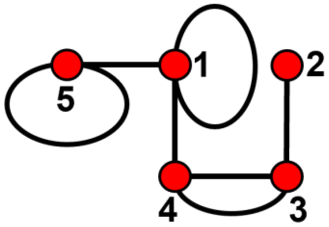
	(6x**1**, 2x**2**, 2x**2**^{1}) **e** 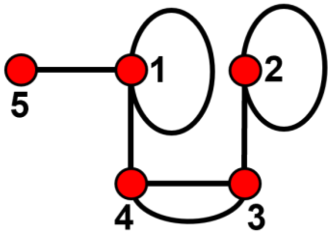	(6x**1**, 2x**2**, 2x**2**^{1}) **f** 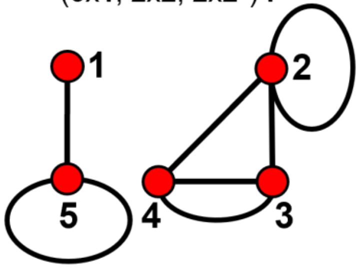	(6x**1**, 2x**2**, 2x**2**^{2}) **a** 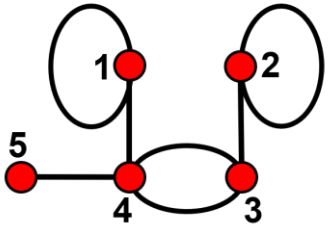	(6x**1**, 2x**2**, 2x**2**^{2}) **b** 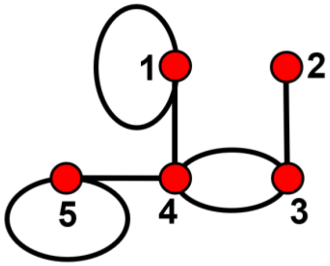

	(6x**1**, 2x**2**, 2x**2**^{2}) **c** 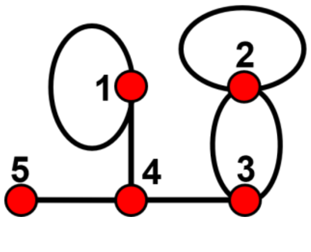	(6x**1**, 2x**2**, 2x**2**^{2}) **d** 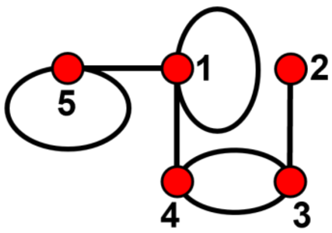	(6x**1**, 2x**2**, 2x**2**^{2}) **e** 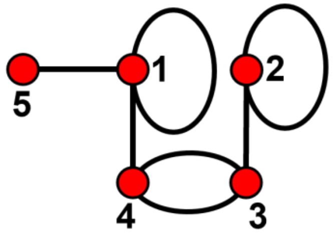	(6x**1**, 2x**2**, 2x**2**^{2}) **f** 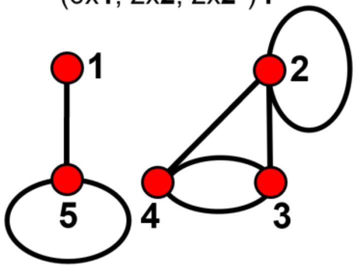
	(6x**1**, 4x**2**^{1}) **a** 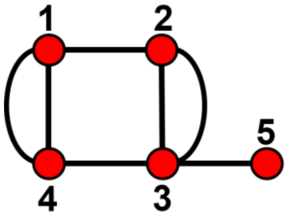	(6x**1**, 4x**2**^{1}) **b** 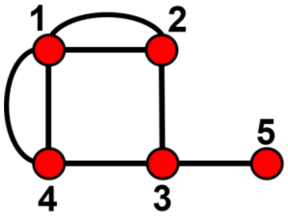	(6x**1**, 4x**2**^{1}) **c** 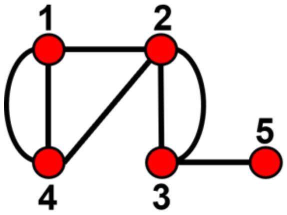	(6x**1**, 2x**2**^{1}, 2x**2**^{2}) **a** 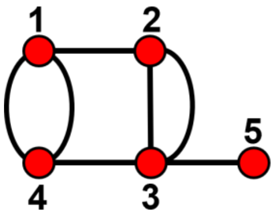
	(6x**1**, 2x**2**^{1}, 2x**2**^{2}) **b** 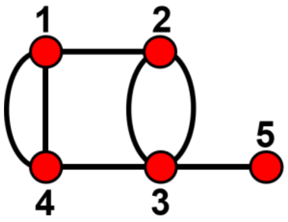	(6x**1**, 2x**2**^{1}, 2x**2**^{2}) **c** 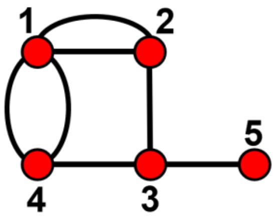	(6x**1**, 2x**2**^{1}, 2x**2**^{2}) **d** 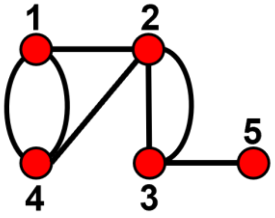	(6x**1**, 2x**2**^{1}, 2x**2**^{2}) **e** 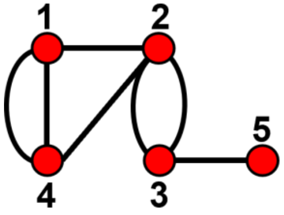
	(6x**1**, 4x**2**^{2}) **a** 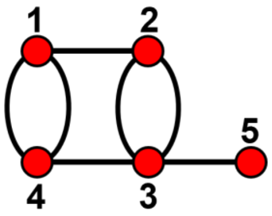	(6x**1**, 4x**2**^{2}) **b** 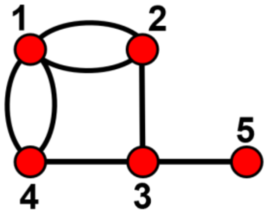	(6x**1**, 4x**2**^{2}) **c** 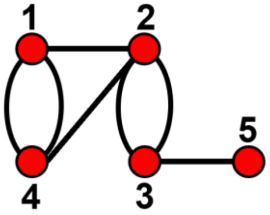	(4x**1**, 3x**2**, 2x**2**^{1}) **a** 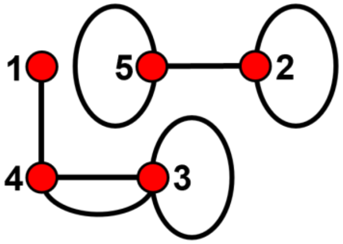

<table>
<tr>
<td></td>
<td>

(4x**1**, 3x**2**, 2x$\mathbf{2}^{1}$) **b**

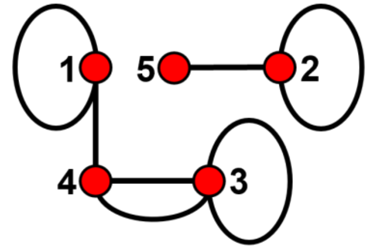

</td>
<td>

(4x**1**, 3x**2**, 2x$\mathbf{2}^{2}$) **a**

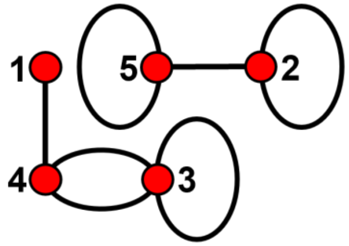

</td>
<td>

(4x**1**, 3x**2**, 2x$\mathbf{2}^{2}$) **b**

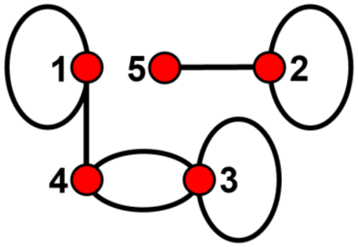

</td>
<td>

(4x**1**, 1x**2**, 4x$\mathbf{2}^{1}$) **a**

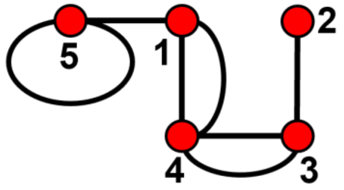

</td>
</tr>
<tr>
<td></td>
<td>

(4x**1**, 1x**2**, 4x$\mathbf{2}^{1}$) **b**

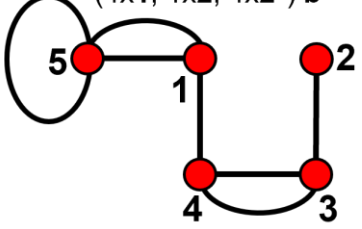

</td>
<td>

(4x**1**, 1x**2**, 4x$\mathbf{2}^{1}$) **c**

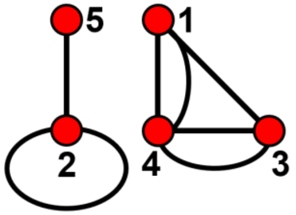

</td>
<td>

(4x**1**, 1x**2**, 2x$\mathbf{2}^{1}$, 2x$\mathbf{2}^{2}$) **a**

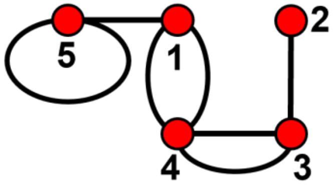

</td>
<td>

(4x**1**, 1x**2**, 2x$\mathbf{2}^{1}$, 2x$\mathbf{2}^{2}$) **b**

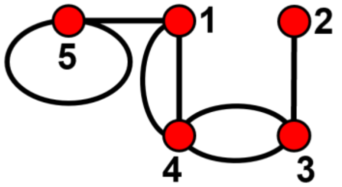

</td>
</tr>
<tr>
<td></td>
<td>

(4x**1**, 1x**2**, 2x$\mathbf{2}^{1}$, 2x$\mathbf{2}^{2}$) **c**

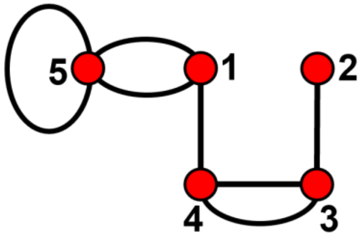

</td>
<td>

(4x**1**, 1x**2**, 2x$\mathbf{2}^{1}$, 2x$\mathbf{2}^{2}$) **d**

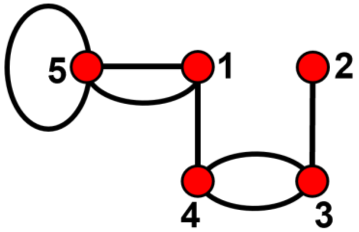

</td>
<td>

(4x**1**, 1x**2**, 2x$\mathbf{2}^{1}$, 2x$\mathbf{2}^{2}$) **e**

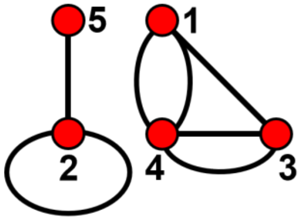

</td>
<td>

(4x**1**, 1x**2**, 4x$\mathbf{2}^{2}$) **a**

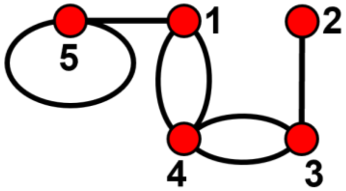

</td>
</tr>
<tr>
<td></td>
<td>

(4x**1**, 1x**2**, 4x$\mathbf{2}^{2}$) **b**

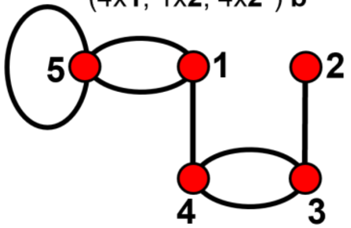

</td>
<td>

(4x**1**, 1x**2**, 4x$\mathbf{2}^{2}$) **c**

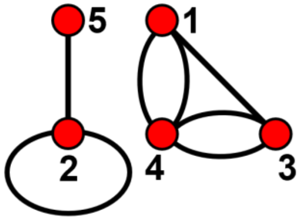

</td>
<td></td>
<td></td>
</tr>
</table>

$^{1}V_{1}{}^{3}V_{3}{}^{4}V_{2}$	NG			
$^{1}V_{1}{}^{3}V_{3}{}^{4}V_{3}$	NG			
$^{1}V_{1}{}^{3}V_{3}{}^{4}V_{4}$	NG			
$^{1}V_{1}{}^{3}V_{5}{}^{4}V_{1}$	NG			
$^{1}V_{1}{}^{3}V_{5}{}^{4}V_{2}$	NG			
$^{1}V_{2}{}^{3}V_{2}{}^{4}V_{1}$	(10x**1**, 1x**2**) 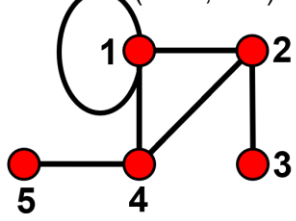	(8x**1**, 2x**2**) **a** 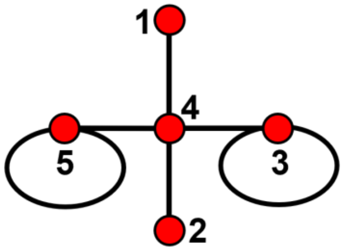	(8x**1**, 2x**2**) **b** 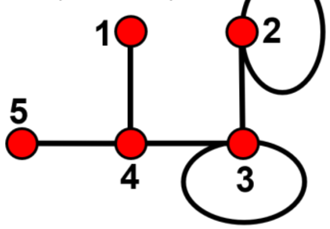	(8x**1**, 2x**2**) **c** 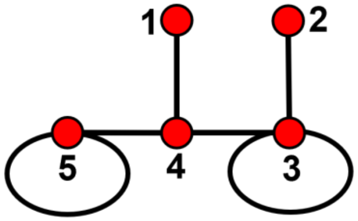
	(8x**1**, 2x**2**^{1}) **a** 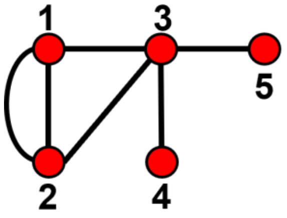	(8x**1**, 2x**2**^{1}) **b** 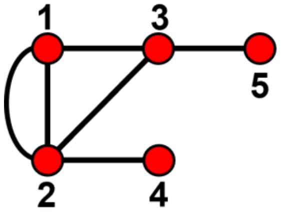	(8x**1**, 2x**2**^{2}) **a** 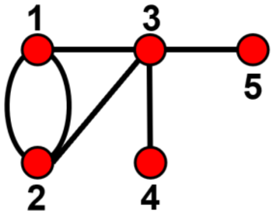	(8x**1**, 2x**2**^{2}) **b** 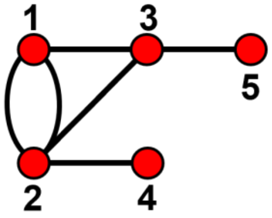
	(6x**1**, 3x**2**) **a** 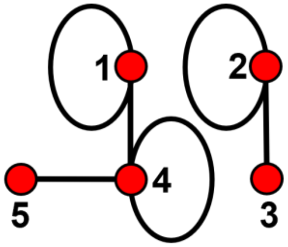	(6x**1**, 3x**2**) **b** 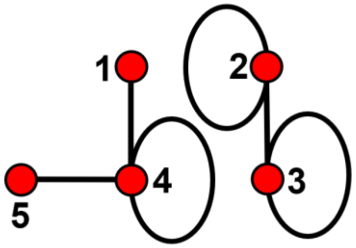	(6x**1**, 3x**2**) **c** 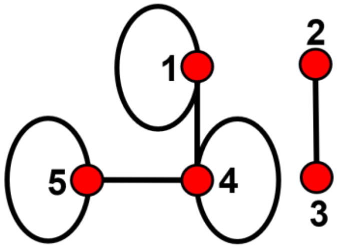	(6x**1**, 1x**2**, 2x**2**^{1}) **a** 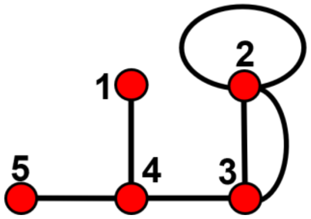

(6x**1**, 1x**2**, 2x$\mathbf{2}^{1}$) **b**

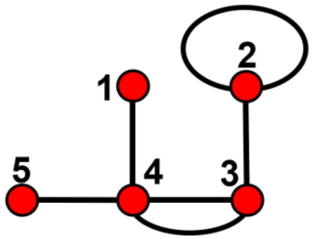

(6x**1**, 1x**2**, 2x$\mathbf{2}^{1}$) **c**

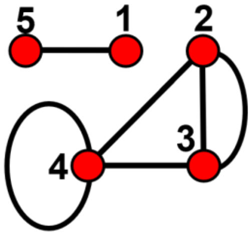

(6x**1**, 1x**2**, 2x$\mathbf{2}^{1}$) **d**

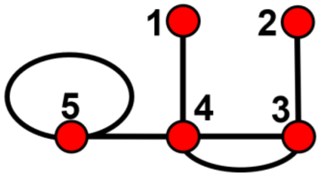

(6x**1**, 1x**2**, 2x$\mathbf{2}^{1}$) **e**

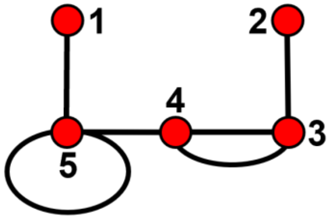

(6x**1**, 1x**2**, 2x$\mathbf{2}^{2}$) **a**

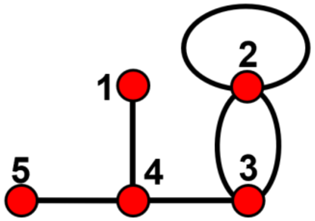

(6x**1**, 1x**2**, 2x$\mathbf{2}^{2}$) **b**

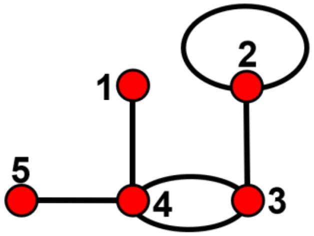

(6x**1**, 1x**2**, 2x$\mathbf{2}^{2}$) **c**

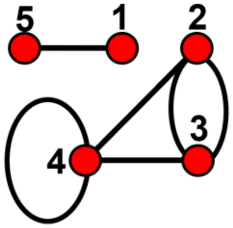

(6x**1**, 1x**2**, 2x$\mathbf{2}^{2}$) **d**

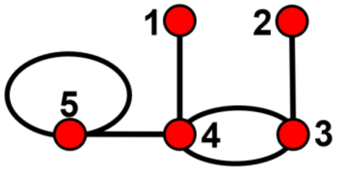

(6x**1**, 1x**2**, 2x$\mathbf{2}^{2}$) **e**

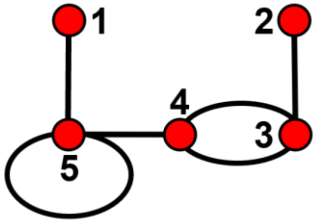

(4x**1**, 2x**2**, 2x$\mathbf{2}^{1}$) **a**

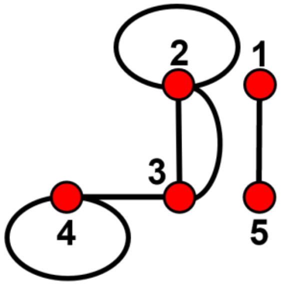

(4x**1**, 2x**2**, 2x$\mathbf{2}^{1}$) **b**

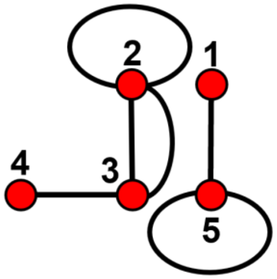

(4x**1**, 2x**2**, 2x$\mathbf{2}^{2}$) **a**

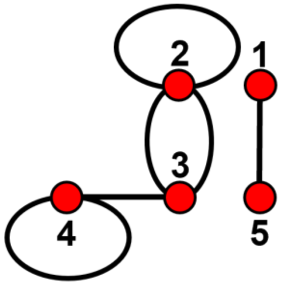

	(4x**1**, 2x**2**, 2x$\mathbf{2}^{2}$) **b**	(4x**1**, 4x$\mathbf{2}^{1}$) **a**	(4x**1**, 4x$\mathbf{2}^{1}$) **b**	(4x**1**, 2x$\mathbf{2}^{1}$, 2x$\mathbf{2}^{2}$) **a**
	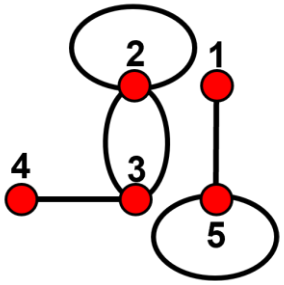	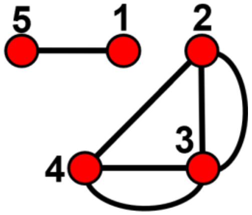	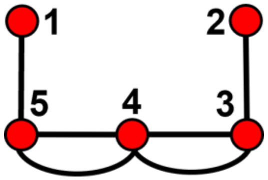	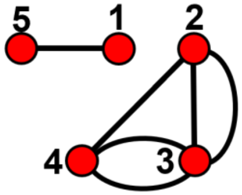
	(4x**1**, 2x$\mathbf{2}^{1}$, 2x$\mathbf{2}^{2}$) **b**	(4x**1**, 4x$\mathbf{2}^{2}$) **a**	(4x**1**, 4x$\mathbf{2}^{2}$) **b**	
	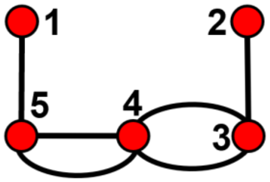	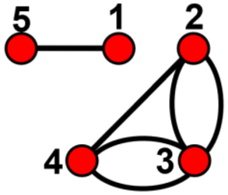	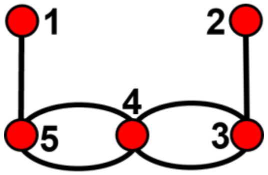	
$^{1}V_{2}{}^{3}V_{2}{}^{4}V_{2}$	NG			
$^{1}V_{2}{}^{3}V_{2}{}^{4}V_{3}$	NG			
$^{1}V_{2}{}^{3}V_{2}{}^{4}V_{4}$	NG			
$^{1}V_{2}{}^{3}V_{4}{}^{4}V_{1}$	NG			
$^{1}V_{2}{}^{3}V_{4}{}^{4}V_{2}$	NG			
$^{1}V_{3}{}^{3}V_{1}{}^{4}V_{1}$	(8x**1**, 1x**2**) **a**<br>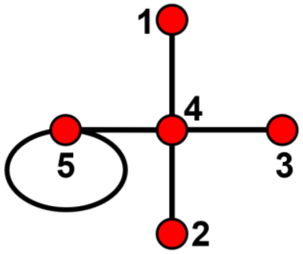	(8x**1**, 1x**2**) **b**<br>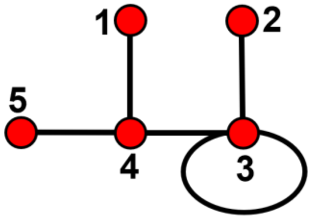	(6x**1**, 2x**2**) **a**<br>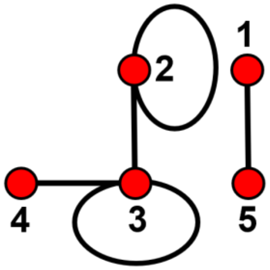	(6x**1**, 2x**2**) **b**<br>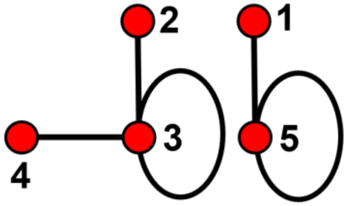

	(6x**1**, 2x$\mathbf{2}^1$)<br>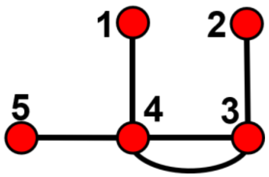	(6x**1**, 2x$\mathbf{2}^2$)<br>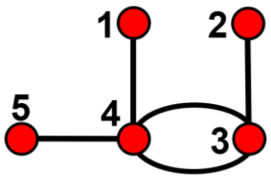	(4x**1**, 1x**2**, 2x$\mathbf{2}^1$)<br>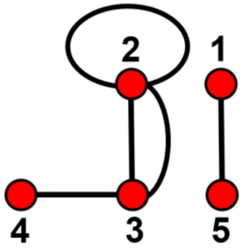	(4x**1**, 1x**2**, 2x$\mathbf{2}^2$)<br>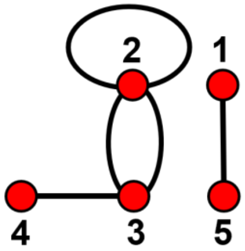
$^1V_3{}^3V_1{}^4V_2$	NG			
$^1V_3{}^3V_1{}^4V_3$	NG			
$^1V_3{}^3V_1{}^4V_4$	NG			
$^1V_3{}^3V_3{}^4V_1$	NG			
$^1V_3{}^3V_3{}^4V_2$	NG			
$^1V_4{}^3V_2{}^4V_1$	NG			
$^1V_4{}^3V_2{}^4V_2$	NG			
$^1V_5{}^3V_1{}^4V_1$	NG			
$^1V_5{}^3V_1{}^4V_2$	NG			
$\mathbf{^2V_r{}^3V_r{}^4V_r}$				
$^2V_1{}^3V_2{}^4V_1$	(10x**1**, 1x**2**)<br>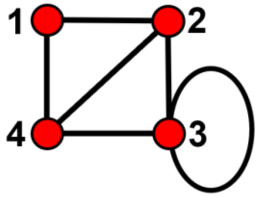	(8x**1**, 2x**2**)<br>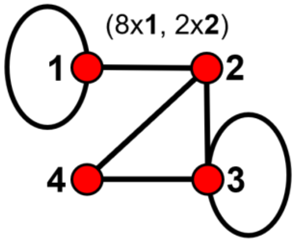	(8x**1**, 2x$\mathbf{2}^1$)<br>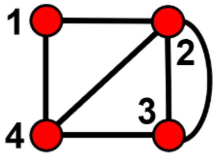	(8x**1**, 2x$\mathbf{2}^2$)<br>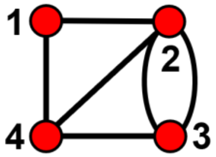

	(6x**1**, 3x**2**) 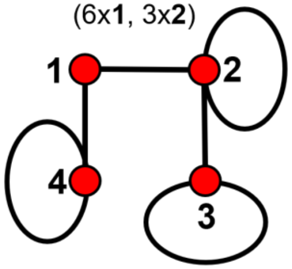	(6x**1**, 1x**2**, 2x**2**^{1}) **a** 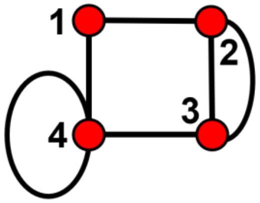	(6x**1**, 1x**2**, 2x**2**^{1}) **b** 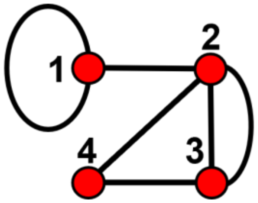	(6x**1**, 1x**2**, 2x**2**^{2}) **a** 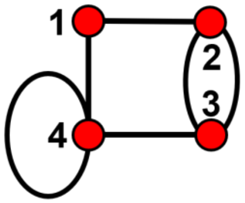
	(6x**1**, 1x**2**, 2x**2**^{2}) **b** 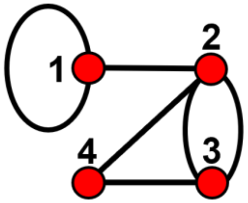	(4x**1**, 4x**2**) 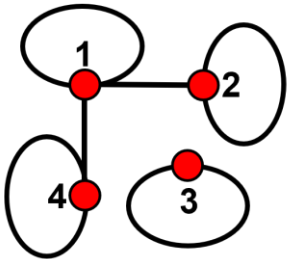	(4x**1**, 2x**2**, 2x**2**^{1}) **a** 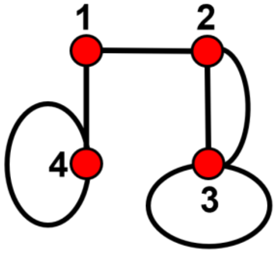	(4x**1**, 2x**2**, 2x**2**^{1}) **b** 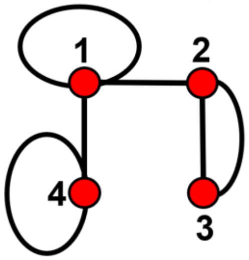
	(4x**1**, 2x**2**, 2x**2**^{1}) **c** 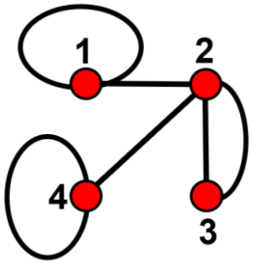	(4x**1**, 2x**2**, 2x**2**^{1}) **d** 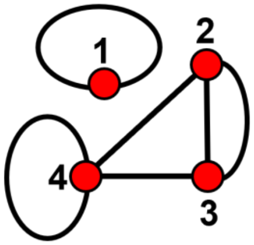	(4x**1**, 2x**2**, 2x**2**^{2}) **a** 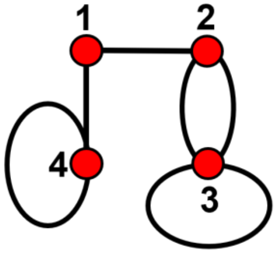	(4x**1**, 2x**2**, 2x**2**^{2}) **b** 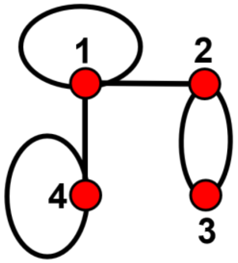

(4x**1**, 2x**2**, 2x$\mathbf{2}^{2}$) **c**

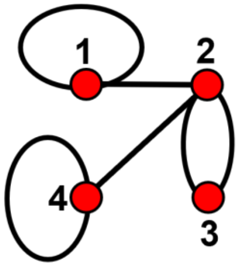

(4x**1**, 2x**2**, 2x$\mathbf{2}^{2}$) **d**

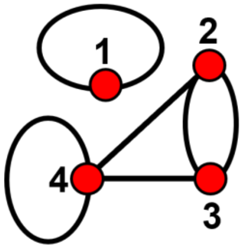

(4x**1**, 4x$\mathbf{2}^{1}$) **a**

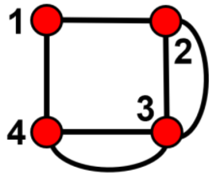

(4x**1**, 4x$\mathbf{2}^{1}$) **b**

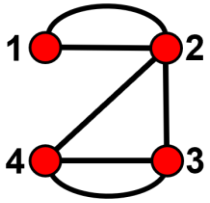

(4x**1**, 2x$\mathbf{2}^{1}$, 2x$\mathbf{2}^{2}$) **a**

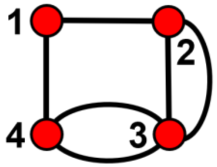

(4x**1**, 2x$\mathbf{2}^{1}$, 2x$\mathbf{2}^{2}$) **b**

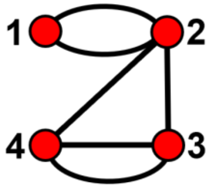

(4x**1**, 2x$\mathbf{2}^{1}$, 2x$\mathbf{2}^{2}$) **c**

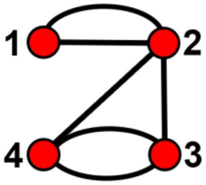

(4x**1**, 4x$\mathbf{2}^{2}$) **a**

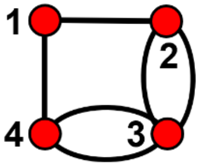

(4x**1**, 4x$\mathbf{2}^{2}$) **b**

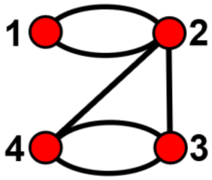

(2x**1**, 3x**2**, 2x$\mathbf{2}^{1}$) **a**

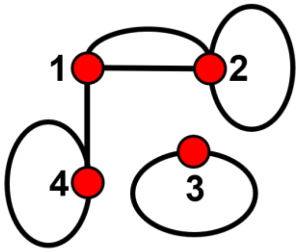

(2x**1**, 3x**2**, 2x$\mathbf{2}^{1}$) **b**

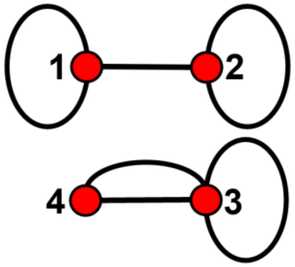

(2x**1**, 3x**2**, 2x$\mathbf{2}^{2}$) **a**

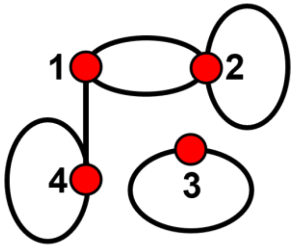

(2x**1**, 3x**2**, 2x$\mathbf{2}^{2}$) **b**

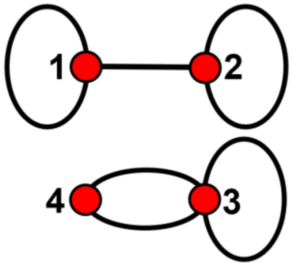

(2x**1**, 1x**2**, 4x$\mathbf{2}^{1}$) **a**

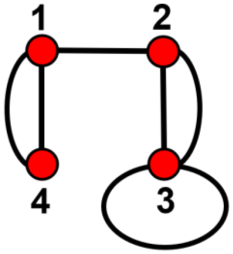

(2x**1**, 1x**2**, 4x$\mathbf{2}^{1}$) **b**

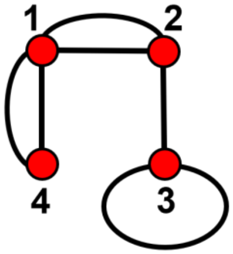

(2x**1**, 1x**2**, 4x$\mathbf{2}^{1}$) **c**

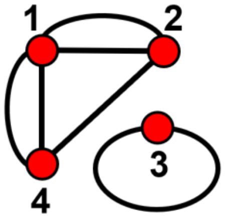

	(2x**1**, 1x**2**, 2x$\mathbf{2}^1$, 2x$\mathbf{2}^2$) **a** 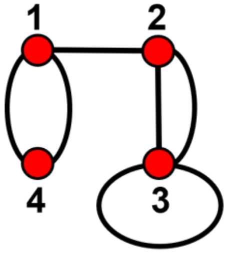	(2x**1**, 1x**2**, 2x$\mathbf{2}^1$, 2x$\mathbf{2}^2$) **b** 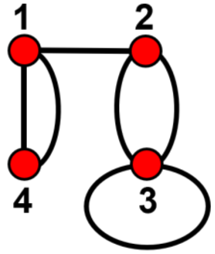	(2x**1**, 1x**2**, 2x$\mathbf{2}^1$, 2x$\mathbf{2}^2$) **c** 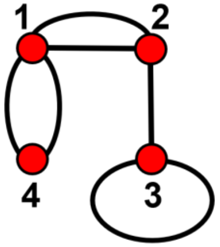	(2x**1**, 1x**2**, 2x$\mathbf{2}^1$, 2x$\mathbf{2}^2$) **d** 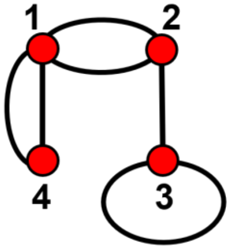
	(2x**1**, 1x**2**, 2x$\mathbf{2}^1$, 2x$\mathbf{2}^2$) **e** 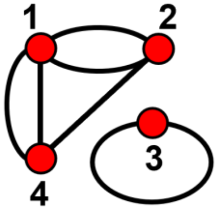	(2x**1**, 1x**2**, 4x$\mathbf{2}^2$) **a** 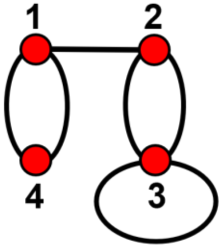	(2x**1**, 1x**2**, 4x$\mathbf{2}^2$) **b** 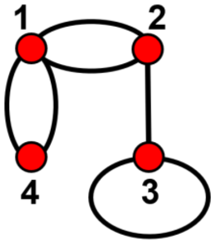	(2x**1**, 1x**2**, 4x$\mathbf{2}^2$) **c** 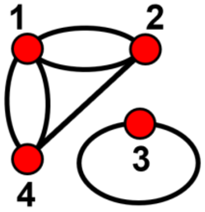
$^2V_1{}^3V_2{}^4V_2$	NG			
$^2V_1{}^3V_2{}^4V_3$	NG			
$^2V_1{}^3V_2{}^4V_4$	NG			
$^2V_1{}^3V_2{}^4V_5$	NG			
$^2V_1{}^3V_4{}^4V_1$	NG			
$^2V_1{}^3V_4{}^4V_2$	NG			
$^2V_1{}^3V_4{}^4V_3$	NG			
$^2V_1{}^3V_6{}^4V_1$	NG			

$^{2}V_{2}{}^{3}V_{2}{}^{4}V_{1}$	(14x**1**) 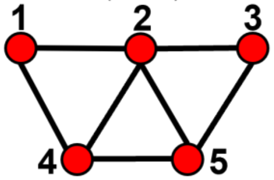	(12x**1**, 1x**2**) **a** 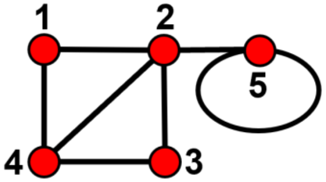	(12x**1**, 1x**2**) **b** 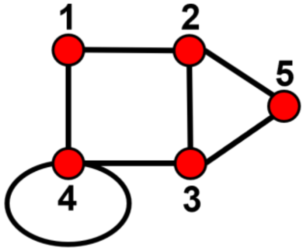	(12x**1**, 1x**2**) **c** 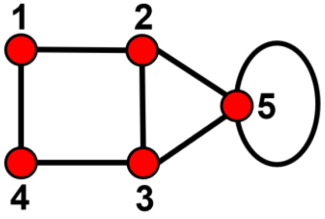
	(12x**1**, 1x**2**) **d** 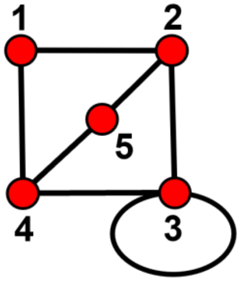	(10x**1**, 2x**2**) **a** 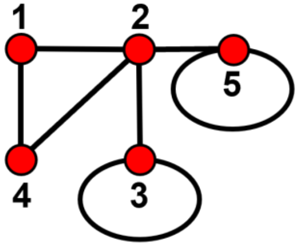	(10x**1**, 2x**2**) **b** 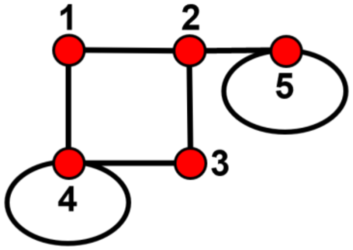	(10x**1**, 2x**2**) **c** 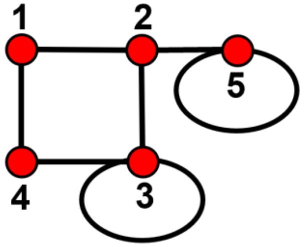
	(10x**1**, 2x**2**) **d** 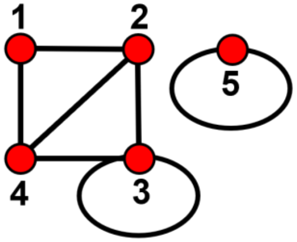	(10x**1**, 2x**2**) **e** 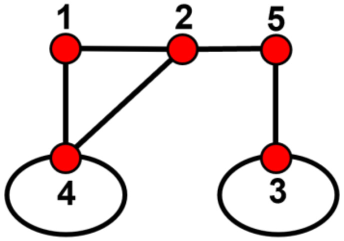	(10x**1**, 2x**2**) **f** 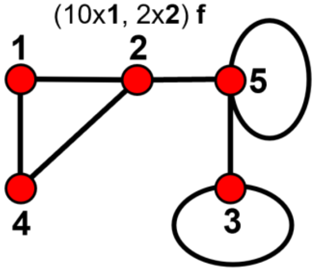	(10x**1**, 2x**2**^{1}) **a** 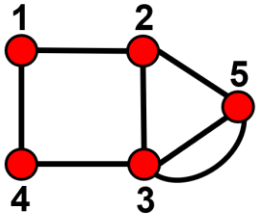

	(10x**1**, 2x**2**^{1}) **b** 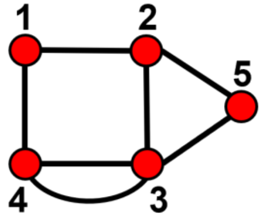	(10x**1**, 2x**2**^{1}) **c** 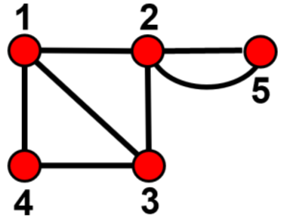	(10x**1**, 2x**2**^{1}) **d** 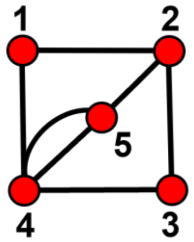	(10x**1**, 2x**2**^{1}) **e** 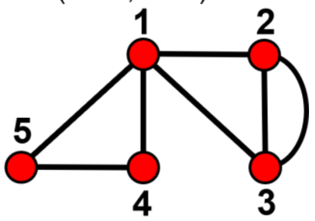
	(10x**1**, 2x**2**^{2}) **a** 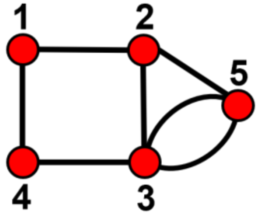	(10x**1**, 2x**2**^{2}) **b** 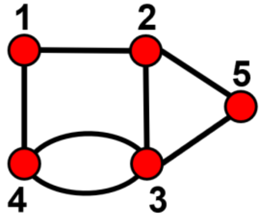	(10x**1**, 2x**2**^{2}) **c** 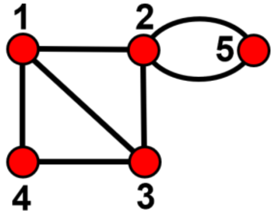	(10x**1**, 2x**2**^{2}) **d** 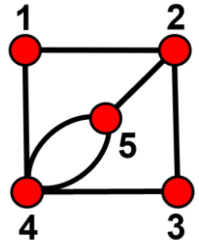
	(10x**1**, 2x**2**^{2}) **e** 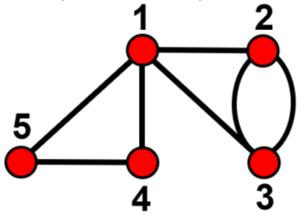	(8x**1**, 3x**2**) **a** 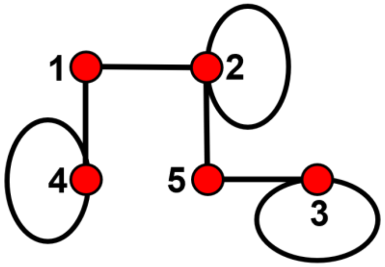	(8x**1**, 3x**2**) **b** 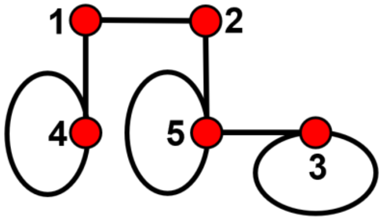	(8x**1**, 3x**2**) **c** 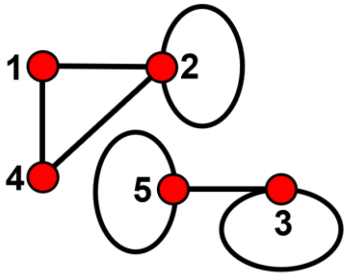

	(8x**1**, 3x**2**) **d**<br>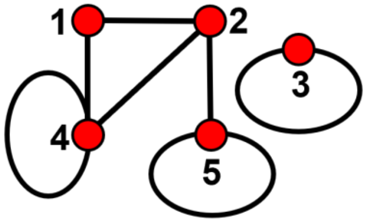 	(8x**1**, 1x**2**, 2x**2**^{1}) **a**<br>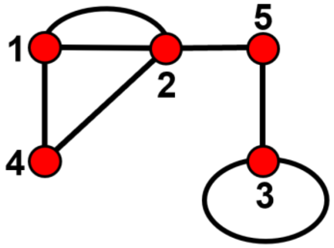 	(8x**1**, 1x**2**, 2x**2**^{1}) **b**<br>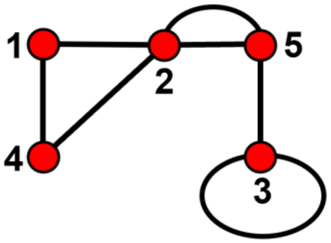 	(8x**1**, 1x**2**, 2x**2**^{1}) **c**<br>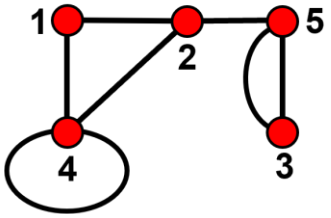
	(8x**1**, 1x**2**, 2x**2**^{1}) **d**<br>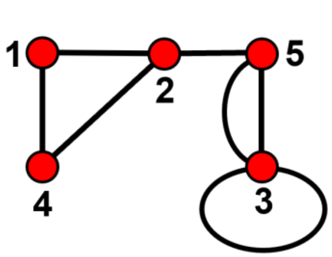 	(8x**1**, 1x**2**, 2x**2**^{1}) **e**<br>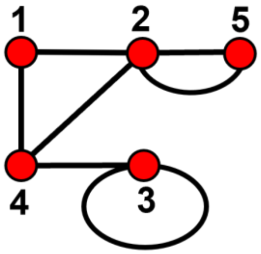 	(8x**1**, 1x**2**, 2x**2**^{1}) **f**<br>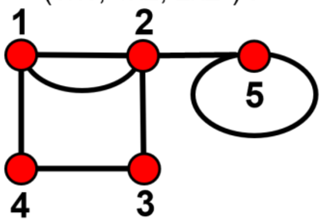 	(8x**1**, 1x**2**, 2x**2**^{1}) **g**<br>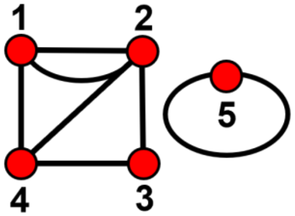
	(8x**1**, 1x**2**, 2x**2**^{1}) **h**<br>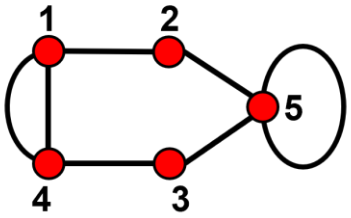 	(8x**1**, 1x**2**, 2x**2**^{1}) **i**<br>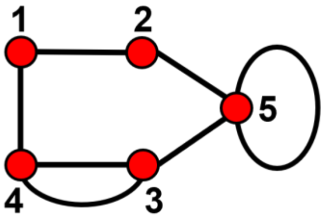 	(8x**1**, 1x**2**, 2x**2**^{2}) **a**<br>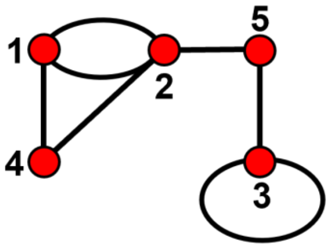 	(8x**1**, 1x**2**, 2x**2**^{2}) **b**<br>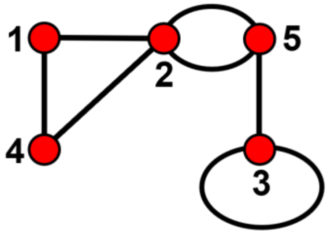
	(8x**1**, 1x**2**, 2x**2**^{2}) **c**<br>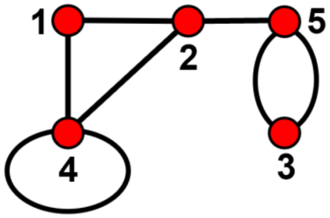 	(8x**1**, 1x**2**, 2x**2**^{2}) **d**<br>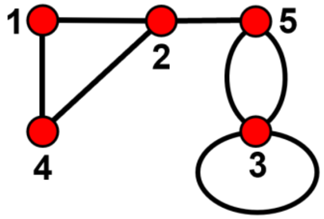 	(8x**1**, 1x**2**, 2x**2**^{2}) **e**<br>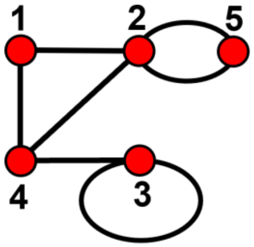 	(8x**1**, 1x**2**, 2x**2**^{2}) **f**<br>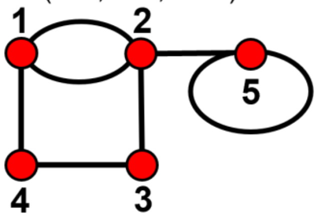

(8x**1**, 1x**2**, 2x$\mathbf{2}^{2}$) **g**

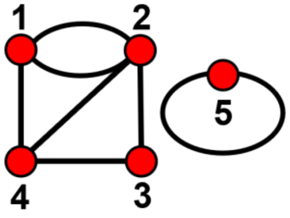

(8x**1**, 1x**2**, 2x$\mathbf{2}^{2}$) **h**

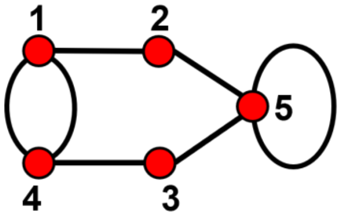

(8x**1**, 1x**2**, 2x$\mathbf{2}^{2}$) **i**

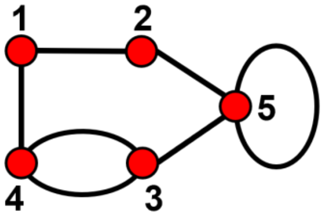

(6x**1**, 4x**2**)

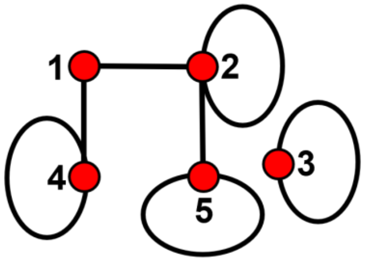

(6x**1**, 2x**2**, 2x$\mathbf{2}^{1}$) **a**

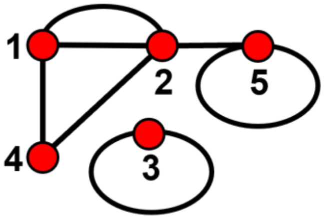

(6x**1**, 2x**2**, 2x$\mathbf{2}^{1}$) **b**

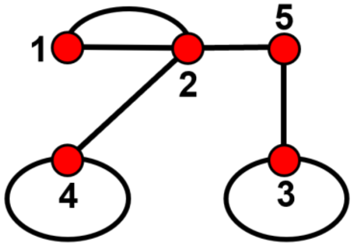

(6x**1**, 2x**2**, 2x$\mathbf{2}^{1}$) **c**

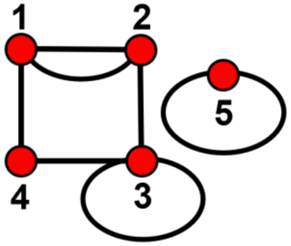

(6x**1**, 2x**2**, 2x$\mathbf{2}^{1}$) **d**

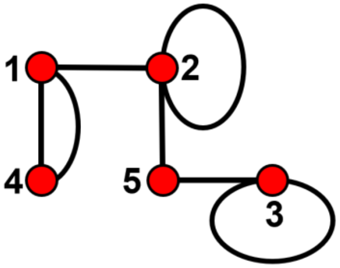

(6x**1**, 2x**2**, 2x$\mathbf{2}^{1}$) **e**

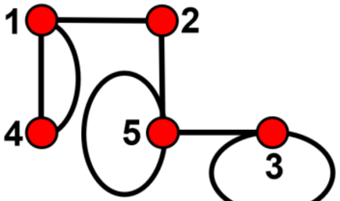

(6x**1**, 2x**2**, 2x$\mathbf{2}^{1}$) **f**

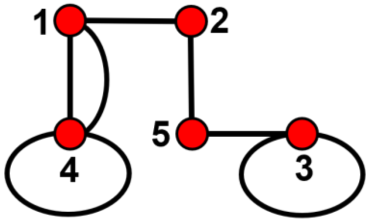

(6x**1**, 2x**2**, 2x$\mathbf{2}^{2}$) **a**

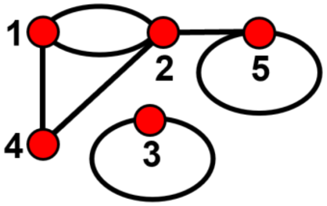

(6x**1**, 2x**2**, 2x$\mathbf{2}^{2}$) **b**

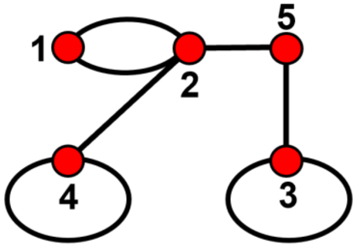

	(6x**1**, 2x**2**, 2x$\mathbf{2}^2$) **c** 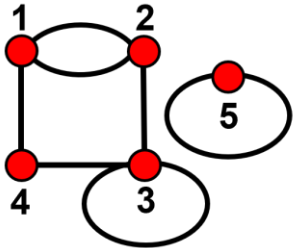	(6x**1**, 2x**2**, 2x$\mathbf{2}^2$) **d** 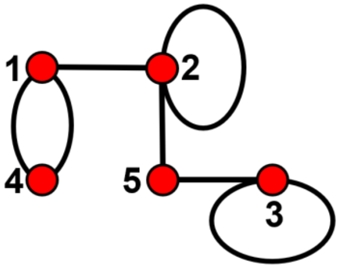	(6x**1**, 2x**2**, 2x$\mathbf{2}^2$) **e** 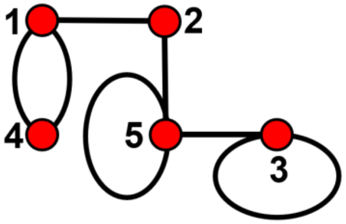	(6x**1**, 2x**2**, 2x$\mathbf{2}^2$) **f** 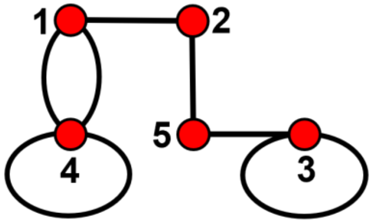
	(6x**1**, 4x$\mathbf{2}^1$) **a** 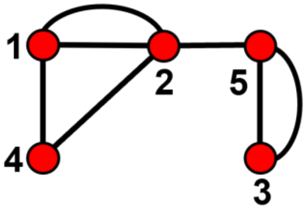	(6x**1**, 4x$\mathbf{2}^1$) **b** 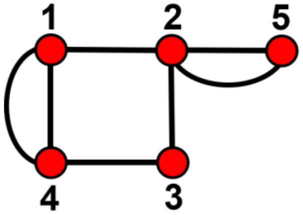	(6x**1**, 4x$\mathbf{2}^1$) **c** 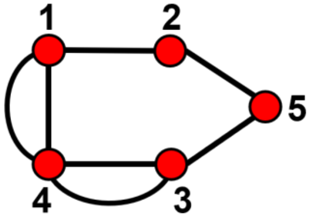	(6x**1**, 2x$\mathbf{2}^1$, 2x$\mathbf{2}^2$) **a** 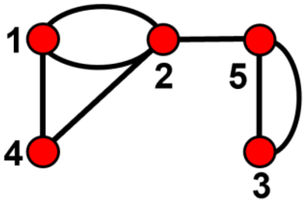
	(6x**1**, 2x$\mathbf{2}^1$, 2x$\mathbf{2}^2$) **b** 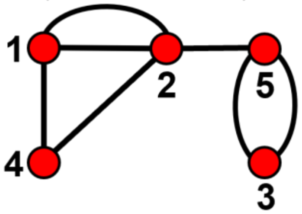	(6x**1**, 2x$\mathbf{2}^1$, 2x$\mathbf{2}^2$) **c** 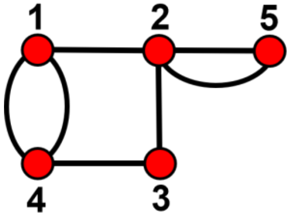	(6x**1**, 2x$\mathbf{2}^1$, 2x$\mathbf{2}^2$) **d** 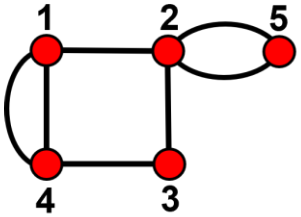	(6x**1**, 2x$\mathbf{2}^1$, 2x$\mathbf{2}^2$) **e** 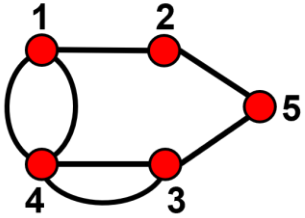

	(6x**1**, 4x$\mathbf{2}^{2}$) **a**	(6x**1**, 4x$\mathbf{2}^{2}$) **b**	(6x**1**, 4x$\mathbf{2}^{2}$) **c**	(4x**1**, 5x**2**)
	(4x**1**, 3x**2**, 2x$\mathbf{2}^{1}$) **a**	(4x**1**, 3x**2**, 2x$\mathbf{2}^{1}$) **b**	(4x**1**, 3x**2**, 2x$\mathbf{2}^{1}$) **c**	(4x**1**, 3x**2**, 2x$\mathbf{2}^{1}$) **d**
	(4x**1**, 3x**2**, 2x$\mathbf{2}^{1}$) **e**	(4x**1**, 3x**2**, 2x$\mathbf{2}^{1}$) **f**	(4x**1**, 3x**2**, 2x$\mathbf{2}^{2}$) **a**	(4x**1**, 3x**2**, 2x$\mathbf{2}^{2}$) **b**

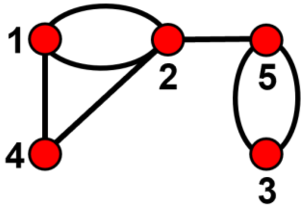

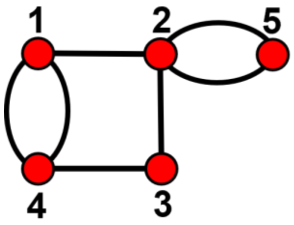

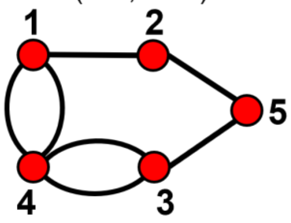

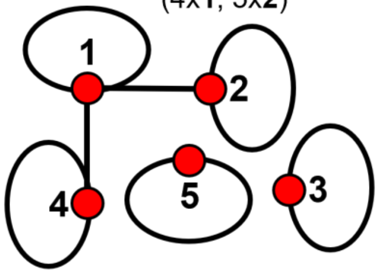

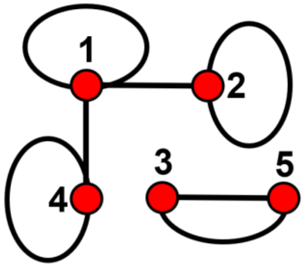

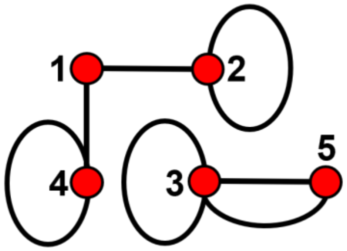

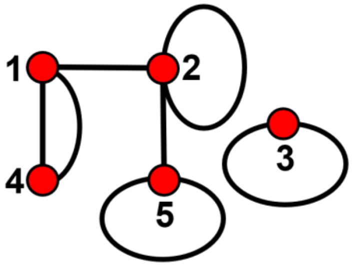

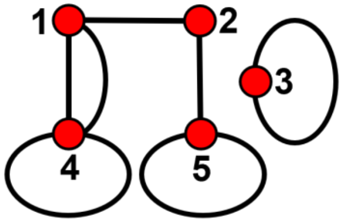

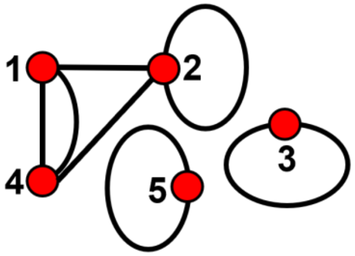

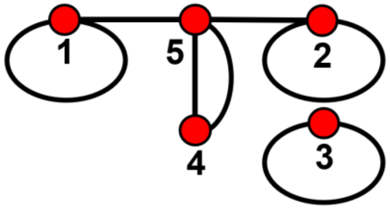

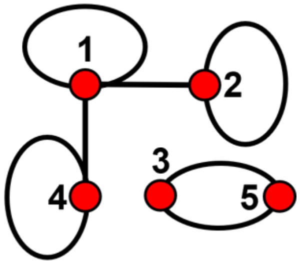

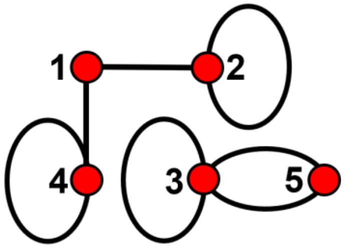

	(4x**1**, 3x**2**, 2x**2**^{2}) **c** 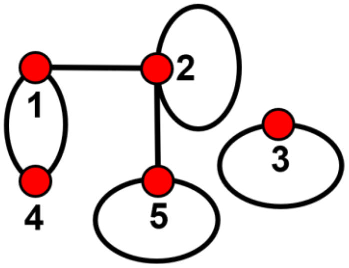	(4x**1**, 3x**2**, 2x**2**^{2}) **d** 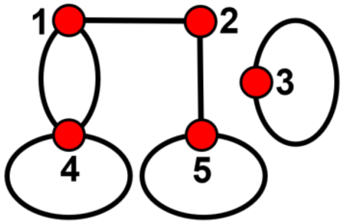	(4x**1**, 3x**2**, 2x**2**^{2}) **e** 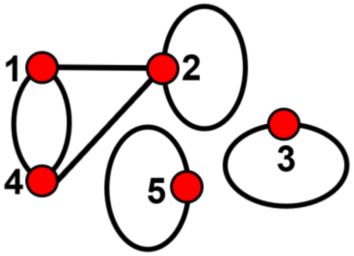	(4x**1**, 3x**2**, 2x**2**^{2}) **f** 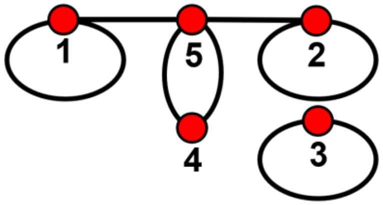
	(4x**1**, 1x**2**, 4x**2**^{1}) **a** 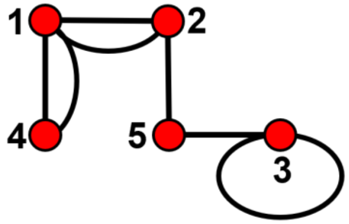	(4x**1**, 1x**2**, 4x**2**^{1}) **b** 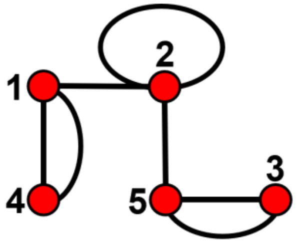	(4x**1**, 1x**2**, 4x**2**^{1}) **c** 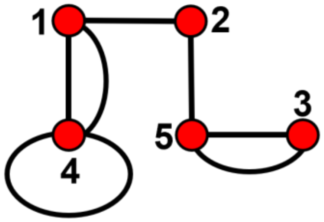	(4x**1**, 1x**2**, 4x**2**^{1}) **d** 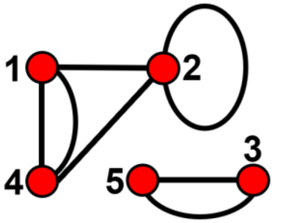
	(4x**1**, 1x**2**, 4x**2**^{1}) **e** 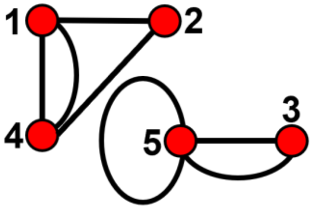	(4x**1**, 1x**2**, 4x**2**^{1}) **f** 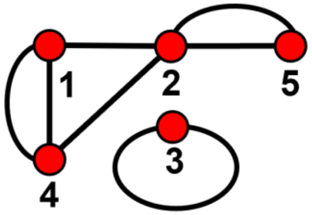	(4x**1**, 1x**2**, 4x**2**^{1}) **g** 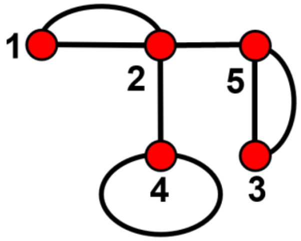	(4x**1**, 1x**2**, 4x**2**^{1}) **h** 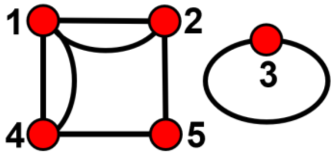
	(4x**1**, 1x**2**, 2x**2**^{1}, 2x**2**^{2}) **a** 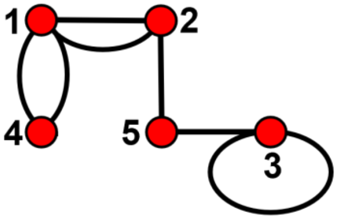	(4x**1**, 1x**2**, 2x**2**^{1}, 2x**2**^{2}) **b** 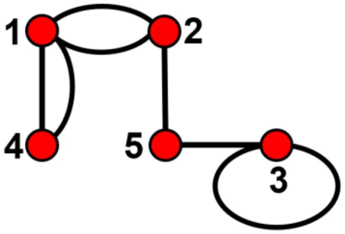	(4x**1**, 1x**2**, 2x**2**^{1}, 2x**2**^{2}) **c** 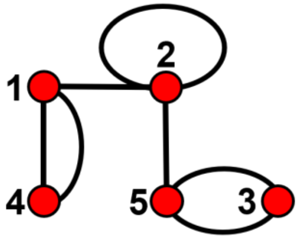	(4x**1**, 1x**2**, 2x**2**^{1}, 2x**2**^{2}) **d** 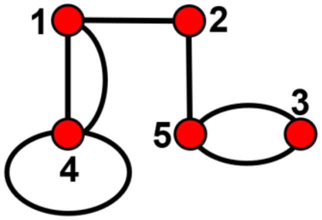

	(4x**1**, 1x**2**, 2x**2**^{1}, 2x**2**^{2}) **e** 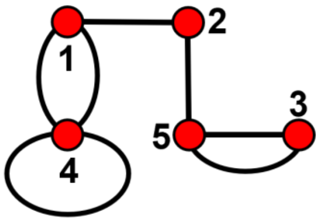	(4x**1**, 1x**2**, 2x**2**^{1}, 2x**2**^{2}) **f** 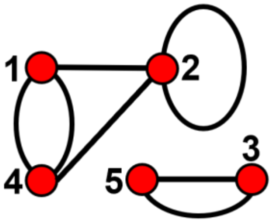	(4x**1**, 1x**2**, 2x**2**^{1}, 2x**2**^{2}) **g** 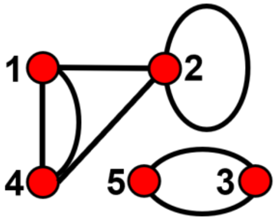	(4x**1**, 1x**2**, 2x**2**^{1}, 2x**2**^{2}) **h** 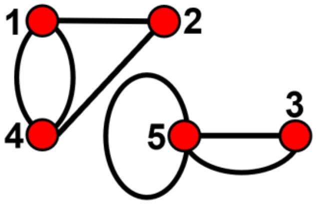
	(4x**1**, 1x**2**, 2x**2**^{1}, 2x**2**^{2}) **i** 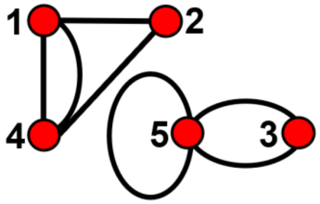	(4x**1**, 1x**2**, 2x**2**^{1}, 2x**2**^{2}) **j** 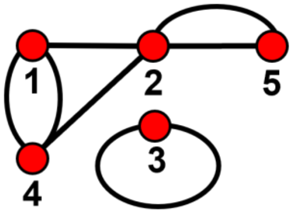	(4x**1**, 1x**2**, 2x**2**^{1}, 2x**2**^{2}) **k** 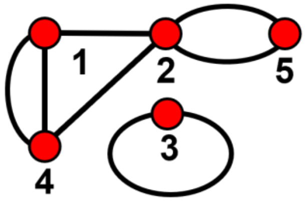	(4x**1**, 1x**2**, 2x**2**^{1}, 2x**2**^{2}) **l** 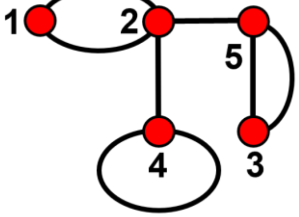
	(4x**1**, 1x**2**, 2x**2**^{1}, 2x**2**^{2}) **m** 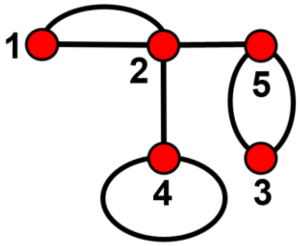	(4x**1**, 1x**2**, 2x**2**^{1}, 2x**2**^{2}) **n** 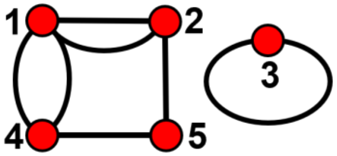	(4x**1**, 1x**2**, 4x**2**^{2}) **a** 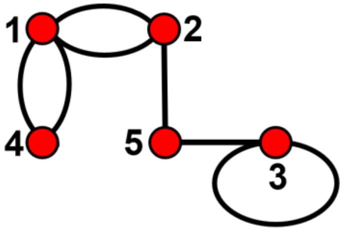	(4x**1**, 1x**2**, 4x**2**^{2}) **b** 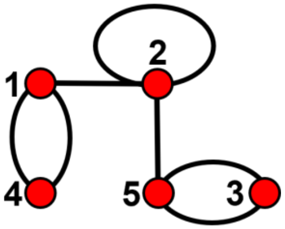
	(4x**1**, 1x**2**, 4x**2**^{2}) **c** 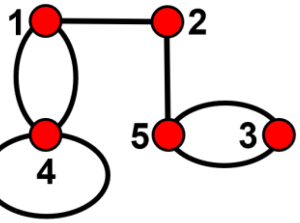	(4x**1**, 1x**2**, 4x**2**^{2}) **d** 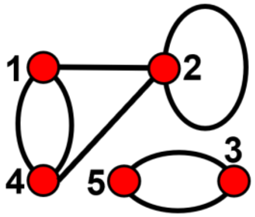	(4x**1**, 1x**2**, 4x**2**^{2}) **e** 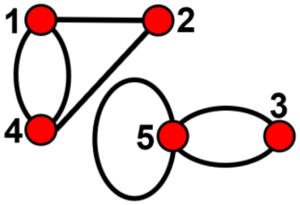	(4x**1**, 1x**2**, 4x**2**^{2}) **f** 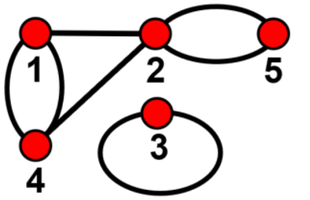

	(4x**1**, 1x**2**, 4x**2**^{2}) **g** 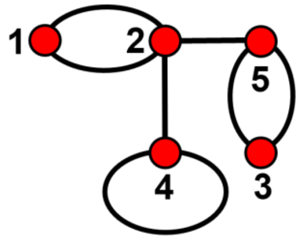	(4x**1**, 1x**2**, 4x**2**^{2}) **h** 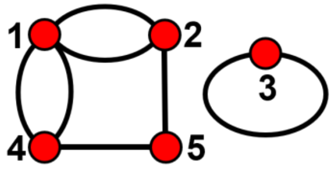	(2x**1**, 4x**2**, 2x**2**^{1}) **a** 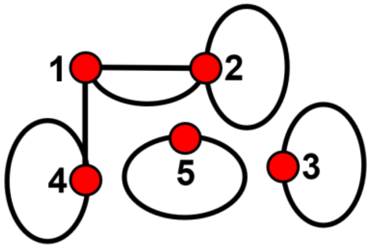	(2x**1**, 4x**2**, 2x**2**^{1}) **b** 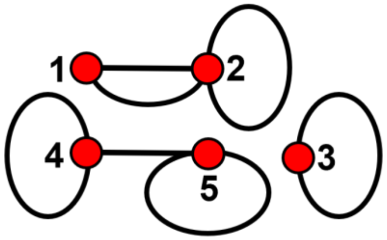
	(2x**1**, 4x**2**, 2x**2**^{2}) **a** 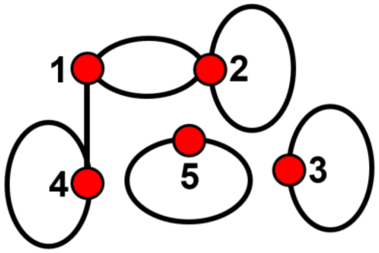	(2x**1**, 4x**2**, 2x**2**^{2}) **b** 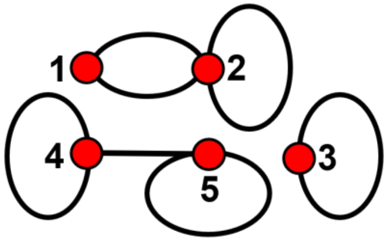	(2x**1**, 2x**2**, 4x**2**^{1}) **a** 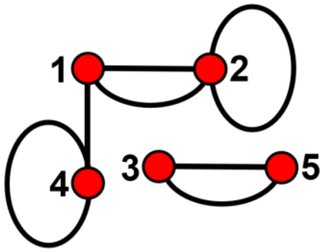	(2x**1**, 2x**2**, 4x**2**^{1}) **b** 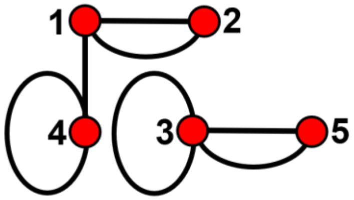
	(2x**1**, 2x**2**, 4x**2**^{1}) **c** 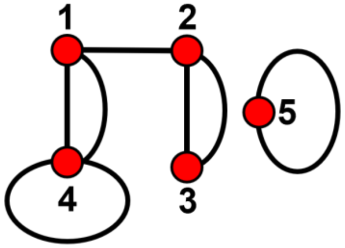	(2x**1**, 2x**2**, 4x**2**^{1}) **d** 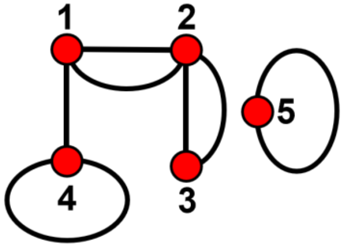	(2x**1**, 2x**2**, 4x**2**^{1}) **e** 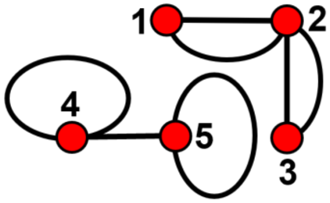	(2x**1**, 2x**2**, 4x**2**^{1}) **f** 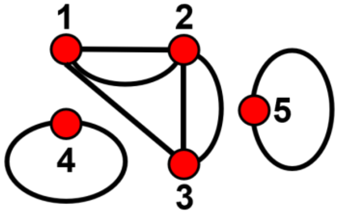
	(2x**1**, 2x**2**, 2x**2**^{1}, 2x**2**^{2}) **a** 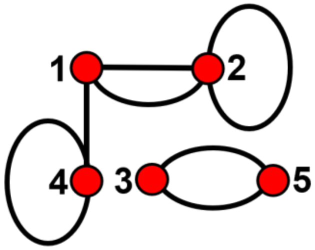	(2x**1**, 2x**2**, 2x**2**^{1}, 2x**2**^{2}) **b** 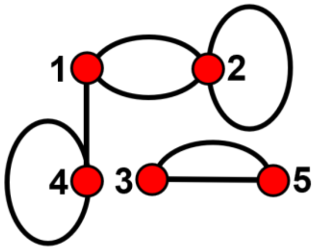	(2x**1**, 2x**2**, 2x**2**^{1}, 2x**2**^{2}) **c** 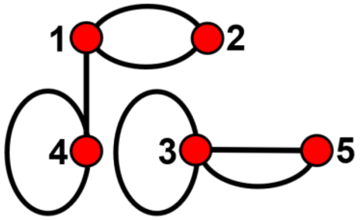	(2x**1**, 2x**2**, 2x**2**^{1}, 2x**2**^{2}) **d** 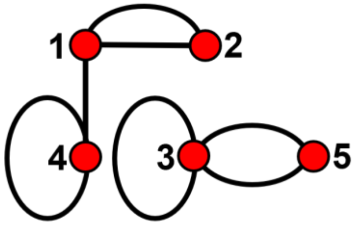

	(2x**1**, 2x**2**, 2x**2**^{1}, 2x**2**^{2}) **e**<br>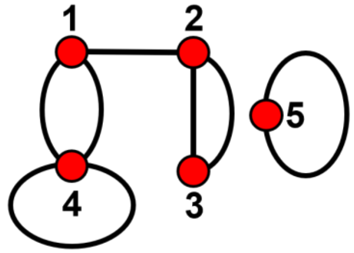	(2x**1**, 2x**2**, 2x**2**^{1}, 2x**2**^{2}) **f**<br>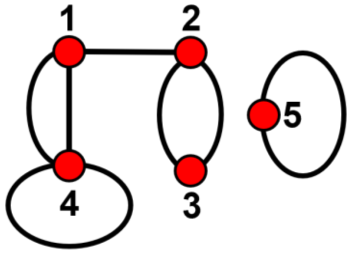	(2x**1**, 2x**2**, 2x**2**^{1}, 2x**2**^{2}) **g**<br>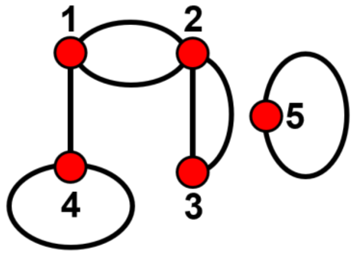	(2x**1**, 2x**2**, 2x**2**^{1}, 2x**2**^{2}) **h**<br>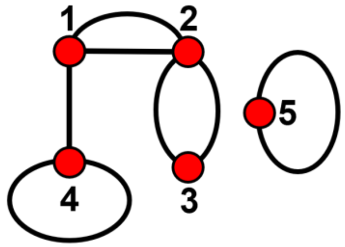
	(2x**1**, 2x**2**, 2x**2**^{1}, 2x**2**^{2}) **i**<br>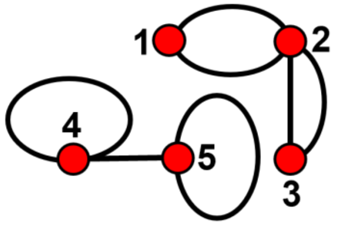	(2x**1**, 2x**2**, 2x**2**^{1}, 2x**2**^{2}) **j**<br>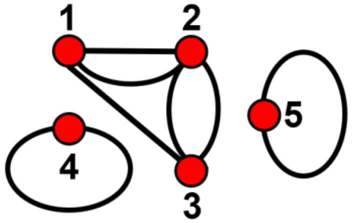	(2x**1**, 2x**2**, 4x**2**^{2}) **a**<br>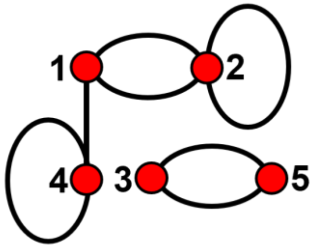	(2x**1**, 2x**2**, 4x**2**^{2}) **b**<br>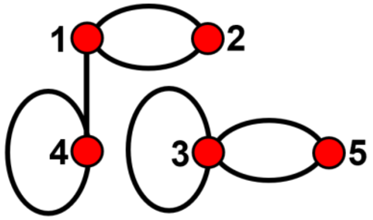
	(2x**1**, 2x**2**, 4x**2**^{2}) **c**<br>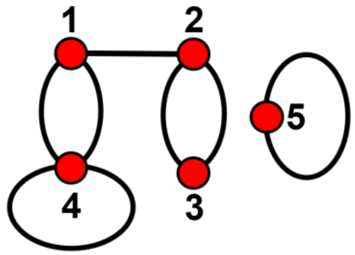	(2x**1**, 2x**2**, 4x**2**^{2}) **d**<br>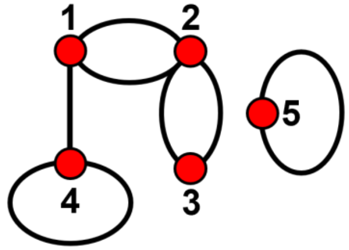	(2x**1**, 2x**2**, 4x**2**^{2}) **e**<br>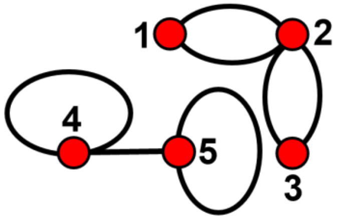	(2x**1**, 2x**2**, 4x**2**^{2}) **f**<br>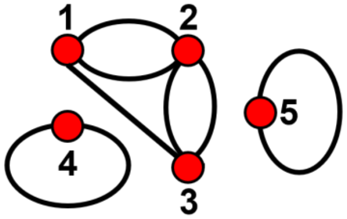
	(2x**1**, 6x**2**^{1}) **a**<br>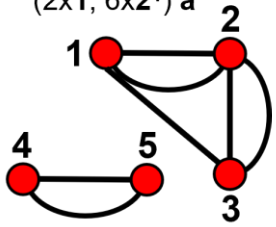	(2x**1**, 6x**2**^{1}) **b**<br>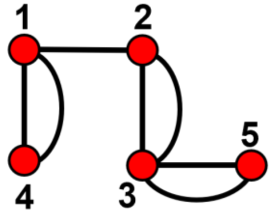	(2x**1**, 4x**2**^{1}, 2x**2**^{2}) **a**<br>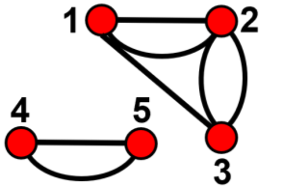	(2x**1**, 4x**2**^{1}, 2x**2**^{2}) **b**<br>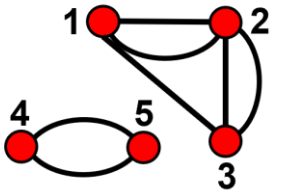

	(2x**1**, 4x**2**^{1}, 2x**2**^{2}) **c** 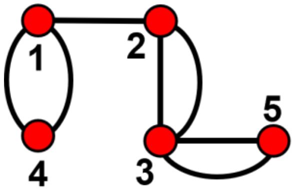	(2x**1**, 4x**2**^{1}, 2x**2**^{2}) **d** 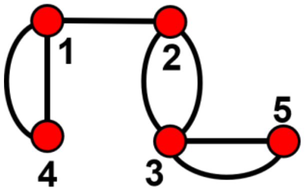	(2x**1**, 4x**2**^{1}, 2x**2**^{2}) **e** 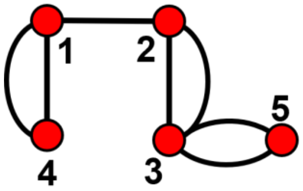	(2x**1**, 2x**2**^{1}, 4x**2**^{2}) **a** 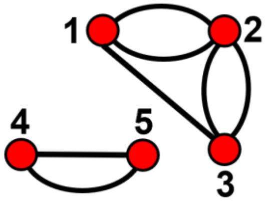
	(2x**1**, 2x**2**^{1}, 4x**2**^{2}) **b** 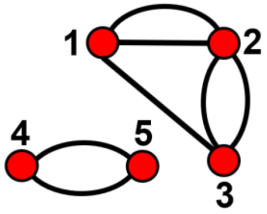	(2x**1**, 2x**2**^{1}, 4x**2**^{2}) **c** 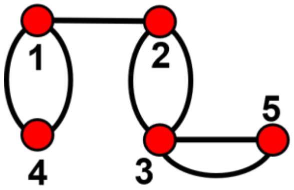	(2x**1**, 2x**2**^{1}, 4x**2**^{2}) **d** 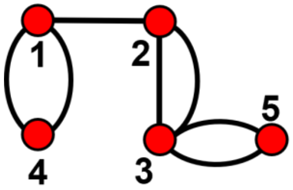	(2x**1**, 2x**2**^{1}, 4x**2**^{2}) **e** 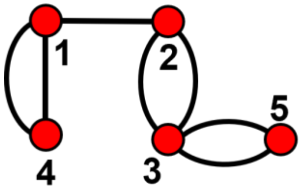
	(2x**1**, 6x**2**^{2}) **a** 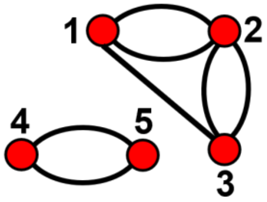	(2x**1**, 6x**2**^{2}) **b** 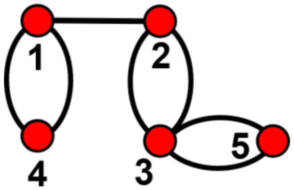		
$^2V_2{}^3V_2{}^4V_2$	NG			
$^2V_2{}^3V_2{}^4V_3$	NG			
$^2V_2{}^3V_2{}^4V_4$	NG			
$^2V_2{}^3V_4{}^4V_1$	NG			
$^2V_2{}^3V_4{}^4V_2$	NG			
$^2V_3{}^3V_2{}^4V_1$	NG			

$^{2}V_{3}{}^{3}V_{2}{}^{4}V_{2}$	NG			
$^{2}V_{3}{}^{3}V_{2}{}^{4}V_{3}$	NG			
$^{2}V_{3}{}^{3}V_{4}{}^{4}V_{1}$	NG			
$^{2}V_{4}{}^{3}V_{2}{}^{4}V_{1}$	NG			
$^{2}V_{4}{}^{3}V_{2}{}^{4}V_{2}$	NG			
$^{2}V_{5}{}^{3}V_{2}{}^{4}V_{1}$	NG			
**Rank 4**				
$\boldsymbol{^{1}V_{r}{}^{2}V_{r}{}^{3}V_{r}{}^{4}V_{r}}$				
$^{1}V_{1}{}^{2}V_{1}{}^{3}V_{1}{}^{4}V_{1}$	(8x**1**, 1x**2**) 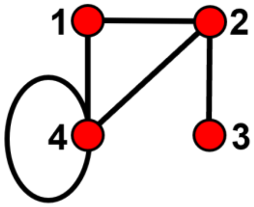	(6x**1**, 2x**2**) **a** 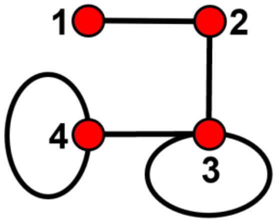	(6x**1**, 2x**2**) **b** 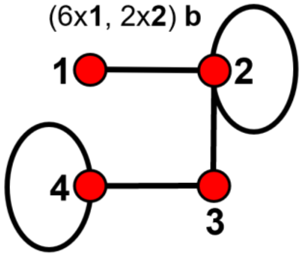	(6x**1**, 2x**2**^{1}) 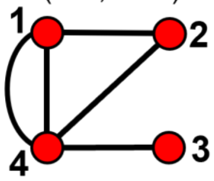
	(6x**1**, 2x**2**^{2}) 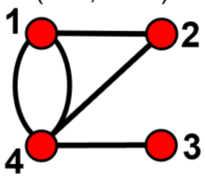	(4x**1**, 3x**2**) 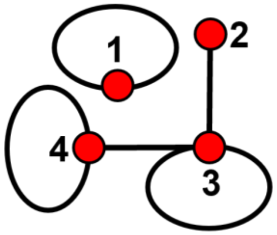	(4x**1**, 1x**2**, 2x**2**^{1}) **a** 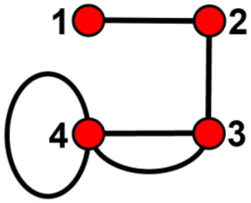	(4x**1**, 1x**2**, 2x**2**^{1}) **b** 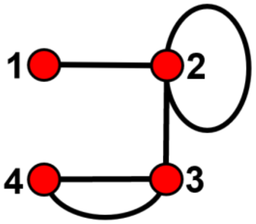

	(4x**1**, 1x**2**, 2x**2**^{1}) **c** 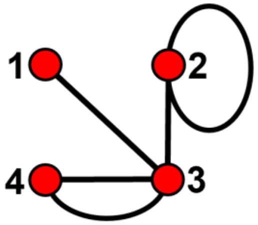	(4x**1**, 1x**2**, 2x**2**^{2}) **a** 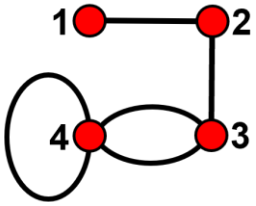	(4x**1**, 1x**2**, 2x**2**^{2}) **b** 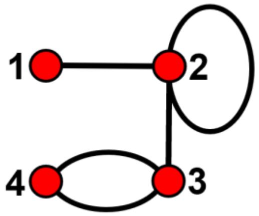	(4x**1**, 1x**2**, 2x**2**^{2}) **c** 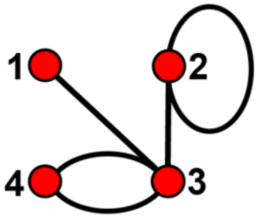
	(2x**1**, 2x**2**, 2x**2**^{1}) **a** 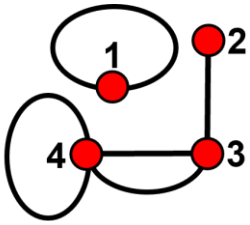	(2x**1**, 2x**2**, 2x**2**^{1}) **b** 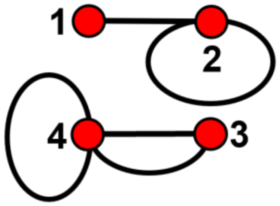	(2x**1**, 2x**2**, 2x**2**^{2}) **a** 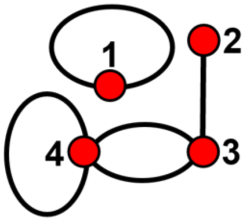	(2x**1**, 2x**2**, 2x**2**^{2}) **b** 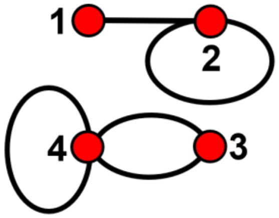
	(2x**1**, 4x**2**^{1}) 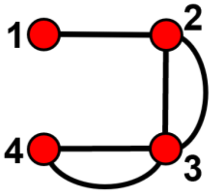	(2x**1**, 2x**2**^{1}, 2x**2**^{2}) **a** 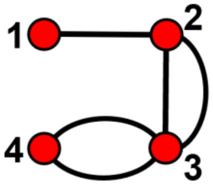	(2x**1**, 2x**2**^{1}, 2x**2**^{2}) **b** 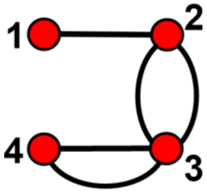	(2x**1**, 4x**2**^{2}) 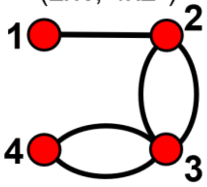
$^{1}V_{1}{}^{2}V_{1}{}^{3}V_{1}{}^{4}V_{2}$	(12x**1**, 1x**2**) 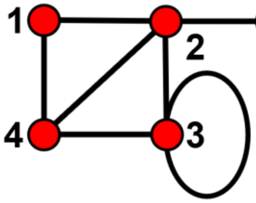	(10x**1**, 2x**2**) **a** 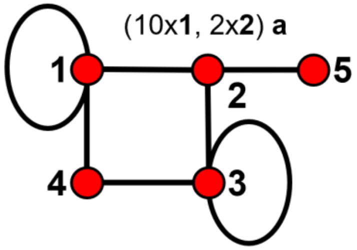	(10x**1**, 2x**2**) **b** 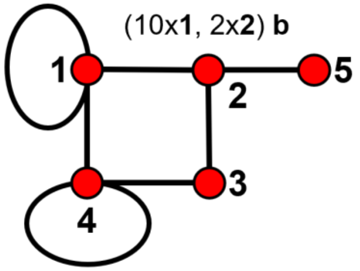	(10x**1**, 2x**2**) **c** 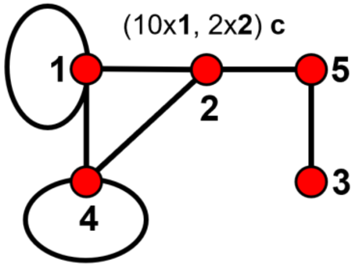

	(10x**1**, 2x**2**) **d**<br>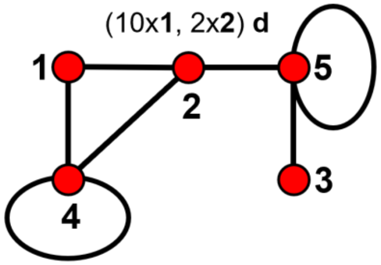	(10x**1**, 2x**2**) **e**<br>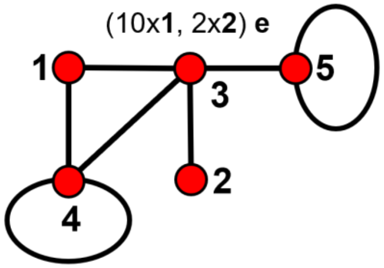	(10x**1**, 2x**2**^{1}) **a**<br>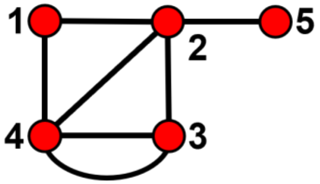	(10x**1**, 2x**2**^{1}) **b**<br>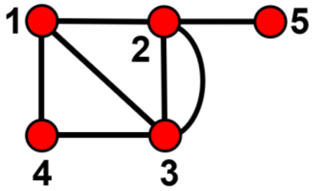
	(10x**1**, 2x**2**^{1}) **c**<br>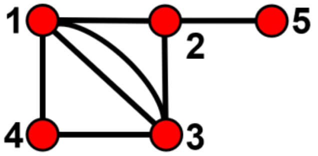	(10x**1**, 2x**2**^{2}) **a**<br>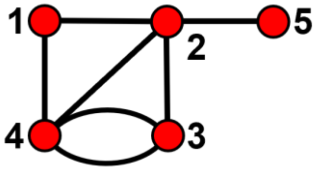	(10x**1**, 2x**2**^{2}) **b**<br>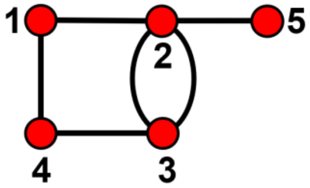	(10x**1**, 2x**2**^{2}) **c**<br>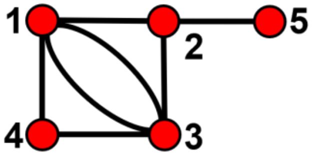
	(8x**1**, 3x**2**) **a**<br>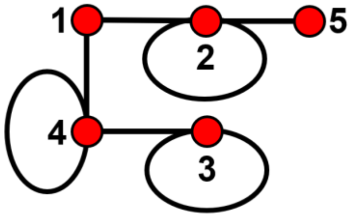	(8x**1**, 3x**2**) **b**<br>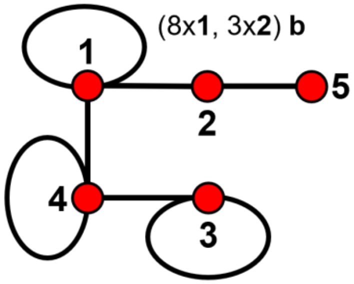	(8x**1**, 3x**2**) **c**<br>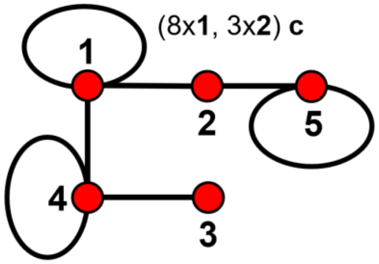	(8x**1**, 3x**2**) **d**<br>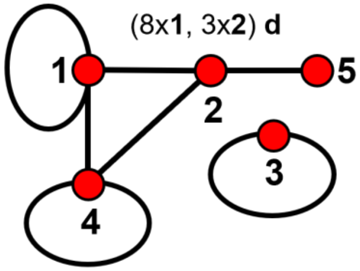
	(8x**1**, 3x**2**) **e**<br>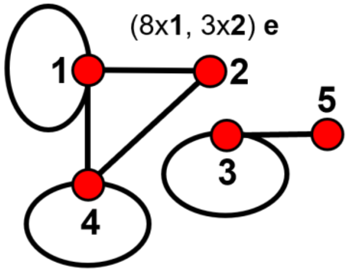	(8x**1**, 1x**2**, 2x**2**^{1}) **a**<br>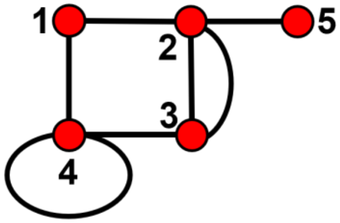	(8x**1**, 1x**2**, 2x**2**^{1}) **b**<br>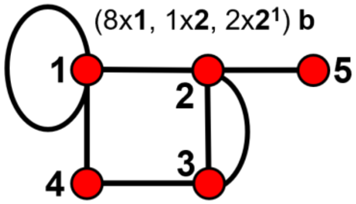	(8x**1**, 1x**2**, 2x**2**^{1}) **c**<br>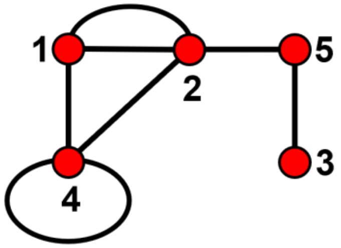

	(8x**1**, 1x**2**, 2x**2**¹) **d** 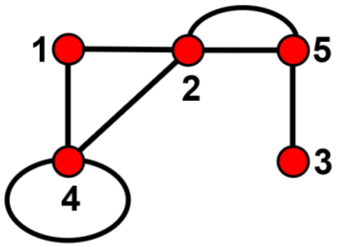	(8x**1**, 1x**2**, 2x**2**¹) **e** 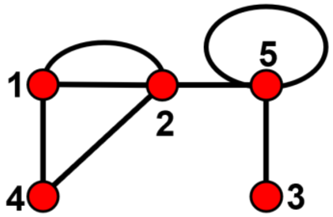	(8x**1**, 1x**2**, 2x**2**¹) **f** 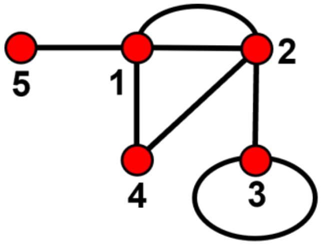	(8x**1**, 1x**2**, 2x**2**¹) **g** 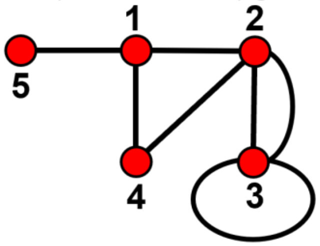
	(8x**1**, 1x**2**, 2x**2**¹) **h** 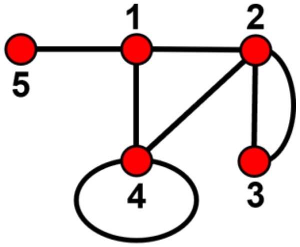	(8x**1**, 1x**2**, 2x**2**²) **a** 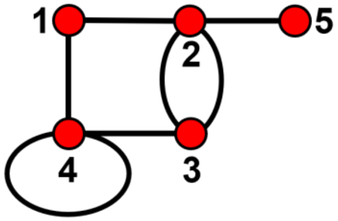	(8x**1**, 1x**2**, 2x**2**²) **b** 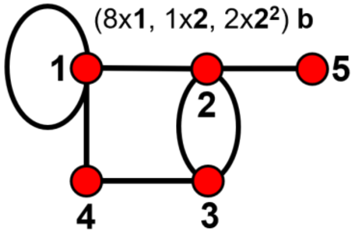	(8x**1**, 1x**2**, 2x**2**²) **c** 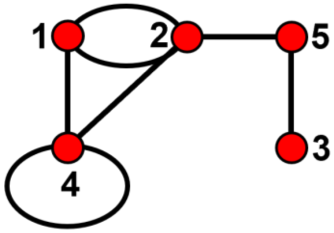
	(8x**1**, 1x**2**, 2x**2**²) **d** 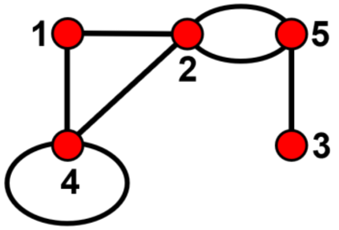	(8x**1**, 1x**2**, 2x**2**²) **e** 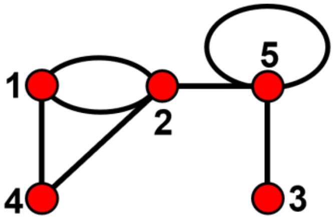	(8x**1**, 1x**2**, 2x**2**²) **f** 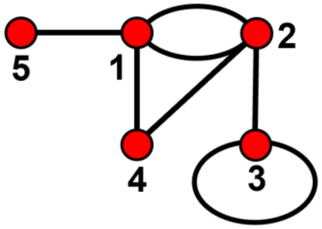	(8x**1**, 1x**2**, 2x**2**²) **g** 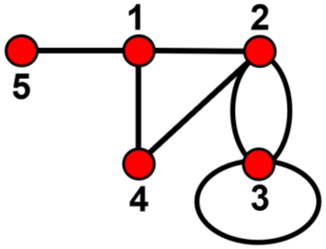
	(8x**1**, 1x**2**, 2x**2**²) **h** 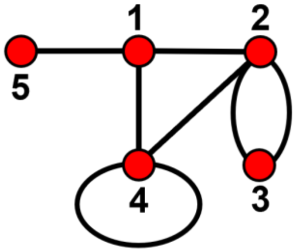	(6x**1**, 4x**2**) 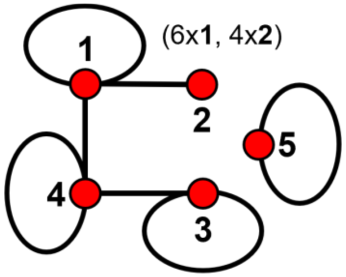	(6x**1**, 2x**2**, 2x**2**¹) **a** 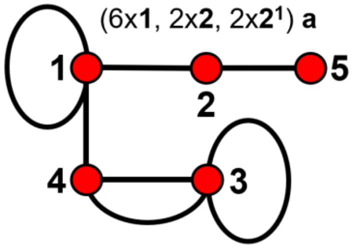	(6x**1**, 2x**2**, 2x**2**¹) **b** 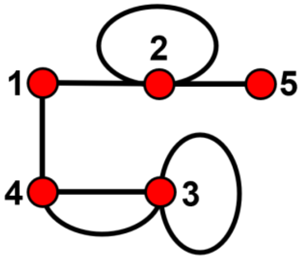

	(6x**1**, 2x**2**, 2x**2**^{1}) **c** 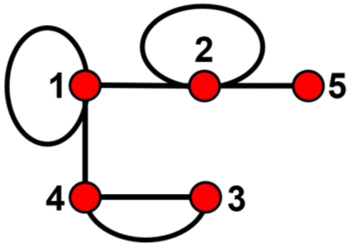	(6x**1**, 2x**2**, 2x**2**^{1}) **d** 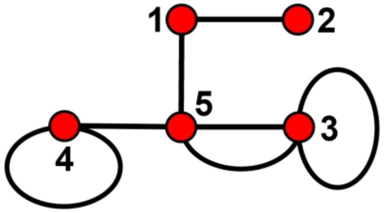	(6x**1**, 2x**2**, 2x**2**^{1}) **e** 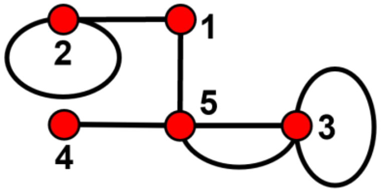	(6x**1**, 2x**2**, 2x**2**^{1}) **f** 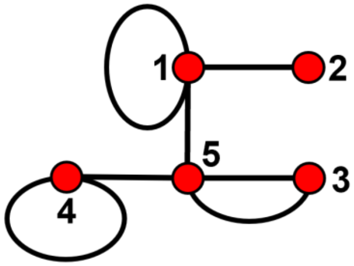
	(6x**1**, 2x**2**, 2x**2**^{1}) **g** 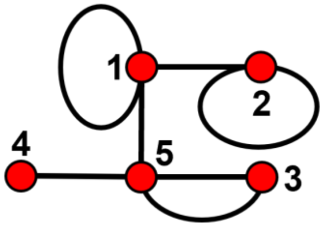	(6x**1**, 2x**2**, 2x**2**^{1}) **h** 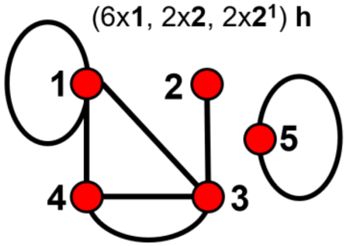	(6x**1**, 2x**2**, 2x**2**^{2}) **a** 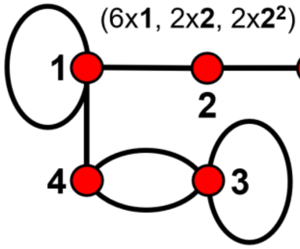	(6x**1**, 2x**2**, 2x**2**^{2}) **b** 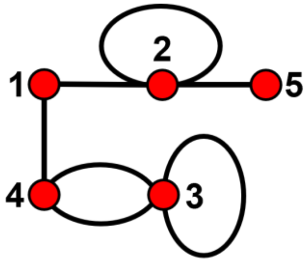
	(6x**1**, 2x**2**, 2x**2**^{2}) **c** 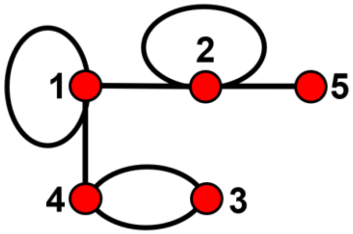	(6x**1**, 2x**2**, 2x**2**^{2}) **d** 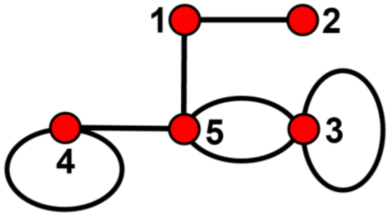	(6x**1**, 2x**2**, 2x**2**^{2}) **e** 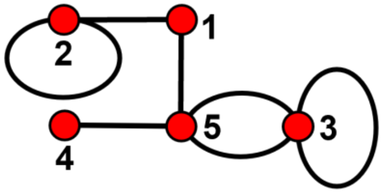	(6x**1**, 2x**2**, 2x**2**^{2}) **f** 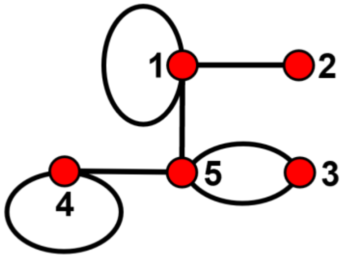
	(6x**1**, 2x**2**, 2x**2**^{2}) **g** 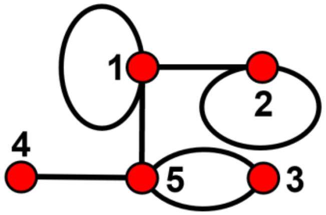	(6x**1**, 2x**2**, 2x**2**^{2}) **h** 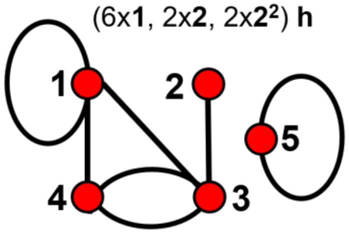	(6x**1**, 4x**2**^{1}) **a** 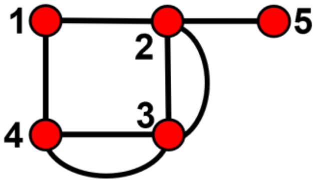	(6x**1**, 4x**2**^{1}) **b** 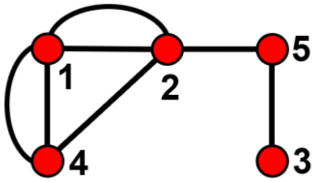

	(6x**1**, 4x$\mathbf{2}^1$) **c** 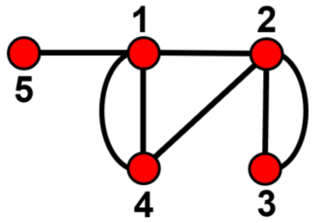	(6x**1**, 2x$\mathbf{2}^1$, 2x$\mathbf{2}^2$) **a** 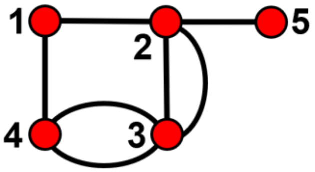	(6x**1**, 2x$\mathbf{2}^1$, 2x$\mathbf{2}^2$) **b** 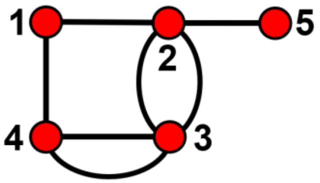	(6x**1**, 2x$\mathbf{2}^1$, 2x$\mathbf{2}^2$) **c** 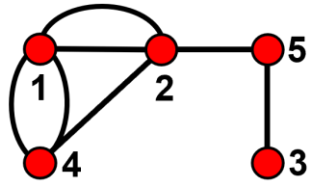
	(6x**1**, 2x$\mathbf{2}^1$, 2x$\mathbf{2}^2$) **d** 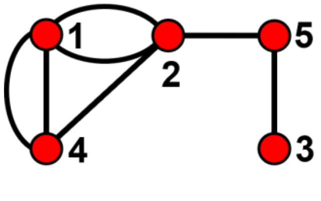	(6x**1**, 2x$\mathbf{2}^1$, 2x$\mathbf{2}^2$) **e** 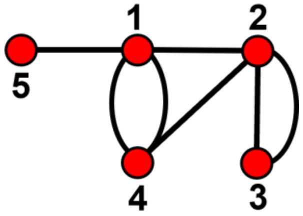	(6x**1**, 2x$\mathbf{2}^1$, 2x$\mathbf{2}^2$) **f** 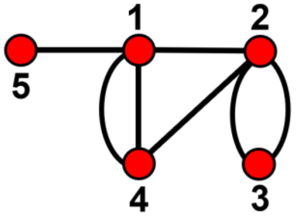	(6x**1**, 4x$\mathbf{2}^2$) **a** 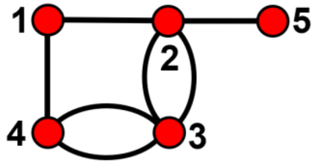
	(6x**1**, 4x$\mathbf{2}^2$) **b** 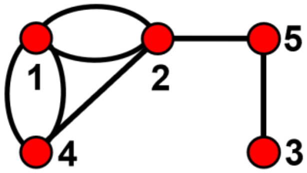	(6x**1**, 4x$\mathbf{2}^2$) **c** 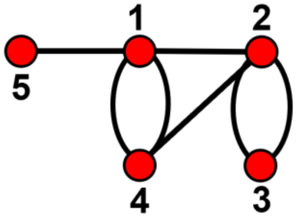	(4x**1**, 3x**2**, 2x$\mathbf{2}^1$) **a** 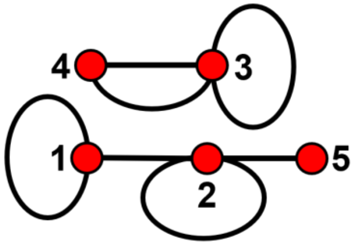	(4x**1**, 3x**2**, 2x$\mathbf{2}^1$) **b** 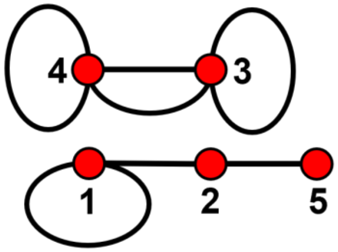

(4x**1**, 3x**2**, 2x$\mathbf{2}^{1}$) **c**

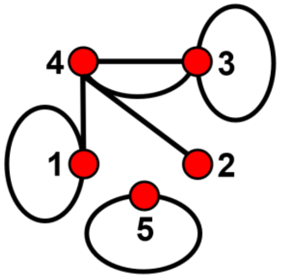

(4x**1**, 3x**2**, 2x$\mathbf{2}^{1}$) **d**

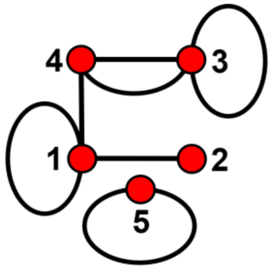

(4x**1**, 3x**2**, 2x$\mathbf{2}^{2}$) **a**

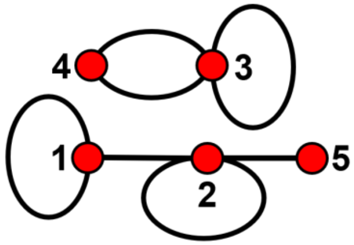

(4x**1**, 3x**2**, 2x$\mathbf{2}^{2}$) **b**

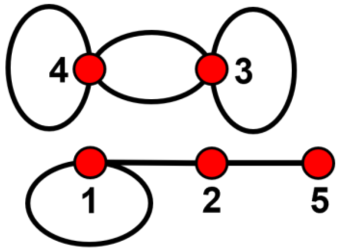

(4x**1**, 3x**2**, 2x$\mathbf{2}^{2}$) **c**

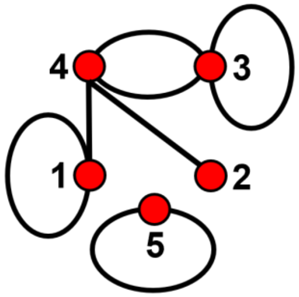

(4x**1**, 3x**2**, 2x$\mathbf{2}^{2}$) **d**

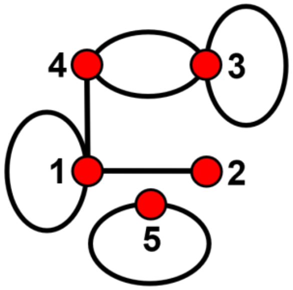

(4x**1**, 1x**2**, 4x$\mathbf{2}^{1}$) **a**

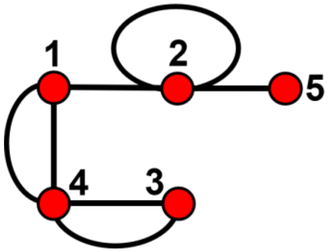

(4x**1**, 1x**2**, 4x$\mathbf{2}^{1}$) **b**

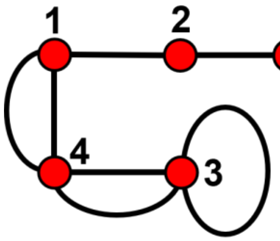

(4x**1**, 1x**2**, 4x$\mathbf{2}^{1}$) **c**

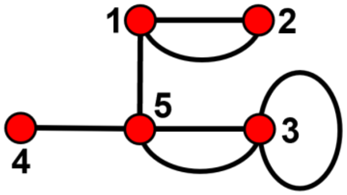

(4x**1**, 1x**2**, 4x$\mathbf{2}^{1}$) **d**

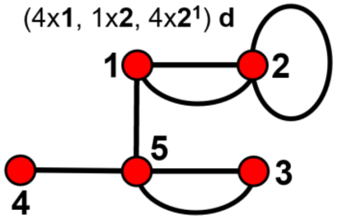

(4x**1**, 1x**2**, 4x$\mathbf{2}^{1}$) **e**

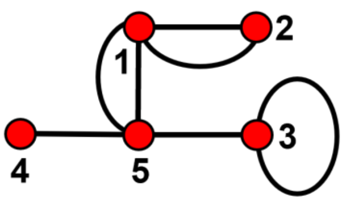

(4x**1**, 1x**2**, 4x$\mathbf{2}^{1}$) **f**

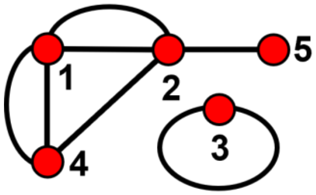

	(4x**1**, 1x**2**, 2x$\mathbf{2}^1$, 2x$\mathbf{2}^2$) **a** 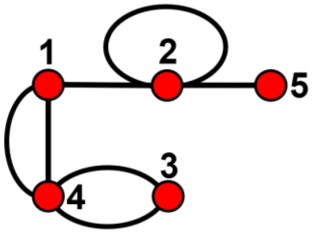	(4x**1**, 1x**2**, 2x$\mathbf{2}^1$, 2x$\mathbf{2}^2$) **b** 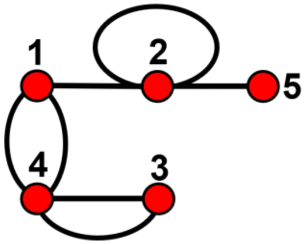	(4x**1**, 1x**2**, 2x$\mathbf{2}^1$, 2x$\mathbf{2}^2$) **c** 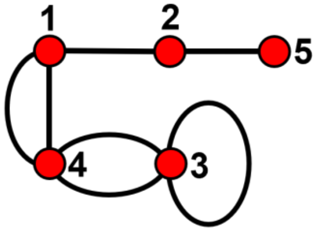	(4x**1**, 1x**2**, 2x$\mathbf{2}^1$, 2x$\mathbf{2}^2$) **d** 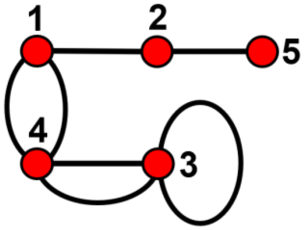
	(4x**1**, 1x**2**, 2x$\mathbf{2}^1$, 2x$\mathbf{2}^2$) **e** 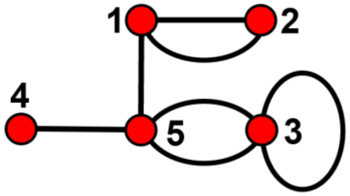	(4x**1**, 1x**2**, 2x$\mathbf{2}^1$, 2x$\mathbf{2}^2$) **f** 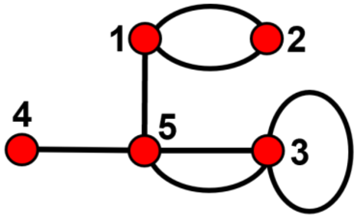	(4x**1**, 1x**2**, 2x$\mathbf{2}^1$, 2x$\mathbf{2}^2$) **g** 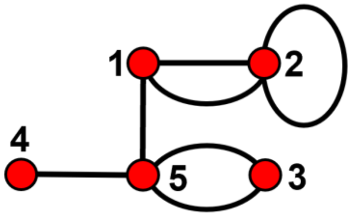	(4x**1**, 1x**2**, 2x$\mathbf{2}^1$, 2x$\mathbf{2}^2$) **h** 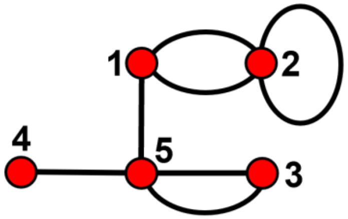
	(4x**1**, 1x**2**, 2x$\mathbf{2}^1$, 2x$\mathbf{2}^2$) **i** 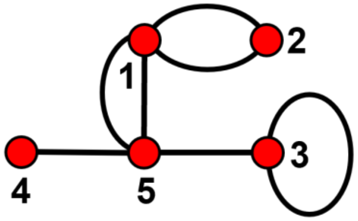	(4x**1**, 1x**2**, 2x$\mathbf{2}^1$, 2x$\mathbf{2}^2$) **j** 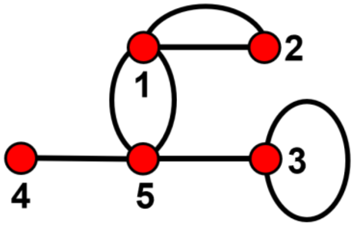	(4x**1**, 1x**2**, 2x$\mathbf{2}^1$, 2x$\mathbf{2}^2$) **k** 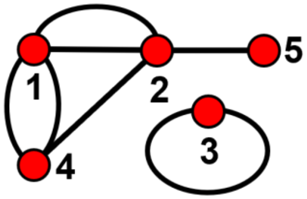	(4x**1**, 1x**2**, 2x$\mathbf{2}^1$, 2x$\mathbf{2}^2$) **l** 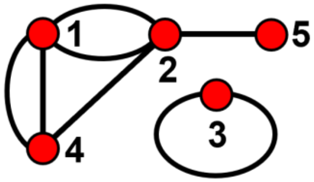
	(4x**1**, 1x**2**, 4x$\mathbf{2}^2$) **a** 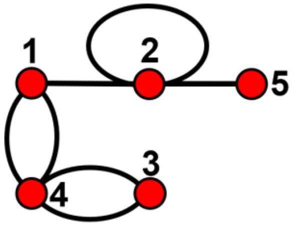	(4x**1**, 1x**2**, 4x$\mathbf{2}^2$) **b** 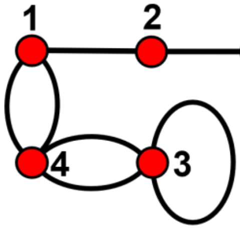	(4x**1**, 1x**2**, 4x$\mathbf{2}^2$) **c** 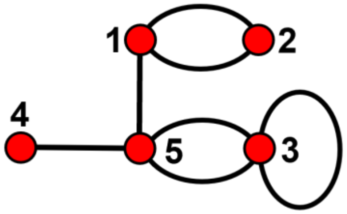	(4x**1**, 1x**2**, 4x$\mathbf{2}^2$) **d** 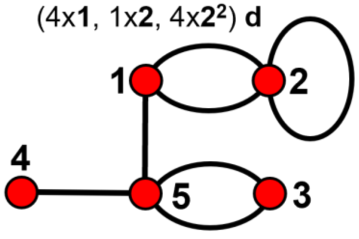

	(4x**1**, 1x**2**, 4x**2**^{2}) **e** 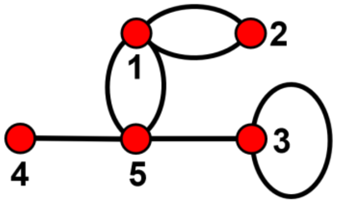	(4x**1**, 1x**2**, 4x**2**^{2}) **f** 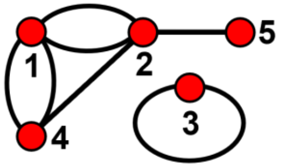	(2x**1**, 4x**2**, 2x**2**^{1}) 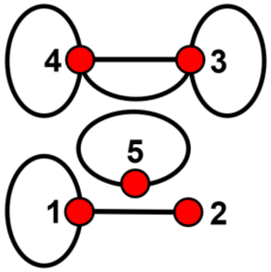	(2x**1**, 4x**2**, 2x**2**^{2}) 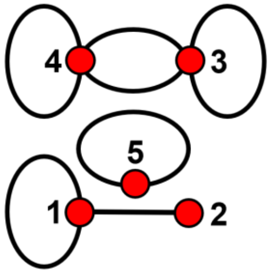
	(2x**1**, 2x**2**, 4x**2**^{1}) **a** 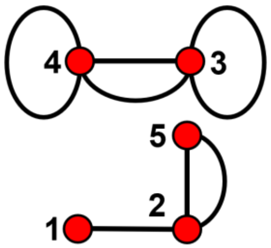	(2x**1**, 2x**2**, 4x**2**^{1}) **b** 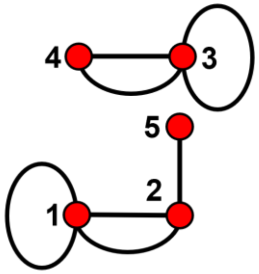	(2x**1**, 2x**2**, 4x**2**^{1}) **c** 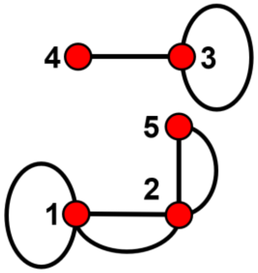	(2x**1**, 2x**2**, 4x**2**^{1}) **d** 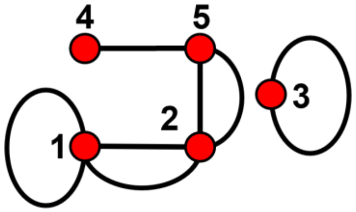
	(2x**1**, 2x**2**, 2x**2**^{1}, 2x**2**^{2}) **a** 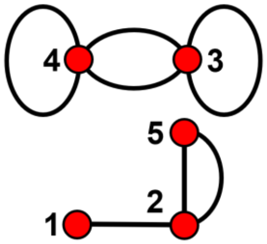	(2x**1**, 2x**2**, 2x**2**^{1}, 2x**2**^{2}) **b** 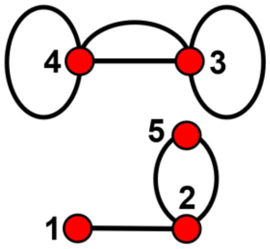	(2x**1**, 2x**2**, 2x**2**^{1}, 2x**2**^{2}) **c** 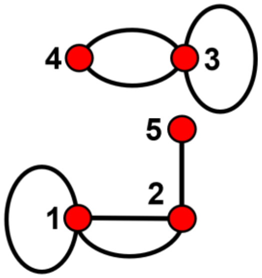	(2x**1**, 2x**2**, 2x**2**^{1}, 2x**2**^{2}) **d** 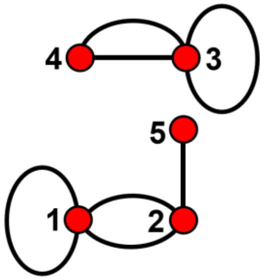

	(2x**1**, 2x**2**, 2x$\mathbf{2}^1$, 2x$\mathbf{2}^2$) **e** 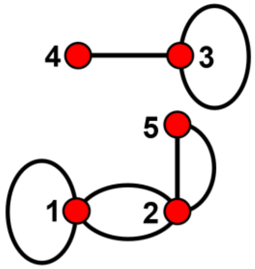	(2x**1**, 2x**2**, 2x$\mathbf{2}^1$, 2x$\mathbf{2}^2$) **f** 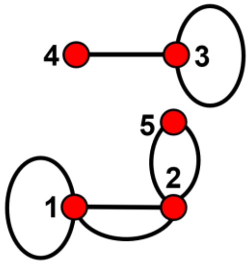	(2x**1**, 2x**2**, 2x$\mathbf{2}^1$, 2x$\mathbf{2}^2$) **g** 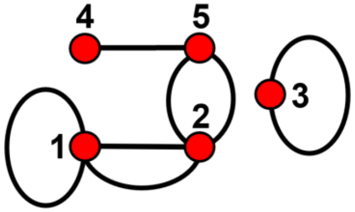	(2x**1**, 2x**2**, 2x$\mathbf{2}^1$, 2x$\mathbf{2}^2$) **h** 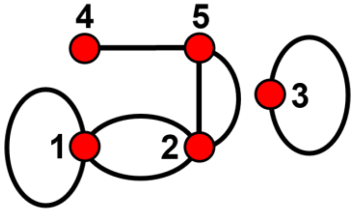
	(2x**1**, 2x**2**, 4x$\mathbf{2}^2$) **a** 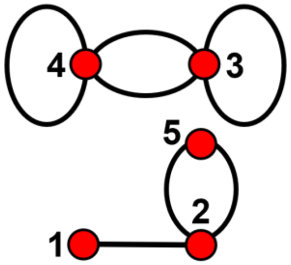	(2x**1**, 2x**2**, 4x$\mathbf{2}^2$) **b** 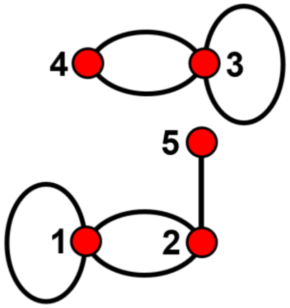	(2x**1**, 2x**2**, 4x$\mathbf{2}^2$) **c** 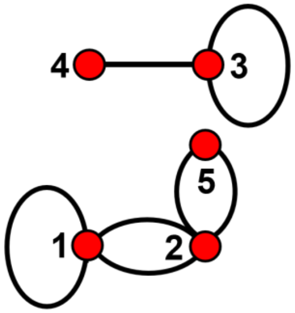	(2x**1**, 2x**2**, 4x$\mathbf{2}^2$) **d** 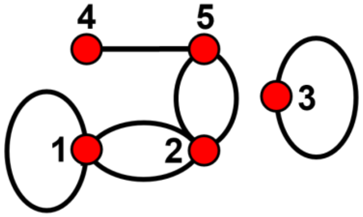
	(2x**1**, 6x$\mathbf{2}^1$) 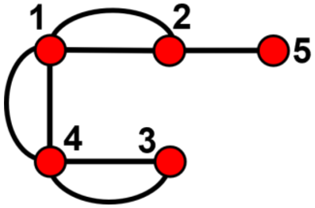	(2x**1**, 4x$\mathbf{2}^1$, 2x$\mathbf{2}^2$) **a** 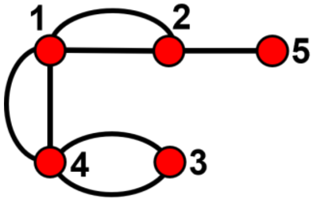	(2x**1**, 4x$\mathbf{2}^1$, 2x$\mathbf{2}^2$) **b** 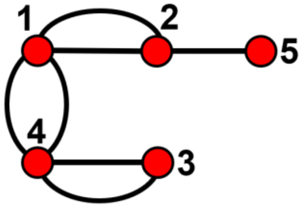	(2x**1**, 4x$\mathbf{2}^1$, 2x$\mathbf{2}^2$) **c** 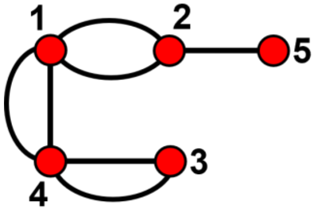

	(2x**1**, 2x$\mathbf{2}^1$, 4x$\mathbf{2}^2$) **a** 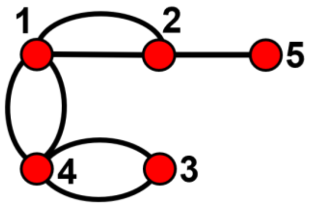	(2x**1**, 2x$\mathbf{2}^1$, 4x$\mathbf{2}^2$) **b** 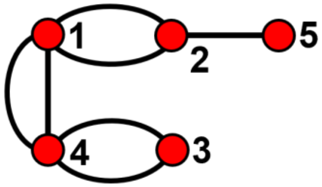	(2x**1**, 2x$\mathbf{2}^1$, 4x$\mathbf{2}^2$) **c** 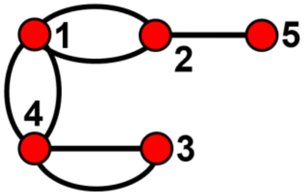	(2x**1**, 6x$\mathbf{2}^2$) 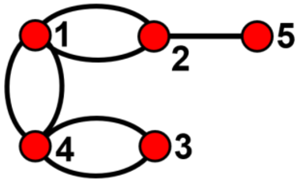
$^1V_1{}^2V_1{}^3V_1{}^4V_3$	NG			
$^1V_1{}^2V_1{}^3V_1{}^4V_4$	NG			
$^1V_1{}^2V_1{}^3V_1{}^4V_5$	NG			
$^1V_1{}^2V_1{}^3V_3{}^4V_1$	NG			
$^1V_1{}^2V_1{}^3V_3{}^4V_2$	NG			
$^1V_1{}^2V_1{}^3V_3{}^4V_3$	NG			
$^1V_1{}^2V_1{}^3V_5{}^4V_1$	NG			
$^1V_1{}^2V_2{}^3V_1{}^4V_1$	(12x**1**) 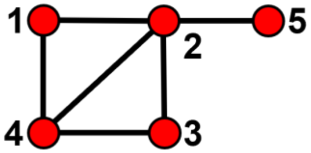	(10x**1**, 1x**2**) **a** 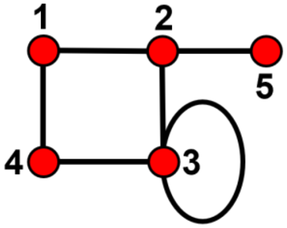	(10x**1**, 1x**2**) **b** 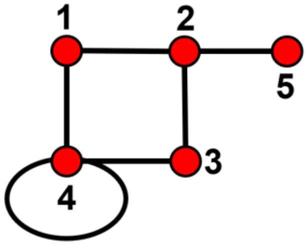	(10x**1**, 1x**2**) **c** 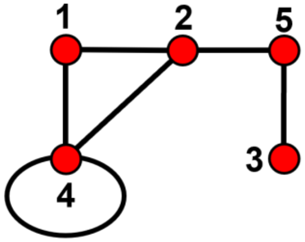

	(10x**1**, 1x**2**) **d**<br>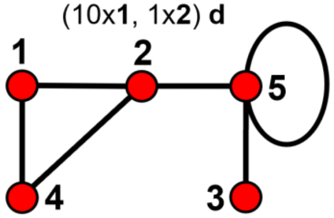	(10x**1**, 1x**2**) **e**<br>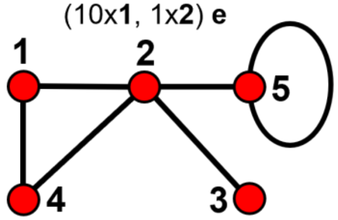	(8x**1**, 2x**2**) **a**<br>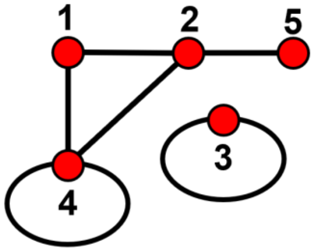	(8x**1**, 2x**2**) **b**<br>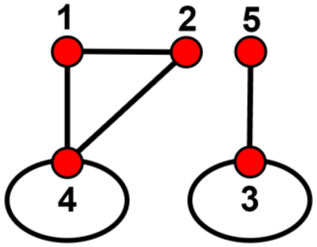
	(8x**1**, 2x**2**) **c**<br>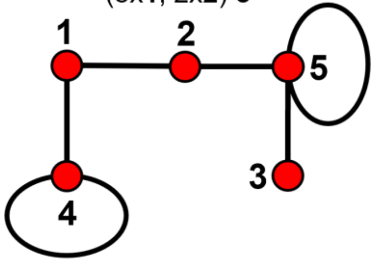	(8x**1**, 2x**2**) **d**<br>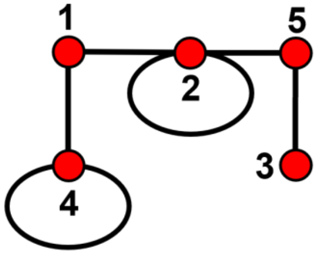	(8x**1**, 2x**2**) **e**<br>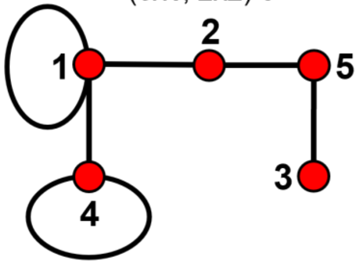	(8x**1**, 2x$\mathbf{2}^1$) **a**<br>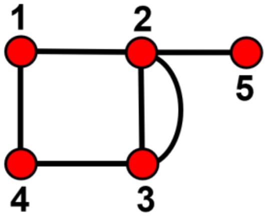
	(8x**1**, 2x$\mathbf{2}^1$) **b**<br>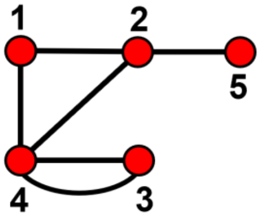	(8x**1**, 2x$\mathbf{2}^1$) **c**<br>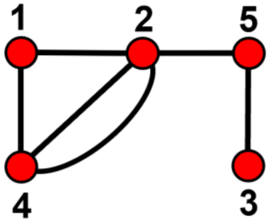	(8x**1**, 2x$\mathbf{2}^1$) **d**<br>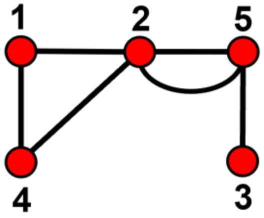	(8x**1**, 2x$\mathbf{2}^2$) **a**<br>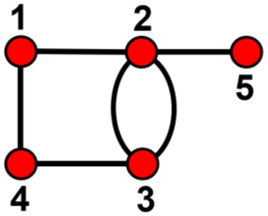

	(8x**1**, 2x$\mathbf{2}^2$) **b** 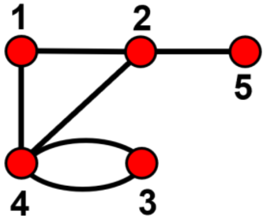	(8x**1**, 2x$\mathbf{2}^2$) **c** 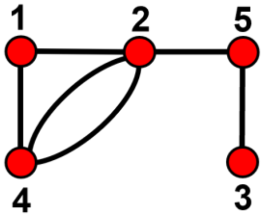	(8x**1**, 2x$\mathbf{2}^2$) **d** 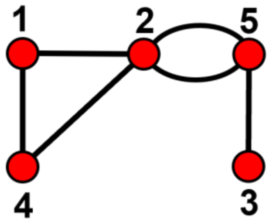	(6x**1**, 3x**2**) **a** 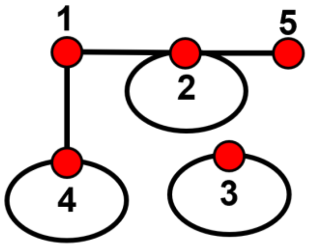
	(6x**1**, 3x**2**) **b** 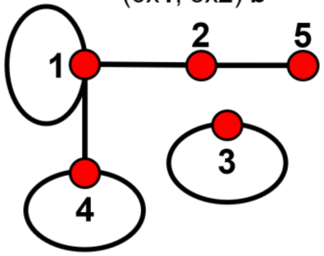	(6x**1**, 1x**2**, 2x$\mathbf{2}^1$) **a** 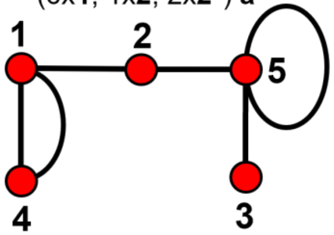	(6x**1**, 1x**2**, 2x$\mathbf{2}^1$) **b** 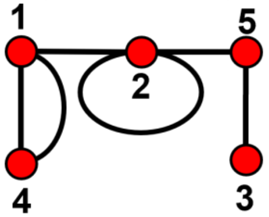	(6x**1**, 1x**2**, 2x$\mathbf{2}^1$) **c** 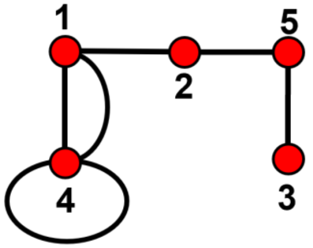
	(6x**1**, 1x**2**, 2x$\mathbf{2}^1$) **d** 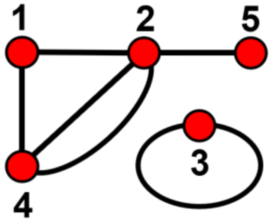	(6x**1**, 1x**2**, 2x$\mathbf{2}^1$) **e** 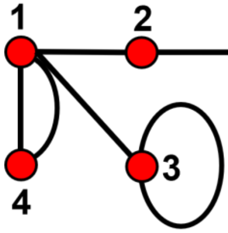	(6x**1**, 1x**2**, 2x$\mathbf{2}^1$) **f** 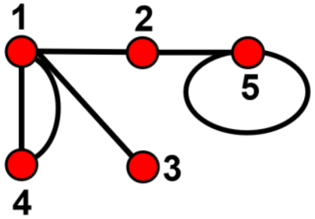	(6x**1**, 1x**2**, 2x$\mathbf{2}^2$) **a** 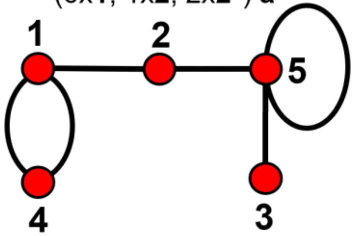
	(6x**1**, 1x**2**, 2x$\mathbf{2}^2$) **b** 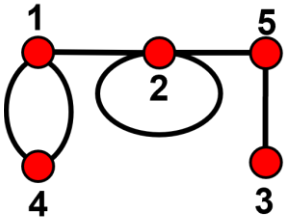	(6x**1**, 1x**2**, 2x$\mathbf{2}^2$) **c** 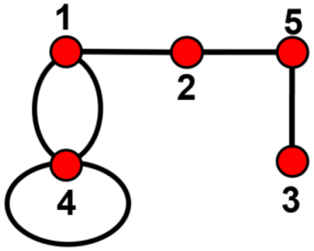	(6x**1**, 1x**2**, 2x$\mathbf{2}^2$) **d** 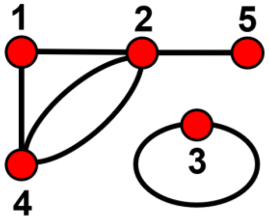	(6x**1**, 1x**2**, 2x$\mathbf{2}^2$) **e** 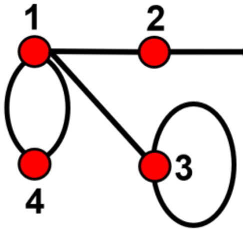

	(6x**1**, 1x**2**, 2x**2**^{2}) **f** 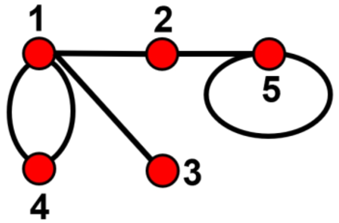	(4x**1**, 4x**2**) 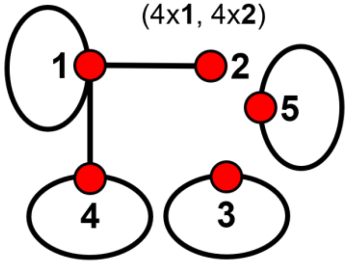	(4x**1**, 2x**2**, 2x**2**^{1}) **a** 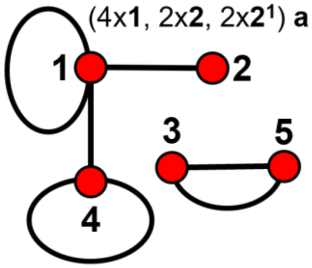	(4x**1**, 2x**2**, 2x**2**^{1}) **b** 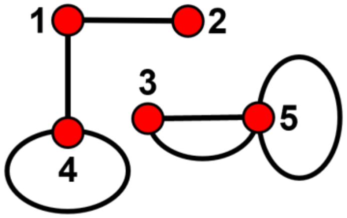
	(4x**1**, 2x**2**, 2x**2**^{1}) **c** 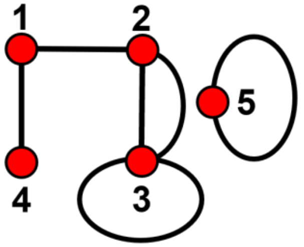	(4x**1**, 2x**2**, 2x**2**^{1}) **d** 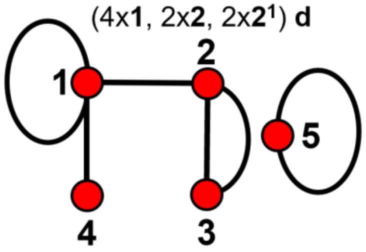	(4x**1**, 2x**2**, 2x**2**^{1}) **e** 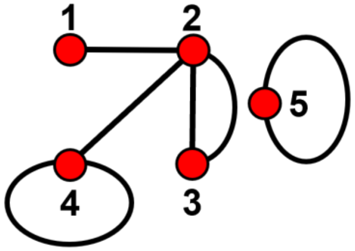	(4x**1**, 2x**2**, 2x**2**^{2}) **a** 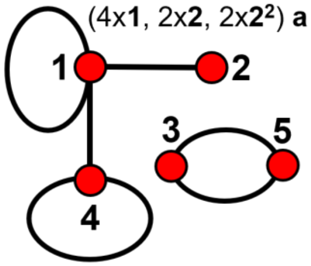
	(4x**1**, 2x**2**, 2x**2**^{2}) **b** 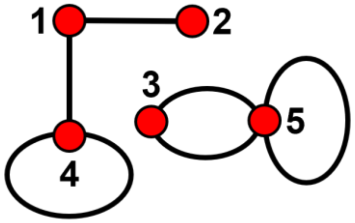	(4x**1**, 2x**2**, 2x**2**^{2}) **c** 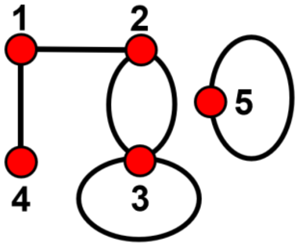	(4x**1**, 2x**2**, 2x**2**^{2}) **d** 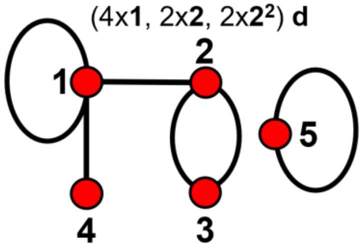	(4x**1**, 2x**2**, 2x**2**^{2}) **e** 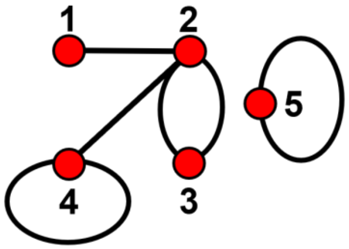
	(4x**1**, 4x**2**^{1}) **a** 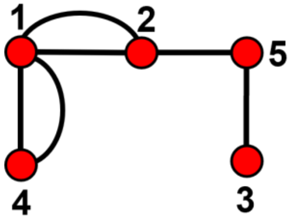	(4x**1**, 4x**2**^{1}) **b** 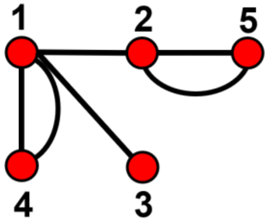	(4x**1**, 2x**2**^{1}, 2x**2**^{2}) **a** 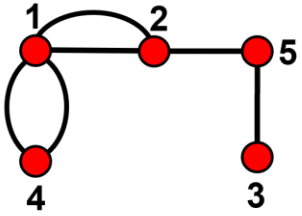	(4x**1**, 2x**2**^{1}, 2x**2**^{2}) **b** 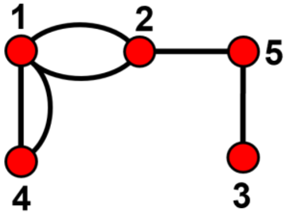

	(4x**1**, 2x$\mathbf{2}^{1}$, 2x$\mathbf{2}^{2}$) **c**	(4x**1**, 2x$\mathbf{2}^{1}$, 2x$\mathbf{2}^{2}$) **d**	(4x**1**, 4x$\mathbf{2}^{2}$) **a**	(4x**1**, 4x$\mathbf{2}^{2}$) **b**
	(2x**1**, 3x**2**, 2x$\mathbf{2}^{1}$) **a**	(2x**1**, 3x**2**, 2x$\mathbf{2}^{1}$) **b**	(2x**1**, 3x**2**, 2x$\mathbf{2}^{2}$) **a**	(2x**1**, 3x**2**, 2x$\mathbf{2}^{2}$) **b**
	(2x**1**, 1x**2**, 4x$\mathbf{2}^{1}$) **a**	(2x**1**, 1x**2**, 4x$\mathbf{2}^{1}$) **b**	(2x**1**, 1x**2**, 4x$\mathbf{2}^{1}$) **c**	(2x**1**, 1x**2**, 4x$\mathbf{2}^{1}$) **d**
	(2x**1**, 1x**2**, 2x$\mathbf{2}^{1}$, 2x$\mathbf{2}^{2}$) **a**	(2x**1**, 1x**2**, 2x$\mathbf{2}^{1}$, 2x$\mathbf{2}^{2}$) **b**	(2x**1**, 1x**2**, 2x$\mathbf{2}^{1}$, 2x$\mathbf{2}^{2}$) **c**	(2x**1**, 1x**2**, 2x$\mathbf{2}^{1}$, 2x$\mathbf{2}^{2}$) **d**

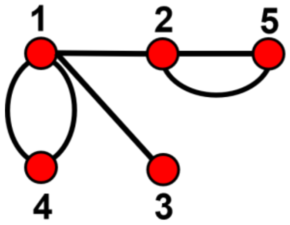

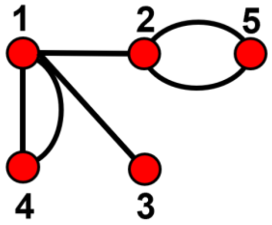

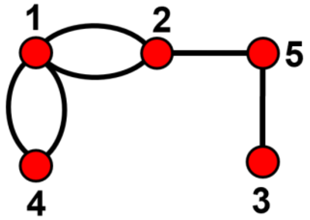

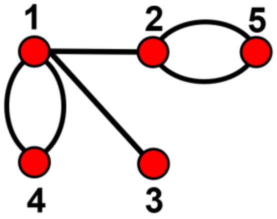

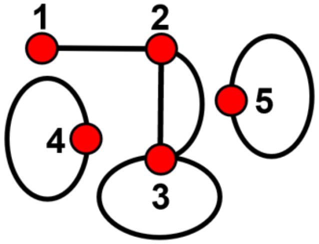

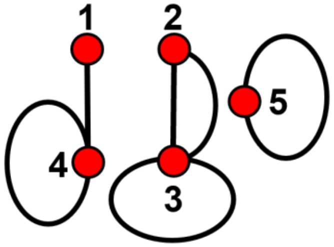

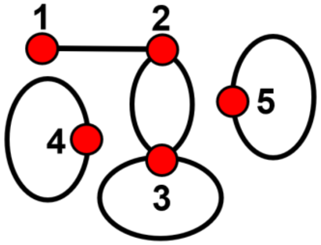

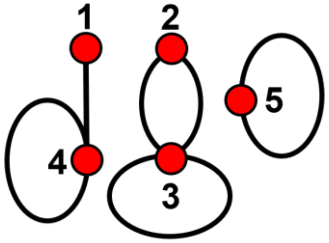

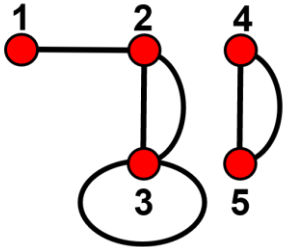

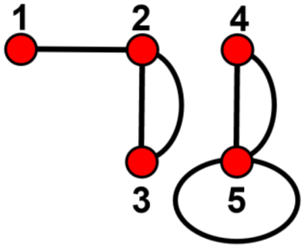

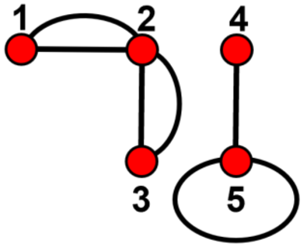

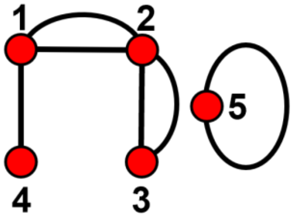

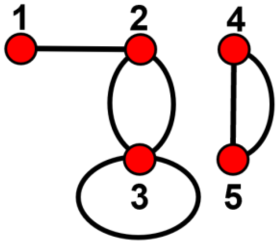

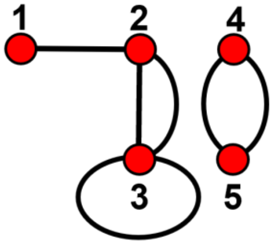

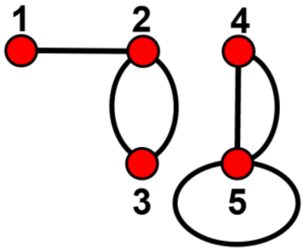

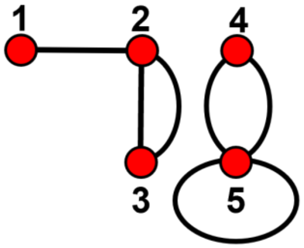

	(2x**1**, 1x**2**, 2x**2**^{1}, 2x**2**^{2}) **e**<br>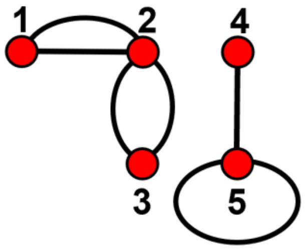	(2x**1**, 1x**2**, 2x**2**^{1}, 2x**2**^{2}) **f**<br>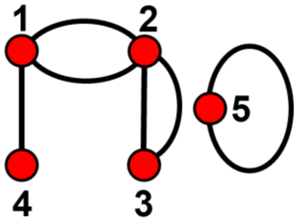	(2x**1**, 1x**2**, 2x**2**^{1}, 2x**2**^{2}) **g**<br>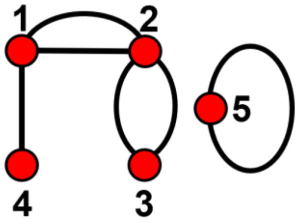	(2x**1**, 1x**2**, 4x**2**^{2}) **a**<br>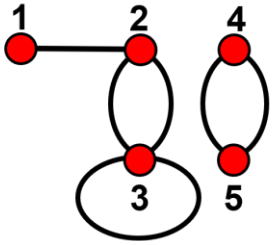
	(2x**1**, 1x**2**, 4x**2**^{2}) **b**<br>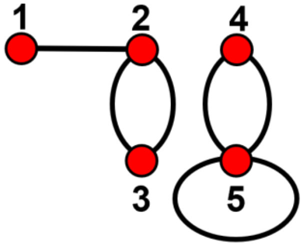	(2x**1**, 1x**2**, 4x**2**^{2}) **c**<br>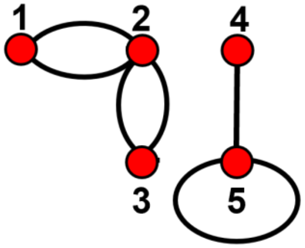	(2x**1**, 1x**2**, 4x**2**^{2}) **d**<br>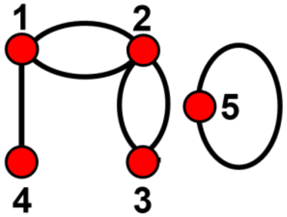	
$^{1}V_1{}^{2}V_2{}^{3}V_1{}^{4}V_2$	NG			
$^{1}V_1{}^{2}V_2{}^{3}V_1{}^{4}V_3$	NG			
$^{1}V_1{}^{2}V_2{}^{3}V_1{}^{4}V_4$	NG			
$^{1}V_1{}^{2}V_2{}^{3}V_3{}^{4}V_1$	NG			
$^{1}V_1{}^{2}V_2{}^{3}V_3{}^{4}V_2$	NG			
$^{1}V_1{}^{2}V_3{}^{3}V_1{}^{4}V_1$	NG			
$^{1}V_1{}^{2}V_3{}^{3}V_1{}^{4}V_2$	NG			
$^{1}V_1{}^{2}V_3{}^{3}V_1{}^{4}V_3$	NG			
$^{1}V_1{}^{2}V_3{}^{3}V_3{}^{4}V_1$	NG			
$^{1}V_1{}^{2}V_4{}^{3}V_1{}^{4}V_1$	NG			
$^{1}V_1{}^{2}V_4{}^{3}V_1{}^{4}V_2$	NG			

$^1V_1{}^2V_5{}^3V_1{}^4V_1$	NG			
$^1V_2{}^2V_1{}^3V_2{}^4V_1$	NG			
$^1V_2{}^2V_1{}^3V_2{}^4V_2$	NG			
$^1V_2{}^2V_1{}^3V_2{}^4V_3$	NG			
$^1V_2{}^2V_1{}^3V_4{}^4V_1$	NG			
$^1V_2{}^2V_2{}^3V_2{}^4V_1$	NG			
$^1V_2{}^2V_2{}^3V_2{}^4V_2$	NG			
$^1V_2{}^2V_3{}^3V_2{}^4V_1$	NG			
$^1V_3{}^2V_1{}^3V_1{}^4V_1$	NG			
$^1V_3{}^2V_1{}^3V_1{}^4V_2$	NG			
$^1V_3{}^2V_1{}^3V_1{}^4V_3$	NG			
$^1V_3{}^2V_1{}^3V_3{}^4V_1$	NG			
$^1V_3{}^2V_2{}^3V_1{}^4V_1$	NG			
$^1V_3{}^2V_2{}^3V_1{}^4V_2$	NG			
$^1V_3{}^2V_3{}^3V_1{}^4V_1$	NG			
$^1V_4{}^2V_1{}^3V_2{}^4V_1$	NG			
$^1V_5{}^2V_1{}^3V_1{}^4V_1$	NG			
Vertex connectivities (^cV_r) where e_A is odd cannot form graphs and are not listed.<br><br>NG = chain graphs not generated.				
