# Supplementary material for: Bond topology of chain, ribbon and tube silicates. Part I. Graph-theory generation of infinite one-dimensional arrangements of (TO4) n− tetrahedra
Source: Acta Crystallogr A Found Adv. 2022 Apr 4;78(Pt 3):212–33. doi: 10.1107/S2053273322001747 (PMC9062827; doi:10.1107/S2053273322001747)
Supplement: Supplementary file 6 [file a-78-00212-sup6.pdf]

FOUNDATIONS
ADVANCES

Volume 78 (2022)

Supporting information for article:

Bond topology of chain, ribbon and tube silicates. Part I. Graph-theory generation of infinite one-dimensional arrangements of $(TO4)^{n-}$ tetrahedra

Maxwell Christopher Day and Frank Christopher Hawthorne

Appendix G. Matrix element combinations and associated non-isomorphic chain graphs for vertex connectivities (${}^{c}V_{r}$) where $\sum r \leq 8$.

**${}^{c}V_{r}$**		
**Rank 1**		
${}^{0}V_{1\text{-}8}$	isolated tetrahedra (nesosilicates)	
${}^{2}V_{1}$	(1x**2**) 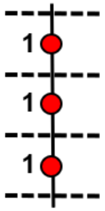	
${}^{2}V_{2}$	(2x**2**) 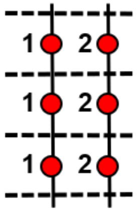	
${}^{2}V_{3}$	(3x**2**) 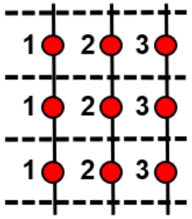	

$^{2}V_{4}$	(6x**1**, 1x**2**) 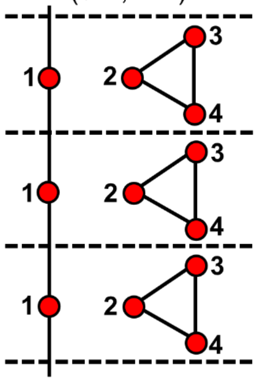	(4x**2**) 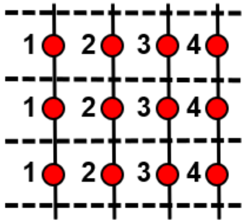	
$^{2}V_{5}$	(8x**1**, 1x**2**) 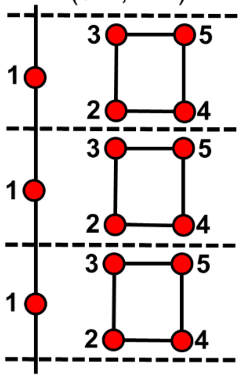	(6x**1**, 2x**2**) 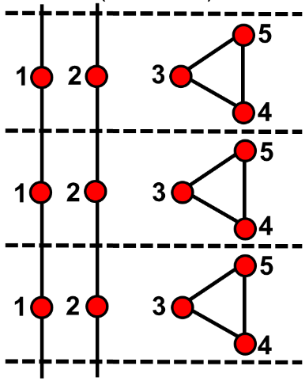	(5x**2**) 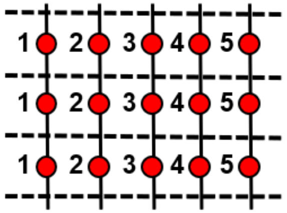

$^{2}V_{6}$	(10x**1**, 1x**2**) 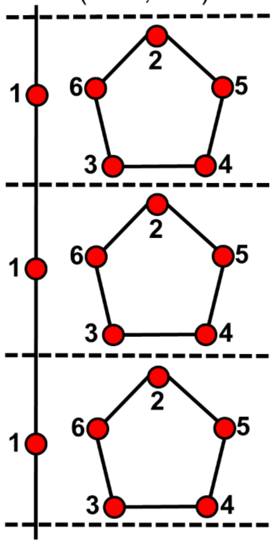	(8x**1**, 2x**2**) 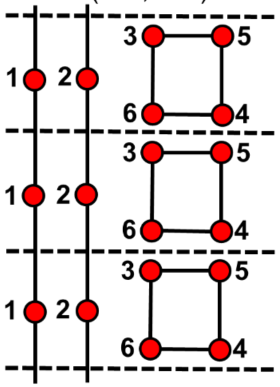	(6x**1**, 3x**2**) 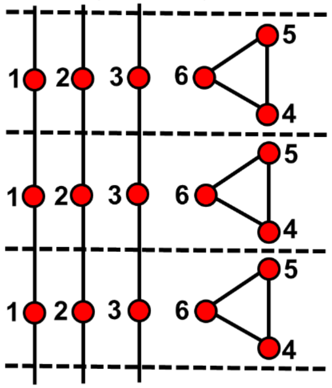
	(6x**2**) 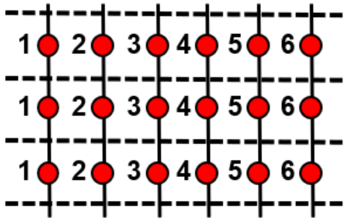		

$^2V_7$	(12x**1**, 1x**2**) **a**<br>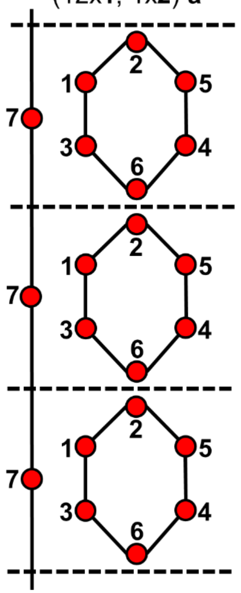	(12x**1**, 1x**2**) **b**<br>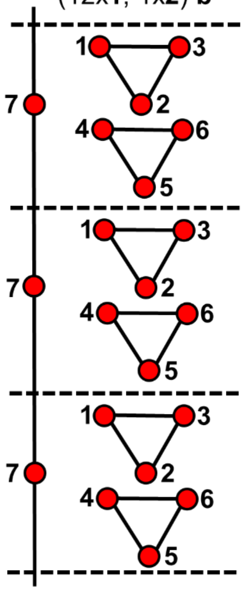	(10x**1**, 2x**2**)<br>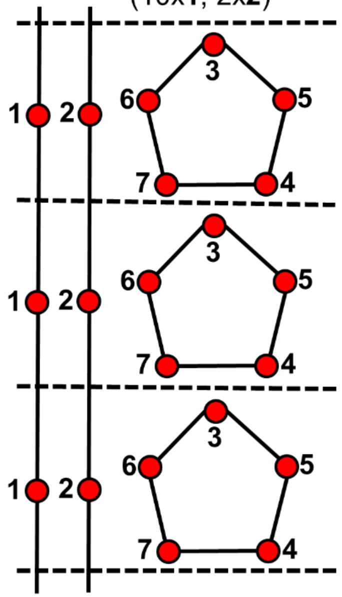
	(8x**1**, 3x**2**)<br>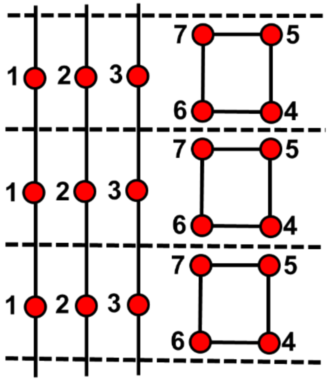	(6x**1**, 4x**2**)<br>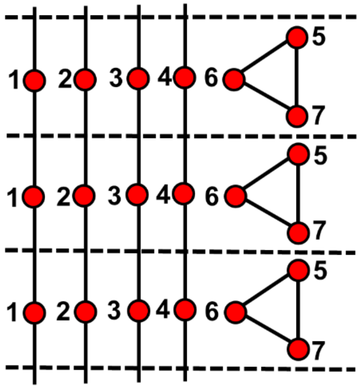	(7x**2**)<br>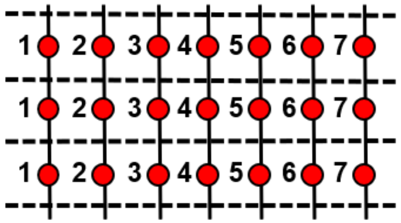

$^2V_8$	(14x**1**, 1x**2**) **a**<br>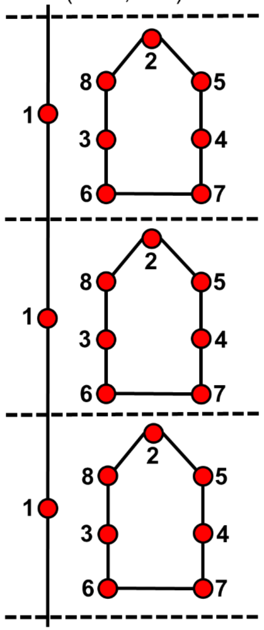	(14x**1**, 1x**2**) **b**<br>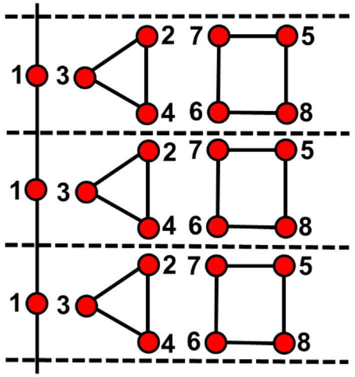	(12x**1**, 2x**2**) **a**<br>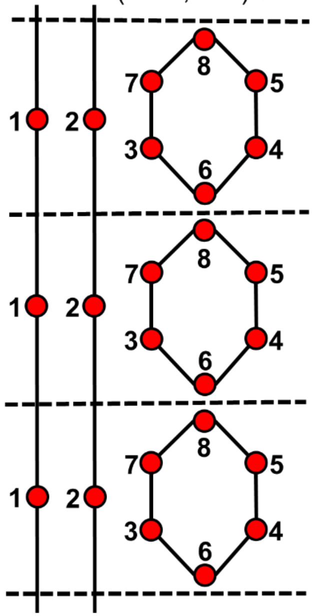

	(12x**1**, 2x**2**) **b**<br>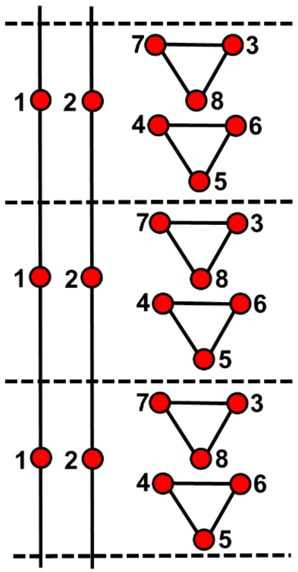	(10x**1**, 3x**2**)<br>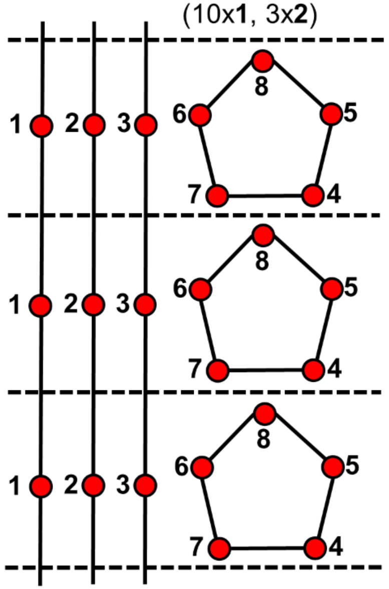	(8x**1**, 4x**2**)<br>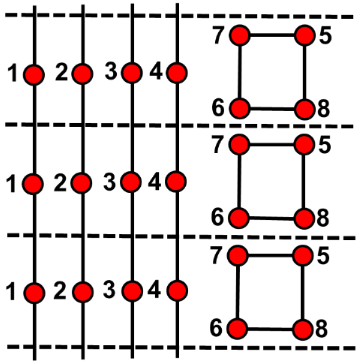
	(6x**1**, 5x**2**)<br>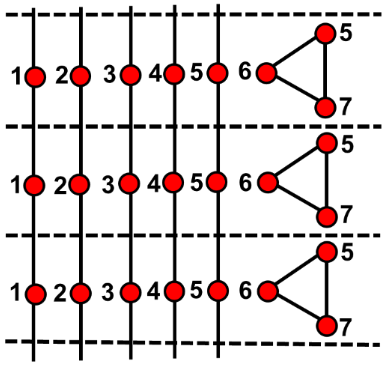	(8x**2**)<br>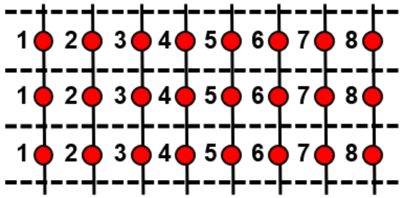	

$^3V_2$	(2x**1**, 2x**2**) 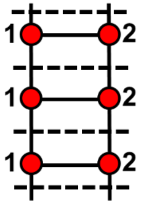		
$^3V_4$	(12x**1**) **a** 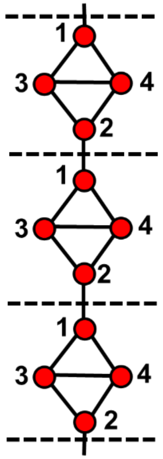	(12x**1**) **b** 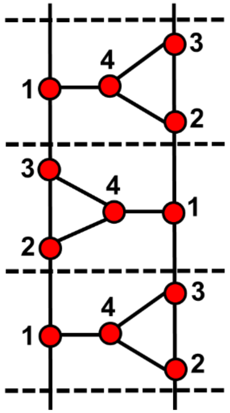	(12x**1**) **c** 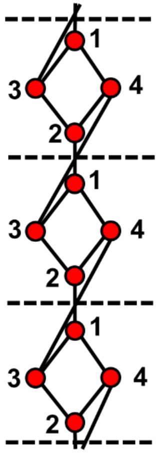

	(12x**1**) **d** 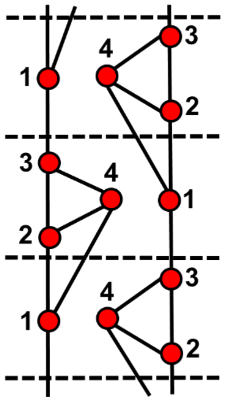	(12x**1**) **e** 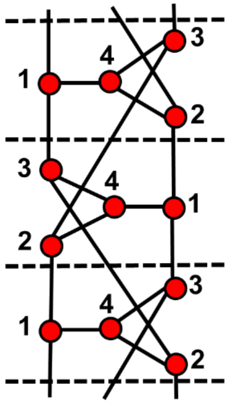	(12x**1**) **f** 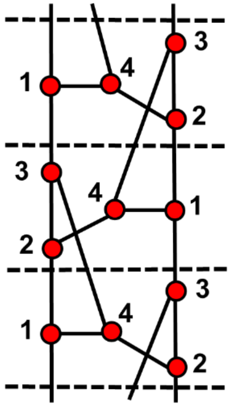
	(12x**1**) **g** 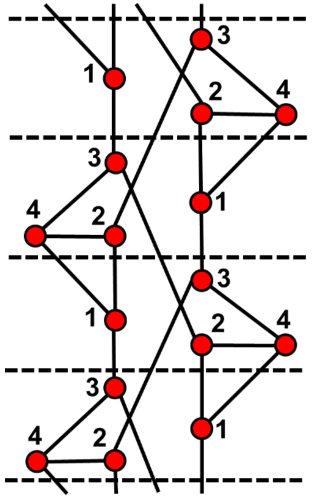	(12x**1**) **h** 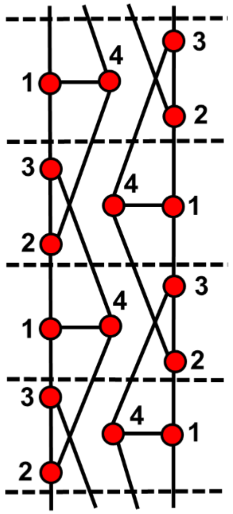	(12x**1**) **i** 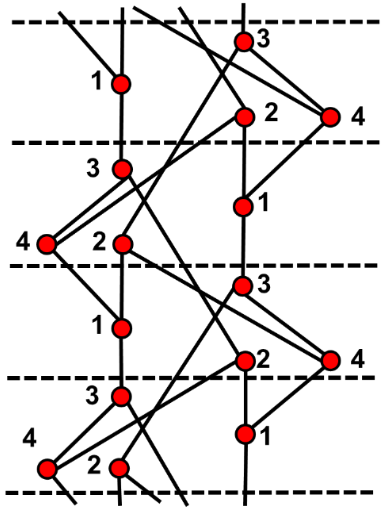

	(12x**1**) **j** 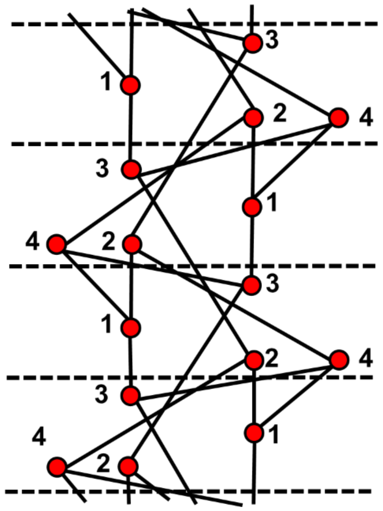	(6x**1**, 3x**2**) 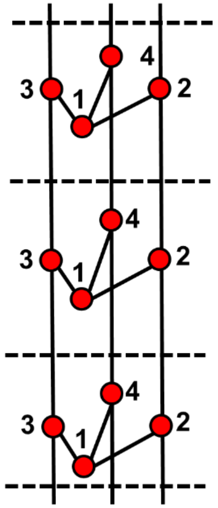	(6x**1**, 1x**2**, 2x**2**^{1}) **a** 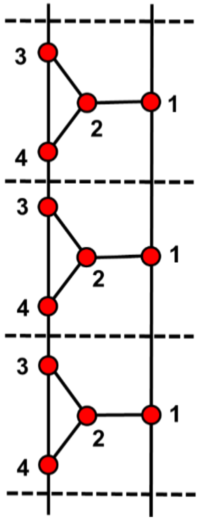

(6x**1**, 1x**2**, 2x**2**^{1}) **b**

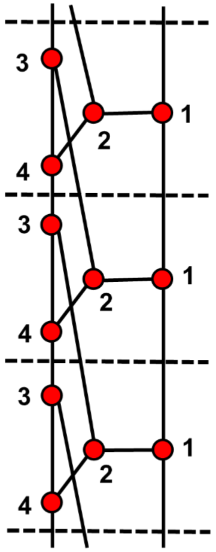

(6x**1**, 1x**2**, 2x**2**^{1}) **c**

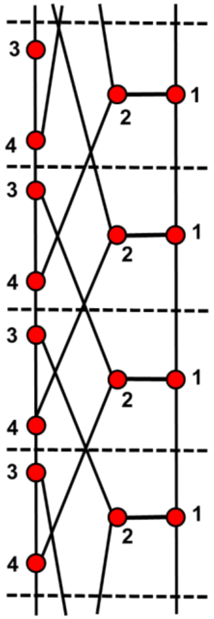

(6x**1**, 1x**2**, 2x**2**^{2}) **a**

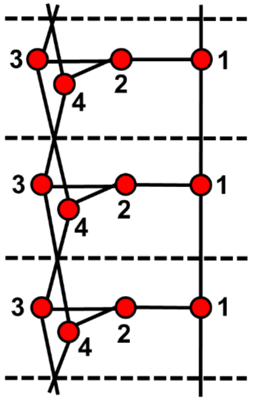

<table>
<tr>
<td></td>
<td>(6x1, 1x2, 2x$\mathbf{2}^{2}$) b
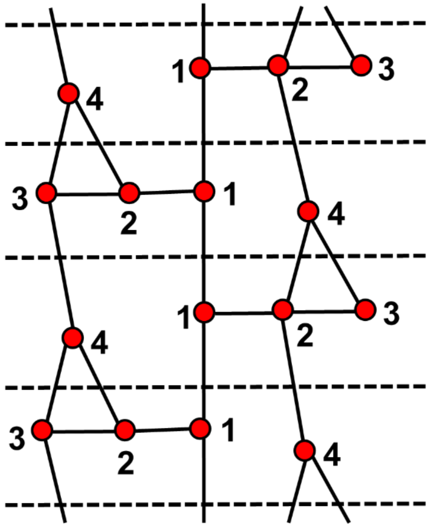
</td>
<td>(6x1, 1x2, 2x$\mathbf{2}^{2}$) c
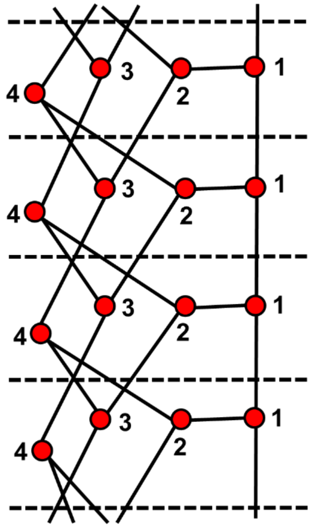
</td>
<td>(4x1, 4x2)
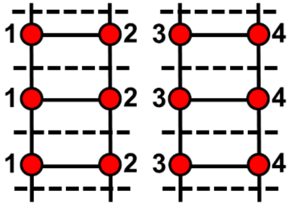
</td>
<td></td>
</tr>
<tr>
<td></td>
<td>(4x1, 2x2, 2x$\mathbf{2}^{1}$)
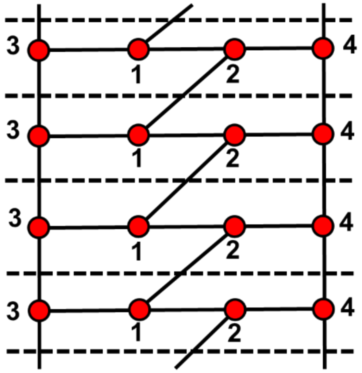
</td>
<td>(4x1, 2x2, 2x$\mathbf{2}^{2}$)
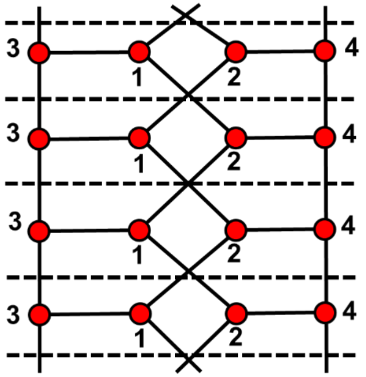
</td>
<td></td>
<td></td>
</tr>
</table>

	(4x**1**, 4x$\mathbf{2}^1$) **a** 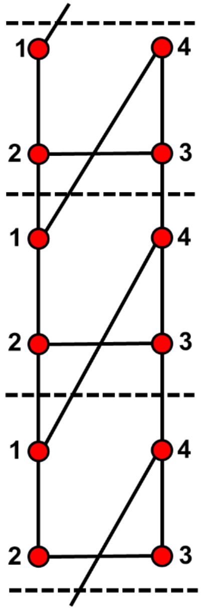	(4x**1**, 4x$\mathbf{2}^1$) **b** 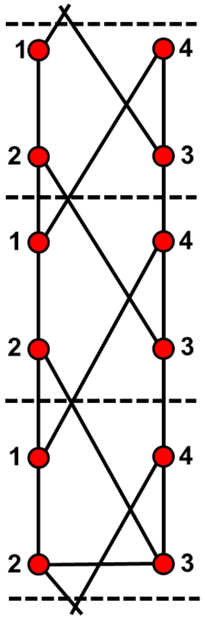	(4x**1**, 2x$\mathbf{2}^1$, 2x$\mathbf{2}^2$) **a** 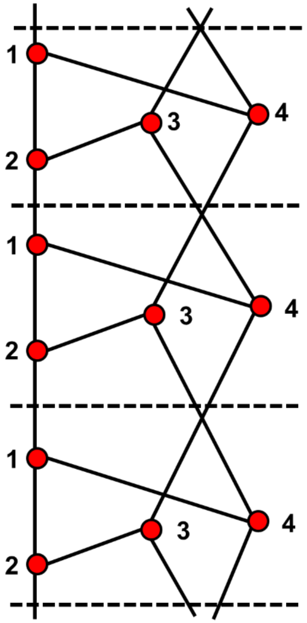	(4x**1**, 2x$\mathbf{2}^1$, 2x$\mathbf{2}^2$) **b** 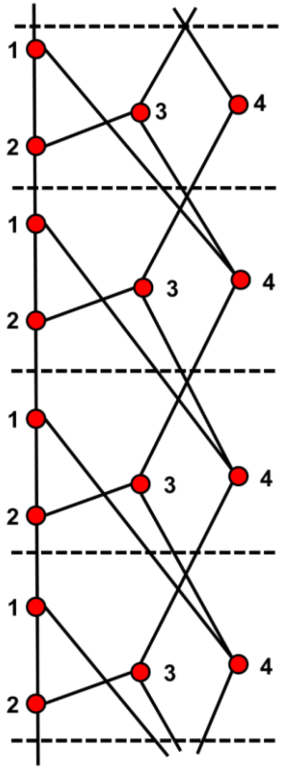

	(4x$\mathbf{1}$, 2x$\mathbf{2}^1$, 2x$\mathbf{2}^2$) c 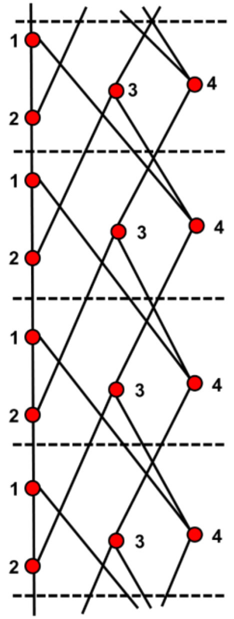	(4x$\mathbf{1}$, 4x$\mathbf{2}^2$) 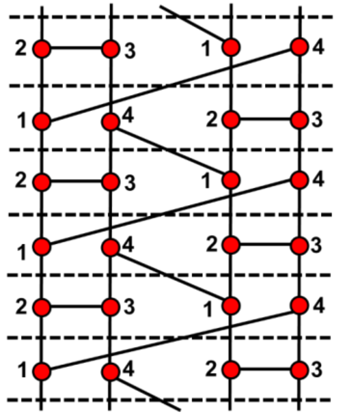		
$^3V_6$	NG			
$^3V_8$	NG			
$^4V_2$	(2x$\mathbf{2}$, 2x$\mathbf{2}^1$) 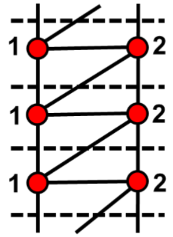	(2x$\mathbf{2}$, 2x$\mathbf{2}^2$) 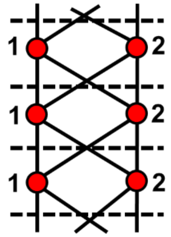		

$^4V_3$	(6x**1**, 3x**2**) **a**<br>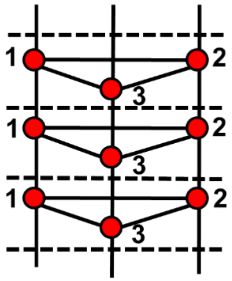	(6x**1**, 3x**2**) **b**<br>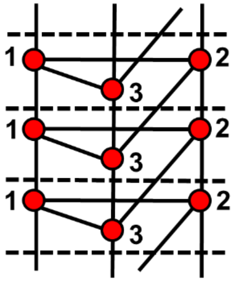	(6x**1**, 3x**2**) **c**<br>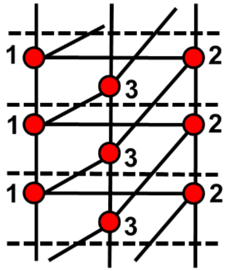	(6x**1**, 3x**2**) **d**<br>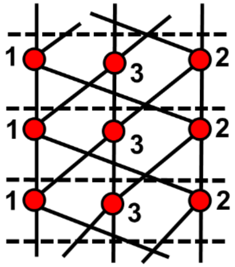
	(2x**2**, 4x**2**^{1})<br>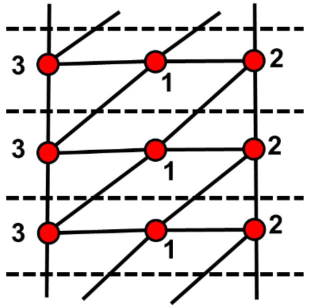	(2x**2**, 2x**2**^{1}, 2x**2**^{2})<br>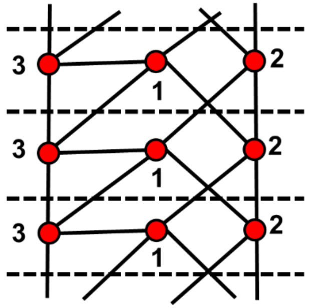	(2x**2**, 4x**2**^{2})<br>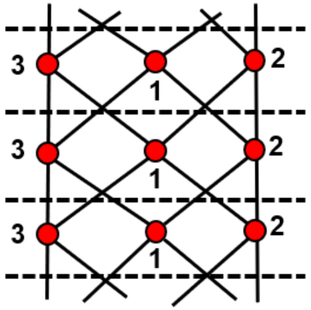	

	(6x**2**¹) **a** 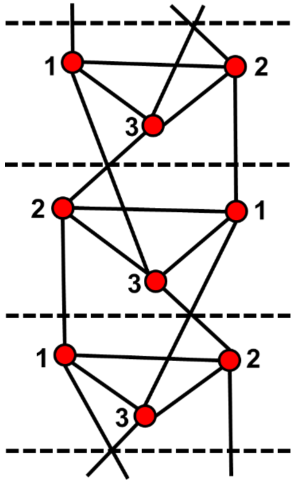	(6x**2**¹) **b** 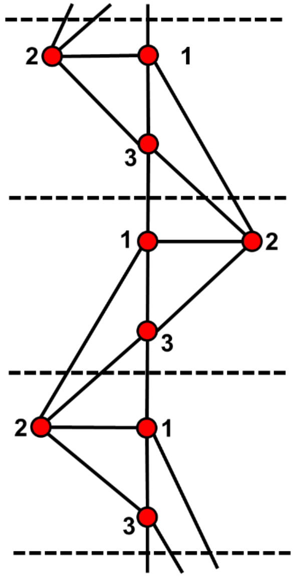	(4x**2**¹, 2x**2**²) **a** 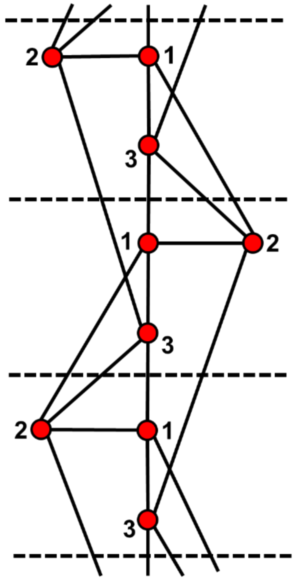	(4x**2**¹, 2x**2**²) **b** 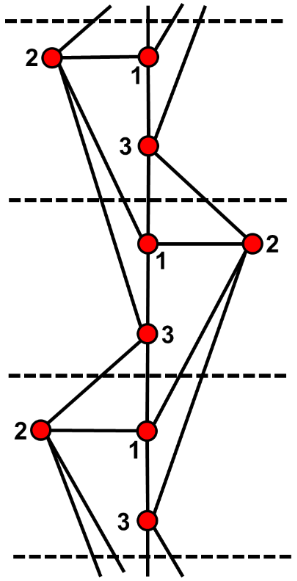

	(2x$\mathbf{2}^1$, 4x$\mathbf{2}^2$)<br>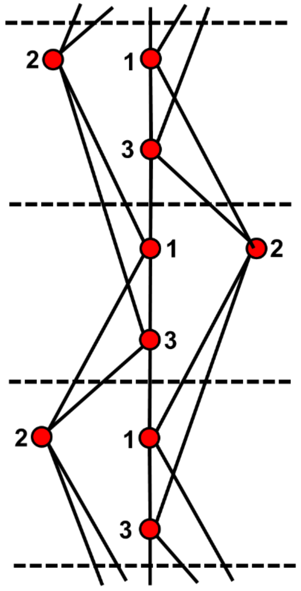	(6x$\mathbf{2}^2$)<br>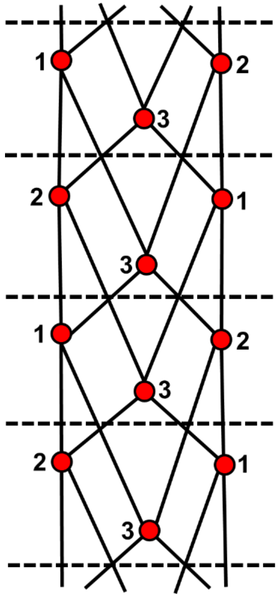		
$^4V_4$	NG			
$^4V_5$	NG			
$^4V_6$	NG			
$^4V_7$	NG			
$^4V_8$	NG			
**Rank 2**				

$^{1}V_{2}{}^{2}V_{1}$	(2x**1**, 1x**2**) 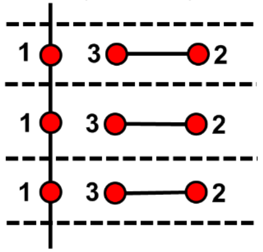			
$^{1}V_{2}{}^{2}V_{2}$	(4x**1**, 1x**2**) 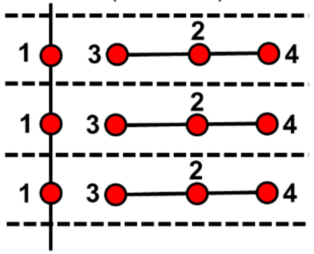	(2x**1**, 2x**2**) 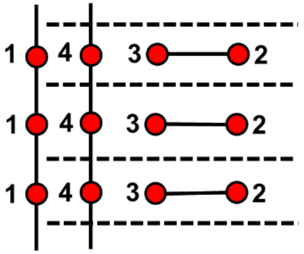		
$^{1}V_{2}{}^{2}V_{3}$	(8x**1**) **b** 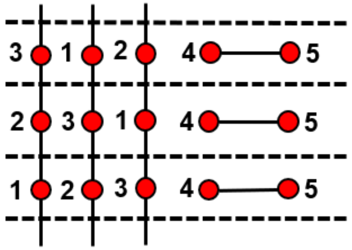	(6x**1**, 1x**2**) 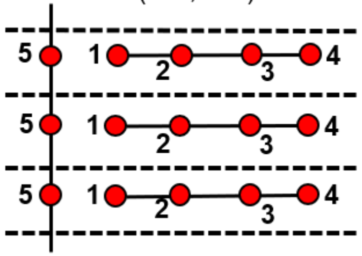	(4x**1**, 2x**2**) 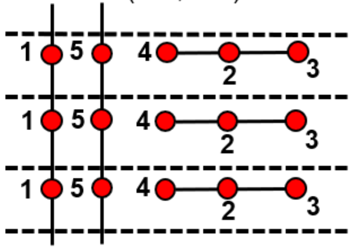	

$^{1}V_{2}{}^{2}V_{4}$	(10x**1**) **b**<br>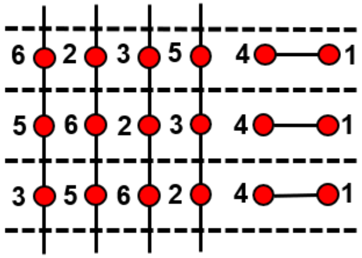	(10x**1**) **c**<br>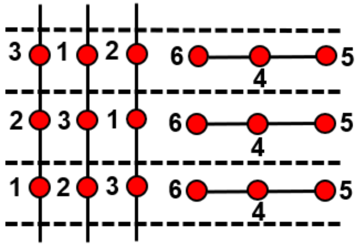	(8x**1**, 1x**2**) **a**<br>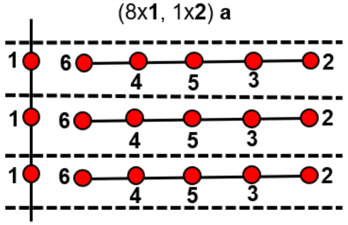	(8x**1**, 1x**2**) **b**<br>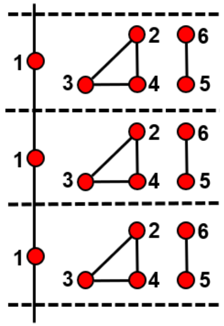
	(6x**1**, 2x**2**^{1})<br>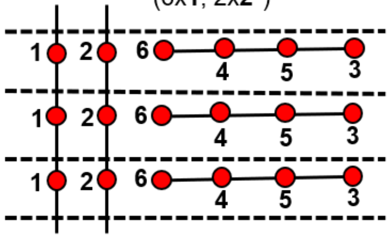			
$^{1}V_{2}{}^{2}V_{5}$	NG			
$^{1}V_{2}{}^{2}V_{6}$	NG			
$^{1}V_{4}{}^{2}V_{1}$	(4x**1**, 1x**2**)<br>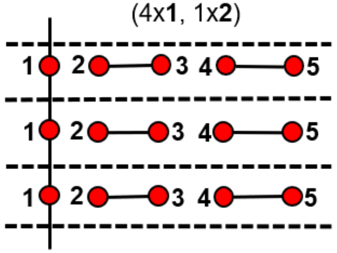			

$^{1}V_{4}{}^{2}V_{2}$	(6x**1**, 1x**2**)<br>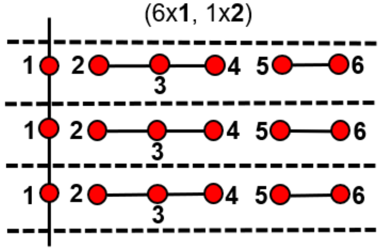	(4x**1**, 2x**2**)<br>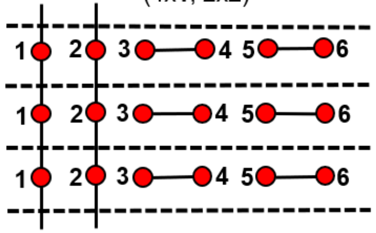		
$^{1}V_{4}{}^{2}V_{3}$	(10x**1**) **a**<br>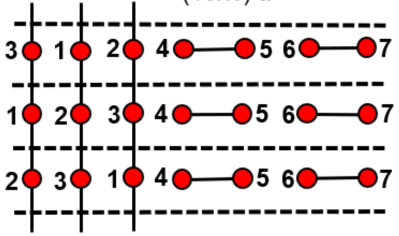	(8x**1**, 1x**2**) **a**<br>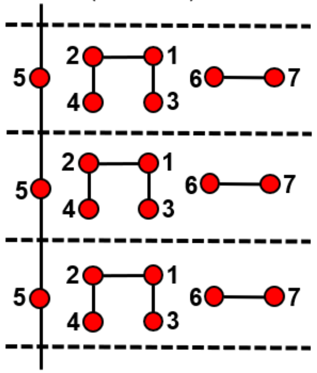	(8x**1**, 1x**2**) **b**<br>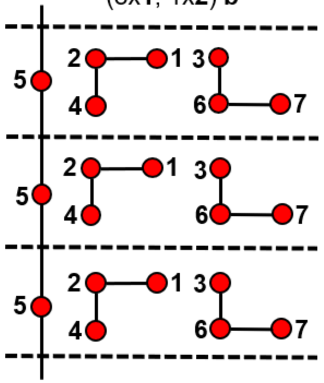	(6x**1**, 2x**2**)<br>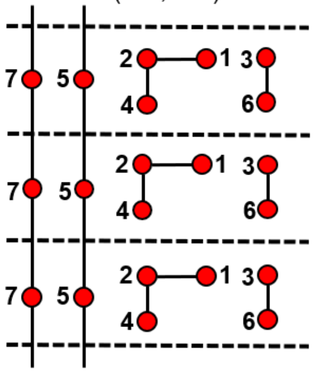
$^{1}V_{4}{}^{2}V_{4}$	(12x**1**) **d**<br>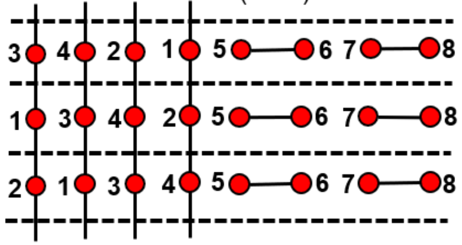	(12x**1**) **e**<br>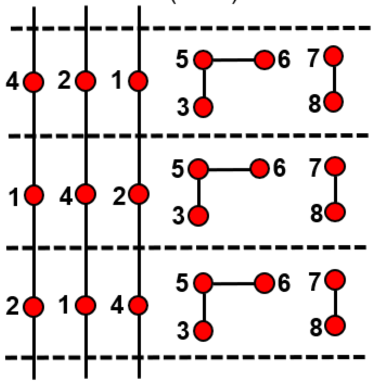	(10x**1**, 1x**2**) **a**<br>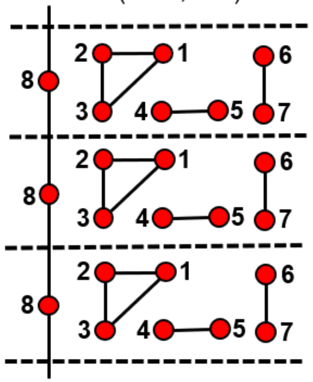	(10x**1**, 1x**2**) **b**<br>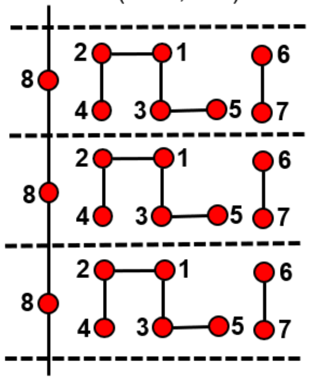

	(10x**1**, 1x**2**) **c** 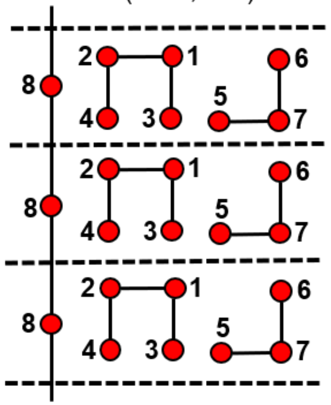	(8x**1**, 2x**2**) **a** 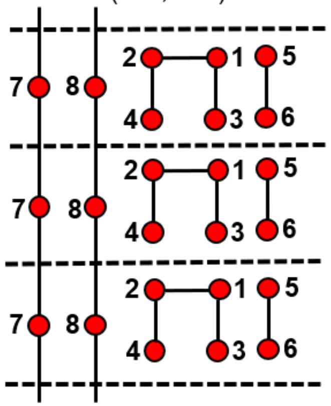	(8x**1**, 2x**2**) **b** 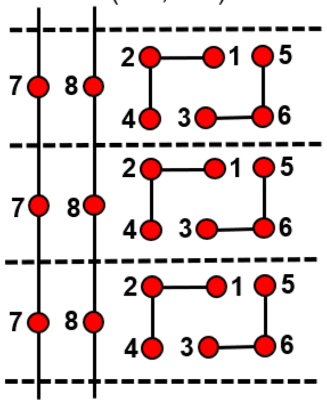	
$^{1}V_{6}{}^{2}V_{1}$	(6x**1**, 1x**2**) 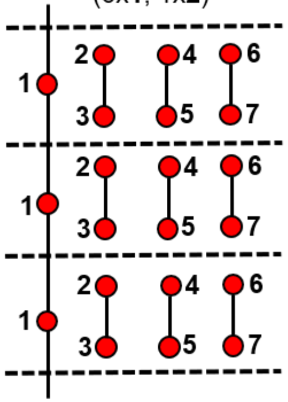			

$^1V_6{}^2V_2$	(8x**1**, 1x**2**) 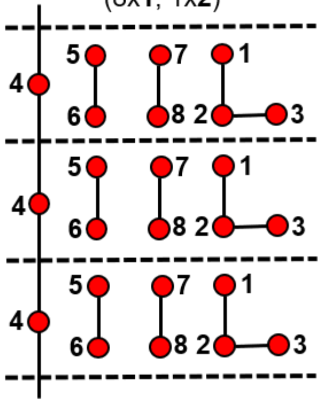	(6x**1**, 2x**2**) 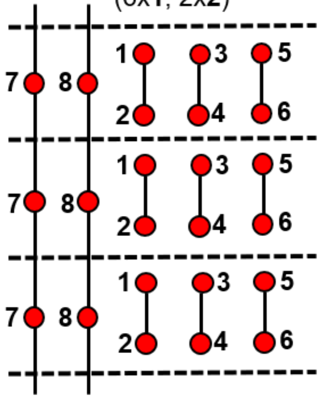		
$\boldsymbol{^1V_r{}^3V_r}$				
$^1V_1{}^3V_1$	(2x**1**, 1x**2**) 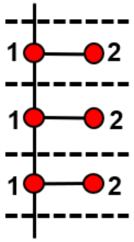			

$^{1}V_{1}{}^{3}V_{3}$	(6x**1**, 2x**2**) 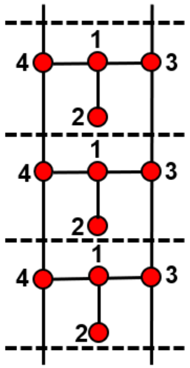	(6x**1**, 2x**2**^{1}) **a** 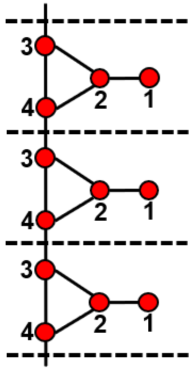	(6x**1**, 2x**2**^{1}) **b** 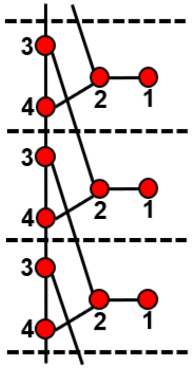	(6x**1**, 2x**2**^{1}) **c** 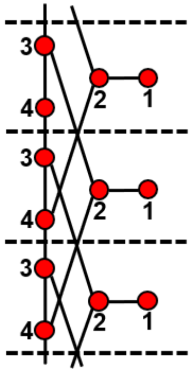
	(6x**1**, 2x**2**^{2}) **a** 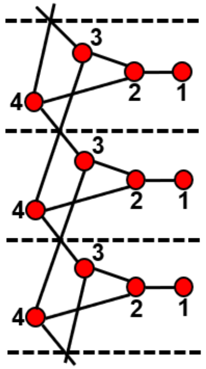	(6x**1**, 2x**2**^{2}) **b** 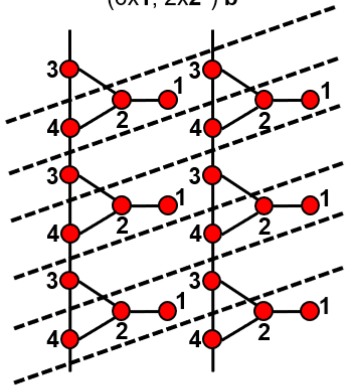	(6x**1**, 2x**2**^{2}) **c** 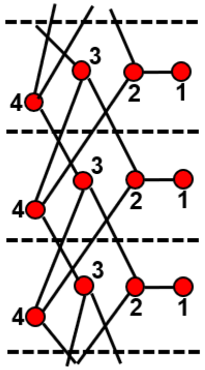	(4x**1**, 3x**2**) 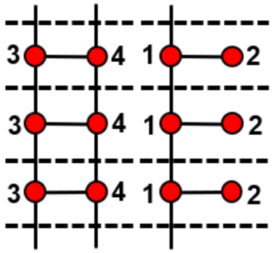

	(4x**1**, 1x**2**, 2x**2**^{1}) 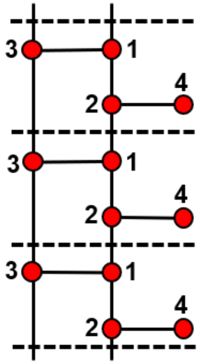	(4x**1**, 1x**2**, 2x**2**^{2}) 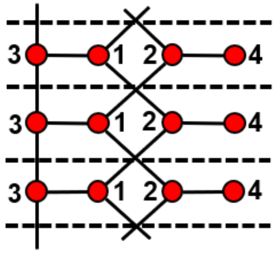		
$^1V_1{}^3V_5$	NG			
$^1V_1{}^3V_7$	NG			
$^1V_2{}^3V_2$	(6x**1**, 1x**2**) 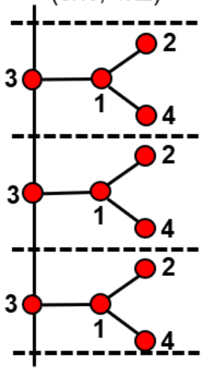	(4x**1**, 2x**2**) **a** 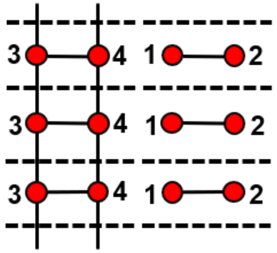	(4x**1**, 2x**2**) **b** 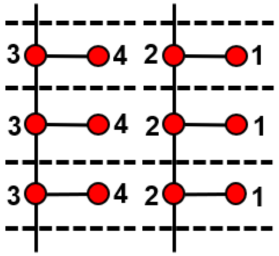	
$^1V_2{}^3V_4$	NG			
$^1V_2{}^3V_6$	NG			

$^1V_3{}^3V_1$	(4x**1**, 1x**2**) 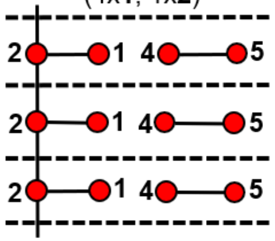			
$^1V_3{}^3V_3$	(10x**1**, 1x**2**) 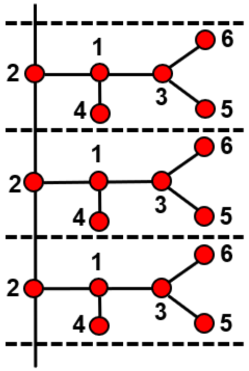	(8x**1**, 2x**2**) **a** 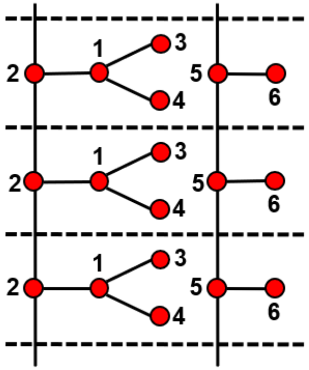	(8x**1**, 2x**2**) **b** 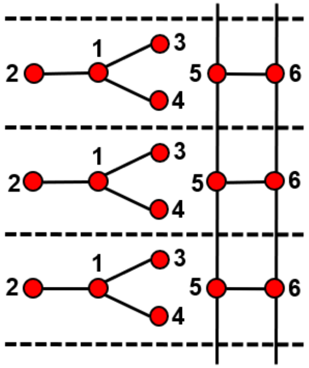	(8x**1**, 2x**2**) **c** 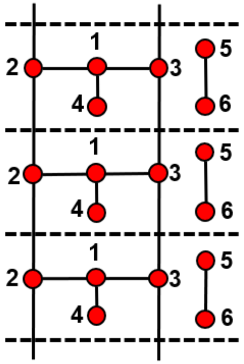
	(8x**1**, 2x**2**^{1}) **a** 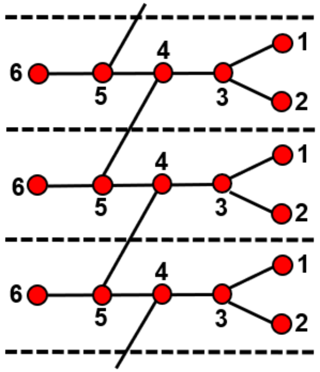	(8x**1**, 2x**2**^{1}) **b-1** 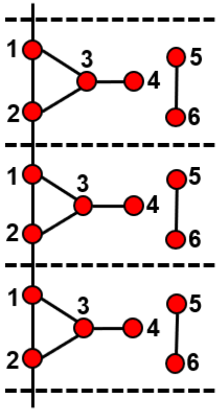	(8x**1**, 2x**2**^{1}) **b-2** 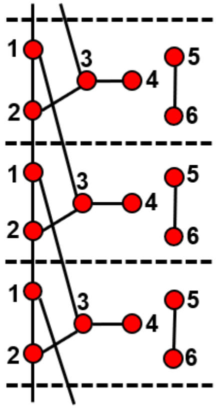	(8x**1**, 2x**2**^{1}) **b-3** 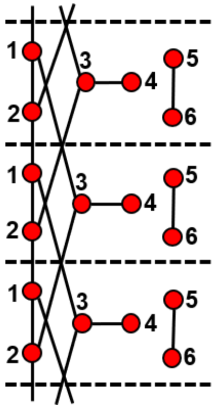

	(8x**1**, 2x**2**^{2}) **a** 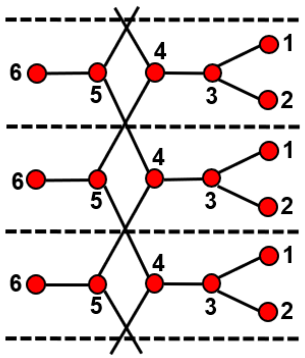	(8x**1**, 2x**2**^{2}) **b-1** 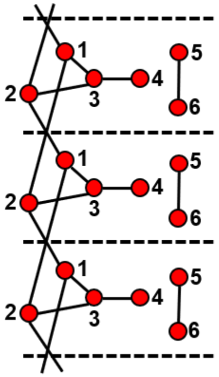	(8x**1**, 2x**2**^{2}) **b-2** 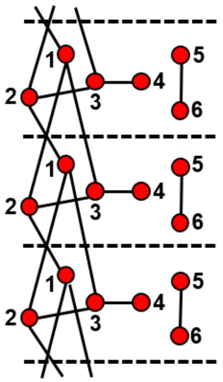	(8x**1**, 2x**2**^{2}) **b-3** 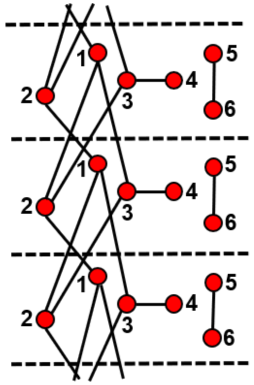
	(6x**1**, 3x**2**) **a** 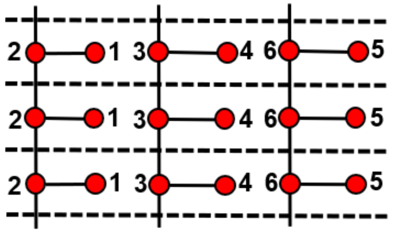	(6x**1**, 3x**2**) **b** 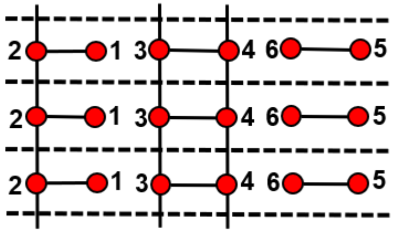		
${}^1V_3{}^3V_5$	NG			
${}^1V_4{}^3V_2$	(8x**1**, 1x**2**) **a** 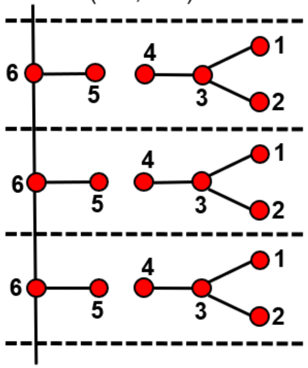	(8x**1**, 1x**2**) **b** 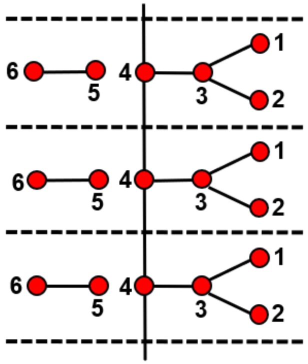	(6x**1**, 2x**2**) **a** 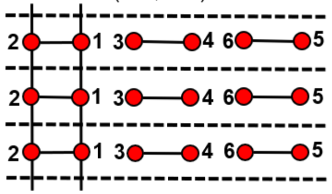	(6x**1**, 2x**2**) **b** 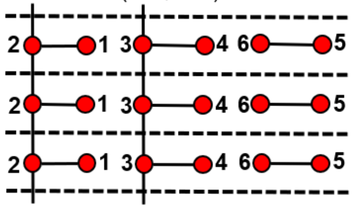

$^1V_4{}^3V_4$	NG			
$^1V_5{}^3V_1$	(6x**1**, 1x**2**) 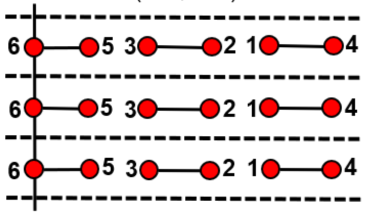			
$^1V_5{}^3V_3$	NG			
$^1V_6{}^3V_2$	NG			
$^1V_7{}^3V_1$	NG			
$\mathbf{^1V_r{}^4V_r}$				
$^1V_2{}^4V_1$	(4x**1**, 1x**2**) 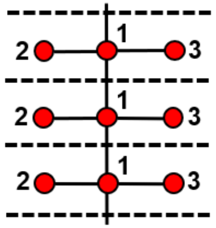			
$^1V_2{}^4V_2$	(6x**1**, 2x**2**) 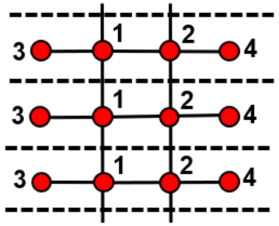	(4x**1**, 1x**2**, 2x**2**^{1}) 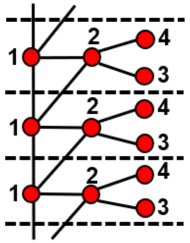	(4x**1**, 1x**2**, 2x**2**^{2}) 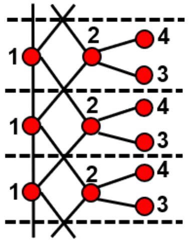	(2x**1**, 2x**2**, 2x**2**^{1}) 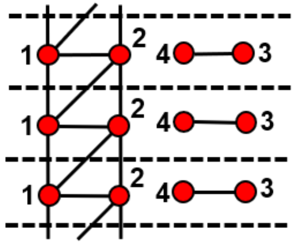

	(2x**1**, 2x**2**, 2x$\mathbf{2}^2$) 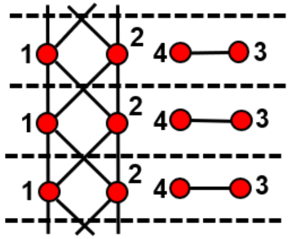			
$^1V_2{}^4V_3$	(10x**1**, 2x**2**) **1** 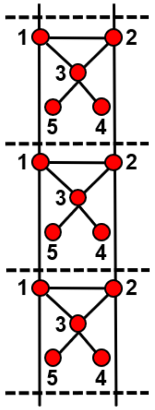	(10x**1**, 2x**2**) **2** 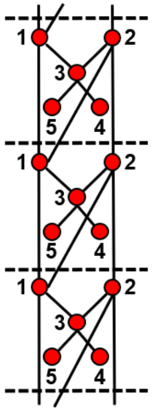	(10x**1**, 2x**2**) **3** 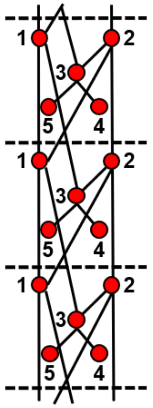	(10x**1**, 2x**2**) **4** 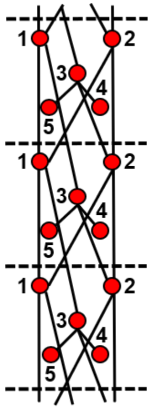
	(8x**1**, 3x**2**) **a-1** 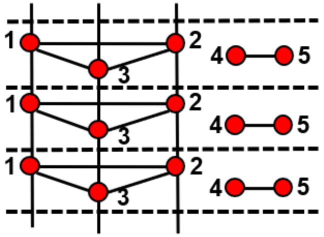	(8x**1**, 3x**2**) **a-2** 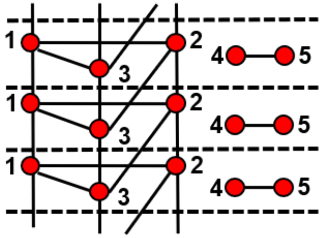	(8x**1**, 3x**2**) **a-3** 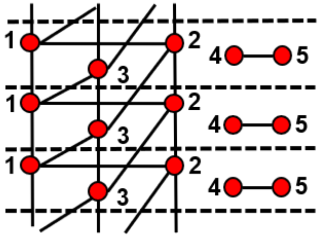	(8x**1**, 3x**2**) **a-4** 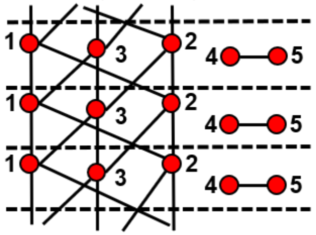

	(8x**1**, 3x**2**) **b**<br>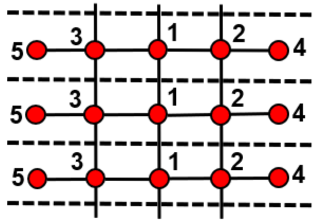	(8x**1**, 1x**2**, 2x**2**^{1}) **1**<br>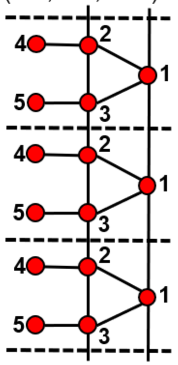	(8x**1**, 1x**2**, 2x**2**^{1}) **2**<br>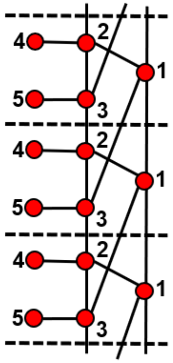	(8x**1**, 1x**2**, 2x**2**^{1}) **3**<br>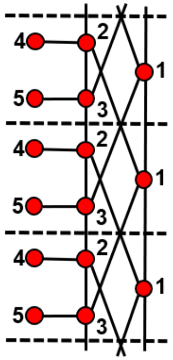
	(8x**1**, 1x**2**, 2x**2**^{2}) **1**<br>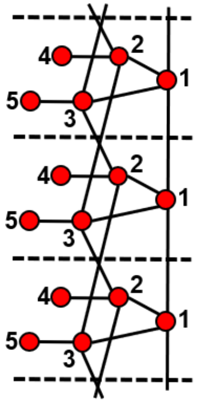	(8x**1**, 1x**2**, 2x**2**^{2}) **2**<br>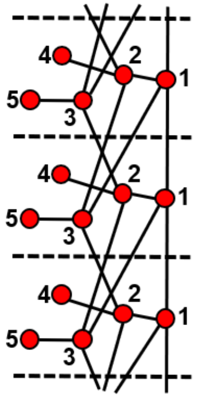	(8x**1**, 1x**2**, 2x**2**^{2}) **3**<br>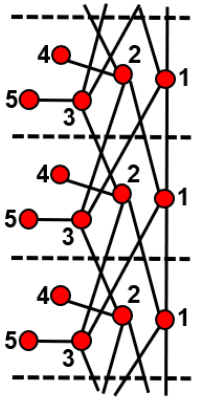	(6x**1**, 2x**2**, 2x**2**^{1})<br>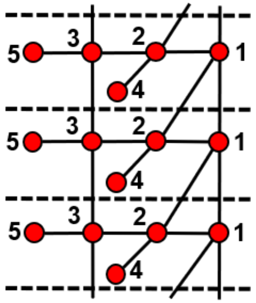

	(6x**1**, 2x**2**, 2x**2**^{2}) 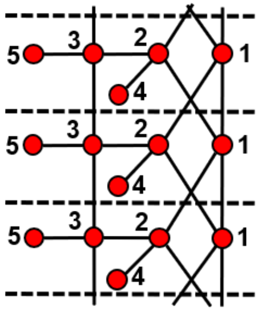	(6x**1**, 4x**2**^{1}) **1** 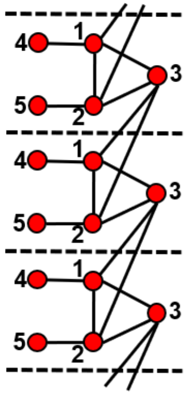	(6x**1**, 4x**2**^{1}) **2** 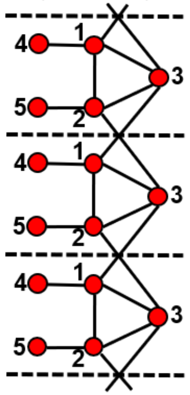	(6x**1**, 4x**2**^{1}) **3** 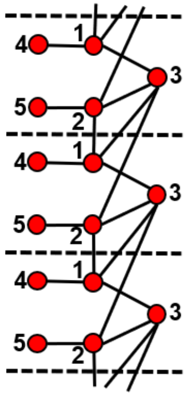
	(6x**1**, 2x**2**^{1}, 2x**2**^{2}) **1** 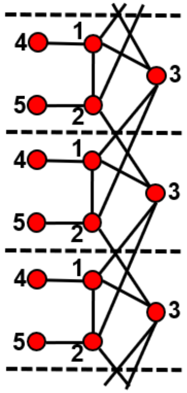	(6x**1**, 2x**2**^{1}, 2x**2**^{2}) **2** 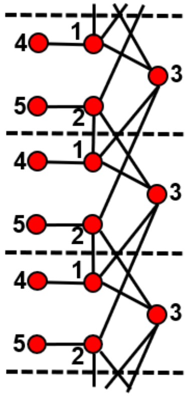	(6x**1**, 4x**2**^{2}) **1** 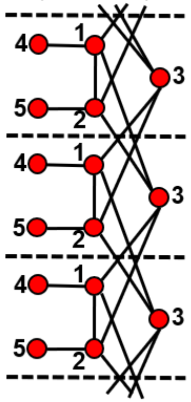	(6x**1**, 4x**2**^{2}) **2** 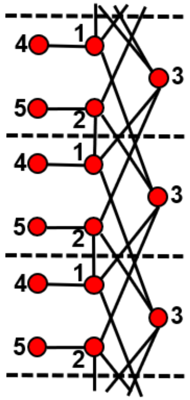
	(4x**1**, 3x**2**, 2x**2**^{1}) 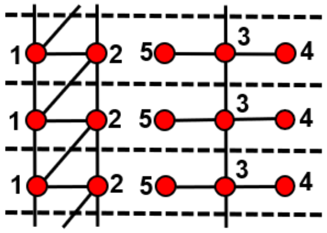	(4x**1**, 3x**2**, 2x**2**^{2}) 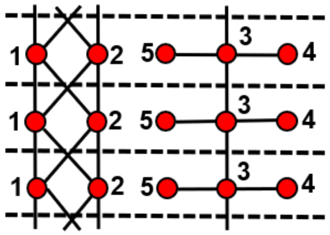	(4x**1**, 1x**2**, 4x**2**^{1}) 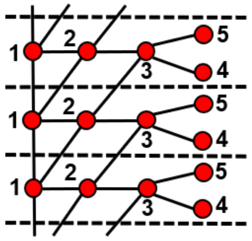	(4x**1**, 1x**2**, 2x**2**^{1}, 2x**2**^{2}) 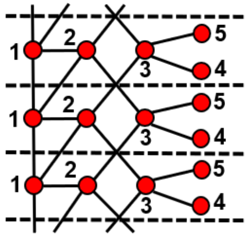

(4x**1**, 1x**2**, 4x$\mathbf{2}^{2}$)

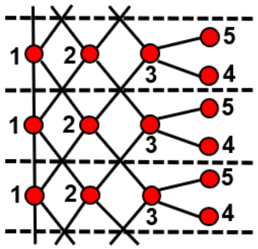

(2x**1**, 2x**2**, 4x$\mathbf{2}^{1}$)

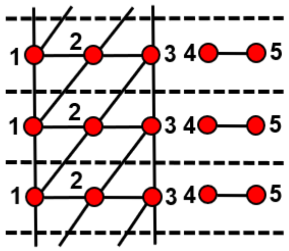

(2x**1**, 2x**2**, 2x$\mathbf{2}^{1}$, 2x$\mathbf{2}^{2}$)

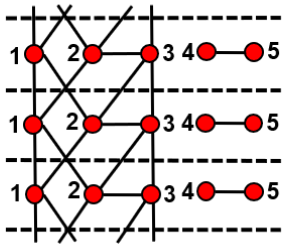

(2x**1**, 2x**2**, 4x$\mathbf{2}^{2}$)

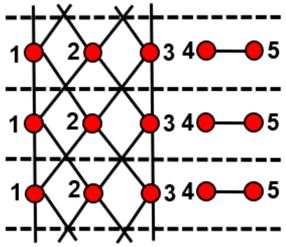

(2x**1**, 6x$\mathbf{2}^{1}$) **1**

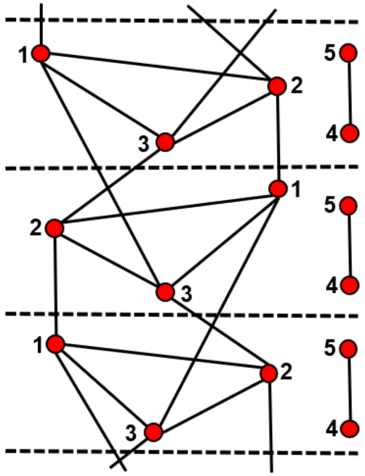

(2x**1**, 6x$\mathbf{2}^{1}$) **2**

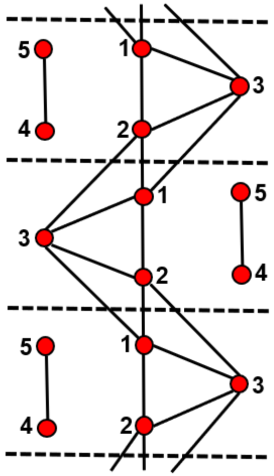

(2x**1**, 4x$\mathbf{2}^{1}$, 2x$\mathbf{2}^{2}$) **1**

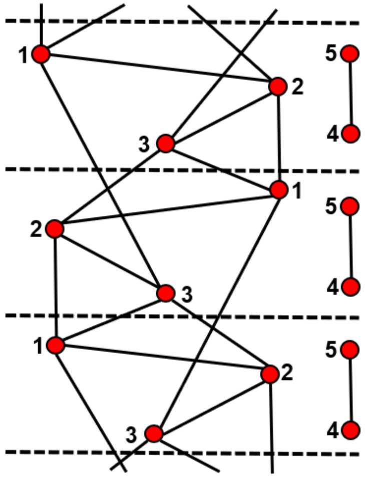

(2x**1**, 4x$\mathbf{2}^{1}$, 2x$\mathbf{2}^{2}$) **2**

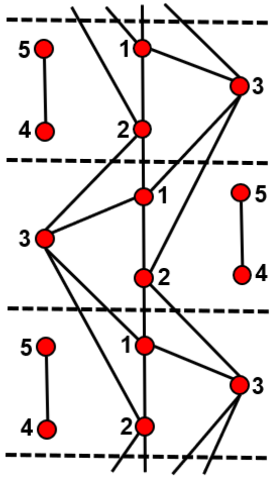

	(2x**1**, 2x$\mathbf{2}^1$, 4x$\mathbf{2}^2$) 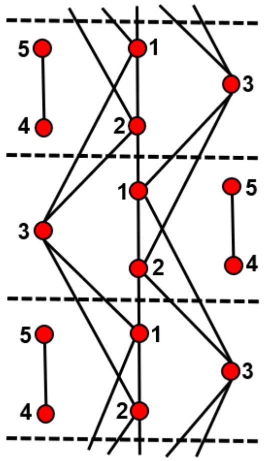	(2x**1**, 6x$\mathbf{2}^2$) 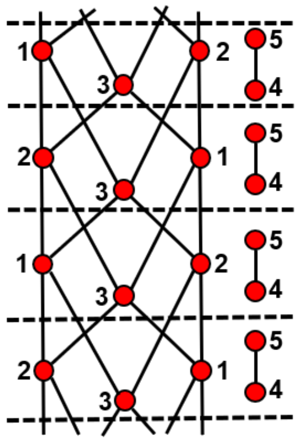		
$^1V_2{}^4V_4$	NG			
$^1V_2{}^4V_5$	NG			
$^1V_2{}^4V_6$	NG			
$^1V_4{}^4V_1$	(6x**1**, 1x**2**) 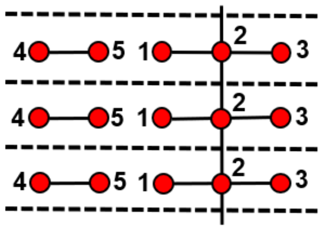			

$^{1}V_4{}^{4}V_2$	(10x**1**, 1x**2**) 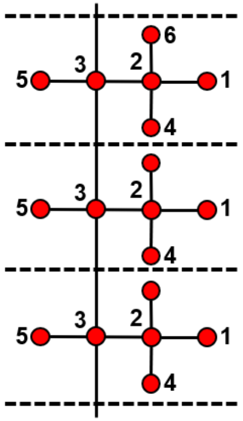	(8x**1**, 2x**2**) **a** 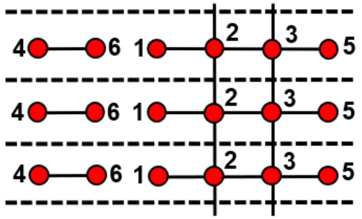	(8x**1**, 2x**2**) **b** 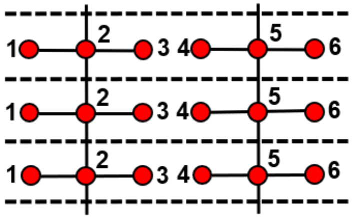	(6x**1**, 1x**2**, 2x**2**^{1}) 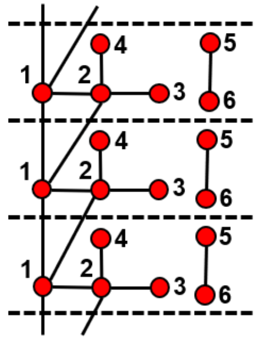
	(6x**1**, 1x**2**, 2x**2**^{2}) 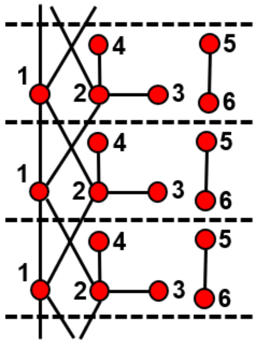	(4x**1**, 2x**2**, 2x**2**^{1}) 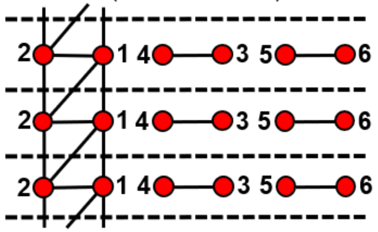	(4x**1**, 2x**2**, 2x**2**^{2}) 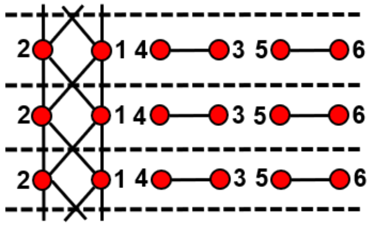	
$^{1}V_4{}^{4}V_3$	NG			
$^{1}V_4{}^{4}V_4$	NG			

$^{1}V_{6}{}^{4}V_{1}$	(8x**1**, 1x**2**) 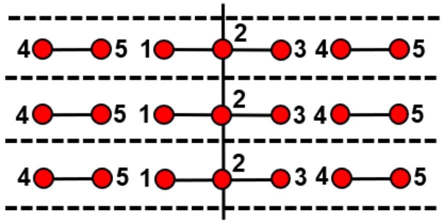			
$^{1}V_{6}{}^{4}V_{2}$	NG			
$\mathbf{^{2}V_{r}{}^{3}V_{r}}$				
$^{2}V_{1}{}^{3}V_{2}$	(4x**1**, 2x**2**) 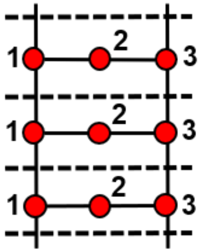	(4x**1**, 2x$\mathbf{2}^1$) **a** 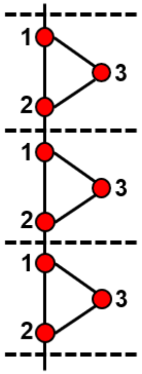	(4x**1**, 2x$\mathbf{2}^1$) **b** 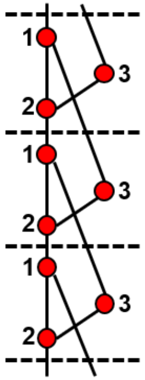	(4x**1**, 2x$\mathbf{2}^1$) **c** 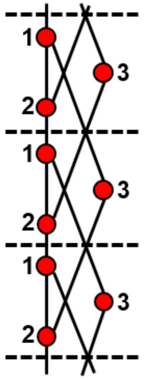
	(4x**1**, 2x$\mathbf{2}^2$) **a** 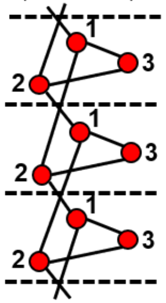	(4x**1**, 2x$\mathbf{2}^2$) **b** 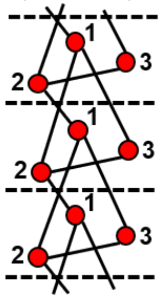	(4x**1**, 2x$\mathbf{2}^2$) **c** 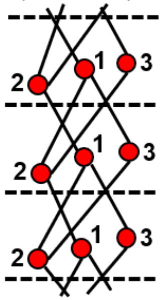	(2x**1**, 3x**2**) 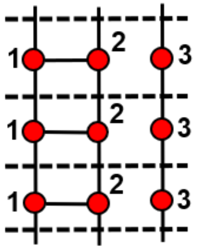

	(2x**1**, 1x**2**, 2x**2**^{1}) 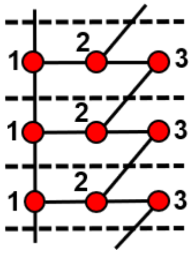	(2x**1**, 1x**2**, 2x**2**^{2}) 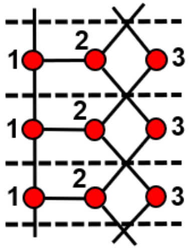		
$^2V_1{}^3V_4$	NG			
$^2V_1{}^3V_6$	NG			
$^2V_2{}^3V_2$	(10x**1**) **a** 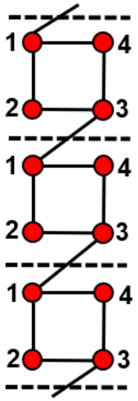	(10x**1**) **b** 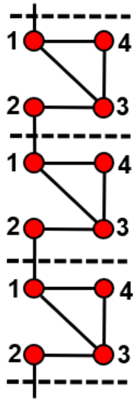	(10x**1**) **c** 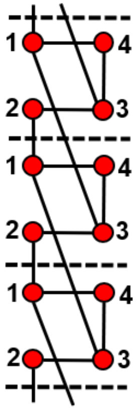	(10x**1**) **d** 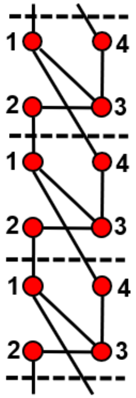

	(10x**1**) **e**<br>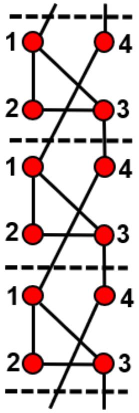	(10x**1**) **f**<br>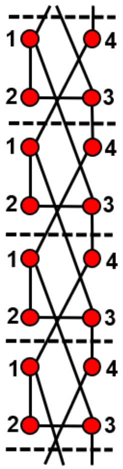	(10x**1**) **g**<br>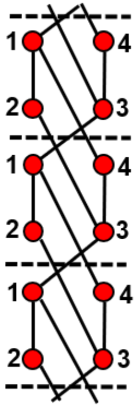	(10x**1**) **h**<br>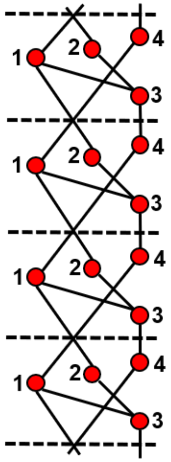
	(10x**1**) **i**<br>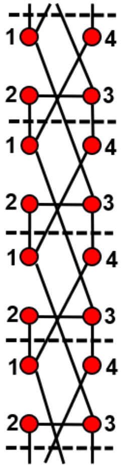	(10x**1**) **j**<br>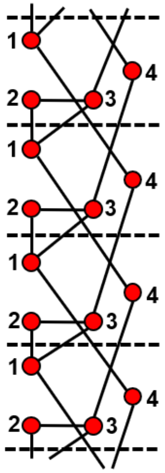	(10x**1**) **k**<br>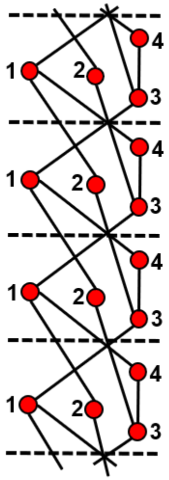	(10x**1**) **l**<br>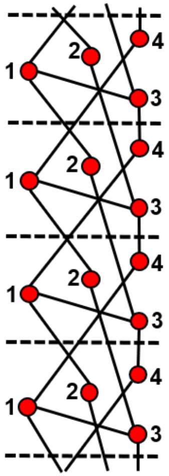

	(10x**1**) **m** 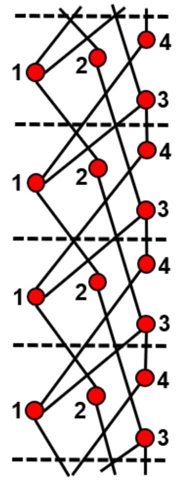	(10x**1**) **n** 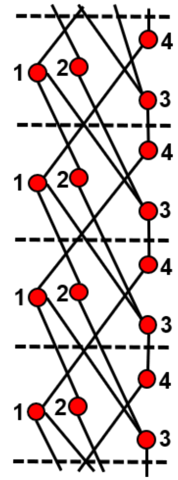	(10x**1**) **o** 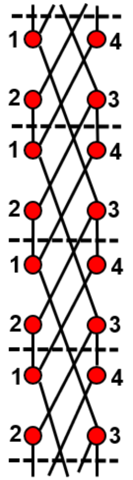	(8x**1**, 1x**2**) **a** 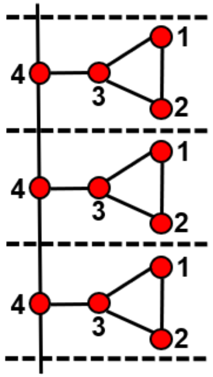
	(8x**1**, 1x**2**) **b** 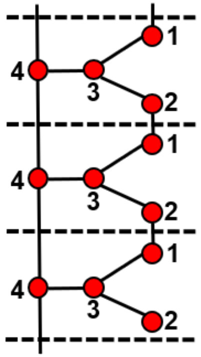	(8x**1**, 1x**2**) **c** 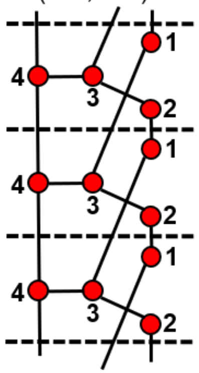	(8x**1**, 1x**2**) **d** 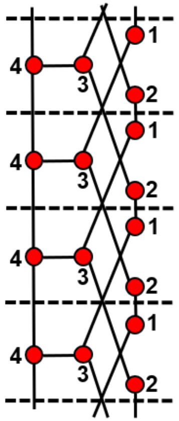	(6x**1**, 2x**2**) 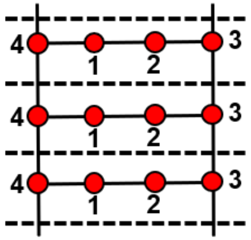

	(6x**1**, 2x$\mathbf{2}^1$) **a** 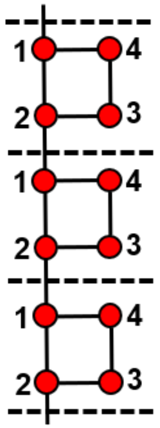	(6x**1**, 2x$\mathbf{2}^1$) **b** 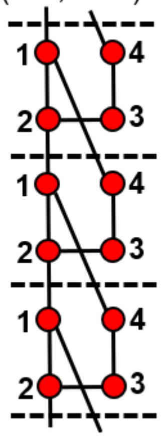	(6x**1**, 2x$\mathbf{2}^1$) **c** 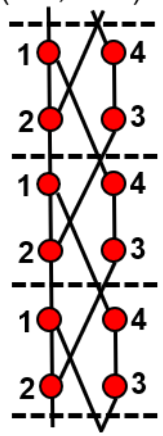	(6x**1**, 2x$\mathbf{2}^1$) **d** 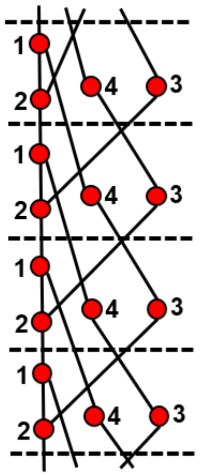
	(6x**1**, 2x$\mathbf{2}^2$) **a** 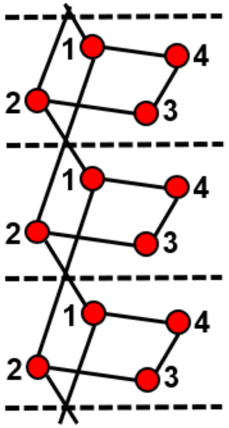	(6x**1**, 2x$\mathbf{2}^2$) **b** 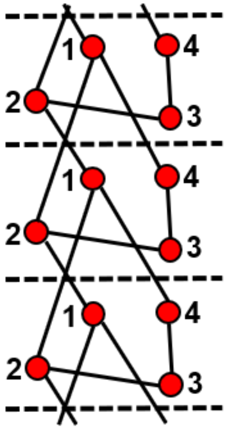	(6x**1**, 2x$\mathbf{2}^2$) **c** 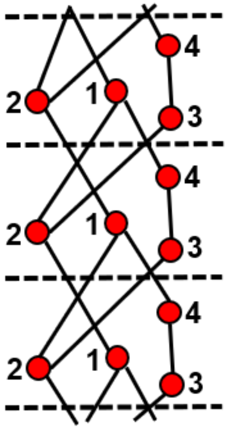	(6x**1**, 2x$\mathbf{2}^2$) **d** 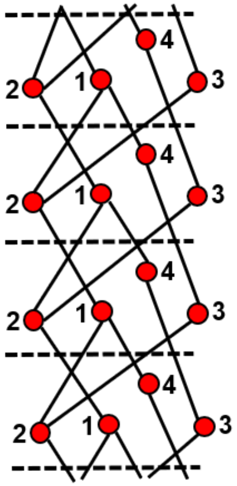

(4x**1**, 3x**2**)

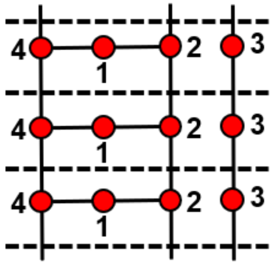

(4x**1**, 1x**2**, 2x$\mathbf{2}^{1}$) **a-1**

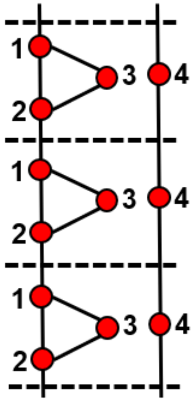

(4x**1**, 1x**2**, 2x$\mathbf{2}^{1}$) **a-2**

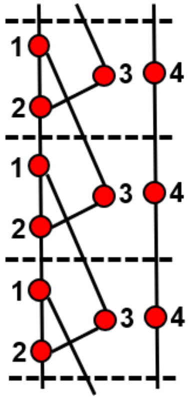

(4x**1**, 1x**2**, 2x$\mathbf{2}^{1}$) **a-3**

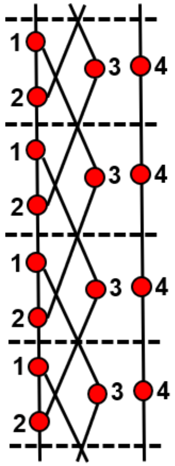

(4x**1**, 1x**2**, 2x$\mathbf{2}^{1}$) **b**

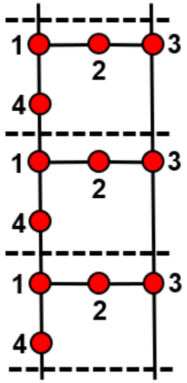

(4x**1**, 1x**2**, 2x$\mathbf{2}^{2}$) **a-1**

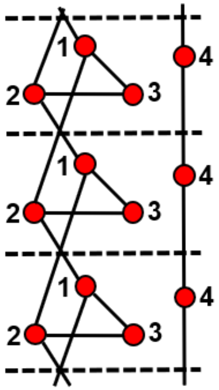

(4x**1**, 1x**2**, 2x$\mathbf{2}^{2}$) **a-2**

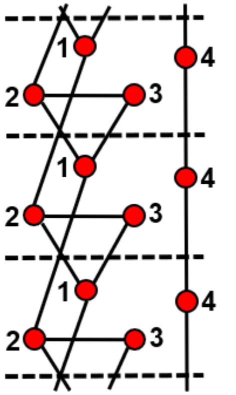

(4x**1**, 1x**2**, 2x$\mathbf{2}^{2}$) **a-3**

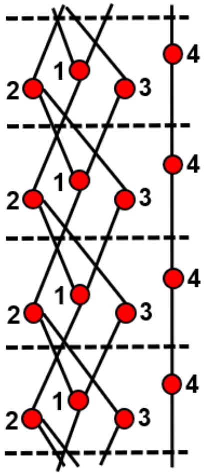

	(4x**1**, 1x**2**, 2x**2**^{2}) **b**<br>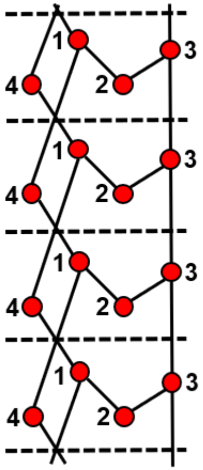	(2x**1**, 4x**2**)<br>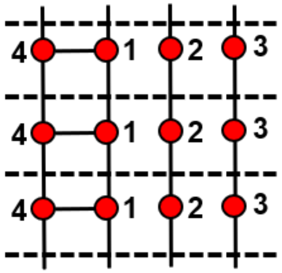	(2x**1**, 2x**2**, 2x**2**^{1}) **b**<br>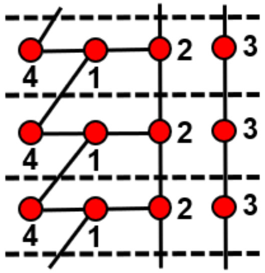	(2x**1**, 2x**2**, 2x**2**^{2}) **b**<br>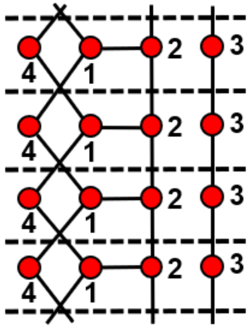
	(2x**1**, 4x**2**^{1})<br>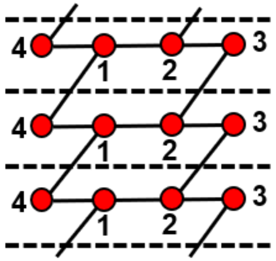	(2x**1**, 2x**2**^{1}, 2x**2**^{2})<br>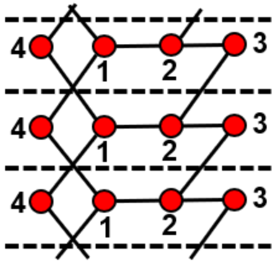	(2x**1**, 4x**2**^{2})<br>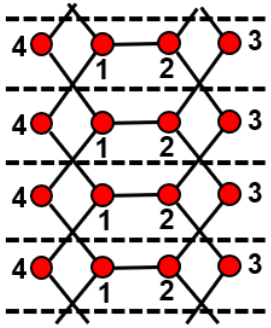	
$^{2}V_{2}{}^{3}V_{4}$	NG			
$^{2}V_{2}{}^{3}V_{6}$	NG			
$^{2}V_{3}{}^{3}V_{2}$	NG			
$^{2}V_{3}{}^{3}V_{4}$	NG			
$^{2}V_{4}{}^{3}V_{2}$	NG			

$^{2}V_{4}{}^{3}V_{4}$	NG			
$^{2}V_{5}{}^{3}V_{2}$	NG			
$^{2}V_{6}{}^{3}V_{2}$	NG			
$\boldsymbol{^{2}V_{r}{}^{4}V_{r}}$				
$^{2}V_{1}{}^{4}V_{1}$	(1x**2**, 2x**2**^{1}) 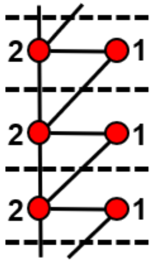	(1x**2**, 2x**2**^{2}) 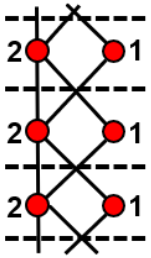		
$^{2}V_{1}{}^{4}V_{2}$	(6x**1**, 2x**2**) **a** 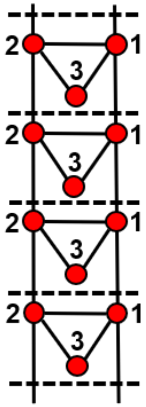	(6x**1**, 2x**2**) **b** 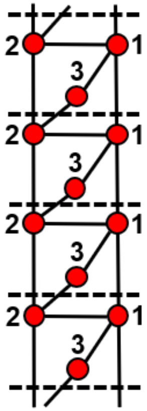	(6x**1**, 2x**2**) **c** 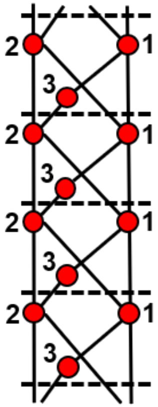	(6x**1**, 2x**2**) **d** 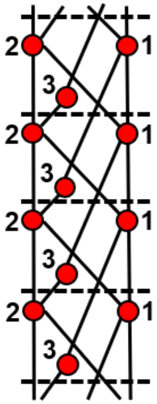

	(3x**2**, 2x**2**^{1}) 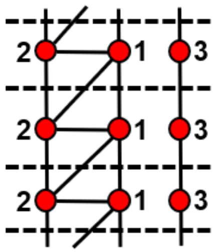	(3x**2**, 2x**2**^{2}) 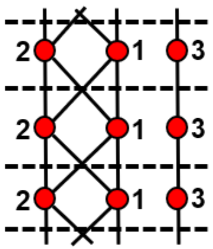	(1x**2**, 4x**2**^{1}) 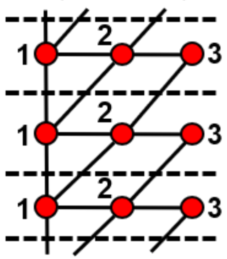	(1x**2**, 2x**2**^{1}, 2x**2**^{2}) **a** 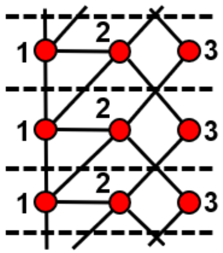
	(1x**2**, 2x**2**^{1}, 2x**2**^{2}) **b** 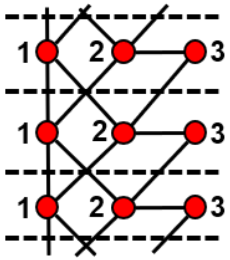	(1x**2**, 4x**2**^{2}) 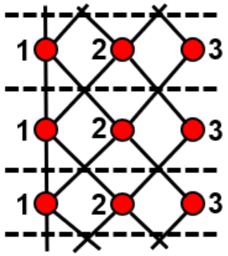		
$^{2}V_{1}{}^{4}V_{3}$	(8x**1**, 3x**2**) **a** 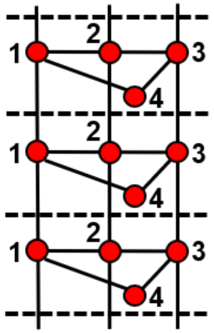	(8x**1**, 3x**2**) **b** 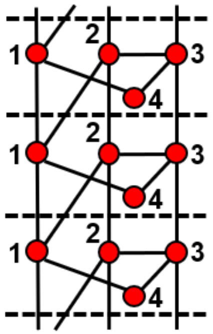	(8x**1**, 3x**2**) **c** 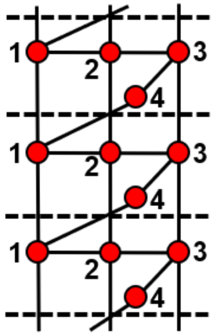	(8x**1**, 3x**2**) **d** 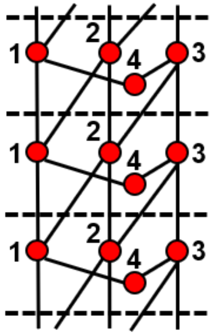

	(8x**1**, 3x**2**) **e** 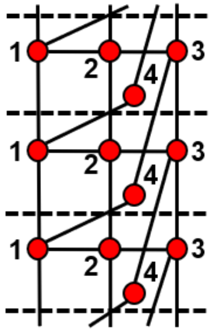	(8x**1**, 3x**2**) **f** 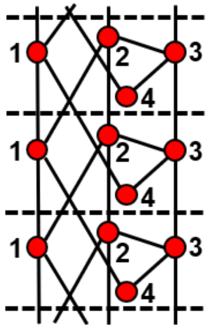	(8x**1**, 3x**2**) **g** 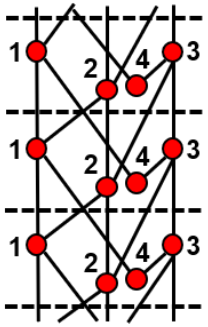	(8x**1**, 3x**2**) **h** 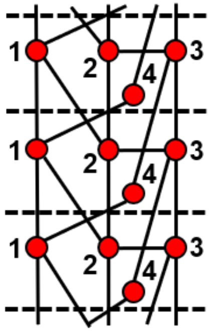
	(8x**1**, 3x**2**) **i** 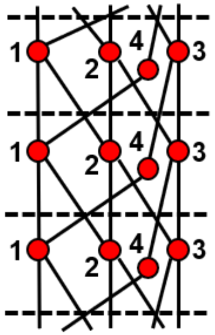	(8x**1**, 1x**2**, 2x$\mathbf{2}^1$) **a** 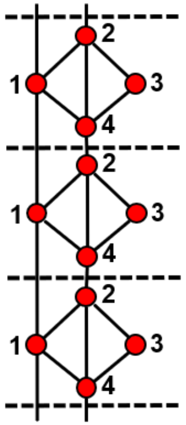	(8x**1**, 1x**2**, 2x$\mathbf{2}^1$) **b** 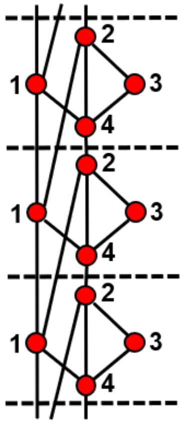	(8x**1**, 1x**2**, 2x$\mathbf{2}^1$) **c** 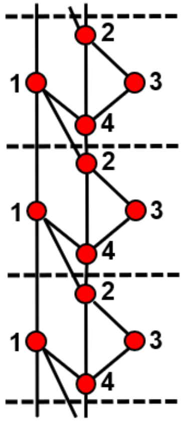

	(8x**1**, 1x**2**, 2x**2**^{1}) **d** 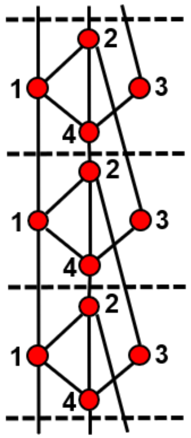	(8x**1**, 1x**2**, 2x**2**^{1}) **e** 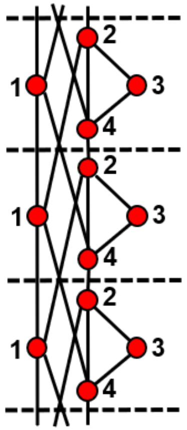	(8x**1**, 1x**2**, 2x**2**^{1}) **f** 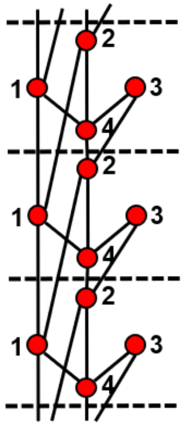	(8x**1**, 1x**2**, 2x**2**^{1}) **g** 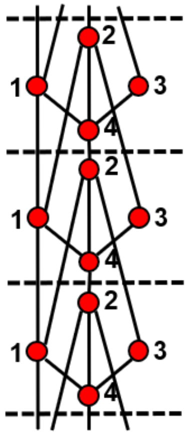
	(8x**1**, 1x**2**, 2x**2**^{1}) **h** 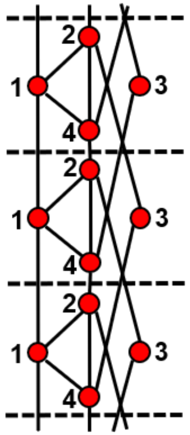	(8x**1**, 1x**2**, 2x**2**^{1}) **i** 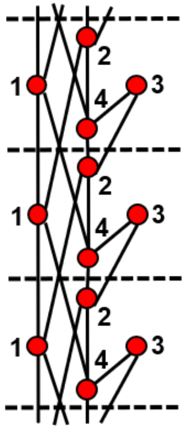	(8x**1**, 1x**2**, 2x**2**^{1}) **j** 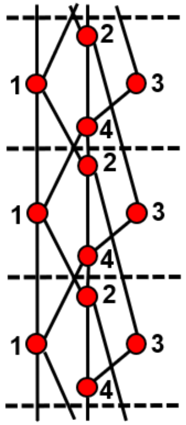	(8x**1**, 1x**2**, 2x**2**^{1}) **k** 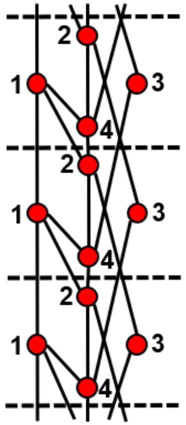

	(8x**1**, 1x**2**, 2x**2**^{1}) **l** 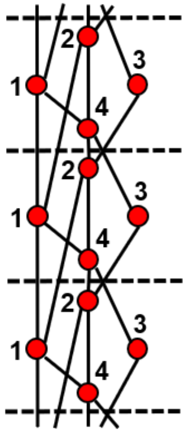	(8x**1**, 1x**2**, 2x**2**^{2}) **a** 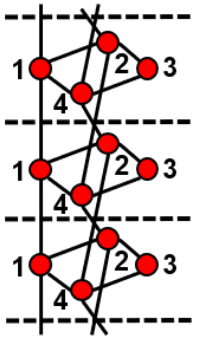	(8x**1**, 1x**2**, 2x**2**^{2}) **b** 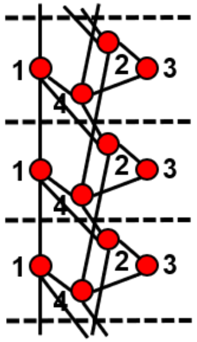	(8x**1**, 1x**2**, 2x**2**^{2}) **c** 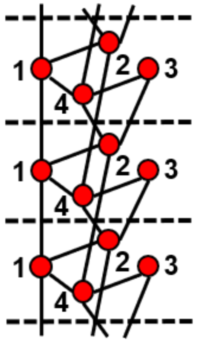
	(8x**1**, 1x**2**, 2x**2**^{2}) **d** 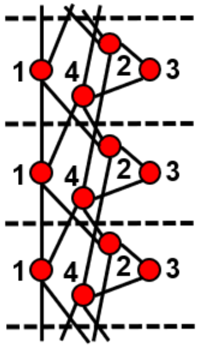	(8x**1**, 1x**2**, 2x**2**^{2}) **e** 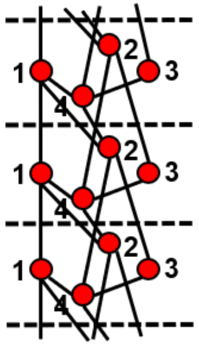	(8x**1**, 1x**2**, 2x**2**^{2}) **f** 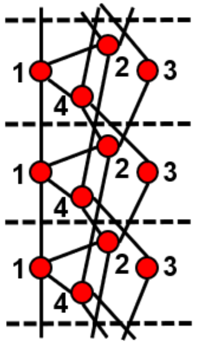	(8x**1**, 1x**2**, 2x**2**^{2}) **g** 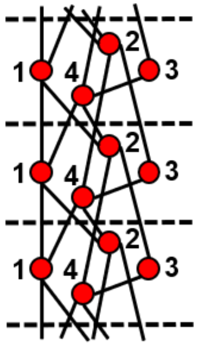

	(8x**1**, 1x**2**, 2x**2**^{2}) **h**<br>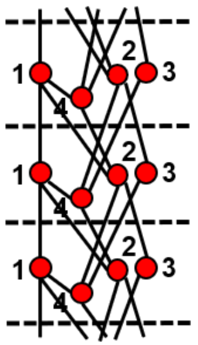	(8x**1**, 1x**2**, 2x**2**^{2}) **i**<br>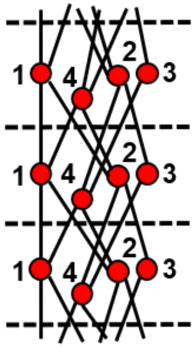	(6x**1**, 4x**2**) **a**<br>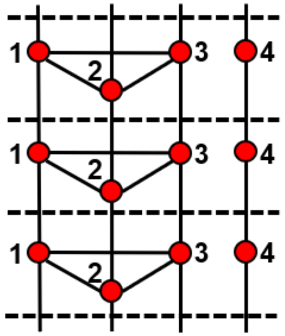	(6x**1**, 4x**2**) **b**<br>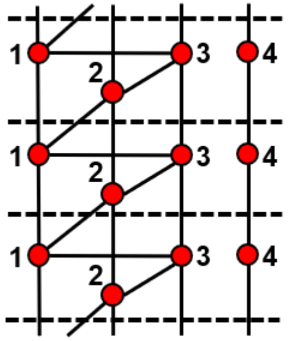
	(6x**1**, 4x**2**) **c**<br>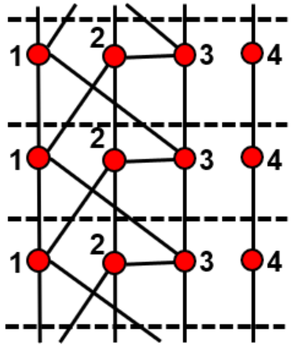	(6x**1**, 4x**2**) **d**<br>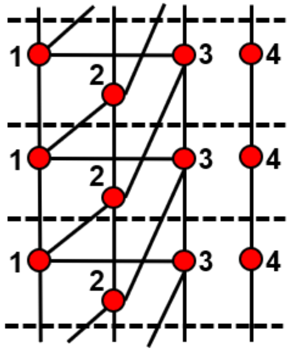	(6x**1**, 4x**2**) **e**<br>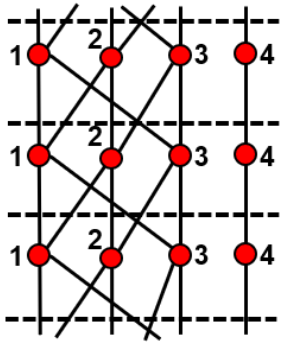	(6x**1**, 2x**2**, 2x**2**^{1}) **a-1**<br>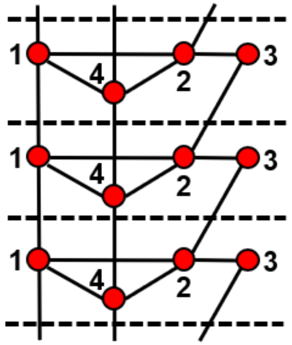

	(6x**1**, 2x**2**, 2x**2**^{1}) **a-2** 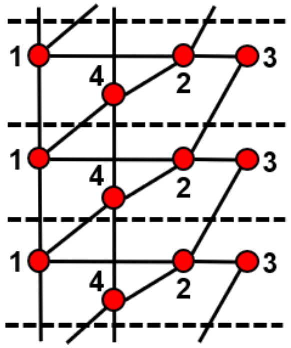	(6x**1**, 2x**2**, 2x**2**^{1}) **a-3** 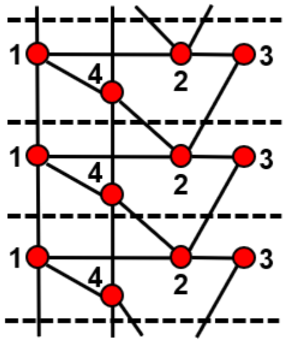	(6x**1**, 2x**2**, 2x**2**^{1}) **a-4** 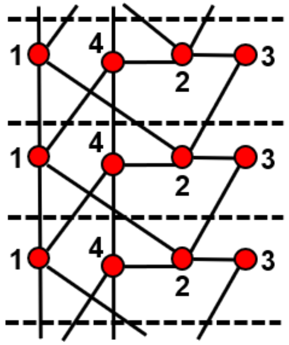	(6x**1**, 2x**2**, 2x**2**^{1}) **a-5** 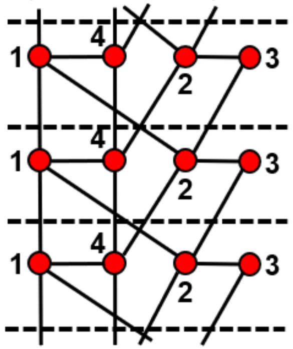
	(6x**1**, 2x**2**, 2x**2**^{1}) **a-6** 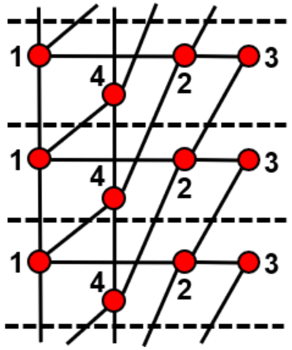	(6x**1**, 2x**2**, 2x**2**^{1}) **a-7** 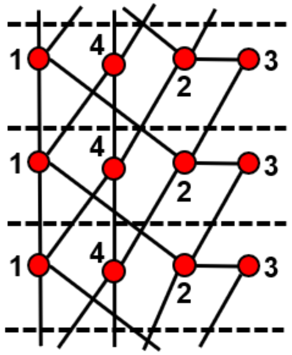	(6x**1**, 2x**2**, 2x**2**^{1}) **b-1** 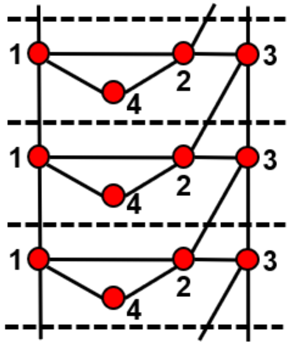	(6x**1**, 2x**2**, 2x**2**^{1}) **b-2** 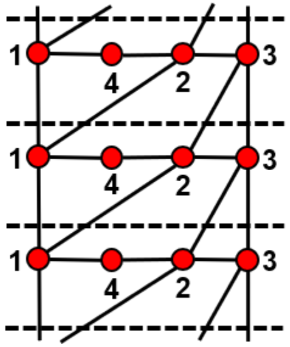

(6x**1**, 2x**2**, 2x$\mathbf{2}^{1}$) **b-3**

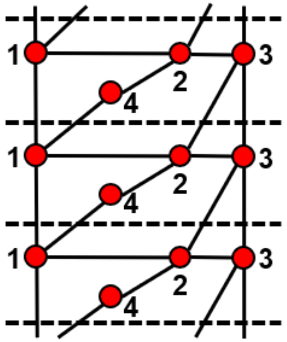

(6x**1**, 2x**2**, 2x$\mathbf{2}^{1}$) **b-4**

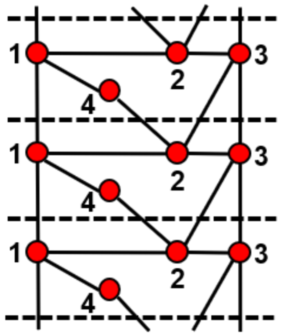

(6x**1**, 2x**2**, 2x$\mathbf{2}^{1}$) **b-5**

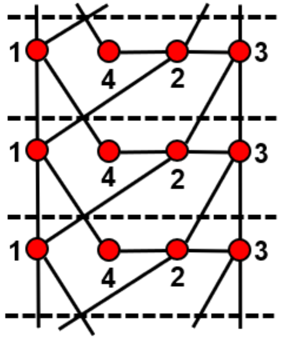

(6x**1**, 2x**2**, 2x$\mathbf{2}^{1}$) **b-6**

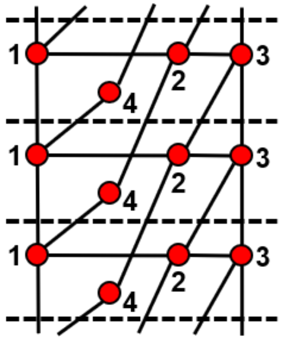

(6x**1**, 2x**2**, 2x$\mathbf{2}^{1}$) **b-7**

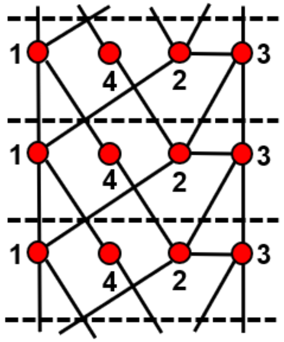

(6x**1**, 2x**2**, 2x$\mathbf{2}^{2}$) **a-1**

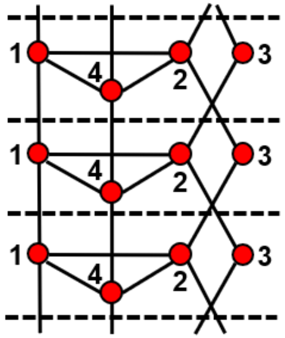

(6x**1**, 2x**2**, 2x$\mathbf{2}^{2}$) **a-2**

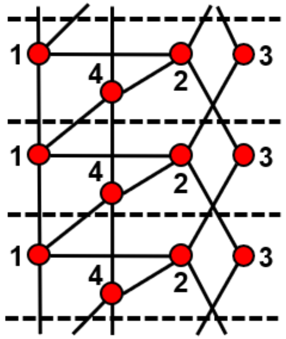

(6x**1**, 2x**2**, 2x$\mathbf{2}^{2}$) **a-3**

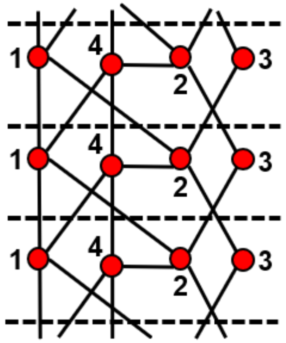

(6x**1**, 2x**2**, 2x$\mathbf{2}^{2}$) **a-4**

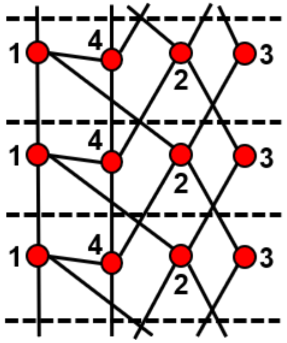

(6x**1**, 2x**2**, 2x$\mathbf{2}^{2}$) **a-5**

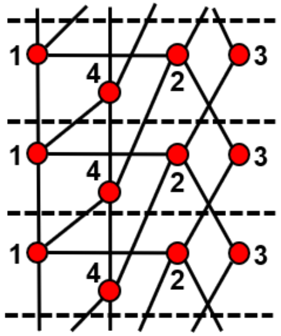

(6x**1**, 2x**2**, 2x$\mathbf{2}^{2}$) **a-6**

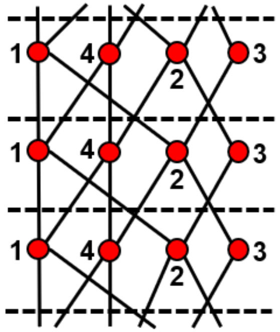

(6x**1**, 2x**2**, 2x$\mathbf{2}^{2}$) **b-1**

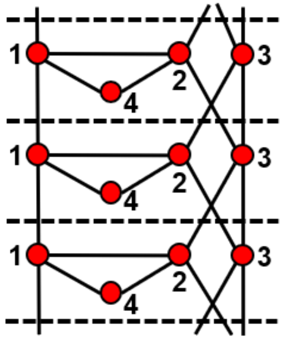

(6x**1**, 2x**2**, 2x$\mathbf{2}^{2}$) **b-2**

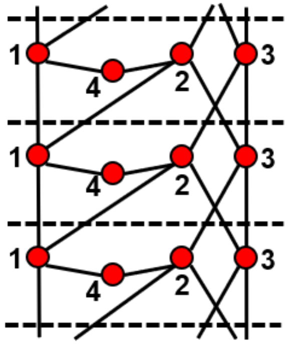

(6x**1**, 2x**2**, 2x$\mathbf{2}^{2}$) **b-3**

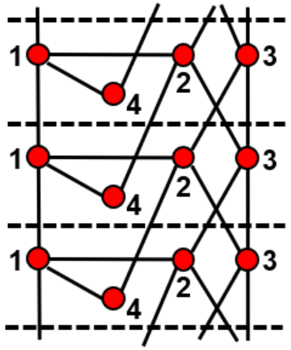

(6x**1**, 2x**2**, 2x$\mathbf{2}^{2}$) **b-4**

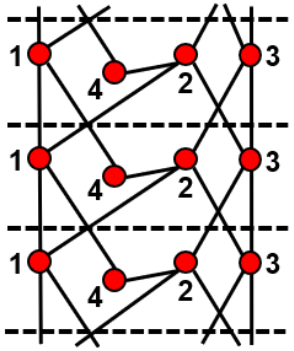

(6x**1**, 2x**2**, 2x$\mathbf{2}^{2}$) **b-5**

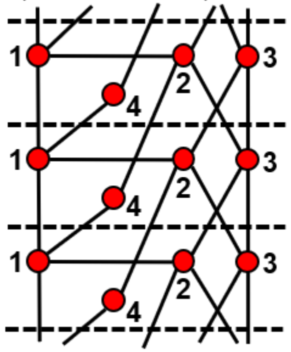

	(6x**1**, 2x**2**, 2x**2**^{2}) **b-6** 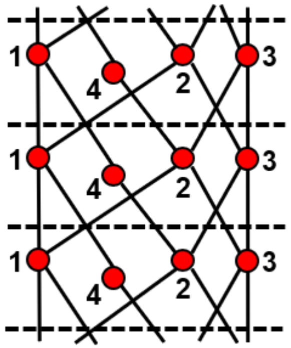	(6x**1**, 4x**2**^{1}) **a** 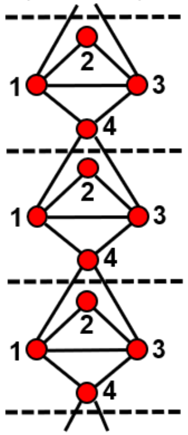	(6x**1**, 4x**2**^{1}) **b** 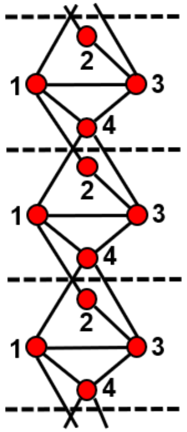	(6x**1**, 4x**2**^{1}) **c** 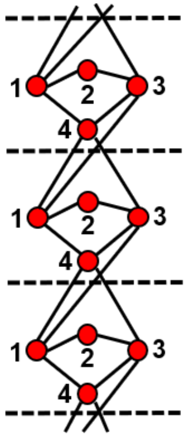
	(6x**1**, 4x**2**^{1}) **d** 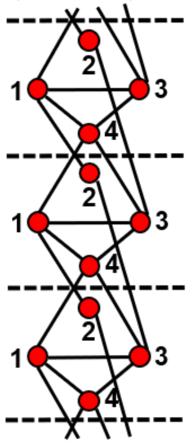	(6x**1**, 4x**2**^{1}) **e** 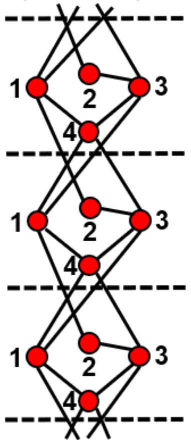	(6x**1**, 4x**2**^{1}) **f** 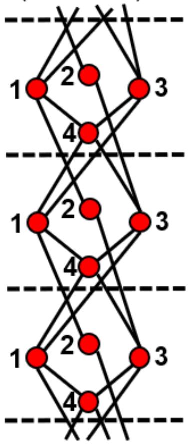	(6x**1**, 2x**2**^{1}, 2x**2**^{2}) **a** 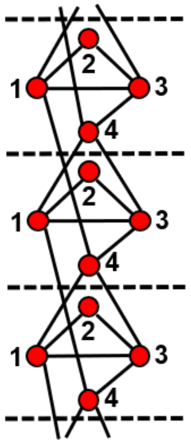

	(6x**1**, 2x**2**^{1}, 2x**2**^{2}) **b**<br>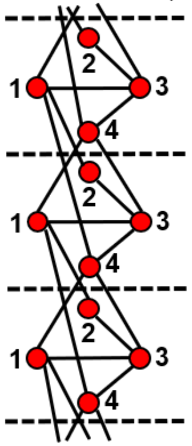	(6x**1**, 2x**2**^{1}, 2x**2**^{2}) **c**<br>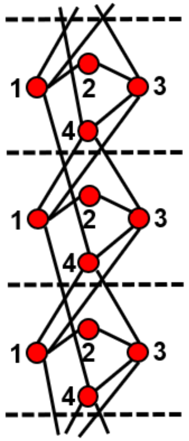	(6x**1**, 2x**2**^{1}, 2x**2**^{2}) **d**<br>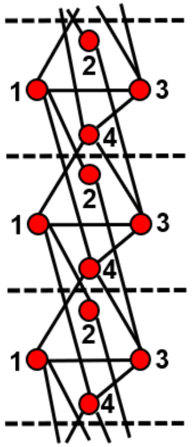	(6x**1**, 2x**2**^{1}, 2x**2**^{2}) **e**<br>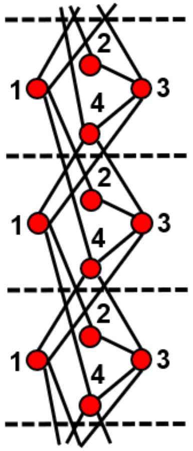
	(6x**1**, 2x**2**^{1}, 2x**2**^{2}) **f**<br>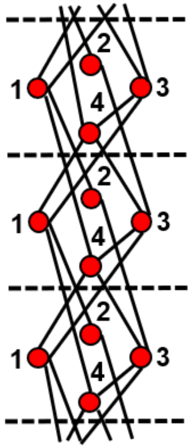	(6x**1**, 4x**2**^{2}) **a**<br>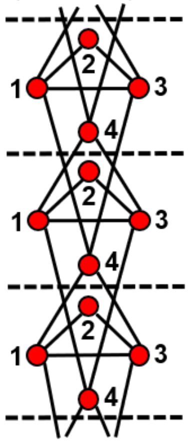	(6x**1**, 4x**2**^{2}) **b**<br>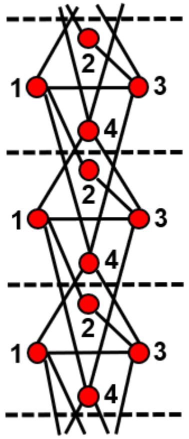	(6x**1**, 4x**2**^{2}) **c**<br>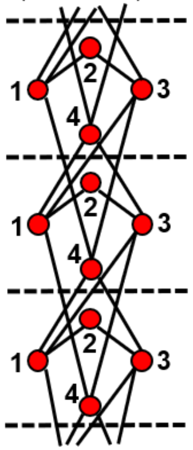

	(6x**1**, 4x**2**^{2}) **d**<br>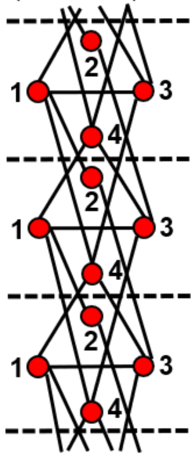	(6x**1**, 4x**2**^{2}) **e**<br>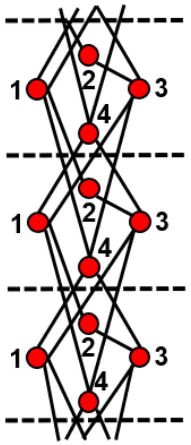	(6x**1**, 4x**2**^{2}) **f**<br>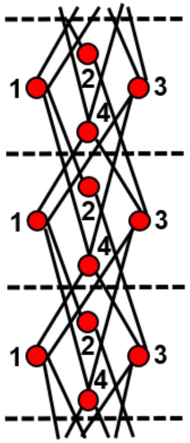	(3x**2**, 4x**2**^{1}) **a-1**<br>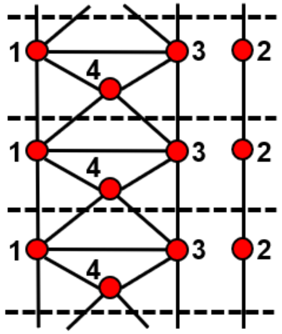
	(3x**2**, 4x**2**^{1}) **a-2**<br>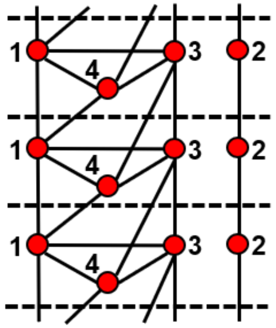	(3x**2**, 4x**2**^{1}) **b**<br>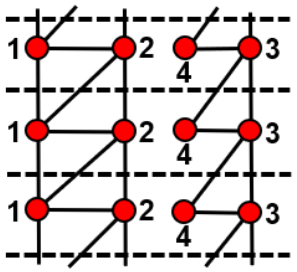	(3x**2**, 2x**2**^{1}, 2x**2**^{2}) **a-1**<br>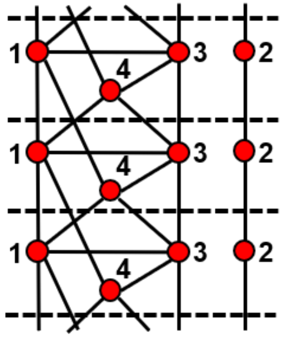	(3x**2**, 2x**2**^{1}, 2x**2**^{2}) **a-2**<br>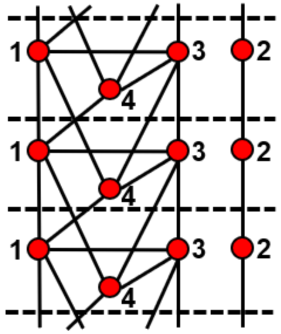

	(3x**2**, 2x$\mathbf{2}^1$, 2x$\mathbf{2}^2$) **b** 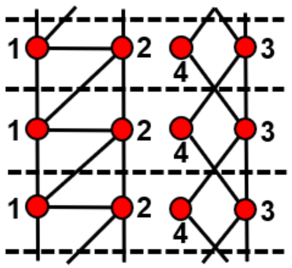	(3x**2**, 2x$\mathbf{2}^1$, 2x$\mathbf{2}^2$) **c** 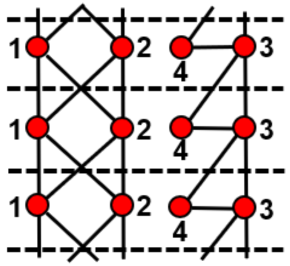	(3x**2**, 4x$\mathbf{2}^2$) **a** 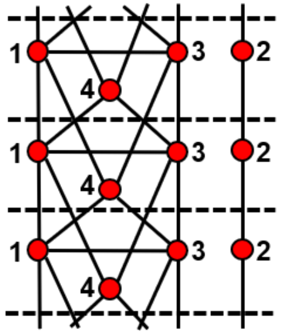	(3x**2**, 4x$\mathbf{2}^2$) **b** 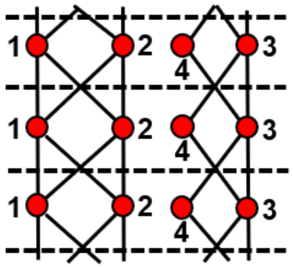
	(1x**2**, 6x$\mathbf{2}^1$) **a-1** 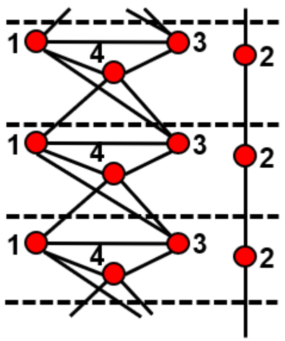	(1x**2**, 6x$\mathbf{2}^1$) **a-2** 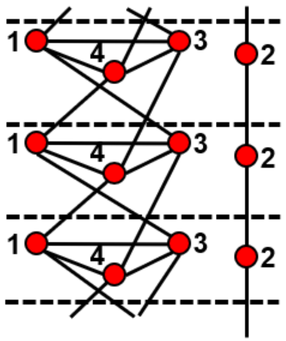	(1x**2**, 6x$\mathbf{2}^1$) **b** 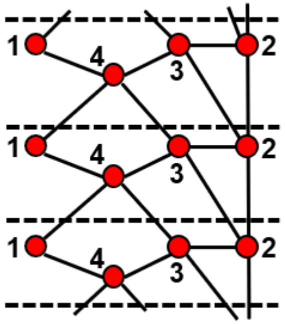	(1x**2**, 4x$\mathbf{2}^1$, 2x$\mathbf{2}^2$) **a-1** 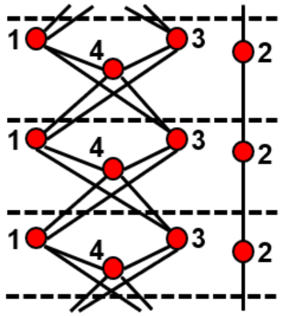
	(1x**2**, 4x$\mathbf{2}^1$, 2x$\mathbf{2}^2$) **a-2** 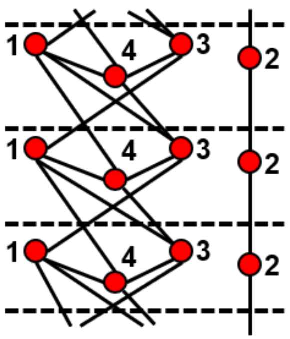	(1x**2**, 4x$\mathbf{2}^1$, 2x$\mathbf{2}^2$) **b** 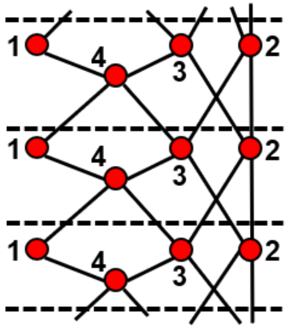	(1x**2**, 4x$\mathbf{2}^1$, 2x$\mathbf{2}^2$) **c** 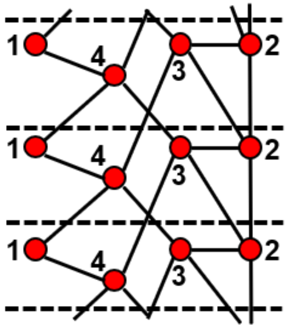	(1x**2**, 2x$\mathbf{2}^1$, 4x$\mathbf{2}^2$) **a** 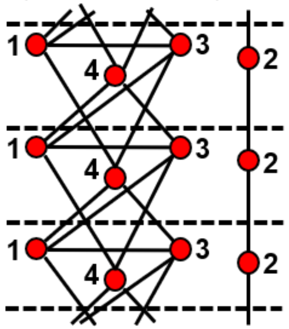

	(1x**2**, 2x$\mathbf{2}^1$, 4x$\mathbf{2}^2$) **b** 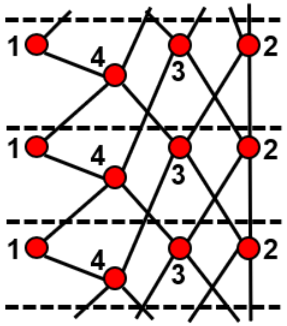	(1x**2**, 2x$\mathbf{2}^1$, 4x$\mathbf{2}^2$) **c** 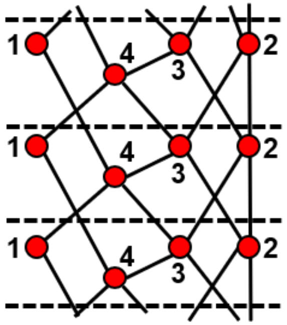	(1x**2**, 2x$\mathbf{2}^1$, 4x$\mathbf{2}^2$) **d** 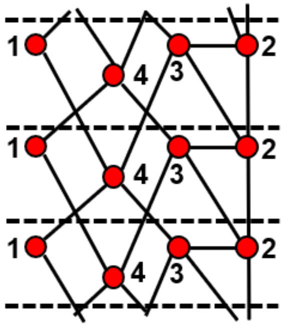	(1x**2**, 6x$\mathbf{2}^2$) **a** 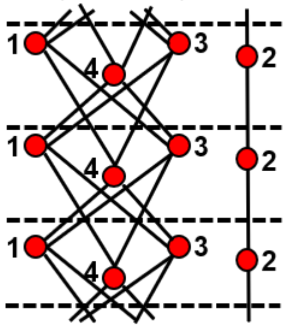
	(1x**2**, 6x$\mathbf{2}^2$) **b** 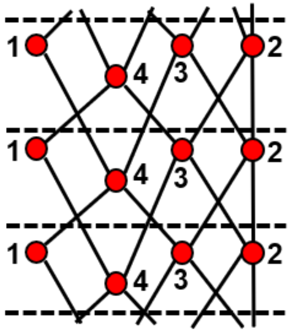			
$^2V_1{}^4V_4$	NG			
$^2V_1{}^4V_5$	NG			
$^2V_1{}^4V_6$	NG			
$^2V_1{}^4V_7$	NG			

$^2V_2{}^4V_1$	(6x**1**, 1x**2**) **a** 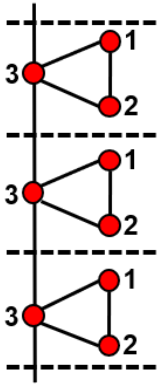	(6x**1**, 1x**2**) **b** 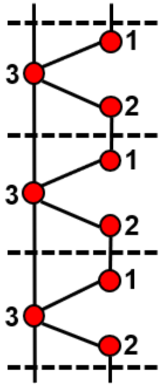	(6x**1**, 1x**2**) **c** 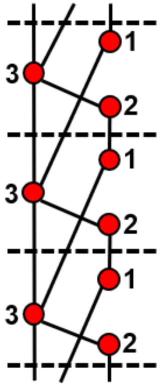	(6x**1**, 1x**2**) **d** 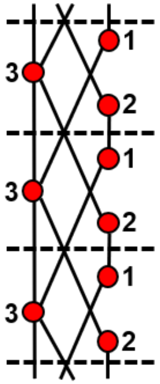
	(2x**2**, 2x**2**^{1}) 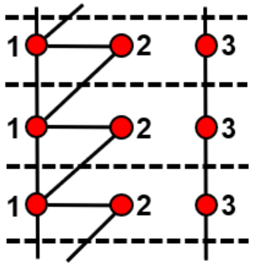	(2x**2**, 2x**2**^{2}) 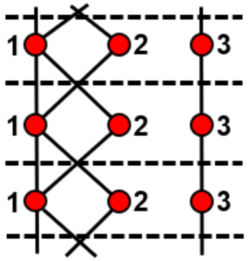	(4x**2**^{1}) 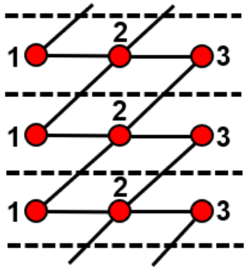	(2x**2**^{1}, 2x**2**^{2}) 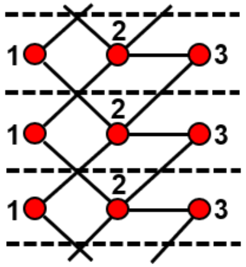
	(4x**2**^{2}) 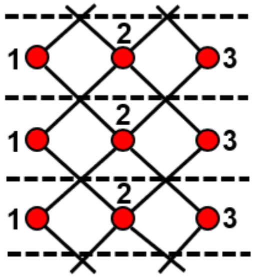			

$^2V_2{}^4V_2$	(8x**1**, 2x**2**) **a-1**<br>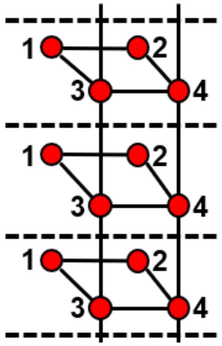	(8x**1**, 2x**2**) **a-2**<br>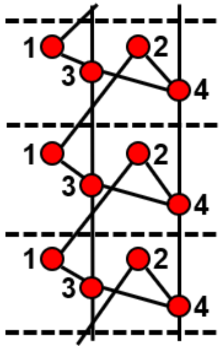	(8x**1**, 2x**2**) **a-3**<br>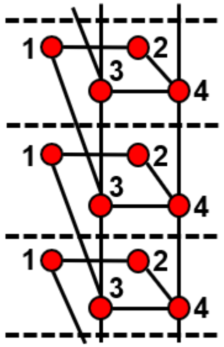	(8x**1**, 2x**2**) **a-4**<br>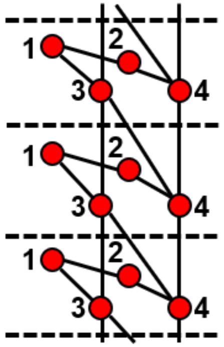
	(8x**1**, 2x**2**) **a-5**<br>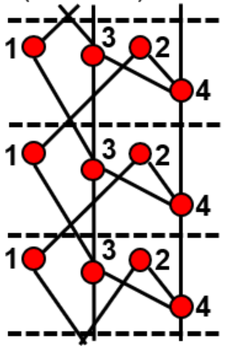	(8x**1**, 2x**2**) **a-6**<br>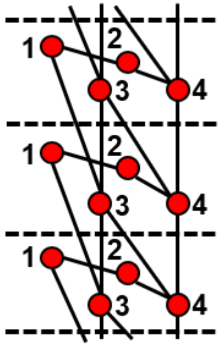	(8x**1**, 2x**2**) **a-7**<br>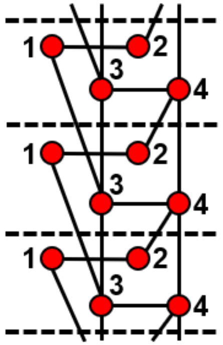	(8x**1**, 2x**2**) **a-8**<br>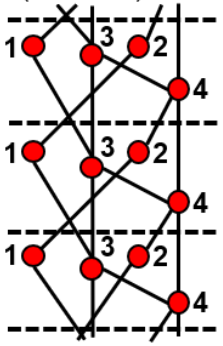

	(8x**1**, 2x**2**) **a-9** 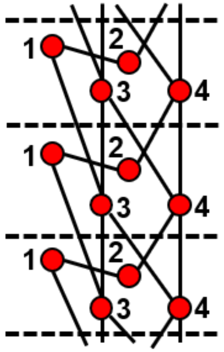	(8x**1**, 2x**2**) **a-10** 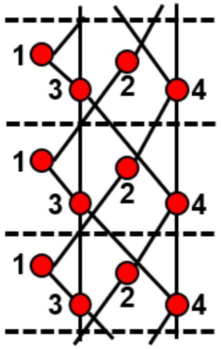	(8x**1**, 2x**2**) **a-11** 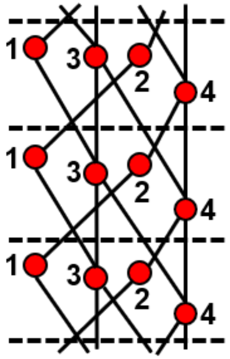	(8x**1**, 2x**2**) **b-1** 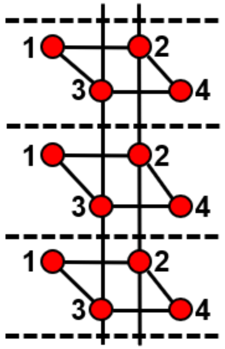
	(8x**1**, 2x**2**) **b-2** 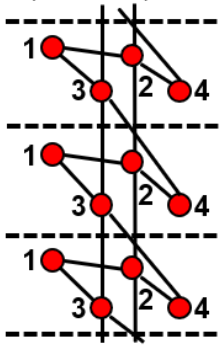	(8x**1**, 2x**2**) **b-3** 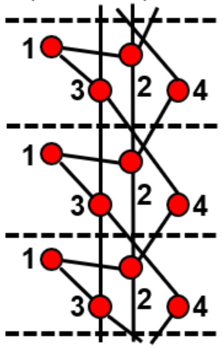	(8x**1**, 2x**2**) **b-4** 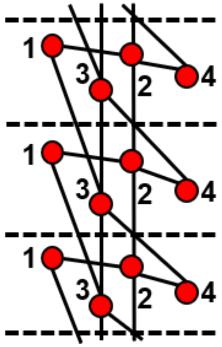	(8x**1**, 2x**2**) **b-5** 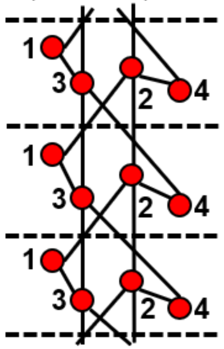

	(8x**1**, 2x**2**) **b-6** 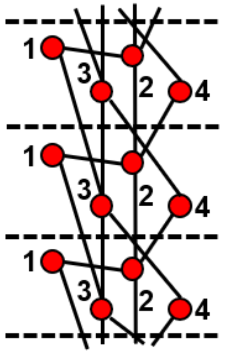	(8x**1**, 2x**2**) **b-7** 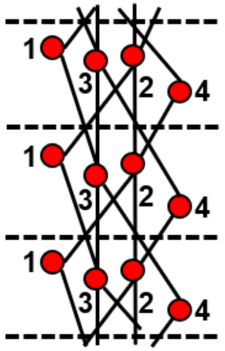	(8x**1**, 2x**2**^{1}) **a** 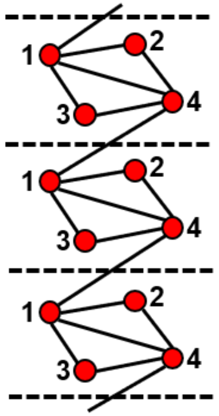	(8x**1**, 2x**2**^{1}) **b** 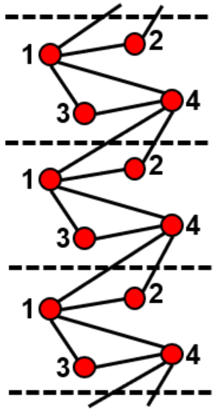
	(8x**1**, 2x**2**^{1}) **c** 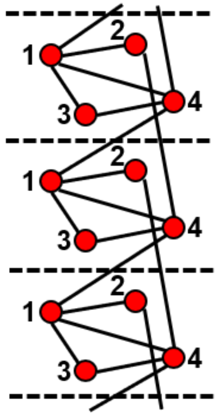	(8x**1**, 2x**2**^{1}) **d** 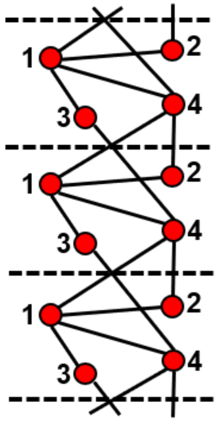	(8x**1**, 2x**2**^{1}) **e** 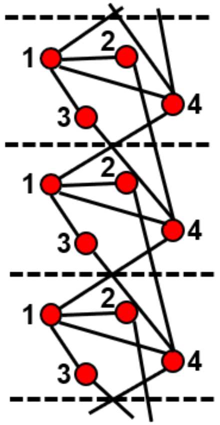	(8x**1**, 2x**2**^{1}) **f** 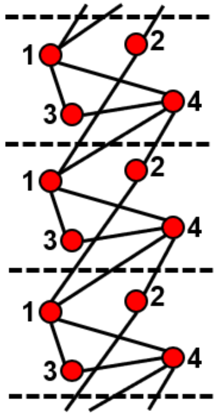

	(8x**1**, 2x**2**^{1}) **g**<br>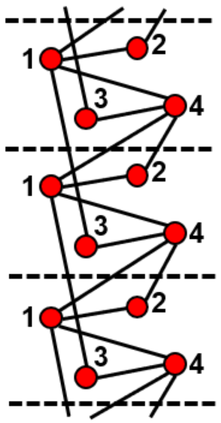 	(8x**1**, 2x**2**^{1}) **h**<br>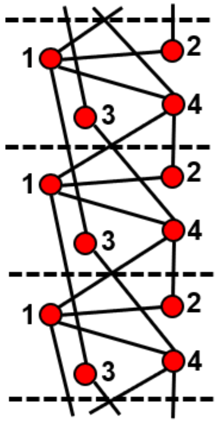 	(8x**1**, 2x**2**^{1}) **i**<br>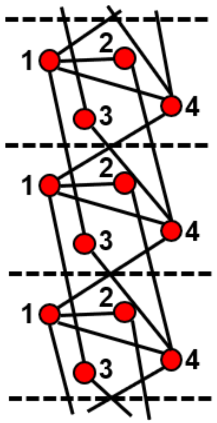 	(8x**1**, 2x**2**^{1}) **j**<br>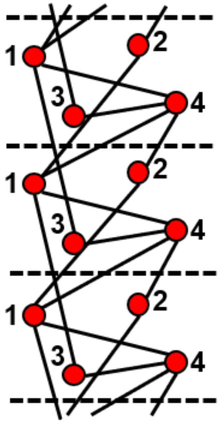
	(8x**1**, 2x**2**^{1}) **k**<br>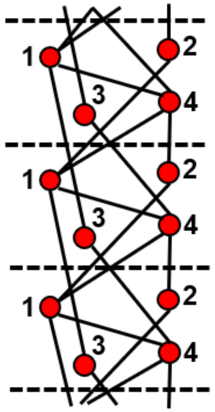 	(8x**1**, 2x**2**^{2}) **a**<br>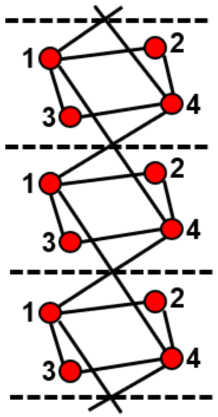 	(8x**1**, 2x**2**^{2}) **b**<br>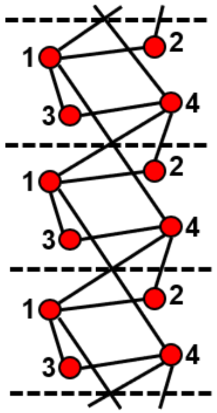 	(8x**1**, 2x**2**^{2}) **c**<br>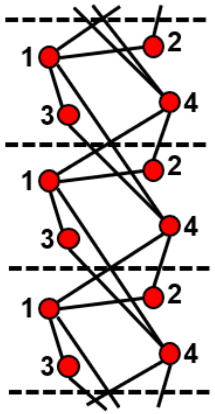

	(8x**1**, 2x**2**^{2}) **d** 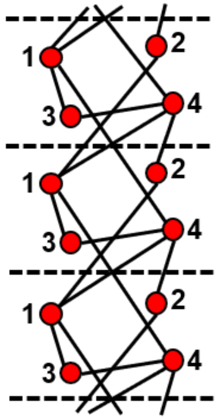	(8x**1**, 2x**2**^{2}) **e** 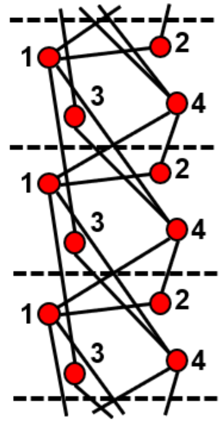	(8x**1**, 2x**2**^{2}) **f** 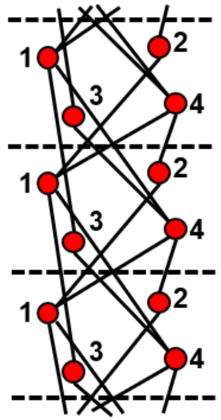	(6x**1**, 3x**2**) **a** 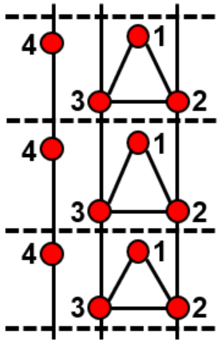
	(6x**1**, 3x**2**) **b** 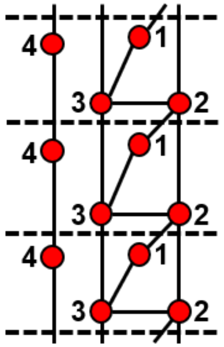	(6x**1**, 3x**2**) **c** 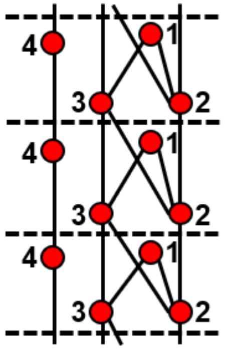	(6x**1**, 3x**2**) **d** 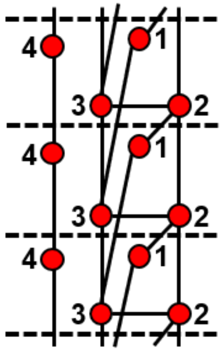	(6x**1**, 3x**2**) **e** 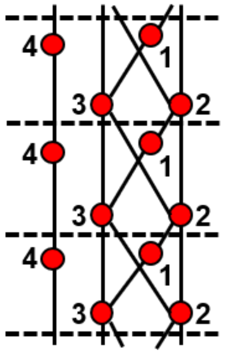

	(6x**1**, 3x**2**) **f** 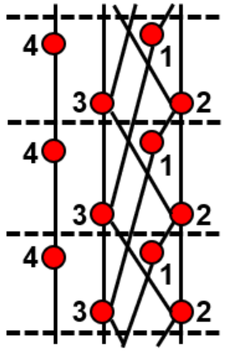	(6x**1**, 1x**2**, 2x**2**^{1}) **a-1** 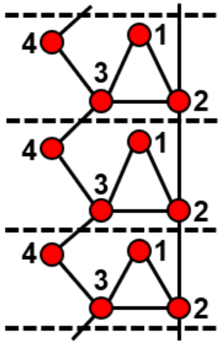	(6x**1**, 1x**2**, 2x**2**^{1}) **a-2** 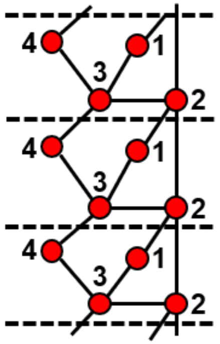	(6x**1**, 1x**2**, 2x**2**^{1}) **a-3** 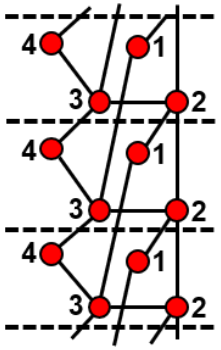
	(6x**1**, 1x**2**, 2x**2**^{1}) **a-4** 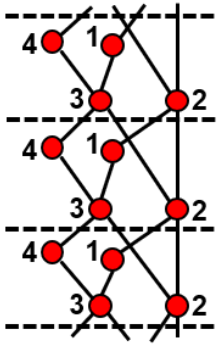	(6x**1**, 1x**2**, 2x**2**^{1}) **a-5** 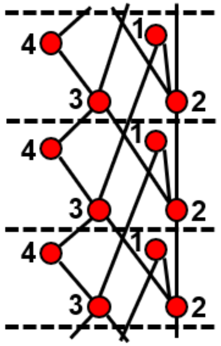	(6x**1**, 1x**2**, 2x**2**^{1}) **a-6** 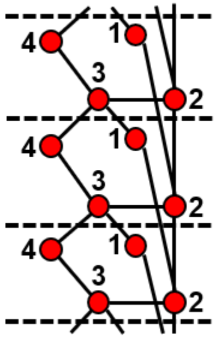	(6x**1**, 1x**2**, 2x**2**^{1}) **a-7** 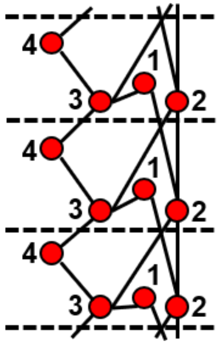

	(6x**1**, 1x**2**, 2x**2**^{1}) **a-8** 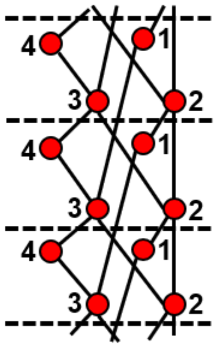	(6x**1**, 1x**2**, 2x**2**^{1}) **b-1** 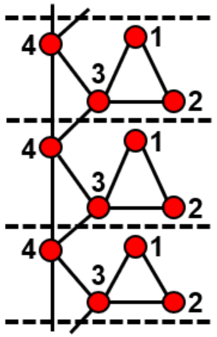	(6x**1**, 1x**2**, 2x**2**^{1}) **b-2** 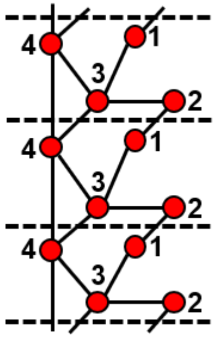	(6x**1**, 1x**2**, 2x**2**^{1}) **b-3** 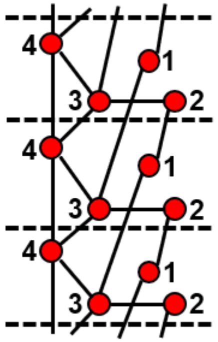
	(6x**1**, 1x**2**, 2x**2**^{1}) **b-4** 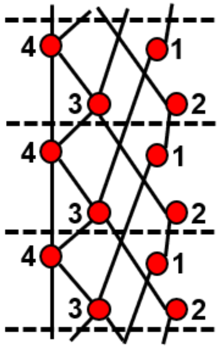	(6x**1**, 1x**2**, 2x**2**^{2}) **a-1** 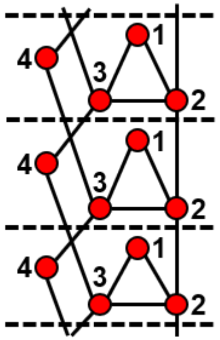	(6x**1**, 1x**2**, 2x**2**^{2}) **a-2** 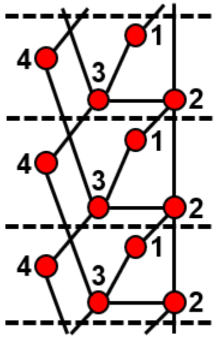	(6x**1**, 1x**2**, 2x**2**^{2}) **a-3** 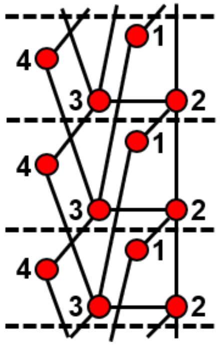

(6x**1**, 1x**2**, 2x$\mathbf{2}^{2}$) **a-4**

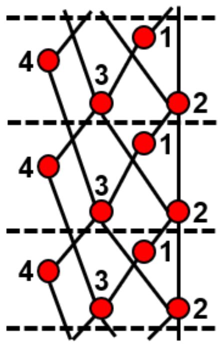

(6x**1**, 1x**2**, 2x$\mathbf{2}^{2}$) **a-5**

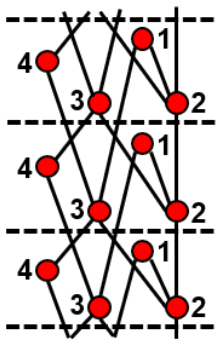

(6x**1**, 1x**2**, 2x$\mathbf{2}^{2}$) **a-6**

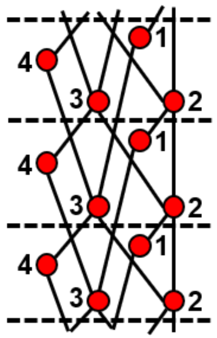

(6x**1**, 1x**2**, 2x$\mathbf{2}^{2}$) **b-1**

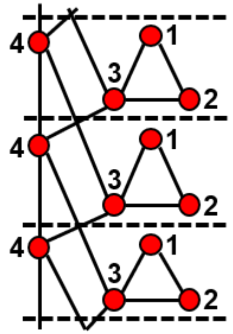

(6x**1**, 1x**2**, 2x$\mathbf{2}^{2}$) **b-2**

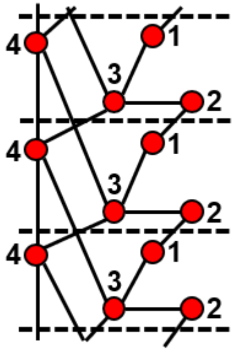

(6x**1**, 1x**2**, 2x$\mathbf{2}^{2}$) **b-3**

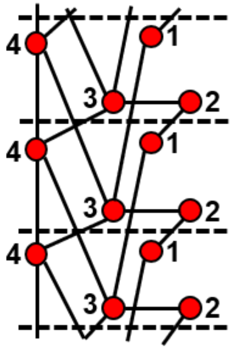

(6x**1**, 1x**2**, 2x$\mathbf{2}^{2}$) **b-4**

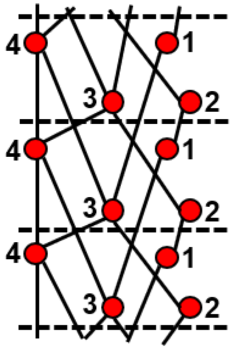

(4x**2**, 2x$\mathbf{2}^{1}$)

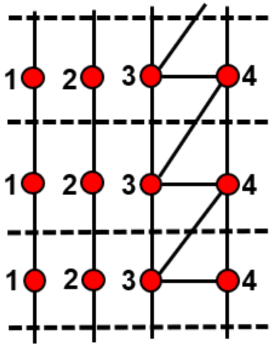

	(4x**2**, 2x**2**²)	(2x**2**, 4x**2**¹) **b**	(2x**2**, 4x**2**²) **c**	(6x**2**¹)
	(4x**2**¹, 2x**2**²) **a**	(4x**2**¹, 2x**2**²) **b**	(2x**2**¹, 4x**2**²) **a**	(2x**2**¹, 4x**2**²) **b**

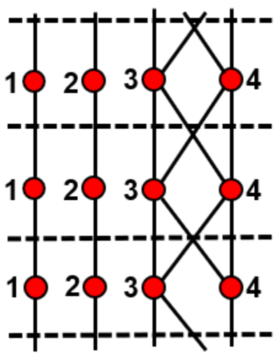

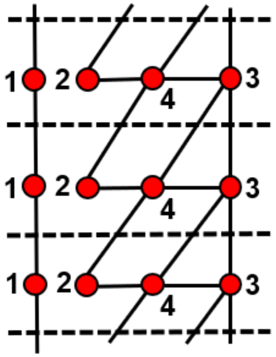

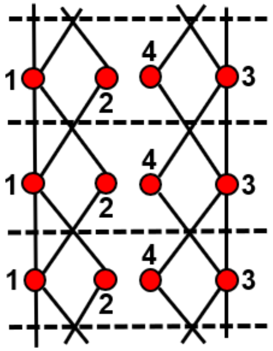

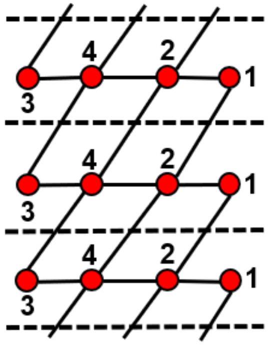

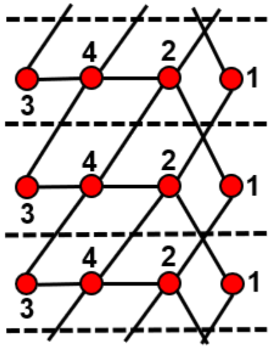

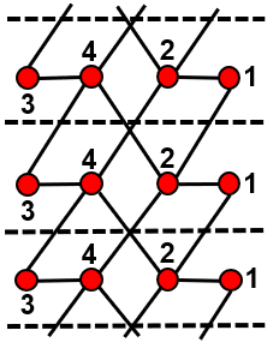

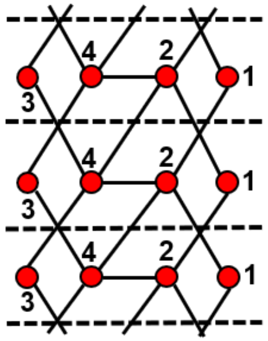

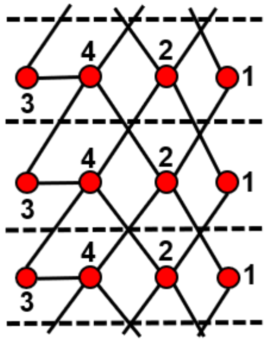

	(6x**2**^{2}) 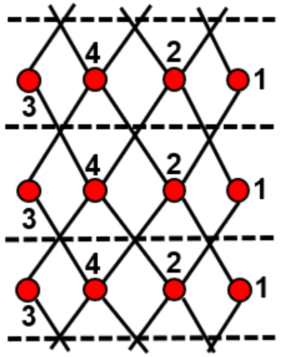			
$^2V_2{}^4V_3$	NG			
$^2V_2{}^4V_4$	NG			
$^2V_2{}^4V_5$	NG			
$^2V_2{}^4V_6$	NG			
$^2V_3{}^4V_1$	(8x**1**, 1x**2**) **a** 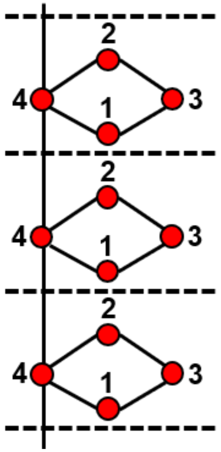	(8x**1**, 1x**2**) **b** 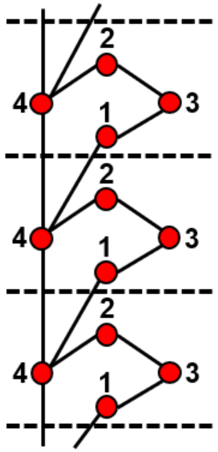	(8x**1**, 1x**2**) **c** 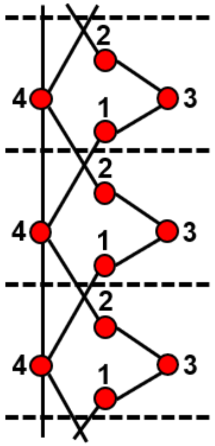	(8x**1**, 1x**2**) **d** 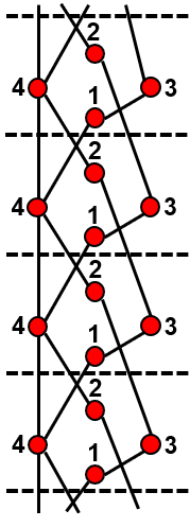

	(8x**1**, 1x**2**) **e** 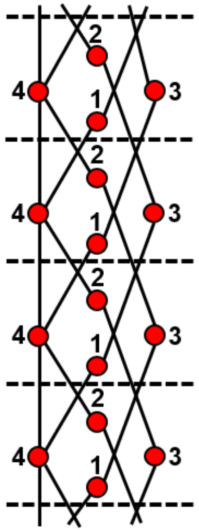	(6x**1**, 2x**2**) **a** 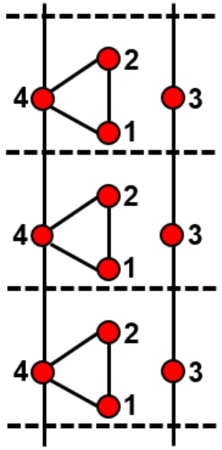	(6x**1**, 2x**2**) **b** 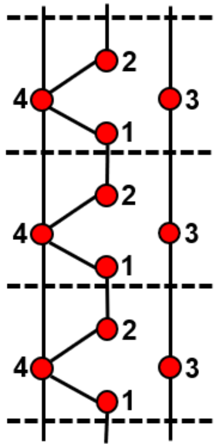	(6x**1**, 2x**2**) **c** 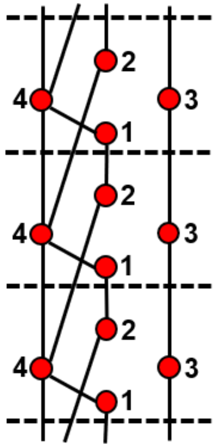
	(6x**1**, 2x**2**) **d** 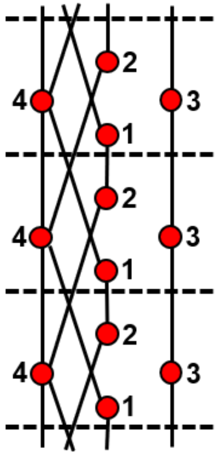	(6x**1**, 2x**2**^{1}) **a** 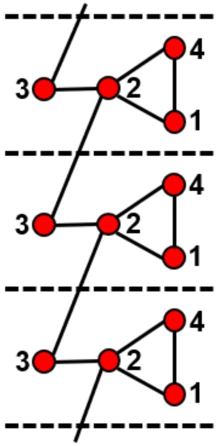	(6x**1**, 2x**2**^{1}) **b** 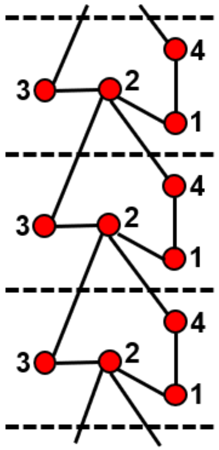	(6x**1**, 2x**2**^{1}) **c** 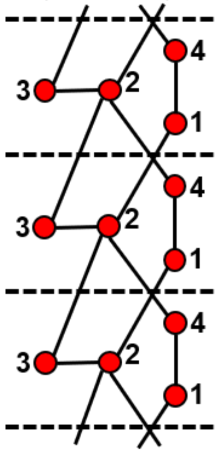

	(6x**1**, 2x**2**¹) **d** 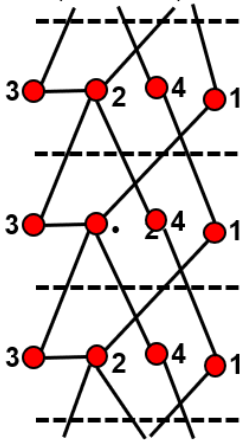	(6x**1**, 2x**2**²) **a** 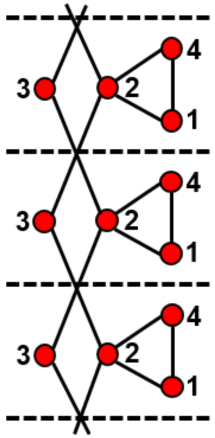	(6x**1**, 2x**2**²) **b** 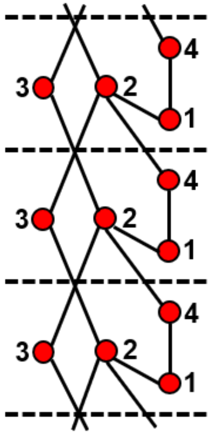	(6x**1**, 2x**2**²) **c** 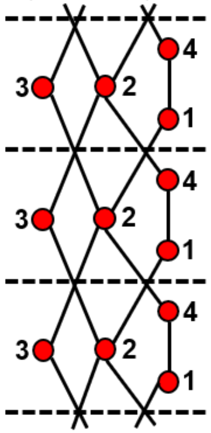
	(6x**1**, 2x**2**²) **d** 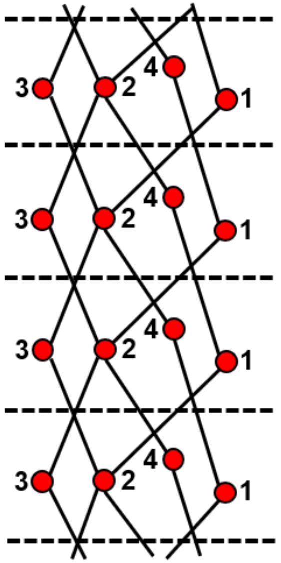	(3x**2**, 2x**2**¹) 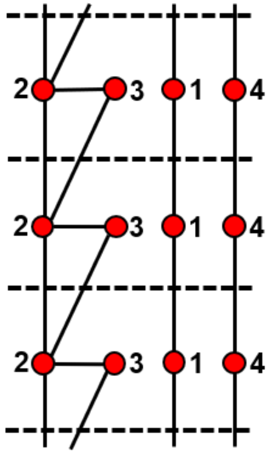	(3x**2**, 2x**2**²) 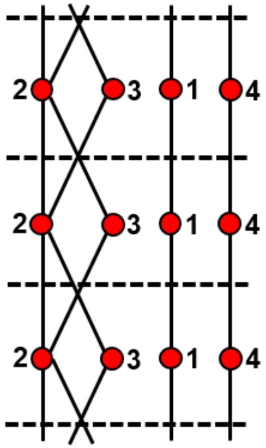	(1x**2**, 4x**2**¹) **b** 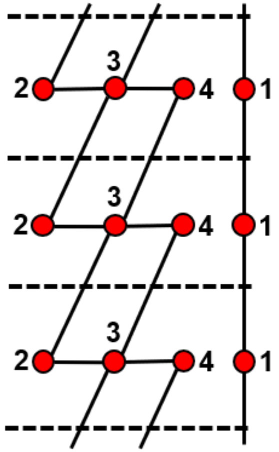

	(1x**2**, 2x**2**[1], 2x**2**[2]) **c**<br>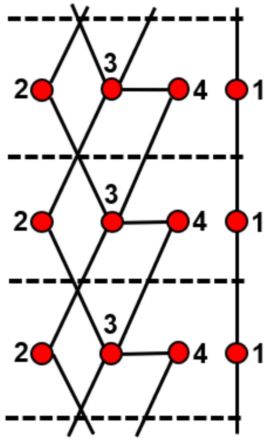	(1x**2**, 4x**2**[2]) **b**<br>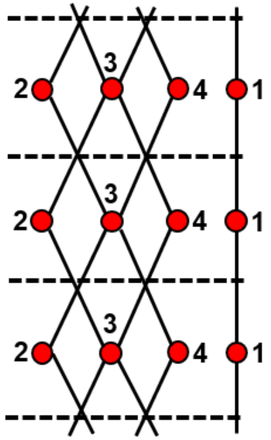		
$^{2}V_{3}{}^{4}V_{2}$	NG			
$^{2}V_{3}{}^{4}V_{3}$	NG			
$^{2}V_{3}{}^{4}V_{4}$	NG			
$^{2}V_{3}{}^{4}V_{5}$	NG			
$^{2}V_{4}{}^{4}V_{1}$	(12x**1**) **a** 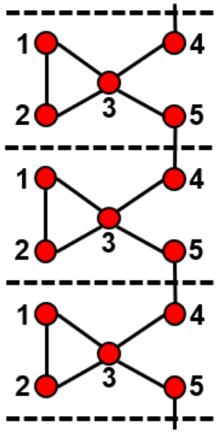	(12x**1**) **b** 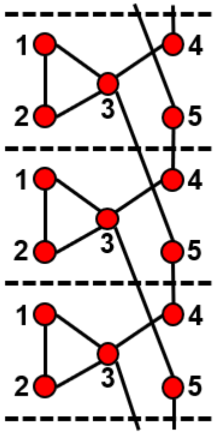	(12x**1**) **c** 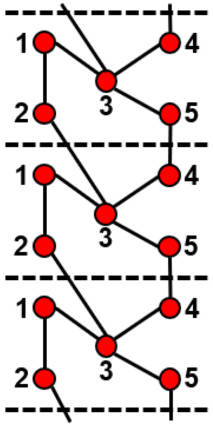	(12x**1**) **d** 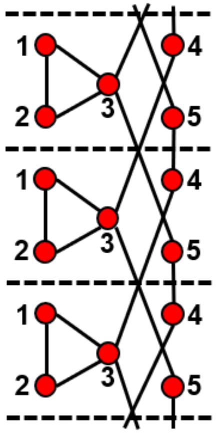

<table>
<tr>
<td></td>
<td>(12x1) e
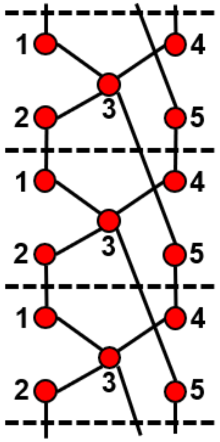

</td>
<td>(12x1) f
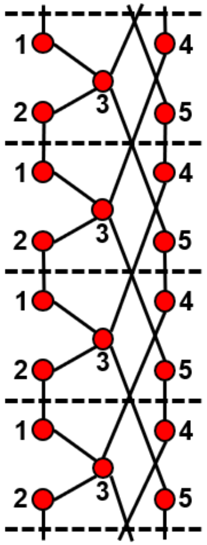

</td>
<td>(12x1) g
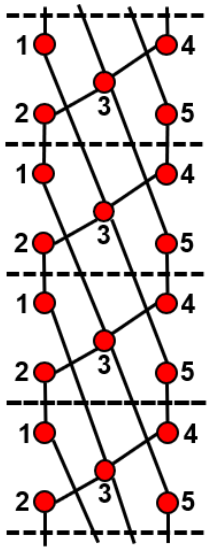

</td>
<td>(12x1) h
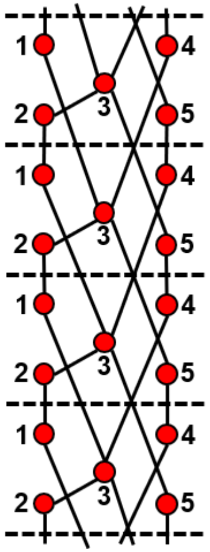

</td>
</tr>
</table>

	(12x**1**) **i**<br>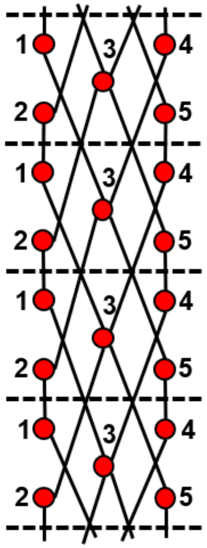 	(10x**1**, 1x**2**) **a**<br>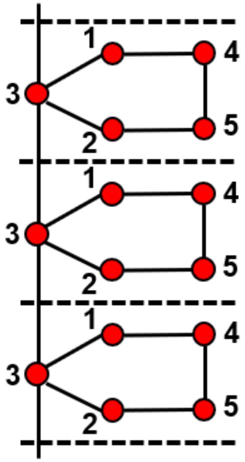 	(10x**1**, 1x**2**) **b**<br>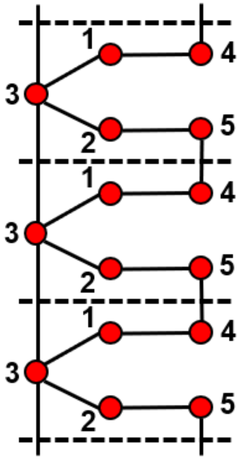 	(10x**1**, 1x**2**) **c**<br>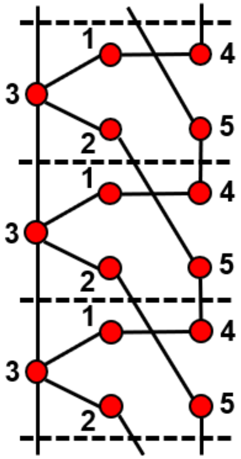

	(10x**1**, 1x**2**) **d**<br>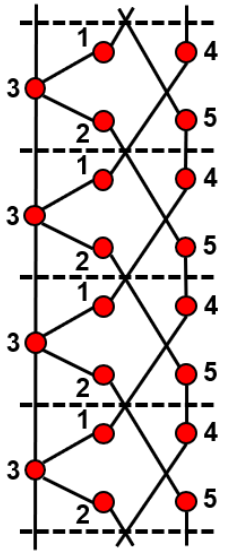 	(10x**1**, 1x**2**) **e**<br>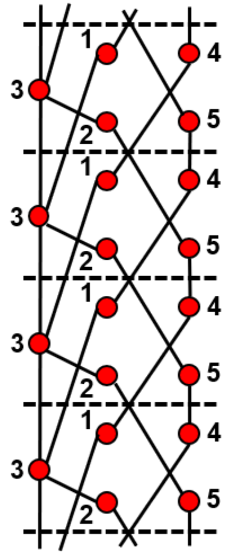 	(10x**1**, 1x**2**) **f**<br>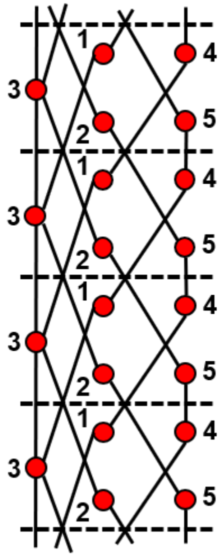 	(8x**1**, 2x**2**) **a**<br>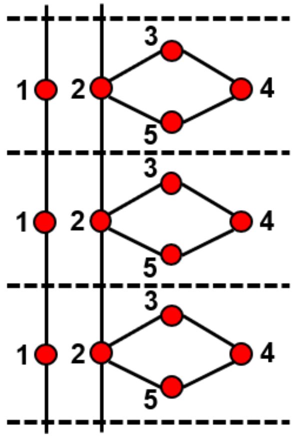

	(8x**1**, 2x**2**) **b** 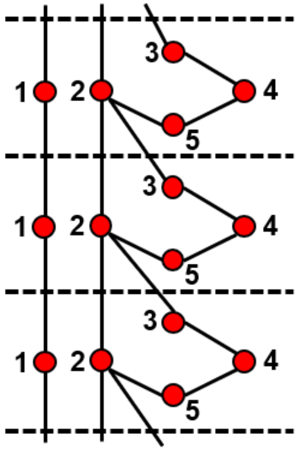	(8x**1**, 2x**2**) **c** 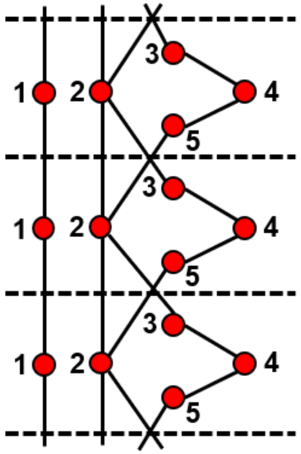	(8x**1**, 2x**2**) **d** 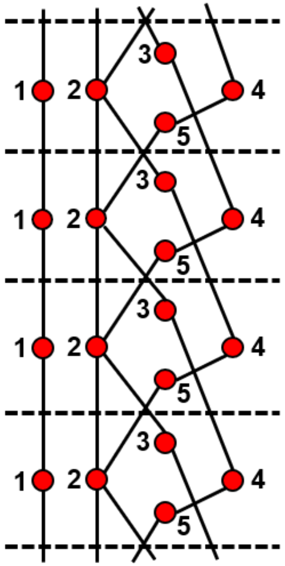	(8x**1**, 2x**2**) **e** 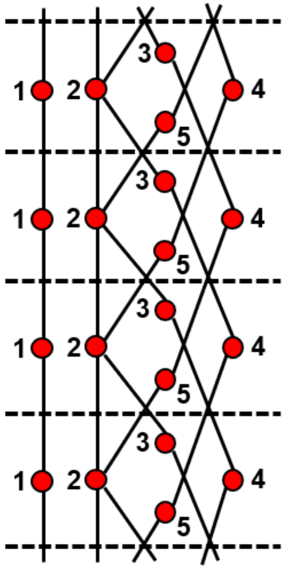

	(8x**1**, 2x**2**^{1}) **a**<br>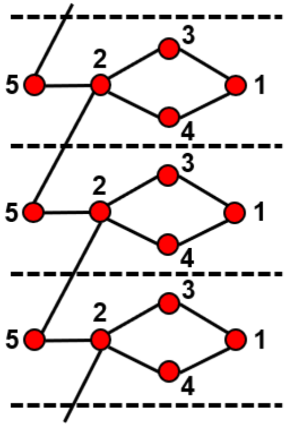	(8x**1**, 2x**2**^{1}) **b**<br>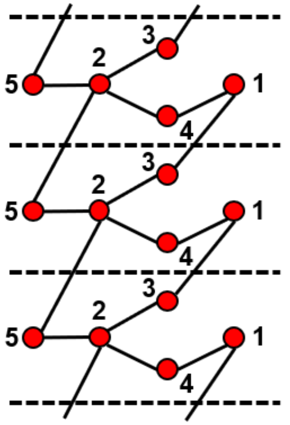	(8x**1**, 2x**2**^{1}) **c**<br>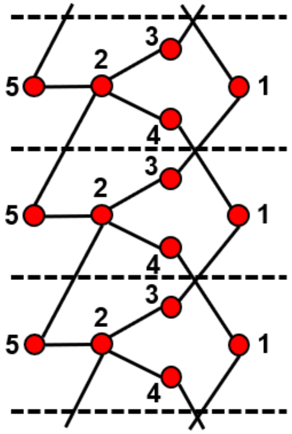	(8x**1**, 2x**2**^{1}) **d**<br>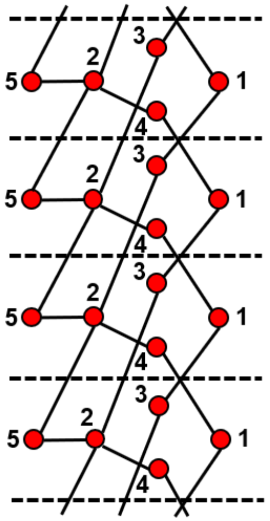
	(8x**1**, 2x**2**^{1}) **e**<br>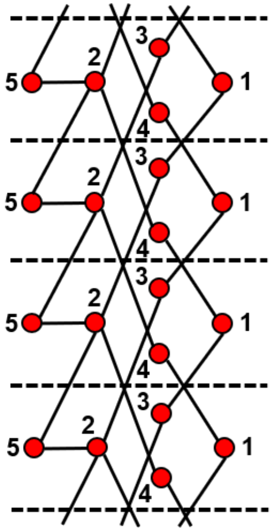	(8x**1**, 2x**2**^{2}) **a**<br>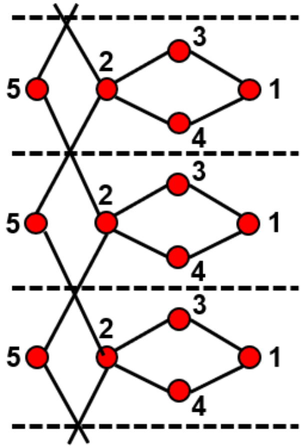	(8x**1**, 2x**2**^{2}) **b**<br>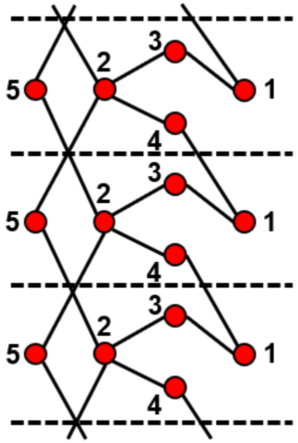	(8x**1**, 2x**2**^{2}) **c**<br>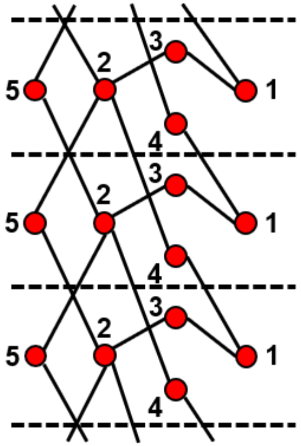

	(8x**1**, 2x**2**^{2}) **d**<br>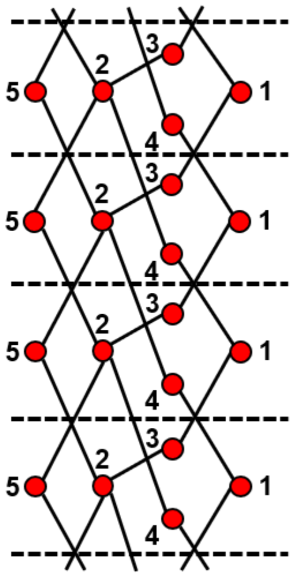 	(8x**1**, 2x**2**^{2}) **e**<br>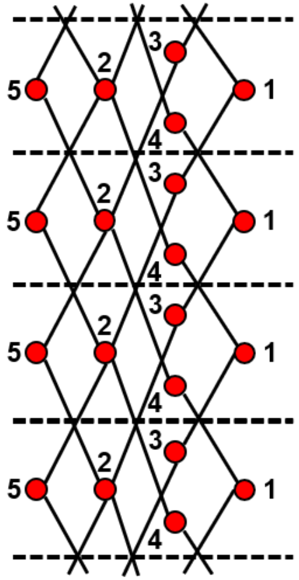 	(6x**1**, 3x**2**) **a**<br>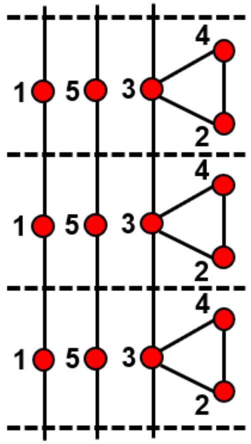 	(6x**1**, 3x**2**) **b**<br>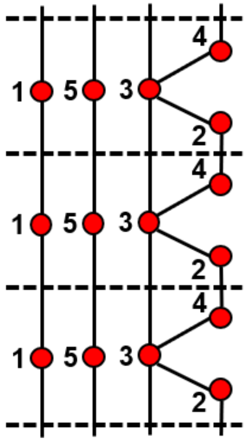

	(6x**1**, 3x**2**) **c** 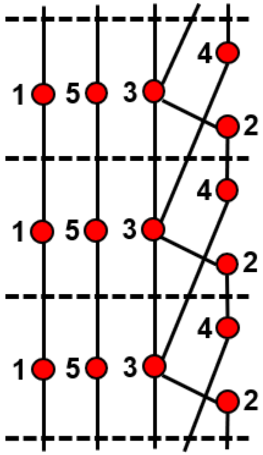	(6x**1**, 3x**2**) **d** 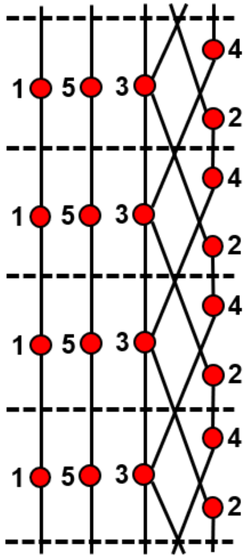	(6x**1**, 1x**2**, 2x$\mathbf{2}^1$) **b-1** 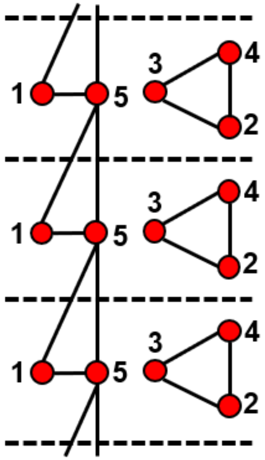	(6x**1**, 1x**2**, 2x$\mathbf{2}^2$) **b-1** 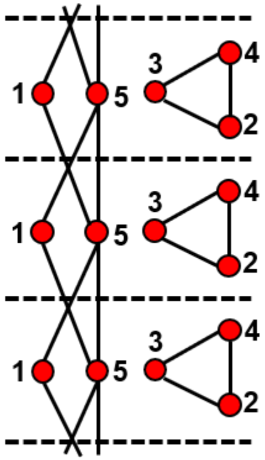
	(6x**1**, 1x**2**, 2x$\mathbf{2}^1$) **c-1** 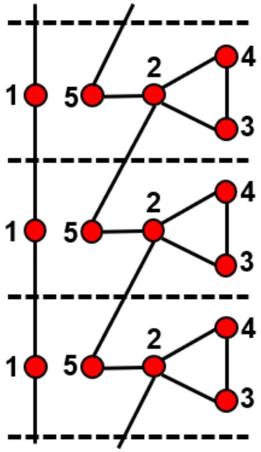	(6x**1**, 1x**2**, 2x$\mathbf{2}^1$) **c-2** 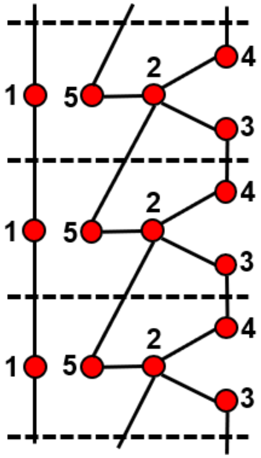	(6x**1**, 1x**2**, 2x$\mathbf{2}^1$) **c-3** 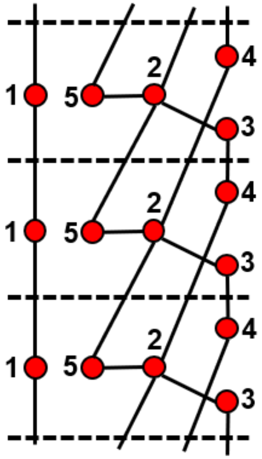	(6x**1**, 1x**2**, 2x$\mathbf{2}^1$) **c-4** 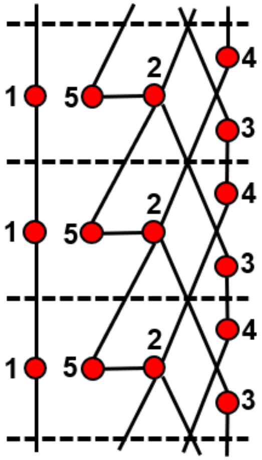

	(6x**1**, 1x**2**, 2x**2**^{2}) **c-1** 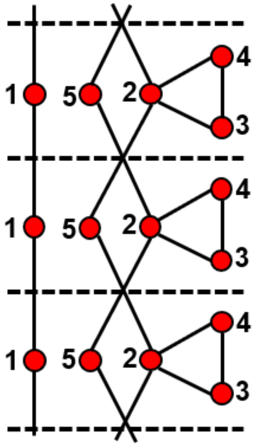	(6x**1**, 1x**2**, 2x**2**^{2}) **c-2** 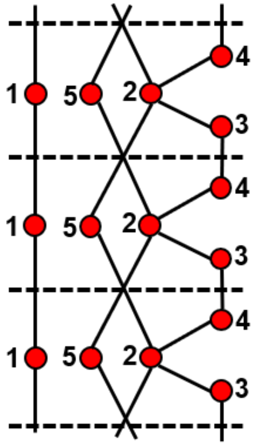	(6x**1**, 1x**2**, 2x**2**^{2}) **c-3** 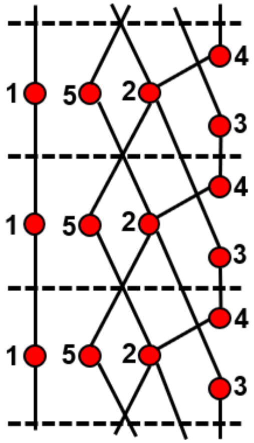	(6x**1**, 1x**2**, 2x**2**^{2}) **c-4** 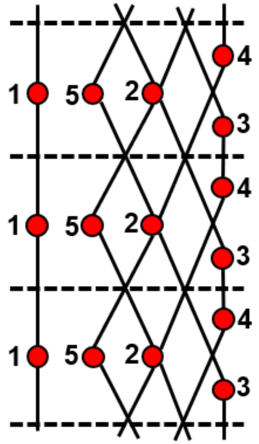
	(4x**2**, 2x**2**^{1}) 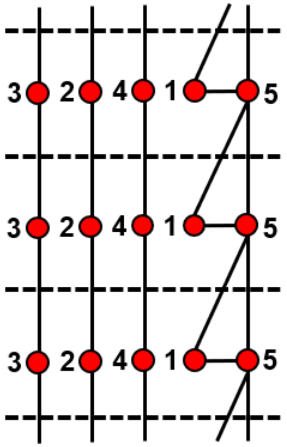	(4x**2**, 2x**2**^{2}) 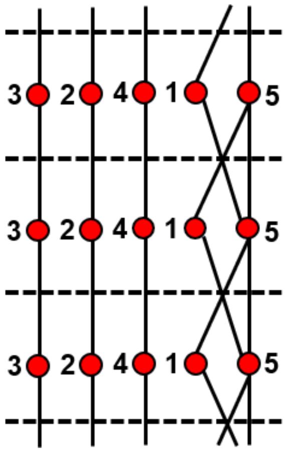	(2x**2**, 4x**2**^{1}) **b** 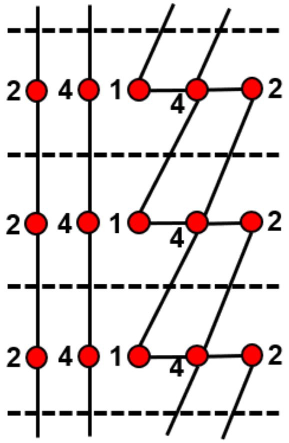	(2x**2**, 2x**2**^{1}, 2x**2**^{2}) **c** 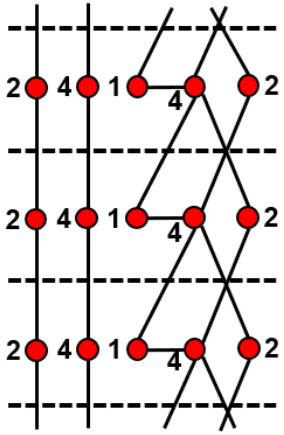

	(2x**2**, 4x**2**^{2}) **b** 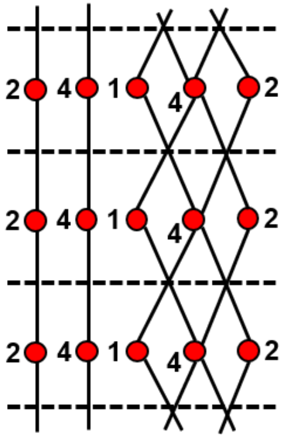			
$^2V_4{}^4V_2$	NG			
$^2V_4{}^4V_3$	NG			
$^2V_4{}^4V_4$	NG			
$^2V_5{}^4V_1$	NG			
$^2V_5{}^4V_2$	NG			
$^2V_5{}^4V_3$	NG			
$^2V_6{}^4V_1$	NG			
$^2V_6{}^4V_2$	NG			
**$^3V_r{}^4V_r$**				

$^3V_2{}^4V_1$	(4x**1**, 3x**2**) 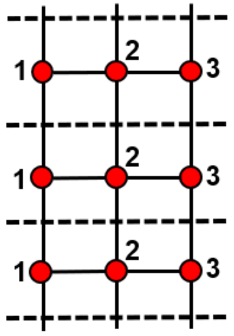	(4x**1**, 1x**2**, 2x$\mathbf{2}^1$) **a** 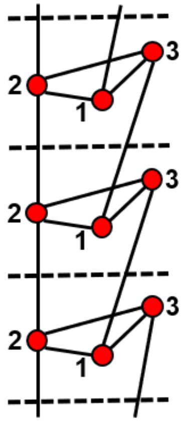	(4x**1**, 1x**2**, 2x$\mathbf{2}^1$) **b** 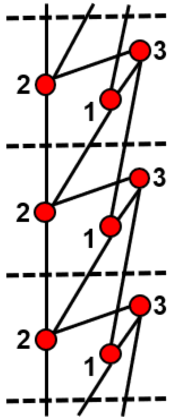	(4x**1**, 1x**2**, 2x$\mathbf{2}^1$) **c** 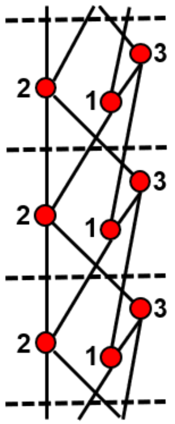
	(4x**1**, 1x**2**, 2x$\mathbf{2}^2$) **a** 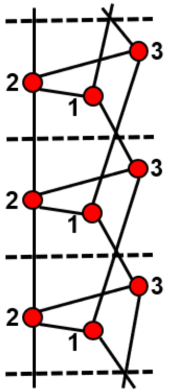	(4x**1**, 1x**2**, 2x$\mathbf{2}^2$) **b** 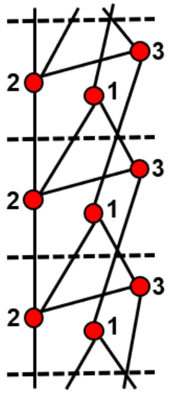	(4x**1**, 1x**2**, 2x$\mathbf{2}^2$) **c** 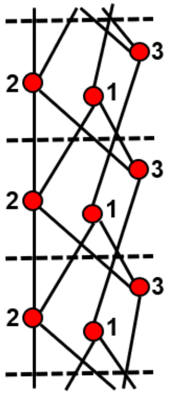	(2x**1**, 2x**2**, 2x$\mathbf{2}^1$) 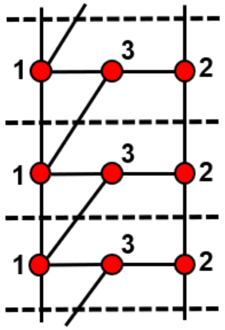

	(2x**1**, 2x**2**, 2x**2**^{2})<br>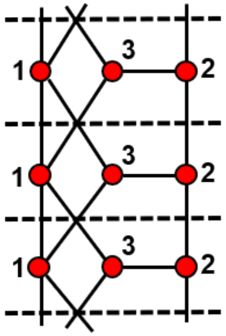	(2x**1**, 4x**2**^{1}) **a**<br>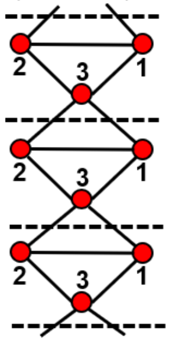	(2x**1**, 4x**2**^{1}) **b**<br>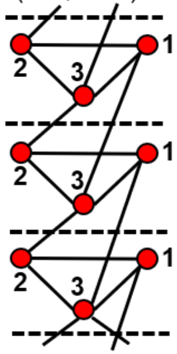	(2x**1**, 4x**2**^{1}) **c**<br>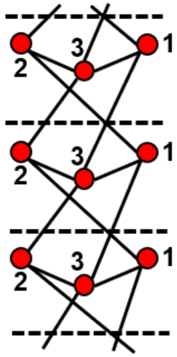
	(2x**1**, 2x**2**^{1}, 2x**2**^{2}) **a**<br>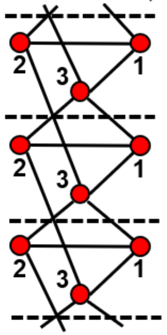	(2x**1**, 2x**2**^{1}, 2x**2**^{2}) **b**<br>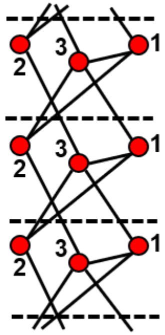	(2x**1**, 4x**2**^{2}) **a**<br>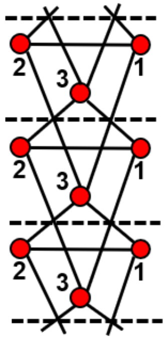	(2x**1**, 4x**2**^{2}) **b**<br>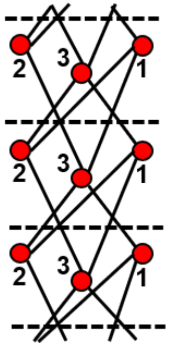
$^{3}V_{2}{}^{4}V_{2}$	NG			
$^{3}V_{2}{}^{4}V_{3}$	NG			
$^{3}V_{2}{}^{4}V_{4}$	NG			
$^{3}V_{2}{}^{4}V_{5}$	NG			
$^{3}V_{2}{}^{4}V_{6}$	NG			
$^{3}V_{4}{}^{4}V_{1}$	NG			
$^{3}V_{4}{}^{4}V_{2}$	NG			

$^{3}V_{4}{}^{4}V_{3}$	NG			
$^{3}V_{4}{}^{4}V_{4}$	NG			
$^{3}V_{6}{}^{4}V_{1}$	NG			
$^{3}V_{6}{}^{4}V_{2}$	NG			
**Rank 3**				
$\mathbf{^{1}V_{r}{}^{2}V_{r}{}^{3}V_{r}}$				
$^{1}V_{1}{}^{2}V_{1}{}^{3}V_{1}$	(4x**1**, 1x**2**) 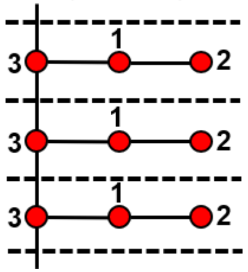	(2x**1**, 2x**2**) 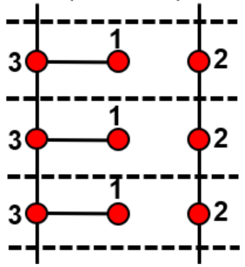	(2x**1**, 2x**2**[1]) 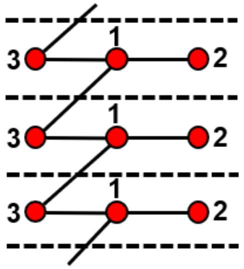	(2x**1**, 2x**2**[2]) 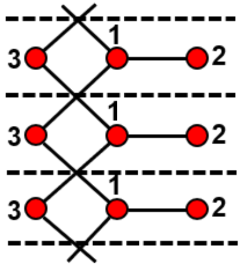
$^{1}V_{1}{}^{2}V_{1}{}^{3}V_{3}$	NG			
$^{1}V_{1}{}^{2}V_{1}{}^{3}V_{5}$	NG			
$^{1}V_{1}{}^{2}V_{2}{}^{3}V_{1}$	(8x**1**) **a** 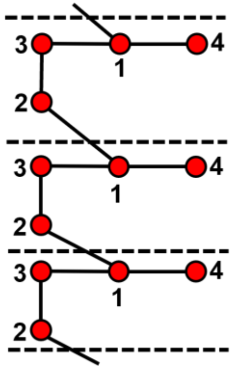	(8x**1**) **b** 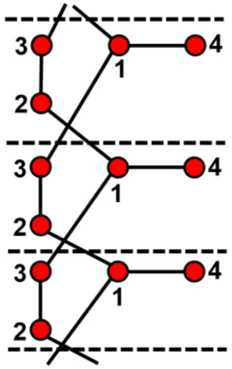	(8x**1**) **c** 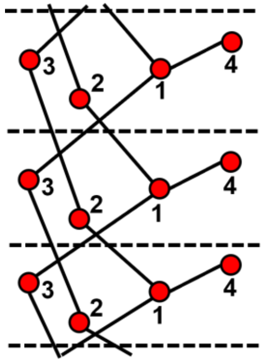	(6x**1**, 1x**2**) 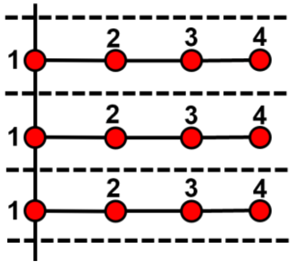

	(4x**1**, 2x**2**) 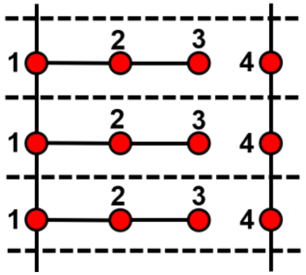	(4x**1**, 2x$\mathbf{2}^1$) 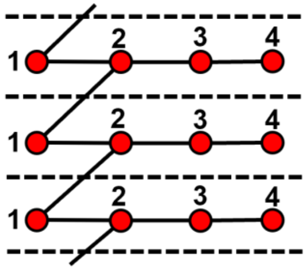	(4x**1**, 2x$\mathbf{2}^2$) 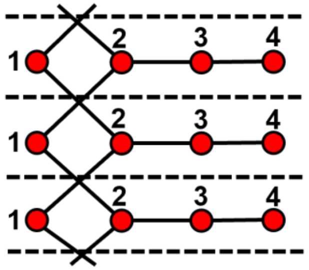	(2x**1**, 3x**2**) 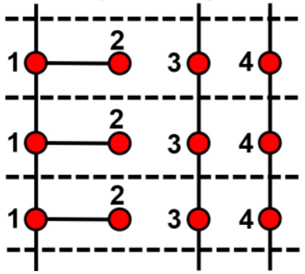
	(2x**1**, 1x2, 2x$\mathbf{2}^1$) **a** 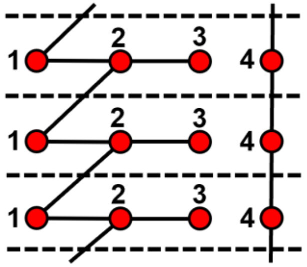	(2x**1**, 1x2, 2x$\mathbf{2}^2$) **a** 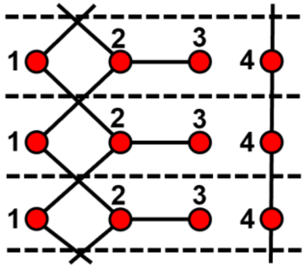		
$^1V_1{}^2V_2{}^3V_3$	NG			
$^1V_1{}^2V_2{}^3V_5$	NG			

$^1V_1{}^2V_3{}^3V_1$	(10x**1**) **a-1** 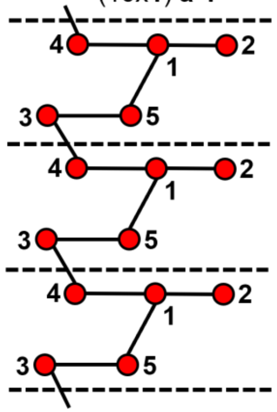	(10x**1**) **a-2** 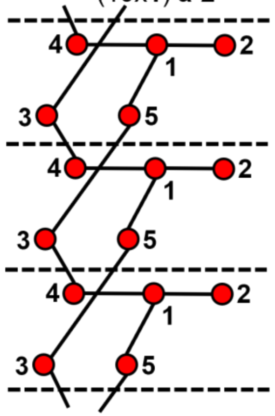	(10x**1**) **a-3** 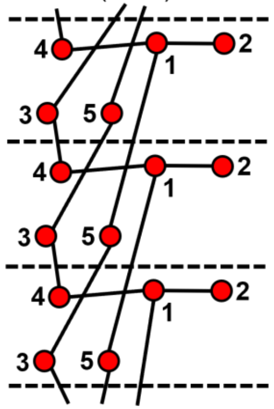	(10x**1**) **a-4** 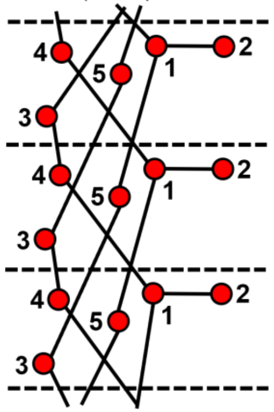
	(10x**1**) **b-1** 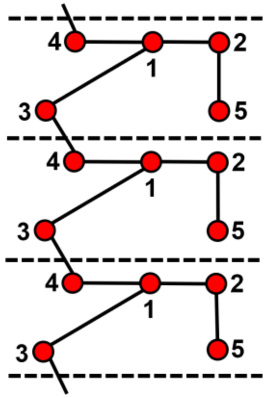	(10x**1**) **b-2** 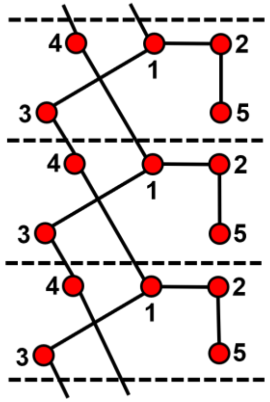	(10x**1**) **b-3** 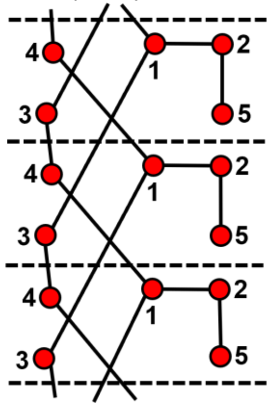	(8x**1**, 1x**2**) **a-1** 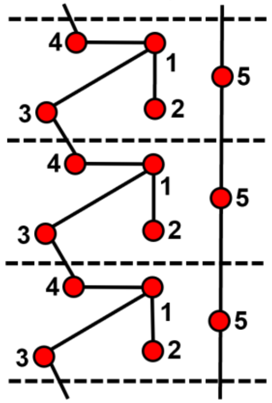

	(8x**1**, 1x**2**) **a-2** 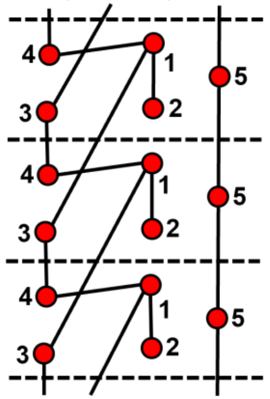	(8x**1**, 1x**2**) **a-3** 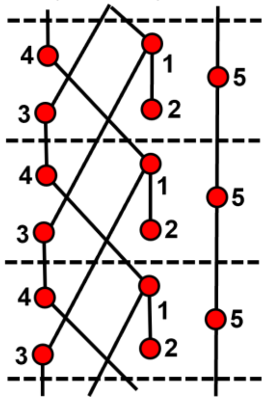	(8x**1**, 1x**2**) **b-1** 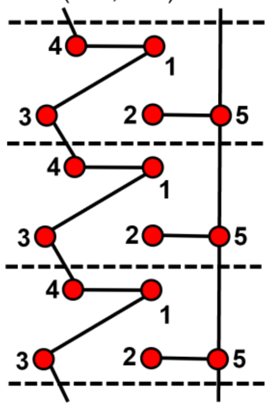	(8x**1**, 1x**2**) **b-2** 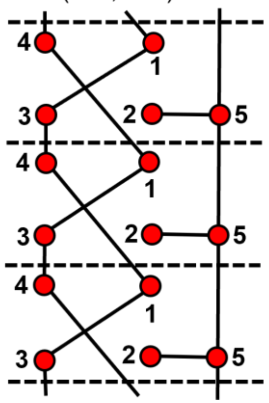
	(8x**1**, 1x**2**) **b-3** 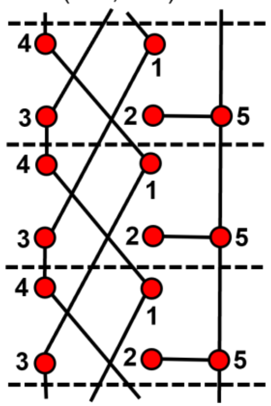	(8x**1**, 1x**2**) **c** 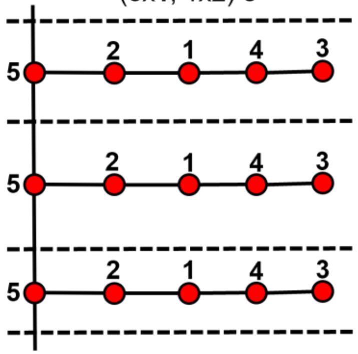	(6x**1**, 2x**2**) 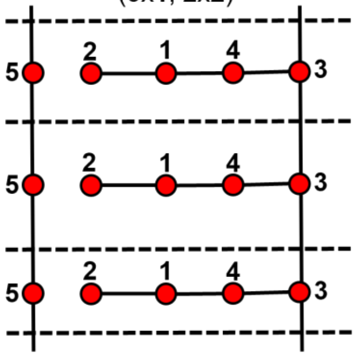	(6x**1**, 2x$\mathbf{2}^1$) 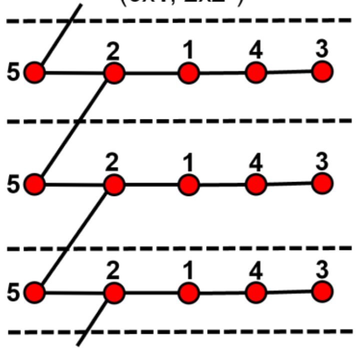

(6x**1**, 2x$\mathbf{2}^{2}$)

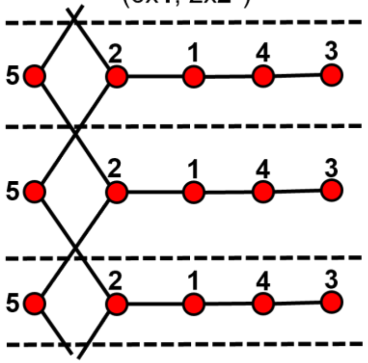

(4x**1**, 3x**2**)

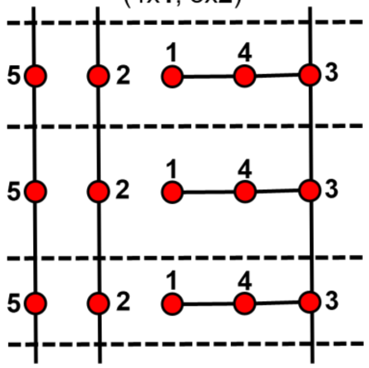

(4x**1**, 1x**2**, 2x$\mathbf{2}^{1}$) **b**

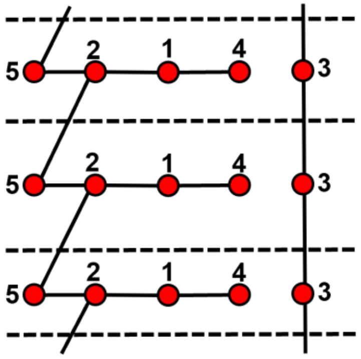

(4x**1**, 1x**2**, 2x$\mathbf{2}^{2}$) **a**

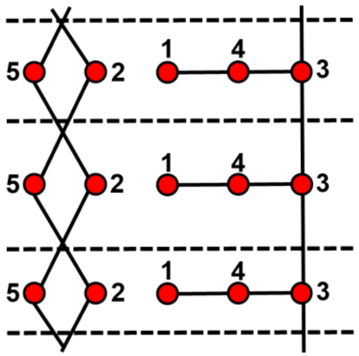

(4x**1**, 1x**2**, 2x$\mathbf{2}^{2}$) **b**

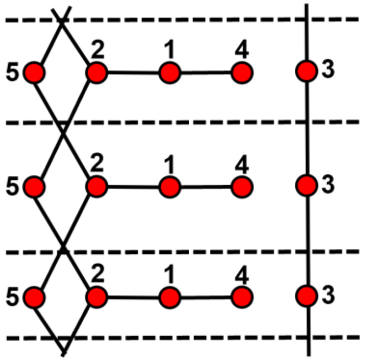

(2x**1**, 4x**2**)

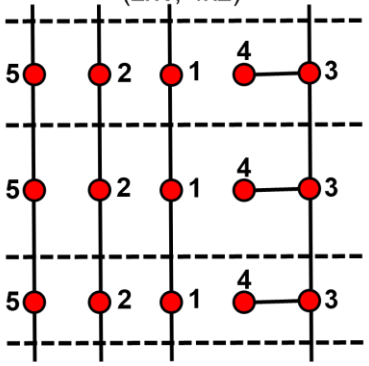

(2x**1**, 2x**2**, 2x$\mathbf{2}^{1}$) **b**

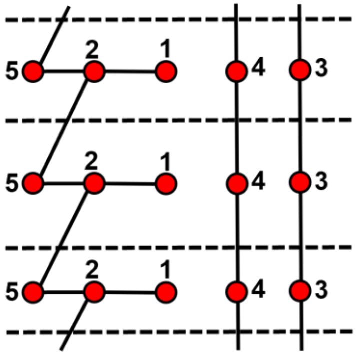

(2x**1**, 2x**2**, 2x$\mathbf{2}^{2}$) **a**

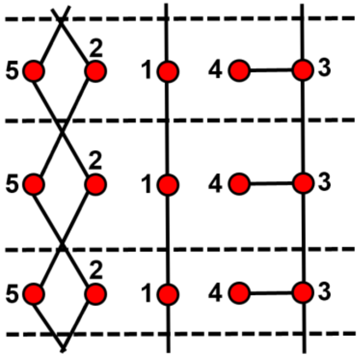

	(2x**1**, 2x**2**, 2x**2**^{2}) **b**<br>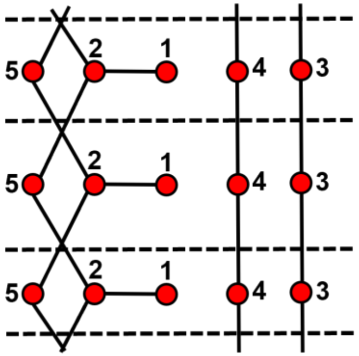	(2x**1**, 2x**2**^{1}, 2x**2**^{2}) **b**<br>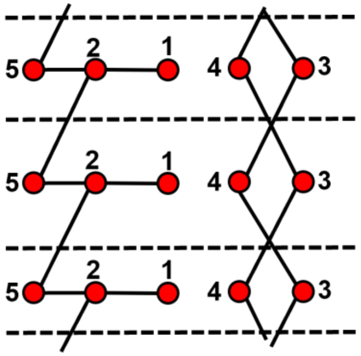	(2x**1**, 4x**2**^{2})<br>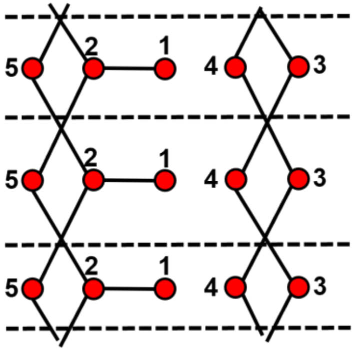	
$^{1}V_{1}{}^{2}V_{3}{}^{3}V_{3}$	NG			
$^{1}V_{1}{}^{2}V_{4}{}^{3}V_{1}$	NG			
$^{1}V_{1}{}^{2}V_{4}{}^{3}V_{3}$	NG			
$^{1}V_{1}{}^{2}V_{5}{}^{3}V_{1}$	NG			
$^{1}V_{1}{}^{2}V_{6}{}^{3}V_{1}$	NG			
$^{1}V_{1}{}^{2}V_{6}{}^{3}V_{3}$	NG			

$^1V_2{}^2V_1{}^3V_2$	(10x**1**) **a** 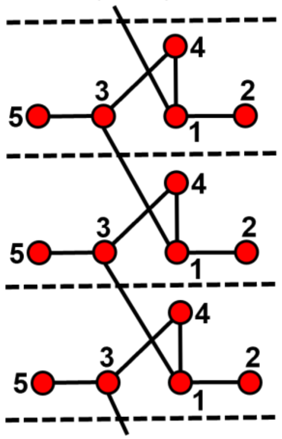	(10x**1**) **b** 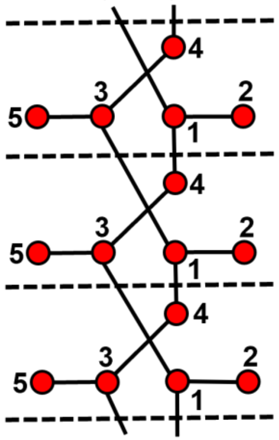	(10x**1**) **c** 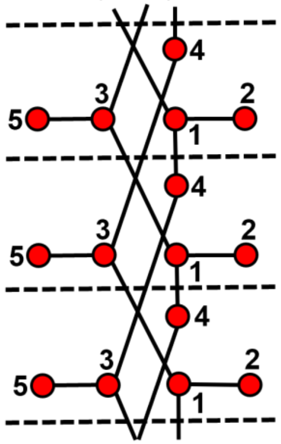	(8x**1**, 1x**2**) **a** 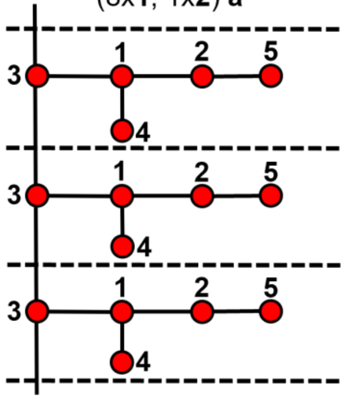
	(8x**1**, 1x**2**) **b** 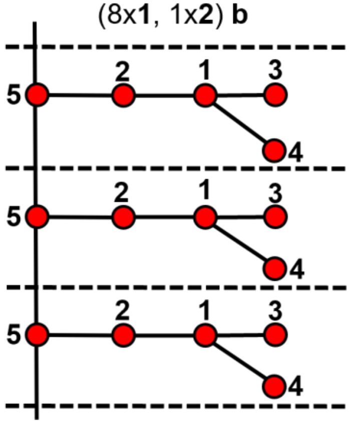	(6x**1**, 2x**2**) **a** 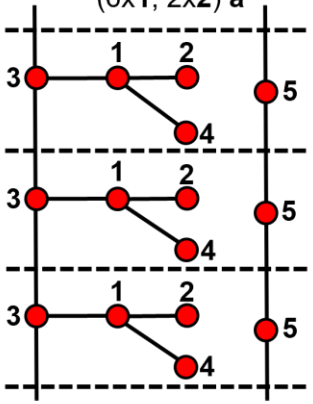	(6x**1**, 2x**2**) **b** 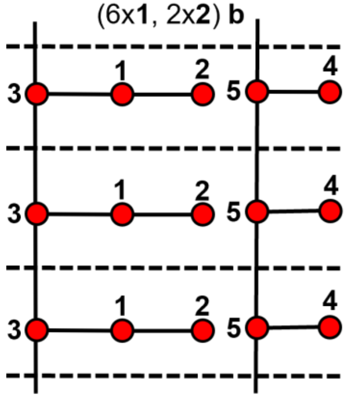	(6x**1**, 2x**2**) **c** 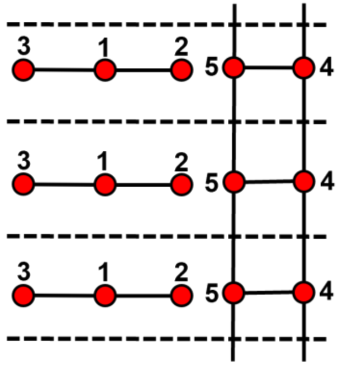

	(6x**1**, 2x**2**) **d** 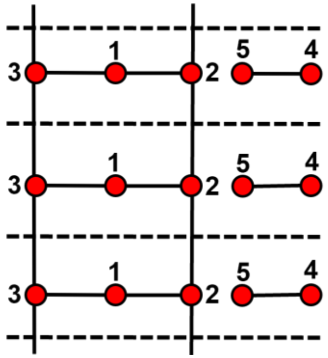	(6x**1**, 2x$\mathbf{2}^1$) **a-1** 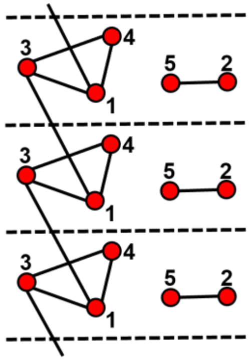	(6x**1**, 2x$\mathbf{2}^1$) **a-2** 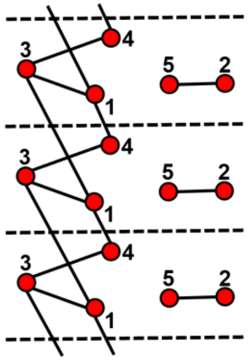	(6x**1**, 2x$\mathbf{2}^1$) **a-3** 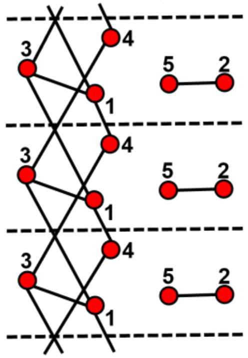
	(6x**1**, 2x$\mathbf{2}^1$) **b** 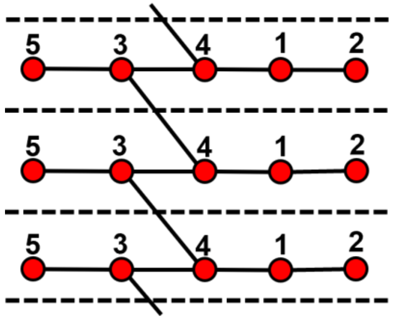	(6x**1**, 2x$\mathbf{2}^1$) **c** 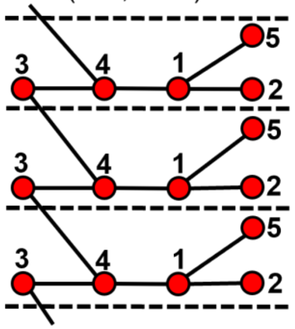	(6x**1**, 2x$\mathbf{2}^2$) **a-1** 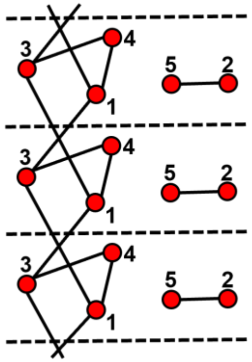	(6x**1**, 2x$\mathbf{2}^2$) **a-2** 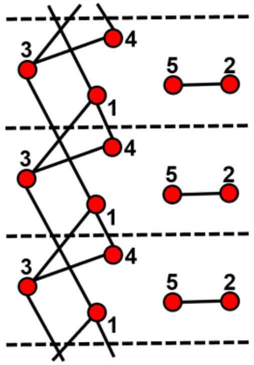

(6x**1**, 2x**2**²) **a-3**

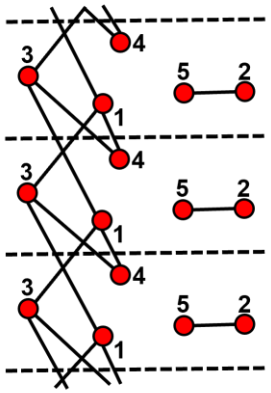

(6x**1**, 2x**2**²) **b**

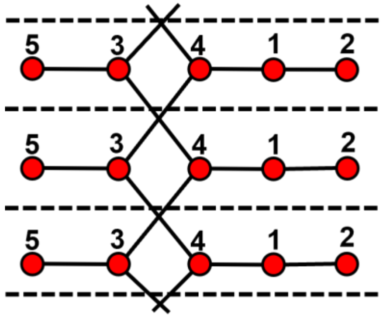

(6x**1**, 2x**2**²) **c**

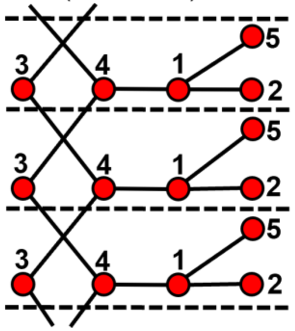

(4x**1**, 3x**2**) **a**

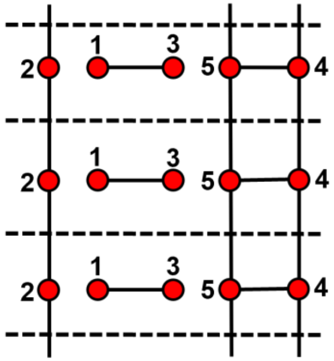

(4x**1**, 3x**2**) **b**

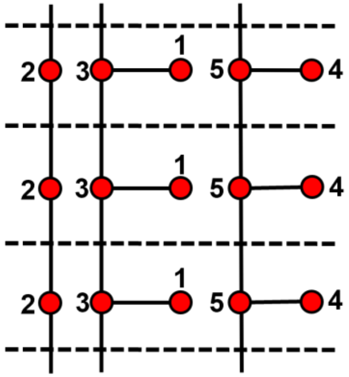

(4x**1**, 1x**2**, 2x**2**¹) **a**

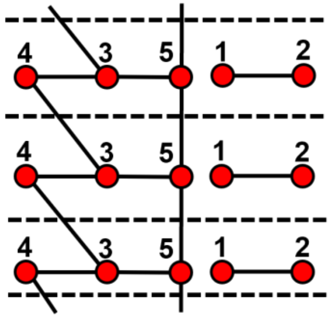

(4x**1**, 1x**2**, 2x**2**¹) **b**

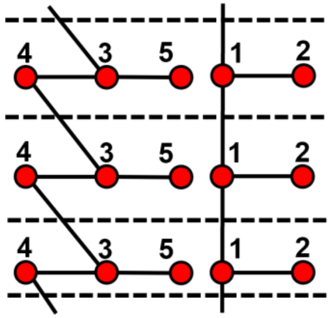

(4x**1**, 1x**2**, 2x**2**²) **a**

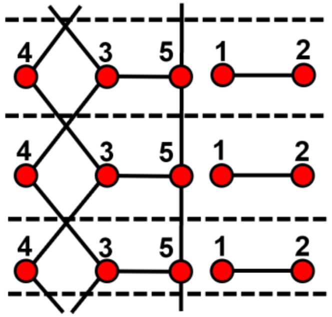

	(4x**1**, 1x**2**, 2x**2**^{2}) **b** 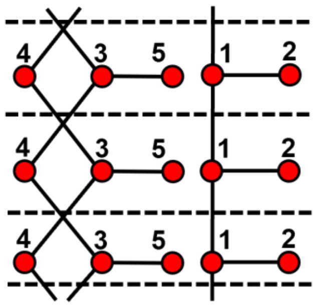	(4x**1**, 1x**2**, 2x**2**^{2}) **c** 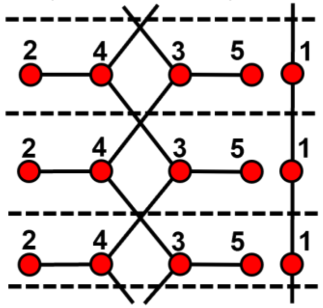		
$^{1}V_{2}{}^{2}V_{1}{}^{3}V_{4}$	NG			
$^{1}V_{2}{}^{2}V_{2}{}^{3}V_{2}$	NG			
$^{1}V_{2}{}^{2}V_{2}{}^{3}V_{4}$	NG			
$^{1}V_{2}{}^{2}V_{3}{}^{3}V_{2}$	NG			
$^{1}V_{2}{}^{2}V_{4}{}^{3}V_{2}$	NG			
$^{1}V_{3}{}^{2}V_{1}{}^{3}V_{1}$	(6x**1**, 1x**2**) **a** 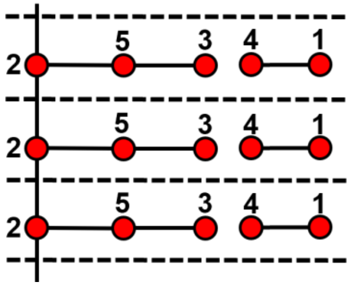	(6x**1**, 1x**2**) **b** 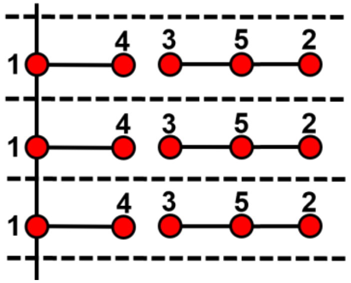	(6x**1**, 1x**2**) **c** 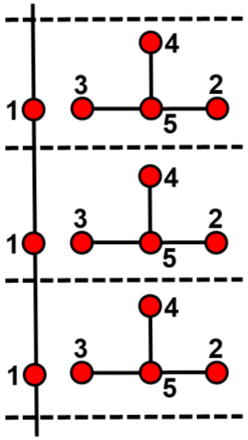	(4x**1**, 2x**2**) 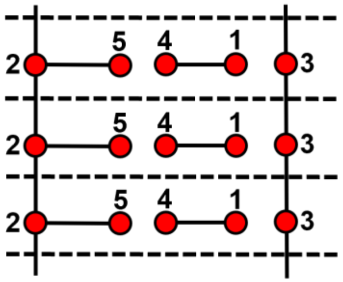

	(4x**1**, 2x**2**^{2})<br>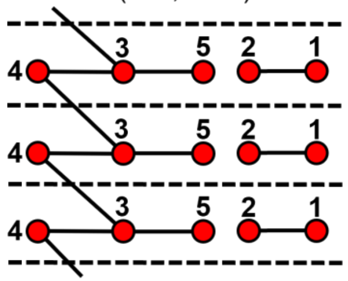	(4x**1**, 2x**2**^{2})<br>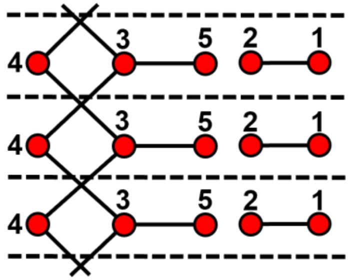		
$^1V_3{}^2V_1{}^3V_3$	NG			
$^1V_3{}^2V_2{}^3V_1$	(10x**1**) **a**<br>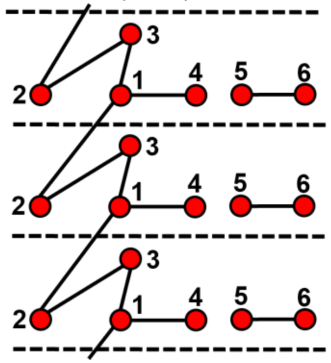	(10x**1**) **b**<br>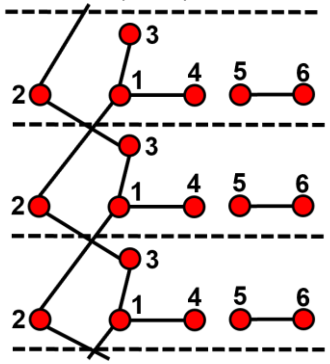	(10x**1**) **c**<br>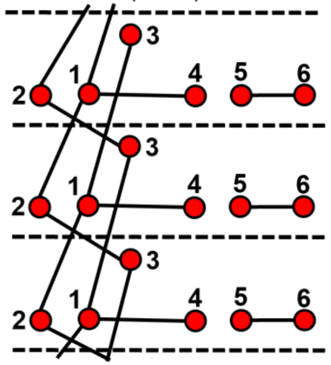	(8x**1**, 1x**2**) **a**<br>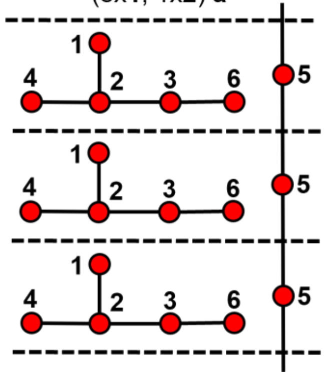

	(8x**1**, 1x**2**) **b**<br>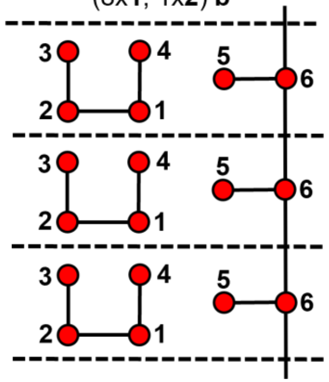	(8x**1**, 1x**2**) **c**<br>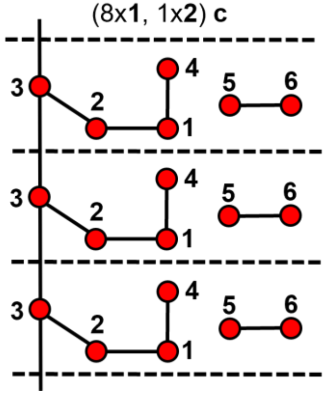	(8x**1**, 1x**2**) **d**<br>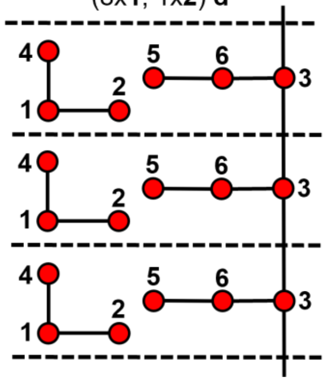	(6x**1**, 2x**2**) **a**<br>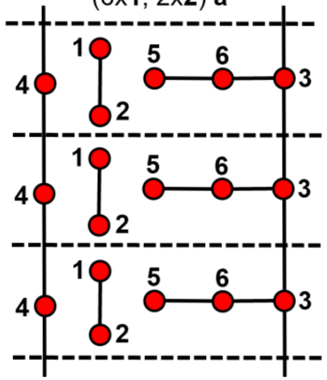
	(6x**1**, 2x**2**) **b**<br>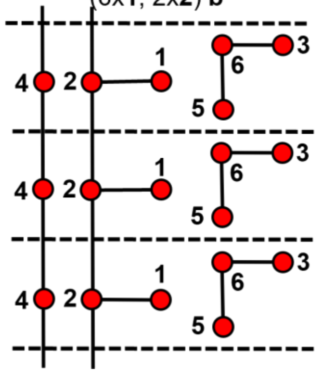	(6x**1**, 2x**2**) **c**<br>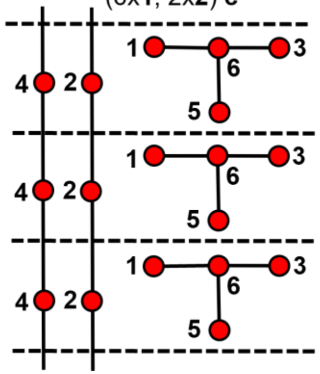	(6x**1**, 2x$\mathbf{2}^1$) **a**<br>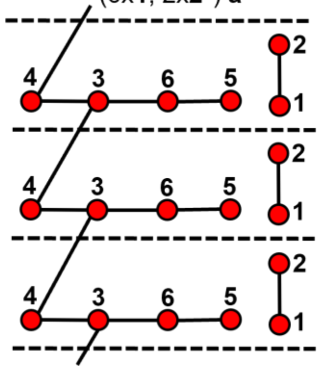	(6x**1**, 2x$\mathbf{2}^1$) **b**<br>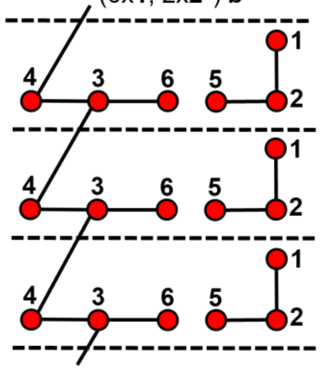

	(6x**1**, 2x$\mathbf{2}^2$) **a** 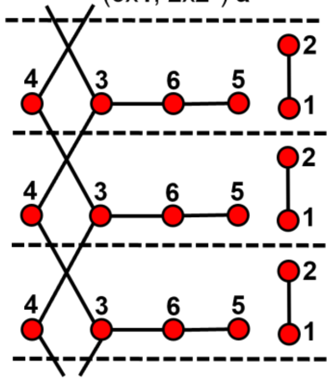	(6x**1**, 2x$\mathbf{2}^2$) **b** 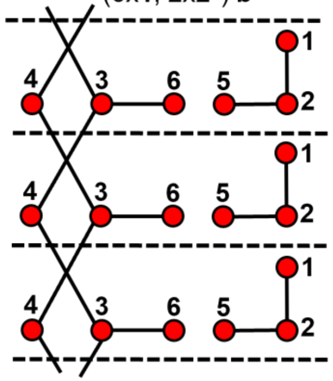	(6x**1**, 2x$\mathbf{2}^2$) **c** 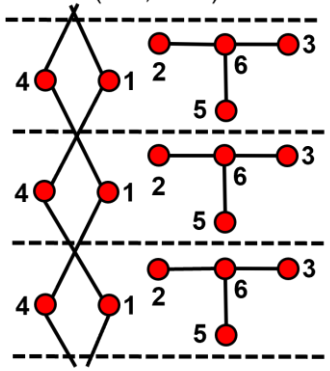	(4x**1**, 3x**2**) 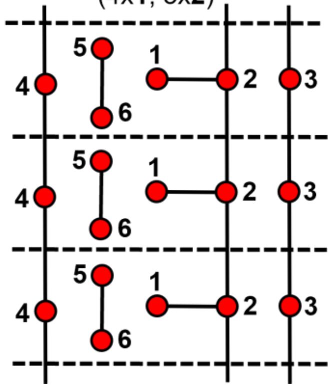
	(4x**1**, 1x**2**, 2x$\mathbf{2}^1$) **a** 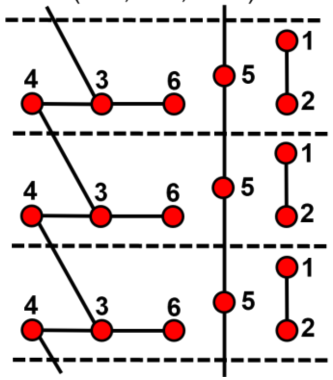	(4x**1**, 1x**2**, 2x$\mathbf{2}^2$) **a** 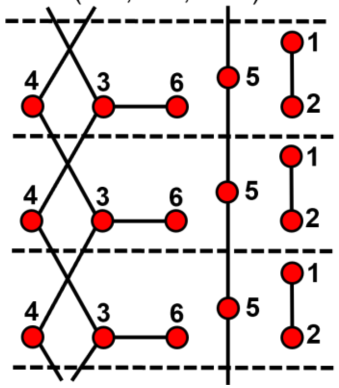	(4x**1**, 1x**2**, 2x$\mathbf{2}^2$) **b** 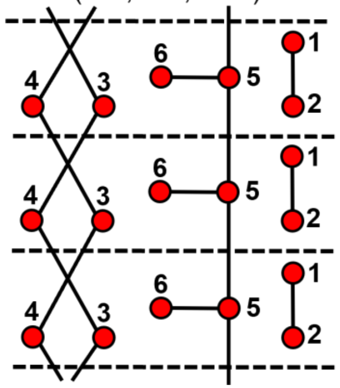	
$^1V_3{}^2V_2{}^3V_3$	NG			
$^1V_3{}^2V_3{}^3V_1$	NG			
$^1V_3{}^2V_4{}^3V_1$	NG			
$^1V_4{}^2V_1{}^3V_2$	NG			
$^1V_4{}^2V_2{}^3V_2$	NG			

$^1V_5{}^2V_1{}^3V_1$	(8x**1**, 1x**2**) **a** 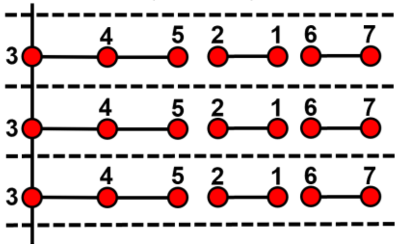	(8x**1**, 1x**2**) **b** 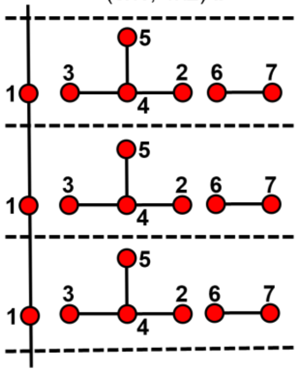	(8x**1**, 1x**2**) **c** 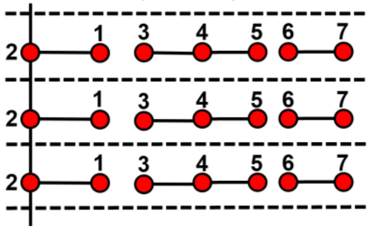	(6x**1**, 2x**2**) 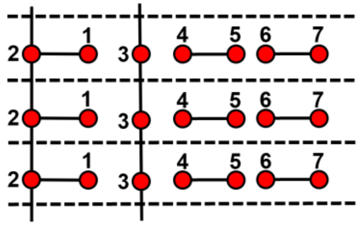
	(6x**1**, 2x**2**^{1}) 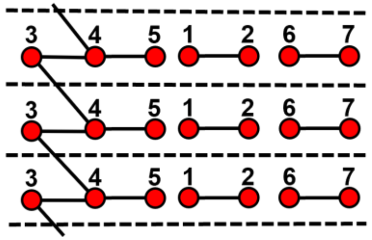	(6x**1**, 2x**2**^{2}) 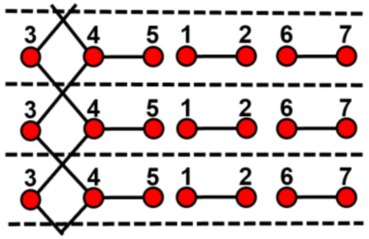		
$^1V_5{}^2V_2{}^3V_1$	NG			
$\mathbf{^1V_r{}^2V_r{}^4V_r}$				
$^1V_2{}^2V_1{}^4V_1$	(6x**1**, 1x**2**) 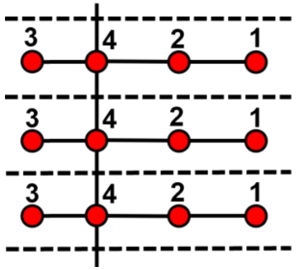	(4x**1**, 2x**2**) 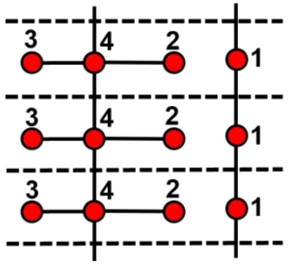	(4x**1**, 2x**2**^{1}) 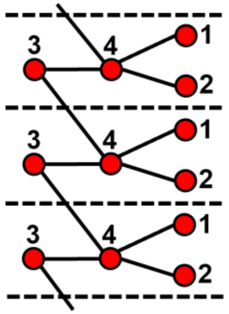	(4x**1**, 2x**2**^{2}) 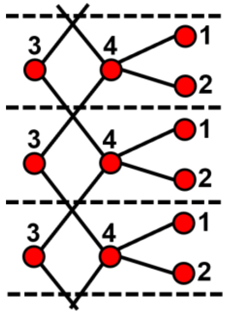

	(2x**1**, 1x**2**, 2x$\mathbf{2}^1$)<br>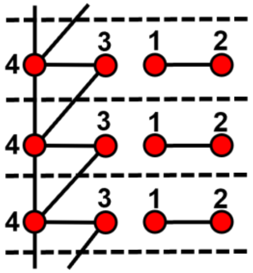 	(2x**1**, 1x**2**, 2x$\mathbf{2}^2$)<br>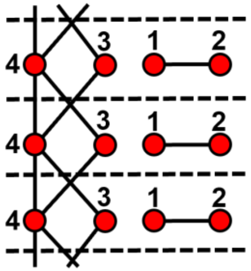 		
$^1V_2{}^2V_1{}^4V_2$	(10x**1**, 1x**2**) **a**<br>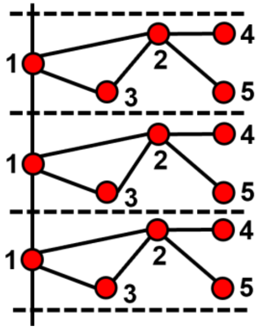 	(10x**1**, 1x**2**) **b**<br>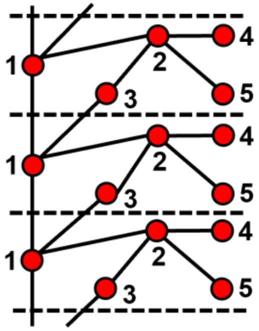 	(10x**1**, 1x**2**) **c**<br>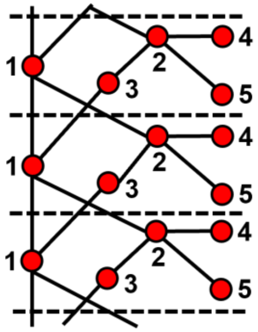 	(10x**1**, 1x**2**) **d**<br>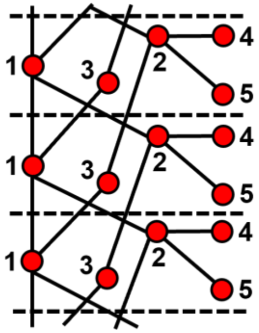
	(8x**1**, 2x**2**) **a**<br>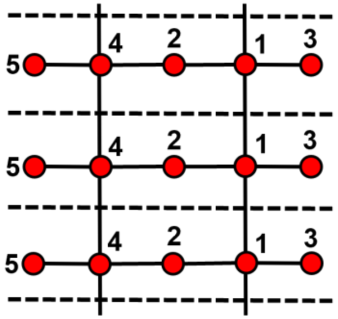 	(8x**1**, 2x2) **b-1**<br>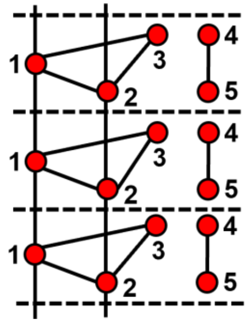 	(8x**1**, 2x**2**) **b-2**<br>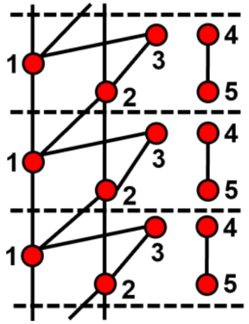 	(8x**1**, 2x**2**) **b-3**<br>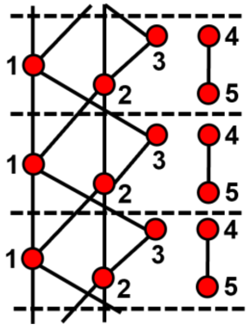

	(8x**1**, 2x**2**) **b-4** 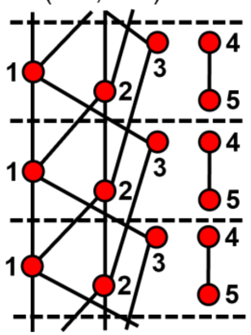	(8x**1**, 2x**2**) **c** 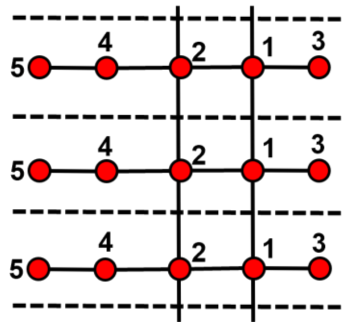	(8x**1**, 2x**2**^{1}) **a** 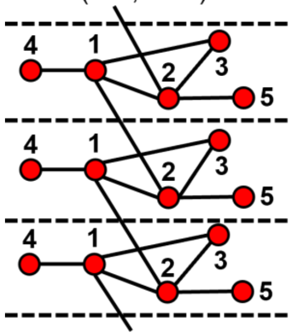	(8x**1**, 2x**2**^{1}) **b** 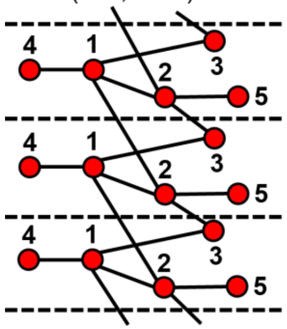
	(8x**1**, 2x**2**^{1}) **c** 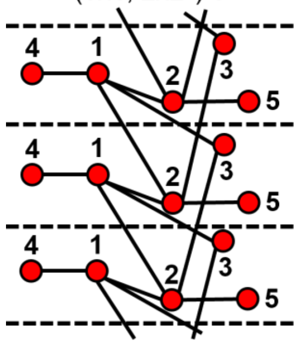	(8x**1**, 2x**2**^{1}) **d** 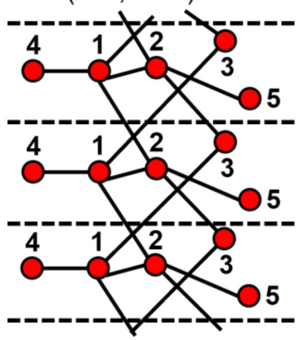	(8x**1**, 2x**2**^{2}) **a** 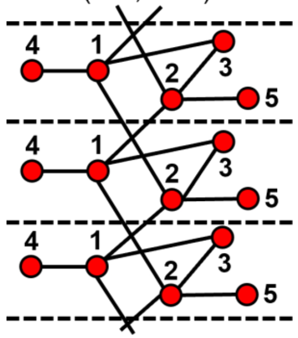	(8x**1**, 2x**2**^{2}) **b** 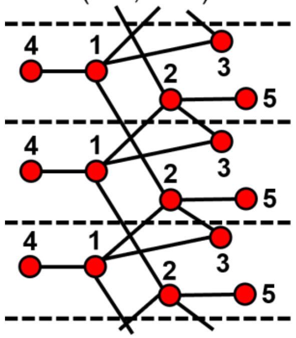
	(8x**1**, 2x**2**^{2}) **c** 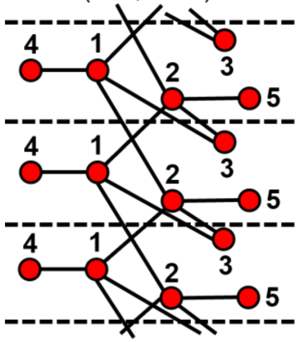	(6x**1**, 3x**2**) 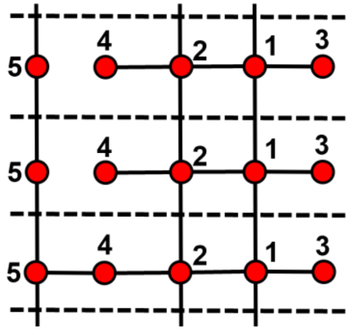	(6x**1**, 1x**2**, 2x**2**^{1}) **a** 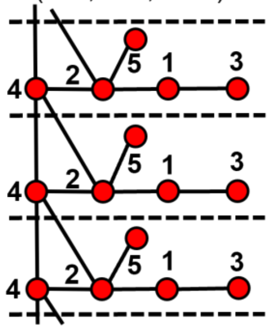	(6x**1**, 1x**2**, 2x**2**^{1}) **b** 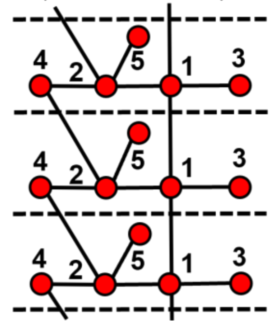

	(6x**1**, 1x**2**, 2x$\mathbf{2}^{2}$) **a**	(6x**1**, 1x**2**, 2x$\mathbf{2}^{2}$) **b**	(4x**1**, 2x**2**, 2x$\mathbf{2}^{1}$) **a**	(4x**1**, 2x**2**, 2x$\mathbf{2}^{1}$) **b**
	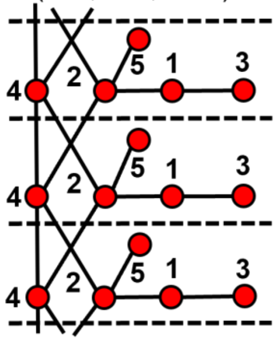	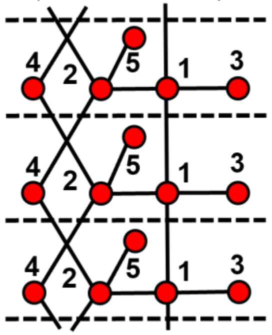	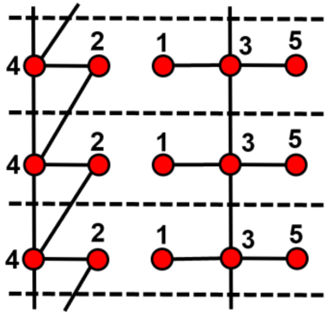	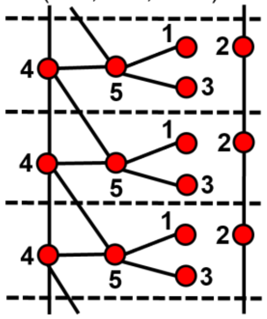
	(4x**1**, 2x**2**, 2x$\mathbf{2}^{1}$) **c**	(4x**1**, 2x**2**, 2x$\mathbf{2}^{2}$) **a**	(4x**1**, 2x**2**, 2x$\mathbf{2}^{2}$) **b**	(4x**1**, 2x**2**, 2x$\mathbf{2}^{2}$) **c**
	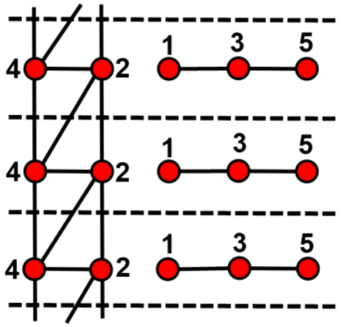	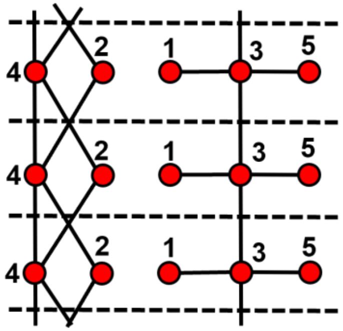	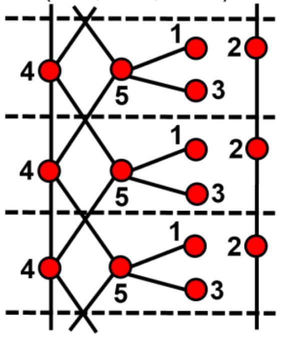	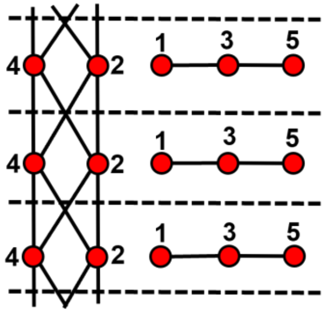
	(4x**1**, 4x$\mathbf{2}^{1}$)	(4x**1**, 2x$\mathbf{2}^{1}$, 2x$\mathbf{2}^{2}$) **a**	(4x**1**, 2x$\mathbf{2}^{1}$, 2x$\mathbf{2}^{2}$) **b**	(4x**1**, 4x$\mathbf{2}^{2}$)
	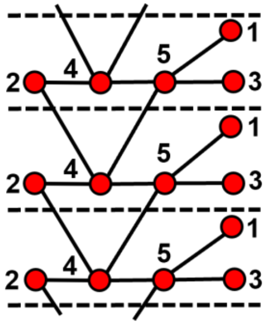	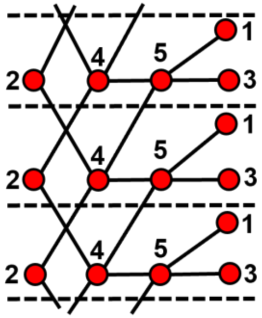	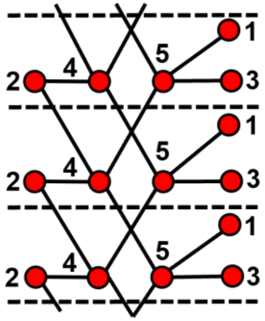	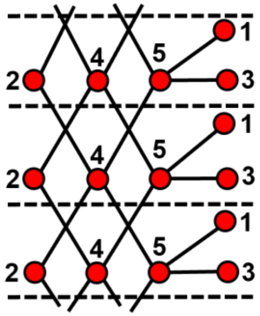

	(2x**1**, 3x**2**, 2x$\mathbf{2}^{1}$)	(2x**1**, 3x**2**, 2x$\mathbf{2}^{2}$)	(2x**1**, 1x**2**, 4x$\mathbf{2}^{1}$)	(2x**1**, 1x**2**, 2x$\mathbf{2}^{1}$, 2x$\mathbf{2}^{2}$) **a**
	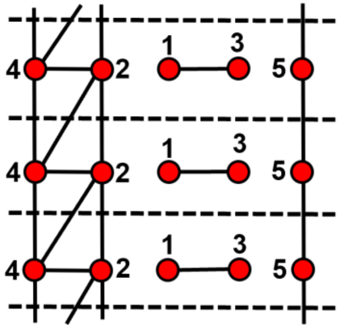	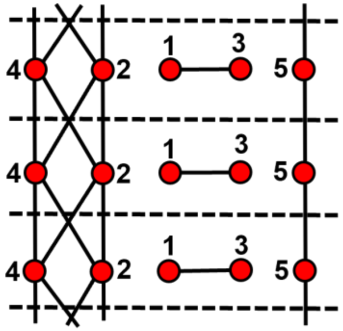	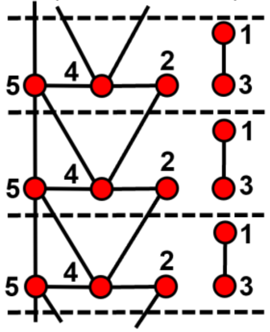	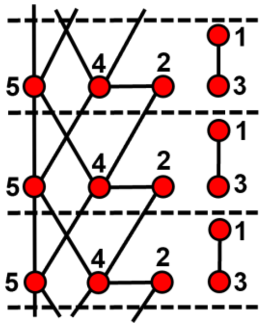
	(2x**1**, 1x**2**, 2x$\mathbf{2}^{1}$, 2x$\mathbf{2}^{2}$) **b**	(2x**1**, 1x**2**, 4x$\mathbf{2}^{2}$)		
	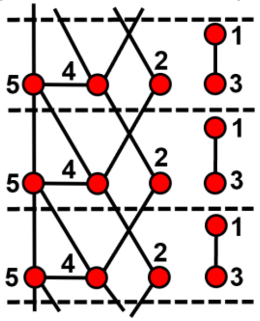	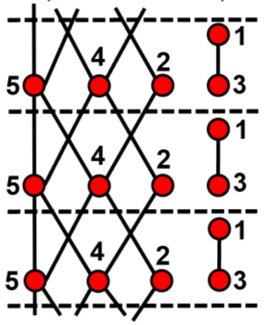		
$^{1}V_{2}{}^{2}V_{1}{}^{4}V_{3}$	NG			
$^{1}V_{2}{}^{2}V_{1}{}^{4}V_{4}$	NG			
$^{1}V_{2}{}^{2}V_{1}{}^{4}V_{5}$	NG			

${}^1V_2{}^2V_2{}^4V_1$	(10x**1**) **a**<br>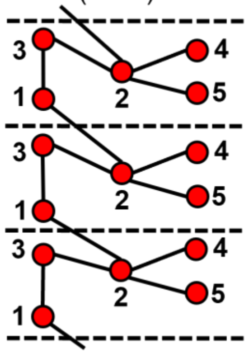	(10x**1**) **b**<br>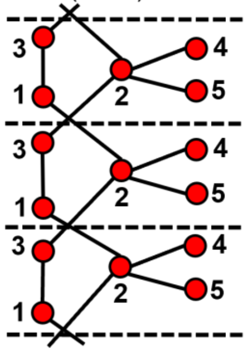	(10x**1**) **c**<br>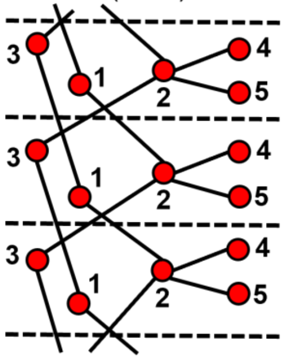	(8x**1**, 1x**2**) **a-1**<br>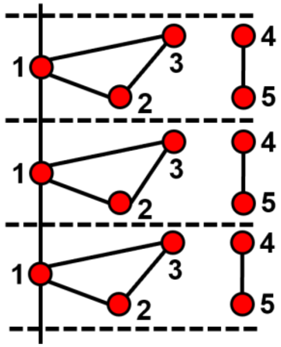
	(8x**1**, 1x**2**) **a-2**<br>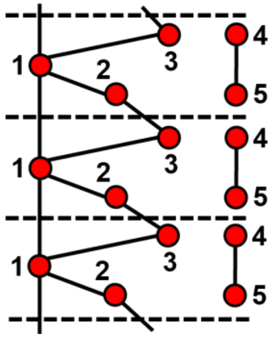	(8x**1**, 1x**2**) **a-3**<br>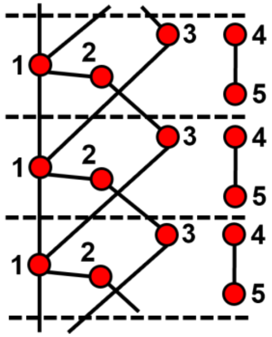	(8x**1**, 1x**2**) **a-4**<br>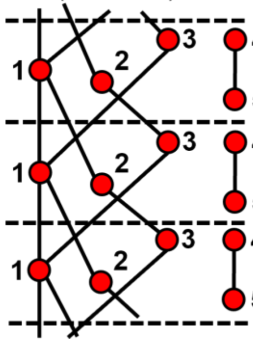	(8x**1**, 1x**2**) **b**<br>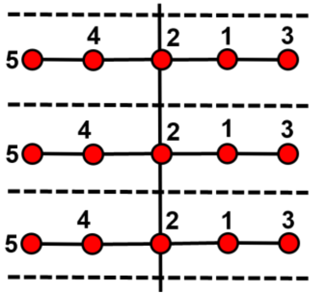
	(8x**1**, 1x**2**) **c**<br>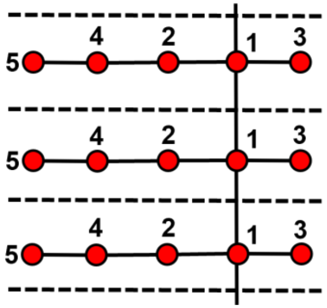	(8x**1**, 1x**2**) **d**<br>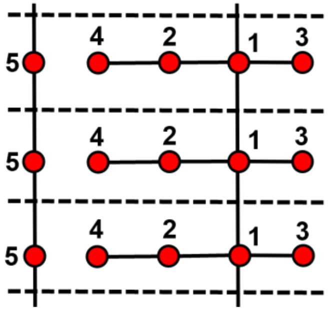	(6x**1**, 2x$\mathbf{2}^1$)<br>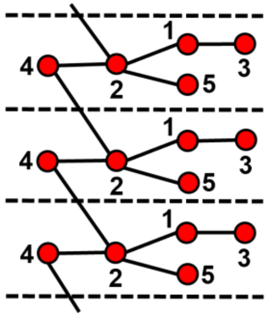	(6x**1**, 2x$\mathbf{2}^2$)<br>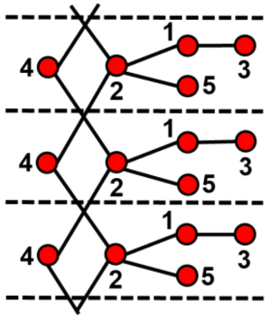

	(4x**1**, 3x**2**)<br>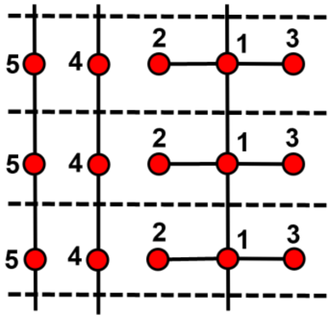	(4x**1**, 1x**2**, 2x**2**^{1}) **a**<br>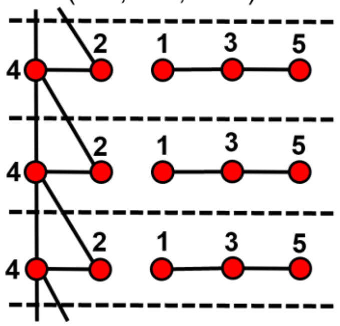	(4x**1**, 1x**2**, 2x**2**^{1}) **c**<br>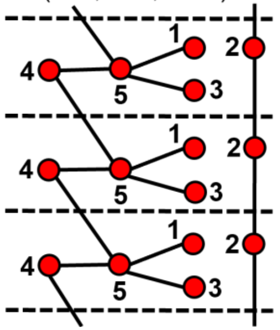	(4x**1**, 1x**2**, 2x**2**^{2}) **a**<br>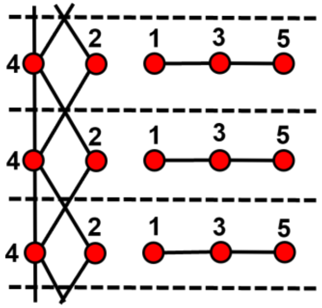
	(4x**1**, 1x**2**, 2x**2**^{2}) **b**<br>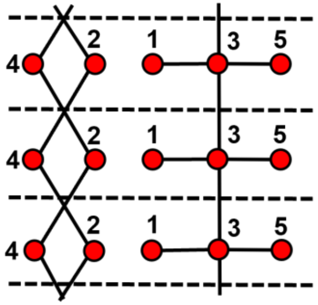	(4x**1**, 1x**2**, 2x**2**^{2}) **c**<br>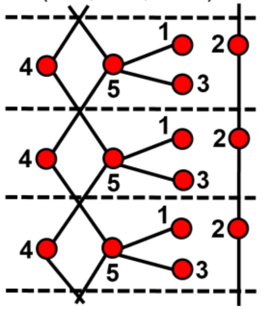	(2x**1**, 2x**2**, 2x**2**^{1})<br>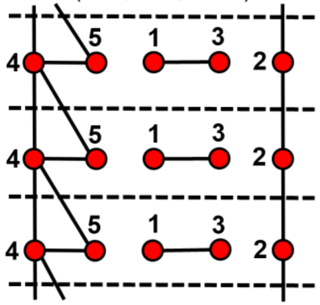	(2x**1**, 2x**2**, 2x**2**^{2})<br>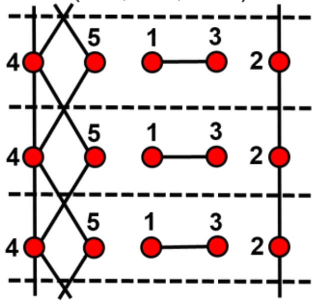
	(2x**1**, 4x**2**^{1})<br>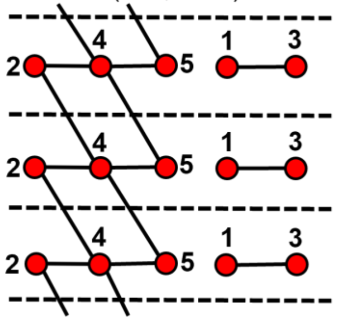	(2x**1**, 2x**2**^{1}, 2x**2**^{2})<br>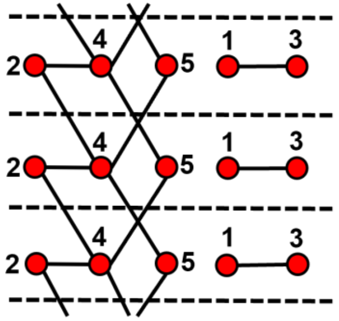	(2x**1**, 4x**2**^{2})<br>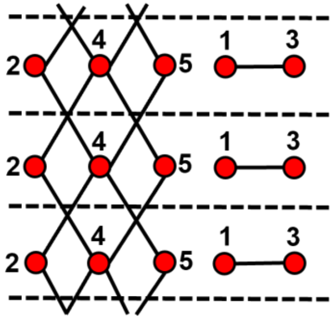	
${}^{1}V_2{}^{2}V_2{}^{4}V_2$	NG			

$^1V_2{}^2V_2{}^4V_3$	NG			
$^1V_2{}^2V_2{}^4V_4$	NG			
$^1V_2{}^2V_3{}^4V_1$	NG			
$^1V_2{}^2V_3{}^4V_2$	NG			
$^1V_2{}^2V_3{}^4V_3$	NG			
$^1V_2{}^2V_4{}^4V_1$	NG			
$^1V_2{}^2V_4{}^4V_2$	NG			
$^1V_2{}^2V_5{}^4V_1$	NG			
$^1V_4{}^2V_1{}^4V_1$	(8x**1**, 1x**2**) **a** 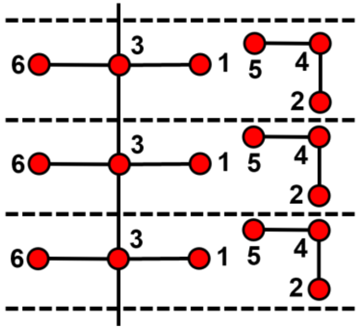	(8x**1**, 1x**2**) **b** 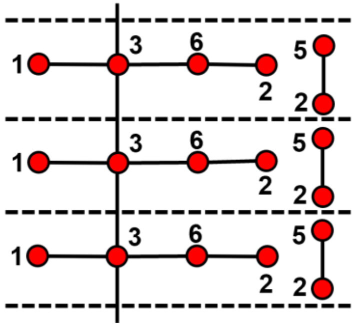	(8x**1**, 1x**2**) **c** 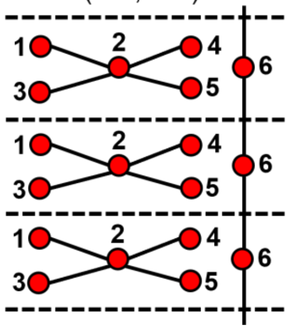	(6x**1**, 2x**2**) 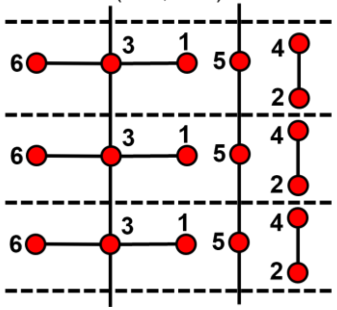
	(6x**1**, 2x$\mathbf{2}^1$) 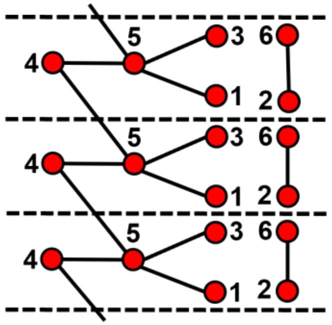	(6x**1**, 2x$\mathbf{2}^2$) 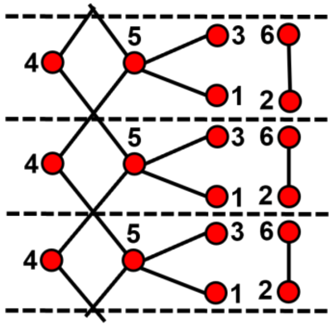	(4x**1**, 1x**2**, 2x$\mathbf{2}^1$) 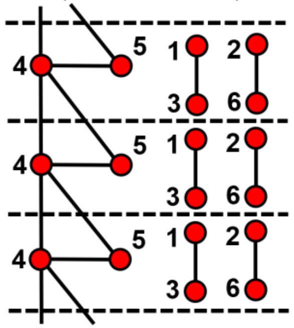	(4x**1**, 1x**2**, 2x$\mathbf{2}^2$) 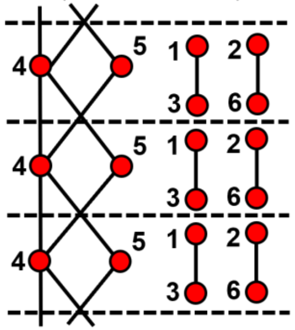

$^{1}V_{4}{}^{2}V_{1}{}^{4}V_{2}$	NG			
$^{1}V_{4}{}^{2}V_{1}{}^{4}V_{3}$	NG			
$^{1}V_{4}{}^{2}V_{2}{}^{4}V_{1}$	NG			
$^{1}V_{4}{}^{2}V_{2}{}^{4}V_{2}$	NG			
$^{1}V_{4}{}^{2}V_{3}{}^{4}V_{1}$	NG			
$^{1}V_{6}{}^{2}V_{1}{}^{4}V_{1}$	NG			
$\mathbf{^{1}V_{r}{}^{3}V_{r}{}^{4}V_{r}}$				
$^{1}V_{1}{}^{3}V_{1}{}^{4}V_{1}$	(4x**1**, 2x**2**) 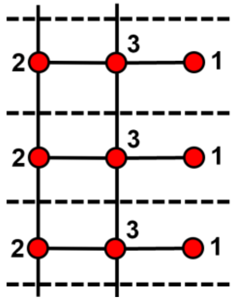	(2x**1**, 1x**2**, 2x**2**^{1}) 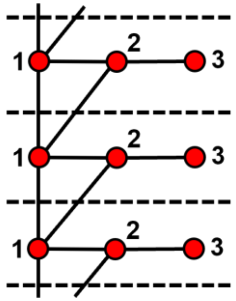	(2x**1**, 1x**2**, 2x**2**^{2}) 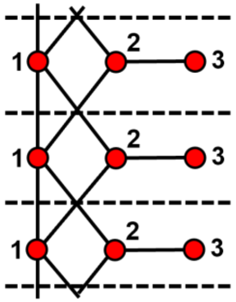	
$^{1}V_{1}{}^{3}V_{1}{}^{4}V_{2}$	(8x**1**, 2x**2**) **a** 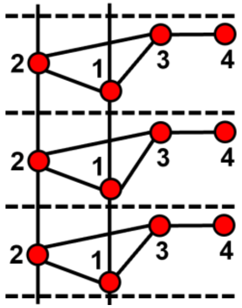	(8x**1**, 2x**2**) **b** 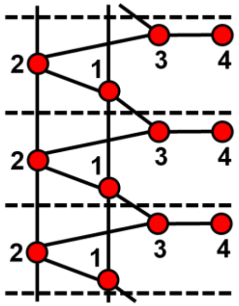	(8x**1**, 2x**2**) **c** 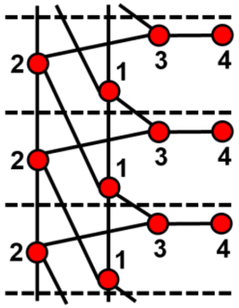	(8x**1**, 2x**2**) **d** 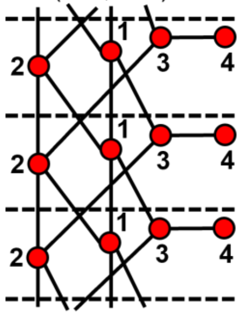

	(6x**1**, 3x**2**) 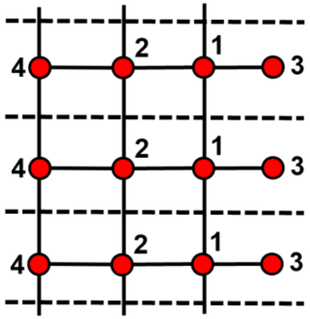	(6x**1**, 1x2, 2x$\mathbf{2}^1$) **a** 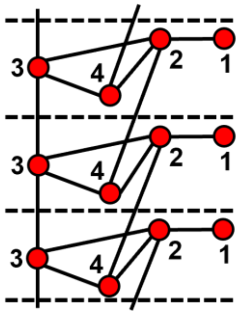	(6x**1**, 1x2, 2x$\mathbf{2}^1$) **b** 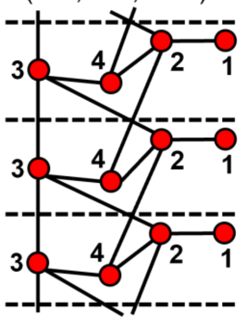	(6x**1**, 1x2, 2x$\mathbf{2}^1$) **c** 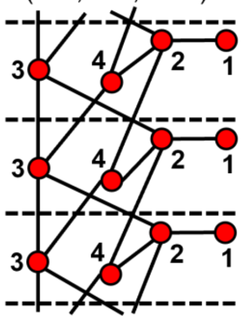
	(6x**1**, 1x2, 2x$\mathbf{2}^2$) **a** 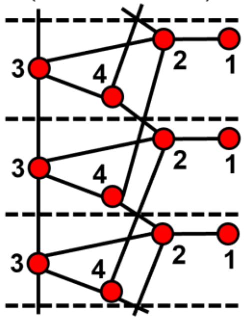	(6x**1**, 1x2, 2x$\mathbf{2}^2$) **b** 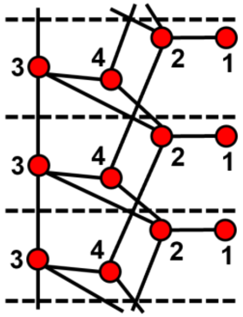	(6x**1**, 1x2, 2x$\mathbf{2}^2$) **c** 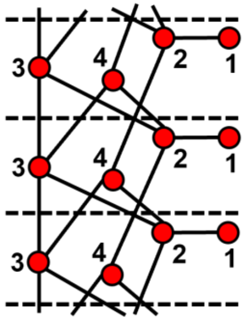	(4x**1**, 2x**2**, 2x$\mathbf{2}^1$) **a** 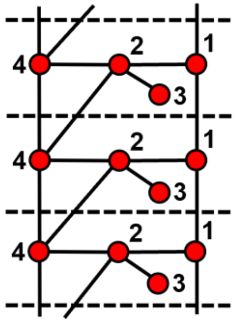
	(4x**1**, 2x**2**, 2x$\mathbf{2}^1$) **b** 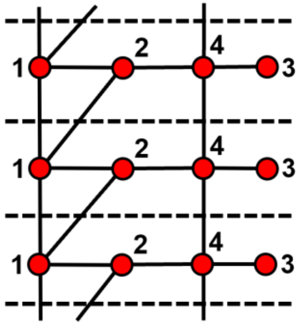	(4x**1**, 2x**2**, 2x$\mathbf{2}^2$) **a** 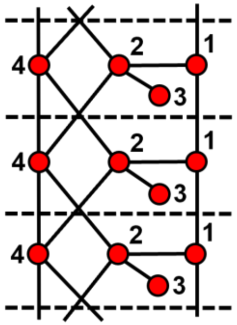	(4x**1**, 2x**2**, 2x$\mathbf{2}^2$) **b** 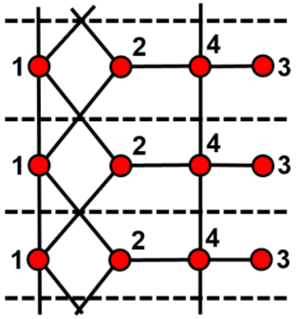	(4x**1**, 4x$\mathbf{2}^1$) **a** 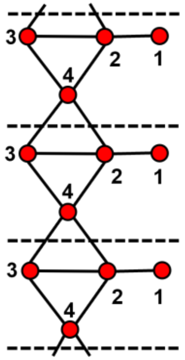

	(4x**1**, 4x**2**^{1}) **b** 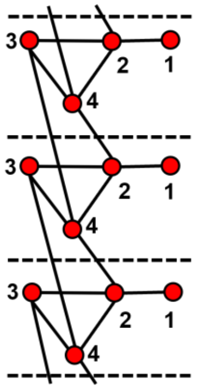	(4x**1**, 4x**2**^{1}) **c** 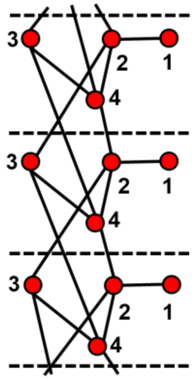	(4x**1**, 2x**2**^{1}, 2x**2**^{2}) **a** 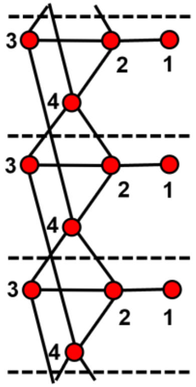	(4x**1**, 2x**2**^{1}, 2x**2**^{2}) **b** 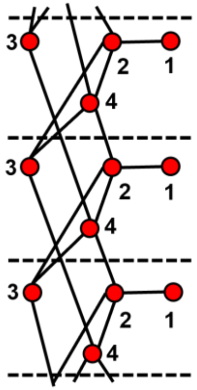
	(4x**1**, 4x**2**^{2}) **a** 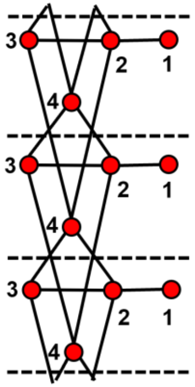	(4x**1**, 4x**2**^{2}) **b** 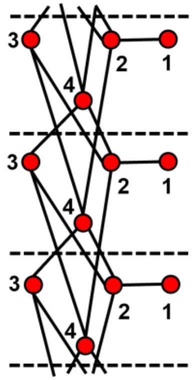	(2x**1**, 3x**2**, 2x**2**^{1}) 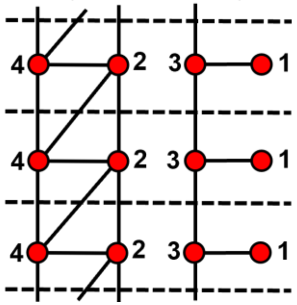	(2x**1**, 3x**2**, 2x**2**^{2}) 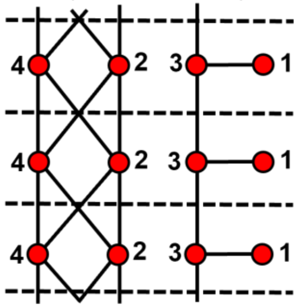

	(2x**1**, 1x**2**, 4x**2**^{1}) 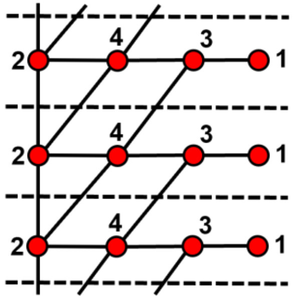	(2x**1**, 1x**2**, 2x**2**^{1}, 2x**2**^{2}) 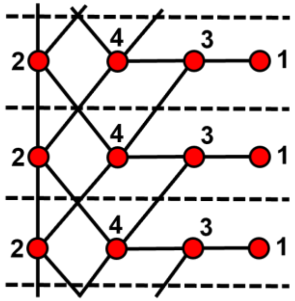	(2x**1**, 1x**2**, 4x**2**^{2}) 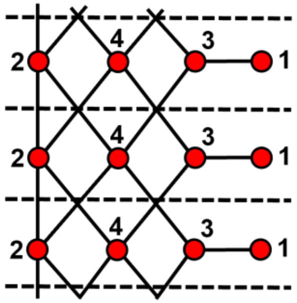	
$^{1}V_{1}{}^{3}V_{1}{}^{4}V_{3}$	NG			
$^{1}V_{1}{}^{3}V_{1}{}^{4}V_{4}$	NG			
$^{1}V_{1}{}^{3}V_{1}{}^{4}V_{5}$	NG			
$^{1}V_{1}{}^{3}V_{1}{}^{4}V_{6}$	NG			
$^{1}V_{1}{}^{3}V_{3}{}^{4}V_{1}$	(14x**1**) **a** 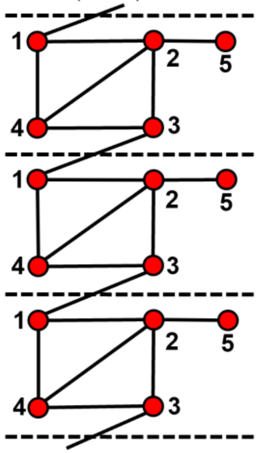	(14x**1**) **b** 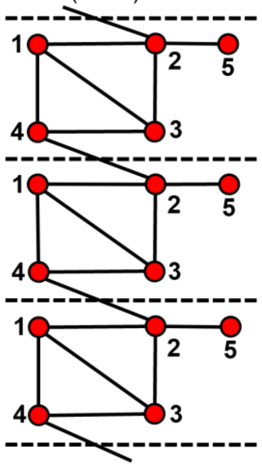	(14x**1**) **c** 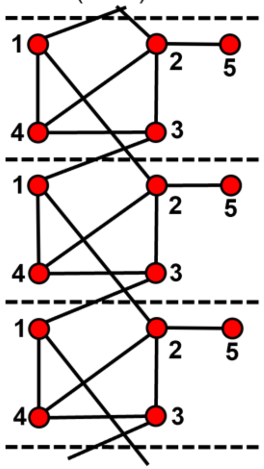	(14x**1**) **d** 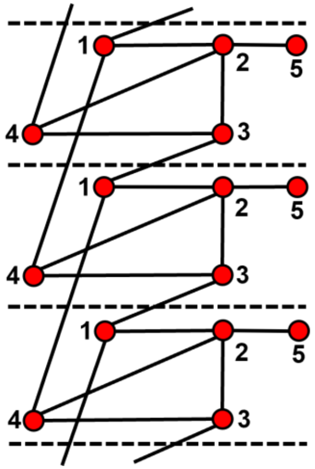

	(14x**1**) **e**<br>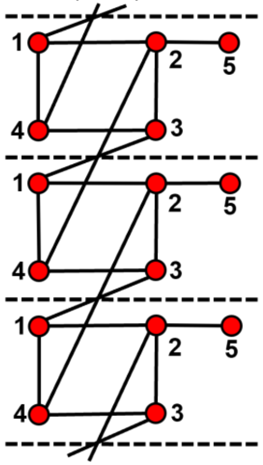	(14x**1**) **f**<br>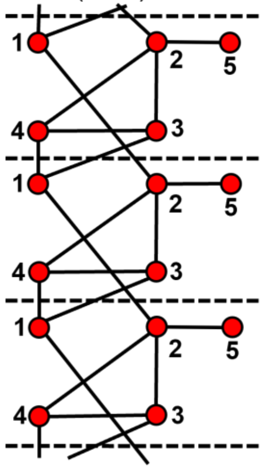	(14x**1**) **g**<br>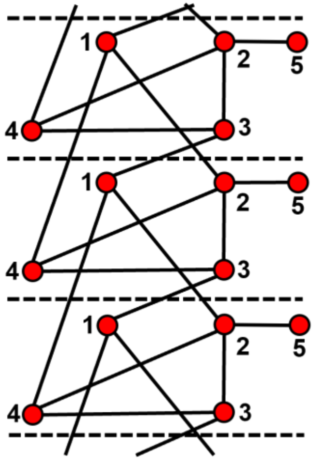	(14x**1**) **h**<br>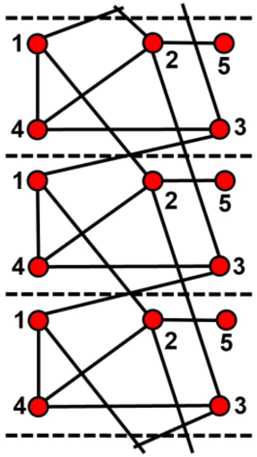
	(14x**1**) **i**<br>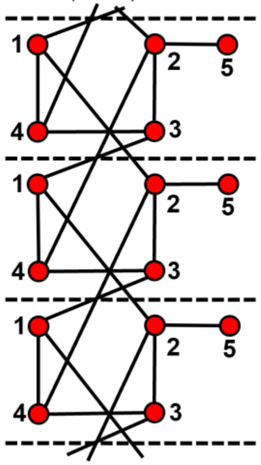	(14x**1**) **j**<br>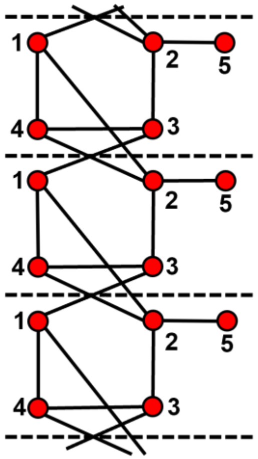	(14x**1**) **k**<br>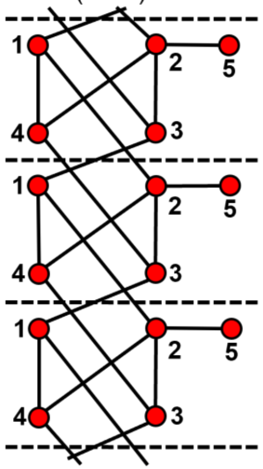	(14x**1**) **l**<br>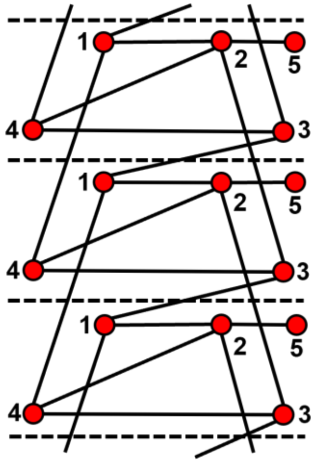

	(14x**1**) **m** 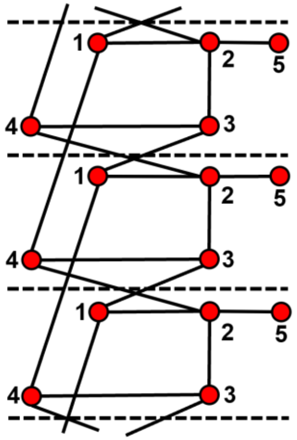	(14x**1**) **n** 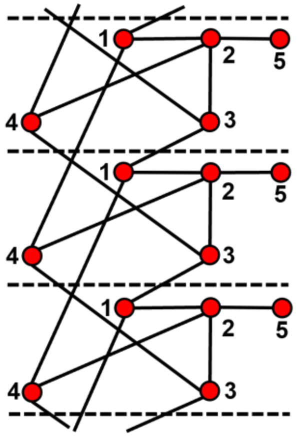	(14x**1**) **o** 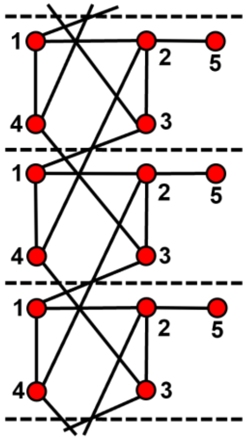	(14x**1**) **p** 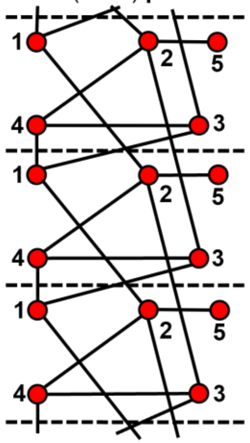
	(14x**1**) **q** 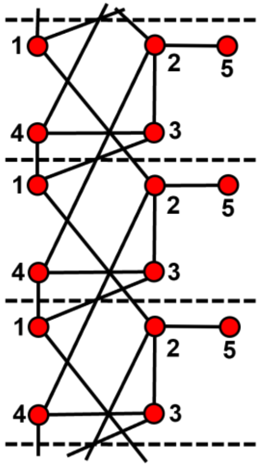	(14x**1**) **r** 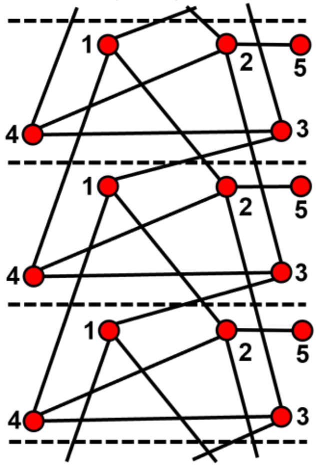	(14x**1**) **s** 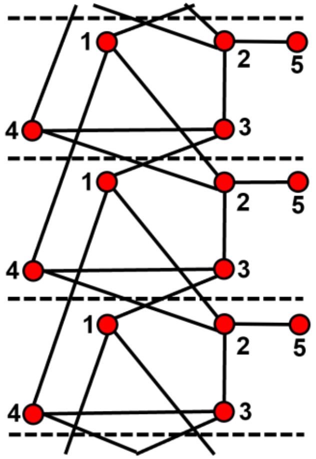	(14x**1**) **t** 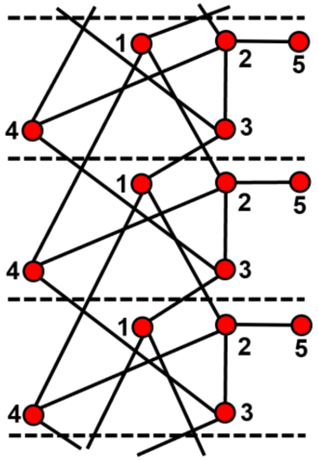

	(14x**1**) **u** 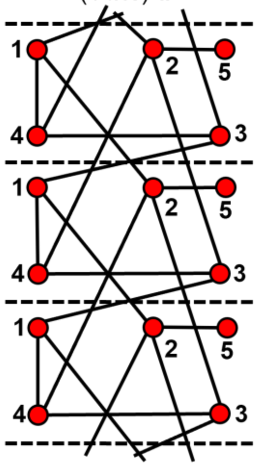	(14x**1**) **v** 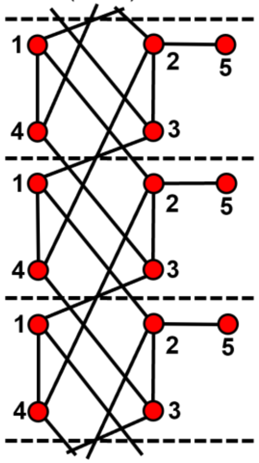	(14x**1**) **w** 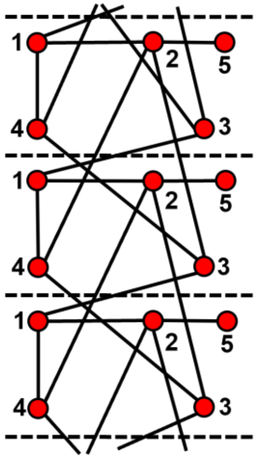	(14x**1**) **x** 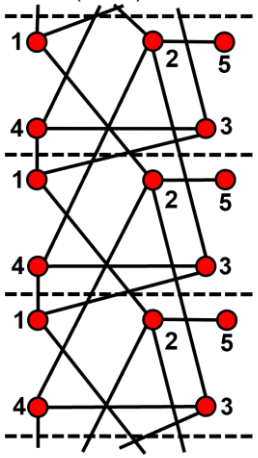
	(14x**1**) **y** 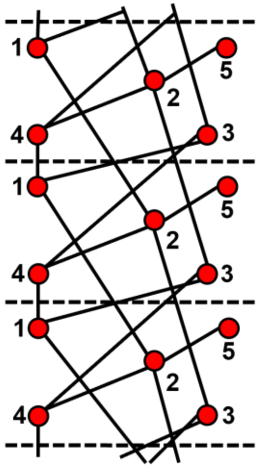	(14x**1**) **z** 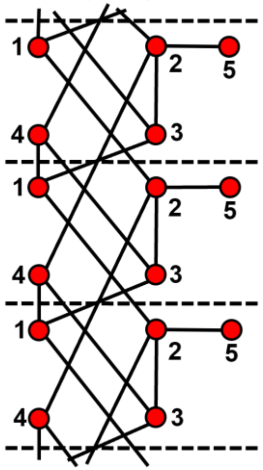	(14x**1**) **a’** 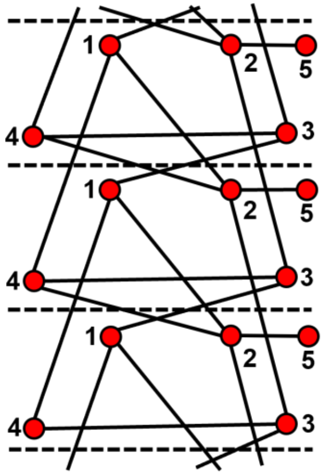	(14x**1**) **b’** 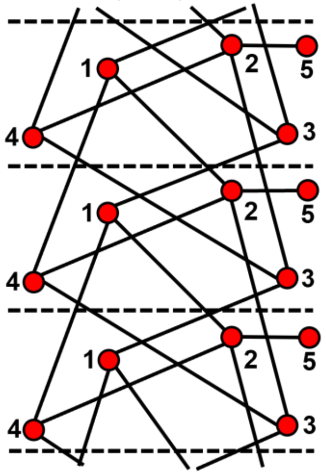

	(14x**1**) **c’** 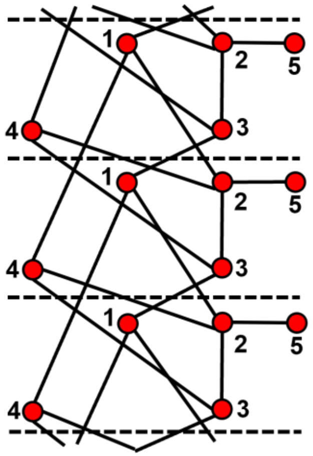	(14x**1**) **d’** 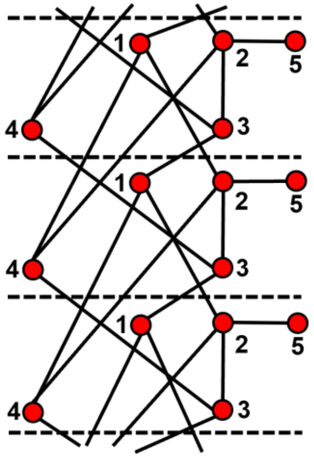	(14x**1**) **e’** 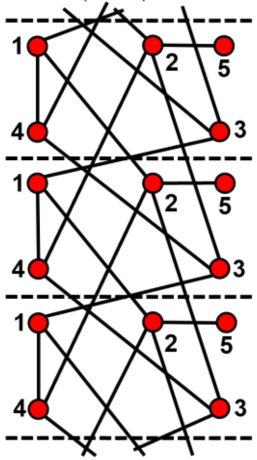	(14x**1**) **f’** 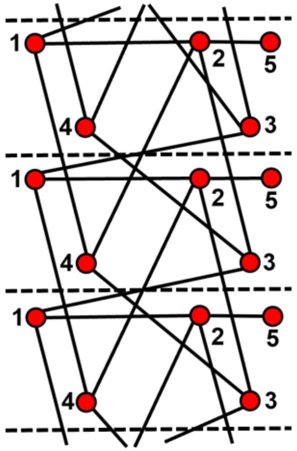
	(14x**1**) **g’** 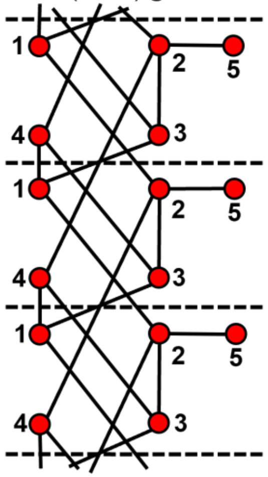	(14x**1**) **h’** 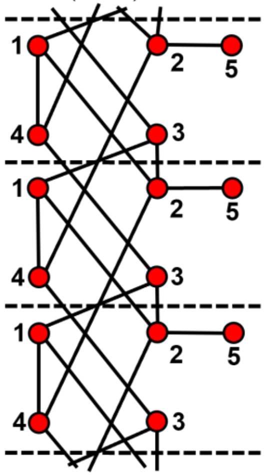	(14x**1**) **i’** 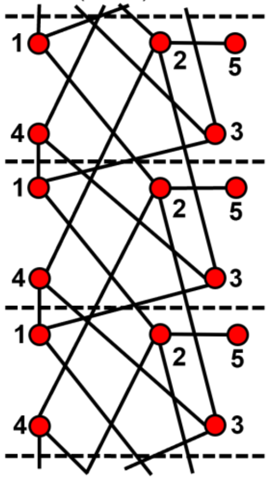	(14x**1**) **j’** 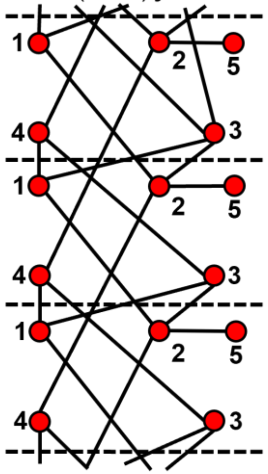

(12x**1**, 1x**2**) **a**

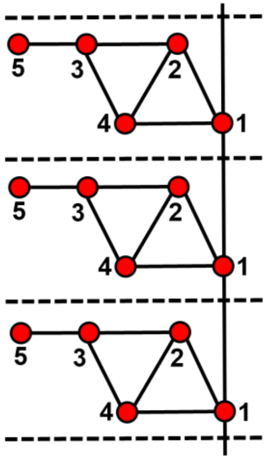

(12x**1**, 1x**2**) **b**

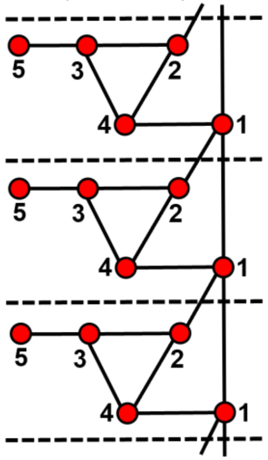

(12x**1**, 1x**2**) **c**

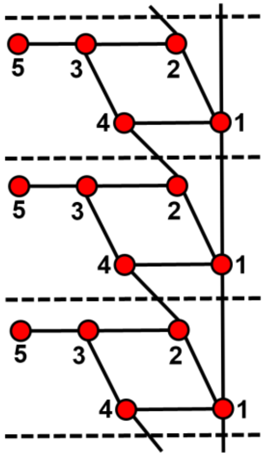

(12x**1**, 1x**2**) **d**

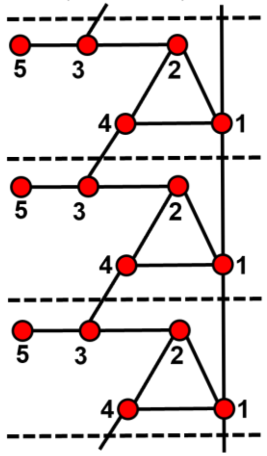

(12x**1**, 1x**2**) **e**

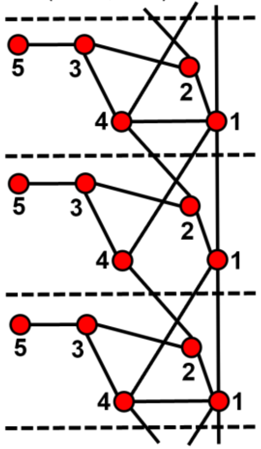

(12x**1**, 1x**2**) **f**

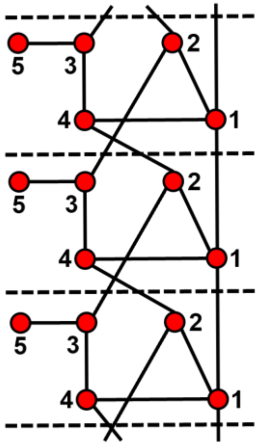

(12x**1**, 1x**2**) **g**

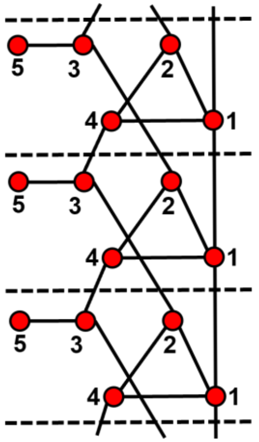

(12x**1**, 1x**2**) **h**

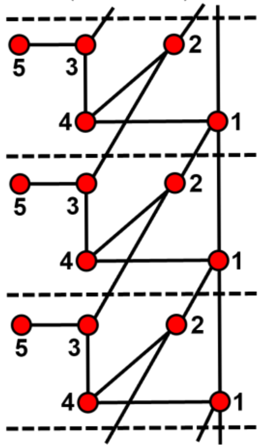

	(12x**1**, 1x**2**) **i** 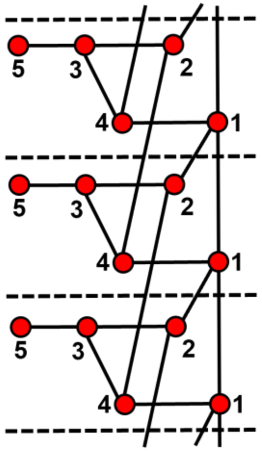	(12x**1**, 1x**2**) **j** 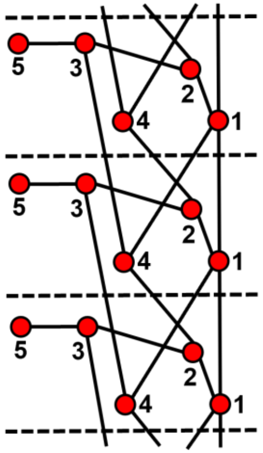	(12x**1**, 1x**2**) **k** 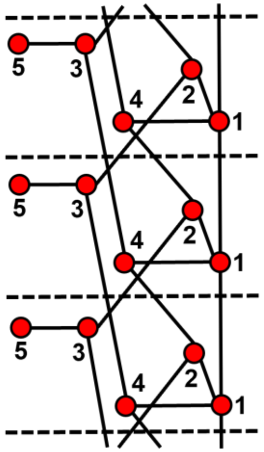	(12x**1**, 1x**2**) **l** 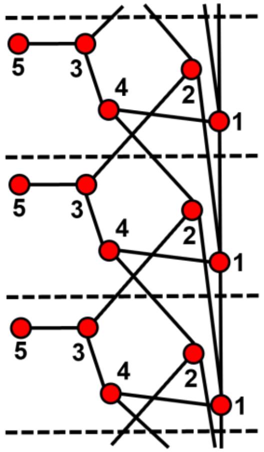
	(12x**1**, 1x**2**) **m** 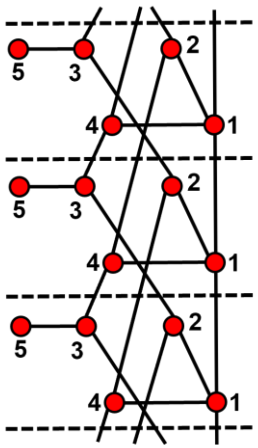	(12x**1**, 1x**2**) **n** 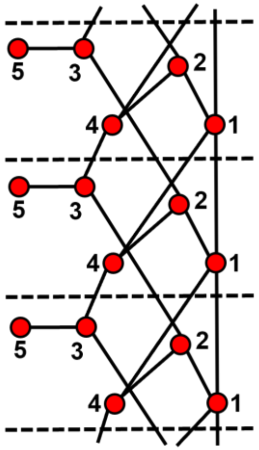	(12x**1**, 1x**2**) **o** 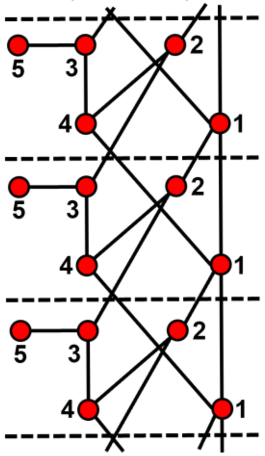	(12x**1**, 1x**2**) **p** 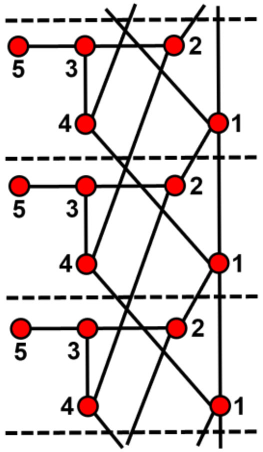

	(12x**1**, 1x**2**) **q** 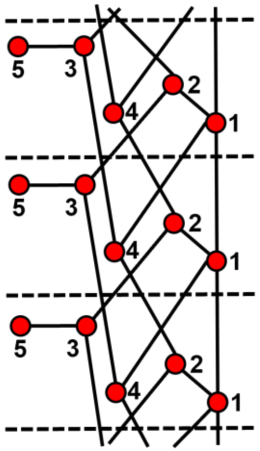	(12x**1**, 1x**2**) **r** 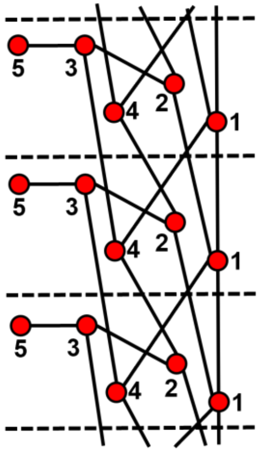	(12x**1**, 1x**2**) **s** 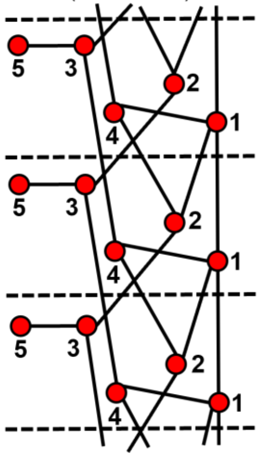	(12x**1**, 1x**2**) **t** 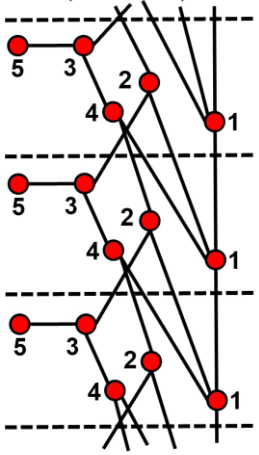
	(12x**1**, 1x**2**) **u** 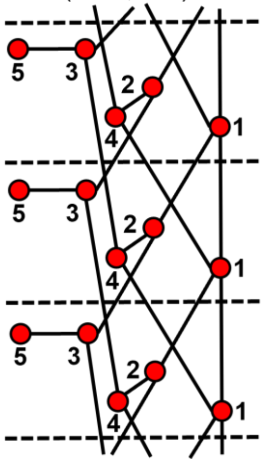	(12x**1**, 1x**2**) **v** 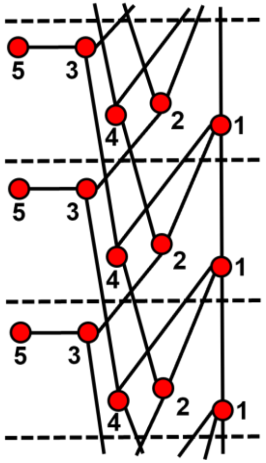	(12x**1**, 1x**2**) **w** 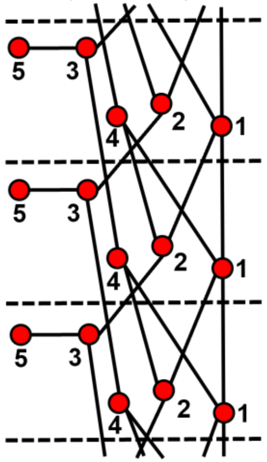	(12x**1**, 1x**2**) **x** 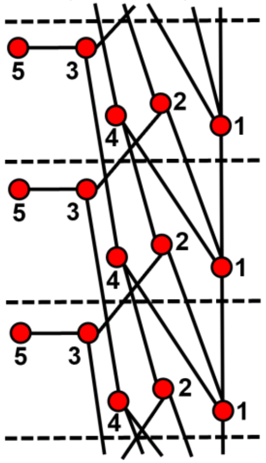

	(10x**1**, 2x**2**) **a** 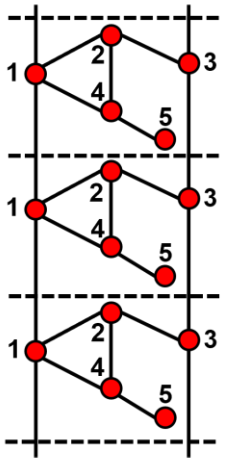	(10x**1**, 2x**2**) **b** 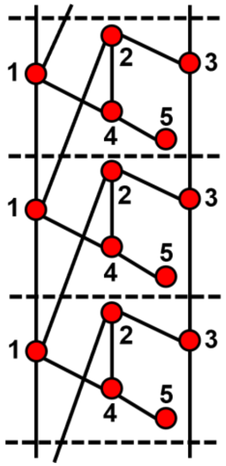	(10x**1**, 2x**2**) **c** 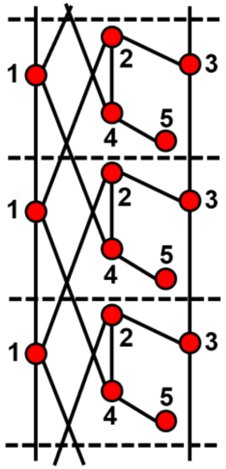	(10x**1**, 2x**2**) **d** 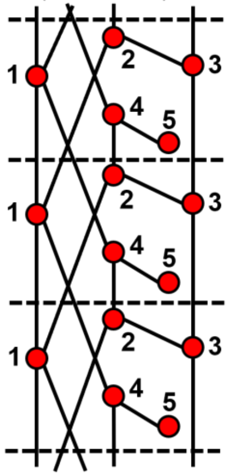
	(10x**1**, 2x**2**^{1}) **a** 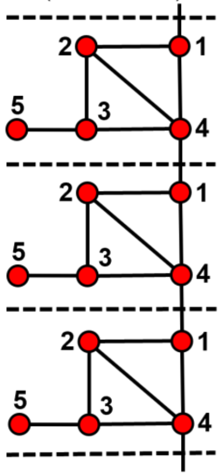	(10x**1**, 2x**2**^{1}) **b** 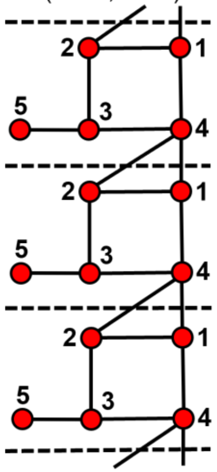	(10x**1**, 2x**2**^{1}) **c** 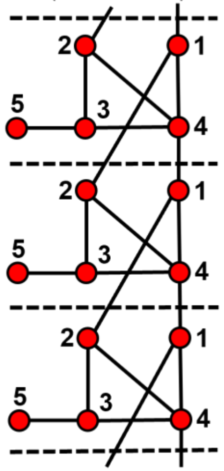	(10x**1**, 2x**2**^{1}) **d** 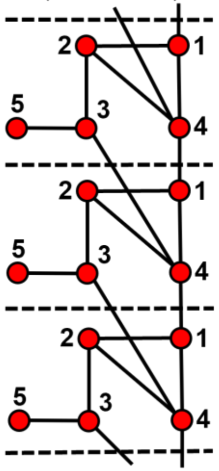

	(10x**1**, 2x$\mathbf{2}^1$) **e** 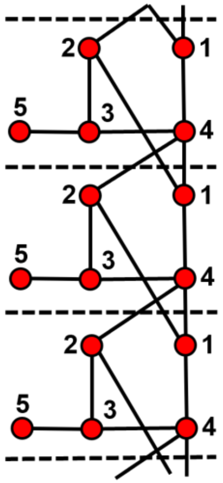	(10x**1**, 2x$\mathbf{2}^1$) **f** 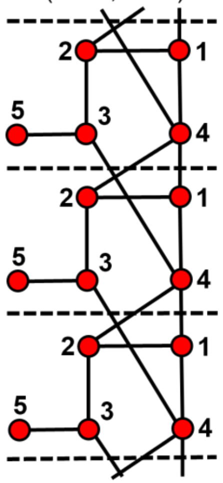	(10x**1**, 2x$\mathbf{2}^1$) **g** 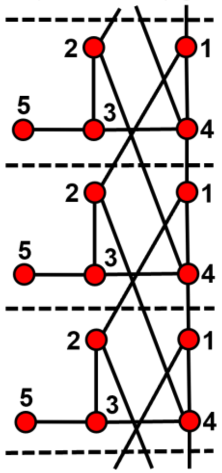	(10x**1**, 2x$\mathbf{2}^1$) **h** 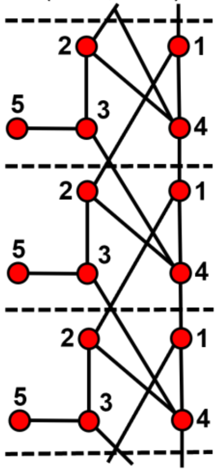
	(10x**1**, 2x$\mathbf{2}^1$) **i** 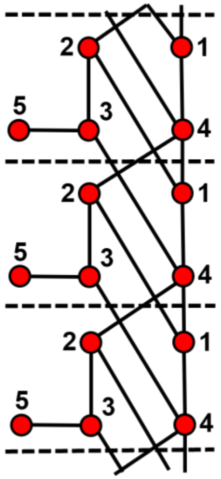	(10x**1**, 2x$\mathbf{2}^1$) **j** 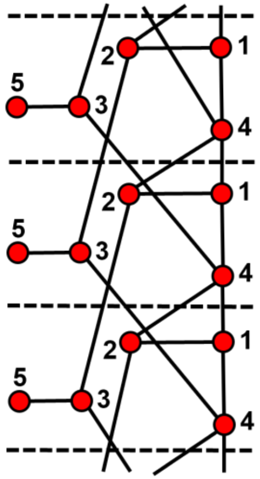	(10x**1**, 2x$\mathbf{2}^1$) **k** 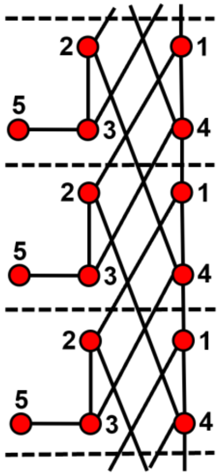	(10x**1**, 2x$\mathbf{2}^1$) **l** 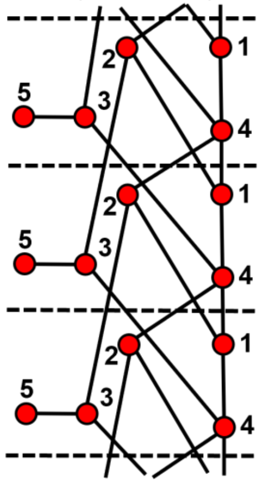

	(10x**1**, 2x**2**^{2}) **a**<br>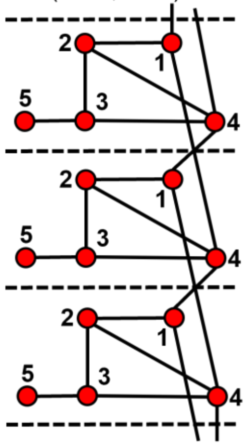	(10x**1**, 2x**2**^{2}) **b**<br>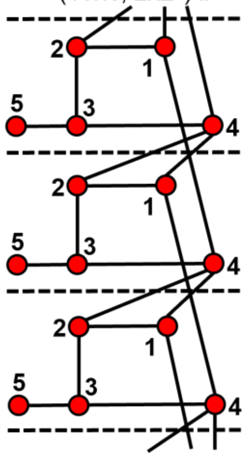	(10x**1**, 2x**2**^{2}) **c**<br>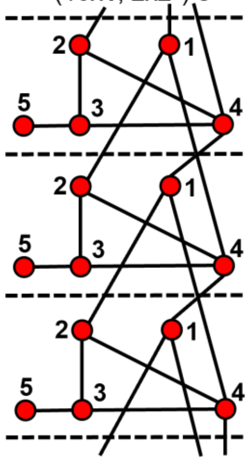	(10x**1**, 2x**2**^{2}) **d**<br>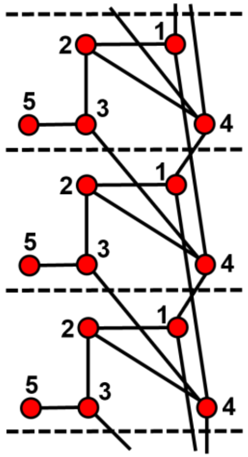
	(10x**1**, 2x**2**^{2}) **e**<br>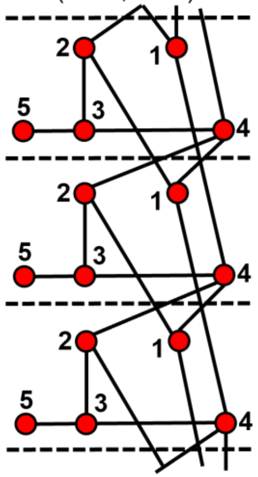	(10x**1**, 2x**2**^{2}) **f**<br>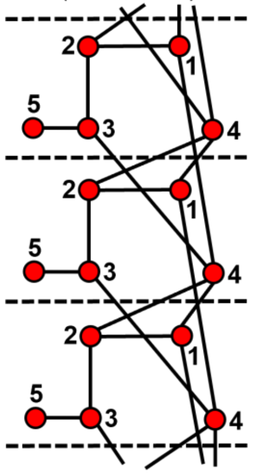	(10x**1**, 2x**2**^{2}) **g**<br>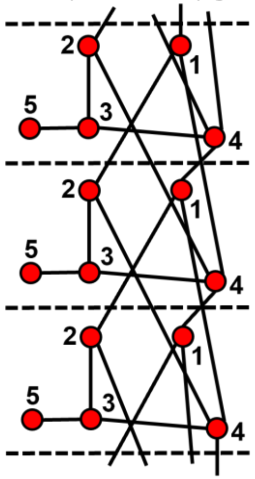	(10x**1**, 2x**2**^{2}) **h**<br>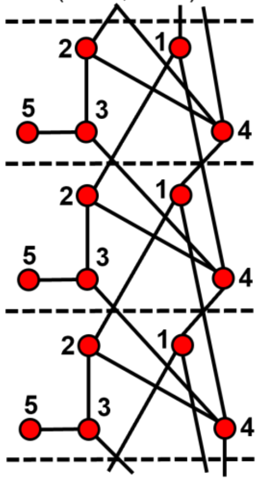

	(10x**1**, 2x**2**^{2}) **i** 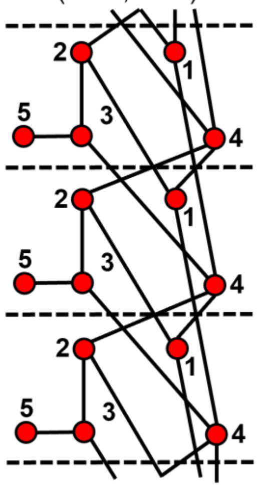	(10x**1**, 2x**2**^{2}) **j** 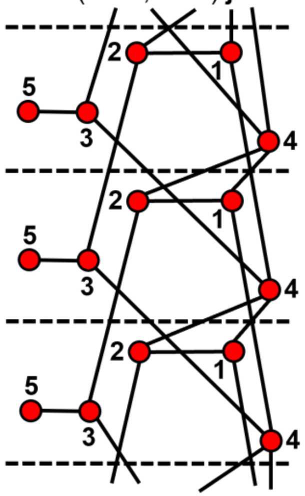	(10x**1**, 2x**2**^{2}) **k** 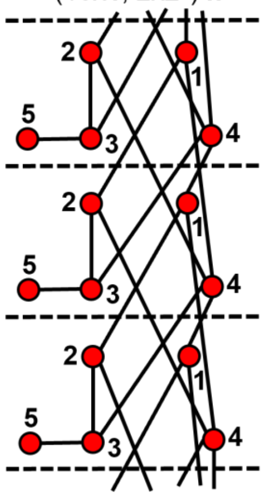	(10x**1**, 2x**2**^{2}) **l** 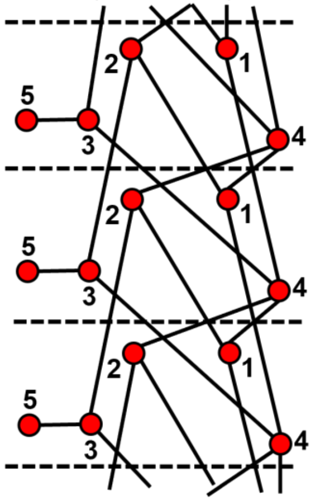
	(8x**1**, 3x**2**) **a** 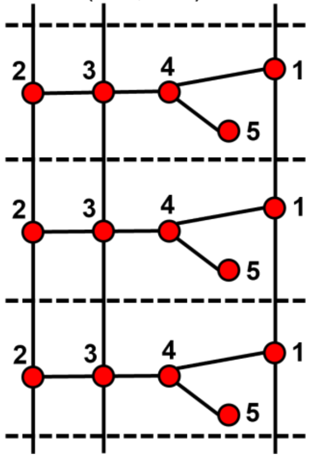	(8x**1**, 3x**2**) **b** 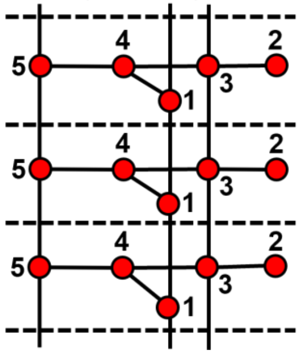	(8x**1**, 3x**2**) **c** 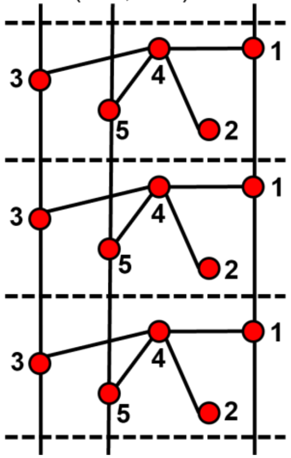	(8x**1**, 1x**2**, 2x**2**^{1}) **a-1** 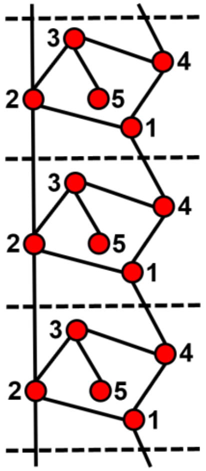

	(8x**1**, 1x**2**, 2x$\mathbf{2}^1$) **a-2** 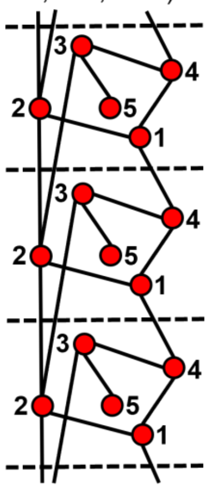	(8x**1**, 1x**2**, 2x$\mathbf{2}^1$) **a-3** 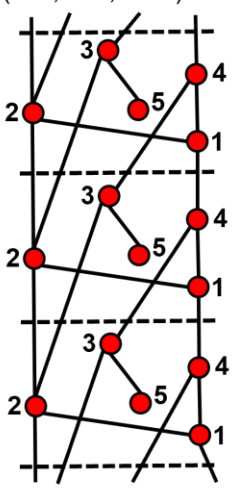	(8x**1**, 1x**2**, 2x$\mathbf{2}^1$) **a-4** 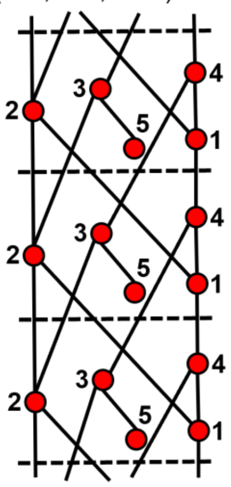	(8x**1**, 1x**2**, 2x$\mathbf{2}^1$) **b-1** 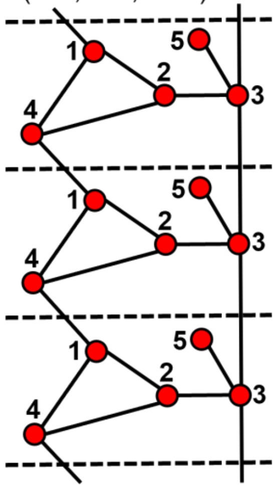
	(8x**1**, 1x**2**, 2x$\mathbf{2}^1$) **b-2** 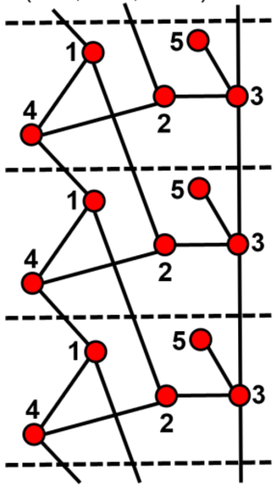	(8x**1**, 1x**2**, 2x$\mathbf{2}^1$) **b-3** 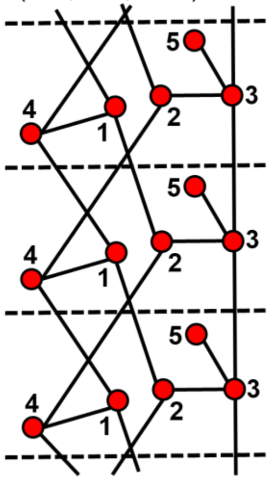	(8x**1**, 1x**2**, 2x$\mathbf{2}^1$) **c-1** 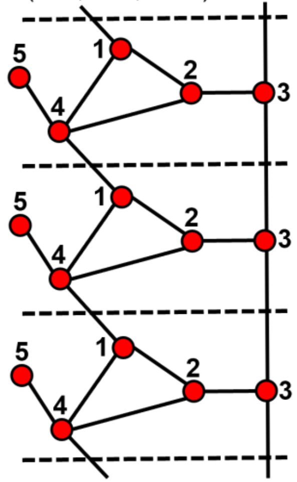	(8x**1**, 1x**2**, 2x$\mathbf{2}^1$) **c-2** 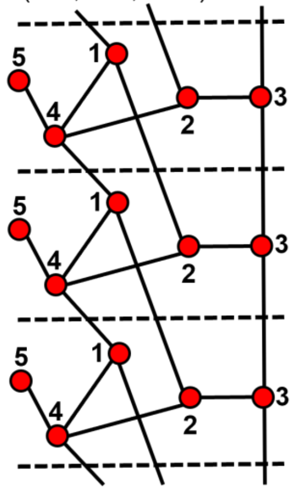

	(8x**1**, 1x**2**, 2x**2**^{1}) **c-3** 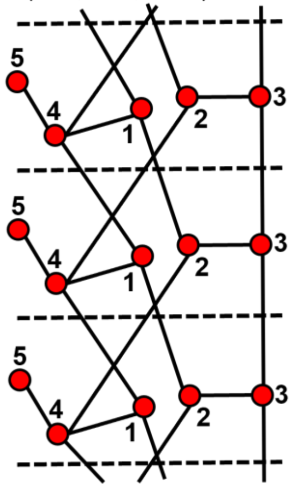	(8x**1**, 1x**2**, 2x**2**^{1}) **d-1** 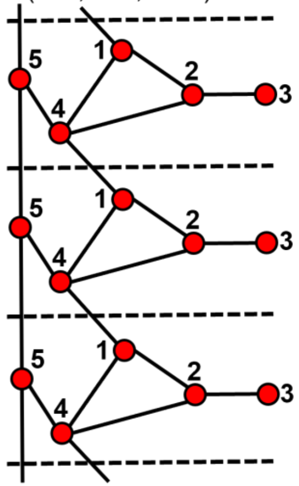	(8x**1**, 1x**2**, 2x**2**^{1}) **d-2** 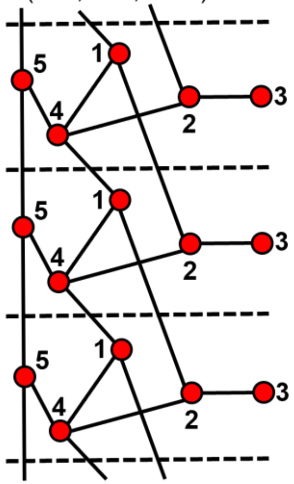	(8x**1**, 1x**2**, 2x**2**^{1}) **d-3** 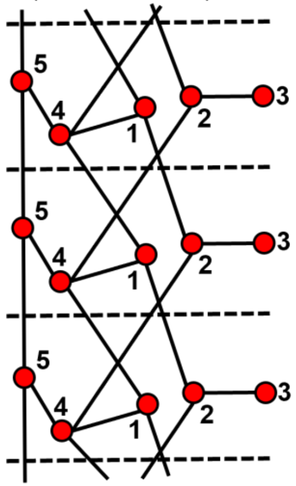
	(8x**1**, 1x**2**, 2x**2**^{1}) **e-1** 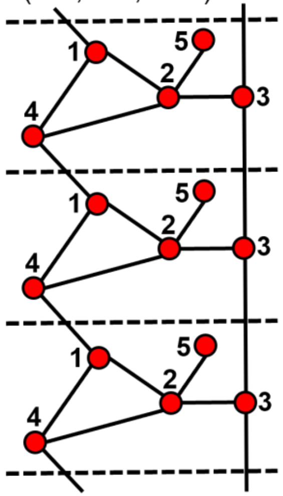	(8x**1**, 1x**2**, 2x**2**^{1}) **e-2** 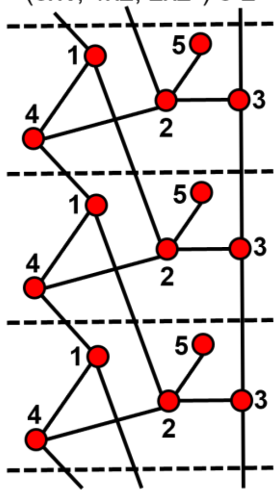	(8x**1**, 1x**2**, 2x**2**^{1}) **e-3** 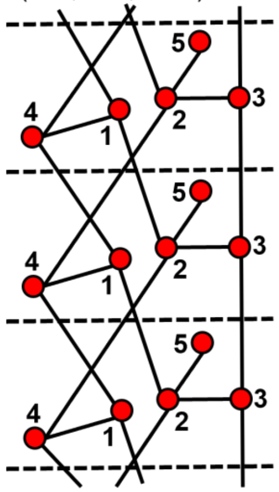	(8x**1**, 1x**2**, 2x**2**^{2}) **a-1** 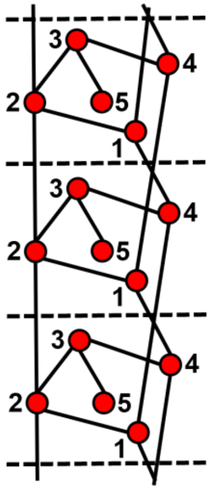

	(8x**1**, 1x**2**, 2x**2**^{2}) **a-2** 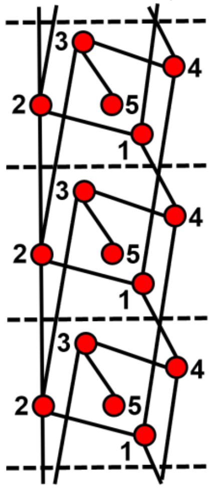	(8x**1**, 1x**2**, 2x**2**^{2}) **a-3** 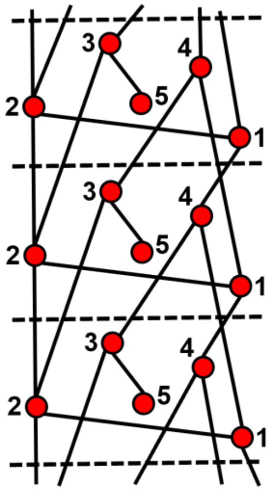	(8x**1**, 1x**2**, 2x**2**^{2}) **a-4** 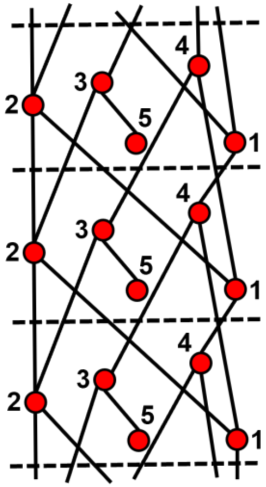	(8x**1**, 1x**2**, 2x**2**^{2}) **b-1** 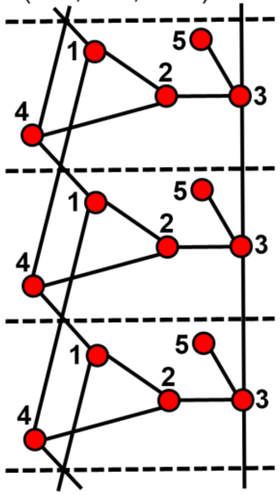
	(8x**1**, 1x**2**, 2x**2**^{2}) **b-2** 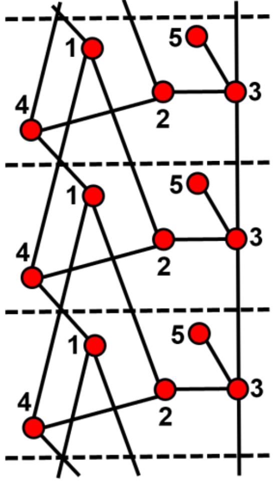	(8x**1**, 1x**2**, 2x**2**^{2}) **b-3** 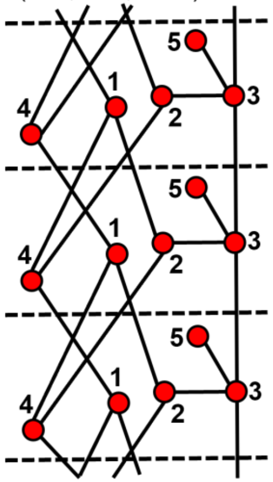	(8x**1**, 1x**2**, 2x**2**^{2}) **c-1** 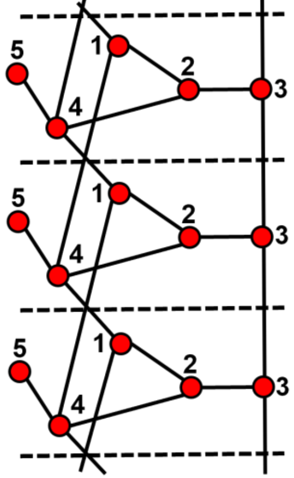	(8x**1**, 1x**2**, 2x**2**^{2}) **c-2** 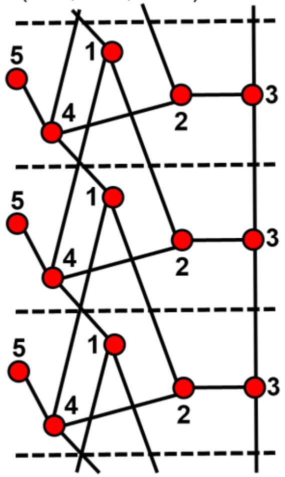

	(8x**1**, 1x**2**, 2x**2**^{2}) **c-3** 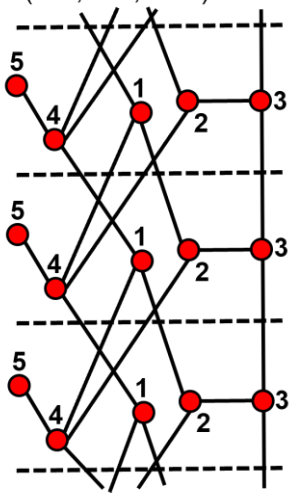	(8x**1**, 1x**2**, 2x**2**^{2}) **d-1** 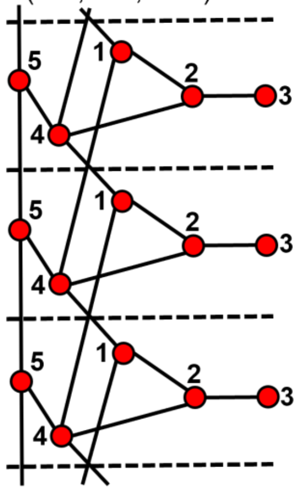	(8x**1**, 1x**2**, 2x**2**^{2}) **d-2** 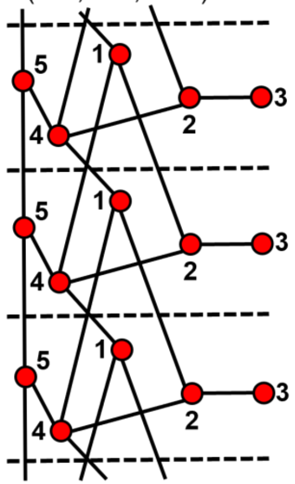	(8x**1**, 1x**2**, 2x**2**^{2}) **d-3** 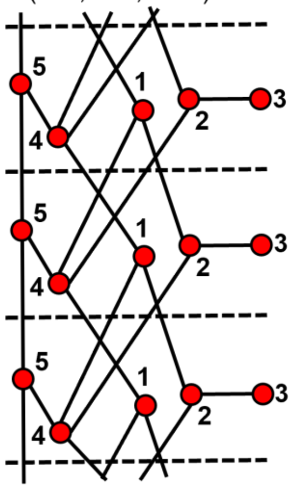
	(8x**1**, 1x**2**, 2x**2**^{2}) **e-1** 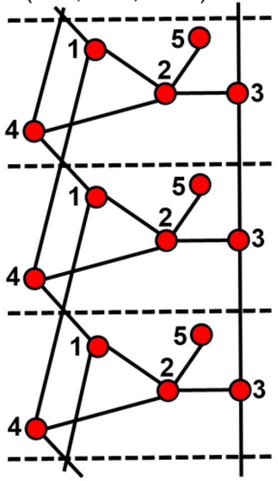	(8x**1**, 1x**2**, 2x**2**^{2}) **e-2** 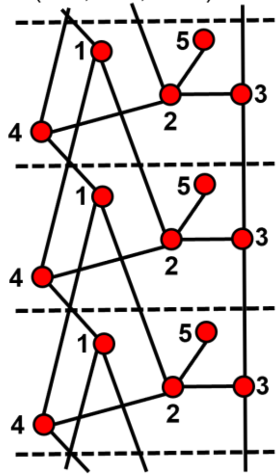	(8x**1**, 1x**2**, 2x**2**^{2}) **e-3** 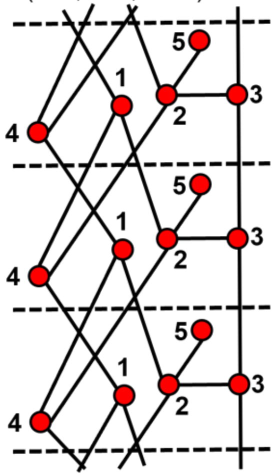	(6x**1**, 4x**2**) **a** 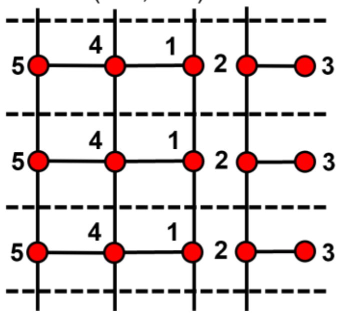

	(6x**1**, 4x**2**) **b** 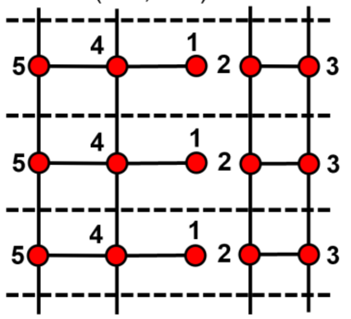	(6x**1**, 2x**2**, 2x**2**^{1}) **a** 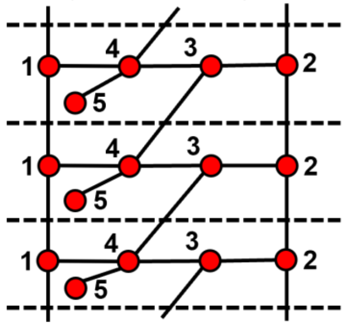	(6x**1**, 2x**2**, 2x**2**^{1}) **b** 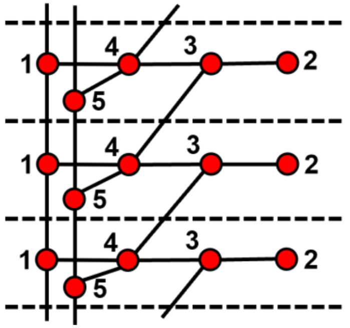	(6x**1**, 2x**2**, 2x**2**^{1}) **c** 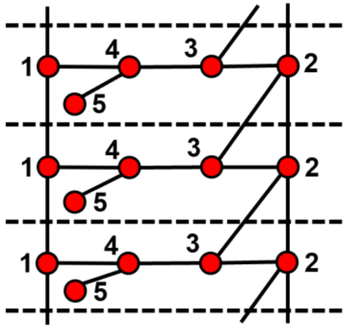
	(6x**1**, 2x**2**, 2x**2**^{1}) **d** 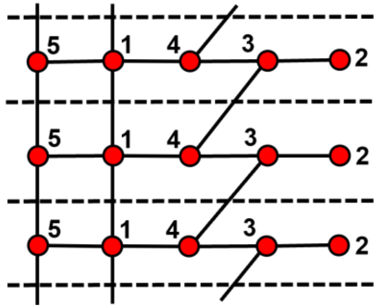	(6x**1**, 2x**2**, 2x**2**^{1}) **e** 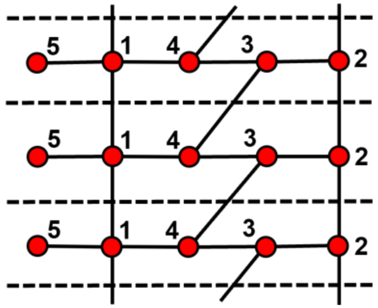	(6x**1**, 2x**2**, 2x**2**^{1}) **f** 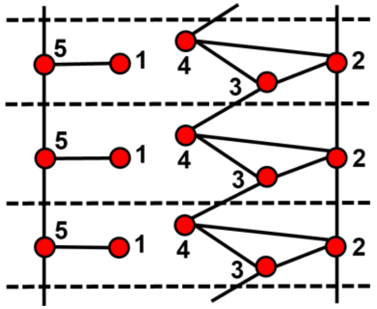	(6x**1**, 2x**2**, 2x**2**^{2}) **a** 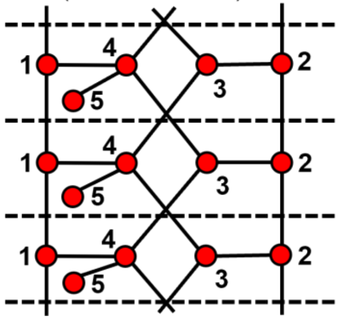
	(6x**1**, 2x**2**, 2x**2**^{2}) **b** 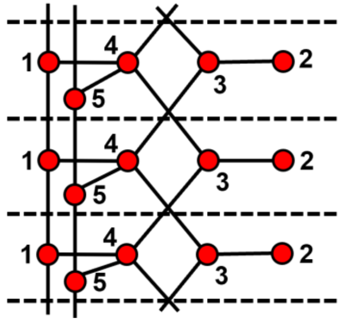	(6x**1**, 2x**2**, 2x**2**^{2}) **c** 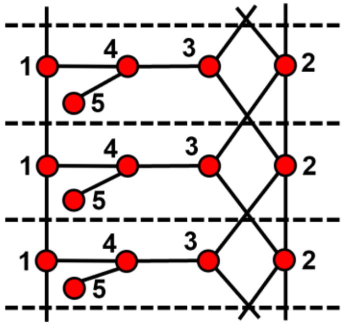	(6x**1**, 2x**2**, 2x**2**^{2}) **d** 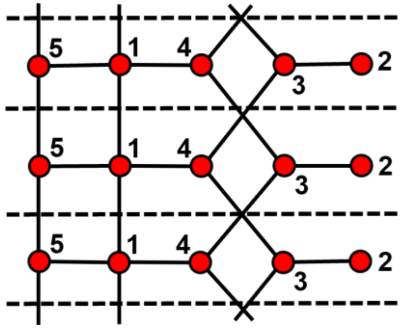	(6x**1**, 2x**2**, 2x**2**^{2}) **e** 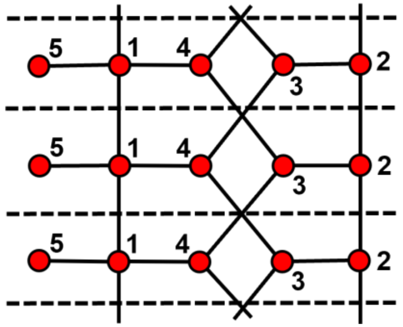

	(6x**1**, 2x**2**, 2x$\mathbf{2}^2$) **f** 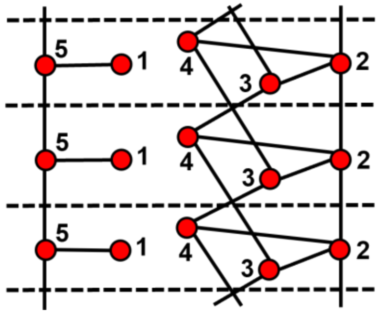	(6x**1**, 4x$\mathbf{2}^1$) **a-1** 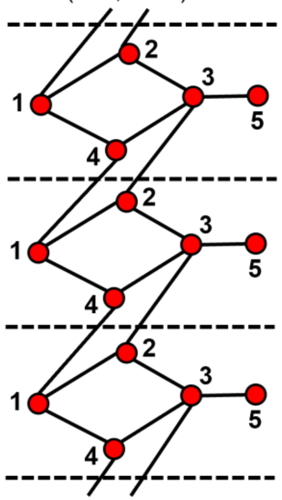	(6x**1**, 4x$\mathbf{2}^1$) **a-2** 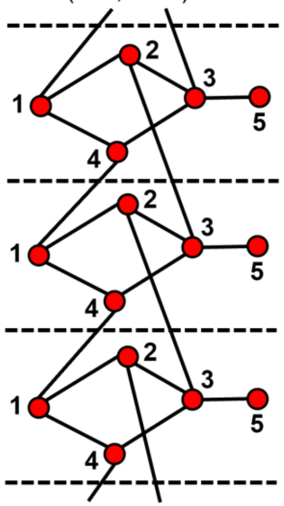	(6x**1**, 4x$\mathbf{2}^1$) **b-1** 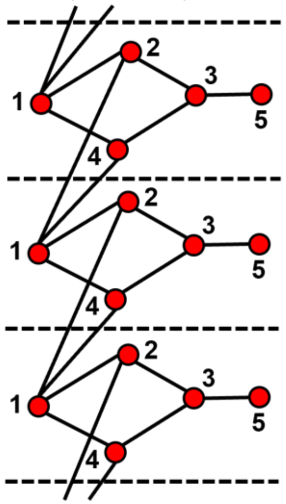
	(6x**1**, 4x$\mathbf{2}^1$) **b-2** 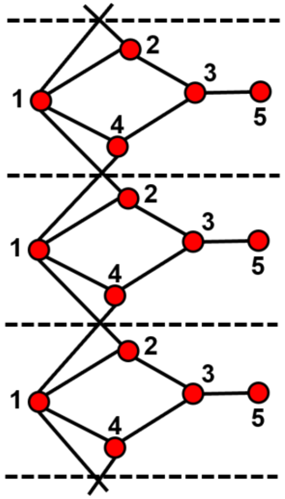	(6x**1**, 4x$\mathbf{2}^1$) **c-1** 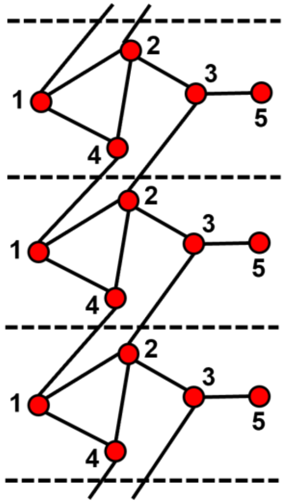	(6x**1**, 4x$\mathbf{2}^1$) **c-2** 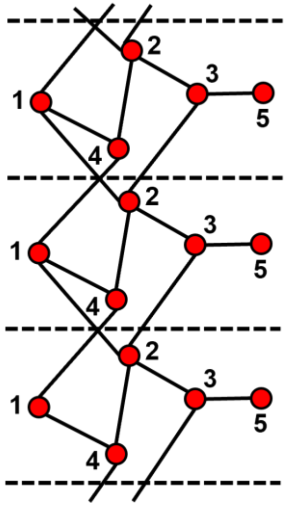	(6x**1**, 2x$\mathbf{2}^1$, 2x$\mathbf{2}^2$) **a-1** 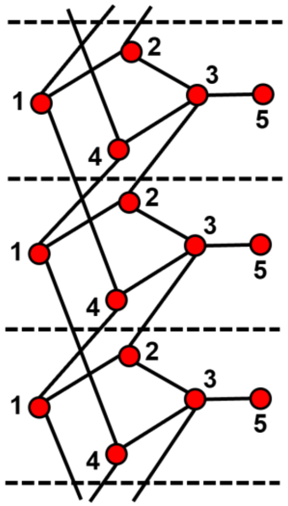

	(6x**1**, 2x**2**^{1}, 2x**2**^{2}) **a-2** 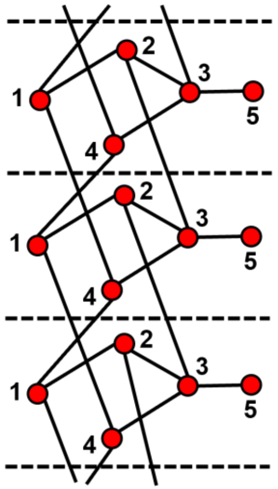	(6x**1**, 2x**2**^{1}, 2x**2**^{2}) **b** 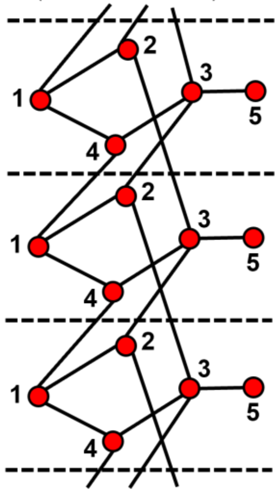	(6x**1**, 2x**2**^{1}, 2x**2**^{2}) **c-1** 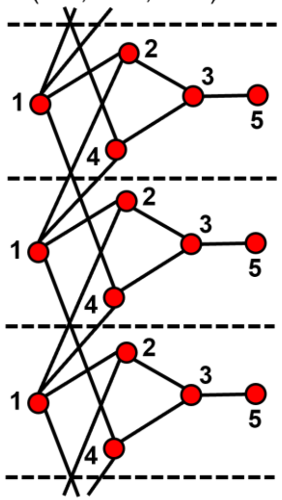	(6x**1**, 2x**2**^{1}, 2x**2**^{2}) **c-2** 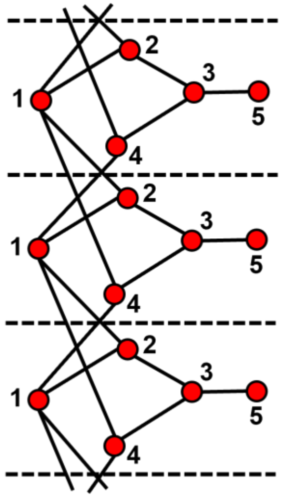
	(6x**1**, 2x**2**^{1}, 2x**2**^{2}) **d-1** 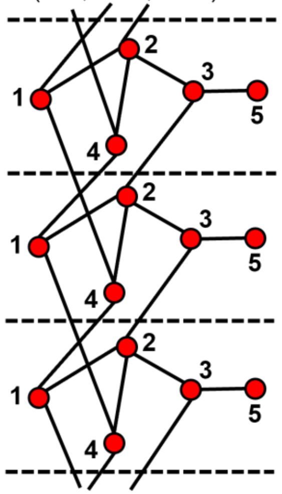	(6x**1**, 2x**2**^{1}, 2x**2**^{2}) **d-2** 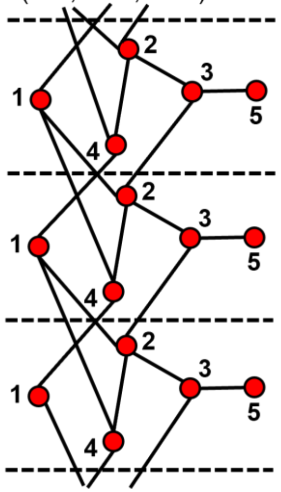	(6x**1**, 2x**2**^{1}, 2x**2**^{1}) **e-1** 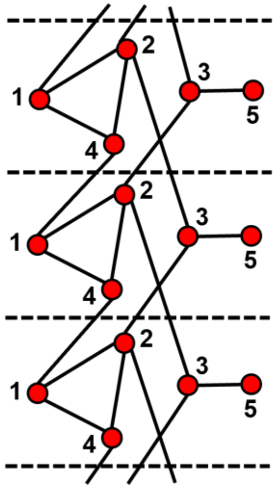	(6x**1**, 2x**2**^{1}, 2x**2**^{1}) **e-2** 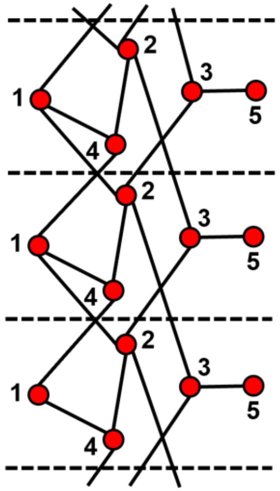

	(6x**1**, 4x$\mathbf{2}^2$) **a-1** 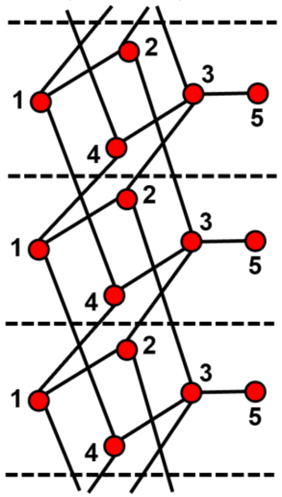	(6x**1**, 4x$\mathbf{2}^2$) **a-2** 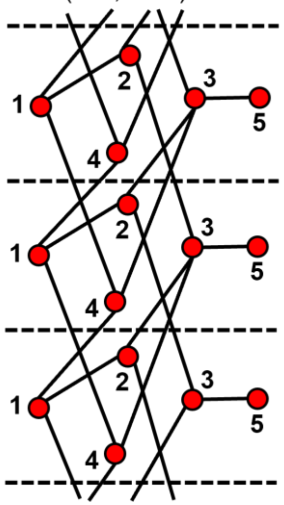	(6x**1**, 4x$\mathbf{2}^2$) **b-1** 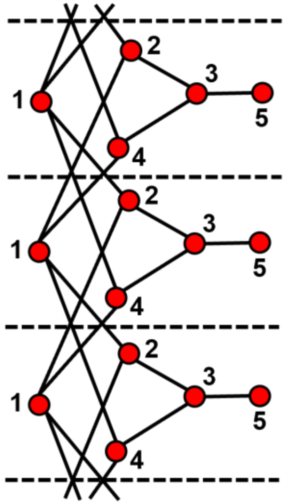	(6x**1**, 4x$\mathbf{2}^2$) **b-2** 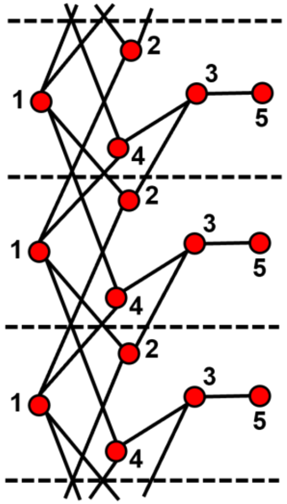
	(6x**1**, 4x$\mathbf{2}^2$) **c-1** 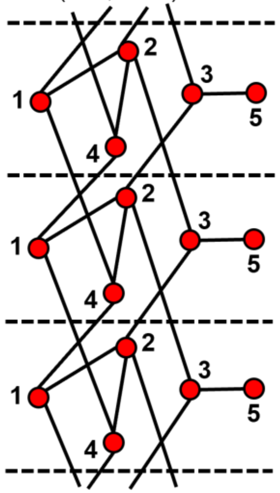	(6x**1**, 4x$\mathbf{2}^2$) **c-2** 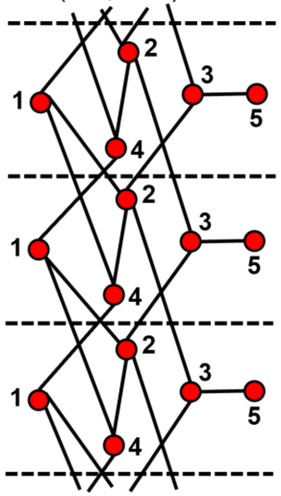	(4x**1**, 3x**2**, 2x$\mathbf{2}^1$) **a** 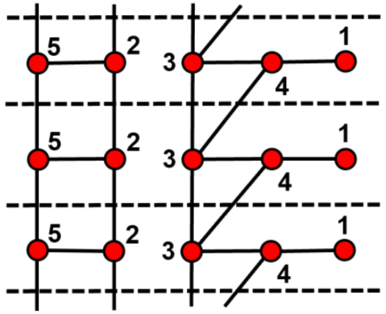	(4x**1**, 3x**2**, 2x$\mathbf{2}^1$) **b** 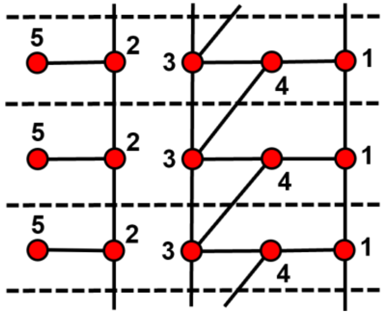

(4x**1**, 3x**2**, 2x$\mathbf{2}^{2}$) **a**

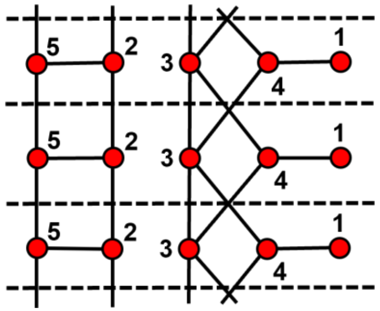

(4x**1**, 3x**2**, 2x$\mathbf{2}^{2}$) **b**

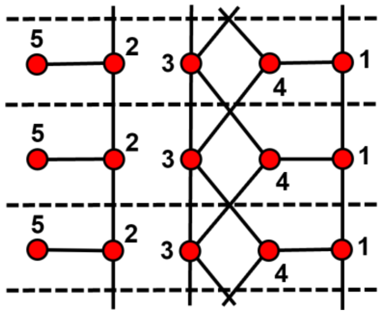

(4x**1**, 1x**2**, 4x$\mathbf{2}^{1}$) **a**

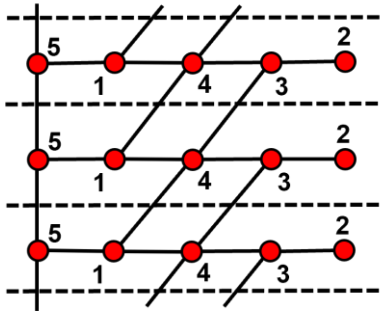

(4x**1**, 1x**2**, 4x$\mathbf{2}^{1}$) **b**

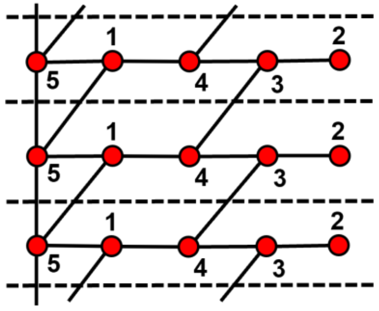

(4x**1**, 1x**2**, 4x$\mathbf{2}^{1}$) **c**

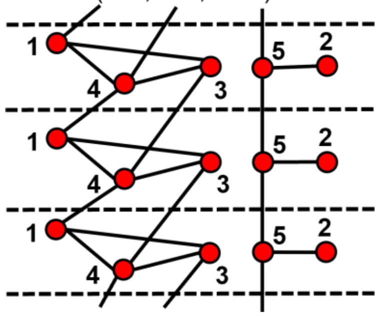

(4x**1**, 1x**2**, 2x$\mathbf{2}^{1}$, 2x$\mathbf{2}^{2}$) **a**

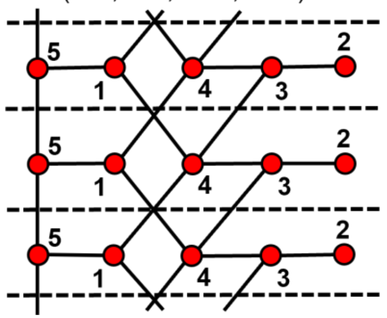

(4x**1**, 1x**2**, 2x$\mathbf{2}^{1}$, 2x$\mathbf{2}^{2}$) **b**

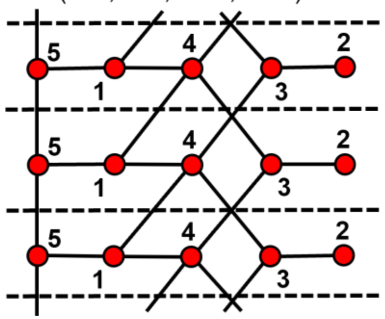

(4x**1**, 1x**2**, 2x$\mathbf{2}^{1}$, 2x$\mathbf{2}^{2}$) **c**

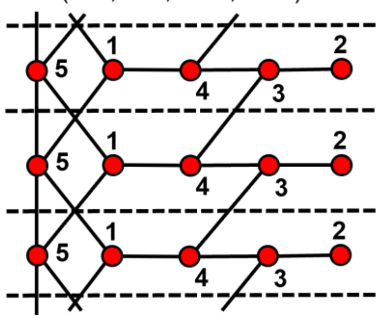

(4x**1**, 1x**2**, 2x$\mathbf{2}^{1}$, 2x$\mathbf{2}^{2}$) **d**

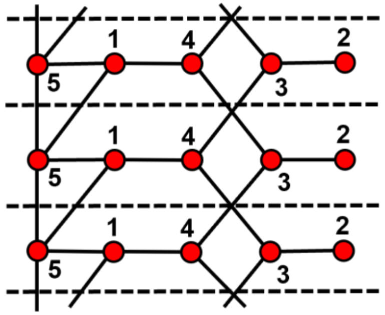

(4x**1**, 1x**2**, 2x$\mathbf{2}^{1}$, 2x$\mathbf{2}^{2}$) **e**

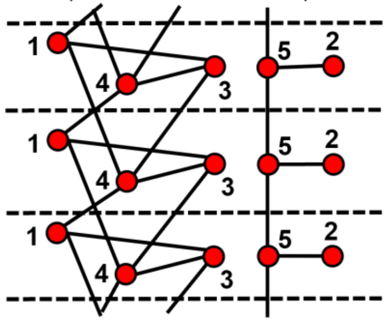

(4x**1**, 1x**2**, 4x$\mathbf{2}^{2}$) **a**

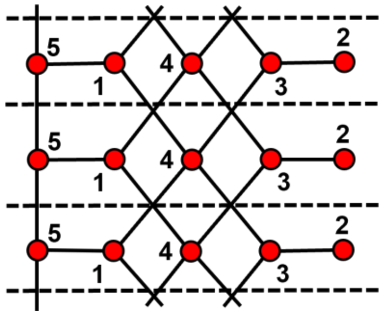

(4x**1**, 1x**2**, 4x$\mathbf{2}^{2}$) **b**

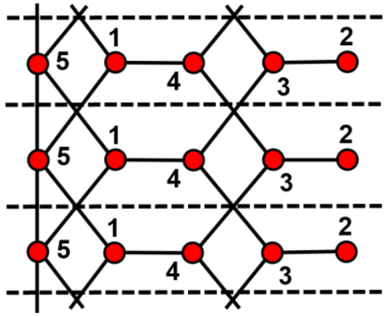

	(4x**1**, 1x**2**, 4x**2**^{2}) **c** 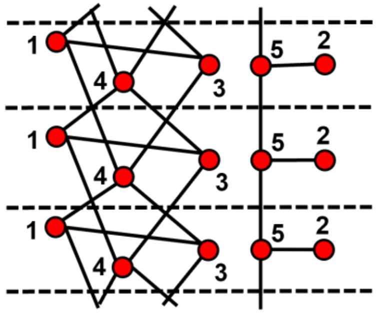			
$^1V_1{}^3V_3{}^4V_2$	NG			
$^1V_1{}^3V_3{}^4V_3$	NG			
$^1V_1{}^3V_3{}^4V_4$	NG			
$^1V_1{}^3V_5{}^4V_1$	NG			
$^1V_1{}^3V_5{}^4V_2$	NG			
$^1V_2{}^3V_2{}^4V_1$	(10x**1**, 1x**2**) **a** 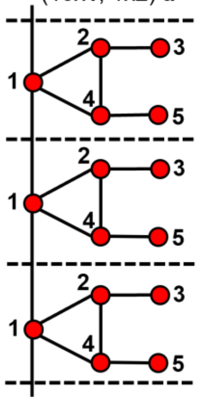	(10x**1**, 1x**2**) **b** 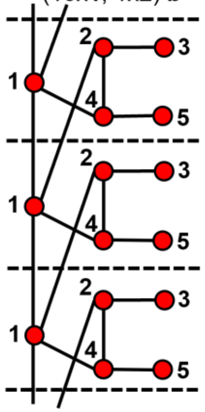	(10x**1**, 1x**2**) **c** 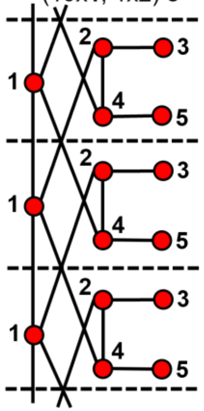	(10x**1**, 1x**2**) **d** 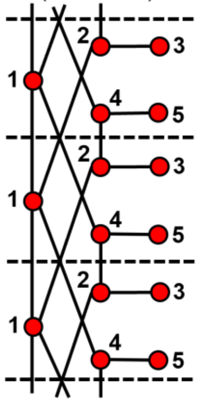

	(8x**1**, 2x**2**) **a** 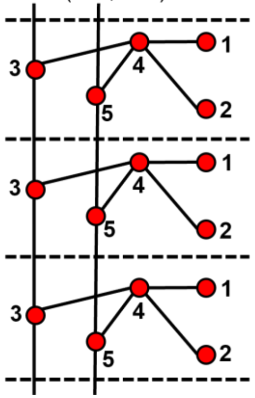	(8x**1**, 2x**2**) **b** 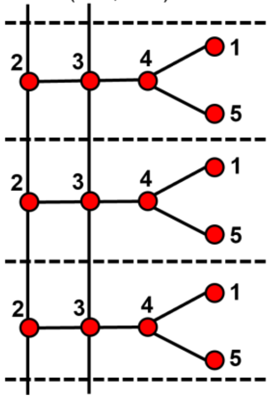	(8x**1**, 2x**2**) **c** 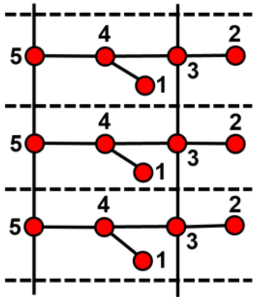	(8x**1**, 2x**2**^{1}) **a-1** 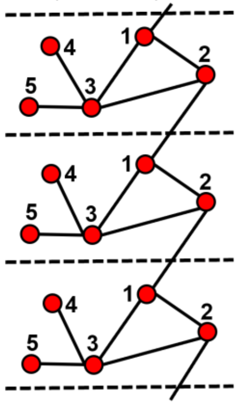
	(8x**1**, 2x**2**^{1}) **a-2** 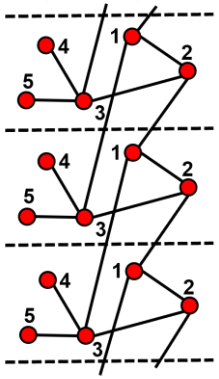	(8x**1**, 2x**2**^{1}) **a-3** 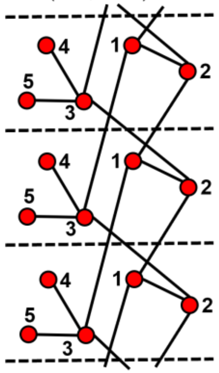	(8x**1**, 2x**2**^{1}) **b-1** 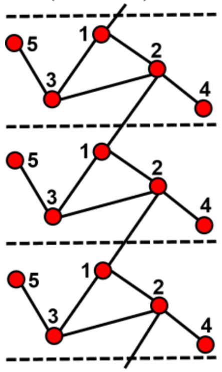	(8x**1**, 2x**2**^{1}) **b-2** 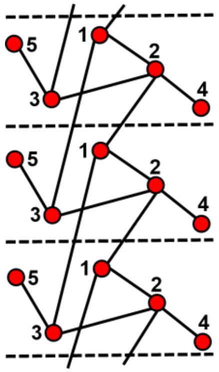

	(8x**1**, 2x$\mathbf{2}^1$) **b-3** 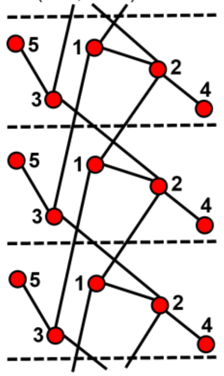	(8x**1**, 2x$\mathbf{2}^2$) **a-1** 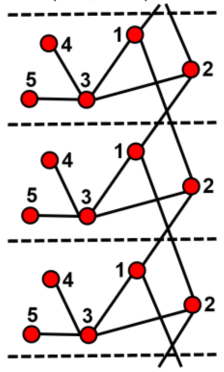	(8x**1**, 2x$\mathbf{2}^2$) **a-2** 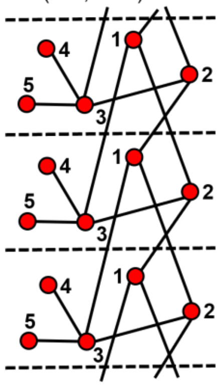	(8x**1**, 2x$\mathbf{2}^2$) **a-3** 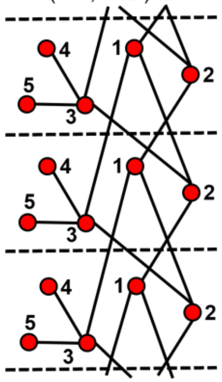
	(8x**1**, 2x$\mathbf{2}^2$) **b-1** 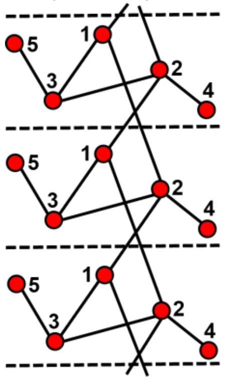	(8x**1**, 2x$\mathbf{2}^2$) **b-2** 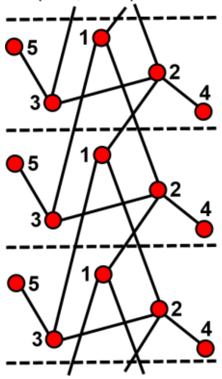	(8x**1**, 2x$\mathbf{2}^2$) **b-3** 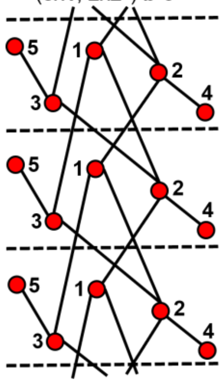	(6x**1**, 3x**2**) **a** 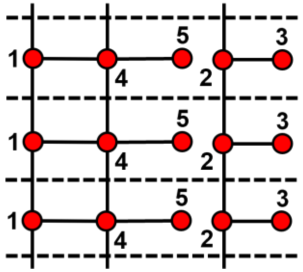

(6x**1**, 3x**2**) **b**

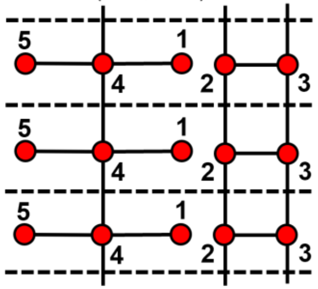

(6x**1**, 3x**2**) **c**

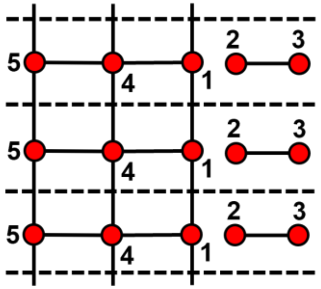

(6x**1**, 1x**2**, 2x$\mathbf{2}^{1}$) **a**

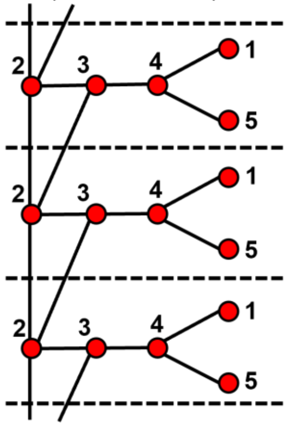

(6x**1**, 1x**2**, 2x$\mathbf{2}^{1}$) **b**

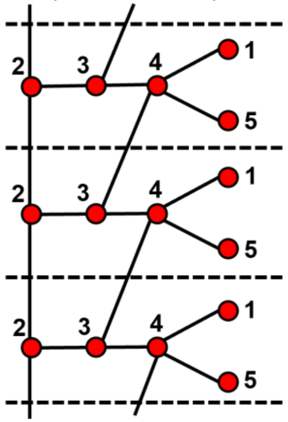

(6x**1**, 1x**2**, 2x$\mathbf{2}^{1}$) **c-1**

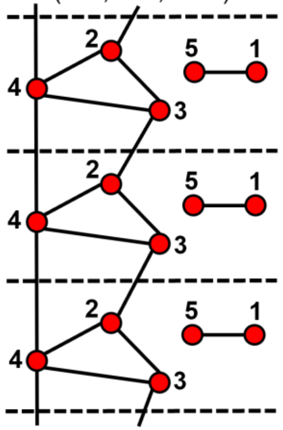

(6x**1**, 1x**2**, 2x$\mathbf{2}^{1}$) **c-2**

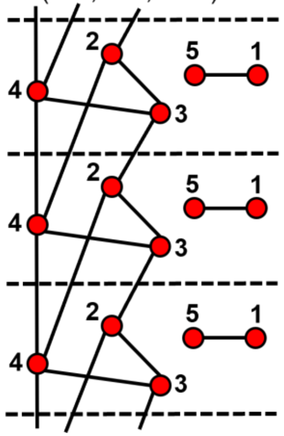

(6x**1**, 1x**2**, 2x$\mathbf{2}^{1}$) **c-3**

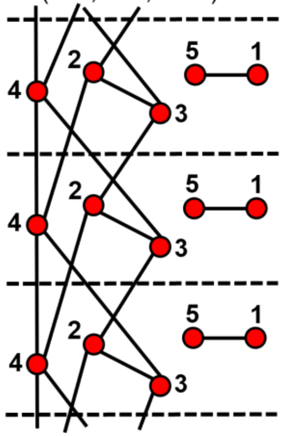

(6x**1**, 1x**2**, 2x$\mathbf{2}^{1}$) **d**

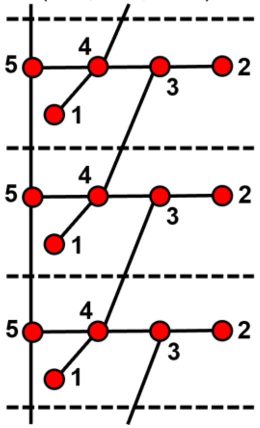

	(6x**1**, 1x**2**, 2x**2**^{1}) **e**<br>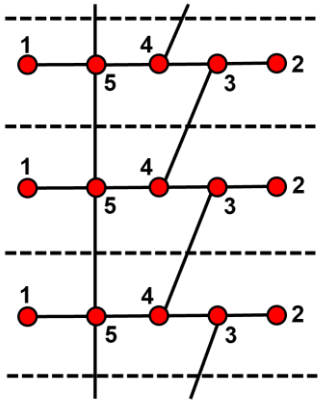	(6x**1**, 1x**2**, 2x**2**^{2}) **a**<br>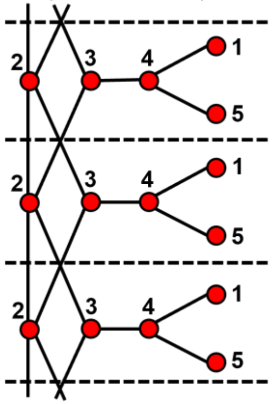	(6x**1**, 1x**2**, 2x**2**^{2}) **b**<br>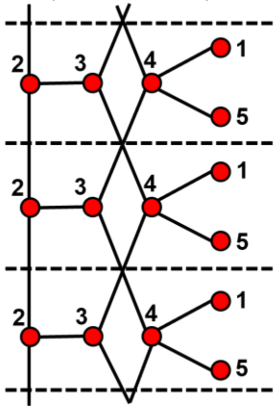	(6x**1**, 1x**2**, 2x**2**^{2}) **c-1**<br>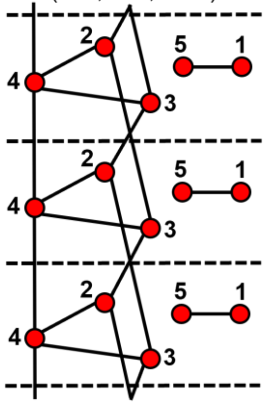
	(6x**1**, 1x**2**, 2x**2**^{2}) **c-2**<br>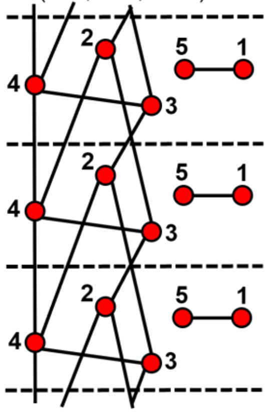	(6x**1**, 1x**2**, 2x**2**^{1}) **c-3**<br>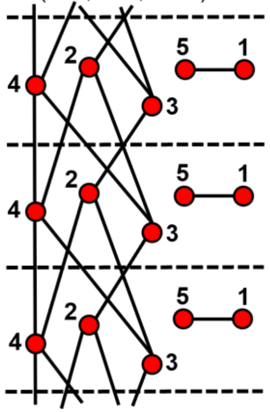	(6x**1**, 1x**2**, 2x**2**^{2}) **d**<br>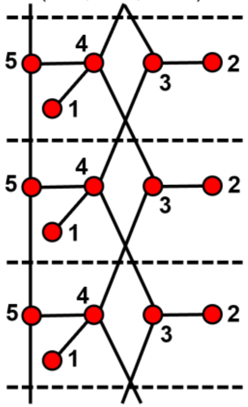	(6x**1**, 1x**2**, 2x**2**^{2}) **e**<br>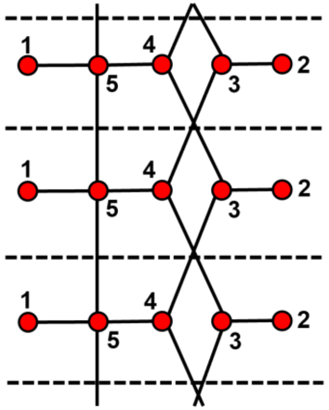

	(4x**1**, 2x**2**, 2x**2**^{1}) **a**<br>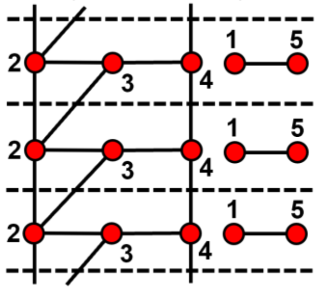	(4x**1**, 2x**2**, 2x**2**^{1}) **b**<br>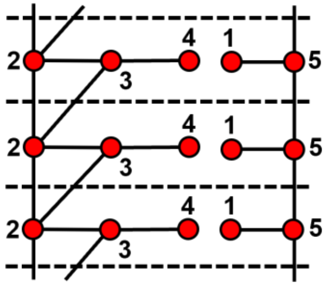	(4x**1**, 2x**2**, 2x**2**^{2}) **a**<br>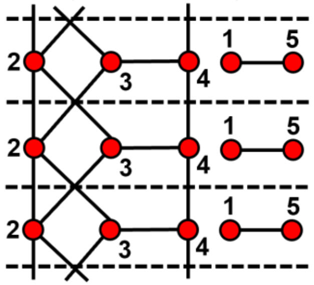	(4x**1**, 2x**2**, 2x**2**^{2}) **b**<br>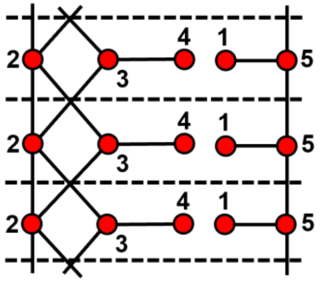
	(4x**1**, 4x**2**^{1}) **a-1**<br>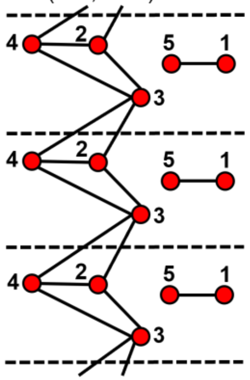	(4x**1**, 4x**2**^{1}) **a-2**<br>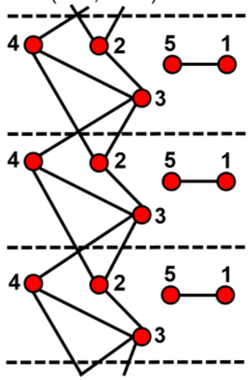	(4x**1**, 4x**2**^{1}) **a-3**<br>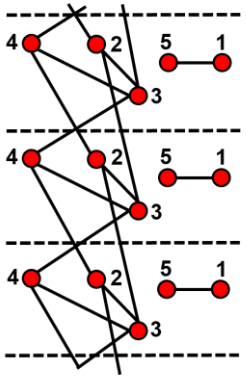	(4x**1**, 4x**2**^{1}) **b**<br>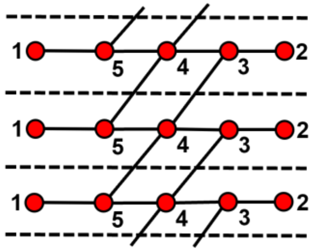
	(4x**1**, 2x**2**^{1}, 2x**2**^{2}) **a-1**<br>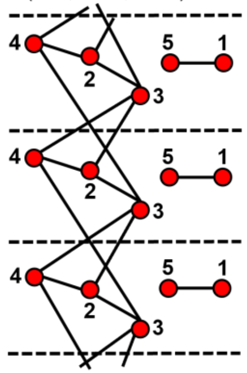	(4x**1**, 2x**2**^{1}, 2x**2**^{2}) **a-2**<br>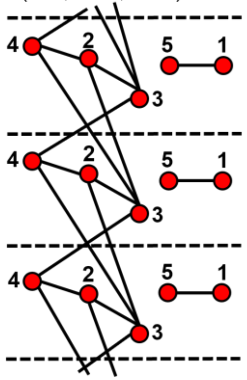	(4x**1**, 2x**2**^{1}, 2x**2**^{2}) **a-3**<br>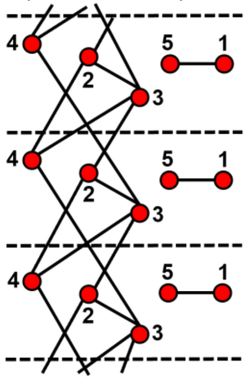	(4x**1**, 2x**2**^{1}, 2x**2**^{2}) **b**<br>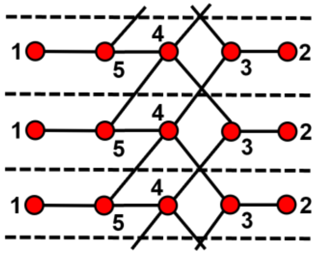

	(4x**1**, 4x**2**^{2}) **a-1** 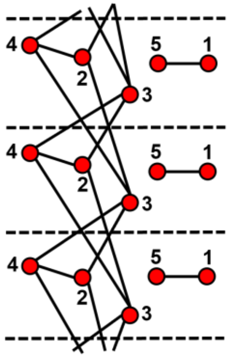	(4x**1**, 4x**2**^{2}) **a-2** 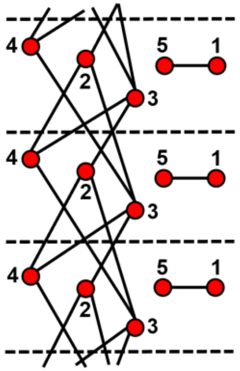	(4x**1**, 4x**2**^{2}) **b** 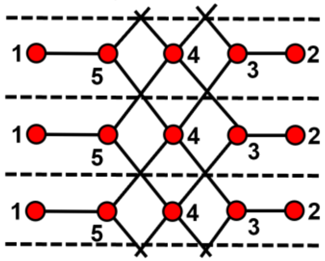	
$^1V_2{}^3V_2{}^4V_2$	NG			
$^1V_2{}^3V_2{}^4V_3$	NG			
$^1V_2{}^3V_2{}^4V_4$	NG			
$^1V_2{}^3V_4{}^4V_1$	NG			
$^1V_2{}^3V_4{}^4V_2$	NG			
$^1V_3{}^3V_1{}^4V_1$	(8x**1**, 1x**2**) **a** 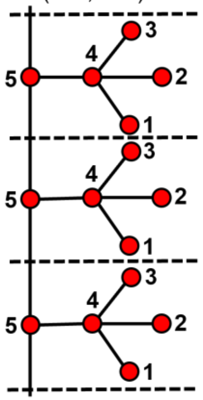	(8x**1**, 1x**2**) **b** 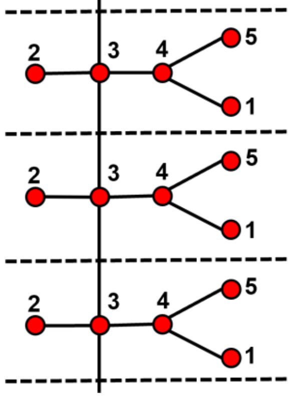	(6x**1**, 2x**2**) **a** 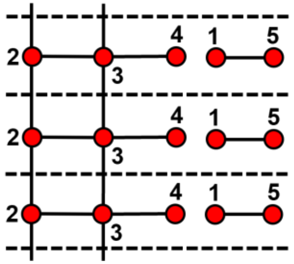	(6x**1**, 2x**2**) **b** 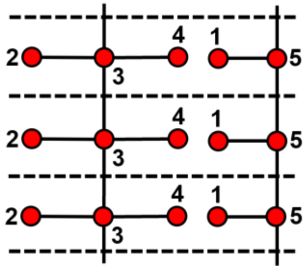

	(6x**1**, 2x**2**^{1})<br>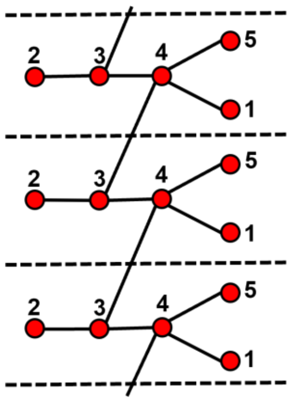	(6x**1**, 2x**2**^{2})<br>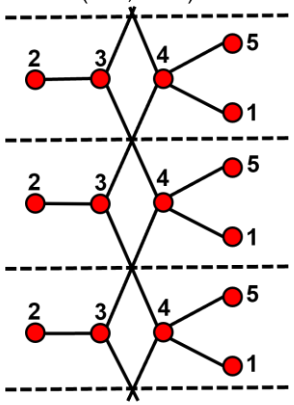	(4x**1**, 1x**2**, 2x**2**^{1})<br>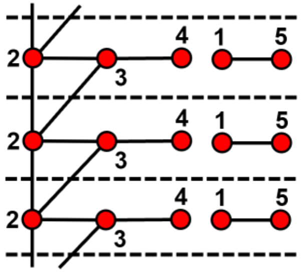	(4x**1**, 1x**2**, 2x**2**^{2})<br>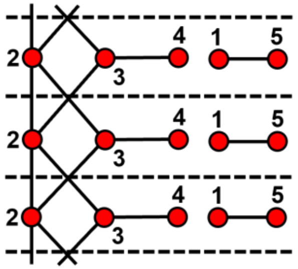
$^1V_3{}^3V_1{}^4V_2$	NG			
$^1V_3{}^3V_1{}^4V_3$	NG			
$^1V_3{}^3V_1{}^4V_4$	NG			
$^1V_3{}^3V_3{}^4V_1$	NG			
$^1V_3{}^3V_3{}^4V_2$	NG			
$^1V_4{}^3V_2{}^4V_1$	NG			
$^1V_4{}^3V_2{}^4V_2$	NG			
$^1V_5{}^3V_1{}^4V_1$	NG			
$^1V_5{}^3V_1{}^4V_2$	NG			
$\boldsymbol{^2V_r{}^3V_r{}^4V_r}$				

$^2V_1{}^3V_2{}^4V_1$	(10x**1**, 1x**2**) **a** 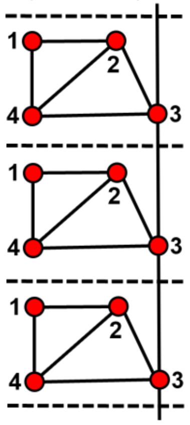	(10x**1**, 1x**2**) **b** 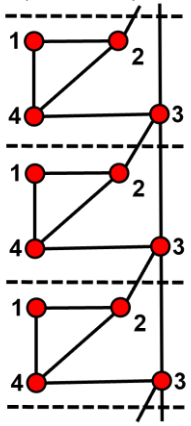	(10x**1**, 1x**2**) **c** 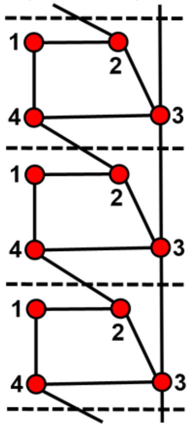	(10x**1**, 1x**2**) **d** 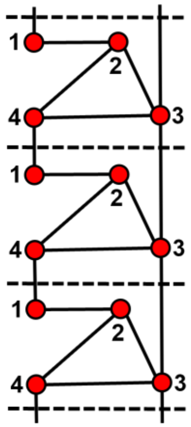
	(10x**1**, 1x**2**) **e** 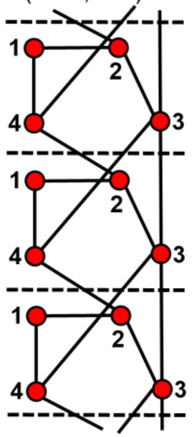	(10x**1**, 1x**2**) **f** 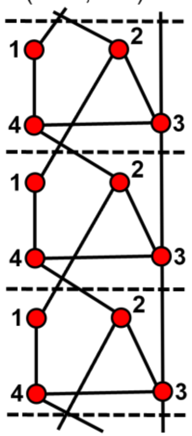	(10x**1**, 1x**2**) **g** 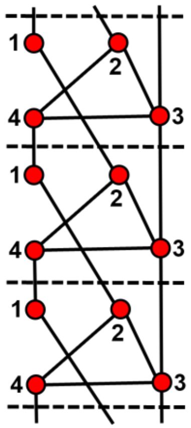	(10x**1**, 1x**2**) **h** 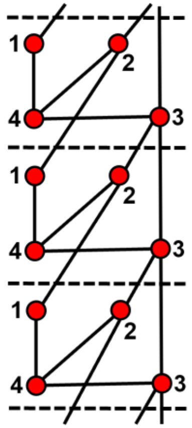

	(10x**1**, 1x**2**) **i** 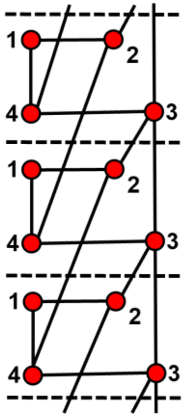	(10x**1**, 1x**2**) **j** 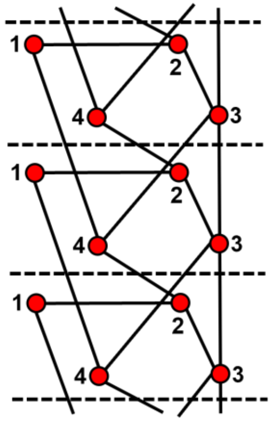	(10x**1**, 1x**2**) **k** 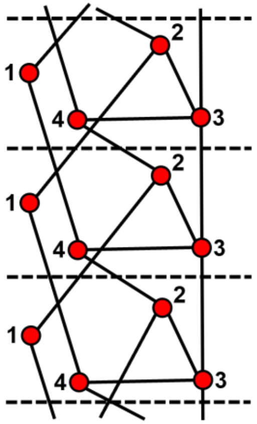	(10x**1**, 1x**2**) **l** 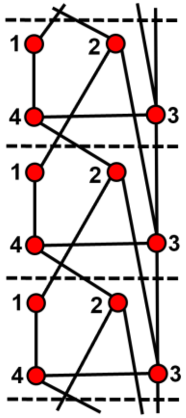
	(10x**1**, 1x**2**) **m** 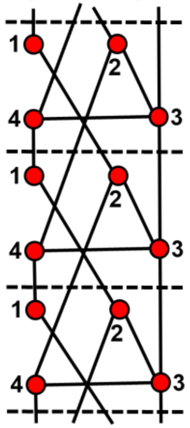	(10x**1**, 1x**2**) **n** 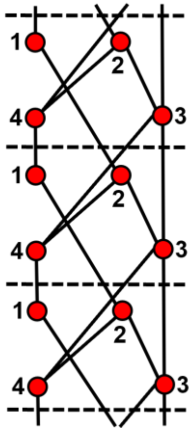	(10x**1**, 1x**2**) **o** 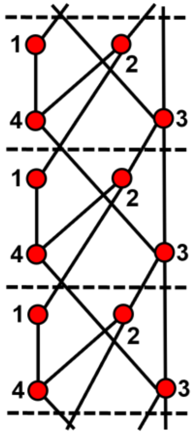	(10x**1**, 1x**2**) **p** 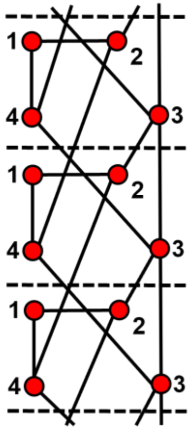

	(10x**1**, 1x**2**) **q** 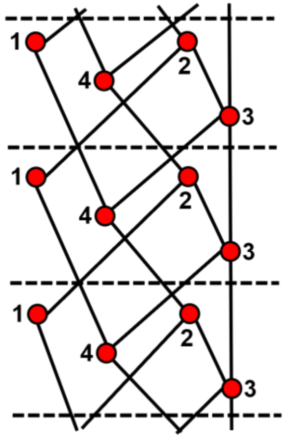	(10x**1**, 1x**2**) **r** 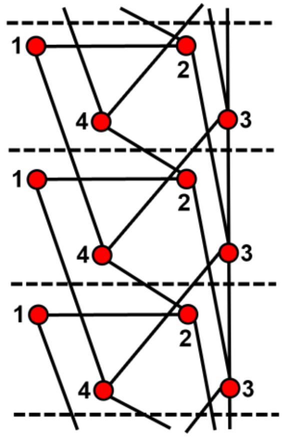	(10x**1**, 1x**2**) **s** 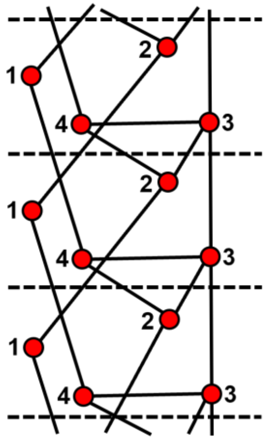	(10x**1**, 1x**2**) **t** 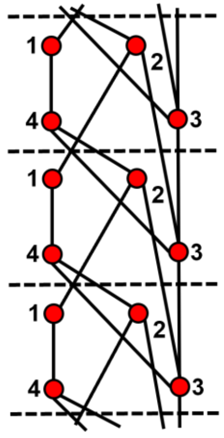
	(10x**1**, 1x**2**) **u** 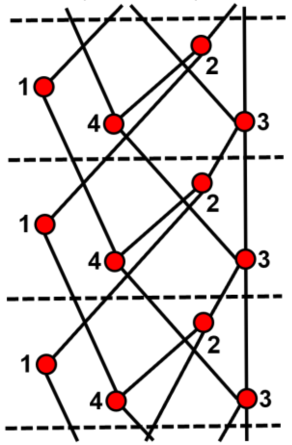	(10x**1**, 1x**2**) **v** 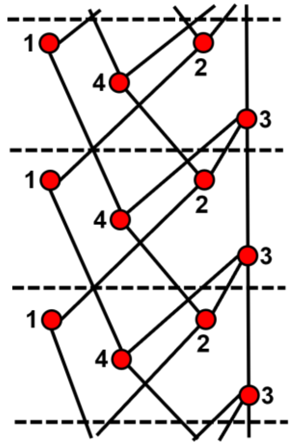	(10x**1**, 1x**2**) **w** 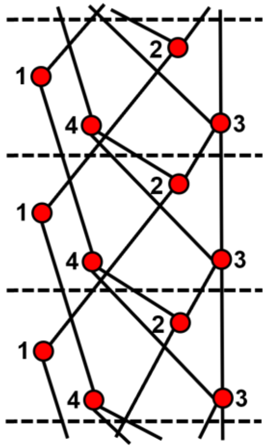	(10x**1**, 1x**2**) **x** 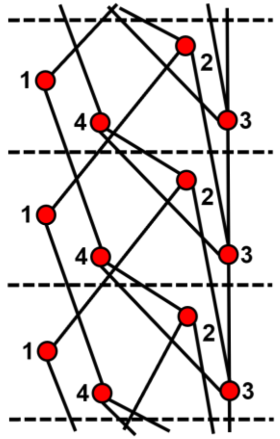

	(8x**1**, 2x**2**) **a** 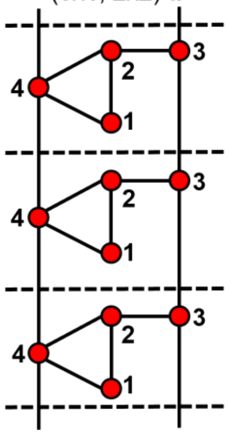	(8x**1**, 2x**2**) **b** 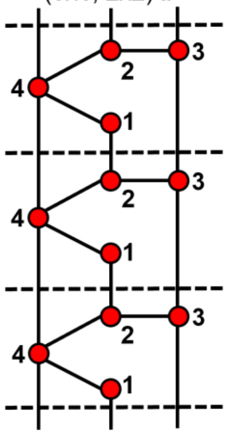	(8x**1**, 2x**2**) **c** 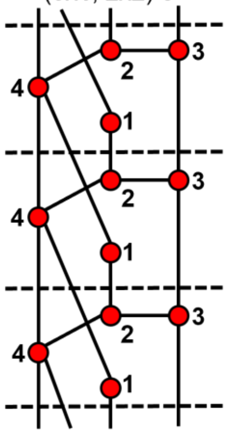	(8x**1**, 2x**2**) **d** 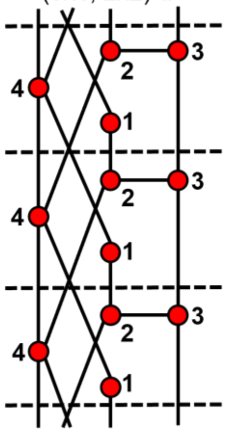
	(8x**1**, 2x**2**^{1}) **a** 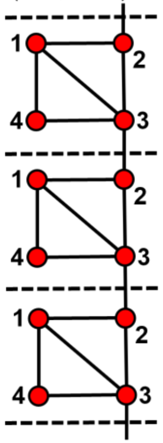	(8x**1**, 2x**2**^{1}) **b** 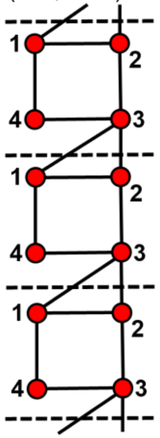	(8x**1**, 2x**2**^{1}) **c** 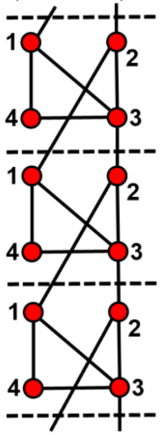	(8x**1**, 2x**2**^{1}) **d** 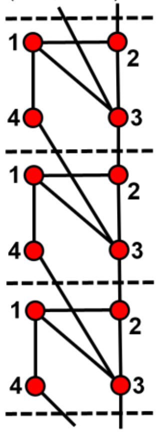

	(8x**1**, 2x$\mathbf{2}^1$) **e** 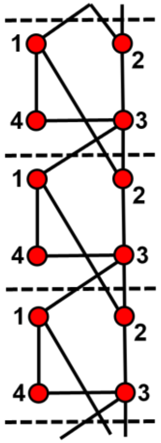	(8x**1**, 2x$\mathbf{2}^1$) **f** 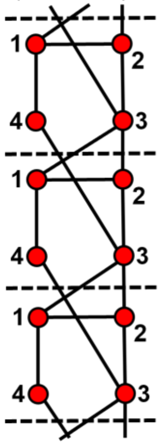	(8x**1**, 2x$\mathbf{2}^1$) **g** 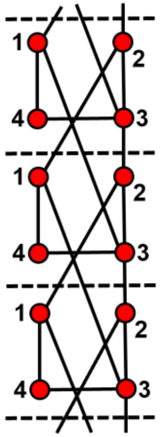	(8x**1**, 2x$\mathbf{2}^1$) **h** 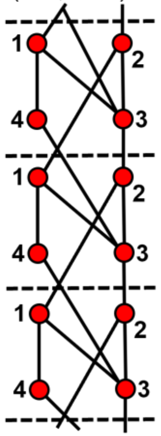
	(8x**1**, 2x$\mathbf{2}^1$) **i** 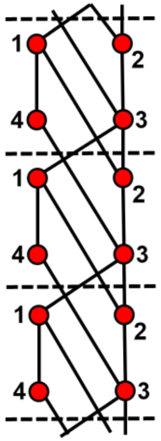	(8x**1**, 2x$\mathbf{2}^1$) **j** 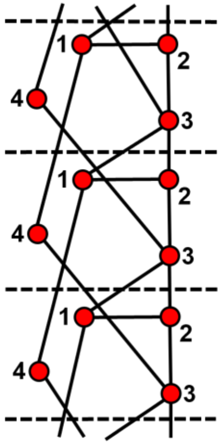	(8x**1**, 2x$\mathbf{2}^1$) **k** 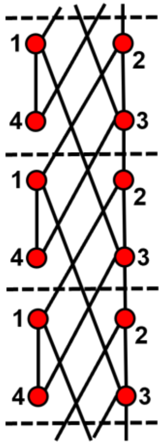	(8x**1**, 2x$\mathbf{2}^1$) **l** 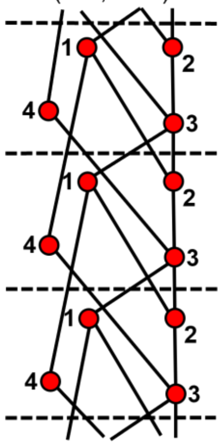

	(10x**1**, 2x**2**^{2}) **a**<br>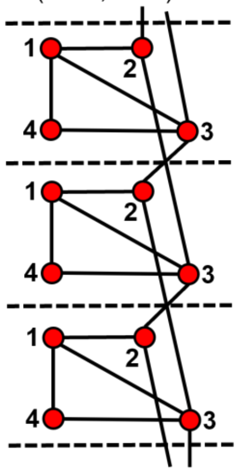	(10x**1**, 2x**2**^{2}) **b**<br>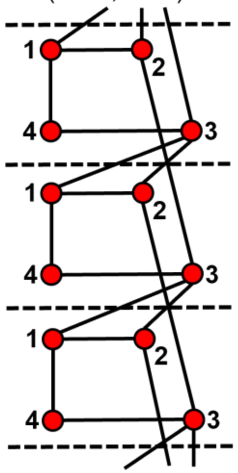	(10x**1**, 2x**2**^{2}) **c**<br>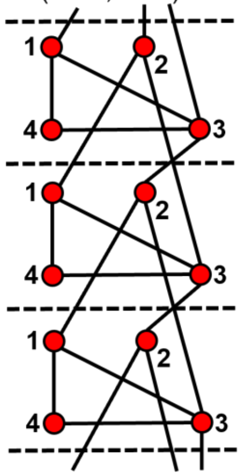	(10x**1**, 2x**2**^{2}) **d**<br>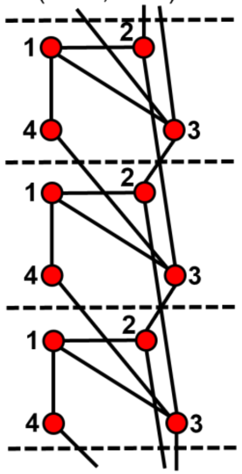
	(10x**1**, 2x**2**^{2}) **e**<br>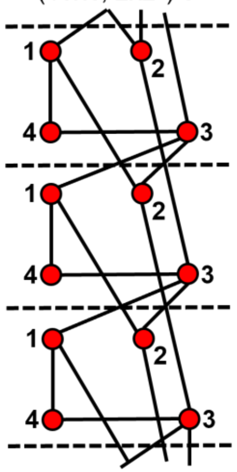	(10x**1**, 2x**2**^{2}) **f**<br>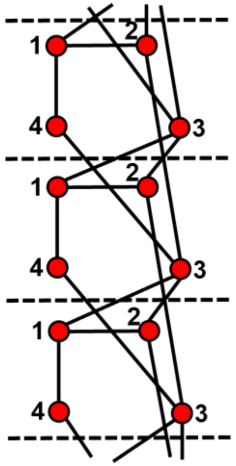	(10x**1**, 2x**2**^{2}) **g**<br>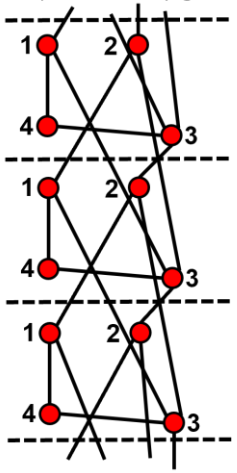	(10x**1**, 2x**2**^{2}) **h**<br>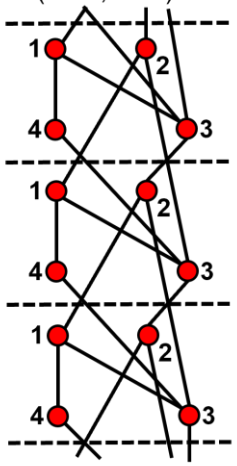

(10x**1**, 2x**2**²) **i**

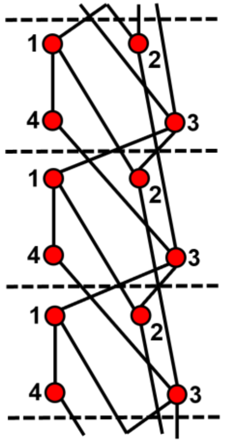

(10x**1**, 2x**2**²) **j**

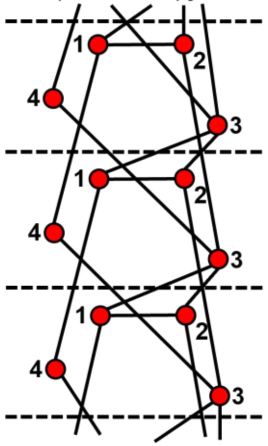

(10x**1**, 2x**2**²) **k**

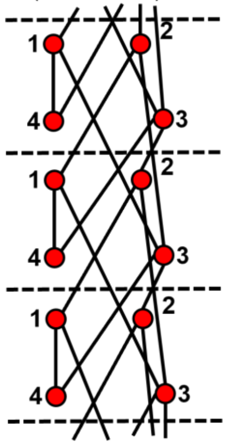

(10x**1**, 2x**2**²) **l**

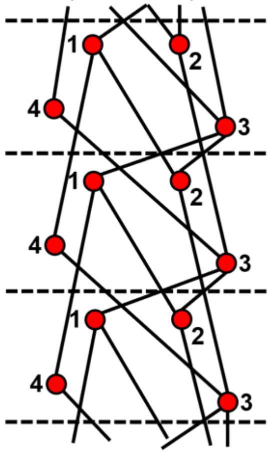

(6x**1**, 3x**2**)

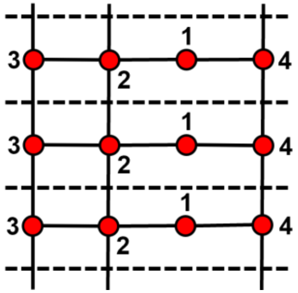

(6x**1**, 1x**2**, 2x**2**¹) **a-1**

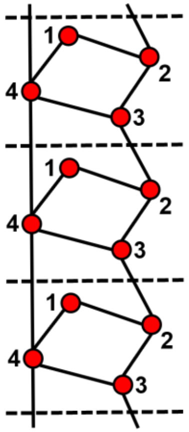

(6x**1**, 1x**2**, 2x**2**¹) **a-2**

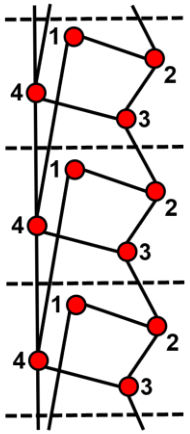

(6x**1**, 1x**2**, 2x**2**¹) **a-3**

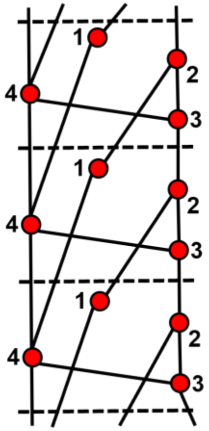

	(6x**1**, 1x**2**, 2x**2**^{1}) **a-4** 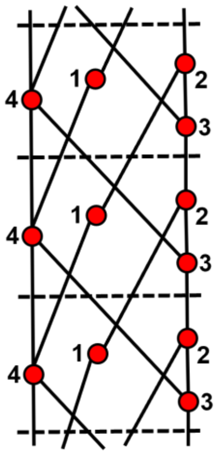	(6x**1**, 1x**2**, 2x**2**^{1}) **b-1** 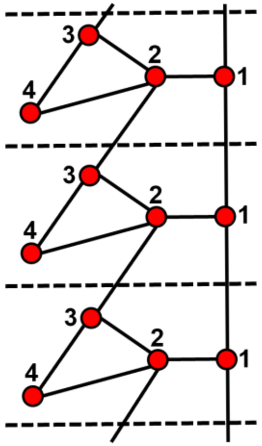	(6x**1**, 1x**2**, 2x**2**^{1}) **b-2** 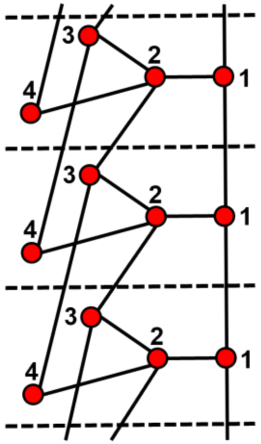	(6x**1**, 1x**2**, 2x**2**^{1}) **b-3** 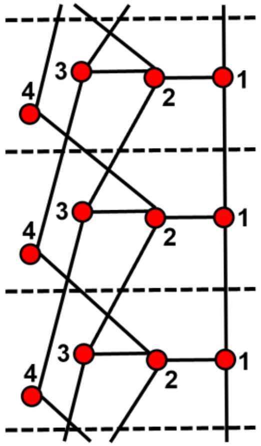
	(6x**1**, 1x**2**, 2x**2**^{2}) **a-1** 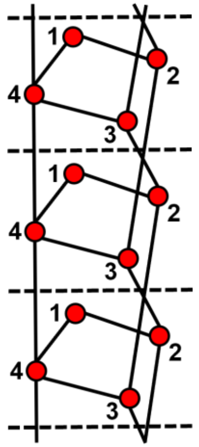	(6x**1**, 1x**2**, 2x**2**^{2}) **a-2** 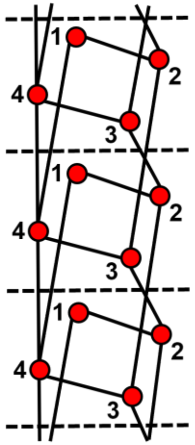	(6x**1**, 1x**2**, 2x**2**^{2}) **a-3** 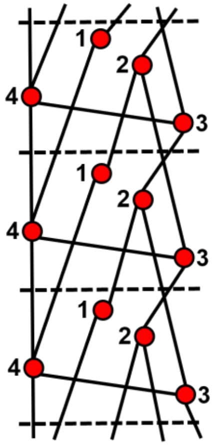	(6x**1**, 1x**2**, 2x**2**^{2}) **a-4** 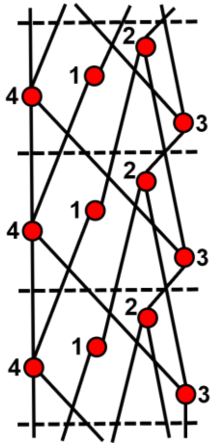

	(6x**1**, 1x**2**, 2x**2**^{2}) **b-1** 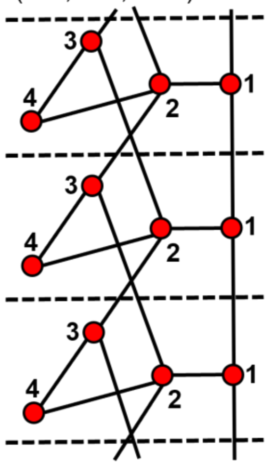	(6x**1**, 1x**2**, 2x**2**^{2}) **b-2** 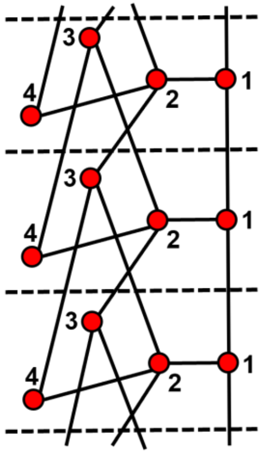	(6x**1**, 1x**2**, 2x**2**^{2}) **b-3** 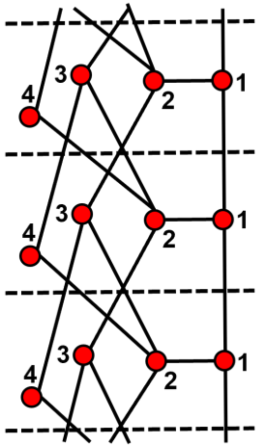	(4x**1**, 4x**2**) 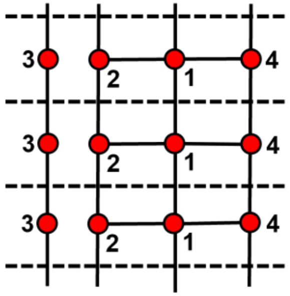
	(4x**1**, 2x**2**, 2x**2**^{1}) **a** 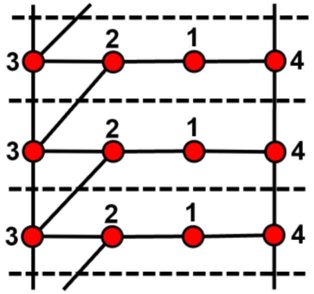	(4x**1**, 2x**2**, 2x**2**^{1}) **b** 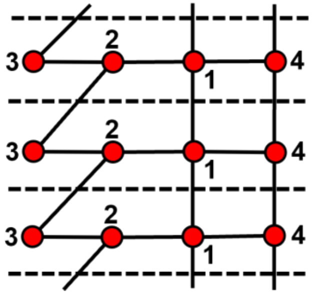	(4x**1**, 2x**2**, 2x**2**^{1}) **c** 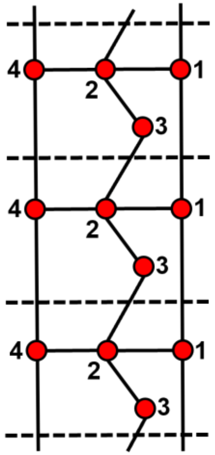	(4x**1**, 2x**2**, 2x**2**^{1}) **d-1** 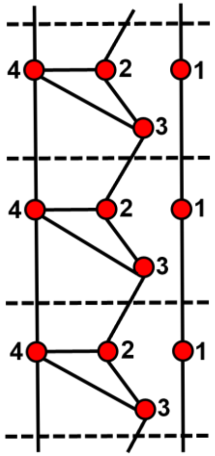

	(4x**1**, 2x**2**, 2x**2**^{1}) **d-2**<br>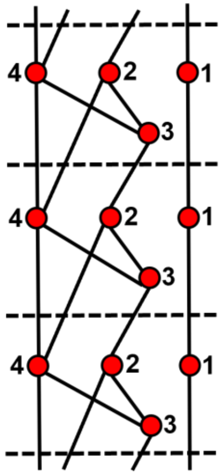	(4x**1**, 2x**2**, 2x**2**^{1}) **d-3**<br>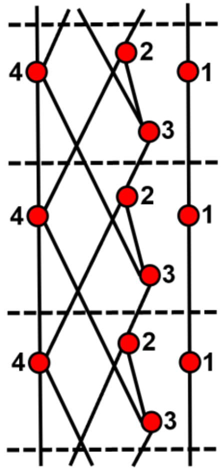	(4x**1**, 2x**2**, 2x**2**^{2}) **a**<br>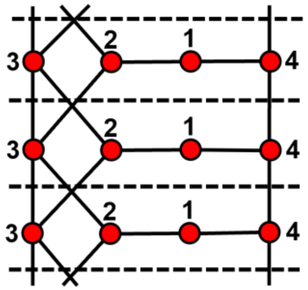	(4x**1**, 2x**2**, 2x**2**^{2}) **b**<br>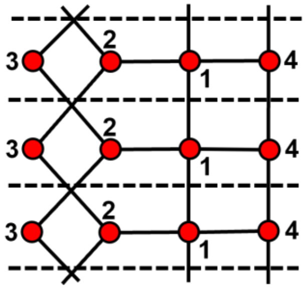
	(4x**1**, 2x**2**, 2x**2**^{2}) **c**<br>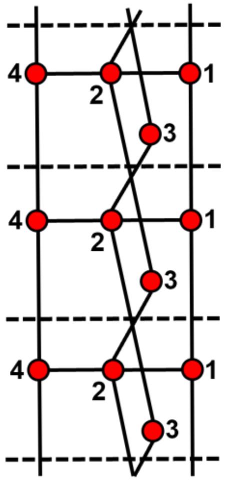	(4x**1**, 2x**2**, 2x**2**^{2}) **d-1**<br>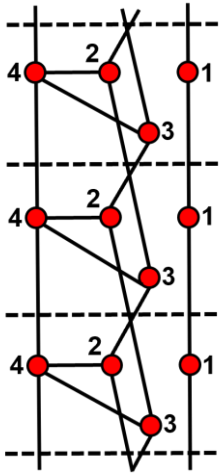	(4x**1**, 2x**2**, 2x**2**^{2}) **d-2**<br>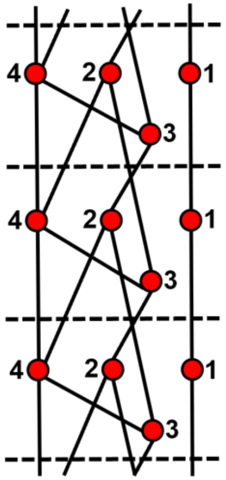	(4x**1**, 2x**2**, 2x**2**^{2}) **d-3**<br>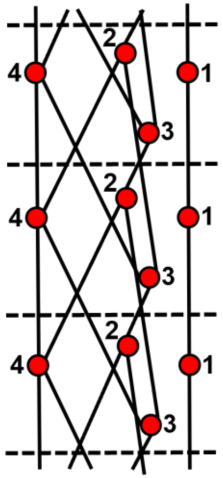

	(4x**1**, 4x$\mathbf{2}^1$) **a-1**<br>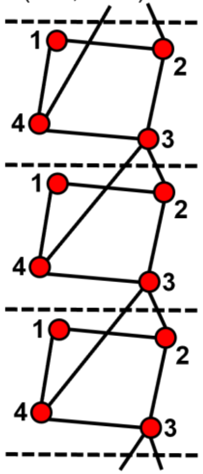	(4x**1**, 4x$\mathbf{2}^1$) **a-2**<br>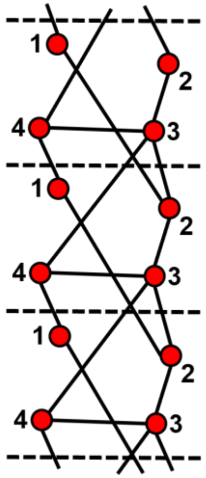	(4x**1**, 4x$\mathbf{2}^1$) **b-1**<br>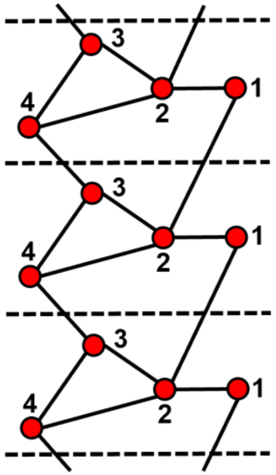	(4x**1**, 4x$\mathbf{2}^1$) **b-2**<br>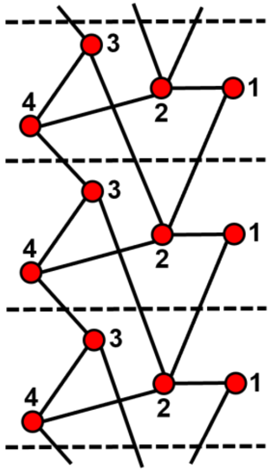
	(4x**1**, 4x$\mathbf{2}^1$) **b-3**<br>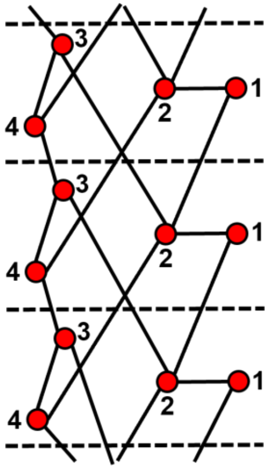	(4x**1**, 2x$\mathbf{2}^1$, 2x$\mathbf{2}^2$) **a-1**<br>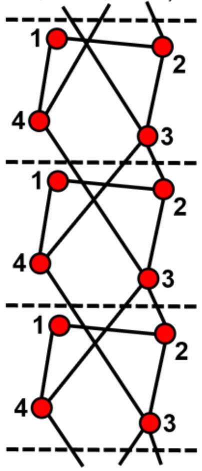	(4x**1**, 2x$\mathbf{2}^1$, 2x$\mathbf{2}^2$) **a-2**<br>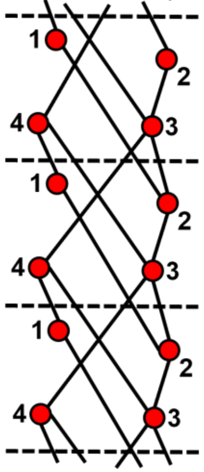	(4x**1**, 2x$\mathbf{2}^1$, 2x$\mathbf{2}^2$) **b-1**<br>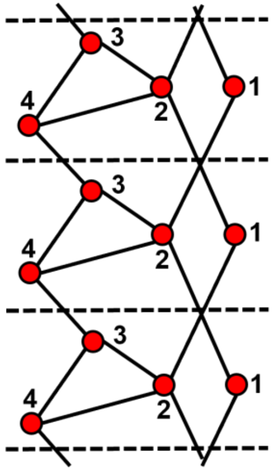

	(4x**1**, 2x$\mathbf{2}^1$, 2x$\mathbf{2}^2$) **b-2**<br>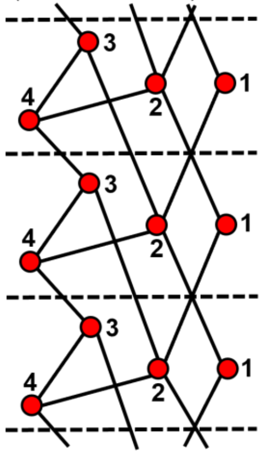	(4x**1**, 2x$\mathbf{2}^1$, 2x$\mathbf{2}^2$) **b-3**<br>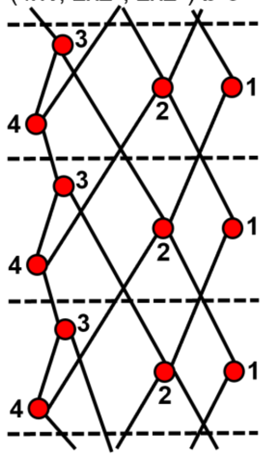	(4x**1**, 2x$\mathbf{2}^1$, 2x$\mathbf{2}^2$) **c-1**<br>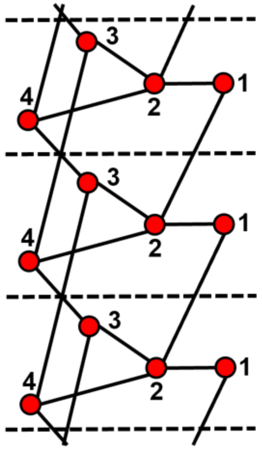	(4x**1**, 2x$\mathbf{2}^1$, 2x$\mathbf{2}^2$) **c-2**<br>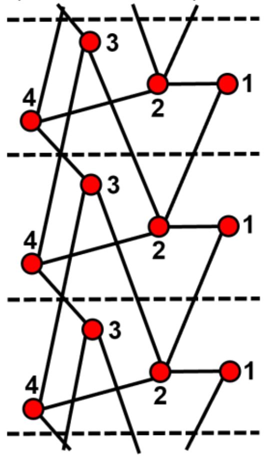
	(4x**1**, 2x$\mathbf{2}^1$, 2x$\mathbf{2}^2$) **c-3**<br>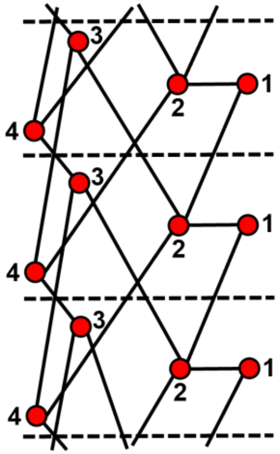	(4x**1**, 4x$\mathbf{2}^2$) **a-1**<br>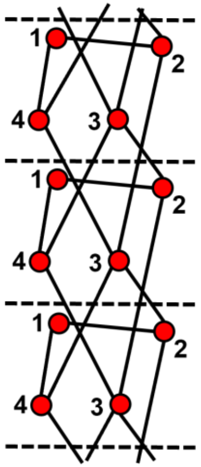	(4x**1**, 4x$\mathbf{2}^2$) **a-2**<br>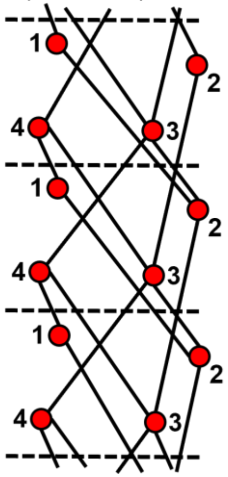	(4x**1**, 4x$\mathbf{2}^2$) **b-1**<br>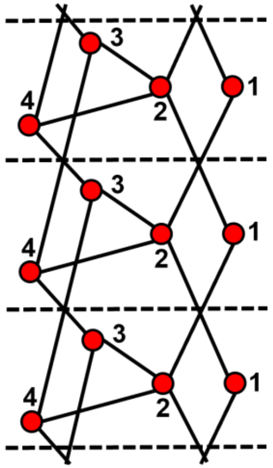

	(4x**1**, 4x**2**^{2}) **b-2** 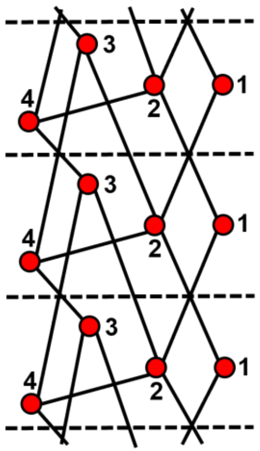	(4x**1**, 4x**2**^{2}) **b-3** 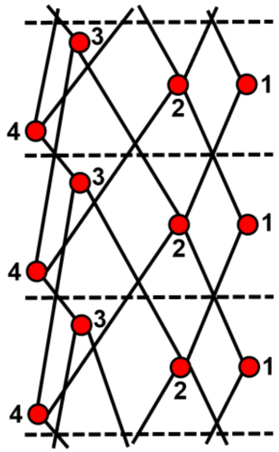	(2x**1**, 3x**2**, 2x**2**^{1}) **a** 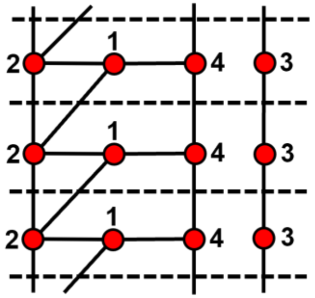	(2x**1**, 3x**2**, 2x**2**^{1}) **b** 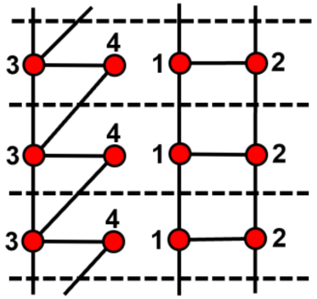
	(2x**1**, 3x**2**, 2x**2**^{2}) **a** 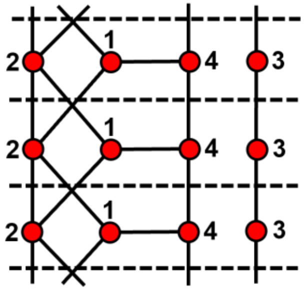	(2x**1**, 3x**2**, 2x**2**^{2}) **b** 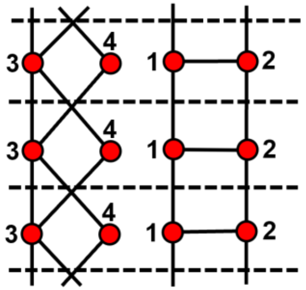	(2x**1**, 1x**2**, 4x**2**^{1}) **a** 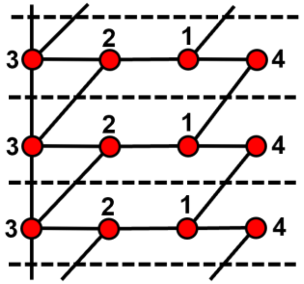	(2x**1**, 1x**2**, 4x**2**^{1}) **b** 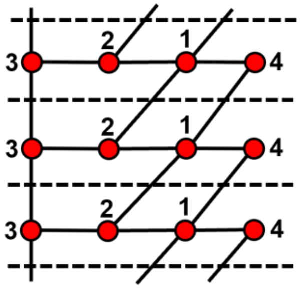

	(2x**1**, 1x**2**, 4x$\mathbf{2}^1$) **c-1** 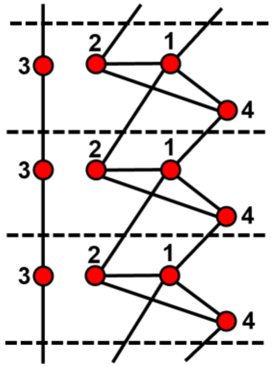	(2x**1**, 1x**2**, 4x$\mathbf{2}^1$) **c-2** 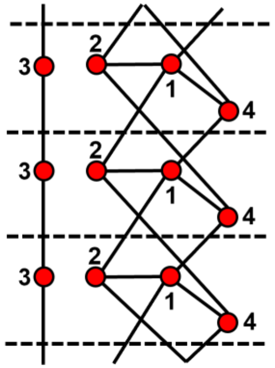	(2x**1**, 1x**2**, 2x$\mathbf{2}^1$, 2x$\mathbf{2}^2$) **a** 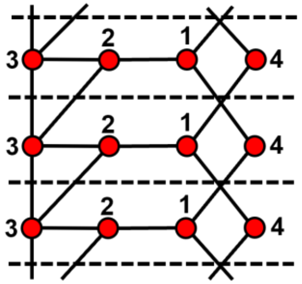	(2x**1**, 1x**2**, 2x$\mathbf{2}^1$, 2x$\mathbf{2}^2$) **b** 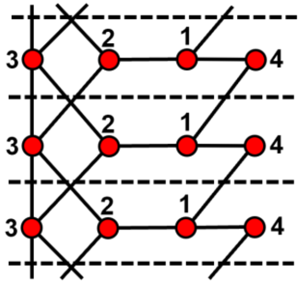
	(2x**1**, 1x**2**, 2x$\mathbf{2}^1$, 2x$\mathbf{2}^2$) **c** 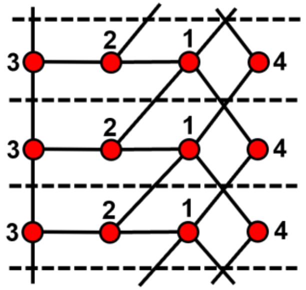	(2x**1**, 1x**2**, 2x$\mathbf{2}^1$, 2x$\mathbf{2}^2$) **d** 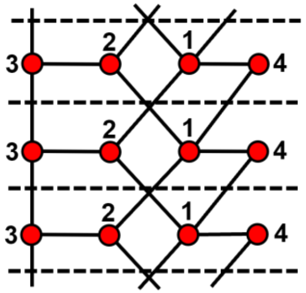	(2x**1**, 1x**2**, 2x$\mathbf{2}^1$, 2x$\mathbf{2}^2$) **e-1** 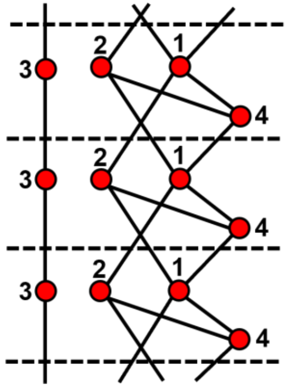	(2x**1**, 1x**2**, 2x$\mathbf{2}^1$, 2x$\mathbf{2}^2$) **e-2** 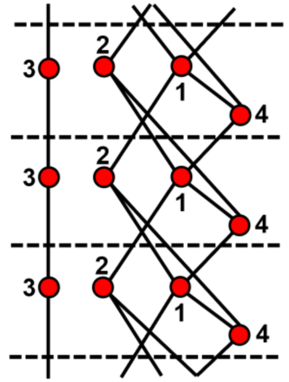

	(2x**1**, 1x**2**, 4x**2**^{2}) **a** 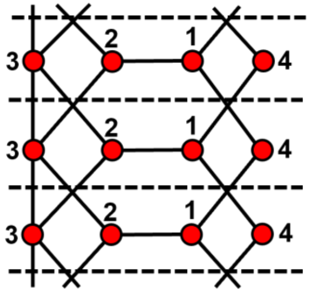	(2x**1**, 1x**2**, 4x**2**^{2}) **b** 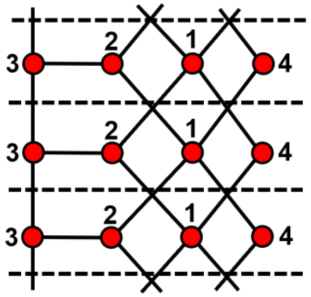	(2x**1**, 1x**2**, 4x**2**^{2}) **c-1** 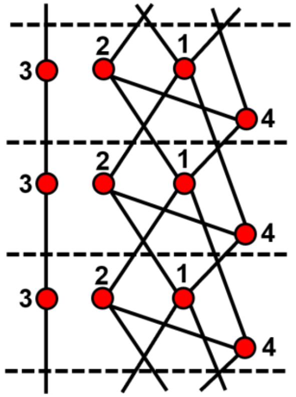	(2x**1**, 1x**2**, 4x**2**^{2}) **c-2** 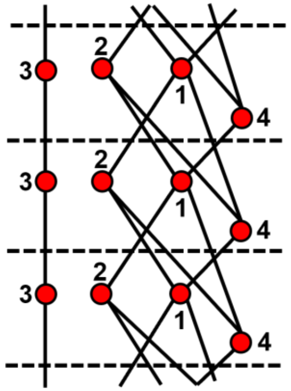
$^2V_1{}^3V_2{}^4V_2$	NG			
$^2V_1{}^3V_2{}^4V_3$	NG			
$^2V_1{}^3V_2{}^4V_4$	NG			
$^2V_1{}^3V_2{}^4V_5$	NG			
$^2V_1{}^3V_4{}^4V_1$	NG			
$^2V_1{}^3V_4{}^4V_2$	NG			
$^2V_1{}^3V_4{}^4V_3$	NG			
$^2V_1{}^3V_6{}^4V_1$	NG			
$^2V_2{}^3V_2{}^4V_1$	NG			
$^2V_2{}^3V_2{}^4V_2$	NG			
$^2V_2{}^3V_2{}^4V_3$	NG			
$^2V_2{}^3V_2{}^4V_4$	NG			
$^2V_2{}^3V_4{}^4V_1$	NG			
$^2V_2{}^3V_4{}^4V_2$	NG			

$^{2}V_{3}{}^{3}V_{2}{}^{4}V_{1}$	NG			
$^{2}V_{3}{}^{3}V_{2}{}^{4}V_{2}$	NG			
$^{2}V_{3}{}^{3}V_{2}{}^{4}V_{3}$	NG			
$^{2}V_{3}{}^{3}V_{4}{}^{4}V_{1}$	NG			
$^{2}V_{4}{}^{3}V_{2}{}^{4}V_{1}$	NG			
$^{2}V_{4}{}^{3}V_{2}{}^{4}V_{2}$	NG			
$^{2}V_{5}{}^{3}V_{2}{}^{4}V_{1}$	NG			
**Rank 4**				
$\boldsymbol{^{1}V_{r}{}^{2}V_{r}{}^{3}V_{r}{}^{4}V_{r}}$				
$^{1}V_{1}{}^{2}V_{1}{}^{3}V_{1}{}^{4}V_{1}$	(8x**1**, 1x**2**) **a** 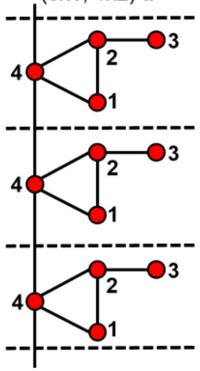	(8x**1**, 1x**2**) **b** 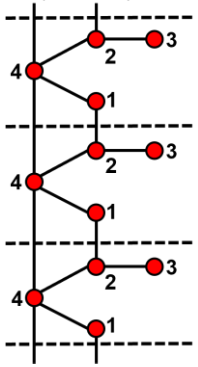	(8x**1**, 1x**2**) **c** 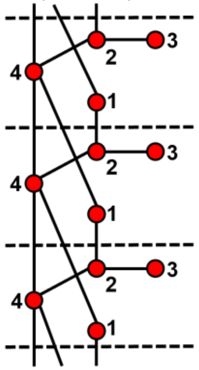	(8x**1**, 1x**2**) **d** 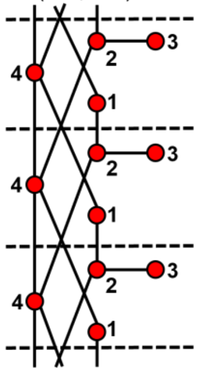

(6x**1**, 2x**2**) **a**

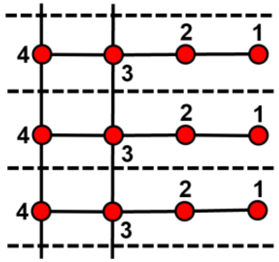

(6x**1**, 2x**2**) **b**

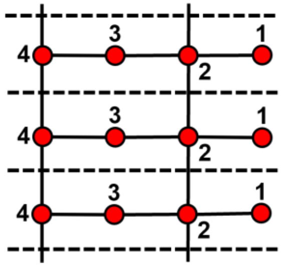

(6x**1**, 2x$\mathbf{2^1}$) **a**

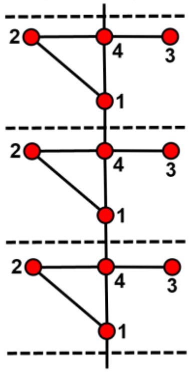

(6x**1**, 2x$\mathbf{2^1}$) **b**

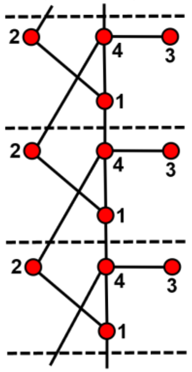

(6x**1**, 2x$\mathbf{2^1}$) **c**

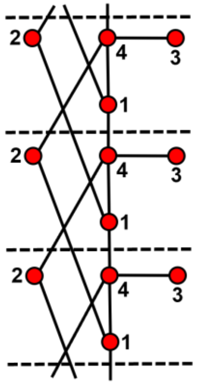

(6x**1**, 2x$\mathbf{2^2}$) **a**

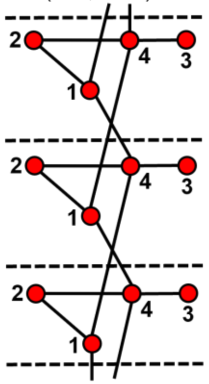

(6x**1**, 2x$\mathbf{2^2}$) **b**

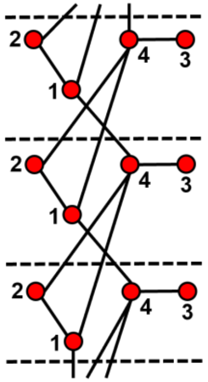

(6x**1**, 2x$\mathbf{2^2}$) **c**

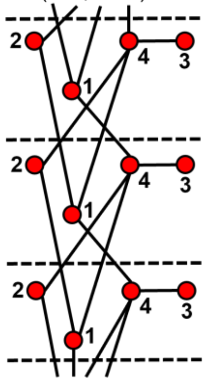

	(4x**1**, 3x**2**) 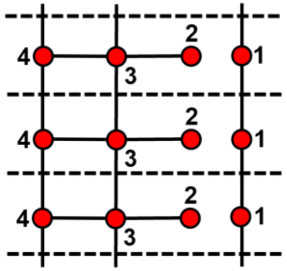	(4x**1**, 1x**2**, 2x**2**^{1}) **a** 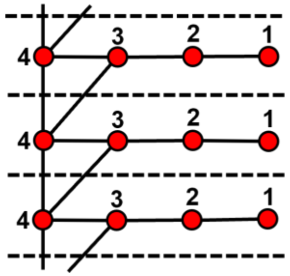	(4x**1**, 1x**2**, 2x**2**^{1}) **b** 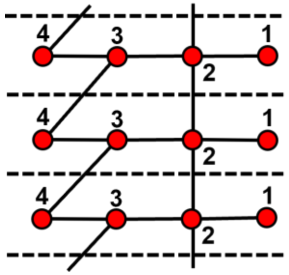	(4x**1**, 1x**2**, 2x**2**^{1}) **c** 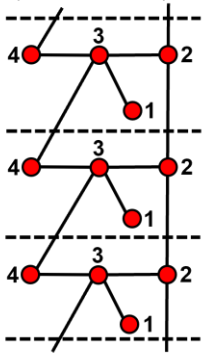
	(4x**1**, 1x**2**, 2x**2**^{2}) **a** 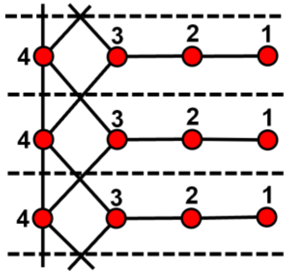	(4x**1**, 1x**2**, 2x**2**^{2}) **b** 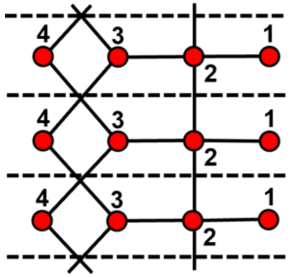	(4x**1**, 1x**2**, 2x**2**^{2}) **c** 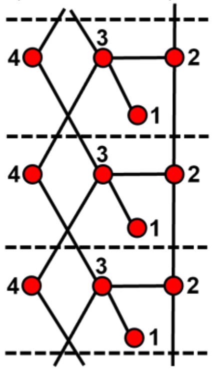	(2x**1**, 2x**2**, 2x**2**^{1}) **a** 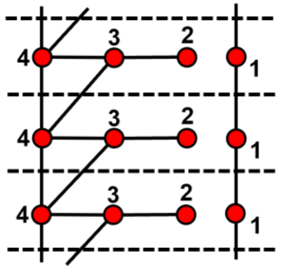
	(2x**1**, 2x**2**, 2x**2**^{1}) **b** 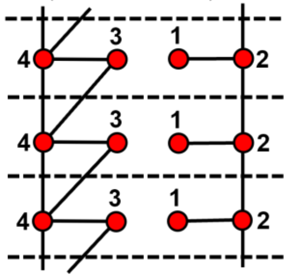	(2x**1**, 2x**2**, 2x**2**^{2}) **a** 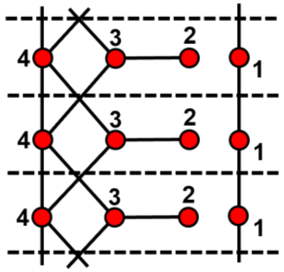	(2x**1**, 2x**2**, 2x**2**^{2}) **b** 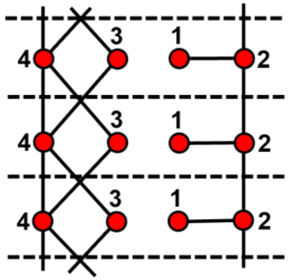	(2x**1**, 4x**2**^{1}) 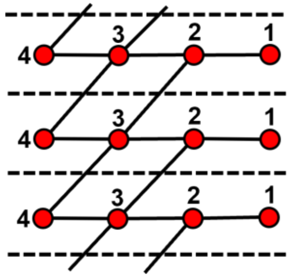

	(2x**1**, 2x$\mathbf{2}^1$, 2x$\mathbf{2}^2$) **a** 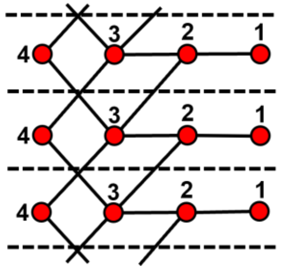	(2x**1**, 2x$\mathbf{2}^1$, 2x$\mathbf{2}^2$) **b** 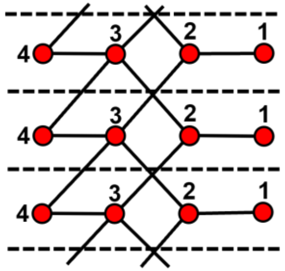	(2x**1**, 4x$\mathbf{2}^2$) 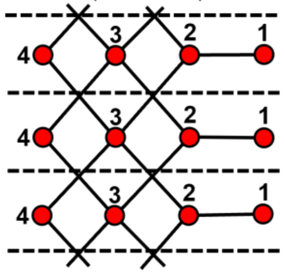	
$^{1}V_{1}{}^{2}V_{1}{}^{3}V_{1}{}^{4}V_{2}$	(12x**1**, 1x**2**) **a** 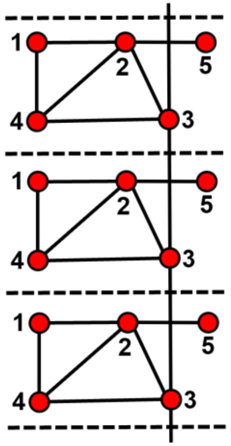	(12x**1**, 1x**2**) **b** 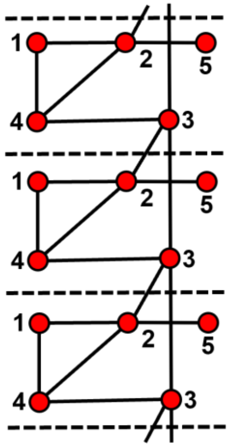	(12x**1**, 1x**2**) **c** 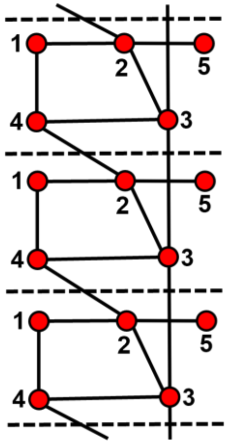	(12x**1**, 1x**2**) **d** 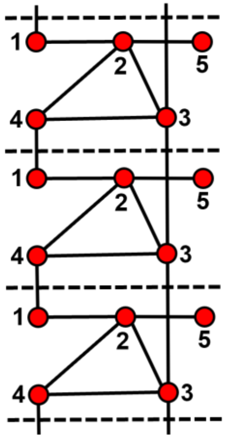

	(12x**1**, 1x**2**) **e** 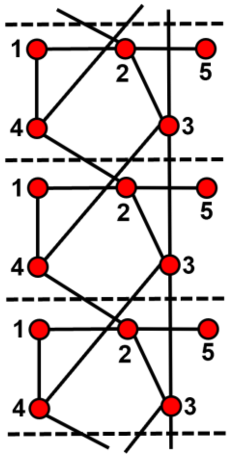	(12x**1**, 1x**2**) **f** 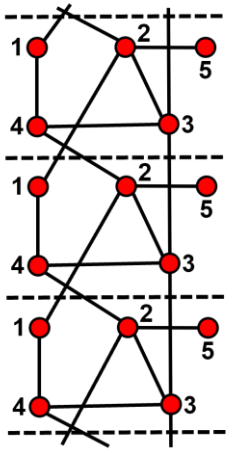	(12x**1**, 1x**2**) **g** 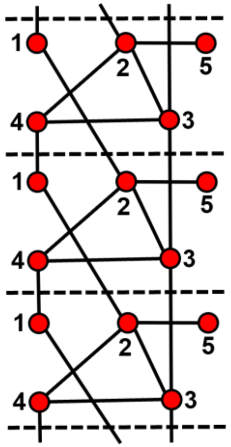	(12x**1**, 1x**2**) **h** 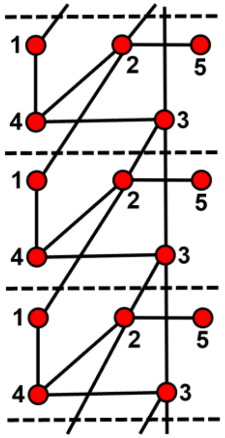
	(12x**1**, 1x**2**) **i** 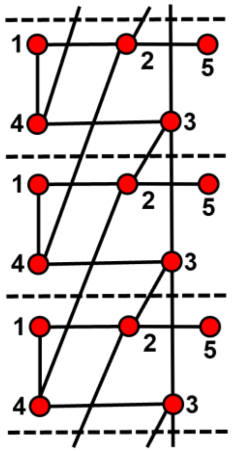	(12x**1**, 1x**2**) **j** 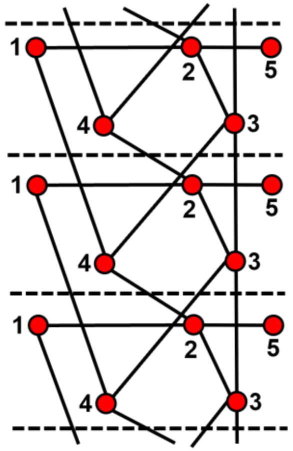	(12x**1**, 1x**2**) **k** 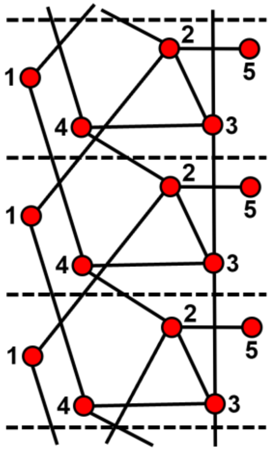	(12x**1**, 1x**2**) **l** 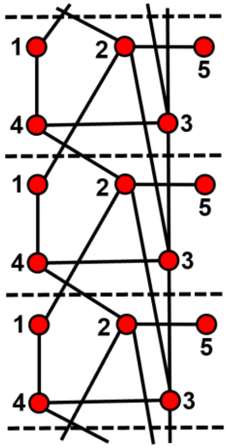

	(12x**1**, 1x**2**) **m** 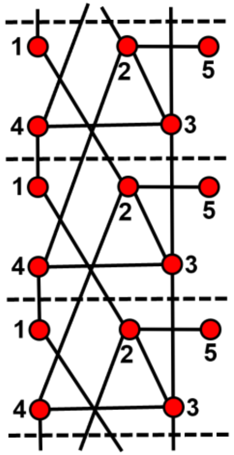	(12x**1**, 1x**2**) **n** 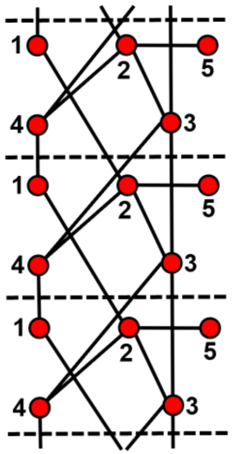	(12x**1**, 1x**2**) **o** 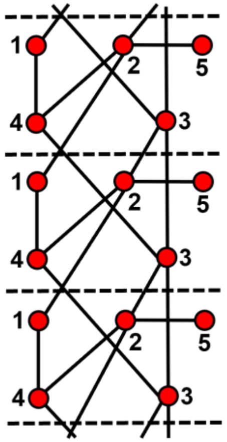	(12x**1**, 1x**2**) **p** 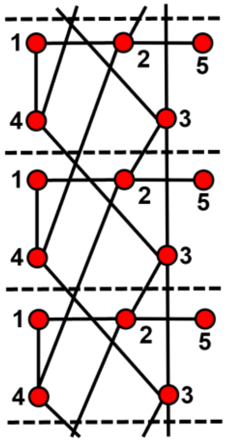
	(12x**1**, 1x**2**) **q** 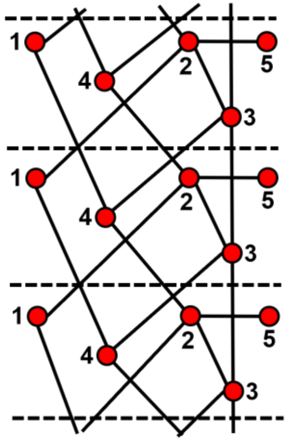	(12x**1**, 1x**2**) **r** 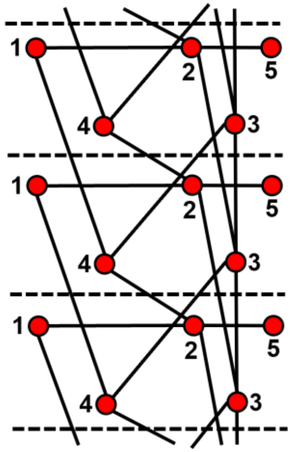	(12x**1**, 1x**2**) **s** 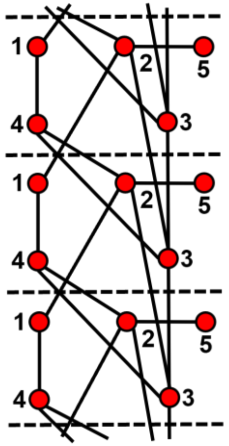	(12x**1**, 1x**2**) **t** 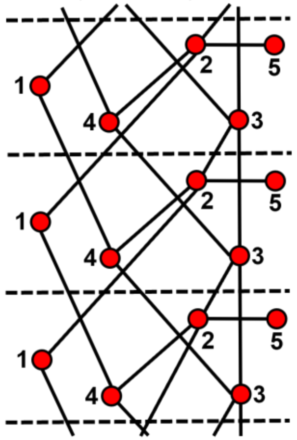

	(12x**1**, 1x**2**) **u** 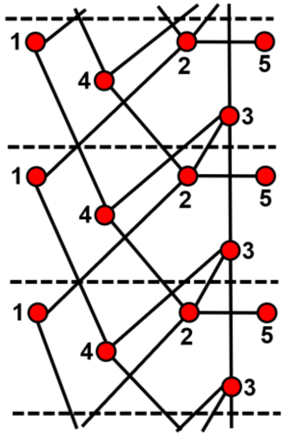	(12x**1**, 1x**2**) **v** 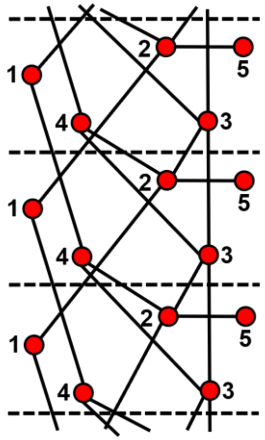	(12x**1**, 1x**2**) **w** 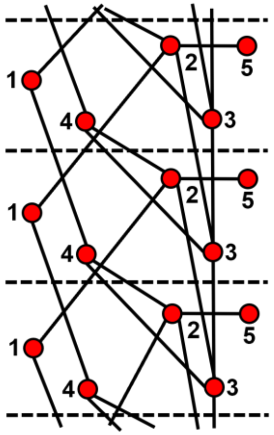	(10x**1**, 2x**2**) **a-1** 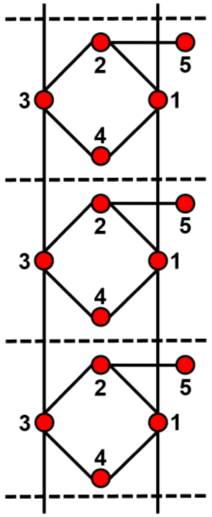
	(10x**1**, 2x**2**) **a-2** 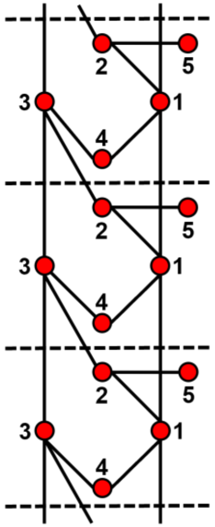	(10x**1**, 2x**2**) **a-3** 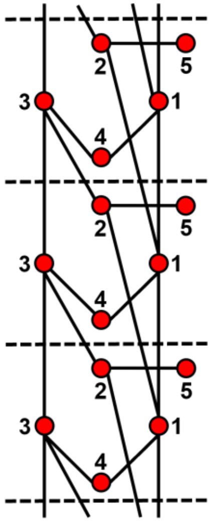	(10x**1**, 2x**2**) **a-4** 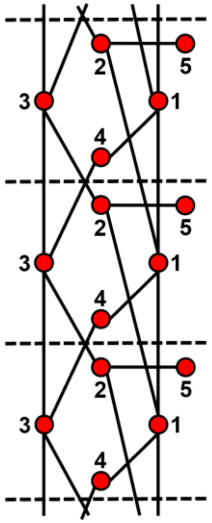	(10x**1**, 2x**2**) **a-5** 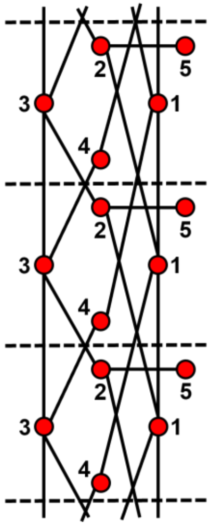

	(10x**1**, 2x**2**) **b-1**<br>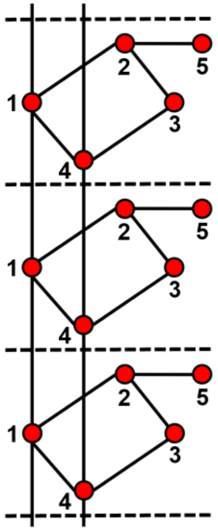 	(10x**1**, 2x**2**) **b-2**<br>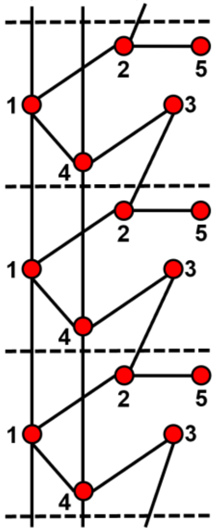 	(10x**1**, 2x**2**) **b-3**<br>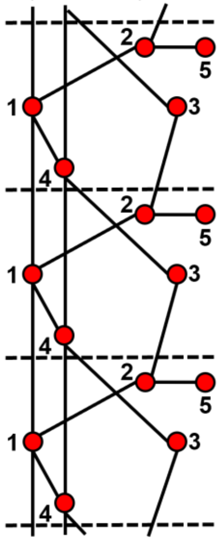 	(10x**1**, 2x**2**) **b-4**<br>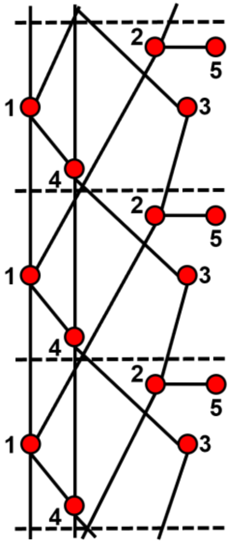

	(10x**1**, 2x**2**) **b-5** 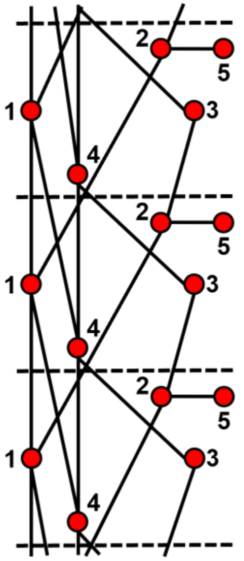	(10x**1**, 2x**2**) **c-1** 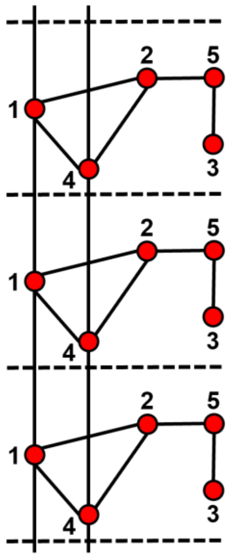	(10x**1**, 2x**2**) **c-2** 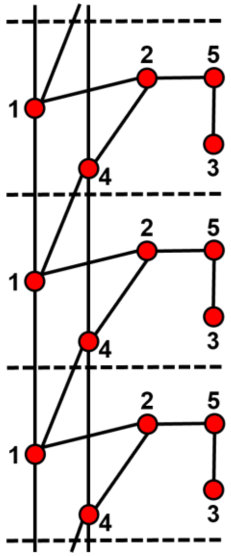	(10x**1**, 2x**2**) **c-3** 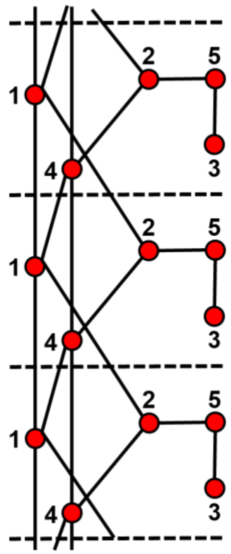

	(10x**1**, 2x**2**) **c-4**<br>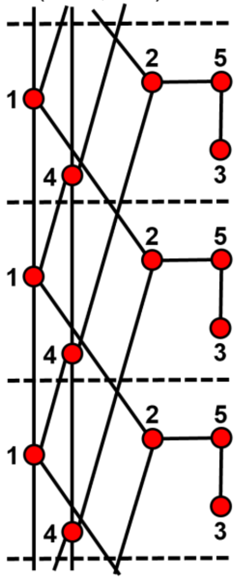 	(10x**1**, 2x**2**) **d-1**<br>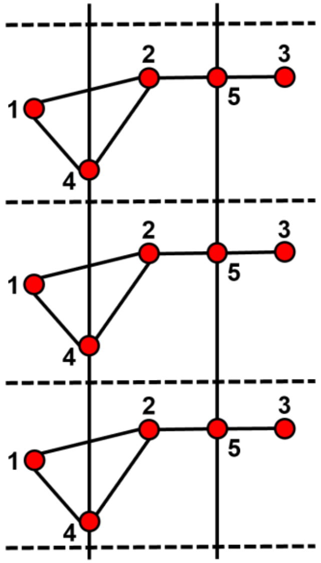 	(10x**1**, 2x**2**) **d-2**<br>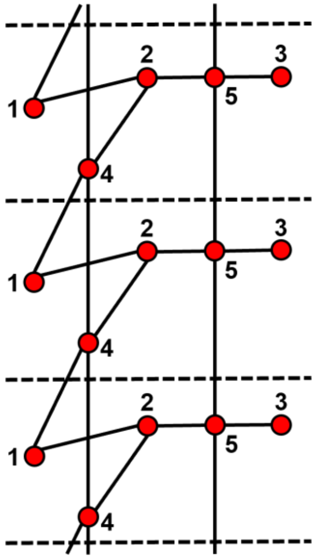 	(10x**1**, 2x**2**) **d-3**<br>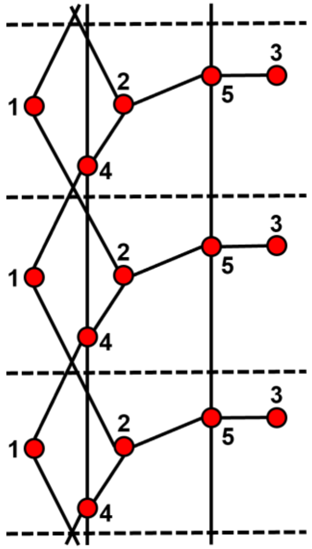

	(10x**1**, 2x**2**) **d-4** 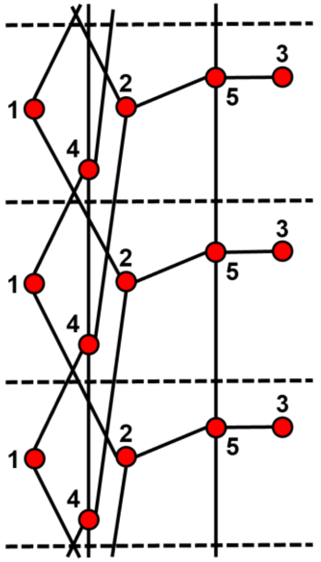	(10x**1**, 2x**2**) **e-1** 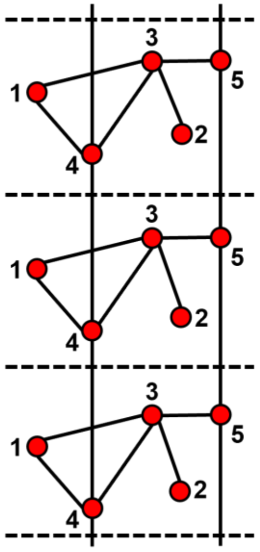	(10x**1**, 2x**2**) **e-2** 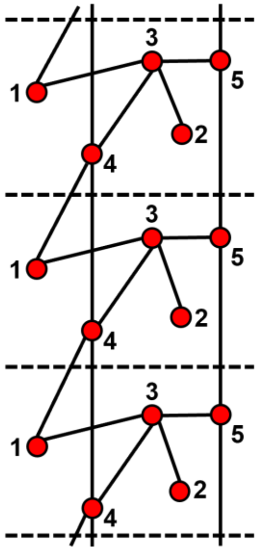	(10x**1**, 2x**2**) **e-3** 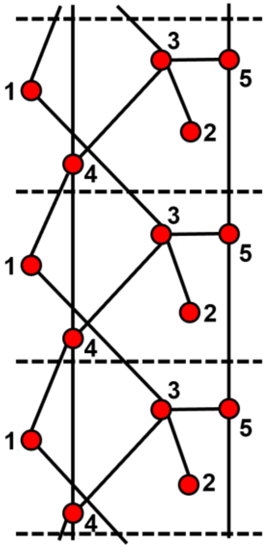

	(10x**1**, 2x**2**) **e-4** 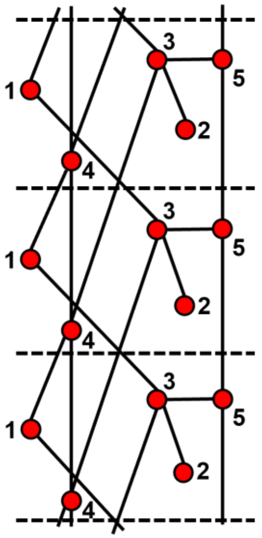	(10x**1**, 2x**2**^{1}) **a-1** 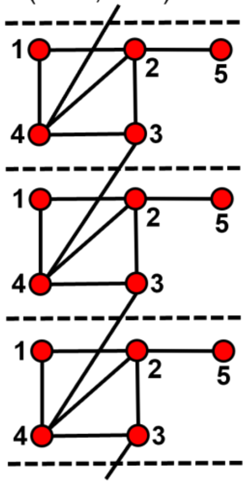	(10x**1**, 2x**2**^{1}) **a-2** 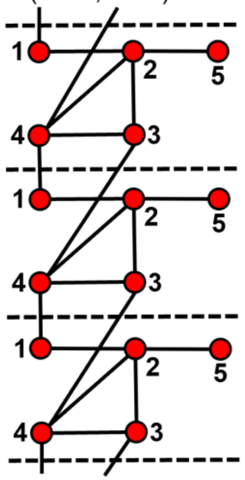	(10x**1**, 2x**2**^{1}) **a-3** 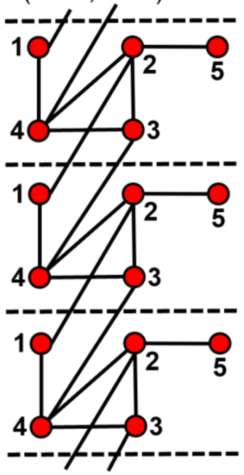
	(10x**1**, 2x**2**^{1}) **a-4** 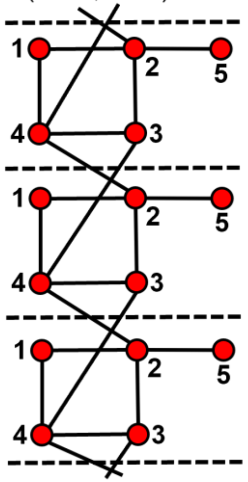	(10x**1**, 2x**2**^{1}) **a-5** 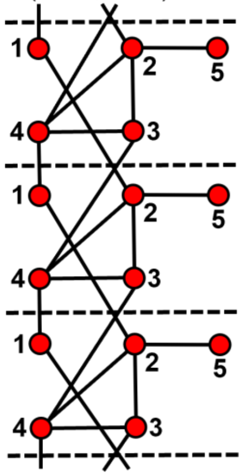	(10x**1**, 2x**2**^{1}) **a-6** 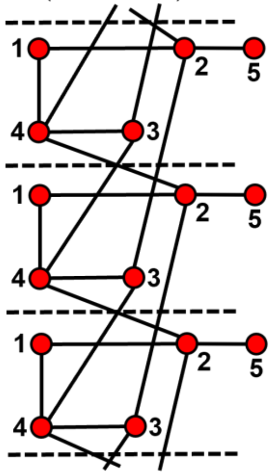	(10x**1**, 2x**2**^{1}) **a-7** 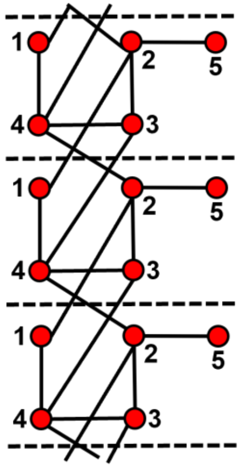

	(10x**1**, 2x**2**^{1}) **a-8**<br>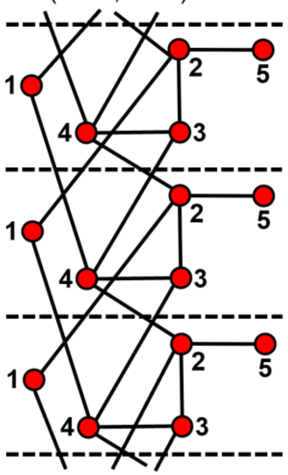	(10x**1**, 2x**2**^{1}) **a-9**<br>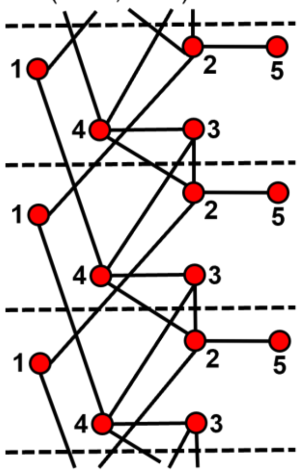	(10x**1**, 2x**2**^{1}) **b-1**<br>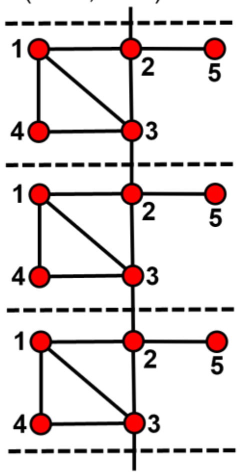	(10x**1**, 2x**2**^{1}) **b-2**<br>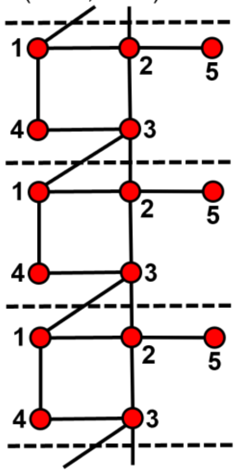
	(10x**1**, 2x**2**^{1}) **b-3**<br>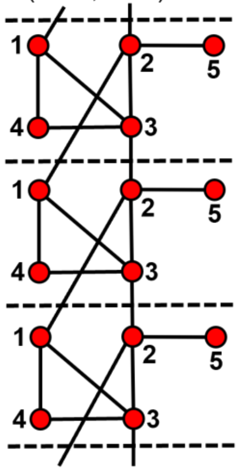	(10x**1**, 2x**2**^{1}) **b-4**<br>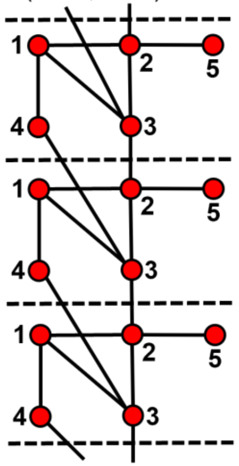	(10x**1**, 2x**2**^{1}) **b-5**<br>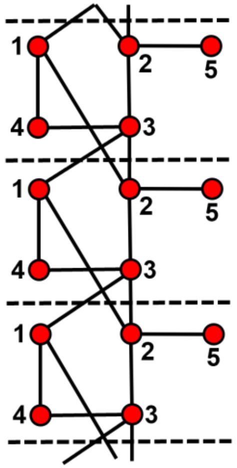	(10x**1**, 2x**2**^{1}) **b-6**<br>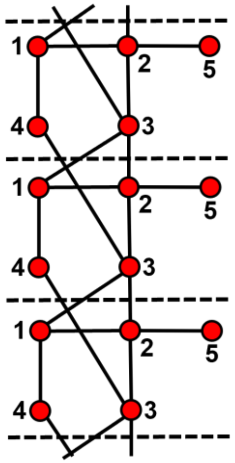

	(10x**1**, 2x**2**^{1}) **b-7**<br>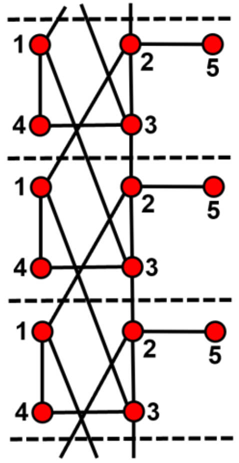	(10x**1**, 2x**2**^{1}) **b-8**<br>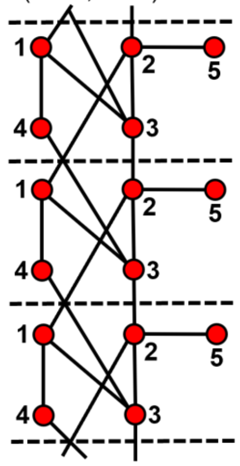	(10x**1**, 2x**2**^{1}) **b-9**<br>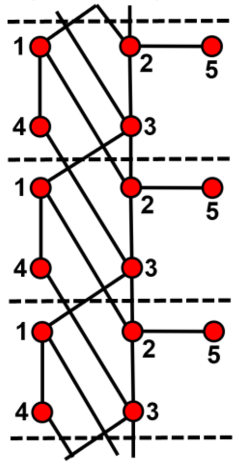	(10x**1**, 2x**2**^{1}) **b-10**<br>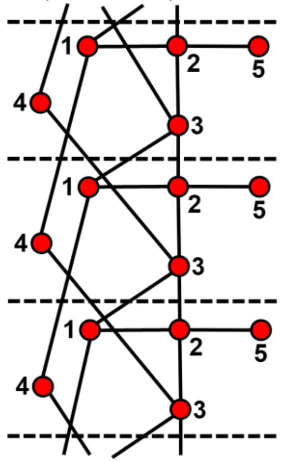
	(10x**1**, 2x**2**^{1}) **b-11**<br>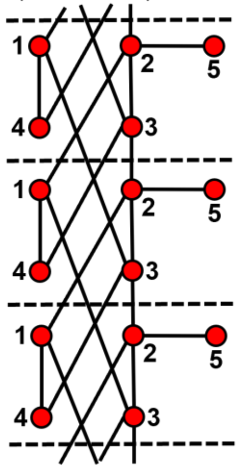	(10x**1**, 2x**2**^{1}) **b-12**<br>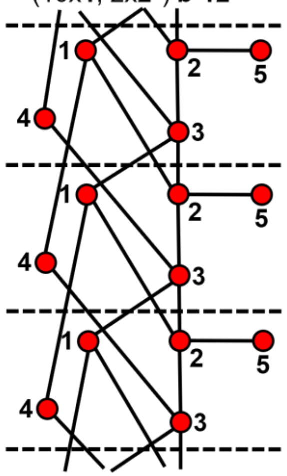	(10x**1**, 2x**2**^{1}) **c-1**<br>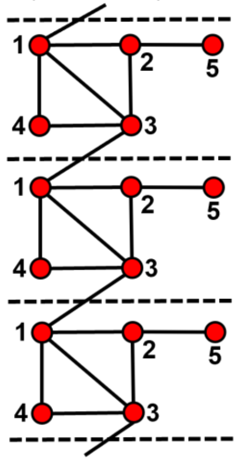	(10x**1**, 2x**2**^{1}) **c-2**<br>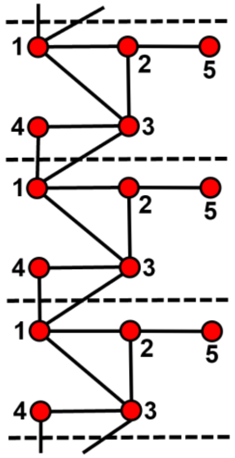

	(10x**1**, 2x**2**^{1}) **c-3** 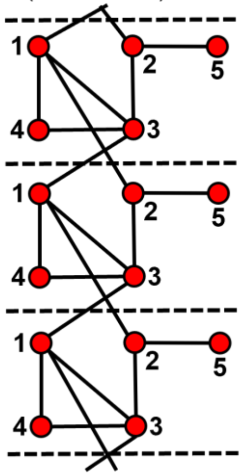	(10x**1**, 2x**2**^{1}) **c-4** 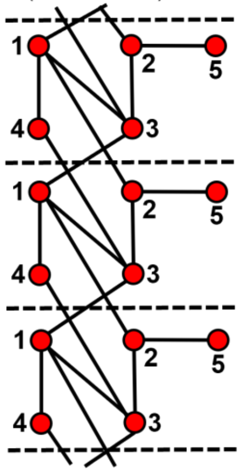	(10x**1**, 2x**2**^{1}) **c-5** 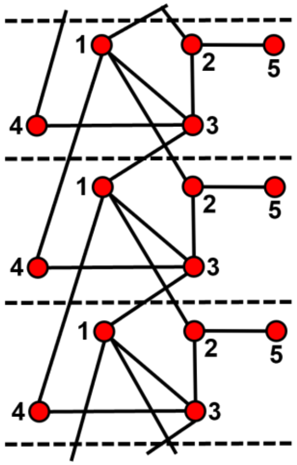	(10x**1**, 2x**2**^{1}) **c-6** 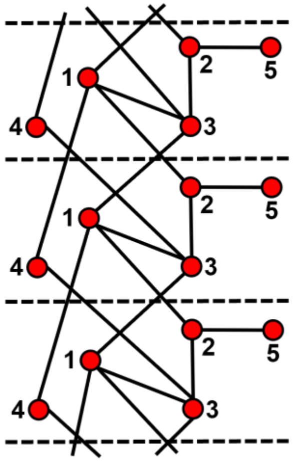
	(10x**1**, 2x**2**^{1}) **c-7** 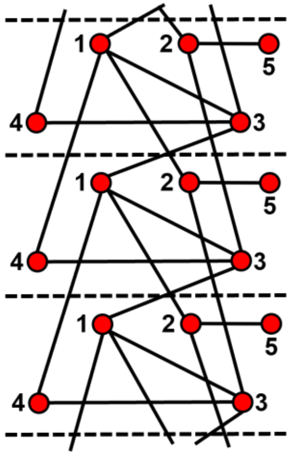	(10x**1**, 2x**2**^{1}) **c-8** 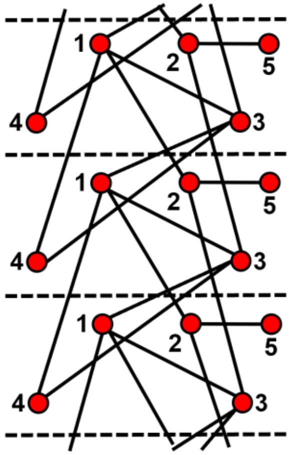	(10x**1**, 2x**2**^{2}) **a-1** 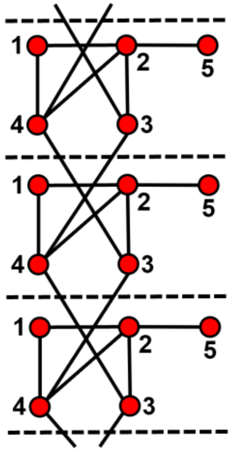	(10x**1**, 2x**2**^{2}) **a-2** 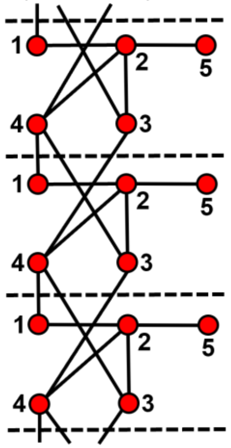

	(10x**1**, 2x**2**^{2}) **a-3** 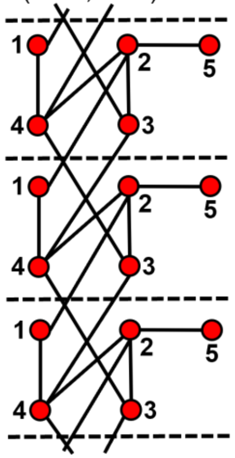	(10x**1**, 2x**2**^{2}) **a-4** 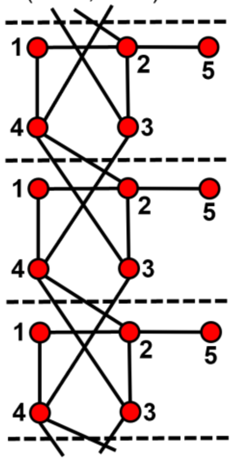	(10x**1**, 2x**2**^{2}) **a-5** 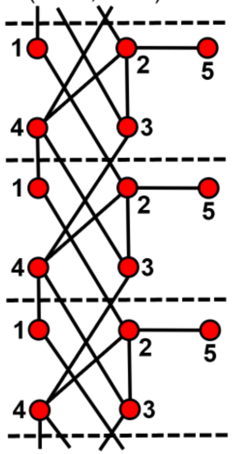	(10x**1**, 2x**2**^{2}) **a-6** 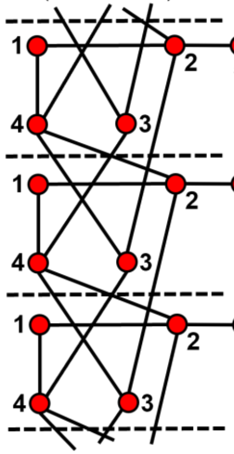
	(10x**1**, 2x**2**^{2}) **a-7** 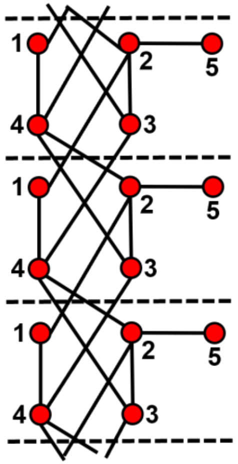	(10x**1**, 2x**2**^{2}) **a-8** 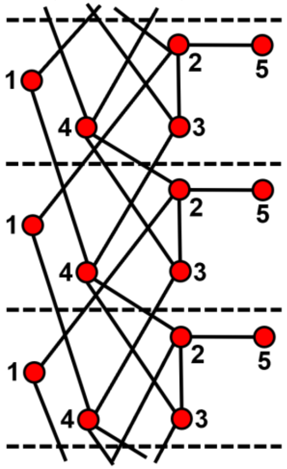	(10x**1**, 2x**2**^{2}) **a-9** 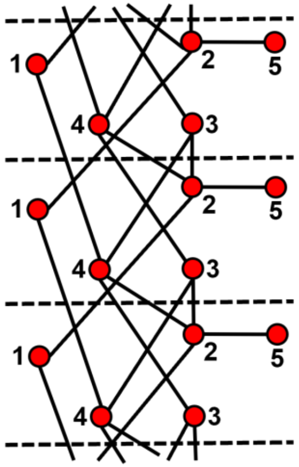	(10x**1**, 2x**2**^{2}) **b-1** 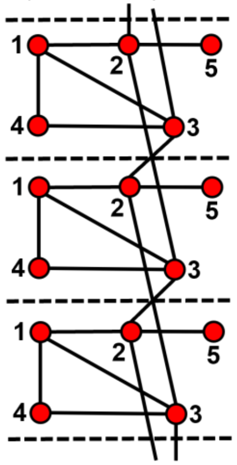

	(10x**1**, 2x**2**^{2}) **b-2**<br>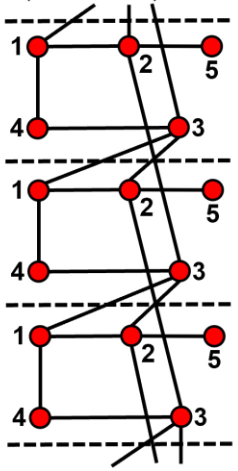	(10x**1**, 2x**2**^{2}) **b-3**<br>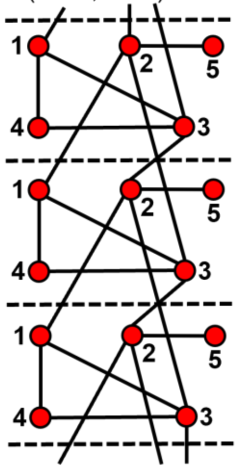	(10x**1**, 2x**2**^{2}) **b-4**<br>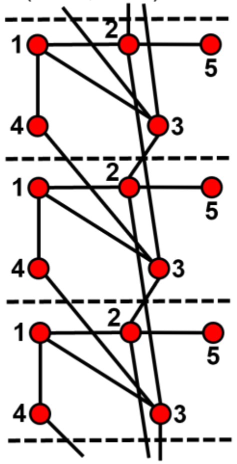	(10x**1**, 2x**2**^{2}) **b-5**<br>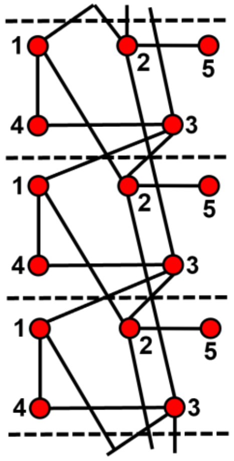
	(10x**1**, 2x**2**^{2}) **b-6**<br>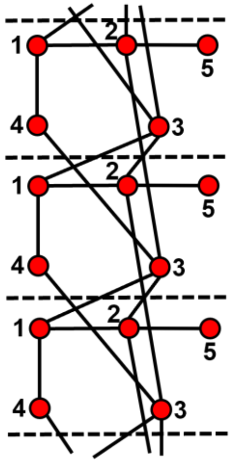	(10x**1**, 2x**2**^{2}) **b-7**<br>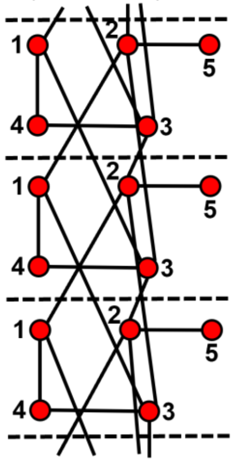	(10x**1**, 2x**2**^{2}) **b-8**<br>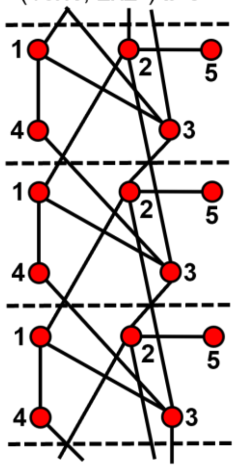	(10x**1**, 2x**2**^{2}) **b-9**<br>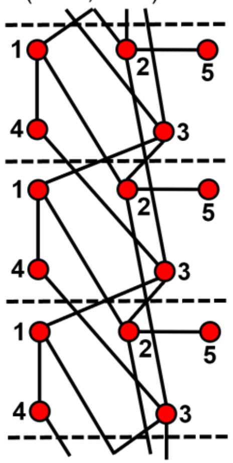

	(10x**1**, 2x**2**^{2}) **b-10** 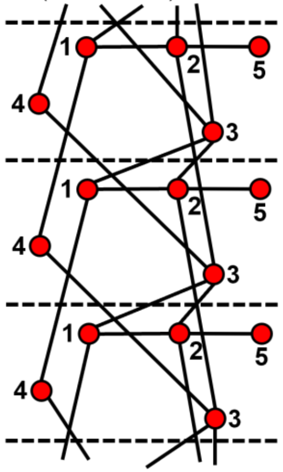	(10x**1**, 2x**2**^{2}) **b-11** 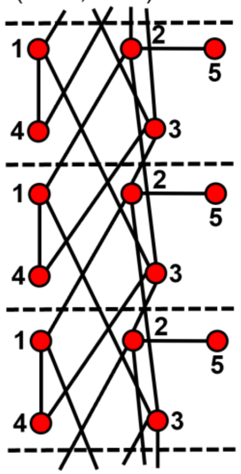	(10x**1**, 2x**2**^{2}) **b-12** 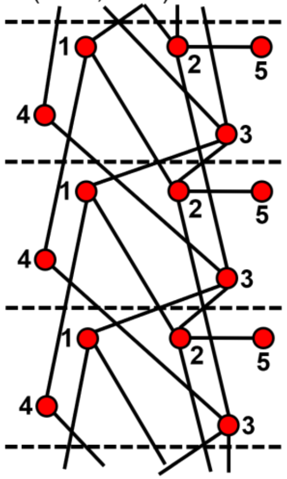	(10x**1**, 2x**2**^{2}) **c-1** 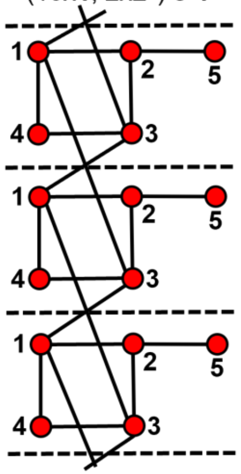
	(10x**1**, 2x**2**^{2}) **c-2** 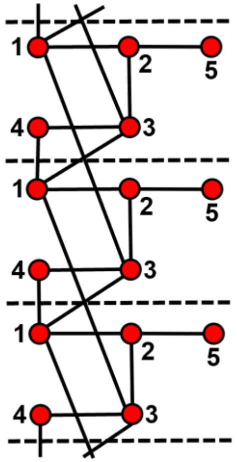	(10x**1**, 2x**2**^{2}) **c-3** 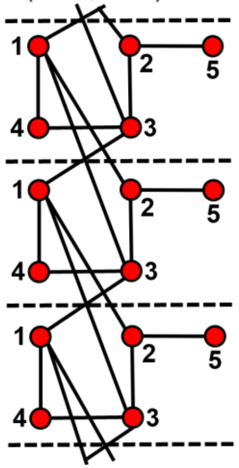	(10x**1**, 2x**2**^{2}) **c-4** 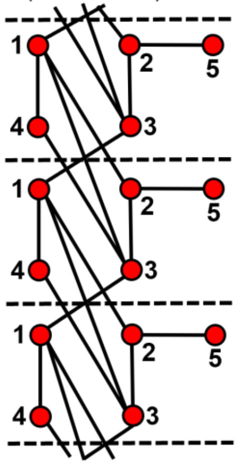	(10x**1**, 2x**2**^{2}) **c-5** 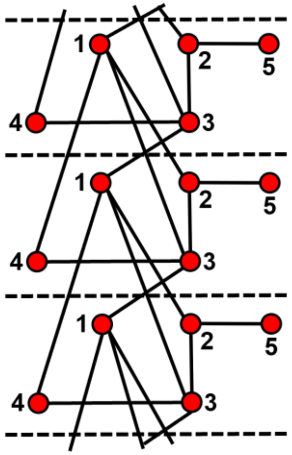

	(10x**1**, 2x**2**^{2}) **c-6** 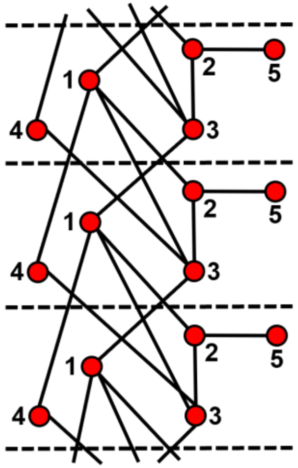	(10x**1**, 2x**2**^{2}) **c-7** 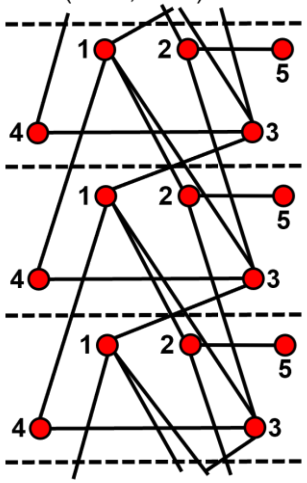	(10x**1**, 2x**2**^{2}) **c-8** 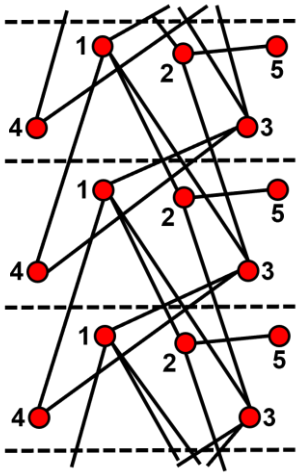	(8x**1**, 3x**2**) **a** 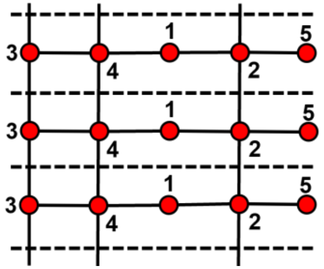
	(8x**1**, 3x**2**) **b** 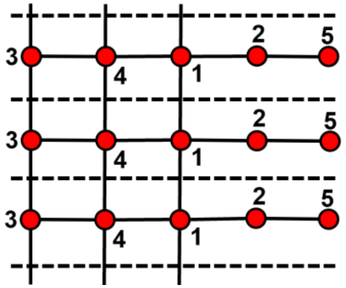	(8x**1**, 3x**2**) **c** 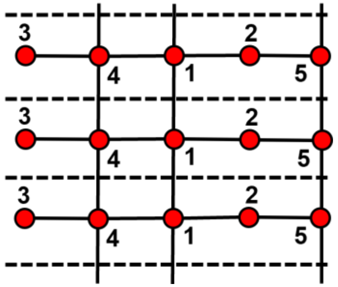	(8x**1**, 3x**2**) **d-1** 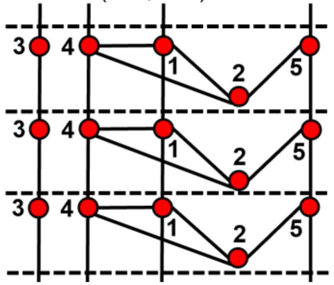	(8x**1**, 3x**2**) **d-2** 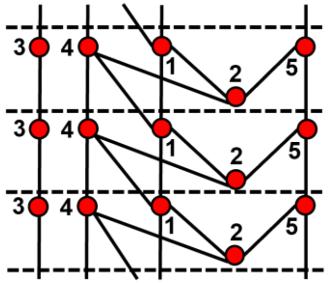

	(8x**1**, 3x**2**) **d-3** 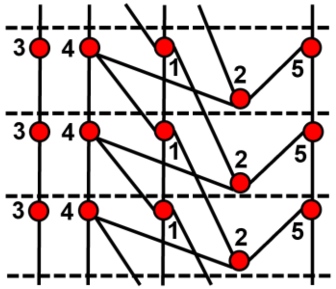	(8x**1**, 3x**2**) **d-4** 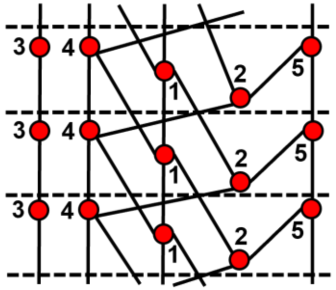	(8x**1**, 3x**2**) **e-1** 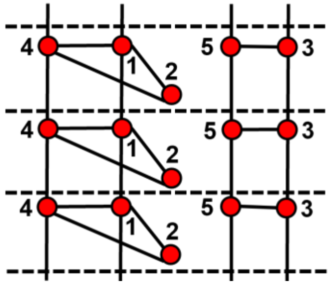	(8x**1**, 3x**2**) **e-2** 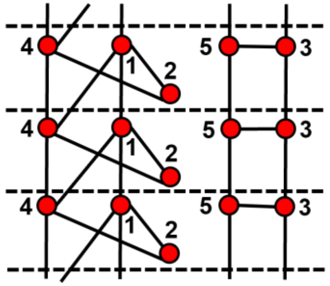
	(8x**1**, 3x**2**) **e-3** 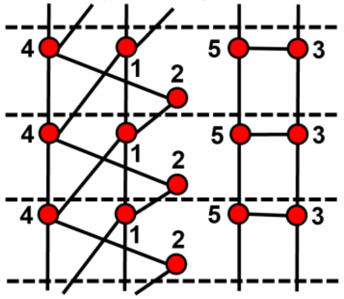	(8x**1**, 3x**2**) **e-4** 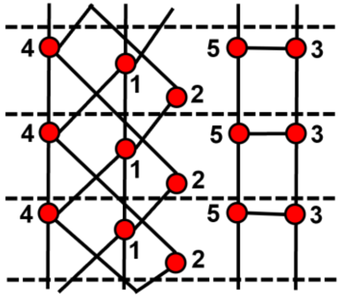	(8x**1**, 1x**2**, 2x**2**^{1}) **a-1** 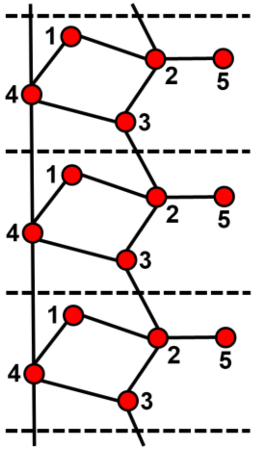	(8x**1**, 1x**2**, 2x**2**^{1}) **a-2** 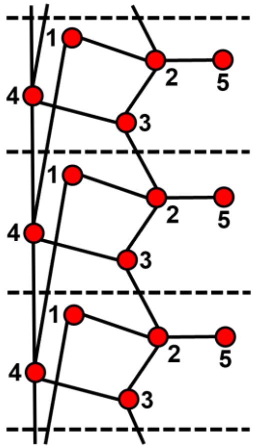

(8x**1**, 1x**2**, 2x$\mathbf{2}^{1}$) **a-3**

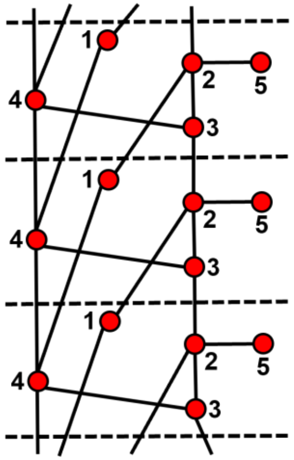

(8x**1**, 1x**2**, 2x$\mathbf{2}^{1}$) **a-4**

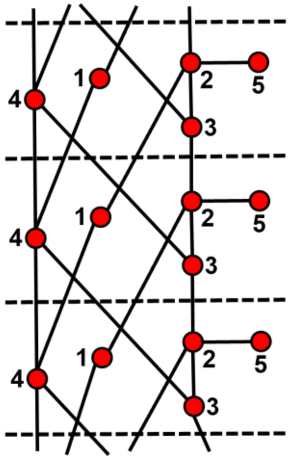

(8x**1**, 1x**2**, 2x$\mathbf{2}^{1}$) **b-1**

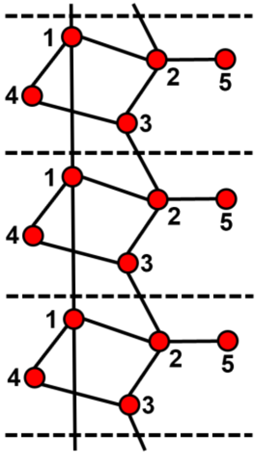

(8x**1**, 1x**2**, 2x$\mathbf{2}^{1}$) **b-2**

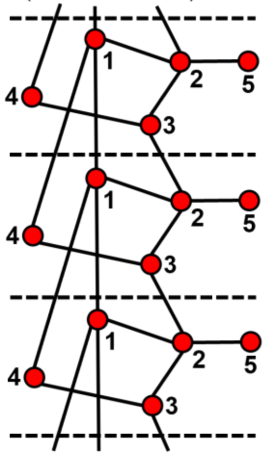

(8x**1**, 1x**2**, 2x$\mathbf{2}^{1}$) **b-3**

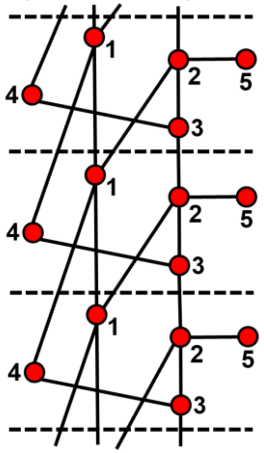

(8x**1**, 1x**2**, 2x$\mathbf{2}^{1}$) **b-4**

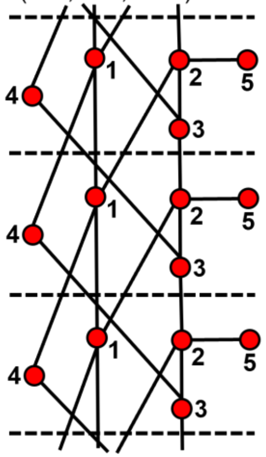

(8x**1**, 1x**2**, 2x$\mathbf{2}^{1}$) **c-1**

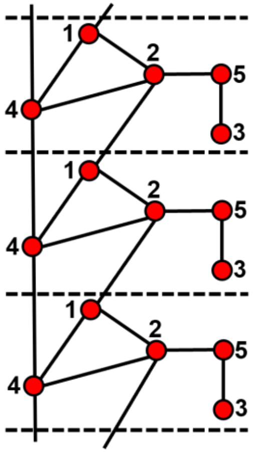

(8x**1**, 1x**2**, 2x$\mathbf{2}^{1}$) **c-2**

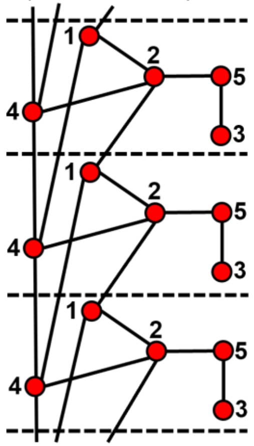

	(8x**1**, 1x**2**, 2x$\mathbf{2}^1$) **c-3** 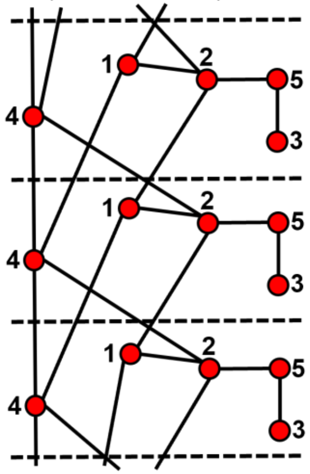	(8x**1**, 1x**2**, 2x$\mathbf{2}^1$) **d-1** 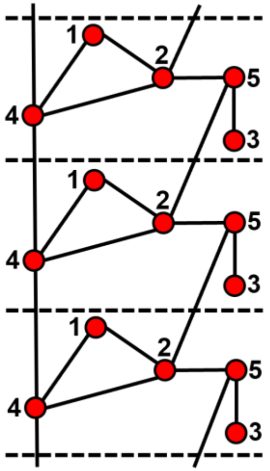	(8x**1**, 1x**2**, 2x$\mathbf{2}^1$) **d-2** 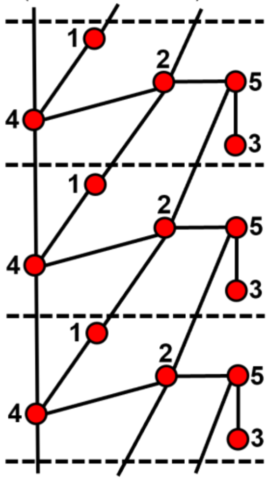	(8x**1**, 1x**2**, 2x$\mathbf{2}^1$) **d-3** 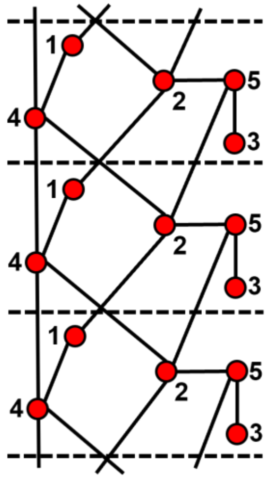
	(8x**1**, 1x**2**, 2x$\mathbf{2}^1$) **d-4** 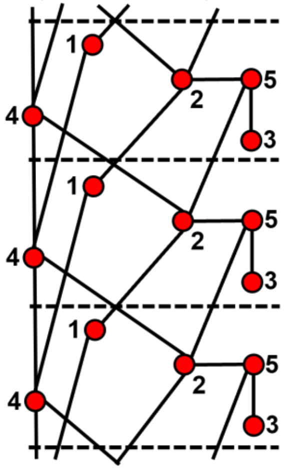	(8x**1**, 1x**2**, 2x$\mathbf{2}^1$) **e-1** 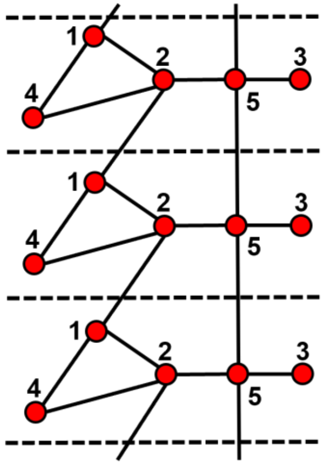	(8x**1**, 1x**2**, 2x$\mathbf{2}^1$) **e-2** 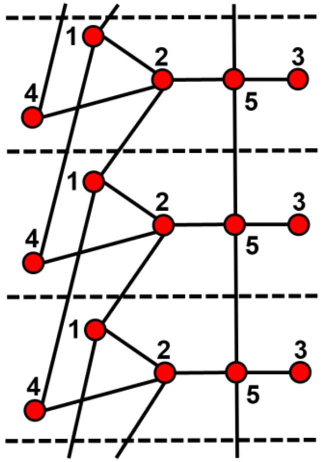	(8x**1**, 1x**2**, 2x$\mathbf{2}^1$) **e-3** 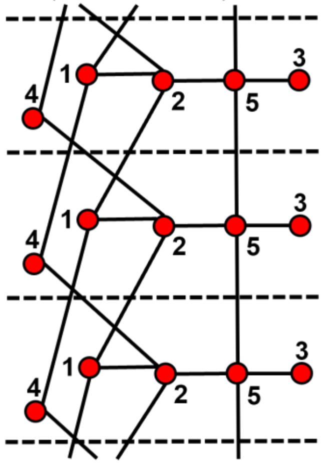

	(8x**1**, 1x**2**, 2x**2**^{1}) **f-1**<br>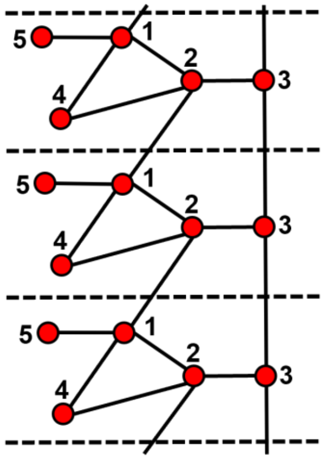	(8x**1**, 1x**2**, 2x**2**^{1}) **f-2**<br>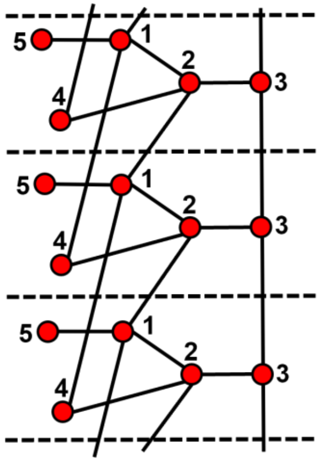	(8x**1**, 1x**2**, 2x**2**^{1}) **f-3**<br>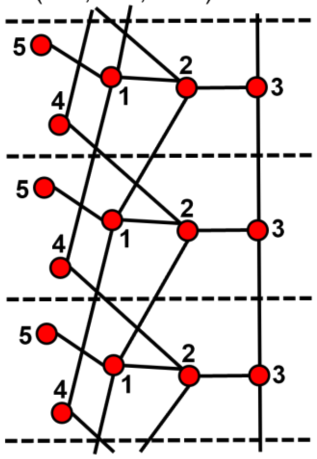	(8x**1**, 1x**2**, 2x**2**^{1}) **g-1**<br>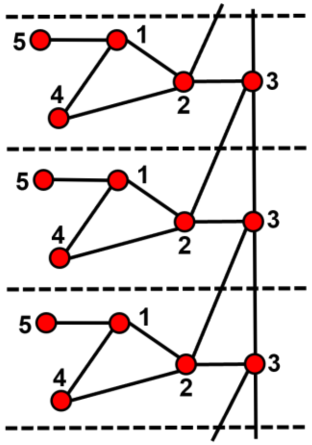
	(8x**1**, 1x**2**, 2x**2**^{1}) **g-2**<br>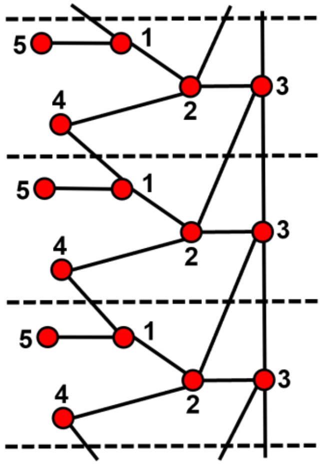	(8x**1**, 1x**2**, 2x**2**^{1}) **g-3**<br>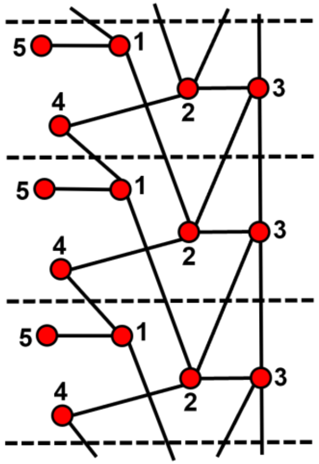	(8x**1**, 1x**2**, 2x**2**^{1}) **g-4**<br>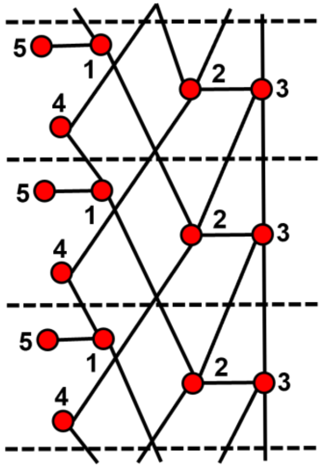	(8x**1**, 1x**2**, 2x**2**^{1}) **h-1**<br>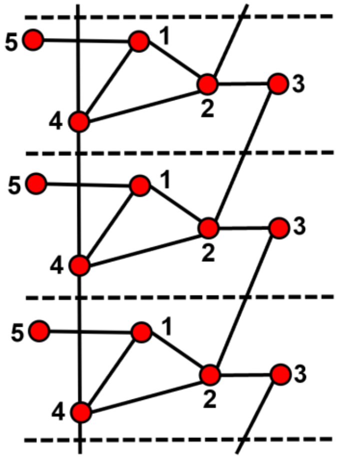

	(8x**1**, 1x**2**, 2x**2**^{1}) **h-2** 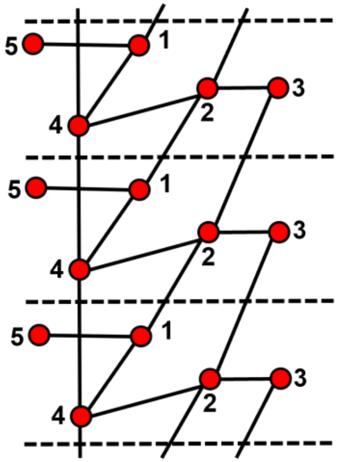	(8x**1**, 1x**2**, 2x**2**^{1}) **h-3** 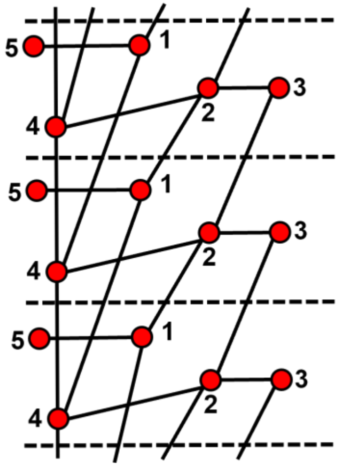	(8x**1**, 1x**2**, 2x**2**^{1}) **h-4** 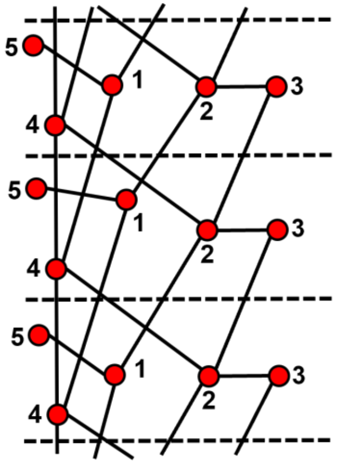	(8x**1**, 1x**2**, 2x**2**^{2}) **a-1** 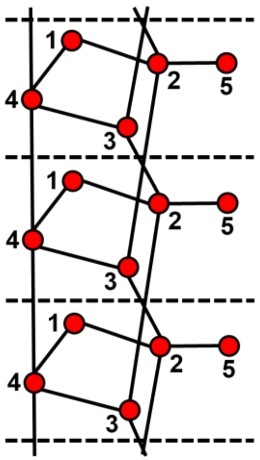
	(8x**1**, 1x**2**, 2x**2**^{2}) **a-2** 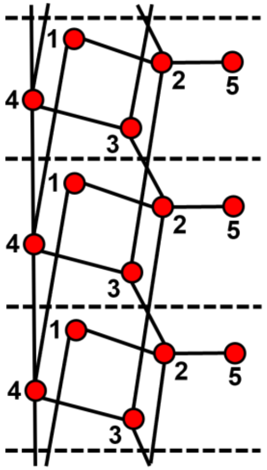	(8x**1**, 1x**2**, 2x**2**^{2}) **a-3** 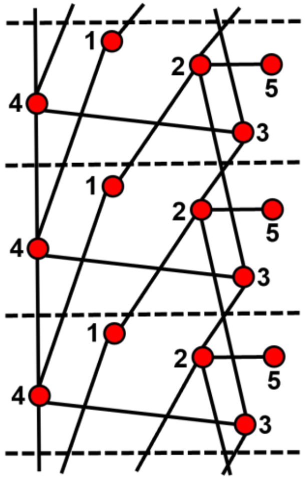	(8x**1**, 1x**2**, 2x**2**^{2}) **a-4** 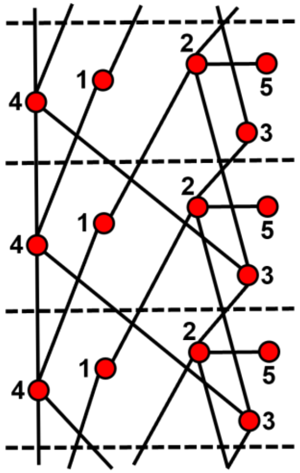	(8x**1**, 1x**2**, 2x**2**^{2}) **b-1** 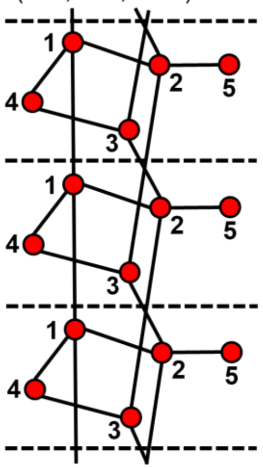

	(8x**1**, 1x**2**, 2x**2**^{2}) **b-2** 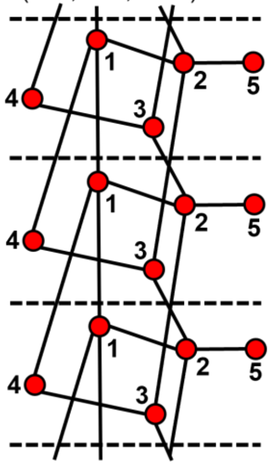	(8x**1**, 1x**2**, 2x**2**^{2}) **b-3** 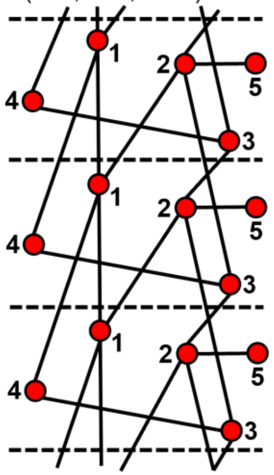	(8x**1**, 1x**2**, 2x**2**^{2}) **b-4** 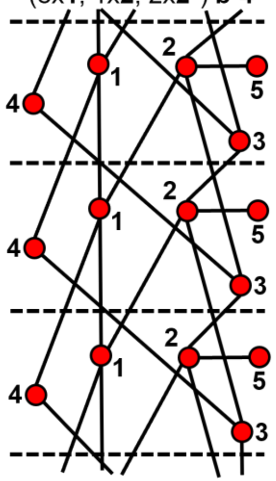	(8x**1**, 1x**2**, 2x**2**^{2}) **c-1** 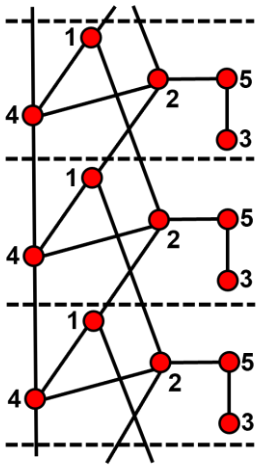
	(8x**1**, 1x**2**, 2x**2**^{2}) **c-2** 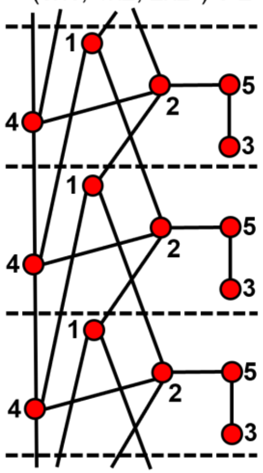	(8x**1**, 1x**2**, 2x**2**^{2}) **c-3** 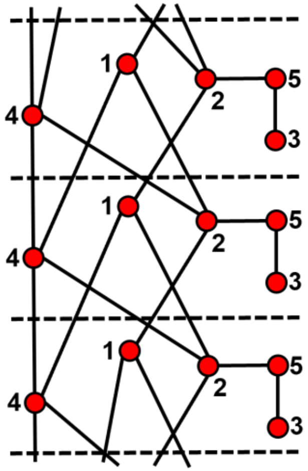	(8x**1**, 1x**2**, 2x**2**^{2}) **d-1** 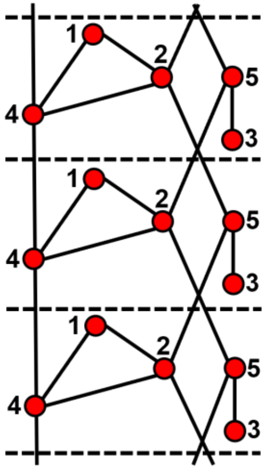	(8x**1**, 1x**2**, 2x**2**^{2}) **d-2** 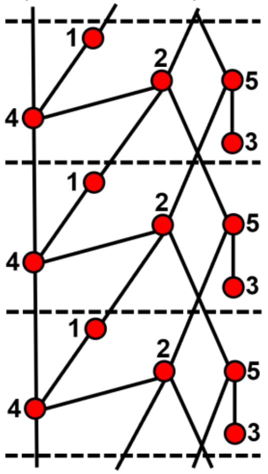

	(8x**1**, 1x**2**, 2x**2**^{2}) **d-3** 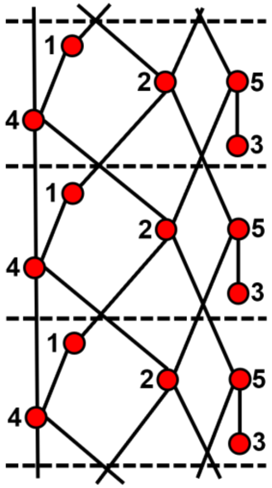	(8x**1**, 1x**2**, 2x**2**^{2}) **d-4** 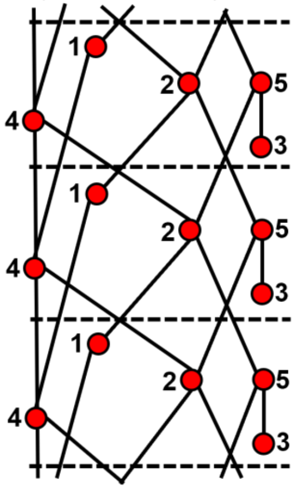	(8x**1**, 1x**2**, 2x**2**^{2}) **e-1** 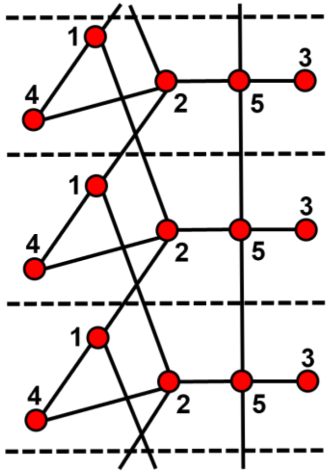	(8x**1**, 1x**2**, 2x**2**^{2}) **e-2** 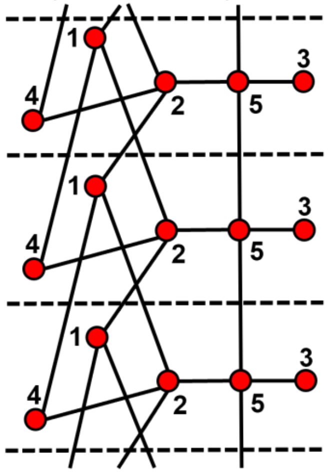
	(8x**1**, 1x**2**, 2x**2**^{2}) **e-3** 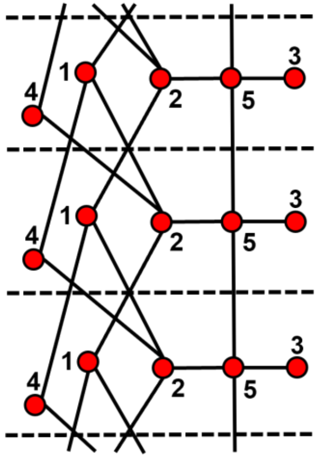	(8x**1**, 1x**2**, 2x**2**^{2}) **f-1** 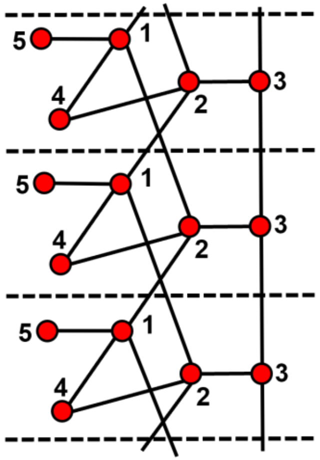	(8x**1**, 1x**2**, 2x**2**^{2}) **f-2** 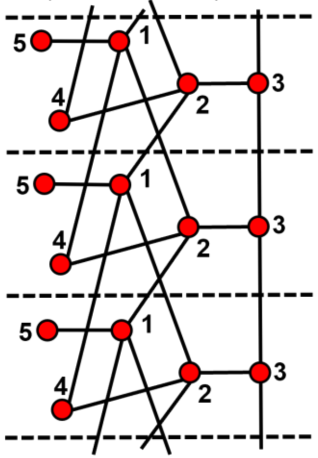	(8x**1**, 1x**2**, 2x**2**^{2}) **f-3** 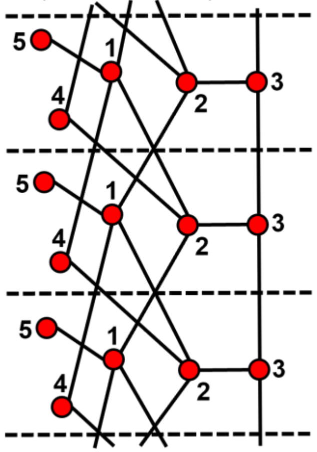

	(8x**1**, 1x**2**, 2x**2**^{2}) **g-1** 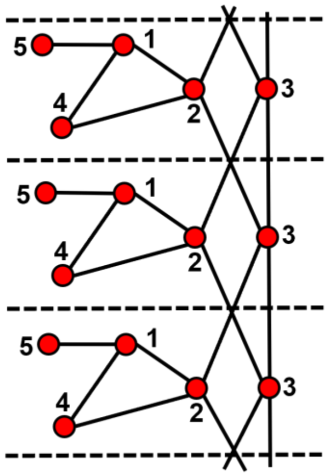	(8x**1**, 1x**2**, 2x**2**^{2}) **g-2** 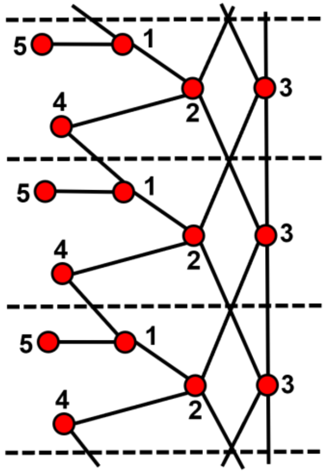	(8x**1**, 1x**2**, 2x**2**^{2}) **g-3** 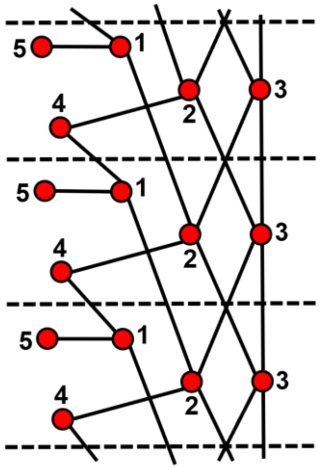	(8x**1**, 1x**2**, 2x**2**^{2}) **g-4** 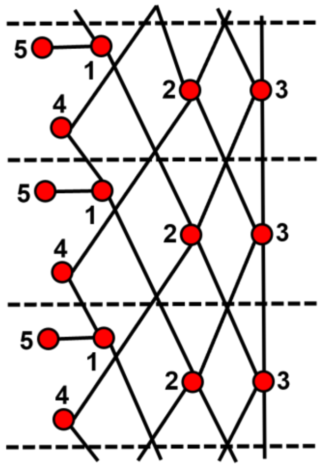
	(8x**1**, 1x**2**, 2x**2**^{2}) **h-1** 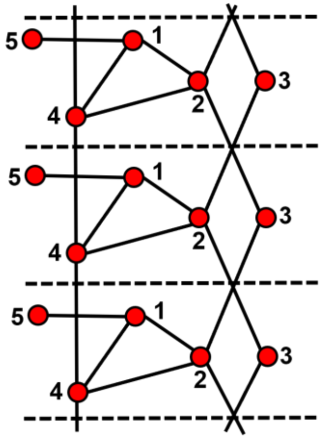	(8x**1**, 1x**2**, 2x**2**^{2}) **h-2** 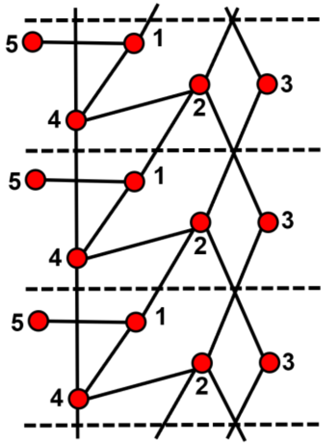	(8x**1**, 1x**2**, 2x**2**^{2}) **h-3** 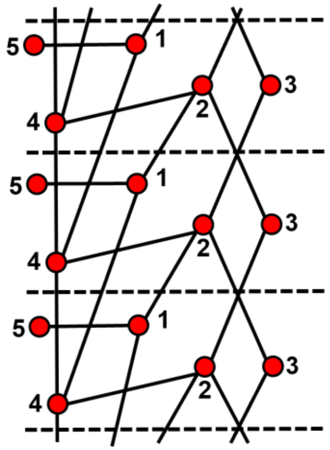	(8x**1**, 1x**2**, 2x**2**^{2}) **h-4** 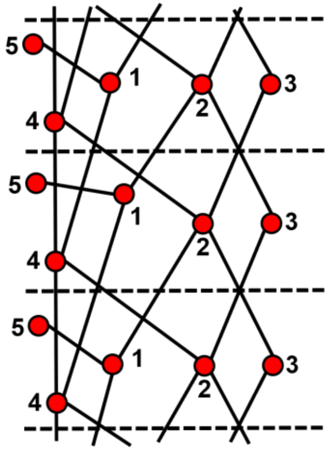

	(6x**1**, 4x**2**) 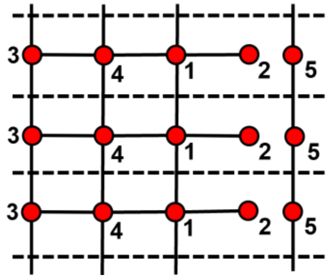	(6x**1**, 2x**2**, 2x**2**^{1}) **a** 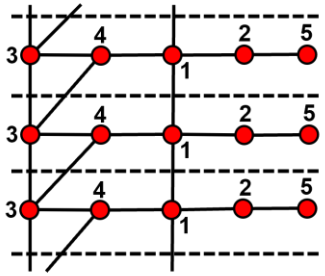	(6x**1**, 2x**2**, 2x**2**^{1}) **b** 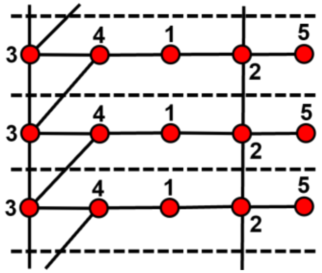	(6x**1**, 2x**2**, 2x**2**^{1}) **c** 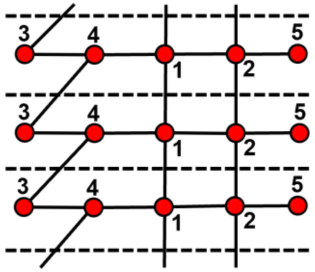
	(6x**1**, 2x**2**, 2x**2**^{1}) **d** 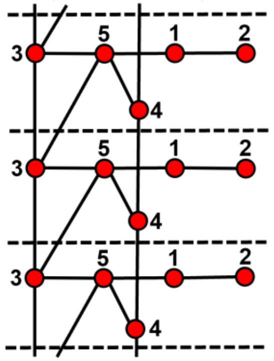	(6x**1**, 2x**2**, 2x**2**^{1}) **e** 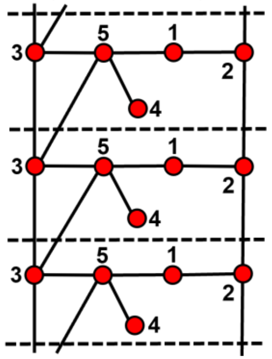	(6x**1**, 2x**2**, 2x**2**^{1}) **f** 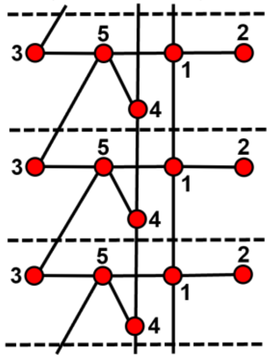	(6x**1**, 2x**2**, 2x**2**^{1}) **g** 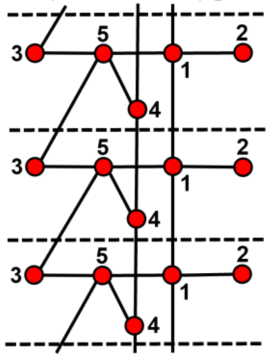
	(6x**1**, 2x**2**, 2x**2**^{1}) **h-1** 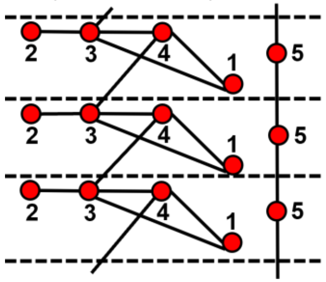	(6x**1**, 2x**2**, 2x**2**^{1}) **h-2** 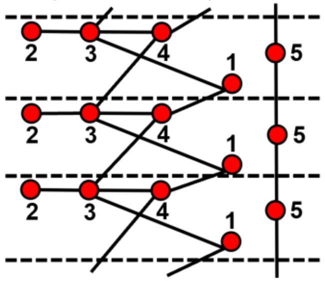	(6x**1**, 2x**2**, 2x**2**^{1}) **h-3** 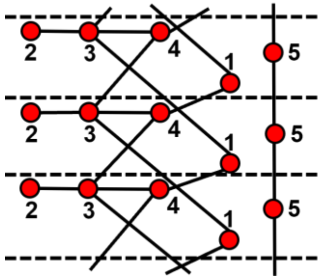	(6x**1**, 2x**2**, 2x**2**^{2}) **a** 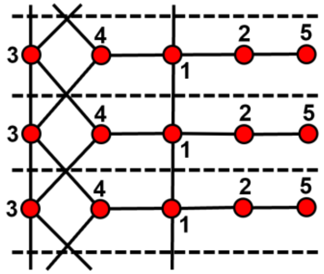

	(6x**1**, 2x**2**, 2x**2**^{2}) **b** 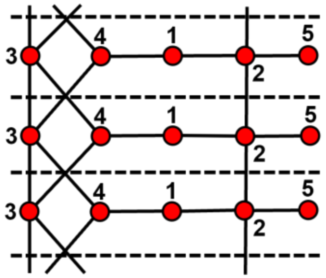	(6x**1**, 2x**2**, 2x**2**^{2}) **c** 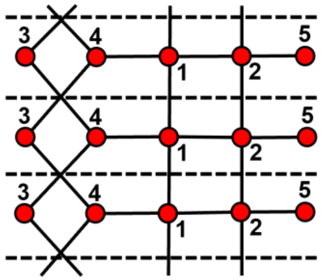	(6x**1**, 2x**2**, 2x**2**^{2}) **d** 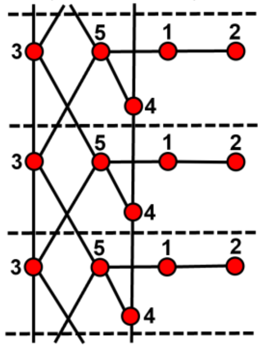	(6x**1**, 2x**2**, 2x**2**^{2}) **e** 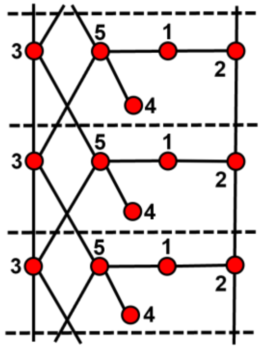
	(6x**1**, 2x**2**, 2x**2**^{2}) **f** 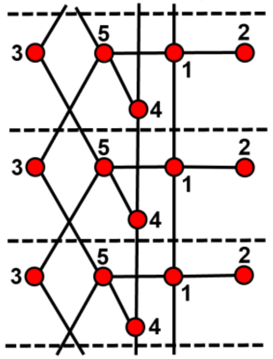	(6x**1**, 2x**2**, 2x**2**^{2}) **g** 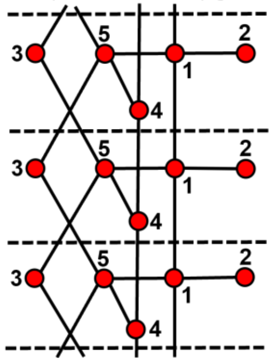	(6x**1**, 2x**2**, 2x**2**^{2}) **h-1** 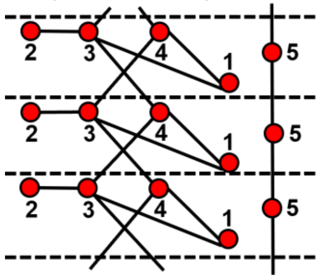	(6x**1**, 2x**2**, 2x**2**^{2}) **h-2** 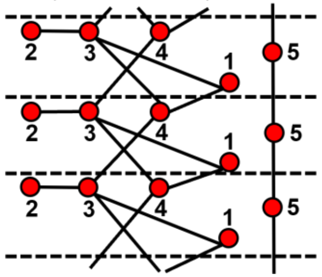

	(6x**1**, 2x**2**, 2x**2**^{2}) **h-3** 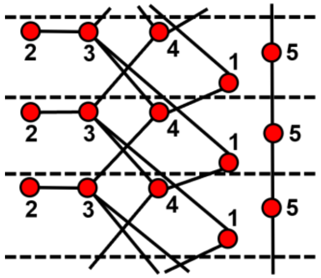	(6x**1**, 4x**2**^{1}) **a-1** 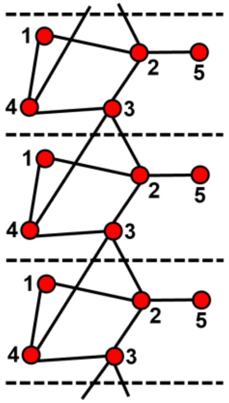	(6x**1**, 4x**2**^{1}) **a-2** 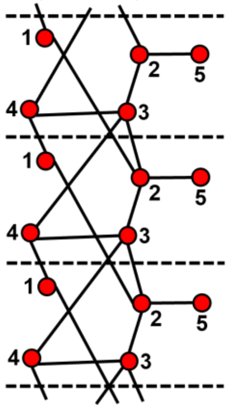	(6x**1**, 4x**2**^{1}) **b-1** 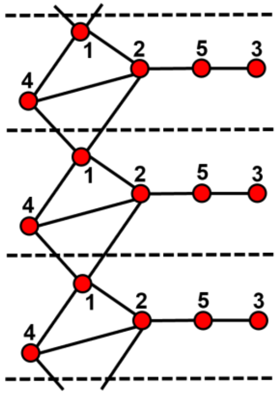
	(6x**1**, 4x**2**^{1}) **b-2** 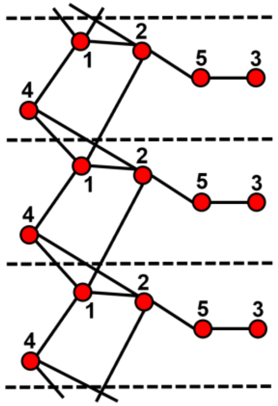	(6x**1**, 4x**2**^{1}) **c-1** 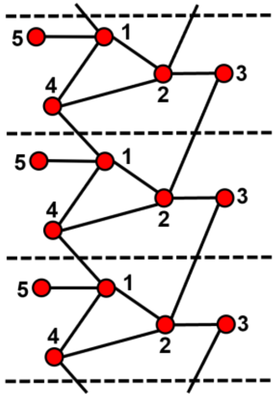	(6x**1**, 4x**2**^{1}) **c-2** 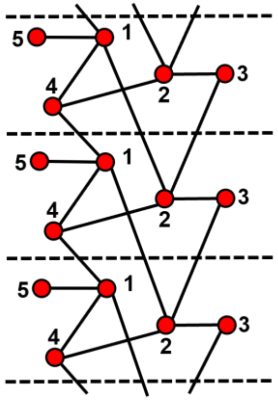	(6x**1**, 4x**2**^{1}) **c-3** 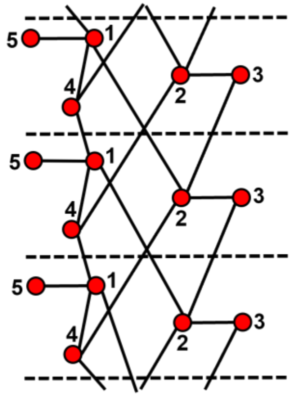

	(6x**1**, 2x**2**^{1}, 2x**2**^{2}) **a-1** 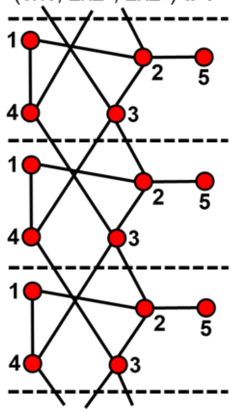	(6x**1**, 2x**2**^{1}, 2x**2**^{2}) **a-2** 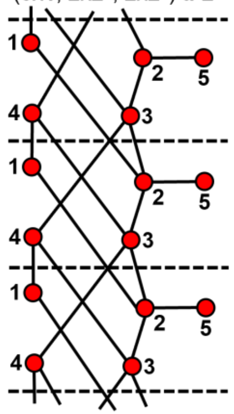	(6x**1**, 2x**2**^{1}, 2x**2**^{2}) **b-1** 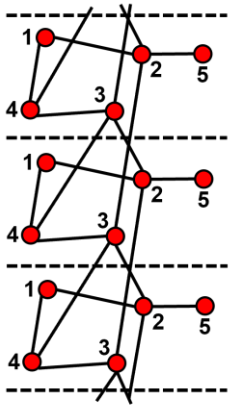	(6x**1**, 2x**2**^{1}, 2x**2**^{2}) **b-2** 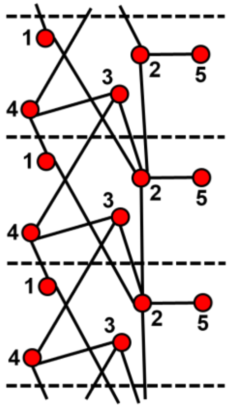
	(6x**1**, 2x**2**^{1}, 2x**2**^{2}) **c-1** 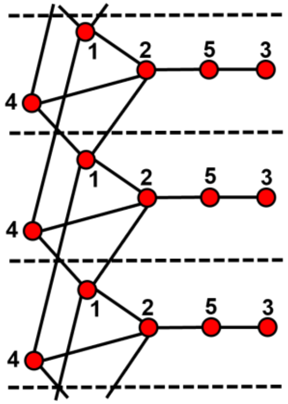	(6x**1**, 2x**2**^{1}, 2x**2**^{2}) **c-2** 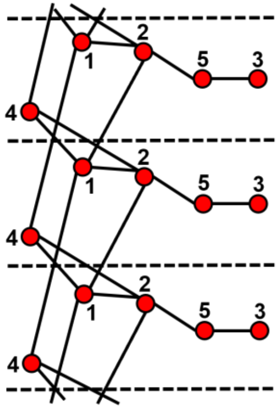	(6x**1**, 2x**2**^{1}, 2x**2**^{2}) **d-1** 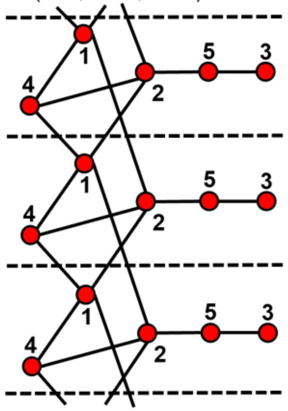	(6x**1**, 2x**2**^{1}, 2x**2**^{2}) **d-2** 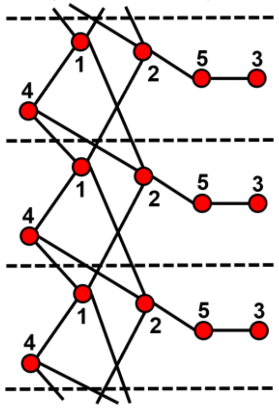

	(6x**1**, 2x**2**^{1}, 2x**2**^{2}) **e-1** 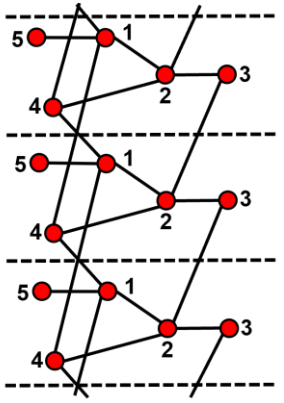	(6x**1**, 2x**2**^{1}, 2x**2**^{2}) **e-2** 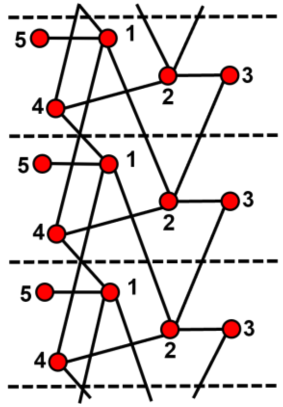	(6x**1**, 2x**2**^{1}, 2x**2**^{2}) **e-3** 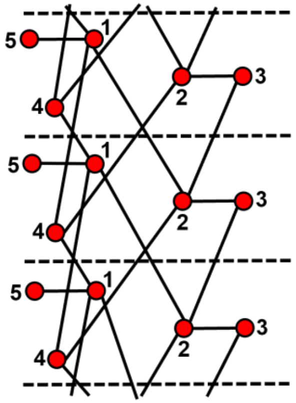	(6x**1**, 2x**2**^{1}, 2x**2**^{2}) **f-1** 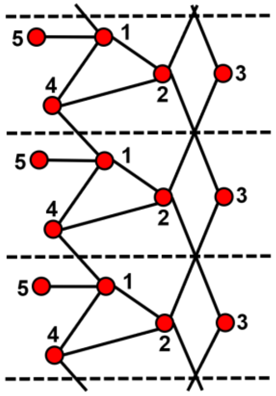
	(6x**1**, 2x**2**^{1}, 2x**2**^{2}) **f-2** 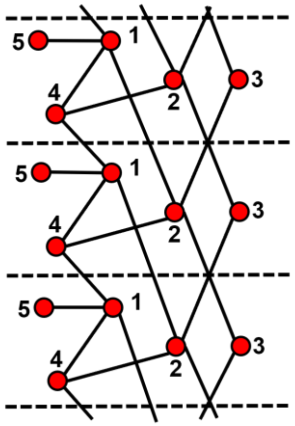	(6x**1**, 2x**2**^{1}, 2x**2**^{2}) **f-3** 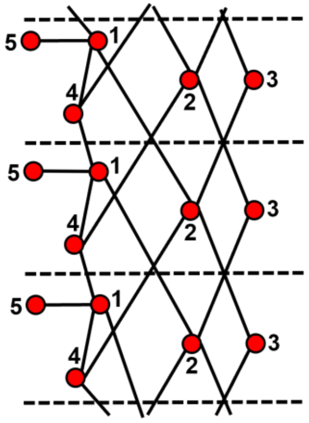	(6x**1**, 4x**2**^{2}) **a-1** 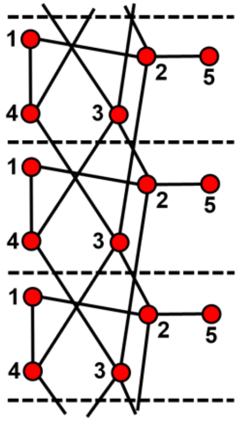	(6x**1**, 4x**2**^{2}) **a-2** 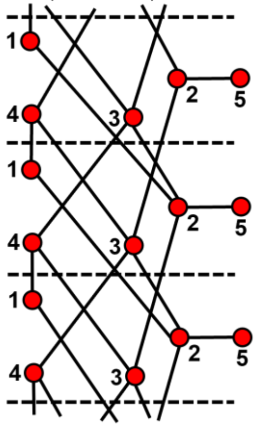

	(6x**1**, 4x**2**^{2}) **b-1** 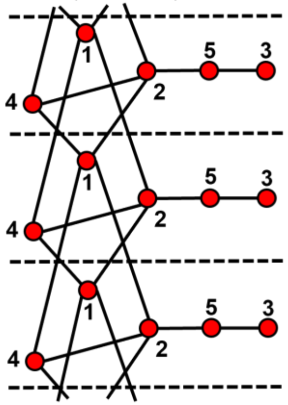	(6x**1**, 4x**2**^{2}) **b-2** 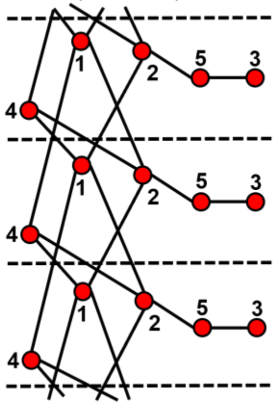	(6x**1**, 4x**2**^{2}) **c-1** 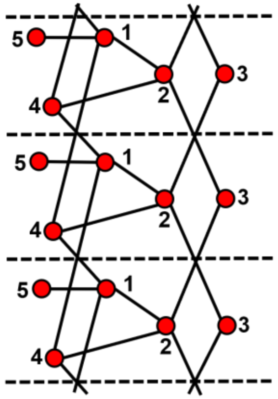	(6x**1**, 4x**2**^{2}) **c-2** 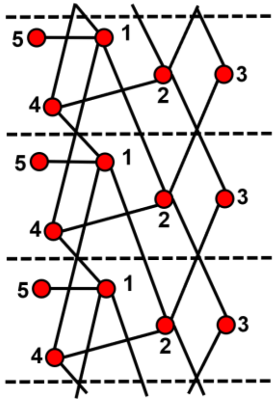
	(6x**1**, 4x**2**^{2}) **c-3** 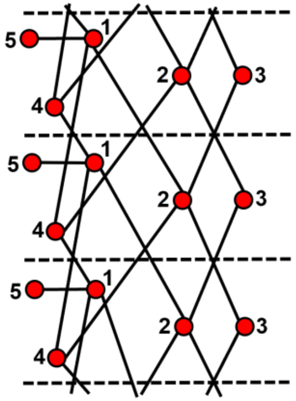	(4x**1**, 3x**2**, 2x**2**^{1}) **a** 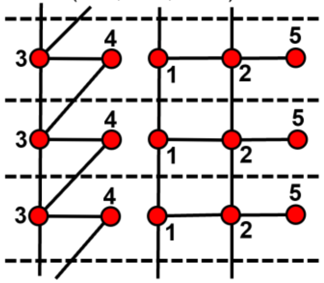	(4x**1**, 3x**2**, 2x**2**^{1}) **b** 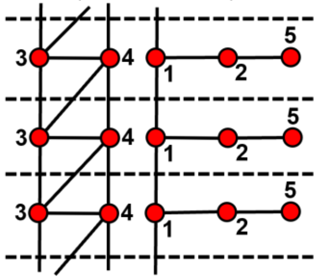	(4x**1**, 3x**2**, 2x**2**^{1}) **c** 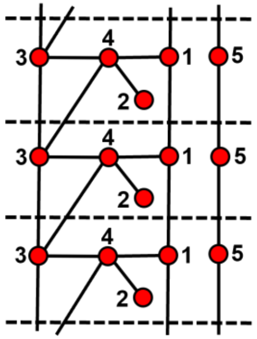

	(4x**1**, 3x**2**, 2x$\mathbf{2}^{1}$) **d**	(4x**1**, 3x**2**, 2x$\mathbf{2}^{2}$) **a**	(4x**1**, 3x**2**, 2x$\mathbf{2}^{2}$) **b**	(4x**1**, 3x**2**, 2x$\mathbf{2}^{2}$) **c**
	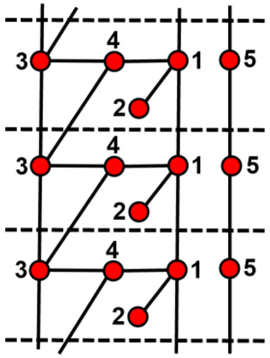	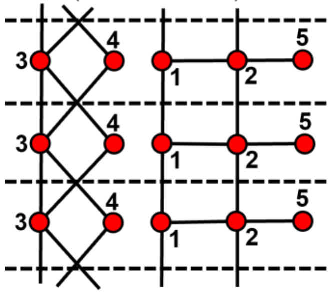	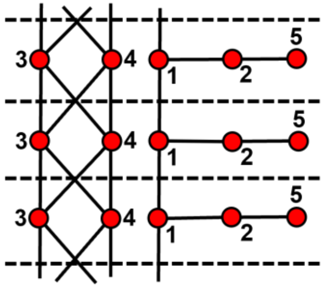	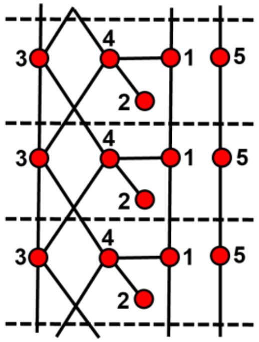
	(4x**1**, 3x**2**, 2x$\mathbf{2}^{2}$) **d**	(4x**1**, 1x**2**, 4x$\mathbf{2}^{1}$) **a**	(4x**1**, 1x**2**, 4x$\mathbf{2}^{1}$) **b**	(4x**1**, 1x**2**, 4x$\mathbf{2}^{1}$) **c**
	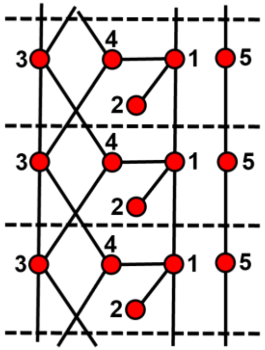	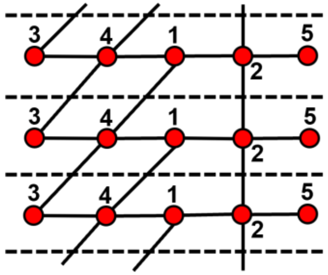	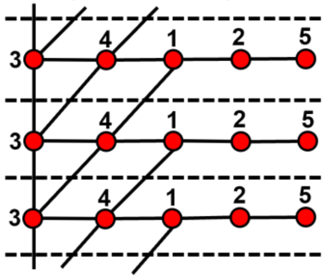	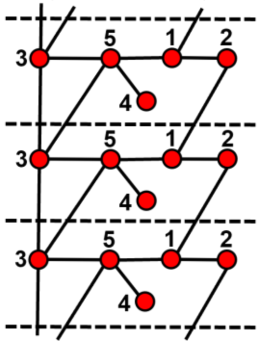

	(4x**1**, 1x**2**, 4x**2**^{1}) **d** 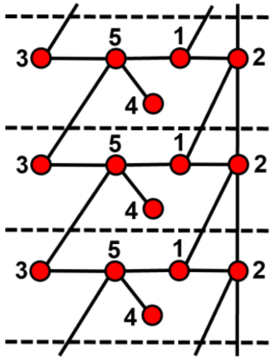	(4x**1**, 1x**2**, 4x**2**^{1}) **e** 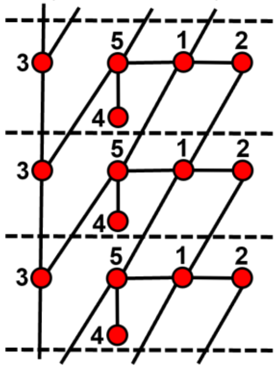	(4x**1**, 1x**2**, 4x**2**^{1}) **f-1** 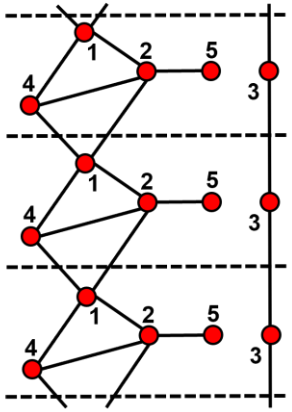	(4x**1**, 1x**2**, 4x**2**^{1}) **f-2** 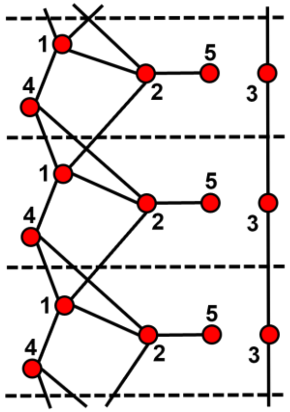
	(4x**1**, 1x**2**, 2x**2**^{1}, 2x**2**^{2}) **a** 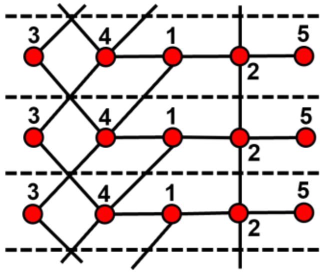	(4x**1**, 1x**2**, 2x**2**^{1}, 2x**2**^{2}) **b** 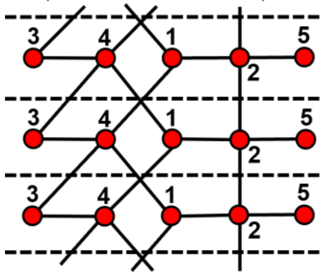	(4x**1**, 1x**2**, 2x**2**^{1}, 2x**2**^{2}) **c** 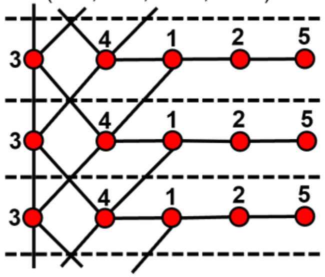	(4x**1**, 1x**2**, 4x**2**^{1}) **d** 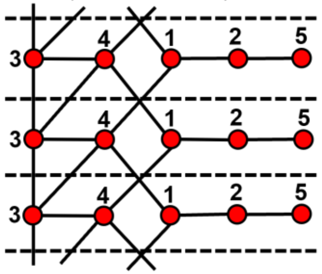

	(4x**1**, 1x**2**, 2x**2**^{1}, 2x**2**^{2}) **e** 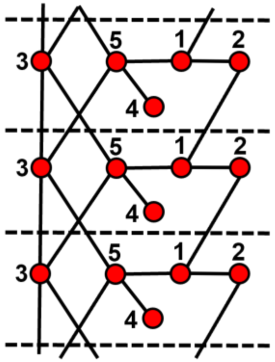	(4x**1**, 1x**2**, 2x**2**^{1}, 2x**2**^{2}) **f** 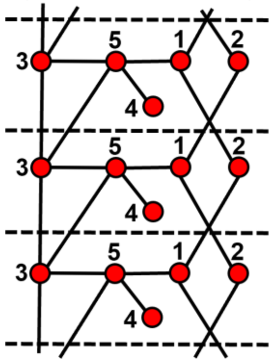	(4x**1**, 1x**2**, 2x**2**^{1}, 2x**2**^{2}) **g** 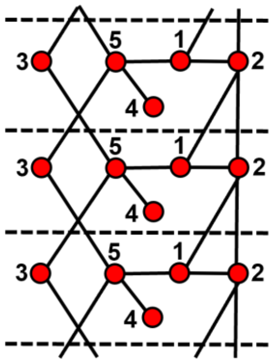	(4x**1**, 1x**2**, 2x**2**^{1}, 2x**2**^{2}) **h** 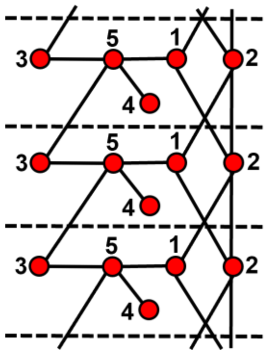
	(4x**1**, 1x**2**, 2x**2**^{1}, 2x**2**^{2}) **i** 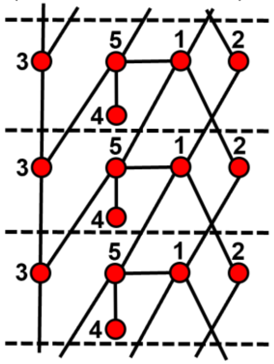	(4x**1**, 1x**2**, 2x**2**^{1}, 2x**2**^{2}) **j** 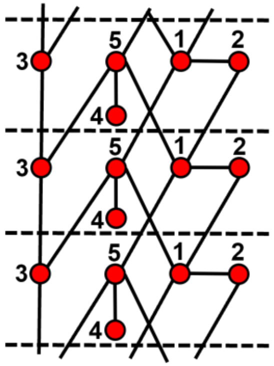	(4x**1**, 1x**2**, 2x**2**^{1}, 2x**2**^{2}) **k-1** 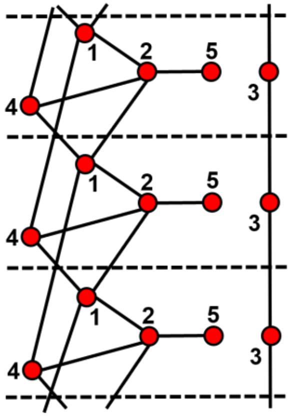	(4x**1**, 1x**2**, 2x**2**^{1}, 2x**2**^{2}) **k-2** 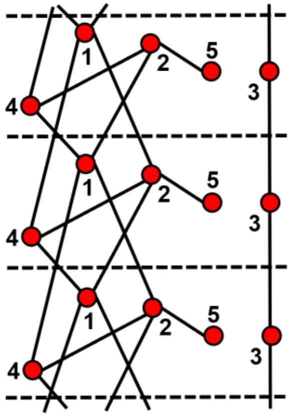

	(4x**1**, 1x**2**, 2x**2**^{1}, 2x**2**^{2}) **l-1** 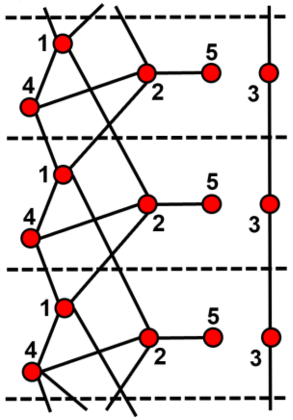	(4x**1**, 1x**2**, 2x**2**^{1}, 2x**2**^{2}) **l-2** 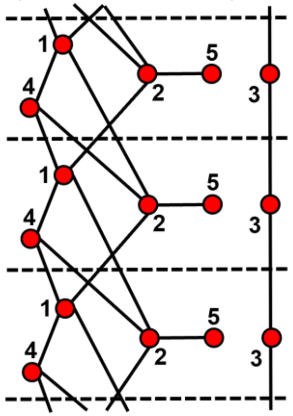	(4x**1**, 1x**2**, 4x**2**^{2}) **a** 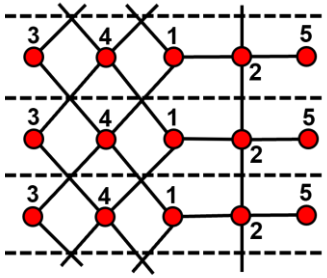	(4x**1**, 1x**2**, 4x**2**^{2}) **b** 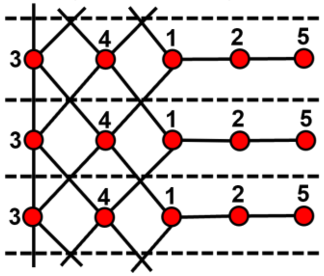
	(4x**1**, 1x**2**, 4x**2**^{2}) **c** 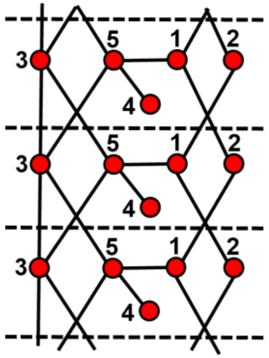	(4x**1**, 1x**2**, 4x**2**^{2}) **d** 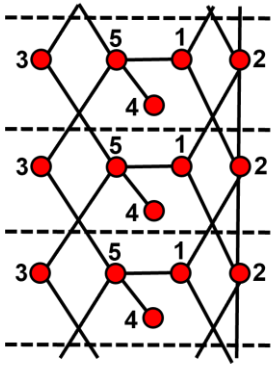	(4x**1**, 1x**2**, 4x**2**^{2}) **e** 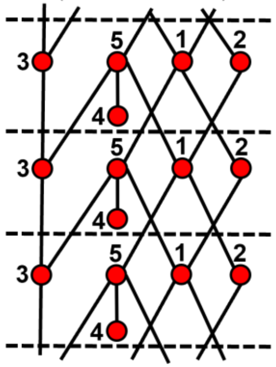	(4x**1**, 1x**2**, 4x**2**^{2}) **f-1** 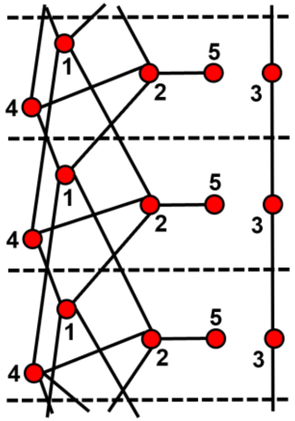

	(4x**1**, 1x**2**, 4x**2**^{2}) **f-2** 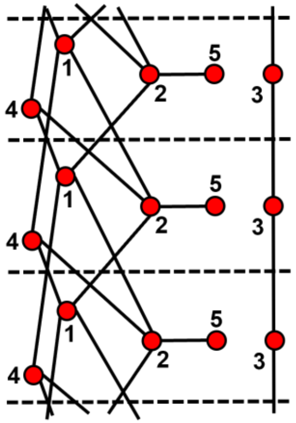	(2x**1**, 4x**2**, 2x**2**^{1}) 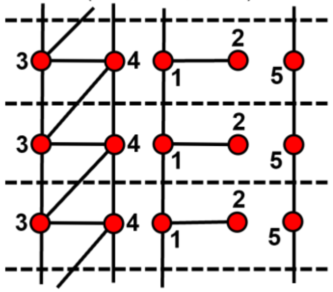	(2x**1**, 4x**2**, 2x**2**^{2}) 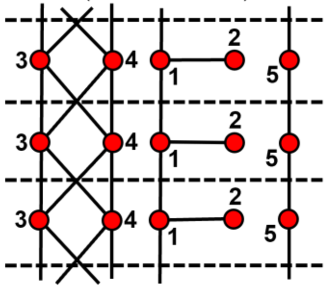	(2x**1**, 2x**2**, 4x**2**^{1}) **a** 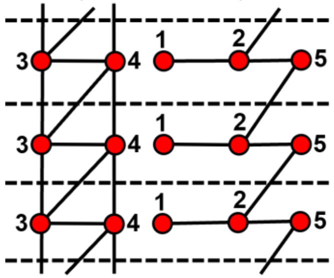
	(2x**1**, 2x**2**, 4x**2**^{1}) **b** 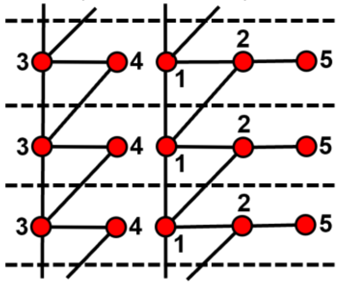	(2x**1**, 2x**2**, 4x**2**^{1}) **c** 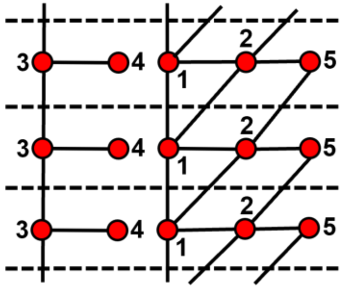	(2x**1**, 2x**2**, 4x**2**^{1}) **d** 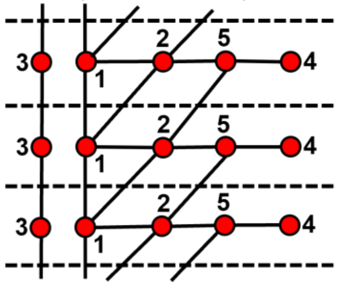	(2x**1**, 2x**2**, 2x**2**^{1}, 2x**2**^{2}) **a** 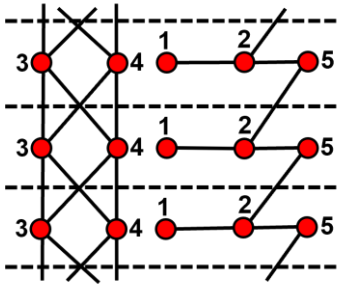
	(2x**1**, 2x**2**, 2x**2**^{1}, 2x**2**^{2}) **b** 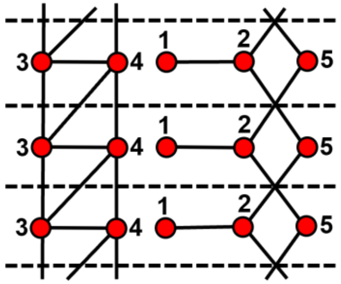	(2x**1**, 2x**2**, 2x**2**^{1}, 2x**2**^{2}) **c** 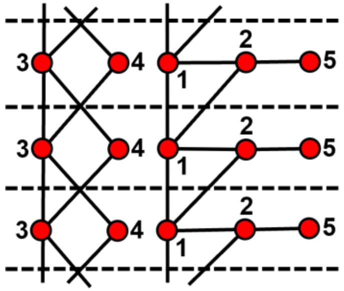	(2x**1**, 2x**2**, 2x**2**^{1}, 2x**2**^{2}) **d** 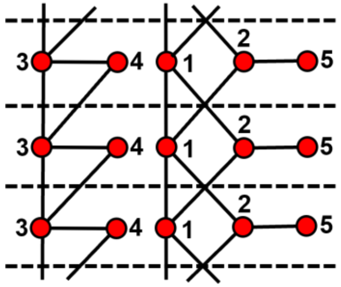	(2x**1**, 2x**2**, 2x**2**^{1}, 2x**2**^{2}) **e** 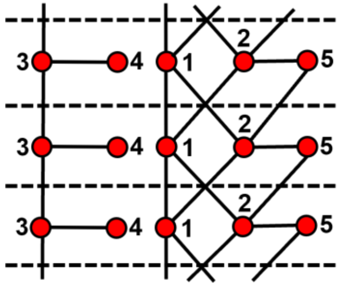

	(2x**1**, 2x**2**, 2x**2**^{1}, 2x**2**^{2}) **f** 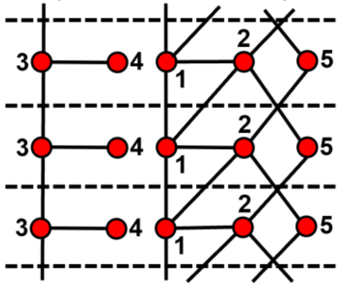	(2x**1**, 2x**2**, 2x**2**^{1}, 2x**2**^{2}) **g** 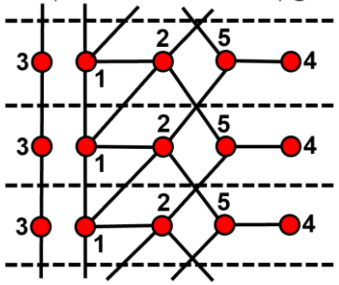	(2x**1**, 2x**2**, 2x**2**^{1}, 2x**2**^{2}) **h** 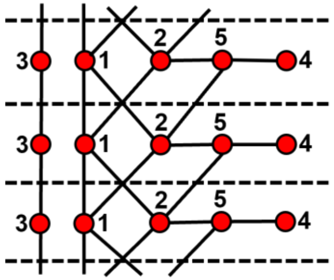	(2x**1**, 2x**2**, 4x**2**^{2}) **a** 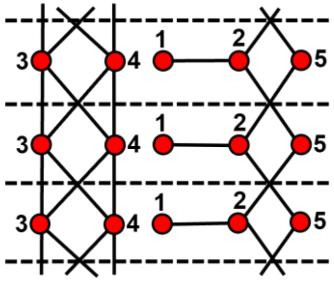
	(2x**1**, 2x**2**, 4x**2**^{2}) **b** 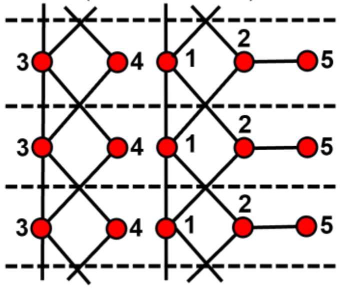	(2x**1**, 2x**2**, 4x**2**^{2}) **c** 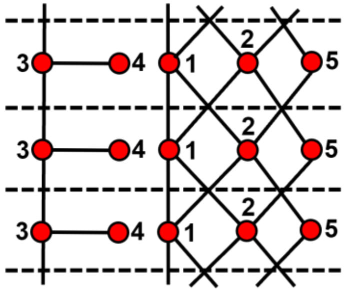	(2x**1**, 2x**2**, 4x**2**^{2}) **d** 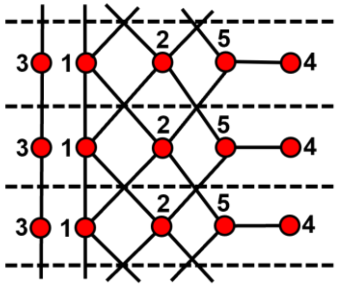	(2x**1**, 6x**2**^{1}) 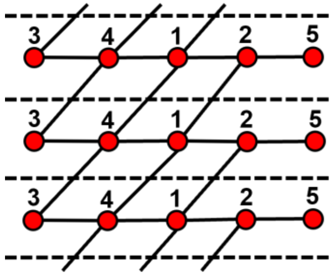
	(2x**1**, 4x**2**^{1}, 2x**2**^{2}) **a** 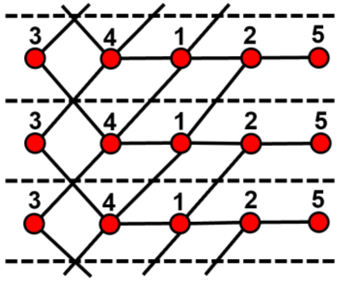	(2x**1**, 4x**2**^{1}, 2x**2**^{2}) **b** 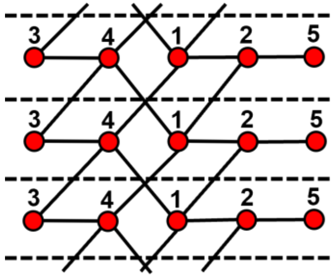	(2x**1**, 4x**2**^{1}, 2x**2**^{2}) **c** 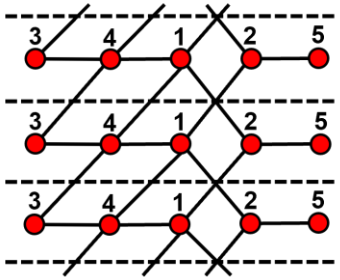	(2x**1**, 2x**2**^{1}, 4x**2**^{2}) **a** 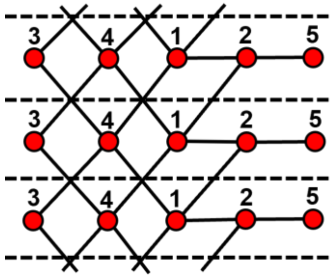

	(2x**1**, 2x**2**^{1}, 4x**2**^{2}) **b** 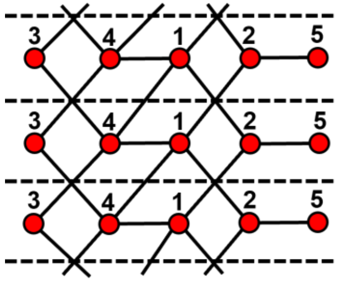	(2x**1**, 2x**2**^{1}, 4x**2**^{2}) **c** 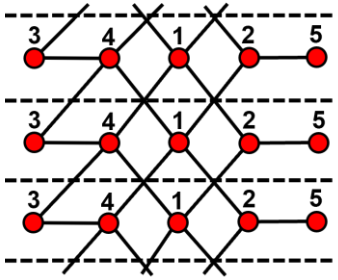	(2x**1**, 6x**2**^{2}) 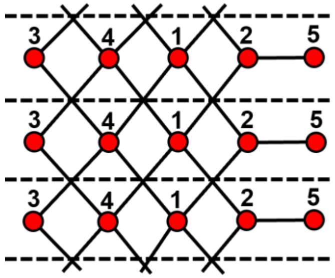	
$^{1}V_{1}{}^{2}V_{1}{}^{3}V_{1}{}^{4}V_{3}$	NG			
$^{1}V_{1}{}^{2}V_{1}{}^{3}V_{1}{}^{4}V_{4}$	NG			
$^{1}V_{1}{}^{2}V_{1}{}^{3}V_{1}{}^{4}V_{5}$	NG			
$^{1}V_{1}{}^{2}V_{1}{}^{3}V_{3}{}^{4}V_{1}$	NG			
$^{1}V_{1}{}^{2}V_{1}{}^{3}V_{3}{}^{4}V_{2}$	NG			
$^{1}V_{1}{}^{2}V_{1}{}^{3}V_{3}{}^{4}V_{3}$	NG			
$^{1}V_{1}{}^{2}V_{1}{}^{3}V_{5}{}^{4}V_{1}$	NG			

$^{1}V_{1}{}^{2}V_{2}{}^{3}V_{1}{}^{4}V_{1}$	(12x**1**) **a**<br>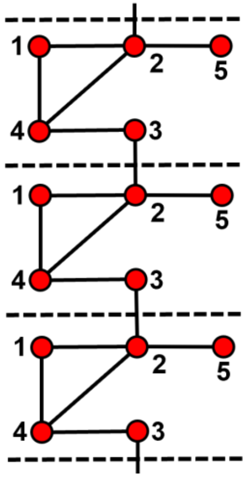	(12x**1**) **b**<br>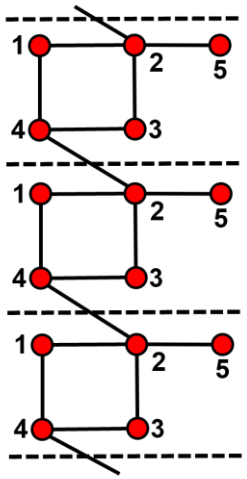	(12x**1**) **c**<br>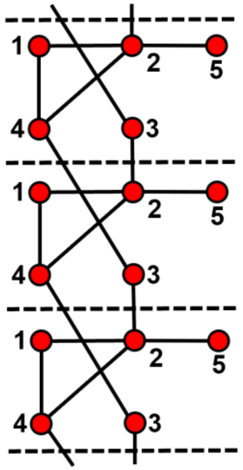	(12x**1**) **d**<br>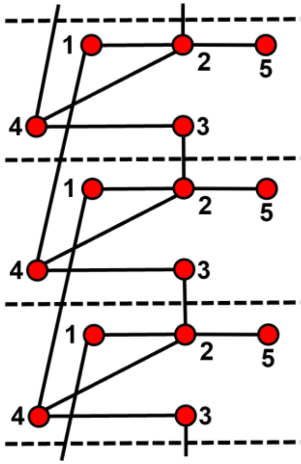
	(12x**1**) **e**<br>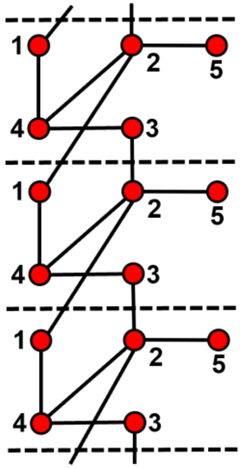	(12x**1**) **f**<br>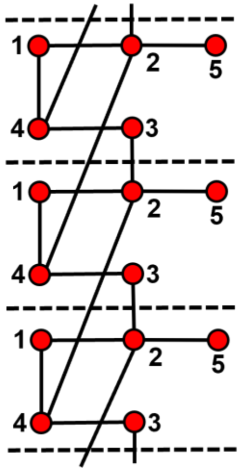	(12x**1**) **g**<br>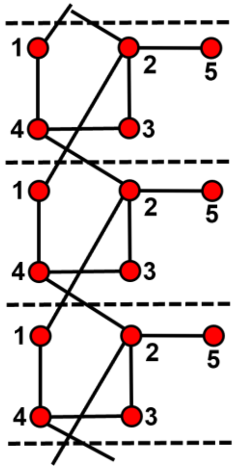	(12x**1**) **h**<br>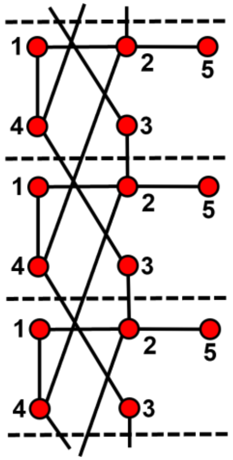

	(12x**1**) **i** 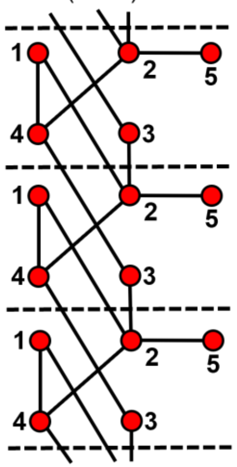	(12x**1**) **j** 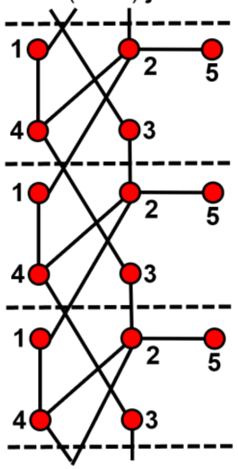	(12x**1**) **k** 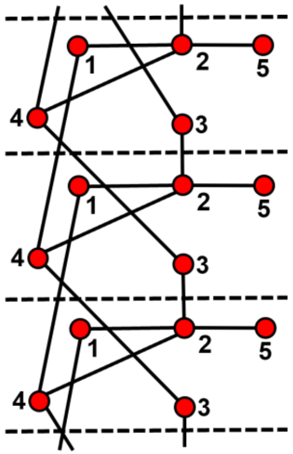	(12x**1**) **l** 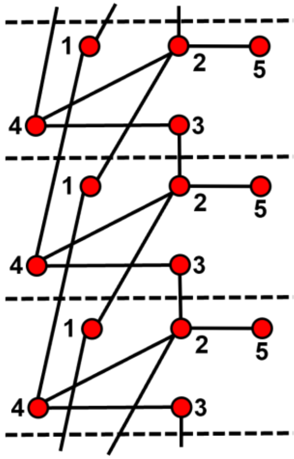
	(12x**1**) **m** 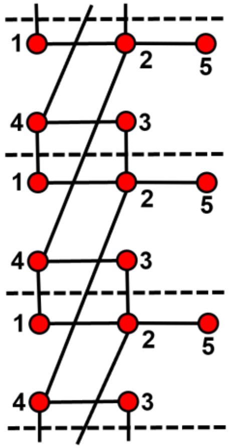	(12x**1**) **n** 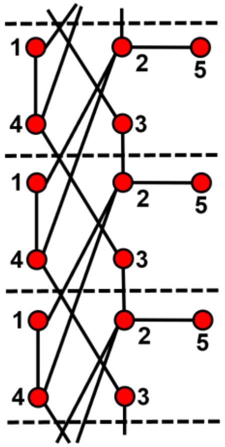	(12x**1**) **o** 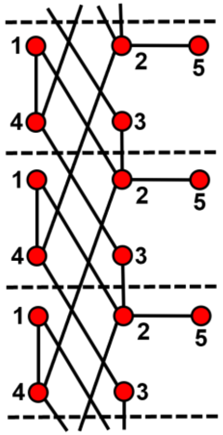	(12x**1**) **p** 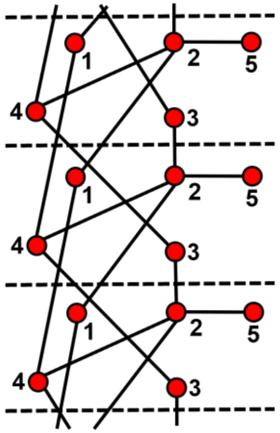

	(12x**1**) **q** 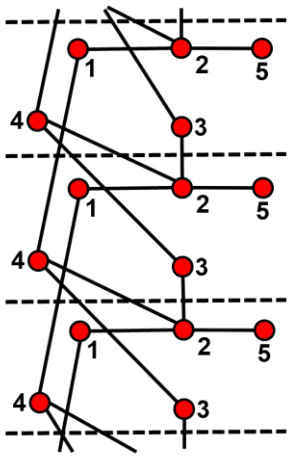	(12x**1**) **r** 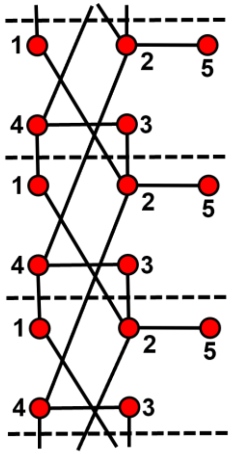	(12x**1**) **s** 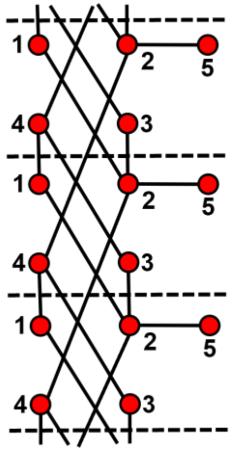	(12x**1**) **t** 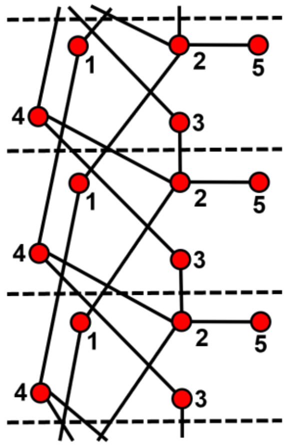
	(12x**1**) **u** 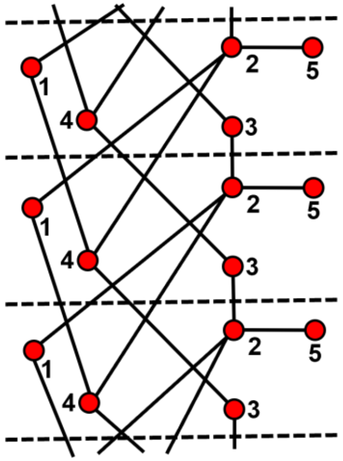	(10x**1**, 1x**2**) **a-1** 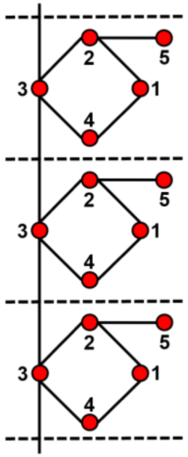	(10x**1**, 1x**2**) **a-2** 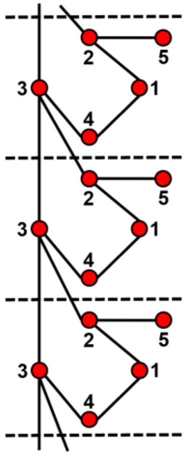	(10x**1**, 1x**2**) **a-3** 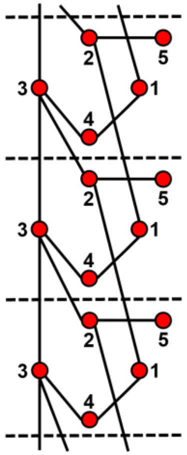

	(10x**1**, 1x**2**) **a-4** 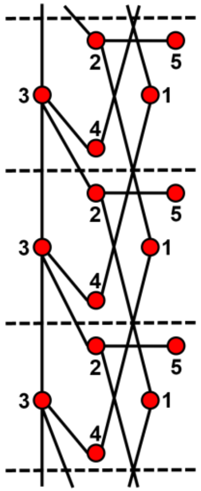	(10x**1**, 1x**2**) **a-5** 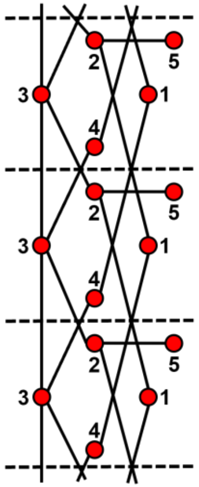	(10x**1**, 1x**2**) **b-1** 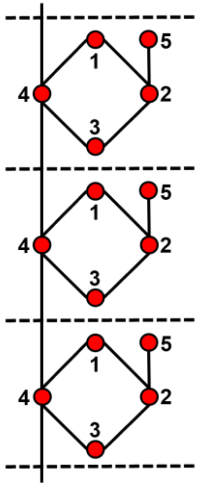	(10x**1**, 1x**2**) **b-2** 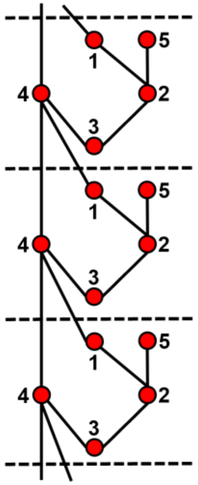
	(10x**1**, 1x**2**) **b-3** 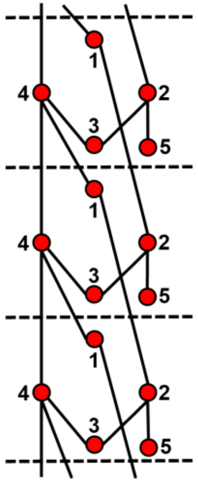	(10x**1**, 1x**2**) **b-4** 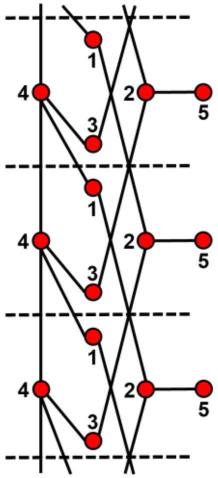	(10x**1**, 1x**2**) **b-5** 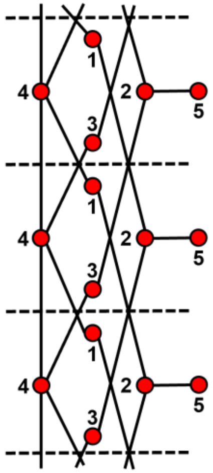	(10x**1**, 1x**2**) **c-1** 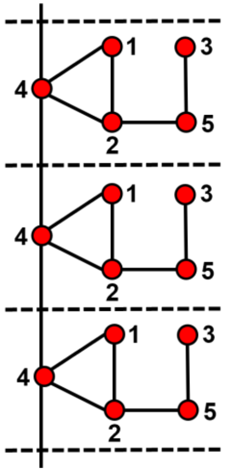

	(10x**1**, 1x**2**) **c-2** 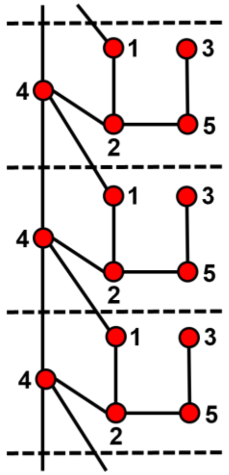	(10x**1**, 1x**2**) **c-3** 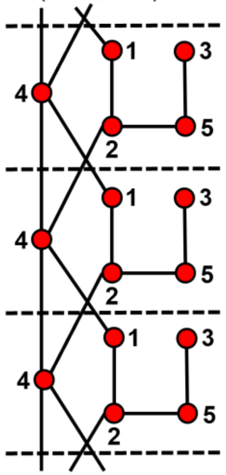	(10x**1**, 1x**2**) **c-4** 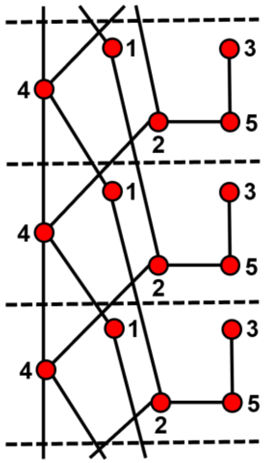	(10x**1**, 1x**2**) **d-1** 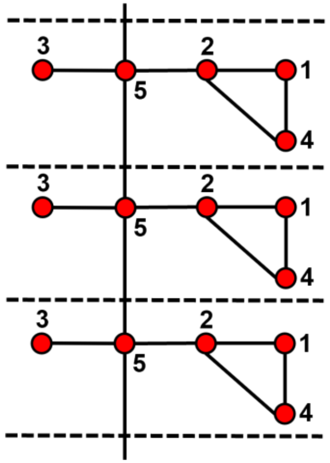
	(10x**1**, 1x**2**) **d-2** 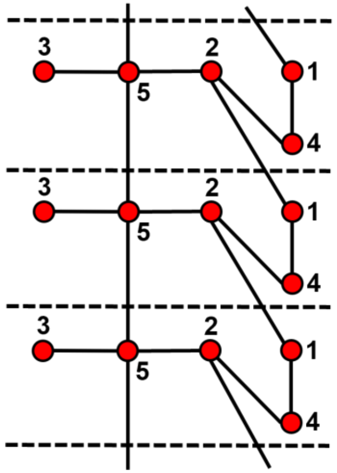	(10x**1**, 1x**2**) **d-3** 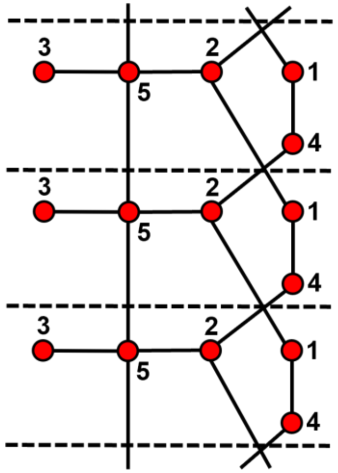	(10x**1**, 1x**2**) **d-4** 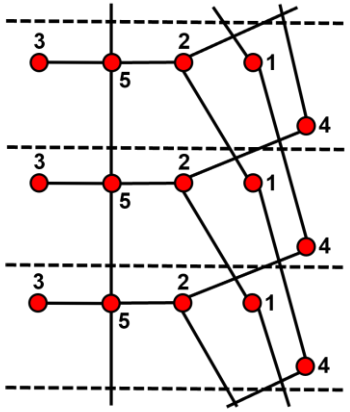	(10x**1**, 1x**2**) **e-1** 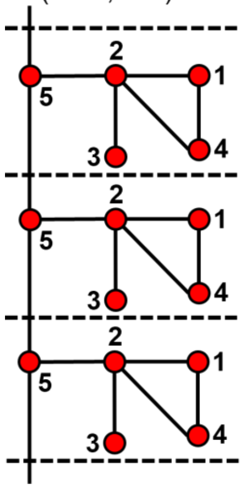

	(10x**1**, 1x**2**) **e-2** 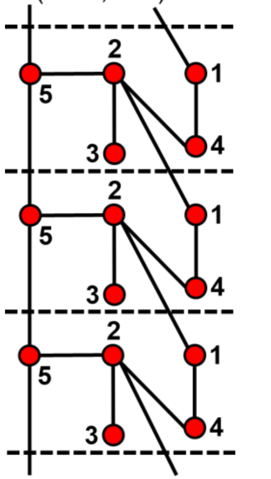	(10x**1**, 1x**2**) **e-3** 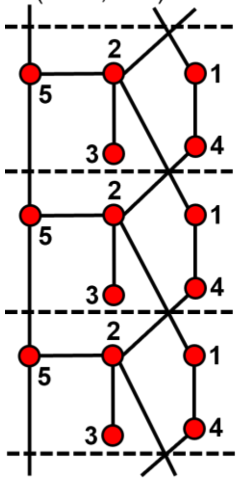	(10x**1**, 1x**2**) **e-4** 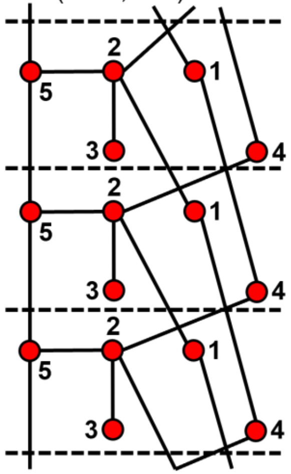	(8x**1**, 2x**2**) **a-1** 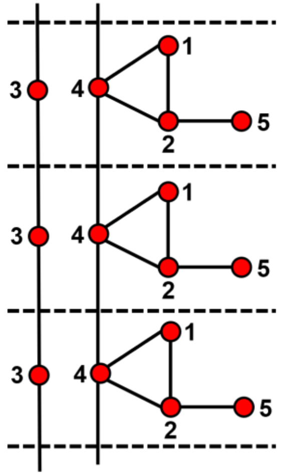
	(8x**1**, 2x**2**) **a-2** 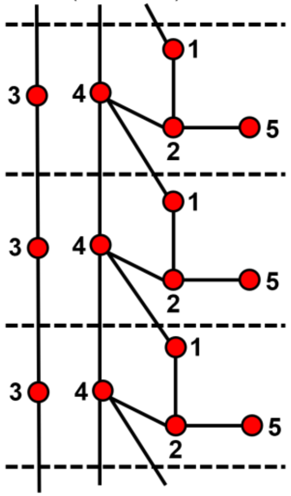	(8x**1**, 2x**2**) **a-3** 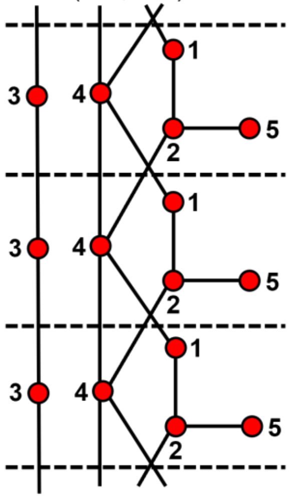	(8x**1**, 2x**2**) **a-4** 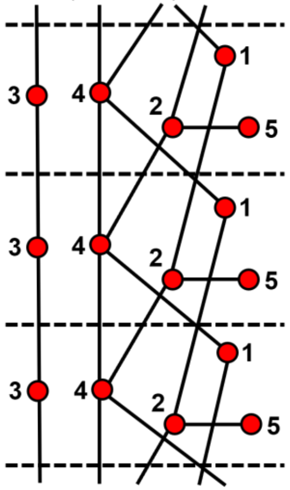	(8x**1**, 2x**2**) **b-1** 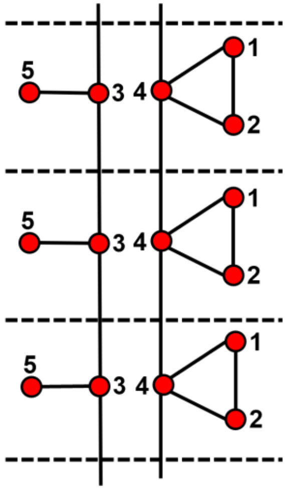

	(8x**1**, 2x**2**) **b-2** 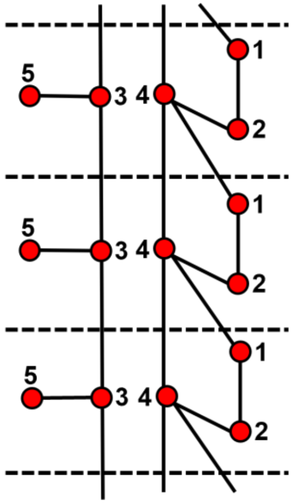	(8x**1**, 2x**2**) **b-3** 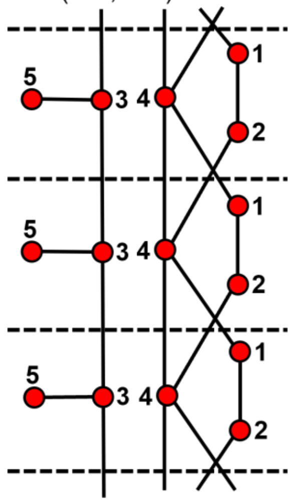	(8x**1**, 2x**2**) **b-4** 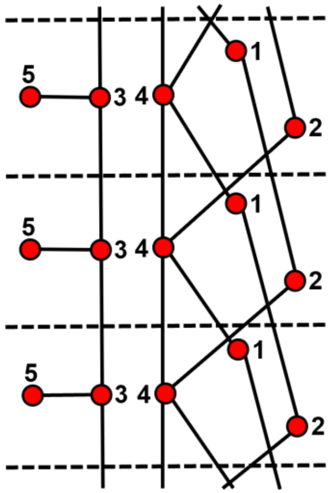	(8x**1**, 2x**2**) **c** 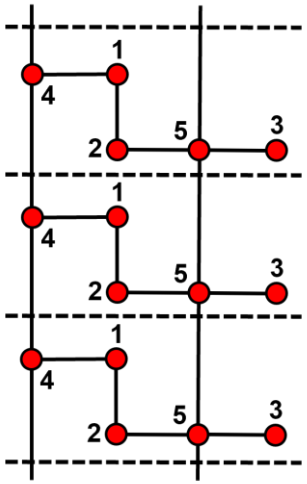
	(8x**1**, 2x**2**) **d** 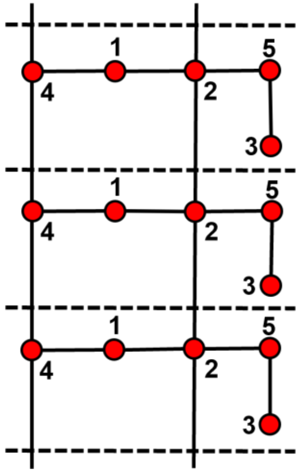	(8x**1**, 2x**2**) **e** 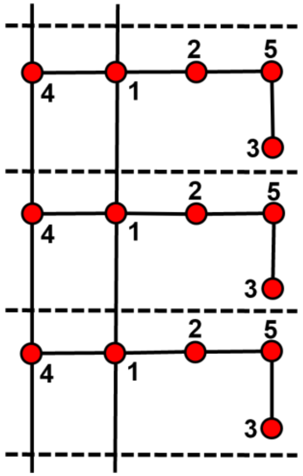	(8x**1**, 2x**2**^{1}) **a-1** 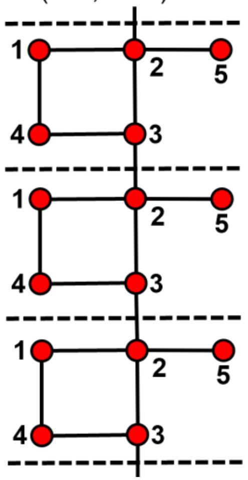	(8x**1**, 2x**2**^{1}) **a-2** 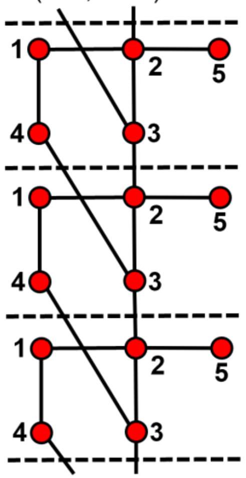

	(8x**1**, 2x**2**^{1}) **a-3**<br>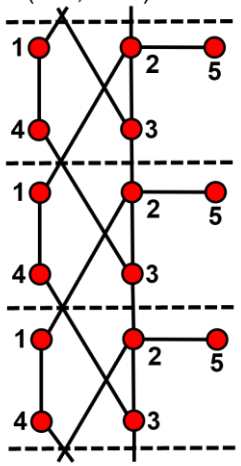	(8x**1**, 2x**2**^{1}) **a-4**<br>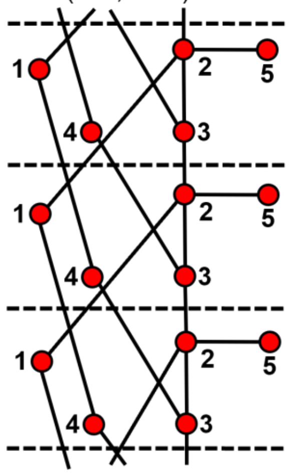	(8x**1**, 2x**2**^{1}) **b-1**<br>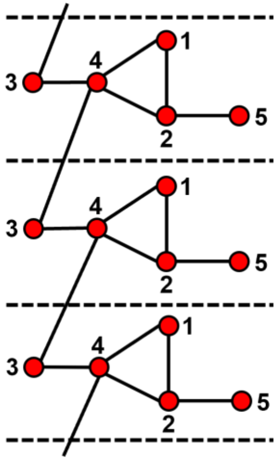	(8x**1**, 2x**2**^{1}) **b-2**<br>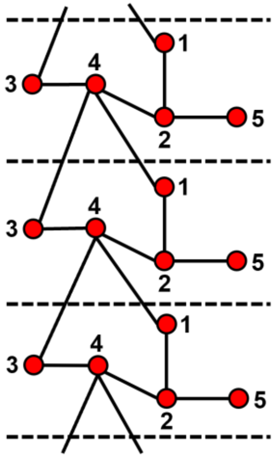
	(8x**1**, 2x**2**^{1}) **b-3**<br>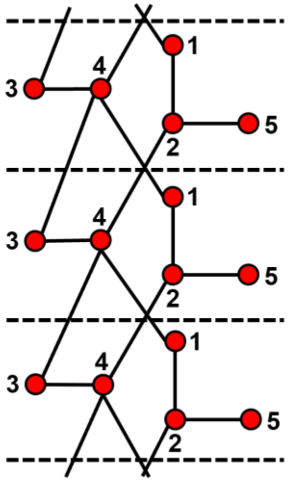	(8x**1**, 2x**2**^{1}) **b-4**<br>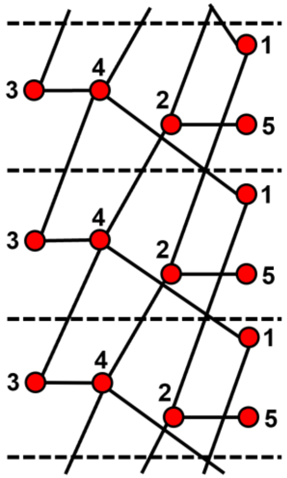	(8x**1**, 2x**2**^{1}) **c-1**<br>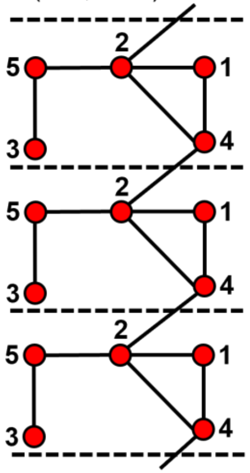	(8x**1**, 2x**2**^{1}) **c-2**<br>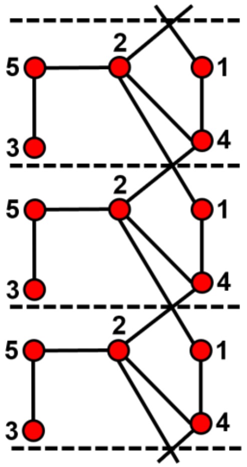

	(8x**1**, 2x$\mathbf{2}^1$) **c-3**<br>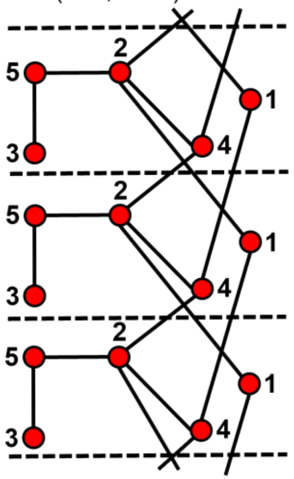	(8x**1**, 2x$\mathbf{2}^1$) **d-1**<br>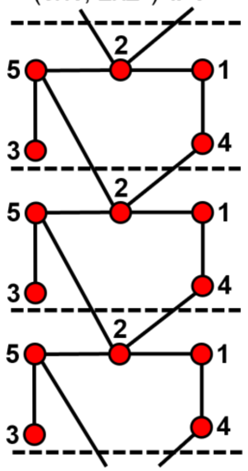	(8x**1**, 2x$\mathbf{2}^1$) **d-2**<br>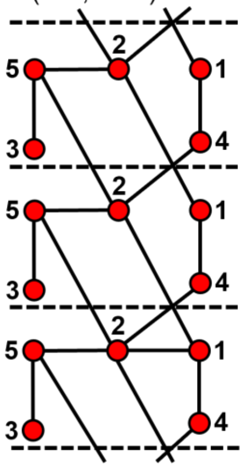	(8x**1**, 2x$\mathbf{2}^1$) **d-3**<br>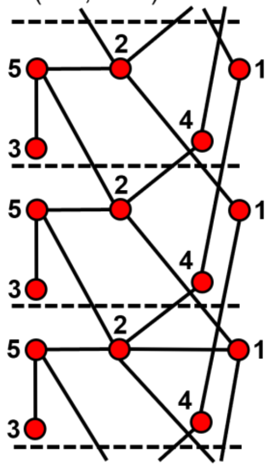
	(8x**1**, 2x$\mathbf{2}^2$) **a-1**<br>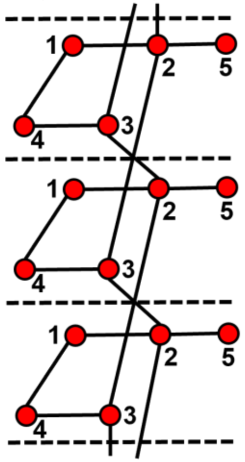	(8x**1**, 2x$\mathbf{2}^2$) **a-2**<br>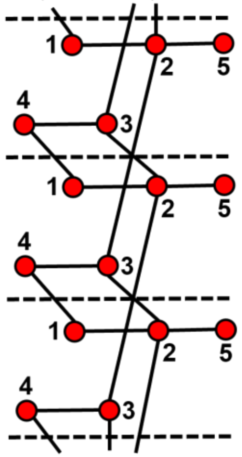	(8x**1**, 2x$\mathbf{2}^2$) **a-3**<br>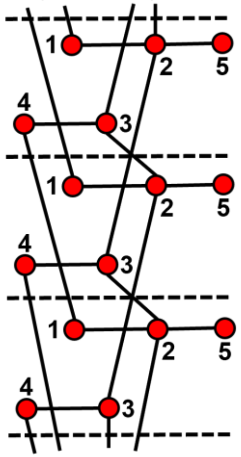	(8x**1**, 2x$\mathbf{2}^2$) **a-4**<br>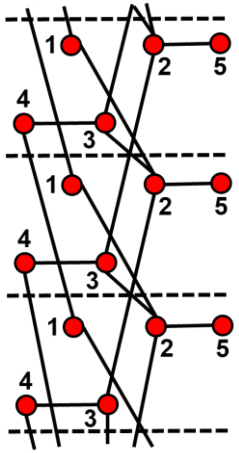

(8x**1**, 2x$\mathbf{2}^{2}$) **b-1**

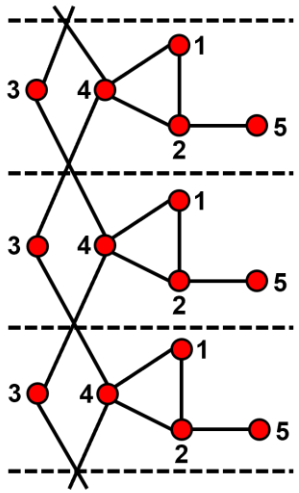

(8x**1**, 2x$\mathbf{2}^{2}$) **b-2**

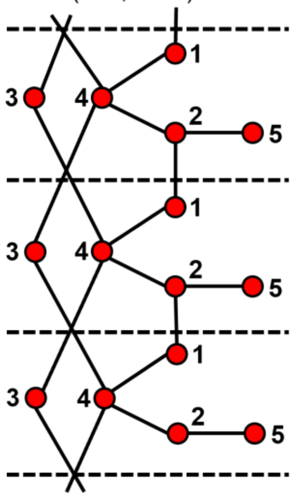

(8x**1**, 2x$\mathbf{2}^{2}$) **b-3**

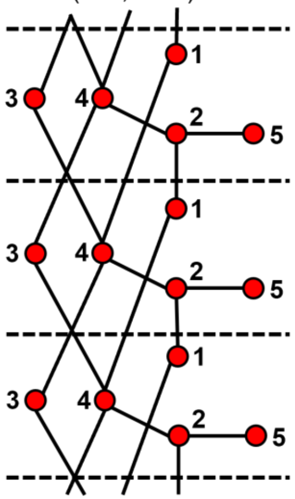

(8x**1**, 2x$\mathbf{2}^{2}$) **b-4**

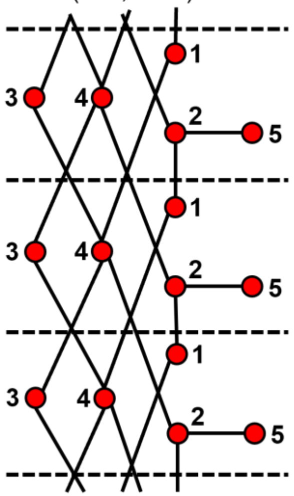

(8x**1**, 2x$\mathbf{2}^{2}$) **c-1**

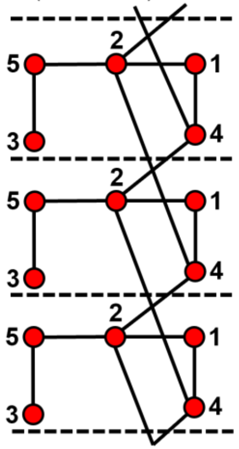

(8x**1**, 2x$\mathbf{2}^{2}$) **c-2**

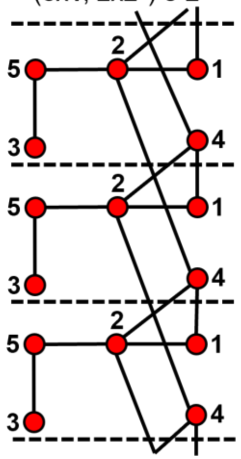

(8x**1**, 2x$\mathbf{2}^{2}$) **c-3**

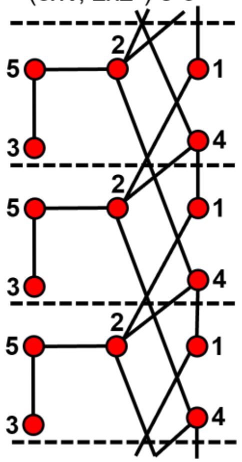

(8x**1**, 2x$\mathbf{2}^{2}$) **d-1**

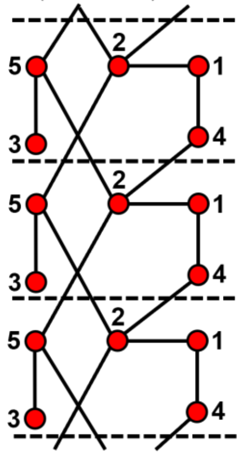

	(8x**1**, 2x**2**^{2}) **d-2** 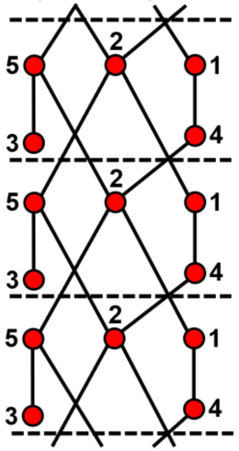	(8x**1**, 2x**2**^{2}) **d-3** 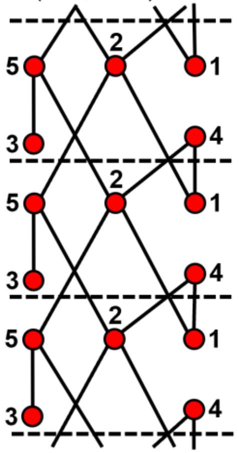	(6x**1**, 3x**2**) **a** 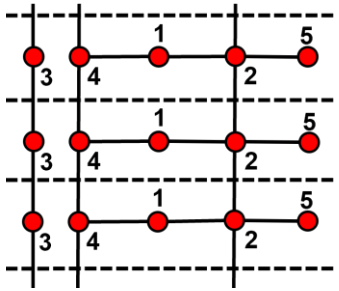	(6x**1**, 3x**2**) **b** 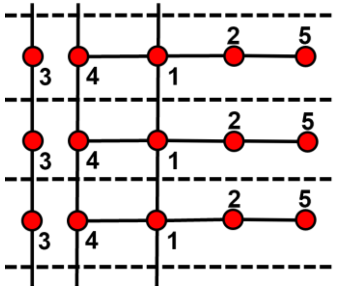
	(6x**1**, 1x**2**, 2x**2**^{1}) **a** 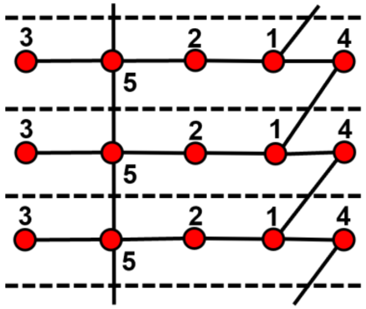	(6x**1**, 1x**2**, 2x**2**^{1}) **b** 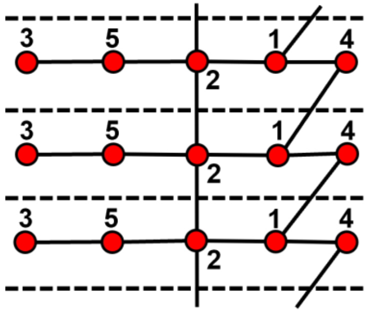	(6x**1**, 1x**2**, 2x**2**^{1}) **c** 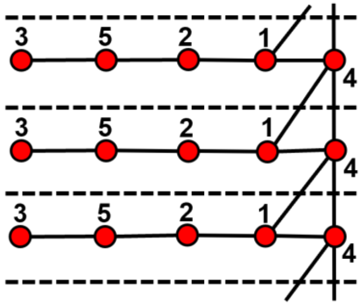	(6x**1**, 1x**2**, 2x**2**^{1}) **d-1** 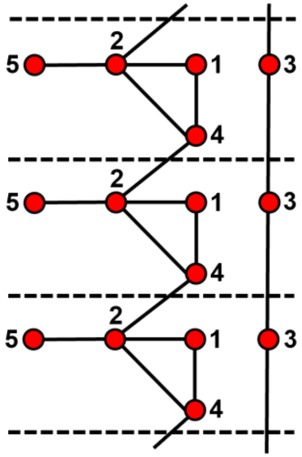

(6x**1**, 1x**2**, 2x$\mathbf{2}^{1}$) **d-2**

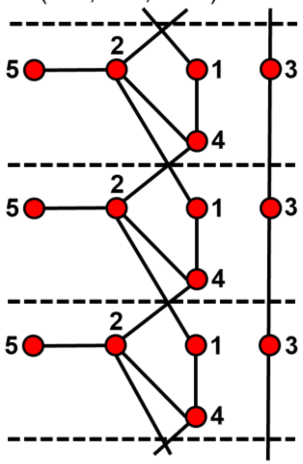

(6x**1**, 1x**2**, 2x$\mathbf{2}^{1}$) **d-3**

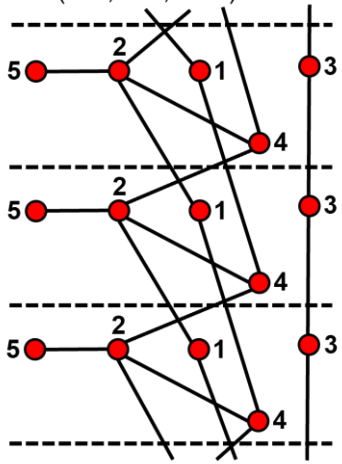

(6x**1**, 1x**2**, 2x$\mathbf{2}^{1}$) **e**

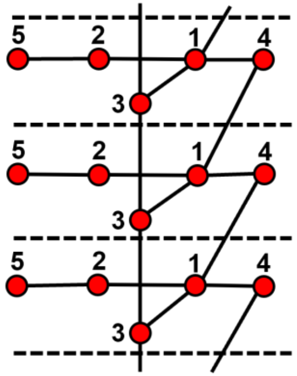

(6x**1**, 1x**2**, 2x$\mathbf{2}^{1}$) **f**

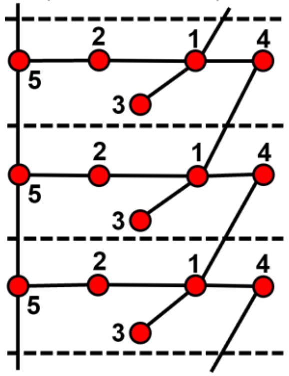

(6x**1**, 1x**2**, 2x$\mathbf{2}^{2}$) **a**

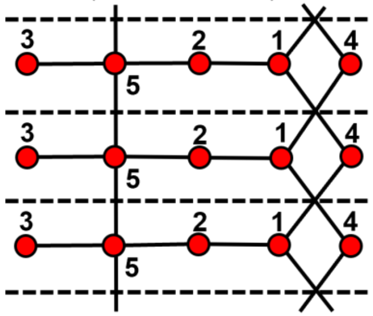

(6x**1**, 1x**2**, 2x$\mathbf{2}^{2}$) **b**

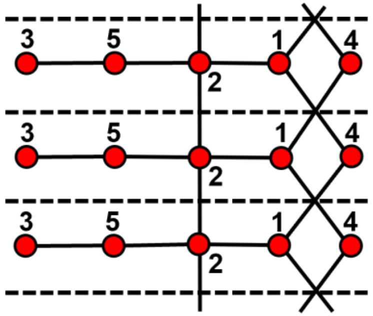

(6x**1**, 1x**2**, 2x$\mathbf{2}^{2}$) **c**

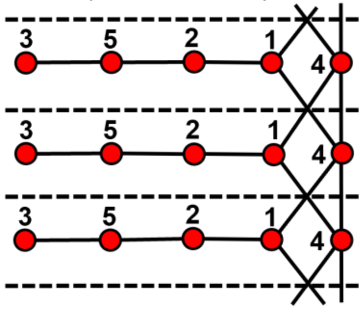

(6x**1**, 1x**2**, 2x$\mathbf{2}^{2}$) **d-1**

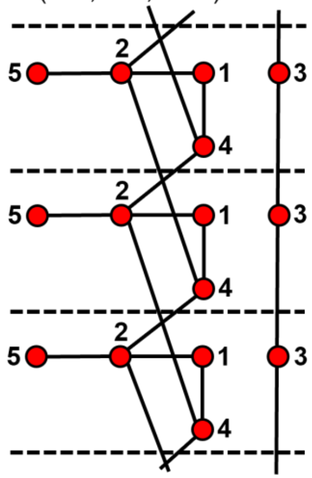

(6x**1**, 1x**2**, 2x$\mathbf{2}^{2}$) **d-2**

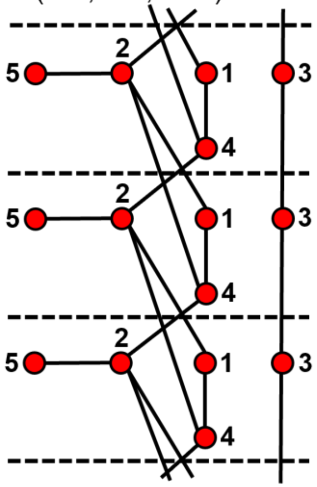

(6x**1**, 1x**2**, 2x$\mathbf{2}^{2}$) **d-3**

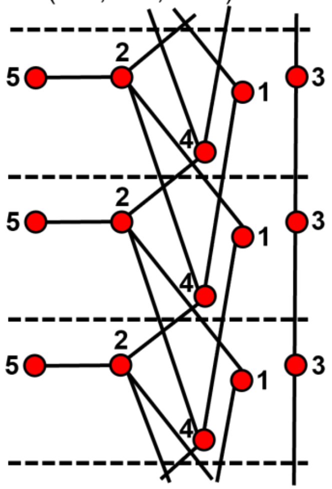

(6x**1**, 1x**2**, 2x$\mathbf{2}^{2}$) **e**

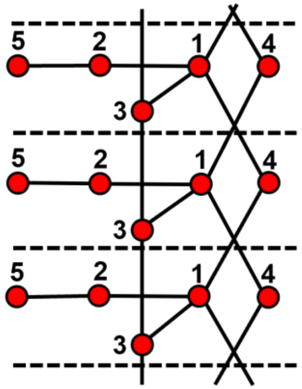

(6x**1**, 1x**2**, 2x$\mathbf{2}^{2}$) **f**

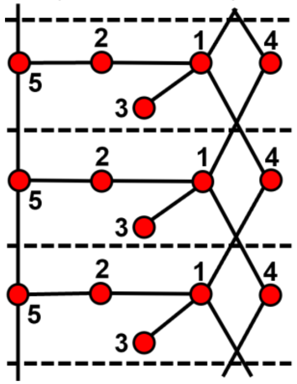

(4x**1**, 4x**2**)

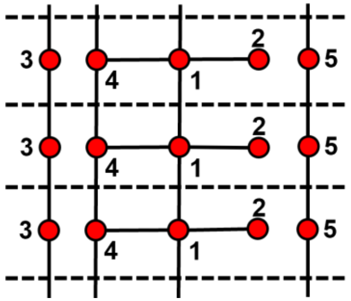

(4x**1**, 2x**2**, 2x$\mathbf{2}^{1}$) **b**

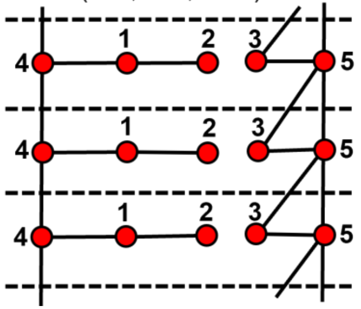

(4x**1**, 2x**2**, 2x$\mathbf{2}^{1}$) **c**

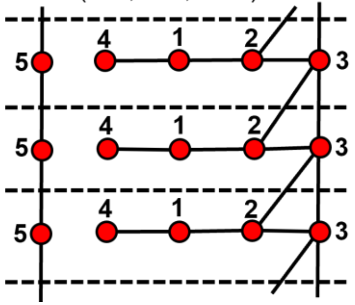

(4x**1**, 2x**2**, 2x$\mathbf{2}^{1}$) **d**

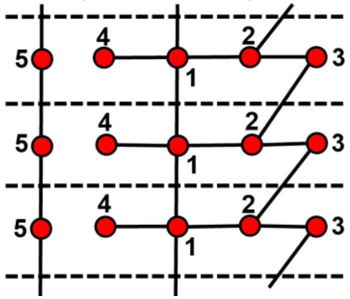

(4x**1**, 2x**2**, 2x$\mathbf{2}^{1}$) **e**

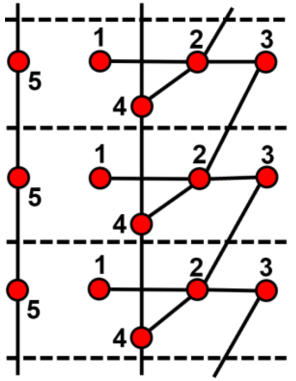

(4x**1**, 2x**2**, 2x$\mathbf{2}^{2}$) **a**

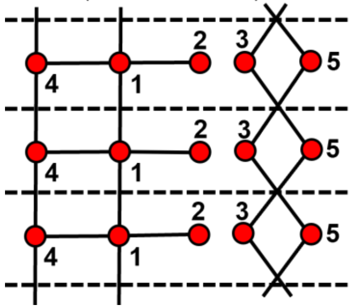

(4x**1**, 2x**2**, 2x$\mathbf{2}^{2}$) **b**

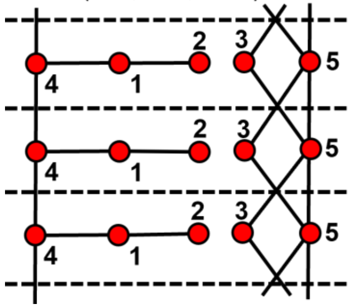

(4x**1**, 2x**2**, 2x$\mathbf{2}^{2}$) **c**

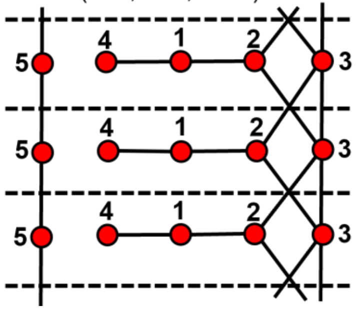

(4x**1**, 2x**2**, 2x$\mathbf{2}^{2}$) **d**

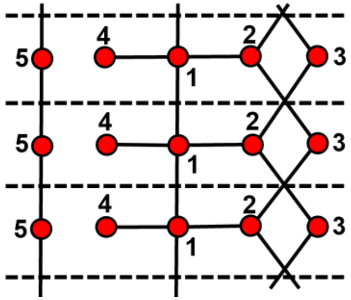

(4x**1**, 2x**2**, 2x$\mathbf{2}^{2}$) **e**

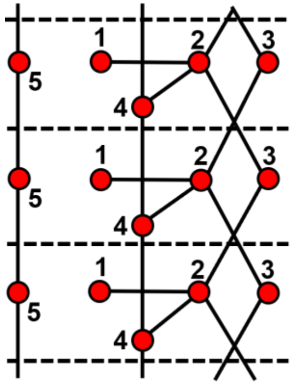

(4x**1**, 4x$\mathbf{2}^{1}$) **a**

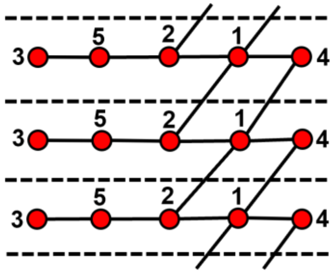

(4x**1**, 4x$\mathbf{2}^{1}$) **b**

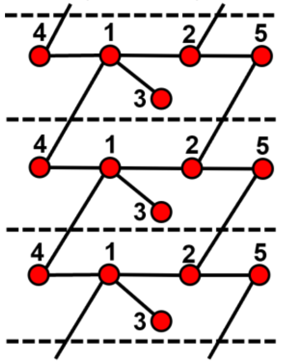

	(4x**1**, 2x**2**^{1}, 2x**2**^{2}) **a**<br>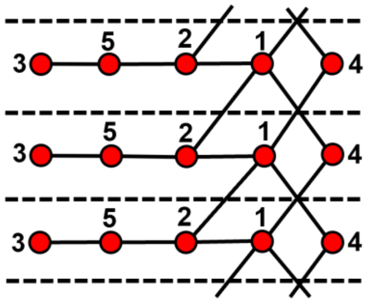 	(4x**1**, 2x**2**^{1}, 2x**2**^{2}) **b**<br>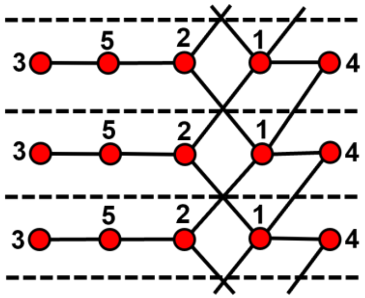 	(4x**1**, 2x**2**^{1}, 2x**2**^{2}) **c**<br>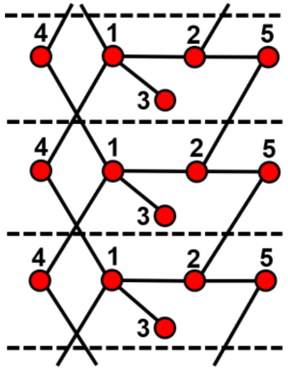 	(4x**1**, 2x**2**^{1}, 2x**2**^{2}) **d**<br>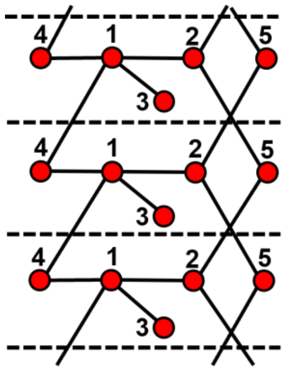
	(4x**1**, 4x**2**^{2}) **a**<br>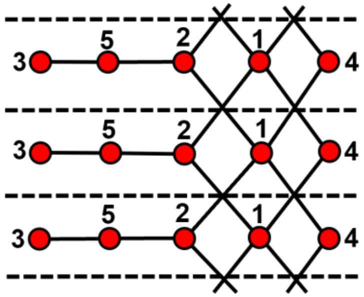 	(4x**1**, 4x**2**^{2}) **b**<br>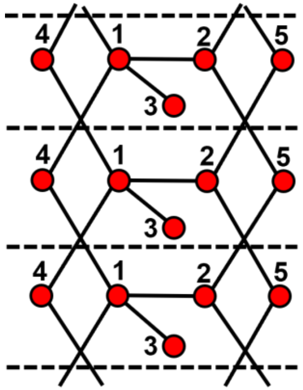 	(2x**1**, 3x**2**, 2x**2**^{1}) **a**<br>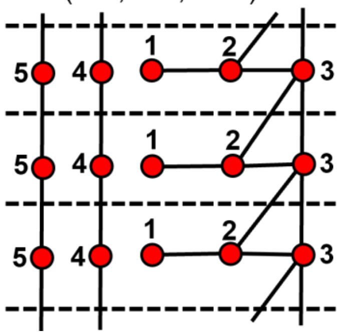 	(2x**1**, 3x**2**, 2x**2**^{1}) **b**<br>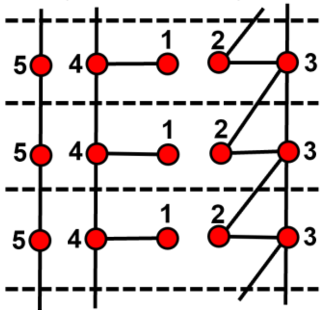

	(2x**1**, 3x**2**, 2x$\mathbf{2}^2$) **a** 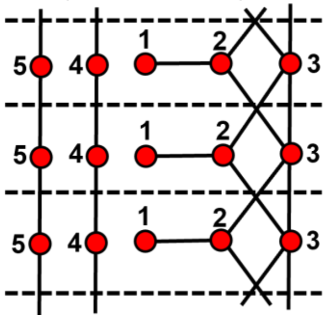	(2x**1**, 3x**2**, 2x$\mathbf{2}^2$) **b** 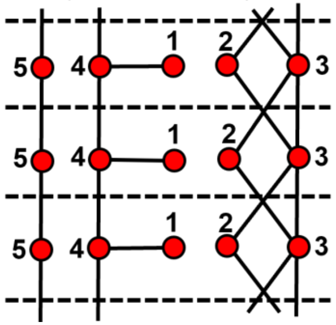	(2x**1**, 1x**2**, 4x$\mathbf{2}^1$) **b** 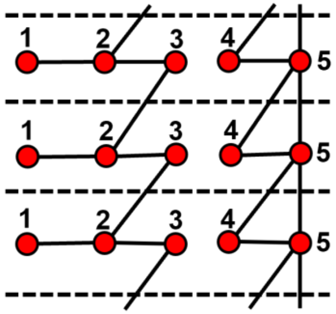	(2x**1**, 1x**2**, 4x$\mathbf{2}^1$) **c** 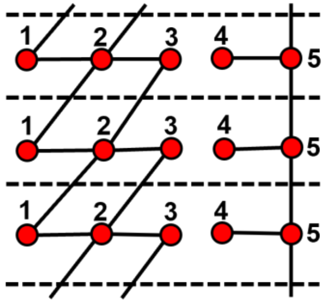
	(2x**1**, 1x**2**, 4x$\mathbf{2}^1$) **d** 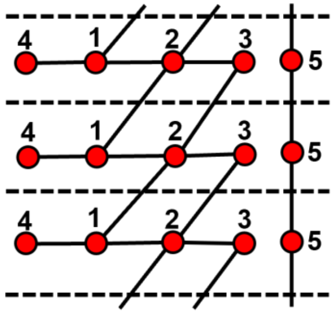	(2x**1**, 1x**2**, 2x$\mathbf{2}^1$, 2x$\mathbf{2}^2$) **b** 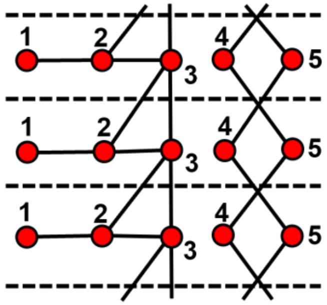	(2x**1**, 1x**2**, 2x$\mathbf{2}^1$, 2x$\mathbf{2}^2$) **c** 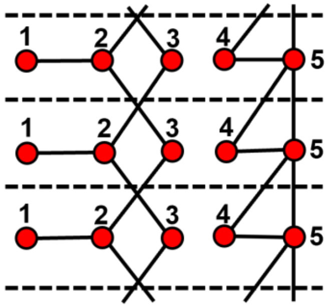	(2x**1**, 1x**2**, 2x$\mathbf{2}^1$, 2x$\mathbf{2}^2$) **d** 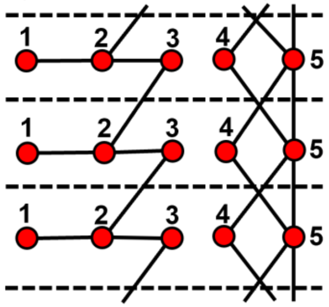
	(2x**1**, 1x**2**, 2x$\mathbf{2}^1$, 2x$\mathbf{2}^2$) **e** 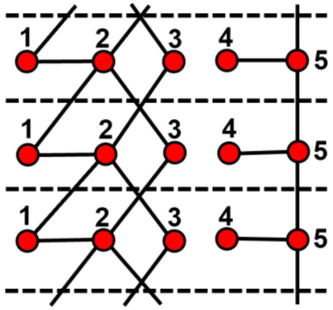	(2x**1**, 1x**2**, 2x$\mathbf{2}^1$, 2x$\mathbf{2}^2$) **f** 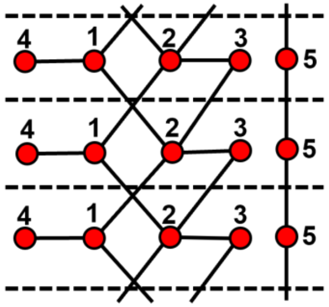	(2x**1**, 1x**2**, 2x$\mathbf{2}^1$, 2x$\mathbf{2}^2$) **g** 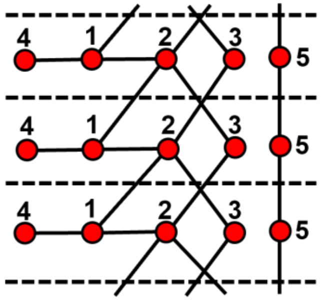	(2x**1**, 1x**2**, 4x$\mathbf{2}^2$) **a** 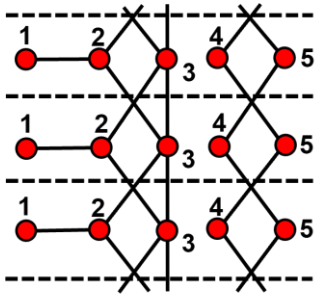

	(2x**1**, 1x**2**, 4x$\mathbf{2}^2$) **b** 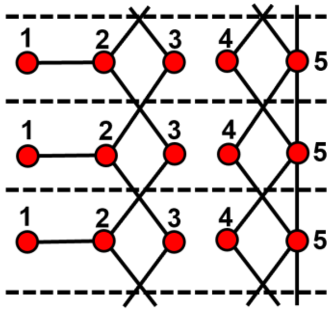	(2x**1**, 1x**2**, 4x$\mathbf{2}^2$) **c** 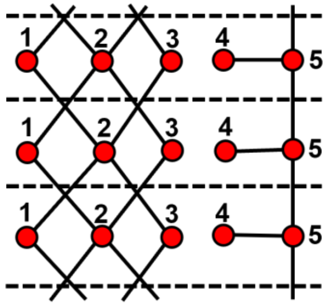	(2x**1**, 1x**2**, 4x$\mathbf{2}^2$) **d** 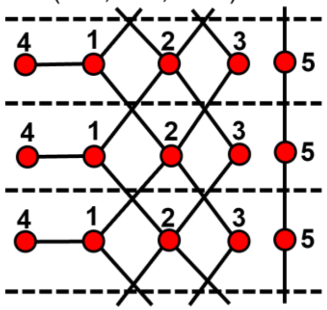	
$^1V_1{}^2V_2{}^3V_1{}^4V_2$	NG			
$^1V_1{}^2V_2{}^3V_1{}^4V_3$	NG			
$^1V_1{}^2V_2{}^3V_1{}^4V_4$	NG			
$^1V_1{}^2V_2{}^3V_3{}^4V_1$	NG			
$^1V_1{}^2V_2{}^3V_3{}^4V_2$	NG			
$^1V_1{}^2V_3{}^3V_1{}^4V_1$	NG			
$^1V_1{}^2V_3{}^3V_1{}^4V_2$	NG			
$^1V_1{}^2V_3{}^3V_1{}^4V_3$	NG			
$^1V_1{}^2V_3{}^3V_3{}^4V_1$	NG			
$^1V_1{}^2V_4{}^3V_1{}^4V_1$	NG			
$^1V_1{}^2V_4{}^3V_1{}^4V_2$	NG			
$^1V_1{}^2V_5{}^3V_1{}^4V_1$	NG			
$^1V_2{}^2V_1{}^3V_2{}^4V_1$	NG			
$^1V_2{}^2V_1{}^3V_2{}^4V_2$	NG			
$^1V_2{}^2V_1{}^3V_2{}^4V_3$	NG			

$^{1}V_{2}{}^{2}V_{1}{}^{3}V_{4}{}^{4}V_{1}$	NG				
$^{1}V_{2}{}^{2}V_{2}{}^{3}V_{2}{}^{4}V_{1}$	NG				
$^{1}V_{2}{}^{2}V_{2}{}^{3}V_{2}{}^{4}V_{2}$	NG				
$^{1}V_{2}{}^{2}V_{3}{}^{3}V_{2}{}^{4}V_{1}$	NG				
$^{1}V_{3}{}^{2}V_{1}{}^{3}V_{1}{}^{4}V_{1}$	NG				
$^{1}V_{3}{}^{2}V_{1}{}^{3}V_{1}{}^{4}V_{2}$	NG				
$^{1}V_{3}{}^{2}V_{1}{}^{3}V_{1}{}^{4}V_{3}$	NG				
$^{1}V_{3}{}^{2}V_{1}{}^{3}V_{3}{}^{4}V_{1}$	NG				
$^{1}V_{3}{}^{2}V_{2}{}^{3}V_{1}{}^{4}V_{1}$	NG				
$^{1}V_{3}{}^{2}V_{2}{}^{3}V_{1}{}^{4}V_{2}$	NG				
$^{1}V_{3}{}^{2}V_{3}{}^{3}V_{1}{}^{4}V_{1}$	NG				
$^{1}V_{4}{}^{2}V_{1}{}^{3}V_{2}{}^{4}V_{1}$	NG				
$^{1}V_{5}{}^{2}V_{1}{}^{3}V_{1}{}^{4}V_{1}$	NG				
Vertex connectivities ($^{c}V_{r}$) where e_A is odd cannot form graphs and are not listed.<br><br>NG = chain graphs not generated.					NG
